# Integrated single-cell and bulk gene expression and ATAC-seq reveals heterogeneity and early changes in pathways associated with resistance to cetuximab in HNSCC-sensitive cell lines

**DOI:** 10.1038/s41416-020-0851-5

**Published:** 2020-05-04

**Authors:** Luciane T. Kagohara, Fernando Zamuner, Emily F. Davis-Marcisak, Gaurav Sharma, Michael Considine, Jawara Allen, Srinivasan Yegnasubramanian, Daria A. Gaykalova, Elana J. Fertig

**Affiliations:** 1 Sidney Kimmel Comprehensive Cancer Center, Johns Hopkins University - School of Medicine, Baltimore, MD USA; 2 Department of Otolaryngology-Head and Neck Surgery, Johns Hopkins University - School of Medicine, Baltimore, MD USA; 3 McKusick-Nathans Institute of the Department of Genetic Medicine, Johns Hopkins University - School of Medicine, Baltimore, MD USA; 4 Department of Medicine, Johns Hopkins University - School of Medicine, Baltimore, MD USA

**Keywords:** Cancer therapeutic resistance, Head and neck cancer

## Abstract

**Background:**

Identifying potential resistance mechanisms while tumour cells still respond to therapy is critical to delay acquired resistance.

**Methods:**

We generated the first comprehensive multi-omics, bulk and single-cell data in sensitive head and neck squamous cell carcinoma (HNSCC) cells to identify immediate responses to cetuximab. Two pathways potentially associated with resistance were focus of the study: regulation of receptor tyrosine kinases by TFAP2A transcription factor, and epithelial-to-mesenchymal transition (EMT).

**Results:**

Single-cell RNA-seq demonstrates heterogeneity, with cell-specific *TFAP2A* and *VIM* expression profiles in response to treatment and also with global changes to various signalling pathways. RNA-seq and ATAC-seq reveal global changes within 5 days of therapy, suggesting early onset of mechanisms of resistance; and corroborates cell line heterogeneity, with different TFAP2A targets or EMT markers affected by therapy. Lack of *TFAP2A* expression is associated with HNSCC decreased growth, with cetuximab and JQ1 increasing the inhibitory effect. Regarding the EMT process, short-term cetuximab therapy has the strongest effect on inhibiting migration. *TFAP2A* silencing does not affect cell migration, supporting an independent role for both mechanisms in resistance.

**Conclusion:**

Overall, we show that immediate adaptive transcriptional and epigenetic changes induced by cetuximab are heterogeneous and cell type dependent; and independent mechanisms of resistance arise while tumour cells are still sensitive to therapy.

## Background

Cancer-targeted therapies are designed to block specific relevant pathways for tumour progression. By doing so, these agents inhibit tumour growth resulting in prolonged patient’s survival.^1^ However, these therapies are not curative and tumours recur or regain growth capability due to acquired resistance that develops within a few years of therapy.^2^ The mechanisms behind the tumour evolution from responsive to resistant state are not fully understood,^3,4^ but can involve mutations to the gene targeted, activation of downstream genes and activation of alternative pathways.^5^ Studies aiming to characterise the mechanisms of resistance have shown an important role of tumour heterogeneity and from cell-adaptive responses to these therapies as the sources of resistance.^6^ The presence of a multitude of cell clones increases the chances of the existence of intrinsic resistant tumour cells that are selected and will keep growing despite the treatment.^6^ In addition, sensitive cell clones have the ability of activating alternative pathways to overcome the blockade of the targeted growth pathway.^7^ Investigating the relevant early adaptive mechanisms that are potential drivers of resistance is critical to introduce early alternative therapies before the phenotype evolves as the dominant feature among the cancer cells.

Currently, cetuximab is the only FDA approved targeted therapeutic for HNSCC,^8^ and was selected based on pervasive overexpression of EGFR and its associations with outcomes in HNSCC.^9,10^ As with other targeted therapies, virtually all HNSCC patients develop acquired resistance limiting its clinical application.^11^ The near universal emergence of resistance and intermediate time rate at which it occurs mark cetuximab treatment in HNSCC as an ideal model system to study resistance. Little is known about the immediate transcriptional and epigenetic changes induced by cetuximab in the very early stages of therapy. We and others have found that compensatory growth factor receptor signalling regulated by *TFAP2A* and EMT, both associated with resistance, are altered while cells are still sensitive to therapy.^12,13^ Therefore, their precise role in resistance and timing at which they induce phenotypic changes remains unknown. It is critical to isolate the timing and effect of each of these pathways during cetuximab response to delineate their subsequent role in resistance.

We hypothesise that the upregulation of mechanisms of resistance arise while HNSCC cells are still sensitive to cetuximab and that some of these mechanisms are associated with chromatin remodelling induced as an immediate response to therapy. Our previous study showed in vitro upregulation of *TFAP2A* 1 day after treatment with cetuximab.^12^ Together with the fact that some of its targets are receptor tyrosine kinases,^14,15^ it is very probable that *TFAP2A* upregulation, or of its targets, is one of the mechanisms activated by HNSCC cells to overcome EGFR blockade and that will induce resistance. Schmitz et al.^13^ also demonstrated that mechanisms of resistance to cetuximab arise early in the course of HNSCC patients’ therapy by detecting EMT upregulation after only 2 weeks of treatment. The stimulation of the EMT phenotype is a common mechanism of resistance to different cancer therapies, including cetuximab.^16–18^ In this study, we focused on these two pathways to investigate how the transcriptional and epigenetic status are rewired while cancer cells are still sensitive to cetuximab.

In order to verify our hypothesis, we performed single-cell RNA sequencing (scRNA-seq) to understand how three HNSCC cell lines and each of their clones respond to a short time course cetuximab therapy. Then, using bulk RNA sequencing (RNA-seq) and assay for transposable-accessible chromatin (ATAC-seq), we investigated the gene expression and chromatin accessibility changes, respectively, of two relevant pathways (TFAP2A and EMT). We verified the heterogeneous and dynamic response to cetuximab among the cell models with cell line-specific adaptive responses to cetuximab and clear disturbances in both pathways. *TFAP2A* regulates HNSCC growth in vitro, and in its absence cells proliferate less. A potential interplay with the EMT was not verified, suggesting that two independent resistance mechanisms to cetuximab are early events in the course of therapy. The response to the combination therapy cetuximab and JQ1, a bromodomain inhibitor known to delay acquired cetuximab resistance,^19^ although heterogeneous, is more efficient to cell growth control than anti-EGFR therapy alone, suggesting that combined therapies blocking multiple growth factors are beneficial in the early stages of therapy.

## Methods

### Cell culture and proliferation assay

UM-SCC-1 (SCC1), UM-SCC-6 (SCC6) and SCC25 cells were cultured in Dulbecco’s Modified Eagle’s Medium and Ham’s F12 supplemented with 10% foetal bovine serum and maintained at 37 °C and 5% CO_2_. A total of 25,000 cells were plated in quintuplicate in six-well plates. Cetuximab (Lilly) was purchased from Johns Hopkins Pharmacy, and JQ1 from Selleck Chemicals. Cell lines were treated daily with cetuximab (100 nM), JQ1 (500 nM), the combination or vehicle (PBS + DMSO; mock) for 5 days. Proliferation was measured using alamarBlue assay (Thermo Scientific). AlamarBlue (10% total volume) was added to each well, and fluorescence (excitation 544 nm, emission 590 nm) was measured after 4 h of incubation at 37 °C. A media only well was used as blank. The measurements were repeated in three independent experiments. Growth rate was calculated using the formula:

1 $$GR = 2^{k(c,t)/k(0)}-1$$

Where *k(0)* = fluorescence measured for non-treated cells and *k(c,t)* = fluorescence for treated cells.^20^

Parental cell lines were authenticated before and after all assays using short tandem repeat (STR) analysis kit PowerPlex16HS (Promega) through the Johns Hopkins University Genetic Resources Core Facility.

### Single-cell RNA sequencing (scRNA-seq)

Cetuximab and untreated HNSCC cell lines were trypsinised, washed and resuspended in PBS. Cell counts and viability were made using Trypan Blue staining (ThermoFisher) in the haemocytometer. Single-cell RNA labelling and library preparations were performed using the 10× Genomics Chromium™ Single Cell system and Chromium™ Single Cell 3′ Library & Gel Bead Kit v2 (10× Genomics), following the manufacturer’s instructions. An input of 8700 was used to recover a total of 5000 cells. Sequencing was performed using the HiSeq platform (Illumina) for 2 × 100 bp sequencing and ~50,000 reads per cell. Samples were sequenced in duplicate. Sequences were filtered and aligned using the CellRanger software (10× Genomics). Data normalisation, pre-processing, dimensionality reduction (method: UMAP), cell clustering (method: louvain), differential expression analysis and visualisation were performed using Monocle 3 alpha (version 2.10.1).

The scRNA-seq data are available at GEO (GSE137524).

### EVA analysis

EVA from the R/Bioconductor package GSReg^21^ version 1.17.0 was used to quantify pathway dysregulation in sets of cells from cetuximab group relative to the set of untreated (PBS) cells. Imputed scRNA-seq data are input to this algorithm. Imputation was performed with MAGIC version 0.1.0.^22^ *P*-values obtained from EVA analysis are FDR adjusted with the Benjamini–Hochberg correction, values below 0.05 considered statistically significant.

### RNA velocity

We used kb-python, a python package that wraps the kallisto and bustools single-cell processing tools,^23,24^ to generate gene count matrices of spliced and unspliced transcripts for each cell line. The cells that were filtered using Monocle 3 were sub-setted for RNA velocity analysis by scVelo.^25^ All the code is available at https://github.com/FertigLab/SingleCellTimeCourse.

### RNA isolation and RNA sequencing

RNA isolation and sequencing were performed from day 0 to 5 of treatment at the Johns Hopkins Medical Institutions Deep Sequencing & Microarray Core Facility. The total RNA was isolated from at least 1000 cells collected on 1 ml of QIazol reagent (Qiagen), following the manufacturer’s instructions. Concentration and quality were measured at the 2100 Bioanalyzer (Agilent), with RNA Integrity Number (RIN) of 7.0 as the minimum threshold. Library preparation used the TrueSeq Stranded Total RNAseq Poly A1 Gold Kit (Illumina), according to the manufacturer’s recommendations, followed by mRNA enrichment using poly(A) enrichment for ribosomal RNA removal. Sequencing was performed using the HiSeq platform (Illumina) for 2 × 100 bp sequencing. Transcript abundance from the RNA-seq reads was inferred using Salmon.^26^ To import Salmon outputs and export into estimated count matrices, we used tximport.^27^ DESeq2 was used for differential expression analysis.

All RNA-seq data are available at GEO (GSE114375).

### ATAC-sequencing

ATAC-seq library preparation was performed as previously described.^28^ Cells were collected after 5 days of treatment (100,000 cells for each group) by scrapping, and were washed and lysed. Nuclei tagmentation and adapter ligation by Tn5 was performed using the Nextera DNA Sample Preparation kit (Illumina), followed by purification with MinElute PCR Purification kit (Qiagen) according to the manufacturers’ instructions. Transposed DNA fragments were amplified using the NEBNext Q5 HotStart HiFi PCR Master Mix with regular forward and reverse barcoded primers. Additional number of amplification cycles were determined by quantitative-PCR using the NEBNext HiFi Master Mix, SYBR Green I (Applied Biosystems) and Custom Nextera Primers. The final product was purified with MinElute PCR Purification kit (Qiagen), and quality checked on 2100 Bioanalyzer (Agilent). Sequencing was performed using the HiSeq platform (Illumina) for 2 × 50 bp sequencing with ~50 million reads per sample.

Sequences quality were assessed using FastQC.^29^ After adapters trimming with Trim Galore! (version 0.5.0), sequences were aligned with Bowtie2 (version 2.3.2) to the human genome (hg19).^30^ Duplicated and mitochondrial reads were removed with Picard Tools (version 2.18),^31^ while unmapped and low-quality reads were removed with SAMtools (version 1.9).^32^ MACS2 was used for peaks calling.^33^ Correlation analysis and differential bound site analysis were performed with DiffBind.^34^ The annotated differential binding sites were filtered for peaks in promoter regions.

All ATAC-seq data are available at GEO (GSE135604).

### *TFAP2A* RNA interference assay

Cells were transfected with a pool of four siRNA sequences (ON-TARGETplus Human TFAP2A pool, Dharmacon) to silence *TFAP2A* expression 1 day after plating. ON-TARGETplus Non-targeting Pool (NTP) and ON-TARGETplus GAPD Control Pool were used as negative and positive transfection controls, respectively. Transfection was performed in serum-free Opti-MEM (Invitrogen) and RNAiMAX Lipofectamine Reagent (Invitrogen). Eight hours after transfection, opti-MEM was replaced with complete medium, and cells were incubated overnight at 37 °C. Treatment with cetuximab, JQ1, the combination or vehicle was performed daily for 5 days. Transfection efficiency and level of the endogenous gene were monitored by qRT-PCR before and 72 h after transfection. Cell proliferation was measured by the alamarBlue assay as described above. Each assay was performed in quintuplicate for each cell line and treatment.

### qRT-PCR analysis

Cell lines were lysed directly in the cell culture plate by adding Qiazol reagent (Qiagen) and RNA isolation followed by the manufacturer’s instructions. Reverse transcription of 300 ng of the total RNA was performed with qScript Master Mix (Quanta Bioscience). Gene expression was determined using TaqMan Universal Master Mix II and TaqMan 20X Gene Expression Assays in a 7900HT equipment (Life Technologies). All assays were quantified in triplicate relative to *GAPDH* using the 2^−ΔΔCt^ method.

### Migration assay

The migration assays were performed in the Culture-Insert 2 Well 24 (Ibidi). In each insert well, 10,000 cells (transfected and not transfected with TFAP2A siRNA) were plated, and 24 h after plating, treated with cetuximab, JQ1, their combination or vehicle. Once, cells were confluent the inserts were removed and gap closure was measured under a microscope at 0 h, 6 h, 12 h and 24 h. The gap area measurements were made using ImageJ,^35^ and closure was determined as the ratio between the initial area and the measured area at each time point. Experiments were performed at least three times.

## Results

### TFAP2A and EMT expression are heterogeneous among cell lines

**Fig. 1:**
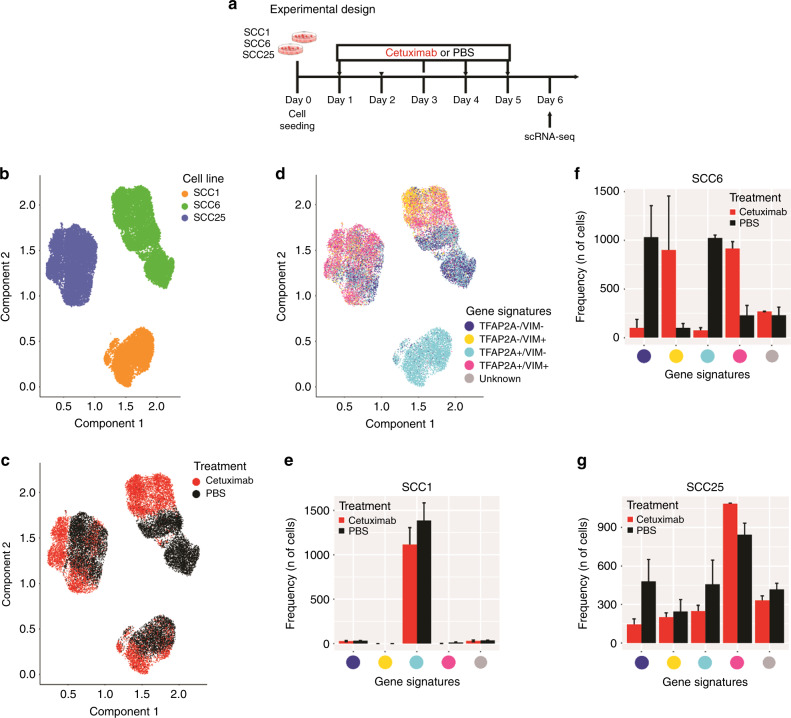
Single-cell RNA-seq profiling of cetuximab-treated and -untreated HNSCC cell lines. **a** SCC1, SCC6 and SCC25 cell lines were treated with cetuximab or PBS (untreated controls) for 5 consecutive days after which cells were collected for single-cell RNA-seq (scRNA-seq). **b** scRNA-seq analysis demonstrates that each cell line presents a specific gene expression profile. **c** In response to cetuximab, the SCC6-treated (red) and untreated (black) clones separate completely, while the SCC1 and SCC25 present some overlap in the distribution regarding the transcriptional profile. **d** Inter-cell heterogeneity is more evident for *TFAP2A* and *VIM* mRNA levels, with SCC1 presenting high levels of *TFAP2A* and no expression of *VIM*. The co-expression analysis shows that in SCC1 there is no change in the levels of *TFAP2A* or *VIM* in response to cetuximab; SCC6-treated cells are *VIM* + (orange and purple), while untreated are negative (green and blue) with different status for *TFAP2A* expression; and most of the SCC25 cells responding with increase in *VIM*, but with some untreated clones presenting the same expression profile for *VIM* and *TFAP2A* (purple) and with *VIM-* clones only detected in the untreated group. **e**, **f**, **g** Bar plots represent the number of treated and untreated cells per each gene signature.

To investigate the heterogeneous responses induced by therapy before resistance developed, sensitive HNSCC models were used to interrogate the immediate changes induced by cetuximab. Based on previous work demonstrating HNSCC cell lines sensitivity to cetuximab^16,36^ and confirmed by proliferation assay, we chose the cell lines SCC1, SCC6 and SCC25 (Supplementary Fig. 1). To verify heterogeneity and how each of the cell clones respond to cetuximab, we performed single-cell RNA-seq (scRNA-seq). The cell lines received cetuximab (treated) or PBS (untreated) and after a total of 5 days the cells were collected in single-cell suspensions for the library preparations and sequencing (Fig. 1a). The PBS (untreated controls) single-cell gene expression levels were measured after a total of 5 days (120 h) of cell culture in order to reflect the same culture conditions as the cetuximab-treated cells.

Based on the whole-transcriptomic profile, each cell line cluster completely separate from each other (Fig. 1b) demonstrating expected inter-cell line heterogeneity. Analysing the cell clusters according to cetuximab therapy, we noted that each cell line presents specific early transcriptional responses. There is a clear separation between treated and untreated cells in SCC6 (Fig. 1c), suggesting that in only 5 days anti-EGFR therapy induces significant transcriptional changes when compared with the untreated (PBS) cells. For SCC1 and SCC25, there are treated cells that cluster with the untreated ones (Fig. 1c), and most probably in these cell lines prolonged exposure is necessary for more significant changes in gene expression.

To investigate the immediate emergence of potential mechanisms of resistance, we investigated the expression of *TFAP2A* and *VIM*, alone or concomitantly, to verify the behaviour of these pathways (transcription regulation by TFAP2A and EMT process) in response to cetuximab. We evaluated the expression of *TFAP2A* and *VIM* genes in the individual cells (Supplementary Fig. 2) and used the individual markers expression levels to classify each individual as double-negative (*TFAP2A-/VIM-*), *TFAP2A*-positive (*TFAP2A* + */VIM-*), *VIM*-positive (*TFAP2A-/VIM* + ) and double-positive (*TFAP2A* + */VIM* + ) (Fig. 1f). The scRNA-seq analysis of the three cell lines show heterogeneity regarding the expression of *TFAP2A* and *VIM* genes. Cetuximab-treated and untreated SCC1 show high levels of *TFAP2A* and absence of *VIM* expression (Supplementary Fig. 2; Fig. 1d, e), suggesting no influence of therapy in these two markers for this specific cell line. SCC6 cells present a definite shift in the expression of *VIM* with the anti-EGFR blockade, with untreated cells presenting downregulation when compared with the treated cells. The shift in *VIM* expression was independent of the *TFAP2A* status (Supplementary Fig. 2; Fig. 1d, f), without apparent variation in the proportions of positive and negative cells in response to cetuximab. Interestingly, the majority of SCC25 cells are double-positive with or without cetuximab therapy. In the presence of EGFR blockade, *VIM* expression is positive among most of the treated clones (double-positive), while the proportions of untreated SCC25 cells expressing or lacking *VIM* are approximate (Supplementary Fig. 2; Fig. 1d, g).

Based on the SCC6 expression profile, there is evidence that cetuximab is capable of inducing *VIM* expression and, corroborating the observation from Schmitz et al.^13^ that cetuximab induces EMT markers early on in the course of therapy. However, most of the transcriptional changes in response to cetuximab are cell type dependent.

### Heterogeneity measurements and RNA velocity show dynamic gene expression changes in response to cetuximab

For a deeper characterisation of single-cell heterogeneity in response to cetuximab therapy, we applied a computational tool, previously developed by our group to quantify dysregulation between two conditions from bulk sequencing data, expression variation analysis (EVA).^21^ We extended EVA for heterogeneity measurement from scRNA-seq,^37^ by performing multivariate statistical analyses of differential variation of expression in gene sets from the scRNA-seq data. Heterogeneity was defined as pathways differentially variable (heterogeneous) between cetuximab-treated and untreated controls (PBS). EVA and gene set enrichment analyses were performed for the Hallmark gene sets in MSigDB version 6.1.^38^

**Fig. 2:**
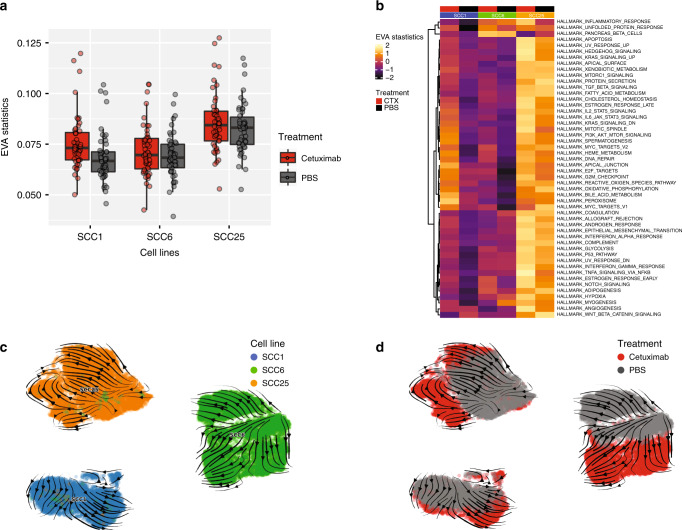
Single-cell heterogeneity and RNA velocity changes as a response to cetuximab. **a**, **b** SCC1 presents increased heterogeneity as measured by EVA statistics in MSigDB Hallmark pathways. Changes in SCC6 and SCC25 heterogeneity are not as significant, but also suggest an adaptive early response to cetuximab. **b**, **c** RNA velocity analysis corroborates the heterogeneity analysis and shows that cetuximab triggers a dynamic process in all three cell lines and suggests that cells would move from a more homeostatic state (PBS, grey) to a state with increased transcriptional complexity (CTX, red) in response to therapy.

EVA analysis indicates that there is increased heterogeneity among hallmark pathways between cetuximab and PBS groups in SCC1, SCC6 and SCC25, although in the last two cell lines the variation is not significant (Fig. 2a, b). SCC1 cetuximab-treated cells show increased heterogeneity in 49 hallmark signalling pathways (Supplementary Table 1), suggesting that anti-EGFR therapy is inducing immediate global changes to different relevant pathways in this cell line. Although not statistically significant, SCC6 and SCC25 present a total of 40 and 39 hallmark pathways, respectively (Supplemental Table 1), changed in response to cetuximab compared with untreated cells. Most probably, these two HNSCC cell lines would need a longer exposure to targeted therapy to present with the same heterogeneity as SCC1 cetuximab-treated cells. The heterogeneity measurements using EVA suggest that during the course of treatment, heterogeneity starts to increase as an immediate response to cetuximab. This effect is probably due to the fact that different cell subclones in the same cell line are activating alternative pathways to overcome EGFR inhibition.

scRNA-seq is a powerful tool that provides quantification of RNA abundance for each individual cell at a specific time point and allows, as demonstrated above, quantification of heterogeneity to different conditions. A new approach, RNA velocity,^39^ allows prediction of cell fate based on the global transcriptional changes captured during dynamic processes, such as response to therapy, in scRNA-seq experiments. RNA velocity uses the ratio unspliced/spliced mRNA to determine cell fate. In a dynamic process, gene upregulation is expected to reflect in increased unspliced mRNA followed by increased spliced variants. The ratio unspliced/spliced mRNA can be used to infer which genes are probably being up- or downregulated or kept stable to maintain homeostasis.

RNA velocity analysis to compare treated and untreated HNSCC cells, demonstrate that cetuximab induces transcriptional changes that reflect a dynamic process (Fig. 2c). In all three cell lines evaluated, the directional flow of untreated cells (grey) is towards the cetuximab-treated cells (red) (Fig. 2d). These results suggest that in the course of treatment, HNSCC cells in vitro would progress from a state where most pathways are in a homeostatic state due to the presence of EGFR activity (here, represented by the untreated cells) and would progress to a state with upregulation of different mRNAs in order to activate alternative pathways to overcome EGFR inhibition (represented by the cetuximab-treated cells).

The heterogeneity measurements and RNA velocity analysis suggest that the immediate response to short time exposure to cetuximab is a dynamic process and reflects in global transcriptional changes in order to overcome the lack of EGFR activation.

### Cetuximab induces immediate gene expression changes in HNSCC in vitro

**Fig. 3:**
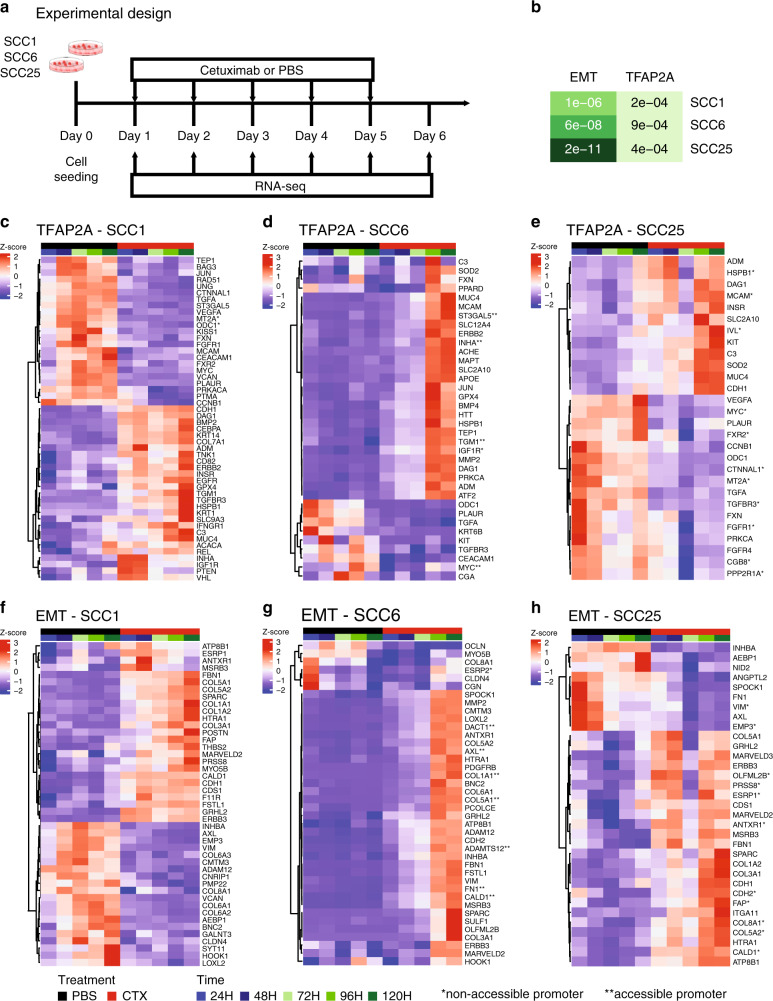
*TFAP2A* targets and EMT markers expression in response to cetuximab. **a** SCC1, SCC6 and SCC25 cell lines were treated with cetuximab or PBS (untreated controls) for 5 consecutive days, and cells were collected daily for bulk RNA-seq (RNA-seq). **b** Among the genes differentially expressed among all three cell lines as a response to cetuximab therapy, the gene set enrichment analysis shows significant presence (*p* ≤ 0.05) of genes that are TFAP2A targets or that participate in the EMT process. When analysed individually, the TFAP2A and EMT differential expressed genes are specific in each of the cell lines. **c**, **f** SCC1 and **e**, **h** SCC25 present changes as soon as 24 h (1 day) after cetuximab therapy, while in (**d**, **g**) SCC6 the changes are only detected at 96 h (4 days) after cells are treated.

In order to evaluate the timing of the changes in the TFAP2A targets and EMT markers and to interrogate each of the pathway genes individually, we performed daily measurements in treated and untreated groups for all three cell lines with bulk RNA sequencing (RNA-seq) (Fig. 3a).

Transcriptional changes induced by cetuximab can be detected genome wide almost immediately after therapy. Differential expression analysis of all timepoints indicate that hundreds of genes have their transcriptional profile changed as a response to anti-EGFR therapy in all three HNSCC cell lines with changes occurring as early as 24 h after treatment (Supplementary Fig. 3A, Supplementary Tables 2–5). In order to investigate the changes in the activity of TFAP2A transcription factor, we followed the expression of its targets identified using the TRANSFAC database.^14,15^ To analyse the status of the EMT pathway, we analysed the EMT markers from the gene signature described by Byers et al. that can predict resistance to anti-EGFR and anti-PI3K therapies.^40^ When each cell line was investigated separately, the gene set enrichment analysis comparing cetuximab and untreated timepoints showed that among the differentially expressed genes in SCC1, 55 are TFAP2A targets (*p* = 2.2e-04) and 49 are EMT markers (*p* = 1.1e-04); in SCC6, there are 46 genes from each pathway (TFAP2A *p* = 9e-04, EMT *p* = 6e-08); and in SCC25, there are 40 TFAP2A targets (*p* = 4.3e-04) and 46 EMT markers (*p* = 2.2e-11) (Fig. 3b). Although there was no variation in the expression of *TFAP2A* and *VIM* in SCC1, there are still significant changes to other markers in both pathways that are potentially associated with future development of acquired cetuximab resistance. The cell lines SCC1 and SCC25 present immediate transcriptional changes to the cetuximab therapy, and most of the genes present expression changes in the first 24 h of therapy (Fig. 3c, f, e, h). SCC6 transcriptional response to anti-EGFR treatment takes longer and most of the changes are noticeable after 96 h of therapy (Fig. 3d, g), which is in agreement with the observed behaviour of this cell line to the cetuximab therapy (Supplementary Fig. 1).

### Chromatin changes can be detected early in the course of cetuximab therapy in vitro

**Fig. 4:**
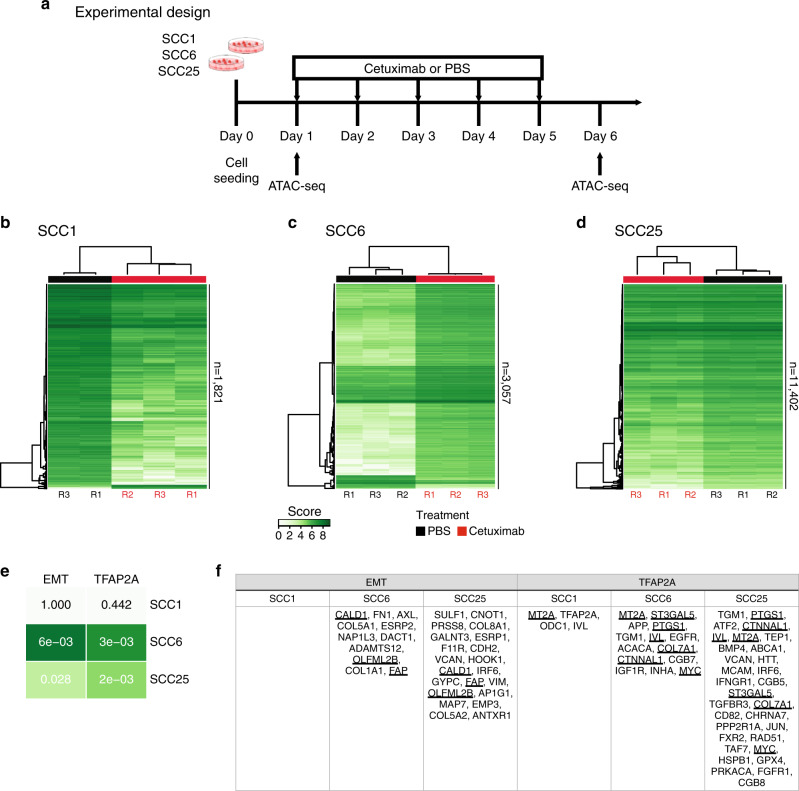
Chromatin structure changes during short time treatment with cetuximab. **a** ATAC-seq was performed after SCC1, SCC6 and SCC25 were treated for 5 days with cetuximab, and also in the untreated (PBS) controls. **b**–**d** Differential binding analysis show that the promoters accessibility changes in response to 5 days of therapy are capable of separating the cetuximab from the PBS replicates in all three cell lines. **e** With the exception of SCC1, there are enrichment for TFAP2A and EMT promoters among the ATAC-seq peaks in SCC6 and SCC25. **f** The differential binding analysis show that SCC25 is the gene with the highest number of genes with chromatin changes in response to cetuximab, and also identified promoters that are changed in more than one cell line (underlined gene names).

We hypothesised that epigenetic rewiring induced by cetuximab is the most probable cause of the adaptive transcriptional changes we detected with RNA-seq. To verify if chromatin remodelling occurs early during cetuximab treatment and if it affects the TFAP2A targets and EMT genes, we measured global chromatin accessibility by ATAC-seq in cells treated with cetuximab and in the untreated controls after five days of therapy (Fig. 4a).

Cetuximab induces significant chromatin changes after only 5 days of therapy (Supplementary Fig. 3B). Differential bound analysis, to identify the accessible protein-DNA binding regions in cetuximab versus untreated groups, shows that there are a total of 1690 binding regions, common to SCC1, SCC6 and SCC25, that have their structure changed as a response to therapy. The unsupervised clustering of these common regions separates the samples that were treated from the untreated controls (Supplementary Fig. 3B, Supplementary Tables 6–9). These findings suggest that epigenetic rewiring is an early event in response to cetuximab, and is probably involved in the regulation of some relevant transcriptional changes observed.

The differential binding analysis was performed for each cell line individually to identify cell-specific chromatin changes in response to cetuximab (Fig. 4b–d). Each of the three cell lines presents specific chromatin changes that separate the groups of treated and untreated replicates. SCC1 and SCC6 show significant promoters reconfiguration as a response to therapy with 1821 and 3057 sites remodelled, respectively (Fig. 4b, c). SCC25 presents the largest number of gene promoters remodelled with 11,402 promoter-binding sites (including genes with more than one binding site) as a result of short-term therapy (Fig. 4d).

The gene set enrichment analysis identified genes from the TFAP2A and EMT pathways in the list of promoters that have chromatin structural changes induced by cetuximab. Promoter region reconfiguration during cetuximab treatment in the SCC1 cell line was detected in only four genes from the TFAP2A pathway, and no changes in EMT promoters is present (Fig. 4e, f). Suggesting that in this cell line, the transcriptional changes in both pathways are not regulated by chromatin remodelling. A total of 11 promoters from the TFAP2A pathway (*p* = 3e-03, Fig. 4e, f) and the same number of EMT gene promoters (*p* = 6e-03, Fig. 4e, f) have their chromatin structure changed by the anti-EGFR therapy in SCC6. The SCC25 cell line presents, as a response to cetuximab, chromatin changes in 31 TFAP2A pathway (*p* = 0.028, Fig. 4e, f) and in 21 EMT promoters (*p* = 2e-03, Fig. 4e, f). Interestingly, all chromatin changes to the SCC25-binding sites make them less accessible when compared with the untreated controls. The ATAC-seq findings suggest that even after a short time exposure of HNSCC cells to cetuximab in vitro, genes from pathways that are associated with acquired resistance present remodelling that could potentially result in altered transcription factors binding.

The genes with transcriptional and chromatin alterations in response to short time treatment with cetuximab are marked with one (non-accessible after cetuximab) or two stars (accessible after cetuximab) in the RNA-seq heatmaps in Fig. 2. As would be expected, the correlation between accessibility and expression is not true for all genes. Although a few relevant genes, such as *AXL* (Fig. 3d), known to be upregulated in acquired resistance to different targeted agents, presents open chromatin combined with upregulation in SCC6-treated cells.

### *TFAP2A* controls HNSCC proliferation in vitro

**Fig. 5:**
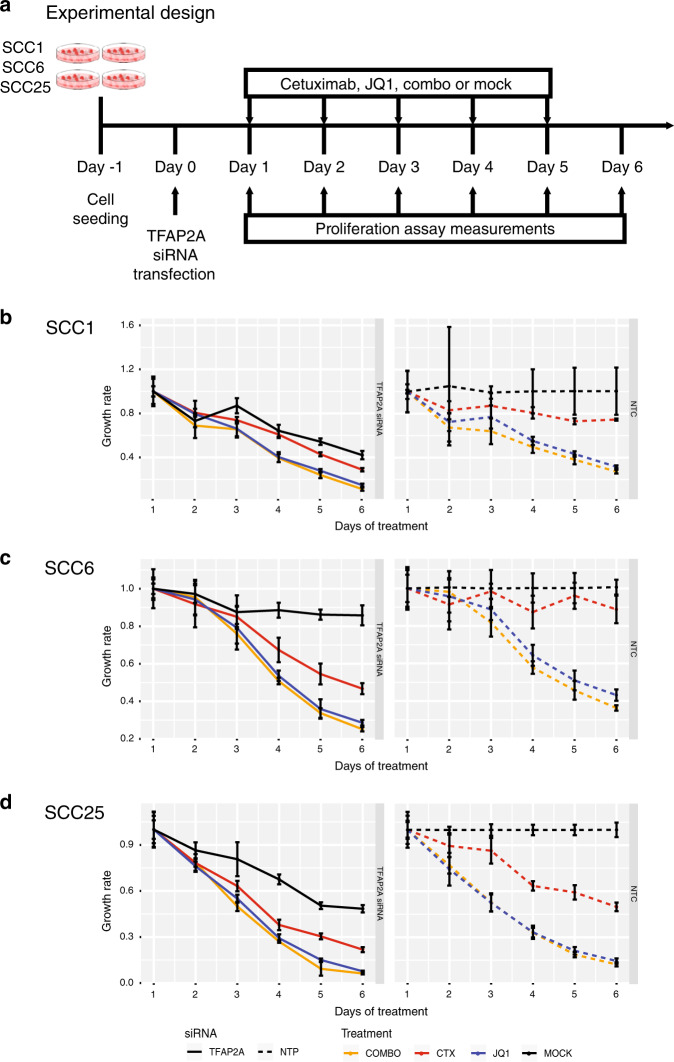
Effect of TFAP2A knockdown in HNSCC cell growth during treatment with cetuximab and JQ1 alone or the combination. **a** Functional validation of the role of *TFAP2A* in HNSCC in vitro was evaluated by siRNA gene silencing in SCC1, SCC6 and SCC25. Cells were treated with cetuximab, JQ1, combination (combo) or vehicle (mock) for 5 days, and the impact of gene knockdown and therapy was determined by measuring proliferation rates. **b**–**d** Transfected groups (full lines, left) were compared with the groups with normal levels of *TFAP2A* (dashed lines, right - NTC). In all cell lines, *TFAP2A* knockdown induce lower proliferation rates (black lines) at different levels, depending on the cell. Cetuximab treatment (red lines) present a synergistic effect, but JQ1 (blue lines) efficacy is even greater in reducing cell growth. Little effect is noted with the combination (orange lines, COMBO) when compared with the effects of JQ1 alone.

The role of *TFAP2A* in HNSCC is poorly characterised. As a transcription factor, it is capable of regulating the expression of several growth factor receptors (EGFR, HER2, TGFBR3, FGFR1, IGFR1 and VEGF).^14,15^ In order to investigate the role of *TFAP2A* in HNSCC cell proliferation in vitro, we used siRNA assay for gene silencing and measured growth rates for 5 days following therapy (Fig. 5a). All transfected cell lines present lower growth rates when compared with the parental cell lines (Fig. 5b–d, black full and dashed lines). The effect of *TFAP2A* is more prominent in SCC1 and SCC25 if compared with SCC6. This is probably related to the fact that both cell lines present *TFAP2A* expression in most of the cell clones, as shown by the scRNA-seq (Fig. 1d).

Combined with the effects of *TFAP2A* transient knockdown, we investigated the role of cetuximab and JQ1 on HNSCC growth. JQ1 is a bromodomain inhibitor that blocks the transcription of cell growth regulators (e.g., c-Myc) and multiple RTKs, and was previously shown to delay acquired cetuximab resistance.^19^ Cetuximab or JQ1 was added to cell culture media once cells were transfected with *TFAP2A* siRNA, and proliferation was measured daily (Fig. 5a). We also verified how cells would respond to the combination (combo) of both drugs in vitro (Fig. 5a).

Cetuximab therapy potentiates growth inhibition in the absence of *TFAP2A* (Fig. 5b–d, red full and dashed lines) with synergistic effect potency dependent on the cell line. SCC1 presents very similar *TFAP2A* expression in treated and untreated cell clones (Fig. 1d), and the effect of gene knockdown with anti-EGFR therapy is not as significant as observed in SCC6 and SCC25. Discrepancies in the growth rates between Fig. 4 and Supplementary Fig. 1 are a result of unintentional cell cycle synchronisation induced by the incubation of cells with serum-free media for at least 8 h for the siRNA transfection assays. Still, a stronger effect on proliferation control was observed with JQ1 treatment (Fig. 5b–d, blue full and dashed lines), most probably due to the silencing of another proliferation factor (c-Myc) and/or RTKs. Interestingly, the combination therapy of cetuximab and JQ1 does not provide a significantly stronger synergistic effect (Fig. 5b–d, orange full and dashed lines). *TFAP2A* transient knockdown was confirmed by qRT-PCR (Fig. 5e–g). These results indicate that in HNSCC in vitro, the transcription factor TFAP2A is an essential regulator of cell growth.

### Cetuximab inhibits HNSCC cell migration in vitro

**Fig. 6:**
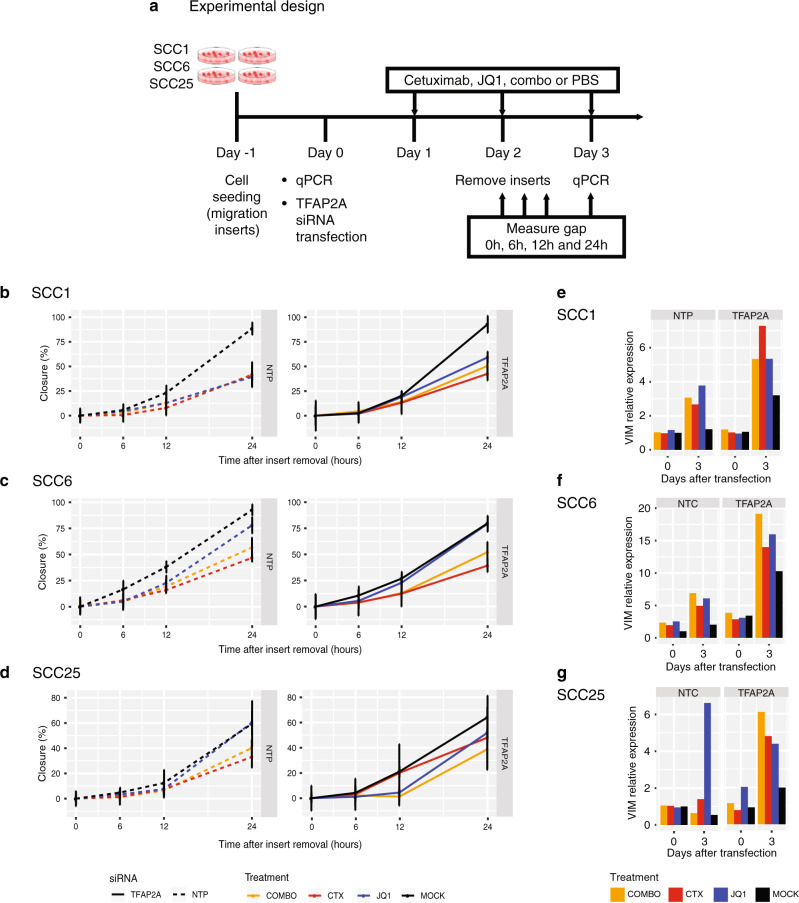
Cell migration in the absence of *TFAP2A* and the effect of cetuximab and JQ1 therapy. **a** To further evaluate the interplay between *TFAP2A* and EMT, cells transfected with siRNA against *TFAP2A* and treated with cetuximab, JQ1, combination (combo) or vehicle (mock) were used for a migration assay. Migration was measured for a total of 24 h immediately after insert removal. **b**–**d** No significant changes in migration was noted when comparing the non-transfected (dashed lines, left) and transfected (full lines, right - NTP) SCC1, SCC6 and SCC25 cells and different treatment groups. **e**–**g** Although migration changes were not observed, there are changes in VIM expression as response to siRNA silencing and the different therapies in all three cell lines.

To investigate the role of cetuximab and JQ1 in the EMT pathway, we performed the scratch assay on SCC1, SCC6 and SCC25 cells treated with both drugs alone or in combination. The cells were seeded in cell migration inserts (Ibidi) and treatment with cetuximab, JQ1, combo or vehicle (mock) 48 h later. Once confluence was reached (72 h after seeding), the insert was removed, and gap closure was measured at 0, 6, 12 and 24 h (Fig. 6a).

Cetuximab treatment resulted in cell migration inhibition in all three cell lines (Fig. 6b–d) when compared with the corresponding untreated cells. The treatment with JQ1 had distinct effects in each of the cell models. Migration of SCC1 with cetuximab, JQ1 or combined therapy did not present any change, and the inhibition effects were the same for all treatment groups when compared with the untreated cells (Fig. 6b). In SCC6, therapy also suppressed migration relative to the absence of treatment. SCC6 cells treated with JQ1 migrate faster than in the presence of cetuximab while the combination therapy reduces migration but not as efficiently as cetuximab monotherapy (Fig. 6c). Although JQ1 was able to reduce SCC1 and SCC6 migration, there was no effect on the migratory abilities of SCC25, and the cells maintain the same rate as untreated cells. Cetuximab had the strongest effect on repressing SCC25 migration and the combination also reduced motility to a lower extent (Fig. 6d).

There is no reference in the literature to a possible interplay between the *TFAP2A* and EMT genes in HNSCC. Since transcriptional factors regulate multiple targets, we also investigated this potential interaction. HNSCC cell lines migration is not impacted by the lack of *TFAP2A* after transfection with siRNA. SCC1, SCC6 and SCC25 transfected with *TFAP2A* siRNA (Fig. 6e–g) present the same migration rates as the non-transfected cell lines (Fig. 6b). The different therapies inhibit migration in a cell type-specific manner (Fig. 6e–g). The scratch assay observations suggest that *TFAP2A* does not directly regulate EMT genes, as silencing of the transcription factor does not affect migration directly. The effects in migration are only associated with cetuximab or JQ1 therapy. The lack of regulation of EMT markers by *TFAP2A* is also noted by the mutually exclusive expression of *TFAP2A* and *VIM* in HNSCC samples from The Cancer Genome Atlas (TCGA) (Supplementary Fig. 4). Also, *EGFR* expression is not present when one of those markers are expressed, suggesting that in patients’ samples these three mechanisms are independently activated by tumour cells. Unfortunately, no assumption regarding possible intrinsic resistance to cetuximab in patients expressing those markers can be made, as there is no information regarding therapy for those patients.

## Discussion

This signalling-based work leads to a novel model of resistance, in which early feedback activating the TFAP2A transcription factor prime cells for resistance through later epigenetic alterations that cause the growth factor receptors regulated by this transcription factor to become re-expressed. It also allowed the confirmation of early rise of changes to the EMT pathway. Using a single-cell and bulk multi-omic approach, we investigated the early responses to cetuximab in HNSCC in vitro models to identify the gene expression and epigenetic mechanisms that are potential drivers of resistance. Treating three HNSCC cell lines for a short period of time, we were able to demonstrate that transcriptional and chromatin rewiring are early events as a response to therapy and that they happen globally and include genes previously described to be involved in resistance to cetuximab. We investigated three HNSCC cell lines (SCC1, SCC6 and SCC25) and their responses to cetuximab in the first few days of therapy.

Approximately 90% of HNSCC present high expression of EGFR protein, and cetuximab seemed to be a reasonable targeted therapy for these tumours.^41^ However, just a small fraction of patients respond to cetuximab, and virtually all responders develop acquired resistance.^42^ To prolong disease control, it is crucial to identify the changes related to resistance while the tumour is still responsive to cetuximab. Currently, there are no biomarkers to predict the drug response, and the mechanisms of resistance are poorly characterised in HNSCC.^40,43^ In a recent time, course study to investigate the transcriptional and DNA methylation signatures driving acquired cetuximab resistance in HNSCC, we found that an essential driver of resistance to anti-EGFR-targeted therapies, *FGFR1*, is epigenetically regulated during chronic exposure to cetuximab and provide strong evidence that epigenetic alterations can drive acquired resistance.^10^

Our scRNA-seq analysis demonstrates that cell lines from the same cancer type present their specific transcriptional signature, with untreated and treated clones clustering separated. Furthermore, heterogeneity measurements and RNA velocity on single cells demonstrate that the response to cetuximab triggers a dynamic process, in which diverse pathways (MSigDB Hallmark pathways) changes in response to EGFR inhibition. These global changes can also be noted in cell fate prediction that demonstrates that treated cells present increased transcriptional activity when compared with untreated controls (RNA velocity direction is from PBS to cetuximab cells).

In addition to single-cell profiling, we further performed a short time course experiment to measure daily the transcriptional changes induced by cetuximab in the three cell lines to verify the cell-specific changes to the TFAP2A targets and EMT genes. Although we did not observe changes in *TFAP2A* and *VIM* in SCC1 at the single-cell level, other genes from these pathways are altered as soon as 24 h after treatment initiation, suggesting that other markers respond with changes in expression to cetuximab. Each cell line presents specific changes to distinct genes from the pathways interrogated. SCC1 and SCC25 present changes after only 24 h of therapy, while in SCC6 those changes are noticed within 96 h of therapy. These results reflect the initial observation in growth rates under cetuximab therapy, where SCC6 presents a resistant-like behaviour with decreased proliferation only after 96 h under cetuximab (Supplementary Fig. 1) or stable slower growth with therapy (Fig. 5c). We have previously observed that anti-EGFR-targeted therapy in vitro is capable of inducing immediate transcriptional changes in the HaCaT keratinocyte cell line model with constitutive *EGFR* activation.^10^ Here, we corroborate this observation by showing that two HNSCC cell lines also present immediate changes to cetuximab and in pathways relevant for resistance. Altogether, this is evidence that adaptive responses to targeted therapies can occur to genes that are involved in driver pathways of resistance and while cancer cells are still sensitive to the therapy.

In another study, we have shown that while SCC25 acquires cetuximab resistance due to chronic exposure,^44^ the transcriptional changes occur a few weeks prior to the promoter hyper- or hypomethylation, with the latter being detected when cells are already resistant. Here, we investigated the hypothesis that some of the genes involved in resistance are controlled by chromatin remodelling that occurs prior to methylation, while the cells are still sensitive to the therapy, and drive a proportion of the expression changes. After 5 days of anti-EGFR blockade, chromatin structure differs between cetuximab and untreated groups in the three HNSCC cell lines as shown by ATAC-seq. We hypothesise that the events that result in acquired resistance go from chromatin changes in the early stages of cetuximab therapy and reflect in transcriptional changes to overcome EGFR inhibition, and that are finally stabilised by gain or loss of methylation. It was previously shown in vitro that *CDKN2A* silencing initially happens through histone modifications leading to loss of gene expression, followed by promoter methylation to lock the repressive state.^45^ Our findings, together with Bachman et al.^45^ suggest that while chromatin rewiring results in gene expression changes, this epigenetic state is still reversible and requires DNA methylation to be maintained and inherited. It is critical to determine the timing in treatment that reversible epigenetic alterations develop to allow alternative therapies to be effective. Short-term exposure to targeted therapies can induce reversible chromatin changes that will lead to resistance, while chronic exposure induces DNA methylation changes that are steadier and more observed in stable resistant states.^46^

*TFAP2A* encodes a transcription factor that binds to growth factor receptors, and is most probably upregulated to overcome the lack of EGFR activity. One proof that this is a potential mechanism of resistance is our previous observation that as a response to anti-EGFR therapy, *TFAP2A* mRNA level is upregulated with only 24 h of therapy initiation in vitro.^12^ The TFAP2A transcription factor has dual-function and can play a role as a tumour suppressor gene (transcriptional repressor) or oncogene (transcriptional activator), depending on the tumour type. Although a previous study showed that in vitro downregulation of *TFAP2A* in HNSCC is associated with decreased proliferation,^47^ another study pointed to the same direction as our findings. In nasopharyngeal carcinoma, *TFAP2A* silencing in vitro and in vivo results in slower cancer cell proliferation and that patients with high tumour levels of the gene present poorer survival compared with those with lower expression.^48^ *TFAP2A* upregulation is a feature of other tumour types, such as neuroblastoma, pancreatic cancer and acute myeloid leukaemia.^49,50,51^ In our in vitro models, *TFAP2A* knockdown resulted in slower cell growth showing the relevance of this transcription factor to HNSCC proliferation in vitro. This finding together with the observation that cetuximab has a synergistic effect is evidence that TFAP2A downstream targets could be new therapeutic markers for combination approaches that will result in prolonged disease control.

The EMT process has also been previously associated with acquired resistance to anti-EGFR-targeted therapies in cells with mesenchymal phenotype.^7,18,41,42^ We found a significant number of EMT gene promoters among those undergoing remodelling after 5 days of therapy in SCC6 and SCC25. Among the EMT genes upregulated by EGFR blockade are a few collagenases, most probably related to providing tumour cells ability to invade the extracellular matrix. One interesting finding is that the gene *AXL* is upregulated after 96 h of cetuximab therapy in the SCC6 cells, and this is also correlated with a more accessible promoter. *AXL* is a receptor tyrosine kinase known to mediate resistance to cetuximab, and is possibly an alternative mechanism HNSCC cells in vitro are activating to keep proliferating under therapy.^40,43,44^ This observation suggests that early chromatin modifications are involved in the development of acquired cetuximab resistance and that they can be detected in the beginning of the treatment.

Since the upregulation of other RTKs, such as *AXL*, is a common finding in acquired anti-EGFR resistance, we tested a combination treatment with cetuximab to evaluate a possible synergistic effect on controlling cell growth more effectively than EGFR-targeted therapy alone. JQ1 is a bromodomain inhibitor that preferentially binds to BRD4, a protein with high affinity for acetylated histone tails, which represses transcription of its targets.^52,53^ Among these target genes are RTKs known to be upregulated as a resistance mechanism to anti-EGFR therapies.^19,54^ In this scenario, BRD4 inhibition seems a reasonable approach by acting as a “multi-targeted” therapy. Also, successful results in delaying acquired cetuximab resistance were shown when JQ1 or BRD4 knockdown were used in combination with cetuximab in HNSCC cell models or patient-derived xenografts.^19^ In our short time course therapeutic model, we could not determine the time of resistance of development, but we observed the inhibitory effects of JQ1 alone or in combination with EGFR blockade. JQ1 has a stronger effect than cetuximab in controlling HNSCC proliferation in vitro, and the addition of cetuximab has diverse impact in reducing cell growth depending on the cell line, with the strongest synergism observed in SCC6 cells. Although including cetuximab to the JQ1 therapy seems to have little effect on reducing proliferation, the combination probably has major impact on disease control by targeting various RTKs at the same time and delays acquired resistance due to reduction of alternative growth pathways tumour cells can use to overcome targeted inhibition. JQ1 is known to have a short half-life reflecting in the necessity of elevated doses that would not be tolerated by cancer patients.^55,56^ Since there are currently other bromodomain inhibitors being evaluated in clinical trials with less toxicity than JQ1, further studies are necessary to identify one which would have a similar effect when combined with cetuximab in HNSCC.

Overall, our study demonstrates that transcriptional and chromatin changes induced by cetuximab therapy are early events that can be detected before acquired resistance develops. Here, we focused on two pathways, TFAP2A and EMT, previously described to be involved in resistance to cetuximab and other anti-EGFR therapies^12,13^ Another major finding is how inter-cell heterogeneity can induce different changes to the same mechanisms of resistance to targeted therapies. Although we observe alterations in both TFAP2A and EMT pathways, the genes affected are different, and in one of the cell lines (SCC1), there is no apparent role of chromatin remodelling in the EMT transcriptional alterations. We demonstrate that two independent mechanisms of resistance present an early onset during the course of cetuximab therapy, suggesting that other mechanisms of resistance could also be deregulated. This observation is relevant since it demonstrates that to overcome resistance acquisition more than one combination therapy would be necessary. Alternatives like JQ1, that targets multiple drivers of resistance, are then promising and would allow the development of clinical trials or clinical decisions to be made without submitting patients to the expensive costs of genetic and genomic tests.

## Supplementary information

Supplementary material

## Data Availability

Unless otherwise specified, all genomics analyses were performed in R. All transcriptional (single cell and bulk) and epigenetic data of the cell lines from this paper have been deposited in GEO (GSE137524, GSE114375 and GSE135604).

## References

[1] Sawyers C (2004). Targeted cancer therapy. Nature.

[2] Hyman, D. M., Taylor, B. S. & Baselga, J. Implementing genome-driven oncology. *Cell* **168**, 584–599 (2017). 10.1016/j.cell.2016.12.015 PMC5463457 28187282

[3] Engelman, J. A., Zejnullahu, K., Mitsudomi, T., Song, Y., Hyland, C., Park, J. O. et al. MET Amplification leads to Gefitinib resistance in lung cancer by activating ERBB3 signaling. *Science* **316**, 1039–1043 (2007). 10.1126/science.1141478 17463250

[4] Hata, A. N., Niederst, M. J., Archibald, H. L., Gomez-Caraballo, M., Siddiqui, F. M., Mulvey, H. E. et al. Tumor cells can follow distinct evolutionary paths to become resistant to epidermal growth factor receptor inhibition. *Nat. Med.* **22**, 262–269 (2016). 10.1038/nm.4040 PMC4900892 26828195

[5] Neel, D. S. & Bivona, T. G. Resistance is futile: overcoming resistance to targeted therapies in lung adenocarcinoma. *npj Precis. Oncol.* http://www.nature.com/articles/s41698-017-0007-0 (2017). 10.1038/s41698-017-0007-0 PMC5687582 29152593

[6] Shaffer, S. M., Dunagin, M. C., Torborg, S. R., Torre, E. A., Emert, B., Krepler, C. et al. Rare cell variability and drug-induced reprogramming as a mode of cancer drug resistance. *Nature* **546**, 431–435 (2017). 10.1038/nature22794 PMC5542814 28607484

[7] Brand, T. M., Iida, M. & Wheeler D. L. Molecular mechanisms of resistance to the EGFR monoclonal antibody cetuximab. *Cancer Biol. Ther.* **11**, 777–792 (2011). 10.4161/cbt.11.9.15050 PMC3100630 21293176

[8] Vincenzi, B., Zoccoli, A., Pantano, F., Venditti, O. & Galluzzo, S. Cetuximab: from bench to bedside. *Curr. Cancer Drug Targets* **10**, 80–95 (2010). 10.2174/156800910790980241 20088790

[9] Grandis, J. R. & Tweardy D. J. Elevated levels of transforming growth factor alpha and epidermal growth factor receptor messenger RNA are early markers of carcinogenesis in head and neck cancer. *Cancer Res*. **53**, 3579–3584 (1993). 8339264

[10] Grandis, J. R., Melhem, M. F., Gooding, W. E., Day, R., Holst, V. A., Wagener, M. M. et al. Levels of TGF-α and EGFR protein in head and neck squamous cell carcinoma and patient survival. *J. Natl Cancer Inst.* **90**, 824–832 (1998). 10.1093/jnci/90.11.824 9625170

[11] Boeckx C, Weyn C, Vanden Bempt I, Deschoolmeester V, Wouters A, Specenier P (2014). Mutation analysis of genes in the EGFR pathway in Head and Neck cancer patients: implications for anti-EGFR treatment response. BMC Res. Notes.

[12] Fertig, E. J., Ozawa, H., Thakar, M., Howard, J. D., Kagohara, L. T., Krigsfeld, G. et al. CoGAPS matrix factorization algorithm identifies transcriptional changes in AP-2alpha target genes in feedback from therapeutic inhibition of the EGFR network. *Oncotarget* **7**, 73845 (2016). 10.18632/oncotarget.12075 PMC5342018 27650546

[13] Schmitz S, Bindea G, Albu RI, Mlecnik B, Machiels J-P (2015). Cetuximab promotes epithelial to mesenchymal transition and cancer associated fibroblasts in patients with head and neck cancer. Oncotarget.

[14] Matys V, Fricke E, Geffers R, Gössling E, Haubrock M, Hehl R (2003). TRANSFAC: transcriptional regulation, from patterns to profiles. Nucleic Acids Res..

[15] Matys V (2006). TRANSFAC(R) and its module TRANSCompel(R): transcriptional gene regulation in eukaryotes. Nucleic Acids Res..

[16] Cheng H, Fertig EJ, Ozawa H, Hatakeyama H, Howard JD, Perez J (2015). Decreased SMAD4 expression is associated with induction of epithelial-to-mesenchymal transition and cetuximab resistance in head and neck squamous cell carcinoma. Cancer Biol. Ther..

[17] Singh A, Settleman J (2010). EMT, cancer stem cells and drug resistance: an emerging axis of evil in the war on cancer. Oncogene.

[18] Fuchs BC, Fujii T, Dorfman JD, Goodwin JM, Zhu AX, Lanuti M (2008). Epithelial-to-mesenchymal transition and integrin-linked kinase mediate sensitivity to epidermal growth factor receptor inhibition in human hepatoma cells. Cancer Res..

[19] Leonard B, Brand TM, O’Keefe RA, Lee ED, Zeng Y, Kemmer JD (2018). BET inhibition overcomes receptor tyrosine kinase-mediated cetuximab resistance in HNSCC. Cancer Res..

[20] Hafner M, Niepel M, Chung M, Sorger PK (2016). Growth rate inhibition metrics correct for confounders in measuring sensitivity to cancer drugs. Nat. Methods.

[21] Afsari B, German D, Fertig EJ (2014). Learning dysregulated pathways in cancers from differential variability analysis. Cancer Inform..

[22] van Dijk D, Sharma R, Nainys J, Yim K, Kathail P, Carr AJ (2018). Recovering gene interactions from single-cell data using data diffusion. Cell.

[23] Bray NL, Pimentel H, Melsted P, Pachter L (2016). Near-optimal probabilistic RNA-seq quantification. Nat. Biotechnol..

[24] Melsted, P., Booeshaghi, A. S., Gao, F., Beltrame, E., Lu, L., Hjorleifsson, K. E. et al. Modular and efficient pre-processing of single-cell RNA-seq [Internet]. *Bioinformatics* http://biorxiv.org/lookup/doi/10.1101/673285 (2019).

[25] Bergen, V., Lange, M., Peidli, S., Wolf, F. A. & Theis F. J. Generalizing RNA velocity to transient cell states through dynamical modeling [Internet]. *Bioinformatics* http://biorxiv.org/lookup/doi/10.1101/820936 (2019). 10.1038/s41587-020-0591-3 32747759

[26] Patro R, Duggal G, Love MI, Irizarry RA, Kingsford C (2017). Salmon provides fast and bias-aware quantification of transcript expression. Nat. Methods.

[27] Soneson C, Love MI, Robinson MD (2015). Differential analyses for RNA-seq: transcript-level estimates improve gene-level inferences. F1000Res.

[28] Buenrostro JD, Giresi PG, Zaba LC, Chang HY, Greenleaf WJ (2013). Transposition of native chromatin for fast and sensitive epigenomic profiling of open chromatin, DNA-binding proteins and nucleosome position. Nat. Methods.

[29] Andrews, S. FastQC: a quality control tool for high throughput sequence data [Internet]. http://www.bioinformatics.babraham.ac.uk/projects/fastqc (2010).

[30] Langmead B, Salzberg SL (2012). Fast gapped-read alignment with Bowtie 2. Nat. Methods.

[31] Wysoker, A., Tibbetts, K. & Fennell, T. Picard Tools Version 2.18. http://broadinstitute.github.io/picard/.

[32] Li H, Handsaker B, Wysoker A, Fennell T, Ruan J, Homer N (2009). The sequence Alignment/Map format and SAMtools. Bioinformatics.

[33] Zhang, Y., Liu, T., Meyer, C. A. & Eeckhoute, J. Model-based analysis of ChIP-Seq (MACS). *Genome Biol*. **9**, R137 [Internet] http://scholar.google.com/scholar?q=related:OjUKu6sM80MJ:scholar.google.com/&hl=en&num=20&as_sdt=0,5 (2008). 10.1186/gb-2008-9-9-r137 PMC2592715 18798982

[34] Ross-Innes, C. S., Stark, R., Teschendorff, A. E., Holmes, K. A., Ali, H. R., Dunning, M. J. et al. Differential oestrogen receptor binding is associated with clinical outcome in breast cancer. *Nature* http://www.nature.com/doifinder/10.1038/nature10730 (2012). 10.1038/nature10730 PMC3272464 22217937

[35] Schneider CA, Rasband WS, Eliceiri KW (2012). NIH Image to ImageJ: 25 years of image analysis. Nat. Methods.

[36] Maseki S, Ijichi K, Nakanishi H, Hasegawa Y, Ogawa T, Murakami S (2013). Efficacy of gemcitabine and cetuximab combination treatment in head and neck squamous cell carcinoma. Mol. Clin. Oncol..

[37] Davis-Marcisak EF, Sherman TD, Orugunta P, Stein-O’Brien GL, Puram SV, Roussos Torres ET (2019). Differential variation analysis enables detection of tumor heterogeneity using single-cell RNA-sequencing data. Cancer Res..

[38] Liberzon A, Birger C, Thorvaldsdóttir H, Ghandi M, Mesirov JP, Tamayo P (2015). The molecular signatures database (MSigDB) hallmark gene set collection. Cell Syst..

[39] La Manno G, Soldatov R, Zeisel A, Braun E, Hochgerner H, Petukhov V (2018). RNA velocity of single cells. Nature.

[40] Byers LA, Diao L, Wang J, Saintigny P, Girard L, Peyton M (2013). An epithelial-mesenchymal transition gene signature predicts resistance to EGFR and PI3K inhibitors and identifies Axl as a therapeutic target for overcoming EGFR inhibitor resistance. Clin. Cancer Res.

[41] Chambers SM, Fasano CA, Papapetrou EP, Tomishima M, Sadelain M, Studer L (2009). Highly efficient neural conversion of human ES and iPS cells by dual inhibition of SMAD signaling. Nat. Biotechnol..

[42] Basu D, Bewley AF, Sperry SM, Montone KT, Gimotty PA, Rasanen K (2013). EGFR inhibition promotes an aggressive invasion pattern mediated by mesenchymal-like tumor cells within squamous cell carcinomas. Mol. Cancer Ther..

[43] Brand TM, Iida M, Stein AP, Corrigan KL, Braverman CM, Luthar N (2014). AXL mediates resistance to cetuximab therapy. Cancer Res..

[44] Stein-O’Brien G, Kagohara LT, Li S, Thakar M, Ranaweera R, Ozawa H (2018). Integrated time course omics analysis distinguishes immediate therapeutic response from acquired resistance. Genome Med..

[45] Bachman KE, Park BH, Rhee I, Rajagopalan H, Herman JG, Baylin SB (2003). Histone modifications and silencing prior to DNA methylation of a tumor suppressor gene. Cancer. Cell.

[46] Brown R, Curry E, Magnani L, Wilhelm-Benartzi CS, Borley J (2014). Poised epigenetic states and acquired drug resistance in cancer. Nat Rev Cancer.

[47] Bennett KL, Romigh T, Eng C, Pazin MJ (2009). AP-2α Induces Epigenetic Silencing of Tumor Suppressive Genes and Microsatellite Instability in Head and Neck Squamous Cell Carcinoma. PLoS ONE.

[48] Shi D, Xie F, Zhang Y, Tian Y, Chen W, Fu L (2014). TFAP2A Regulates Nasopharyngeal Carcinoma Growth and Survival by Targeting HIF-1 Signaling Pathway. Cancer Prev Res..

[49] Carrière C, Mirocha S, Deharvengt S, Gunn JR, Korc M (2011). Aberrant expressions of AP-2α splice variants in pancreatic cancer. Pancreas.

[50] Schulte JH, Kirfel J, Lim S, Schramm A, Friedrichs N, Deubzer HE (2008). Transcription factor AP2alpha (TFAP2a) regulates differentiation and proliferation of neuroblastoma cells. Cancer Lett..

[51] Ding X, Yang Z, Zhou F, Wang F, Li X, Chen C (2013). Transcription factor AP-2α regulates acute myeloid leukemia cell proliferation by influencing Hoxa gene expression. Int J. Biochem Cell Biol..

[52] Filippakopoulos P, Qi J, Picaud S, Shen Y, Smith WB, Fedorov O (2010). Selective inhibition of BET bromodomains. Nature.

[53] Decker T-M, Kluge M, Krebs S, Shah N, Blum H, Friedel CC (2017). Transcriptome analysis of dominant-negative Brd4 mutants identifies Brd4-specific target genes of small molecule inhibitor JQ1. Sci. Rep..

[54] Stratikopoulos EE, Dendy M, Szabolcs M, Khaykin AJ, Lefebvre C, Zhou M-M (2015). Kinase and BET inhibitors together clamp inhibition of PI3K signaling and overcome resistance to therapy. Cancer Cell..

[55] Alqahtani A, Choucair K, Ashraf M, Hammouda DM, Alloghbi A, Khan T (2019). Bromodomain and extra-terminal motif inhibitors: a review of preclinical and clinical advances in cancer therapy. Future Sci. OA.

[56] Andrieu G, Belkina AC, Denis GV (2016). Clinical trials for BET inhibitors run ahead of the science. Drug Disco. Today Technol..

[57] Kagohara, L. T., Zamuner, F., Considine, M., Stein-O’Brien, G., Sherman, T., Gaykalova, D. A. & Fertig, E. J. Abstract 3024: Transcriptional and epigenetic regulation of resistance markers in cetuximab sensitive HNSCC cells. in *Experimental and Molecular Therapeutics* 3024–3024 (American Association for Cancer Research, 2019).

[58] Kagohara, L. T., Zamuner, F., Considine, M., Allen, J., Yegnasubramanian, S., Gaykalova, D. A. & Fertig, E. J. Integrated single cell and bulk multi-omics reveals heterogeneity and early changes in pathways associated with cetuximab resistance in HNSCC sensitive cell lines (Cancer Biology). Preprint at 10.1101/729384 (2019).

