# Correction: Integrated single-cell and bulk gene expression and ATAC-seq reveals heterogeneity and early changes in pathways associated with resistance to cetuximab in HNSCC-sensitive cell lines

**DOI:** 10.1038/s41416-020-0998-0

**Published:** 2020-07-21

**Authors:** Luciane T. Kagohara, Fernando Zamuner, Emily F. Davis-Marcisak, Gaurav Sharma, Michael Considine, Jawara Allen, Srinivasan Yegnasubramanian, Daria A. Gaykalova, Elana J. Fertig

**Affiliations:** 1 Sidney Kimmel Comprehensive Cancer Center, Johns Hopkins University - School of Medicine, Baltimore, MD USA; 2 Department of Otolaryngology-Head and Neck Surgery, Johns Hopkins University - School of Medicine, Baltimore, MD USA; 3 McKusick-Nathans Institute of the Department of Genetic Medicine, Johns Hopkins University - School of Medicine, Baltimore, MD USA; 4 Department of Medicine, Johns Hopkins University - School of Medicine, Baltimore, MD USA

**Keywords:** Cancer therapeutic resistance, Head and neck cancer

Correction to: *British Journal of Cancer* (2020) 123, 101–113;10.1038/s41416-020-0851-5, published online 04 May 2020

The original version of this Article contained an error in Fig. 1b. The figure and legend mislabelled SCC1 as blue and SCC25 as orange, where it should have labelled SCC1 as orange and SCC25 as blue. This has been corrected below. The authors apologise for this oversight.

**Fig. 1:**
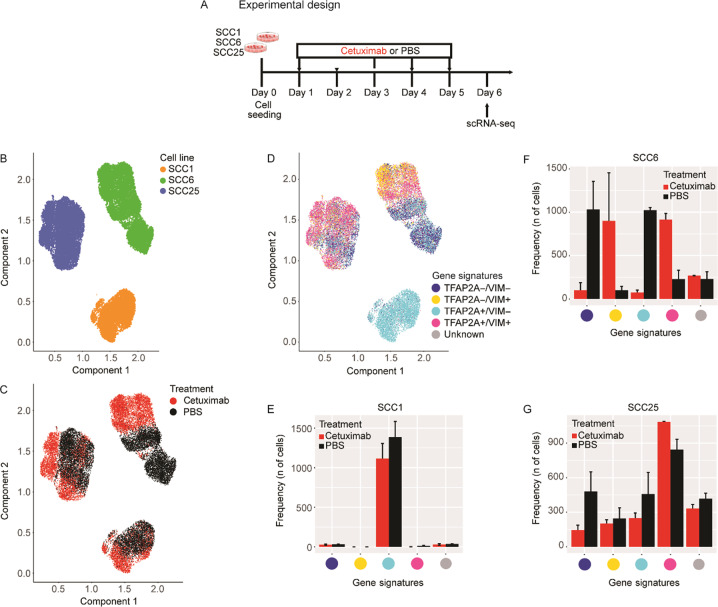
Single-cell RNA-seq profiling of cetuximab-treated and -untreated HNSCC cell lines. **a** SCC1, SCC6 and SCC25 cell lines were treated with cetuximab or PBS (untreated controls) for 5 consecutive days, after which cells were collected for single-cell RNA-seq (scRNA-seq). **b** scRNA-seq analysis demonstrates that each cell line presents a specific gene expression profile. **c** In response to cetuximab, the SCC6-treated (red) and untreated (black) clones separate completely, while the SCC1 and SCC25 present some overlap in the distribution regarding the transcriptional profile. **d** Inter-cell heterogeneity is more evident for TFAP2A and VIM mRNA levels, with SCC1 presenting high levels of TFAP2A and no expression of VIM. The co-expression analysis shows that in SCC1 there is no change in the levels of TFAP2A or VIM in response to cetuximab; SCC6-treated cells are VIM+ (orange and purple), while untreated are negative (green and blue) with different status for TFAP2A expression; and most of the SCC25 cells responding with increase in VIM, but with some untreated clones presenting the same expression profile for VIM and TFAP2A (purple) and with VIM− clones only detected in the untreated group. **e**–**g** Bar plots represent the number of treated and untreated cells per each gene signature.

