# Supplementary material for: Integrated single-cell and bulk gene expression and ATAC-seq reveals heterogeneity and early changes in pathways associated with resistance to cetuximab in HNSCC-sensitive cell lines
Source: Br J Cancer. 2020 May 4;123(1):101–13. doi: 10.1038/s41416-020-0851-5 (PMC7341752; doi:10.1038/s41416-020-0851-5)
Supplement: Supplementary file 1 — Supplementary material [file 41416_2020_851_MOESM1_ESM.pdf]

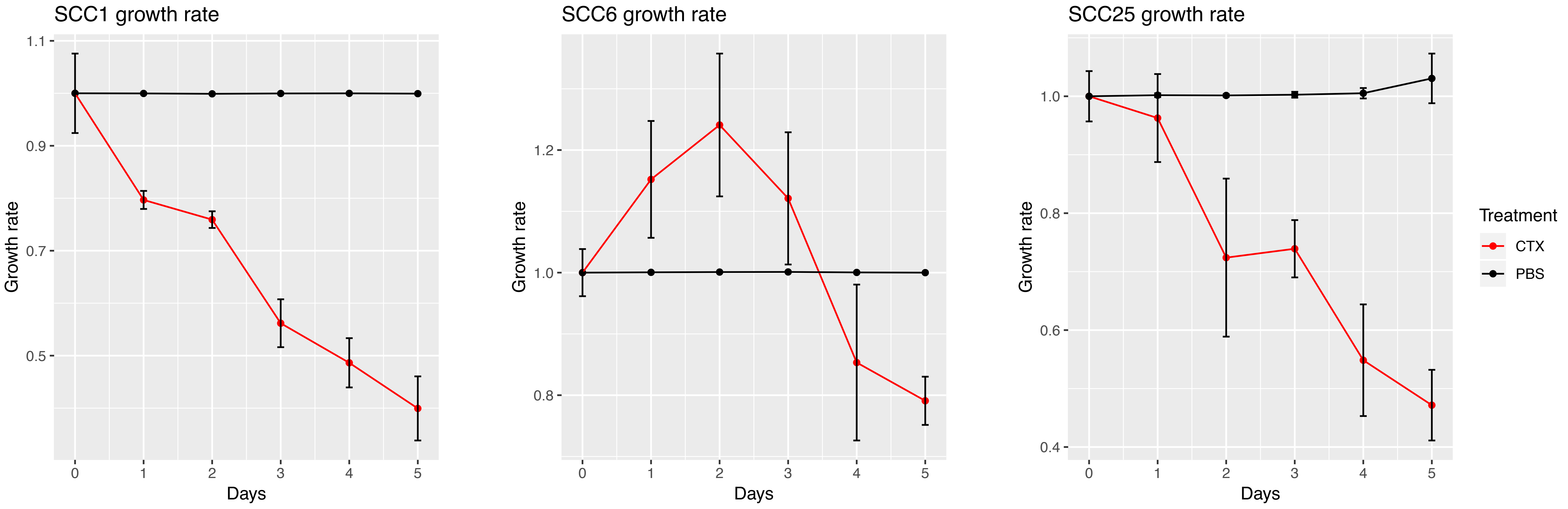

Supplementary Figure 1 - Proliferation assay to verify SCC1, SCC6 and SCC25 sensitivity to cetuximab. HNSCC cells were seeded in 6-well plates and treated with cetuximab or PBS daily for five days. Proliferation was measured every 24 hours using the alamar blue assay. The fluorescence measured was used to determine the growth rate ($GR = 2k^{(c,t)/k(0)} - 1$).

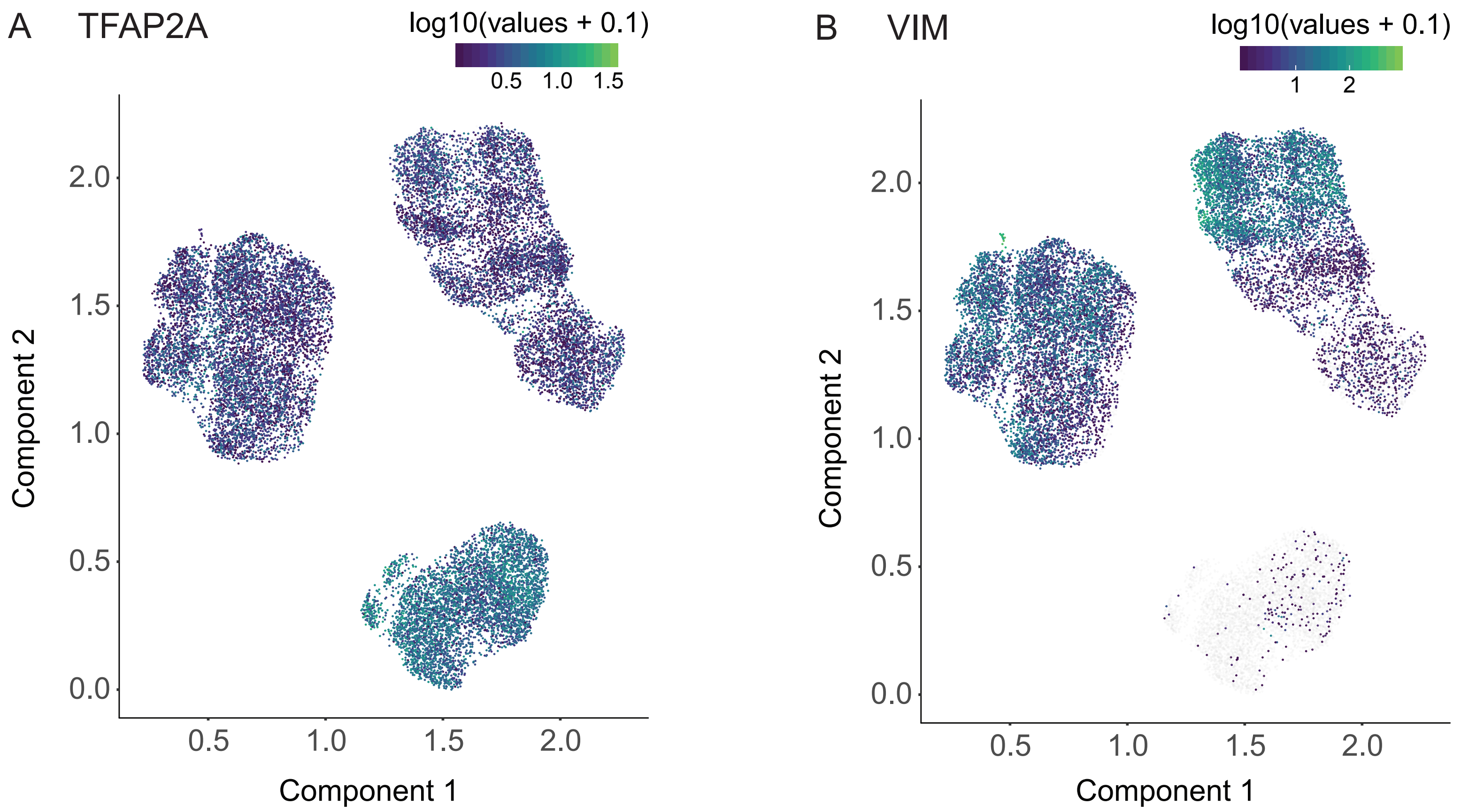

Supplementary Figure 2 - Single cell expression of TFAP2A and VIM in SCC1, SCC6 and SCC25.

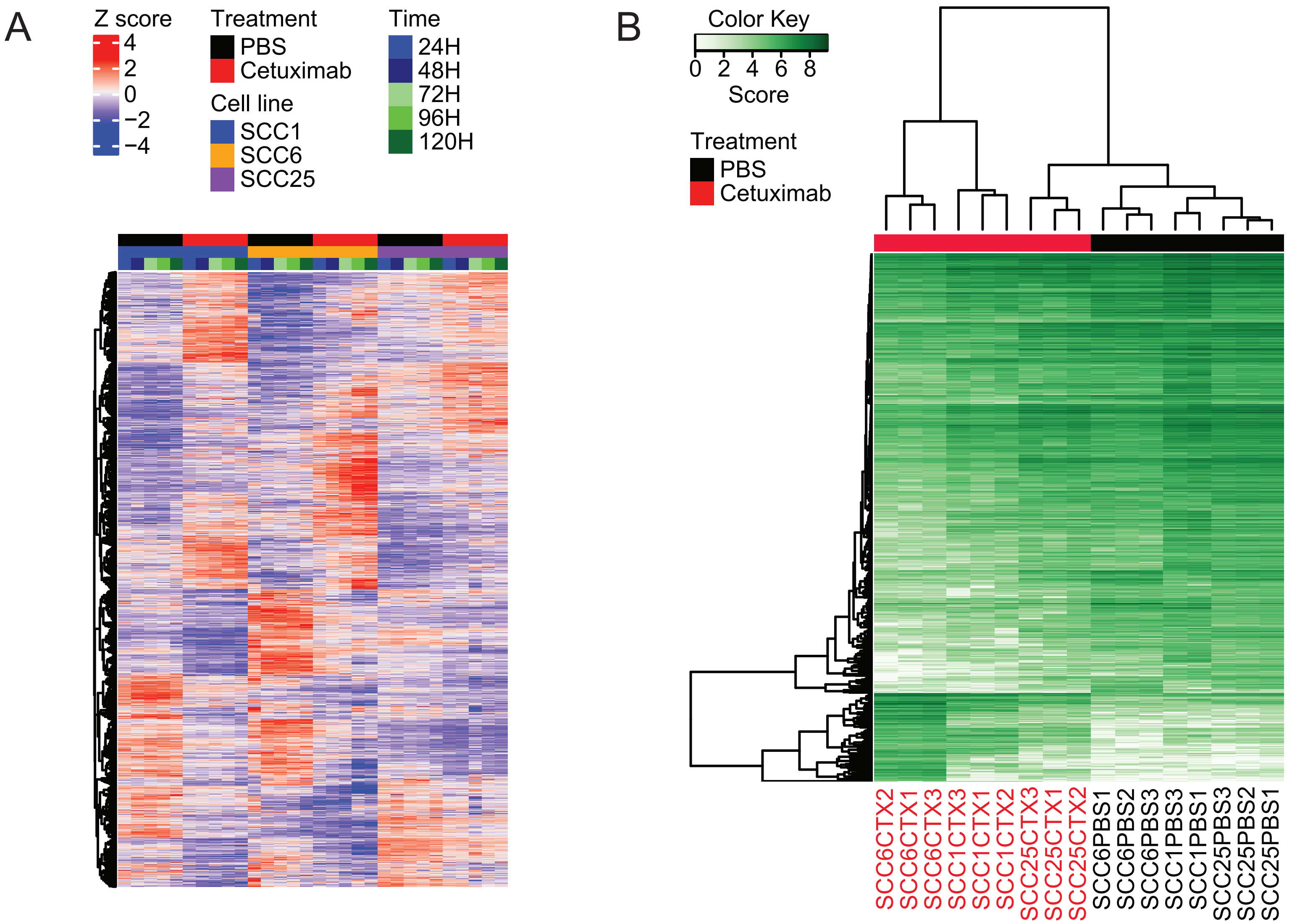

Supplementary Figure 3 - Differential expression analysis (A) and differential binding analysis (B) for all SCC1, SCC6 and SCC25 cell lines. RNA-seq analysis for differentially expressed genes was performed to identify the common genes that change overtime in all three cell lines. Differential binding analysis on the ATAC-seq peaks was also performed in all three cell lines at the same time to identify the common bound sites with accessibility changes in response to cetuximab therapy.

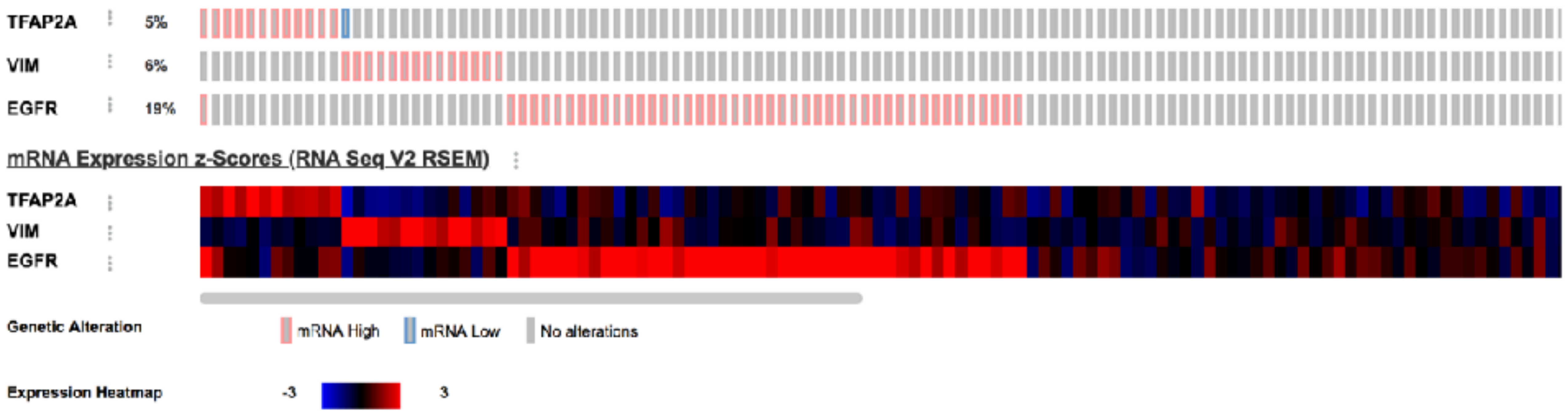

Supplemental Figure 4 - Expression profile of TFAP2A, VIM and EGFR in HNSCC from TCGA. HNSCC prior to treatment do not express the three genes concomitantly.

Supplementary Table 1 - Adjusted p-values from EVA statistics heterogeneity measurements.

MSigDB Pa	adjusted p-values		
	SCC1	SCC6	SCC25
HALLMARK	0.000812	0.490234	0
HALLMARK	0	0	0.003074
HALLMARK	0	0.005829	0.000154
HALLMARK	0	0	0
HALLMARK	0	0	0.483371
HALLMARK	0	0	0
HALLMARK	0.000002	0.05274	0
HALLMARK	0	0.005633	0
HALLMARK	0	0	0.000473
HALLMARK	0	0.19979	0.000018
HALLMARK	0	0	0.000154
HALLMARK	0	0.000065	0
HALLMARK	0	0	0
HALLMARK	0	0	0
HALLMARK	0	0.619539	0
HALLMARK	0	0	0.001882
HALLMARK	0	0.004358	0
HALLMARK	0	0.000008	0.015
HALLMARK	0	0.224466	0
HALLMARK	0	0.000001	0.19858
HALLMARK	0.000003	0	0.446284
HALLMARK	0	0	0.07457
HALLMARK	0.000001	0	0.859886
HALLMARK	0	0	0
HALLMARK	0.149524	0	0
HALLMARK	0.000002	0	0
HALLMARK	0	0	0.000079
HALLMARK	0	0	0
HALLMARK	0	0	0
HALLMARK	0	0.000003	0.000003
HALLMARK	0	0	0
HALLMARK	0	0	0
HALLMARK	0	0	0.264325
HALLMARK	0	0	0
HALLMARK	0	0	0
HALLMARK	0	0	0
HALLMARK	0	0.490234	0
HALLMARK	0	0	0
HALLMARK	0.000006	0	0.613957
HALLMARK	0	0.001074	0.313264
HALLMARK	0	0	0.000005
HALLMARK	0.000001	0.000266	0.000007
HALLMARK	0	0	0

HALLMARK	0	0	0
HALLMARK	0	0	0.41951
HALLMARK	0	0.323679	0.398591
HALLMARK	0	0.490234	0
HALLMARK	0	0.735571	0
HALLMARK	0.000047	0	0.315747
HALLMARK	0	0.128772	0

Highligthed in grey are the non-significant adjusted p-values (>0.05).

Supplementary Table 2 - Differentially expressed genes between cetuximab and PBS across SCC1, SCC6

Genes	log2FoldCh	pvalue
AAAS	0.206396	0.011988
AADAC	0.619799	0.000583
AADACP1	0.577236	0.000743
AAK1	-0.30949	9.08E-05
AAMP	0.28913	4.47E-06
AAR2	0.157147	0.032178
AARS2	0.204389	0.037484
AARSD1	0.268212	5.23E-05
AATBC	-0.87642	0.001856
AATF	0.413164	9.24E-18
ABC7-4240	-0.66022	0.000542
ABCA13	0.314583	0.001919
ABCA5	-0.39625	0.000243
ABCA7	-0.42198	0.023204
ABCB10P4	-0.8964	0.000599
ABCB6	-0.36045	0.032022
ABCC1	0.26157	3.49E-05
ABCC3	-0.31405	0.000533
ABCC4	0.189735	0.000689
ABCC6	-0.59541	0.004841
ABCC9	0.2306	0.032643
ABCE1	0.283118	0.000207
ABCF1	0.179991	0.001446
ABCF2	0.193768	0.001011
ABHD10	0.171474	0.039893
ABHD15	-0.42369	0.000227
ABHD17B	-0.20072	0.002645
ABHD17C	0.22863	0.008926
ABHD2	0.168455	0.010303
ABHD4	-0.40824	6.33E-06
ABHD6	-0.25962	0.021904
ABI2	0.192862	0.043282
ABI3BP	-0.42256	0.000995
ABL2	0.283326	0.022797
ABLIM2	0.214235	0.040311
ABLIM3	-0.17753	0.001968
ABO	-0.21985	0.002927
ABR	0.224153	0.030557
ABT1	0.210547	0.018118
ABTB2	0.370665	0.001686
AC002398.	-0.59001	0.018062
AC002480.	-0.43398	0.01241
AC002550.	-1.0171	0.023409
AC003092.	0.96803	0.021092

AC003991.	-0.81086	0.00551
AC004870.	-0.89696	0.013507
AC005154.	-0.3082	7.99E-05
AC005154.	-1.23527	0.019045
AC005523.	0.275479	0.002497
AC005537.	-0.51236	0.024073
AC005618.	-0.77152	0.003875
AC005682.	0.299162	0.006003
AC006014.	-0.62608	0.025769
AC006116.	-0.76269	0.012543
AC006128.	0.544396	0.049204
AC006372.	-3.02082	0.0337
AC006509.	-1.20554	0.016664
AC007285.	-0.61757	0.021708
AC007326.	-0.44166	0.019819
AC007383.	-0.42678	0.016529
AC007743.	-0.44371	0.005455
AC009502.	0.439453	0.022332
AC009948.	-0.34136	0.000154
AC010731.	-1.36632	0.029667
AC010970.	0.282687	0.002884
AC011286.	-1.3696	0.027086
AC011294.	0.884193	0.044009
AC011330.	-0.38001	0.04293
AC011530.	2.779948	0.035291
AC011738.	-0.69807	0.005078
AC012146.	0.412796	0.017797
AC012501.	-0.81546	0.042896
AC015849.	2.990632	0.024478
AC016745.	-1.27014	0.001558
AC016999.	1.024976	0.039633
AC017116.	-2.75949	0.03162
AC020571.	0.972398	0.043507
AC020956.	-1.46552	0.016534
AC024560.	-0.19001	0.028695
AC024937.	-0.54338	0.034097
AC025171.	-0.5835	0.000185
AC034220.	-0.46933	0.015626
AC053503.	-3.63315	0.007078
AC058791.	0.872763	2.29E-05
AC063976.	-0.87519	0.000194
AC068134.	-0.86106	0.00127
AC068134.	-2.58542	0.00246
AC073115.	-3.50053	0.020821
AC073333.	0.964604	0.028512
AC074183.	0.61887	0.01325
AC074286.	-0.35916	0.015549

AC078883.	1.002262	0.03182
AC079610.	-0.49647	0.029209
AC084809.	-0.88635	0.049713
AC091729.	-0.24589	0.028963
AC092066.	-0.43116	0.00078
AC092641.	1.949835	0.048413
AC093162.	-0.45011	0.017049
AC093732.	-1.35719	0.048584
AC093838.	0.361818	0.000345
AC093899.	-2.91085	0.040519
AC098614.	-0.39436	0.013057
AC104389.	0.724252	0.007562
AC105760.	-0.31525	0.042976
AC108142.	0.420206	0.008414
AC108868.	3.001787	0.024158
AC109333.	1.747231	0.011398
AC113189.	0.467255	0.009554
AC114730.	1.436695	0.006981
AC116366.	-0.65004	0.010873
AC124789.	0.801315	0.001171
AC133785.	0.896389	0.004673
AC135048.	-1.04557	6.85E-06
AC137932.	0.801259	0.005604
AC141586.	0.233142	0.001695
AC145124.	-0.60165	0.014346
AC145343.	-0.64939	0.018961
AC159540.	-0.49071	5.45E-06
ACADM	-0.24643	0.010867
ACADSB	-0.40753	1.04E-05
ACAN	0.930348	0.046279
ACAT2	0.19221	0.019114
ACBD4	-0.62784	0.000118
ACBD5	-0.14244	0.040784
ACCS	-0.49774	6.15E-08
ACD	0.441856	0.006534
ACE2	-0.99496	0.005034
ACIN1	0.163746	0.00067
ACKR3	-1.14047	3.54E-15
ACLY	0.185685	0.000143
ACOT1	-0.44423	1.61E-05
ACOT13	-0.24046	0.000492
ACOT4	-0.45116	0.001692
ACOT7	0.569718	1.90E-07
ACOX1	-0.11313	0.033888
ACOXL	0.628251	0.000182
ACP5	-0.49178	0.007249
ACSBG1	-0.58876	0.022396

ACSF2	-0.25767	0.001876
ACSL1	-0.38828	8.71E-08
ACSL3	-0.15518	0.009999
ACSL5	-0.24208	0.000118
ACTA1	-2.74511	0.003851
ACTA2	-1.19024	8.15E-07
ACTBL2	-0.49912	0.003937
ACTG2	-1.45141	1.37E-12
ACTL6A	0.190204	0.002422
ACTR10	-0.14687	0.019586
ACVR1B	0.162145	0.001402
ACY1	0.303738	0.001976
ACYP2	-0.36367	0.000235
ADAL	-0.22089	0.02118
ADAM12	-0.27395	0.012808
ADAM15	0.411276	0.000685
ADAM19	0.822072	1.69E-09
ADAM23	-0.4737	8.95E-07
ADAM28	-0.42777	0.00128
ADAM32	-0.20754	0.034562
ADAM8	1.309405	7.47E-14
ADAMTS12	0.252782	0.020708
ADAMTS15	-0.2001	0.024363
ADAMTS17	0.358941	0.036027
ADAMTS20	-0.23785	0.044185
ADAMTS6	0.660353	1.63E-09
ADAMTS9	-0.87039	7.29E-08
ADAMTSL1	0.633641	1.15E-05
ADAMTSL3	-0.50389	0.019551
ADAT2	0.254804	0.00889
ADCY1	0.374984	0.001374
ADCY3	0.366982	5.50E-05
ADCY4	-1.00495	0.002516
ADCY5	-0.8921	0.027315
ADCY6	-0.64385	6.41E-08
ADCY7	0.423193	3.08E-05
ADCY9	-0.22085	0.018147
ADD2	0.326256	0.000315
ADGRA2	-1.14287	2.71E-08
ADGRE5	0.308607	0.002669
ADGRF1	0.828952	0.000253
ADGRG6	0.409602	3.79E-05
ADGRL3	-0.54872	0.038537
ADGRV1	-0.46847	0.01411
ADH5	-0.12201	0.033609
ADH7	-2.34565	1.43E-12
ADHFE1	-0.98732	9.36E-07

ADM	-0.46471	0.00026
ADORA2B	0.587395	3.45E-15
ADPRH	-0.61478	0.000799
ADRA1B	0.602201	2.07E-06
ADRA1D	1.420734	0.000202
ADRB1	0.543083	0.002199
ADRB2	0.238181	0.010894
ADRM1	0.477278	0.001063
ADSL	0.244867	0.000548
ADSSL1	-0.44754	0.000147
ADTRP	0.442134	0.001624
AEBP1	0.851407	2.61E-07
AEN	0.557023	1.11E-16
AF003625.:	0.889021	1.67E-06
AF131217.:	1.263499	0.016876
AFAP1	0.347676	4.48E-05
AFAP1-AS1	0.164719	0.046682
AFAP1L1	0.349537	8.36E-05
AFF1	-0.26601	0.008341
AFG3L2	0.098302	0.014275
AGA	-0.36477	0.001354
AGAP10P	-0.79703	0.006633
AGBL5	0.190712	0.044837
AGGF1P1	-2.21318	0.015008
AGK	0.174442	0.013215
AGL	-0.28316	4.04E-05
AGMAT	0.503766	3.99E-06
AGO2	0.431734	2.85E-11
AGO4	-0.60574	1.33E-11
AGPAT3	-0.44182	0.000113
AGPAT4	-0.21155	0.000895
AGPAT5	0.362048	9.88E-06
AGPS	-0.18877	0.026456
AGR2	0.842853	5.22E-06
AHCTF1	0.233426	0.001575
AHCY	0.402688	1.99E-06
AHCYL2	-0.38046	7.91E-05
AHSA1	0.280527	1.53E-05
AIF1L	0.345331	1.39E-06
AIFM3	-0.74703	0.004957
AIM1	0.163075	0.003813
AIMP2	0.29582	0.00019
AIP	0.201008	0.039561
AJAP1	0.976149	3.47E-11
AK2	0.289244	2.64E-07
AK4	0.120785	0.033952
AKAP1	0.318198	1.15E-12

AKAP12	1.211928	4.15E-45
AKAP2	0.413284	6.17E-12
AKAP6	-0.60698	9.57E-05
AKAP8L	0.172693	0.03536
AKAP9	-0.27853	0.019767
AKR1B1	0.213174	0.009724
AKR1B10	-0.61024	0.02455
AKR1B15	-0.47376	0.037807
AKR1C2	-0.78658	0.00012
AKR1C3	-0.65716	0.000123
AKR7A3	0.59156	0.04812
AKT2	0.24962	0.016287
AKT3	-0.17868	0.049564
AKTIP	-0.37946	0.000135
AL022344.[illegible]	-0.68116	0.005522
AL050303.[illegible]	-2.65316	0.017359
ALAD	-0.24094	0.000864
ALDH18A1	0.099126	0.018053
ALDH1B1	-0.39432	1.57E-09
ALDH3A1	-1.15097	5.49E-07
ALDH3A2	-0.17389	0.028915
ALDH3B1	-0.42204	0.012697
ALDH3B2	-2.33628	2.81E-05
ALDH5A1	-0.56877	8.36E-09
ALDH6A1	-0.30255	0.001089
ALDH7A1	-0.17379	0.004531
ALG1	0.264863	0.010669
ALG3	0.274164	0.013267
ALKBH2	0.185961	0.048946
ALKBH5	0.121251	0.047949
ALOX12	0.792294	1.49E-11
ALOXE3	1.072208	2.41E-09
ALPK1	-0.58599	1.94E-10
ALPP	-0.98296	0.000647
ALX4	-0.39567	0.023348
ALYREF	0.341379	0.001418
AMACR	-0.32656	7.29E-06
AMBRA1	0.180816	0.011469
AMIGO1	-0.48519	0.001427
AMMECR1	0.303374	1.84E-05
AMOT	-1.22747	0.001918
AMOTL1	0.27557	5.69E-09
AMOTL2	0.224637	0.020857
AMPD2	0.357144	0.001587
AMTN	0.284755	0.008609
ANAPC1	0.154708	0.008082
ANAPC5	0.104213	0.021097

ANAPC7	0.139639	0.004402
ANG	-0.34015	0.011028
ANGEL1	0.140885	0.024042
ANGPT1	-0.86808	4.15E-06
ANGPT2	1.065721	0.000786
ANGPTL2	0.966293	9.90E-05
ANGPTL4	0.545135	7.87E-05
ANGPTL7	-2.06744	0.024992
ANGPTL8	1.69043	0.034662
ANK3	-0.32535	0.00055
ANKEF1	-0.33229	1.91E-06
ANKH	-0.17768	0.036671
ANKMY1	0.268069	0.004498
ANKMY2	-0.19821	0.00733
ANKRA2	-0.2687	0.023586
ANKRD10	0.462312	1.60E-07
ANKRD10-I	0.537119	3.77E-06
ANKRD11	0.215729	0.032416
ANKRD13A	-0.1605	0.047331
ANKRD13B	0.533316	0.000252
ANKRD13C	-0.13225	0.039055
ANKRD18C	-1.18522	0.009259
ANKRD20A	-0.95364	3.91E-06
ANKRD22	0.6968	0.047561
ANKRD29	0.17098	0.037958
ANKRD33B	0.421994	4.94E-05
ANKRD34B	-1.8458	0.009926
ANKRD65	-0.67908	0.002512
ANKRD9	-0.48694	0.000824
ANKS6	0.355118	2.28E-06
ANKZF1	0.15003	0.019393
ANLN	0.219538	0.003484
ANO1	0.799759	4.26E-15
ANO1-AS2	1.872609	0.00473
ANO7	0.526755	0.033795
ANOS1	-0.69676	6.61E-09
ANP32A	0.178834	0.032135
ANP32B	0.233902	0.003925
ANPEP	0.79455	2.04E-13
ANTXR1	-0.36065	1.80E-12
ANTXR2	0.767898	3.28E-08
ANXA1	0.170413	0.019845
ANXA10	1.720885	2.39E-14
ANXA13	-1.22572	0.000371
ANXA3	-0.51047	2.04E-09
ANXA4	-0.23082	0.006391
ANXA5	0.289891	4.57E-05

ANXA6	0.440996	9.71E-05
ANXA8	-0.16202	0.037298
ANXA8L1	-0.18505	0.006094
ANXA9	-0.47582	0.00491
AOX1	0.306074	0.004419
AP000275.	-3.12442	0.013272
AP000350.	-1.62877	0.031601
AP000350.	-0.80977	0.000496
AP000593.	1.106718	0.000334
AP000695.	-0.39268	0.019897
AP000892.	-0.93587	1.40E-08
AP001258.	-0.41458	0.003858
AP001372.	-0.39559	0.0108
AP002954.	-0.70844	0.019777
AP003068.	-0.27658	0.015162
AP003068.	0.809458	0.029252
AP004782.	0.844377	0.033169
AP1M2	-0.19986	0.021333
AP1S2	0.221962	0.023624
AP2B1	0.105758	0.018998
AP2S1	0.244684	0.001004
AP3B2	-0.50929	1.38E-07
AP4B1	0.156117	0.015851
AP4M1	0.247438	0.008804
AP5M1	-0.29172	8.75E-05
APBA2	0.56048	0.005914
APCDD1	-1.69581	0.000359
APCDD1L	0.338107	0.020219
APCDD1L-A	0.344808	0.002547
APEH	0.171097	0.045215
APEX1	0.228796	0.000288
APEX2	0.326038	0.000499
APH1B	-0.36666	0.001207
APIP	0.188202	0.030941
APITD1-CO	0.616881	0.003284
APLF	-0.31055	0.014122
APLN	0.526609	0.000188
APLP1	-0.44306	0.030362
APOB	-1.30499	0.021509
APOBEC3B	0.424554	0.000858
APOBEC3D	-0.32077	0.004994
APOBEC3F	-0.31863	0.000921
APOBR	-1.29443	0.00919
APOE	-0.48108	0.028244
APOL2	-0.39024	0.007932
APOL3	-0.43857	0.000333
APOL6	-0.37981	0.030762

APOM	-0.33012	0.029192
APOO	0.202058	0.023452
APOOL	-0.20525	0.009109
APOPT1	0.115502	0.024281
APP	-0.17378	0.013643
APPBP2	-0.30108	0.0002
APRT	0.378589	0.013738
AQP1	0.467016	0.015826
AQP10	-0.44581	0.047737
AQP11	-0.49756	0.041621
AQP7	-0.59295	0.014225
ARAF	0.236622	0.037336
ARAP1-AS2	0.725963	0.026441
ARAP3	0.511264	8.27E-07
ARC	0.985949	1.98E-06
AREG	1.121364	5.09E-31
ARF1	0.203444	0.019623
ARF6	0.123371	0.010175
ARFGAP2	0.091439	0.032971
ARFIP2	0.127481	0.027496
ARFRP1	0.343096	0.000743
ARG2	0.85027	1.70E-14
ARHGAP11	0.260198	0.020252
ARHGAP17	0.145971	0.018644
ARHGAP18	0.412463	0.000587
ARHGAP19	0.286904	0.002501
ARHGAP22	-0.30702	0.018172
ARHGAP23	0.369108	0.006961
ARHGAP24	0.244043	0.00788
ARHGAP25	0.500111	0.000184
ARHGAP26	-0.53371	1.53E-08
ARHGAP27	-0.37627	0.010475
ARHGAP29	0.460688	6.57E-08
ARHGAP5	-0.2816	0.008245
ARHGDIA	0.321311	0.043925
ARHGDIB	0.24408	0.004515
ARHGEF1	0.524666	7.98E-05
ARHGEF10I	-0.70405	4.19E-06
ARHGEF18	0.281807	0.046667
ARHGEF2	0.605296	1.39E-08
ARHGEF34I	-0.20254	0.014751
ARHGEF37	-0.79658	0.000289
ARHGEF6	-0.22838	0.036032
ARID3B	0.822731	4.84E-14
ARID4A	-0.25778	0.010991
ARID5A	0.304313	0.038667
ARID5B	-0.21946	0.049575

ARIH2	0.122138	0.012292
ARL11	-0.65315	0.001949
ARL14	0.425762	0.01009
ARL15	-0.32271	0.024371
ARL2	0.427454	0.001363
ARL6IP5	-0.32178	6.13E-11
ARL9	0.402034	0.027202
ARMC6	0.437487	0.003269
ARMCX3	-0.3364	2.60E-06
ARMCX4	-0.27683	0.00889
ARNTL	0.395238	1.80E-05
ARNTL2	0.28635	0.000423
ARPC1A	0.109314	0.021477
ARPC1B	0.453461	0.000969
ARPC5	-0.26992	1.18E-05
ARPIN	-0.37508	1.23E-07
ARRDC4	0.319032	0.011089
ARSB	-0.17354	0.019483
ARSD	-0.58016	1.08E-09
ARSG	-0.62088	1.01E-05
ARSJ	0.161681	0.041817
ARV1	-0.274	0.000524
AS3MT	-0.43545	0.012187
ASAH1	-0.64785	1.41E-14
ASAP1	0.255868	5.78E-06
ASAP3	-0.17302	0.046188
ASB1	0.396443	9.94E-10
ASB6	0.264184	0.005884
ASF1B	0.260456	0.022787
ASH1L	-0.16478	0.008839
ASIC2	0.824579	0.008193
ASL	0.220575	0.047208
ASPH	-0.13003	0.032474
ASRGL1	0.67699	0.001006
ASS1	-0.38562	0.048251
ASTL	1.636589	0.047011
ASXL1	0.200409	0.000144
ATAD2	0.16149	0.010949
ATAD2B	-0.1903	0.008687
ATAD3A	0.548576	0.001723
ATAD3B	0.579987	4.05E-05
ATAD5	0.185413	0.02144
ATF1	0.217671	0.005246
ATF4	0.125953	0.043217
ATF7IP2	-0.45165	0.000478
ATG10	-0.24801	0.022464
ATG101	0.281288	0.008405

ATG16L1	0.23961	0.00015
ATG2B	-0.22344	0.00233
ATG4A	-0.1963	0.037699
ATIC	0.343868	2.25E-12
ATL1	-0.86856	3.19E-07
ATL2	0.217451	0.006017
ATL3	-0.13562	0.046547
ATOH8	-1.65898	1.34E-05
ATP11C	0.171048	0.031245
ATP13A5	-1.82208	0.004702
ATP1A3	0.926323	0.025221
ATP1B1	-0.32987	2.71E-11
ATP1B3	-0.17623	0.0044
ATP2A2	-0.16237	0.001123
ATP5A1	0.124233	0.000303
ATP5B	0.158582	0.000443
ATP5F1	0.121169	0.028142
ATP5G1P6	1.040817	0.016262
ATP5G2	0.195681	0.009119
ATP5J2-PT(	0.314516	0.027282
ATP5L2	2.162291	0.013217
ATP6AP1L	-0.65814	0.034378
ATP6AP2	-0.1923	0.009313
ATP6V0A1	-0.3487	3.09E-07
ATP6V0B	0.222546	0.022118
ATP6V0D2	-0.39398	0.000986
ATP6V1A	-0.38413	2.79E-06
ATP6V1B1-	0.550211	0.03182
ATP6V1C2	-0.44267	0.009688
ATP6V1E1	-0.22612	0.000779
ATP7A	-0.3737	5.84E-05
ATP7B	-0.29212	0.044112
ATP8B1	-0.43007	2.45E-06
ATP9A	-0.16543	0.047585
ATRIP	0.312097	0.010091
ATRN	-0.25886	4.71E-09
ATXN1	-0.32017	6.61E-09
ATXN10	-0.16289	0.00022
ATXN1L	-0.09435	0.030431
ATXN2L	0.217598	0.0256
ATXN7L2	0.504801	0.000376
ATXN7L3	0.154237	0.023218
AUNIP	0.253808	0.000379
AUP1	0.225168	0.008081
AURKA	0.370178	0.001246
AURKAPS1	1.427833	0.011602
AURKB	0.521576	3.56E-06

AUTS2	-0.20245	0.012993
AVPI1	0.352683	0.018969
AXDND1	-0.83483	0.000895
AXIN1	0.401618	0.00268
AXIN2	-1.18078	0.000533
AXL	0.62153	1.06E-06
AZGP1	-2.15219	0.032326
B2M	-0.35611	0.00151
B3GALT4	-0.36669	0.029162
B3GLCT	-0.3044	1.63E-05
B3GNT2	-0.26003	0.0054
B3GNT3	0.638515	1.26E-07
B4GALNT2	-0.95163	1.40E-09
B4GALNT3	0.426388	0.000237
B4GALT1	-0.13139	0.008761
B4GALT2	0.285353	0.03765
B4GALT3	0.256849	0.000258
B4GALT4	-0.17468	0.022943
B9D1	0.24737	0.00518
BACE1	-0.13905	0.027539
BAG6	0.342106	0.00022
BAIAP2L2	0.845597	0.000128
BAIAP3	0.658622	0.009367
BAK1	0.201424	0.003327
BAMBI	0.348143	0.036943
BANF1	0.296669	0.000125
BANK1	-0.79018	0.002271
BAP1	0.21232	0.033598
BATF3	0.828235	0.004774
BAX	0.400908	0.000126
BAZ2B	-0.31346	0.008778
BBOF1	-0.24051	0.025727
BBOX1	-1.93257	0.001267
BBS2	-0.20831	0.001342
BBS5	-0.18163	0.015885
BCAP31	0.140524	0.011084
BCAR1	0.423054	0.044139
BCAR3	0.53943	5.03E-08
BCAS3	-0.46269	6.50E-09
BCAT1	0.345531	0.000589
BCCIP	0.305594	4.11E-05
BCDIN3D	-0.24402	0.00606
BCDIN3D-A	-0.51272	0.028458
BCHE	-0.41843	8.07E-05
BCKDHB	-0.35275	1.15E-06
BCL10	0.495036	1.61E-06
BCL2	0.728646	1.78E-05

BCL2L1	0.305122	0.000539
BCL2L10	-0.42148	0.043354
BCL2L12	0.402787	0.00695
BCL2L14	-1.08287	0.000587
BCL2L2-PAI	0.559288	0.003169
BCL7A	-0.20062	0.042118
BCO1	-1.05573	0.040191
BCR	0.225893	0.02547
BCS1L	0.486639	1.70E-12
BDH2	-0.38005	2.30E-07
BEND3	0.266124	0.036372
BEND3P3	0.327078	0.020215
BEND6	-0.35243	2.01E-05
BEND7	-0.2735	0.020618
BEST1	-0.21426	0.022907
BFSP1	-0.23943	0.01242
BHLHB9	-0.23067	0.025775
BHLHE40	0.284575	0.02871
BHLHE41	-0.58704	8.93E-13
BICDL2	-0.67346	0.020952
BIK	0.647148	0.000296
BIN1	0.270246	0.038863
BIN3	0.181018	0.042731
BIRC5	0.334617	0.000178
BISPR	-0.46212	0.004251
BLM	0.360049	0.000194
BLMH	0.203539	0.001739
BLOC1S6	-0.15061	0.03736
BLVRA	-0.22668	0.013369
BLZF2P	-1.72166	0.026417
BMP6	1.27916	0.000187
BMPR1A	-0.16105	0.033276
BMPR1B	0.263103	0.031767
BMPR2	-0.32199	6.85E-05
BMS1	0.14761	0.032419
BMS1P10	0.398868	0.038739
BMT2	-0.34622	0.004887
BNC1	0.244237	8.16E-07
BNIP1	0.251452	0.002331
BNIP3L	-0.1796	0.023748
BOD1	0.226523	0.00014
BOLA1	0.302425	0.04857
BOLA2	0.406448	8.76E-05
BOLA2B	0.455722	0.000497
BOLA3	0.285429	2.20E-05
BOP1	0.689501	2.98E-05
BORA	0.208512	0.005531

BORCS7	-0.34051	2.23E-05
BPHL	-0.20244	0.00305
BPTF	-0.18567	0.005459
BRCA2	0.237502	0.00025
BRD4	0.239944	0.005738
BRD9	0.220831	0.005681
BRI3BP	0.159169	0.031304
BRIX1	0.36047	2.33E-09
BRPF1	0.258014	0.00459
BRWD1	-0.24495	0.035851
BSDC1	-0.14574	0.033275
BTBD16	-1.16905	0.046884
BTBD19	0.328671	0.026684
BTBD2	0.365687	0.045021
BTBD6	0.198544	0.011844
BTBD7	-0.16318	0.012897
BTBD8	-0.63391	7.31E-06
BTBD9	-0.3	0.004967
BTC	-0.61461	0.009464
BTF3	0.135914	0.015947
BTF3P2	2.483348	1.12E-08
BTG2	-0.32388	1.28E-05
BTN2A2	-0.1904	0.01606
BTN3A1	-0.27273	0.002109
BTN3A2	-0.22986	0.004657
BTN3A3	-0.40073	0.000112
BUB1	0.409931	9.34E-05
BUB1B	0.393731	0.001661
BUB3	0.230101	7.81E-05
BUD13	0.188322	0.002265
BVES	-0.35763	6.35E-07
BX322557.	-0.31779	0.004524
BYSL	0.375377	1.18E-08
BZW1	0.221627	0.00637
BZW1P2	0.467293	7.98E-10
BZW2	0.339595	3.16E-13
C10orf67	-0.39974	0.041666
C10orf99	-1.54594	0.025198
C11orf52	-0.93964	1.27E-06
C11orf54	-0.25005	0.027757
C11orf63	-0.25644	0.004235
C11orf68	0.465126	0.000286
C11orf80	-0.28772	0.009333
C11orf84	0.285847	0.034748
C11orf88	-1.12605	0.00093
C11orf98	0.353341	9.07E-06
C12orf10	0.336484	0.003702

C12orf43	0.192871	0.012973
C12orf49	-0.13243	0.00811
C12orf50	-0.3717	0.003709
C12orf54	-0.46815	0.01004
C12orf56	-0.54555	0.025331
C14orf132	0.241219	0.011577
C14orf166	0.16902	0.002498
C14orf28	-0.48296	4.81E-05
C14orf37	-0.35209	0.023904
C15orf38-A	0.230223	0.044053
C15orf39	0.344781	0.022238
C15orf48	-0.93094	0.02487
C15orf52	-0.28698	0.000766
C15orf59	-0.88829	0.000151
C16orf45	0.273244	0.012768
C16orf52	0.395574	0.000836
C16orf62	-0.16775	0.001207
C16orf74	0.492262	0.000263
C16orf91	0.297465	0.019703
C17orf49	0.368687	0.006499
C17orf51	0.472284	3.27E-08
C17orf53	0.387962	0.002049
C18orf32	-0.16816	0.04056
C19orf24	0.495302	0.022364
C19orf25	0.357093	0.008921
C19orf33	0.418166	0.002165
C19orf48	0.527318	6.95E-06
C19orf53	0.263759	0.002906
C19orf66	-0.39799	0.021338
C1GALT1C1	-0.17639	0.018481
C1orf109	0.211775	0.031892
C1orf112	0.198113	0.01663
C1orf132	-0.6909	8.35E-06
C1orf186	-0.52419	8.11E-06
C1orf198	-0.26566	3.66E-05
C1orf21	-0.49043	5.46E-06
C1orf226	0.264028	0.006218
C1orf43	0.11682	0.011528
C1orf61	0.593782	0.031275
C1orf74	-0.14169	0.020809
C1orf94	1.266317	0.02552
C1QBP	0.458609	2.78E-09
C1QTNF2	-1.16027	0.008409
C1QTNF3	-0.94154	0.012638
C1QTNF3-A	1.828971	0.034487
C1R	-0.97035	1.69E-10
C1RL	-0.64202	5.00E-06

C1S	-1.09885	2.07E-17
C2	-0.28359	0.049949
C20orf194	-0.40545	1.07E-08
C20orf197	0.71967	0.000237
C20orf27	0.613341	0.000818
C21orf59	0.208954	0.003302
C21orf91	-0.23798	0.023612
C22orf23	-0.54831	0.004577
C22orf34	1.70928	0.008257
C2orf27A	-0.36399	0.024025
C2orf54	-1.19035	0.047529
C2orf70	1.300722	0.000949
C3	-1.40036	1.40E-06
C3orf52	0.207658	0.015747
C3orf62	-0.21193	0.021415
C3orf67	0.199207	0.037555
C4BPAP1	3.466297	0.026534
C4orf3	-0.17156	0.01645
C4orf33	-0.44838	2.46E-06
C4orf36	-0.35022	0.004624
C4orf47	-0.75764	0.017256
C5AR2	0.399548	0.048971
C5orf15	-0.24638	0.001245
C5orf17	-0.53669	0.035698
C5orf30	0.373175	0.000187
C5orf42	-0.31405	0.002708
C5orf56	-0.471	0.007719
C5orf63	-0.40081	0.000198
C5orf66	0.554217	0.002081
C6orf1	0.558617	9.40E-05
C6orf10	-1.11184	0.000953
C6orf136	0.292208	0.021681
C6orf141	-0.30632	0.036965
C6orf201	-0.99866	0.009604
C6orf62	-0.1846	0.001226
C6orf89	-0.19482	0.000173
C6orf99	0.662758	0.024335
C7orf26	0.262765	0.007084
C7orf31	-0.35814	0.001621
C7orf57	0.311582	0.008848
C7orf65	-1.33564	0.007874
C8orf31	-0.98807	0.00488
C8orf33	0.278873	4.72E-06
C8orf4	-0.64495	0.029512
C8orf44	-0.28766	0.034669
C8orf46	-0.72498	0.02778
C8orf48	-0.35951	0.013481

C9orf142	0.448391	0.031956
C9orf3	-0.34286	1.62E-05
C9orf47	0.621903	0.039202
C9orf78	0.151719	0.002218
CA11	-0.64685	0.000162
CA3-AS1	-1.11127	0.00339
CAAP1	-0.1695	0.046639
CACFD1	-0.47473	0.01031
CACNA1A	-0.36239	0.004329
CACNA1H	1.227809	6.75E-06
CACNG4	-1.33204	2.50E-19
CACTIN	0.399456	0.02766
CACTIN-AS	1.034563	0.039399
CACYBP	0.25058	0.001507
CAD	0.390237	2.46E-06
CADM1	0.441589	0.000492
CADPS2	-0.56645	0.000462
CALB1	0.641071	1.63E-12
CALCOCO1	-0.37808	0.000423
CALCOCO2	-0.21023	0.007787
CALD1	-0.79128	9.10E-24
CALHM2	0.668384	1.02E-05
CALHM3	0.899781	0.002148
CALM3	0.190119	0.031544
CALU	-0.14059	0.038429
CAMK1D	-0.80025	3.25E-08
CAMK2D	0.167173	0.015173
CAMK2N1	0.266881	0.005094
CAMK4	0.841493	2.79E-05
CAMLG	0.163527	0.0092
CAMSAP1	0.154122	0.005459
CANT1	0.206496	0.024374
CAP2	-0.15227	0.044514
CAPN10	0.299858	0.036912
CAPN13	-2.35096	0.002318
CAPN14	-0.55609	0.047851
CAPN3	-0.60509	0.004943
CAPRIN2	0.556025	1.93E-09
CAPS2	-0.40704	0.003526
CARD10	0.510448	0.002845
CARD11	0.540032	3.24E-06
CARD16	-0.45862	2.08E-05
CARD17	-1.51814	5.21E-06
CARD6	-0.26777	0.014919
CARD8	-0.25008	4.23E-05
CARF	-0.55183	2.07E-05
CARM1	0.447032	4.19E-05

CARMIL2	0.657405	0.038339
CARMIL3	-1.08023	0.005172
CARMN	-0.96452	1.93E-08
CARNMT1	0.2951	0.000774
CARNS1	-0.58181	0.020985
CASC8	0.834208	0.000191
CASC9	-0.25714	0.018587
CASP10	-0.41005	0.0004
CASP14	-1.24647	0.001224
CASP1P2	-0.47368	0.039803
CASP6	-0.28472	0.00092
CASP7	-0.34467	0.000228
CASP9	-0.32788	0.011039
CASQ2	-0.69516	0.001635
CAT	-0.33996	5.79E-08
CATSPER2	-0.39849	0.006723
CAV1	0.178044	0.031133
CBFA2T2	-0.29733	2.85E-05
CBFA2T3	-0.63748	0.003742
CBFB	0.220793	0.003519
CBL	0.19481	0.013516
CBLN3	-0.45293	0.006784
CBR1	-0.24301	0.033218
CBR3-AS1	-0.67219	0.000352
CC2D2A	0.232245	0.010697
CCAR2	0.261344	0.001922
CCAT1	0.607586	0.017501
CCDC102B	-0.40407	0.01463
CCDC103	-0.74142	0.004611
CCDC115	-0.1694	0.020939
CCDC12	0.19999	0.032524
CCDC122	-0.22367	0.043427
CCDC124	0.364713	0.047983
CCDC126	-0.41347	0.00015
CCDC138	0.248091	0.002568
CCDC144A	-0.41438	0.000936
CCDC144B	-0.57549	0.000843
CCDC144CI	-0.77312	1.65E-06
CCDC144N	-0.6518	0.018085
CCDC146	-0.96295	1.62E-09
CCDC149	-0.47888	0.00053
CCDC150	0.365163	0.004405
CCDC162P	-0.27521	0.018258
CCDC173	-1.09902	0.013991
CCDC186	-0.25842	0.012951
CCDC189	0.48548	0.016979
CCDC18-AS	-0.41482	1.84E-05

CCDC191	-0.50857	0.010585
CCDC22	0.286997	0.020027
CCDC3	0.578272	1.12E-07
CCDC34	0.229625	0.049724
CCDC39	-0.45911	0.0057
CCDC50	-0.19263	0.014809
CCDC57	0.230677	0.03861
CCDC58	0.285145	0.001484
CCDC59	0.212857	0.011896
CCDC69	1.088982	3.85E-15
CCDC71L	0.426114	0.000721
CCDC74B	0.681594	0.030714
CCDC80	-0.8874	1.27E-07
CCDC85C	0.435469	1.33E-07
CCDC86	0.552823	4.76E-06
CCDC9	0.208205	0.048639
CCDC92	-0.37304	0.003881
CCHCR1	0.257076	0.007578
CCK	1.055894	0.021511
CCL20	1.134802	2.82E-08
CCL28	-0.33548	0.000434
CCL5	-1.25164	0.017902
CCNA1	1.252005	2.44E-23
CCNA2	0.253772	0.015309
CCNB1	0.308282	0.000284
CCNB1IP1	0.141568	0.028908
CCNB2	0.25906	0.000197
CCNB3	0.706749	0.03838
CCND2	0.585914	2.10E-13
CCND3	0.328194	0.00092
CCNDBP1	-0.19637	0.014072
CCNG1	0.232729	0.001727
CCNH	0.214908	0.002709
CCNJL	-0.43723	0.000348
CCNK	0.238241	1.59E-05
CCNO	0.620691	0.002026
CCNY	0.098955	0.049255
CCPG1	-0.39222	0.000788
CCT2	0.243787	0.000504
CCT3	0.233138	1.33E-06
CCT4	0.291094	4.01E-06
CCT5	0.280573	5.22E-07
CCT6A	0.288487	5.40E-08
CCT6B	-0.57468	0.028478
CCT7	0.378112	2.29E-13
CCT8	0.232996	0.00098
CD101	-0.51358	0.004907

CD109	0.186873	0.029944
CD160	-0.43581	0.049176
CD163L1	-0.36683	0.03354
CD24	-0.46957	7.36E-17
CD27-AS1	-0.24328	0.003726
CD320	0.532197	0.002941
CD33	0.960828	0.034089
CD36	-1.13581	7.08E-12
CD37	-0.54455	0.004185
CD38	-0.89417	0.03021
CD3EAP	0.409501	0.000615
CD44	0.367626	5.10E-07
CD46	-0.23654	0.004349
CD47	-0.23559	0.002711
CD55	0.520165	1.67E-06
CD59	0.179856	0.010823
CD6	0.61558	0.013021
CD68	0.318464	0.000577
CD83	0.234372	0.019356
CDA	0.275924	0.002762
CDC123	0.233439	0.0001
CDC20	0.574142	3.25E-06
CDC25A	0.560815	1.63E-06
CDC25B	0.371427	0.001547
CDC34	0.384826	0.007324
CDC37	0.361092	0.000255
CDC42BPA	-0.35106	7.20E-07
CDC42EP1	0.709751	4.50E-05
CDC42EP2	0.529342	0.005032
CDC42EP3	-0.48101	3.11E-08
CDC42SE2	-0.29451	0.0001
CDC45	0.395982	6.70E-09
CDC6	0.249646	1.64E-05
CDCA2	0.300536	0.00259
CDCA3	0.446315	0.000253
CDCA4	0.328889	0.012192
CDCA5	0.409401	5.02E-05
CDCA7	0.212976	0.040753
CDCA7L	0.197715	6.40E-05
CDCA8	0.35423	0.007436
CDCP1	0.265852	9.20E-07
CDH1	-0.43477	0.000858
CDH11	0.798677	3.29E-08
CDH13	-0.2407	0.000448
CDH2	-0.30136	0.000906
CDHR2	-0.83662	0.000119
CDK1	0.289864	0.007905

CDK10	0.293303	0.00823
CDK11B	0.156235	0.039128
CDK12	0.150063	0.009355
CDK17	0.254458	0.001409
CDK19	-0.28493	0.004213
CDK2	0.233068	0.001138
CDK2AP1	0.123529	0.038356
CDK4	0.416238	2.99E-05
CDK5RAP1	0.226581	4.38E-05
CDK5RAP2	0.142881	0.002602
CDK5RAP3	0.166243	0.018381
CDK6	0.283801	0.001689
CDKL3	-0.48833	0.02017
CDKL5	-0.35071	0.00019
CDKN1B	-0.19659	0.007359
CDKN2AIPN	0.195277	0.001384
CDKN2B	-0.72314	1.48E-10
CDKN3	0.159162	0.023505
CDNF	-0.5905	0.005554
CDPF1	0.350077	0.027746
CDRT1	-0.6483	3.04E-05
CDS1	-0.19202	0.007464
CDT1	0.453889	0.026219
CDYL	0.19917	2.94E-05
CEACAM19	-0.46524	0.01525
CEBPZ	0.203698	0.005341
CECR7	-0.3097	0.025651
CELF1	0.175281	0.002519
CELSR3	-0.32694	0.031774
CEMIP	0.191725	0.000808
CENPA	0.332133	0.045487
CENPH	0.31286	0.00013
CENPN	0.199835	0.007761
CENPO	0.262316	0.003877
CENPP	0.133671	0.040393
CENPU	0.212633	0.004163
CENPV	0.415577	4.54E-05
CENPW	0.315171	0.000549
CEP112	-0.40938	0.021662
CEP126	-0.24059	0.003248
CEP128	0.227505	0.008125
CEP131	0.407242	0.017906
CEP135	0.193662	0.047056
CEP162	-0.20373	0.026864
CEP164	0.257613	0.000154
CEP170	0.314607	0.002595
CEP19	-0.38006	0.000537

CEP250	0.245277	0.008711
CEP55	0.307519	1.05E-05
CEP70	-0.36573	0.000278
CEP72	0.389892	0.000405
CEP78	0.162376	0.004808
CEP83	0.278432	0.003828
CEP85	0.161874	0.003623
CEP89	0.14071	0.027083
CERCAM	-0.30512	0.024672
CERKL	-0.40307	5.65E-06
CERS2	0.213204	0.001996
CERS3	-0.42637	0.002887
CERS4	-0.3591	0.027219
CES4A	-0.66109	0.000147
CETP	-0.98571	0.032922
CFAP20	0.201024	0.000775
CFAP206	-0.76214	0.003276
CFAP44	-0.65161	2.18E-06
CFAP46	0.739832	0.003603
CFAP57	-0.22114	0.032067
CFAP70	-0.50249	0.004583
CFAP97	0.172044	0.038606
CFB	-1.01617	0.018698
CFH	-0.18197	0.019403
CFI	-2.25472	3.93E-05
CFL2	-0.20039	0.006499
CFLAR	-0.4613	0.000135
CGB7	0.316543	0.035541
CGB8	0.676258	0.001652
CGNL1	0.264672	0.004091
CH17-195P	-0.44291	0.029569
CH17-264L	-0.40732	0.00189
CH17-296N	0.531698	0.008757
CH507-236	-0.77566	0.034069
CH507-338	-0.77566	0.034069
CH507-513	-0.28867	2.75E-05
CH507-528	-0.2882	2.90E-05
CH507-9B2	-0.51241	0.041887
CH507-9B2	0.399049	6.98E-06
CHAC1	0.299244	0.039536
CHAF1A	0.347479	0.00016
CHAF1B	0.180874	0.004463
CHAMP1	0.165028	0.031447
CHCHD2	0.189172	0.034347
CHCHD3	0.275024	3.69E-07
CHCHD4	0.570867	2.74E-09
CHCHD5	0.234114	0.012734

CHD1	0.211446	0.00406
CHD1L	0.174644	0.010207
CHD4	0.162333	0.004795
CHD6	-0.19093	0.004889
CHD7	0.213804	0.005748
CHEK1	0.214944	0.001444
CHERP	0.412558	0.001719
CHI3L1	-1.19957	0.019007
CHIC1	-0.20418	0.029558
CHIC2	0.336806	0.000425
CHID1	0.189345	0.016725
CHM	-0.1749	0.048384
CHMP1B	-0.22323	0.006013
CHMP1B2F	-0.5132	0.043011
CHMP3	-0.1333	0.013617
CHN1	-0.3276	0.040352
CHN2	-0.27591	0.040068
CHODL	-0.45317	0.036104
CHP1	-0.14183	0.032254
CHRAC1	0.120664	0.018598
CHRNA5	0.440127	0.000891
CHST11	0.144578	0.011484
CHST15	-0.12674	0.049831
CHSY3	-0.44499	0.025414
CHTF18	0.675876	0.002967
CHTOP	0.115476	0.048322
CIAO1	0.099942	0.042341
CIB1	0.31922	0.001079
CIITA	-0.44125	0.006146
CISD3	0.277433	0.032331
CISH	0.386119	0.036874
CIT	0.236777	0.002732
CIZ1	0.263981	0.004791
CKAP2L	0.225748	0.006563
CKAP5	0.174885	0.003686
CKMT1B	-0.20035	0.014244
CKS1B	0.408817	2.78E-06
CKS2	0.270222	0.022819
CLASP1	-0.14983	0.01545
CLCA2	-0.89519	0.00122
CLCF1	0.341728	0.001228
CLCN3	-0.17279	0.005176
CLCN6	0.138235	0.021177
CLDN1	-1.26645	3.87E-41
CLDN16	-1.00609	4.07E-10
CLDN23	-0.89035	1.36E-06
CLDN4	-0.43107	0.025839

CLEC12A	-0.87216	0.001014
CLEC2D	-0.56636	0.000827
CLEC7A	-1.24331	8.07E-06
CLHC1	-0.39998	0.008359
CLIC1	0.180374	0.000193
CLIC3	-1.29536	2.10E-05
CLIC4	-0.37484	0.001067
CLIP1	-0.39151	0.000426
CLIP3	-0.68239	0.000856
CLIP4	-0.21532	0.039746
CLK2	0.176303	0.010671
CLK3	0.179864	0.009184
CLMN	0.318689	0.027694
CLMP	0.684722	7.52E-17
CLN5	-0.21746	0.004781
CLN6	0.318104	0.001764
CLN8	-0.25574	0.000123
CLNS1A	0.195417	0.003225
CLPB	0.364832	7.06E-07
CLPP	0.374203	0.028858
CLPTM1L	0.188429	0.009506
CLU	-0.38549	0.001853
CLUAP1	-0.20731	0.018849
CLUH	0.593618	0.000146
CLUHP3	-0.18839	0.022974
CLYBL	-0.52505	0.004351
CMAHP	-0.55642	0.04996
CMBL	0.273121	0.001917
CMP21-97(	-3.89142	0.002305
CMSS1	0.135744	0.046049
CMTM4	-0.36115	6.18E-12
CMTM7	0.407232	9.04E-05
CMYA5	-0.37562	0.042602
CNBP	0.251867	7.61E-06
CNGB1	-0.64373	0.000253
CNIH3	0.482407	0.004611
CNNM2	-0.25889	0.004658
CNOT10	0.132733	0.015661
CNOT3	0.290533	0.028843
CNOT7	0.14602	0.018561
CNOT9	0.13688	0.01512
CNPY1	-3.25228	0.020018
CNPY4	-0.2416	0.01044
CNTN1	0.225244	0.02842
CNTNAP3	0.256368	0.017027
COA4	0.265432	0.000562
COA5	-0.32862	1.24E-05

COA6	0.10866	0.045106
COBLL1	-0.39954	0.009411
COL12A1	-0.17824	0.004162
COL13A1	0.379901	0.008229
COL17A1	0.25408	0.00016
COL1A1	-0.39562	0.013141
COL1A2	-0.68956	2.84E-06
COL21A1	-0.9611	0.000279
COL3A1	-1.61735	3.27E-14
COL4A1	0.239629	0.002874
COL4A2	0.317346	0.01431
COL4A4	-1.00113	0.035204
COL4A5	-0.50939	2.39E-06
COL4A6	-0.12724	0.036065
COL5A1	-0.42113	0.001121
COL5A1-AS	1.101142	0.044219
COL5A2	-0.92276	7.85E-18
COL5A3	-0.35204	0.013503
COL6A3	0.603284	0.024965
COL8A1	-0.60755	1.22E-11
COL9A2	-0.69957	0.000936
COLCA1	-1.0778	0.000179
COLCA2	-2.08501	4.99E-06
COLGALT2	-1.86099	0.02535
COMMD2	-0.17913	0.010782
COMMD3	-0.1374	0.01746
COMMD4	0.18791	0.020252
COPRS	0.273593	0.012712
COPS3	0.276572	9.38E-08
COPS6	0.178561	0.001765
COPS7A	0.191936	0.018817
COPS8	0.139717	0.016434
COPZ1	0.116464	0.00516
COPZ2	-0.29252	0.023156
COQ2	0.266806	0.019864
COQ8A	0.277418	0.000839
CORO1A	0.630724	1.20E-05
CORO1C	0.096135	0.012637
CORO2A	0.154826	0.003131
COTL1	0.358224	0.000409
COX10	0.287896	0.000696
CPA4	-0.86094	6.44E-06
CPE	-0.59224	1.21E-07
CPEB2	-0.32842	0.035056
CPNE1	0.284464	1.64E-05
CPNE3	-0.16332	0.042393
CPNE7	0.639301	3.77E-05

CPOX	0.151812	0.033772
CPQ	-0.55222	1.80E-05
CPS1	-0.18636	0.000973
CPSF1	0.432043	0.001177
CPSF3	0.169181	0.005384
CPSF4	0.291911	0.00336
CPSF6	0.114631	0.018421
CPT2	-0.25308	0.000497
CPVL	-0.13683	0.00401
CPZ	-0.50503	0.012438
CR1	-0.86223	0.025964
CRACR2A	0.400981	9.83E-08
CRAMP1	0.140846	0.032124
CRCP	0.163767	0.007025
CREB3L2	-0.13427	0.036448
CREB5	-0.43773	0.03399
CREBL2	-0.17125	0.019609
CREG1	-0.46464	5.60E-06
CREG2	-0.3871	9.55E-06
CRELD1	-0.47573	0.006219
CRIM1	-0.24737	0.000212
CRISPLD1	-0.57013	0.000296
CRISPLD2	0.39703	0.027843
CROCC2	1.271823	0.027998
CROT	-0.45318	1.08E-06
CRYAB	-0.74108	7.45E-05
CRYBG3	-0.19856	0.013201
CRYL1	-0.22694	0.037948
CRYZL1	-0.20153	0.014686
CS	0.18177	4.10E-06
CSDC2	-0.96675	0.001683
CSE1L	0.304857	9.47E-07
CSF1	-0.44876	2.00E-05
CSF1R	-0.29721	0.040117
CSF2	0.883607	4.59E-07
CSMD2	0.736498	1.62E-07
CSNK1E	0.452124	1.93E-07
CSNK2A1	0.140207	0.00156
CSNK2A2	0.171676	0.001957
CSNK2B	0.172986	0.029828
CSPG4	0.353738	0.02123
CSRNP1	0.566332	1.37E-05
CSRNP3	-1.22465	2.09E-11
CSRP1	-0.27222	0.000177
CSRP2	0.12899	0.017851
CSTF2	0.267526	5.61E-06
CTA-212A2	-1.45122	0.0011

CTA-293F1	-0.56682	0.008451
CTA-414D7	0.516458	0.037298
CTAGE5	-0.204	0.038256
CTB-113P1	-0.52569	0.031249
CTB-133P2	0.711158	5.32E-05
CTB-151G2	-0.59539	0.000118
CTB-193M1	0.398781	0.000106
CTB-58E17	-0.78213	0.034226
CTB-5E10.3	0.663494	0.02417
CTB-60B18	0.567882	0.00243
CTBP2	0.221407	2.84E-09
CTBS	-0.23031	0.001294
CTC-261N6	-3.584	0.028451
CTC-351M1	-0.39992	0.02132
CTC-429L19	-1.57137	0.047582
CTC-471F3.	-0.6334	0.0058
CTC-479C5	0.468224	0.048784
CTC-490E2	-0.36593	0.009283
CTC-510F12	-0.63315	0.004206
CTCF	0.113045	0.002383
CTD-2008L	-0.43051	0.008562
CTD-2008P	-0.39543	0.043186
CTD-2036P	-0.83597	0.027792
CTD-2047H	-3.32247	0.001238
CTD-2058B	0.568054	1.65E-07
CTD-2066L	0.394521	0.029996
CTD-2192J1	-0.40411	0.007337
CTD-2269F	-0.92671	1.07E-05
CTD-2284J1	-0.68907	0.003017
CTD-2292M	-0.40685	0.012161
CTD-2303H	-0.78827	4.72E-06
CTD-2309C	-1.34772	0.008691
CTD-2319I1	-1.14915	0.004327
CTD-2323K	0.750485	0.007726
CTD-2354A	0.708553	8.63E-09
CTD-2382E	1.152389	0.004526
CTD-2396E	-0.8613	0.000674
CTD-2510F	0.292565	0.014733
CTD-2514C	-0.47617	0.032075
CTD-2521M	-0.85007	0.01454
CTD-2541J1	0.970815	0.007796
CTD-2547L	-0.83201	0.000182
CTD-2547L	-0.4321	0.019043
CTD-2553L	-1.32692	0.027046
CTD-2554C	-0.4231	0.031368
CTD-2562J1	1.521608	0.024403
CTD-2583A	0.47156	0.025475

CTD-2587H	1.049221	0.001836
CTD-2619J	-0.44796	0.031114
CTD-2623N	-0.83667	0.037825
CTD-2626G	-0.35714	0.001174
CTD-2647L	-0.43385	0.029578
CTD-2666L	-0.86162	0.029009
CTD-3006G	-0.64292	0.018839
CTD-3088G	-0.46835	0.03915
CTD-3105H	-1.70781	0.014965
CTD-3105H	-2.37191	0.018099
CTD-3113P	-0.25583	0.033998
CTD-3131K	-2.28283	0.049858
CTD-3214H	-3.32053	4.42E-05
CTD-3214K	-1.30301	0.036838
CTD-3247H	-1.96634	0.036003
CTDNEP1	0.269964	0.036036
CTF1	-0.50853	0.005479
CTNNAL1	0.597584	2.59E-13
CTNNBL1	0.17796	0.000885
CTPS1	0.267546	2.54E-05
CTSA	-0.26102	0.022606
CTSB	-0.39996	0.00015
CTSF	-0.68683	4.64E-08
CTSH	-0.2645	0.004078
CTSK	-0.36153	2.97E-05
CTSO	-1.016	2.33E-08
CTU2	0.428595	0.007142
CUL2	0.137417	0.016665
CUL4A	0.134877	0.001623
CUL5	-0.18585	0.018963
CUTC	0.174661	0.01461
CWC25	0.13854	0.032474
CWF19L1	0.130389	0.046128
CXADR	-0.46453	0.000803
CXCL1	0.451796	0.006025
CXCL10	-1.91374	0.009711
CXCL11	-0.7519	0.01932
CXCL12	-0.69316	0.02998
CXCL14	-1.20418	0.000142
CXCL16	-0.53452	5.43E-05
CXCL9	-2.25266	0.006199
CXCR2	-2.11892	0.036255
CXorf23	-0.36254	0.00076
CXorf40B	0.155977	0.035017
CXorf57	0.452323	6.39E-05
CXXC1	0.243598	0.033163
CXXC5	-0.87962	2.82E-07

CYB561	-0.1965	0.019155
CYB561A3	-0.33269	0.001231
CYB561D2	0.237743	0.028004
CYB5B	0.127115	0.006664
CYB5D1	0.275652	0.001205
CYB5D2	-0.45901	0.000101
CYB5R1	-0.38137	0.00074
CYB5R2	-0.57944	5.80E-05
CYBRD1	-0.71054	3.51E-19
CYC1	0.285502	0.015478
CYLD	-0.33664	0.00299
CYP1A1	-0.91322	0.000241
CYP1B1-AS	0.260814	0.000583
CYP26B1	0.493398	4.63E-06
CYP27B1	0.949862	2.81E-12
CYP27C1	0.286218	0.002262
CYP2B6	1.09515	1.97E-05
CYP2B7P	0.931738	5.06E-07
CYP2R1	-1.13338	0.012172
CYP2S1	0.343829	0.010955
CYP2U1	-1.07472	3.47E-10
CYP39A1	-0.90591	1.31E-08
CYP3A5	-0.4333	0.008245
CYP3A7	-0.71481	0.001148
CYP3A7-CY	-1.86692	0.007912
CYP4B1	-2.12595	0.003643
CYP4X1	-0.91092	5.38E-11
CYTH2	0.182303	0.023101
CYTH4	1.233503	0.001173
CYTOR	0.349804	0.000289
DAG1	-0.50358	7.50E-06
DAGLB	0.265896	0.002537
DANCR	0.363294	0.00079
DAP3	0.146498	0.013635
DAPK1	-0.68864	3.13E-07
DAPL1	-1.18584	0.000847
DARS	0.163458	0.019295
DARS2	0.213964	0.000418
DAW1	0.795209	0.003323
DAXX	0.195814	0.000544
DAZAP1	0.367171	0.000374
DBF4	0.287269	0.007644
DBF4B	0.254722	0.007174
DBH	0.917213	0.001903
DBNDD2	0.263534	0.035792
DBT	-0.1859	0.029843
DCAF1	0.177855	0.003741

DCAF13	0.270814	0.000433
DCAF15	0.307102	0.048825
DCAF5	-0.24829	9.29E-06
DCAF6	-0.1862	0.000221
DCBLD1	-0.68929	1.42E-22
DCDC1	1.149282	0.02441
DCK	0.162342	0.022972
DCLK2	-0.58516	0.049746
DCLRE1B	0.229673	0.004874
DCN	-0.97742	1.61E-08
DCPS	0.193725	0.017784
DCTD	0.313707	1.23E-07
DCTPP1	0.384788	4.77E-05
DCUN1D2-/	1.823254	0.023296
DCUN1D5	0.256897	0.000115
DDB1	0.101372	0.007693
DDB2	0.167479	0.009609
DDIAS	0.249569	0.005068
DDN	1.153527	4.07E-07
DDO	-0.70011	0.003277
DDX10	0.256611	0.00035
DDX11	0.291713	0.010513
DDX18	0.210932	0.004949
DDX19A	0.127021	0.035145
DDX20	0.140049	0.006626
DDX21	0.367908	3.58E-06
DDX23	0.167844	0.003879
DDX24	0.153312	0.003462
DDX27	0.186649	3.17E-05
DDX28	0.283698	0.012809
DDX31	0.368274	1.97E-11
DDX39A	0.386223	0.000124
DDX39B	0.309822	1.56E-08
DDX41	0.241634	0.007958
DDX49	0.335037	0.00522
DDX50	0.172095	0.012894
DDX50P1	0.584275	0.045057
DDX54	0.444164	0.002883
DDX55	0.182838	0.007629
DDX56	0.313799	4.41E-05
DECR1	-0.53477	8.15E-07
DEDD2	0.405647	0.005058
DEF6	0.293469	0.008636
DEFB1	-1.87885	0.000644
DEGS1	-0.16189	0.037567
DENND1B	-0.48619	2.18E-05
DENND1C	-0.64184	0.004509

DENND2D	-0.22509	0.037578
DENND3	0.246017	0.001095
DENND4C	-0.28291	0.011156
DEPDC1B	0.241179	0.003546
DFFA	0.132796	0.030576
DGKA	0.267414	1.22E-07
DGKD	0.383873	1.03E-09
DGKI	0.401129	0.00022
DGKZ	0.404853	0.012767
DHCR7	0.307727	0.000311
DHODH	0.192905	0.032549
DHPS	0.219519	0.002514
DHRS1	-0.20747	0.012409
DHRS11	0.348362	0.016345
DHRS12	-0.64065	0.000332
DHRS4-AS1	-0.20952	0.004098
DHRS7	-0.2171	0.002302
DHRS9	1.512584	1.23E-07
DHX15	0.194521	0.002852
DHX30	0.3656	0.000318
DHX33	0.476848	2.69E-28
DHX34	0.412087	0.012594
DHX35	0.192	0.000591
DHX37	0.423648	0.001224
DHX38	0.206839	0.000157
DHX40	-0.14707	0.04359
DHX9	0.154724	0.01094
DIABLO	0.1912	0.000233
DIAPH1	0.167816	5.47E-05
DIAPH2	0.242654	0.004905
DIEXF	0.258016	8.83E-06
DIMT1	0.314566	6.77E-06
DIRC2	-0.23028	0.000835
DIRC3	0.783458	0.046096
DIS3	0.205907	0.000906
DISC1	-0.95796	0.000239
DISP1	-0.40544	0.012152
DISP3	0.379851	0.048741
DIXDC1	-0.5235	6.36E-05
DKC1	0.397938	1.74E-07
DKK1	0.299189	2.18E-06
DKK3	-0.33719	4.59E-07
DLAT	0.190043	0.003734
DLC1	0.416154	9.14E-05
DLEC1	-1.63358	0.016864
DLEU1	0.261853	0.026911
DLG1-AS1	-0.44457	0.008585

DLG4	0.640623	0.000678
DLGAP1	-0.7315	0.000527
DLGAP1-AS	-1.30081	0.040838
DLGAP5	0.284151	0.010072
DLST	0.128563	0.004409
DLX1	0.322833	0.03023
DLX2	0.564255	0.019214
DLX3	-0.44331	0.00355
DLX4	-0.61386	0.000115
DMD	-0.1768	0.023163
DMKN	0.183312	0.00917
DMRT2	0.861303	0.020057
DMRT3	-1.43474	0.018255
DMRTC2	2.230182	0.012308
DMXL1	-0.3374	0.000758
DNA2	0.18693	0.0101
DNAAF3	0.633678	0.005164
DNAAF5	0.430821	0.000199
DNAH17	-1.19457	0.000302
DNAH5	-0.37907	0.000655
DNAJA3	0.328903	8.98E-06
DNAJA4	0.258522	0.017149
DNAJB2	-0.15593	0.028372
DNAJB4	-0.37435	0.027674
DNAJB9	-0.29206	0.029182
DNAJC1	-0.33704	7.94E-05
DNAJC11	0.241293	0.000555
DNAJC14	0.156744	0.006777
DNAJC16	-0.18981	0.011295
DNAJC2	0.256906	0.000624
DNAJC3	-0.2089	0.018483
DNAJC5	0.194328	0.012521
DNAJC7	0.156841	0.00092
DNAJC8	0.113482	0.019081
DNAJC9	0.239906	7.91E-08
DNAL1	-0.16725	0.047783
DNAL4	-0.23024	0.021886
DNASE2	-0.32002	0.000155
DNER	0.484932	2.21E-07
DNLZ	0.506209	0.017108
DNM1P35	-0.6561	0.044332
DNM2	0.198239	0.040849
DNM3	0.421556	0.009985
DNMBP	0.468896	1.18E-20
DNMT1	0.34768	3.64E-08
DNMT3L	-3.04462	0.035058
DNTTIP1	0.256403	0.001813

DOCK10	0.484376	4.00E-06
DOCK4	0.818826	1.30E-07
DOCK6	0.282905	0.021948
DOCK8	-0.76086	0.002018
DOCK9	0.236037	8.08E-05
DOPEY2	-0.37236	0.002582
DPAGT1	0.302478	8.51E-07
DPF1	0.446868	0.001198
DPF2	0.193583	0.001313
DPF3	0.450176	0.000966
DPH2	0.340744	0.000638
DPH7	0.298248	1.74E-05
DPM2	0.34486	4.33E-05
DPP4	-0.61558	0.000472
DPP8	-0.16852	0.035752
DPP9	0.434668	0.00085
DQX1	-0.52599	0.040879
DRAM1	-0.78259	8.52E-14
DRAM2	-0.15738	0.014479
DRAP1	0.520587	0.000312
DRD2	0.736999	0.037901
DRG1	0.197057	0.001369
DRG2	0.383123	3.06E-06
DSC1	-1.93571	0.000138
DSC2	-0.48647	0.000718
DSC3	-0.20864	0.023068
DSCR3	-0.13964	0.045821
DSEL	0.232556	0.007114
DSG3	-0.44794	0.006738
DST	-0.34227	1.37E-05
DSTN	-0.3828	2.09E-09
DSTYK	-0.2461	2.98E-06
DTD1	0.260047	0.001585
DTL	0.328195	2.23E-06
DTNA	-0.48231	0.003681
DTWD2	-0.48917	5.88E-05
DTX4	-0.57809	4.33E-06
DTYMK	0.372365	0.004295
DUBR	-0.49369	7.15E-05
DUOX2	-0.44124	0.028099
DUS1L	0.284698	0.026319
DUS2	0.198562	0.001091
DUS3L	0.560661	0.000456
DUSP11	0.193515	0.021771
DUSP12	0.187588	0.001448
DUSP14	-0.17991	0.025741
DUSP16	-0.8121	3.35E-07

DUSP2	0.892248	2.73E-08
DUSP3	-0.26188	0.000778
DUSP4	1.62	1.87E-65
DUSP5	1.341816	5.19E-30
DUSP5P1	-0.68481	0.010145
DUSP6	1.223475	8.96E-51
DUSP7	0.518338	1.91E-05
DUSP8	-1.25416	0.001156
DUSP9	0.500753	0.017125
DUXAP10	-0.20508	0.004069
DUXAP8	-0.23029	0.002689
DVL1	0.622558	0.002397
DYNC1LI1	0.14254	0.00963
DYNC1LI2	-0.16767	0.024144
DYNLT3	-0.29302	0.001116
DYRK3	0.370057	0.00014
DYSF	-0.29267	0.004153
DYX1C1	0.630966	0.010443
DYX1C1-CC	-6.821	2.13E-05
E2F1	0.297554	0.008992
E2F3	0.19434	0.000753
E2F4	0.412249	1.12E-06
E2F6P4	-0.46724	0.018073
E2F7	0.589011	1.58E-10
E2F8	0.199639	0.033944
EARS2	0.203804	0.00638
EBF3	-0.10255	0.016713
EBF4	-0.78027	0.00151
EBNA1BP2	0.406377	2.80E-08
ECE2	0.448933	2.81E-06
ECEL1P1	-1.39428	0.006814
ECEL1P2	-1.58128	0.006173
ECH1	-0.20497	0.019075
ECHDC2	-0.72298	1.14E-06
ECI2	0.151583	0.02913
ECM1	0.43914	0.006643
ECSIT	0.457349	2.32E-05
ECT2	0.220575	0.018465
EDARADD	-0.38825	1.03E-07
EDEM2	-0.16114	0.031872
EDEM3	-0.23402	0.013719
EDIL3	-1.52058	1.03E-13
EDN1	-0.98003	2.58E-24
EDN2	-0.67392	0.013163
EDNRA	0.545524	6.23E-06
EDNRB	-0.24188	0.016963
EEF1A1	0.160406	0.003907

EEF1A1P11	1.244977	0.032234
EEF1B2	0.21759	4.97E-05
EEF1D	0.369466	0.000744
EEF1E1	0.324111	0.001421
EEF1G	0.263776	4.46E-06
EEF2	0.383332	7.26E-05
EEF2KMT	0.253929	0.016912
EFCAB1	0.42332	0.035618
EFCAB14	-0.18066	0.0012
EFCAB2	-0.23982	0.022167
EFCAB6	-0.75024	0.001126
EFEMP1	-0.11078	0.01536
EFHD1	-0.84995	2.99E-09
EFHD2	0.701936	1.96E-05
EFNA1	-0.31209	0.000664
EFNA5	-0.33942	8.64E-07
EFNB1	0.623732	0.000277
EFNB2	0.214289	0.000342
EFTUD2	0.240923	1.87E-06
EGF	-0.41876	3.57E-05
EGFL6	-1.35095	0.002286
EGFR	-0.08277	0.024581
EGLN1	-0.24636	0.002631
EGR1	2.444051	1.50E-42
EGR2	1.974086	4.20E-15
EGR3	1.794795	4.97E-21
EHBP1	-0.19223	0.016482
EHD1	0.3013	0.035324
EHD4	0.179654	0.022926
EHHADH	-0.3854	1.38E-06
EHMT2	0.200526	0.044308
EIF1	0.156396	0.006643
EIF1AD	0.154419	0.020396
EIF2A	0.147878	0.048341
EIF2AK3	-0.23474	0.03218
EIF2B2	0.295367	2.76E-08
EIF2B3	0.140808	0.021847
EIF2B4	0.17456	0.01176
EIF2B5	0.148535	0.004864
EIF2D	0.155625	0.025155
EIF2S2	0.152818	0.012121
EIF3A	0.16138	0.010194
EIF3B	0.316912	1.06E-07
EIF3C	0.332059	3.36E-10
EIF3D	0.329118	3.11E-15
EIF3E	0.210986	0.004911
EIF3G	0.314873	0.003462

EIF3H	0.173839	0.000245
EIF3I	0.238973	7.89E-09
EIF3J	0.142632	0.027232
EIF3K	0.176736	0.001836
EIF3L	0.182931	0.000176
EIF3M	0.185424	0.003992
EIF4A1	0.355481	1.13E-14
EIF4B	0.252085	5.50E-07
EIF4E3	-0.5733	0.000717
EIF4EBP1	0.57625	0.000254
EIF4EBP2	-0.15455	0.021601
EIF4G1	0.238675	0.00126
EIF4H	0.164055	0.001003
EIF5A	0.479559	4.82E-07
EIF5B	0.198812	0.004952
EIF6	0.377617	3.42E-06
ELAC2	0.315822	1.31E-07
ELAVL1	0.148448	0.032231
ELFN2	0.824107	1.04E-07
ELK1	0.355704	0.004024
ELK3	0.561209	1.62E-11
ELL	0.376416	0.005382
ELL3	-0.36464	0.033215
ELMO1	0.155271	0.044715
ELMOD2	-0.33408	0.000509
ELOA	0.12165	0.012439
ELOF1	0.202846	0.008933
ELOVL1	0.232474	0.001399
ELOVL2	1.402872	1.45E-17
ELOVL4	0.444818	3.64E-05
ELOVL6	0.454973	1.22E-09
ELOVL7	-0.67454	0.000221
ELP3	0.127898	0.03735
ELP5	0.300326	0.002413
EMC2	-0.24484	0.01201
EMC3-AS1	0.31071	0.000134
EMC4	0.154554	0.009278
EMC8	0.248535	0.005553
EMG1	0.348215	1.14E-07
EML2-AS1	0.951115	0.032802
EML3	0.309716	0.015044
EMP1	0.630386	1.09E-10
EMP3	0.592357	2.91E-05
EN1	-0.46591	0.001935
EN2	0.406206	0.016637
ENAH	-0.42089	0.000146
ENDOD1	-0.35256	2.29E-07

ENO1	0.278405	4.36E-07
ENOPH1	0.186984	0.00619
ENPP6	-1.42749	0.035247
ENTPD3	-0.59042	0.001161
ENTPD3-AS	-0.6445	0.002635
ENTPD4	-0.15814	0.010497
ENTPD6	0.256709	0.014416
EPB41	-0.23918	0.004806
EPB41L1	-0.38837	0.023861
EPB41L2	0.270142	4.04E-05
EPB41L4A	-0.63451	0.046656
EPB41L4A-.	0.275325	0.010681
EPHA1-AS1	-0.51565	0.024905
EPHA2	0.641366	4.49E-07
EPHA3	-0.1744	0.01249
EPHB2	0.263478	0.016993
EPHX1	-0.27115	0.011386
EPHX2	-0.52601	7.65E-05
EPM2A	-0.27592	0.002787
EPM2AIP1	-0.36258	2.51E-07
EPN2	0.230913	0.010336
EPOR	0.48069	0.000752
EPRS	0.150215	0.025896
EPS15L1	0.160137	0.00364
ERAL1	0.190926	0.006931
ERAP1	-0.23707	0.017081
ERAP2	-0.33411	0.001169
ERBB3	-0.48979	1.46E-12
ERCC1	0.290445	0.000978
ERCC6L	0.258562	0.027736
EREG	1.362277	8.63E-23
ERFE	0.513718	0.000109
ERG	0.62641	1.52E-09
ERH	0.159868	0.041498
ERI1	0.254147	0.000687
ERI3	0.164489	0.036569
ERICH5	-1.3042	0.001286
ERLEC1	-0.1449	0.005836
ERLIN1	0.151725	0.0065
ERMAP	-0.33199	1.77E-05
ERMP1	-0.22845	0.004045
ERO1B	-0.42196	0.000159
ERRFI1	0.972068	7.01E-14
ERV3-1	-0.42605	0.001115
ERVH-1	0.553723	0.000673
ERVMER34	0.299111	0.00046
ERVW-1	-0.408	0.008626

ESD	0.140637	0.024458
ESF1	0.212523	0.040617
ESM1	1.324292	0.000535
ESPL1	0.405441	0.000103
ESR2	-0.36059	0.048737
ESRG	0.263817	3.22E-05
ESRP1	-0.19368	0.001408
ESYT1	0.208952	0.005508
ESYT3	-0.48805	0.013717
ETAA1	-0.29519	0.00017
ETFBKMT	-0.68192	0.007903
ETFDH	-0.24093	0.000506
ETFRF1	-0.31203	0.000653
ETS1	0.550776	6.27E-12
ETV1	0.643846	0.000142
ETV4	1.092004	3.00E-18
ETV5	0.303729	0.000439
ETV7	-0.70212	0.002749
EVA1C	-0.39567	0.038528
EVC2	0.200741	0.019816
EVI2A	0.410357	0.009288
EVPL	0.462827	0.008192
EWSR1	0.150924	0.001507
EXD2	-0.21491	0.000974
EXOC3L4	-0.65995	0.037836
EXOSC1	0.131905	0.026815
EXOSC10	0.180434	0.000232
EXOSC2	0.335237	2.51E-05
EXOSC3	0.221257	0.001755
EXOSC4	0.387818	0.044347
EXOSC5	0.456995	5.89E-05
EXOSC7	0.339664	6.00E-08
EXOSC8	0.309437	0.000318
EXOSC9	0.244103	0.000525
EXT2	-0.12453	0.047384
EZH2	0.190245	0.000543
EZR	0.310739	6.90E-13
F12	0.54687	0.046881
F3	0.144913	0.026061
F8	-0.51724	0.00027
FAAHP1	-0.49396	0.01467
FAAP100	0.402298	0.03216
FABP5	0.537895	2.63E-05
FADS2	-0.30874	0.011317
FAF1	0.207346	0.00194
FAHD2CP	-0.7252	0.000256
FAIM	-0.41416	9.42E-07

FAM106A	-0.70045	0.023031
FAM107B	0.280594	0.005127
FAM110C	-0.32296	0.007753
FAM114A1	-0.39385	2.79E-07
FAM117B	-0.20199	0.030875
FAM120A	0.122433	0.00632
FAM120B	-0.17924	0.002345
FAM129B	0.51383	1.74E-05
FAM131A	0.268799	0.012994
FAM131B	-0.96653	0.035945
FAM134B	-0.94915	5.76E-09
FAM136A	0.266947	0.00065
FAM138E	-3.98813	0.021764
FAM13A-AS	-0.46445	0.005601
FAM13B	-0.37601	1.95E-05
FAM151B	-0.43931	0.044789
FAM161A	-0.19209	0.027724
FAM167A	0.836859	7.64E-07
FAM168A	-0.13231	0.00705
FAM171A1	0.232807	0.006726
FAM172A	-0.36381	2.25E-05
FAM174A	-0.37693	3.18E-05
FAM177A1	-0.16822	0.001473
FAM182B	-0.82941	0.030175
FAM183A	-0.87525	0.028625
FAM185A	-0.34468	0.023162
FAM185BP	-1.49593	0.021848
FAM189B	0.396887	0.033967
FAM196B	0.95672	2.27E-13
FAM198B	-0.58086	1.43E-09
FAM19A2	-0.83085	0.001841
FAM200A	-0.31211	0.001027
FAM204A	0.171976	0.0288
FAM207A	0.508469	0.004404
FAM212B	-0.54213	1.02E-06
FAM212B-A	-0.93053	0.001454
FAM214A	-0.39936	3.29E-07
FAM216A	0.658416	1.06E-06
FAM21EP	-0.7514	0.007436
FAM21FP	-0.59228	0.049186
FAM222A-A	1.156893	0.024623
FAM227A	-0.428	0.004123
FAM228B	-0.28975	0.003364
FAM26E	-0.67982	0.012094
FAM27C	0.72011	0.001501
FAM46A	-0.46996	0.015186
FAM49A	-1.2309	0.003045

FAM49B	0.187416	0.002525
FAM53C	-0.22723	0.032641
FAM58A	0.26424	0.031513
FAM64A	0.491277	0.000204
FAM69A	-0.31312	0.000612
FAM72A	0.414859	0.00951
FAM72B	0.400095	0.007064
FAM72C	0.536199	0.00045
FAM72D	0.428604	0.022185
FAM78A	-0.61902	0.02093
FAM83A	0.692951	4.03E-16
FAM83A-AS	0.663815	3.99E-09
FAM83D	0.375938	0.018702
FAM83G	0.370047	0.000778
FAM8A1	-0.29007	0.003529
FAM90A12	-2.59421	0.016865
FAN1	-0.14993	0.03934
FANCA	0.407406	1.09E-06
FANCC	0.116499	0.012268
FANCD2	0.275752	1.93E-06
FANCE	0.195775	0.026906
FANCG	0.404601	0.000416
FANCI	0.26866	0.000459
FANK1	-0.40912	0.000477
FAP	-0.4766	8.45E-06
FAR2	0.388748	0.010567
FARSA	0.342446	0.003847
FARSB	0.329252	9.95E-09
FAS	-0.21993	0.011437
FASN	0.622518	0.000208
FAT1	-1.67413	0.004281
FAT2	-0.57621	4.15E-18
FAT3	0.585193	0.034551
FAT4	-0.26481	0.013307
FAU	0.332705	0.00093
FAXDC2	-0.78137	2.62E-07
FBL	0.476078	1.93E-07
FBLN1	-0.47923	1.25E-05
FBN1	-0.52305	4.01E-09
FBN2	0.350634	1.58E-05
FBP1	-0.9714	0.038784
FBXL17	-0.80222	3.33E-09
FBXL20	-0.34396	0.000294
FBXL5	-0.28455	0.000177
FBXL6	0.352427	0.027038
FBXO10	-0.37148	0.003658
FBXO17	0.233154	0.010534

FBXO2	-0.30658	0.030199
FBXO27	-0.36325	7.03E-05
FBXO31	0.220128	0.019279
FBXO32	-0.65485	1.51E-12
FBXO45	0.240091	0.024071
FBXO9	-0.2605	0.00498
FBXW10	-0.96549	0.000654
FBXW2	-0.19279	0.002278
FBXW4	-0.27805	0.006526
FBXW7	0.227692	0.009564
FCGBP	-0.74015	0.016203
FCGR1A	-1.11098	0.001808
FCGR1B	-1.03004	0.027679
FCHSD2	0.174966	0.011299
FCMR	0.788076	0.000256
FDPS	0.187313	0.002418
FECH	-0.27321	7.74E-05
FEN1	0.267694	0.011724
FER1L4	-0.5891	0.000522
FERMT1	0.213852	0.001391
FERMT3	0.717296	0.010649
FETUB	-2.90999	0.048123
FGD3	-0.64015	0.000124
FGF11	-0.46408	2.63E-10
FGF5	-0.37945	0.010776
FGFBP1	0.60405	1.24E-09
FGFR1	0.299905	0.002439
FGFR1OP	0.186439	0.013459
FGFR2	-0.28614	1.62E-05
FGFR4	0.613202	0.00294
FH	0.199579	4.59E-05
FHL1	0.33302	4.52E-07
FHL2	0.187715	0.025838
FHL3	0.421125	9.83E-05
FHOD1	0.389622	0.006235
FHOD3	-0.187	0.007979
FIBIN	-0.98649	7.07E-05
FICD	-0.31543	0.005611
FIG4	-0.47196	0.000137
FIGNL1	0.37168	1.97E-06
FILIP1L	-1.58702	0.006191
FIP1L1	0.179036	0.003552
FKBP10	0.301583	0.025004
FKBP11	0.336766	0.000275
FKBP15	0.101962	0.01343
FKBP1A	0.267855	0.000653
FKBP4	0.373703	6.19E-10

FKBP7	-0.32237	0.030788
FKBP9	-0.23849	0.002734
FLAD1	0.185778	0.031396
FLG	0.476562	0.003242
FLI1	0.541519	7.68E-08
FLII	0.208154	0.02921
FLJ16779	-1.48926	4.23E-07
FLJ20021	0.433668	0.005735
FLJ22447	-0.67349	0.0009
FLJ27354	0.809123	0.001232
FLJ31356	-1.09566	2.62E-06
FLJ37035	-0.59112	0.045102
FLNB	0.512989	8.02E-06
FLNB-AS1	0.785667	0.001737
FLNC	0.681577	1.11E-06
FLOT2	-0.29194	0.000992
FLRT2	0.285192	0.035312
FLRT3	-0.27743	0.000704
FLVCR1	0.228864	0.030496
FMNL2	0.188621	0.038098
FMNL3	0.42245	9.23E-06
FMO4	-0.40392	0.002776
FN1	0.561576	9.16E-05
FNBP1	0.225394	0.00039
FNBP1L	-0.17828	0.034194
FNDC3B	-0.22515	0.001574
FOLH1	-0.9591	0.000198
FOLR1	-2.03903	1.02E-12
FOLR3	-0.38049	0.00318
FOS	0.971668	8.37E-06
FOSB	1.064062	8.10E-15
FOSL1	1.111995	2.38E-19
FOSL2	-0.32374	0.000412
FOXA2	0.933364	0.007977
FOXC2	0.515009	0.000135
FOXD2-AS1	0.355494	0.04051
FOXD3-AS1	-1.12859	0.043433
FOXD4L3	-3.72677	0.015836
FOXE3	1.217338	0.015237
FOXF2	0.363477	0.004934
FOXG1-AS1	0.901146	2.12E-05
FOXJ3	0.172223	0.002008
FOXK2	0.193544	0.019479
FOXL1	0.428369	0.005813
FOXL2	-0.50019	0.032123
FOXL2NB	-0.4634	0.038514
FOXM1	0.324669	0.000696

FOXN1	-0.95539	0.003879
FOXN2	0.29179	0.006124
FOXN3	-0.25388	0.000596
FOXO4	-0.52563	3.67E-05
FOXO6	-0.59384	0.025187
FOXP2	-1.18637	1.64E-06
FOXRED1	0.151919	0.040199
FPGT	-0.28462	0.001525
FPR1	0.386329	0.043258
FRAS1	-0.73831	1.15E-09
FRG1	-0.2853	0.031059
FRG1BP	-0.2941	0.014395
FRG2C	-2.88046	0.029969
FRK	-0.52326	2.05E-06
FRMD3	0.407241	0.028729
FRMD5	0.271422	0.011462
FRS2	-0.21702	0.00553
FSCN1	0.591076	0.003294
FSCN2	-0.91797	0.009469
FSD1	2.300387	0.018816
FSD2	-1.00759	0.011219
FSIP2	-0.48257	4.10E-05
FST	0.675321	0.000277
FSTL1	-0.21909	0.019929
FSTL4	-1.15965	3.13E-24
FTO	-0.14294	0.004928
FTSJ1	0.178302	0.034691
FTSJ3	0.197728	0.003611
FTX	-0.52378	1.71E-06
FUBP1	0.175443	0.005439
FUBP3	0.100472	0.026738
FUCA1	-0.30456	2.14E-05
FUOM	0.390809	0.018607
FURIN	0.376395	0.010159
FUS	0.222638	0.005248
FUT2	-0.30618	0.044088
FUT4	0.275307	0.044272
FUT8	0.191609	0.007722
FUT8-AS1	1.24486	0.016712
FXN	0.197064	0.008198
FXR2	0.290323	0.001854
FXYD3	-0.77412	0.000118
FXYD5	0.385293	0.000158
FXYD7	1.401149	0.038611
FYTTD1	-0.23635	0.000653
FZD1	-0.58965	1.02E-08
FZD10	0.542835	0.000215

FZD5	-0.28681	6.15E-05
FZD7	-0.21492	0.020478
FZD9	0.605526	0.017509
G0S2	0.616464	1.54E-05
G6PC3	0.362649	0.002802
GAB1	-0.45033	6.26E-05
GABARAPL	-0.68992	0.000209
GABARAPL	-0.13208	0.033123
GABRA3	-0.61931	0.000779
GABRP	0.341709	0.002469
GAD1	-0.62783	0.000891
GAGE12F	1.148663	0.017227
GAL	1.138128	5.01E-13
GAL3ST4	-0.44539	0.018946
GALC	-0.46544	5.02E-08
GALE	0.370815	0.000126
GALK2	0.14097	0.014337
GALNT1	-0.23507	0.002731
GALNT10	-0.23298	0.000313
GALNT11	-0.27736	1.70E-07
GALNT14	0.398275	1.35E-05
GALNT18	-0.22298	0.009276
GALNT2	-0.26198	0.000898
GALNT5	-0.30009	6.31E-07
GALNT6	-0.1204	0.014804
GAPDH	0.239013	0.001354
GAPDHP62	1.113936	0.005467
GAR1	0.216298	0.031357
GARS	0.199686	0.013182
GART	0.320582	1.57E-07
GAS5	0.237616	0.001323
GAS6-AS2	-0.25055	0.024874
GATA6	0.295468	0.007087
GATAD1	-0.26609	7.03E-05
GATAD2A	0.395281	9.61E-05
GATAD2B	0.167224	0.014049
GATS	-0.83731	8.36E-10
GATSL3	-0.45928	0.008958
GBA	-0.1811	0.029075
GBE1	-0.18495	0.017845
GBGT1	0.441259	0.016885
GBP1	-0.95124	2.31E-07
GBP1P1	-1.09685	4.34E-05
GBP2	-1.45721	4.59E-07
GBP4	-1.15557	0.007406
GBP5	-0.67888	0.038645
GBP6	-0.49154	0.000274

GCA	-0.46434	0.002717
GCAT	0.341452	0.011667
GCDH	0.273688	0.002094
GCFC2	0.197329	0.008371
GCH1	-0.4371	0.002614
GCN1	0.221993	0.000397
GCNT1	-0.31083	8.45E-07
GCNT4	-0.81139	3.87E-08
GDA	0.426424	0.000118
GDF11	0.593257	2.60E-06
GDF15	0.73006	0.049369
GDPD1	-0.65516	0.025787
GDPD2	-0.42959	0.032408
GDPD5	0.375327	0.025801
GEM	0.530234	6.96E-05
GEMIN4	0.356156	2.96E-05
GEMIN5	0.402697	5.82E-15
GEMIN7	0.225445	0.01745
GFM1	0.171952	0.000608
GFM2	-0.23489	0.001265
GFOD1	0.101813	0.038865
GFOD2	0.197593	0.019997
GFPT2	1.064919	8.55E-25
GFY	2.299338	0.04425
GGA1	0.295818	0.005092
GGACT	-0.70673	0.002119
GGCT	0.210581	0.000434
GGPS1	-0.19662	0.034255
GGT6	-0.8067	0.007791
GGT7	-0.31088	0.012859
GHc-857G6	-1.04008	0.026038
GHET1	-0.5379	0.000564
GHR	-0.65559	6.44E-05
GIF	-3.2812	0.044624
GIMAP6	0.420794	0.042162
GIMAP8	1.416711	0.010428
GINM1	-0.23555	0.00118
GINS3	0.179492	0.017222
GINS4	0.261684	0.002997
GIPR	-0.4408	0.024805
GIT1	0.435974	0.000151
GJA1	-0.51686	0.000158
GJA3	1.504617	2.88E-10
GJB2	0.812267	7.52E-31
GJB3	0.655448	1.22E-09
GJB4	0.814618	8.64E-13
GJB5	0.541298	2.61E-12

GJD3	-3.11746	0.03818
GK	0.704696	1.54E-08
GKAP1	-0.67002	0.003323
GLB1L2	0.668403	6.50E-05
GLDN	-0.47907	0.009473
GLIPR2	-0.2876	0.003662
GLIS3	-0.32496	0.016632
GLO1	0.170615	0.014682
GLOD4	0.181271	0.0002
GLP2R	-1.3649	0.00015
GLRB	-1.39411	0.001332
GLRX	-0.48839	0.000636
GLRX3	0.187802	0.000363
GLRX5	0.205362	0.013032
GLS	-0.29455	0.015042
GLT8D2	-0.49808	0.000528
GLTSCR2	0.400074	0.00885
GLUD1	0.300039	8.50E-08
GLYCTK	0.656605	0.000245
GMCL1	-0.19485	0.011348
GMDS	0.265768	0.001525
GMDS-AS1	-0.357	0.041537
GMEB2	0.23597	0.032801
GMIP	0.188756	0.049027
GMPR	-0.61746	0.007289
GMPR2	-0.17056	0.001888
GMPS	0.258738	0.00011
GNA12	0.293493	0.000175
GNA15	0.380835	0.000405
GNAI1	-0.22722	0.001317
GNAO1	-0.47291	0.010028
GNE	0.228574	0.047686
GNG11	0.865886	9.22E-05
GNG2	0.343671	0.025903
GNG5	0.222135	0.03068
GNL1	0.21367	0.006214
GNL2	0.252656	8.04E-06
GNL3	0.287951	4.41E-06
GNL3L	0.295383	0.000265
GNPDA1	0.198694	0.000311
GNPNAT1	0.238079	0.000188
GNPTAB	-0.42136	2.64E-07
GNS	-0.21112	1.34E-06
GOLGA1	-0.17375	0.001541
GOLGA2P5	-0.36473	0.000697
GOLGA6L1	-4.53318	0.000256
GOLGA6L5	-0.48447	0.002185

GOLGA6L9	-0.25145	0.014435
GOLGA8CP	-1.05629	0.026558
GOLGA8EP	-3.596	0.012038
GOLGA8M	-1.44361	0.036833
GOLGA8N	-0.80056	2.20E-10
GOLGA8R	-0.71184	0.005432
GOLGB1	-0.34014	0.000304
GOLIM4	0.116636	0.049747
GOLM1	0.21986	1.28E-05
GOPC	-0.40031	3.64E-08
GOT1	0.306626	1.31E-08
GOT2	0.189706	4.22E-05
GP6	-1.35664	0.000397
GPANK1	0.254167	0.025341
GPAT2	0.403926	7.58E-05
GPAT3	0.901051	5.62E-17
GPAT4	0.139718	0.012295
GPATCH4	0.253154	0.001432
GPHN	0.29692	7.40E-05
GPI	0.202657	0.003024
GPKOW	0.203893	0.002068
GPM6A	-0.76379	0.001542
GPN1	0.194226	0.001581
GPN3	0.173227	0.016115
GPNMB	-0.78947	9.93E-09
GPR1	-0.60354	2.94E-10
GPR107	-0.21367	0.000204
GPR132	-0.62428	0.005586
GPR135	-0.27974	0.02611
GPR137C	-0.39263	0.021583
GPR153	0.927863	1.47E-08
GPR155	-0.74143	1.88E-06
GPR158	-0.29216	0.000511
GPR161	-0.32813	7.41E-05
GPR176	0.253864	0.02327
GPR179	-1.10678	0.035272
GPR3	0.78303	0.007675
GPR50	0.960522	4.65E-12
GPR68	-0.31052	0.029447
GPR87	0.218352	0.007901
GPRASP2	-0.27809	0.005238
GPRC5B	0.211245	0.00999
GPRC5C	-0.31701	0.022666
GPRIN1	0.369819	0.004854
GPRIN2	-1.03287	1.19E-05
GPSM1	0.484125	0.032015
GPSM3	1.056917	1.11E-06

GPX3	0.302442	0.004444
GPX7	-0.4805	0.000188
GRAMD1A	0.362313	0.004802
GRAMD1C	-0.60393	0.000578
GRAMD2	-0.6213	4.91E-09
GRAMD3	-0.42307	1.54E-06
GRB10	0.399251	5.53E-07
GRB14	0.33989	0.029351
GRB2	0.102286	0.048898
GRB7	0.572061	2.97E-05
GREM1	-0.40479	0.001489
GRHL2	-0.20545	0.001371
GRIA1	0.429419	0.001756
GRIK2	-0.3556	0.001331
GRIN2A	-0.97073	0.040277
GRK5	0.60628	1.68E-10
GRK6	0.289729	0.024445
GRK7	-1.77605	0.009625
GRM1	-0.91738	0.005736
GRPEL1	0.401142	1.51E-10
GRSF1	0.175534	0.016626
GRWD1	0.3672	0.002367
GS1-24F4.2	-2.3473	0.044389
GS1-259H1	-0.66101	0.032418
GS1-304P7	-2.76959	0.041417
GS1-358P8	-0.81337	2.03E-06
GSAP	-0.81239	0.000215
GSDMB	-0.45097	0.000545
GSG2	0.416056	8.86E-05
GSK3A	0.281694	0.00555
GSK3B	-0.1429	0.0059
GSPT1	0.219794	2.12E-05
GSPT2	0.237756	0.000338
GSS	0.164129	0.010213
GSTA4	-0.33247	0.000209
GSTK1	-0.389	2.92E-06
GSTM2	-0.55302	1.79E-05
GSTO1	0.472199	1.65E-14
GTF2F1	0.224305	0.016376
GTF2H2B	0.293372	0.049543
GTF2H4	0.190324	0.008173
GTF2I	-0.19031	0.005218
GTF2IP12	-0.60653	0.000153
GTF2IP4	-0.28068	2.45E-05
GTF2IRD2B	-0.54908	3.16E-09
GTF3A	0.254931	1.31E-05
GTF3C2	0.207472	0.007675

GTF3C5	0.302517	0.002926
GTF3C6	0.195404	0.044669
GTPBP1	0.180006	0.019796
GTPBP4	0.272353	2.14E-06
GTSE1	0.34196	0.023132
GUCY1B2	-2.12738	0.001284
GUCY2C	-0.51477	0.034805
GXYLT2	-0.66986	4.44E-07
GYG2	-0.74838	0.041305
H19	-0.80633	0.000342
H2AFV	0.116342	0.014417
H2AFY	0.117383	0.019644
H2AFZ	0.269943	0.000189
H3F3B	0.128573	0.002586
H6PD	-0.28399	0.000268
HACD1	-0.33953	4.28E-07
HACD2	-0.57008	1.99E-17
HACD4	-0.1611	0.041059
HADH	-0.35824	3.54E-10
HAGH	-0.41759	0.002308
HAP1	-0.45788	0.00456
HARS	0.209966	0.000154
HAS2	0.635417	0.001536
HAS2-AS1	0.521015	0.044775
HAUS1	0.273221	0.018137
HAUS2	0.164981	0.009137
HAUS4	0.181172	0.000757
HAUS5	0.415379	0.002801
HAUS7	0.433306	4.67E-05
HAUS8	0.29095	0.002109
HAX1	0.154824	0.001695
HBE1	0.55129	0.04564
HBEGF	1.045006	1.51E-12
HBP1	-0.36144	0.007059
HCAR2	-0.74435	0.039833
HCFC1	0.305493	0.042959
HCG27	-0.67688	0.002366
HCK	-1.1985	0.010512
HCN3	-0.46166	0.04398
HCP5	-0.4765	7.07E-09
HDAC11	-0.2293	0.047277
HDAC2	0.151116	0.017857
HDAC3	0.194372	0.001687
HDAC7	0.237934	0.04759
HDAC9	0.415916	0.000114
HDDC2	0.174616	0.003602
HDDC3	0.256568	0.04955

HDGF	0.196749	0.003993
HDHD5	0.37487	0.000244
HEATR1	0.207787	0.003853
HEATR4	-0.63077	0.0001
HEATR5A	-0.30958	0.034233
HEBP2	-0.16229	0.001842
HECTD4	-0.2342	0.001887
HEG1	-1.15009	1.97E-15
HELLPAR	-0.34105	3.80E-05
HELLS	0.196597	0.015131
HELZ	-0.41219	1.64E-06
HEPHL1	0.390059	0.018983
HERC2	-0.38451	1.63E-14
HERC2P9	-0.31699	0.000269
HERC3	0.227043	0.003711
HERPUD2	-0.17648	0.003435
HES1	0.210358	0.009295
HES2	-0.65685	0.000142
HES6	0.604548	0.029762
HEXA	-0.14386	0.005556
HEXIM1	-0.37282	3.05E-06
HEY1	0.678376	0.006268
HEY2	0.546277	0.012807
HFE	-0.3451	7.28E-08
HGH1	0.389257	0.003663
HGS	0.324283	0.035448
HGSNAT	-0.36832	4.09E-08
HHAT	-0.41973	6.74E-05
HHIP	-0.94055	0.004895
HHLA1	0.550178	1.70E-10
HIBCH	-0.25235	0.004166
HIC2	0.383786	0.005765
HID1	-0.86194	0.029309
HIGD2A	0.262401	0.037031
HINT3	-0.18398	0.027351
HIST1H1C	-0.51102	1.35E-05
HIST1H2AC	-0.49952	2.82E-09
HIST1H2AE	-0.45713	0.012022
HIST1H2AG	-0.34329	0.003055
HIST1H2AI	-0.68877	0.004904
HIST1H2BC	-0.60095	4.85E-06
HIST1H2BD	-0.41529	0.000143
HIST1H2BE	-0.76825	0.002806
HIST1H2BF	-0.94165	0.000105
HIST1H2BG	-0.8343	3.19E-16
HIST1H2BN	-0.66994	4.89E-07
HIST1H3D	-0.38179	0.023137

HIST1H3E	-0.9484	1.68E-11
HIST1H3H	-0.77062	6.98E-07
HIST1H4E	-0.84634	4.97E-05
HIST1H4H	-1.1631	2.07E-09
HIST2H2BE	-0.60841	1.05E-08
HIST2H2BF	-0.55642	3.67E-05
HIST2H4B	-0.52827	6.00E-08
HIST4H4	-0.47303	0.009191
HIVEP2	-0.18309	0.023132
HIVEP3	0.252581	0.017627
HJURP	0.306337	0.0167
HK1	0.186599	0.010506
HK2	0.599366	4.18E-09
HKDC1	0.422359	0.00233
HLA-B	-0.39318	0.005365
HLA-DPB1	0.394048	0.040492
HLA-DRA	-1.76504	0.022387
HLA-F	-0.35296	0.040346
HLA-F-AS1	-0.29208	0.005073
HLCS	-0.36383	2.25E-09
HLX	0.620158	0.001548
HMBS	0.257264	0.001211
HMCES	0.17743	0.003136
HMCN1	-0.40691	0.020265
HMGA1	1.13051	6.65E-15
HMGA2	0.758345	7.20E-07
HMGB1	0.218369	0.012992
HMGB1P6	0.32239	5.85E-05
HMGCL	-0.20813	0.001288
HMGCS2	-3.34373	0.001176
HMGN2	0.253871	0.000293
HMGN3	-0.32029	3.45E-06
HMGN3-AS	-0.34144	0.009288
HMGN5	-0.27786	0.031737
HMGXB3	0.202841	0.001148
HMMR	0.309404	0.006363
HN1	0.193777	0.03559
HNF4A	1.287063	0.027734
HNRNPA1	0.270523	0.000167
HNRNPA1P	1.475313	0.034435
HNRNPAB	0.301138	3.52E-07
HNRNPC	0.166387	0.002924
HNRNPD	0.219086	0.000654
HNRNPDL	0.129906	0.002072
HNRNPF	0.277068	1.20E-13
HNRNPK	0.1565	0.005166
HNRNPL	0.315001	3.45E-07

HNRNPM	0.177265	0.011398
HNRNPR	0.168289	0.020302
HNRNPUL1	0.230183	0.008239
HOMER1	0.241362	0.000954
HOMER2	0.167757	0.007649
HOMEZ	-0.37743	2.69E-07
HOPX	0.715384	0.010328
HOTAIR	-0.46859	0.00511
HOTAIRM1	0.179943	0.020215
HOTTIP	-1.09422	0.001265
HOXA11-AS	-1.30893	0.00882
HOXA13	-0.36332	0.007557
HOXA4	-0.44475	0.02658
HOXA5	-0.41283	0.025064
HOXA-AS2	-0.54666	0.024423
HOXB3	0.200987	0.046392
HOXB8	-0.47583	0.040435
HOXC12	-1.3912	0.024456
HOXC13-AS	-0.29155	0.026994
HOXD10	-0.23906	2.13E-05
HOXD13	0.438644	0.005342
HOXD8	-0.27801	0.023941
HOXD9	-0.26722	0.040122
HPCAL1	0.292318	0.031094
HPDL	0.566638	0.013608
HPRT1	0.25202	0.00099
HPS3	-0.27443	5.05E-05
HR	0.446129	0.010646
HRAS	0.271127	0.022231
HRH1	0.289857	0.002441
HRH2	-0.66642	1.01E-05
HRK	-1.09397	0.006886
HS3ST1	1.295209	1.17E-15
HS3ST2	-0.46838	0.003258
HS3ST3A1	0.635738	0.001367
HS6ST1	-0.43946	0.001635
HS6ST2	0.637067	2.23E-08
HSD11B1	1.071678	0.001323
HSD11B2	0.417522	0.000951
HSD17B2	0.651757	0.00027
HSD17B4	-0.18831	0.008486
HSD17B6	-0.49281	0.00775
HSD3B7	-0.62484	0.003821
HSF1	0.382874	0.012301
HSFX1	-0.46906	0.029953
HSP90AA1	0.257608	0.001353
HSP90AB1	0.254027	0.000226

HSPA12A	-0.3984	1.03E-06
HSPA1A	0.289536	0.02716
HSPA4	0.212049	0.003651
HSPA4L	-0.32721	0.018142
HSPA9	0.254505	3.65E-06
HSPB1	-0.56695	0.007199
HSPB11	-0.19417	0.016254
HSPB3	-0.9734	9.82E-11
HSPB8	-0.4726	1.21E-06
HSPBP1	0.364208	0.019148
HSPD1	0.395913	1.37E-05
HSPE1	0.217051	0.026034
HSPH1	0.25222	0.027899
HTATIP2	-0.24375	0.000737
HTR1D	-0.4399	0.000822
HTR2C	-0.61812	0.001272
HTR7	0.595994	1.24E-07
HTRA1	-0.78711	4.02E-12
HUS1	0.224798	0.019876
HYAL1	-0.37821	0.000822
IAH1	-0.16013	0.036945
IARS	0.297421	1.18E-07
ICAM1	-0.47371	0.008216
ICK	-0.16537	0.01734
ICOSLG	-0.73206	2.35E-09
IDH1	-0.65749	2.65E-06
IDH3A	0.169783	0.017587
IDH3B	0.233287	0.017615
IDNK	-0.44954	0.006023
IDO1	-1.98249	0.018634
IER2	0.451493	0.001643
IER3	0.932557	1.48E-14
IFFO2	-0.41998	0.004157
IFI27L1	-0.19285	0.028678
IFI30	-0.23836	0.003995
IFI35	-0.4193	0.044582
IFIT5	-0.30882	0.034678
IFITM10	-0.50754	0.004184
IFNAR2	-0.22091	0.009108
IFNE	0.354804	0.036675
IFNGR2	0.303292	0.000121
IFNWP19	0.325391	0.025125
IFRD2	0.501156	2.29E-05
IFT43	-0.18268	0.017096
IFT80	-0.27064	0.000725
IFT81	-0.38352	9.43E-06
IGDCC4	0.728416	0.028638

IGF1R	0.111713	0.040173
IGF2	0.674857	0.022582
IGF2BP1	0.208469	0.015371
IGF2BP2	0.184638	0.001959
IGF2BP2-AS	1.086468	3.99E-05
IGF2R	-0.23451	0.000343
IGFBP1	0.922071	0.016324
IGFBP4	0.304883	0.017863
IGFBP5	-0.67761	0.004508
IGFBP7	-0.57519	1.08E-07
IGFL2-AS1	-0.41211	0.007188
IGHMBP2	0.249492	0.006673
IGIP	-0.57684	0.002312
IGSF1	-0.48791	0.001496
IGSF3	-0.30922	3.99E-05
IGSF9B	0.936225	0.002232
IKBKAP	0.134874	0.00469
IKZF2	-0.76939	2.49E-16
IKZF3	0.347073	0.012128
IL10RB	-0.23525	0.000385
IL11	1.174137	1.43E-24
IL11RA	-0.44287	0.009663
IL12A	0.412677	0.048466
IL13RA2	0.644715	0.000176
IL1A	0.548891	0.000131
IL1B	0.530313	0.001031
IL1R1	-0.51628	0.001209
IL1R2	0.925435	0.002834
IL1RAP	-0.16557	0.039826
IL1RL1	1.886944	9.22E-14
IL1RN	0.828387	5.72E-12
IL20	1.784179	7.32E-06
IL24	2.349054	1.95E-30
IL27RA	0.489693	1.46E-08
IL32	-1.25427	2.33E-12
IL37	1.700642	5.91E-06
IL3RA	-0.71692	0.000465
IL4R	0.235304	0.000467
IL6R	0.370221	3.22E-09
IL7	-0.89872	0.002068
IL7R	0.531548	6.81E-07
ILDR1	-1.03809	0.001833
ILF2	0.19969	0.000878
ILF3	0.355272	3.55E-07
ILKAP	0.245525	0.000202
IMMT	0.117951	0.028162
IMP4	0.421207	1.02E-05

IMPA2	-0.22517	0.035121
IMPDH1	0.330923	0.010166
IMPDH2	0.432496	2.42E-21
INAFM2	-0.50733	9.35E-07
INCA1	-0.3934	0.004458
INCENP	0.344021	0.006044
INF2	0.322568	0.047581
ING1	0.206417	0.032066
ING4	-0.47444	4.23E-08
INHA	-0.72033	0.023087
INHBA	0.668588	1.94E-08
INHBB	-0.74126	1.34E-05
INO80E	0.310847	0.001715
INPP1	-0.30063	0.043134
INPP4A	-0.16742	0.015981
INPPL1	-0.31248	0.001486
INSC	0.86305	0.042828
INSIG2	-0.24886	0.004475
INSL3	-1.18503	0.027271
INSR	-0.21842	0.008001
INTS10	0.111244	0.046538
INTS11	0.297223	0.00361
INTS13	0.321039	0.000626
INTS14	0.159342	0.006324
INTS4	0.258297	3.23E-05
INTS6L	-0.29206	0.009506
IPO11	0.23908	0.000102
IPO4	0.61436	3.57E-07
IPO5	0.30307	9.71E-07
IPO5P1	-0.31297	0.020873
IPO7	0.188357	0.024701
IPO9	0.155522	0.001498
IPP	-0.25238	0.001532
IPPK	0.119886	0.039829
IQCA1	-0.63212	1.07E-16
IQCF3	-1.30765	0.030917
IQCH-AS1	-0.36399	0.024614
IQGAP2	-0.42878	0.048014
IRAK1	0.423292	0.000578
IRAK1BP1	-0.26319	0.001749
IRAK2	0.257336	0.004459
IRAK3	-0.36	0.000131
IRF2	-0.18321	0.015706
IRF3	0.262208	0.013435
IRF4	1.003872	1.08E-21
IRGQ	-0.239	0.000193
IRS2	0.316159	0.005425

ISCA1	0.111628	0.047454
ISCA1P1	0.867929	0.001888
ISCU	0.148487	0.010906
ISM1	0.712769	0.000177
ISOC2	0.347143	0.044183
ISPD	-0.43508	0.003277
ISY1-RAB43	0.24652	0.008449
ITCH	0.17855	0.033866
ITFG1	-0.3204	1.53E-05
ITFG2	0.165103	0.028735
ITGA1	-0.32035	0.000215
ITGA11	-0.70718	8.49E-05
ITGA2	0.378965	0.001666
ITGA3	0.288602	0.004825
ITGA4	-0.28002	9.63E-06
ITGA5	0.269014	0.008869
ITGA6	0.176444	0.006793
ITGA7	0.861549	8.70E-05
ITGAV	-0.44293	4.45E-05
ITGB1BP1	0.215851	0.003117
ITGB2	0.423323	0.004184
ITGB3	-0.89145	1.57E-06
ITGB6	-0.49238	3.39E-07
ITGB8	-0.52426	2.92E-06
ITGBL1	-0.28643	5.65E-07
ITM2A	-0.62157	0.001704
ITM2B	-0.44866	0.000214
ITPA	0.254944	0.00124
ITPK1	0.402866	0.000251
ITPR1	0.18045	0.016017
ITPR3	0.368756	9.12E-07
ITPRIP	0.396369	7.32E-05
ITPRIPL1	0.624206	0.021887
ITSN2	-0.1733	0.048681
IVL	-1.22579	0.003469
IYD	-1.1593	0.016059
JADE2	0.30015	0.000261
JAG1	0.547581	1.26E-14
JAK3	-0.4433	0.045412
JAKMIP3	-0.37057	0.002702
JARID2	0.271318	1.19E-08
JKAMP	-0.1866	0.005283
JPH2	-0.79272	3.43E-08
JPX	-0.20029	0.033563
JRKL	-0.25024	0.044251
JUND	-0.64568	0.040011
KANK1	-0.2075	0.001991

KANSL1L	-0.33684	0.01244
KANSL2	0.234499	0.003273
KARS	0.311657	4.35E-10
KAT14	-0.17594	0.01336
KAT2A	0.332661	0.00159
KAT2B	-0.35749	0.000178
KAT5	0.223034	0.009771
KAT6A	0.126893	0.018133
KAT7	0.176294	0.001735
KATNAL1	-0.2199	0.031225
KATNB1	0.434767	0.002092
KB-1460A1	-0.83032	0.035317
KB-1836B5	-0.94362	0.037176
KBTBD3	-0.64409	0.000344
KC6	-0.38216	0.003144
KCMF1	0.220918	8.67E-05
KCNA2	-0.81279	4.31E-15
KCNAB1	-0.61116	0.001434
KCNAB2	0.274661	0.020914
KCND3	-0.88382	0.002823
KCNE4	-0.55016	0.025332
KCNF1	-0.6551	0.013081
KCNG1	0.644066	0.000131
KCNH3	0.944825	0.000801
KCNJ8	1.075708	0.04366
KCNK1	0.361229	0.000138
KCNK3	-0.93717	0.027982
KCNK5	0.712368	1.41E-09
KCNK7	0.977057	0.031939
KCNMA1	0.409339	0.000189
KCNN2	-1.79708	0.015672
KCNN4	0.388943	0.017297
KCNQ1OT1	-0.23795	0.001713
KCNQ5	0.39596	3.08E-05
KCNS3	-0.60238	4.98E-08
KCNV1	-0.5316	0.015265
KCTD10	-0.1379	0.007889
KCTD12	-0.2771	0.016863
KCTD15	0.207817	0.014217
KCTD17	0.387469	0.019911
KCTD18	-0.2998	2.95E-05
KCTD20	-0.207	8.98E-05
KCTD21	-0.34091	0.001876
KCTD5	0.237282	0.00373
KCTD6	-0.29444	0.009918
KCTD7	-0.28438	0.002628
KCTD8	1.408823	0.003107

KDELC2	-0.24708	0.000411
KDM1A	0.140522	0.000954
KDM4C	-0.24315	0.035704
KDM5A	-0.14892	0.049638
KDM5B	-0.36425	4.86E-08
KDM5C	0.152264	0.025356
KDM6A	-0.24857	0.000479
KDM7A	0.492607	0.027585
KDSR	0.2008	9.77E-05
KHDRBS1	0.171634	0.001499
KHDRBS3	0.573362	5.89E-07
KHNYN	-0.1545	0.000275
KHSRP	0.3735	0.002302
KHSRPP1	-1.08934	1.00E-06
KIAA0100	0.157312	0.000516
KIAA0232	-0.37695	0.000715
KIAA0319L	-0.43115	4.62E-07
KIAA0895	-0.51325	1.22E-05
KIAA1109	-0.22632	0.023444
KIAA1147	-0.48455	7.22E-06
KIAA1161	-0.26029	0.000476
KIAA1217	-0.22889	0.00453
KIAA1328	-0.19644	0.026857
KIAA1462	0.380038	0.00027
KIAA1524	0.236087	0.020421
KIAA1549	0.271541	0.000119
KIAA1549L	0.767445	5.33E-05
KIAA1614	-0.71511	0.000251
KIAA1644	-0.23444	0.006994
KIAA1683	-1.54733	3.34E-05
KIAA1841	-0.27189	0.004324
KIAA1958	0.283634	0.016202
KIDINS220	-0.24555	0.001202
KIF11	0.204246	0.038731
KIF12	-0.66401	0.005011
KIF13A	-0.20806	0.002629
KIF14	0.301494	0.007025
KIF15	0.417952	0.000226
KIF17	1.079783	1.60E-11
KIF18A	0.268096	0.014283
KIF18B	0.486162	0.000445
KIF1A	1.8468	1.23E-05
KIF1B	-0.16448	0.040064
KIF1C	0.199889	0.025962
KIF20A	0.303443	0.00036
KIF21B	0.454565	0.000264
KIF22	0.396675	8.04E-05

KIF24	0.231343	0.032786
KIF28P	-1.00258	0.015373
KIF2C	0.40681	7.68E-06
KIF4A	0.262093	0.003462
KIFC1	0.355404	0.008091
KIT	-1.22322	2.76E-05
KITLG	0.297277	0.002908
KL	3.416699	0.026227
KLC2	0.303399	0.01242
KLC3	0.390518	0.020671
KLF16	0.478314	0.006524
KLF4	0.329412	0.023094
KLF5	0.332508	0.001259
KLF7	0.165732	0.033177
KLF8	-0.45707	7.45E-07
KLHDC1	-0.64256	0.005825
KLHDC2	-0.16515	0.006896
KLHDC4	0.230408	0.029522
KLHDC8B	-0.85949	1.20E-06
KLHDC9	-0.62692	0.026203
KLHL13	-0.25958	9.44E-05
KLHL18	0.134956	0.028299
KLHL21	0.206667	0.035677
KLHL23	-0.25146	0.02447
KLHL24	-0.59357	0.000127
KLHL3	-0.34207	0.012109
KLHL33	-0.45598	0.017382
KLHL36	-0.25149	1.73E-05
KLHL41	-1.59909	0.041361
KLHL42	-0.2049	0.004246
KLK13	1.342254	0.000367
KLK5	-0.57568	0.001678
KLRC4-KLRI	-3.93016	0.008694
KMO	-0.82679	1.73E-10
KMT2B	0.348967	0.004069
KMT2C	-0.2507	0.0094
KMT2E	-0.21268	0.005728
KMT5A	0.209022	0.000445
KMT5B	-0.14752	0.017781
KNOP1	0.208589	0.002719
KNSTRN	0.220114	0.011824
KPNA2	0.264932	0.000925
KPNA2P3	-0.64037	0.035935
KPNA5	-0.18984	0.021276
KPNB1	0.219371	9.59E-05
KPTN	0.287071	0.014215
KRBA1	-0.33249	0.009666

KRBA2	-0.5531	0.000264
KRCC1	-0.57271	4.40E-08
KREMEN1	-0.18685	0.035088
KRI1	0.167545	0.016879
KRR1	0.404048	3.31E-07
KRT16P2	1.013896	0.017869
KRT17	-0.73193	0.000291
KRT18	0.481223	1.01E-09
KRT5	-0.20833	0.01983
KRT75	-1.76285	0.000769
KRT8	0.272479	0.018113
KRT80	-0.27329	0.009466
KRTAP10-2	-1.14167	0.049311
KRTAP5-AS	-1.27477	0.016835
KRTCAP3	0.261498	0.020578
KTN1-AS1	0.226809	0.007271
KXD1	0.215262	0.010727
KYAT1	0.318029	0.023881
L1CAM	-0.92941	1.05E-14
L3MBTL1	-0.76446	1.83E-07
L3MBTL4	-0.71085	0.000563
LA16c-358I	0.31375	0.008425
LA16c-380I	0.721879	0.002766
LA16c-390I	0.792842	0.00663
LACC1	-0.27658	0.015804
LACE1	-0.2185	0.020029
LACTB	-0.28355	0.000868
LACTB2	-0.16446	0.015597
LAD1	0.54031	2.70E-05
LAMA3	0.383805	2.79E-11
LAMA4	0.890685	2.55E-11
LAMB3	0.344926	0.00115
LAMC2	0.351216	1.47E-05
LAMC3	0.537218	0.001937
LAMP1	-0.16488	0.014066
LAMP2	-0.29858	0.000437
LAMP3	-0.65718	0.00312
LAMTOR3	-0.18363	0.027254
LANCL1	-0.21509	0.001515
LANCL2	0.152143	0.0369
LAPTM5	0.597844	1.20E-06
LARGE1	-0.55559	0.004921
LARP1	0.140939	0.010163
LARS	0.204264	0.006647
LARS2	0.15474	0.008432
LAS1L	0.315462	5.14E-09
LASP1	0.170911	0.035802

LAYN	-0.45357	3.42E-08
LBH	-0.76261	7.86E-13
LBR	0.18195	0.035833
LCK	1.235339	1.03E-12
LDB3	-1.2541	6.32E-06
LDHB	0.338272	3.36E-05
LDLR	0.373643	8.41E-06
LDLRAD3	0.441912	2.55E-12
LDLRAD4	-1.56217	2.05E-27
LDOC1L	-0.25237	0.004463
LEFTY2	-2.5368	0.009544
LENG1	0.395308	0.002628
LENG9	0.407076	0.047893
LEPROT	-0.33133	3.00E-07
LEPROTL1	-0.17701	0.02196
LETM1	0.260848	0.001024
LETM2	0.41875	2.82E-09
LGALS3	0.335441	0.000414
LGALS8	-0.26938	0.00214
LGALSL	-0.51948	0.000877
LGMN	-0.22667	0.012849
LGR4	-0.3379	2.69E-07
LGR5	-2.24013	0.000108
LHFPL2	0.463457	3.77E-15
LHX1	0.647679	0.005646
LHX2	0.877496	0.031023
LHX9	1.042641	0.036251
LIF	0.444856	0.04264
LIG1	0.354756	0.000833
LIG3	0.240256	1.90E-05
LIG4	-0.45798	7.41E-05
LIMA1	0.15268	0.010434
LIMCH1	-0.85897	1.30E-13
LIMK2	-0.30755	2.32E-05
LIMS2	-0.55003	0.004979
LIN28B	0.538602	0.003499
LINC00205	-0.3571	0.0011
LINC00294	-0.40739	1.66E-05
LINC00314	1.231261	0.026658
LINC00337	0.901349	0.016662
LINC00342	-0.40011	0.008733
LINC00346	-0.55764	6.43E-08
LINC00365	-0.50719	0.044434
LINC00472	0.622483	0.00083
LINC00484	-1.79438	0.021935
LINC00504	-0.33911	0.000678
LINC00511	-0.34046	0.000323

LINC00519	-0.47247	0.000474
LINC00520	1.199398	0.000967
LINC00578	-1.89037	0.022309
LINC00626	-1.69207	0.002902
LINC00638	-0.3941	0.022271
LINC00640	-1.07295	0.00029
LINC00641	-0.29141	0.003843
LINC00649	-0.32263	4.00E-06
LINC00665	-0.36836	0.041863
LINC00667	-0.2042	0.00016
LINC00674	-0.56877	0.00074
LINC00675	-3.31844	0.042276
LINC00707	0.92463	2.64E-21
LINC00863	-0.42773	9.39E-05
LINC00865	-0.60074	6.63E-09
LINC00882	-0.4264	0.019589
LINC00884	-0.69459	0.00729
LINC00885	-1.19399	0.0045
LINC00886	-0.43825	1.17E-05
LINC00888	-0.33236	3.96E-06
LINC00893	-0.64051	0.002444
LINC00898	-0.96013	0.024006
LINC00909	-0.29026	0.002832
LINC00941	0.51379	3.18E-06
LINC00954	-0.83215	0.009674
LINC00958	0.169398	0.020552
LINC00960	-0.8831	1.05E-05
LINC00963	-0.20804	0.002754
LINC01003	-0.68476	4.73E-05
LINC01010	-0.50968	0.00972
LINC01011	-0.5539	0.010237
LINC01021	-0.59541	9.09E-05
LINC01089	-0.31032	0.014706
LINC01116	0.268113	0.029996
LINC01123	0.351413	0.040514
LINC01133	-0.67489	0.007843
LINC01138	-0.26779	0.019748
LINC01139	-0.30004	0.015958
LINC01154	2.917649	0.032079
LINC01204	0.684586	0.020017
LINC01220	-1.37204	0.016807
LINC01232	-0.24125	0.015379
LINC01285	-0.90953	0.000573
LINC01297	-0.45783	0.03757
LINC01322	0.455092	0.000658
LINC01341	-1.07387	0.000188
LINC01345	-2.80456	0.006589

LINC01347	-0.3307	0.023902
LINC01348	-0.53824	0.039136
LINC01355	-0.41643	0.007791
LINC01356	0.464108	0.00202
LINC01376	-0.5283	0.01051
LINC01410	-0.28353	0.02615
LINC01451	-1.22621	0.034815
LINC01460	-0.71973	0.014976
LINC01468	0.352532	0.007388
LINC01474	2.165009	0.046322
LINC01508	-0.61774	0.048163
LINC01537	0.695917	0.001136
LINC01551	1.278283	0.012941
LINC01588	-0.31872	0.002912
LINC01605	0.331664	0.000308
LINC01609	-0.48467	0.005303
LINC01649	-1.55203	0.020345
LINC01653	-0.96733	0.031426
LINC01719	-0.45818	0.006136
LINC01722	-0.42975	0.025094
LINC01727	-1.52156	0.009785
LINC01748	-0.39409	0.0008
LINC01762	-0.68787	0.048161
LINC01833	-0.70719	0.000815
LINC01873	-0.30018	0.006521
LINC01943	-0.678	0.048932
LINC01979	-1.23335	0.011731
LINC02015	-1.08207	8.06E-05
LINC02031	-1.31176	0.043226
LINC02035	-0.76949	1.24E-07
LINC02068	-1.51818	9.98E-05
LINC02154	1.061303	7.69E-05
LINC02178	0.959478	6.76E-05
LINC02186	-0.95369	0.045202
LINC02187	-1.6323	0.000155
LINC02198	-1.83506	0.022401
LINP1	0.272413	0.004741
LIPA	-0.37038	5.66E-05
LIPE	0.451707	0.048235
LITAF	-0.28266	0.00089
LL0XNC01-[illegible]	-0.37471	0.028867
LL22NC03-[illegible]	-0.29917	0.000297
LLGL1	0.349322	0.017593
LLNLR-222[illegible]	-0.38567	0.04238
LLNLR-307[illegible]	2.781687	0.04467
LMAN2L	-0.19782	0.00031
LMBRD1	-0.30584	0.00056

LMBRD2	-0.3968	7.15E-05
LMLN	-0.22473	0.001938
LMNA	0.316365	0.029248
LMNB2	0.383974	0.00347
LMO4	-0.21938	4.28E-05
LMO7	-0.5538	8.20E-08
LMOD1	-1.95931	3.33E-15
LOXL1	-0.52511	0.00801
LOXL3	0.243636	0.048463
LPAR1	0.266292	7.24E-05
LPAR3	0.251864	0.001279
LPAR6	-0.52348	3.78E-05
LPCAT1	0.196299	0.002059
LPCAT4	0.569297	3.04E-08
LPGAT1	-0.16969	0.019243
LPP	-0.41785	1.17E-05
LPP-AS2	-0.52748	0.005907
LPXN	0.751365	4.67E-07
LRCH3	-0.12897	0.027248
LRP11	-0.23923	0.029227
LRP5L	0.243737	0.014446
LRPPRC	0.26902	0.000197
LRR1	0.196756	0.021828
LRRC17	-0.59355	7.81E-05
LRRC27	-0.42527	2.52E-05
LRRC3	-0.2455	0.040379
LRRC32	-1.38482	5.49E-07
LRRC37A16	-0.42795	0.003733
LRRC37A3	-0.4543	3.70E-05
LRRC37B	-0.20364	0.023514
LRRC55	-3.02821	0.016884
LRRC57	-0.1471	0.031947
LRRC59	0.234589	1.53E-05
LRRC7	-1.17674	3.05E-39
LRRC75A-A	0.175742	0.001451
LRRC8A	0.447105	2.00E-05
LRRC8C	0.611559	6.66E-09
LRRC8D	0.107393	0.03242
LRRK1	0.199273	0.000381
LRRN1	-0.74985	0.048897
LRWD1	0.609079	9.31E-05
LSG1	0.176994	5.66E-05
LSM12	0.178805	0.006927
LSM12P1	0.306252	0.00326
LSM2	0.288206	0.011029
LSM5	0.165223	0.02496
LSM6	0.228527	0.014557

LSM7	0.329067	0.001679
LSP1	-0.35253	0.014038
LTA4H	0.155501	0.010662
LTB	-1.0725	1.56E-08
LTBP1	0.504908	1.18E-15
LTV1	0.194898	0.007097
LUC7L	0.268984	0.000132
LUM	-1.52243	5.72E-08
LURAP1L	-0.64678	0.001922
LVCAT1	-0.99414	7.18E-05
LY6K	0.349049	0.00132
LY75	-0.27643	0.025016
LYAR	0.549294	2.80E-10
LYN	-0.49546	4.10E-05
LYNX1	-0.61276	0.020674
LYPLA2	0.249909	0.020567
LYPLAL1	-0.24182	0.003395
LYRM4	0.185262	0.011829
LYRM9	-0.4686	0.019534
LZIC	0.128977	0.047523
LZTS1	0.506359	0.000169
MAB21L3	-0.50017	1.28E-05
MACC1	-0.67287	0.000234
MACROD2	-0.64441	0.000234
MAD1L1	0.484809	0.000511
MAD2L1	0.35421	4.46E-05
MAD2L2	0.436568	0.000976
MAFA-AS1	-1.28329	0.017103
MAFB	-1.20038	7.64E-07
MAFF	0.746804	1.19E-12
MAGED1	-0.17621	0.024296
MAGT1	-0.21287	0.005364
MAIP1	0.239856	5.60E-05
MAK16	0.266129	0.000844
MALAT1	-0.28392	0.01241
MALL	1.109519	2.22E-76
MALSU1	0.17208	0.001552
MAMDC2	-0.44703	0.000722
MAMDC2-A	-0.71515	0.004151
MAN1A2	-0.15097	0.039204
MAN2B2	-0.33722	0.003833
MAN2C1	0.273419	0.041366
MANBA	-0.57168	8.58E-11
MANSC4	-2.60383	0.046724
MAOB	1.055788	3.15E-13
MAP10	-0.4034	0.006659
MAP1B	-0.20097	0.009199

MAP1LC3A	-0.45466	0.00442
MAP2K1	0.330222	7.91E-11
MAP2K3	0.376138	0.000447
MAP2K4	0.15053	0.01678
MAP2K5	-0.16531	0.034817
MAP2K6	-0.82309	0.001691
MAP2K7	0.327051	0.000516
MAP3K13	-0.20586	0.000789
MAP3K14	0.490613	0.000239
MAP3K2	-0.20305	0.028419
MAP3K20	0.14915	0.020858
MAP3K4	-0.1499	0.005808
MAP3K5	-0.39782	0.00941
MAP3K8	-0.33602	0.031004
MAP4K4	0.48096	1.19E-09
MAP6	-0.71349	0.002946
MAP6D1	0.250397	0.038389
MAP7D2	-1.15033	0.000259
MAPK10	-0.59531	0.000104
MAPK13	-0.24596	0.042467
MAPK14	-0.19924	8.07E-06
MAPKAPK2	0.149166	0.030087
MAPKAPK3	0.545083	6.10E-13
MAPKAPK5	0.157147	0.000992
MAPKAPK5	0.14217	0.048402
MAPKBP1	0.317999	5.47E-05
MAPRE2	0.301221	1.17E-05
MAPRE3	-0.26657	0.006552
2-Mar	-0.60381	2.91E-05
2-Mar	-0.45379	0.003257
4-Mar	1.018324	1.09E-06
8-Mar	-0.16249	0.0173
9-Mar	-0.38312	0.005771
MARCKS	-0.46447	2.91E-10
MARCKSL1	0.44572	0.001774
MARK1	-0.49039	1.76E-13
MARK2P16	3.165082	0.039378
MARK4	0.280672	0.040705
MARS	0.220326	0.000535
MARS2	0.30914	5.79E-05
MARVELD2	-0.22399	0.000739
MARVELD3	-0.6501	2.35E-09
MASP1	-0.67136	0.034889
MATN1-AS	0.316657	0.048917
MATN2	-1.33965	1.01E-10
MAVS	-0.07561	0.035992
MBD1	0.215751	0.001402

MBD6	0.299055	0.022882
MBNL1-AS1	-1.03798	9.30E-13
MBNL2	-0.27143	0.019916
MBOAT1	0.209306	0.005662
MBOAT2	0.577084	4.95E-16
MBOAT7	0.295879	0.049133
MBTPS1	-0.19468	0.000509
MCAM	-0.48041	4.84E-06
MCAT	0.269026	0.013797
MCCC1	-0.11892	0.039518
MCCC2	0.12073	0.02036
MCEE	-0.22033	0.019291
MCF2L	0.446654	0.034392
MCF2L2	-0.31059	0.028915
MCFD2	0.134088	0.048817
MCIDAS	0.620404	0.008588
MCM10	0.413256	1.23E-06
MCM2	0.322645	0.003303
MCM3AP	0.114464	0.013874
MCM4	0.299367	1.17E-05
MCM5	0.279068	0.014016
MCM6	0.103769	0.03647
MCM7	0.36924	1.19E-06
MCM8	0.1834	0.025875
MCMBP	0.234104	2.59E-08
MCMDC2	-0.49992	0.014905
MCOLN3	-0.20768	0.040593
MCRIP1	0.333136	0.029125
MCRS1	0.214785	0.011036
MCUB	-0.38312	0.012486
MDC1	0.291523	0.001935
MDFI	0.55093	0.006536
MDH2	0.224876	0.017485
MDN1	0.232803	1.36E-05
ME1	-0.23748	0.00497
ME2	0.231047	0.000435
ME3	0.931991	0.000554
MEA1	0.223556	0.003064
MED1	0.150481	0.035686
MED15	0.266739	0.02566
MED22	0.269972	0.032644
MED24	0.180358	0.037668
MED27	0.183818	0.000331
MED8	0.188914	0.000314
MEG3	0.670928	2.63E-17
MEG8	4.210708	0.002032
MEGF9	-0.47579	1.48E-08

MEIS1	-0.25639	0.003457
MEIS2	-0.23593	0.012716
MELK	0.227796	0.005068
MEMO1	0.240892	0.00015
MEMO1P1	-1.28558	0.004016
MERTK	0.712767	2.86E-06
MEST	-0.33229	0.000244
MET	0.218446	0.000952
METAP1	0.217634	0.000566
METAP1D	-0.23218	0.003523
METAP2	0.222732	0.003146
METRNL	0.431709	0.039123
METTL1	0.261827	0.003134
METTL12	0.276656	0.014757
METTL16	0.276629	2.96E-09
METTL17	0.129831	0.003168
METTL21B	0.237956	0.01372
METTL25	-0.47211	7.67E-06
METTL7A	-1.13819	6.58E-17
METTL9	0.170842	0.001544
MFAP3L	-0.43394	0.037876
MFAP5	-0.30918	0.049882
MFN2	0.205192	0.000372
MFNG	0.450168	0.030055
MFSD1	-0.30587	3.32E-06
MFSD10	0.462358	0.016418
MFSD11	-0.1865	0.005387
MFSD14A	-0.16292	0.028057
MFSD2A	0.568251	4.54E-08
MFSD6	-0.87185	3.82E-09
MFSD8	-0.16782	0.039184
MFSD9	-0.22788	0.010745
MGAM	-1.03933	0.000105
MGAT4A	-0.66196	0.027212
MGAT5	0.218713	1.34E-05
MGC32805	1.452479	1.60E-06
MGST2	-0.17358	0.027424
MGST3	-0.14261	0.012704
MICAL2	0.303249	4.00E-05
MID1	-0.20669	0.000373
MID1IP1	0.337964	0.023757
MID2	-0.17293	0.015138
MIDN	0.440117	0.002727
MIGA1	-0.16745	0.033827
MIGA2	0.276279	0.015193
MIIP	0.596335	7.00E-05
MINDY1	-0.31114	0.001089

MINPP1	-0.13666	0.017629
MIPEP	0.22844	0.001362
MIR100HG	0.661153	1.09E-06
MIR193BH(	-0.41617	0.00615
MIR210HG	-0.40359	0.028556
MIR22HG	-0.55543	9.17E-07
MIR27B	-1.31358	0.001146
MIR31HG	0.362623	0.004769
MIR331	-2.03394	0.017491
MIR4292	0.616225	0.03133
MIR4435-2	0.332486	0.000263
MIR4458H(	-0.1938	0.044927
MIR503HG	-0.46667	0.018766
MIR570	-0.33451	0.021709
MIR635	-0.7981	0.046886
MIRLET7BH	-0.55839	0.000551
MIRLET7D	-0.56558	0.027139
MIS18A	0.255045	0.000133
MIS18BP1	0.178093	0.032913
MISP	0.423259	0.00498
MITF	-0.36594	0.011899
MKI67	0.255238	0.015185
MKL2	-0.14134	0.007078
MKNK2	0.507233	0.000133
MLKL	0.280617	1.28E-05
MLLT1	0.366836	0.014308
MLLT3	-0.27816	0.001874
MLST8	0.533082	0.002834
MLXIPL	-0.5555	0.00529
MLYCD	-0.33092	0.000611
MMACHC	0.210336	0.008203
MME	-0.87907	5.89E-06
MME-AS1	-1.33617	0.022121
MMP12	-1.56342	0.008383
MMP13	-0.5206	1.86E-05
MMP14	-0.28382	0.001582
MMP15	-0.53995	0.000445
MMP2	-0.30407	0.010171
MMP24	-0.56571	0.016053
MMP28	-0.44969	0.029911
MMP3	1.479932	4.93E-10
MMP7	-0.98055	0.000111
MMP9	1.568207	5.28E-05
MMS19	0.327821	5.13E-10
MMS22L	0.186719	0.013269
MOAP1	-0.33283	1.28E-05
MOB1A	-0.13967	0.049921

MOB3A	0.228438	0.032737
MOB3C	-0.20614	0.035255
MOCS1	0.305482	0.009698
MOK	0.543728	6.90E-07
MON1A	0.392868	0.008669
MON1B	-0.13892	0.023206
MORC2	0.098139	0.032576
MORN4	-0.2248	0.006559
MOSPD1	-0.3554	0.000448
MOV10L1	-0.45044	0.003812
MOXD1	-1.11648	1.17E-08
MPDU1	0.273536	0.003239
MPI	0.139621	0.022128
MPP2	0.506521	0.004896
MPP3	0.309514	0.008146
MPP4	-1.30411	0.002087
MPP6	0.557349	3.83E-12
MPP7	-0.31464	6.50E-05
MPPE1	-0.29943	5.73E-06
MPRIP	0.529725	2.01E-11
MPV17	0.184362	0.019993
MPZL1	-0.1056	0.022936
MR1	-0.55576	5.57E-14
MRAS	-1.09254	1.98E-11
MRFAP1	0.100975	0.02755
MRFAP1L1	-0.12388	0.048437
MRGBP	0.291402	0.00149
MRM3	0.315949	0.00145
MROH7	-1.06418	0.00248
MROH8	-0.72342	0.001755
MRPL1	0.268093	0.000211
MRPL10	0.31645	0.000409
MRPL11	0.221456	0.027236
MRPL12	0.396268	0.016915
MRPL14	0.169786	0.008929
MRPL15	0.22315	8.62E-05
MRPL16	0.28478	1.88E-06
MRPL17	0.24654	0.010942
MRPL18	0.197043	0.000321
MRPL2	0.34377	0.000832
MRPL20	0.22667	0.00178
MRPL21	0.25502	0.000638
MRPL22	0.16791	0.019189
MRPL23	0.317306	0.011566
MRPL24	0.203388	0.000996
MRPL27	0.230985	0.000251
MRPL28	0.318072	0.008112

MRPL3	0.270694	3.15E-05
MRPL32	0.214899	0.001053
MRPL36	0.372866	0.000953
MRPL37	0.33029	0.000311
MRPL38	0.331415	0.030811
MRPL39	0.18101	0.037082
MRPL4	0.434134	0.027669
MRPL42	0.191151	0.020513
MRPL45	0.191315	0.000572
MRPL46	0.193186	0.00185
MRPL47	0.150099	0.048939
MRPL48	0.124594	0.031836
MRPL49	0.17727	0.015671
MRPL53	0.224704	0.042556
MRPL58	0.291679	1.50E-05
MRPL9	0.289038	4.11E-05
MRPS10	0.239978	5.32E-06
MRPS11	0.198415	0.031004
MRPS12	0.362752	0.018343
MRPS15	0.186992	0.006198
MRPS16	0.13369	0.022848
MRPS18A	0.30873	0.001757
MRPS18B	0.303192	3.81E-05
MRPS2	0.549823	5.09E-05
MRPS22	0.194811	0.001201
MRPS23	0.225044	0.002217
MRPS25	0.246934	0.000174
MRPS27	0.202648	2.29E-06
MRPS28	0.20031	0.003672
MRPS30	0.249073	1.08E-05
MRPS35	0.194675	0.000684
MRPS5	0.201623	0.001247
MRPS6	0.402341	1.46E-09
MRPS7	0.237692	0.003405
MRRF	0.176755	0.002119
MRTO4	0.433148	3.23E-07
MRVI1	-0.87789	0.001037
MSANTD3	0.186569	0.00716
MSC-AS1	-0.22388	0.005515
MSH2	0.205828	0.008378
MSH5	0.330656	0.000222
MSI2	-0.27337	0.000151
MSRB2	-0.44296	0.000594
MSRB3	-0.47526	2.98E-09
MST1R	0.474491	6.21E-05
MSTO1	0.221402	0.021598
MSX1	0.358701	0.022778

MT1E	0.645834	5.12E-07
MT1L	1.018581	6.39E-10
MT1X	0.406437	2.52E-05
MT2A	1.031482	1.07E-12
MTA1	0.389543	0.006131
MTA2	0.200017	0.000895
MTAP	0.503583	3.60E-11
MTCH2	0.116363	0.040941
MT-CO1	-0.18941	0.018698
MTERF2	-0.23655	0.003388
MTERF3	0.225036	0.014787
MTFP1	0.46896	0.000182
MTFR1L	-0.22042	0.002052
MTFR2	0.288363	0.000483
MTG1	0.327058	0.002962
MTHFD1	0.255012	3.90E-06
MTHFD1L	0.482094	8.71E-14
MTHFD2	0.231535	0.025126
MTMR11	0.49346	1.28E-05
MTMR3	-0.23526	0.023074
MT-ND1	0.211351	0.040264
MT-ND2	0.30851	0.003336
MTND4P23	-3.31201	0.018308
MT-ND5	-0.20837	0.000772
MT-ND6	-0.24479	0.020208
MTOR	0.121501	0.007046
MTPAP	0.159203	0.035811
MTRF1	-0.20454	0.013137
MT-RNR2	-0.31967	2.65E-07
MTRR	0.197414	0.001792
MTSS1	0.388466	0.000252
MT-TA	-0.43311	0.000668
MT-TG	-0.41204	0.017083
MT-TN	-0.4565	0.021282
MT-TP	-0.33568	0.004333
MTURN	0.306655	0.016379
MTUS1	-0.3314	1.98E-05
MTUS2	-1.14932	9.20E-08
MUC1	-0.96784	0.014048
MUC16	-1.76661	0.000148
MUC2	2.105361	0.011588
MUC20	-1.02902	0.000272
MUC4	-1.14677	0.001337
MUCL1	2.974817	0.025023
MUS81	0.232078	0.040158
MUTYH	0.402846	0.000557
MVP	-0.28341	0.042805

MXD4	-0.32996	0.014598
MXI1	-0.15463	0.035111
MXRA5	-1.17758	1.18E-23
MXRA5Y	-1.15509	9.30E-06
MXRA7	-0.1786	2.07E-05
MYB	0.459021	0.013062
MYBBP1A	0.547452	8.92E-06
MYBL2	0.270605	0.008274
MYBPC2	-0.535	0.048083
MYC	1.048231	6.72E-21
MYCL	-0.93356	3.95E-12
MYEF2	0.355783	0.045139
MYEOV	0.730904	5.27E-06
MYH10	0.173584	0.009554
MYH14	-0.43565	0.007765
MYH15	1.228371	3.63E-08
MYH16	0.730068	4.29E-11
MYH3	-0.39605	0.000189
MYL12A	-0.37447	3.66E-06
MYL2	-0.9897	9.15E-05
MYL6	-0.43373	8.83E-09
MYL6B	0.216681	0.029505
MYL7	-2.2241	5.67E-07
MYL9	-1.38446	6.78E-15
MYLIP	-0.52679	0.005563
MYLK	-0.64138	9.08E-10
MYLK3	-0.42358	0.019031
MYO10	0.604106	8.10E-34
MYO16	-0.52954	0.000283
MYO16-AS	-1.16334	0.000234
MYO19	0.317365	6.49E-06
MYO3B	-0.97436	7.96E-21
MYO5C	-0.58891	0.018628
MYO7B	0.611743	7.38E-05
MYOM1	-1.21904	5.21E-05
MYOM2	-0.56059	5.03E-05
MYOSLID	0.358934	4.31E-05
MYPN	0.452408	0.011335
MYRF	0.631178	3.76E-05
MYRIP	-3.4842	0.013842
MZF1-AS1	-0.42069	0.035623
N4BP2L1	-0.66754	0.000165
N4BP2L2	-0.22395	0.005185
N4BP3	0.315349	0.017832
NAA10	0.303746	0.002445
NAA15	0.292285	4.28E-05
NAA25	0.168208	0.024759

NAALAD2	-0.61157	0.01593
NAB2	0.652852	4.37E-10
NABP1	-0.55809	5.14E-08
NACA	0.202151	8.13E-05
NACAD	0.469329	0.01082
NACC1	0.323283	0.026474
NADSYN1	-0.35422	0.000741
NAE1	0.159661	0.012764
NAF1	0.255415	0.001353
NAGS	0.659408	5.77E-06
NAIF1	0.210484	0.014212
NAIP	-0.30313	0.024928
NALCN	-0.41355	0.000286
NAMPT	0.225858	0.033565
NANOS1	-0.28387	0.025302
NANS	0.244143	5.33E-05
NAP1L1	0.281152	3.85E-05
NAP1L2	-0.36657	3.38E-05
NAP1L4	0.118251	0.005115
NAP1L5	-0.49988	0.008263
NAPB	-0.23886	0.012663
NAPEPLD	-0.26232	0.014003
NARS2	0.279991	1.60E-05
NASP	0.19498	0.003913
NAT1	-0.28929	0.014482
NAT10	0.398215	8.32E-11
NAT9	0.223318	0.005131
NATD1	-0.22681	0.043422
NAV2	-0.39886	1.02E-07
NAV3	1.055934	8.08E-31
NBDY	-0.1912	0.003989
NBEAL1	-0.39408	0.001863
NBEAL2	0.308457	0.03656
NBL1	0.339636	0.040551
NBPF12	-0.29344	4.26E-05
NBPF14	-0.14224	0.023932
NBPF19	-0.20808	0.007015
NBPF20	-0.19972	0.033549
NBR2	-0.30468	0.005077
NCAPD2	0.300473	5.20E-05
NCAPD3	0.290832	5.72E-08
NCAPG	0.322225	0.001139
NCAPG2	0.193811	0.007107
NCAPH	0.377671	4.20E-08
NCAPH2	0.476696	3.80E-05
NCBP1	0.188355	0.000869
NCBP3	0.124794	0.038475

NCEH1	0.155664	0.041215
NCF2	-0.35813	0.004944
NCK1-AS1	-0.24909	0.040646
NCKAP5	-0.46308	0.000173
NCKIPSD	0.314118	0.007037
NCL	0.301082	3.99E-05
NCOA1	-0.34673	0.000298
NCOA2	-0.24498	2.12E-07
NCSTN	-0.23387	0.000291
NDC1	0.320241	0.00011
NDC80	0.279931	0.022838
NDE1	0.181325	0.004085
NDFIP1	-0.15402	0.033379
NDFIP2	-0.30593	0.002484
NDRG2	0.172783	0.003523
NDRG3	-0.12745	0.00921
NDST1	-0.2853	0.00132
NDUFAB1	0.224967	0.018665
NDUFAF2	0.25982	0.003845
NDUFAF4	0.245207	0.012276
NDUFAF8	0.351701	0.049472
NDUFB1	-0.12692	0.031112
NDUFB8	0.194107	0.01595
NDUFB9	0.220705	6.63E-06
NDUFC1	0.184389	0.029445
NDUFS2	0.09634	0.033737
NDUFS3	0.23989	0.003248
NDUFV1	0.279108	0.01142
NDUFV2	0.14976	0.001963
NEBL	-0.3143	0.014013
NECTIN1	0.361659	0.000773
NECTIN3	0.343867	0.005476
NEDD1	-0.18716	0.046345
NEDD4L	0.200772	0.017442
NEDD9	-0.34212	2.06E-07
NEFL	-0.9151	1.56E-13
NEIL3	0.200533	0.007821
NEK11	-0.22904	0.028935
NEK2	0.2625	0.014468
NEK9	-0.13928	0.001764
NELFA	0.313924	0.033808
NELFB	0.296758	0.023205
NELFCD	0.273575	2.31E-07
NEMP2	0.401808	1.79E-09
NEO1	0.482826	7.94E-06
NES	0.530188	1.88E-05
NET1	0.194807	0.003322

NETO2	0.235045	0.00028
NEURL1	1.306379	4.84E-08
NEURL1B	-0.62736	1.59E-15
NEURL3	-0.797	0.014926
NEURL4	0.315086	0.000986
NEUROG2	-1.47937	0.018478
NEXN	-0.74601	9.02E-12
NFAM1	-0.71655	0.006344
NFATC2	0.85016	3.99E-15
NFE2L3	0.190496	0.012098
NFKBIE	0.28739	0.012281
NFRKB	0.158133	0.0212
NFS1	0.217331	0.000905
NFX1	0.195453	0.001176
NGDN	0.215076	0.000299
NGEF	0.901793	4.69E-11
NGFR	0.790971	2.75E-06
NHEJ1	0.159486	0.017499
NHLH2	-0.91816	4.05E-11
NHLRC3	-0.42646	3.63E-06
NHP2	0.331161	0.000973
NHSL1	0.168871	0.012556
NHSL2	0.318568	2.41E-05
NICN1	-0.34113	0.012239
NID2	0.418516	0.000255
NIF3L1	0.165026	0.016466
NIFK	0.18858	0.000534
NIFK-AS1	-0.48067	0.007976
NIN	0.190721	0.005988
NINJ1	-0.49663	0.009212
NIP7	0.270252	0.000962
NIPA1	-0.23274	0.02707
NIPAL1	0.401063	1.78E-05
NIPAL2	-0.20165	0.042692
NIPSNAP3A	-0.27803	2.29E-05
NKD2	0.482126	0.041262
NKRF	0.235797	0.008155
NKX2-2-AS1	-4.12565	0.012683
NLE1	0.568745	4.98E-12
NLN	0.223937	0.000754
NLRP10	1.020014	7.61E-08
NLRP3	0.537849	0.000952
NLRP6	-1.27341	0.005302
NLRP7	-0.67499	0.001392
NME1	0.455088	1.52E-08
NME2	0.290854	0.000755
NME9	-0.80437	0.022872

NMI	-0.27979	0.013265
NMRK1	-0.27195	0.000904
NNMT	-0.4578	6.18E-05
NNT	-0.18084	0.002581
NOB1	0.433148	6.29E-15
NOC2L	0.454742	2.46E-05
NOC3L	0.326076	0.001432
NOC4L	0.457665	0.019123
NOD1	0.17847	0.036897
NOD2	-0.64971	0.000234
NOL10	0.223515	0.000304
NOL11	0.208079	0.002415
NOL12	0.290817	0.002436
NOL6	0.55562	6.91E-08
NOL7	0.225858	7.34E-05
NOL8	0.130854	0.042568
NOL9	0.143071	0.021436
NOLC1	0.417982	1.52E-14
NOM1	0.14773	0.044524
NONO	0.154327	6.39E-05
NOP10	0.224061	4.52E-06
NOP14	0.359329	6.08E-13
NOP16	0.468703	2.38E-11
NOP2	0.360963	1.89E-07
NOP56	0.354487	3.04E-05
NOP58	0.226448	0.000253
NOP9	0.124091	0.00625
NORAD	-0.25733	0.000237
NOS2P3	0.785123	0.047417
NOV	0.277261	0.000598
NOXA1	-0.69588	0.032091
NPB	-2.09558	0.020274
NPC1	0.253432	0.014101
NPC1L1	-1.63541	0.023307
NPHP3	-0.15133	0.038052
NPHP4	0.258796	0.01934
NPIPA2	-1.41099	0.002331
NPL	-0.39775	0.02707
NPM1	0.381209	3.12E-05
NPM2	-0.56488	0.022336
NPM3	0.58652	1.54E-08
NPNT	-0.86091	0.012894
NPR3	0.91651	2.95E-14
NPRL3	0.363964	0.003194
NPSR1-AS1	-0.68041	0.041625
NPTN-IT1	0.332512	0.010709
NPTX1	0.925927	0.003116

NPTXR	-0.42587	8.51E-05
NQO1	-0.16709	0.000247
NR1D1	0.648988	2.31E-06
NR2C2AP	0.291962	0.000411
NR2E1	-0.54365	1.64E-06
NR3C1	0.206903	0.01617
NR4A1	0.501513	0.000814
NRBP2	-0.45203	0.000219
NRCAM	-0.46077	7.67E-06
NREP	-0.39218	1.07E-06
NRG1	0.27527	0.000114
NRG3	0.866984	0.044836
NRG4	-0.53762	5.33E-06
NRGN	0.999937	0.004034
NRIP3	0.251494	0.006207
NRM	0.248934	0.041345
NRP2	0.33141	0.000236
NRXN2	-0.29818	0.021048
NSD1	-0.15866	0.001222
NSD2	0.321225	1.06E-06
NSMF	0.424028	0.005052
NSUN2	0.275595	2.12E-10
NSUN5	0.255574	0.021997
NT5C2	-0.36656	1.38E-05
NT5DC1	-0.18093	0.001571
NT5DC3	0.359879	4.90E-07
NT5E	0.619332	1.61E-12
NTF3	0.557009	0.001697
NTHL1	0.427825	0.01437
NTMT1	0.482192	1.34E-08
NTN1	-0.83938	0.001129
NTN4	-0.54992	2.44E-11
NTN5	-0.85113	0.015651
NTNG1	0.497752	0.00242
NTRK1	1.054501	0.020818
NTSR1	2.218346	0.000812
NUB1	-0.26105	0.001694
NUBP1	0.205839	0.00793
NUCB2	-0.18831	0.024653
NUDC	0.311753	0.000912
NUDCD1	0.21267	0.019166
NUDCD2	0.20243	0.001338
NUDT1	0.394624	0.000249
NUDT11	0.191853	0.035283
NUDT15	0.314271	4.32E-07
NUDT16	-0.32538	2.75E-05
NUDT21	0.139227	0.033225

NUDT4P1	1.483783	0.014446
NUDT4P2	0.265758	0.004902
NUDT6	-0.35934	0.020748
NUDT7	-0.25352	0.040075
NUF2	0.185507	0.033872
NUFIP1	0.243656	0.00522
NUMBL	0.362152	0.030564
NUP107	0.214038	0.002911
NUP153	0.198621	0.000305
NUP155	0.220585	0.000514
NUP160	0.17988	0.006092
NUP188	0.358317	7.78E-09
NUP205	0.23428	0.000535
NUP214	0.135651	0.019039
NUP35	0.314859	4.71E-05
NUP37	0.165282	0.02101
NUP50	0.12271	0.020216
NUP58	0.131342	0.029135
NUP62	0.239275	0.029312
NUP62CL	0.541005	7.79E-06
NUP85	0.157817	0.002287
NUP88	0.315941	7.23E-09
NUP93	0.372485	1.94E-10
NUP98	0.177333	3.67E-05
NUPR1	-0.50885	0.005504
NUTF2	0.186636	0.008236
NUTM2D	-0.42926	0.002162
NWD1	-0.86689	0.000186
NXF1	0.182197	0.002609
NXPE3	-0.30277	2.45E-09
NXT1	0.253146	0.006336
NYNRIN	-0.44354	0.009876
OACYLP	-2.79318	0.021597
OAF	0.789164	5.67E-10
OAZ1	0.225772	0.01042
OCIAD1	-0.12534	0.037512
OCIAD2	0.350246	0.000223
OCLN	-0.55753	0.01943
ODAM	-2.1267	0.000914
ODC1	0.801113	2.32E-18
ODF2	0.318201	4.90E-08
OGDHL	0.779424	0.035721
OIP5	0.306021	0.000514
OIP5-AS1	-0.23095	0.001484
OLA1	0.21225	0.005179
OLFML2A	-0.32533	0.005009
OLFML2B	-0.6812	0.002286

OLFML3	-0.77618	8.22E-08
OLMALINC	0.534077	1.86E-07
OLR1	-1.03992	0.00357
OPHN1	-0.21108	0.007106
OPN4	-3.45349	0.010796
OPRD1	-0.60666	0.032383
OPTN	-0.30114	0.000527
OR11H4	-0.75053	0.04642
OR2A1-AS1	-0.39583	0.006765
OR2A9P	-0.56944	0.005953
OR4A14P	1.793311	0.03423
OR7E14P	1.837432	0.009049
OR8R1P	-1.15856	0.016983
ORAI2	0.372536	0.000935
ORAI3	-0.61695	7.29E-06
ORAOV1	-0.11868	0.041406
ORC1	0.435273	6.97E-05
ORC4	-0.21526	0.00253
ORC6	0.155037	0.021901
ORMDL3	-0.26815	7.40E-07
OS9	-0.18877	0.001457
OSBP2	0.264816	0.048208
OSBPL10	0.19567	0.000276
OSBPL1A	-0.16305	0.0179
OSBPL6	0.322506	0.005701
OSGEP	0.1987	0.000482
OSGEPL1	-0.18617	0.046866
OSTM1-AS1	-1.61034	0.016699
OTUD1	-0.37892	0.018071
OVCH2	-0.66279	0.000292
OVGP1	-0.88536	0.003897
OVOL1	0.732007	0.002551
OXA1L	0.129655	0.025041
OXNAD1	0.2846	0.005568
OXSR1	0.209935	0.001748
OXTR	0.439795	0.005966
P2RX4	-0.48749	2.21E-05
P2RX5	0.790179	8.22E-07
P2RX6	-0.72689	0.000367
P2RX6P	-2.20997	0.026726
P2RX7	-0.64983	5.45E-08
P2RY1	-0.31239	0.040297
P2RY6	-0.57188	0.000881
P3H2	-0.24572	0.000901
P4HA2	-0.22332	0.008791
P4HTM	-0.54551	0.009994
PA2G4	0.355286	5.41E-06

PABPC1	0.221648	1.04E-06
PABPC1L	0.451661	3.74E-05
PABPC4	0.333509	5.44E-07
PADI1	0.634051	0.000871
PAF1	0.158873	0.029902
PAFAH1B1	-0.12209	0.004876
PAFAH1B3	0.40231	0.011688
PAFAH2	-0.25393	0.000432
PAG1	0.687874	9.46E-18
PAGR1	0.390924	0.015459
PAICS	0.375816	8.75E-07
PAK1	0.151561	0.007631
PAK1IP1	0.34195	2.45E-07
PAK2	-0.13543	0.04994
PAK3	0.504955	0.006169
PAK4	0.399267	0.042038
PALD1	0.471164	0.035393
PALLD	-0.8677	1.94E-23
PALM	-0.89958	0.006528
PALM2-AK/	-0.19263	0.031355
PALMD	-0.78083	2.28E-09
PAM	-0.57973	4.29E-07
PAM16	0.374926	0.004761
PAMR1	-3.1052	3.29E-12
PANK4	0.167594	0.032303
PAOX	-0.30371	0.040005
PAPD4	-0.16448	0.031243
PAPLN	-0.9852	1.86E-10
PAPPA2	0.530749	0.009162
PAPPA-AS1	-0.98088	0.020533
PAQR5	0.608721	6.72E-09
PAQR7	-0.32466	0.001096
PARD3B	-1.01721	0.005283
PARD6B	0.387097	0.026785
PARL	0.137265	0.00872
PARP1	0.175563	0.003108
PARP14	-0.6133	0.035628
PARP1P1	3.560917	0.008555
PARP2	0.149768	0.035027
PARP3	-0.29833	0.004294
PARP4	-0.15188	0.010527
PARP8	-0.19794	0.016097
PARPBP	0.227832	0.040452
PARVB	0.267743	0.004791
PASK	0.284995	0.000539
PATJ	-0.27118	0.001041
PATL1	0.08883	0.039356

PATL2	-0.4697	0.004395
PAX6	-0.29976	0.000742
PAX8-AS1	-0.19264	0.000207
PAX9	0.588123	6.45E-09
PAXBP1	0.280121	0.001638
PAXIP1-AS1	-0.39016	0.004327
PAXIP1-AS2	-0.50125	1.38E-05
PBDC1	0.138306	0.028727
PBLD	-0.34256	0.000223
PBX1	-1.06773	4.34E-10
PBX2	0.196266	0.010579
PBX3	0.499153	1.50E-10
PBXIP1	-0.58262	9.87E-08
PC	0.518116	6.92E-06
PCAT14	-0.35229	0.007962
PCAT7	-0.50848	3.66E-05
PCBP1	0.19614	0.010775
PCBP2	0.171369	0.000428
PCBP3-OT1	1.241922	0.014718
PCCB	0.182212	0.000465
PCDH1	0.463641	1.39E-06
PCDH10	-0.29675	0.00834
PCDH18	-0.643	0.013376
PCDH7	-0.35258	0.001819
PCDHAC2	0.326516	0.014895
PCDHB10	-0.72476	0.00106
PCDHB12	-0.40332	0.008042
PCDHB13	-0.59792	0.004189
PCDHB14	-0.7108	2.22E-10
PCDHB6	-0.45488	0.003209
PCDHB9	-0.83867	7.84E-06
PCDHGA1	-0.85336	1.50E-08
PCDHGA4	-0.39106	0.024718
PCDHGB1	-0.47459	0.000378
PCDHGC5	0.778728	1.75E-07
PCF11	-0.15501	0.036571
PCGF5	-0.26356	0.012838
PCGF6	0.18914	0.010945
PCID2	0.256284	0.000396
PCLAF	0.278763	0.015089
PCLO	-0.35188	0.00012
PCMT1	0.151015	0.001527
PCMTD1	-0.41913	0.000115
PCMTD2	-0.33216	1.56E-07
PCNA	0.236755	4.60E-06
PCNT	0.212373	0.001899
PCOLCE2	0.223235	0.009273

PCOTH	0.626764	0.000194
PCSK6	-0.25115	0.006694
PCTP	-0.26516	0.00073
PCYOX1	-0.45739	3.67E-15
PCYOX1L	-0.30885	0.003631
PCYT1A	-0.13146	0.011669
PDCD11	0.37616	2.15E-08
PDCD1LG2	-0.4069	0.002354
PDCD2	0.180019	0.000678
PDCD2L	0.406989	1.90E-05
PDCD5	0.199833	0.000528
PDE12	0.172674	0.003934
PDE1A	-0.85033	0.017727
PDE2A	0.352134	0.045872
PDE3A	-1.02904	0.001207
PDE5A	-0.69706	2.86E-05
PDE8B	-0.33201	0.007388
PDE9A	0.949436	0.001661
PDGFB	-0.30796	0.010596
PDGFC	-0.41472	1.46E-08
PDGFRA	0.712157	0.004053
PDHA1	0.193545	0.000574
PDIA3	-0.12963	0.005576
PDIA3P1	-0.29092	0.004688
PDK3	-0.26617	0.002464
PDLIM1	-0.36002	0.000547
PDLIM2	0.575974	0.000764
PDP2	0.187961	0.00485
PDRG1	0.151497	0.013103
PDS5B	0.215812	0.013206
PDSS1	0.234632	0.002043
PEA15	0.160622	0.001272
PEAK1	-0.34983	0.000286
PEAR1	0.513393	1.01E-09
PECR	-0.28848	0.040856
PEF1	0.188569	0.009616
PELP1	0.406083	0.000202
PEMT	0.534311	0.002167
PER1	0.457211	0.001605
PER2	0.22114	0.003804
PERP	-0.39031	2.12E-07
PES1	0.39878	1.40E-07
PEX1	-0.29679	0.000252
PEX10	0.32881	0.016387
PEX11B	-0.17147	0.044472
PEX13	-0.35819	9.47E-05
PEX2	-0.29873	0.000106

PEX5	0.241519	1.85E-07
PF4	1.596807	0.048838
PFAS	0.668615	1.05E-07
PFDN1	0.114963	0.036739
PFDN2	0.339926	3.84E-07
PFKM	0.447725	2.85E-11
PFKP	0.318401	0.000637
PGAM5	0.406532	1.61E-09
PGAP1	-0.28539	0.005062
PGAP2	0.200339	0.013554
PGAP3	-0.32799	0.005087
PGBD1	-0.19818	0.033076
PGF	0.94291	2.74E-05
PGGT1B	-0.16366	0.048389
PGM2L1	-0.27856	0.00879
PGPEP1	-0.36875	0.003779
PGRMC2	-0.27657	3.22E-05
PHACTR3	0.571114	0.011943
PHACTR4	-0.23501	0.000186
PHB	0.318913	5.71E-06
PHB2	0.355544	6.63E-05
PHBP12	1.932919	0.011129
PHC1	0.334367	7.89E-05
PHC1P1	0.356174	0.046883
PHC2	0.466805	7.29E-13
PHF10	-0.17804	0.006581
PHF11	-0.35291	0.001557
PHF19	0.473901	9.68E-06
PHF21A	-0.19307	0.006175
PHF21B	0.910297	0.007231
PHF5A	0.330994	2.38E-05
PHGDH	0.24334	0.039602
PHKA2	0.325459	4.14E-08
PHKB	-0.34502	8.61E-05
PHKG1	0.516432	0.022701
PHLDA1	0.615856	4.61E-16
PHLDB2	-0.32489	0.011294
PHLPP2	-0.17498	0.014774
PHOSPHO2	-0.33634	0.048286
PHRF1	0.336418	0.02578
PHTF1	-0.18179	0.043532
PHYH	-0.37604	1.33E-07
PI15	-1.04178	0.00056
PI4K2B	-0.26278	0.005332
PI4KAP1	0.595024	4.74E-06
PI4KAP2	0.437208	0.00289
PID1	0.504956	9.32E-07

PIF1	0.425709	0.02143
PIGB	-0.18834	0.048077
PIGCP1	-0.36567	0.003331
PIGL	0.2283	0.000952
PIGN	-0.22565	0.000651
PIGP	-0.1841	0.030315
PIGV	-0.18666	0.016329
PIGX	-0.19219	0.003566
PIGY	0.89076	0.017286
PIGZ	-0.52701	0.000187
PIH1D1	0.264519	0.001012
PIK3C2A	-0.18477	0.027439
PIK3C2B	-0.45013	0.001838
PIK3CB	-0.22062	0.005356
PIK3IP1	-0.72533	3.48E-13
PIK3R1	-0.18824	0.008974
PIK3R3	-0.32108	0.013352
PIM1	0.709355	1.83E-09
PIM2	0.262288	0.016574
PINK1-AS	-0.19492	0.014427
PINX1	0.348159	6.26E-05
PIP4K2A	0.115446	0.049611
PIP5K1A	0.107809	0.016039
PISD	0.296305	3.66E-05
PITPNA-AS:	0.405373	0.012731
PITPNC1	0.568981	8.30E-13
PITRM1	0.176763	0.000253
PJA1	0.248321	6.39E-07
PJA2	-0.22543	0.039802
PKD2	-0.2077	0.016098
PKDREJ	-0.72844	0.003101
PKHD1	-0.77109	0.002048
PKMYT1	0.489488	0.026735
PKP2	0.267949	1.01E-08
PKP4	0.143437	0.001285
PLA2G1B	-2.56837	0.040556
PLA2G4A	0.53055	1.38E-06
PLA2G4C	-0.72792	1.36E-06
PLA2G4F	-0.66137	0.025568
PLA2R1	-0.55959	4.57E-10
PLAA	0.140576	0.011852
PLAU	0.298438	0.002777
PLAUR	0.463584	0.000113
PLB1	-0.329	0.013223
PLBD1-AS1	-0.72385	0.008434
PLBD2	-0.16836	0.011916
PLCB1	-0.46302	0.000563

PLCB2	0.849978	0.000352
PLCB4	-0.48686	0.000402
PLCE1	-0.40423	0.001044
PLCL1	-1.18392	0.000884
PLD1	-0.7	6.89E-09
PLD6	0.387954	0.000186
PLEC	0.430027	0.046626
PLEK2	0.671327	8.55E-13
PLEKHA1	-0.27123	0.001601
PLEKHA2	-0.28756	3.00E-05
PLEKHA6	0.347143	0.015829
PLEKHB1	-0.72665	0.020179
PLEKHF2	-0.3042	4.79E-06
PLEKHG1	-0.39952	0.00035
PLEKHG2	0.254213	0.030088
PLEKHG3	0.63201	2.70E-09
PLEKHG4	0.55784	0.002987
PLEKHG5	0.638986	2.44E-05
PLEKHH1	-0.26617	0.012276
PLEKHM3	-0.25249	0.013901
PLEKHO1	-0.33663	0.001212
PLIN3	0.276141	0.002253
PLK1	0.540842	7.47E-05
PLK2	-0.13989	0.046318
PLK3	0.708931	1.11E-11
PLK4	0.30612	1.99E-05
PLOD2	-0.44419	0.001293
PLPP1	-0.67499	8.75E-16
PLPP3	-0.2343	0.038684
PLPP4	-0.58214	1.17E-05
PLPP5	0.1225	0.018739
PLPP6	-0.32481	0.001497
PLPPR5	0.991055	0.017918
PLRG1	0.132876	0.017577
PLS1	0.490421	0.021333
PLS3-AS1	-0.74498	0.016107
PLSCR3	0.398793	0.002268
PLSCR4	-0.86817	3.13E-16
PLXDC2	-0.98921	1.48E-14
PLXNA2	0.525208	2.51E-05
PMF1	0.222188	0.007904
PMFBP1	-0.21334	0.01231
PMP22	0.322793	0.031445
PMPCA	0.277777	0.000593
PMS2P6	-0.48745	0.002032
PNKP	0.37741	0.019885
PNMA1	0.223167	0.001275

PNO1	0.324805	1.38E-07
PNP	0.471623	2.51E-16
PNPO	0.195423	0.01002
PNPT1	0.18917	0.010015
PNRC1	-0.30122	0.025235
POC1A	0.215158	0.047092
PODNL1	-0.7507	0.000269
PODXL	0.492001	2.42E-06
POLA1	0.236749	7.93E-05
POLA2	0.201252	0.01509
POLD1	0.448631	0.024635
POLD2	0.503417	1.11E-06
POLDIP2	0.171322	0.026094
POLDIP3	0.207881	0.000126
POLE	0.337477	0.000963
POLE3	0.242177	2.55E-05
POLE4	0.249743	0.018053
POLG	0.32564	3.40E-06
POLI	-0.27747	0.001509
POLR1A	0.222119	0.000437
POLR1B	0.42684	3.57E-12
POLR1C	0.382294	8.43E-10
POLR1D	0.111335	0.023853
POLR1E	0.455444	1.82E-09
POLR2C	0.178322	5.08E-06
POLR2E	0.278476	0.045516
POLR2F	0.226045	0.013219
POLR2H	0.308345	9.97E-08
POLR2I	0.210697	0.025392
POLR2J	0.225375	0.018111
POLR3A	0.218763	5.51E-07
POLR3C	0.116964	0.04116
POLR3D	0.279065	0.000802
POLR3E	0.298644	0.000203
POLR3G	0.711011	8.79E-16
POLR3GL	-0.17907	0.03006
POLR3K	0.377922	3.07E-05
POLRMT	0.478402	0.006758
POM121C	0.212724	0.014153
POMGNT2	0.388131	0.013596
PON2	-0.20312	0.001227
POP1	0.20226	0.001701
POP5	0.153717	0.033532
POP7	0.332315	0.041112
POR	0.250581	0.03998
POT1-AS1	-0.45045	0.002434
POU2AF1	-0.85618	3.77E-05

POU2F2	0.843127	0.000625
POU6F2	-0.59175	0.003691
POU6F2-AS	-1.04453	0.010845
PP14571	-0.9339	0.011801
PPAN	0.463012	0.001293
PPARA	-0.32418	7.58E-06
PPARD	0.297764	0.021356
PPARG	0.517002	1.77E-09
PPARGC1A	-1.73166	4.12E-17
PPARGC1B	0.202757	0.039024
PPAT	0.352462	9.31E-06
PPCDC	0.277583	0.006271
PPFIBP1	0.188437	0.004886
PPIA	0.140957	0.010374
PPIC	-0.51059	4.92E-09
PPIE	0.113387	0.012157
PPIF	0.312372	4.66E-07
PPIH	0.216876	0.000949
PPIL1	0.17345	0.000424
PPIP5K1	-0.16576	0.000851
PPM1A	-0.14957	0.040318
PPM1B	-0.22404	0.021064
PPM1F	0.379136	0.004838
PPM1G	0.329994	1.02E-08
PPM1H	-0.78191	0.000101
PPM1K	-0.52206	0.003128
PPM1L	-0.41257	0.003892
PPME1	0.124515	0.027379
PPP1CC	0.202566	0.01187
PPP1R11	0.172053	0.010818
PPP1R15A	0.544021	4.34E-08
PPP1R16A	0.449104	0.04331
PPP1R18	0.347655	1.79E-05
PPP1R26	0.252247	0.037152
PPP1R3B	-0.45011	0.00518
PPP1R3D	-0.17451	0.043612
PPP1R8	0.127644	0.007436
PPP2R1A	0.264568	0.007002
PPP2R2B	-1.4095	1.08E-05
PPP2R2D	0.140877	0.005671
PPP2R3A	-0.25271	0.00232
PPP2R5B	-0.25472	0.024431
PPP2R5C	-0.10549	0.020123
PPP2R5D	0.161128	0.04053
PPP4R1	0.200596	9.78E-07
PPP4R4	-0.13587	0.04301
PPP5C	0.34792	1.92E-05

PPP6R1	0.375339	0.00415
PPRC1	0.48109	4.44E-08
PPT1	-0.0855	0.026747
PPT2	0.301978	0.000998
PQBP1	0.347267	0.000179
PQLC3	-0.38381	3.34E-06
PRAP1	-2.62348	0.005219
PRC1	0.230754	0.00011
PRCP	-0.25352	0.000221
PRDM8	0.741065	5.83E-05
PRDM9	1.111444	0.021019
PRDX4	0.146513	0.010778
PREB	0.19618	0.015092
PRELID1	0.374618	0.000565
PRELID3A	0.352231	0.017482
PREX1	0.321826	0.000646
PRICKLE2	-0.44665	3.99E-07
PRIM1	0.358843	8.56E-06
PRIM2	0.175939	0.028156
PRKAB2	-0.23549	0.000979
PRKAR1B	0.276739	0.025137
PRKAR2A	0.104198	0.047074
PRKCA	0.19724	0.002478
PRKCI	-0.16359	0.039496
PRKCSH	0.203029	0.017927
PRKDC	0.212497	3.95E-06
PRKG1	-0.37958	0.017272
PRMT1	0.393699	3.64E-05
PRMT3	0.387594	8.68E-06
PRMT5	0.363435	4.21E-10
PRMT7	0.230604	0.02214
PRMT9	-0.21663	0.016787
PRNP	0.109709	0.029336
PROC	-0.36307	0.023554
PROCR	0.84755	2.02E-17
PRODH	-0.99764	0.001419
PROM2	-0.5727	0.000611
PROS1	-0.49342	2.36E-05
PROSC	0.118061	0.019302
PROSER1	0.155782	0.006718
PROSER2	0.341095	0.008247
PRPF19	0.260414	0.002165
PRPF31	0.259727	0.001799
PRPF38A	0.180688	0.023236
PRPF4	0.284827	1.65E-06
PRPF6	0.153181	0.023135
PRPF8	0.231557	1.60E-05

PRPS1	0.228721	0.00013
PRPS2	0.303245	3.05E-09
PRR11	0.291496	0.001537
PRR5	0.467099	0.01116
PRR5-ARH(	0.651595	0.03299
PRR9	2.687286	0.000233
PRRC2A	0.288358	0.007066
PRRC2B	0.139918	0.039145
PRRC2C	0.116019	0.030993
PRRG2	-0.32836	0.031701
PRRG4	-0.8589	2.10E-05
PRRT2	-1.27085	1.71E-05
PRRT4	0.484776	0.007415
PRRX2	0.652306	0.026501
PRSS16	-0.47906	7.11E-08
PRSS3	2.28468	1.73E-19
PRSS8	-0.51141	0.000133
PRUNE1	-0.14292	0.021246
PRUNE2	-0.65227	0.004347
PSAP	-0.36773	0.000381
PSAT1	0.318225	0.004047
PSD4	0.191528	0.038635
PSEN1	-0.18936	0.002388
PSENEN	0.140841	0.024241
PSG1	-0.7351	0.004834
PSG2	-1.13703	0.021926
PSG3	-0.88041	0.008556
PSG4	-1.01444	2.34E-10
PSG5	-1.02161	0.00011
PSG7	-1.07692	0.001653
PSG8	-1.42745	0.003994
PSG9	-0.8284	0.000494
PSMA3-AS:	-0.22987	0.018241
PSMA7	0.303189	2.55E-05
PSMB2	0.129879	0.002554
PSMB3	0.214397	7.01E-05
PSMB5	0.221573	4.57E-05
PSMB6	0.235985	8.98E-06
PSMB7	0.181051	0.003429
PSMB8-AS:	-0.35018	0.008578
PSMB9	-0.28501	0.006617
PSMC3	0.156275	0.00766
PSMC3IP	0.225168	0.020248
PSMC4	0.199669	0.000101
PSMC5	0.151952	0.005448
PSMD11	0.118773	0.021539
PSMD13	0.202032	1.13E-05

PSMD2	0.311706	1.28E-06
PSMD3	0.158867	0.043151
PSMD4	0.114063	0.034413
PSMD5-AS:	-0.32714	5.04E-06
PSMD6	0.126	0.012204
PSMD8	0.225323	0.007874
PSME1	-0.21111	0.011842
PSME3	0.185674	0.012187
PSMF1	0.177571	0.028841
PSMG1	0.308541	3.09E-07
PSMG2	0.185837	0.011424
PSMG3	0.260218	0.029215
PSMG3-AS:	-0.44912	0.000918
PSMG4	0.361445	0.008894
PSPH	0.147652	0.00442
PSRC1	0.330098	0.03572
PTBP1	0.380242	0.000825
PTCD3	0.206772	9.41E-05
PTDSS1	0.204795	0.002353
PTGER2	0.319125	0.018046
PTGES2	0.406314	0.006666
PTGES3	0.194062	0.006268
PTGES3P1	-1.47975	0.00703
PTGFRN	-0.16103	0.01304
PTGS2	0.512885	0.000302
PTK2B	0.267648	0.014935
PTN	-0.2271	0.044088
PTOV1-AS1	-0.25421	0.005556
PTOV1-AS2	0.490371	0.028202
PTP4A2	0.089851	0.040534
PTPN1	0.226355	0.000194
PTPN14	-0.14657	0.010992
PTPN2	0.178296	0.019735
PTPN21	-0.31627	7.30E-06
PTPN3	-0.1779	0.000257
PTPN4	-0.17057	0.048782
PTPRC	-1.00829	0.041411
PTPRCAP	-2.95225	0.031072
PTPRE	0.494218	3.95E-16
PTPRH	1.49883	2.07E-09
PTPRM	-0.20629	0.004595
PTPRQ	-0.52019	0.048307
PTPRR	0.303955	0.019802
PTTG1	0.208156	0.001362
PUF60	0.361529	0.002495
PUM3	0.322776	1.92E-05
PURB	-0.22447	0.008043

PUS1	0.386921	0.00167
PUS10	-0.44092	1.19E-05
PUS7	0.197095	0.003686
PUSL1	0.422621	0.04387
PVT1	0.361793	7.08E-07
PWP1	0.183517	0.001101
PWWP2A	-0.16562	0.049533
PXMP4	-0.41143	0.002215
PXN	0.347216	0.000297
PYCR1	0.354387	0.001637
PYGB	0.471767	5.00E-07
PYROXD2	-0.51738	1.11E-06
QDPR	0.157085	0.03562
QPRT	-0.35957	0.032463
QRICH2	-0.47373	3.99E-05
QRSL1	0.190021	0.003751
QSOX2	0.332817	1.12E-08
QTRT1	0.423988	0.001511
QTRT2	0.154058	0.021825
R3HCC1	0.235197	0.035051
R3HDM1	0.192074	0.001
R3HDM4	0.359326	0.007782
RAB11FIP1	-0.2456	0.003667
RAB11FIP1	-0.33213	0.047217
RAB11FIP4	-0.20716	0.04893
RAB17	0.332519	0.022635
RAB23	-0.20001	0.001622
RAB25	-0.47872	0.001242
RAB26	-0.70574	7.54E-05
RAB27A	-0.36119	0.000231
RAB30	-0.5587	3.16E-10
RAB31	0.167011	0.008853
RAB32	-0.48648	6.80E-05
RAB34	0.256561	0.040335
RAB38	0.279184	0.004606
RAB3D	-0.28704	0.006235
RAB3IL1	0.376192	0.01558
RAB3IP	-0.3019	0.0013
RAB40A	-0.48955	0.016251
RAB40B	-0.23151	0.042196
RAB43P1	0.542968	0.012193
RAB4A	-0.32782	1.22E-07
RAB5B	-0.28132	1.46E-05
RAB6B	-0.45505	0.044659
RAB8A	0.144322	0.007965
RABEP1	0.16899	0.00558
RABEPK	0.391512	6.48E-08

RABGAP1L	-0.30563	0.002139
RABL2A	-0.27321	0.022011
RABL3	-0.20928	0.005087
RABL6	0.285053	0.041427
RAC2	0.743591	2.77E-10
RAC3	0.386661	0.023691
RACGAP1	0.251399	0.001412
RACK1	0.369221	2.29E-10
RAD1	0.148999	0.00355
RAD18	0.339587	8.15E-05
RAD21	0.161068	0.032056
RAD21-AS1	-0.62162	0.02308
RAD23A	0.189924	0.018384
RAD51AP1	0.21938	0.023547
RAD51-AS1	-0.34855	0.010055
RAD54B	0.297184	0.012324
RAD54L	0.464503	1.50E-07
RAE1	0.154095	0.004318
RAF1	0.103372	0.021737
RAG1	-0.17563	0.047902
RAI14	-0.18513	0.028459
RALA	0.14037	0.030871
RALB	0.187431	0.014987
RALBP1	0.121122	0.006027
RALGAPA2	-0.32589	0.007146
RALGDS	0.273345	0.020159
RALGPS1	-0.50563	0.000312
RALGPS2	0.207076	0.04525
RALY	0.310085	0.007206
RALYL	-0.91225	0.034528
RAN	0.238294	8.44E-05
RANBP1	0.350817	4.92E-05
RANBP3	0.228662	0.045687
RANGAP1	0.450781	1.50E-07
RANGRF	0.327252	0.004074
RAP1GAP2	0.663113	1.03E-13
RAP1GDS1	0.113473	0.048478
RAP2B	0.170759	0.012989
RAP2C-AS1	-0.62729	0.003073
RAPGEF1	0.189883	0.028322
RAPGEF3	0.352942	0.001311
RARA	0.39362	0.028851
RARB	-1.20915	0.000108
RASA3	0.487928	4.91E-07
RASAL2	0.274707	0.004426
RASAL2-AS	0.268222	0.012862
RASD2	-0.85968	1.64E-06

RASGRF1	0.344987	0.024189
RASIP1	0.509565	0.00132
RASL11B	-0.35817	9.38E-05
RASSF5	0.357828	0.038752
RASSF6	-0.70168	9.76E-06
RASSF8	0.274612	0.004336
RBBP7	0.138319	0.031731
RBBP8	0.129119	0.040487
RBFA	0.30725	3.21E-05
RBFADN	-0.62013	0.000867
RBFOX2	-0.10533	0.0475
RBL1	0.176084	0.021465
RBL2	-0.27214	2.41E-07
RBM10	0.270954	0.024182
RBM15	0.20849	0.001148
RBM17	0.122219	0.003477
RBM19	0.308992	2.61E-10
RBM20	-0.60461	0.026143
RBM22	0.172044	0.001037
RBM26-AS:	-0.33647	0.045975
RBM28	0.211453	0.00078
RBM38	0.339219	0.044108
RBMS3	-0.66308	2.92E-10
RBMX	0.18208	0.005715
RBMX2	0.174408	0.002797
RC3H1	-0.21513	0.018178
RCAN1	-0.43441	0.000166
RCAN2	-1.96017	0.001202
RCAN3	-0.18237	0.006908
RCBTB2	-0.30022	0.013548
RCC1	0.458794	1.21E-18
RCC1L	0.246241	0.001628
RCC2	0.163931	0.001193
RCL1	0.446442	1.34E-12
RCN1	-0.15961	0.007981
RCOR3	-0.22074	3.36E-05
RDH13	-0.29663	0.009455
RDH16	-0.52343	0.009544
RECK	-0.43001	1.84E-06
RECQL4	0.549886	0.003782
REEP1	-1.63392	3.67E-08
REEP4	0.326077	0.031355
RELA	0.215009	0.044583
RELN	2.1094	0.006353
RELT	0.472094	0.001416
RERG	-0.94182	0.021945
RET	-0.64232	5.16E-08

REXO4	0.29355	8.99E-08
RFC2	0.261866	0.0003
RFC3	0.226347	0.005776
RFC4	0.166374	0.017822
RFC5	0.270027	1.34E-06
RFLNB	0.273115	0.021121
RFT1	0.313107	3.22E-05
RFTN1	0.16074	0.008506
RFWD3	0.212573	0.00804
RFX2	0.298264	0.012955
RFX5	-0.17767	0.004877
RFX8	1.169235	3.32E-07
RGAG1	0.671365	0.01359
RGCC	1.053317	1.79E-14
RGL2	0.282011	0.005666
RGL4	-0.41875	0.025046
RGS10	0.398108	0.000258
RGS14	0.453558	0.012868
RGS16	1.017049	0.016626
RGS17	0.495962	0.022442
RGS19	0.371232	0.048188
RGS2	-0.26848	0.001465
RGS20	0.319071	7.76E-05
RHBDL2	-0.36967	4.94E-05
RHCG	0.834428	0.046275
RHNO1	0.234572	0.005041
RHOB	0.294161	0.006808
RHOBTB1	-0.41251	8.18E-05
RHOBTB3	-0.456	0.010514
RHOD	0.452657	0.021137
RHOG	0.305942	0.011894
RHOQ	-0.33612	1.35E-05
RHOQP3	3.089849	0.03903
RHOT2	0.342122	0.040277
RHOU	-0.5701	1.43E-12
RHOXF1-AS	-0.83805	2.24E-05
RIC3	-1.42945	0.004548
RIC8B	-0.2006	0.002136
RIMBP2	-0.48412	0.002361
RIMBP3	-0.8899	0.00276
RIMKLB	-0.22042	0.038476
RIMS2	-0.50372	0.000661
RIMS3	-0.68935	9.82E-05
RIN1	0.540118	0.012289
RIN2	0.118977	0.012217
RINT1	0.199615	0.021177
RIOK1	0.301868	2.32E-06

RIPK1	-0.20686	0.001855
RIPK3	-0.50893	0.000526
RIPPLY2	0.630254	0.007677
RIPPLY3	-0.65806	0.031513
RIT1	-0.28308	0.040244
RMDN2	-0.25654	0.021175
RMND1	0.187086	0.016327
RN7SL1	-0.5018	0.016256
RN7SL2	-0.92114	0.005105
RN7SL274F	1.146932	0.04856
RN7SL376F	-2.03414	0.003966
RN7SL45P	-2.30809	0.017021
RN7SL847F	3.696163	0.006865
RNA5SP15!	2.951635	0.034853
RNASE1	-0.35793	0.02295
RNASE10	-0.43803	0.019159
RNASE4	-0.28823	0.029701
RNASE7	0.407939	4.91E-07
RNASEH2A	0.333625	0.004948
RNASEL	-0.51293	3.54E-06
RNASET2	-0.48244	8.83E-05
RND3	-0.42335	0.022572
RNF103	-0.3145	7.34E-06
RNF103-CH	1.057214	0.015172
RNF11	-0.12901	0.036164
RNF115	-0.12823	0.036762
RNF125	0.375029	3.69E-05
RNF13	-0.2239	0.010131
RNF135	-0.27943	0.001378
RNF138	0.24467	0.003619
RNF14	-0.14613	0.011924
RNF141	-0.25061	0.000162
RNF144A	0.830676	3.37E-22
RNF144A-A	0.662261	0.003234
RNF146	-0.40898	0.000324
RNF150	-1.53185	0.001917
RNF152	-0.61757	4.27E-05
RNF167	0.232255	0.028104
RNF169	0.281473	0.000588
RNF170	-0.34444	9.92E-05
RNF182	1.01017	0.0045
RNF19B	0.313497	0.001171
RNF212B	-0.39546	0.006182
RNF213	-0.36953	0.000736
RNF216	0.151057	0.007461
RNF216P1	0.292097	0.000318
RNF217-AS	-0.59336	2.61E-05

RNF26	0.27935	0.038206
RNF40	0.171382	0.049462
RNPC3	-0.26031	0.004553
RNPS1	0.346908	1.43E-06
RNU7-18P	1.350651	0.045225
RNU7-75P	1.935506	0.015166
RNY3	-3.74388	0.003138
ROBO3	0.659087	0.001054
ROCK1P1	-0.83056	0.023396
ROCK2	-0.24116	0.00397
ROS1	-1.93342	1.41E-07
RP11-1000	-0.51454	0.019559
RP11-1020	-0.5987	0.011924
RP11-102N	-1.27692	0.00058
RP11-1033	1.64252	0.015048
RP11-1094	0.579821	0.003131
RP11-1094	-1.71205	0.031759
RP11-10A1	-0.49493	0.006886
RP11-10C2	-0.36674	0.010295
RP11-1102	0.710848	0.00028
RP11-1145	-0.72914	0.034891
RP11-114G	-1.01428	0.019745
RP11-114H	0.861666	5.38E-14
RP11-114H	1.240475	1.07E-20
RP11-1151	1.203639	0.042346
RP11-1152	-3.14478	0.031384
RP11-115D	-0.61766	0.000662
RP11-1167	-2.06396	0.003809
RP11-117L	-2.72103	0.036566
RP11-119D	-1.00365	0.00095
RP11-1246	-0.45249	0.002202
RP11-12G1	0.456347	9.52E-05
RP11-131N	-1.31105	0.000829
RP11-132A	0.681022	8.76E-08
RP11-1334	-0.48906	0.010146
RP11-134L	-0.5422	0.042555
RP11-13K1	-1.48103	0.043671
RP11-141N	-1.20906	0.000123
RP11-142A	-1.07018	0.048694
RP11-145A	-1.09371	1.71E-08
RP11-145N	-1.69571	0.024549
RP11-147L	-0.52615	7.87E-09
RP11-147L	-0.42871	0.046432
RP11-152K	-1.74906	0.047421
RP11-156P	0.573125	0.000124
RP11-158I1	-2.07353	0.000232
RP11-159F	-0.75051	0.001946

RP11-159N	-0.8784	0.003106
RP11-15A1	-0.92958	0.010577
RP11-15F1:	-0.87926	0.031375
RP11-160N	-1.00525	0.005313
RP11-161M	1.470804	0.001716
RP11-164C	-0.88611	0.019519
RP11-164J1	-0.68084	0.001528
RP11-165F:	-2.43003	0.019792
RP11-165J3	-1.12822	0.013751
RP11-169D	2.623955	0.022176
RP11-170M	0.734954	0.043573
RP11-171I2	-1.25387	0.036212
RP11-171I2	-0.88014	0.00288
RP11-173A	1.861279	0.049095
RP11-173P	5.382989	0.014957
RP11-174G	-0.60182	6.46E-07
RP11-174O	-2.31223	0.003204
RP11-175P	-0.91071	0.003062
RP11-181B	-2.26281	0.009762
RP11-181C	3.60299	0.048616
RP11-181K	2.069081	0.021125
RP11-186B	0.395258	0.000315
RP11-187C	-0.72633	0.024995
RP11-18H2	1.034953	0.002138
RP11-196G	0.514908	0.039154
RP11-196G	-0.64081	3.01E-09
RP11-196G	-1.0451	0.00852
RP11-196G	-1.11623	6.98E-05
RP11-197K	-0.3646	0.010604
RP11-1E6.1	2.231883	3.33E-12
RP11-206L:	-0.41686	0.00202
RP11-20G1	-2.89815	0.039385
RP11-210K	-2.8872	0.049616
RP11-211G	-1.9406	0.007386
RP11-212P	-0.32692	0.002104
RP11-215P	1.200424	0.045599
RP11-217C	1.258483	0.025152
RP11-218M	-0.43336	0.001546
RP11-219A	-0.77927	0.005412
RP11-219A	1.299475	0.005965
RP11-221N	0.772764	0.000599
RP11-228B	-0.69041	0.008034
RP11-229P	1.394531	0.000257
RP11-22B2	0.601796	1.73E-05
RP11-22C1	-0.72324	0.018069
RP11-22D3	-3.02485	0.026334
RP11-22N1	0.93438	0.045775

RP11-234A	1.013178	0.00529
RP11-23J18	-0.96476	0.008243
RP11-244F	-1.69189	0.003907
RP11-244H	-0.60818	0.000629
RP11-244O	-0.23269	0.010712
RP11-248G	5.364594	3.61E-05
RP1-124C6	-1.49275	2.72E-05
RP11-250B	-0.9394	0.018606
RP11-250B	-0.4985	0.032008
RP11-253M	-0.65037	0.002891
RP11-254I2	-2.99164	0.016017
RP11-256I9	0.278202	0.042962
RP11-257K	-3.78068	0.005028
RP11-259N	0.272389	0.028031
RP11-259O	-0.58122	0.006317
RP11-259O	-0.27589	0.014708
RP1-125I3.	1.467841	0.001058
RP11-265B	-3.07096	0.028387
RP11-265N	-5.95795	1.64E-06
RP11-266K	-0.37683	0.024808
RP11-266L9	-3.51601	0.003766
RP11-267M	0.579251	0.004389
RP11-272P	1.50148	0.012181
RP11-274B	-0.43059	0.004099
RP11-274H	-0.74472	0.009381
RP11-277P	-0.64695	2.31E-05
RP11-280G	1.944391	0.035985
RP11-281A	-3.33449	0.037199
RP11-283I3	-0.6351	5.81E-07
RP11-285F	-0.5812	0.04008
RP11-286N	1.119396	0.000634
RP11-286N	-0.27772	0.010122
RP11-288L9	-1.12937	0.034706
RP11-290L1	1.53418	0.049252
RP11-295D	0.592079	0.000931
RP11-295K	-0.6214	0.00498
RP11-297P	0.929934	1.46E-21
RP11-298I3	-0.49489	1.43E-05
RP11-299G	-0.45357	0.001011
RP11-29B2	-3.90623	0.002355
RP11-2L8.1	-1.90433	0.021545
RP11-305K	-0.82445	0.032309
RP11-306O	-0.49564	0.001914
RP11-307E	-0.22908	0.046152
RP11-309M	1.853594	0.029735
RP11-30P6	0.754302	0.000449
RP11-314N	-0.7952	0.044755

RP11-319G	-0.54464	0.034944
RP11-321N	-1.45472	0.011282
RP11-323P	-0.66914	0.019675
RP11-324E	-0.86879	0.03902
RP11-324I2	-0.95807	0.03337
RP11-325L1	-1.11157	0.044258
RP11-326C	0.76866	0.044417
RP11-326G	-0.59067	0.009134
RP11-326I1	-0.42799	0.026422
RP11-326I1	-1.26759	0.015524
RP11-327F1	-0.64705	0.00993
RP11-32B5	-1.20647	0.003725
RP11-32B5	-1.06841	0.033764
RP11-334C	-0.43539	0.025089
RP11-335E	1.29833	0.009977
RP11-337C	-0.72508	0.007664
RP11-337C	-0.33738	0.01561
RP11-337N	-0.81585	0.028273
RP11-338K	0.840671	0.047082
RP11-33B1	-0.22417	0.02806
RP11-33B1	1.221873	0.037712
RP11-342K	3.414943	0.035781
RP11-342M	0.455668	0.02345
RP11-343C	0.367883	7.93E-05
RP11-345J1	-2.57377	0.043063
RP11-345J4	0.226692	0.000847
RP11-345P	-1.56345	0.022385
RP11-346D	-0.41968	0.00018
RP11-347C	-2.05105	0.042835
RP11-350N	2.505188	0.043447
RP11-350N	0.933476	0.007609
RP11-350N	0.782311	0.0233
RP11-353K	-1.2704	0.045449
RP11-356J5	-0.42233	2.10E-05
RP11-359E	-0.64926	0.006756
RP11-35J10	-0.96598	0.042513
RP11-360L9	2.499473	1.49E-05
RP11-361L1	-0.75014	0.011082
RP11-363E	-0.89532	0.034987
RP11-364P	-1.17856	0.000274
RP11-366L1	0.969765	0.012938
RP11-367G	-0.64854	6.83E-05
RP11-368N	-0.68968	0.04876
RP11-373L1	-0.58196	1.88E-05
RP11-378A	-0.36742	0.04014
RP11-379B	-0.37421	0.000411
RP11-379F4	-1.64805	0.016679

RP11-379H	-0.39763	5.03E-05
RP11-37L2	-1.7091	0.000419
RP11-382B	-0.32465	6.95E-05
RP11-383J2	-0.89493	0.009021
RP11-384C	3.300657	0.040335
RP11-385D	-1.13345	0.000162
RP11-387H	-3.06435	0.033097
RP11-38L1	-0.5051	5.33E-06
RP11-391H	-2.09011	0.006402
RP11-391L1	-2.14799	0.029477
RP11-395I1	-0.60852	0.040204
RP11-395N	-0.55578	0.002258
RP11-397A	-0.39498	0.012795
RP11-398C	0.358873	7.56E-05
RP11-3B12	-4.05863	0.000632
RP11-400K	-1.15909	0.003263
RP11-400K	-0.65298	2.18E-14
RP11-403A	0.751488	0.040416
RP11-405M	1.383694	0.040575
RP11-406D	-0.66825	0.045088
RP11-408P	0.380356	0.001027
RP1-140K8	-0.49928	0.000455
RP11-412H	2.005412	0.010794
RP11-413N	-0.57053	0.039061
RP11-414H	-3.02035	0.019066
RP11-415J8	0.316145	0.009668
RP11-416I2	1.115488	2.96E-10
RP11-416N	0.514521	0.016592
RP11-420A	-0.87529	0.007086
RP11-420L9	-0.55366	3.31E-05
RP11-420N	-2.77275	0.040971
RP11-420N	3.222507	0.049755
RP11-421F	-0.36772	0.00185
RP11-424C	1.090978	0.001931
RP11-439E	-0.7126	0.015363
RP11-439E	-1.26865	0.001154
RP11-440D	0.265141	0.036784
RP11-445F	0.848191	1.71E-05
RP11-445H	-2.78178	0.009424
RP11-445N	2.967724	0.02403
RP11-446N	-0.72436	0.018799
RP11-448A	-0.49877	0.004876
RP11-449H	0.389827	0.010975
RP11-44B1	-1.65104	0.041486
RP11-44F1	-0.67464	0.021459
RP11-44F2	-0.53	0.000871
RP11-44N2	-0.428	0.013998

RP11-456N	1.397711	0.026006
RP11-456P	0.676113	0.028269
RP11-457M	-0.80908	0.036765
RP11-45M2	0.373045	0.012982
RP11-461O	0.891544	0.040021
RP11-463O	-7.15363	0.0168
RP11-467J1	-0.70817	0.006721
RP11-468E	-0.27717	0.024431
RP11-46A1	-2.19208	0.025967
RP11-46C2	-0.31827	0.016688
RP11-473M	-0.90718	0.039595
RP11-473O	-2.21467	0.036566
RP11-474L1	0.996536	0.009802
RP11-478P	-0.90193	0.048942
RP11-481A	-0.47762	0.047411
RP11-481C	0.453132	0.0278
RP11-481F	-1.77608	1.65E-05
RP11-482H	-1.83989	0.02658
RP11-483F	-0.89492	0.043499
RP11-483I1	-0.67335	0.028779
RP11-486L1	-0.61397	0.00256
RP11-486O	-1.00001	0.001754
RP11-488P	1.309366	7.25E-21
RP11-489E	-1.47768	0.028661
RP11-494M	-0.4606	0.004816
RP11-495P	0.686571	0.047324
RP11-496H	-0.35502	0.009145
RP11-498B	1.986259	0.037019
RP11-498E	0.690386	0.01111
RP11-499E	-0.82439	0.00778
RP11-499F	-1.67376	0.023226
RP11-49I11	-1.30867	0.00862
RP11-504P	-0.28159	0.030307
RP11-504P	-0.68514	0.018859
RP11-517C	1.444868	0.030494
RP11-517I3	-1.03443	0.011471
RP11-517I3	-0.97939	2.05E-05
RP1-151F1	-0.37545	0.019429
RP11-520H	-1.53507	0.036087
RP11-521B	0.463805	0.000934
RP11-521B	0.429183	1.75E-06
RP11-521C	0.740768	0.007195
RP11-523L1	-1.88664	0.042003
RP11-525A	1.5461	0.010527
RP11-529K	0.689287	0.003993
RP11-530N	-0.53499	0.044922
RP11-532F	-0.46141	0.018554

RP11-538D	-0.48032	0.029953
RP11-539G	-0.72967	0.0387
RP11-53B2	-1.71055	4.72E-05
RP11-53B2	-2.05014	0.003361
RP11-541N	-0.51654	0.008116
RP11-544M	-0.39891	0.026698
RP11-545A	-1.60185	0.018632
RP11-546J1	-0.81893	0.005429
RP11-548K	-3.14728	0.034712
RP11-54A9	0.73975	0.001379
RP11-54H7	-0.134	0.020742
RP11-54O7	-2.91607	0.042937
RP11-553A	-0.38369	0.047554
RP11-553L	-0.50106	0.010267
RP11-557H	-0.814	0.011861
RP11-561C	-1.06179	0.028331
RP11-566K	1.197676	0.025513
RP11-567J2	0.705874	0.038242
RP11-56B1	-0.60224	0.037647
RP11-574H	1.126993	0.010567
RP11-575F	-0.40068	0.037321
RP11-578F	-3.66589	0.020735
RP11-57A1	3.404232	0.030187
RP11-57H1	-0.57764	0.035331
RP11-57H1	-0.45732	5.91E-05
RP11-582J1	0.360619	0.017297
RP11-583F	-2.21245	0.003262
RP11-585P	0.416364	0.000386
RP11-585P	-0.76102	0.038641
RP11-587D	1.147485	0.034401
RP11-587P	3.045422	0.027818
RP11-588K	-1.93528	0.022769
RP11-58O9	0.435099	0.001033
RP11-596C	-0.9689	0.0002
RP11-597D	-0.49282	0.003506
RP11-598P	-7.14465	0.018333
RP1-159A1	-2.03625	0.037788
RP11-59C5	0.429864	0.012009
RP11-59D5	1.798825	2.71E-12
RP11-59H7	-0.61532	0.046943
RP11-59H7	-1.00524	0.047932
RP11-5C23	-0.51614	0.019913
RP11-603J2	-2.43762	0.03821
RP11-609N	0.69415	0.000525
RP11-611L	-2.90165	0.027175
RP11-616M	3.092187	0.010171
RP11-617F	-0.59541	0.000353

RP11-618P	1.496999	0.044814
RP11-61N2	0.535238	0.007997
RP11-624A	-0.21385	0.027377
RP11-624L	-0.22504	0.037692
RP11-625H	-0.52918	0.047753
RP11-629E	-1.13626	0.014528
RP11-631N	-0.46229	0.000787
RP11-638I8	2.826176	0.037057
RP11-63P1	2.00371	0.005619
RP11-641D	0.212048	0.001221
RP11-655M	1.816527	0.018105
RP11-65G9	0.902932	1.35E-05
RP11-661A	0.445366	0.004142
RP11-667M	-3.13165	0.023652
RP11-66B2	0.249032	0.037979
RP11-66B2	1.139788	3.66E-06
RP11-677M	-0.49679	0.018345
RP11-677M	1.411436	0.013319
RP11-680A	0.536791	0.016044
RP11-686D	-0.64184	0.018815
RP11-68I3.	3.118022	0.018627
RP11-696N	-0.50786	0.002954
RP11-69C1	-1.12605	0.005751
RP11-69I8.	0.616727	1.56E-09
RP11-701H	-2.4209	0.039922
RP11-701H	-0.93743	0.030954
RP11-701H	-1.44776	0.012974
RP11-703I1	-1.96465	0.001032
RP11-706P	-1.19595	0.018313
RP11-70D2	-1.28049	0.046635
RP11-70L8.	0.797505	0.002831
RP11-719K	-3.26876	0.046794
RP11-729I1	1.739658	0.037202
RP11-731C	-0.66116	0.005301
RP11-734K	-0.48746	0.000531
RP11-736N	-0.74699	0.024529
RP11-739P	-0.81977	0.008725
RP11-739P	-0.6635	0.016561
RP11-73E1	-0.48301	0.012699
RP11-73M1	-0.44224	0.041274
RP11-745L1	-0.85172	0.018297
RP11-74M1	-0.60829	0.029148
RP11-752G	-3.72459	0.007895
RP11-752L1	-3.13365	0.029329
RP11-753A	2.061293	0.04591
RP11-757F	-0.29269	0.044279
RP11-757G	-0.97352	6.54E-05

RP11-75I2.	-3.08039	0.010662
RP11-761I4	-1.73723	0.005189
RP11-762I7	-1.46161	0.038578
RP11-766F	-0.9437	0.001481
RP11-774O	-1.015	8.99E-07
RP11-775C	-1.41277	0.028908
RP11-777F	-2.08898	0.044382
RP11-781P	-2.91876	0.029059
RP11-783K	0.380816	0.007926
RP11-7K24	-0.45472	0.047571
RP11-800A	-1.04274	0.005307
RP11-806O	-1.6516	0.000364
RP11-80I15	-0.99624	0.032784
RP11-810P	-1.26264	0.043062
RP11-81A2	-0.39676	0.014077
RP11-822E	0.650088	0.000439
RP11-823E	1.443114	2.21E-05
RP11-834C	0.718851	0.004008
RP11-834C	-1.52636	0.026877
RP11-834C	0.40952	0.029201
RP11-83A2	-0.26235	0.02978
RP11-83M1	-0.70094	0.006787
RP11-83M1	-0.74465	0.001306
RP11-844P	2.901615	0.008433
RP11-844P	0.566533	0.007371
RP11-845M	-1.01629	0.00098
RP11-848G	-1.50532	0.003536
RP11-848G	-0.43854	0.026541
RP11-848P	-0.43875	0.003752
RP11-849F	-0.52268	0.023912
RP11-849H	-0.35491	0.021496
RP11-867G	-1.47414	0.006489
RP11-879F	2.609088	0.004674
RP11-884K	-0.5376	0.003713
RP11-885B	-1.93974	0.032738
RP11-88I18	-0.36832	0.046564
RP11-890B	-0.43687	0.00032
RP11-903H	-0.71741	0.003104
RP11-90J19	-0.47767	2.47E-07
RP11-90L1.	-0.47822	2.14E-05
RP11-932O	0.857784	0.025858
RP11-932O	-1.04636	0.016443
RP11-967K	-0.47339	0.000572
RP11-96B2	1.840783	0.005209
RP11-96B5	-2.91182	0.033582
RP11-96D1	-0.55571	0.001378
RP1-197B1	-0.49682	0.011799

RP11-989E	0.470498	0.020712
RP1-198K1	0.761372	0.021962
RP1-199J3.	-0.77532	0.017568
RP1-221C1	-0.66996	2.55E-05
RP1-261G2	0.743634	4.79E-05
RP1-27O5.:	0.56706	0.006008
RP13-104F.	-0.6049	0.005247
RP13-204A	-1.33099	0.000279
RP13-210D	-1.80056	0.006313
RP13-467H	-1.40857	0.03174
RP1-34B20	-1.00114	0.004055
RP1-34M2:	2.528473	0.001429
RP13-766D	-1.15304	0.005707
RP13-942N	0.74596	0.000974
RP1-80N2.:	-0.69298	0.000421
RP1-80N2.4	-0.70461	0.016961
RP1-95L4.4	-0.50702	0.013453
RP1L1	0.440816	0.012162
RP3-331H2	0.477526	8.68E-05
RP3-333H2	2.985463	0.022407
RP3-368A4	-0.51125	0.019406
RP3-395M2	0.936135	0.033792
RP3-395M2	-0.69948	0.01636
RP3-400B1	-0.95502	0.01302
RP3-426I6.	3.666794	0.006098
RP3-428L1(	-0.33572	0.018746
RP3-429G5	-1.78572	0.013366
RP3-439F8	-0.55133	0.000707
RP3-465N2	3.502516	0.006975
RP3-467D1	0.286723	0.00435
RP3-473L9.	-2.3544	0.02895
RP3-508I15	1.676066	0.041437
RP4-530I15	0.851305	0.000988
RP4-536B2	-0.84096	0.018594
RP4-545C2	-0.69174	0.00128
RP4-555D2	0.28028	0.019424
RP4-568C1	-0.30674	0.000244
RP4-584D1	-3.29829	0.033973
RP4-614C1	0.237675	0.013141
RP4-635E1	-0.72005	5.32E-05
RP4-663N1	2.527137	0.042538
RP4-665N4	-2.98064	0.022672
RP4-668J24	0.736418	0.018072
RP4-673M1	-1.1638	0.00439
RP4-714D9	-0.27549	0.021109
RP4-758J18	-0.22927	0.034493
RP4-781K5	0.288953	0.012084

RP4-798P1	-0.45992	0.000181
RP4-800G7	-0.43188	0.043025
RP5-1028K	0.71487	8.43E-06
RP5-1037N	1.956362	0.020174
RP5-1039K	-0.40745	0.001257
RP5-1039K	-1.80007	0.002225
RP5-1039K	-0.30215	0.002222
RP5-1042K	0.839454	0.008704
RP5-1061H	-0.57379	0.031433
RP5-1063M	0.482648	0.013635
RP5-1065J2	-0.39765	0.034707
RP5-1074L1	-0.41162	0.000885
RP5-1086K	-0.5677	0.008436
RP5-1148A	-0.45103	0.031703
RP5-821D1	-0.32571	0.032528
RP5-827C2	-0.8455	0.02933
RP5-855D2	-0.67065	0.040445
RP5-855D2	-1.13006	0.024676
RP5-857K2	-0.63988	0.035497
RP5-864K1	0.684461	0.044373
RP5-874C2	-1.42922	0.041069
RP5-875H1	0.57384	0.002009
RP5-882C2	-0.59514	0.010386
RP5-965G2	0.551075	0.000107
RP5-965G2	0.511108	0.044544
RP5-967N2	0.739891	0.010339
RP6-114E2	-0.5665	0.049945
RP6-218J18	-3.29249	0.043903
RP9P	0.221441	0.012826
RPA1	0.148548	0.006832
RPA2	0.1325	0.019863
RPA3	0.174197	0.010233
RPAP1	0.204413	0.012616
RPAP2	-0.16811	0.0309
RPAP3	0.163481	0.048062
RPE65	0.45309	0.000221
RPEP6	-1.03595	0.001212
RPF1	0.177666	0.011981
RPF2	0.319143	0.000111
RPGR	0.186266	0.020385
RPIA	0.314367	3.27E-06
RPL10	0.179508	0.02134
RPL10A	0.252566	4.47E-06
RPL10P13	1.574233	6.49E-07
RPL11	0.221437	7.05E-08
RPL12	0.295855	3.82E-08
RPL13A	0.307605	0.000316

RPL13AP5	0.70628	0.02296
RPL13AP7	0.680226	0.040632
RPL13P12	1.564355	0.007874
RPL14	0.2612	3.87E-06
RPL15	0.158794	0.000196
RPL17	0.215601	0.000439
RPL18	0.369851	6.23E-06
RPL18A	0.327987	0.007941
RPL19	0.224535	0.012433
RPL21P16	0.41407	0.013218
RPL22L1	0.346164	0.000559
RPL23	0.182037	0.007052
RPL23A	0.355616	3.22E-05
RPL24	0.193401	0.00767
RPL26	0.198848	0.013274
RPL27	0.19293	0.000369
RPL27A	0.290279	0.000581
RPL28	0.235358	0.007541
RPL29	0.281066	1.97E-05
RPL3	0.29533	5.63E-11
RPL30	0.119699	0.034427
RPL31	0.170825	0.005601
RPL32	0.27019	4.37E-09
RPL34	0.226854	9.32E-05
RPL35	0.337322	1.48E-05
RPL35A	0.238647	0.000226
RPL36	0.404819	4.98E-05
RPL36A	0.19333	0.009208
RPL37	0.153922	0.013575
RPL37A	0.149354	0.034291
RPL38	0.221829	6.23E-05
RPL39L	0.226164	0.032935
RPL4	0.279991	1.41E-12
RPL41	0.220481	9.68E-06
RPL5	0.226832	0.000881
RPL6	0.249678	9.72E-09
RPL7	0.179846	0.021087
RPL7A	0.300175	1.18E-07
RPL7L1	0.174262	0.002595
RPL8	0.43216	0.001267
RPL9P7	2.442514	0.012456
RPLP0	0.262324	3.41E-06
RPLP0P2	0.522853	0.005638
RPLP0P6	0.96891	0.0043
RPLP2	0.419256	0.001682
RPN1	0.10153	0.042841
RPP21	0.327638	0.005883

RPP25L	0.234718	0.005309
RPP30	0.234655	0.00792
RPP40	0.324181	6.16E-05
RPRM	-1.90103	0.03211
RPS10	0.281617	2.91E-07
RPS11	0.155722	0.017982
RPS12	0.255461	1.68E-07
RPS13	0.258293	4.68E-07
RPS14	0.332518	4.33E-06
RPS15	0.36845	0.031579
RPS15A	0.173938	0.001881
RPS16	0.346631	1.69E-05
RPS17	0.221809	0.000177
RPS18	0.233188	2.96E-05
RPS19	0.276778	0.000437
RPS19BP1	0.320932	0.023939
RPS2	0.410287	0.029252
RPS20	0.176218	0.000333
RPS21	0.281565	4.12E-05
RPS24	0.219734	0.000257
RPS27A	0.151344	0.030974
RPS28	0.305804	0.00305
RPS28P7	0.29446	0.00924
RPS2P46	0.493431	0.000875
RPS2P5	0.242214	0.021279
RPS3	0.268313	0.000149
RPS3A	0.164241	0.041227
RPS4X	0.23733	9.87E-07
RPS4XP16	-0.66419	0.026273
RPS5	0.351743	8.04E-05
RPS6	0.221883	0.001241
RPS6KA1	0.272979	0.002456
RPS6KA2	0.277293	0.010465
RPS6KA2-I1	0.870288	0.008388
RPS6KA4	0.345749	0.02583
RPS7	0.17614	0.002985
RPS7P11	2.139618	0.03196
RPS8	0.298625	1.86E-09
RPS9	0.301849	6.88E-07
RPSA	0.251943	8.86E-05
RPSAP52	1.367317	2.65E-32
RPUSD3	0.311559	0.021036
RPUSD4	0.177826	0.014023
RRAGB	-0.20562	0.004543
RRAGC	-0.167	0.048862
RRM1	0.127283	0.043969
RRM2	0.351478	0.001015

RRP1	0.555862	1.10E-07
RRP12	0.368205	1.20E-06
RRP15	0.178405	0.035214
RRP1B	0.327211	0.0005
RRP7A	0.32801	0.001484
RRP7BP	0.234652	0.035451
RRP8	0.200335	0.000564
RRP9	0.470428	8.58E-05
RRS1	0.306409	0.006078
RSAD1	0.373152	0.000206
RSC1A1	0.286209	0.006627
RSL1D1	0.384557	3.12E-08
RSL24D1	0.204005	0.018707
RSU1	-0.13943	0.010507
RTCB	0.120924	0.04181
RTEL1	0.436976	0.000133
RTKN	0.303573	0.028272
RTKN2	-0.44439	0.000403
RTN1	-0.96065	0.048589
RTP3	0.223916	0.042295
RTP4	-0.82629	0.017469
RTTN	0.330677	0.000202
RUNX3	0.380775	0.008316
RUSC2	0.566413	5.58E-07
RUVBL1	0.39521	1.49E-14
RUVBL2	0.451618	0.000418
RWDD2A	-0.46791	3.67E-05
RWDD3	-0.17894	0.038855
RXRG	-0.87867	0.035797
S100A10	0.177439	0.005712
S100A11	0.225016	0.008356
S100A13	0.141882	0.009446
S100A16	0.310859	0.000262
S100A2	0.284852	5.13E-05
S100A3	0.297929	0.038849
S100A5	1.490497	0.017127
S1PR1	0.304375	0.008789
S1PR2	-0.34206	0.001538
S1PR3	0.25762	0.000724
S1PR5	-0.56835	0.000711
SAA1	-0.75631	1.36E-11
SAA2	-1.00233	1.71E-10
SAA4	-1.69352	0.004001
SAAL1	0.317251	6.79E-06
SACS	0.299329	0.004077
SAE1	0.229083	3.25E-05
SAFB	0.266616	0.000738

SAFB2	0.238869	0.005478
SALL1	0.419734	0.015077
SAMD12	-1.11469	3.81E-07
SAMD4A	-0.53262	0.000138
SAMD4B	0.306952	0.002976
SAMD9L	-0.80073	0.014194
SAMHD1	-0.48757	0.020819
SAP130	0.182115	0.017902
SAP18	0.113352	0.02542
SAP30	0.424496	0.001983
SAP30BP	0.153861	0.001071
SAP30L	-0.20036	0.016926
SAP30L-AS:	-0.38781	0.039611
SAPCD1-AS	-2.07348	0.03517
SAR1B	-0.13766	0.025862
SARDH	0.439238	0.026553
SARM1	-0.50534	0.005928
SARS	0.172859	0.012311
SARS2	0.344423	0.011062
SART1	0.278411	0.006642
SART3	0.223785	3.52E-05
SASH1	-0.336	0.001267
SATB1	-0.61961	0.005096
SATB2	-0.24075	0.015024
SAV1	0.158239	0.021093
SBF1	0.316477	0.027178
SBF2-AS1	-0.29003	0.001869
SCAF4	0.217222	0.000925
SCAF8	0.119069	0.020177
SCAP	0.238396	0.032716
SCARB1	0.344993	0.000659
SCARB2	-0.14594	0.021126
SCARF1	-0.86224	0.00567
SCARNA13	0.509846	0.040068
SCART1	-0.4427	0.035297
SCCPDH	-0.20017	0.000434
SCD	0.216225	0.029589
SCD5	-0.57493	1.51E-06
SCFD2	0.137987	0.029943
SCGB3A2	0.614119	1.53E-12
SCLT1	0.203827	0.005851
SCLY	0.337671	0.001276
SCN1B	-0.51096	0.006396
SCN2A	-0.65613	1.04E-06
SCN3B	-0.50845	0.0142
SCN4B	-0.43065	9.06E-05
SCN8A	-0.3859	0.010004

SCN9A	0.587866	0.000904
SCO1	0.156071	0.005523
SCP2	-0.24699	0.002587
SCPEP1	-0.65671	5.55E-08
SCRN3	-0.39932	0.000131
SCUBE3	-0.42856	0.006317
SCYL1	0.221397	0.041491
SCYL3	-0.22028	0.03878
SDAD1	0.12113	0.017334
SDCCAG3	0.420361	8.37E-09
SDCCAG8	0.149142	0.012865
SDHA	0.188545	0.001853
SDHAF3	0.241558	0.005096
SDHB	0.288209	0.000292
SDHC	-0.11082	0.041575
SDK1	-0.58521	2.06E-05
SDK2	-0.40723	0.001588
SDPR	-0.54278	0.003391
SDR39U1	0.325277	0.04287
SDR42E1	-0.28228	0.009288
SDSL	-0.35033	0.007315
SEC13	0.147658	0.048108
SEC14L1	0.114541	0.021925
SEC14L2	0.387018	2.74E-07
SEC16B	-0.46106	0.00014
SEC1P	-0.85974	0.015866
SEC22A	-0.19763	0.033932
SEC22B	-0.14216	0.044003
SEC23A	-0.21156	0.011282
SECISBP2L	-0.21663	0.030149
SEH1L	0.34521	1.75E-09
SEL1L3	-0.25607	0.021043
SELENOH	0.267085	0.021559
SELENOP	-1.5098	0.000241
SELL	-1.21169	0.000105
SEMA3C	-0.19963	0.026552
SEMA3F	-0.30184	0.020532
SEMA4B	0.311099	0.013092
SEMA4D	0.234175	0.010534
SEMA4G	-0.28717	0.043266
SEMA6D	-0.55692	0.007319
SEMA7A	0.710453	2.14E-09
SENCR	0.660936	0.014682
SENP1	0.211536	0.000381
SENP3	0.341777	0.002639
SENP7	-0.30618	0.011064
SENP8	-0.27244	0.018105

SEPHS1	0.141315	0.035582
SEPSECS-AS	-0.21485	0.048716
11-Sep	0.199956	0.002489
SEPT14P19	-1.47555	0.000878
4-Sep	-0.48596	0.030941
6-Sep	-0.18499	0.005095
8-Sep	-0.16683	0.001972
9-Sep	0.324057	0.009048
SERAC1	-0.24239	0.032159
SERBP1	0.300248	1.56E-13
SERINC3	-0.14975	0.031648
SERPINB13	-0.83196	0.033
SERPINB2	1.804278	8.00E-44
SERPINB5	0.333285	5.80E-05
SERPINB8	0.269631	0.005625
SERPINB9P	-0.70507	3.10E-06
SERPIND1	1.154538	0.028912
SERPINE1	0.314605	8.57E-05
SERPINE2	0.905308	5.06E-06
SERPINF1	-0.20512	0.034484
SERPINF2	-0.49864	0.004829
SERTAD1	0.304939	0.025647
SERTAD2	0.182452	0.004419
SERTAD3	-0.29193	0.000928
SERTAD4	-0.35526	4.82E-05
SESN1	-0.17878	0.0439
SESN2	0.346616	0.012273
SESN3	-0.51354	0.001049
SET	0.240644	0.000455
SETBP1	0.395332	1.96E-06
SETD1A	0.364311	0.009677
SETD3	0.117935	0.019068
SF1	0.246684	0.00952
SF3A1	0.282561	5.07E-07
SF3A2	0.41266	0.041281
SF3A3	0.23024	1.19E-05
SF3B2	0.230871	1.09E-07
SF3B3	0.295716	3.86E-07
SF3B5	0.298314	0.008286
SFMBT2	0.264384	0.006271
SFN	0.416662	0.001438
SFR1	0.182424	0.034047
SFRP1	-0.23253	5.17E-05
SFSWAP	0.263708	0.000251
SFT2D2	-0.2518	0.003984
SFTA1P	1.067599	2.22E-15
SFXN1	0.175209	0.006294

SFXN4	0.365591	7.12E-07
SGCB	-0.2554	7.56E-05
SGF29	0.249151	0.011526
SGK1	-0.16952	0.025159
SGMS1-AS1	-0.81448	3.49E-05
SGMS2	0.190603	0.014985
SGPP1	-0.1904	0.029804
SGTA	0.462872	0.001479
SH2B2	0.717961	0.017055
SH2B3	0.444556	1.68E-12
SH2D2A	1.136017	5.92E-11
SH2D3A	0.516085	0.000229
SH2D3C	-0.64873	0.041908
SH2D5	1.116941	8.64E-16
SH3BGRL	-0.54396	7.61E-11
SH3BGRL3	0.483016	0.00031
SH3BP5-AS	-0.49695	0.001923
SH3D19	-0.21814	0.000248
SH3GL1	0.419286	0.003162
SH3GLB1	-0.1535	0.03421
SH3KBP1	0.35927	1.28E-06
SH3RF1	0.213436	0.003683
SH3RF2	-0.29697	0.000513
SH3RF3	0.440458	4.84E-05
SH3TC1	0.592784	0.000226
SH3TC2	0.37302	8.53E-06
SH3YL1	-0.36607	0.004665
SHANK2	-0.30087	0.002972
SHC1	0.2745	0.000459
SHC2	-0.63456	0.00946
SHC3	-0.30083	0.002692
SHCBP1	0.24518	0.001836
SHISA4	-0.73444	5.59E-06
SHISA9	-0.36255	0.004155
SHMT2	0.404132	8.95E-12
SHQ1P1	-1.70253	0.001799
SHROOM3	0.394473	1.24E-12
SHROOM4	-0.40201	2.34E-07
SHTN1	-0.34837	0.000283
SIAE	-0.61316	5.94E-10
SIDT2	-0.55822	3.60E-07
SIGMAR1	0.457507	0.000898
SIKE1	-0.20531	0.008253
SIMC1	0.409328	0.000606
SIN3A	0.127214	0.016598
SIPA1L3	0.343493	0.014006
SIRPA	-0.2812	0.002155

SIRT5	-0.13112	0.043401
SIX1	0.148802	0.032794
SIX2	0.383798	0.031294
SKA1	0.256472	0.003575
SKA3	0.456931	1.22E-05
SKI	0.210366	0.037742
SKIV2L	0.275795	0.000612
SLAIN2	-0.18997	0.041137
SLBP	0.165142	0.012704
SLC12A7	0.300359	0.012476
SLC12A8	-0.4352	0.000105
SLC13A5	0.571858	0.000253
SLC15A2	-0.30069	0.000131
SLC16A1	0.135202	0.046862
SLC16A13	-0.27769	0.032895
SLC16A14	-0.46203	0.000632
SLC16A4	-0.46287	0.000881
SLC17A5	-0.42003	1.98E-09
SLC19A1	0.494464	0.000703
SLC1A3	-0.18402	0.005432
SLC1A5	0.252055	0.01194
SLC20A1	0.804186	3.07E-28
SLC22A2	0.438385	0.048337
SLC22A23	-0.41023	0.000307
SLC22A5	-0.17362	0.017451
SLC24A3	-0.61233	6.00E-05
SLC25A15	0.293579	9.60E-05
SLC25A19	0.473375	1.23E-09
SLC25A1P5	1.706846	0.017599
SLC25A22	0.534071	0.002487
SLC25A23	-0.37183	0.000837
SLC25A25	0.437608	6.06E-10
SLC25A27	-0.39905	0.001352
SLC25A3	0.112909	0.041129
SLC25A32	0.243046	0.001103
SLC25A33	0.144271	0.035206
SLC25A37	0.334657	2.01E-06
SLC25A39	0.309586	0.007975
SLC25A4	-0.29216	0.001603
SLC25A42	-0.28814	0.010774
SLC25A46	-0.14941	0.024665
SLC25A5	0.276842	1.97E-08
SLC25A52	1.376293	0.017357
SLC25A5-A	0.453586	0.04363
SLC25A6	0.350282	0.005957
SLC26A2	-0.29587	0.010115
SLC26A6	0.194122	0.043917

SLC27A3	-0.38762	0.005377
SLC27A4	0.347342	0.002945
SLC27A6	-2.90641	0.036244
SLC28A3	-0.56094	0.003884
SLC29A1	0.715274	8.34E-11
SLC2A10	-0.72496	0.001767
SLC2A11	-0.4683	0.000106
SLC2A12	-1.74565	6.13E-16
SLC2A1-AS1	-0.67682	0.029127
SLC2A3	1.469032	6.77E-25
SLC2A5	-0.25486	0.020915
SLC2A9	-0.28456	0.000327
SLC30A10	-0.85746	0.005968
SLC30A4	-0.68462	1.62E-09
SLC30A6	-0.19674	0.024099
SLC33A1	-0.24202	0.002467
SLC34A1	1.685756	0.033476
SLC35A5	-0.17413	0.001418
SLC35B3	-0.21907	0.0185
SLC35B4	-0.19618	0.039856
SLC35D1	-0.30059	0.000205
SLC35E2B	-0.17496	0.004587
SLC35E3	-0.13525	0.047544
SLC35F2	0.236026	4.39E-05
SLC35F5	-0.16077	0.040416
SLC37A2	0.349751	0.000846
SLC37A4	0.200209	0.010216
SLC38A4	-0.47481	1.42E-10
SLC38A5	0.329048	0.002685
SLC38A6	-0.21637	0.002193
SLC38A7	-0.17264	0.014019
SLC38A9	-0.15668	0.006981
SLC39A10	0.387483	0.000385
SLC39A11	-0.361	9.04E-07
SLC39A14	0.132138	0.002408
SLC39A4	0.610059	0.003595
SLC40A1	-1.58615	7.57E-05
SLC41A1	0.166162	0.009624
SLC41A2	-0.31904	0.032288
SLC43A1	0.447449	0.020696
SLC43A3	0.468655	6.34E-10
SLC44A2	-0.38371	0.000315
SLC44A3	-0.60441	6.83E-09
SLC44A3-A	-0.44119	9.64E-05
SLC44A5	-0.36115	0.011067
SLC45A1	1.86628	0.034479
SLC45A3	0.382039	0.007899

SLC46A1	-0.53558	8.19E-09
SLC47A2	-0.63621	0.006988
SLC4A11	0.32898	0.038927
SLC4A7	0.333786	0.007877
SLC4A8	-0.36895	0.003622
SLC51A	-0.48981	0.000156
SLC52A1	-0.42831	1.64E-05
SLC5A12	-0.70276	4.97E-09
SLC5A3	0.287289	0.008447
SLC5A5	-1.37252	0.008946
SLC5A6	0.406961	0.000352
SLC6A15	0.388066	3.25E-05
SLC6A2	-1.18395	5.62E-07
SLC6A20	0.436565	0.02894
SLC6A8	0.275513	0.004453
SLC7A2	0.808545	4.21E-16
SLC7A4	-0.98306	0.021735
SLC7A5P1	0.964914	0.041258
SLC7A7	-1.06744	2.47E-14
SLC8A1	-0.25406	0.033961
SLC9A1	0.323475	0.003236
SLC9A3R2	0.638332	0.001647
SLC9A7P1	-0.90747	0.011863
SLC9A8	-0.14967	0.040622
SLC9A9	-0.56575	6.25E-06
SLC9B2	0.354907	0.000527
SLC9C1	-0.92129	0.011203
SLCO1A2	-0.86955	0.000173
SLCO2A1	0.293032	0.028095
SLCO4A1	1.374674	6.21E-36
SLCO4C1	-0.92859	0.034482
SLCO6A1	-1.55932	0.004521
SLFN11	0.439777	1.87E-11
SLFN13	-0.29481	0.015704
SLIRP	0.198797	0.004805
SLIT2	0.652986	0.000468
SLPI	-0.57949	0.021429
SMAD3	0.190624	0.018211
SMAD6	-0.53522	0.021602
SMAGP	0.50144	1.86E-06
SMARCA1	-0.29673	0.000413
SMARCA2	-0.15264	0.008103
SMARCA4	0.377114	0.000454
SMARCB1	0.226602	0.013066
SMARCC1	0.184204	0.0006
SMARCC2	0.159554	0.001909
SMARCD1	0.322468	1.50E-07

SMARCD3	-0.26011	0.039182
SMC1A	0.147306	0.001585
SMC2	0.279395	0.002036
SMC2-AS1	-0.58708	0.001526
SMC4	0.195387	0.033864
SMG1P2	0.376979	0.004471
SMG1P4	0.421903	0.028716
SMG1P7	-0.32563	0.005156
SMG5	0.211263	0.00119
SMG9	0.232	0.0013
SMIM14	-0.54173	0.001637
SMIM20	0.19083	0.02642
SMIM3	0.700766	1.42E-18
SMIM7	-0.12244	0.04947
SMN2	0.214956	0.005243
SMNDC1	0.162759	0.038086
SMO	-0.37211	0.01143
SMOX	0.494937	2.51E-08
SMPD2	0.340189	0.001424
SMPD3	0.553529	0.01528
SMPD4	0.268654	0.005297
SMPDL3A	-0.18606	0.037505
SMPDL3B	-0.65883	0.000256
SMS	0.26297	1.52E-05
SMTN	0.512464	0.000969
SMURF1	0.185637	0.009522
SMURF2	0.170279	0.040629
SMYD5	0.404259	1.79E-08
SNAI1	0.836286	0.000132
SNAI2	0.42696	2.27E-09
SNAPC2	0.368515	0.035531
SNAPC3	-0.17097	0.048308
SNAPC4	0.434259	0.005959
SNCAIP	-0.32576	0.020136
SNCB	1.139291	0.000457
SNCG	-1.18193	7.49E-11
SNHG1	0.366108	1.95E-05
SNHG12	0.22958	0.013891
SNHG14	-0.36625	2.07E-05
SNHG15	0.470374	6.55E-08
SNHG16	0.209468	0.007788
SNHG17	0.37196	0.000118
SNHG23	0.701304	0.00187
SNHG4	0.203943	0.029679
SNHG5	0.256891	0.003152
SNHG6	0.128355	0.031259
SNHG9	0.366463	0.033797

SNN	-0.25966	0.000449
SNORA20	-1.83584	0.028213
SNORA70	0.873252	0.002309
SNORA71C	1.215881	7.51E-05
SNORA73B	0.651287	0.041907
SNORD17	0.634473	0.000214
SNORD46	1.064963	0.043813
SNPH	-0.34431	0.031111
SNRK	0.20348	0.001541
SNRNP200	0.169968	5.64E-05
SNRNP25	0.278882	0.001757
SNRNP40	0.306012	5.22E-08
SNRPA	0.388156	0.004496
SNRPA1	0.248473	2.55E-05
SNRPB	0.439655	2.25E-07
SNRPB2	0.153515	0.035022
SNRPC	0.214285	0.005963
SNRPD1	0.313287	6.07E-05
SNRPD2	0.20045	0.001976
SNRPE	0.250308	0.002264
SNRPF	0.308855	8.06E-06
SNRPG	0.229115	0.001368
SNRPN	-0.18819	0.024339
SNTB2	-0.43214	5.98E-05
SNU13	0.328205	2.49E-06
SNURF	-1.28435	0.000386
SNX12	0.130249	0.008814
SNX13	-0.16275	0.029042
SNX14	-0.19443	0.010278
SNX19	-0.23165	1.58E-05
SNX24	-0.31043	0.001308
SNX29	0.204256	0.017796
SNX33	-0.31453	0.000107
SNX4	-0.25976	2.50E-05
SNX5	0.299883	9.23E-10
SNX7	-0.22412	0.005326
SNX9	0.316896	1.43E-08
SOCS2	0.400662	0.000688
SOCS3	0.601633	0.012175
SOD2	-0.59032	4.66E-05
SOD3	-1.47049	4.20E-05
SOGA1	-0.163	0.049212
SOGA3	-0.42774	0.037186
SORCS3	2.649865	1.29E-05
SORD	0.387735	3.71E-18
SORD2P	0.443839	3.95E-05
SORL1	0.240312	2.81E-05

SORT1	-0.69412	4.54E-11
SOS1	-0.33177	3.61E-05
SOS2	-0.42114	7.93E-07
SOWAHB	-1.29309	0.000321
SOX13	0.567531	2.28E-07
SOX2	-0.39142	0.007156
SOX4	-0.24415	0.001387
SOX6	-0.49617	1.71E-05
SOX7	0.265472	0.011742
SOX9	0.90106	4.92E-21
SP140L	-0.20427	0.001956
SP2	0.231748	0.021944
SPAG16	-0.24787	0.006526
SPAG5	0.249932	0.000543
SPAG7	0.186394	0.010782
SPANXA1	0.984758	0.027389
SPANXA2	0.984758	0.027389
SPANXB1	0.815957	0.027168
SPARC	-0.54203	0.007911
SPATA13	0.408066	6.62E-08
SPATS2L	0.127735	0.007182
SPDL1	0.199978	0.044247
SPECC1	-0.16575	0.000168
SPEF2	-0.5516	0.014871
SPEG	-0.3669	0.014627
SPG11	-0.22658	0.008344
SPG21	0.170922	0.003781
SPIDR	-0.19184	0.008093
SPIN4	0.276197	0.000795
SPINK5	-0.43132	0.000537
SPINT1	-0.19026	0.049272
SPOCK1	0.593239	6.09E-07
SPOCK2	-0.64119	0.015381
SPOUT1	0.334822	0.000246
SPPL2A	-0.32433	1.83E-06
SPPL3	-0.14314	0.016598
SPRR1B	1.255878	1.36E-05
SPRR2A	1.268681	0.00625
SPRR2C	2.897736	0.021295
SPRR2F	3.688058	0.005052
SPRY2	1.049006	3.53E-35
SPRY4	0.681325	4.31E-23
SPRYD3	0.233622	0.000489
SPTB	0.482973	0.016436
SPTBN5	0.768934	0.018155
SPTSSA	-0.32066	0.002765
SQLE	0.307575	6.51E-05

SRA1	0.186262	0.041563
SRBD1	0.139281	0.016838
SRCAP	0.176013	0.010392
SRFBP1	0.209042	0.020617
SRGAP1	0.229784	0.037875
SRGAP2	0.180114	0.001929
SRGAP2D	0.408101	7.64E-05
SRGAP3	-0.38105	0.00013
SRL	-1.87085	0.012785
SRM	0.551433	0.000264
SRP14-AS1	-0.41046	8.08E-05
SRP68	0.173534	0.000678
SRP72	0.213032	0.001454
SRPK1	0.214756	0.007798
SRPRB	0.154845	0.00417
SRPX	0.244827	0.009562
SRRM1	0.140265	0.010103
SRRT	0.307206	0.001002
SRSF7	0.170955	0.032021
SRSF8	-0.16304	0.027762
SRSF9	0.268493	0.000119
SSB	0.198938	0.045289
SSBP1	0.160409	0.041755
SSC5D	-1.11087	0.00555
SSFA2	0.345484	5.94E-07
SSNA1	0.299827	0.015306
SSPN	-0.8891	3.30E-26
SSR2	0.14749	0.011265
SSRP1	0.375719	8.92E-11
SSSCA1	0.370691	0.018155
ST3GAL5	0.365757	0.025112
ST3GAL6	0.3113	0.002085
ST3GAL6-A	0.870343	0.000534
ST6GAL1	-0.25415	0.000286
ST6GALNA(	0.370627	0.027509
ST7	0.142711	0.034487
ST7-OT4	-1.27632	0.027038
ST8SIA4	0.700303	0.005569
ST8SIA6	-1.43538	0.000487
STAB1	2.628852	0.044174
STAC	0.470748	6.52E-06
STAG1	0.193522	0.009679
STAG3L1	-0.32159	0.005938
STAM	0.167111	0.011299
STAMBPL1	0.490441	8.02E-09
STARD10	-0.29103	0.025098
STARD4-AS	-0.29795	0.049276

STARD5	-0.54051	0.013657
STARD7	-0.13903	0.001316
STAT2	-0.45589	0.016218
STAT3	-0.24258	0.047218
STAT5A	-0.32556	0.017714
STAU1	0.145663	0.00017
STC2	0.672554	2.88E-14
STEAP1	0.496115	6.21E-07
STEAP1B	2.279118	0.023819
STIL	0.249747	0.002159
STIP1	0.324485	8.34E-08
STK10	0.359958	6.47E-07
STK11IP	0.243428	0.005623
STK17B	0.479156	4.35E-07
STK24	0.236185	4.60E-07
STK31	-0.77436	8.75E-08
STK32A	-1.23919	0.002954
STK32C	0.317504	0.012221
STK35	0.166707	0.023406
STK38	-0.32649	1.53E-15
STK38L	-0.42582	8.91E-05
STK4	0.254221	0.000579
STMN3	1.169688	1.24E-08
STN1	0.148775	0.006359
STOM	0.15104	0.017807
STOML2	0.388059	5.02E-05
STON1	-0.7529	6.18E-07
STON2	-0.28283	0.002146
STOX2	-0.83102	2.50E-11
STRAP	0.181754	0.000383
STRIP1	0.113135	0.035961
STRIP2	0.194011	0.014322
STS	-0.34925	0.0013
STX10	0.207003	0.048137
STX12	-0.35516	0.000309
STX19	-1.13025	0.004324
STX1A	0.619781	6.52E-06
STX7	-0.23047	0.005219
STXBP5	-0.25255	0.008802
SUCLG1	0.108868	0.023986
SUCLG2-AS	-0.80508	0.047682
SUDS3	-0.14143	0.020543
SUGP2	0.106235	0.03937
SULF1	-0.59798	0.035281
SULT1E1	-0.84594	0.000161
SUMF1	-0.35869	0.000101
SUMO3	0.144596	0.039506

SUN3	0.402299	0.011627
SUOX	-0.2378	0.0243
SUPT16H	0.185301	0.002333
SUPT5H	0.176702	0.005979
SUPT7L	-0.11203	0.02412
SUPV3L1	0.149292	0.02033
SURF2	0.504959	0.000877
SURF6	0.333158	0.020864
SUSD2	-0.69306	0.009696
SUSD4	0.705551	0.000186
SUV39H1	0.350937	0.008995
SVEP1	-0.20572	0.038427
SVIL	-0.18699	0.005189
SWI5	0.211595	0.017707
SYBU	-0.34224	0.0143
SYCP2L	-0.5192	0.041231
SYDE2	-0.34112	0.019621
SYMPK	0.284898	0.000333
SYNC	-0.72826	1.60E-15
SYNCRIP	0.166392	0.0269
SYNE1	-0.28556	6.69E-05
SYNE2	-0.37407	1.38E-05
SYNE3	0.966121	1.80E-35
SYNJ2	0.420771	1.09E-16
SYNJ2BP	-0.28252	4.16E-05
SYNPO	-0.67417	1.12E-06
SYNPO2	-1.56438	2.84E-16
SYS1	0.176785	0.004987
SYT15	-0.67549	1.32E-09
SYT17	-0.47229	0.005134
SYT7	0.382385	0.00047
SYTL2	-0.81822	0.001342
SYTL3	0.30368	1.81E-07
SZRD1	0.231233	0.00022
TACC1	0.186862	0.006343
TACC3	0.359514	0.004671
TACSTD2	-0.81788	5.04E-07
TAF12	0.137309	0.047754
TAF1A	0.301035	0.011006
TAF1D	0.245236	0.00811
TAF3	0.170968	0.018936
TAF4	0.29969	3.70E-06
TAF4B	0.25387	3.40E-05
TAF5	0.241863	0.042447
TAF5L	0.127103	0.033094
TAF6	0.248236	0.026435
TAF9	0.166325	0.011085

TAGLN	-1.18486	6.85E-12
TAGLN3	1.683003	1.92E-15
TAL2	-3.18482	0.0275
TANC1	0.103657	0.040667
TANGO6	0.327493	6.28E-08
TANK	-0.18346	0.048233
TAP1	-0.39802	0.00611
TAP2	0.182376	0.000595
TAPBP	-0.23126	0.014198
TAPBPL	-0.29064	0.022219
TAPT1	-0.23406	0.008435
TARS	0.179967	0.007213
TARS2	0.173912	0.005256
TAS1R3	0.724129	0.034264
TAS2R4	-0.99977	0.021874
TATDN2	0.192881	0.002349
TBC1D1	0.224235	0.000139
TBC1D10C	-0.97763	0.004409
TBC1D12	-0.15858	0.017241
TBC1D20	-0.17653	0.001304
TBC1D23	-0.29296	0.002483
TBC1D26	-2.06352	0.031046
TBC1D31	0.217017	0.005683
TBC1D32	-0.33305	0.007692
TBC1D8	0.349008	2.16E-06
TBC1D8B	-0.36943	7.54E-06
TBC1D9	-0.68961	3.88E-11
TBCD	0.185527	0.032683
TBCEL	-0.55439	2.96E-09
TBCK	-0.27682	0.002282
TBK1	-0.19877	0.018645
TBKBP1	0.524102	0.002615
TBL1X	0.134627	0.047489
TBL1XR1	0.180924	0.02312
TBL3	0.42255	0.015705
TBP	0.135143	0.02707
TBPL1	0.162327	0.013132
TBRG4	0.447737	9.57E-09
TBX2	1.286965	0.017508
TBX21	2.359813	1.41E-17
TCEAL4	-0.32844	0.000471
TCEAL8	-0.19555	0.013759
TCEANC	-0.3624	0.044707
TCERG1	0.259578	9.14E-06
TCERG1L	0.763767	2.83E-11
TCF19	0.205929	0.013217
TCF20	0.228726	1.39E-05

TCF21	0.690502	0.046194
TCF3	0.479455	0.005393
TCF4	-0.18408	0.015898
TCF7	0.389551	0.00093
TCHHL1	-2.38321	0.01805
TCHP	0.217136	0.001808
TCN2	-0.457	0.007526
TCOF1	0.71716	1.44E-19
TCP1	0.308189	4.43E-06
TCP11L1	-0.30422	0.00034
TCP11L2	-0.23781	0.012669
TCTN1	-0.19429	0.003219
TDO2	-0.39394	0.016971
TDP1	0.112218	0.030145
TDRD7	-0.64831	0.004569
TDRP	-0.27987	0.000123
TEAD4	0.53579	5.96E-07
TELO2	0.421472	0.041561
TENM1	-0.67686	0.00019
TENM2	-0.44156	2.92E-12
TENM3	0.262742	0.014729
TENM4	-0.30322	0.005458
TERF2	0.138656	0.044524
TERT	1.784748	8.45E-06
TET1	-1.55809	1.60E-14
TET3	0.176137	0.003084
TEX10	0.35508	1.88E-07
TEX14	-0.78089	0.023086
TEX19	1.211146	0.019352
TEX30	0.232532	0.00176
TEX40	0.640126	1.77E-06
TFAM	0.259055	0.0027
TFAP4	0.486734	0.000414
TFCP2L1	-0.72306	3.12E-18
TFDP1	0.179183	0.000137
TFDP2	-0.24748	4.99E-05
TFPI	-0.27633	0.012763
TFPI2	1.561024	5.93E-23
TFRC	0.276013	0.016342
TG	-0.6021	0.002275
TGFA	0.57661	1.07E-13
TGFB1	0.365226	0.030535
TGFB2	-1.15297	2.26E-26
TGFB2-AS1	-1.17316	3.05E-13
TGFB2-OT1	-7.18399	0.017698
TGFB3	-0.30598	0.037904
TGFBR3	0.580175	1.77E-08

TGIF2-C20c	0.603278	0.016207
TGM5	-1.38112	0.045133
TGS1	0.177126	0.006336
THADA	0.246339	5.89E-05
THAP4	0.50261	6.45E-05
THAP7-AS1	-0.39738	0.001824
THBD	0.406024	0.020019
THBS2	-0.46377	0.03752
THBS3	-0.40237	0.000606
THCAT158	-1.48719	0.027965
THEG	1.102546	0.001644
THEM4	0.152026	0.042726
THOC5	0.220385	1.85E-05
THOP1	0.427621	0.003683
THRB	-0.26545	0.04678
THSD4	-0.29458	0.001964
THY1	0.679379	0.00179
TIAM1	0.630449	1.55E-18
TIAM2	-0.30105	0.009633
TICRR	0.352585	0.00745
TIGD2	-0.35964	3.29E-06
TIGD6	-0.18887	0.027847
TIMELESS	0.28089	1.53E-06
TIMM10	0.440136	3.59E-05
TIMM13	0.330554	0.010234
TIMM17B	0.284386	0.014731
TIMM22	0.241855	0.000552
TIMM23	0.200775	6.46E-05
TIMM23B	0.171599	0.013328
TIMM44	0.349824	0.00064
TIMM50	0.330614	3.61E-05
TIMM8A	0.175791	0.041964
TIMM9	0.17121	0.004234
TIMP1	0.724555	1.31E-05
TIMP2	-0.5531	3.68E-11
TIPIN	0.32929	0.000112
TIRAP	-0.31059	0.000878
TJP2	0.234829	2.31E-07
TJP3	-1.35522	0.014528
TK1	0.314195	0.017131
TK2	-0.15119	0.018274
TKFC	0.236636	0.033612
TKT	0.216658	0.035123
TLCD2	-0.76481	1.26E-07
TLDC2	-1.18642	0.004363
TLE3	0.342918	0.003528
TLE4	-0.31694	0.001396

TLL1	-0.2946	0.000397
TLR2	-0.36234	0.000628
TLR5	-0.54335	0.000655
TM4SF1	0.150052	0.031043
TM4SF19	0.483589	6.84E-07
TM4SF19-A	0.321489	0.018935
TM7SF3	-0.36018	8.94E-05
TM9SF2	-0.17865	0.001687
TM9SF4	-0.18761	0.026429
TMA16	0.192757	0.007828
TMBIM1	-0.14159	0.049048
TMBIM4	-0.17364	0.015144
TMC1	-0.70722	0.030714
TMC3	-0.46799	0.023995
TMC4	-1.10781	8.09E-06
TMC7	0.218073	0.03091
TMCC2	-0.41817	0.015535
TMCC3	0.459035	0.000236
TMCO3	-0.15005	0.003931
TMCO6	0.328725	0.000187
TMED1	0.229279	0.028042
TMED10	-0.12929	0.00687
TMED10P2	-1.32388	0.012155
TMED4	-0.16402	0.028072
TMED7-TIC	-0.53644	0.000299
TMEM106B	-0.30572	0.002145
TMEM106C	0.097053	0.041451
TMEM109	0.188594	0.027916
TMEM11	0.29272	0.000425
TMEM116	-0.24402	0.039576
TMEM117	-0.36907	0.000156
TMEM123	-0.22175	0.001721
TMEM126A	0.191539	0.035759
TMEM128	-0.23132	0.004349
TMEM132B	-1.1621	0.004111
TMEM133	-0.54623	0.000825
TMEM140	-0.45022	0.004355
TMEM141	0.309626	0.00134
TMEM144	-0.27827	0.002736
TMEM145	0.75029	0.004466
TMEM147	0.341592	0.008704
TMEM150C	-0.93535	0.009231
TMEM154	0.227299	0.009443
TMEM156	0.362363	0.012629
TMEM159	-0.35437	0.005984
TMEM161B	-0.22018	0.037457
TMEM164	0.344383	4.81E-08

TMEM165	-0.30658	7.25E-11
TMEM168	-0.3153	0.005963
TMEM170A	0.185634	0.042912
TMEM171	0.314287	0.047949
TMEM177	0.291675	0.004077
TMEM178E	-1.26006	0.000178
TMEM18	0.132946	0.023888
TMEM185A	-0.25174	0.006546
TMEM19	-0.2342	0.00183
TMEM200A	0.992477	8.57E-12
TMEM201	0.680823	0.000144
TMEM204	0.506867	0.003473
TMEM206	0.300104	0.000299
TMEM208	0.133942	0.027529
TMEM209	0.149647	0.032517
TMEM229E	-0.3148	0.00341
TMEM232	-1.24018	0.027072
TMEM237	-0.20025	0.000114
TMEM254	-0.22793	0.000414
TMEM255A	-1.30856	1.62E-17
TMEM258	0.120481	0.032377
TMEM260	-0.28114	0.000154
TMEM261	0.282403	0.007108
TMEM268	0.250642	2.83E-06
TMEM27	-0.46923	6.76E-05
TMEM30B	-0.37996	1.53E-08
TMEM33	0.204823	0.001953
TMEM35B	-0.18859	0.047153
TMEM38B	0.212756	0.035526
TMEM39B	0.292527	0.011
TMEM44-A	-0.3687	0.017566
TMEM45A	-0.24493	0.009628
TMEM5	0.127312	0.015198
TMEM50A	-0.11865	0.018529
TMEM50B	-0.25924	0.001827
TMEM52B	-0.67294	0.013836
TMEM56	-0.40248	0.037724
TMEM59	-0.33947	0.000214
TMEM62	-0.57145	6.96E-07
TMEM63B	-0.31053	0.005329
TMEM63C	-0.50444	0.002649
TMEM69	0.173227	0.005858
TMEM86A	-0.66923	0.000338
TMEM9	-0.21518	0.00915
TMEM92	0.387342	0.001682
TMEM97	0.314039	0.001636
TMEM9B	-0.14045	0.009496

TMEM9B-A	-0.78132	0.006807
TMLHE	-0.22894	0.005889
TMOD1	-0.8558	0.021587
TMOD2	-0.69539	2.03E-07
TMPO	0.202233	0.002978
TMPRSS11I	0.534939	0.009937
TMPRSS13	-0.98973	0.000622
TMSB10	0.240146	4.21E-06
TMTC1	-0.27247	0.030489
TMTC2	-0.32557	0.000687
TMX2	0.156631	0.01452
TNC	-0.92164	7.33E-20
TNFAIP2	-0.92237	0.003197
TNFAIP8L2	-1.8994	0.02252
TNFRSF10A	0.458171	1.16E-08
TNFRSF10B	0.188253	0.004207
TNFRSF10D	0.494149	0.005657
TNFRSF12A	0.402153	0.003213
TNFRSF14	-0.40461	0.02258
TNFRSF19	0.61905	2.27E-17
TNFRSF1B	0.319124	0.006016
TNFRSF25	0.448898	0.004473
TNFRSF6B	1.822481	0.002551
TNFRSF8	1.132568	1.14E-08
TNFRSF9	-0.59671	0.000192
TNFSF10	-1.20118	0.000673
TNFSF13B	-1.29714	0.000205
TNFSF18	0.835492	0.04538
TNIK	0.797997	3.63E-27
TNIP1	0.213307	0.011193
TNIP2	0.315104	0.006272
TNIP3	-0.85607	7.31E-06
TNNT1	0.399694	0.004751
TNNT3	-1.02376	0.003607
TNPO2	0.163944	0.003384
TNRC6A	-0.1609	0.004182
TNRC6C-AS	-0.43301	0.000971
TNS3	-0.35736	0.000147
TNS4	0.622394	9.43E-08
TOE1	0.276721	0.014464
TOMM22	0.243976	0.000117
TOMM34	0.660941	2.71E-13
TOMM40	0.544346	1.69E-05
TOMM5	0.350022	5.48E-06
TOMM6	0.254812	0.001108
TONSL	0.481525	0.010437
TOP1	0.179863	0.008405

TOP1MT	0.461189	1.31E-05
TOP2A	0.250712	0.005771
TOP3A	0.292758	2.71E-06
TOP3B	0.172803	0.036285
TOPBP1	0.172202	0.000469
TOPORS-AS	-0.39349	0.038754
TOR4A	0.590791	0.00687
TP53AIP1	-0.53074	1.48E-06
TP53I3	-0.39714	7.25E-05
TP53INP1	-0.98024	4.04E-11
TP53INP2	-0.73685	1.71E-10
TP53TG1	-0.41451	0.003412
TPBG	0.286458	0.00265
TPD52L1	-0.15564	0.010765
TPD52L2	0.193585	8.98E-05
TPI1	0.16279	0.007392
TPI1P2	-0.66991	0.01033
TPM1	-0.70118	8.68E-26
TPM3	0.139323	0.008719
TPM4	-0.2729	3.59E-08
TPP2	0.143203	0.028831
TPRN	0.461801	0.011307
TPT1	0.170123	0.007436
TPTEP1	-0.36069	0.006998
TPX2	0.345583	5.10E-06
TRABD2A	0.705245	2.44E-14
TRAF2	0.284632	0.018204
TRAF3IP1	-0.17401	0.025748
TRAF3IP2-A	0.353223	0.020211
TRAF3IP3	-0.84011	0.005091
TRAF5	-0.21633	0.019828
TRAF7	0.273105	0.025586
TRAFD1	-0.36504	0.002367
TRAK2	-0.21139	0.002105
TRAM1	-0.35298	0.000537
TRAM2	0.192625	0.005479
TRANK1	-0.89196	0.003913
TRAP1	0.516473	5.65E-11
TRAPPC2	-0.14605	0.023692
TRAPPC6A	-0.55792	0.001318
TRAPPC9	-0.34886	0.006268
TRBC2	1.749956	1.31E-08
TREML2	1.626195	0.003437
TRERF1	0.393388	1.82E-06
TRIB1	0.628944	2.04E-08
TRIB3	0.427623	0.002344
TRIM16	-0.26641	0.00019

TRIM16L	-0.28872	0.005382
TRIM2	-0.76171	1.05E-10
TRIM21	-0.31085	0.00964
TRIM22	-1.13214	0.001847
TRIM24	-0.14899	0.02658
TRIM28	0.39488	0.00112
TRIM29	-0.3643	0.033735
TRIM34	-0.53349	1.33E-08
TRIM36	0.263381	0.027399
TRIM38	-0.2819	0.00285
TRIM4	-0.23103	0.010412
TRIM55	-0.7935	0.005852
TRIM56	-0.41351	7.26E-09
TRIM6	-0.45773	7.47E-09
TRIM68	-0.12788	0.038931
TRIM69	-0.5251	0.00046
TRIM73	-0.37997	0.018303
TRIM8	0.271727	0.010241
TRIO	-0.13083	0.003072
TRIP13	0.397812	6.75E-05
TRIQK	-0.35293	0.002054
TRIT1	0.114851	0.042584
TRMT1	0.530927	3.80E-05
TRMT112	0.302285	0.000343
TRMT1L	-0.22089	0.015132
TRMT2A	0.283071	0.046166
TRMT6	0.31958	2.74E-09
TRMT61A	0.471789	0.011222
TRNP1	0.211586	0.041711
TROAP	0.434073	0.001949
TRPA1	-0.71562	0.025624
TRPM2	0.539778	0.002087
TRPM4	-0.34175	0.012686
TRPM7	-0.15909	0.025128
TRPV1	-0.27929	0.016417
TRPV3	0.612857	0.000947
TRPV6	-1.42047	0.000144
TRRAP	0.206856	0.000349
TRUB2	0.226977	1.19E-05
TSC1	-0.12341	0.025264
TSC22D1	0.304276	7.35E-06
TSC22D3	-0.22487	0.006307
TSEN2	0.270919	0.001675
TSEN54	0.243326	0.042583
TSFM	0.174202	0.005889
TSHZ2	-0.3437	0.012062
TSHZ3	0.172118	0.03371

TSIX	0.259346	0.008474
TSPAN2	-0.53605	4.06E-06
TSPAN3	-0.2114	0.000183
TSPAN31	-0.35902	0.001314
TSPAN32	-1.34721	0.035161
TSPAN5	0.206977	0.002464
TSPEAR-AS	-0.72297	0.010241
TSPEAR-AS	-0.92864	0.005242
TSPOAP1	-0.83902	0.001177
TSPOAP1-A	-1.18372	0.040182
TSPYL2	0.28014	0.002849
TSR1	0.410721	4.16E-18
TSSC1	0.210184	0.008923
TSSK3	-0.75431	0.002854
TSTA3	0.3297	0.001685
TSTD3	-0.45987	0.006278
TTC13	-0.62507	1.64E-05
TTC23	-0.16842	0.0145
TTC27	0.218356	0.003289
TTC28	-0.45685	0.018659
TTC28-AS1	-0.67409	1.16E-10
TTC3	-0.21587	0.000487
TTC30A	-0.41153	2.12E-06
TTC30B	-0.30092	0.005078
TTC34	-0.48846	0.008365
TTC3P1	-0.33753	0.034682
TTC4	0.172735	0.00072
TTC9B	1.209038	0.036082
TTF1	0.123555	0.045228
TTI1	0.23107	0.000475
TTI2	0.166275	0.034074
TTK	0.344484	0.000987
TTL	0.257637	4.41E-07
TTLL1	-0.53241	0.007911
TTLL12	0.591935	2.11E-05
TTLL4	0.39426	1.86E-10
TTLL5	0.106743	0.022925
TTLL7	-0.57852	2.91E-07
TTN	-0.27412	0.004701
TTYH2	0.426778	0.000627
TUB	0.335308	0.005059
TUBA1A	-0.63073	1.36E-09
TUBA1B	0.337865	1.70E-05
TUBA1C	0.229706	0.01363
TUBB	0.182022	0.012944
TUBB3	0.283911	0.041849
TUBB4B	0.3632	0.005697

TUBB6	0.442635	2.95E-05
TUBB8P12	-1.25134	0.032954
TUBG1	0.351193	3.88E-05
TUBGCP2	0.250334	0.004053
TUBGCP3	0.141158	0.003187
TUBGCP4	0.129426	0.036802
TUFM	0.401163	2.30E-06
TUG1	-0.18357	0.005287
TULP4	-0.14031	0.022685
TUT1	0.289161	0.001937
TWIST1	0.339026	0.010457
TWISTNB	0.175318	0.008959
TWNK	0.372334	1.03E-07
TXLNG	0.236155	0.000582
TXNDC12	0.138949	0.00152
TXNDC15	-0.15835	0.014909
TXNDC17	0.236399	0.005876
TXNDC5	0.215228	0.000214
TXNL4A	0.155607	0.014244
TXNRD3	-0.25504	0.007121
TYMS	0.162616	0.037136
U2AF1	0.212577	0.003104
U2AF2	0.373535	0.000317
U47924.27	-0.4368	0.028361
UACA	0.294256	0.000104
UAP1	0.425948	1.56E-06
UBA1	0.191734	0.001335
UBA2	0.249308	5.04E-06
UBA52	0.146183	0.005043
UBA7	-0.51827	0.005419
UBALD2	0.29757	0.005855
UBAP2L	0.109485	0.038186
UBASH3B	0.488501	5.49E-12
UBD	-1.79047	9.06E-05
UBE2C	0.463893	0.000443
UBE2E2	0.198619	0.041062
UBE2H	-0.24369	0.009073
UBE2I	0.235821	0.000272
UBE2J1	-0.1703	0.00804
UBE2M	0.263436	0.005485
UBE2N	0.151606	0.007623
UBE2O	0.21835	0.010591
UBE2Q2P1	-0.54228	0.014308
UBE2QL1	-1.41196	1.14E-08
UBE2R2	0.147055	0.027837
UBE2S	0.453139	0.031221
UBE2T	0.279163	0.000117

UBE3B	-0.15261	0.01848
UBIAD1	0.225265	0.025241
UBL3	-0.35476	4.69E-06
UBL4A	0.203698	0.010398
UBL7	0.247181	0.008515
UBN2	-0.34827	0.000297
UBOX5	0.280089	0.001678
UBQLN2	-0.18112	0.00184
UBQLN4	0.23647	7.24E-05
UBQLN4P1	1.06482	0.009859
UBR4	0.125843	0.008787
UBXN1	0.235866	0.014238
UBXN11	0.314162	0.001603
UBXN2B	-0.39139	1.29E-05
UBXN4	-0.2094	0.0143
UCHL3	0.311221	0.000161
UCHL5	0.152574	0.049059
UCK2	0.442662	1.69E-08
UCKL1	0.236598	0.048262
UCP2	0.483314	0.037983
UEVLD	-0.29637	0.000327
UGCG	0.345408	0.002216
UGDH-AS1	-0.36884	4.32E-05
UGGT2	-0.34788	0.00164
UGP2	-0.18384	0.019998
UGT1A1	-0.30858	0.003555
UGT1A10	-0.74039	2.42E-13
UGT1A2P	-0.70886	0.049284
UGT1A3	-0.66854	0.008005
UGT1A6	-0.41993	1.22E-05
UGT1A7	-0.76422	0.008366
UGT1A8	-0.95738	1.85E-10
UGT2B17	-1.61104	1.55E-06
UGT8	0.60976	9.25E-06
UHRF1	0.546319	1.01E-06
UIMC1	0.24159	0.004682
ULBP3	0.261527	0.031356
ULK2	-0.38315	5.88E-07
UNC13B	-0.2472	0.013637
UNC13C	-0.77059	0.049708
UNC13D	0.507698	0.001162
UNC50	-0.1344	0.045441
UNC93A	-0.72385	4.87E-05
UNG	0.195027	0.022619
UNGP3	-1.31565	0.00152
UPF1	0.229451	0.03383
UPK2	-0.86048	0.005962

UPK3B	-1.10386	0.000116
UPP1	0.959374	2.24E-21
UQCRC1	0.18953	0.045116
UQCRFS1	0.209366	0.000347
URB1	0.265844	1.62E-10
URB2	0.309152	0.000488
URM1	0.243658	0.005493
USE1	0.235401	0.039931
USP1	0.178198	0.033518
USP10	0.129615	0.011298
USP17L4	3.404487	0.002231
USP2-AS1	0.948903	5.03E-07
USP3	0.192499	0.001469
USP30-AS1	-1.00921	0.025624
USP31	-0.28983	0.001008
USP32P1	-0.3552	0.013045
USP32P2	-0.53044	0.001361
USP32P3	-0.44962	0.00081
USP33	-0.24537	0.002856
USP36	0.340447	1.46E-07
USP39	0.165995	0.00347
USP4	0.138352	0.040219
USP46-AS1	-0.74002	4.89E-06
USP49	-0.29199	0.001672
USP9X	-0.12948	0.040515
UST	-0.60228	1.73E-12
UTAT33	-0.97683	0.0434
UTP11	0.250829	0.001107
UTP14A	0.285875	3.56E-06
UTP14C	-0.18593	0.042346
UTP18	0.367198	7.23E-08
UTP20	0.220287	0.002032
UTP4	0.380298	2.73E-19
UTP6	0.187802	0.015699
UTRN	-0.30115	2.37E-06
UXT	0.167726	0.019505
VAC14	0.281028	0.008684
VAMP1	-0.17376	0.046605
VAMP4	-0.19511	0.046835
VAMP8	-0.23967	0.000848
VANGL2	-0.34422	3.38E-06
VARS	0.541136	3.90E-05
VASH1	-0.39461	0.008615
VASN	0.5742	0.027674
VASP	0.434153	0.000226
VAV2	0.228418	0.002905
VAV3	-0.3559	1.57E-06

VCPKMT	-0.16519	0.032094
VDAC1	0.244055	9.88E-05
VEGFA	0.616538	6.78E-09
VEGFC	0.141056	0.019184
VEPH1	-0.3988	0.010566
VGF	1.042971	0.00156
VGLL1	-0.70391	0.004943
VGLL4	-0.14973	0.028338
VIL1	-1.60408	0.008909
VIM	0.474398	0.004237
VIPAS39	-0.11088	0.03914
VIPR1	-0.9762	3.72E-17
VIPR1-AS1	-1.03765	0.0038
VMAC	-0.40715	0.032765
VMP1	-0.16652	0.042294
VOPP1	0.188579	0.005944
VPS13A	-0.20076	0.049862
VPS13B	-0.20451	0.038859
VPS13C	-0.42745	0.000268
VPS13D	-0.19006	0.013199
VPS16	0.177967	0.017477
VPS25	0.120121	0.036724
VPS28	0.224285	0.022539
VPS37B	0.286012	0.025936
VPS39	-0.14445	0.024543
VPS45	-0.20167	0.002063
VPS50	-0.16441	0.047463
VPS52	0.124303	0.027864
VPS54	-0.18939	0.01279
VPS8	-0.1991	0.018012
VPS9D1-AS	0.735607	0.000227
VRK1	0.302373	0.00164
VSIG10	-0.3257	5.05E-05
VSIG10L	-0.35625	0.033698
VSTM2A	2.931255	0.046147
VSTM5	-1.05057	0.044983
VTCN1	-1.69787	0.001866
VWA2	-0.39494	0.004406
VWA3A	-2.68292	0.033201
VWA5A	-0.91804	7.30E-06
VWA8	-0.2419	0.007997
VWA8-AS1	-0.83489	0.021379
VWDE	-0.54603	6.58E-05
VWF	-0.51164	0.017024
WAC-AS1	-0.39987	3.19E-10
WARS2	0.136014	0.025494
WARS2-IT1	-0.42406	0.026615

WASF1	-0.1994	0.003831
WASHC1	0.395602	0.005199
WASHC2C	-0.10244	0.033846
WASHC3	-0.25967	3.84E-05
WBP11	0.192156	0.003544
WBP1L	-0.14885	0.003156
WBP2NL	-0.50249	0.042332
WBSCR22	0.241867	0.000456
WDFY3	-0.14963	0.037598
WDHD1	0.194066	0.019651
WDR11	-0.15338	0.004605
WDR12	0.286452	1.37E-05
WDR3	0.299194	0.000115
WDR31	-0.51981	3.17E-06
WDR36	0.173381	0.033253
WDR37	-0.38556	0.000452
WDR4	0.371646	0.003211
WDR43	0.344787	2.22E-08
WDR44	-0.15436	0.035163
WDR46	0.32728	0.000121
WDR5	0.262662	0.000359
WDR54	0.395413	0.000123
WDR55	0.171221	0.015621
WDR5B	-0.23623	0.046367
WDR62	0.440696	0.001705
WDR64	-2.81751	0.020144
WDR72	-0.23294	0.001703
WDR74	0.44784	2.21E-05
WDR75	0.1673	0.016948
WDR88	-0.5378	0.022713
WDR90	0.552879	0.0037
WDR91	-0.21522	0.005535
WDSUB1	-0.27858	0.006044
WDTC1	-0.18277	0.045022
WFDC2	-1.07351	0.001205
WFDC21P	-0.90602	0.00125
WHAMMP:	-0.35004	0.00437
WHAMMP:	-0.28613	0.028822
WIPI1	-0.64306	5.74E-10
WNK1	-0.11424	0.011281
WNT10A	-0.64258	0.000926
WNT10B	0.621892	0.0164
WNT11	-1.2834	0.007802
WNT2B	-0.54724	1.95E-05
WNT4	-1.15177	1.20E-07
WNT6	-1.23655	0.000126
WRAP53	0.409319	1.35E-05

WRB	-0.21173	0.025971
WRNIP1	0.186176	0.004982
WSCD1	1.322938	4.22E-11
WWC2-AS2	-0.6743	0.035296
WWP1	-0.13853	0.040177
XAB2	0.340838	0.006655
XAF1	-1.03376	0.048418
XCL1	-0.73469	0.00016
XCR1	-1.42385	0.030538
XDH	0.205027	0.008399
XG	-0.74001	6.30E-26
XPO4	0.196701	0.009637
XPO5	0.363685	6.54E-17
XPO6	0.25069	2.12E-07
XPO7	0.170562	0.000884
XPOT	0.215085	0.009298
XPR1	-0.47636	0.000123
XRCC1	0.257117	0.001399
XRCC3	0.44402	0.00189
XRCC5	0.17004	0.002531
XRCC6	0.165801	0.000535
XRN1	-0.22334	0.031602
XRN2	0.190444	0.000319
XRRA1	0.228936	0.038497
XXbac-BPG	-0.33316	0.019176
XXbac-BPG	-0.38534	0.028769
XXyac-YRM	-0.26551	0.041302
XYLB	0.439518	0.002213
XYLT1	0.466776	1.36E-07
YAP1	0.140448	0.031505
YARS	0.256989	2.62E-05
YARS2	0.221455	0.000867
YBX1	0.424077	1.07E-07
YBX1P1	0.515076	0.049572
YBX3	0.185329	0.000253
YEATS2	0.139406	0.020473
YIPF6	-0.2446	0.004203
YKT6	0.169271	0.000739
YOD1	0.334888	0.001683
YPEL3	-0.55921	0.00223
YPEL5	-0.54215	3.14E-07
YRDC	0.409959	1.93E-08
YTHDF3	-0.11588	0.03688
YWHAE	0.165406	0.004579
YWHAG	-0.20092	0.001787
Z84812.4	3.834982	0.001106
ZBED1	-0.38066	0.000511

ZBED2	0.847359	1.93E-08
ZBED3	-0.23789	0.040169
ZBED4	0.204134	0.000191
ZBED5	-0.12855	0.02906
ZBED5-AS1	-0.31599	0.026186
ZBED9	-0.27277	0.001814
ZBTB1	-0.25396	0.001416
ZBTB17	0.413897	0.001666
ZBTB2	0.352896	0.000114
ZBTB38	-0.14854	0.013822
ZBTB39	0.19	0.005181
ZBTB47	-0.28538	0.049521
ZBTB48	0.203423	0.035938
ZBTB5	-0.19945	0.027193
ZBTB9	0.231263	0.002468
ZC3H10	0.265141	0.024953
ZC3H18	0.264334	0.009878
ZC3H7B	0.205405	0.031083
ZC3HAV1	-0.29133	0.010275
ZCCHC2	-0.18196	0.034299
ZCCHC4	0.200473	0.019497
ZCWPW1	-0.21456	0.048548
ZDBF2	-0.28972	0.026405
ZDHHC11B	-0.54209	0.005681
ZDHHC18	0.178343	0.024099
ZDHHC20P	-1.84896	0.033449
ZDHHC21	-0.50555	0.000527
ZDHHC23	-0.44126	0.000735
ZDHHC6	0.110553	0.019049
ZDHHC7	-0.13617	0.016918
ZFAND2B	0.206657	0.024557
ZFAND5	-0.15201	0.006695
ZFAS1	0.192243	0.005942
ZFHX2	-0.88713	0.0059
ZFHX4	-0.24943	0.000966
ZFP1	0.225764	0.005158
ZFP14	-0.59736	4.69E-05
ZFP3	-0.85064	2.33E-05
ZFP30	-0.25029	0.014797
ZFP36	0.38498	0.025604
ZFP36L1	0.482824	3.24E-11
ZFP36L2	0.376225	3.37E-05
ZFP57	-0.7151	0.006325
ZFP69B	0.451804	0.004021
ZFP90	-0.20858	0.000153
ZFPM2-AS1	-0.2966	0.002313
ZFYVE16	-0.17423	0.02955

ZFYVE19	0.193095	0.003042
ZFYVE26	-0.15988	0.006893
ZGPAT	0.355157	0.003293
ZHX1	-0.1771	0.039306
ZIC1	-0.39345	6.92E-05
ZIC2	0.414573	0.001056
ZIC4	-0.7625	8.32E-07
ZKSCAN1	-0.31721	5.58E-06
ZKSCAN3	-0.57181	3.19E-08
ZKSCAN4	-0.26287	0.001933
ZMAT3	-0.21722	0.01879
ZMIZ1-AS1	-0.6615	0.001433
ZMYND19	0.425667	5.60E-06
ZMYND8	0.325337	4.74E-05
ZNF10	-0.44829	6.79E-05
ZNF117	-0.63347	5.38E-06
ZNF124	-0.27742	0.03316
ZNF134	-0.19791	0.004416
ZNF142	0.269813	0.000514
ZNF143	0.212499	0.004996
ZNF182	-0.23957	0.014223
ZNF189	-0.17647	0.031299
ZNF19	-0.41817	0.004335
ZNF202	0.165932	0.007393
ZNF204P	-0.48096	8.32E-06
ZNF211	-0.33004	4.13E-05
ZNF212	0.192752	0.022369
ZNF223	-0.29103	0.030518
ZNF224	-0.26446	0.021028
ZNF226	-0.28031	0.003609
ZNF23	-0.16214	0.038424
ZNF248	-0.40992	5.18E-05
ZNF25	-0.4611	0.00512
ZNF251	-0.2151	0.02755
ZNF252P	-0.27296	0.00461
ZNF253	-0.331	0.024657
ZNF254	-0.47279	0.000108
ZNF260	-0.23445	0.027952
ZNF263	0.162661	0.013045
ZNF264	-0.33188	0.000637
ZNF274	0.159695	0.046766
ZNF276	0.223455	0.022935
ZNF28	-0.21866	0.039202
ZNF280D	-0.27008	0.001403
ZNF281	-0.19464	0.006945
ZNF282	0.437101	0.000951
ZNF30	-0.26776	0.028243

ZNF302	-0.32717	0.000144
ZNF311	-0.32499	0.020241
ZNF319	-0.27636	0.029645
ZNF320	-0.33567	0.004714
ZNF321P	-0.45251	0.00648
ZNF322	-0.22776	0.02458
ZNF329	-0.2244	0.005165
ZNF330	0.24558	0.000299
ZNF334	-0.41448	2.19E-05
ZNF335	0.25023	0.028689
ZNF337-AS	-0.33849	0.00972
ZNF33A	-0.24706	0.001774
ZNF33B	-0.19917	0.007459
ZNF343	0.178233	0.00979
ZNF350	-0.26595	0.013834
ZNF37BP	-0.20905	0.028949
ZNF384	0.210172	0.000219
ZNF385C	-0.65767	0.036806
ZNF391	-0.40764	1.44E-05
ZNF396	-0.71577	0.001096
ZNF416	-0.25915	0.03743
ZNF417	-0.31171	4.15E-05
ZNF420	-0.33156	0.042856
ZNF425	-0.6153	0.000763
ZNF433	-0.34804	0.03646
ZNF436	-0.18611	0.001668
ZNF439	-0.59494	0.030522
ZNF45	-0.17601	0.006609
ZNF474	0.485415	1.97E-05
ZNF48	-0.35262	0.012818
ZNF485	0.252178	0.045496
ZNF496	0.222523	0.014079
ZNF503-AS	-0.60143	0.017652
ZNF506	-0.22091	0.012186
ZNF510	-0.19721	0.026744
ZNF511	0.365014	0.007039
ZNF514	-0.3187	0.000977
ZNF528	-0.33459	1.05E-05
ZNF528-AS	-0.30122	0.010262
ZNF532	-0.1319	0.009029
ZNF540	-0.89245	0.004332
ZNF546	-0.38372	0.042649
ZNF547	-0.38644	0.020667
ZNF552	-0.25174	0.022654
ZNF554	-0.463	0.026282
ZNF561	-0.28108	0.000327
ZNF561-AS	-0.35736	0.004078

ZNF563	-0.39213	0.004243
ZNF570	-0.32915	0.005323
ZNF571	-0.3063	0.032736
ZNF572	-0.29665	0.013704
ZNF575	-0.87831	0.007468
ZNF585A	-0.24554	0.016108
ZNF585B	-0.32514	0.002199
ZNF587	-0.19752	0.022455
ZNF592	0.146873	0.016078
ZNF593	0.447482	0.027021
ZNF594	-0.25398	0.011684
ZNF596	-0.46864	0.009605
ZNF598	0.483051	0.008722
ZNF599	-0.41235	0.000141
ZNF605	-0.28158	0.000641
ZNF606	-0.43664	2.47E-06
ZNF608	-0.31541	0.00106
ZNF611	-0.33478	0.003036
ZNF615	-0.2922	0.004279
ZNF622	0.153835	0.018884
ZNF625-ZN	-0.4383	0.021788
ZNF629	-0.30066	0.000708
ZNF638	-0.19688	0.008033
ZNF641	-0.23322	0.009611
ZNF652	-0.24239	0.00773
ZNF658	-0.35588	0.000607
ZNF658B	-0.55988	0.002708
ZNF692	0.34973	0.001153
ZNF697	0.493713	1.01E-07
ZNF70	-0.2578	0.014099
ZNF704	-0.52232	9.48E-13
ZNF706	0.1874	0.001434
ZNF713	-0.30408	0.011355
ZNF720	-0.32397	0.004078
ZNF74	0.260261	0.014653
ZNF75D	-0.26289	0.00252
ZNF765	-0.27878	0.005647
ZNF767P	-0.21453	0.017739
ZNF772	-0.19228	0.005149
ZNF773	-0.24366	0.016068
ZNF774	-0.54649	2.16E-07
ZNF776	-0.18341	0.022998
ZNF778	0.192781	0.012114
ZNF780A	-0.36708	0.00322
ZNF780B	-0.35037	0.000609
ZNF789	-0.26126	0.006967
ZNF791	-0.2396	0.001624

ZNF81	-0.35586	0.000643
ZNF812P	-1.60881	9.63E-05
ZNF813	-0.22595	0.049038
ZNF814	-0.32415	0.000164
ZNF815P	-0.47054	0.036365
ZNF816	-0.24208	0.044762
ZNF836	-0.31534	0.001853
ZNF84	-0.27347	0.00337
ZNF844	-0.60192	0.015405
ZNF846	-0.73198	2.86E-08
ZNF862	-0.76423	6.43E-07
ZNF879	-0.5522	0.00287
ZNF891	-0.25418	0.032
ZNF91	-0.39037	1.21E-06
ZNHIT6	0.235514	0.018986
ZNRD1	0.469791	2.89E-08
ZNRD1ASP	-0.3931	0.000179
ZPR1	0.311996	1.04E-08
ZRANB2-AS	-1.61763	0.044329
ZSCAN16	-0.43466	0.040123
ZSCAN26	-0.22891	0.013763
ZSCAN30	-0.27589	8.66E-06
ZSCAN31	-0.7196	5.43E-07
ZW10	0.162156	0.01029
ZWILCH	0.264002	0.000595
ZWINT	0.322697	9.98E-06
ZYG11B	-0.28327	0.011301

and SCC25 cell lines.

Supplementary Table 3 - SCC1 differentially expressed genes between cetuximab and PBS.

Genes	log2FoldCh	pvalue
A2ML1	-2.12506	4.51E-05
A2MP1	-4.24349	9.14E-10
A4GALT	-1.24609	8.31E-10
AACS	-0.20151	0.00972
AACSP1	-1.21101	0.006581
AADAC	5.442025	3.17E-41
AADACL2-A	2.881813	0.005418
AADACP1	3.603728	1.80E-25
AAED1	0.21517	0.020749
AAK1	-0.16272	0.001678
AAR2	-0.16266	0.006384
AARSD1	0.131327	0.043541
AASDH	-0.36099	0.001448
AATBC	-1.465	0.001301
AATF	0.528368	3.37E-20
ABALON	0.940439	2.31E-09
ABAT	-1.34877	1.74E-25
ABC13-474	1.324514	4.61E-06
ABC7-4240	-2.70727	5.46E-11
ABCA12	-1.23276	6.16E-13
ABCA2	-1.09102	1.41E-07
ABCA5	-0.81377	5.64E-07
ABCA9-AS1	-2.32786	0.00013
ABCB1	2.761011	9.19E-06
ABCB10P1	-3.02095	2.82E-07
ABCB10P4	-2.01006	0.002424
ABCB5	-1.17067	0.024459
ABCB6	-1.87622	4.54E-22
ABCB7	0.196305	0.004206
ABCC1	-0.27228	0.00219
ABCC2	-1.07397	4.48E-18
ABCC3	-0.91582	2.19E-06
ABCC5	-0.32171	3.84E-06
ABCC6	-0.81898	0.001532
ABCC6P1	-1.52227	0.005583
ABCC6P2	-1.9744	0.000418
ABCE1	0.295166	5.88E-05
ABCF1	0.149891	0.001313
ABCF2	0.231821	3.49E-08
ABCF3	-0.19132	0.001681
ABCG2	-1.37667	1.02E-26
ABHD10	0.283932	0.00022
ABHD11	0.311646	0.012341
ABHD14A	-0.27631	0.013145

ABHD15	-0.63858	1.17E-12
ABHD16A	-0.25217	0.00017
ABHD17A	0.416972	0.01144
ABHD2	0.2223	1.20E-05
ABHD3	-0.1572	0.037444
ABHD4	-1.4326	1.56E-41
ABHD5	0.51463	6.80E-05
ABI3	2.886743	0.01792
ABI3BP	-0.77932	4.61E-09
ABL1	0.126056	0.043449
ABL2	1.008494	6.35E-14
ABLIM1	-0.30324	4.21E-08
ABLIM2	-2.55674	6.85E-14
ABLIM3	-1.11572	4.40E-18
ABR	0.788093	6.21E-17
ABT1	0.277673	0.000133
ABTB1	-0.49463	0.000427
ABTB2	0.269228	0.004019
AC000403.	-0.5935	0.012023
AC002116.	0.855374	0.026015
AC003973.	-2.96167	0.040597
AC004156.	-0.55946	0.037737
AC004381.	0.347865	0.001389
AC004471.	-1.42217	0.038437
AC004490.	-1.3103	0.043622
AC004951.	-0.96343	0.009985
AC004967.	0.449745	0.017364
AC005077.	0.929838	0.016118
AC005154.	-0.41003	4.43E-06
AC005262.	3.130281	0.009636
AC005336.	-1.21855	0.000224
AC005540.	2.220988	1.87E-05
AC005578.	-1.07604	0.033347
AC005592.	0.900915	0.005685
AC005618.	-0.80354	0.008671
AC005618.	-2.14784	0.000328
AC005775.	0.910839	0.047371
AC005944.	-0.49786	0.010233
AC006014.	-1.13069	0.000937
AC006116.	-3.00649	4.84E-08
AC006116.	-1.24937	0.011983
AC006262.	-1.64336	5.86E-06
AC006277.	-0.70058	0.017873
AC006946.	-0.9348	0.013162
AC006978.	-0.80778	0.000124
AC007038.	-0.84585	1.75E-07
AC007041.	2.391292	0.033702

AC007050.	2.701559	0.016205
AC007126.	-0.55667	0.021717
AC007163.	2.815384	0.008131
AC007192.	7.187853	0.016508
AC007193.	-3.20808	2.31E-10
AC007246.	0.237223	0.039706
AC007292.	-0.81211	0.038395
AC007326.	-0.78071	4.36E-09
AC007326.	-0.53148	0.003328
AC007750.	-0.65388	0.019616
AC008277.	-1.46295	0.003681
AC008694.	4.431606	0.000172
AC009005.	0.858981	0.000944
AC009120.	-0.86997	0.014514
AC009120.	-0.34347	0.004042
AC009133.	-0.35872	0.016839
AC009133.	6.389369	0.023996
AC009133.	-5.18489	0.002642
AC009229.	-3.49141	0.026966
AC009404.	-0.49486	0.026375
AC009495.	-0.68102	0.042017
AC009542.	-1.40528	0.042224
AC010127.	0.636943	0.02132
AC010642.	0.320549	0.022439
AC010761.	0.581381	0.000178
AC010761.	0.744116	0.007315
AC010907.	-4.5986	0.00413
AC011288.	-0.49185	0.047617
AC011330.	-0.64597	0.002367
AC012065.	-4.07566	0.003982
AC012146.	0.341089	0.035905
AC012360.	0.938801	7.73E-05
AC012531.	-0.72601	0.008151
AC012613.	-1.5416	1.23E-05
AC016582.	-1.1235	0.004312
AC016735.	-1.85086	0.020538
AC017002.	1.613021	0.012843
AC017006.	-1.74679	0.001001
AC018641.	-0.94802	0.048082
AC019186.	2.933032	0.023196
AC020571.	3.479718	0.005623
AC024560.	-0.87425	0.048641
AC024937.	-0.86181	0.006382
AC026150.	-1.37683	0.007151
AC026202.	1.246526	0.00871
AC034220.	-1.48545	3.25E-20
AC046143.	0.483836	0.006493

AC058791.	2.145166	3.32E-65
AC062028.	1.603154	0.043384
AC062029.	-0.61109	0.00543
AC068134.	-3.19772	0.033176
AC068279.	-0.76179	8.23E-05
AC068282.	-0.83549	0.000119
AC068533.	0.843269	0.007019
AC068580.	-0.86803	3.04E-07
AC068831.	-0.99239	0.003124
AC068831.	2.277969	3.90E-11
AC069257.	1.563865	0.001093
AC072062.	-0.63277	2.98E-05
AC073046.	-0.69908	0.000197
AC073072.	0.776637	0.006891
AC074117.	-0.28758	0.003744
AC074212.	-1.85806	1.05E-05
AC074212.	-1.90745	0.000171
AC079922.	0.966551	0.000271
AC083843.	-0.23722	0.033805
AC083843.	-0.99301	0.000135
AC087491.	-2.93855	0.047628
AC090616.	-1.16462	0.00753
AC091633.	-1.80746	0.043469
AC091729.	-0.31399	0.029039
AC091814.	-0.40713	0.043669
AC092597.	-1.46569	0.002953
AC092675.	3.027999	0.017333
AC093162.	-0.63732	0.000128
AC093609.	-2.92855	0.045676
AC093616.	1.088665	0.000372
AC093818.	0.533713	0.002123
AC093838.	0.261032	0.011961
AC097374.	-0.401	0.000267
AC097467.	-1.61917	0.018945
AC098614.	0.653327	0.002199
AC098617.	-0.99226	0.031417
AC098826.	2.346579	0.046058
AC107079.	-2.94611	0.021682
AC108142.	-0.33355	0.021809
AC108676.	0.206407	0.018078
AC112229.	-0.73522	0.003026
AC113189.	1.696585	7.62E-14
AC113404.	2.13167	0.048125
AC114776.	-0.96841	0.018622
AC114812.	-3.51992	0.012351
AC114812.	-1.77152	0.000269
AC115617.	-3.42037	0.004117

AC116366.	-1.51039	6.97E-11
AC124861.	-1.8415	0.003881
AC124997.	-3.06257	0.032316
AC125232.	-0.49237	3.22E-05
AC129778.	1.690332	0.000795
AC135048.	-0.91181	1.52E-05
AC137932.	0.394308	0.004683
AC137932.	2.582988	1.75E-06
AC139099.	-1.47173	0.003604
AC141586.	0.553535	4.69E-07
AC142472.	-0.88955	0.002361
AC144831.	-3.25997	2.11E-07
AC144831.	-4.20733	0.000398
AC145124.	-0.84873	0.000538
AC159540.	-0.51393	1.45E-08
AC226118.	-1.77376	0.00371
ACAA1	-0.24803	0.002446
ACAA2	0.215038	0.025127
ACACA	-0.34887	0.001193
ACAD10	-0.42651	2.35E-09
ACAD11	-0.32069	0.000821
ACADL	-1.21511	7.22E-11
ACADM	0.237317	0.002658
ACADSB	-0.4538	2.44E-09
ACADVL	0.218258	0.000898
ACAP2	0.17787	0.013433
ACAP3	-0.33885	0.012864
ACBD4	-0.38027	0.000105
ACBD5	-0.28035	7.85E-07
ACBD6	-0.15401	0.013656
ACCS	-1.2403	7.60E-18
ACD	0.680813	5.30E-17
ACE2	-4.39045	4.33E-19
ACER3	0.615284	4.92E-14
ACIN1	0.186728	0.00064
ACKR2	-0.63213	0.033358
ACKR3	-5.32846	2.55E-22
ACKR4	-0.67865	0.018875
ACO1	0.207051	0.005972
ACOD1	5.095827	3.06E-06
ACOT1	-0.713	3.12E-06
ACOT11	0.251966	0.028939
ACOT13	-0.27888	0.00077
ACOT7	0.784685	1.27E-18
ACOT9	0.309904	2.32E-05
ACOX1	-0.28662	0.001834
ACOX2	1.217395	0.034313

ACOXL	-0.4134	2.92E-05
ACOXL-AS1	-1.09668	0.001389
ACP1	0.261295	3.64E-05
ACP2	-0.23029	0.005127
ACP5	-0.23594	0.005893
ACP7	2.302647	5.25E-33
ACPP	-3.09509	2.81E-15
ACSF2	-0.54272	1.64E-09
ACSL1	-1.00522	4.07E-28
ACSL3	-0.4222	1.71E-06
ACSL4	0.789816	2.12E-13
ACSM3	-0.63975	0.004792
ACSS1	-1.24549	7.93E-30
ACTA2	1.206018	1.92E-15
ACTA2-AS1	0.902239	0.040272
ACTB	0.593843	3.08E-18
ACTBL2	-1.86116	7.78E-05
ACTC1	-2.64027	1.03E-06
ACTG1	0.248136	2.25E-05
ACTG1P20	0.949439	0.03187
ACTG2	-1.6657	0.000506
ACTL6A	0.381171	3.32E-06
ACTN1	0.658844	3.34E-18
ACTR1A	0.126052	0.020276
ACTR1B	-0.25953	0.003097
ACTR2	0.161323	0.004966
ACTR3	0.214645	0.001158
ACTR3B	-0.32005	0.000805
ACTR3C	-0.55158	7.44E-09
ACVR1C	0.513606	0.00714
ACVR2A	-0.27037	0.021392
ACY1	0.323378	0.000154
ACY3	-0.52741	0.012712
ADAD2	-2.38369	0.009516
ADAL	-0.39206	2.91E-05
ADAM10	-0.19701	5.94E-05
ADAM12	2.398696	3.18E-11
ADAM15	0.414912	6.98E-08
ADAM18	-3.07612	5.25E-06
ADAM19	2.885992	2.50E-53
ADAM20P1	-1.79123	0.006702
ADAM23	-1.00178	0.00146
ADAM28	-1.45412	6.37E-20
ADAM8	2.817453	7.72E-17
ADAMTS1	0.527338	2.08E-05
ADAMTS13	-1.4101	2.00E-09
ADAMTS15	-2.3883	2.98E-11

ADAMTS16	0.999761	0.000158
ADAMTS3	0.879584	2.66E-10
ADAMTS6	1.125519	1.76E-13
ADAMTS7	-1.26217	0.000262
ADAMTS7F	-1.10745	0.040205
ADAMTS9	-1.85278	0.007867
ADAMTSL4	-2.08058	1.53E-10
ADAP1	-0.76293	2.77E-06
ADAT1	0.136924	0.040496
ADAT2	-0.1735	0.022476
ADAT3	0.897789	0.015471
ADCK1	-0.42737	5.12E-05
ADCK2	0.248335	0.006663
ADCY1	1.084851	0.001083
ADCY3	0.47488	7.80E-14
ADCY6	-0.31412	0.003151
ADCY9	-0.48832	1.79E-05
ADD2	0.92245	3.55E-07
ADGRA2	-1.28228	4.40E-06
ADGRA3	-0.35755	7.12E-09
ADGRE2	0.847757	1.22E-05
ADGRE5	0.545323	1.36E-06
ADGRF3	-1.08162	1.57E-08
ADGRF4	-1.83178	9.48E-27
ADGRF5	0.729055	0.000742
ADGRF5P1	-1.00118	0.04394
ADGRG1	-0.73081	9.92E-32
ADGRG2	-0.66849	0.00572
ADGRG6	0.431641	5.12E-05
ADGRL1	-0.21394	0.030001
ADGRL2	-0.83122	1.79E-10
ADGRL3	-1.71626	1.98E-16
ADGRV1	-0.55674	5.43E-05
ADH1A	-2.24262	0.025441
ADH4	-1.0042	0.007448
ADH5	-0.12375	0.019327
ADHFE1	-0.5693	0.000841
ADI1	-0.18875	0.000329
ADIRF	1.385944	0.0001
ADIRF-AS1	0.932815	3.55E-06
ADM	-0.76347	3.30E-18
ADORA2A	-1.03297	0.012456
ADORA2A-/	-0.96925	0.000874
ADORA2B	1.583436	3.90E-71
ADPRH	-0.35088	0.003701
ADRA1B	-1.65675	0.000593
ADRB1	0.766918	5.34E-05

ADRM1	0.735237	1.25E-12
ADSL	0.241183	0.000147
ADSSL1	-1.46021	1.27E-12
ADTRP	1.252309	2.83E-15
AE000658.:	3.281287	0.048756
AEBP1	3.960193	4.35E-08
AEN	1.142587	6.46E-32
AF064858.:	-1.06492	0.024169
AF064858.:	-2.08237	0.02025
AF064858.(	-1.37319	0.019077
AF064858.:	-1.84941	0.012169
AF121898.:	1.843918	1.07E-12
AF165138.:	-2.22005	3.52E-07
AFAP1	0.265074	3.79E-07
AFAP1L2	-0.26427	3.53E-05
AFDN	0.223017	0.001186
AFTPH	-0.17483	0.009448
AGA	-0.28856	0.001176
AGAP2	0.412572	0.000554
AGAP2-AS1	0.434541	0.005789
AGAP4	-0.17983	0.0196
AGBL2	-0.45867	0.02172
AGBL4	-0.67394	0.013052
AGGF1	-0.19153	0.002876
AGGF1P1	-1.21085	0.021586
AGK	0.131555	0.00842
AGL	-0.18896	0.003004
AGMAT	0.958893	1.05E-15
AGO1	0.178648	0.004891
AGO2	0.522596	1.29E-11
AGO3	0.164063	0.0284
AGO4	-0.41585	1.95E-10
AGPAT1	0.159264	0.030232
AGPAT3	-0.21293	0.0146
AGPAT4	-0.68452	1.53E-09
AGPAT5	0.614954	4.68E-16
AGRN	-0.44303	0.021541
AHCTF1	0.31748	1.74E-05
AHCY	0.246736	2.11E-06
AHCYL2	-0.40057	0.002483
AHDC1	-0.4749	0.000782
AHI1	0.280721	0.029112
AHNAK	-0.39346	1.17E-07
AHNAK2	-1.34261	2.70E-08
AHR	-0.62751	4.31E-13
AHRR	-1.53838	1.76E-68
AHSA1	0.420365	3.99E-09

AHSA2	-0.25598	0.014477
AIDA	0.581794	8.25E-19
AIFM3	-0.69232	3.03E-06
AIG1	-0.21553	0.009287
AIM1	-0.54846	1.68E-18
AIM1L	-0.72444	2.37E-05
AIPL1	-1.58134	5.05E-07
AJAP1	2.459745	1.66E-15
AJUBA	-1.22173	5.97E-17
AK1	0.279582	0.001271
AK2	0.168147	0.002783
AK3	-0.36613	0.001915
AK3P5	1.201926	0.035521
AK6	0.506888	1.61E-08
AK7	-0.7929	0.017337
AKAP12	2.003456	7.53E-51
AKAP13	-0.562	1.36E-06
AKAP17A	0.503411	1.54E-08
AKAP2	0.493658	0.00302
AKAP3	-0.41203	0.047435
AKAP5	0.472411	0.000171
AKAP8L	0.15779	0.035602
AKIRIN1	0.171439	0.009171
AKIRIN2	0.207372	0.012891
AKNA	-0.23144	0.043657
AKR1A1	-0.20205	0.007447
AKR1B10	-4.20793	2.86E-56
AKR1B15	-1.92703	5.79E-14
AKR1C1	-1.31238	0.00011
AKR1C2	-3.93026	2.74E-14
AKR1C3	-2.21911	2.25E-13
AKR7A2	0.189583	0.030029
AKT1	0.320172	1.33E-07
AKT3	0.343262	0.000659
AKTIP	-0.42773	1.76E-08
AL022344.[illegible]	-1.0582	0.03774
AL590763.[illegible]	1.494806	0.007831
ALAD	-0.5615	2.74E-24
ALAS1	0.425209	4.22E-05
ALDH16A1	0.455604	0.00058
ALDH1A1	-1.72058	0.044599
ALDH1A2	-1.10523	0.010173
ALDH1A3	0.338711	0.014232
ALDH1B1	-0.32859	1.78E-05
ALDH1L2	-0.8747	2.20E-16
ALDH3A1	-1.63208	1.36E-34
ALDH3A2	-0.71574	8.11E-14

ALDH3B1	-1.8358	3.18E-32
ALDH3B2	-2.52121	2.49E-16
ALDH4A1	-0.78542	5.63E-21
ALDH5A1	-0.21266	0.001352
ALDH6A1	-0.48095	5.34E-12
ALDH7A1	-0.55107	9.42E-14
ALDH9A1	0.374166	1.15E-08
ALDOA	0.40243	5.71E-11
ALG1	0.239633	0.024144
ALG10	0.373215	7.76E-06
ALG13	0.198443	0.00894
ALG14	-0.19312	0.021745
ALG1L	-0.70776	0.000442
ALG1L9P	-0.76181	0.017214
ALG5	0.21932	0.007591
ALG8	0.173472	0.003405
ALKBH1	0.214382	0.003968
ALKBH5	0.165049	0.013022
ALKBH6	0.241217	0.003394
ALOX12	0.601222	0.000826
ALOX12-AS	-0.37175	0.020931
ALOX5AP	2.862456	6.00E-41
ALOXE3	1.539236	5.06E-18
ALPK1	-0.58403	3.24E-10
ALPK2	-0.55927	0.043317
ALPP	-4.77389	2.18E-17
ALPPL2	-5.95572	3.61E-11
ALS2CL	-0.59031	8.03E-07
ALX4	-3.00665	0.000561
ALYREF	0.673778	4.05E-16
AMACR	-0.19444	0.032101
AMDHD1	-0.38319	0.049537
AMDHD2	0.279442	0.014018
AMER1	-0.6504	1.27E-09
AMFR	-0.22119	0.006683
AMMECR1	-0.18319	0.004721
AMOTL1	0.60146	5.63E-14
AMOTL2	-0.43488	5.08E-06
AMPD2	0.365689	0.000261
AMY2B	-0.88292	0.020785
ANAPC1	0.204805	3.26E-05
ANAPC10	0.221521	0.009663
ANAPC5	-0.10343	0.04984
ANGPT2	1.134108	0.000556
ANGPTL4	3.451673	5.10E-22
ANK1	-0.73246	3.86E-05
ANK2	-0.52337	0.005623

ANK3	-1.46772	1.91E-12
ANKEF1	-0.44843	2.36E-05
ANKK1	-0.95023	0.000229
ANKLE1	1.066268	4.59E-10
ANKMY2	-0.28571	0.001248
ANKRA2	-0.33075	0.000176
ANKRD10	0.253041	0.024029
ANKRD11	0.420837	7.38E-06
ANKRD13C	-0.18696	0.007949
ANKRD16	-0.45744	0.002453
ANKRD18A	0.237058	0.030544
ANKRD18B	0.684725	1.52E-09
ANKRD2	-2.70892	1.42E-32
ANKRD20A	-1.25569	9.81E-05
ANKRD22	1.445306	4.12E-06
ANKRD28	0.141343	0.017482
ANKRD33B	0.352026	0.001607
ANKRD34B	-1.42536	0.002664
ANKRD35	-4.50346	0.000516
ANKRD36B	-0.42564	1.14E-06
ANKRD36B	-6.96895	0.001092
ANKRD37	0.409226	0.03158
ANKRD40	0.187301	0.005735
ANKRD49	-0.41797	1.90E-05
ANKRD50	0.315621	0.002992
ANKRD52	-0.59855	2.08E-12
ANKRD65	-0.82768	4.11E-20
ANKRD9	-0.54676	8.03E-05
ANKUB1	-0.94783	0.024161
ANKZF1	-0.26599	0.02803
ANLN	0.404221	7.93E-06
ANO1	-1.59026	0.024311
ANO6	-0.34096	2.47E-07
ANO7	1.436495	2.78E-11
ANO7L1	-1.0053	0.000305
ANO8	-0.53647	7.02E-06
ANO9	-0.76413	2.47E-07
ANP32A	0.203283	0.023315
ANP32B	0.338757	3.94E-05
ANP32E	0.24903	0.003568
ANPEP	2.660998	2.19E-35
ANTXR1	-0.23819	0.001819
ANTXR2	3.618769	1.24E-107
ANXA1	0.563664	2.26E-12
ANXA10	-2.03843	0.018533
ANXA2R	0.845667	5.52E-05
ANXA3	0.218975	0.015173

ANXA4	-0.4861	1.69E-25
ANXA5	0.663548	2.39E-11
ANXA6	1.233837	2.67E-13
ANXA8	-1.10595	2.33E-35
ANXA8L1	-1.01658	6.89E-39
ANXA9	-0.69825	1.39E-05
AOC1	-3.19375	0.013211
AOX1	1.688799	2.95E-17
AP000295.	2.171812	0.033049
AP000350.	-0.62638	0.005727
AP000569.	2.78084	0.027239
AP000662.	-0.86776	0.000519
AP000892.	-0.69953	3.89E-06
AP000962.	0.459181	0.00122
AP000997.	1.864366	0.046361
AP001258.	-0.19452	0.037999
AP001372.	-0.64648	1.06E-05
AP003068.	-0.40539	7.82E-05
AP005901.	2.001145	1.19E-07
AP006621.	-0.46271	0.011406
AP1AR	-0.18124	0.003276
AP1B1	0.32793	2.85E-06
AP1M1	0.234565	0.000932
AP1S2	0.436784	5.92E-05
AP2B1	0.204241	2.36E-05
AP2S1	0.484265	5.28E-10
AP3D1	0.18799	0.0125
AP3S1	0.231056	0.000376
AP3S2	-0.16605	0.044227
AP4B1	0.25925	0.000235
AP5B1	-0.5026	0.000124
AP5M1	-0.25695	0.001285
AP5S1	-0.19805	0.010515
APAF1	0.207712	0.012191
APBB2	-0.24724	0.019505
APBB3	-0.71443	4.95E-08
APC2	-0.50887	0.005777
APEH	0.271023	7.10E-05
APEX2	0.299615	1.38E-08
APH1B	-0.53613	3.62E-06
API5	0.120839	0.04779
APIP	0.276712	0.005208
APITD1-CO	1.690063	1.65E-05
APLN	2.784638	2.24E-19
APLP2	0.142065	0.017304
APMAP	-0.19069	0.000185
APOBEC3B	0.584389	0.002595

APOBEC3C	0.153705	0.04264
APOC1	0.366347	0.013488
APOD	-1.52687	5.43E-18
APOL1	-1.08893	8.76E-09
APOL2	-0.45699	1.98E-06
APOL3	-0.82717	5.98E-10
APOL4	-1.96442	7.86E-15
APOL6	-0.74525	1.89E-05
APOM	-0.58993	0.033809
APOO	0.496856	0.000295
APOOL	-0.30877	9.00E-05
APOPT1	0.346327	7.76E-06
APPBP2	-0.25696	0.000112
APPL1	0.168911	0.040442
AQP10	-2.24889	0.0015
AQP11	-1.02879	5.28E-10
AQP3	0.694291	0.013031
ARAP1-AS2	0.825029	0.033232
ARAP3	1.115863	4.83E-18
ARC	2.59824	7.84E-06
ARCN1	0.320526	7.07E-08
AREG	1.549583	5.18E-37
AREL1	0.158562	0.000313
ARF1	0.415715	2.58E-11
ARF3	0.178381	8.29E-05
ARF4	0.167937	0.014155
ARF6	0.275164	1.62E-06
ARFGAP3	0.250781	0.007111
ARFIP1	0.233744	0.005958
ARFRP1	0.209493	0.025163
ARG2	1.710132	7.23E-20
ARHGAP1	0.149553	0.010267
ARHGAP10	0.226706	0.00279
ARHGAP11	0.614886	4.91E-06
ARHGAP18	0.694414	2.74E-12
ARHGAP19	0.344579	3.95E-05
ARHGAP21	-0.24136	5.81E-05
ARHGAP22	-1.3399	1.01E-06
ARHGAP24	0.954315	2.64E-05
ARHGAP25	1.770058	5.49E-30
ARHGAP26	-1.35741	3.00E-17
ARHGAP27	0.377223	0.002531
ARHGAP27	-0.41058	0.002016
ARHGAP29	0.783089	1.22E-22
ARHGAP30	-0.67815	0.00182
ARHGAP31	1.887955	3.48E-52
ARHGAP35	-0.32545	3.72E-10

ARHGAP4	-1.15666	0.000122
ARHGAP44	-0.57606	0.020345
ARHGAP5	-0.25749	0.001128
ARHGAP9	1.366026	0.003505
ARHGDIA	0.582453	3.72E-08
ARHGDIB	1.085823	2.60E-28
ARHGEF1	0.533834	1.01E-07
ARHGEF10I	-0.45312	0.000484
ARHGEF16	0.499435	0.000147
ARHGEF17	-0.85553	1.70E-34
ARHGEF18	-0.2392	0.011761
ARHGEF2	1.327357	5.30E-22
ARHGEF25	-0.37939	0.003273
ARHGEF26-	-0.44953	0.020372
ARHGEF28	0.465716	5.02E-05
ARHGEF3	-0.18464	0.023166
ARHGEF37	-2.64522	3.40E-16
ARHGEF4	-0.89466	1.72E-12
ARHGEF40	0.905925	8.72E-13
ARHGEF5	0.162553	0.011587
ARHGEF9	-0.39696	1.14E-05
ARID2	-0.27232	0.000759
ARID3A	0.488644	0.000373
ARID3B	1.176109	5.13E-17
ARID4A	-0.34311	8.26E-07
ARID4B	-0.12735	0.030298
ARID5A	0.327144	0.010629
ARID5B	-1.86545	3.04E-28
ARIH2OS	-0.70972	0.009496
ARL11	-0.76616	6.23E-09
ARL15	-0.27024	0.033416
ARL2BP	0.528961	1.24E-12
ARL2-SNX1	2.987774	0.043961
ARL4A	-0.59582	5.14E-11
ARL4C	1.5046	3.28E-32
ARL4D	-1.12114	6.58E-17
ARL6IP5	-0.85193	6.37E-24
ARL6IP6	0.319352	2.02E-05
ARMC1	0.115547	0.04813
ARMC12	-1.06857	0.045575
ARMC4	1.103719	0.000496
ARMC5	0.336231	0.008913
ARMC6	0.238007	0.009806
ARMCX1	0.34922	0.000176
ARMCX2	-0.28473	0.002706
ARMCX3	-0.23865	0.005911
ARMCX6	-0.30617	1.69E-06

ARNT	-0.3657	5.38E-10
ARNTL2	0.977094	6.00E-13
ARNTL2-AS	0.987375	0.039698
ARPC1A	0.320835	8.58E-06
ARPC1B	0.581264	2.47E-09
ARPC2	0.201102	0.005694
ARPC3	0.284402	5.37E-07
ARPC3P1	1.185367	0.000211
ARPC4	0.284077	1.59E-06
ARPC4-TTL	-0.43857	0.01152
ARPC5L	0.550366	1.90E-13
ARPIN	-1.10281	1.97E-16
ARPP19	0.146967	0.039759
ARRB1	0.790871	0.000101
ARRB2	0.57092	9.57E-07
ARRDC3-AS	-0.75228	1.39E-07
ARSA	-0.56245	0.002652
ARSD	-0.87924	1.21E-13
ARSG	-0.39732	0.000123
ARSJ	0.409169	7.91E-05
ARSK	-0.25479	0.008835
ART4	-1.79786	0.003234
ARTN	-0.48983	6.37E-09
ARV1	-0.2453	0.035043
ARVCF	0.97431	1.98E-10
AS3MT	-0.47795	0.001636
ASAH1	-0.64716	1.77E-07
ASAH2	-0.26318	0.015072
ASAP1	0.346269	2.21E-10
ASAP2	0.462758	2.94E-12
ASAP3	-1.90509	2.42E-31
ASB1	0.502829	2.35E-05
ASB13	-0.24324	0.002893
ASB16	-0.50608	0.037346
ASB2	2.474537	0.049156
ASB3	0.17277	0.032521
ASB8	-0.14756	0.014542
ASCC2	0.178027	0.000878
ASF1B	0.552816	3.95E-10
ASGR1	1.753208	1.27E-08
ASH1L	-0.18034	8.32E-05
ASIC1	-0.2592	0.010532
ASL	0.531654	3.90E-11
ASMTL	-0.59521	1.59E-06
ASNSD1	0.328943	1.88E-05
ASPA	-0.29112	0.027476
ASRGL1	1.023478	1.71E-06

ASS1	-1.53922	6.69E-37
ASTE1	-0.30367	0.001067
ASXL1	0.135275	0.003796
ASXL2	0.114331	0.035334
ATAD2	0.723269	1.53E-14
ATAD2B	-0.17319	0.012965
ATAD3A	0.652022	6.36E-09
ATAD3B	0.675215	2.60E-09
ATAD5	0.641536	3.81E-09
ATF3	-0.83873	1.40E-05
ATF4	0.245183	0.000332
ATF6B	-0.36088	5.90E-09
ATF7	0.136944	0.016105
ATF7IP	-0.38273	9.03E-08
ATG101	0.344722	3.42E-05
ATG14	-0.39829	3.70E-10
ATG16L2	0.356078	0.009267
ATG3	0.157341	0.003665
ATG7	0.231297	0.002202
ATG9A	-0.4634	1.21E-08
ATIC	0.273001	4.05E-07
ATL1	-0.62659	4.11E-14
ATL3	-0.33911	3.12E-07
ATN1	0.508463	5.50E-08
ATOH8	-3.54329	1.18E-14
ATP10D	-0.48552	1.14E-07
ATP13A2	0.502717	1.85E-06
ATP13A3	0.447828	3.64E-05
ATP1A1-AS	-0.46836	0.033985
ATP1B3	0.41086	2.51E-06
ATP2B4	-1.14771	5.15E-14
ATP2C1	0.156276	0.004218
ATP5A1	0.107118	0.008466
ATP5B	0.107004	0.018353
ATP5D	0.45344	0.007721
ATP5F1	0.218917	1.83E-06
ATP5G1	0.149056	0.009695
ATP5G1P4	0.806353	0.03474
ATP5H	-0.14542	0.001932
ATP5J	0.220413	0.012135
ATP5J2	0.124989	0.007083
ATP5L	0.115479	0.03703
ATP5L2	-2.26666	0.029726
ATP5S	-0.24275	0.000639
ATP5SL	0.127295	0.024275
ATP6AP1	-0.29524	1.06E-06
ATP6AP2	-0.12204	0.019554

ATP6V0A4	0.33249	0.028694
ATP6V0B	0.24075	0.004186
ATP6V0C	0.431459	0.00613
ATP6V0E1	-0.16938	0.01885
ATP6V0E2-	1.222148	0.00534
ATP6V1B1	-3.7326	1.20E-08
ATP6V1B1-	2.361592	2.31E-05
ATP6V1B2	0.264922	4.54E-05
ATP6V1C1	0.165052	0.013693
ATP6V1D	0.634196	1.27E-19
ATP6V1E1	-0.16593	0.025873
ATP7B	-0.41648	0.001121
ATP8A1	-1.35141	2.13E-09
ATP8A2	2.83704	2.49E-12
ATP8B1	-0.19964	0.000547
ATP8B2	0.713289	6.57E-07
ATP8B4	-2.35623	7.21E-11
ATPAF1	0.148069	0.021604
ATPAF2	-0.25673	0.00596
ATRAID	-0.15856	0.000128
ATRIP	0.181668	0.036468
ATRN	-0.42133	7.66E-09
ATRNL1	0.951348	0.040387
ATXN1	-1.10443	4.54E-20
ATXN10	-0.18608	0.002232
ATXN1L	-0.18853	8.27E-06
ATXN2L	0.160537	0.021054
ATXN3	-0.25522	1.41E-08
ATXN7L1	-0.42862	4.64E-07
ATXN7L2	0.311126	0.00195
ATXN7L3B	-0.3462	4.07E-05
ATXN8OS	-1.45911	0.047022
AUH	-0.29112	0.005836
AUNIP	0.277404	0.004484
AUP1	0.215836	8.71E-05
AURKAIP1	0.47685	0.000292
AURKB	0.827604	2.93E-21
AVEN	0.305162	1.14E-06
AVPI1	-0.28025	0.01034
AXIN1	0.17778	0.015737
AXL	1.197768	1.50E-26
AZI2	-0.18167	0.023459
B2M	-0.4046	0.016354
B3GALNT1	0.307116	6.46E-05
B3GALT1	-0.4371	0.020835
B3GALT4	-1.32021	2.10E-15
B3GNT2	0.190634	0.041106

B3GNT3	1.970023	6.25E-29
B3GNT4	-0.84884	0.000179
B3GNT9	-0.36277	0.013799
B4GALT1	-0.44799	2.70E-07
B4GALT2	-0.17362	0.003117
B4GALT3	0.193159	0.014491
B4GALT4	-0.62751	5.45E-16
B4GALT6	1.398081	2.87E-20
B4GAT1	-0.28526	0.003693
BAALC	-1.22326	0.012843
BAALC-AS1	-0.31624	0.015175
BACE2	0.494882	1.90E-06
BACH2	-1.23296	3.15E-12
BAG2	0.402998	0.000835
BAG3	0.370655	6.19E-07
BAG5	0.112329	0.026057
BAG6	0.424507	4.21E-08
BAHD1	0.115785	0.047825
BAIAP2	-0.50986	2.97E-08
BAIAP2-AS:	-0.5787	6.68E-05
BAIAP2L1	0.316899	1.62E-06
BAIAP2L2	2.082191	4.10E-37
BAIAP3	0.542406	0.03566
BANF1	0.392095	8.37E-08
BANK1	-0.72474	0.012737
BANP	0.459075	0.014033
BARX2	0.81539	2.72E-13
BASP1	0.396006	3.56E-05
BATF	-3.63256	0.002841
BATF3	1.39999	5.32E-08
BAX	0.341089	2.57E-05
BAZ1A	0.357448	6.11E-06
BAZ1B	0.223138	0.000271
BAZ2B	-0.61881	6.11E-15
BBOF1	-0.49994	0.000309
BBOX1	-3.99568	4.39E-14
BBS1	-0.74555	4.93E-14
BBS12	-0.31809	0.005321
BBS2	-0.40803	1.13E-09
BBS4	-0.18192	0.002289
BBS5	-0.40391	0.000432
BBS9	-0.33525	8.82E-05
BBX	-0.45271	1.09E-19
BCAM	-1.62176	2.13E-24
BCAP29	0.433468	1.57E-07
BCAP31	0.302023	0.000128
BCAR1	1.159223	8.96E-14

BCAR3	1.02051	8.74E-15
BCAS2	0.329594	4.27E-05
BCAS3	-0.47871	1.50E-05
BCAS4	-0.46443	5.38E-07
BCCIP	0.47486	4.11E-10
BCDIN3D	-0.44061	0.001852
BCDIN3D-A	-0.47032	0.006855
BCHE	-0.49824	0.005657
BCKDHB	-0.42343	4.28E-08
BCKDK	0.50777	2.35E-07
BCL10	0.620862	2.76E-11
BCL2	0.769173	1.07E-07
BCL2A1	2.783523	0.038223
BCL2L1	0.715579	1.94E-09
BCL2L10	-1.04388	8.55E-05
BCL2L11	-0.33322	0.000817
BCL2L12	0.697169	8.87E-21
BCL2L13	-0.14168	0.041995
BCL2L2	-0.30665	8.12E-05
BCL2L2-PAI	0.45757	0.004883
BCL3	-0.45887	0.003231
BCL6	-0.99595	1.03E-14
BCL7A	-0.33616	0.001168
BCL7B	0.423692	8.08E-10
BCL7C	0.358031	7.01E-05
BCL9	-0.27073	0.023144
BCO1	-2.97792	0.016283
BCO2	-1.81157	3.34E-09
BCOR	-0.35717	2.20E-05
BCR	0.312905	0.000126
BCRP3	1.989543	0.044291
BCS1L	0.29297	0.000377
BDH2	-0.7516	6.30E-13
BDKRB1	-1.74161	5.34E-19
BDKRB2	-0.4565	2.78E-05
BDP1	0.169514	0.044338
BEGAIN	0.781922	0.043107
BEND3P1	-0.39935	0.000154
BEND3P3	-0.81286	0.006414
BEND5	-1.16921	0.020944
BEND6	-0.56598	5.47E-07
BEST1	-0.74092	7.03E-05
BEST2	-2.38223	2.60E-06
BET1L	-0.13256	0.036916
BEX3	-0.34634	5.50E-08
BEX5	0.461967	0.045113
BFSP1	1.121441	1.84E-12

BGN	-1.12759	0.003462
BHLHA15	1.596797	0.000199
BHLHB9	-0.39656	1.47E-08
BHLHE40	0.422266	0.001503
BHLHE41	-0.52884	1.57E-05
BICD1	0.42467	9.05E-06
BICDL1	-0.62284	4.87E-06
BICDL2	-0.64768	9.87E-05
BIK	0.672541	0.000161
BIRC5	0.434833	0.000816
BISPR	-0.54015	0.001318
BIVM-ERCC	-0.27626	5.08E-06
BLACAT1	1.774548	2.45E-12
BLM	0.997892	3.32E-20
BLNK	-2.61007	0.000688
BLVRA	-0.17688	0.003697
BLVRB	-0.38138	8.88E-06
BLZF1	0.214009	0.040829
BMF	-2.01755	1.08E-25
BMP1	-1.23661	2.08E-15
BMP2	-1.01946	1.51E-20
BMP2K	0.353904	2.86E-10
BMP3	1.645346	0.016047
BMPER	1.156776	2.59E-07
BMPR1A	-0.27073	7.36E-07
BMPR2	-0.44169	2.45E-13
BMS1	0.373634	1.88E-07
BMS1P10	0.678424	0.020119
BMS1P14	1.676141	0.04383
BMT2	-0.79641	6.62E-17
BNC1	0.212508	0.049834
BNC2	1.235884	0.000165
BNIP1	0.500437	2.59E-06
BNIP3	0.680635	5.74E-10
BNIP3L	-0.24184	0.011545
BNIP3P4	3.882544	4.27E-05
BNIPL	-1.56273	8.42E-06
BOLA2	0.480517	8.89E-08
BOLA2B	0.521084	3.16E-07
BOLA3	0.491823	8.61E-13
BOP1	0.754983	7.88E-14
BORCS6	-0.27468	0.026162
BORCS7	-0.59257	4.14E-16
BPHL	-0.6301	2.32E-21
BPIFA4P	-2.38858	0.022219
BRCA1	0.648678	5.31E-13
BRCA2	0.658183	1.22E-08

BRD2	0.140899	0.008294
BRD3	-0.17049	0.019112
BRD4	0.415377	3.54E-06
BRD7	0.203808	0.000706
BRD7P6	4.195284	0.001203
BRD8	-0.21494	0.01222
BRD9	0.381019	2.75E-08
BRF2	0.203759	0.01079
BRI3BP	0.245635	5.65E-07
BRIP1	0.669032	6.50E-06
BRIX1	0.758785	1.73E-20
BRMS1	0.147054	0.034811
BRMS1L	-0.28034	0.004204
BRPF1	0.33205	1.97E-06
BRPF3	0.261356	6.83E-05
BRSK1	1.226551	9.49E-06
BRWD1	-0.27147	5.87E-06
BSCL2	-0.65786	8.09E-07
BSDC1	-0.24222	0.000586
BSPRY	0.737441	1.32E-06
BTBD1	0.206832	0.000596
BTBD10	0.274399	0.00054
BTBD11	0.92923	0.000436
BTBD16	-1.41076	0.030484
BTBD19	1.569827	5.96E-26
BTBD3	-0.45382	0.000331
BTBD6	0.195034	0.000114
BTBD7	0.218246	0.000499
BTBD8	-0.78748	4.03E-05
BTBD9	-0.26063	0.019568
BTD	-0.49709	4.85E-07
BTF3	0.222142	0.000323
BTF3L4P2	0.345015	0.000561
BTG2	-1.41959	1.32E-22
BTG3	0.317024	0.001905
BTN2A1	0.161085	0.010631
BTN3A1	-0.87752	5.45E-15
BTN3A2	-0.64882	7.59E-10
BTN3A3	-0.92847	4.84E-16
BTNL8	-2.76346	0.044081
BTRC	-0.22722	0.00023
BUB1	0.465184	7.75E-05
BUB1B	0.553271	1.52E-05
BUB3	0.364817	2.82E-09
BUD13	0.294364	2.15E-05
BUD31	0.296939	5.44E-10
BX322557.	-0.44225	0.001117

BYSL	0.310959	6.33E-06
BZW1	0.892274	1.38E-32
BZW1P2	1.028125	9.96E-27
BZW2	0.178147	0.001357
C10orf35	0.571094	1.02E-08
C10orf55	0.948525	2.42E-07
C10orf67	-1.39982	9.78E-06
C10orf76	-0.12575	0.041566
C11orf24	0.228453	0.000713
C11orf45	-0.43885	7.35E-06
C11orf49	-0.29977	1.53E-06
C11orf52	-0.82002	1.08E-11
C11orf54	-0.50177	2.00E-08
C11orf68	0.322661	0.005798
C11orf70	-0.83153	1.36E-07
C11orf71	-0.53736	0.002253
C11orf84	0.464198	1.04E-08
C11orf91	3.442438	2.27E-06
C11orf95	0.316132	0.012351
C11orf98	0.326983	4.30E-06
C12orf29	0.214035	0.015984
C12orf4	0.291019	0.008113
C12orf43	0.217882	3.08E-05
C12orf75	0.888521	2.09E-12
C12orf76	-0.44147	4.69E-06
C14orf1	-0.30712	0.000374
C14orf159	-0.37705	1.44E-05
C14orf166	0.200355	0.001929
C14orf178	-1.90324	0.028376
C14orf2	0.150259	0.024696
C14orf28	-0.63889	5.59E-05
C14orf37	-0.78761	0.006508
C14orf79	-0.38919	0.00056
C14orf80	0.744813	2.34E-10
C14orf93	-0.32996	0.000387
C15orf38-A	0.215674	0.009484
C15orf39	0.340598	0.001331
C15orf40	-0.28771	0.003464
C15orf48	1.211123	4.43E-05
C15orf52	1.114089	3.70E-14
C15orf54	3.094838	6.61E-12
C15orf56	1.873936	0.001057
C15orf57	0.194313	0.007021
C15orf65	-0.8305	6.09E-05
C16orf52	0.838868	1.76E-22
C16orf58	-0.22085	0.009675
C16orf59	0.520711	0.000231

C16orf62	-0.35688	2.56E-12
C16orf74	1.20935	7.68E-30
C16orf87	-0.49289	1.95E-06
C17orf47	-2.56637	0.038211
C17orf49	0.303531	8.61E-05
C17orf51	1.196237	2.24E-22
C17orf53	0.568359	7.32E-08
C17orf80	0.158347	0.017901
C18orf21	0.20597	0.023441
C18orf54	0.998999	1.00E-23
C18orf8	0.372633	4.46E-05
C19orf24	0.360471	0.009978
C19orf33	0.563884	9.93E-06
C19orf43	0.37551	0.002776
C19orf47	0.481879	2.53E-06
C19orf48	0.41227	1.06E-15
C19orf53	0.371289	1.61E-06
C19orf66	-0.47553	0.000273
C19orf68	-0.4188	0.000699
C19orf71	-0.51026	0.000448
C1orf101	-1.33122	6.41E-08
C1orf106	0.830485	4.20E-15
C1orf112	0.496395	4.06E-10
C1orf115	0.434667	7.79E-05
C1orf116	-0.72598	7.37E-06
C1orf122	0.356761	0.021235
C1orf131	-0.35949	2.89E-05
C1orf174	0.272542	0.000321
C1orf186	-0.43127	0.014638
C1orf198	-0.19879	0.000613
C1orf204	0.508757	0.0418
C1orf220	-0.83827	0.00162
C1orf228	-1.14547	0.005422
C1orf35	0.303453	0.000404
C1orf53	-0.41079	0.046154
C1orf56	-0.26292	0.014301
C1QBP	0.443662	7.13E-14
C1QTNF3	-1.31919	0.000385
C1QTNF6	-0.56011	1.58E-06
C1R	-1.95888	1.16E-10
C1RL	-1.21552	1.66E-15
C1S	-1.77779	2.43E-12
C2	-0.52356	0.000327
C20orf197	1.815582	0.00426
C20orf24	0.477946	7.03E-09
C20orf27	0.372239	0.001573
C20orf96	-0.54465	0.000106

C21orf59	0.279634	2.05E-05
C21orf91	-0.55046	1.94E-07
C22orf23	-1.08363	7.06E-17
C22orf24	0.70739	0.027373
C22orf29	-0.26858	0.001108
C22orf46	-0.34748	0.000881
C2CD2	0.521074	4.13E-06
C2CD2L	0.737668	3.39E-13
C2orf16	-0.30731	0.014269
C2orf27A	-0.72308	4.13E-05
C2orf42	-0.21521	0.019679
C2orf48	0.567568	0.046832
C2orf49	-0.18841	0.013976
C2orf66	-1.40111	0.037766
C2orf68	-0.51301	5.20E-08
C2orf81	-0.54012	0.000326
C3	-0.86227	2.18E-06
C3orf33	-0.2572	0.009396
C3orf52	0.302205	4.93E-05
C3orf58	-0.24856	0.004219
C3orf62	-0.17826	0.02638
C3orf67	0.927328	9.17E-24
C3orf70	0.732848	0.012326
C4A	-0.79858	0.006181
C4B	-1.62227	0.0008
C4BPB	-1.42095	5.58E-10
C4orf19	1.691581	0.009497
C4orf22	1.28599	0.045446
C4orf3	-0.35658	2.14E-09
C4orf33	-0.37512	6.85E-05
C4orf46	0.40461	0.001476
C4orf47	-0.52137	0.036139
C4orf48	0.96778	0.035923
C5AR1	-0.87141	0.002357
C5AR2	1.02742	2.49E-08
C5orf15	-0.58078	3.09E-17
C5orf34	0.575755	6.82E-07
C5orf38	-1.16008	1.39E-10
C5orf42	-0.51175	2.69E-06
C5orf46	-3.56995	0.004442
C5orf51	0.13397	0.029979
C5orf56	-1.05156	3.71E-10
C5orf63	-0.60744	2.58E-05
C5orf64	-1.29967	0.047955
C6orf106	-0.70946	4.92E-22
C6orf120	-0.41347	2.63E-05
C6orf136	0.215028	0.021596

C6orf141	0.925003	6.10E-24
C6orf223	1.401259	0.029821
C6orf47	-0.23649	0.000305
C6orf48	-0.17214	0.001173
C6orf52	0.847484	0.030867
C6orf58	1.041061	0.004726
C6orf62	-0.19531	8.12E-05
C6orf89	-0.18217	0.002739
C7orf26	0.244878	0.007196
C7orf31	-0.4038	0.000288
C7orf49	-0.26217	7.75E-05
C7orf55-LU	0.318874	0.010102
C7orf57	1.749538	1.87E-07
C7orf73	-0.36259	0.000146
C8orf33	0.344986	1.69E-08
C8orf37	-0.3246	0.001411
C8orf4	-2.87928	0.000443
C8orf44	-0.60043	5.37E-06
C8orf46	-1.01256	0.00654
C8orf48	-0.46193	0.011532
C8orf88	0.794096	7.34E-06
C9orf116	-0.32918	0.013444
C9orf142	0.3618	0.000546
C9orf16	-0.38182	0.021804
C9orf3	-0.97585	1.91E-26
C9orf40	0.319596	0.017853
C9orf47	1.278837	0.017706
C9orf69	-0.31119	8.17E-05
C9orf78	0.262134	3.80E-07
C9orf84	-0.64019	0.000583
CA11	-0.64022	9.53E-08
CA12	-1.81555	6.32E-15
CA13	1.292954	7.92E-22
CA14	0.838068	0.002684
CA15P1	-3.69692	0.002574
CA5B	-0.24816	0.027556
CA5BP1	0.295352	0.005041
CAB39	0.687714	3.66E-26
CABLES1	-0.62692	7.33E-07
CABLES2	0.2629	0.016842
CACFD1	-0.97008	2.78E-11
CACNG4	-2.86155	1.50E-21
CACNG8	0.937173	0.000317
CACUL1	0.12679	0.037633
CACYBP	0.610827	1.73E-18
CAD	0.20264	0.00297
CADM1	1.55818	3.05E-40

CADPS2	-0.73958	4.73E-06
CALCOCO1	-0.70122	4.84E-22
CALCOCO2	-0.30469	0.00019
CALCRL	-0.77603	8.58E-08
CALD1	-0.82464	3.26E-21
CALM1	0.122988	0.010328
CALM2	0.596587	4.05E-19
CALM3	0.164083	0.002209
CALML6	-3.0592	0.022121
CALU	0.326305	8.14E-06
CAMK1D	-1.24351	8.92E-10
CAMK1G	2.240456	0.001242
CAMK2G	-0.58202	3.97E-14
CAMK2N1	1.077423	1.33E-15
CAMKK1	-0.28428	0.025959
CAMKK2	-0.32022	1.00E-05
CAMSAP1	0.262976	3.34E-05
CAMTA2	0.410354	0.000306
CAND1	0.156655	0.0135
CANT1	0.518165	3.66E-09
CAP1	0.53734	3.22E-14
CAPN1	-0.38293	2.59E-07
CAPN15	0.67368	7.27E-05
CAPN2	0.636919	1.20E-17
CAPN3	-1.53903	9.42E-08
CAPN5	0.916723	2.08E-10
CAPNS1	0.308382	4.75E-05
CAPNS2	-2.0517	0.001987
CAPRIN1	0.212086	7.00E-05
CAPRIN2	1.403662	4.80E-54
CAPS	-1.01742	1.04E-06
CAPS2	-0.52615	0.000735
CAPZA1	0.396147	1.44E-10
CAPZA2	0.143833	0.02145
CARD10	0.592929	1.84E-08
CARD11	0.592507	1.52E-06
CARD14	-0.97541	4.84E-19
CARD19	-0.42924	0.000116
CARD8-AS1	0.910347	1.05E-10
CARF	-0.45576	0.007049
CARHSP1	-0.25317	0.00228
CARM1	0.222194	0.016041
CARMIL1	-0.14119	0.016458
CARMIL2	1.265464	1.55E-07
CARS	0.115036	0.035668
CARS2	-0.1963	0.003423
CASC10	-0.92157	7.01E-05

CASC15	-0.87138	2.64E-05
CASC19	1.305259	2.75E-13
CASC21	1.891086	0.012728
CASC3	0.180213	0.000639
CASC4	-0.11857	0.022417
CASC9	-0.75016	7.29E-08
CASK	-0.26376	0.00421
CASKIN2	0.380594	0.003388
CASP10	-0.36185	0.000318
CASP14	-1.48143	0.006834
CASP1P2	-1.44937	0.001683
CASP3	0.437071	2.93E-10
CASP4	0.380384	0.003825
CASP6	-0.17775	0.045205
CASP7	-0.64984	3.70E-12
CASP8AP2	0.189389	0.00444
CASS4	0.764556	0.049034
CASZ1	-1.02089	1.98E-09
CAT	-0.44636	3.22E-12
CATSPERG	-0.77185	0.002106
CAV2	-0.44784	1.13E-05
CBARP	0.407654	0.045532
CBFA2T2	-0.40992	3.00E-07
CBFB	0.563563	2.00E-19
CBL	0.538716	4.26E-16
CBLB	-0.6367	6.94E-10
CBLC	-0.35931	0.015044
CBR1	-0.30849	0.004136
CBR3	0.806074	2.19E-06
CBR3-AS1	-0.49906	0.000136
CBR4	-0.32146	0.00298
CBWD3	0.377317	0.006044
CBX3	0.315372	6.62E-05
CBX4	-0.34526	0.00414
CBX5	0.629186	3.20E-13
CBX6	-0.34854	0.012009
CBX7	-0.44558	0.002463
CBY1	-0.27674	0.000297
CCAR1	0.200784	0.013334
CCAR2	0.138126	0.003489
CCAT1	0.990908	6.45E-16
CCDC102B	-0.46741	0.026331
CCDC113	-0.66034	1.75E-21
CCDC115	-0.47351	4.77E-19
CCDC120	0.750049	1.20E-11
CCDC121	-0.93881	9.27E-06
CCDC124	0.347072	0.016524

CCDC127	0.184296	0.003613
CCDC136	-0.37045	0.035583
CCDC137	0.160537	0.004826
CCDC138	0.340532	0.000208
CCDC144A	-0.38769	5.09E-05
CCDC144B	-0.5759	4.83E-08
CCDC144CI	-1.12882	5.36E-07
CCDC146	-1.02248	3.08E-05
CCDC149	-0.88119	0.012944
CCDC15	0.599147	1.05E-07
CCDC150	0.435397	0.003346
CCDC150P:	0.548508	0.034716
CCDC153	-0.90861	1.87E-05
CCDC158	-1.21312	0.000273
CCDC159	-0.69288	2.26E-05
CCDC162P	-0.4336	2.37E-06
CCDC167	-0.30401	0.016825
CCDC168	1.917999	0.014123
CCDC169	0.359797	0.00335
CCDC170	-1.47775	0.046134
CCDC171	-0.35707	0.035567
CCDC18	0.542212	0.001094
CCDC18-AS	-0.88265	5.13E-13
CCDC191	-0.62657	1.90E-10
CCDC22	0.264279	0.013562
CCDC24	-0.31342	0.001097
CCDC25	-0.15705	0.019429
CCDC28B	-0.3095	0.048287
CCDC30	-0.61227	0.001493
CCDC39	-0.45812	0.000802
CCDC40	-0.89468	0.001932
CCDC51	-0.34973	2.28E-05
CCDC54	-2.75206	0.012954
CCDC58	0.513363	7.34E-07
CCDC59	0.365017	1.78E-06
CCDC68	0.632153	7.83E-10
CCDC69	2.198547	4.62E-49
CCDC71L	0.944818	2.23E-11
CCDC77	0.20226	0.042306
CCDC80	-2.12977	5.16E-40
CCDC82	0.247531	0.007436
CCDC85B	2.102364	4.36E-08
CCDC85C	-0.41657	5.99E-07
CCDC86	0.63429	1.14E-15
CCDC87	-0.82793	3.20E-07
CCDC88A	0.424711	0.000112
CCDC88B	-0.6518	0.000137

CCDC89	-4.42421	0.000329
CCDC91	0.200565	0.00366
CCDC92	-0.65423	0.000826
CCDC93	0.269701	0.00015
CCDC96	0.42579	0.015129
CCDC97	-0.14023	0.038822
CCHCR1	0.221693	0.003208
CCL20	2.463316	3.08E-08
CCL28	-0.25229	0.005413
CCL5	-0.98464	0.009464
CCM2	0.457335	1.36E-10
CCNA2	0.297643	0.016722
CCNB1	0.367109	0.004378
CCNC	0.283896	0.000208
CCND2	1.178117	1.57E-20
CCND3	0.331441	0.000166
CCNE1	0.654622	1.39E-12
CCNE2	0.864946	2.23E-09
CCNF	0.407955	0.003135
CCNH	0.293274	2.17E-05
CCNI	0.212986	0.000271
CCNJ	0.348901	0.003265
CCNJL	0.509143	0.000219
CCNK	0.248789	0.00024
CCNL2	-0.24564	0.005073
CCNT1	0.13447	0.003675
CCPG1	-0.41238	1.02E-06
CCR1	-1.5499	0.036604
CCR10	0.790753	0.00232
CCR3	-2.1089	9.24E-09
CCRL2	2.161771	8.02E-09
CCS	-0.26302	7.83E-05
CCSAP	-0.40752	0.000694
CCSER1	-1.41037	4.27E-08
CCT2	0.457394	9.69E-17
CCT3	0.37153	1.15E-17
CCT4	0.473938	7.61E-15
CCT5	0.486606	6.80E-16
CCT6A	0.495976	1.23E-20
CCT6B	-0.97584	0.005522
CCT7	0.252454	2.92E-09
CCT8	0.493333	2.61E-16
CCT8P1	-1.21379	0.001258
CCZ1	0.186034	0.002079
CD109	-1.03731	6.92E-16
CD151	-0.50308	2.47E-07
CD160	-1.05356	0.001308

CD163L1	1.42032	1.19E-11
CD177	2.11182	2.70E-09
CD1D	-1.38351	4.80E-06
CD200	3.623132	9.81E-18
CD22	1.685261	0.005345
CD24	-2.44362	1.87E-12
CD274	2.256636	1.79E-33
CD276	-0.3449	0.000838
CD27-AS1	-0.75711	1.17E-07
CD302	0.290481	0.016773
CD320	0.877846	7.78E-16
CD34	1.25528	0.019632
CD36	-3.37474	1.92E-16
CD38	-1.93043	1.15E-20
CD3EAP	0.543429	7.84E-10
CD40	0.172511	0.049267
CD44	1.020603	6.14E-53
CD46	-0.35493	1.80E-06
CD55	-0.46912	0.00268
CD59	1.256885	2.01E-34
CD63	0.193378	0.036455
CD68	2.176137	7.76E-26
CD7	-2.28631	0.002667
CD81	-0.11825	0.013541
CD82	-0.46376	4.01E-06
CD83	1.103226	5.80E-19
CD86	-2.53203	0.0002
CD93	1.476159	0.037219
CD99	0.244008	0.002379
CD99L2	-0.88945	1.19E-17
CDA	0.424412	8.21E-06
CDADC1	-0.41174	7.71E-08
CDAN1	0.170479	0.016259
CDC123	0.256008	2.88E-05
CDC14B	-0.1588	0.038662
CDC16	0.156243	0.000848
CDC20	0.729278	4.76E-09
CDC23	-0.17931	0.014496
CDC25A	0.999405	1.97E-23
CDC25B	-0.43232	2.49E-06
CDC26	0.203362	0.003498
CDC27	0.250684	0.000331
CDC34	0.321922	0.000823
CDC37	0.469323	2.54E-08
CDC37L1	-0.40071	5.32E-05
CDC37P1	-2.38048	0.013172
CDC42BPG	-0.75064	7.83E-09

CDC42EP1	0.841088	6.41E-16
CDC42EP2	1.621737	7.61E-24
CDC42EP3	-0.30577	4.59E-07
CDC42EP5	1.444989	1.02E-07
CDC42SE2	-0.39069	1.91E-06
CDC45	0.818874	5.11E-21
CDC5L	0.252746	8.29E-05
CDC6	1.026573	2.63E-26
CDC73	0.202596	0.000823
CDCA3	0.422368	0.007519
CDCA4	0.59953	1.26E-13
CDCA4P1	-1.8286	0.042485
CDCA5	0.819709	3.64E-28
CDCA7	0.628607	0.001385
CDCA7L	0.597492	6.09E-13
CDCA8	0.437625	0.006802
CDCP1	1.134072	4.22E-27
CDH1	-0.96424	2.28E-18
CDH10	-2.32977	0.006126
CDH12P1	-0.96752	0.015443
CDH12P3	-0.96752	0.015443
CDH13	0.6356	9.50E-14
CDH16	-2.88184	0.001768
CDH18	-2.05799	5.77E-09
CDH24	0.179984	0.024059
CDH26	-1.00371	0.000954
CDH3	-0.19997	0.00277
CDH8	-0.40657	0.012162
CDHR2	-1.29111	0.000955
CDHR4	-2.38869	0.000456
CDIPT	-0.29222	0.001013
CDK1	0.596913	7.13E-09
CDK11A	0.267446	0.004616
CDK11B	0.354241	1.20E-06
CDK14	-0.59325	9.20E-07
CDK17	1.375081	2.71E-32
CDK18	-0.74749	5.59E-06
CDK19	-0.36026	2.07E-06
CDK2	0.54323	5.00E-22
CDK20	-0.54462	0.000538
CDK2AP1	0.238461	9.70E-07
CDK2AP2	0.210624	0.004469
CDK3	-0.49012	0.004484
CDK4	0.408436	3.54E-09
CDK5R1	0.484553	2.77E-12
CDK5RAP1	0.263229	6.66E-05
CDK5RAP3	-0.44378	1.42E-06

CDK6	0.273405	2.03E-06
CDK7	0.282635	0.000318
CDK8	0.186005	0.003114
CDKL5	-0.42595	0.000201
CDKN1A	0.756425	1.06E-05
CDKN1B	-0.22052	0.030233
CDKN1C	-2.65257	2.84E-09
CDKN2AIP	0.413109	1.70E-08
CDKN2AIPN	0.298834	1.05E-05
CDKN2B	-1.83627	2.29E-51
CDKN3	0.396704	0.04892
CDNF	-0.69475	0.016526
CDR2	0.608976	6.12E-13
CDR2L	-1.96503	3.28E-09
CDRT1	-1.84624	5.55E-26
CDRT15P1	-1.00651	0.015727
CDS1	-0.49726	7.52E-13
CDT1	0.928466	4.09E-12
CDV3	0.353172	3.99E-08
CDYL	-0.19748	0.023116
CDYL2	0.833485	7.23E-05
CEACAM1	1.286802	4.69E-05
CEACAM19	-0.88273	9.20E-10
CEACAM5	0.770198	0.006798
CEBPA	-3.20681	1.91E-24
CEBPA-AS1	-1.44715	0.007317
CEBPB	1.076475	1.07E-08
CEBPZ	0.362917	1.98E-06
CEBPZOS	-0.26981	1.35E-05
CECR7	-0.24981	0.035162
CELF1	0.19309	0.000239
CELSR1	-1.017	3.95E-21
CELSR2	-0.98196	8.43E-14
CELSR3	-0.62604	1.17E-07
CEMIP	0.340248	0.044485
CENPF	0.331553	0.021972
CENPH	0.475963	2.44E-07
CENPI	0.650901	6.61E-13
CENPJ	0.460718	0.000433
CENPK	0.698614	3.97E-10
CENPL	0.366257	0.002008
CENPM	0.564944	4.19E-08
CENPN	0.502094	2.46E-10
CENPO	0.745774	1.19E-14
CENPP	0.303874	0.007394
CENPQ	0.501325	1.24E-07
CENPS	0.171455	0.035645

CENPT	-0.38038	4.78E-05
CENPU	0.660006	1.62E-09
CENPV	0.6218	4.22E-09
CENPW	0.700051	9.53E-11
CENPX	0.360743	7.20E-08
CEP120	0.191751	0.002947
CEP126	-0.67071	7.92E-07
CEP128	0.799077	5.64E-18
CEP131	0.728148	9.60E-10
CEP135	0.589086	1.39E-14
CEP170	1.675783	4.03E-38
CEP170P1	1.90337	1.07E-11
CEP350	-0.14831	0.017176
CEP41	0.336453	4.15E-05
CEP55	0.69366	1.32E-09
CEP57L1	0.342527	2.96E-05
CEP63	-0.27029	0.000874
CEP68	-0.64999	0.000305
CEP70	-0.30276	0.000842
CEP72	0.569868	7.69E-08
CEP76	0.304733	0.000586
CEP78	0.520595	1.76E-14
CEP85	0.194287	0.003564
CERCAM	-0.93275	1.03E-18
CERK	0.51482	5.77E-05
CERS2	0.502679	7.39E-14
CERS3	-1.17754	9.12E-13
CERS4	-1.73804	4.09E-06
CERS5	0.157346	0.008125
CES2	-0.77073	2.48E-21
CES4A	-1.11018	7.46E-12
CFAP157	-0.31916	0.028537
CFAP20	0.413528	6.83E-08
CFAP206	-2.22699	4.00E-06
CFAP44	-0.41669	0.00311
CFAP45	1.617847	2.11E-12
CFAP47	-2.16322	0.00476
CFAP53	-0.68174	0.013941
CFAP70	-0.80283	4.07E-05
CFB	-1.14484	0.00611
CFH	-1.52523	0.002853
CFHR3	-0.91714	3.94E-06
CFI	-0.8434	0.014823
CFL1	0.384673	4.84E-10
CFLAR	-0.33739	0.006138
CGNL1	-0.88926	0.000154
CGREF1	-0.34914	0.032392

CH17-264L	-0.75129	3.02E-09
CH17-296N	0.578231	0.00561
CH17-302N	-1.70428	1.86E-05
CH17-335B	-0.85734	2.65E-08
CH17-340N	-0.57491	0.003349
CH17-360D	-1.71259	8.83E-12
CH17-360D	-0.31026	0.000837
CH17-360D	-0.47466	3.14E-09
CH17-472G	0.417404	2.65E-08
CH507-154	0.428484	0.001556
CH507-254	0.433403	0.020537
CH507-254	0.642337	0.000395
CH507-338	0.321056	0.03514
CH507-42P	-0.57165	0.0001
CH507-513	-0.24082	0.006872
CH507-528	-0.24157	0.006604
CH507-9B2	-0.28632	2.82E-05
CH507-9B2	0.258343	2.73E-05
CHAC2	0.578439	9.51E-07
CHAD	-0.64399	0.007053
CHAF1A	0.496764	5.92E-11
CHAF1B	0.663095	1.65E-13
CHAMP1	0.619606	6.42E-14
CHAT	-3.30569	1.01E-06
CHCHD2	0.25817	0.000547
CHCHD3	0.327809	5.26E-10
CHCHD4	0.437137	4.88E-06
CHD1	0.222354	0.010689
CHD1L	0.338778	2.57E-10
CHD2	0.267985	0.035443
CHD3	-0.4463	1.89E-20
CHD4	0.239967	5.10E-05
CHD6	-0.5327	2.74E-22
CHD7	0.232487	7.95E-05
CHDH	0.444337	0.001894
CHEK1	0.647825	2.70E-22
CHERP	0.575039	1.40E-09
CHGB	1.021491	0.003969
CHIC2	0.97538	1.86E-18
CHID1	0.183033	0.000759
CHKA	0.310835	0.002468
CHKB-CPT1	-0.63022	0.018964
CHML	0.66912	8.22E-08
CHMP2B	0.32614	7.54E-05
CHMP4B	0.203243	0.003299
CHMP4C	0.338588	1.03E-07
CHMP6	0.293066	0.006061

CHMP7	0.191945	0.020108
CHN1	0.856901	7.26E-05
CHORDC1	0.546861	1.15E-11
CHP1	-0.16602	0.001104
CHP2	1.274226	9.09E-08
CHRAC1	0.239326	0.001146
CHRNA5	0.547443	4.20E-10
CHRNB1	0.400849	4.49E-05
CHST10	0.555466	0.009737
CHST11	1.137979	1.38E-17
CHST14	0.235851	0.017117
CHST2	0.406435	0.001472
CHST3	0.571783	1.57E-13
CHST7	0.368789	0.009728
CHSY1	0.178901	0.009542
CHSY3	1.346283	7.25E-11
CHTF18	0.963557	3.45E-21
CHUK	0.32366	7.42E-05
CHURC1	-0.3444	3.92E-06
CIART	-0.67964	0.032719
CIB1	0.439803	3.37E-09
CICP14	-0.55265	1.90E-07
CICP16	-0.7041	0.021826
CICP27	-0.4648	5.30E-05
CIDEA	3.742851	2.39E-10
CIDECP	-0.34326	0.003027
CIR1	-0.21259	0.003069
CIRBP	-0.24128	3.92E-05
CISD1	0.171454	0.002817
CISH	0.468011	0.000927
CIT	0.464377	4.26E-07
CITED2	-0.6705	5.65E-08
CITED4	1.22279	4.05E-06
CITF22-49E	-2.60595	0.005205
CIZ1	0.209364	0.003627
CKAP2L	0.601354	2.09E-06
CKB	-0.54074	0.013158
CKMT1A	-0.49803	1.20E-09
CKMT1B	-0.71181	2.66E-14
CKS1B	0.643986	1.25E-09
CKS2	0.302861	0.021637
CLASP1	-0.57597	1.46E-10
CLASP2	0.144017	0.041717
CLASRP	0.443301	4.36E-06
CLCA2	-2.93626	2.45E-19
CLCF1	0.719288	2.08E-08
CLCN2	0.286171	0.002076

CLCN3	-0.43851	5.94E-11
CLCN4	-0.96641	4.57E-22
CLCNKA	-3.19897	0.008714
CLCNKB	-2.12528	0.00366
CLDN1	-1.27868	1.74E-06
CLDN12	0.47034	5.15E-08
CLDN16	-2.18981	1.38E-17
CLDN23	-0.46399	0.002887
CLDN4	0.338143	0.004781
CLDN9	-1.2439	3.85E-06
CLEC2B	-0.4778	0.007331
CLEC7A	-2.18992	1.83E-05
CLHC1	-0.36462	0.010607
CLIC1	0.482804	2.12E-08
CLIC2	-0.80895	0.029502
CLIC3	-2.92592	5.79E-44
CLIP1	-0.33015	0.007694
CLIP2	1.109679	1.71E-12
CLIP4	-0.35462	1.33E-09
CLMN	-0.73452	5.43E-12
CLMP	1.365252	1.63E-32
CLN3	-0.36774	1.91E-06
CLN6	0.383874	6.40E-05
CLPB	0.549385	4.98E-07
CLPP	0.515967	4.38E-05
CLPTM1L	0.26795	2.45E-05
CLSPN	1.035527	7.86E-30
CLSTN1	0.157058	0.004954
CLSTN3	-0.19325	0.007636
CLTA	0.307867	0.024737
CLTB	0.323556	0.00294
CLU	-0.99704	1.48E-23
CLUH	0.359	3.41E-06
CLUHP3	-0.25263	0.006096
CLYBL	-0.62416	0.001397
CMBL	0.301353	0.018189
CMC2	0.395259	1.68E-12
CMC4	0.225127	0.045068
CMIP	0.255963	0.000453
CMPK1	0.161049	0.009857
CMSS1	0.43894	3.63E-11
CMTM3	0.643874	2.97E-10
CMTM7	1.118668	9.94E-19
CMTR1	-0.20172	0.012652
CMYA5	-0.66874	0.000146
CNDP2	-0.42714	1.02E-07
CNGB3	-0.5356	0.001662

CNIH1	0.107183	0.040293
CNKSR3	-0.35086	0.000489
CNN3	0.757881	2.05E-21
CNNM2	-0.22931	0.006378
CNNM4	-0.17329	0.010398
CNOT10	0.295914	4.43E-08
CNOT11	0.161582	0.00884
CNOT3	0.245477	0.001758
CNOT4	0.268836	1.12E-05
CNOT6L	-0.25953	0.000628
CNOT7	0.238051	1.84E-06
CNOT9	0.219474	0.000503
CNRIP1	1.548363	1.02E-07
CNST	0.420941	2.02E-08
CNTLN	0.259866	0.021454
CNTN1	0.538198	0.00232
CNTNAP1	0.430886	0.000474
CNTNAP3	0.347586	0.000715
CNTNAP3B	0.232243	0.03397
CNTNAP3P	0.8098	0.003085
COA3	0.219225	0.007162
COA5	-0.58336	4.46E-10
COA6	0.206763	0.013507
COA7	0.146331	0.002398
COBLL1	-0.2981	0.002241
COCH	1.520023	4.70E-24
COG3	0.187601	0.008863
COG7	-0.28062	0.000335
COG8	-0.13959	0.04575
COIL	0.186652	0.004954
COL11A1	1.409241	0.025084
COL12A1	-1.39903	3.79E-28
COL16A1	-1.14419	1.93E-39
COL17A1	0.154908	0.028259
COL18A1	-1.2985	0.004965
COL1A1	-1.6958	2.07E-08
COL1A2	-2.00495	3.07E-10
COL21A1	-1.25828	0.017527
COL24A1	-1.15957	0.047719
COL27A1	-0.51114	7.80E-08
COL28A1	-0.52822	0.00774
COL2A1	-0.93585	0.003695
COL3A1	-3.78993	0.001318
COL4A2	0.239657	0.00263
COL4A3	-3.28287	1.51E-13
COL4A3BP	-0.37362	1.75E-10
COL4A4	-3.23309	9.31E-13

COL4A5	-1.44496	1.06E-23
COL4A6	-0.88111	1.56E-15
COL5A1	-2.21339	1.15E-09
COL5A2	-1.23167	1.71E-19
COL6A1	1.501173	5.30E-17
COL6A2	1.833865	9.51E-11
COL6A3	1.413313	9.50E-08
COL7A1	-1.74877	2.38E-11
COL8A1	0.576767	0.000342
COMMD2	-0.33562	3.17E-07
COMMD4	0.243533	8.98E-05
COMMD6	-0.24963	1.10E-05
COMMD9	-0.15643	0.024117
COPA	0.204835	0.000958
COPB1	0.281445	1.58E-05
COPG2	-0.39484	1.24E-10
COPRS	0.362778	7.24E-07
COPS3	0.446614	6.65E-13
COPS6	0.34482	1.22E-11
COPS8	0.280021	5.02E-07
COPZ2	-0.70927	7.56E-08
COQ4	-0.319	0.000267
COQ8B	-0.53124	1.10E-07
CORO1A	2.516968	3.21E-79
CORO1C	0.456079	1.62E-12
CORO2B	2.099742	8.12E-21
CORO6	-0.7499	9.92E-11
COTL1	0.907725	2.04E-17
COX10	0.149975	0.039231
COX14	-0.48809	6.16E-13
COX15	0.115423	0.04675
COX17	0.451717	2.08E-10
COX5A	0.25323	0.000235
COX6CP1	3.063842	0.030098
COX8A	0.218343	0.005036
CPA4	-2.99525	3.28E-09
CPA5	-2.17328	0.000421
CPE	-0.89795	6.82E-11
CPEB2	-0.34641	0.006891
CPEB3	-0.44765	0.00367
CPEB4	0.418054	1.76E-05
CPED1	2.139782	6.65E-21
CPNE2	0.847685	2.28E-15
CPNE5	3.164958	5.35E-13
CPNE8	0.525027	1.16E-06
CPOX	0.160573	0.009668
CPPED1	0.307062	5.96E-05

CPSF1	0.236986	0.000143
CPSF1P1	-1.14279	8.95E-05
CPSF2	0.144258	0.024885
CPSF3	0.145964	0.042601
CPSF4	0.118031	0.032839
CPSF6	0.198351	0.000629
CPT1A	-0.2833	0.000772
CPT1B	-0.51804	0.012122
CPT2	-0.38576	4.02E-05
CRACR2A	0.57421	9.46E-11
CRBN	-0.1718	0.003629
CRCP	0.289543	1.45E-08
CRCT1	4.747146	5.55E-05
CREB3	0.147636	0.037917
CREB3L2	0.300471	4.88E-06
CREB5	1.245767	1.04E-06
CREBRF	-0.2362	0.008576
CREG1	-0.9574	7.10E-15
CREG2	-2.82868	1.10E-26
CRELD1	-0.43895	0.000359
CREM	0.581335	1.00E-08
CRIM1	-0.34634	0.001587
CRIPAK	-0.46727	6.61E-05
CRISP3	-2.09092	0.000798
CRISPLD1	-1.04791	2.27E-06
CRK	0.09896	0.026483
CRKL	0.083731	0.010938
CRLF3	0.401018	1.64E-07
CRMP1	0.317543	0.000112
CROCCP3	-0.50776	0.015213
CROT	-0.73889	2.10E-14
CRTC2	0.12947	0.031893
CRYAB	-3.10624	5.94E-33
CS	0.191079	2.20E-06
CSAD	-0.74029	1.56E-15
CSAG2	-2.00407	0.002884
CSAG3	-2.46351	0.013989
CSE1L	0.609018	4.81E-17
CSF1	-0.93809	3.63E-10
CSF1R	-2.05589	1.22E-15
CSF2	4.415103	3.87E-17
CSF3	6.384485	1.12E-19
CSGALNAC	0.192546	0.018364
CSK	0.372766	1.82E-07
CSMD3	1.016986	0.001753
CSNK1A1	0.141237	0.004809
CSNK1E	0.704383	2.84E-21

CSNK2A1	0.280844	2.87E-07
CSNK2B	0.331914	6.96E-05
CSPG5	-0.74747	0.009955
CSRNP1	0.932666	4.61E-08
CSRNP3	-1.06262	6.91E-09
CST3	-0.7559	5.15E-11
CST6	0.907562	1.91E-05
CSTB	0.300936	0.00038
CSTF2	0.455346	1.39E-11
CSTF3	0.298534	0.00017
CT62	0.727662	0.000698
CTA-204B4	-0.42934	0.022867
CTA-212A2	-1.27255	0.000714
CTA-228A9	-0.81954	0.004201
CTA-243E7	-0.39673	0.039108
CTA-293F1	-1.97402	2.72E-11
CTA-29F11	0.634681	0.045386
CTA-363E1	-1.05415	0.008813
CTA-384D8	-3.36901	0.037311
CTA-398F1	-0.96068	0.025327
CTA-440B3	1.462391	0.043209
CTA-445C9	-0.58146	0.002856
CTA-989H1	-0.77236	0.035055
CTA-992D9	0.734702	0.021399
CTAGE5	-0.40851	1.56E-06
CTAGE7P	-0.52923	0.00912
CTB-113P1	-1.98879	1.49E-07
CTB-131B5	-0.61176	0.011221
CTB-131K1	0.306808	0.000161
CTB-133P2	1.757059	2.28E-11
CTB-13F3.1	0.542115	0.004725
CTB-147N1	-1.23283	1.84E-07
CTB-151G2	-1.09972	9.39E-10
CTB-167G5	-2.84077	0.025237
CTB-193M1	0.580415	7.20E-05
CTB-25B13	-0.57637	0.0001
CTB-50L17.	0.22762	0.018575
CTB-55O6.1	0.503072	0.003176
CTB-58E17	-0.84491	0.000674
CTB-5E10.3	-1.60486	0.030243
CTB-60B18	-1.25904	4.28E-06
CTB-60B18	-0.81778	0.005009
CTB-91J4.1	-1.02854	0.011814
CTBP1	0.295455	0.000814
CTBP2	0.394709	1.69E-09
CTC1	0.278529	0.00641
CTC-251D1	-1.07484	3.94E-05

CTC-260E6	-1.37528	0.008638
CTC-270D5	-1.72365	3.52E-05
CTC-339O9	-1.05076	0.015277
CTC-351M1	-0.54783	0.008065
CTC-360J11	-1.99153	0.023621
CTC-378H2	4.261671	0.000994
CTC-429P9	-0.87248	0.004601
CTC-429P9	-0.97666	0.02713
CTC-454I21	-3.40834	5.41E-05
CTC-463N1	0.479861	0.037949
CTC-487M2	0.69302	0.04222
CTC-487M2	0.428952	0.012409
CTC-490E2	-1.53256	0.003466
CTC-490G2	2.764588	0.001046
CTC-498J12	-2.64904	1.90E-05
CTC-499B1	0.788354	0.002491
CTC-505O3	-2.91838	0.045858
CTC-510F1	-0.76265	0.000242
CTC-518B2	-2.04067	0.03535
CTC-524C5	-0.46225	0.000263
CTC-525D6	-1.13879	0.018494
CTC-573M9	-1.60528	0.042669
CTC-573N1	-0.96863	0.023915
CTD-2003C	-1.70521	0.005663
CTD-2006C	2.749621	0.045444
CTD-2006C	-1.79978	0.025246
CTD-2006H	-0.4767	0.039555
CTD-2008P	-1.5613	2.77E-09
CTD-2013N	-0.79474	0.011731
CTD-2020K	0.613871	0.000607
CTD-2020K	-0.71044	0.021577
CTD-2024F	3.406961	0.004241
CTD-2026K	-0.64323	0.001566
CTD-2035E	1.495088	0.045929
CTD-2035E	1.475221	1.78E-05
CTD-2047H	-1.2522	0.000965
CTD-2058B	1.534875	3.63E-10
CTD-2066L	3.47752	0.006659
CTD-2066L	2.10336	0.009749
CTD-2066L	2.49549	4.92E-06
CTD-2083E	1.444678	0.036999
CTD-2095E	-1.36806	0.037355
CTD-2095E	-0.71856	0.040529
CTD-2104P	-1.58888	0.035206
CTD-2116N	0.584387	4.37E-09
CTD-2132N	-0.57507	0.003129
CTD-2162K	0.609875	0.033336

CTD-2165H	-0.98164	0.001749
CTD-2175A	2.188877	0.003027
CTD-2184D	-1.87451	0.000651
CTD-2196E	1.276225	0.049548
CTD-2201I:	-3.17195	0.012757
CTD-2207C	-1.94341	0.001065
CTD-2207P	1.97699	0.007542
CTD-2224J!	-1.44114	0.012575
CTD-2228K	-3.04264	0.018337
CTD-2228K	-0.84935	6.53E-10
CTD-2231E	1.183177	0.00043
CTD-2245F	3.219797	0.027649
CTD-2263F	2.655607	0.015772
CTD-2267D	-0.66508	2.00E-05
CTD-2269F	-1.28599	0.005798
CTD-2270L	0.405508	0.046681
CTD-2292M	-0.91553	8.05E-05
CTD-2303H	-0.6001	5.84E-06
CTD-2309H	-1.72093	0.003961
CTD-2323K	0.510309	0.03936
CTD-2330K	1.674673	0.006697
CTD-2341M	-0.86073	0.001847
CTD-2354A	2.014674	1.06E-20
CTD-2366F	2.736227	3.14E-09
CTD-2369P	1.392981	1.74E-05
CTD-2377C	-1.36116	0.01936
CTD-2382E	0.847346	0.001856
CTD-2383I2	0.770444	0.022762
CTD-2396E	-0.56976	2.24E-05
CTD-2510F	0.593161	4.57E-05
CTD-2514K	-2.76565	0.037243
CTD-2517C	-1.63612	2.72E-06
CTD-2525I3	1.606905	0.006356
CTD-2530H	-2.61326	0.036909
CTD-2530N	-0.86793	0.007637
CTD-2547L	-1.32687	0.001529
CTD-2547L	-1.3122	2.03E-07
CTD-2550C	1.58401	0.01303
CTD-2555C	0.652156	0.014304
CTD-2574D	-0.2787	0.048854
CTD-2574D	-2.099	0.004764
CTD-2587H	2.466128	3.44E-07
CTD-2589H	0.46986	0.029395
CTD-2589M	1.770092	0.041404
CTD-2619J:	-2.09525	0.008123
CTD-2619J:	-0.45415	0.001084
CTD-2619J:	-0.77815	0.002006

CTD-2619J1	-0.50848	0.010243
CTD-2619J1	-1.40001	0.024511
CTD-2623N	-1.20777	0.000698
CTD-2626G	-0.87062	1.00E-10
CTD-2647L	-0.38029	0.027347
CTD-2649C	-0.71672	0.006396
CTD-2650P	-0.80915	0.02851
CTD-3010D	-0.69378	0.029093
CTD-3023L	-3.86008	0.000981
CTD-3074C	-0.43621	0.028849
CTD-3088G	-2.28334	0.043413
CTD-3088G	-1.74978	2.04E-08
CTD-3094K	-1.6658	0.026778
CTD-3113P	0.225775	0.028193
CTD-3184A	-0.47356	0.037384
CTD-3203P	2.050835	0.00339
CTD-3222D	-4.12784	0.015196
CTDNEP1	0.321165	4.85E-05
CTDSP2	-0.2647	0.002466
CTDSPL	-0.60958	1.36E-09
CTGF	0.957112	3.82E-23
CTHRC1	0.981869	2.14E-12
CTIF	-0.54912	6.58E-06
CTNNAL1	1.694223	1.37E-34
CTNNB1	0.370097	2.26E-07
CTNNBIP1	-0.38142	1.28E-05
CTNNBL1	0.118424	0.032302
CTNS	0.252991	0.012471
CTPS1	0.685268	1.37E-39
CTPS2	0.16966	0.045457
CTR9	0.153282	0.010369
CTRB2	1.302505	0.007749
CTSA	-0.46482	1.61E-06
CTSB	0.218136	0.049468
CTSC	-0.71007	3.04E-06
CTSD	-1.00968	9.94E-13
CTSE	2.608732	0.000236
CTSF	-0.86878	1.65E-10
CTSH	-1.2579	9.44E-13
CTSL	0.653558	1.01E-09
CTSO	-1.19954	5.25E-05
CTSV	-0.67643	0.000461
CTSZ	0.158906	0.001941
CTTNBP2NI	0.39557	9.13E-07
CTU1	0.729593	0.003912
CUEDC2	-0.15844	0.038534
CUL1	0.129563	0.001271

CUL2	0.308404	7.06E-05
CUL5	-0.19007	0.003833
CUX1	-0.49312	2.62E-08
CWC22	0.293064	5.52E-06
CWC25	0.221083	0.001083
CWC27	0.220495	0.000217
CWF19L1	0.190946	0.001275
CWF19L2	0.194434	0.037833
CXADR	-0.63073	6.12E-21
CXCL1	3.361266	3.78E-15
CXCL2	2.365417	3.95E-11
CXCL3	1.403328	9.01E-08
CXCL8	2.754155	5.96E-11
CXCL9	-3.70996	2.09E-05
CXCR6	1.285083	1.04E-07
CXorf49	1.375568	0.007737
CXorf49B	1.3845	0.008369
CXorf57	0.413157	0.011735
CXXC5	-1.33561	3.38E-26
CYB561	-0.5235	2.48E-11
CYB561A3	-0.5638	4.93E-13
CYB561D1	-0.60852	2.04E-07
CYB5B	0.405975	1.14E-08
CYB5D2	-0.54345	1.07E-06
CYB5R1	-0.63735	7.25E-07
CYB5R2	-1.98768	3.58E-06
CYB5R3	0.28318	0.001194
CYBRD1	-1.40395	2.60E-32
CYCS	0.27745	8.60E-05
CYFIP1	0.206051	0.000183
CYGB	1.075986	7.38E-11
CYHR1	-0.44813	4.37E-06
CYP17A1	-3.29391	2.50E-08
CYP19A1	-0.78036	0.007515
CYP1A1	-3.94578	8.33E-36
CYP1A2	-0.87051	5.39E-05
CYP1B1	-2.3424	3.34E-34
CYP1B1-AS	1.049438	3.74E-16
CYP20A1	0.222865	0.003148
CYP27B1	2.309595	1.80E-35
CYP27C1	0.887421	1.37E-05
CYP2U1	-0.45318	4.61E-06
CYP2W1	-3.31598	8.73E-08
CYP39A1	-2.0739	7.33E-16
CYP3A7	-2.18761	0.003892
CYP4B1	-2.8996	3.11E-06
CYP4F11	-0.69168	0.000285

CYP4F26P	1.015721	0.041662
CYP4F3	-0.98044	0.028505
CYR61	0.400572	0.001383
CYSTM1	0.4885	3.46E-05
CYTH2	0.38206	2.49E-09
CYTH3	-0.64101	1.24E-19
CYTOR	1.143442	1.91E-23
CYYR1	0.62416	0.002342
D21S2088E	1.052086	3.20E-10
D2HGDH	-0.48609	0.000646
DAAM1	-0.42072	0.000731
DAB2IP	-1.61438	1.85E-27
DACH1	-0.83722	8.21E-06
DACT2	-2.08009	0.009352
DAG1	-1.35718	7.79E-53
DAGLB	-0.27749	0.003979
DAP	0.570317	1.57E-07
DAP3	0.347905	2.39E-09
DAPK1	-1.6415	8.50E-09
DAPK3	0.306948	0.00772
DAPP1	0.28471	0.022135
DARS	0.207863	0.000171
DARS2	0.229029	0.000306
DAW1	1.2678	1.00E-11
DAXX	0.277505	8.40E-09
DAZAP1	0.3674	1.53E-06
DAZAP2	-0.16628	0.001507
DBF4	0.512128	2.86E-06
DBF4B	0.516669	1.24E-05
DBN1	0.810649	1.17E-10
DBNDD1	-0.27034	0.019084
DBNDD2	0.554677	3.77E-08
DBNL	0.2978	0.002015
DBP	-0.80341	1.79E-06
DBR1	0.136745	0.019485
DBT	-0.50854	2.96E-10
DCAF1	0.104375	0.029492
DCAF10	-0.25009	0.010959
DCAF11	-0.33815	2.19E-13
DCAF12	-0.12554	0.023252
DCAF13	0.284293	1.23E-05
DCAF15	0.486391	1.04E-08
DCAF17	-0.23985	0.000279
DCAF4	-0.49023	3.81E-05
DCAF5	-0.46278	2.23E-11
DCAF6	-0.39668	4.14E-11
DCAF7	-0.096	0.005517

DCAF8	-0.35649	9.49E-06
DCAKD	-0.54356	5.73E-07
DCBLD1	-0.43915	0.000135
DCBLD2	1.504612	2.72E-32
DCK	0.522539	1.91E-09
DCLRE1A	0.249466	0.002178
DCLRE1B	0.544953	2.01E-08
DCLRE1C	0.185014	0.01452
DCN	-2.93201	1.97E-15
DCP1A	0.282752	0.000222
DCP2	-0.22607	0.005045
DCST2	-1.96925	2.43E-06
DCTD	0.357318	3.09E-10
DCTN1	0.203056	0.001103
DCTPP1	0.239942	0.000245
DCUN1D5	0.18979	0.007443
DDAH1	0.570777	1.26E-08
DDAH2	-0.27397	0.000549
DDB2	-0.18041	0.032383
DDHD2	-0.14764	0.016201
DDI2	-0.24041	1.62E-05
DDIAS	0.749848	2.44E-12
DDIT4L	2.303176	3.35E-17
DDN	0.667563	0.0007
DDR1	-1.0055	8.13E-24
DDR2	0.603254	0.025039
DDRGK1	0.319718	0.001381
DDT	0.245712	0.048103
DDX1	0.310837	1.32E-06
DDX10	0.159951	0.003601
DDX11	0.3982	0.001495
DDX12P	0.590958	0.000447
DDX18	0.377505	2.04E-08
DDX20	0.261841	2.95E-06
DDX21	0.439665	1.37E-07
DDX23	0.10047	0.013845
DDX24	0.275694	0.000143
DDX27	0.31142	1.08E-08
DDX28	0.162934	0.039931
DDX31	0.23562	0.000535
DDX39A	0.570768	2.48E-07
DDX39B	0.215849	2.09E-07
DDX41	0.187611	5.29E-05
DDX46	0.266835	0.000233
DDX49	-0.14256	0.047994
DDX50	0.291276	6.31E-05
DDX51	0.217795	0.012728

DDX52	0.290567	1.44E-05
DDX54	0.385195	3.45E-05
DDX55	0.279726	3.42E-07
DDX56	0.277986	3.40E-06
DECR1	-0.48495	3.69E-08
DECR2	-0.57026	0.000187
DEDD	0.153995	0.007184
DEDD2	0.705491	1.67E-09
DEF6	0.258052	0.005197
DEK	0.355465	0.001714
DENND1A	0.258456	1.43E-05
DENND1B	-0.42609	2.29E-07
DENND1C	-0.54798	1.20E-07
DENND2A	1.305575	1.17E-11
DENND2C	-0.6266	3.87E-05
DENND2D	-0.32857	0.001364
DENND4B	0.275344	0.000194
DENR	0.227832	2.15E-06
DEPDC1B	0.456414	0.000534
DEPDC7	-1.14322	7.79E-12
DERL1	0.124382	0.017675
DERL2	0.202657	0.007829
DESI1	0.303405	1.99E-06
DESI2	0.215522	0.00082
DFFA	0.120119	0.046825
DFNA5	-0.48026	1.34E-14
DFNB59	-1.34342	0.004292
DGCR10	-3.23204	0.025397
DGCR11	0.242677	0.026968
DGCR2	-0.69552	6.84E-12
DGCR6	-0.78317	4.07E-08
DGCR8	0.185945	0.027904
DGKA	0.743172	8.94E-24
DGKE	0.161259	0.014601
DGKI	0.929297	3.91E-08
DGKQ	-0.31917	0.008947
DGKZ	0.477149	2.53E-10
DGUOK	0.224407	0.00055
DHFR	0.508753	4.91E-05
DHFR2	-0.24714	0.001615
DHFRP1	0.944004	7.74E-18
DHODH	-0.17543	0.011017
DHPS	0.151911	0.035028
DHRS1	-0.44699	3.04E-08
DHRS12	-0.81917	1.84E-09
DHRS2	1.129812	1.17E-17
DHRS3	-1.06881	0.006715

DHRS4	-0.41069	0.002928
DHRS4-AS1	-0.29184	0.019707
DHRS4L2	-0.28034	0.024732
DHRS7	-0.16452	0.047899
DHRS7B	0.196077	0.019546
DHX15	0.223126	9.68E-05
DHX16	0.151861	0.011171
DHX29	0.139913	0.039612
DHX30	0.242237	0.000887
DHX32	-0.1927	0.006554
DHX33	0.247119	2.75E-07
DHX34	0.380723	4.13E-05
DHX35	0.175162	0.006718
DHX37	0.168364	0.014089
DHX38	0.156359	0.008537
DHX40	-0.22061	3.59E-05
DHX40P1	-0.44986	0.048201
DHX9	0.360729	3.70E-07
DIAPH1	0.446479	3.01E-16
DIAPH3	0.639212	4.50E-10
DIEXF	0.47133	6.04E-09
DIMT1	0.156652	0.048539
DIO2	-1.5375	4.01E-07
DIP2A	0.108485	0.033788
DIP2C	-0.86644	6.16E-11
DIS3	0.319537	8.53E-05
DIS3L	-0.37421	1.25E-06
DISC1	0.56885	0.005382
DISP1	-0.30649	0.034737
DISP2	1.184722	4.61E-16
DIXDC1	-1.04708	2.01E-16
DKC1	0.372529	3.11E-09
DLAT	0.174074	0.00893
DLC1	0.96686	3.21E-51
DLEU1_1	0.924464	0.03372
DLEU1_2	0.705382	0.005775
DLEU2	0.390596	0.02799
DLG1-AS1	-0.61024	0.013123
DLG4	1.768399	2.28E-19
DLG5	0.372142	3.97E-10
DLGAP1-AS	-0.50223	9.65E-06
DLGAP4	0.421728	1.97E-07
DLGAP5	0.403492	0.012359
DLK2	-1.23124	2.56E-15
DLL1	-1.30918	1.78E-15
DLL3	0.996741	6.61E-08
DLL4	3.822316	7.95E-25

DLST	0.514205	3.79E-18
DLX1	0.224929	0.038167
DLX2	-0.5035	0.002369
DLX3	-1.69497	3.11E-14
DLX4	-1.181	0.000341
DLX5	-1.74347	8.77E-10
DLX6-AS1	-0.99873	1.26E-15
DMD	-0.24082	0.012361
DMGDH	-1.01074	0.032533
DMKN	0.736109	3.69E-13
DMPK	-0.93673	1.05E-12
DMRTA1	-0.89434	3.18E-08
DMRTA2	0.617912	2.71E-05
DMRTC2	2.369842	0.000986
DMTF1	0.222637	0.000107
DMTN	0.871859	3.71E-06
DMXL1	-0.37214	2.16E-05
DNA2	0.367324	0.000114
DNAAF5	0.300302	0.000602
DNAH1	-0.9473	0.000127
DNAH11	-0.96938	3.31E-10
DNAH14	0.306366	0.000198
DNAH2	0.391317	0.000414
DNAJA1	0.404224	5.25E-10
DNAJA2	0.296956	7.84E-09
DNAJA3	0.242328	3.20E-05
DNAJA4	1.120462	6.25E-28
DNAJB1	0.208983	0.006052
DNAJB11	0.145552	0.048535
DNAJB2	-0.65603	2.63E-12
DNAJB3	-2.7274	0.0241
DNAJB4	-0.75439	4.60E-09
DNAJB6	0.171207	0.004133
DNAJB9	0.500666	0.00885
DNAJC10	0.395048	3.30E-06
DNAJC11	0.113229	0.031317
DNAJC12	-0.73077	0.037556
DNAJC15	-1.89994	1.46E-27
DNAJC16	-0.22338	0.004446
DNAJC18	-0.25172	0.010717
DNAJC2	0.544137	2.03E-13
DNAJC22	0.354276	0.013475
DNAJC27	-0.47149	0.000174
DNAJC3	0.251663	0.031776
DNAJC5	0.219874	0.005023
DNAJC6	-0.40277	0.036024
DNAJC7	0.345224	2.10E-09

DNAJC8	0.275402	0.00022
DNAJC9	0.635216	2.01E-27
DNAL4	-0.46721	4.77E-08
DNASE1L3	-1.61542	0.002002
DNASE2	-0.54969	1.12E-13
DNER	0.747488	1.79E-12
DNHD1	-0.32594	0.031438
DNLZ	0.270004	0.042913
DNM1	-0.52221	2.43E-11
DNM2	0.138557	0.021979
DNMBP	1.63494	1.72E-39
DNMT1	0.927962	2.36E-37
DNMT3B	-0.39821	0.002269
DNPEP	-0.38877	1.23E-08
DNTTIP1	0.261372	2.86E-05
DNTTIP2	0.409763	4.04E-06
DOC2A	-0.64013	0.000892
DOCK1	-0.1136	0.019409
DOCK11	0.366572	0.001565
DOCK2	1.89121	0.003598
DOCK4	3.443992	1.26E-21
DOCK5	0.324347	3.24E-13
DOCK9-AS2	-0.93311	0.013667
DOHH	0.462976	0.002609
DOK4	-0.5691	3.13E-12
DOK7	1.202245	4.19E-09
DOLPP1	0.201133	0.005112
DONSON	0.719753	2.30E-21
DOPEY2	-0.54636	0.000255
DPAGT1	0.14487	0.013218
DPF1	0.798162	5.71E-05
DPF2	0.137121	0.014912
DPH3	0.152501	0.040628
DPM2	0.174325	0.008082
DPP4	-0.60174	0.02205
DPP8	-0.30408	1.35E-06
DPP9	0.565978	4.39E-08
DPRXP4	-1.25807	0.034005
DPY19L1	0.371584	6.82E-07
DPY19L3	0.340316	9.11E-06
DPY30	0.226183	0.045769
DPYSL2	-0.32432	4.46E-06
DPYSL3	1.919552	1.03E-19
DQX1	-1.13508	2.91E-08
DR1	0.137046	0.017428
DRAM1	-1.05516	6.96E-13
DRAM2	-0.18958	0.004587

DRAP1	1.171952	7.23E-22
DRC1	-1.25936	0.041106
DRG1	0.28221	3.57E-08
DROSHA	0.217367	0.000306
DRP2	0.764319	0.025159
DSC1	-2.59058	0.013691
DSC2	-0.51893	1.12E-06
DSC3	-1.03222	1.88E-16
DSCAM	4.581467	3.06E-40
DSCC1	0.67531	3.00E-14
DSE	0.790389	6.85E-12
DSEL	-0.67974	6.28E-07
DSG2	0.283573	2.36E-05
DSG3	-2.09204	1.06E-15
DSN1	0.557285	5.73E-09
DSP	-0.56686	5.31E-08
DST	-0.98394	2.01E-39
DSTYK	-0.39142	3.53E-08
DTD1	0.27803	5.37E-05
DTL	0.947021	3.82E-23
DTWD2	-0.39248	0.001171
DTX2P1	0.588771	0.001856
DTX3	-0.22194	0.00307
DTX4	-1.28226	3.81E-37
DTYMK	0.605157	3.50E-12
DUBR	-0.83265	2.02E-10
DUOX1	-1.05631	8.99E-20
DUOX2	-1.52712	2.70E-18
DUOXA1	-0.92185	1.68E-17
DUOXA2	-1.43191	2.05E-06
DUS1L	0.161047	0.036409
DUS3L	0.706513	3.54E-08
DUSP10	-0.36694	0.016794
DUSP11	0.273924	3.73E-05
DUSP12	0.299745	2.69E-05
DUSP14	-0.20415	5.28E-05
DUSP16	-0.463	0.022247
DUSP19	-0.45353	0.02641
DUSP23	0.774562	0.000207
DUSP28	-0.50179	0.000355
DUSP3	-0.42881	9.45E-10
DUSP4	2.688468	3.39E-40
DUSP5	2.13026	7.69E-48
DUSP6	3.208729	2.67E-57
DUSP8	-1.2254	5.33E-05
DUSP9	-1.33275	1.53E-16
DUT	0.413079	9.67E-07

DUXAP10	-0.39016	0.00038
DUXAP8	-0.42279	1.32E-06
DUXAP9	-0.4605	1.10E-05
DVL1	0.659178	4.89E-06
DVL3	-0.18368	0.006962
DYNC1I1	0.848275	0.00372
DYNC1I2	0.190162	0.000119
DYNC1LI1	0.209092	0.000961
DYNC1LI2	-0.15006	0.024063
DYNC2LI1	-0.36723	2.81E-05
DYNLT1	-0.31195	2.01E-05
DYRK1A	-0.16272	0.001655
DYRK1B	-0.29567	0.03503
DYRK3	0.799925	1.09E-14
DYSF	2.251843	5.62E-13
DZANK1	-0.44186	0.00012
DZIP1	0.216548	0.003255
DZIP3	-0.28739	0.001486
E2F1	0.607748	3.57E-11
E2F2	-0.30471	0.038989
E2F3	0.417563	1.07E-05
E2F4	0.414073	2.74E-17
E2F5	-0.18336	0.02675
E2F7	0.901529	6.13E-45
E2F8	0.633726	4.87E-05
EAF1	0.377255	1.79E-06
EAPP	-0.28378	9.03E-06
EARS2	0.148507	0.005675
EBI3	1.47641	6.71E-09
EBLN3P	-0.18604	0.000294
EBNA1BP2	0.501731	7.87E-12
EBPL	-0.12803	0.023319
ECD	0.344544	2.27E-07
ECE1	0.125384	0.047113
ECEL1P1	-3.97914	0.003577
ECH1	-0.46964	2.96E-06
ECHDC1	0.137359	0.04862
ECHDC2	-0.84776	6.12E-16
ECHDC3	-1.32755	6.65E-12
ECM1	0.959704	3.78E-11
ECM2	-1.30502	0.004146
ECSCR	6.043036	5.23E-08
ECT2	0.498369	2.17E-07
EDAR	-1.82207	0.00405
EDARADD	-0.37528	0.000148
EDC3	-0.4177	1.87E-13
EDC4	0.283388	0.000383

EDEM1	0.322428	0.00529
EDEM2	-0.39586	1.72E-07
EDIL3	-2.62819	2.53E-34
EDN1	-0.46539	0.013164
EDN2	-5.88595	1.25E-06
EDNRA	1.032206	0.002519
EDNRB	-4.99805	2.55E-10
EED	0.36193	8.96E-07
EEF1A1P4	1.457075	0.001492
EEF1AKMT	0.40825	0.000115
EEF1B2	0.346308	2.17E-08
EEF1D	0.289489	9.45E-05
EEF1E1	0.530419	1.05E-11
EEF1E1-BL(	-4.07941	0.046409
EEF2	0.155959	0.04086
EEF2KMT	0.310869	0.003963
EEFSEC	-0.29142	0.018697
EFCAB1	-0.59535	0.027522
EFCAB12	-0.68372	0.00434
EFCAB7	-0.25992	0.009801
EFEMP1	-0.41443	1.97E-05
EFHC1	-0.40214	0.000142
EFHC2	-2.01099	0.041774
EFHD2	0.889464	3.35E-18
EFNA1	-0.61188	3.34E-09
EFNA3	-0.5077	0.012724
EFNA4	-0.30873	0.000522
EFNA5	-1.2604	1.65E-15
EFNB1	0.959095	1.67E-15
EFNB2	0.981299	1.21E-15
EFR3A	-0.12209	0.038419
EFR3B	0.775404	0.037514
EFS	-2.24292	1.93E-16
EFTUD2	0.315737	1.13E-10
EGF	-1.64816	2.29E-06
EGFL7	1.635019	7.33E-05
EGFR	-0.56328	1.92E-11
EGFR-AS1	-0.4299	0.019796
EGLN1	0.125637	0.032935
EGLN3	-1.03915	3.09E-12
EGR1	3.664812	2.50E-25
EGR3	2.22327	2.64E-05
EGR4	2.680573	0.006443
EHBP1L1	0.360611	0.000187
EHD1	0.830734	2.43E-09
EHD2	-0.27598	0.010839
EHD4	0.809687	1.81E-24

EHF	1.405331	2.10E-13
EHHADH	-0.52744	7.09E-16
EI24	0.115756	0.004877
EID2	-0.22966	0.045137
EIF1	0.162929	0.007878
EIF1AD	0.382007	3.36E-07
EIF1AX	0.236083	0.00047
EIF1B	0.135763	0.040775
EIF1B-AS1	-0.8587	0.012565
EIF2A	0.358258	2.17E-06
EIF2B1	0.116587	0.009147
EIF2B2	0.563488	4.94E-16
EIF2B3	0.417754	2.44E-05
EIF2B4	0.180758	0.002449
EIF2B5	0.167979	0.01941
EIF2S1	0.263908	5.56E-06
EIF2S2	0.336754	1.34E-06
EIF2S3	0.229326	0.000342
EIF3A	0.241746	7.12E-05
EIF3B	0.225736	2.61E-07
EIF3C	0.364789	7.94E-13
EIF3CL	0.87926	0.021449
EIF3D	0.396626	1.48E-11
EIF3EP1	-0.68568	0.004234
EIF3G	0.300934	0.000209
EIF3H	0.126011	0.00768
EIF3I	0.290186	1.23E-07
EIF3J	0.264907	1.73E-05
EIF3J-AS1	-0.45622	0.002869
EIF3K	0.205997	0.000862
EIF3M	0.21491	0.00019
EIF4A1	0.401571	1.42E-15
EIF4A2	-0.26725	3.69E-05
EIF4E	0.240936	5.52E-05
EIF4E2	-0.2674	2.19E-07
EIF4E3	-0.5072	8.52E-05
EIF4EBP1	0.705408	3.36E-16
EIF4G1	0.356511	3.63E-09
EIF4G2	0.126732	0.002384
EIF4H	0.251362	1.24E-07
EIF5	0.379198	2.77E-06
EIF5A	0.570557	1.34E-19
EIF5A2	0.525371	2.11E-10
EIF5B	0.285705	3.35E-06
EIF6	0.330473	1.11E-05
ELAC1	-0.26385	0.040482
ELAC2	0.242463	0.000336

ELAVL1	0.293407	6.81E-06
ELAVL2	0.486057	0.00656
ELF1	-0.37626	0.000184
ELF2	-0.25589	0.000103
ELF3-AS1	-0.65902	0.038712
ELF4	-0.29894	1.24E-07
ELFN2	1.670884	0.000432
ELK1	0.345259	2.94E-06
ELK3	1.540143	1.25E-45
ELK4	0.115272	0.039656
ELL	0.659596	7.14E-08
ELL2	1.191644	4.02E-57
ELMO1	0.313101	0.022941
ELMO2	-0.13777	0.017436
ELMO3	-0.35376	4.80E-05
ELMOD2	-0.24183	0.001468
ELMOD3	-0.25866	0.003772
ELMSAN1	-0.1158	0.031916
ELN	-1.02727	0.043725
ELOA	0.326667	4.97E-10
ELOC	0.262737	0.00163
ELOF1	0.197529	0.014144
ELOVL1	0.50897	4.32E-07
ELOVL2	0.931475	0.012516
ELOVL3	-1.19649	0.000213
ELOVL4	-0.19418	0.029975
ELOVL6	0.337302	0.000544
ELOVL7	0.279942	0.008522
ELP5	0.266536	0.012296
ELP6	0.166296	0.001535
EMC2	-0.16206	0.014457
EMC3-AS1	0.562556	5.17E-09
EMC6	0.394513	0.002623
EMC8	0.412729	9.90E-08
EMC9	0.252171	0.047218
EME1	0.58868	3.80E-06
EMG1	0.45592	3.25E-15
EMID1	-0.91715	1.59E-06
EMILIN2	0.784011	2.80E-10
EML2	0.232133	0.040956
EML2-AS1	2.149372	0.01047
EML4	0.27699	0.000152
EML6	-0.34269	0.022865
EMP1	-0.95741	0.000414
EMP2	-0.25867	0.037424
EMP3	1.44369	3.74E-15
ENAH	-0.97728	8.43E-27

ENC1	1.119431	5.68E-24
ENDOD1	-0.61388	7.70E-14
ENDOV	-0.33434	0.001614
ENG	1.350405	2.07E-17
ENGASE	-0.31279	0.010067
ENKD1	0.479953	4.54E-06
ENKUR	0.684081	0.034153
ENO1	0.380835	2.24E-13
ENO1P1	-5.4703	0.046655
ENO2	1.219431	6.63E-13
ENO3	0.40002	0.01271
ENOPH1	0.399134	4.66E-13
ENOSF1	-0.1782	0.005175
ENPEP	-0.55781	0.025405
ENSA	0.144954	0.01116
ENTPD2	1.596788	1.40E-23
ENTPD3-AS	-0.48388	0.000817
ENTPD4	-0.17922	0.035282
ENTPD6	0.378121	3.58E-07
ENTPD7	0.433548	0.00057
EOMES	1.045665	8.91E-06
EP400	0.266294	0.000292
EPAS1	-0.55221	8.65E-08
EPB41L1	-0.61431	2.36E-15
EPB41L2	0.646285	7.83E-30
EPB41L4B	-0.8275	9.74E-10
EPCAM	0.747224	8.53E-11
EPG5	0.596687	4.68E-06
EPHA1	0.298519	0.004605
EPHA10	-1.16872	0.007183
EPHA1-AS1	1.237989	0.000144
EPHA2	1.715518	2.77E-12
EPHA3	-1.54673	0.000112
EPHA4	-1.79663	0.004081
EPHB2	0.86289	3.42E-22
EPHB3	-1.5341	6.72E-14
EPHB6	-1.74923	0.017124
EPHX1	-0.97869	1.33E-22
EPHX2	-1.02449	7.56E-11
EPHX4	0.7903	1.13E-07
EPM2A	-0.62953	8.64E-08
EPM2AIP1	-0.38375	0.001433
EPN1	0.337745	0.004986
EPN3	-1.12485	2.71E-22
EPO	-1.90296	0.01825
EPOP	0.986147	3.78E-12
EPPK1	-1.34634	3.78E-05

EPRS	0.226184	0.001527
EPS8	-0.84622	4.80E-19
EPS8L2	-0.79257	2.15E-20
EPSTI1	-0.85165	0.016188
ERAP1	-0.27302	0.007578
ERBB2	-0.50038	1.74E-10
ERBB3	-0.86831	4.74E-35
ERBB4	-3.1863	1.54E-07
ERC1	-0.19181	2.14E-06
ERCC1	0.622152	1.36E-08
ERCC2	0.159088	0.03697
ERCC3	0.263849	1.02E-06
ERCC4	-0.18113	0.021994
ERCC5	-0.2643	0.009741
ERCC6	-0.23237	1.41E-05
ERCC6L	0.849752	6.37E-14
ERCC6L2	0.238804	0.002266
EREG	1.33879	4.40E-16
ERFE	1.113998	0.009176
ERG	1.34427	1.93E-10
ERGIC1	-0.35465	5.00E-07
ERGIC2	0.254179	1.35E-05
ERGIC3	-0.21911	3.15E-06
ERH	0.300236	0.000395
ERI1	0.551656	7.97E-10
ERI3	-0.18066	0.000787
ERI3-IT1	-1.06621	0.005049
ERICH5	-0.45689	0.005708
ERLIN1	0.339485	1.92E-05
ERMAP	-0.37181	0.013375
ERMARD	-0.20613	0.007136
ERN1	0.806847	6.96E-11
ERO1A	0.369385	5.21E-05
ERO1B	0.514826	0.001684
ERP27	-1.10242	0.012228
ERRFI1	0.527495	1.82E-07
ERV3-1	-0.53738	2.00E-08
ERVH-1	1.768589	9.51E-19
ERVK13-1	-0.27453	0.003307
ERVK-28	-1.22722	2.05E-08
ERVK3-1	0.128944	0.0298
ERVMER34	0.424429	0.000486
ERVV-2	-1.12818	0.046334
ERVW-1	-0.45328	0.001107
ESAM	1.17758	4.23E-05
ESCO2	0.756891	1.86E-11
ESD	0.241803	4.76E-06

ESF1	0.313648	7.74E-05
ESM1	11.31345	1.56E-25
ESPL1	0.412332	0.000202
ESPN	-1.61209	4.03E-05
ESPNP	-1.53402	0.012384
ESR1	-1.6339	1.25E-26
ESRG	1.021812	1.13E-16
ESRP1	-0.24041	2.56E-06
ESYT1	0.3796	2.28E-05
ESYT2	-0.24957	8.29E-09
ETAA1	-0.39104	2.83E-05
ETF1	0.293207	2.87E-05
ETFB	-0.17528	0.002334
ETFBKMT	-0.95743	3.52E-06
ETFRF1	-0.6037	1.22E-10
ETHE1	-0.37047	2.21E-05
ETNK2	0.342137	0.001075
ETS1	1.459611	5.97E-40
ETS2	-1.14593	3.60E-15
ETV3	0.25438	6.42E-05
ETV4	0.47272	7.77E-05
ETV7	-0.58556	0.00613
EVA1A	0.36083	0.021221
EVA1B	0.750109	0.002286
EVC	0.446917	8.60E-06
EVC2	0.443793	6.77E-05
EVI2B	2.536429	0.000191
EVI5L	-0.58517	5.19E-06
EVL	-0.38473	0.003126
EVPLL	-1.4888	2.76E-16
EXO1	0.844058	1.06E-18
EXO5	0.328842	6.54E-05
EXOC1	-0.16824	0.019868
EXOC3-AS1	-0.4567	0.012722
EXOC3L1	-0.76766	0.02039
EXOC4	-0.2113	0.000257
EXOC6	0.316122	0.003929
EXOC6B	-0.67397	1.18E-31
EXOSC1	0.162029	0.013051
EXOSC10	0.476594	6.13E-18
EXOSC2	0.317355	2.44E-08
EXOSC3	0.505605	5.85E-11
EXOSC4	0.378583	0.015936
EXOSC8	0.530618	1.78E-08
EXOSC9	0.490001	1.00E-08
EXPH5	-0.32276	0.003064
EXT1	-0.18435	0.018394

EXTL3	0.135636	0.038767
EXTL3-AS1	-0.7145	0.019281
EYA3	0.329748	1.53E-05
EZH2	0.624694	8.22E-09
F10-AS1	-2.50221	0.047747
F11R	-0.32304	2.62E-09
F12	0.800234	8.95E-05
F2RL1	0.909097	5.81E-22
F2RL2	-1.41491	0.000879
F8	-0.57195	0.000104
F8A1	0.231679	0.008335
FA2H	0.978032	0.002761
FAAH	-1.09834	1.74E-09
FAAH2	-0.33105	0.027499
FAAP20	0.464468	0.00392
FABP2	-1.10099	0.012514
FABP4	-2.61047	0.039288
FABP5	-0.38623	0.036974
FADS3	-0.9858	1.02E-17
FAF1	0.335782	7.62E-11
FAHD2B	-0.32894	0.000292
FAHD2CP	-0.72069	0.00223
FAIM	-0.44873	4.47E-09
FAM102A	-0.32312	1.11E-05
FAM103A2	0.450171	0.018204
FAM104A	0.200655	0.0024
FAM104B	0.363457	0.000333
FAM105A	1.048159	5.35E-10
FAM106A	-0.49677	0.004894
FAM107B	0.733609	5.11E-19
FAM109A	0.248324	0.008775
FAM110C	2.183677	3.84E-29
FAM111A	0.261099	4.63E-05
FAM111B	0.823744	3.07E-14
FAM114A1	-0.30867	0.003522
FAM117B	-0.35175	0.006196
FAM120B	-0.26281	6.51E-10
FAM120C	-0.2667	0.027026
FAM126A	0.314989	2.52E-05
FAM127A	-0.35657	0.000615
FAM127C	-0.30374	0.029403
FAM129A	0.244223	0.041544
FAM129B	0.709997	7.04E-07
FAM134A	-0.18651	0.000549
FAM134B	-1.7061	5.30E-17
FAM134C	-0.2156	0.000508
FAM135A	-0.40961	0.000165

FAM136A	0.395254	6.76E-09
FAM13A	-0.1765	0.020425
FAM13A-A!	-0.4678	0.00764
FAM13B	-0.23556	0.005608
FAM157A	0.740021	3.43E-06
FAM160A1	-0.30254	0.007294
FAM160B1	-0.3787	4.20E-09
FAM160B2	-0.43893	2.59E-07
FAM162A	0.38249	5.78E-08
FAM167A	1.194404	1.07E-10
FAM168A	-0.31241	5.16E-07
FAM169A	-0.2271	0.000111
FAM171A1	0.478702	2.16E-07
FAM171B	-0.91916	3.02E-14
FAM172A	-0.27576	0.000384
FAM172BP	2.884496	4.48E-16
FAM174A	-0.40313	0.001717
FAM174B	-0.63361	0.032352
FAM177A1	-0.1102	0.039982
FAM181B	-1.41451	5.07E-06
FAM183A	-0.88419	0.026709
FAM184A	0.673361	0.015326
FAM185A	-0.30293	0.010523
FAM187A	0.338514	0.030018
FAM189B	0.331006	0.001775
FAM196B	4.741314	4.79E-18
FAM198B	-2.31924	2.13E-19
FAM19A3	1.059719	0.014971
FAM206A	0.165317	0.002753
FAM207A	0.776018	2.04E-06
FAM208B	0.191837	0.001796
FAM20B	0.178474	0.008764
FAM210A	0.242504	0.001066
FAM210B	-0.29627	8.00E-05
FAM212A	-0.81285	1.98E-07
FAM212B	-0.34996	0.027171
FAM213A	-0.65328	4.71E-07
FAM214A	-0.46091	2.15E-08
FAM214B	0.98261	1.22E-08
FAM215B	-0.38488	0.005128
FAM216A	0.698659	4.90E-13
FAM21FP	-0.5577	0.039218
FAM222A	1.075892	2.46E-07
FAM223A	-1.68693	0.014275
FAM223B	-1.64089	0.017501
FAM227A	-0.79206	0.000117
FAM229A	-0.66074	0.026845

FAM230C	-1.72558	0.04524
FAM234A	-0.85482	2.48E-11
FAM234B	-0.64456	1.71E-09
FAM26E	-0.61847	0.000287
FAM26F	0.453376	0.035834
FAM30A	-1.49486	0.026324
FAM35BP	-1.53467	2.70E-06
FAM35DP	-0.77173	0.022894
FAM41C	-0.6329	0.029324
FAM43A	1.237426	7.73E-08
FAM45A	-0.2492	2.63E-08
FAM46A	-0.52443	0.007116
FAM46B	-2.86612	3.19E-18
FAM47E	-0.38868	0.00791
FAM47E-ST	-0.64706	9.87E-06
FAM49B	0.23393	0.000565
FAM50A	0.186468	0.039911
FAM53B	-0.39673	1.74E-05
FAM53C	-0.13867	0.011301
FAM58A	0.249614	0.004399
FAM65A	0.468857	3.82E-05
FAM65B	-0.56384	0.00056
FAM69A	-0.63347	3.56E-12
FAM71E1	-0.64319	0.021051
FAM71F2	-0.62808	0.013758
FAM72A	0.604961	0.001191
FAM72B	0.375031	0.044811
FAM72C	0.602682	0.004074
FAM72D	0.529151	0.002979
FAM76A	-0.49861	1.49E-06
FAM78A	-1.0064	9.42E-09
FAM81B	-2.93999	0.021028
FAM83A	1.595956	1.26E-38
FAM83A-AS	1.668029	1.18E-31
FAM83B	0.236719	0.003647
FAM83E	-1.31665	0.024203
FAM83G	0.423657	2.12E-05
FAM83H	0.285027	0.019418
FAM83H-A	-0.28205	0.025568
FAM84A	-0.38624	0.021932
FAM84B	0.727432	2.25E-06
FAM86B1	-0.48188	0.029361
FAM86B3P	-0.38355	0.001508
FAM86C1	-0.40793	0.001248
FAM86C2P	-0.65465	0.016531
FAM87A	-0.92499	0.018454
FAM91A1	0.288013	0.000149

FAM96A	0.198539	0.041409
FAM98A	0.153777	0.02229
FAM98B	0.343216	0.000286
FAM9A	-1.70957	0.036521
FANCA	0.734753	4.22E-19
FANCB	0.711593	1.73E-06
FANCD2	0.706083	3.17E-12
FANCD2OS	-1.66159	0.043781
FANCF	-0.31725	0.000942
FANCG	0.700005	5.35E-17
FANCI	0.715556	9.44E-17
FANCM	0.504059	1.60E-07
FANK1	-0.99148	4.17E-08
FAP	-0.85106	3.31E-08
FAR2	0.85581	2.03E-07
FAR2P4	-3.02206	0.004511
FARP1	0.721934	2.04E-17
FARP2	-0.42592	7.02E-05
FARSA	0.322972	2.65E-05
FARSB	0.371456	4.03E-12
FAS	0.529	2.36E-13
FASN	0.305929	0.018289
FASTK	-0.33001	0.000195
FASTKD2	0.158391	0.006944
FAT1	-0.27705	0.001061
FAT2	-2.10444	7.27E-34
FAT4	-2.04459	1.88E-36
FAU	0.235003	0.000108
FAXDC2	-0.87784	4.24E-07
FBL	0.463938	6.25E-12
FBLIM1	0.951359	3.46E-16
FBLN1	-0.50234	3.03E-06
FBLN2	-2.52379	2.88E-13
FBN1	-1.69234	1.20E-34
FBN2	0.335098	1.08E-08
FBRSL1	0.381094	0.005741
FBXL14	0.562449	1.57E-06
FBXL17	-0.44609	0.000485
FBXL18	0.350791	0.004972
FBXL19	0.353053	0.004975
FBXL19-AS1	-0.32935	0.000271
FBXL2	-0.6352	7.91E-11
FBXL20	-0.63607	3.94E-15
FBXL3	-0.1961	0.002158
FBXL4	-0.24792	2.28E-05
FBXL6	0.444917	2.18E-08
FBXL8	-0.36432	0.031542

FBXO10	-0.59806	2.18E-16
FBXO11	0.187683	0.002632
FBXO15	-0.30416	0.006403
FBXO2	-0.48915	0.004356
FBXO22	0.176802	0.004137
FBXO24	-0.78814	0.011898
FBXO25	-0.13791	0.02267
FBXO27	0.35145	0.004566
FBXO28	0.295019	8.32E-07
FBXO32	-1.03579	8.16E-19
FBXO34	-0.15366	0.015509
FBXO36	-0.37995	0.018398
FBXO39	-1.31493	0.035713
FBXO44	-0.39191	0.000752
FBXO45	0.484518	4.94E-16
FBXO48	0.264446	0.048035
FBXO5	0.499223	2.43E-08
FBXO7	-0.10118	0.048281
FBXW10	-0.95483	0.035126
FBXW11P1	-3.41332	0.003059
FBXW2	-0.21432	0.000146
FBXW4	-0.66605	3.17E-09
FBXW4P1	0.743629	0.001319
FBXW5	0.314123	0.018593
FBXW8	-0.4479	6.24E-07
FBXW9	-0.47324	0.00082
FCGR1A	-0.63998	0.024882
FCGR1B	-1.15063	0.000845
FCGRT	0.30526	0.015214
FCHO1	0.954673	0.000335
FCHSD2	0.590381	7.88E-11
FCRLB	1.018399	0.000159
FDXR	-0.39512	1.58E-05
FECH	-0.86237	1.44E-19
FEM1A	0.394142	0.025433
FEM1B	-0.3149	0.003139
FEN1	0.715746	3.33E-33
FER	-0.21413	0.001676
FER1L5	-0.51419	0.035456
FER1L6	0.814366	0.016181
FES	0.538977	1.81E-06
FEZ2	0.327658	2.87E-06
FGD1	0.42482	9.64E-07
FGD3	-2.96339	6.99E-37
FGD4	-0.33301	1.25E-05
FGD6	0.396119	5.11E-06
FGF1	1.697026	5.19E-07

FGF11	-0.75074	3.70E-11
FGF2	0.682811	3.55E-07
FGF5	1.754008	3.63E-35
FGF7	-0.74561	0.012424
FGFBP1	-1.0641	2.87E-26
FGFR1	0.834481	0.001053
FGFR1OP	0.217505	0.008615
FGFR1OP2	0.233383	0.00047
FGFR2	-1.60211	1.53E-14
FGFR3	-2.68804	2.51E-09
FGGY	-0.24741	0.005829
FGR	-2.14395	0.030122
FH	0.447516	2.61E-19
FHAD1	0.836539	0.005251
FHDC1	0.534561	2.33E-08
FHL1	1.437649	3.77E-23
FHL2	0.850576	3.64E-15
FHL3	0.604472	2.49E-10
FHOD1	0.944382	1.26E-09
FIBP	0.150438	0.013827
FICD	0.274135	0.010569
FIGN	-0.41029	0.0002
FIGNL1	0.478069	9.36E-07
FILIP1L	-0.70081	0.028367
FIP1L1	0.37974	3.76E-12
FITM2	-0.36146	0.000794
FIZ1	0.163018	0.046299
FJX1	1.081072	2.41E-13
FKBP10	0.37609	6.62E-11
FKBP15	0.159612	0.002392
FKBP1A	1.554915	2.49E-27
FKBP1B	-0.30837	0.010969
FKBP1C	2.357179	0.003073
FKBP4	0.235246	0.000764
FKBP5	-1.27879	8.31E-16
FKBP9	-0.65749	5.60E-27
FLCN	-0.27251	0.000467
FLG	-2.12814	1.27E-06
FLG-AS1	-1.34864	0.000404
FLI1	2.01117	9.56E-40
FLII	0.363731	0.000164
FLJ12825	-0.67064	0.00092
FLJ22447	-0.36651	0.031001
FLJ30679	-1.09871	0.019743
FLJ31356	-2.83121	1.09E-13
FLJ37453	-0.46893	0.0304
FLJ46284	-0.64045	0.027948

FLNA	0.305932	0.002626
FLNB	0.44758	6.67E-11
FLNB-AS1	0.51941	0.000105
FLNC	1.415402	1.45E-18
FLOT1	-0.2946	3.83E-05
FLOT2	-0.38275	1.06E-14
FLRT3	-1.37942	6.77E-10
FLT1	1.139145	5.36E-06
FLVCR1	-0.12952	0.041122
FLVCR2	-0.72648	1.89E-09
FMC1	-0.54038	1.15E-05
FMN1	0.340992	0.002696
FMNL1	0.719687	1.25E-08
FMNL2	0.413552	1.54E-08
FMNL3	1.289282	5.90E-18
FMO4	-0.59185	4.41E-08
FMO5	-0.50584	1.72E-05
FMR1-IT1	-1.66809	0.004496
FN1	-0.26562	0.030382
FN3K	-0.54952	0.000242
FN3KRP	0.17351	0.005898
FNBP1L	-0.19124	0.000229
FNBP1P1	-0.42689	0.015417
FNBP4	-0.22062	0.000876
FNDC10	-0.68814	0.000241
FNDC4	0.368795	0.028761
FNTB	0.206577	0.007103
FOLR1	-3.18232	7.17E-07
FOS	2.110401	9.54E-56
FOSL1	2.700517	1.02E-55
FOSL2	-1.26978	1.18E-23
FOXA1	1.573026	6.53E-05
FOXA2	4.877347	8.24E-09
FOXB1	-0.40237	0.048321
FOXC1	-0.76705	1.17E-06
FOXC2	1.520487	1.55E-06
FOXD1	2.025459	2.83E-31
FOXD1-AS1	1.764191	5.38E-11
FOXD2	0.788931	0.002957
FOXD3-AS1	-0.5328	0.049992
FOXE1	0.361608	0.010826
FOXH1	-3.44611	0.002944
FOXJ2	-0.21137	0.001296
FOXJ3	0.142265	0.015816
FOXK1	0.191677	0.029598
FOXL1	1.224414	1.20E-07
FOXL2NB	-0.41087	0.021493

FOXM1	0.645212	6.59E-12
FOXN1	-3.3518	1.66E-05
FOXN2	0.321257	0.000205
FOXN3	-0.36145	4.85E-06
FOXO3	-0.1604	0.018044
FOXO4	-0.93306	7.49E-21
FOXP1	0.370863	5.16E-05
FOXP4	0.435828	3.82E-05
FOXQ1	3.695115	6.17E-43
FOXRED1	0.203469	0.000166
FOXRED2	-0.20156	0.004541
FOXS1	-2.23397	0.005411
FPGS	0.3331	0.000241
FPGT	-0.47565	2.75E-07
FRAS1	-0.68405	2.50E-09
FRAT1	-0.39003	0.047977
FREM2	-0.35794	0.030669
FRG1	0.338482	2.00E-05
FRG1BP	-0.34245	0.002595
FRG1HP	-0.25651	0.003583
FRG1KP	-3.04572	4.36E-05
FRK	-0.66583	6.57E-16
FRMD3	-0.97378	0.004812
FRMD4A	1.219051	2.84E-20
FRMD4B	-0.22878	0.000365
FRMD5	0.534023	6.91E-06
FRMD6	0.639098	6.66E-13
FRRS1	-0.50551	9.65E-14
FRS2	-0.39697	3.99E-07
FRS3	0.298333	0.013796
FSCN1	1.264981	6.34E-14
FSCN2	-0.90598	0.044581
FSTL1	-0.41216	1.53E-06
FSTL3	-1.68465	4.71E-23
FTH1	-0.22592	0.026678
FTH1P11	-0.80858	0.031451
FTO	-0.22519	0.000525
FTSJ1	0.172502	0.001078
FTSJ3	0.250892	1.09E-06
FTX	-0.63265	2.48E-15
FUBP1	0.30068	0.001272
FUBP3	0.162516	0.000275
FUK	-0.41646	1.49E-05
FUOM	0.86845	5.09E-05
FURIN	0.510934	6.98E-06
FUS	0.482618	0.001072
FUT1	0.662192	1.14E-10

FUT10	-0.34961	0.000444
FUT2	0.38487	0.039897
FUT3	1.220863	7.53E-10
FUT4	0.831733	1.46E-11
FUT8	0.964451	2.99E-16
FUT8-AS1	1.607697	0.0056
FUT9	-2.49142	1.32E-07
FXN	0.424173	4.64E-05
FXR2	0.280278	0.000378
FXYD3	-2.25296	1.00E-15
FXYD5	0.772772	1.11E-16
FYB	-1.34947	0.000276
FYCO1	-0.2315	0.038172
FZD1	-0.82184	1.17E-13
FZD3	-0.3544	4.53E-05
FZD4	-0.35525	0.041053
FZD5	-0.14588	0.043714
FZD6	-0.23961	0.000576
FZD7	-0.85696	3.21E-07
G6PD	-0.60296	4.42E-08
GAA	-1.48706	3.20E-15
GAB1	-0.58335	6.73E-17
GAB3	0.704926	0.031695
GABARAP	-0.19171	4.44E-05
GABARAPL	-1.66275	7.56E-47
GABBR1	-0.60416	2.26E-06
GABBR2	1.437101	0.035883
GABPB1	0.287519	2.37E-05
GABPB1-AS	-0.6296	4.36E-12
GABPB2	-0.24944	0.008518
GABRG1	-1.39589	0.001161
GADD45A	-0.61856	5.47E-05
GADD45B	-1.00546	1.96E-09
GADD45GI	0.389517	0.00048
GAK	0.212367	0.037176
GAL	1.058948	0.003376
GAL3ST4	-1.4414	1.57E-23
GALC	-0.5242	0.000861
GALE	0.467114	8.22E-11
GALK1	-0.31121	0.045516
GALK2	0.326172	0.000195
GALNS	-0.84351	3.94E-10
GALNT11	-0.28817	2.93E-05
GALNT12	-0.44219	0.001031
GALNT13	-1.24203	0.004029
GALNT14	0.971897	0.000236
GALNT15	-3.03089	1.37E-12

GALNT2	-0.48615	3.32E-15
GALNT3	0.258572	0.001475
GALNT4	0.343969	0.014068
GALR2	1.183864	0.041824
GALT	-0.26897	0.000289
GAMT	-0.18847	0.03012
GANC	-0.36916	6.91E-05
GAPDH	0.252852	2.98E-05
GAPDHP21	-0.91216	0.045745
GAR1	0.215884	0.002198
GAREM1	-0.98297	1.70E-13
GARNL3	-0.90001	5.16E-06
GARS	0.190319	0.005956
GART	0.545408	3.00E-19
GAS6	-0.3956	0.006611
GAS6-AS1	-1.29435	0.003075
GAS8	-0.26826	1.02E-05
GATA1	-2.4959	0.045567
GATA2	-0.8277	4.89E-06
GATA2-AS1	-0.52183	3.48E-05
GATA3	-0.54326	0.000608
GATA6	-0.70922	2.32E-11
GATAD1	-0.17506	0.000416
GATAD2A	0.544863	3.98E-10
GATS	-1.18074	3.66E-21
GATSL3	-1.40487	2.86E-21
GBA	-0.15262	0.041632
GBA2	0.239853	0.001479
GBE1	-0.21615	0.000179
GBF1	0.149788	0.002478
GBP1	-1.03934	0.00853
GBP1P1	-1.4551	0.000114
GBP2	-1.99815	3.59E-06
GBP6	-1.34439	1.71E-08
GBX2	1.416408	8.68E-07
GCDH	0.178795	0.01523
GCFC2	0.369937	5.05E-08
GCH1	0.630853	8.42E-08
GCLC	-1.29007	6.41E-36
GCN1	0.272787	9.88E-08
GCNT2	0.738704	1.53E-07
GCNT3	-1.28013	9.40E-05
GCNT4	-1.10325	0.000189
GCOM1	0.839756	1.04E-06
GCSAM	-0.88916	1.10E-08
GDAP1	0.239957	0.040159
GDE1	-0.26756	3.01E-05

GDF1	3.285617	0.022907
GDF11	0.31952	0.000118
GDF15	1.214283	3.96E-08
GDI1	0.211358	0.007115
GDI2	0.144782	0.000289
GDPD3	-0.5724	0.000242
GDPD5	1.738011	2.72E-19
GEM	1.941858	3.86E-10
GEMIN4	0.27524	1.35E-05
GEMIN5	0.336766	1.01E-06
GEMIN6	0.156644	0.011976
GEMIN7	0.295264	0.002216
GEN1	0.192984	0.035483
GET4	0.384451	0.00012
GFER	0.365628	2.27E-06
GFI1B	-0.31161	0.018043
GFM1	0.161252	0.001033
GFM2	-0.1528	0.018148
GFOD1	-0.41372	0.000459
GFOD2	0.192674	0.043807
GFPT1	0.254109	0.025974
GFPT2	1.101265	2.47E-13
GGACT	-0.38407	0.012264
GGH	0.323363	0.002689
GGT6	-1.83595	3.04E-46
GGT7	-0.22514	0.018242
GHR	-0.82695	0.017491
GID4	-0.20223	0.039822
GIMAP2	-0.60728	0.00087
GINS1	0.485554	1.72E-07
GINS2	0.770762	2.22E-12
GINS3	0.602942	8.81E-15
GINS4	0.566585	6.26E-10
GIT1	0.406958	4.76E-07
GIT2	0.223416	0.000136
GJA1	0.32865	0.012654
GJA5	-1.72015	0.027117
GJB2	1.613618	1.20E-38
GJB3	0.794772	3.61E-10
GJB4	0.893945	1.65E-05
GJB5	0.446347	0.007689
GJB7	-1.33269	0.000199
GJC1	0.912194	3.27E-23
GJC2	1.696004	4.60E-07
GJD3	2.974099	0.00734
GK	0.765225	1.16E-06
GLA	0.25149	0.014775

GLB1	-0.57173	8.57E-10
GLB1L	-0.73361	5.20E-07
GLCCI1	-0.92965	4.14E-17
GLCE	0.279671	0.000464
GLDN	-0.9684	1.48E-05
GLE1	0.150865	0.011894
GLI1	-1.38555	0.016508
GLI2	-1.42955	0.000528
GLI4	-0.50993	0.000684
GLIDR	-0.43385	0.033448
GLIPR2	-0.99397	3.37E-12
GLIS2	-1.06882	3.01E-15
GLIS3	-0.74231	0.001596
GLMN	0.158749	0.040292
GLOD4	0.127689	0.011046
GLP2R	-4.59078	0.000607
GLRB	0.340344	0.030877
GLRX2	0.519077	1.97E-09
GLRX3	0.374375	8.46E-07
GLS	0.325043	0.000243
GLT8D1	-0.45625	1.91E-09
GLT8D2	-0.19803	0.008225
GLTP	0.442289	7.18E-08
GLTSCR1L	-0.28945	0.000184
GLTSCR2	0.278987	0.013401
GLUD1	0.334776	1.28E-09
GLUD1P3	-0.55004	0.009898
GLYCTK	1.005325	1.02E-24
GM2A	-0.11921	0.036809
GMEB1	0.355942	5.23E-07
GMEB2	0.207321	0.01505
GMFB	0.206558	0.005803
GMIP	0.452771	0.002818
GMNN	0.546196	8.38E-12
GMPPB	0.397066	1.96E-07
GMPR	-1.06572	2.36E-05
GMPR2	-0.22226	0.000751
GMPS	0.488689	1.13E-13
GNA11	0.264238	0.002223
GNA12	0.31518	7.40E-05
GNA15	0.719147	5.41E-08
GNAI1	-0.77748	1.16E-21
GNAI2	0.407237	8.96E-13
GNAI3	0.199455	0.001641
GNAL	-0.92484	1.52E-06
GNAO1	-3.54594	1.00E-23
GNAQ	-0.38857	6.35E-11

GNAS	-0.11511	0.008851
GNAT3	-4.11591	0.000377
GNB1	0.27801	4.95E-07
GNB4	0.383429	4.47E-05
GNB5	-0.30279	0.000841
GNG11	2.022976	1.60E-22
GNG5	0.514942	2.65E-10
GNGT1	-0.45024	0.032371
GNL2	0.301122	0.002275
GNL3	0.300197	7.96E-05
GNL3L	0.364198	1.51E-06
GNL3LP1	0.635764	0.007871
GNPNAT1	0.492522	5.81E-12
GNPTAB	-0.66381	2.08E-11
GNRHR	1.23705	0.036058
GNRHR2	-0.50036	0.015099
GNS	-0.36689	0.000291
GOLGA2	-0.16092	0.004215
GOLGA2P5	-0.65522	9.28E-05
GOLGA3	0.205996	0.010003
GOLGA6L5	-0.3676	0.006188
GOLGA6L9	-0.26798	0.020639
GOLGA8A	-0.33766	0.001126
GOLGA8B	-0.49125	3.75E-06
GOLGA8F	-3.77822	3.06E-12
GOLGA8G	-3.8431	6.95E-12
GOLGA8N	-1.28035	2.68E-08
GOLGA8R	-1.15942	0.002609
GOLGB1	-0.38483	7.60E-08
GOLT1A	1.253804	2.45E-08
GOLT1B	0.181297	0.014308
GOPC	-0.41525	7.00E-09
GORASP1	-0.24567	1.16E-05
GORASP2	0.205375	0.000738
GOSR2	0.152432	0.000851
GOT1	0.219847	3.07E-05
GOT2	0.332751	7.75E-11
GP1BA	-0.86448	0.002905
GPAA1	0.223507	0.027738
GPALPP1	-0.20243	0.007633
GPANK1	0.288488	0.002886
GPAT3	0.901876	8.45E-08
GPAT4	0.33007	3.77E-07
GPATCH11	0.418591	3.06E-08
GPATCH2	-0.20025	0.010372
GPATCH3	-0.2235	0.001775
GPATCH4	0.296909	1.40E-05

GPBP1L1	-0.11958	0.005275
GPC1	-1.6359	7.35E-11
GPHN	0.422738	3.91E-09
GPI	0.375713	6.48E-11
GPKOW	0.170954	0.022084
GPLD1	-0.64485	0.005191
GPM6A	-0.87026	4.96E-08
GPN1	0.1764	0.001241
GPN3	0.439247	1.37E-07
GPNMB	-1.98121	4.80E-30
GPR1	-3.27907	4.09E-20
GPR107	-0.30798	3.15E-11
GPR108	-0.23048	0.000859
GPR135	-0.33778	0.013503
GPR150	-1.50966	0.037929
GPR155	-1.06527	6.33E-11
GPR158	-0.4428	1.05E-08
GPR160	0.591355	9.35E-05
GPR161	-0.32683	6.62E-06
GPR17	-0.8539	0.006322
GPR176	0.377081	0.000172
GPR179	-2.37694	0.003869
GPR18	2.531212	0.022807
GPR180	0.182528	0.002768
GPR3	0.756478	0.000106
GPR37	-0.57079	6.82E-05
GPR37L1	-1.29394	0.002425
GPR4	6.102897	2.61E-20
GPR68	-1.71319	2.56E-19
GPR75	0.315211	0.043412
GPR85	-0.96557	1.22E-07
GPR87	-0.6359	4.48E-12
GPR89A	0.122551	0.01691
GPRASP1	-0.58234	0.005883
GPRC5A	-0.76318	0.045278
GPRC5B	-0.9907	7.44E-09
GPRIN1	0.778323	3.86E-22
GPRIN2	-2.2523	6.71E-19
GPS1	0.250227	0.001276
GPS2	0.409666	1.01E-05
GPSM3	2.845599	9.19E-31
GPT	-1.12339	0.005954
GPX1	0.43872	0.00382
GPX2	-1.14216	0.001313
GPX4	-0.39616	0.000191
GPX7	-0.69718	0.00016
GRAMD1A	0.447624	0.000522

GRAMD1B	1.448042	3.13E-05
GRAMD1C	-0.87465	7.79E-20
GRAMD2	-2.90302	1.45E-16
GRAMD4	-0.45214	0.001347
GRB14	-0.26132	0.043726
GRB2	0.161476	0.000328
GRB7	0.589112	0.000163
GREB1	-0.658	1.02E-05
GREM1	2.384338	2.16E-20
GRHL1	-0.42442	2.29E-05
GRHL2	-0.78126	2.59E-19
GRHPR	-0.18782	0.009486
GRIK2	-2.28069	4.56E-12
GRIN2C	-0.79027	0.00168
GRIN2D	0.683977	3.10E-07
GRIN3A	-1.04	0.045065
GRIP1	-0.54882	0.021636
GRK5	0.391368	0.019968
GRK6	0.205234	0.015338
GRM1	-1.42868	0.02868
GRN	-0.38869	0.000118
GRPEL1	0.642593	1.37E-15
GRPR	2.203189	0.000372
GRSF1	0.215011	6.42E-05
GRTP1	-0.4705	0.000123
GRWD1	0.296814	1.41E-05
GS1-114I9.	-0.53288	0.030774
GS1-358P8	-1.01357	0.003302
GS1-393G1	1.096286	0.031409
GS1-594A7	-2.932	0.027391
GSDMA	4.745198	4.64E-41
GSDMB	-0.64569	3.04E-05
GSDMC	0.85282	2.59E-06
GSE1	-0.34672	0.000311
GSG1	-1.46127	0.008293
GSG2	0.769409	1.18E-16
GSK3A	0.261153	3.58E-05
GSN	-0.33819	0.010914
GSPT1	0.428107	7.30E-11
GSR	-0.4863	1.37E-06
GSTA4	-1.58713	1.88E-07
GSTCD	0.455669	1.34E-06
GSTK1	-0.76931	2.20E-20
GSTM2	-1.54384	1.22E-15
GSTM3	-1.25284	1.20E-14
GSTM4	-3.04522	6.52E-23
GSTM5	-3.62545	0.022477

GSTO1	1.340993	6.26E-22
GSTO2	-0.30262	0.028178
GSTP1	-0.61337	7.93E-11
GSTT2B	-0.32898	0.014633
GSTZ1	-0.43893	1.24E-06
GTDC1	0.178114	0.029562
GTF2A1	0.257818	0.030135
GTF2A2	0.370575	1.06E-07
GTF2B	0.180332	0.029837
GTF2E2	0.237155	0.000512
GTF2F1	0.267359	1.14E-05
GTF2H1	0.109645	0.042152
GTF2H2	0.361816	0.0022
GTF2H2B	0.739701	3.13E-05
GTF2H5	0.200809	0.010018
GTF2I	-0.26068	5.18E-11
GTF2IP1	-0.49257	8.95E-10
GTF2IP4	-0.53948	8.23E-14
GTF2IRD2	-0.45433	1.08E-05
GTF2IRD2B	-0.56793	8.25E-09
GTF3A	0.251574	7.11E-05
GTF3C2	0.132316	0.020754
GTF3C5	0.225977	2.80E-06
GTF3C6	0.437898	2.92E-09
GTPBP10	0.246825	0.000176
GTPBP4	0.407639	7.96E-09
GTSE1	0.534217	4.08E-06
GUCY1A3	-1.30687	1.49E-05
GUCY1B2	-0.62764	0.000497
GUK1	0.385898	1.22E-05
GULP1	0.849747	1.47E-05
GVQW1	0.556851	0.004629
GXYLT1	0.552141	8.79E-10
GXYLT2	-0.50257	7.56E-05
GYG1	0.309601	3.37E-05
GYG2	-1.08552	1.44E-10
H19	-2.24483	6.38E-10
H2AFV	0.210932	0.049297
H2AFX	0.792499	1.45E-05
H2AFY	0.329993	3.28E-11
H2AFY2	-0.43659	0.000287
H2AFZ	0.590833	4.05E-11
H3F3B	0.355412	4.75E-14
H6PD	-1.36443	8.58E-19
HACD1	-0.44867	2.13E-06
HACD2	-1.14717	1.19E-26
HACD3	0.264703	1.32E-06

HACD4	-0.43593	7.18E-09
HADHA	-0.14241	0.000147
HADHB	-0.2974	1.13E-11
HAGLR	0.482332	0.004477
HAGLROS	0.952462	0.003076
HAP1	-1.17691	4.39E-07
HAPLN3	0.446701	0.009708
HARS	0.316065	2.71E-09
HAS3	0.936729	3.53E-08
HAT1	0.411411	6.72E-08
HAUS1	0.516161	3.79E-08
HAUS2	0.528415	1.45E-12
HAUS3	0.29696	0.000179
HAUS5	0.301388	0.000125
HAUS6	0.307352	1.81E-05
HAUS7	0.502635	1.49E-08
HAUS8	0.630241	2.80E-08
HBEGF	3.007939	1.21E-20
HBP1	-0.62056	5.25E-35
HBS1L	0.185921	0.002881
HCAR2	-2.6285	7.01E-20
HCAR3	-1.51307	5.17E-19
HCCS	0.157248	0.013527
HCFC1	0.350554	4.74E-05
HCG27	0.826623	0.007465
HCLS1	2.628094	5.41E-24
HCN3	-0.53486	1.77E-06
HCP5	-1.00498	3.00E-10
HCP5B	-1.65579	0.00628
HDAC1	0.227057	0.000335
HDAC11	-0.87654	1.97E-20
HDAC2	0.228524	0.00011
HDAC3	0.146082	0.0077
HDAC4	-0.62727	1.60E-07
HDAC5	-0.39273	2.51E-06
HDAC6	-0.50062	7.71E-09
HDAC9	1.087142	2.92E-08
HDGF	0.49769	2.30E-06
HDHD2	-0.25325	0.004018
HDHD3	-0.47039	9.94E-09
HDX	2.936706	0.036437
HEATR1	0.185238	0.00074
HEATR4	-0.99651	0.000279
HEATR6	-0.23036	8.87E-05
HECTD2	0.451805	9.26E-06
HECTD4	-0.2873	0.004236
HECW2	-0.84625	4.50E-07

HEG1	-2.49035	1.19E-46
HELLS	0.999299	9.97E-19
HELZ	-1.00156	1.01E-34
HENMT1	-0.17498	0.0222
HERC2	-0.22251	0.000151
HERC2P9	-0.25478	7.86E-05
HERPUD1	0.421324	0.005362
HERPUD2	-0.26229	4.42E-05
HES1	-0.27296	0.040859
HES2	-2.67664	1.15E-13
HES7	1.266766	0.000126
HESX1	-0.81242	0.017026
HEXA	-0.31249	7.08E-06
HEXA-AS1	-0.5688	0.044732
HEXDC	-0.39615	0.002378
HEXIM2	-0.44754	0.005374
HEY1	0.672384	1.98E-06
HFE	-0.74433	6.42E-26
HGH1	0.248658	0.003491
HGS	0.33657	0.001971
HGSNAT	-0.53551	3.29E-09
HHAT	-0.74266	2.70E-08
HHEX	0.433572	0.0042
HHIP-AS1	1.274238	9.11E-05
HHIPL1	-1.07292	0.022029
HHIPL2	-1.44185	0.003641
HHLA1	1.852893	1.08E-25
HHLA3	-0.3268	0.00119
HIBADH	-0.34572	4.81E-05
HIBCH	-0.23958	0.000122
HIC1	2.329713	3.89E-11
HIC2	-0.33142	4.58E-08
HID1	-0.58318	0.015148
HIF1AN	-0.2419	4.53E-07
HINFP	-0.16421	0.015763
HINT3	-0.71212	9.27E-17
HIP1	1.023351	2.70E-22
HIP1R	-0.13776	0.032813
HIPK3	-0.13522	0.041922
HIRIP3	0.530498	8.99E-08
HIST1H1C	-1.43235	3.47E-17
HIST1H1PS	-0.96361	0.008713
HIST1H2AC	-1.28448	1.26E-34
HIST1H2AG	-0.70589	0.000903
HIST1H2AI	-1.1218	0.011859
HIST1H2BC	-0.53798	0.013444
HIST1H2BD	-1.03921	2.53E-11

HIST1H2BE	-0.901	0.026306
HIST1H2BK	-0.50304	0.0102
HIST1H2BN	-0.84548	1.15E-05
HIST1H3D	-0.94781	0.000154
HIST1H3E	-0.85117	8.53E-06
HIST1H3G	-1.02027	0.003224
HIST1H3H	-1.16005	0.000171
HIST1H4E	-0.66506	0.022969
HIST1H4H	-1.41233	1.88E-11
HIST1H4I	-0.75435	9.13E-08
HIST1H4J	-0.52769	0.03369
HIST2H2AA	-0.39475	0.026595
HIST2H2AC	1.256956	0.029998
HIST2H2BA	-1.03557	0.034768
HIST2H2BC	-0.40189	0.014188
HIST2H2BE	-1.21055	6.48E-38
HIST2H2BF	-0.50861	4.42E-05
HIST2H4B	-1.15814	0.009823
HIST4H4	-0.98397	7.45E-06
HIVEP3	0.716079	2.99E-06
HK1	0.457811	9.42E-05
HLA-B	-0.54287	0.024361
HLA-C	-0.39069	0.007863
HLA-DMA	-0.95655	5.85E-12
HLA-DOB	-1.34666	4.45E-05
HLA-E	-0.34439	0.012201
HLA-F	-0.84934	0.001669
HLA-V	-0.82565	4.82E-10
HLCS	-0.148	0.030127
HLTF	0.163433	0.016057
HM13	0.183727	0.018177
HMCES	0.268933	1.72E-07
HMG20A	-0.11637	0.044887
HMG20B	-0.56781	8.44E-11
HMGA1	2.186747	1.70E-40
HMGA2	2.895602	1.74E-61
HMGB1	0.445618	1.82E-06
HMGB1P1	-1.35168	0.012595
HMGB1P3	1.49673	0.00066
HMGB1P6	0.562628	9.55E-10
HMGB2	0.396001	0.020045
HMGB3	-0.33566	0.00208
HMGCL	-0.42485	6.73E-06
HMGCR	-0.38436	0.000838
HMGCS1	-0.51641	0.00046
HMGN1	0.260932	0.001107
HMGN1P3	1.87596	0.003071

HMGN2	0.233222	0.004052
HMGN2P1!	1.938778	0.039517
HMGN3	-0.58878	9.33E-06
HMGN3-AS	-0.72566	4.15E-05
HMGXB3	0.173833	0.017497
HMGXB4	0.125747	0.036634
HMMR	0.437983	0.006814
HMOX2	0.453742	9.78E-08
HMX2	1.922823	0.010309
HN1	0.273092	0.001202
HN1L	0.179956	0.014537
HNRNPA1	0.22441	0.007905
HNRNPA1P	-0.52895	3.40E-09
HNRNPA1P	0.467521	0.01399
HNRNPA1P	-0.36985	0.025795
HNRNPA2B	0.27849	0.00277
HNRNPA3	0.290228	0.0058
HNRNPA3P	0.806938	0.008292
HNRNPAB	0.541963	4.04E-32
HNRNPC	0.157881	0.014827
HNRNPD	0.350872	1.60E-06
HNRNPF	0.182411	1.78E-05
HNRNPK	0.185238	0.000104
HNRNPL	0.310227	3.80E-10
HNRNPLP2	-1.34915	0.04892
HNRNPM	0.325807	3.22E-05
HNRNPU	0.320941	1.52E-06
HNRNPUL1	0.4149	7.99E-09
HOGA1	-1.66568	5.28E-09
HOMER1	0.192971	0.032305
HOMER2	-0.23171	0.015044
HOMER3	0.72533	1.26E-06
HOMEZ	-0.61466	4.64E-12
HOOK1	0.416345	0.000423
HOOK2	-0.16066	0.047408
HORMAD2-	-3.23324	0.041601
HOTAIR	-0.89376	0.001205
HOXA1	0.251312	0.005152
HOXA10-AS	0.653164	0.005056
HOXA11	0.548485	0.002083
HOXA13	-0.61815	0.008967
HOXA2	0.500707	0.048194
HOXA3	0.644828	1.31E-05
HOXA6	-0.51617	0.032919
HOXA9	0.522612	0.001452
HOXB13	-0.36996	0.001236
HOXB2	-3.1094	0.014284

HOXB3	-0.48492	0.007149
HOXB9	0.341993	0.00084
HOXC11	-0.67561	1.49E-05
HOXC12	-0.90301	0.009075
HOXC13	-1.63078	1.86E-23
HOXC13-AS	-2.25835	4.68E-27
HOXC4	-0.4158	0.018334
HOXC6	-0.39559	0.019013
HOXD1	1.311384	2.03E-07
HOXD10	-0.71962	1.51E-19
HOXD11	-0.45382	0.000157
HOXD13	0.314168	0.015816
HOXD4	-3.6428	0.015753
HOXD9	-0.97232	5.38E-12
HOXD-AS2	-1.04672	1.99E-06
HPCAL1	0.978755	1.54E-14
HPCAL4	2.119883	9.49E-16
HPRT1	0.55825	3.49E-11
HPS1	-0.35841	6.56E-07
HPS4	-0.10059	0.023909
HPS5	-0.41383	1.21E-08
HR	-1.76401	6.35E-18
HRASLS2	-3.94034	1.38E-15
HRASLS5	-3.43269	0.002785
HRCT1	-0.67439	0.014532
HRH1	0.923265	2.29E-26
HRK	-2.30263	5.59E-08
HRNR	-2.49139	4.11E-20
HS1BP3	-0.26487	0.000338
HS1BP3-IT1	1.122841	0.006621
HS2ST1	-0.1811	0.024613
HS3ST1	1.682893	5.59E-20
HS3ST5	-2.60987	0.012074
HS6ST1	-0.58246	9.21E-06
HS6ST2	0.340616	0.000687
HS6ST3	-1.72016	6.11E-05
HSD11B1	1.612523	0.046019
HSD11B1L	-0.94683	7.06E-05
HSD17B1P1	-1.00007	0.019863
HSD17B2	0.535795	0.002006
HSD17B4	-0.29694	1.29E-05
HSD17B6	-0.98129	4.32E-12
HSD17B8	-0.51701	2.32E-07
HSD3B1	1.58493	0.04
HSD3BP5	1.573346	0.000648
HSDL2	-0.29529	5.70E-07
HSF1	0.18475	0.023933

HSF4	-0.77822	0.00011
HSFX1	-1.53151	0.001073
HSFX2	4.169257	0.017792
HSP90AA1	0.753624	1.27E-22
HSP90AB1	0.419718	3.34E-12
HSP90B1	0.2999	0.000655
HSPA12A	-1.56939	8.27E-11
HSPA13	0.373065	6.12E-05
HSPA14	0.355407	0.000552
HSPA1A	0.586098	5.23E-05
HSPA4	0.240599	1.17E-05
HSPA5	0.296749	0.012695
HSPA6	1.450599	6.08E-09
HSPA8	0.734199	4.28E-25
HSPA9	0.274776	2.84E-06
HSPB1	-1.39796	7.46E-07
HSPB1P1	-1.5539	0.049017
HSPB8	-1.54627	0.000604
HSPB9	-1.13896	0.021113
HSPBP1	0.331577	0.00564
HSPD1	0.457652	5.31E-17
HSPD1P11	-2.40343	5.45E-08
HSPE1	0.418131	1.80E-08
HSPE1-MO	0.475396	0.033971
HSPG2	-1.46878	3.90E-09
HSPH1	0.691854	1.13E-24
HTATIP2	-0.36127	2.01E-07
HTATSF1P2	0.582648	0.027051
HTR1D	-0.76419	4.27E-09
HTR3E-AS1	1.608869	0.049078
HTR7	1.771314	4.10E-22
HTR7P1	-0.53408	0.00201
HTRA1	-1.15612	4.62E-11
HTRA2	0.21365	0.001669
HTT	-0.1257	0.042383
HUS1	0.474272	6.42E-11
HUS1B	1.967514	0.01103
HUWE1	-0.27343	1.59E-06
HVCN1	0.514288	0.00563
HYAL1	-0.79365	7.45E-09
HYI	1.348226	3.74E-50
HYOU1	0.277466	0.000928
IARS	0.227576	4.32E-05
IARS2P1	0.884173	0.049056
IBA57	0.35983	1.60E-05
IBSP	-0.56599	0.025488
ICA1	0.304527	0.038664

ICA1L	0.399412	0.001422
ICAM1	0.951843	7.61E-08
ICAM4	1.890351	0.011084
ICK	-0.73335	6.65E-25
ICMT	0.231133	3.79E-05
ICOSLG	-0.51241	0.001379
ID1	-1.51616	0.000523
ID2	-2.00604	9.83E-12
ID2-AS1	-2.64591	0.03085
ID3	-1.46451	1.38E-06
ID4	-2.16967	0.000133
IDH1	-0.92505	1.53E-34
IDH2	-0.46965	8.99E-06
IDH3A	0.457494	3.99E-09
IDH3B	0.235791	6.61E-05
IDH3G	0.264183	0.002574
IDI2-AS1	0.946565	0.013709
IDS	0.831586	2.77E-23
IDUA	-1.2309	2.81E-08
IER2	1.397641	2.55E-14
IER3	1.912189	7.52E-28
IER5	1.382652	1.29E-41
IER5L	0.867919	0.001094
IFFO1	2.320107	1.85E-20
IFI30	-1.16239	3.01E-29
IFITM10	-3.10363	1.60E-08
IFNA1	3.043855	0.030543
IFNAR1	0.148866	0.012637
IFNE	2.180681	4.18E-23
IFNGR1	-0.51146	8.99E-05
IFNGR2	0.605547	4.62E-14
IFNWP19	2.471469	1.97E-37
IFRD2	0.468306	5.12E-13
IFT122	-0.3229	0.000478
IFT140	-0.42834	2.18E-05
IFT172	-0.20373	0.006748
IFT27	-0.27966	0.021859
IFT43	-0.67256	2.57E-19
IFT74	-0.33505	0.000612
IFT80	-0.44182	5.22E-07
IFT81	-0.40552	0.013436
IGBP1	-0.18822	0.007994
IGF1R	-0.49048	1.32E-05
IGF2	-3.11279	5.85E-13
IGF2-AS	-2.74724	1.57E-06
IGF2BP1	0.167739	0.000903
IGF2BP2	0.476589	3.71E-11

IGF2BP2-AS	1.631801	5.72E-05
IGF2BP3	-0.13744	0.033008
IGFBP3	-2.54079	3.07E-07
IGFBP4	0.600227	1.31E-10
IGFBP6	1.054545	5.35E-10
IGFL1	-2.80716	1.81E-09
IGFL1P1	-3.84883	5.67E-19
IGFL2-AS1	-2.9826	2.59E-16
IGFN1	3.485852	4.76E-06
IGHMBP2	0.414672	0.000151
IGIP	-0.33684	0.023887
IGKV1OR-2	4.130683	0.000905
IGSF23	-1.50671	0.039559
IGSF3	-0.67185	6.52E-15
IGSF8	-0.32282	0.043385
IGSF9	-1.14305	5.94E-09
IK	0.170748	0.002963
IKBIP	0.241779	0.005652
IKBKAP	0.156159	0.008719
IKBKB	-0.24579	0.01276
IKBKE	0.26948	0.001779
IKZF2	-1.12333	1.76E-13
IKZF3	0.585794	4.35E-05
IL10RB	-0.3087	0.000116
IL11	2.827605	6.57E-33
IL11RA	-0.28797	0.03816
IL12A	1.207375	8.51E-06
IL12RB2	-1.81528	3.55E-27
IL13RA1	0.189253	0.044164
IL13RA2	4.359177	2.76E-16
IL15	-0.88284	8.59E-10
IL15RA	-0.383	0.000347
IL17RA	-0.21798	0.007993
IL17RC	-0.44464	6.86E-05
IL17RE	-0.77815	3.79E-15
IL18	-1.35068	3.78E-15
IL18BP	-0.54616	1.80E-06
IL1A	2.061664	8.75E-16
IL1B	2.962589	1.69E-30
IL1R1	-0.20743	0.029968
IL1R2	1.857306	9.32E-08
IL1RAPL1	1.608951	0.003889
IL1RL2	0.528415	0.001085
IL1RN	-1.92114	2.02E-11
IL20	0.839947	0.037753
IL20RB	-1.23128	1.10E-12
IL22RA1	-1.26611	7.81E-17

IL22RA2	-3.80222	2.02E-11
IL23A	1.856584	1.57E-15
IL24	3.150239	3.55E-49
IL27RA	0.98256	1.01E-13
IL31RA	0.486424	0.003165
IL34	-3.52823	1.87E-05
IL36RN	-2.32441	1.95E-27
IL4I1	2.106116	7.97E-10
IL4R	0.795813	5.28E-18
IL6	0.856679	0.02195
IL6R	0.416261	0.002644
IL6ST	0.54837	6.61E-08
IL7R	0.589893	0.00016
ILDR1	-0.87308	0.000793
ILF2	0.359665	9.03E-11
ILF3	0.200032	0.000416
ILK	0.30719	1.10E-05
ILKAP	0.255397	3.80E-05
IMMP2L	-0.53954	1.03E-06
IMMT	0.33951	3.13E-07
IMP4	0.341914	1.18E-05
IMPA1	0.263542	0.001587
IMPA2	-1.14162	3.18E-19
IMPACT	-0.38404	1.03E-12
IMPDH2	0.251816	0.000195
INAFM2	0.800648	1.24E-07
INCENP	0.412721	5.23E-06
INF2	0.28676	0.019505
ING2	0.249839	0.005097
ING4	-0.67102	1.26E-07
INHA	-1.96937	1.84E-05
INHBA	1.206337	8.78E-19
INHBA-AS1	1.473	1.13E-13
INHBE	-1.45273	0.000921
INIP	0.170004	0.009045
INMT	-0.88607	0.038973
INMT-MINI	4.144021	2.09E-05
INO80C	0.330729	3.79E-05
INO80D	0.229117	0.023147
INO80E	0.278096	3.65E-06
INPP4A	-0.48627	4.42E-11
INPP4B	0.73526	2.70E-13
INPP5B	-0.38925	0.00016
INPP5E	0.171207	0.022247
INPP5J	-0.61089	0.002289
INPPL1	-0.29175	9.78E-05
INSIG1	-0.43265	0.011383

INSR	-0.50384	3.04E-09
INSRR	3.70842	0.003477
INTS1	0.231825	0.043271
INTS11	0.2014	0.00508
INTS13	0.52546	3.40E-09
INTS4	0.267198	1.16E-06
INTS5	0.137759	0.016392
INTS6	0.148819	0.012756
INTS6-AS1	-0.45944	0.041193
INTS7	0.342934	8.95E-05
INTU	-0.5166	3.72E-11
IP6K3	-4.03932	1.12E-14
IPMK	-0.29949	0.009441
IPO11	0.208588	0.001372
IPO13	-0.20311	0.020137
IPO4	0.554403	1.97E-14
IPO5	0.589627	7.29E-21
IPO5P1	-0.88257	8.97E-05
IPO7	0.328741	1.52E-06
IPO8	0.32375	4.80E-06
IPO8P1	3.275136	0.034075
IPO9	0.324572	9.58E-09
IPP	-0.19403	0.010499
IQCB1	0.110159	0.045938
IQCD	0.584473	0.000289
IQCE	-0.864	5.43E-13
IQCJ-SCHIP	-0.65656	3.37E-18
IQGAP1	0.303465	3.23E-08
IQGAP3	0.358045	0.000389
IQSEC1	-0.45926	0.000141
IQSEC2	-0.84985	1.25E-10
IRAK1	0.684993	3.14E-13
IRAK1BP1	-0.44421	0.00263
IRAK2	0.832356	6.33E-09
IRAK3	-0.49829	1.01E-05
IREB2	-0.46554	1.15E-09
IRF1	-0.58006	2.32E-11
IRF2	-0.15304	0.043345
IRF2BP2	0.308724	0.005072
IRF3	0.135813	0.025222
IRF5	-0.41266	6.16E-05
IRS2	-0.31039	0.000585
IRX2	-1.14663	4.75E-21
IRX4	1.280799	0.0287
ISG20	0.952578	3.04E-05
ISG20L2	0.325051	9.84E-08
ISM2	-1.13703	0.001783

ISPD	-0.73629	8.42E-08
ISY1	0.179787	0.005792
ISY1-RAB43	-0.27615	0.001596
ITCH	0.566556	1.15E-13
ITFG1	-0.45924	4.80E-06
ITFG2	0.163247	0.046555
ITGA1	0.909196	1.92E-11
ITGA10	1.365317	4.94E-07
ITGA2	2.608117	2.21E-80
ITGA3	1.15179	9.38E-29
ITGA5	1.628376	7.29E-34
ITGA6	0.402049	3.48E-13
ITGA9	-1.31827	0.038266
ITGA9-AS1	-0.79926	0.000203
ITGAE	0.320616	0.002549
ITGAV	-0.55053	5.93E-07
ITGAX	1.167965	0.000684
ITGB1	0.749656	6.99E-18
ITGB1BP1	0.336711	5.82E-07
ITGB2	1.46724	6.60E-09
ITGB2-AS1	1.239207	0.000929
ITGB3BP	0.537377	7.11E-07
ITGB4	0.347837	0.006045
ITGB5	0.453901	0.000726
ITGB6	-1.07672	2.88E-18
ITGB7	-3.61533	0.013965
ITGB8	-1.89997	1.87E-23
ITGBL1	0.892337	0.00388
ITM2B	-0.67376	2.27E-11
ITPK1	0.386219	1.23E-07
ITPKA	1.28026	8.89E-05
ITPKB	0.720417	1.81E-08
ITPKC	-0.55491	1.25E-09
ITPR1	-0.31327	0.000768
ITPR2	0.279652	0.029532
ITPR3	0.506082	1.53E-10
ITPRIP	1.357412	1.13E-36
ITPRIPL1	0.978857	4.96E-09
ITSN1	-0.25337	0.000151
ITSN2	0.312519	1.40E-05
IVNS1ABP	-0.71083	1.34E-20
IWS1	0.197674	0.000362
JADE2	-0.51418	8.50E-12
JAG1	0.624631	8.50E-08
JAG2	-1.27169	7.67E-17
JAK1	-0.1522	0.004225
JAKMIP3	-1.23501	0.000672

JAM3	0.514469	0.002237
JARID2	0.186535	0.005167
JARID2-AS1	-2.31507	0.022025
JDP2	-0.60108	6.63E-14
JKAMP	-0.23734	0.00347
JMJD1C	0.2855	4.34E-07
JMJD6	0.566051	1.08E-12
JMJD7-PLA	-0.49455	0.002599
JMY	-0.58876	1.05E-17
JOSD1	0.112135	0.039311
JPH2	-1.10535	5.30E-21
JPX	-0.152	0.004628
JRKL	-0.21375	0.047272
JTB	0.157004	0.018629
JUN	0.633388	3.58E-05
JUP	-0.46072	1.63E-10
KANK1	-0.5533	6.03E-11
KANK4	-3.64282	1.53E-17
KANSL1	-0.13205	0.020733
KANSL1L	-0.43624	0.000114
KANTR	-0.31572	0.000815
KARS	0.247124	2.76E-10
KAT14	-0.6134	1.13E-07
KAT2A	0.151467	0.008006
KAT6A	0.197936	3.00E-05
KAT6B	-0.47943	5.09E-11
KAT7	0.263124	8.00E-05
KAT8	0.157796	0.039951
KATNB1	0.317998	0.000419
KAZALD1	-0.56573	0.003596
KAZN	-0.635	1.20E-12
KB-1460A1	-1.00489	0.013475
KB-1732A1	-1.55925	1.03E-09
KB-318B8.7	-0.7554	0.007635
KBTBD11	1.619848	1.15E-07
KBTBD2	0.420268	3.20E-08
KBTBD3	-0.85054	7.48E-10
KBTBD7	-0.68619	4.33E-14
KBTBD8	0.530523	0.000354
KC6	-0.69524	0.00562
KCMF1	0.271117	7.65E-07
KCND1	0.535914	0.028441
KCND2	-0.71043	0.002426
KCNE5	1.482247	0.004599
KCNG1	0.648753	0.004678
KCNG3	0.949934	0.040753
KCNJ12	2.14432	1.57E-10

KCNJ15	0.277096	0.002082
KCNK15	-3.49284	0.022497
KCNK15-AS	-1.27523	0.000675
KCNK2	2.440539	4.92E-24
KCNK6	0.503926	1.74E-08
KCNN4	-0.32859	0.000445
KCNQ1OT1	-0.14328	0.036522
KCNQ5	1.862616	7.97E-25
KCNQ5-IT1	3.742123	0.000107
KCNS2	0.590664	0.018176
KCP	-1.23205	1.69E-07
KCTD1	-0.83506	3.73E-17
KCTD10	0.148285	0.015975
KCTD11	-0.41677	0.000136
KCTD12	0.245369	0.017426
KCTD13	-0.2533	0.019264
KCTD14	0.98757	3.47E-17
KCTD15	-0.34972	0.003224
KCTD17	0.510944	0.001112
KCTD18	-0.5942	5.97E-18
KCTD2	-0.2081	0.000236
KCTD20	-0.31834	1.10E-05
KCTD21	-0.31787	0.000337
KCTD5	0.392981	4.25E-11
KDELC1	-0.30025	0.006649
KDELR3	0.281281	0.004795
KDM1A	-0.54058	4.98E-12
KDM3A	-0.29314	2.71E-06
KDM3B	-0.14599	0.001049
KDM4A	-0.36391	8.62E-12
KDM4B	-0.4098	0.000473
KDM5A	-0.13734	0.011086
KDM5B	-0.49711	5.49E-16
KDM6A	-0.53078	3.65E-06
KDM6B	0.393449	0.004561
KDM7A	0.445978	2.98E-05
KEAP1	-0.16579	0.031075
KHDC1L	0.735042	0.003426
KHDRBS1	0.297837	7.00E-06
KHNYN	-0.52147	1.19E-14
KHSRP	0.507902	2.15E-13
KHSRPP1	-0.92786	0.036748
KIAA0040	-0.6659	6.55E-13
KIAA0100	0.10272	0.046777
KIAA0232	-0.33865	2.57E-08
KIAA0319	-1.01266	9.32E-06
KIAA0319L	-0.46105	1.57E-08

KIAA0556	-0.5838	1.53E-12
KIAA0586	0.188401	0.010723
KIAA0753	0.228746	0.001239
KIAA0825	-0.41973	0.025592
KIAA0895	-0.81549	1.34E-14
KIAA0922	-0.32289	7.61E-07
KIAA0930	-0.55643	1.09E-13
KIAA1024L	-1.31452	0.007055
KIAA1109	-0.29191	0.001359
KIAA1143	-0.17944	0.01204
KIAA1161	-0.28592	0.001532
KIAA1211L	-1.23373	2.90E-22
KIAA1217	0.192899	0.046978
KIAA1324	-0.69463	0.001038
KIAA1324L	-0.20345	0.004529
KIAA1429	0.19187	0.000721
KIAA1522	-0.39333	8.31E-09
KIAA1524	0.913503	8.03E-25
KIAA1549	0.267448	0.001715
KIAA1549L	2.88902	1.79E-26
KIAA1551	0.555249	1.05E-10
KIAA1586	0.444816	3.81E-05
KIAA1614	-0.73302	1.16E-06
KIAA1755	2.109018	0.001177
KIAA2026	-0.15448	0.001428
KIDINS220	-0.20823	3.49E-05
KIF11	0.295629	0.032182
KIF12	-0.98161	0.001941
KIF13A	-0.59708	4.30E-12
KIF14	0.640392	9.06E-06
KIF15	0.53143	2.09E-05
KIF17	1.627845	1.25E-05
KIF18B	0.44011	3.37E-05
KIF18BP1	-2.43487	0.000458
KIF1A	2.683815	0.035369
KIF1BP	0.273921	4.49E-08
KIF20B	0.531271	6.66E-06
KIF21B	0.910544	0.007983
KIF24	0.450506	0.000385
KIF26B	-1.13341	3.11E-06
KIF28P	-1.26171	0.003413
KIF2A	0.25026	0.000533
KIF2C	0.654742	1.71E-10
KIF3B	-0.15658	0.000117
KIF3C	0.410657	0.000151
KIF4A	0.582879	6.18E-08
KIF5A	0.73624	0.048191

KIF5B	0.14888	0.000824
KIF9	0.282819	0.037183
KIF9-AS1	-0.42614	0.019127
KIFC1	0.576662	1.51E-05
KIFC2	-1.00031	2.91E-10
KIFC3	0.721961	9.03E-08
KIRREL	0.704829	1.95E-10
KIRREL-IT1	1.446368	0.031765
KISS1	3.084725	8.11E-10
KIT	-1.00759	0.0214
KITLG	-0.26651	0.03345
KLC2	0.681362	1.44E-10
KLC4	-0.55905	2.70E-10
KLF10	-0.34317	8.39E-05
KLF11	-0.77048	1.19E-12
KLF15	-1.20134	0.000994
KLF16	0.422305	0.000104
KLF4	-0.30549	0.013164
KLF5	-0.50972	8.96E-05
KLF6	0.493641	0.001116
KLF7	0.492767	2.94E-11
KLF7-IT1	1.349251	0.019552
KLF8	-0.91247	1.02E-16
KLF9	-2.20285	2.13E-45
KLHDC10	-0.17303	0.009513
KLHDC2	-0.39353	5.04E-08
KLHDC3	-0.46544	3.64E-09
KLHDC7B	-1.4527	0.020951
KLHDC8B	-1.52125	1.19E-44
KLHL10	-1.79249	0.041119
KLHL11	0.195539	0.030177
KLHL13	-1.1473	5.75E-12
KLHL15	0.286695	0.005563
KLHL18	0.208097	0.004107
KLHL2	0.197321	0.038003
KLHL21	0.307257	0.005462
KLHL22	-0.38051	2.76E-05
KLHL23	0.596546	8.50E-08
KLHL24	-1.0699	4.56E-82
KLHL28	-0.35521	0.001779
KLHL3	-0.88478	2.54E-05
KLHL30	-1.15076	0.028296
KLHL31	-0.83825	1.98E-05
KLHL36	-0.32507	0.000365
KLHL5	0.399022	0.000406
KLHL7	-0.21245	0.00242
KLHL8	0.146422	0.047317

KLHL9	-0.29862	0.000124
KLK13	2.648437	0.001354
KLK4	-0.93201	0.001015
KLK6	1.678073	0.002501
KLK8	-2.38025	0.031286
KLLN	-0.68785	0.003952
KLRD1	-0.43168	0.009832
KLRG2	-0.70601	0.004046
KMO	-0.7109	0.015064
KMT2B	0.326848	9.81E-06
KMT2C	-0.25007	0.000678
KMT2D	0.156276	0.027909
KMT2E	-0.17115	0.005426
KMT5A	0.358165	1.98E-12
KNL1	0.731964	2.67E-13
KNSTRN	0.515783	1.34E-08
KNTC1	0.537531	8.17E-10
KPNA1	0.149877	0.00137
KPNA2	0.423455	4.77E-06
KPNA2P1	4.236592	0.000124
KPNA2P3	-0.78055	0.001136
KPNA4	0.308927	1.46E-06
KPNB1	0.451373	6.39E-13
KRBA2	-0.44005	0.045187
KRBOX4	0.259283	0.000725
KRCC1	-0.63018	2.08E-06
KREMEN1	-0.93321	1.57E-20
KREMEN2	-1.10453	8.27E-06
KRI1	0.177363	0.007781
KRR1	0.198234	0.002341
KRT1	-5.38822	4.72E-05
KRT13	-5.61123	4.41E-17
KRT14	-1.82621	5.47E-13
KRT15	-2.46242	2.78E-36
KRT16	-1.84697	1.54E-08
KRT16P5	1.124797	0.000597
KRT16P6	0.771756	2.23E-05
KRT17	-2.44975	1.34E-13
KRT17P1	-1.8983	4.51E-10
KRT17P2	-1.85939	3.24E-08
KRT18	1.21092	9.58E-22
KRT18P3	-4.35	2.47E-05
KRT19	-1.16161	2.76E-19
KRT34	2.149157	2.40E-06
KRT4	-5.84124	2.11E-05
KRT5	-2.5595	2.14E-11
KRT6A	-2.72757	2.72E-11

KRT6C	-1.80736	0.001058
KRT7	-0.14964	0.024406
KRT7-AS	-0.38598	1.33E-05
KRT8	0.602002	8.97E-06
KRT80	-1.11196	8.11E-14
KRT87P	-1.08272	1.88E-07
KRT8P36	1.944826	0.036935
KRT8P46	-0.65217	0.000842
KRT9	-1.21797	1.93E-05
KRTDAP	-3.26647	0.001351
KTI12	0.305217	0.000792
KXD1	0.362511	3.01E-07
L1CAM	-1.22609	1.45E-54
L29074.3	-2.71895	0.029329
L2HGDH	0.22869	0.001077
L3HYPDH	-0.26337	0.011333
L3MBTL2	-0.18991	0.009413
L3MBTL3	-0.24917	0.01126
LA16c-358I	0.271088	0.037489
LA16c-360I	-1.6127	0.003812
LA16c-380I	-0.84863	6.91E-06
LA16c-431I	0.32624	0.04718
LACC1	0.433275	4.30E-05
LACE1	-0.27363	0.005059
LAD1	0.613905	3.42E-11
LAMA1	0.491881	1.71E-05
LAMA3	0.518852	0.0007
LAMA4	1.281603	1.22E-07
LAMA5	-0.96138	6.64E-08
LAMA5-AS1	-2.20302	0.024375
LAMB2	-1.33631	1.98E-17
LAMB3	1.252859	7.20E-14
LAMB4	1.841332	0.008196
LAMC1	-0.29741	0.000109
LAMC2	1.127456	4.00E-17
LAMP2	-0.31691	2.46E-09
LAMTOR4	-0.18763	0.001129
LANCL1	-0.48035	1.06E-12
LAPTM4A	-0.60875	7.93E-16
LAPTM5	3.660925	3.10E-18
LARGE1	-0.68686	0.00033
LARGE2	-1.39364	5.45E-11
LARP4	0.144152	0.010543
LARS	0.282682	0.000401
LAS1L	0.297056	4.47E-10
LASP1	0.340878	9.27E-08
LAT2	2.196076	3.80E-30

LATS1	0.146439	0.04503
LAYN	-0.6806	0.003241
LBH	-1.77348	8.12E-07
LBR	0.550169	4.55E-07
LCA10	-1.47061	0.001327
LCA5	-0.7416	1.32E-07
LCAT	-0.40108	0.004357
LCK	2.907435	1.00E-19
LCN15	1.478439	6.02E-09
LCOR	0.343761	2.92E-05
LCP1	1.299693	6.31E-18
LCTL	0.725236	0.036009
LDB1	-0.50367	7.49E-11
LDHA	0.305033	0.000294
LDHB	0.31571	1.23E-08
LDHD	-0.58687	0.000644
LDLR	0.617146	9.00E-06
LDLRAD2	-1.18611	0.017879
LDLRAP1	-0.17637	0.014239
LDOC1L	-0.71395	3.93E-16
LEF1	0.638448	0.003627
LEKR1	-0.63233	0.02178
LEMD1	1.739172	0.000136
LEMD3	0.189368	0.000196
LENEP	-3.60569	0.004387
LEPR	-0.29355	0.014886
LEPROT	-0.56118	1.93E-15
LETM2	0.828413	3.90E-08
LETMD1	-0.19842	0.005299
LEXM	-2.28545	0.014432
LFNG	-2.46807	2.69E-24
LGALS1	1.293137	4.53E-07
LGALS3	-0.48099	2.77E-13
LGALS3BP	-0.44842	0.003194
LGALS7	-1.9007	0.002192
LGALS7B	-1.19659	0.001297
LGALS8	0.292276	0.001723
LGI3	-3.20755	0.020115
LGMN	0.17877	0.02363
LGR4	-0.43311	1.81E-05
LGSN	-0.98747	0.035674
LHFP	-0.44329	2.16E-07
LHFPL2	-0.23732	0.009023
LHFPL5	-0.36264	0.033942
LHPP	-1.1152	6.46E-18
LHX1	2.515109	5.68E-29
LHX4	-0.44677	0.011974

LIF	2.950692	1.41E-20
LIFR	0.626386	0.006912
LIG1	0.543524	1.49E-10
LIG3	0.185558	0.001037
LIMA1	0.714976	9.65E-22
LIMCH1	-0.91929	1.22E-36
LIMD1	-0.16024	0.043789
LIMD2	1.480275	2.03E-10
LIMK2	-1.00697	6.43E-29
LIMS2	-0.46687	0.001172
LIN54	0.310208	2.82E-05
LIN7A	-1.72151	0.048946
LIN7B	-0.57652	0.010318
LIN9	0.633414	8.55E-10
LINC00114	-1.44423	0.005915
LINC00173	2.281092	0.006543
LINC00174	-0.21945	0.03735
LINC00205	-0.59038	1.35E-05
LINC00216	-0.68746	0.025529
LINC00221	-0.76801	0.01934
LINC00240	-0.50465	0.03105
LINC00265	-0.75758	2.15E-12
LINC00294	-1.00034	3.30E-34
LINC00310	0.767691	0.008288
LINC00319	-2.39968	0.001745
LINC00339	-0.35648	0.001824
LINC00342	-0.24972	0.006776
LINC00346	-0.82187	5.05E-18
LINC00460	1.030379	0.010806
LINC00467	0.473955	1.78E-06
LINC00472	0.592857	0.035999
LINC00491	-0.40508	0.003977
LINC00511	-1.86777	1.98E-27
LINC00520	3.122089	0.003919
LINC00525	-2.5899	3.19E-09
LINC00565	3.745941	0.009777
LINC00589	1.514302	0.035653
LINC00598	-0.67347	0.004009
LINC00607	-1.4325	0.004858
LINC00622	1.291611	0.023371
LINC00623	0.49621	0.000719
LINC00628	1.530468	0.000164
LINC00638	-0.8447	8.79E-05
LINC00640	-1.3703	0.041869
LINC00641	-0.31943	0.0123
LINC00648	-0.78561	7.18E-05
LINC00649	-0.75727	4.16E-15

LINC00652	-0.63734	0.008738
LINC00664	-2.14636	0.001165
LINC00665	-0.37278	0.014549
LINC00667	-0.71894	1.79E-08
LINC00678	-0.92088	0.000287
LINC00680	0.20267	0.005986
LINC00704	1.709123	3.74E-11
LINC00705	2.34497	6.03E-05
LINC00707	0.960704	3.06E-08
LINC00840	-1.66513	0.006388
LINC00847	-0.67289	1.57E-10
LINC00862	1.25922	1.45E-09
LINC00863	-0.38763	5.11E-05
LINC00885	-3.37701	5.00E-19
LINC00887	-1.72695	0.001297
LINC00893	-1.02137	0.002245
LINC00894	-1.02103	3.05E-07
LINC00898	-2.65048	0.006381
LINC00909	-0.36182	0.012651
LINC00910	-0.43192	0.000402
LINC00920	-0.52221	0.03039
LINC00923	-1.59489	0.001562
LINC00941	1.877437	1.77E-25
LINC00954	-1.51372	1.88E-06
LINC00959	-0.75383	0.002519
LINC00963	-0.44237	3.67E-07
LINC00964	-1.02555	3.91E-12
LINC00973	1.668806	0.011615
LINC00976	0.390086	0.028465
LINC00987	-0.93417	0.006393
LINC00998	-0.14655	0.024533
LINC01001	0.329229	0.005123
LINC01002	0.253495	0.000343
LINC01003	-0.56355	0.011388
LINC01011	-1.16751	9.20E-07
LINC01021	-0.8482	0.001095
LINC01060	1.357936	0.01358
LINC01067	-0.68331	0.028063
LINC01085	-3.38517	0.04018
LINC01096	0.828544	0.017104
LINC01106	-0.34984	0.005708
LINC01111	-1.85992	0.046721
LINC01116	0.374872	0.010968
LINC01119	-0.62672	0.018594
LINC01123	-0.73733	2.22E-10
LINC01127	1.76785	9.61E-07
LINC01128	0.307254	0.000139

LINC01133	-1.97448	0.000135
LINC01139	-0.76516	0.002099
LINC01140	-1.42451	9.24E-05
LINC01160	-0.59382	1.53E-07
LINC01204	1.207161	0.026405
LINC01213	-2.66983	1.78E-09
LINC01224	-0.6256	0.005309
LINC01232	-0.6166	1.12E-07
LINC01239	-0.84863	2.05E-06
LINC01247	-1.66124	0.026222
LINC01270	-0.37747	0.033941
LINC01271	-1.39525	0.003629
LINC01287	-0.97772	1.45E-05
LINC01293	1.892114	2.73E-20
LINC01315	-1.54632	0.038059
LINC01322	1.320781	3.38E-08
LINC01341	-1.89632	1.85E-06
LINC01355	-0.48016	0.004395
LINC01376	-0.7611	0.014362
LINC01391	-2.56613	0.000124
LINC01410	-0.58596	0.001041
LINC01422	-0.98882	0.002654
LINC01429	-1.60275	0.02111
LINC01435	-0.5797	0.041188
LINC01451	-2.29027	0.000433
LINC01460	-1.08187	0.033952
LINC01503	-0.36043	0.003911
LINC01515	0.324258	0.005766
LINC01518	-1.84503	0.049011
LINC01537	2.040963	1.57E-17
LINC01547	0.484973	0.036636
LINC01564	-2.48915	3.22E-11
LINC01605	1.830601	1.39E-17
LINC01615	-0.97598	2.46E-08
LINC01655	-3.69236	0.002586
LINC01679	-0.7586	0.000278
LINC01698	-1.12347	0.010247
LINC01719	-0.3225	0.007942
LINC01722	-1.08175	1.59E-10
LINC01730	-1.13614	0.027319
LINC01747	1.448405	0.00504
LINC01785	2.952468	0.001406
LINC01792	1.686449	0.02027
LINC01808	-2.25997	0.007588
LINC01812	3.331462	0.043192
LINC01819	-1.06353	3.78E-07
LINC01835	0.94578	1.75E-05

LINC01836	-1.00119	0.000737
LINC01842	-0.67178	0.040552
LINC01876	-0.66704	6.10E-05
LINC01881	0.30038	0.049733
LINC01922	-1.16549	0.008162
LINC01940	1.722026	0.042646
LINC01948	-3.47934	0.000477
LINC01963	-0.45984	0.016918
LINC01990	-1.24875	0.006257
LINC02001	-0.44086	0.009542
LINC02015	-1.66844	0.000393
LINC02021	-0.69874	0.041465
LINC02035	-0.64915	2.53E-09
LINC02043	-0.47232	0.039527
LINC02071	-0.95137	0.025916
LINC02081	0.597088	0.002868
LINC02100	-1.05868	0.003348
LINC02167	-0.81033	0.000543
LINC02177	-0.49999	0.043149
LINC02198	-2.61286	0.000419
LINC02210	-0.21078	0.003972
LINGO2	-2.73379	0.013879
LIPA	-0.39482	3.96E-08
LIPG	0.485738	5.44E-07
LIPM	2.630548	0.017704
LIPT2	-0.39191	0.040805
LITAF	-0.18133	0.035981
LIX1L	0.4497	4.24E-05
LL21NC02-	0.865795	0.012235
LL22NC03-	-0.31036	0.025809
LL22NC03-	0.262025	9.50E-05
LL22NC03-	-0.88021	2.20E-09
LLGL1	0.318583	6.84E-06
LLGL2	-0.28994	0.017624
LLNLF-173	0.404841	0.013108
LLNLR-284	-1.9026	0.037993
LMAN2L	-0.35775	0.000171
LMBR1	0.16819	0.007481
LMBRD1	-0.21198	0.0026
LMNB1	0.48131	0.001537
LMNB2	0.286129	0.000388
LMNTD1	-1.3023	0.002825
LMO2	0.988217	0.000342
LMO7	-0.96866	1.03E-11
LMO7-AS1	-0.74566	0.01855
LMOD1	-1.20999	0.009804
LMTK2	0.124937	0.047881

LNCSRLR	0.876316	0.000418
LNPEP	0.30185	0.002002
LNPK	0.229113	0.0187
LONRF1	-0.29246	0.002276
LONRF3	1.075026	4.77E-09
LOX	0.633827	1.05E-06
LOXL1-AS1	0.78816	0.015723
LOXL2	0.258667	0.004364
LOXL3	0.739955	1.53E-07
LPAR1	0.248331	0.002815
LPAR3	0.875062	7.07E-18
LPAR5	0.351686	0.005549
LPAR6	-1.07516	1.20E-12
LPCAT3	-0.2373	0.01236
LPCAT4	0.258093	0.009933
LPGAT1	0.243306	0.000272
LPP	-0.42715	6.91E-12
LPP-AS2	-0.52466	0.005629
LRCH1	0.478575	6.76E-24
LRCH3	0.331059	1.73E-05
LRCH4	0.352381	0.015159
LRFN3	0.485478	0.000259
LRFN4	0.496092	0.005532
LRG1	0.795936	1.22E-10
LRIG2	-0.23466	6.12E-05
LRP1	-0.80026	7.09E-16
LRP11	-1.29703	8.01E-21
LRP12	0.209306	0.002717
LRP1B	-1.71095	1.98E-16
LRP2	1.335836	0.018799
LRP4	-0.6514	1.77E-11
LRP5L	0.205824	0.034738
LRPAP1	-0.30636	0.002672
LRPPRC	0.211646	0.004172
LRR1	0.569681	1.72E-11
LRRC1	-0.13497	0.047078
LRRC2	0.736352	0.019218
LRRC20	0.24544	0.006874
LRRC23	-0.54248	3.05E-05
LRRC27	-0.70144	4.22E-08
LRRC28	-0.24155	0.0039
LRRC37A15	-0.62511	2.47E-05
LRRC37A16	-0.40443	9.50E-06
LRRC37A2	-0.17105	0.013776
LRRC37A3	-0.28784	0.011678
LRRC37A4F	0.197	0.011606
LRRC37B	-0.21564	0.007699

LRRC38	6.058543	2.06E-51
LRRC39	-0.73479	5.15E-05
LRRC4	0.990488	0.035934
LRRC40	0.324686	0.000481
LRRC45	0.284935	0.024033
LRRC49	0.372553	0.003022
LRRC59	0.483203	3.01E-15
LRRC66	1.519599	0.029602
LRRC7	-1.11822	2.97E-08
LRRC75A-A	-0.14984	0.000133
LRRC8A	0.919757	4.82E-13
LRRC8B	0.52192	6.19E-07
LRRC8C	2.244582	6.69E-71
LRRC8D	-0.34243	1.04E-10
LRRC8E	0.718166	9.19E-10
LRRFIP1	0.226125	0.00019
LRRFIP2	-0.38762	9.08E-14
LRRK1	0.717765	3.79E-11
LRRN4	1.413858	2.40E-05
LRSAM1	-0.29301	0.000196
LRWD1	0.630419	1.74E-07
LSM10	0.201015	0.043126
LSM12	0.254416	0.000172
LSM12P1	0.460157	0.00013
LSM2	0.311354	5.37E-05
LSM3	0.440464	1.94E-12
LSM4	0.368318	9.44E-08
LSM5	0.260161	0.006229
LSM6	0.564833	2.77E-08
LSM7	0.131187	0.047404
LSMEM1	-0.44921	0.016798
LSP1	-1.72846	6.64E-14
LSR	0.335719	0.000539
LSS	-0.56757	2.81E-16
LST1	1.189612	0.015162
LTA	2.440202	0.001359
LTB	2.54584	8.86E-34
LTB4R	-0.3008	0.004382
LTB4R2	-0.42718	0.005024
LTBP1	-0.83841	0.003691
LTBP4	0.525135	0.001366
LTBR	-0.18689	0.045854
LTF	-1.83333	0.049163
LTN1	0.255129	0.00272
LTV1	0.207519	0.007228
LUC7L	0.305757	0.000174
LUM	-1.21684	0.017067

LURAP1	1.084508	0.049412
LURAP1L	-0.73029	0.00502
LUZP1	0.178404	0.000117
LVCAT1	-0.37363	0.036421
LY6D	-3.51512	6.58E-19
LY6G5C	0.57691	0.009461
LY6G6C	-1.66689	0.011124
LY6K	0.314511	0.000511
LY75	-0.21246	0.047851
LY9	-1.66473	0.011749
LY96	1.584203	0.000162
LYAR	0.859829	3.74E-24
LYL1	1.011222	0.001104
LYN	-0.26232	0.001851
LYNX1	-2.57562	0.000119
LYPD3	-0.85743	7.79E-11
LYPD5	-0.54821	0.006154
LYPD6B	-1.39659	1.05E-07
LYPLA1	0.56012	3.39E-16
LYPLA2	0.464341	1.73E-07
LYPLAL1	-0.17852	0.018502
LYRM1	0.210612	0.006703
LYRM9	-1.1383	2.23E-05
LYSMD4	-0.47727	0.004091
LZIC	0.214359	0.000887
LZTFL1	-0.24709	0.03612
LZTR1	-0.19864	0.00727
LZTS2	-0.61967	1.79E-08
LZTS3	-0.84509	6.89E-07
M6PR	0.160672	0.001518
MACF1	0.564492	1.53E-11
MAD1L1	0.52556	2.33E-09
MAD2L1	0.729823	1.00E-10
MAD2L2	0.453744	1.46E-06
MAEA	0.185723	0.026577
MAFF	0.752023	6.09E-11
MAFG	0.275369	0.000307
MAFG-AS1	0.466504	0.046469
MAGEB17	3.198726	7.19E-25
MAGEC2	-0.47629	0.009221
MAGED1	-0.30796	1.30E-05
MAGEE1	0.436433	0.024691
MAGEF1	-0.3056	0.029011
MAGI3	0.242306	0.040821
MAGOH	0.362803	1.02E-06
MAGOHB	0.478178	3.22E-07
MAIP1	0.177374	0.035336

MAK16	0.342974	1.11E-06
MAL2	0.269472	0.002771
MALAT1	-0.31541	0.000752
MALL	0.909622	1.16E-26
MALSU1	0.327374	8.84E-07
MALT1	0.423869	1.93E-05
MAMDC2	0.354683	0.025122
MAML2	-0.53177	3.94E-10
MAML3	-0.47734	0.001954
MAN1A1	0.435208	0.009945
MAN1B1	-0.1559	0.020331
MAN2B1	-0.309	0.007145
MAN2B2	-0.44954	2.39E-06
MANBA	-0.89411	2.36E-20
MANEAL	-0.40274	1.72E-05
MANF	0.571183	1.36E-08
MAP1LC3B	0.256867	3.26E-05
MAP1S	0.993409	8.21E-09
MAP2	-3.24157	1.09E-20
MAP2K1	0.682493	2.93E-19
MAP2K3	1.191828	6.15E-35
MAP2K5	-0.40494	0.000147
MAP2K6	-1.20377	3.74E-17
MAP2K7	0.405099	2.51E-06
MAP3K1	-0.23684	0.003697
MAP3K11	0.204719	0.03752
MAP3K13	-0.19661	0.002084
MAP3K14	0.502783	0.000141
MAP3K14-/	-0.5169	0.000103
MAP3K2	-0.52127	1.51E-13
MAP3K20	0.202663	0.001043
MAP3K3	0.140974	0.043116
MAP3K4	0.230758	0.000228
MAP3K5	-0.28814	0.043734
MAP3K6	-0.21532	0.019203
MAP3K7	0.140105	0.008368
MAP3K7CL	0.491802	0.000598
MAP3K8	-0.65045	6.31E-05
MAP4	-0.32925	5.25E-14
MAP4K2	0.221236	0.019055
MAP4K4	0.957477	1.39E-23
MAP6D1	0.385383	0.047575
MAP7	0.157248	0.019034
MAP7D1	0.608187	6.55E-08
MAP7D3	0.301962	1.41E-05
MAPK10	-1.80479	5.60E-12
MAPK12	0.515157	0.000154

MAPK13	0.836451	2.12E-20
MAPK14	-0.22308	1.22E-05
MAPK1IP1I	0.167928	0.017579
MAPK3	-0.2717	4.95E-05
MAPK6	-0.48292	1.10E-13
MAPK7	0.214588	0.002483
MAPK8IP1	-0.34848	0.044062
MAPK8IP2	-0.66992	0.025939
MAPKAP1	0.224459	0.006671
MAPKAPK2	0.140514	0.016135
MAPKAPK3	0.584554	6.57E-09
MAPKBP1	0.878468	7.51E-14
MAPRE1	0.308726	8.95E-08
MAPRE3	-0.47782	0.000152
1-Mar	0.456861	3.19E-08
2-Mar	-0.15809	0.031328
1-Mar	-0.3171	0.036708
4-Mar	2.86847	1.30E-37
8-Mar	-0.42161	2.60E-06
9-Mar	0.383407	0.006091
MARCKS	-0.58547	8.22E-17
MARCKSL1	0.298328	4.69E-05
MARK1	-0.31367	0.007922
MARK2	0.141192	0.025652
MARK3	0.234033	8.01E-07
MARS2	0.34204	0.000135
MARVELD2	-0.24648	0.003074
MARVELD3	-0.26208	0.012695
MASP2	-0.62103	0.000943
MAST1	0.60779	0.010812
MAST3	-0.20987	0.049144
MAST4	0.50628	3.10E-05
MASTL	0.30354	0.011951
MATN1-AS	-0.80383	0.013529
MATN2	-2.52366	5.41E-21
MATR3	0.182434	0.010271
MAVS	-0.27046	1.16E-08
MAX	0.183975	0.003045
MAZ	-0.45395	4.50E-07
MB	-1.58899	2.50E-06
MB21D1	0.812603	4.31E-17
MB21D2	0.591273	1.09E-08
MBD2	-0.21315	6.28E-05
MBD3	0.289666	0.00141
MBD6	0.227557	0.002518
MBLAC1	0.505067	0.023384
MBLAC2	-0.50762	1.08E-07

MBNL1	0.179383	0.001718
MBNL1-AS:	-1.63074	2.53E-12
MBNL2	-1.03258	1.43E-23
MBNL3	-0.2608	0.005255
MBOAT1	0.536179	0.001143
MBOAT2	0.755274	1.46E-28
MBP	0.631205	1.67E-13
MBTD1	-0.37138	1.22E-05
MBTPS1	-0.20566	8.63E-05
MC1R	-0.21637	0.037653
MCAM	0.499782	0.00019
MCAT	0.308383	0.001769
MCC	-0.4352	5.56E-07
MCCC1	-0.33467	0.000236
MCCC1-AS:	-0.48819	0.015586
MCFD2	0.402067	6.99E-07
MCIDAS	0.39046	0.035063
MCL1	0.250307	0.016087
MCM10	0.843546	3.80E-20
MCM2	0.648608	1.98E-17
MCM3	0.480818	1.40E-09
MCM3AP	0.149622	0.033808
MCM4	0.542052	3.24E-11
MCM5	0.80244	2.17E-19
MCM6	0.326099	8.14E-06
MCM7	0.612547	5.68E-13
MCM8	0.691421	7.33E-25
MCM9	-0.27769	0.000698
MCMBP	0.422544	5.54E-09
MCMDC2	-0.71843	0.00078
MCPH1	0.377262	1.81E-08
MCPH1-AS	0.894025	0.004055
MCRIP1	0.352082	9.25E-05
MCRIP2	0.492599	0.003559
MCTP1	1.704646	4.96E-19
MCTS1	0.368072	7.62E-07
MCUB	0.68177	5.92E-12
MDC1	0.46726	1.24E-09
MDFI	1.052064	4.21E-11
MDFIC	-0.27742	0.000457
MDGA1	-1.99452	5.44E-07
MDH1	-0.09346	0.02036
MDM2	0.383318	0.005249
MDN1	0.149251	0.005798
MDS2	-1.12883	0.039912
ME1	-0.62361	1.60E-08
ME2	0.283907	0.000477

MEA1	0.298105	8.88E-07
MEAF6	0.477164	1.00E-14
MECOM	0.392795	0.01445
MECP2	-0.18415	0.005988
MECR	-0.19504	0.022177
MED1	0.35925	2.48E-07
MED10	0.270952	2.54E-05
MED11	-0.3039	0.000678
MED12L	-0.34883	0.002096
MED15	0.36624	7.22E-06
MED16	0.383626	0.001673
MED19	0.167773	0.049721
MED20	-0.26828	0.000406
MED27	0.233942	0.000278
MED28	-0.16009	0.034162
MED29	-0.12003	0.013392
MED30	0.237328	0.003888
MED31	0.224462	0.013606
MED4-AS1	1.688807	0.026934
MED6	0.223471	0.003778
MED8	0.432307	1.27E-09
MEDAG	2.677088	1.38E-05
MEF2C	-0.97933	0.000238
MEF2D	0.282579	0.010902
MEGF6	-1.44195	2.14E-06
MEGF8	-0.37163	0.000316
MEGF9	-0.72767	1.00E-08
MEI1	1.131793	0.002357
MEIS1	-0.543	5.26E-05
MELK	0.609963	1.54E-10
MELTF	-0.64138	4.66E-08
MEMO1	0.279515	0.003056
MEPCE	0.226263	0.0031
MERTK	0.374834	0.005858
MESDC1	1.019448	1.26E-10
MESDC2	0.150815	0.00372
MEST	-0.28233	0.001324
METAP1	0.20973	2.54E-05
METAP2	0.32134	1.44E-05
METTL14	-0.13496	0.046988
METTL16	0.103381	0.044017
METTL17	0.248804	0.001455
METTL26	0.323637	0.00067
METTL2A	0.255565	0.000121
METTL2B	0.354145	1.19E-07
METTL3	0.288082	0.000434
METTL7A	-2.6074	3.00E-31

METTL8	-0.22679	0.004643
METTL9	0.519789	8.72E-12
MEX3C	-0.24699	1.59E-05
MFAP3	-0.15692	0.02892
MFAP3L	-1.57307	6.21E-20
MFAP4	-0.97853	0.032161
MFAP5	-2.36401	9.57E-13
MFGE8	-0.37719	0.001308
MFHAS1	0.541208	8.09E-13
MFN2	0.13478	0.042136
MFNG	0.731582	1.06E-06
MFSD11	-0.10462	0.045731
MFSD14A	-0.16975	0.033297
MFSD14C	-0.24555	0.007131
MFSD6	-0.16961	0.000341
MGA	0.152784	0.002841
MGAM	-1.68086	1.82E-09
MGAT4C	-1.02346	0.018699
MGAT5	0.622421	3.51E-14
MGAT5B	0.912223	0.001102
MGC32805	2.472178	0.013212
MGEA5	-0.28683	0.007103
MGLL	1.103092	3.30E-13
MGME1	0.290572	1.86E-05
MIB1	-0.27552	0.000643
MICAL1	0.321987	0.006485
MICAL2	0.769284	2.38E-14
MICALCL	0.909444	2.27E-16
MICALL1	-0.24682	0.007179
MICALL2	-0.91937	6.47E-16
MICB	0.460215	0.000133
MICU2	0.337237	0.000139
MID1	-0.93067	5.21E-21
MIDN	0.861352	5.92E-08
MIEF1	0.187111	0.014099
MIEF2	-0.31922	0.000545
MIEN1	-0.17774	0.047148
MIF	0.548503	0.005878
MILR1	1.425784	9.21E-10
MINCR	-0.70483	0.002484
MINDY1	-0.22484	0.026558
MINDY2	-0.14516	0.017753
MINK1	-0.23959	0.008541
MINOS1	0.147046	0.034123
MINPP1	-0.12674	0.009609
MIPEP	-0.24957	0.000368
MIR100HG	0.748736	2.06E-08

MIR137HG	-1.77485	0.007499
MIR155HG	1.199238	0.022389
MIR17HG	0.371887	0.03191
MIR186	-0.81373	0.0035
MIR193BH(	-1.33761	1.75E-11
MIR205HG	-0.36884	0.00408
MIR210HG	-0.98056	0.000991
MIR2117H(	-0.8329	9.57E-07
MIR222HG	0.466194	0.008463
MIR29A	3.032333	0.046587
MIR30C2	3.27181	0.029597
MIR3138	0.738336	0.011121
MIR3142H(	2.124892	9.42E-07
MIR3176	1.192198	0.023423
MIR3180-4	-0.87889	0.038134
MIR31HG	2.442432	2.91E-32
MIR374B	-2.61998	0.012803
MIR3936	-1.53127	0.003216
MIR3945H(	-1.50132	0.038375
MIR4260	1.953994	0.044098
MIR4435-2	0.927196	6.76E-19
MIR4677	3.092532	0.031789
MIR4783	-3.18653	0.031636
MIR503HG	0.916162	0.009345
MIR589	2.216861	0.001204
MIR623	3.084576	0.016184
MIR635	-0.711	0.02297
MIR663A	3.075489	0.030411
MIR762HG	-0.57594	0.038806
MIR9-3HG	-2.1748	1.06E-07
MIR99AHG	-1.67713	1.31E-06
MIS12	0.34024	1.85E-05
MIS18A	0.567598	9.22E-16
MIS18BP1	0.378504	0.000195
MITF	-0.49355	0.002607
MKI67	0.655139	5.18E-08
MKL1	-0.38874	9.48E-07
MKL2	-0.1696	0.005519
MKNK1	-0.30017	1.47E-08
MKRN1	-0.22837	1.65E-07
MKRN2	0.254737	1.18E-05
MKRN2OS	-0.50217	0.001024
MKX	-0.69663	0.024651
MLEC	-0.16598	0.008924
MLF1	-0.31285	0.003707
MLH1	0.210257	0.000476
MLH3	0.180793	0.027374

MLKL	1.178763	1.22E-18
MLLT1	0.202134	0.030676
MLLT10	-0.21703	3.26E-05
MLLT3	-0.60416	6.32E-09
MLLT6	-0.23976	2.67E-05
MLPH	0.992793	3.22E-22
MLST8	0.245659	0.018874
MLX	0.430286	2.69E-14
MLYCD	-0.72881	2.18E-12
MMAA	-0.62999	1.49E-05
MMAB	-0.32959	4.59E-06
MMACHC	0.183665	0.008797
MMADHC	0.314519	4.17E-07
MMD	0.551825	1.29E-09
MME	0.741436	1.28E-07
MME-AS1	1.569106	0.010616
MMGT1	0.201095	0.001001
MMP1	5.755912	7.26E-19
MMP11	-0.67326	0.007839
MMP12	-1.22586	9.38E-07
MMP16	0.375716	0.001485
MMP19	0.867916	6.83E-07
MMP24	-1.06991	0.000739
MMP24-AS	-0.31931	0.000711
MMP25	-1.0394	0.000273
MMP28	-3.18183	3.40E-16
MMP3	4.077866	8.43E-50
MMP7	-2.19818	2.74E-05
MMP9	3.240497	9.67E-34
MMRN2	-0.98951	1.47E-07
MMS22L	0.522481	6.00E-17
MN1	-0.53857	0.000798
MNAT1	0.263204	9.43E-05
MND1	0.984274	4.91E-10
MNS1	0.657618	1.61E-07
MNX1	0.567018	0.001068
MOB1B	0.434886	1.77E-10
MOB3B	0.338028	4.99E-05
MOB3C	-0.26796	0.000527
MOB4	0.323788	0.000661
MOCOS	-0.23651	0.027487
MOCS1	0.229051	0.009342
MOCS2	0.147784	0.02761
MOGAT3	0.940954	0.013517
MOK	0.695588	5.06E-12
MON1A	0.587334	2.02E-08
MORC3	0.192295	0.00406

MORF4L1	-0.13438	0.011186
MORF4L2	-0.2079	0.001434
MORN1	-0.37888	0.002572
MORN3	-0.36256	0.030452
MOSPD3	-0.25446	0.030454
MOV10	-0.53445	4.98E-07
MOXD1	-0.286	0.025588
MPDU1	0.276192	0.001803
MPHOSPH1	0.195807	0.003169
MPHOSPH6	0.591129	4.26E-13
MPHOSPH9	0.330185	0.000147
MPO	5.542034	1.96E-06
MPP1	0.434565	9.74E-05
MPP5	-0.32113	2.01E-06
MPP6	0.251061	0.001116
MPP7	-0.82214	9.97E-14
MPPE1	-0.49879	2.37E-07
MPST	0.415908	0.000133
MPV17	0.153628	0.014091
MPZL1	-0.23405	3.24E-05
MPZL2	0.507912	0.000882
MPZL3	-0.21707	0.014408
MR1	-0.57627	1.07E-06
MRAS	-2.3996	2.56E-33
MRE11	0.234834	0.007548
MREG	0.485764	1.44E-05
MRFAP1	0.156046	0.000312
MRFAP1L1	-0.23349	0.000238
MRGBP	0.465322	1.17E-09
MRGPRX3	1.121775	1.76E-06
MRGPRX4	4.971443	8.70E-05
MRM2	0.245883	2.19E-07
MRM3	0.331567	1.62E-07
MROH2A	-4.02244	5.59E-08
MROH3P	1.408432	0.000635
MROH6	0.71057	3.53E-06
MROH8	-1.12248	1.14E-05
MRPL1	0.304678	5.29E-05
MRPL10	0.221543	0.000104
MRPL11	0.389845	1.53E-08
MRPL12	0.519832	1.86E-06
MRPL14	0.165412	0.00521
MRPL15	0.53091	4.92E-15
MRPL16	0.172893	0.001463
MRPL18	0.307945	2.35E-06
MRPL19	0.228	4.60E-07
MRPL2	0.161131	0.042721

MRPL20	0.376851	4.82E-08
MRPL21	0.343377	3.64E-08
MRPL22	0.183019	0.013048
MRPL23	0.793913	1.69E-11
MRPL23-AS	-1.51261	5.79E-06
MRPL27	0.124852	0.020665
MRPL28	0.319745	0.000107
MRPL3	0.336391	1.37E-06
MRPL30	0.280268	0.000558
MRPL32	0.169576	0.013589
MRPL33	0.233566	0.000121
MRPL35	0.234936	0.002252
MRPL36	0.336851	0.002196
MRPL37	0.401143	1.16E-08
MRPL38	0.252266	0.001731
MRPL39	0.466999	5.64E-09
MRPL4	0.409156	0.000195
MRPL41	0.426133	0.01854
MRPL42	0.267428	0.00238
MRPL45	0.251224	2.74E-06
MRPL47	0.310805	8.85E-07
MRPL48	0.273631	0.000881
MRPL52	0.350237	0.001067
MRPL57	0.33939	0.007888
MRPL58	0.345571	4.01E-05
MRPL9	0.444732	4.78E-18
MRPS10	0.193125	0.00234
MRPS11	0.196154	0.001829
MRPS12	0.266711	0.00192
MRPS15	0.400425	1.59E-10
MRPS18A	0.165573	0.022422
MRPS18B	0.264363	2.76E-07
MRPS2	0.509881	6.40E-10
MRPS21	-0.12173	0.017454
MRPS22	0.240102	3.64E-06
MRPS23	0.316452	1.13E-06
MRPS24	0.269308	0.005654
MRPS26	0.275109	0.038414
MRPS27	0.19469	0.00085
MRPS28	0.238291	7.56E-06
MRPS30	0.303715	5.18E-06
MRPS31	0.388985	9.03E-07
MRPS33	0.144747	0.029917
MRPS34	0.310066	0.014239
MRPS35	0.267246	2.40E-06
MRPS36	0.144102	0.048794
MRPS5	0.306398	7.00E-07

MRPS6	0.430691	5.16E-10
MRPS7	0.252265	2.14E-07
MRPS9	0.277183	4.76E-06
MRTO4	0.516383	2.25E-20
MRVI1	-2.32183	2.49E-17
MS4A10	-1.24741	0.020049
MS4A6A	-1.06339	0.021355
MSANTD1	-0.81588	0.035185
MSANTD2	0.237953	0.009991
MSANTD3	0.800788	8.03E-14
MSANTD3-	0.79753	0.000379
MSC	-1.34889	3.37E-14
MSC-AS1	-1.34697	9.08E-24
MSH2	0.482088	5.96E-10
MSH3	0.181844	0.007063
MSH5-SAP(	-1.26023	0.000773
MSH6	0.299411	4.75E-05
MSI2	-0.57844	3.59E-19
MSL2	0.153464	0.006091
MSLN	-1.45001	7.57E-09
MSLNL	-3.0944	0.033252
MSMO1	-0.308	0.009202
MSN	0.720419	2.36E-12
MSRA	0.814079	3.28E-10
MSRB1	-0.40175	3.85E-06
MSRB3	-0.33527	0.006569
MST1	-0.56781	0.000872
MST1L	-1.19661	0.012115
MST1R	1.102587	7.63E-28
MSTO1	0.230302	0.000208
MSTO2P	0.511294	0.000228
MSX1	1.637843	3.19E-12
MT1E	1.357971	5.29E-77
MT1F	0.384149	0.010856
MT1JP	1.514751	2.29E-08
MT1L	3.127735	2.48E-45
MT1M	1.181459	3.86E-17
MT1X	-0.51255	0.000488
MT2A	2.725396	7.87E-97
MTA1	0.229749	0.008735
MTA3	-0.24575	9.09E-07
MTAP	0.585916	8.75E-18
MT-ATP8	0.26621	0.027346
MTBP	0.496373	1.33E-05
MTCH1	0.109289	0.039391
MTCH2	0.128711	0.038071
MT-CO1	-0.20582	0.000944

MT-CO2	-0.20406	0.00443
MTDH	0.273402	1.92E-05
MTERF2	-0.54455	8.21E-08
MTERF3	0.362724	4.15E-08
MTF2	0.152323	0.039128
MTFP1	0.785359	4.40E-17
MTFR1L	-0.33871	6.79E-07
MTFR2	0.300784	0.024452
MTHFD1	0.416885	3.30E-11
MTHFD1P1	1.617939	0.006685
MTHFD2	0.253634	0.000586
MTHFD2L	0.193804	0.031751
MTHFS	0.22298	0.000549
MTIF2	0.120834	0.044272
MTMR11	0.600547	3.03E-08
MTMR12	0.25155	1.59E-06
MTMR14	0.135873	0.033891
MTMR3	-0.47137	1.56E-10
MTMR6	0.258789	6.87E-05
MTMR9	-0.26292	0.000165
MT-ND2	0.315029	0.003624
MTND2P28	0.292505	0.007394
MT-ND5	-0.33289	0.00251
MT-ND6	-0.35559	0.005546
MTO1	0.139001	0.016033
MTPN	0.22942	0.00022
MTRF1L	0.277519	2.61E-07
MT-RNR2	-0.72019	2.02E-07
MTRR	0.340223	2.87E-09
MTSS1L	-1.37775	1.78E-26
MT-TA	-0.41574	0.00843
MT-TC	-0.59113	0.000456
MT-TG	0.775992	7.91E-05
MT-TH	0.821596	0.005669
MT-TL2	0.495279	0.00339
MT-TN	-0.76769	2.73E-05
MT-TP	-0.68218	0.000134
MT-TY	-0.40507	0.014837
MTUS1	-1.82142	7.67E-19
MTX1P1	-0.87433	0.011737
MTX2	0.169415	0.003543
MUC1	-2.36164	2.26E-05
MUC15	1.496377	0.037872
MUC16	-1.73384	3.74E-06
MUC20	-0.61459	0.006705
MUC4	-1.34176	0.001768
MURC	0.934522	0.000758

MUS81	0.32375	6.87E-05
MUT	-0.12497	0.023127
MUTYH	0.405695	0.000731
MVK	-0.39702	2.18E-06
MVP	-0.43363	0.00032
MXD1	0.608407	6.98E-09
MXD3	-0.51547	0.005303
MXD4	-0.40467	0.0017
MXI1	-0.7128	5.27E-21
MXRA5	-2.54252	8.76E-18
MYBBP1A	0.27438	0.000297
MYBL1	0.55152	7.42E-06
MYBL2	0.486921	8.96E-12
MYBPHL	1.192348	0.03649
MYC	0.86588	7.95E-11
MYCBP	0.283353	0.000412
MYCL	-1.98275	2.66E-26
MYCT1	4.088605	0.000342
MYD88	-0.38207	0.000883
MYDGF	0.31155	0.000432
MYEF2	0.738809	2.36E-07
MYEOV	0.727952	0.00562
MYH10	0.307504	7.61E-05
MYH14	-1.08642	1.18E-11
MYH15	4.446055	7.83E-35
MYH16	3.670958	8.92E-19
MYH3	-0.69392	0.007084
MYH9	0.139018	0.010212
MYL12A	0.38385	5.23E-10
MYL12B	0.364322	2.63E-09
MYL5	-0.40536	0.001993
MYL6	-0.28705	5.52E-12
MYL6B	0.355366	0.000535
MYL7	-1.75512	1.43E-06
MYL9	-1.24224	2.43E-31
MYLIP	-0.45145	0.019035
MYLK4	-0.86803	0.001698
MYLK-AS1	-1.12791	3.03E-06
MYO10	1.325617	4.53E-50
MYO16	-1.34146	2.03E-05
MYO16-AS	-3.7335	4.46E-09
MYO18A	-0.20214	0.003161
MYO19	0.310563	2.64E-08
MYO1D	0.296837	0.000201
MYO1E	-0.28852	0.001458
MYO3B	-1.26432	0.018646
MYO5B	-0.55165	2.04E-05

MYO5C	0.526123	1.18E-05
MYO9A	-0.52275	1.87E-12
MYO9B	0.213267	0.002861
MYOF	-0.28156	0.000737
MYOM1	-1.18415	0.0006
MYOZ1	-1.39847	1.44E-10
MYPN	-2.24616	7.44E-05
MYRF	2.103687	2.24E-17
MYZAP	0.745523	2.84E-09
MZF1	-0.38752	0.000142
MZT1	0.371694	6.03E-05
MZT2A	0.486006	0.000354
MZT2B	0.543751	0.003931
N4BP2	0.369026	4.55E-05
N4BP2L1	-0.63638	0.000128
N4BP2L2	-0.25497	0.001222
N4BP3	1.490088	8.46E-16
NAA10	0.396904	1.42E-08
NAA15	0.453237	6.61E-09
NAA16	0.437463	2.65E-05
NAA25	0.161407	0.035532
NAA40	0.201012	0.008447
NAA50	0.304964	2.15E-07
NAAA	-0.45676	8.61E-06
NAALADL2	-1.39012	1.11E-06
NAB1	0.278317	0.000321
NAB2	0.243795	0.004413
NABP2	0.273456	0.000396
NACAD	0.8419	3.49E-07
NACC1	0.267057	0.002593
NACC2	-0.77497	5.39E-08
NADK2	-0.5738	4.29E-10
NADSYN1	-0.62563	9.01E-21
NAE1	0.347363	2.32E-07
NAF1	0.466784	2.58E-06
NAGK	0.153801	0.004029
NAGLU	-0.4296	0.000956
NAGS	1.053686	7.58E-15
NAIP	-0.46562	3.90E-05
NAMPT	0.250865	0.03253
NANOS1	-0.84113	1.24E-05
NANP	0.306956	0.002267
NANS	0.425822	1.44E-11
NAP1L1	0.485196	5.47E-07
NAP1L4	0.293128	3.63E-12
NAPB	-0.45273	6.10E-05
NAPEPLD	-0.30709	0.002694

NAPRT	0.296918	0.03376
NAPSA	-0.86075	0.042957
NARFL	-0.19593	0.025299
NARS2	0.220694	0.000318
NASP	0.390872	3.23E-05
NAT10	0.258831	9.73E-09
NAT14	-0.32698	0.002881
NATD1	-0.37069	0.003137
NAV1	0.823117	1.67E-10
NAV2	0.233205	0.005219
NAV3	2.654973	3.56E-50
NAXD	-0.28437	1.31E-05
NAXE	0.190388	0.005054
NBAS	0.174753	0.037949
NBDY	-0.21741	0.021572
NBEAL1	-0.26453	0.004667
NBL1	0.181942	0.012099
NBN	0.19355	0.027446
NBPF11	-0.13485	0.031488
NBPF12	-0.2095	0.014444
NBPF19	-0.38746	3.35E-10
NBPF20	0.448674	8.36E-06
NBPF26	-0.2329	0.001625
NBPF3	0.195881	0.003063
NBPF8	0.468872	3.28E-09
NBPF9	0.414082	8.22E-10
NBR2	-0.45442	3.20E-05
NCAPD2	0.321418	0.008269
NCAPD3	0.463392	2.97E-14
NCAPG	0.746329	3.18E-10
NCAPG2	0.534637	4.57E-06
NCAPH	0.685889	3.52E-17
NCAPH2	0.590049	9.94E-18
NCBP1	0.355538	1.29E-05
NCBP3	0.231021	1.96E-06
NCCRP1	-1.03077	0.008262
NCEH1	0.948703	9.91E-23
NCF1	0.849437	0.006864
NCF1B	1.398285	0.002958
NCK1	-0.31733	1.44E-06
NCK1-AS1	-0.50463	0.000304
NCKAP1L	2.251972	2.04E-08
NCKAP5L	-0.19114	0.015032
NCL	0.281445	2.24E-08
NCOA1	-0.38784	4.50E-13
NCOA2	-0.72561	6.85E-14
NCOA3	-0.54586	1.59E-17

NCOA5	0.122168	0.041161
NCOA7	-1.28321	1.86E-07
NCOR2	0.510608	0.000556
NCS1	-0.24439	0.003359
NCSTN	-0.31142	0.001125
NDC1	0.389636	2.63E-06
NDC80	0.492576	0.0001
NDE1	0.233283	0.003063
NDFIP1	-0.16401	0.003627
NDFIP2	-0.48185	8.62E-08
NDNF	-1.04659	0.006964
NDOR1	0.360841	6.64E-05
NDRG1	0.256774	0.010587
NDRG2	0.483827	9.46E-05
NDRG4	-0.31004	0.007943
NDST1	-0.80942	4.30E-23
NDST2	-0.27203	0.001113
NDUFA1	-0.20499	4.22E-05
NDUFA10	-0.12281	0.013522
NDUFA12	0.145394	0.00604
NDUFA2	-0.27413	0.001391
NDUFA4	0.22737	0.000366
NDUFA4L2	-0.8398	0.001594
NDUFA5	-0.20374	0.006996
NDUFA6	0.166321	0.018979
NDUFAB1	0.341888	5.15E-05
NDUFAF1	0.281733	0.00029
NDUFAF2	0.263709	0.000313
NDUFAF3	0.457267	0.000208
NDUFAF4	0.495653	9.33E-11
NDUFAF6	0.149115	0.043017
NDUFAF8	0.387028	0.000961
NDUFB1	-0.13215	0.045746
NDUFB11	0.151239	0.049825
NDUFB2-AS	-0.46914	0.012859
NDUFB8	0.153529	0.0029
NDUFB9	0.322003	1.41E-08
NDUFC1	0.298629	0.000513
NDUFC2	0.216333	0.014231
NDUFS2	0.16547	0.002938
NDUFS3	0.13298	0.041181
NDUFS6	0.330417	0.000167
NDUFS7	0.337363	0.034986
NDUFV2	0.149446	0.011673
NDUFV2P1	0.280511	0.012791
NEBL	-2.71727	1.84E-13
NECAB2	-2.05398	0.040511

NECAP1	0.265701	0.000534
NECAP2	0.227221	0.000533
NECTIN1	0.213682	0.043425
NECTIN2	-0.43422	9.16E-05
NECTIN3	0.133754	0.041626
NEDD4	0.50205	1.29E-11
NEDD4L	0.734337	7.10E-07
NEDD8	0.235273	0.000219
NEDD9	-1.15243	1.21E-19
NEIL1	-0.48066	0.004899
NEIL3	0.495752	0.001727
NEK11	-0.47036	1.28E-05
NEK2	0.318948	0.024926
NEK6	0.302604	0.000169
NEK7	0.291982	0.00011
NEK8	-0.17971	0.041463
NEK9	-0.40965	4.22E-09
NELFA	0.271987	0.003785
NELFB	0.290452	0.000116
NELFCD	0.131315	0.001132
NELFE	0.218762	0.002042
NELL2	1.116217	0.02403
NEMP1	0.484512	7.47E-09
NEMP2	0.702756	1.65E-12
NEO1	-0.34572	4.02E-11
NES	2.82452	3.57E-31
NET1	0.948305	2.21E-37
NETO2	0.691247	3.40E-27
NEU3	0.227884	0.017724
NEURL1	0.626585	7.74E-09
NEURL1B	-0.79139	7.31E-05
NEXN	-1.03882	8.12E-06
NF2	0.371255	6.60E-05
NFAM1	-2.36424	8.57E-05
NFASC	-1.84323	1.89E-18
NFAT5	0.230691	0.006883
NFATC1	1.428061	0.035703
NFE2	-1.23471	0.000957
NFE2L1	-0.61865	1.93E-05
NFE2L2	-0.18656	0.008835
NFE2L3	0.729391	8.89E-11
NFIA	-0.65106	1.65E-10
NFIB	-0.36951	1.54E-05
NFIL3	-0.70916	1.44E-10
NFKB1	0.349567	2.26E-06
NFKB2	0.71774	6.88E-11
NFKBIB	0.32929	0.001057

NFKBID	0.55346	0.000582
NFKBIE	0.886207	2.51E-10
NFKBIZ	0.978343	0.000196
NFRKB	0.224801	5.36E-08
NFS1	-0.18387	0.001678
NFU1	0.389101	3.32E-07
NFXL1	0.459201	1.98E-07
NFYA	0.29452	2.12E-05
NGDN	0.247247	0.000203
NGF	1.751672	1.61E-11
NGFR	0.865422	0.013058
NHEJ1	-0.20819	0.008092
NHLRC1	-0.33079	0.033377
NHLRC2	0.19813	0.000999
NHP2	0.181304	0.00291
NHS	-0.5395	0.000653
NHSL1	0.285651	0.016506
NHSL2	1.784227	6.23E-20
NICN1	-0.40268	0.020194
NIFK	0.269992	0.000117
NIN	0.194448	0.001841
NINJ1	-0.60883	5.81E-06
NIP7	0.442654	2.80E-08
NIPA1	0.368561	2.35E-06
NIPA2	0.304566	3.84E-07
NIPAL1	0.643111	6.91E-08
NIPAL2	-0.40398	0.002731
NIPAL3	-0.24076	0.016724
NIPSNAP1	-0.27589	0.000373
NIPSNAP3A	-0.76031	1.18E-16
NIPSNAP3E	-0.62409	0.040865
NISCH	-0.38588	4.95E-07
NKAIN1	-1.4351	0.042365
NKAPP1	-0.50234	0.009172
NKIRAS1	-0.26179	0.01196
NKIRAS2	-0.27425	0.000228
NKPD1	-1.92372	0.002768
NKRF	0.174858	0.007198
NKX1-2	-1.5071	3.53E-07
NKX2-5	0.654191	0.013069
NKX3-1	0.414239	0.001034
NKX6-1	0.571025	0.046265
NLE1	0.360667	2.91E-09
NLGN2	-0.62703	2.85E-11
NLGN3	-1.08229	0.002124
NLK	0.723813	2.23E-16
NLRC5	-0.64572	0.036328

NLRP1	-0.36934	0.000667
NLRP2	-1.06036	0.015083
NLRP3	2.351367	7.88E-55
NLRP3P1	0.693236	0.012218
NLRP7	0.603488	0.023244
NLRX1	-0.63308	9.24E-09
NMB	0.477406	0.000282
NMD3	0.204155	0.005692
NME1	0.562285	4.15E-15
NME2	0.237699	0.000433
NME3	-0.72872	0.000431
NME9	-0.54027	0.011151
NMI	-0.42399	0.000855
NMNAT1	-0.26102	0.018832
NMNAT2	0.542366	0.000186
NMRAL2P	-0.74371	0.000219
NMT1	0.127492	0.014777
NMT2	0.405067	6.70E-05
NMU	0.53116	0.011717
NMUR1	0.838238	0.003399
NNMT	-0.97522	9.30E-08
NNT	-0.19361	0.001375
NNT-AS1	0.165998	0.00937
NOB1	0.23613	9.87E-07
NOC2L	0.442441	6.00E-10
NOC3L	0.318704	3.68E-07
NOC4L	0.481417	2.51E-06
NOCT	0.544935	5.10E-05
NOD1	-0.26896	0.000239
NOG	1.07383	0.014124
NOL10	0.245525	1.01E-05
NOL11	0.35362	4.03E-08
NOL12	0.417008	0.000338
NOL3	-0.24525	0.023327
NOL4L	0.663883	2.77E-06
NOL6	0.432107	1.53E-09
NOL7	0.480992	1.16E-10
NOL9	0.454374	5.06E-09
NOLC1	0.431298	1.46E-10
NOM1	0.296749	1.55E-07
NONO	0.25603	1.19E-09
NONOP2	-0.59323	0.013559
NOP10	0.29689	8.62E-06
NOP14	0.451043	9.98E-12
NOP16	0.303485	2.02E-10
NOP2	0.311935	2.84E-08
NOP56	0.340455	6.86E-08

NOP58	0.392214	1.68E-06
NORAD	-0.22239	0.003705
NOS1AP	0.609625	0.001523
NOS2P3	1.161581	0.013409
NOS3	1.389257	1.38E-05
NOSIP	0.255786	0.010529
NOSTRIN	0.555405	0.037108
NOTCH2	-0.14515	0.00249
NOTCH3	-1.46767	5.74E-06
NOTCH4	1.752046	1.12E-07
NOXA1	-1.00688	0.003783
NOXRED1	-0.90451	0.000987
NPAT	0.337303	1.82E-05
NPBWR1	0.972184	0.032418
NPC1	0.725233	1.80E-12
NPC2	-0.16224	0.001985
NPDC1	0.375792	0.010082
NPEPL1	-0.32018	0.002294
NPIPB11	0.354262	0.017836
NPIPB2	-0.76063	0.013056
NPIPB9	-1.07237	0.041288
NPM1	0.466767	8.96E-11
NPM1P35	2.72092	0.037926
NPM1P9	1.338752	0.018311
NPM3	0.340944	1.30E-05
NPNT	-3.97463	9.37E-17
NPPA-AS1	0.638368	0.012103
NPPB	-1.60376	0.008473
NPR1	-1.60226	2.99E-32
NPR2	1.542801	5.62E-08
NPRL3	0.259399	0.008716
NPTN	-0.21364	4.93E-06
NPTN-IT1	-0.30527	0.026654
NPTX1	1.171707	4.27E-05
NPTXR	-1.38173	2.75E-08
NPY1R	-1.12451	2.33E-11
NPY4R	-1.62051	5.88E-11
NQO1	-0.43003	0.010073
NR1D1	0.857991	4.02E-14
NR1D2	-0.1863	0.014918
NR1H3	-0.5591	3.43E-08
NR1I2	2.01418	0.001738
NR2E3	0.984713	0.017566
NR2F2	-1.03317	4.18E-27
NR2F2-AS1	-1.32644	1.10E-08
NR2F6	0.350579	0.022271
NR3C1	-0.5228	6.05E-12

NR4A2	0.559231	8.08E-05
NR5A2	0.775246	8.44E-05
NR6A1	-0.34475	0.015732
NRARP	-0.61855	0.000421
NRAS	0.308174	0.000185
NRBP1	-0.4695	8.75E-15
NRBP2	-1.05891	1.09E-12
NRCAM	-0.52697	6.82E-05
NRDC	0.178912	0.004748
NRF1	0.301692	9.44E-06
NRG1	-0.87868	1.30E-06
NRG2	2.936066	0.00455
NRG4	-1.1353	5.42E-08
NRGN	1.578649	5.54E-18
NRIP1	1.155127	4.44E-10
NRIP3	2.18077	2.60E-29
NRP1	2.692851	2.57E-54
NRROS	2.659666	4.36E-13
NRSN2-AS1	-0.44696	0.000307
NSA2	0.21874	0.000216
NSD1	-0.36343	1.29E-11
NSD2	0.799683	1.20E-17
NSD3	-0.14384	0.048847
NSFL1C	0.175795	0.00319
NSFP1	6.362365	0.03578
NSG1	-3.06299	2.68E-21
NSL1	0.176853	0.010757
NSMCE4A	0.283103	1.74E-05
NSMF	0.857018	6.65E-14
NSRP1	0.217253	0.001478
NSUN2	0.436007	3.91E-15
NSUN5	0.328657	0.000351
NT5C	0.247386	0.012949
NT5C1B	0.483936	0.00651
NT5C2	-0.16591	0.0198
NT5C3B	0.344196	1.63E-05
NT5DC2	0.350806	0.049153
NT5DC3	0.440696	5.78E-06
NT5DC4	0.92189	0.002761
NT5E	3.198047	1.43E-61
NTAN1	0.529799	1.98E-14
NTF4	-0.80424	1.51E-10
NTMT1	0.549077	1.89E-13
NTN4	-1.67117	5.05E-10
NTN5	-2.00009	0.000346
NTNG1	1.983565	1.02E-09
NTPCR	-0.31169	3.77E-05

NTRK1	3.536991	0.000645
NTSR1	6.352081	1.85E-09
NUAK2	0.739637	2.15E-08
NUB1	-0.41686	1.39E-06
NUBPL	-0.23792	0.000471
NUCB1	-0.1369	0.014432
NUCB2	0.371316	0.002309
NUCKS1	0.236046	0.003393
NUDC	0.448319	1.06E-09
NUDCD1	0.569142	2.11E-16
NUDT1	0.374923	0.000325
NUDT12	-0.51336	4.20E-13
NUDT13	-0.26406	0.026233
NUDT14	-0.21283	0.0229
NUDT15	0.646724	3.09E-15
NUDT16	-0.43866	4.22E-09
NUDT18	-0.99675	3.10E-13
NUDT21	0.280737	0.001015
NUDT3	-0.27889	0.008112
NUDT4	0.242798	0.000455
NUDT4P2	0.26868	0.039304
NUDT7	-0.90109	1.38E-05
NUDT9	-0.2821	1.75E-06
NUF2	0.749824	1.96E-09
NUFIP1	0.216029	0.001958
NUMB	0.218789	1.70E-05
NUMBL	-0.29885	0.009929
NUP107	0.571946	5.13E-13
NUP153	0.28272	0.000309
NUP155	0.487921	6.29E-17
NUP188	0.42504	1.29E-16
NUP205	0.41498	1.04E-11
NUP210	0.558532	4.57E-08
NUP214	0.190435	0.000386
NUP35	0.2544	0.030791
NUP37	0.309562	1.39E-05
NUP43	0.243673	3.06E-05
NUP50	0.491852	1.32E-10
NUP54	0.360567	1.43E-06
NUP58	0.430629	2.82E-10
NUP62	0.308354	1.71E-05
NUP62CL	0.729585	3.38E-10
NUP85	0.386887	1.88E-09
NUP88	0.458205	9.89E-10
NUP93	0.431344	4.05E-14
NUP98	0.186894	9.11E-05
NUPR1	-3.33326	1.90E-22

NUS1	0.237532	0.000504
NUS1P1	2.69039	1.28E-05
NUSAP1	0.302372	0.023691
NUTF2	0.270938	3.31E-05
NUTM2B	-0.63083	0.001967
NUTM2D	-0.72378	2.20E-07
NXF1	0.163194	0.017919
NXN	-0.20194	0.002069
NXPE3	-0.27049	0.009544
NXT1	0.402871	9.57E-11
NYNRIN	-1.33809	2.02E-19
OAF	1.485417	7.52E-17
OAT	-0.23429	0.003558
OAZ1	0.174391	0.001947
OAZ3	-0.37578	0.038344
OBSCN	0.247953	0.049604
OBSL1	-0.73586	8.24E-13
OCIAD2	1.02932	7.43E-16
ODAM	-5.20158	8.47E-07
ODC1	1.339492	4.47E-22
ODF2	0.199999	0.008386
OFCC1	-1.03861	0.004955
OGDHL	-0.80839	7.69E-08
OGFOD2	-0.22775	0.044148
OGFOD3	-0.2595	0.000235
OGT	-0.23579	1.45E-05
OIP5	0.411738	0.000612
OLA1	0.358896	1.32E-08
OLFML2A	-2.79034	9.73E-23
OLFML3	-1.12508	7.07E-07
OLMALINC	1.491737	1.49E-16
OLR1	-4.6417	6.42E-37
OMA1	-0.26505	0.005656
OPLAH	-0.69128	6.16E-05
OPN3	-0.40877	8.20E-05
OPRD1	-0.73733	0.00956
OPRM1	1.261343	0.001433
OR10AE1P	1.461568	0.039071
OR10Y1P	-0.8788	0.034251
OR2A1-AS1	-0.30433	0.011209
OR2A7	-0.28492	0.01111
OR2A9P	1.100805	0.007012
OR7E38P	0.269984	0.012142
ORAI1	0.580846	2.41E-05
ORAI2	0.487855	8.04E-06
ORAI3	-0.49388	8.57E-06
ORAOV1	-0.20435	0.002908

ORC1	0.647853	7.19E-08
ORC2	0.298921	1.19E-08
ORC3	0.365378	1.01E-05
ORC5	0.247256	0.000149
ORC6	0.275636	2.88E-05
ORMDL1	0.131519	0.028196
ORMDL2	0.204522	0.007945
ORMDL3	-0.32601	3.30E-07
OS9	-0.31584	5.07E-09
OSBPL10	0.86852	1.27E-22
OSBPL1A	-1.40967	2.21E-41
OSBPL5	-1.10554	0.000161
OSBPL6	0.772247	4.52E-08
OSBPL7	-0.67151	0.003504
OSBPL8	0.297716	3.08E-05
OSCAR	0.897386	0.024415
OSCP1	-0.81174	1.99E-05
OSER1	-0.17217	0.015396
OSER1-AS1	-0.60938	0.000556
OSGEP	0.347772	3.50E-07
OSGEPL1	-0.36725	0.001644
OSGIN1	-0.54051	0.004896
OSMR	0.313616	0.036692
OSR1	-2.51827	4.38E-08
OSR2	-0.68619	5.45E-05
OST4	0.238245	0.001787
OTUB2	-0.3944	0.003814
OTUD1	-1.21517	4.47E-17
OTUD4	0.196079	0.000849
OTUD6B	0.262485	0.018888
OTUD7A	1.010718	0.038381
OVAAL	-4.3147	0.000125
OVCA2	0.396333	0.000247
OVCH2	-2.78092	2.77E-09
OVGP1	-0.69361	0.025051
OVOL2	-0.40264	0.004623
OXCT1	0.313847	4.93E-09
OXCT1-AS1	1.837223	0.004926
OXLD1	-0.60072	3.43E-06
OXNAD1	0.193174	0.021614
OXR1	-0.19391	0.014146
OXSM	-0.17657	0.044005
OXSR1	-0.24631	0.003493
P2RX5	1.267826	5.44E-16
P2RX6	-0.86246	0.006023
P2RY1	0.472896	0.000227
P2RY2	0.193596	0.005287

P3H3	0.423781	0.021305
P3H4	-0.23543	0.02251
P4HA2	-0.3331	0.00017
P4HTM	-1.06385	5.46E-21
PA2G4	0.446207	1.58E-07
PABPC1	0.189362	5.84E-07
PABPC4	0.24782	2.59E-09
PABPN1	0.221274	0.029547
PACSIN2	0.220887	0.002117
PACSIN3	-0.4487	9.43E-09
PADI2	-1.51755	0.000245
PAF1	0.260088	0.000748
PAFAH1B2	0.14355	0.006608
PAFAH1B3	0.219549	0.046082
PAFAH2	-0.36865	2.59E-06
PAG1	1.481345	5.92E-44
PAGR1	0.631222	1.49E-16
PAICS	0.436889	4.59E-08
PAIP1	0.154848	0.024547
PAIP2	0.217484	0.000571
PAIP2B	-0.54404	0.000134
PAK1IP1	0.46383	1.60E-11
PAK2	0.198689	0.000673
PAK4	0.272631	0.023334
PAK6	-0.17114	0.043909
PALB2	0.261569	0.004983
PALD1	0.61821	0.041595
PALLD	-1.8264	2.48E-39
PALM	-1.83599	2.71E-05
PALM2-AKA	1.360317	1.59E-09
PALM3	-1.29894	1.99E-08
PALMD	-1.24723	0.02239
PAM	-0.66	3.29E-09
PAM16	0.593223	1.80E-06
PAN2	-0.45122	0.003738
PANK1	-0.70997	3.86E-12
PANK2	0.303141	4.54E-05
PANK3	-0.16539	0.026104
PANK4	0.531202	2.90E-05
PANX1	0.16216	0.015373
PAPD7	0.22706	0.00021
PAPOLA	0.273309	3.85E-07
PAPPA	-0.45507	0.036682
PAQR3	0.369616	0.000148
PAQR5	0.316657	1.23E-06
PAQR6	-0.98167	0.00037
PAQR7	-0.53664	2.13E-05

PAQR9	1.973077	3.36E-05
PARD6A	0.90885	4.26E-05
PARD6B	0.934756	7.02E-13
PARD6G	-0.83346	5.99E-06
PARD6G-AS	-0.57494	0.047549
PARK7	0.104501	0.048292
PARL	0.139547	0.026226
PARM1	0.536923	0.036052
PARN	-0.09554	0.045972
PARP1	0.619747	7.11E-22
PARP12	-0.34292	0.011371
PARP2	0.481549	4.21E-10
PARP3	0.237296	0.000987
PARPBP	0.522509	4.86E-10
PART1	-0.85489	0.0243
PASK	0.210006	0.027298
PATJ	-0.30169	3.31E-06
PATL1	0.191887	0.020668
PATL2	-0.57241	0.00761
PAX6	1.218926	0.000166
PAX8	0.439091	0.000107
PAX8-AS1	0.455366	2.54E-07
PAXIP1	0.191037	0.001328
PAXIP1-AS1	-0.5621	0.00105
PAXIP1-AS2	-0.47433	5.94E-06
PBDC1	0.448391	1.47E-11
PBK	0.47801	7.38E-06
PBLD	-0.68935	8.62E-18
PBOV1	-3.19404	0.011469
PBRM1	0.205934	0.004809
PBX1	-1.13108	5.42E-13
PBX2	0.136025	0.031042
PBX3	0.714504	3.30E-22
PBXIP1	-1.20685	8.68E-32
PCAT14	-0.82227	1.10E-11
PCBD1	0.189503	0.021182
PCBP1	0.285549	7.62E-05
PCBP1-AS1	-0.31613	0.014752
PCBP2	0.203865	1.82E-05
PCCA-AS1	-1.90096	0.045698
PCDH1	0.924354	4.62E-28
PCDH18	-2.87752	1.00E-18
PCDH7	0.501438	0.001
PCDH9	-0.39888	0.025261
PCDHB10	-0.45878	0.018664
PCDHB11	-1.05711	0.00015
PCDHB13	-0.61593	4.19E-09

PCDHB14	-0.93609	3.89E-11
PCDHB15	-0.99049	4.70E-07
PCDHB2	-0.34518	0.001481
PCDHB4	-1.63613	0.017854
PCDHB5	-0.45447	0.012329
PCDHB8	-0.74731	4.76E-07
PCDHB9	-0.48771	0.000818
PCDHGA1	-1.94669	1.08E-05
PCDHGA10	-1.27886	6.87E-05
PCDHGA3	-1.70885	0.00958
PCDHGA4	-1.1087	1.19E-05
PCDHGA9	-1.32211	2.98E-10
PCDHGB1	-0.96732	2.24E-08
PCDHGB2	-1.10097	2.75E-12
PCDHGB3	-1.1181	0.001729
PCDHGB4	-0.31718	0.001766
PCDHGB7	-1.03981	6.25E-11
PCDHGC5	0.976676	0.017118
PCED1A	-0.59328	2.45E-07
PCF11	-0.13631	0.042582
PCGF2	0.43625	4.42E-08
PCGF5	-0.43652	2.19E-05
PCGF6	0.331219	6.99E-05
PCID2	0.170409	0.00128
PCLAF	0.597619	1.12E-05
PCMT1	0.366421	6.44E-15
PCMTD1	-0.57194	3.74E-14
PCMTD2	-0.50399	1.19E-11
PCNA	0.524842	2.09E-12
PCNP	0.257881	0.001019
PCNPP3	2.889854	0.00062
PCNT	0.314754	2.40E-05
PCNX1	-0.23707	0.006267
PCNX4	0.401951	1.37E-08
PCOLCE2	0.553501	1.06E-06
PCOTH	-1.27082	0.043111
PCSK6	-0.66534	0.004038
PCSK9	-0.76303	1.05E-22
PCYOX1	-0.72151	2.51E-27
PCYOX1L	-0.89067	4.82E-09
PCYT2	-0.28141	3.54E-06
PDAP1	0.33346	1.34E-07
PDCD10	0.300132	1.13E-05
PDCD11	0.331819	3.48E-12
PDCD1LG2	0.689326	0.032076
PDCD2	0.142299	0.007166
PDCD2L	0.369141	6.92E-05

PDCD4	-0.84618	1.66E-16
PDCD5	0.462731	7.07E-08
PDCD6	0.349688	6.55E-05
PDCD7	0.228319	0.010193
PDE12	0.355494	8.76E-07
PDE2A	3.235761	1.15E-10
PDE4B	-1.69173	0.003445
PDE5A	-1.01049	1.11E-19
PDE6A	-1.46629	6.07E-14
PDE6D	0.260874	0.004798
PDE7A	-0.31566	0.001041
PDE8A	0.243558	6.43E-05
PDE8B	-0.63138	2.84E-05
PDF	0.482277	0.001805
PDGFA	0.324508	0.020906
PDGFC	-0.30487	0.003626
PDGFD	-0.86455	1.10E-07
PDIA3	0.131575	0.045358
PDIA4	0.301698	0.000146
PDIA5	0.243213	0.000635
PDIA6	0.18517	0.008356
PDIK1L	-0.44038	7.28E-06
PDK1	0.401632	6.23E-08
PDK2	-0.60528	5.50E-10
PDLIM1	-0.62957	1.02E-13
PDLIM2	0.995701	1.52E-06
PDLIM4	0.958832	5.88E-12
PDLIM7	0.738608	7.62E-10
PDP1	0.607537	1.46E-08
PDPK2P	0.348837	0.007191
PDPN	0.924128	9.32E-07
PDRG1	0.144065	0.011917
PDS5A	0.288281	1.49E-05
PDS5B	0.299492	2.37E-05
PDSS1	0.732775	5.15E-09
PDX1	0.874396	3.39E-05
PDXDC2P	-0.31319	0.004246
PDXP	0.746263	0.00076
PDZD11	0.147431	0.010721
PDZD7	-0.34302	0.028049
PDZD8	0.123183	0.032536
PDZK1	-0.9096	0.000316
PDZK1IP1	-0.81387	0.012144
PEA15	0.569342	2.44E-14
PEAR1	0.994441	1.17E-17
PEBP1	-0.13135	0.006368
PECAM1	-0.5827	0.033183

PECR	-0.23239	0.030296
PEG10	-0.42811	0.007558
PELI1	0.740344	3.21E-09
PELI3	-0.35427	0.000352
PELO	0.359828	4.45E-07
PELP1	0.25342	1.09E-05
PEPD	-0.24411	0.008642
PER1	-0.99028	1.21E-16
PER2	0.421641	0.003259
PERP	-1.68853	4.72E-29
PES1	0.301372	9.29E-06
PET117	-0.33764	0.000546
PEX1	-0.24245	0.001346
PEX11A	-0.31833	0.000851
PEX11B	-0.43832	6.97E-10
PEX11G	-0.69484	0.001445
PEX2	-0.4142	1.75E-05
PEX26	0.108875	0.032157
PEX5	-0.22188	0.003015
PEX6	-0.29695	0.006915
PEX7	-0.36407	0.000488
PFAS	0.540748	2.00E-19
PFDN1	0.33625	1.26E-07
PFDN2	0.737634	8.34E-19
PFDN4	0.331929	0.000907
PFKFB2	-0.20253	0.007575
PFKFB3	0.592217	1.18E-07
PFKFB4	-0.7635	1.95E-07
PFKL	-0.22686	0.012023
PFKP	0.126753	0.04911
PFN1	0.899885	7.93E-15
PGAM1	0.251449	1.14E-06
PGAM1P5	-1.63396	0.000764
PGAM1P7	1.665521	0.024147
PGAM1P8	-0.64431	0.018524
PGAM5	0.538112	2.63E-21
PGAP1	-0.64431	3.34E-09
PGAP2	-0.21739	0.01071
PGAP3	-1.34338	4.07E-27
PGBD1	0.246644	0.029885
PGBD2	-0.24214	0.015577
PGF	1.892043	4.59E-18
PGGHG	-0.44588	0.009772
PGGT1B	-0.14667	0.027695
PGLS	-0.255	0.026769
PGM1	-0.20845	0.018768
PGM3	0.320871	3.36E-05

PGM5P2	0.513491	0.039017
PGP	0.419362	0.000394
PGPEP1	0.3954	0.002517
PGRMC1	0.326762	6.93E-10
PGRMC2	-0.79382	1.24E-21
PGS1	0.170814	0.021478
PHACTR2	0.352303	1.44E-05
PHACTR3	-1.44137	5.19E-09
PHACTR4	-0.31918	2.20E-06
PHB	0.318392	5.83E-08
PHB2	0.312576	1.85E-10
PHC1	0.371295	1.79E-05
PHC1P1	0.581798	0.001072
PHC2	0.873595	1.25E-39
PHEX	-0.87404	1.82E-05
PHF10	-0.18324	0.000189
PHF19	0.730354	1.08E-16
PHF2	-0.13034	0.017332
PHF20	-0.21095	0.000445
PHF20L1	0.148385	0.027113
PHF21A	-0.3449	6.33E-06
PHF5A	0.288125	0.000224
PHF6	0.26812	0.000284
PHF7	-0.308	0.031714
PHGDH	-0.48167	0.000138
PHKA2	-0.16374	0.043544
PHKB	-0.36947	1.34E-09
PHKG2	-0.1445	0.006712
PHLDA1	3.688564	6.21E-46
PHLDA2	1.926321	2.09E-09
PHLDA3	-0.60136	2.57E-07
PHLPP2	-0.33215	4.84E-05
PHOSPHO2	-0.35817	0.036497
PHPT1	-0.19415	0.010333
PHRF1	0.300496	0.001831
PHTF2	-0.26089	0.000223
PHYH	-0.7891	1.12E-11
PHYKPL	-0.36123	1.11E-05
PI15	-2.13074	6.71E-07
PI4K2A	0.226223	0.005878
PI4K2B	-0.26936	0.007042
PIAS1	-0.25941	0.001529
PIAS3	-0.27032	5.72E-05
PIBF1	0.295249	0.001186
PICALM	0.317746	3.89E-05
PIEZO1	-0.28591	0.021825
PIFO	-0.73456	4.80E-06

PIGB	-0.25355	0.000652
PIGC	-0.34441	0.012611
PIGCP1	-0.93988	4.86E-11
PIGF	-0.33551	7.75E-05
PIGH	0.268068	0.002985
PIGK	0.237202	0.003037
PIGN	-0.12054	0.021192
PIGO	-0.21115	0.01549
PIGS	-0.47483	1.01E-08
PIGT	-0.17279	0.031477
PIGV	-0.34065	9.60E-05
PIGZ	-1.20036	4.30E-13
PIH1D1	0.272971	0.000102
PIK3C2B	-1.23948	4.12E-45
PIK3C2G	-1.70418	0.000146
PIK3C3	-0.12631	0.03518
PIK3CB	0.226139	0.006759
PIK3CD	0.823697	6.61E-10
PIK3CD-AS:	-1.47765	6.13E-07
PIK3CG	1.132492	0.017881
PIK3IP1	-0.58863	2.19E-05
PIK3R1	-0.91314	1.32E-11
PIK3R3	-0.58035	0.000279
PIK3R4	0.136359	0.008385
PILRB	-0.56468	2.35E-05
PIM1	0.339319	0.001809
PIM3	-0.44247	0.006149
PIN4	-0.16101	0.037403
PINK1-AS	-0.30005	0.000595
PINLYP	-0.72033	0.000143
PINX1	0.544696	1.49E-10
PIP4K2A	0.474993	3.67E-09
PIP4K2B	-0.1005	0.014478
PIPOX	-0.74431	0.020096
PIR	-1.09002	9.39E-17
PITHD1	0.306525	7.65E-06
PITPNA	-0.10727	0.027785
PITPNB	0.293827	0.000794
PITPNM3	-1.86338	1.44E-17
PITRM1	0.160994	0.005666
PJA1	0.291693	0.000131
PKD2	-0.4777	2.82E-09
PKDREJ	-1.34417	0.000613
PKIA	0.602017	2.55E-07
PKIB	1.552857	1.29E-10
PKIG	0.265712	0.012655
PKM	0.281964	2.08E-06

PKMYT1	0.687455	4.77E-17
PKN1	1.107758	8.02E-14
PKN3	0.482905	1.51E-11
PKP1	-2.82337	7.38E-22
PKP2	0.633398	4.95E-11
PLA2G10	-1.38964	1.37E-06
PLA2G16	-0.70885	2.00E-11
PLA2G4A	-0.47892	0.000289
PLA2G4F	-0.50663	0.030769
PLA2G6	-0.58294	2.50E-07
PLA2G7	-1.48493	2.73E-06
PLA2R1	-1.04133	2.31E-46
PLAA	0.294217	4.53E-06
PLAC8	-0.82985	0.000687
PLAC9	-2.26912	0.012872
PLAG1	-0.45516	7.57E-09
PLAGL2	0.173132	0.013492
PLAU	1.476127	5.13E-18
PLAUR	1.967211	1.68E-25
PLB1	-1.26113	1.33E-09
PLCB1	-0.93139	4.56E-19
PLCB2	1.172397	1.14E-06
PLCB4	1.262897	4.48E-11
PLCD1	-1.87299	1.48E-09
PLCD3	-0.84504	4.84E-10
PLCD4	0.787235	4.82E-14
PLCG1	0.30722	1.14E-06
PLCG2	-0.33122	6.78E-05
PLCL1	-0.77378	0.012201
PLCL2	1.26943	0.006895
PLCXD1	0.839316	1.39E-10
PLCXD2	0.517661	3.38E-07
PLD1	-1.46574	9.33E-18
PLD2	-0.42782	1.10E-15
PLD3	-0.48799	5.41E-05
PLD6	0.243903	0.017877
PLEC	0.545013	0.001645
PLEK2	1.907375	1.94E-51
PLEKHA1	-0.36109	3.01E-05
PLEKHA2	-0.73998	2.23E-16
PLEKHA4	0.493803	0.00053
PLEKHA5	0.331661	5.63E-05
PLEKHA6	0.168026	0.041264
PLEKHA7	-1.91209	3.49E-06
PLEKHA8P1	-0.26096	0.031681
PLEKHB1	0.501297	0.04215
PLEKHB2	0.315999	0.001286

PLEKHF1	0.969869	6.97E-05
PLEKHF2	-0.4956	6.16E-12
PLEKHG2	1.061441	2.70E-18
PLEKHG3	0.739735	1.42E-15
PLEKHG4	0.479054	0.025529
PLEKHG4B	0.991508	0.0347
PLEKHG5	1.09604	3.67E-17
PLEKHG6	-0.88951	3.85E-08
PLEKHH1	-0.24147	0.047154
PLEKHH3	-0.73609	1.60E-07
PLEKHJ1	0.156553	0.035835
PLEKHM3	-0.39733	4.40E-08
PLEKHN1	0.414012	0.005125
PLEKHO2	0.435797	0.000164
PLEKHS1	-1.18531	0.043239
PLIN2	0.666354	1.68E-05
PLIN3	0.202428	0.024078
PLIN4	-1.36033	0.001256
PLK3	1.757434	3.47E-31
PLK4	0.671914	2.02E-08
PLOD2	0.608273	5.27E-09
PLOD3	0.381372	8.45E-07
PLP2	0.356563	4.69E-07
PLPP1	-0.65805	1.50E-08
PLPP2	0.355444	0.003019
PLPP3	-0.52028	1.99E-06
PLPP5	0.262504	0.002946
PLPP6	-0.61818	2.79E-06
PLRG1	0.151183	0.015016
PLS1	0.309523	0.000554
PLS3-AS1	-0.99678	4.91E-05
PLSCR3	0.295169	0.001125
PLSCR4	-1.25925	9.18E-17
PLTP	-0.70443	2.76E-14
PLXDC2	-1.81329	1.73E-16
PLXNA1	-0.23333	0.023371
PLXNA2	0.903995	3.06E-16
PLXNB1	-1.12759	4.41E-22
PLXNB2	-0.41867	5.01E-05
PLXND1	0.543309	1.45E-06
PM20D2	0.453783	3.50E-05
PMAIP1	0.792766	3.64E-07
PMEL	-0.58633	0.000927
PMF1	0.151368	0.001433
PMF1-BGL/	-0.67194	0.004137
PMFBP1	-1.3196	0.002011
PMP22	0.801441	0.000184

PMPCA	0.209426	0.000593
PMS2	-0.13195	0.012517
PMS2P6	-0.27676	0.037473
PMS2P7	-0.59447	0.003286
PNISR	-0.26928	0.02015
PNKP	0.637584	1.41E-15
PNMT	-1.4902	0.010578
PNN	0.373766	1.27E-05
PNO1	0.259275	8.56E-06
PNOC	-4.27428	0.000525
PNP	1.337463	1.21E-41
PNPLA2	-0.40972	0.005128
PNPLA4	-0.40064	0.000541
PNPT1	0.287565	0.007687
PNRC1	-0.44788	0.000357
PNRC2	0.205203	0.017343
POC1A	0.339425	0.000325
POC1B	0.433287	8.11E-06
POC5	0.278673	0.000832
PODNL1	-1.00203	6.12E-08
PODXL	2.465252	6.34E-51
POF1B	-0.95711	6.78E-19
POGK	0.181535	0.001384
POGZ	0.107525	0.042783
POLA1	0.634521	1.47E-10
POLA2	0.643482	2.62E-27
POLB	0.2917	0.000117
POLD1	0.621062	2.62E-11
POLD2	0.438457	1.90E-07
POLD3	0.287102	3.24E-05
POLE	0.610503	2.78E-11
POLE2	0.9218	2.27E-25
POLE3	0.300085	4.78E-09
POLE4	0.392139	0.000179
POLG	0.194319	0.000556
POLI	-0.57011	1.21E-07
POLL	-0.20108	0.010686
POLM	0.441145	3.79E-06
POLQ	0.554475	2.66E-06
POLR1B	0.31345	5.96E-14
POLR1C	0.176678	0.020177
POLR1E	0.74713	5.03E-33
POLR2A	0.312353	1.07E-07
POLR2C	0.258443	2.40E-07
POLR2D	0.2385	6.19E-05
POLR2F	0.236115	1.28E-05
POLR2H	0.223493	9.13E-06

POLR2I	0.532131	7.17E-08
POLR2J	0.212537	0.003548
POLR2J2	0.863004	1.91E-08
POLR2J3	-0.42665	5.89E-05
POLR2L	0.293245	0.003289
POLR3A	0.320201	4.30E-07
POLR3C	0.22254	0.000703
POLR3E	0.178377	0.014817
POLR3G	1.113819	6.46E-26
POLR3K	0.5493	8.90E-12
POLRMT	0.501461	0.00039
POM121	0.290234	7.88E-06
POM121B	0.844573	0.001332
POM121C	0.400487	1.00E-09
POMGNT2	0.618586	1.75E-10
POMP	0.433525	2.04E-07
POMT1	-0.19389	0.003147
POMT2	-0.31289	2.91E-06
POMZP3	0.300626	0.003133
PON2	-0.36119	5.82E-09
POP1	0.667435	2.60E-12
POP4	0.250462	0.0001
POP7	0.504589	4.52E-07
POPDC3	0.57936	0.000417
PORCN	-0.40666	5.41E-05
POSTN	-1.79486	1.86E-06
POT1-AS1	-0.80827	1.09E-06
POU2F2	3.86831	7.24E-35
POU2F3	-1.67619	7.27E-08
POU5F2	-0.5163	0.033497
POU6F1	-0.48083	0.000518
PPA2	-0.15792	0.043914
PPAN	0.521591	9.07E-07
PPARA	-0.32477	0.002612
PPARGC1B	-0.62456	9.99E-07
PPAT	0.312933	0.000317
PPCDC	0.585516	2.23E-09
PPCS	-0.30779	1.42E-06
PPDPF	0.3519	0.021729
PPFIBP1	0.133024	0.019933
PPIA	0.360238	9.50E-08
PPIAL4C	1.801232	0.001109
PPIAP22	1.002742	9.91E-08
PPIB	0.131402	0.026235
PPIC	-0.70923	6.90E-09
PPID	0.200094	0.001902
PPIF	0.579048	1.41E-06

PPIH	0.494991	2.38E-06
PPIL1	0.360316	5.38E-15
PPIL4	0.240741	0.000839
PPIP5K1	-0.24926	0.002531
PPL	-1.20634	7.57E-19
PPM1A	-0.19071	0.003063
PPM1B	-0.74281	3.99E-22
PPM1E	-0.66643	0.008443
PPM1F	0.481538	1.45E-08
PPM1G	0.4867	4.05E-17
PPM1H	-0.97512	6.14E-20
PPM1M	0.501739	0.000108
PPP1CA	0.24696	0.000572
PPP1CB	-0.26611	0.001781
PPP1CC	0.237697	4.97E-05
PPP1R11	0.165688	0.003537
PPP1R12A	0.159478	0.014012
PPP1R12B	-0.37605	0.007684
PPP1R13B	0.273652	0.000506
PPP1R14B	0.519677	3.98E-06
PPP1R14C	-0.83695	2.52E-06
PPP1R15A	0.917624	2.33E-14
PPP1R18	1.052358	3.45E-21
PPP1R1B	1.52779	0.007685
PPP1R21	-0.25563	2.38E-06
PPP1R26	0.34143	0.000591
PPP1R26-A	-0.36818	6.42E-05
PPP1R35	0.323743	0.036232
PPP1R3B	0.79877	1.03E-06
PPP1R3D	-0.44331	2.41E-07
PPP1R3E	-0.35729	0.025267
PPP1R3G	-1.38246	0.000284
PPP1R42	-2.32962	0.008248
PPP1R8	0.212726	0.000537
PPP2CA	0.180003	0.012326
PPP2CB	0.224015	0.000412
PPP2R1B	0.165898	0.002063
PPP2R2A	0.215874	3.10E-05
PPP2R2B	-2.32994	1.01E-17
PPP2R2D	0.245303	0.00015
PPP2R3A	-1.03561	6.11E-26
PPP2R3C	0.284741	0.002998
PPP2R5A	-0.16364	0.008551
PPP2R5C	-0.19194	5.85E-06
PPP3CA	0.189563	0.042428
PPP3CB	0.460713	1.23E-08
PPP3CB-AS	-0.4468	6.19E-07

PPP3CC	0.44113	0.00031
PPP3R1	0.365996	6.67E-07
PPP4C	0.238297	0.000583
PPP4R1	0.722257	2.05E-27
PPP4R2	0.187621	0.007983
PPP4R3A	0.130224	0.005104
PPP5C	0.236559	0.000188
PPP6R1	0.321675	0.00022
PPRC1	0.414756	1.81E-13
PQBP1	0.33738	1.51E-05
PQLC2	0.434331	6.65E-05
PQLC3	-0.82785	3.24E-21
PRADC1	0.511739	1.41E-08
PRAF2	-0.68486	3.64E-09
PRAG1	-0.37787	0.002825
PRAL	-1.95608	1.43E-07
PRAME	0.404352	5.24E-05
PRC1	0.482154	6.48E-06
PRC1-AS1	0.828286	0.023257
PRCD	0.659357	0.006283
PRCP	-0.56962	1.11E-14
PRDM1	2.339068	3.02E-33
PRDM10	0.364039	0.005387
PRDM11	-0.81926	1.04E-12
PRDM8	2.892078	6.29E-60
PRDM9	-0.97192	0.021573
PRDX3	0.279135	3.53E-06
PRDX5	-0.24151	0.000644
PREB	0.230463	0.003586
PRELID1	0.519546	3.61E-10
PRELID2P1	-2.92404	0.027626
PRELID3A	0.384599	0.001723
PRELID3B	0.253855	0.000515
PREP	-0.37434	1.36E-10
PREPL	-0.10211	0.026662
PRICKLE1	-0.57704	0.009694
PRICKLE2	-0.90326	3.11E-07
PRIM1	0.795123	1.89E-13
PRIM2	0.634696	4.14E-19
PRIMPOL	0.403202	5.06E-05
PRKAB2	-0.32183	4.51E-05
PRKACA	0.185296	0.002937
PRKAG2	0.793589	4.81E-14
PRKAG3	-1.35017	0.033036
PRKAR1A	0.372604	4.07E-10
PRKAR2A	0.337036	6.93E-08
PRKAR2A-A	-0.61424	0.001705

PRKAR2B	0.913624	4.66E-08
PRKCD	-0.31676	3.61E-06
PRKCH	0.191941	0.021751
PRKCI	-0.16863	0.016733
PRKD3	0.314188	3.19E-09
PRKDC	0.268167	3.72E-06
PRKG2	-0.75485	2.67E-05
PRKXP1	-1.21059	0.000646
PRMT1	0.280465	0.000102
PRMT5	0.533897	5.02E-17
PRMT6	-0.36084	0.000284
PRMT7	0.146374	0.010144
PRNP	0.17556	0.016377
PROB1	0.496115	0.000316
PROC	-2.02731	7.46E-33
PROCR	0.418717	1.36E-06
PRODH	-4.33963	6.06E-17
PROM2	-1.51758	1.48E-21
PROS1	-0.83219	1.12E-08
PROSC	0.416917	5.28E-13
PROSER1	0.202888	0.000197
PRPF19	0.202095	0.010035
PRPF3	0.351922	4.35E-06
PRPF38A	0.313469	0.001744
PRPF38B	0.203072	5.09E-05
PRPF4	0.377519	8.22E-10
PRPF40A	0.147755	0.026155
PRPF6	0.122174	0.018055
PRPF8	0.350621	4.69E-10
PRPS1	0.407416	7.90E-09
PRPS1P2	0.310704	0.021197
PRPS2	0.513592	2.20E-13
PRR11	0.744472	2.35E-10
PRR13	0.343783	1.79E-05
PRR14	0.261585	0.008536
PRR14L	-0.24096	0.000246
PRR15L	-3.99169	1.69E-08
PRR33	-3.09394	1.75E-08
PRR36	1.543477	0.000671
PRR7	1.262735	9.53E-06
PRR7-AS1	0.952194	0.000146
PRRC2A	0.190504	0.001428
PRRC2C	0.16654	0.003233
PRRG2	-0.24093	0.02829
PRRG4	-1.1653	1.12E-13
PRRT1	-0.6459	0.042009
PRRT2	-0.99252	0.000529

PRRT4	-1.12881	0.027641
PRSS12	-0.31221	0.030571
PRSS16	-0.46754	6.64E-07
PRSS23	0.275749	0.000349
PRSS3	4.616999	4.15E-22
PRSS56	-1.82488	0.009205
PRSS8	-0.82085	2.87E-09
PRUNE1	-0.19366	0.00218
PRX	-0.64453	1.82E-05
PSAP	-0.52269	7.79E-09
PSAPL1	-3.46999	6.54E-05
PSCA	-1.82686	4.95E-06
PSEN1	-0.23037	0.000903
PSG5	-3.06394	7.92E-05
PSG9	-1.66074	0.00034
PSIP1	0.305279	0.00173
PSKH1	-0.47676	2.50E-08
PSMA1	0.3229	5.47E-07
PSMA2	0.185981	0.003821
PSMA5	0.150118	0.022043
PSMA6	0.123742	0.043085
PSMA7	0.385852	1.50E-09
PSMB1	0.132852	0.020855
PSMB10	-0.67797	2.61E-07
PSMB2	0.331072	2.74E-09
PSMB3	0.257999	3.00E-06
PSMB4	0.128474	0.019783
PSMB6	0.150655	0.004052
PSMB7	0.340912	7.77E-06
PSMB8	-0.27532	0.013563
PSMB8-AS1	-0.85015	1.15E-06
PSMB9	-0.34631	0.000785
PSMC1	0.305466	7.64E-06
PSMC2	0.198451	0.000389
PSMC3	0.267271	2.96E-05
PSMC3IP	1.022459	1.05E-22
PSMC4	0.294251	4.47E-07
PSMC5	0.268395	3.56E-07
PSMC6	0.222922	0.000969
PSMD1	0.21467	0.000263
PSMD10	-0.20248	0.000507
PSMD11	0.326551	3.37E-08
PSMD12	0.326729	5.11E-05
PSMD13	0.386361	3.08E-14
PSMD14	0.39379	5.81E-07
PSMD2	0.929627	4.11E-26
PSMD3	0.420117	2.28E-10

PSMD4	0.299985	1.05E-06
PSMD5-AS1	-0.607	1.33E-12
PSMD6	0.174736	0.002555
PSMD8	0.312733	9.03E-06
PSMD9	0.161278	0.002716
PSME1	-0.48344	3.42E-07
PSME3	0.360758	2.43E-08
PSMF1	0.123674	0.035275
PSMG1	0.219637	0.010528
PSMG3-AS1	-0.7394	0.005658
PSPC1	0.26676	0.048566
PSPH	-0.23793	0.017549
PSTPIP1	0.81648	0.034848
PSTPIP2	-0.34162	0.00018
PTAFR	0.798351	6.25E-12
PTAR1	0.198529	0.009439
PTBP1	0.517777	2.37E-16
PTCH1	-0.5918	6.14E-07
PTDSS1	0.344021	1.74E-15
PTEN	-0.14512	0.005554
PTENP1	0.873213	0.038389
PTER	-0.22877	0.019861
PTGER4	0.705437	2.53E-05
PTGES	-2.25633	4.30E-14
PTGES2	0.217293	0.01489
PTGES3	0.363378	1.12E-06
PTGES3L	0.368132	0.035093
PTGES3L-A	-0.8696	0.002281
PTGES3P1	-2.6343	0.005008
PTGFRN	-0.23993	0.002625
PTGIR	2.348169	5.93E-07
PTGR1	-0.89161	1.56E-10
PTGS2	1.107001	1.38E-05
PTHLH	2.084063	8.59E-08
PTK2B	0.465838	8.80E-10
PTK6	1.138376	3.35E-09
PTK7	-0.31474	7.72E-06
PTMA	0.415875	9.64E-06
PTMAP5	1.126752	0.001004
PTN	0.942525	6.76E-07
PTOV1	-0.30278	1.90E-05
PTOV1-AS1	-0.3797	6.22E-05
PTOV1-AS2	0.399018	0.038885
PTP4A2	0.322302	1.10E-07
PTP4A3	0.474782	0.036022
PTPN1	0.496227	2.07E-12
PTPN11	0.295465	1.04E-05

PTPN12	0.711749	6.15E-15
PTPN13	-1.03937	1.78E-17
PTPN14	-0.17359	0.000458
PTPN18	-0.36076	0.000252
PTPN2	0.267077	0.000112
PTPN3	-0.24489	0.000102
PTPN4	-0.4189	1.81E-08
PTPN7	0.846049	0.041726
PTPN9	0.33738	2.39E-08
PTPRA	-0.18011	0.001269
PTPRB	0.352041	0.003981
PTPRE	0.6778	2.50E-08
PTPRG	0.617758	0.000397
PTPRG-AS1	0.697476	1.61E-10
PTPRJ	-0.61178	4.16E-06
PTPRM	-1.05463	9.05E-11
PTPRQ	-1.83406	8.76E-06
PTPRS	-1.08956	1.19E-09
PTPRU	-1.99907	8.85E-20
PTPRZ1	1.528833	2.62E-06
PTRH2	0.175357	0.017251
PTTG1	0.517929	0.000136
PTTG1IP	0.199673	0.00052
PTX3	0.866609	3.91E-06
PUF60	0.22937	0.002803
PUM3	0.36541	1.24E-09
PUS1	0.201321	0.00099
PUS10	-0.3019	0.004555
PUS3	0.257582	0.000329
PUSL1	0.46834	0.000114
PVR	0.335675	0.006219
PVRIG	-0.4907	0.028353
PVT1	0.175115	0.0097
PVT1_1	1.258288	0.042478
PWP1	0.333896	7.36E-09
PWWP2A	0.226803	0.018488
PXDC1	-0.68247	8.98E-12
PXDNL	-0.77392	0.035144
PXMP4	-0.71024	1.44E-08
PXN	0.692693	8.30E-22
PYCARD-AS	-1.65065	0.021412
PYCR2	0.136075	0.030686
PYGB	0.29208	0.000511
PYGL	-0.2084	8.52E-05
PYGO2	-0.33644	0.001289
PYROXD2	-0.65013	3.51E-09
PYY	1.260281	0.040128

PYY2	-0.76811	1.75E-09
QDPR	0.33212	2.57E-05
QKI	0.364669	2.87E-08
QPCT	0.714965	7.92E-08
QPRT	-1.18656	4.96E-25
QRICH2	-0.90348	0.000959
QSOX1	0.903082	1.97E-19
QSOX2	0.227512	0.001343
R3HCC1	0.1849	0.009621
R3HDM1	0.254388	0.000128
R3HDM2	-0.28239	0.021017
R3HDM4	0.311379	0.000138
RAB10	-0.11816	0.042482
RAB11B	0.340955	0.005048
RAB11FIP1	-0.26645	0.02282
RAB11FIP1	-1.07365	2.68E-09
RAB11FIP4	-0.69478	1.46E-20
RAB13	0.338512	0.001333
RAB14	0.103035	0.035998
RAB17	0.527162	7.17E-07
RAB1A	0.179632	0.011327
RAB1B	0.171251	0.003359
RAB20	-0.60056	2.33E-10
RAB24	-0.30576	0.003647
RAB27A	0.348118	2.54E-05
RAB27B	-1.01473	1.02E-16
RAB30	-1.00237	1.09E-12
RAB31	0.696557	1.62E-10
RAB32	-0.72777	2.87E-13
RAB33B	-0.29246	0.001759
RAB34	0.501105	2.27E-11
RAB35	0.317119	1.06E-06
RAB3B	2.384973	1.65E-33
RAB3C	-2.79637	0.047285
RAB3D	-0.58287	1.22E-16
RAB3GAP1	-0.13893	0.007188
RAB40A	-0.49892	0.006631
RAB40B	-0.64188	0.000113
RAB40C	-0.30299	0.003916
RAB41	-0.64481	0.043465
RAB4B	-0.25127	0.048752
RAB5B	-0.35995	8.17E-10
RAB5C	0.225809	0.000223
RAB6A	0.13348	0.019631
RAB7A	0.285847	0.000124
RAB7B	-2.15643	6.76E-07
RAB8A	0.359574	4.93E-10

RAB8B	0.356244	3.34E-06
RAB9A	-0.40738	2.43E-05
RABEPK	0.162583	0.043735
RABGAP1	-0.30559	2.50E-06
RABGEF1	0.269045	1.25E-05
RABL2A	-0.59441	4.40E-05
RABL2B	-0.36655	3.89E-06
RABL3	-0.23694	6.69E-05
RABL6	0.231626	0.000852
RAC1	0.360984	4.37E-13
RAC2	1.233507	1.61E-21
RAC3	-0.26082	0.010452
RACGAP1	0.319388	0.003924
RACK1	0.277557	7.45E-07
RAD1	0.311484	5.95E-07
RAD17	-0.19279	0.011668
RAD18	0.786403	6.82E-16
RAD21	0.266343	0.000287
RAD21-AS1	-0.49418	0.027701
RAD23A	0.176176	0.018564
RAD23B	0.249549	0.000338
RAD51	0.638905	1.70E-15
RAD51AP1	0.502704	2.91E-07
RAD51-AS1	-0.80433	2.06E-08
RAD51B	0.316113	0.017718
RAD51C	0.36376	7.08E-05
RAD54B	0.788429	8.72E-12
RAD54L	0.82301	3.55E-17
RAD9A	0.166451	0.012147
RAE1	0.251412	0.000654
RAET1E	-0.76168	4.78E-06
RAET1G	-1.20635	4.93E-12
RAET1K	0.74392	0.033013
RAET1L	-1.8445	3.22E-06
RAI14	1.203132	8.37E-26
RALB	0.635761	4.54E-22
RALGAPA1	0.615296	0.044337
RALGAPA2	-0.29034	0.00781
RALGDS	-0.46976	5.93E-06
RALGPS1	-0.99104	6.06E-12
RALGPS2	-0.38539	0.001838
RALY	0.43025	4.29E-10
RAMP2	2.815561	6.88E-14
RAMP2-AS	0.394779	0.042869
RAN	0.453566	9.87E-27
RANBP1	0.76988	2.50E-26
RANBP10	0.135192	0.035397

RANBP3	0.206386	0.001588
RANBP9	0.283923	0.00646
RANGAP1	0.663302	2.97E-22
RAP1B	0.469446	2.50E-11
RAP1GAP	-0.69503	8.11E-06
RAP1GAP2	0.41615	6.95E-10
RAP1GDS1	0.340133	2.54E-11
RAP2B	0.799114	1.29E-13
RAP2C	0.253851	0.001091
RAP2C-AS1	-0.48387	0.002188
RAPGEF1	-0.48652	2.57E-11
RAPGEF2	-0.1816	0.008097
RAPGEF3	2.988129	1.06E-36
RAPGEF4	0.679088	0.004522
RAPGEF6	0.28604	0.000135
RAPGEFL1	-1.69535	1.47E-19
RAPH1	0.512863	1.28E-08
RARA	0.27863	0.026498
RARB	-1.03728	0.000406
RARG	-1.26832	3.13E-20
RARRES1	-0.56874	0.011464
RARRES3	-2.36233	7.02E-12
RASA2	0.345579	5.03E-05
RASA3	1.438921	2.38E-22
RASA4	-0.67026	0.001578
RASD1	1.358403	0.003173
RASEF	-0.25274	0.001664
RASGEF1A	-0.29389	0.013435
RASGEF1B	1.475395	0.026709
RASGRF1	1.03063	6.29E-12
RASGRP1	-2.081	9.45E-14
RASIP1	0.91774	1.67E-11
RASL10A	1.457163	0.00091
RASL11A	0.518629	0.024028
RASL11B	-1.29915	0.000123
RASSF1	0.293707	1.86E-05
RASSF2	0.666102	0.039864
RASSF3	0.416745	4.25E-09
RASSF6	-1.35447	2.15E-16
RASSF8	0.793014	8.09E-13
RASSF8-AS:	0.804264	3.09E-05
RAVER1	0.63177	1.68E-05
RB1	0.497325	1.13E-12
RB1CC1	-0.14588	0.019928
RBBP4	0.225866	0.00064
RBBP5	0.212372	0.003355
RBBP7	0.379467	1.65E-07

RBBP8	0.392749	9.07E-05
RBBP8NL	-2.15349	2.86E-06
RBBP9	-0.18233	0.008349
RBFA	0.172963	0.012436
RBFOX2	-0.15692	0.006937
RBL1	0.481955	5.51E-07
RBL2	-0.42818	3.26E-10
RBM10	0.269718	4.92E-05
RBM14	0.488045	1.80E-07
RBM15	0.213152	0.007117
RBM17	0.163422	0.009446
RBM19	0.515555	1.72E-11
RBM20	-1.03352	0.000705
RBM22	0.451059	1.34E-12
RBM24	0.81061	0.028373
RBM25	0.205588	0.005959
RBM26	0.144119	0.01318
RBM27	0.149852	0.020154
RBM28	0.220793	8.03E-06
RBM34	0.235505	0.00102
RBM38	0.385755	0.000429
RBM42	0.337822	0.007253
RBM43	-0.65942	5.42E-10
RBM47	-0.3844	8.21E-09
RBM5	-0.14465	0.029463
RBM8A	0.123832	0.027831
RBMS2	0.549974	1.81E-10
RBMS3	-1.01459	2.28E-14
RBMX	0.249046	0.000472
RBMX2	0.206403	0.00306
RBP7	0.405321	0.000444
RBPJ	0.190681	0.006042
RBPMS2	1.462172	0.009311
RBPMS-AS1	-1.19507	0.023743
RBX1	0.254839	0.000143
RCAN3	-0.35449	8.35E-08
RCBTB1	-0.18757	0.00692
RCBTB2	-0.42546	0.000355
RCC1	0.217043	0.001811
RCC1L	0.124698	0.042946
RCL1	0.31161	0.0006
RCN1	-0.17046	0.03177
RCN3	1.077452	8.35E-05
RCOR1	0.170786	0.001568
RCOR3	-0.33671	5.83E-11
RDH13	-0.32931	0.010027
RDH16	-2.19091	2.18E-14

RDX	-0.28871	5.30E-06
RECK	-0.30648	0.002349
RECQL	0.461483	4.38E-10
RECQL4	0.605354	2.36E-12
RECQL5	-0.21517	0.026256
REEP1	-1.34778	0.001196
REEP3	-0.25259	0.000253
REEP4	-0.25581	0.037045
REEP5	-0.24211	2.20E-06
REEP6	-0.56891	0.000237
REL	-0.32399	0.003078
RELA	0.256893	0.001095
RELB	0.381551	0.003808
RELL1	0.344619	0.006022
RELL2	0.482304	4.61E-09
RELN	3.367726	2.55E-12
RELT	1.059628	4.97E-37
REN	3.126964	7.95E-09
REPS1	0.198662	0.003093
RER1	0.175957	0.000457
RET	1.137804	0.049387
RETSAT	-0.3098	0.000275
REV3L	-0.66532	1.87E-14
REXO1	0.596763	2.78E-05
REXO2	0.25731	0.000272
REXO4	0.181357	0.00077
RFC1	0.26544	5.20E-05
RFC2	0.646363	4.38E-21
RFC3	0.717165	3.03E-09
RFC4	0.576029	1.03E-12
RFC5	0.668079	9.52E-13
RFFL	0.228867	0.003262
RFK	0.271373	0.001291
RFLNB	1.04641	5.41E-11
RFPL4A	2.177904	0.044233
RFT1	0.177295	0.002019
RFTN1	1.550374	8.60E-43
RFWD3	0.542872	7.59E-11
RFX1	0.480069	9.79E-05
RFX3	0.482381	0.000132
RFX7	0.617193	1.23E-09
RGCC	1.769396	0.000133
RGMB	1.047578	1.86E-27
RGMB-AS1	0.878535	0.002612
RGP1	0.11468	0.019047
RGS10	1.375777	5.60E-36
RGS14	0.565036	9.90E-08

RGS18	2.427489	0.003466
RGS19	0.671483	0.000111
RGS2	0.687745	1.09E-07
RGS3	0.865003	5.99E-13
RGS4	3.631914	6.30E-10
RGS5	-0.24122	0.041182
RHBDD1	-0.24518	0.003612
RHBDF2	0.387841	0.001397
RHBDL2	-0.54497	5.61E-05
RHCG	-0.56533	0.015936
RHEB	0.27969	4.51E-06
RHEBP1	-0.69047	0.003362
RHEBP2	-3.08626	0.009811
RHNO1	0.342306	3.05E-06
RHOA	0.151166	0.000673
RHOB	1.331412	2.69E-24
RHOBTB1	-1.12855	0.026093
RHOBTB2	-0.3434	6.93E-05
RHOBTB3	-1.73602	8.34E-32
RHOC	0.162155	0.034787
RHOD	-0.47369	0.000333
RHOF	2.049874	4.41E-20
RHOG	0.256155	0.00306
RHOQ	-0.41154	8.18E-08
RHOT2	0.286048	0.001566
RHOV	-1.00181	3.90E-05
RHOXF1-AS	-1.12656	0.001214
RIC1	-0.44082	4.88E-10
RIC8B	-0.14177	0.027577
RIF1	0.361387	5.24E-06
RILPL1	-0.372	0.00041
RIMBP3C	-5.50989	1.78E-05
RIMKLB	-0.40093	1.87E-07
RIMS2	0.466334	1.97E-05
RIN2	-0.51697	9.13E-13
RIN3	0.264639	0.024485
RING1	-0.23382	0.000676
RINL	1.207774	2.48E-08
RINT1	0.358813	2.83E-05
RIOK1	0.504766	4.65E-14
RIPK3	-0.43233	0.011059
RIPK4	-0.75057	5.72E-06
RIT1	-0.30554	0.000115
RITA1	0.204137	0.005424
RLF	0.135816	0.047284
RLIM	0.179058	0.001805
RMI1	0.26515	0.016819

RMI2	0.3298	0.00057
RMND1	0.136626	0.019843
RMND5A	-0.29099	4.99E-07
RN7SKP116	-2.65801	8.75E-11
RN7SKP131	2.78597	2.15E-10
RN7SKP16	1.059266	0.046077
RN7SKP160	2.698338	0.040753
RN7SKP272	-3.50368	0.026457
RN7SKP63	2.78597	2.15E-10
RN7SKP80	-3.16394	0.045459
RN7SL1	0.658357	0.000143
RN7SL2	1.00333	2.20E-07
RN7SL263F	1.701749	0.035066
RN7SL268F	-1.13235	0.001559
RN7SL308F	-2.12042	0.001949
RN7SL32P	-2.31195	0.019343
RN7SL441F	-1.17363	0.022647
RN7SL472F	3.379016	0.009183
RN7SL474F	-1.03448	0.039024
RN7SL689F	-1.26493	0.000275
RN7SL81P	0.692182	0.02072
RNA5SP118	-3.38458	0.001342
RNA5SP187	-1.01464	0.021923
RNA5SP216	-3.19806	0.046318
RNASE10	-0.80818	5.20E-06
RNASE7	2.071116	3.45E-35
RNASEH1	0.21453	0.000876
RNASEH1-A	0.456817	0.000653
RNASEH2A	0.502461	3.15E-08
RNASEH2B	0.277187	0.000403
RNASEH2C	0.476098	1.02E-05
RNASEL	-0.71165	6.68E-13
RNASET2	-0.40328	0.001249
RNF10	-0.12189	0.020738
RNF103	-0.54056	2.01E-07
RNF123	-0.18724	0.029007
RNF125	0.662078	7.26E-05
RNF126	0.470772	0.004673
RNF128	-1.45946	7.56E-07
RNF13	-0.45328	1.10E-11
RNF135	-0.53451	4.24E-05
RNF138	0.393773	2.35E-05
RNF141	-0.34829	6.45E-07
RNF144A	1.680796	2.77E-15
RNF144B	-1.4272	1.31E-44
RNF145	-0.25956	0.033506
RNF146	-0.30714	1.92E-05

RNF150	-0.93033	3.66E-05
RNF152	-1.31466	4.89E-11
RNF166	0.172984	0.047479
RNF168	0.140057	0.012846
RNF169	0.211748	0.018685
RNF170	-0.50664	2.74E-08
RNF185	-0.10618	0.046755
RNF207	-0.36288	0.01178
RNF213	-0.66256	0.00038
RNF214	0.168117	0.025758
RNF216P1	0.418146	2.36E-07
RNF217	-0.51158	2.27E-08
RNF217-AS	-1.07598	0.000217
RNF219	0.263424	0.00106
RNF224	-1.16711	0.027982
RNF225	-2.6958	0.0116
RNF24	0.198603	0.04781
RNF25	0.168097	0.028505
RNF38	-0.29512	6.81E-06
RNF39	-0.90321	2.42E-05
RNF4	0.227452	4.82E-05
RNF40	0.245475	1.21E-05
RNF41	-0.24821	0.000386
RNF43	-0.98877	3.49E-13
RNF8	-0.13222	0.010965
RNGTT	0.202549	0.000382
RNPC3	-0.29506	0.008017
RNPS1	0.333109	3.90E-09
RNU2-7P	2.267385	0.004883
RNU6-126F	1.727605	0.043154
RNU6-140F	-1.76945	0.001667
RNU6-26P	1.412992	0.002743
RNU6-282F	-2.55677	0.010623
RNU6-403F	-3.86463	0.001811
RNU6-611F	-2.93932	0.04518
RNU6-7	1.289657	0.020459
RNU6-702F	-2.2627	0.016533
RNU6-8	1.289657	0.020459
ROBO1	-0.61933	1.53E-10
ROBO3	1.821613	4.37E-12
ROBO4	2.14191	1.56E-11
ROCK1P1	-0.60974	0.000135
ROGDI	-0.51647	6.66E-05
ROS1	-2.06594	9.05E-14
RP1-101A2	-0.87915	0.041565
RP1-102K2	2.591197	4.80E-11
RP11-1000	-2.01898	0.000205

RP11-1000	-0.93639	0.012139
RP11-1007	-0.76725	0.01389
RP11-1007	-0.4301	0.044716
RP11-1017	-0.55231	0.015914
RP11-1020	-0.52021	0.00457
RP11-1024	1.48541	0.000768
RP11-102N	-0.80459	0.037461
RP11-1035	1.778546	5.03E-06
RP11-1038	-1.04167	0.004502
RP11-1055	0.718862	0.044445
RP11-105N	-1.21008	0.018475
RP11-1069	-1.18695	0.040693
RP11-1070	-2.14848	0.009921
RP11-1072	-0.59761	0.022067
RP11-107M	-2.61772	0.039125
RP11-1084	-1.22463	0.019934
RP11-108H	2.987103	0.042047
RP11-108K	-1.79407	2.31E-05
RP11-108M	-1.18905	0.02094
RP11-1094	0.996586	2.16E-06
RP11-1099	-0.38444	0.005493
RP11-10A1	0.929851	0.004236
RP11-10A1	1.085885	0.005794
RP11-10C2	-0.29623	0.021984
RP11-10C2	-0.5399	0.020749
RP11-10C2	-0.45553	0.000751
RP11-10H3	-2.86008	0.049839
RP11-10J2	1.088525	2.16E-05
RP11-10N1	-1.11743	0.035468
RP11-1103	0.347597	0.025159
RP11-110G	-0.38141	0.003632
RP11-1143	-0.50796	0.03608
RP11-1148	-0.79957	4.99E-05
RP11-1149	1.757222	0.000298
RP11-1149	0.736156	7.25E-06
RP11-1149	0.369288	0.002467
RP11-114H	1.993468	0.010032
RP11-114H	2.979679	0.01256
RP11-115D	-3.06391	1.41E-09
RP11-115J	0.954613	0.004545
RP11-116D	2.740889	3.68E-06
RP11-118B	-1.83077	0.000764
RP11-119D	-2.02636	5.78E-08
RP11-119F	-2.3059	8.06E-07
RP11-120K	-0.98061	0.024195
RP11-121L	-0.52038	0.026174
RP11-121P	1.473108	4.18E-09

RP11-1223	-1.92236	0.02003
RP11-1223	-2.69831	0.030502
RP11-1228	0.725503	0.029465
RP11-1246	-1.35156	4.13E-13
RP11-124D	-1.39883	0.041839
RP11-1275	-0.91039	0.000259
RP11-1275	-1.39028	3.61E-06
RP11-128L!	-2.36035	0.003943
RP11-12G1	0.647288	3.03E-07
RP11-12J1(	-0.81095	0.010486
RP11-12J1(	-2.61231	0.016074
RP11-130L:	-3.17897	0.00074
RP11-131L:	-0.65822	0.047197
RP11-131M	-2.58576	0.000134
RP11-132A	0.534708	0.000164
RP11-1348	-1.67609	0.049232
RP11-134L:	-0.90461	9.78E-05
RP11-136I1	1.144199	0.017279
RP11-1379.	-0.58888	0.049148
RP11-138C	1.312884	1.86E-07
RP11-138I1	0.238323	0.024648
RP11-1391.	0.982614	5.97E-05
RP11-140K	-0.4347	0.048219
RP11-1415	-0.50226	8.56E-05
RP11-141C	-0.83408	0.013539
RP11-141M	-1.7625	1.50E-05
RP11-141M	-1.69807	0.014852
RP11-142A	-3.16117	1.55E-06
RP11-144O	-1.82308	0.018734
RP11-145A	-1.98295	1.48E-05
RP11-145G	-2.35093	0.004375
RP11-146E	-2.63757	0.001166
RP11-146F	0.531399	0.040459
RP11-147L:	-0.33812	1.02E-08
RP11-147L:	-0.51258	0.003604
RP11-148B	4.568882	7.11E-05
RP11-14K3	-3.78314	0.000802
RP11-14N7	0.55405	4.63E-07
RP11-152L:	-2.02682	0.004875
RP11-152N	0.518912	0.000791
RP11-153F	-1.00378	0.012552
RP11-156E	-1.69158	0.002354
RP11-156P	0.401817	0.048613
RP11-157P	-0.63679	2.89E-05
RP11-159D	-0.50693	0.025898
RP11-159F:	-0.68733	4.29E-05
RP11-159N	-1.95917	1.74E-07

RP11-15A1	-0.79907	0.038204
RP11-15B2	1.441329	0.030124
RP11-15F1	-2.07126	0.00024
RP11-15F1	-1.07677	0.034185
RP11-15H2	-0.63753	0.015757
RP11-164J1	-2.18083	2.08E-08
RP11-164P	-0.42262	0.046652
RP11-166O	-0.59947	0.018523
RP11-166P	-0.68709	0.042741
RP11-168K	-3.06172	0.029971
RP11-169F	-1.39585	0.023059
RP11-169K	2.259322	0.003475
RP11-16K1	1.251927	0.000175
RP11-170M	1.123854	0.01983
RP11-174G	-0.98265	1.24E-12
RP11-174O	0.604553	0.000976
RP11-177C	-0.55508	0.033876
RP11-177G	-1.35377	0.008948
RP11-178C	-0.35743	0.035478
RP11-179B	-0.77576	0.023632
RP11-180C	3.4019	4.23E-10
RP11-180C	1.919579	4.91E-07
RP11-182J1	-0.76169	0.030376
RP11-182J2	-1.12521	0.015208
RP11-182L1	-0.49836	0.005369
RP11-18H2	2.786504	0.037982
RP1-118J21	-1.34354	0.046795
RP11-191G	1.585598	0.028444
RP11-192H	-1.20634	3.82E-05
RP11-193H	3.646679	0.002457
RP11-196G	-0.77638	3.08E-16
RP11-196G	-1.33076	2.50E-07
RP11-201K	0.881896	0.028235
RP11-204L1	-1.66135	4.88E-08
RP11-206L1	0.235796	0.012008
RP11-206L1	-0.49845	6.39E-05
RP11-20B2	-0.77588	2.02E-08
RP11-20B2	-1.22502	0.024486
RP11-20G6	1.014719	9.33E-06
RP11-20I23	0.493069	0.015268
RP11-20I23	0.306973	0.049648
RP11-210N	-0.88997	0.022925
RP11-217E	2.533506	0.001009
RP11-218M	-0.56964	1.61E-05
RP11-219A	-0.61929	2.61E-08
RP11-219A	1.22609	0.045049
RP11-219B	1.355095	0.038397

RP11-21L2:	-1.73952	4.20E-14
RP11-21L2:	-1.7638	0.021792
RP11-220I1	-3.34813	0.036806
RP11-221J2	1.516896	0.047339
RP11-221N	2.178966	2.09E-18
RP11-221N	2.614893	0.002544
RP11-226L:	-0.26692	0.03597
RP11-227H	-0.80932	0.002041
RP11-228B	-0.62355	0.006602
RP11-22B2	0.631194	5.65E-05
RP11-230G	2.386048	1.25E-06
RP11-230L:	-1.61727	0.000301
RP11-234B	0.474006	0.012844
RP11-236F!	2.793218	0.041224
RP11-236L:	-2.82637	2.73E-05
RP11-239L:	-2.79163	0.047555
RP11-23E1	-1.24291	0.018418
RP11-23J9.	-0.65817	0.011391
RP11-23N2	-1.45205	8.93E-06
RP11-242D	-0.31823	0.012715
RP11-244F	-1.23253	0.001773
RP11-244F	-1.4201	0.00186
RP11-244H	-0.80936	0.000713
RP11-244O	-0.5697	0.010505
RP11-249C	3.042077	0.01383
RP1-124C6	-5.14657	3.71E-26
RP11-250B	-0.90375	0.009569
RP11-250B	-0.66916	0.049793
RP11-250B	-1.17714	0.000827
RP11-250B	-0.60106	0.00207
RP11-253E	-0.38134	0.015564
RP11-253M	-1.66646	2.09E-17
RP11-255E	-3.12728	0.00882
RP11-255G	1.891491	0.008474
RP11-255M	-1.59847	0.027292
RP11-256I9	1.441653	6.77E-12
RP11-260M	-1.04586	0.003568
RP11-261C	1.544553	4.11E-05
RP11-262A	-1.26074	4.61E-08
RP11-262H	-0.5917	0.00548
RP11-264B	-0.60678	0.032774
RP11-267M	0.421618	0.026544
RP11-268J1	-0.30582	0.028431
RP11-269F:	0.470295	0.000344
RP11-269G	-3.3324	0.043431
RP11-26J3.	1.10617	0.000248
RP11-274B	-1.14902	0.013994

RP11-274H	0.554349	0.03557
RP11-276H	1.293698	0.004586
RP11-276H	1.841557	1.45E-06
RP11-277P	-1.13089	3.37E-09
RP11-279N	-0.60902	0.031111
RP11-27G1	1.13684	0.000813
RP11-280G	2.847143	9.32E-06
RP11-281P	0.598977	0.032531
RP11-283G	-1.00934	0.030698
RP11-283G	2.427548	0.017522
RP11-283I3	-0.78089	2.46E-05
RP11-284E	0.539637	2.69E-06
RP11-284F.	0.781007	1.03E-07
RP11-284F.	0.843383	0.018539
RP11-285F	-0.57397	0.011756
RP11-286E	1.788671	0.048156
RP11-286H	-1.59395	0.01058
RP11-288C	-0.5585	2.92E-05
RP11-288H	0.21787	0.033467
RP11-290F.	-1.18294	0.001748
RP11-290L	4.547895	7.39E-07
RP11-290L	1.639809	0.00859
RP11-293M	-0.60678	0.02599
RP11-294C	-1.81813	0.032644
RP11-295G	0.278347	0.014937
RP11-295K	-2.95946	3.85E-19
RP11-297N	-0.85019	0.022152
RP11-298I3	-1.04603	3.41E-06
RP11-298J2	-0.45305	0.000163
RP11-299H	1.268829	0.049778
RP11-29H2	-0.73637	0.006402
RP11-2B6.2	-0.48416	0.014347
RP11-2E11	-0.88127	0.027875
RP11-2N1.2	0.587511	0.039856
RP11-301L	-3.20567	1.58E-07
RP11-303E	0.337108	0.016718
RP11-304L	-0.72003	0.035015
RP11-305M	0.748703	0.00054
RP11-307E	-0.68261	0.001235
RP11-309L	-1.05341	0.000118
RP11-30K9	-0.65266	0.000651
RP11-30P6	1.62482	1.10E-12
RP11-311F	-2.62643	3.45E-21
RP11-317N	-1.06322	0.025884
RP11-318A	-1.32809	0.010643
RP11-319E	-2.07506	0.007802
RP11-319E	-2.94646	0.042943

RP11-319G	-0.56566	0.042998
RP11-320N	2.028703	9.80E-06
RP11-320N	-2.70196	0.010866
RP11-324E	-2.06306	2.21E-05
RP11-324J1	3.815814	0.001564
RP11-326C	-0.50234	0.022376
RP11-326I1	-0.75733	0.00044
RP11-327E	-1.41601	0.000611
RP11-327P	-0.54838	0.023293
RP11-32B5	-0.94839	7.34E-06
RP11-32B5	-0.98083	6.58E-07
RP11-331F	0.612013	9.60E-05
RP11-334C	-0.45966	0.004857
RP11-336A	1.278412	0.025607
RP11-337C	-0.386	0.000579
RP11-33B1	-0.21793	0.035924
RP11-33E1	-0.54069	0.02071
RP11-33O4	-0.46036	0.034068
RP11-341G	0.721027	0.002678
RP11-342N	0.58666	0.014736
RP11-343C	0.408889	0.000843
RP11-344N	-0.39531	0.030697
RP11-345J	-1.21237	0.0306
RP11-345J	0.250996	0.000394
RP11-345P	-0.44042	0.008328
RP11-347C	-0.33704	0.044184
RP11-347C	-1.21237	0.0306
RP11-347C	1.098113	0.002785
RP11-349A	-3.36144	0.032298
RP11-34A1	-3.3168	0.011924
RP11-34P1	0.554388	0.04873
RP11-34P1	1.305392	0.001858
RP11-350J	1.108637	0.0252
RP11-350J	-2.77687	0.012012
RP11-350N	1.879771	0.003956
RP11-352B	-3.06448	0.016925
RP11-353N	-2.41136	0.048954
RP11-354N	-1.27371	0.000145
RP11-355F	-1.24013	0.011807
RP11-355O	-0.39406	0.030098
RP11-356B	-1.45578	0.013038
RP11-356I2	0.765156	0.000241
RP11-356J5	-1.08725	5.57E-08
RP11-357H	0.291702	0.021173
RP11-359B	-0.54328	0.01676
RP11-359E	1.116095	2.80E-06
RP11-359E	-0.56673	0.005256

RP11-359G	-2.41359	0.004241
RP11-359K	1.091128	3.97E-05
RP11-35J1(	-3.29091	0.014892
RP11-35O1	-1.77143	0.005307
RP11-360L!	3.882481	0.042114
RP11-360C	1.156801	0.047076
RP11-361L:	3.144792	0.004461
RP11-361L:	-0.92327	0.002187
RP11-362K	-4.42908	0.001606
RP11-363E	-0.33621	0.027179
RP11-363N	-0.54878	0.04169
RP11-364P	-0.77736	0.012664
RP11-366H	-0.4216	0.045987
RP11-366L:	1.877322	4.91E-06
RP11-366M	0.422239	0.039781
RP11-367F:	-2.21832	0.046903
RP11-367G	-2.46053	1.28E-17
RP11-367G	-2.93536	0.0148
RP11-367H	-3.04948	0.001332
RP11-367N	0.769834	0.043492
RP11-368J:	2.794276	0.04035
RP11-368P	0.824673	0.003417
RP11-370B	-1.47268	0.003611
RP11-373D	-3.15415	0.000102
RP11-377G	2.01707	0.005758
RP11-378A	-0.55589	0.031288
RP11-378E	1.141673	0.007348
RP11-379B	-0.66735	0.004925
RP11-379B	-0.89103	0.000292
RP11-379H	-0.80997	9.12E-06
RP11-37B2	-0.23364	0.04138
RP11-37C7	-0.79677	0.000723
RP11-37L2.	-1.74338	0.001974
RP11-381E	0.60873	0.040071
RP11-381C	2.482436	1.90E-15
RP11-382A	0.435369	3.73E-11
RP11-383J:	-1.99624	0.010233
RP11-384K	-0.91271	0.044412
RP11-385D	-1.31459	0.000113
RP11-386G	3.043857	3.26E-05
RP11-386G	0.683191	0.020773
RP11-387H	1.644953	0.001076
RP11-38L1!	-0.4017	0.017431
RP11-38L1!	-1.72865	2.95E-21
RP11-390E	-0.3143	0.044441
RP11-390P	-0.51996	0.032302
RP11-391H	-1.92781	0.023016

RP11-392P	-0.36752	0.000723
RP11-394B	-1.20846	2.41E-10
RP11-394J1	1.645874	0.020866
RP11-396M	3.021505	0.035201
RP11-398C	0.393426	1.20E-05
RP11-3B12	-3.18172	0.000701
RP11-3B7.1	-2.91354	0.046856
RP11-3P17	3.439918	0.036742
RP11-400K	-2.13409	0.00011
RP11-400K	-2.03051	5.27E-25
RP11-400L	-1.28383	0.045595
RP11-400N	-0.43579	0.005156
RP11-401F	-2.34588	0.000211
RP11-403B	-0.7162	0.00201
RP11-404F	-0.96016	4.81E-07
RP11-404O	-1.18801	0.008451
RP11-404P	-1.21105	0.010154
RP11-404P	-1.45413	0.000327
RP11-407G	0.947615	0.002785
RP11-408A	-2.08749	0.04896
RP11-408A	-0.79727	0.020475
RP1-140K8	-2.84219	1.07E-18
RP11-410C	-0.83257	0.010029
RP11-411G	3.247963	0.026397
RP11-415F	-1.05393	0.024975
RP11-415J8	0.763955	3.24E-07
RP11-415J8	-2.48586	0.001216
RP11-416I2	-0.33952	0.017334
RP11-417O	3.801371	0.01433
RP11-420A	-1.23343	4.30E-07
RP11-420L9	-3.30893	0.035572
RP11-420L9	-0.70951	4.77E-05
RP11-424C	0.930031	0.039987
RP11-424M	1.334164	0.000162
RP11-428J1	-0.72196	0.002733
RP11-429P	-0.587	0.020193
RP11-431J2	2.563031	2.27E-28
RP11-434D	-1.18083	0.005587
RP11-434D	-1.38187	1.70E-05
RP11-434D	-3.05283	3.22E-05
RP11-435F	0.903548	0.015261
RP11-435O	-0.86827	0.027939
RP11-439A	-3.61531	0.001726
RP11-439E	-1.24332	0.000133
RP11-441O	-0.46038	0.018077
RP11-443B	0.598135	3.19E-05
RP11-445F	1.904175	1.39E-15

RP11-445F	1.978269	1.62E-07
RP11-445P	-1.0172	0.029008
RP11-446H	-1.71232	0.02463
RP11-448A	-0.32803	0.002614
RP11-449H	0.685482	9.91E-07
RP11-449P	-0.33818	0.001578
RP11-44F1	-0.50307	0.030197
RP11-44F1	-0.83409	0.002719
RP11-44N1	0.509727	0.033537
RP11-44N2	-1.39879	0.000159
RP11-451F	3.363789	0.02285
RP11-452G	0.96068	0.034488
RP11-452L	-1.71981	0.00079
RP11-454E	-0.56997	0.014168
RP11-454K	-0.49004	0.005598
RP11-454L	-0.50473	0.02299
RP11-457M	-0.74446	0.000583
RP11-458J	-0.55371	0.002421
RP11-45A1	-1.33869	0.002538
RP11-462G	-1.15647	0.018325
RP11-462L	2.924624	3.67E-12
RP11-463I	-7.37762	0.01485
RP11-463O	0.307977	0.027479
RP11-464C	-2.42422	4.52E-09
RP11-465B	-0.59881	0.025282
RP11-465L	3.703806	0.003912
RP11-467D	-0.6599	0.034951
RP11-467J	-0.54649	0.016477
RP11-468E	-0.72961	0.000318
RP11-469A	1.169288	0.000464
RP11-46A1	-0.72723	0.000364
RP11-46C2	-0.51699	0.036298
RP11-46D6	-0.38379	0.004056
RP11-46H1	-0.79273	0.023958
RP11-472G	-3.16878	0.025218
RP11-473M	-0.68424	0.029934
RP11-474G	0.787887	0.003661
RP11-474P	-0.74518	0.026885
RP11-475I	-4.52966	4.32E-05
RP11-477D	-0.67612	0.000418
RP11-477N	-2.60917	0.002096
RP11-479G	0.723192	0.001369
RP11-47A8	1.2407	2.88E-05
RP11-47I2	1.941397	2.71E-07
RP11-483I1	-1.22927	0.008021
RP11-486L	-1.94371	9.64E-08
RP11-486M	3.519205	0.02091

RP11-486M	4.54821	1.41E-14
RP11-488C	-1.83228	0.010613
RP11-488L	0.195335	0.01814
RP11-488P	3.122348	5.07E-47
RP11-489E	-1.39249	0.027755
RP11-48B3	-0.76991	0.002639
RP11-48B3	-0.75029	0.00498
RP11-48D4	-3.18653	0.031636
RP11-490M	-0.74812	0.00156
RP11-494M	-2.03239	1.70E-05
RP11-496H	-0.54766	0.006132
RP11-496H	-0.469	0.046037
RP11-496I2	-3.31453	0.03444
RP11-496I9	-1.26149	0.003586
RP11-499O	-1.89926	2.48E-09
RP11-49K2	0.577865	0.003948
RP11-49K2	-1.46701	0.007111
RP11-4L24.	-0.42525	0.014215
RP11-4O1.:	-0.45884	0.008358
RP11-500C	1.151356	1.33E-06
RP11-500G	-1.5467	0.004398
RP11-500M	-0.60792	0.012569
RP11-504P	-0.37434	0.003835
RP11-504P	-0.51553	0.02091
RP11-505K	-0.93324	0.000583
RP11-505K	0.931493	0.021632
RP11-506K	-1.84636	8.97E-08
RP11-511H	-0.82255	0.002559
RP11-514P	-1.87966	5.29E-07
RP11-517B	-0.49985	0.035655
RP1-151F1	-0.9464	0.001901
RP1-151F1	-0.80485	2.28E-06
RP11-520H	-0.98332	0.024815
RP11-521B	0.465176	4.22E-06
RP11-521L	1.237245	0.003747
RP11-521C	1.66244	2.11E-05
RP11-522B	-1.52671	2.94E-08
RP11-523C	-0.43122	0.033374
RP11-524H	-0.76598	0.002923
RP11-525A	0.891106	4.39E-05
RP11-527D	1.571523	0.000559
RP11-527N	2.343142	5.23E-05
RP11-529K	0.529189	0.047725
RP11-530N	-0.67092	0.009052
RP11-530N	-1.46065	0.003803
RP11-532F	-1.36848	0.002722
RP11-536C	-2.16531	0.043589

RP11-536C	0.662151	0.000504
RP11-537E	-1.01079	0.012617
RP11-537H	-0.86173	0.037183
RP11-538D	-0.40481	0.024113
RP11-539L	-1.18244	0.009555
RP11-53B2	-1.49274	0.040865
RP11-53O1	-0.40285	0.008099
RP11-53O1	-0.45064	0.000427
RP11-540C	-0.84801	0.002465
RP11-541N	-0.77388	0.003186
RP11-544A	1.103812	0.034568
RP11-545A	-0.90854	0.000724
RP11-545P	1.380606	0.0443
RP11-548H	0.731825	0.002063
RP11-548K	-2.50663	0.023008
RP11-54A9	4.784284	1.03E-28
RP11-54D1	-1.60255	0.000464
RP11-54H7	0.238754	0.001209
RP11-54O7	-1.15558	0.028032
RP11-54O7	-3.31988	0.005861
RP11-550F	0.627911	0.002404
RP11-55K1	-1.07642	0.0005
RP11-561C	-1.53688	0.001816
RP11-561C	0.801809	0.007793
RP11-566E	-0.623	0.000553
RP11-567M	-0.4749	0.012282
RP11-568J	0.633288	0.030945
RP11-568K	-0.46091	0.008792
RP11-571M	2.292068	0.000423
RP11-573D	0.430164	0.028623
RP11-573M	-3.48673	0.018325
RP11-574E	4.894721	0.000217
RP11-574K	-1.00145	0.004903
RP11-57B2	-1.54559	0.003311
RP11-57C1	1.313358	0.028504
RP11-57H1	-0.73915	0.023974
RP11-57H1	-0.92132	2.38E-14
RP11-582E	-0.69657	0.000115
RP11-585P	-1.52228	0.022322
RP11-588K	-2.54818	0.00161
RP11-58O9	-1.60653	2.10E-12
RP11-593F	-1.85526	0.019322
RP11-597D	-1.74749	5.67E-13
RP11-598F	-2.61526	0.024886
RP11-59D5	2.077644	2.41E-11
RP11-5C23	-0.38399	0.033647
RP11-5N19	-1.67612	3.86E-15

RP11-5O17	-0.63349	0.034151
RP11-600F:	-0.57295	0.01651
RP11-611O	0.499171	0.00376
RP11-613M	0.682798	0.013142
RP11-618G	0.50985	0.015655
RP11-61J19	-0.81822	0.008764
RP11-61L2:	-1.42627	0.001338
RP11-61N2	0.52536	0.047189
RP11-626K	0.706715	0.006605
RP11-627G	-1.87737	0.022821
RP11-632K	-0.27805	0.035801
RP11-635L:	-1.12624	0.037915
RP11-638I2	-1.20503	0.0176
RP11-63C8	-1.5282	0.014008
RP11-63G1	0.869493	7.52E-10
RP11-640L9	-1.59499	0.035431
RP11-640N	-0.56477	0.0099
RP11-641D	0.315818	0.001551
RP11-642A	1.178048	5.40E-05
RP11-643A	-1.55191	0.042716
RP11-644F.	-0.56292	0.025131
RP11-644F.	-0.41407	0.000252
RP11-646I6	-0.97692	0.019891
RP11-649G	-1.24223	0.007688
RP11-64D2	4.499613	1.55E-15
RP11-64K1	2.542948	0.001447
RP11-650L:	0.416608	0.023543
RP11-651P	0.78803	0.010357
RP11-657O	-1.02817	0.0087
RP11-658F:	-0.66236	4.12E-08
RP11-660L:	-0.32328	0.0278
RP11-661A	-1.01111	0.035723
RP11-661A	0.379271	0.02362
RP11-662M	-1.58104	0.013752
RP11-666A	-1.69367	1.92E-11
RP11-666A	-2.52251	0.000583
RP11-666O	-1.51233	0.043057
RP11-667K	-0.73498	0.005047
RP11-66B2	-2.2212	0.030937
RP11-66B2	-0.44624	0.000895
RP11-66B2	-0.35979	0.001601
RP11-66N2	-0.49225	0.005089
RP11-676B	-0.5036	0.013219
RP11-676J:	0.657265	0.003326
RP11-676J:	0.854679	7.70E-06
RP11-676J:	-0.66899	0.03957
RP11-677M	2.240542	0.008515

RP11-678G	-0.78346	0.034222
RP11-680A	0.535097	0.005593
RP11-685M	-1.56595	0.016193
RP11-686D	-0.26658	0.049911
RP11-694I1	-1.48611	0.002132
RP11-696N	-0.54355	0.004569
RP11-69I8.	1.875281	3.13E-25
RP11-69I8.	3.655579	0.002033
RP11-69L1(	-1.23586	0.046837
RP11-6N17	-0.28101	0.027473
RP11-6O2.:	-0.64439	0.012745
RP11-701P	-1.24127	0.026009
RP11-702H	-2.78003	0.04299
RP11-706C	0.511603	7.87E-06
RP11-706C	0.539146	3.98E-21
RP11-706C	4.25887	0.010274
RP11-707G	3.602298	0.043497
RP11-707M	-0.80079	0.024729
RP11-707P	-0.57729	0.027484
RP11-70C1	0.788324	0.000929
RP11-70F1	-2.07066	0.008274
RP11-70F1	-2.39543	1.67E-05
RP11-710F	-4.11748	0.000603
RP11-717F	0.48882	0.000857
RP11-719K	-0.55561	0.02695
RP11-723C	-1.12192	0.011573
RP11-725P	-0.75099	0.002671
RP11-727A	-0.49908	2.76E-05
RP11-727F	-0.8082	0.001672
RP11-731C	-0.5609	0.011187
RP11-732A	1.114865	0.001966
RP11-734K	-0.52141	0.002274
RP11-734K	-0.77703	1.40E-07
RP11-737C	2.240907	0.019032
RP11-739L:	-0.97561	0.031476
RP11-739N	1.293715	0.000132
RP11-739P	-1.37828	0.004383
RP11-73K9	-1.02034	0.008451
RP11-745A	0.530575	0.035092
RP11-74M:	-3.02867	0.01093
RP11-750H	1.079267	0.017217
RP11-753H	1.703766	0.006123
RP11-756P	0.409101	0.005071
RP11-757F	2.019931	4.27E-05
RP11-757F	-0.35807	0.044224
RP11-758M	1.196511	0.01137
RP11-758M	-1.43086	0.000632

RP11-758N	3.442512	0.012881
RP11-75C1	3.73196	0.013887
RP11-766F	-2.06237	5.27E-06
RP11-767N	-0.47827	0.031211
RP11-770E	-1.29788	0.002047
RP11-774O	-1.86283	5.43E-18
RP11-777F	-0.92382	0.047104
RP11-77K1	-1.0789	0.000323
RP11-77M!	-0.78708	0.03963
RP11-785D	-3.36407	0.04413
RP11-789C	-1.55188	0.001326
RP11-792A	-0.26943	0.042598
RP11-793H	-1.15019	0.022909
RP11-796A	-1.42054	0.034545
RP11-797H	-0.76826	0.014422
RP11-798G	-0.53679	0.002472
RP11-799B	-1.24398	0.000517
RP11-799D	-0.61176	0.024113
RP1-179N1	-0.93068	0.002431
RP11-79N2	-1.81283	0.00054
RP11-7K24	-0.9155	0.006056
RP11-800A	-0.20191	0.030452
RP11-802E	-1.04598	0.011658
RP11-803D	1.224637	8.30E-06
RP11-806L	-0.61708	0.016753
RP11-806O	-2.22597	0.033565
RP11-80A1	-1.96733	0.009967
RP11-815M	-2.26574	1.90E-06
RP11-817I4	0.431346	0.024366
RP11-818F	-0.90156	0.000139
RP11-81A1	-0.66363	0.04408
RP11-81A1	-0.39069	0.017301
RP11-81A2	-1.03669	4.04E-06
RP11-81A2	-1.95042	0.009665
RP11-81H1	-1.47987	0.045312
RP11-81H3	-0.42532	0.029776
RP11-822E	0.987753	6.73E-05
RP11-823E	3.555951	2.21E-16
RP11-829H	1.326594	0.026923
RP11-82O1	-0.55561	0.02695
RP11-834C	-1.71153	0.022992
RP11-834C	-2.62468	0.020476
RP11-834C	-1.49418	0.022701
RP11-834C	0.684241	0.00144
RP11-83A2	-0.35264	0.004671
RP11-83J1	-2.18995	0.010769
RP11-83M	-1.43236	0.017238

RP11-83M1	-1.78205	0.001307
RP11-83M1	-2.0798	8.91E-06
RP11-83N9	-1.23467	0.012431
RP11-845M	-1.77035	0.002812
RP11-848P	-0.83052	0.002521
RP11-848P	-0.75356	8.05E-07
RP11-84N1	-3.49937	0.023593
RP11-854K	-0.88017	1.39E-06
RP11-85G2	2.208984	0.000124
RP11-861E	-0.34638	0.033512
RP11-876N	0.609007	0.000947
RP11-879F	1.760935	1.74E-08
RP11-87H9	-0.35378	0.004423
RP11-87M1	-1.94255	0.027271
RP11-87N3	1.413997	0.045051
RP11-881M	1.178925	0.028812
RP11-884K	-0.35922	0.010055
RP11-886H	4.422743	0.002694
RP11-890B	-0.72996	2.64E-10
RP11-894J1	0.895992	0.007799
RP11-89K1	-1.32616	0.047875
RP11-8L2.1	-1.00873	3.26E-05
RP11-907D	4.815965	7.07E-06
RP11-90D4	-2.10333	0.025225
RP11-90J19	-0.87699	2.90E-11
RP11-90L1.	-1.3843	1.70E-17
RP11-91G2	-0.88252	0.009515
RP11-92G1	0.681134	0.012957
RP11-932O	-1.96787	0.000128
RP11-93B1	0.728769	0.009807
RP1-193H1	-2.19807	5.50E-11
RP11-93H2	-0.60361	0.016005
RP11-949J7	-1.27414	0.000241
RP11-94H1	1.928899	0.021216
RP11-94M1	-3.07827	1.27E-05
RP11-955H	3.040601	0.005142
RP11-95M1	1.803176	0.001254
RP11-96D1	-0.85287	4.23E-05
RP11-977G	0.512802	0.021635
RP11-98J23	-0.63229	0.00622
RP1-206D1	-0.61546	0.018645
RP1-221C1	-0.58217	0.015694
RP1-223H1	-3.08073	0.018395
RP1-228H1	0.393857	0.002757
RP1-228P1	-0.69489	0.034858
RP1-261G2	2.632478	0.00031
RP1-261G2	1.684297	2.32E-15

RP1-272L1(	-3.20486	0.000628
RP1-278C1	1.208803	0.005985
RP1-27K12	-0.57069	0.016405
RP1-27K12	-1.42553	0.000361
RP1-283E3	-0.3781	0.004765
RP1-283E3	-0.27274	0.001236
RP13-104F:	-0.37334	0.047071
RP1-313I6.	0.478045	0.029639
RP1-317E2	-0.87188	0.021004
RP13-188A	-0.70887	0.010788
RP13-204A	-1.11202	0.009942
RP13-463N	1.750505	1.72E-07
RP13-476E	-1.64813	0.002568
RP13-631K	1.069132	9.21E-05
RP13-631K	2.052282	0.002502
RP13-753N	0.576245	0.032918
RP13-766D	-1.68534	0.014954
RP1-37N7.:	-1.09775	0.008981
RP1-39G22	-0.41678	0.034285
RP1-5O6.7	1.118242	0.007062
RP1-68D18	0.679803	0.016618
RP1-68D18	0.635062	0.002607
RP1-80N2.4	-0.86578	0.041979
RP1-81D8.3	-3.0354	0.015911
RP1-90G24	-0.91029	0.015024
RP1-90J20.	-1.48603	0.048154
RP1-92O14	0.526862	0.017247
RP1-95L4.4	-0.91791	0.001798
RP3-342P2	-0.74531	0.029663
RP3-355L5.	-0.70601	0.003965
RP3-355L5.	-2.92457	0.04018
RP3-368A4	-0.84713	1.48E-10
RP3-382I1(	-1.23885	2.15E-06
RP3-394A1	-0.27508	0.003637
RP3-405J1(	0.561619	0.001312
RP3-406P2	-0.93681	0.033613
RP3-412A9	2.457966	7.95E-06
RP3-414A1	-0.96006	0.002714
RP3-425C1	-0.76986	0.015366
RP3-428L1(	0.803546	0.000899
RP3-430N8	-4.57251	0.002048
RP3-439F8	-1.84077	7.85E-07
RP3-460G2	-1.33605	0.003146
RP3-467D1	0.755966	1.29E-09
RP3-523K2	0.16767	0.026832
RP4-530I15	0.646253	0.03074
RP4-536B2	-1.09734	0.010546

RP4-539M(	1.025847	3.59E-07
RP4-539M(	1.645679	6.20E-07
RP4-541C2	2.514586	0.013417
RP4-545C2	0.994195	0.002461
RP4-568C1	-1.15264	1.12E-16
RP4-569D1	-1.82155	0.026002
RP4-575N6	1.061775	0.042161
RP4-584D1	-2.25104	0.025595
RP4-590F2	-2.47373	0.023259
RP4-591C2	0.985381	2.30E-12
RP4-605O3	-0.8661	0.012394
RP4-614O4	-1.07399	4.82E-05
RP4-622L5.	4.570339	0.004963
RP4-622L5.	-1.45458	0.030408
RP4-625H1	-1.73169	0.041222
RP4-635E1	-0.74094	0.000221
RP4-665J2	-0.58177	0.040328
RP4-669L1	0.665303	0.013253
RP4-671O1	0.655901	0.000318
RP4-673M1	-0.68879	0.021443
RP4-694A7	0.804384	6.85E-10
RP4-706A1	1.360183	0.035436
RP4-734G2	-0.63768	0.022883
RP4-740C4	-1.42309	1.45E-06
RP4-742C1	4.181728	0.003783
RP4-742J2	-0.83611	0.015474
RP4-758J1	-0.34193	0.004697
RP4-773N1	-0.40958	0.000844
RP4-781K5	0.391484	0.001303
RP4-784A1	1.121628	0.021545
RP4-794I6.	-0.45414	0.044535
RP4-798P1	-0.97413	0.002871
RP4-800G7	-0.61994	0.005663
RP4-816N1	-0.93516	0.016965
RP5-1024G	-0.79217	0.012691
RP5-1028K	1.298294	0.00015
RP5-1039K	-0.87928	9.59E-15
RP5-1039K	-0.42511	2.59E-06
RP5-1044O	-0.50115	2.50E-05
RP5-1050D	1.225192	0.013079
RP5-1052I5	-2.48225	0.000643
RP5-1056H	-0.77923	0.001035
RP5-1112D	-0.88884	0.002351
RP5-1112D	-0.59768	0.040472
RP5-1120P	0.675407	0.025576
RP5-1121A	0.98655	0.040481
RP5-1125A	-1.19423	0.003328

RP5-1125A	-0.66872	0.007038
RP5-1142A	-0.6935	0.000243
RP5-1148A	-0.59898	0.011536
RP5-1157N	1.084397	0.017933
RP5-1165K	-1.49003	1.26E-05
RP5-1180D	0.266135	0.040032
RP5-821D1	-0.41119	0.013501
RP5-827C2	-0.7485	0.035328
RP5-827C2	-0.95334	0.045305
RP5-855D2	-0.59188	0.037666
RP5-862P8	3.334611	0.036415
RP5-864K1	-0.63415	0.000289
RP5-866L2(	-3.44464	5.20E-11
RP5-874C2	-2.36459	0.012132
RP5-875H1	1.678548	2.99E-09
RP5-882C2	-0.70594	0.003736
RP5-900K1	0.785383	1.96E-05
RP5-965G2	-0.59289	0.023635
RP5-966M1	-1.20397	0.002186
RP5-968P1	-0.70854	0.01302
RP5-991G2	0.356431	8.13E-06
RP5-994D1	-0.48858	0.038872
RP9	0.375247	7.42E-06
RPA1	0.178183	0.003812
RPA2	0.429684	2.52E-09
RPA3	0.419585	2.42E-05
RPAP3	0.256009	0.002245
RPARP-AS1	-0.36911	0.000688
RPE65	1.132935	8.60E-09
RPF1	0.410528	6.41E-08
RPF2	0.353933	2.74E-06
RPGR	0.652312	1.25E-05
RPIA	0.315445	0.000135
RPL11	0.086769	0.02673
RPL12	0.190451	0.000911
RPL13	0.179763	0.025883
RPL13P12	0.979794	0.004133
RPL14	0.265215	2.48E-09
RPL15	0.102011	0.025837
RPL17	0.263794	1.30E-08
RPL18	0.261795	2.11E-05
RPL18A	0.195971	0.041886
RPL19	0.224634	0.001636
RPL21	0.207351	0.002927
RPL21P16	0.281857	1.94E-05
RPL23	0.235797	6.13E-06
RPL23A	0.326329	3.94E-09

RPL23AP21	1.644408	0.037923
RPL23AP24	-4.08111	0.00645
RPL23AP42	0.369301	3.30E-07
RPL23AP53	0.532533	0.035027
RPL23AP7	0.318805	0.002464
RPL23AP82	0.407489	5.05E-08
RPL23AP97	3.140124	0.006763
RPL24	0.235075	5.12E-06
RPL26L1	0.235074	0.003731
RPL27	0.22268	1.31E-06
RPL27A	0.169168	0.013447
RPL28	0.22252	0.013998
RPL29P19	-0.97906	6.13E-07
RPL30	0.106549	0.035199
RPL32	0.239954	6.18E-08
RPL32P3	-0.37148	0.002436
RPL35	0.228324	0.00013
RPL35A	0.150932	0.000603
RPL36	0.226718	0.00557
RPL36A	0.167404	0.026479
RPL36AL	0.213429	0.004746
RPL38	0.284481	2.90E-07
RPL39L	0.628069	0.005015
RPL4	0.18174	0.000151
RPL5	0.145331	0.005476
RPL6	0.253688	1.68E-10
RPL6P27	0.645209	0.025505
RPL7	0.111054	0.019946
RPL7AP10	-0.90848	0.008719
RPL7L1	0.095362	0.032177
RPL8	0.181565	0.03956
RPL9	0.127713	0.049918
RPL9P9	0.131509	0.01272
RPLP0	0.140834	0.004767
RPLP2	0.15014	0.035157
RPN1	0.234084	3.17E-06
RPP21	0.326522	3.99E-05
RPP30	0.412595	3.00E-10
RPP40	0.175412	0.015306
RPRD1A	-0.23763	0.001439
RPS10-NUD	0.435092	0.000292
RPS13	0.132024	0.000547
RPS15	0.392355	0.000677
RPS15A	0.08786	0.024707
RPS15AP16	0.997796	0.033898
RPS16	0.329177	4.26E-09
RPS17	0.162348	0.002145

RPS19BP1	0.616508	5.69E-07
RPS2	0.255424	0.006549
RPS20	0.317866	1.33E-09
RPS20P35	0.748964	0.036303
RPS21	0.344161	4.78E-07
RPS23	0.115073	0.014733
RPS24	0.125064	0.005313
RPS25	0.165235	0.001031
RPS27A	0.158277	0.009351
RPS28	0.208738	0.016534
RPS28P7	0.306965	0.004225
RPS2P5	0.322171	0.00238
RPS3AP5	1.557566	0.0336
RPS5	0.226611	0.000543
RPS6KA2	-0.62339	0.022075
RPS6KA4	0.227642	0.04988
RPS6KA5	-0.76848	8.63E-13
RPS6KB2	0.113515	0.04327
RPS7	0.183894	0.000178
RPS9	0.140673	0.001932
RPS9P1	-1.74482	0.045579
RPSA	0.160685	0.000978
RPSAP26	3.536052	0.020375
RPSAP52	3.065502	9.22E-16
RPSAP53	4.099984	0.004977
RPUSD1	0.310732	0.035062
RPUSD3	0.173837	0.017898
RRAD	-1.78342	7.17E-11
RRAGB	-0.42901	1.38E-07
RRM1	0.67574	3.72E-15
RRM2	1.030503	1.17E-35
RRN3	0.262577	0.000326
RRN3P3	0.479139	5.54E-05
RRNAD1	-0.39795	0.000724
RRP1	0.369154	3.85E-06
RRP12	0.327692	3.23E-09
RRP15	0.30546	1.29E-05
RRP1B	0.292844	1.90E-08
RRP36	0.223156	0.002565
RRP7A	0.482089	1.51E-11
RRP7BP	0.415186	6.03E-05
RRP9	0.318994	0.000448
RRS1	0.382117	7.02E-05
RSAD1	0.175653	0.022572
RSC1A1	0.536508	1.94E-07
RSL1D1	0.471049	6.68E-12
RSL24D1	0.28579	0.000111

RSPH1	-1.1917	0.01715
RSPH3	-0.60594	1.14E-13
RSPRY1	0.190197	0.000295
RSRC2	0.155181	0.025725
RTCB	0.144564	0.001342
RTEL1	0.818061	2.60E-17
RTEL1-TNFI	0.368417	0.009379
RTKN	0.296038	0.000952
RTKN2	0.396559	0.00185
RTN2	-0.41529	0.000417
RTN4	0.270528	0.001232
RTN4RL1	-2.94611	0.028163
RTP4	-1.38587	0.003534
RTTN	0.531584	1.49E-09
RUBCN	0.198854	0.049843
RUFY3	-0.32427	3.11E-05
RUNDC3A-/	-0.60193	9.84E-09
RUNX1	-0.2064	0.020407
RUNX2	-0.84447	1.75E-14
RUNX3	1.22046	1.78E-09
RUSC2	0.886855	7.53E-14
RUVBL1	0.246026	0.002491
RUVBL2	0.314343	1.91E-05
RWDD1	0.146682	0.007978
RWDD2A	0.242132	0.025625
RWDD2B	-0.24954	0.001772
RWDD3	-0.45875	0.000137
RWDD4	0.163722	0.01213
RXRA	-0.88277	9.78E-20
RXRB	-0.29203	0.000282
RYBP	-0.17143	0.005493
RYR1	-0.76872	0.002483
S100A10	-0.94931	3.77E-15
S100A13	0.682079	2.28E-19
S100A14	-0.49234	1.02E-12
S100A16	0.369546	0.000295
S100A2	0.404146	0.00021
S100A3	0.525364	4.10E-05
S100A4	-0.84067	1.51E-10
S100A6	-0.32156	0.001913
S100A9	1.351858	1.77E-06
S100P	-2.17926	0.000403
S1PR1	2.030591	1.32E-28
S1PR3	0.692491	4.91E-06
S1PR5	-1.58503	7.49E-22
SAA1	-1.29947	1.82E-05
SAA2	-0.98363	0.028267

SAAL1	0.539933	4.43E-14
SACS	1.076553	1.55E-27
SAE1	0.304916	1.96E-07
SAFB	0.36188	3.82E-07
SAFB2	0.344672	0.000105
SAMD1	0.267929	0.003567
SAMD11	-2.45275	8.32E-05
SAMD12	-0.39916	0.020132
SAMD14	0.913697	0.00496
SAMD4A	-1.72922	1.26E-18
SAMD4B	0.362545	1.40E-07
SAMD5	0.367089	0.042035
SAMM50	0.243376	0.00028
SAMSN1	-0.51471	0.024707
SAMSN1-A!	-3.20197	0.00557
SAP130	0.296639	0.000125
SAP30	0.511316	0.000203
SAP30BP	0.259407	1.83E-06
SAP30L	-0.5284	2.06E-09
SAPCD2	0.541883	0.001121
SARAF	-0.22486	0.000322
SARM1	-0.59603	0.000671
SARNP	0.275898	0.003396
SARS2	0.259774	0.00196
SART1	0.40456	1.03E-07
SART3	0.23402	3.15E-06
SASS6	0.344274	0.000555
SAT1	0.988432	2.04E-10
SAT2	-0.32303	0.000907
SAV1	0.171147	0.016165
SAYSD1	-0.23264	0.024985
SBDS	0.491137	1.77E-10
SBDSP1	0.482351	6.72E-09
SBF2	-0.20212	0.037166
SBK1	0.776716	0.042349
SBNO1	0.346688	5.35E-14
SC5D	-0.21618	0.01364
SCAF1	0.372813	0.008465
SCAF11	-0.1593	0.002995
SCAF4	0.469708	5.40E-10
SCAMP1	-0.32482	6.87E-05
SCAMP1-A!	-0.78028	6.10E-11
SCAMP5	-0.34172	1.53E-05
SCAND2P	-0.22668	0.012903
SCARA3	-0.63644	2.19E-17
SCARB1	-0.80758	9.71E-07
SCARB2	-0.51737	1.20E-07

SCARF1	0.857816	0.002794
SCARF2	1.906951	2.20E-13
SCARNA22	1.456224	0.017576
SCART1	-0.5509	0.042564
SCD	-0.49641	0.000616
SCD5	-1.2634	5.42E-16
SCEL	-3.22021	1.04E-08
SCFD1	0.192546	0.010997
SCG5	2.850237	1.49E-29
SCGB3A2	1.873547	1.52E-20
SCLT1	0.605274	5.45E-10
SCN2A	-0.62092	0.018787
SCN3B	-0.82325	0.005784
SCN4B	-0.88842	0.021091
SCN9A	0.578601	0.004186
SCNN1A	-1.38506	1.84E-13
SCNN1G	1.064219	0.041451
SCOC	-0.17542	0.040484
SCP2	-0.26635	2.35E-05
SCPEP1	-0.92611	2.96E-18
SCRIB	0.261274	0.035551
SCRN1	0.281716	2.42E-06
SCRN2	-0.63484	4.55E-10
SCRN3	-0.28648	0.001154
SCUBE3	-0.5734	0.004144
SCYL1	0.18277	0.035071
SCYL3	-0.24443	0.005032
SDAD1	0.133514	0.038398
SDAD1P1	-0.52378	0.028791
SDC2	1.495347	0.043471
SDC3	-0.25693	0.000598
SDCBP	0.705865	1.58E-14
SDCBP2	0.842857	1.21E-06
SDCCAG3	0.280857	1.83E-05
SDCCAG8	0.495888	1.16E-10
SDE2	0.233572	0.000434
SDF2L1	0.78435	1.46E-07
SDHA	0.58413	3.09E-11
SDHAF2	0.295582	0.000633
SDHAP3	-1.2334	0.011048
SDHB	0.294632	2.02E-06
SDK1	-3.20858	2.67E-20
SDK2	2.059237	0.006088
SDPR	-0.99507	3.07E-10
SDR42E1	-0.28466	7.48E-05
SDSL	-0.29829	0.011573
SEC11C	0.362099	3.78E-06

SEC14L1	0.152611	0.013972
SEC14L2	0.980478	3.59E-31
SEC16B	-0.77518	0.004916
SEC22C	-0.13795	0.008284
SEC23B	0.402363	1.99E-07
SEC23IP	0.126945	0.019647
SEC24A	0.475241	1.79E-07
SEC31A	0.14734	0.020335
SEC31B	-1.03542	1.25E-06
SEC61A2	-0.29083	0.012138
SEC61G	0.306158	0.000114
SEC63	0.231946	3.99E-05
SECISBP2	0.190492	0.002204
SECISBP2L	-0.21641	0.003987
SECTM1	-1.84065	1.42E-18
SEH1L	0.186072	0.000314
SEL1L3	-0.82252	2.68E-09
SELENBP1	-0.78494	0.006684
SELENOH	0.318658	0.018631
SELENOK	0.37161	9.87E-05
SELENOP	-2.56492	9.91E-11
SELENOS	0.278361	3.66E-06
SELENOT	0.233853	0.002626
SELPLG	0.725471	0.029998
SEM1	-0.21457	0.001391
SEMA3A	2.150915	6.32E-12
SEMA3B	-0.95769	2.64E-10
SEMA3C	-0.45379	1.37E-05
SEMA3D	-0.56776	0.009986
SEMA3E	-2.72706	1.70E-74
SEMA4B	0.335861	0.004403
SEMA4G	-0.34087	0.005417
SEMA5B	-4.84484	6.45E-09
SEMA6B	2.987717	2.72E-22
SEMA6C	-0.64135	0.000125
SEMA6D	-2.4782	0.010351
SEMA7A	2.516201	1.50E-57
SENCR	2.946375	9.70E-13
SENP1	0.353523	1.90E-08
SENP3	0.237145	4.78E-05
SENP3-EIF4	0.762075	0.021931
SENP5	0.089433	0.043014
SENP6	-0.12465	0.041714
SENP8	-0.58497	5.07E-05
SEPHS1	0.174207	0.041142
SEPSECS	-0.48325	8.15E-10
SEPSECS-AS	-0.33185	4.08E-05

10-Sep	0.227174	5.88E-05
11-Sep	0.350332	2.83E-08
SEPT4-AS1	-1.26736	0.045491
5-Sep	0.909088	0.000621
6-Sep	-0.36852	2.60E-08
7-Sep	0.273117	6.46E-05
SEPT7P2	-0.30456	0.000398
8-Sep	0.156184	0.004477
SERAC1	-0.26074	0.000611
SERBP1	0.217158	4.90E-08
SERGEF	-0.36172	0.000242
SERINC1	-0.14421	0.030536
SERINC2	0.575672	2.45E-06
SERINC5	-0.96166	4.55E-19
SERP1	0.214368	0.004235
SERPINA1	1.094859	5.55E-08
SERPINA3	-1.97939	6.56E-06
SERPINB1	0.739684	8.22E-05
SERPINB13	-5.2195	2.82E-25
SERPINB2	1.584816	8.75E-28
SERPINB3	-0.98351	0.029274
SERPINB5	-0.6078	1.28E-07
SERPINB6	0.174099	0.00048
SERPINB7	-1.17477	1.26E-18
SERPINB8	0.989045	7.27E-22
SERPINB9	0.865166	1.05E-08
SERPIND1	1.84214	0.020563
SERPINE1	0.221216	0.009363
SERPINE2	1.359437	4.03E-07
SERTAD1	0.704604	6.72E-11
SERTAD3	-0.4346	1.18E-07
SERTAD4	-0.90784	5.23E-12
SESN1	-0.67574	2.89E-08
SESN2	0.236475	0.006945
SESTD1	0.40278	0.001322
SET	0.284371	6.81E-08
SETD1A	0.358838	1.39E-05
SETD1B	0.137198	0.048702
SETD2	-0.15868	0.000825
SETD3	0.187246	0.000246
SETD5	-0.18173	0.000714
SETDB1	-0.19037	0.004395
SETX	0.367166	1.20E-05
SEZ6L2	-0.56705	1.11E-07
SF1	0.364369	2.38E-05
SF3A1	0.465313	2.57E-11
SF3A2	0.365452	0.000747

SF3A3	0.42701	8.05E-17
SF3B2	0.310048	1.36E-07
SF3B3	0.227698	6.78E-07
SF3B4	0.451141	1.19E-14
SF3B6	0.219702	0.000179
SFI1	0.186933	0.019336
SFPQ	0.343754	0.001589
SFR1	0.898934	6.38E-18
SFRP1	-1.01346	0.024959
SFSWAP	0.331999	2.34E-06
SFT2D1	0.30132	1.10E-07
SFT2D2	-0.16345	0.028369
SFTA1P	1.621933	1.54E-14
SFXN1	0.463462	4.48E-21
SFXN2	0.539123	2.30E-13
SFXN4	0.450483	7.40E-12
SGCE	-0.40979	2.41E-11
SGK1	1.013004	2.62E-25
SGMS1	-0.34812	1.34E-05
SGMS1-AS:	-0.59628	0.00012
SGMS2	0.288642	0.00099
SGO1	0.791039	8.48E-10
SGO2	0.525898	0.000489
SGSM2	-0.29722	7.17E-05
SGSM3	-0.44385	1.37E-05
SGTA	0.397398	1.96E-06
SH2B1	-0.29421	0.016734
SH2B2	-0.46605	0.004827
SH2B3	2.281239	3.83E-68
SH2D2A	3.717443	2.50E-42
SH2D3A	0.72621	3.08E-11
SH2D4A	0.296723	0.000578
SH2D5	2.37686	9.44E-13
SH2D6	0.722725	0.030822
SH3BGRL	-0.57638	4.39E-16
SH3BGRL3	0.925057	3.45E-15
SH3BP1	0.603339	3.57E-11
SH3BP2	-0.36169	9.85E-06
SH3BP5-AS	-0.57787	0.00022
SH3BP5L	-0.16704	0.033675
SH3D19	-0.41863	2.13E-15
SH3GL1	-0.2126	0.00983
SH3GLB2	-0.41185	4.68E-05
SH3KBP1	1.525664	6.55E-40
SH3PXD2A-	0.94083	0.0001
SH3RF1	0.27911	2.38E-05
SH3RF2	-0.9755	1.39E-22

SH3RF3	2.310756	0.011824
SH3TC2	0.340231	0.010102
SH3YL1	-0.42465	6.57E-05
SHANK2	0.35407	0.015084
SHANK3	1.307146	1.52E-10
SHB	0.690127	7.18E-08
SHC1	0.44801	2.76E-09
SHC1P2	-2.66256	0.004831
SHC3	-1.1802	2.96E-07
SHCBP1	0.719219	7.35E-23
SHE	-0.65643	0.000268
SHISA2	3.654676	8.38E-40
SHISA4	-0.86873	1.96E-22
SHMT1	-0.15774	0.016229
SHMT2	0.316938	1.03E-08
SHOC2	-0.20412	0.004881
SHPK	0.43959	6.63E-08
SHPRH	-0.14491	0.048012
SHQ1	0.106909	0.045532
SHQ1P1	-0.9347	0.012636
SHROOM1	0.50299	2.81E-06
SHROOM2	-0.34073	0.0001
SHROOM3	-0.3796	2.08E-05
SHTN1	-0.63702	1.48E-30
SIAE	-0.85364	4.60E-12
SIAH1	-0.21667	0.002487
SIDT2	-0.88681	4.80E-23
SIGIRR	0.98463	2.41E-07
SIGLEC15	0.832224	0.002044
SIGMAR1	0.304201	1.83E-05
SIK1	-0.39696	0.006072
SIK2	-0.13152	0.01761
SIKE1	-0.22472	0.004355
SIM2	0.48568	0.000295
SIN3A	-0.19339	0.019856
SIPA1	0.47567	0.000486
SIPA1L2	-0.42211	0.021241
SIPA1L3	0.637045	5.56E-07
SIRPA	-0.46368	9.12E-08
SIRT3	-0.45929	2.72E-05
SIRT5	-0.29636	9.08E-07
SIVA1	0.401339	2.67E-05
SIX4	0.404821	1.39E-06
SIX5	-0.51196	0.007514
SKA1	0.710045	2.55E-16
SKA2	0.27161	0.007673
SKA3	0.985606	2.06E-18

SKI	0.314962	4.57E-05
SKIDA1	-1.13641	3.65E-05
SKIL	-0.40761	1.64E-07
SKIV2L	0.115958	0.043845
SKIV2L2	0.334999	2.57E-06
SKP2	-0.41448	7.37E-08
SLBP	0.408525	6.04E-08
SLC10A4	1.084456	0.025
SLC11A2	-0.32516	6.57E-05
SLC12A2	0.370636	1.01E-07
SLC12A9	-0.38008	0.000217
SLC13A4	-0.62185	0.018088
SLC15A1	1.414939	4.15E-05
SLC15A2	-1.00484	4.36E-21
SLC15A4	0.278403	0.001135
SLC16A1	-0.25005	0.0017
SLC16A12	-2.50478	3.00E-32
SLC16A13	-0.44263	1.29E-06
SLC16A14	-1.00569	0.000682
SLC16A1-A	0.185867	0.006457
SLC16A2	0.330342	0.001946
SLC16A3	0.755882	3.35E-09
SLC16A5	-0.90115	2.54E-11
SLC16A7	-0.49353	0.001305
SLC16A8	-0.77455	0.022673
SLC16A9	0.746072	0.049133
SLC17A5	-0.47819	0.000112
SLC18A3	-3.66434	1.67E-05
SLC18B1	-0.49871	6.59E-07
SLC19A1	0.884478	5.90E-16
SLC19A2	-0.1446	0.024526
SLC19A3	-0.50598	0.009057
SLC1A1	-1.22325	1.20E-12
SLC1A3	-0.44386	1.99E-06
SLC1A4	-0.51499	3.28E-07
SLC20A1	1.339155	1.49E-49
SLC22A1	1.648235	4.46E-16
SLC22A18A	1.378205	0.008171
SLC22A20	-0.42177	0.005315
SLC22A23	-1.65143	3.76E-28
SLC22A3	1.029068	1.76E-16
SLC22A4	0.307144	0.013089
SLC22A5	-1.08753	1.45E-09
SLC24A1	0.208409	0.032892
SLC25A10	0.540025	4.80E-09
SLC25A12	-0.13572	0.021603
SLC25A13	-0.33533	1.08E-10

SLC25A17	0.194628	0.002034
SLC25A18	-0.89034	0.000948
SLC25A19	0.805499	4.16E-17
SLC25A20	0.25909	0.01211
SLC25A23	-0.27528	0.00276
SLC25A24	0.247076	0.00043
SLC25A25	0.759124	1.49E-14
SLC25A25-/	-0.51012	0.000348
SLC25A26	-0.24554	0.000813
SLC25A27	-1.27721	7.93E-11
SLC25A28	-0.32645	0.024366
SLC25A30	-0.23584	0.002401
SLC25A32	0.586066	1.54E-09
SLC25A36	-0.36493	9.75E-08
SLC25A37	1.304622	1.40E-31
SLC25A39	0.307791	4.28E-06
SLC25A4	-0.23713	0.014963
SLC25A40	0.223033	0.018965
SLC25A5	0.505566	3.59E-23
SLC25A51	0.397675	4.02E-07
SLC26A1	-0.53477	0.01251
SLC26A11	-0.47243	3.54E-07
SLC26A2	-0.56926	6.46E-06
SLC26A6	-0.63548	0.000118
SLC27A1	-0.47122	0.000304
SLC27A2	-1.07073	2.59E-21
SLC27A3	-0.98884	5.31E-18
SLC28A3	1.787779	2.47E-06
SLC29A1	1.118459	9.97E-25
SLC29A3	-1.32409	2.70E-22
SLC2A1	-0.67006	7.09E-14
SLC2A11	-0.41923	7.08E-08
SLC2A12	-1.42562	1.53E-08
SLC2A1-AS:	-0.80618	0.003511
SLC2A4RG	-0.20385	0.048164
SLC2A5	-0.93115	0.045315
SLC2A6	0.244956	0.038187
SLC2A9	-0.23783	0.000384
SLC30A1	-0.5003	6.31E-06
SLC30A10	-1.30987	0.023678
SLC30A2	-0.65983	0.028762
SLC31A2	-0.47527	5.43E-07
SLC35A1	-0.38614	0.001314
SLC35A3	-0.325	0.000686
SLC35B1	0.273868	1.54E-08
SLC35D1	-1.0772	3.00E-27
SLC35E2	-0.35154	0.008745

SLC35E2B	-0.33803	5.06E-13
SLC35E4	0.914205	1.67E-06
SLC35F2	0.618203	2.76E-19
SLC35F3	1.392061	0.036174
SLC35F5	-0.35112	7.89E-06
SLC35G2	0.515761	4.74E-07
SLC36A1	-0.29373	0.000995
SLC37A2	0.644613	4.66E-20
SLC38A1	0.126042	0.024935
SLC38A10	-0.48808	0.000224
SLC38A11	-1.9016	0.031171
SLC38A2	-0.25995	0.013259
SLC38A4	-1.57657	2.52E-09
SLC38A7	-0.34354	3.74E-07
SLC39A10	0.862826	3.17E-16
SLC39A11	-0.68984	7.17E-07
SLC39A14	0.402455	2.18E-07
SLC39A4	0.762839	1.86E-06
SLC39A7	-0.18176	0.001902
SLC39A9	-0.21443	0.002535
SLC3A2	-0.6693	9.71E-13
SLC40A1	-1.8704	1.17E-27
SLC41A1	0.438091	1.54E-11
SLC41A2	-0.26209	0.009168
SLC43A1	0.745934	4.43E-05
SLC43A3	2.053102	8.12E-54
SLC44A1	-0.33926	1.52E-08
SLC44A2	-0.42625	5.61E-13
SLC44A3	-0.5423	8.63E-18
SLC44A3-A	-0.55356	1.68E-05
SLC44A5	-0.62317	2.00E-09
SLC45A1	-0.97178	0.04617
SLC45A3	1.814667	8.14E-33
SLC45A4	0.500375	9.87E-07
SLC46A1	-0.48379	1.55E-05
SLC47A2	-2.99396	2.51E-55
SLC4A10	-1.02854	0.002208
SLC4A11	0.434794	0.003373
SLC4A1AP	0.131811	0.009383
SLC4A2	0.199092	0.009047
SLC4A5	-0.55897	0.000246
SLC4A7	0.993535	7.05E-25
SLC50A1	-0.31051	8.36E-06
SLC52A1	-1.35963	4.40E-19
SLC52A2	0.286089	0.034664
SLC5A12	-1.3226	0.003786
SLC5A6	0.146521	0.016171

SLC6A15	0.265166	0.000205
SLC6A2	-3.08552	0.00144
SLC6A20	-0.94802	0.017983
SLC6A8	-0.44192	4.85E-07
SLC6A9	-1.53662	5.62E-36
SLC7A1	0.117782	0.036449
SLC7A11	-0.55086	0.004669
SLC7A11-A	-0.67536	0.00313
SLC7A5	-0.7425	2.75E-12
SLC7A5P1	0.727908	0.048876
SLC7A6	-0.15493	0.034839
SLC7A6OS	0.140013	0.002499
SLC7A7	-2.46177	4.65E-22
SLC7A8	-1.35971	5.32E-13
SLC8A1	0.264664	0.046
SLC8B1	0.237891	0.033459
SLC9A1	0.398438	3.90E-10
SLC9A2	0.564018	0.023766
SLC9A3	-0.73486	0.002157
SLC9A3R2	2.236969	2.06E-15
SLC9A5	0.742774	3.02E-05
SLC9A7	0.605723	3.69E-05
SLC9A7P1	-1.12628	4.21E-16
SLC9A8	-0.24829	0.000152
SLC9A9	-2.71961	7.68E-18
SLCO1B1	1.276919	0.044961
SLCO1B3	0.868413	6.88E-17
SLCO1B7	1.017731	0.039783
SLCO3A1	-1.17077	7.92E-22
SLCO4A1	2.496672	8.75E-17
SLCO4A1-A	3.436888	8.41E-06
SLCO4C1	-2.12405	1.80E-05
SLCO6A1	-1.12009	0.031975
SLF1	0.392861	1.77E-05
SLF2	0.316052	0.000998
SLFN11	0.943011	7.37E-15
SLFN12L	0.395623	0.020929
SLFNL1-AS1	0.685438	0.000263
SLIRP	0.441573	9.24E-09
SLIT2	1.532298	1.00E-44
SLIT2-IT1	3.398251	0.01766
SLITRK6	-0.91742	0.030007
SLK	0.308986	1.22E-08
SLMAP	0.384277	4.38E-09
SLPI	-1.36708	7.27E-13
SLTM	0.267556	0.000103
SLX1A	0.573358	0.000599

SLX4	0.374464	1.36E-06
SMAD3	0.668011	2.54E-12
SMAD6	-4.60813	2.38E-09
SMAD7	-0.71878	2.28E-12
SMAD9	-0.41045	0.025131
SMAGP	1.122342	1.75E-19
SMAP2	0.235534	0.00712
SMARCA4	0.409732	1.03E-06
SMARCA5	0.179415	0.016608
SMARCAD1	0.226192	0.004998
SMARCAL1	0.140973	0.034286
SMARCB1	0.258797	3.76E-05
SMARCD1	0.346809	9.45E-08
SMARCD3	-1.08749	4.76E-09
SMC1A	0.38412	5.44E-08
SMC2	0.584901	1.26E-08
SMC2-AS1	-0.48078	0.010751
SMC3	0.360833	9.29E-06
SMC4	0.566093	3.23E-08
SMC5	0.145396	0.049838
SMC6	0.166375	0.016572
SMCHD1	0.141028	0.048813
SMCO3	-2.16483	6.74E-08
SMCO4	0.810572	1.56E-05
SMCR8	0.112919	0.026286
SMDT1	-0.19095	0.041463
SMG1P4	-0.27643	0.033165
SMG5	0.111114	0.025725
SMG6	-0.15334	0.011173
SMG9	0.393718	2.74E-11
SMIM12	0.151299	0.024248
SMIM13	0.151011	0.025196
SMIM14	-0.73841	4.49E-07
SMIM15	0.418559	3.50E-08
SMIM22	0.459151	0.003268
SMIM24	1.338814	0.002262
SMIM25	0.968647	0.01756
SMN1	0.319774	7.01E-07
SMNDC1	0.202893	0.000479
SMO	-0.83307	7.28E-11
SMOC2	-1.23769	0.000175
SMOX	1.174498	1.13E-14
SMPD2	0.247532	0.022278
SMPD4	0.307734	9.18E-09
SMPDL3A	-0.57141	8.61E-09
SMPDL3B	-0.27901	0.01773
SMS	0.511251	1.55E-10

SMTN	2.558129	6.00E-57
SMTNL2	-3.93463	0.000243
SMURF1	0.295521	0.000246
SMURF2	1.960559	6.75E-63
SMURF2P1	0.655608	0.037726
SMYD2	0.137593	0.017806
SNAI1	1.0155	3.20E-06
SNAP23	0.522324	4.71E-12
SNAP29	0.154342	0.009505
SNAP91	-0.60307	0.000375
SNAPC1	0.259072	0.00241
SNAPC2	0.22203	0.047301
SNAPC3	0.218938	0.001739
SNAPC4	0.55001	7.76E-11
SNAPC5	-0.14703	0.044975
SNCA	-0.74934	5.70E-22
SNCG	-1.23447	3.26E-28
SNED1	-0.60749	0.001487
SNHG11	-0.35289	0.000237
SNHG14	0.258439	1.50E-05
SNHG15	0.227046	0.011025
SNHG16	0.264875	0.003637
SNHG17	0.431988	4.13E-08
SNHG19	0.395121	0.017943
SNHG20	-0.2602	0.044278
SNHG22	-1.72076	0.030532
SNHG4	-0.36213	0.010708
SNHG5	0.186577	0.026695
SNHG7	0.225139	0.002312
SNN	-1.07881	1.57E-27
SNORA14B	0.634097	0.032605
SNORA2	0.9168	0.035678
SNORA71C	1.183087	6.14E-05
SNORA80B	1.689309	0.023372
SNORD17	1.080614	1.33E-11
SNORD53	-3.64733	0.006468
SNORD89	-1.21024	0.016624
SNPH	-0.36541	0.010812
SNRK	0.387916	2.32E-07
SNRNP200	0.135962	0.000258
SNRNP25	0.68628	2.34E-11
SNRNP27	0.356787	6.36E-10
SNRNP40	0.351605	6.15E-11
SNRNP70	0.327685	0.001356
SNRPA	0.355311	0.000643
SNRPA1	0.325514	5.16E-06
SNRPB	0.312444	6.04E-10

SNRPB2	0.321612	2.37E-06
SNRPD1	0.495055	7.66E-11
SNRPD2	0.267847	1.61E-05
SNRPD3	0.260633	0.000395
SNRPE	0.525076	9.72E-12
SNRPEP4	4.586662	4.73E-05
SNRPF	0.449795	1.19E-10
SNRPG	0.433863	1.83E-09
SNTB2	-0.16228	0.016611
SNU13	0.242867	0.000423
SNUPN	0.134201	0.024061
SNW1	0.173534	0.00065
SNX1	-0.20344	5.58E-05
SNX10	0.690892	3.89E-11
SNX11	-0.27023	0.000709
SNX12	0.10702	0.007898
SNX18	0.276362	0.000612
SNX18P3	0.424454	0.003782
SNX18P7	1.042956	0.003795
SNX19	-0.14725	0.017095
SNX21	-0.32047	0.017367
SNX25	0.684531	6.20E-11
SNX3	0.216677	4.65E-05
SNX30	0.1597	0.018919
SNX33	-0.37855	2.28E-07
SNX4	-0.23966	7.14E-05
SNX5	0.31909	3.36E-09
SNX8	0.324887	0.000226
SNX9	1.02377	3.88E-24
SOAT1	0.255481	0.00017
SOCS1	0.615777	0.049276
SOCS2	0.548187	1.89E-06
SOCS3	0.986682	0.00492
SOCS4	0.158699	0.014706
SOCS6	-0.33866	0.001583
SOD1	-0.21942	0.000444
SOGA1	-0.29598	0.00507
SORBS1	1.537453	1.50E-13
SORBS2	-1.94658	8.28E-44
SORBS3	0.762625	1.25E-13
SORCS2	-1.80523	1.25E-17
SORD2P	0.28121	0.012113
SORL1	-0.23343	1.73E-06
SOS1	-0.27295	1.28E-07
SOS2	-0.31985	3.69E-08
SOWAHA	0.952916	0.034413
SOWAHB	-1.00516	1.71E-06

SOWAHD	-1.73655	0.00139
SOX12	-0.56354	2.37E-05
SOX13	0.658387	3.50E-12
SOX15	0.659047	6.68E-07
SOX18	-3.39256	0.000553
SOX6	-1.53581	4.61E-25
SOX7	1.228241	2.90E-18
SOX9	2.836428	3.58E-54
SP140	2.490638	5.64E-26
SP2-AS1	-0.33937	0.017887
SP6	-0.96834	0.011308
SP8	0.85343	0.000548
SP9	3.160469	0.001998
SPACA6	-0.42233	0.000525
SPACA9	-0.49958	0.001504
SPAG1	0.899052	2.13E-30
SPAG16	-0.4303	0.000489
SPAG5	0.475167	3.73E-09
SPAG9	0.140592	0.029368
SPANXD	1.293134	0.044603
SPARC	-1.9303	1.98E-32
SPAST	-0.20639	0.00012
SPATA13	0.562312	0.023475
SPATA18	-0.89265	9.86E-08
SPATA25	-0.71157	0.027088
SPATA5	0.48692	4.56E-10
SPATA5L1	0.345894	3.56E-07
SPATA7	-0.45404	2.78E-05
SPATA9	-1.11934	0.020101
SPATS2	0.335704	0.000216
SPC24	0.564474	1.76E-05
SPC25	0.701232	4.39E-08
SPCS2	0.168765	0.02768
SPCS3	0.314057	1.71E-05
SPDL1	0.410947	0.002889
SPDYE9P	-0.93734	0.048824
SPECC1	-0.42442	5.09E-14
SPEF2	-0.63061	3.55E-05
SPEG	-0.55326	1.32E-05
SPG11	-0.2958	0.002378
SPG20	-0.19712	0.004642
SPHK1	1.820645	5.50E-35
SPHK2	0.605588	3.55E-05
SPICE1	0.334052	7.47E-06
SPIDR	-0.2079	0.01302
SPIN1	-0.34664	9.26E-10
SPIN4	0.22828	0.019613

SPINK5	-1.37411	5.97E-12
SPINT2	0.22499	0.00717
SPNS1	-0.20114	0.033758
SPOCD1	1.390147	9.11E-17
SPOCK2	-1.04554	0.000503
SPON2	-2.73697	4.02E-12
SPOPL	0.314334	0.00027
SPOUT1	0.131304	0.048633
SPPL2B	-0.53695	4.09E-05
SPR	-0.24193	0.000753
SPRED1	0.157984	0.019202
SPRED2	0.362216	1.67E-05
SPRED3	2.036313	4.88E-20
SPRTN	-0.14686	0.030102
SPRY2	1.331541	7.10E-18
SPRY3	-0.56167	2.63E-07
SPRY4	1.955893	4.04E-25
SPRY4-IT1	1.90403	1.59E-08
SPRYD4	-0.26171	0.005251
SPRYD7	-0.30891	7.57E-05
SPSB1	-0.49545	2.45E-06
SPSB2	-0.41865	0.000186
SPTAN1	0.131984	0.035908
SPTB	2.52608	1.27E-13
SPTBN1	-0.40784	4.44E-08
SPTLC3	-0.79443	1.12E-06
SPTSSA	-0.45798	5.23E-07
SPTY2D1-A	-0.41385	0.049968
SQRDL	0.565495	0.000442
SRA1	0.270567	0.000685
SRBD1	0.26238	0.000644
SRCAP	0.383064	7.32E-05
SRCIN1	0.356167	0.029429
SRD5A3	-0.34637	0.000974
SREBF2	-0.20357	0.00374
SRFBP1	0.286887	4.34E-06
SRGAP1	0.608863	8.46E-17
SRGAP2	0.243074	0.002509
SRGAP2D	0.330668	0.00063
SRGAP3	-2.14173	2.26E-29
SRI	-0.17445	0.00096
SRM	0.631426	9.39E-11
SRP14-AS1	-0.30524	0.002692
SRP19	0.172785	0.010126
SRP54	0.153583	0.007832
SRP68	0.247654	1.33E-08
SRP72	0.361056	9.94E-08

SRP9	0.205707	0.003801
SRP9P1	0.717945	0.010303
SRPK1	0.326951	5.04E-09
SRPK2	-0.40687	4.78E-11
SRPX	0.683937	0.004468
SRPX2	0.846663	8.63E-08
SRRD	0.252564	0.000542
SRRM1	0.361167	6.42E-08
SRRM2	0.261699	2.67E-05
SRRM2-AS1	-1.38755	2.72E-05
SRRT	0.41955	0.000127
SRSF1	0.311298	0.008347
SRSF4	0.202829	0.004882
SRSF7	0.176165	0.047745
SRSF9	0.355697	6.80E-15
SRXN1	-0.59124	0.000282
SS18L1	-0.20335	0.002159
SS18L2	0.282985	0.009547
SSBP1	0.293533	1.54E-05
SSBP2	-0.60686	0.000334
SSBP3	-0.50093	1.01E-09
SSBP4	0.719587	0.0001
SSC5D	1.738648	0.003153
SSFA2	0.679683	3.60E-29
SSH2	-0.17069	0.029438
SSH3	-0.3906	5.31E-06
SSNA1	0.177462	0.027082
SSPN	-1.15977	1.07E-19
SSR2	0.234241	1.19E-05
SSR3	0.60896	5.16E-14
SSRP1	0.3887	7.21E-08
SSSCA1	0.307215	0.004362
SSU72	0.154244	0.007706
SSX2IP	0.269337	0.014211
ST3GAL2	0.551232	1.42E-05
ST3GAL3	-0.28653	0.014038
ST3GAL4	-0.24862	0.031757
ST3GAL4-A	-0.90243	3.12E-11
ST3GAL5	1.072556	5.80E-22
ST3GAL6	0.712202	7.05E-05
ST3GAL6-A	1.36138	0.005394
ST5	-0.45306	4.84E-09
ST6GALNA(	-1.74188	1.38E-14
ST6GALNA(	0.371745	0.000361
ST6GALNA(	-0.84451	0.000981
ST7-AS1	-1.01619	0.003465
STAC2	-2.26043	3.74E-05

STAG1	0.373493	1.96E-06
STAG2	0.225007	0.002755
STAG3	-0.8406	2.03E-12
STAG3L1	-0.77216	6.39E-07
STAG3L2	-0.2797	0.038747
STAG3L5P	-0.64258	2.23E-05
STAG3L5P-	-0.58674	9.73E-06
STAM	0.330804	8.12E-05
STAMBP	-0.15415	0.015257
STAMBPL1	1.731134	9.78E-41
STAP2	0.321694	0.0006
STARD3	0.43002	3.02E-06
STARD3NL	0.263828	1.75E-05
STARD4-AS	-0.61631	0.04633
STARD5	-1.50226	4.72E-12
STARD8	0.956245	0.012703
STAT2	-0.82522	6.39E-06
STAT4	-1.32256	6.19E-06
STAT5A	-0.33328	0.012468
STAT5B	-0.21247	0.016473
STAU1	0.203623	0.008912
STBD1	-0.58871	1.29E-08
STEAP1	2.973998	5.65E-33
STEAP1B	1.963601	0.008593
STEAP2	1.230613	6.75E-09
STEAP3	-0.82499	1.18E-13
STEAP4	-1.50728	2.21E-08
STIL	0.697285	1.41E-13
STIM1	-0.29869	0.01219
STIP1	0.353856	1.13E-10
STK10	1.009564	2.10E-25
STK11IP	-0.16104	0.037664
STK17A	0.654057	1.23E-13
STK19	-0.45119	1.62E-07
STK24	0.151408	0.001947
STK26	-0.18432	0.002872
STK31	-0.70356	0.002277
STK32A	-1.49281	0.001884
STK38	-0.41713	4.71E-14
STK38L	-0.37953	9.82E-06
STK39	0.729278	6.29E-13
STK4	0.399463	3.74E-19
STK40	0.330359	0.002345
STMN1	0.659268	7.99E-09
STMN3	1.38796	5.18E-19
STN1	0.191613	0.002794
STOM	-0.77174	1.10E-12

STOML2	0.304501	8.00E-07
STON1	-0.76945	0.037417
STON2	-0.60596	4.36E-08
STOX2	-0.53491	4.02E-07
STRAP	0.190151	0.003817
STRBP	0.243014	0.000862
STRC	-1.05731	0.008892
STRCP1	-1.41156	0.008488
STRIP2	1.171394	1.75E-17
STRN3	0.206661	0.00754
STT3A	0.268721	9.02E-10
STT3B	0.2426	0.000361
STX17	-0.38328	5.82E-07
STX18	0.263377	0.000745
STX18-AS1	-0.43832	0.010235
STX19	-1.85805	1.85E-16
STX1A	0.626079	5.17E-09
STX1B	-0.61427	0.018301
STX2	0.562971	1.29E-10
STX3	0.346442	2.70E-07
STX6	0.143743	0.016509
STX7	-0.20735	0.000145
STXBP3	-0.14124	0.025146
STXBP5	0.450955	1.01E-06
STXBP5-AS	0.530335	0.044114
STYK1	0.777312	4.90E-17
STYXL1	0.196701	0.000682
SUB1	0.339212	1.40E-05
SUCLG1	0.157653	0.001443
SUCLG2-AS	0.50293	0.00727
SUDS3	-0.24657	0.000131
SUFU	-0.30971	0.000254
SUGT1	0.466193	5.36E-15
SUGT1P2	-1.81081	0.049455
SULT1A1	-0.55043	0.01533
SULT1A2	-0.40658	0.024138
SULT1B1	-0.74437	0.001923
SULT1E1	-6.50719	4.14E-12
SULT2B1	-1.59029	6.39E-05
SUMF1	-0.32791	6.61E-08
SUMF2	-0.23909	1.09E-07
SUMO1	0.23627	0.00214
SUMO2	0.397967	1.97E-09
SUMO3	0.343216	7.12E-10
SUN1	-0.19518	2.34E-06
SUN2	-0.22899	0.005692
SUN3	1.574696	2.75E-05

SUOX	-0.16826	0.011677
SUPT16H	0.269547	1.46E-05
SUPT4H1	0.111098	0.030454
SUPT5H	0.141591	0.001808
SUPT7L	-0.20436	4.16E-05
SUPV3L1	0.193542	0.006346
SURF1	-0.16997	0.001649
SURF2	0.680757	9.28E-12
SURF4	0.142038	0.026541
SURF6	0.379957	8.51E-05
SUSD1	-0.28284	0.005552
SUSD2	-0.82039	0.000295
SUSD4	5.027803	2.24E-08
SUSD5	-0.30429	0.029327
SUV39H1	0.590598	3.20E-21
SUV39H2	0.475938	2.25E-07
SUZ12	0.226509	0.001624
SVEP1	-0.86656	1.99E-05
SVOPL	-0.97158	7.09E-11
SWAP70	-0.20967	0.00031
SWI5	0.248859	0.015563
SYBU	-1.12593	1.10E-32
SYDE1	1.051551	5.17E-08
SYN1	2.078162	0.022454
SYNC	-1.26935	3.05E-14
SYNCRIP	0.180985	0.015075
SYNE1	-0.56092	2.39E-09
SYNE2	-0.5561	1.32E-07
SYNE3	0.681212	0.00026
SYNE4	-0.96018	0.027098
SYNGAP1	-0.1815	0.033344
SYNJ1	0.365036	1.45E-06
SYNJ2	1.302257	3.08E-33
SYNJ2BP	-0.19483	0.012101
SYNM	-0.34149	0.013086
SYNPO2	-0.94247	4.53E-06
SYS1	0.157586	0.007836
SYT1	-0.26839	0.003064
SYT11	0.718013	0.006924
SYT12	-1.28433	3.00E-07
SYT14	0.284879	0.044481
SYT15	-1.66783	3.38E-19
SYT16	-0.75385	1.85E-06
SYT17	-0.99455	9.94E-11
SYT8	-2.40528	6.28E-16
SYTL1	-0.76033	3.68E-12
SYTL3	-0.48112	0.015146

SYTL4	0.798032	2.48E-11
SYVN1	0.357425	3.06E-05
SZRD1	0.300448	1.11E-10
TAB1	-0.18124	0.040124
TAB2	0.16726	0.001494
TAB3	-0.52787	2.10E-15
TACC1	0.24329	0.00169
TACC2	-0.93063	3.23E-15
TACC3	0.536108	2.61E-10
TACO1	0.152037	0.028966
TACSTD2	-0.53659	0.000219
TADA2A	0.162101	0.043187
TADA2B	0.271741	2.77E-05
TAF10	0.219466	0.006227
TAF11	-0.40041	2.37E-05
TAF12	0.197814	0.000285
TAF13	0.196454	0.041828
TAF1A	0.710872	2.04E-17
TAF1B	0.376916	2.51E-07
TAF2	0.180411	0.014806
TAF3	0.43992	6.58E-08
TAF4	0.404092	6.97E-09
TAF4B	0.40532	1.02E-05
TAF5	0.51907	2.43E-08
TAF5L	0.293622	9.66E-06
TAF6	0.302037	1.48E-05
TAF9B	0.330714	3.32E-05
TAGLN	-0.7104	2.24E-05
TAGLN3	3.612495	2.65E-39
TALDO1	-0.22922	0.023115
TANC1	-0.22459	5.17E-07
TANC2	-0.18089	0.001584
TANGO6	0.26246	0.0078
TAOK3	0.247564	0.002105
TAP1	-0.49561	0.001109
TAPBP	-0.33931	0.003135
TAPBPL	-0.92884	1.95E-11
TAPT1-AS1	-1.22121	7.28E-06
TARBP1	-0.27605	0.000528
TARDBP	0.17344	0.006024
TARS	0.41331	1.74E-09
TARSL2	-0.63769	5.18E-08
TAS2R38	-1.1626	0.031655
TAS2R4	-0.86479	0.009184
TASP1	-0.18486	0.021016
TATDN2P2	0.643071	5.16E-10
TAX1BP1	-0.24196	8.39E-05

TBC1D1	0.592099	1.33E-12
TBC1D10B	0.359014	2.65E-06
TBC1D10C	-2.34687	0.006425
TBC1D12	-0.17516	0.043241
TBC1D13	-0.18304	0.007663
TBC1D17	-0.26774	0.002279
TBC1D19	-0.39676	0.000176
TBC1D2	0.316191	0.018122
TBC1D20	-0.19457	6.11E-05
TBC1D22A	-0.18108	0.024395
TBC1D24	0.371324	0.001143
TBC1D29	-1.00659	0.037005
TBC1D2B	0.391181	4.30E-10
TBC1D30	0.292984	0.008143
TBC1D31	0.266229	0.000823
TBC1D32	-0.21806	0.018594
TBC1D3B	-1.23405	1.47E-05
TBC1D3L	-0.89504	3.70E-09
TBC1D3P5	-1.18944	0.003458
TBC1D8	-0.5817	1.72E-09
TBC1D8-AS	-0.52652	0.005277
TBC1D9	-0.61578	1.62E-06
TBC1D9B	-0.27128	0.000914
TBCA	0.150129	0.035276
TBCB	0.345761	4.62E-05
TBCC	-0.18314	0.041613
TBCEL	-0.53776	9.16E-14
TBL1XR1	0.192147	0.015994
TBL3	0.406617	0.000203
TBP	0.212927	0.000253
TBPL1	0.160035	0.014438
TBRG1	-0.32841	6.25E-08
TBRG4	0.170429	0.008986
TBX18	-0.22801	0.028603
TBX18-AS1	-2.7205	0.010054
TBX3	2.481149	1.08E-57
TC2N	0.217055	0.004532
TCAF1P1	-0.21112	0.030333
TCAF2	-0.76616	9.76E-05
TCAF2P1	-1.38221	0.040873
TCEA1	0.205112	0.005163
TCEA2	-0.65948	0.000103
TCEA3	-1.43342	1.55E-21
TCEAL1	-0.8581	3.30E-19
TCEAL3	-0.31243	0.002568
TCEAL4	-1.13194	7.30E-22
TCEANC	-0.40499	0.048771

TCERG1	0.303872	0.000202
TCF19	0.847387	7.06E-27
TCF20	0.125996	0.040588
TCF25	-0.23956	0.002138
TCF3	0.290311	0.008304
TCF4	-0.41352	0.002071
TCF7	1.658016	5.10E-22
TCF7L1	-0.51823	1.32E-06
TCFL5	-0.28341	3.78E-05
TCHH	-0.47827	0.049392
TCHHL1	-2.10267	7.00E-05
TCHP	0.497842	3.02E-09
TCOF1	0.888162	5.35E-32
TCP1	0.531905	1.50E-22
TCP11L1	-0.78157	2.36E-12
TCP11L2	-0.72606	3.47E-11
TCTA	-0.50122	5.30E-11
TCTEX1D2	0.519091	9.92E-06
TCTEX1D4	1.755959	0.001069
TCTN1	-0.42466	6.74E-06
TCTN2	-0.19114	0.013443
TCTN3	-0.27376	4.29E-07
TDG	-0.26554	0.005707
TDP1	0.375064	2.21E-07
TDRD10	-1.22156	0.016847
TDRD3	-0.18944	0.016339
TDRD6	-2.39234	2.31E-05
TEAD2	0.279677	0.03005
TEAD3	-0.44289	1.28E-08
TEAD4	0.341009	0.000118
TECPR2	-0.45243	1.33E-07
TEKT4P2	-0.45006	0.000363
TELO2	0.557249	9.58E-06
TENM2	-2.1218	1.11E-07
TENM3	-1.51197	9.05E-17
TEP1	0.329124	0.000861
TERF2IP	0.218161	0.012596
TERT	1.213881	1.38E-12
TESK2	-0.82498	1.12E-05
TET1	-1.45226	8.46E-12
TET2	-0.53255	3.58E-12
TET3	-0.6252	9.10E-07
TEX10	0.654388	3.53E-12
TEX15	0.892854	3.81E-15
TEX264	-0.26793	0.005458
TEX30	0.875001	2.47E-15
TFAM	0.459253	1.57E-07

TFAP2C	-1.08265	1.36E-20
TFB2M	0.29077	5.77E-05
TFDP1	0.440393	1.07E-13
TFDP2	-0.48821	8.50E-13
TFE3	0.272784	0.004109
TFEB	-0.74635	9.57E-08
TFIP11	0.170936	0.000943
TFP1	-2.9452	0.040946
TFPI	-0.34041	0.000598
TG	-1.60143	0.00058
TGDS	0.17782	0.041056
TGFA	2.037949	1.09E-81
TGFB1	1.112343	1.01E-10
TGFB1I1	0.747099	2.56E-11
TGFB2	-2.64762	8.22E-34
TGFB2-AS1	-2.1816	2.34E-05
TGFBR1	-0.37802	3.86E-07
TGFBR2	0.913709	1.42E-26
TGFBR3	-1.21712	2.07E-17
TGFBRAP1	0.165946	0.026117
TGIF1	-0.51883	1.01E-12
TGIF2	0.727325	1.46E-13
TGM1	-1.93782	6.39E-13
TGM2	1.812975	2.15E-21
TGM5	-3.10488	0.000513
TGS1	0.210089	0.015516
TH	1.368726	0.000116
THADA	0.181484	0.000359
THAP1	0.427292	1.09E-06
THAP10	0.508498	4.05E-06
THAP12	0.303811	7.11E-07
THAP4	0.224157	0.002689
THAP7-AS1	-0.57143	0.015087
THAP8	-0.41028	0.014716
THBS2	-0.75889	2.71E-05
THBS3	-0.63232	4.20E-06
THEM4	-0.16541	0.048355
THEM6	-1.16424	2.51E-14
THOC1	0.241408	0.003103
THOC3	0.302049	8.19E-06
THOC5	0.443517	2.43E-16
THOP1	0.447612	1.34E-06
THRA	-0.72168	3.41E-11
THRB	-0.32079	0.004114
THSD4	0.588719	8.26E-05
THSD7B	-1.92348	4.43E-06
THUMPD2	0.324385	9.52E-05

TIA1	-0.30439	0.000115
TIAL1	0.116561	0.009143
TIAM2	-0.74206	2.86E-14
TICAM1	0.714079	5.93E-07
TICAM2	1.150548	0.000695
TICRR	0.436707	0.000429
TIE1	5.104565	1.36E-15
TIFA	0.479324	2.45E-07
TIGD6	-0.21807	0.01869
TIGD7	-0.87111	0.000277
TIMELESS	0.329332	1.68E-05
TIMM10	0.39237	0.000587
TIMM13	0.439162	9.60E-05
TIMM17A	0.250241	0.011564
TIMM17B	0.158066	0.026939
TIMM21	0.296268	0.000803
TIMM22	0.246657	5.67E-07
TIMM23	0.217582	0.000105
TIMM44	0.237708	2.69E-05
TIMM50	0.418219	6.31E-08
TIMM8A	0.267671	0.002197
TIMM8B	-0.21713	0.004153
TIMM9	0.223229	0.002623
TIMP1	0.849248	6.32E-21
TIMP2	-0.50959	1.11E-11
TIMP3	-2.18906	2.09E-11
TINAGL1	0.77586	2.90E-06
TINCR	-2.69089	4.59E-09
TINF2	0.158976	0.012624
TIPARP	-0.43898	0.00153
TIPIN	0.78927	4.14E-12
TIRAP	0.2646	0.000526
TJP2	0.3313	0.000705
TK1	0.648166	8.40E-09
TK2	-0.67001	5.18E-08
TKFC	-0.3549	3.44E-05
TKT	-0.70567	1.64E-13
TLCD1	0.191535	0.046107
TLCD2	-0.83618	1.38E-17
TLE1	-0.27213	0.005237
TLE3	0.349583	0.000953
TLK1	-0.16522	0.01852
TLL1	-1.43303	2.33E-20
TLN1	0.398227	4.38E-08
TLN2	-0.48416	0.015114
TLR3	-0.99806	4.79E-10
TLR6	1.005426	7.80E-07

TLX2	-1.57121	0.040596
TM2D2	-0.21659	0.005998
TM4SF19	2.945281	7.09E-19
TM4SF19-A	2.514695	1.05E-25
TM4SF19-T	2.754278	1.33E-14
TM6SF1	1.768324	8.79E-07
TM6SF2	-2.30618	0.006683
TM7SF2	-0.59346	3.79E-05
TM9SF2	-0.1425	0.00145
TM9SF3	0.13034	0.036392
TMA16	0.22933	0.009321
TMA7	0.13993	0.036235
TMBIM4	-0.30586	0.000178
TMBIM6	0.07938	0.017808
TMC1	-1.36302	0.038039
TMC4	-0.46069	1.25E-07
TMC5	0.731204	0.000907
TMC7	0.417853	0.000327
TMC8	2.202039	4.67E-11
TMCC3	1.033976	3.41E-11
TMCO3	-0.31787	4.94E-06
TMCO4	-0.41872	1.61E-05
TMED10	0.135927	0.023634
TMED2	0.135465	0.024126
TMED4	0.212372	0.022476
TMED5	0.210513	0.003593
TMED7	0.161852	0.024654
TMED7-TIC	0.65641	8.14E-05
TMED8	0.187012	0.034798
TMED9	0.217842	0.001231
TMEFF1	1.547699	0.000664
TMEM104	0.148489	0.026863
TMEM105	-0.9655	0.000322
TMEM106A	0.351377	0.002933
TMEM106B	-0.29934	0.017206
TMEM106C	0.328778	1.92E-07
TMEM107	0.306291	0.007661
TMEM109	-0.26843	0.006359
TMEM11	0.280514	2.45E-06
TMEM110	-0.21441	0.000744
TMEM117	-1.57387	2.65E-19
TMEM125	0.634167	2.21E-06
TMEM126B	0.156776	0.000785
TMEM127	-0.14371	0.009205
TMEM128	-0.17707	0.017251
TMEM129	-0.51578	4.03E-07
TMEM134	-0.69257	6.46E-11

TMEM138	-0.31138	0.000827
TMEM140	-0.89037	8.22E-11
TMEM145	0.897711	0.000393
TMEM147	0.207055	0.023858
TMEM14B	0.314456	0.000296
TMEM150A	-0.31922	0.000771
TMEM154	0.563459	3.49E-11
TMEM156	3.139093	8.60E-25
TMEM159	-0.70303	3.28E-10
TMEM160	0.804411	0.007961
TMEM161B	-0.29758	0.012459
TMEM165	-0.20809	0.003069
TMEM167B	-0.18515	0.010315
TMEM168	-0.40945	1.44E-06
TMEM170A	0.429586	8.35E-11
TMEM171	1.314594	4.84E-11
TMEM173	0.279476	0.007213
TMEM175	-0.32748	0.042814
TMEM178A	-2.07366	0.023234
TMEM178B	-2.01961	0.038412
TMEM179B	-0.20035	0.007452
TMEM184A	-0.51882	0.000653
TMEM184B	0.271901	0.001994
TMEM184C	-0.16976	0.004584
TMEM185A	-0.33684	8.51E-06
TMEM185B	0.378443	2.74E-06
TMEM186	-0.22981	0.00544
TMEM189	-0.52626	1.69E-15
TMEM19	-0.27059	0.001253
TMEM191C	-0.66447	0.001031
TMEM198B	-0.39482	0.02113
TMEM199	0.222191	0.001599
TMEM2	-0.20478	0.027245
TMEM200A	1.681342	3.57E-10
TMEM201	0.683063	2.98E-11
TMEM206	0.420263	0.000175
TMEM209	0.363798	4.78E-09
TMEM214	0.196091	0.009773
TMEM217	0.442889	0.012693
TMEM218	-0.31761	0.016596
TMEM229B	-0.34642	0.005978
TMEM237	-0.52328	3.79E-09
TMEM243	-0.18619	0.009087
TMEM25	-0.14997	0.014857
TMEM254	-1.00001	8.34E-20
TMEM254-	-1.08953	0.000299
TMEM255A	-2.19829	1.99E-10

TMEM258	0.237701	0.0004
TMEM260	-0.28236	3.32E-05
TMEM263	0.1792	0.017008
TMEM266	-0.39766	0.046453
TMEM267	-0.23318	0.007443
TMEM27	-1.5614	0.000328
TMEM30B	0.167467	0.048466
TMEM33	0.375595	1.59E-07
TMEM38A	-0.70857	1.33E-07
TMEM38B	0.26556	0.004219
TMEM39A	0.191026	0.004777
TMEM39B	0.539782	2.41E-08
TMEM41A	0.177224	0.004627
TMEM41B	0.21448	0.006393
TMEM42	-0.28578	0.000459
TMEM43	-0.36555	2.17E-07
TMEM44-A	-0.31495	0.033547
TMEM45B	1.841627	1.03E-12
TMEM47	-0.51192	0.0088
TMEM50A	-0.11496	0.03606
TMEM51	0.200379	0.003511
TMEM52B	-3.0652	5.32E-19
TMEM53	-0.72362	6.98E-11
TMEM55A	0.352685	1.17E-06
TMEM55B	-0.18446	0.020236
TMEM56	-0.37056	0.000475
TMEM56-R	-0.30917	0.037152
TMEM59	-0.3672	5.63E-05
TMEM60	-0.38639	4.06E-05
TMEM63A	0.421374	7.75E-06
TMEM63B	-0.48283	2.73E-11
TMEM63C	-2.1654	0.010736
TMEM68	-0.19624	0.011818
TMEM70	0.313026	9.52E-05
TMEM71	2.053904	7.63E-09
TMEM74	1.271464	0.00569
TMEM82	3.372151	0.007238
TMEM86A	-0.57634	0.035356
TMEM8A	0.718714	7.36E-09
TMEM8B	-0.91097	0.000108
TMEM9	-0.40471	1.01E-10
TMEM91	-0.56546	0.006552
TMEM92	2.085298	9.74E-22
TMEM92-A	1.47896	0.000129
TMEM94	-0.28027	1.82E-06
TMEM97	0.289223	0.030983
TMEM98	0.299498	0.001633

TMEM9B	0.15356	0.000985
TMEM9B-A	-1.18242	3.61E-05
TMPO	0.563744	2.11E-10
TMPRSS11I	-2.41393	0.000181
TMPRSS13	-0.87012	1.61E-05
TMPRSS7	-1.49975	0.004932
TMPRSS9	-0.6303	0.002901
TMSB10	0.611688	3.64E-12
TMTC2	-0.44438	0.01763
TMTC4	-0.28364	0.001148
TMUB2	-0.36062	1.79E-05
TMX1	0.186442	0.008901
TMX2P1	0.287035	0.017268
TNC	-0.73645	3.31E-05
TNF	1.003565	0.001522
TNFAIP2	0.286941	0.006646
TNFAIP3	1.140972	9.95E-15
TNFAIP8	-0.4049	0.001043
TNFAIP8L3	0.302011	0.00151
TNFRSF10A	0.969741	1.61E-13
TNFRSF10B	0.494097	4.30E-07
TNFRSF10D	0.580057	4.48E-06
TNFRSF11A	0.5132	0.00044
TNFRSF12A	0.391286	0.000518
TNFRSF14	-0.64498	0.009603
TNFRSF21	-0.97305	2.90E-19
TNFRSF25	0.801064	1.29E-06
TNFSF10	-1.9203	1.58E-06
TNFSF13B	-0.80439	0.029411
TNFSF15	2.6296	4.29E-15
TNFSF4	-1.00022	0.007433
TNFSF8	3.536743	4.99E-07
TNIK	0.607448	8.16E-09
TNIP1	0.67833	2.86E-07
TNIP2	0.257159	0.000103
TNK1	-0.40697	4.65E-09
TNKS1BP1	-0.60763	1.77E-16
TNNC1	-2.16619	2.09E-09
TNNI2	-2.88694	6.23E-18
TNNT1	0.505482	0.001797
TNNT2	-2.00201	4.29E-05
TNNT3	-3.59483	9.21E-21
TNPO1	0.350998	2.08E-07
TNPO3	0.097581	0.029504
TNRC6A	-0.25114	0.000258
TNRC6C-AS	-0.32262	0.019644
TNS1	0.299056	0.022523

TNS2	0.628646	1.94E-05
TNS3	-0.92843	2.78E-39
TNS4	-0.82869	7.22E-06
TNXB	-0.93996	5.61E-06
TOB1	-0.50262	1.07E-07
TOE1	0.417762	1.13E-05
TOGARAM1	-0.27254	0.000457
TOM1	-0.6156	5.10E-09
TOM1L1	0.155862	0.049299
TOM1L2	-0.6113	3.93E-06
TOMM22	0.382207	1.86E-09
TOMM34	0.784541	9.49E-22
TOMM40	0.599822	1.92E-19
TOMM40L	0.458602	8.51E-09
TOMM5	0.286667	3.70E-06
TOMM6	0.317595	2.24E-05
TOMM7	-0.19586	0.005952
TOMM70	0.138601	0.013952
TONSL	0.752548	7.92E-19
TONSL-AS1	-1.14577	0.003455
TOP1	0.479754	3.51E-17
TOP3A	0.280318	0.000646
TOPBP1	0.546813	2.91E-12
TOPORS	0.127171	0.015985
TOR1AIP1	0.335259	0.000118
TOR1B	0.209645	0.013558
TOR3A	0.224972	0.000146
TOR4A	0.989277	1.26E-08
TP53	-0.75469	4.36E-06
TP53BP2	0.61567	1.67E-11
TP53I3	-0.78912	0.001355
TP53INP1	-1.06891	3.91E-16
TP53INP2	-0.71091	1.79E-07
TP53TG1	-1.1209	1.85E-20
TP63	-0.51075	5.03E-06
TP73	1.177915	1.40E-09
TP73-AS1	0.537656	1.70E-06
TPBG	0.424426	0.000393
TPCN1	0.231657	0.015672
TPD52L1	-0.51905	7.34E-14
TPD52L2	0.438399	2.82E-14
TPGS2	0.184779	0.001531
TPI1	0.166574	9.53E-05
TPM1	-1.35274	3.27E-17
TPM2	0.237735	0.003422
TPM3	0.578779	1.11E-21
TPM4	0.467852	6.18E-16

TPP1	-0.29832	0.000441
TPP2	0.369324	3.44E-08
TPPP	0.658446	0.00382
TPRA1	-0.39152	2.48E-05
TPRG1	-0.91276	1.29E-07
TPRG1-AS1	-1.48178	0.000349
TPRG1L	-0.35505	7.65E-05
TPRKB	0.398125	1.67E-05
TPRKBP2	3.074458	0.020113
TPRN	0.599067	7.60E-08
TPRXL	-0.90385	0.038417
TPST1	0.252183	0.000226
TPST2	-0.52736	5.48E-07
TPT1-AS1	-0.32884	0.000889
TPTEP1	-0.62468	1.82E-07
TPX2	0.476043	3.39E-05
TRA2B	0.294909	0.001777
TRABD	0.382134	0.002529
TRABD2A	0.904656	2.09E-11
TRADD	-0.37704	0.005406
TRAF2	0.334723	3.37E-05
TRAF3	0.322789	1.24E-07
TRAF3IP1	-0.22367	0.003089
TRAF3IP2	-0.62027	3.20E-26
TRAFD1	-0.75878	7.19E-15
TRAIP	0.351681	0.005551
TRAK1	-0.20713	0.000137
TRAM2	0.209011	0.002049
TRANK1	-1.2176	1.20E-05
TRAP1	0.387355	1.99E-10
TRAPPC10	0.186503	0.003764
TRAPPC12-	-1.24553	0.009909
TRAPPC13	-0.19632	0.030628
TRAPPC6A	-1.65459	4.69E-36
TRAPPC9	-0.29348	0.013062
TRDMT1	0.536611	0.000283
TREM2	-1.74678	0.014251
TREX1	-0.40354	0.016703
TRHDE	2.395829	5.34E-08
TRHDE-AS1	0.940283	0.006095
TRIAP1	-0.24104	0.020497
TRIB1	2.167423	7.76E-36
TRIB3	0.422621	0.000916
TRIM11	0.329657	0.00027
TRIM13	-0.34596	5.72E-11
TRIM15	1.894918	0.002437
TRIM16	-1.14602	5.17E-15

TRIM16L	-0.91398	2.77E-08
TRIM22	-1.46632	0.007848
TRIM24	-0.30837	0.000325
TRIM26	-0.23481	0.002375
TRIM27	0.449857	6.06E-09
TRIM28	0.382396	9.39E-10
TRIM29	-1.99815	1.75E-16
TRIM34	-0.61442	2.30E-06
TRIM35	-0.36043	0.003878
TRIM36	0.825102	1.50E-07
TRIM37	-0.19212	0.006495
TRIM38	-0.24851	0.000315
TRIM4	-0.24626	0.001229
TRIM52	-0.3273	0.006082
TRIM56	-0.38011	6.51E-09
TRIM58	-1.14776	0.041531
TRIM6	-0.54444	9.50E-11
TRIM62	-0.56318	3.43E-09
TRIM66	-0.39191	0.006079
TRIM68	-0.33788	0.000577
TRIM69	-0.33044	0.040913
TRIM73	-0.72043	0.002344
TRIM8	0.902629	3.12E-11
TRIM9	1.533975	0.000631
TRIML2	1.572417	5.79E-22
TRIP12	0.186944	0.004181
TRIP13	0.91778	1.88E-18
TRIQK	-0.27228	0.000572
TRMT1	0.343186	9.06E-06
TRMT11	0.242006	0.001908
TRMT112	0.178875	0.003889
TRMT13	-0.18352	0.045526
TRMT2A	0.372254	6.66E-06
TRMT2B	0.541728	7.12E-14
TRMT44	0.270919	0.000171
TRMT6	0.409194	3.07E-09
TRMT61A	0.334766	0.002643
TRMT61B	-0.20827	0.017732
TRMU	0.293117	0.000278
TRNAU1AP	0.21725	0.002892
TRNP1	-0.3214	0.017353
TROAP	0.334261	0.014021
TROVE2	0.150451	0.029546
TRPA1	-1.03951	0.003538
TRPM3	-1.36959	0.003062
TRPM4	-0.94539	1.21E-09
TRPM6	-0.95517	1.52E-08

TRPS1	-0.37033	0.025612
TRPV1	-0.38318	0.001411
TRPV3	1.60083	7.05E-09
TRPV4	-1.2314	1.89E-07
TRRAP	0.283465	2.62E-07
TSC1	-0.17121	0.005707
TSC22D2	0.321392	0.004349
TSC22D3	-2.38519	9.39E-19
TSEN54	0.157329	0.019339
TSKS	-2.17526	8.09E-05
TSKU	-0.83355	1.96E-11
TSN	0.153501	0.041827
TSNARE1	-0.56103	5.90E-05
TSPAN1	0.404781	0.005713
TSPAN10	-0.86968	9.62E-07
TSPAN12	-0.29005	0.00951
TSPAN14	0.587586	3.06E-05
TSPAN15	-0.21153	0.002162
TSPAN17	-0.43881	6.18E-15
TSPAN18	2.876288	2.90E-05
TSPAN2	-0.46165	0.000417
TSPAN31	-0.28249	2.17E-07
TSPAN4	0.950774	2.01E-12
TSPAN5	1.611633	2.22E-30
TSPAN6	0.221286	0.004619
TSPAN9	-0.19532	0.046308
TSPOAP1	-0.87232	0.006781
TSPY26P	-1.20168	0.003812
TSPYL2	0.571073	1.55E-05
TSR1	0.386812	1.48E-12
TSR3	0.320474	0.00168
TSSC2	-0.5155	0.000365
TSSC4	0.197319	0.040399
TSSK4	-0.57358	0.003258
TSSK5P	-0.82567	2.44E-06
TSTA3	0.336747	8.06E-06
TSTD1	-0.55621	0.001826
TSTD2	-0.16829	0.029901
TSTD3	-0.37733	0.028327
TTC12	-0.50414	1.29E-07
TTC17	-0.20898	0.022733
TTC19	0.178219	0.006653
TTC21A	-0.88594	1.97E-09
TTC22	-0.93615	8.13E-14
TTC23	-0.42776	2.98E-10
TTC26	0.241943	0.012076
TTC27	0.18464	0.00476

TTC3	-0.17311	0.006346
TTC30A	-0.50633	0.001234
TTC30B	-0.68181	2.90E-06
TTC32	-0.54302	0.00081
TTC39B	-0.75628	5.77E-09
TTC39C	-0.18346	0.001538
TTC3P1	-0.4891	0.003355
TTC4	0.200866	0.000233
TTC41P	0.387701	0.033027
TTC7A	-0.46727	4.52E-07
TTC8	-0.2741	0.000469
TTC9	-1.22877	4.35E-21
TTF1	0.209845	0.001356
TTF2	0.257214	2.45E-06
TTI1	0.280699	2.47E-10
TTI2	0.280108	1.51E-05
TTK	0.540674	2.20E-05
TTL	0.3599	5.29E-07
TTLL1	-0.82868	0.012031
TTLL11-IT1	0.637887	0.032413
TTLL12	0.326458	3.91E-06
TTLL3	-0.58438	2.30E-11
TTLL4	0.389796	2.70E-08
TTLL5	0.242953	0.001581
TTN	-0.30842	0.001308
TUBA1A	-0.56082	2.28E-07
TUBA1B	0.616752	6.21E-20
TUBA1C	0.443755	6.26E-12
TUBAL3	0.762442	0.039147
TUBB	0.412898	2.50E-16
TUBB3	0.457715	0.000366
TUBB4B	0.470993	3.23E-18
TUBB6	0.676097	5.14E-15
TUBBP5	-0.61204	0.029132
TUBD1	-0.35584	0.000488
TUBG1	0.572649	4.87E-27
TUBG1P	-1.92378	0.036072
TUBGCP2	0.201621	0.023095
TUBGCP3	0.243908	1.81E-05
TUBGCP4	0.251888	0.000159
TUFM	0.186768	0.001669
TUFT1	-0.54065	2.61E-05
TUG1	-0.19783	0.000299
TULP3	0.230249	0.000384
TULP4	-0.33145	5.45E-11
TUSC3	-0.86685	0.005608
TVP23C	0.505251	1.09E-08

TWF2	0.654848	6.82E-09
TWIST1	-0.30812	0.000897
TWISTNB	0.325168	2.29E-08
TWNK	0.185785	0.000322
TXK	0.82935	0.008575
TXLNA	0.106282	0.024887
TXLNG	0.403975	1.96E-09
TXNDC12	0.193552	5.63E-06
TXNDC17	0.266823	6.19E-05
TXNDC5	0.375678	3.17E-07
TXNDC9	0.497975	4.93E-08
TXNL1	0.291172	2.24E-05
TXNL4A	0.307021	1.86E-06
TXNRD3	-0.74777	4.21E-09
TYMS	0.837542	9.98E-22
TYRO3	-0.29236	0.000101
TYRP1	-0.91276	0.028996
TYSND1	-0.91733	4.17E-17
U2AF1	0.446391	6.81E-07
U2AF1L4	-0.56322	0.005616
U2AF2	0.503393	1.58E-11
U2SURP	0.359294	6.06E-07
U91328.21	1.965556	0.014598
UACA	0.284877	4.85E-05
UAP1	1.225028	4.58E-26
UBA1	0.317659	1.08E-06
UBA2	0.399688	2.94E-11
UBA5	0.107581	0.019971
UBA52	0.245987	4.44E-06
UBA6	0.66637	2.57E-11
UBA6-AS1	0.263972	0.005669
UBA7	-0.87723	1.23E-05
UBAC2	-0.27109	0.000106
UBALD2	0.604752	4.69E-09
UBAP1L	-0.6302	0.023045
UBAP2	0.11512	0.046261
UBAP2L	0.213464	0.000546
UBASH3B	-0.42937	2.23E-05
UBB	0.170914	0.032776
UBE2C	0.580384	4.05E-05
UBE2D2	0.211459	0.002935
UBE2D3	0.124588	0.01447
UBE2E1	0.18187	0.008188
UBE2E3	0.162047	0.028294
UBE2F	0.43013	3.41E-07
UBE2G1	-0.11553	0.039982
UBE2H	-0.4645	4.80E-22

UBE2I	0.471287	7.87E-14
UBE2J1	0.272691	0.00125
UBE2J2	0.303254	0.000792
UBE2L3	0.151525	0.000496
UBE2L6	-0.71609	0.007638
UBE2M	0.342349	0.000777
UBE2N	0.316788	3.00E-08
UBE2Q2	-0.21494	0.000267
UBE2S	0.742201	3.43E-09
UBE2T	0.701127	4.82E-18
UBE2V1	0.204139	6.77E-06
UBE3A	0.218419	0.003799
UBE3B	-0.20746	0.000159
UBE3D	0.231014	0.031318
UBE4B	-0.14638	0.017155
UBL3	-0.32974	1.71E-09
UBL4A	0.189299	0.013364
UBN2	-0.30644	0.000517
UBQLN1	0.242073	0.000178
UBQLNL	-1.54444	0.032153
UBR1	0.241304	0.0003
UBR2	-0.2625	8.21E-05
UBR4	0.256836	7.45E-05
UBR7	0.411286	1.21E-07
UBTD1	0.650999	0.000309
UBTD2	-0.27483	0.00024
UBXN1	0.214694	0.000939
UBXN2A	0.113185	0.044292
UBXN4	-0.19329	0.01906
UCA1	0.967842	4.51E-07
UCHL3	0.424636	6.42E-09
UCHL5	0.517692	4.21E-13
UCK2	0.385222	2.93E-11
UCN2	-0.94062	1.56E-05
UCP2	0.917137	2.87E-08
UCP3	-1.06786	0.045581
UFD1L	0.255473	2.77E-09
UFL1	-0.19453	0.000892
UGCG	0.321582	8.35E-05
UGDH	0.285163	0.004867
UGGT1	0.198437	0.001956
UGP2	-0.28742	7.43E-05
UGT1A1	-2.10389	8.41E-11
UGT1A10	-4.29092	1.02E-14
UGT1A3	-4.27415	2.98E-07
UGT1A4	-3.95681	0.003532
UGT1A6	-3.19481	1.39E-18

UGT1A7	-4.36938	1.80E-06
UGT1A8	-4.10581	0.046975
UGT2B17	-3.71656	0.043737
UGT3A2	-2.36756	2.42E-05
UHMK1	0.177603	0.015587
UHRF1	0.874724	2.38E-16
UHRF1BP1	-0.41016	1.35E-09
UHRF1BP1I	0.296658	3.29E-05
UHRF2	0.228594	0.001786
UIMC1	0.28541	5.81E-06
ULBP2	0.635987	1.37E-11
ULK1	-0.67087	3.82E-09
ULK2	-0.32298	2.07E-05
UMPS	0.171816	0.002004
UNC119	-0.24994	8.25E-05
UNC119B	-0.23269	0.001204
UNC50	-0.13809	0.023055
UNC5B	-3.18204	2.90E-11
UNC79	-0.75482	0.012686
UNC80	-0.31952	0.045339
UNG	0.533896	3.98E-14
UPF1	0.25959	0.001095
UPF3B	0.210568	0.015535
UPK2	-1.89296	1.81E-20
UPK3B	-1.84386	2.10E-26
UPK3BL	-1.74874	3.03E-07
UPP1	2.419463	9.52E-44
UPRT	0.222941	0.003627
UQCC2	-0.16551	0.025586
UQCRC2	0.116728	0.029878
UQCRFS1P1	2.255847	0.033492
UQCRH	0.295265	1.21E-06
URB1	0.304891	0.000175
URB2	0.484059	3.44E-15
UROD	0.240761	0.000363
UROS	-0.13734	0.013812
USB1	0.316132	2.02E-05
USF2	0.339198	0.001195
USF3	-0.18224	0.003089
USH1G	-4.31114	7.54E-10
USMG5	0.13087	0.015604
USP1	0.392409	6.78E-05
USP10	0.195994	0.001897
USP12	0.369984	1.36E-08
USP14	0.159369	0.006997
USP19	-0.22649	2.64E-05
USP24	-0.19023	0.008107

USP25	-0.21686	0.013249
USP2-AS1	0.558193	0.045753
USP3	0.134736	0.046364
USP30	-0.2428	0.000897
USP30-AS1	-1.25125	0.011277
USP31	-0.36013	2.62E-05
USP32	0.177959	0.010398
USP32P1	-0.47351	7.90E-06
USP32P3	-1.58389	5.24E-09
USP33	-0.14129	0.017229
USP34	0.176611	0.001418
USP36	0.378086	1.09E-10
USP39	0.209607	1.76E-05
USP4	0.193222	0.005492
USP40	-0.50226	5.16E-12
USP41	-1.46614	0.019063
USP43	-0.30741	0.001953
USP46	-0.16055	0.003803
USP46-AS1	-0.63806	1.39E-05
USP49	-0.4095	5.33E-07
USP5	-0.20641	1.12E-05
USP54	-0.14096	0.035364
USP6	-0.30576	0.006994
USP7	0.278241	3.31E-08
USP9X	-0.13043	0.023928
USPL1	0.174076	0.009281
UST	-0.42234	0.000199
UTP11	0.637469	2.49E-19
UTP14A	0.297114	2.28E-05
UTP14C	-0.12076	0.046919
UTP18	0.510984	1.71E-14
UTP20	0.224616	0.000468
UTP3	0.215814	0.013191
UTP4	0.480046	1.25E-14
UTP6	0.390794	2.26E-12
UTRN	-0.63261	1.49E-15
VAC14-AS1	0.772517	0.00059
VAMP1	-0.68089	0.001636
VAMP2	-0.23487	0.00049
VAMP8	-0.11974	0.045232
VANGL1	-0.40699	1.06E-05
VANGL2	-0.71712	3.65E-28
VAPA	0.21329	0.001274
VARS	0.623684	8.67E-14
VARS2	-0.23692	0.002181
VASP	0.603132	1.90E-09
VAV3	-3.57738	1.88E-48

VAX1	1.451877	0.004012
VBP1	0.244605	0.000454
VCAN	2.327051	1.95E-30
VCP	0.173658	0.021032
VCPKMT	-0.25153	0.004756
VDAC1	0.409615	3.98E-22
VDAC1P8	-0.26714	0.02375
VDAC2	0.212336	0.000995
VDAC3	0.244547	1.16E-06
VDR	1.2891	2.64E-29
VEGFA	1.381035	5.29E-21
VEGFC	0.925794	1.77E-17
VEPH1	0.924046	7.04E-12
VEZF1	0.168013	0.00641
VEZT	0.24797	0.00399
VGF	1.236006	0.005308
VGLL1	-0.68188	8.45E-08
VGLL3	-0.35317	0.017005
VHL	-0.19219	0.000157
VILL	-2.94948	1.19E-09
VIM	3.902596	6.61E-30
VIM-AS1	2.321982	6.99E-10
VIPAS39	-0.26871	0.00294
VIPR1	-1.76846	1.63E-53
VIPR1-AS1	-1.70499	1.72E-08
VIT	-4.21801	0.001745
VKORC1	0.247233	0.003789
VMAC	-0.71494	0.000526
VMP1	0.37922	9.44E-08
VOPP1	0.337822	4.57E-06
VPS13B	-0.2942	3.41E-05
VPS13C	-0.40757	0.000296
VPS13D	-0.33653	0.000469
VPS26B	-0.25455	0.000169
VPS37A	0.183108	0.011858
VPS37B	0.642637	1.72E-08
VPS39	-0.40526	2.00E-09
VPS45	-0.14995	0.016477
VPS51	0.35469	0.015374
VPS52	0.177024	0.000597
VPS72	0.13995	0.020228
VPS8	-0.2573	0.005227
VPS9D1	-0.29211	0.001997
VPS9D1-AS	0.284805	0.02842
VRK1	0.641778	2.97E-09
VSIG1	0.443723	0.032565
VSIG10	-0.41086	1.01E-08

VSIG10L	-0.94198	0.000271
VSIR	-1.08962	8.47E-07
VSNL1	-1.53368	3.24E-09
VSTM1	4.625345	2.12E-05
VSTM5	-1.21553	0.001748
VTA1	-0.11974	0.035161
VTA1P2	3.551811	0.005022
VTCN1	-5.57537	1.15E-16
VTN	-2.84271	3.09E-07
VWA2	-0.70994	0.000112
VWA5A	-1.22546	1.91E-05
VWA7	-0.88397	5.72E-06
VWA8-AS1	-1.59825	0.028589
VWDE	-0.58067	5.27E-06
WAC-AS1	-0.63538	3.97E-10
WAPL	0.145484	0.031267
WARS	0.262072	0.005734
WASF1	-0.70144	1.46E-09
WASH3P	-0.21309	0.004267
WASHC3	-0.29581	0.001045
WBP1	-0.68499	1.02E-13
WBP11	0.262926	0.000126
WBP1L	-0.35768	6.70E-09
WBP1LP2	0.875556	0.031425
WBP4	0.208367	0.004472
WBSCR27	0.668605	0.005577
WDCP	0.326199	0.00634
WDFY1	-0.21416	1.72E-05
WDHD1	0.7598	3.58E-16
WDR1	0.264275	2.30E-09
WDR12	0.237233	0.000148
WDR18	0.347527	0.024123
WDR19	-0.29036	5.65E-05
WDR20	0.163721	0.014879
WDR24	0.351775	0.002173
WDR26	0.149461	0.039455
WDR3	0.181499	9.77E-05
WDR31	-0.34317	0.009445
WDR34	0.368434	2.47E-06
WDR36	0.265534	8.04E-06
WDR4	0.43476	4.29E-06
WDR41	-0.27603	0.000561
WDR43	0.417243	3.02E-08
WDR48	-0.13748	0.014899
WDR5	0.155829	0.00167
WDR53	0.347815	5.23E-07
WDR54	0.53357	3.53E-11

WDR59	-0.2341	3.06E-05
WDR5B	-0.28041	0.033257
WDR6	-0.17289	0.000887
WDR62	0.835866	2.38E-18
WDR66	0.270207	0.00031
WDR74	0.245724	1.26E-05
WDR75	0.255545	1.26E-05
WDR76	0.789589	1.78E-15
WDR77	0.403302	1.89E-10
WDR81	-0.25441	0.035956
WDR83	-0.24249	0.011764
WDR86	-1.1063	0.044672
WDR88	-0.70566	0.002028
WDR89	0.162594	0.038083
WDR91	-0.58207	9.38E-10
WDR92	-0.22969	0.031621
WDSUB1	-0.45052	5.63E-10
WDTC1	-0.28164	1.66E-05
WDYHV1	0.213317	0.019213
WFDC1	0.60293	0.038657
WFDC2	-1.00406	3.69E-10
WFDC21P	-2.85318	2.08E-12
WHAMM	0.161059	0.026203
WHAMMP:	0.311983	0.002477
WHRN	-0.75221	0.011539
WIPF1	0.32234	0.026251
WIPF3	0.585032	0.02838
WIPI1	-0.53245	3.65E-05
WISP2	-1.1765	0.000605
WIZ	0.413411	3.96E-05
WLS	-0.32361	6.11E-06
WNK1	-0.14473	0.036453
WNK4	0.619762	0.000342
WNT10A	-3.19615	9.43E-24
WNT2B	-0.44046	0.004053
WNT3	0.434297	0.000444
WNT3A	1.507626	0.00231
WNT4	-2.14503	4.41E-13
WNT5B	1.442854	2.85E-05
WNT6	-3.33476	1.47E-12
WNT7A	1.400371	1.83E-16
WNT7B	-0.52179	0.006829
WRAP53	0.317193	6.29E-05
WRAP73	0.294497	0.001152
WRNIP1	0.236943	0.000825
WSB1	-0.18504	0.016162
WSCD1	1.928718	3.88E-07

WWC2	0.508208	1.80E-09
WWC3	-0.52013	2.57E-05
WWTR1	0.204505	0.001003
XBP1	0.256543	0.000772
XCL1	-0.77274	0.000718
XDH	1.019161	7.97E-24
XG	-0.29443	0.008005
XIAPP3	-2.54868	0.02668
XIRP2	2.816154	0.018558
XK	-0.71983	0.0156
XKR4	-0.81919	0.030314
XPC	-0.51214	2.63E-07
XPO4	0.31545	3.57E-07
XPO5	0.193557	0.00111
XPO6	0.237471	3.30E-05
XPO7	0.150492	0.004974
XPR1	-0.48288	3.24E-22
XRCC1	0.344884	4.47E-07
XRCC2	0.600916	1.34E-09
XRCC3	0.462301	4.74E-09
XRCC4	0.43114	1.86E-06
XRCC5	0.36376	1.10E-09
XRCC6	0.331953	1.20E-06
XRN1	-0.26338	0.00208
XRN2	0.326193	2.10E-05
XXbac-B461	-0.56715	0.008446
XXbac-B476	-0.37248	0.006951
XXbac-BPG	-0.51955	0.027989
XXbac-BPG	1.307783	2.24E-08
XXbac-BPG	-0.29089	0.015433
XXbac-BPG	-2.71181	0.03831
XXbac-BPG	-0.57086	5.98E-06
XXYLT1	-0.23584	0.010567
XYLB	0.344715	0.000662
YAP1	0.187523	2.32E-05
YARS	0.330251	1.64E-16
YARS2	0.282192	0.000209
YBX1	0.405624	2.18E-10
YBX1P1	1.182982	2.99E-05
YBX3	-0.17005	0.000447
YEATS2	0.325006	9.00E-06
YEATS4	0.231751	0.042538
YIPF1	-0.34667	4.48E-07
YIPF4	-0.24682	0.000167
YJEFN3	-0.53815	0.013596
YKT6	0.183123	4.20E-05
YLPM1	0.173105	0.006812

YOD1	0.298782	0.007378
YPEL2	-0.38434	0.000411
YPEL3	-0.68487	4.84E-08
YPEL4	-0.63595	0.014572
YPEL5	-0.34655	6.14E-05
YRDC	0.628652	1.81E-09
YTHDF2	0.14035	0.011236
YWHAB	0.253781	8.01E-05
YWHABP2	1.397473	0.033293
YWHAE	0.207494	4.38E-08
YWHAH	0.38495	1.16E-17
YWHAZ	0.29184	2.91E-06
ZACN	-1.23263	0.019484
ZAN	-1.62184	0.000609
ZBED2	0.900888	0.026027
ZBED3	-0.5096	3.58E-09
ZBED4	0.198574	0.000278
ZBED5	-0.34523	0.006248
ZBED5-AS1	-0.80147	2.52E-06
ZBED8	-0.3748	0.011766
ZBTB10	-0.26109	0.006784
ZBTB16	-3.19734	1.45E-30
ZBTB2	0.382922	0.000493
ZBTB22	-0.37356	7.76E-08
ZBTB24	-0.13605	0.031279
ZBTB33	-0.26481	0.000375
ZBTB37	-0.32885	0.000138
ZBTB38	-0.21902	0.000124
ZBTB4	-0.51137	9.55E-09
ZBTB41	-0.23547	0.014552
ZBTB44	-0.19992	0.000379
ZBTB47	-0.78922	7.63E-09
ZBTB5	-0.53254	1.73E-11
ZBTB7B	-0.81319	1.93E-08
ZC2HC1A	-0.30747	0.012786
ZC2HC1C	0.633482	2.83E-05
ZC3H12A	0.548472	0.003513
ZC3H12B	-0.64985	0.023699
ZC3H12C	0.968892	2.14E-11
ZC3H14	0.257416	7.81E-07
ZC3H15	0.340326	8.68E-09
ZC3H18	0.458518	3.51E-12
ZC3H3	0.227187	0.011067
ZC3H7A	0.203935	0.000521
ZC3H7B	0.303839	5.63E-05
ZC3HC1	0.136918	0.025897
ZCCHC11	-0.3116	8.34E-08

ZCCHC17	0.184229	0.004875
ZCCHC2	-0.42804	0.000255
ZCCHC4	0.385444	2.59E-05
ZCCHC8	0.264788	0.001296
ZDHHC1	-0.83035	0.000165
ZDHHC13	0.161279	0.020288
ZDHHC14	0.26005	0.015059
ZDHHC15	-0.78036	0.002505
ZDHHC16	0.126799	0.028634
ZDHHC17	-0.32536	0.000117
ZDHHC2	0.300246	0.003079
ZDHHC23	-0.31397	0.000375
ZDHHC24	-0.54182	1.36E-05
ZDHHC3	-0.17087	0.004703
ZDHHC4P1	-1.65951	0.017219
ZDHHC6	0.335676	9.27E-08
ZDHHC7	-0.22968	0.009447
ZDHHC9	-0.16552	0.012128
ZEB1	2.193606	5.16E-21
ZEB1-AS1	0.609484	0.002438
ZER1	-0.48618	2.70E-06
ZFAND2A	0.477644	3.53E-08
ZFAND2B	0.16642	0.04043
ZFHX2	0.481753	0.014262
ZFHX3	0.227314	0.043469
ZFHX4	-0.7511	1.14E-14
ZFP1	0.410713	4.46E-06
ZFP14	-0.51345	2.70E-05
ZFP2	-2.44486	8.89E-05
ZFP3	-0.32168	0.009096
ZFP30	-0.35741	0.000927
ZFP36L1	0.886375	1.79E-09
ZFP36L2	-0.42474	0.00296
ZFP64	0.205277	0.006348
ZFP69B	0.557706	7.81E-08
ZFP90	-0.2019	0.006618
ZFPM2	-0.39419	0.001296
ZFR	0.195864	0.037025
ZFYVE1	-0.33479	1.89E-08
ZFYVE26	-0.20373	0.003192
ZFYVE28	-0.73855	3.67E-09
ZG16	-2.16539	8.23E-05
ZGLP1	-0.58281	0.039575
ZGPAT	0.294157	0.002419
ZGRF1	0.657175	7.54E-09
ZHX1	-0.35227	0.00016
ZHX2	0.278283	0.013767

ZIC2	0.76874	7.77E-12
ZIC5	0.395931	0.00105
ZKSCAN3	-0.41654	0.000844
ZKSCAN4	-0.57109	5.21E-07
ZMAT3	-0.26944	0.000371
ZMIZ2	0.297762	0.005322
ZMYM2	-0.38681	7.44E-13
ZMYM3	-0.1263	0.016192
ZMYM5	-0.20029	0.02085
ZMYM6	-0.21626	0.003124
ZMYND11	-0.14496	0.001869
ZMYND15	-0.75203	0.004295
ZMYND19	0.295885	1.97E-05
ZMYND8	0.128725	0.037972
ZNF10	-0.3104	0.000752
ZNF101	0.302877	0.003407
ZNF106	0.444975	2.10E-12
ZNF112	-0.26399	0.027004
ZNF117	-0.53886	1.21E-06
ZNF121	0.361179	0.001021
ZNF124	0.348427	0.003682
ZNF132	-0.7795	0.000437
ZNF133	-0.34047	8.42E-05
ZNF134	-0.2998	8.58E-06
ZNF142	0.358445	1.45E-10
ZNF143	0.560602	1.59E-12
ZNF154	0.774275	0.007296
ZNF160	-0.19044	0.008617
ZNF165	0.918382	8.32E-20
ZNF169	-0.36637	0.03107
ZNF18	-0.42859	1.17E-05
ZNF180	0.385797	0.001162
ZNF181	-0.34275	0.027591
ZNF19	-0.57934	4.28E-07
ZNF2	-0.19047	0.010449
ZNF200	0.181034	0.020933
ZNF204P	-0.34688	0.002738
ZNF205	0.267074	0.026475
ZNF211	-0.37065	1.99E-05
ZNF212	-0.19629	0.01384
ZNF217	-0.14768	0.010911
ZNF219	0.607876	0.000733
ZNF22	0.220927	0.026733
ZNF221	-0.38916	0.014654
ZNF223	-0.56429	7.24E-06
ZNF224	-0.55597	6.16E-10
ZNF225	-0.42942	0.000263

ZNF226	-0.21819	0.00417
ZNF23	0.184549	0.016987
ZNF230	-0.26854	0.043431
ZNF239	0.493409	0.00816
ZNF248	-0.41491	0.003733
ZNF25	-0.30195	0.049521
ZNF250	-0.31081	0.007412
ZNF251	-0.53476	9.68E-09
ZNF252P	-0.22723	0.001495
ZNF26	0.311082	1.41E-05
ZNF264	-0.27569	0.004533
ZNF267	0.320401	8.99E-05
ZNF274	0.264461	0.00105
ZNF275	0.246072	0.019574
ZNF280D	-0.19407	0.001487
ZNF281	0.510055	7.09E-08
ZNF287	-0.3394	0.017303
ZNF300P1	3.383047	0.005965
ZNF302	-0.35123	0.015822
ZNF304	-0.15802	0.044461
ZNF311	-0.55915	0.001698
ZNF319	0.272348	0.024215
ZNF320	-0.1866	0.003242
ZNF321P	-0.45964	0.01493
ZNF322	-0.21903	0.010316
ZNF326	0.20849	0.027484
ZNF331	-1.46227	3.45E-07
ZNF335	0.257045	0.005489
ZNF337	-0.29243	0.000437
ZNF33A	-0.38032	3.22E-08
ZNF341	0.260856	0.024452
ZNF343	0.166807	0.03847
ZNF346	-0.20605	0.011647
ZNF350-AS	-1.04484	0.000111
ZNF354A	0.253472	0.000193
ZNF358	-0.34334	0.014183
ZNF362	-1.40578	1.05E-37
ZNF367	0.502628	1.21E-06
ZNF37BP	-0.2162	0.045377
ZNF383	-0.44731	4.18E-05
ZNF384	0.130106	0.026966
ZNF385C	-0.65606	0.005625
ZNF391	-0.26264	0.000882
ZNF394	0.203394	0.003422
ZNF395	-0.72114	1.21E-28
ZNF396	-1.28422	4.61E-07
ZNF397	-0.47576	7.45E-09

ZNF398	-0.31249	6.71E-06
ZNF407	0.25732	0.004062
ZNF408	0.260913	0.007672
ZNF41	0.20501	0.019195
ZNF414	0.40009	0.038436
ZNF419	-0.30072	0.000124
ZNF420	-0.27421	0.01197
ZNF425	-0.92641	2.32E-07
ZNF428	0.3648	0.035272
ZNF429	-0.36692	0.001943
ZNF431	-0.41447	0.000101
ZNF432	0.191495	0.012077
ZNF433	-0.27701	0.031599
ZNF436	-0.48666	1.88E-07
ZNF438	0.870454	2.90E-08
ZNF444	0.244571	0.024512
ZNF446	-0.6261	2.55E-07
ZNF449	-0.24612	0.00104
ZNF45	-0.18497	0.031701
ZNF451	0.280245	0.002734
ZNF462	0.296488	0.000399
ZNF469	-1.04837	0.005679
ZNF473	0.230825	0.000565
ZNF488	-1.9667	2.49E-23
ZNF491	-1.08776	0.031004
ZNF493	-0.29506	0.014166
ZNF496	0.358662	8.98E-06
ZNF502	-0.30752	0.012959
ZNF506	-0.55293	0.024319
ZNF510	-0.15505	0.031736
ZNF511	0.427274	4.39E-07
ZNF512B	-0.32342	0.002181
ZNF513	-0.40468	1.05E-05
ZNF514	-0.29139	0.002516
ZNF516	-0.48175	7.44E-06
ZNF517	-0.40661	0.00046
ZNF518A	-0.19775	0.005091
ZNF528	-0.67246	0.004526
ZNF530	0.321014	0.000578
ZNF532	-0.1143	0.035434
ZNF544	0.162856	0.028429
ZNF550	-0.19635	0.039562
ZNF551	0.234934	0.000611
ZNF554	-0.3179	0.026107
ZNF561-AS	-0.38325	0.002226
ZNF562	0.376118	4.11E-07
ZNF565	-0.35018	0.049554

ZNF568	-0.79067	0.003202
ZNF57	-0.25935	0.013474
ZNF571	-0.26777	0.029405
ZNF577	-0.3305	0.035684
ZNF579	0.603497	0.00688
ZNF580	-0.39438	0.002934
ZNF582-AS	0.495704	0.042063
ZNF583	-1.38641	0.043783
ZNF592	0.206762	0.000288
ZNF593	0.381792	0.019711
ZNF598	0.788671	1.33E-07
ZNF599	-0.34259	0.004954
ZNF605	-0.3448	4.18E-07
ZNF607	-0.29985	0.00062
ZNF609	0.168604	0.001203
ZNF615	-0.28789	0.000274
ZNF618	-1.0656	4.18E-11
ZNF620	0.664519	4.98E-05
ZNF621	0.192871	0.002936
ZNF622	0.24205	0.002455
ZNF623	-0.35017	1.25E-07
ZNF625-ZN	-0.38806	0.022239
ZNF627	-0.24792	0.032885
ZNF628	0.57952	0.009585
ZNF629	-0.24605	4.03E-05
ZNF639	0.177645	0.039864
ZNF652	-0.58842	1.51E-15
ZNF669	0.236566	0.006729
ZNF670	0.352173	0.016575
ZNF672	0.407319	1.64E-05
ZNF678	0.297911	0.009471
ZNF687	-0.32097	1.24E-06
ZNF689	0.269916	0.001028
ZNF691	-0.239	0.008607
ZNF696	0.308399	0.008744
ZNF697	1.50796	1.17E-31
ZNF7	-0.29703	3.12E-05
ZNF70	0.225415	0.039453
ZNF703	0.826549	8.58E-07
ZNF704	-1.42881	5.88E-12
ZNF706	0.339655	3.30E-09
ZNF710	0.300315	0.020932
ZNF711	-0.44032	7.66E-06
ZNF714	0.272713	0.012891
ZNF718	0.413515	2.89E-07
ZNF725P	-2.93827	0.023527
ZNF736	0.404197	0.019749

ZNF737	-0.72746	0.022062
ZNF740	-0.26051	4.78E-06
ZNF750	-1.42682	0.000334
ZNF75A	-0.1981	0.021665
ZNF761	-0.26257	0.006844
ZNF764	0.250328	0.016861
ZNF766	-0.32617	0.000243
ZNF767P	-0.31607	0.002619
ZNF772	-0.24636	0.00165
ZNF773	-0.42857	1.87E-05
ZNF774	-0.54539	3.99E-05
ZNF778	0.478177	7.48E-08
ZNF780A	-0.15811	0.038482
ZNF780B	-0.3052	0.025051
ZNF783	-0.36892	8.34E-05
ZNF785	-0.23406	0.010457
ZNF787	0.599808	2.34E-05
ZNF789	-0.2621	0.041734
ZNF79	0.231361	0.034684
ZNF8	-0.2829	7.37E-05
ZNF800	0.163886	0.026913
ZNF804A	-3.20632	0.011054
ZNF805	0.332261	0.041006
ZNF81	-0.24375	0.003786
ZNF812P	-3.46924	0.003229
ZNF815P	-0.47116	0.004832
ZNF821	-0.36355	0.003007
ZNF827	-0.21515	0.00232
ZNF829	-1.16334	0.017026
ZNF84	-0.20691	0.000968
ZNF841	0.291186	2.62E-06
ZNF846	-0.53127	0.000193
ZNF85	0.362491	0.005962
ZNF850	0.354568	0.001008
ZNF853	1.274331	2.48E-06
ZNF860	0.629256	5.52E-08
ZNF862	-1.28483	9.40E-05
ZNF865	0.492484	0.013996
ZNF878	0.677077	0.005039
ZNF887P	1.02657	0.000648
ZNF891	-0.27084	0.001567
ZNF91	-0.36279	0.001926
ZNF92	0.358046	0.001077
ZNFX1	-0.39556	0.01101
ZNHIT2	0.507993	0.032481
ZNHIT3	0.145232	0.012109
ZNHIT6	0.335045	5.49E-07

ZNRD1	0.33624	0.000426
ZNRD1ASP	-0.46844	0.010399
ZNRF2	-0.26614	0.013087
ZNRF2P1	1.651681	0.010491
ZP4	2.702543	1.06E-14
ZPR1	0.311231	1.53E-06
ZRANB1	-0.21218	0.022134
ZRSR2	0.249018	0.027586
ZSCAN12P1	1.140395	9.53E-05
ZSCAN22	0.299379	0.005749
ZSCAN23	-0.57969	0.000116
ZSCAN25	-0.2544	0.000619
ZSCAN31	-1.60692	6.71E-32
ZSCAN32	-0.17261	0.015415
ZSWIM4	0.400473	0.011502
ZSWIM5	-0.78436	2.85E-07
ZSWIM6	0.727156	3.92E-20
ZW10	0.23012	0.007012
ZWILCH	0.563284	1.38E-06
ZWINT	0.738517	1.06E-17
ZXDB	0.236328	0.025286
ZYG11B	-0.32121	0.000584
ZYX	0.23054	0.004696

Supplementary Table 4 - SCC6 differentially expressed genes between cetuximab and PBS.

Genes	log2FoldCh	pvalue
A1BG-AS1	-0.74105	0.004184
A2ML1	3.031981	2.84E-06
AACS	-0.25264	0.009431
AADAC	3.085607	1.98E-27
AADACL2-A	1.539981	0.031452
AADACP1	2.576761	2.34E-19
AAMDC	-0.76957	0.000555
AARSD1	0.270296	0.038229
AASDHPPT	0.543471	0.000112
AASS	-1.23959	0.001112
AATF	0.605308	4.97E-10
ABAT	-1.69722	1.07E-19
ABC12-492	-3.27583	0.007895
ABC13-474	-1.07726	6.11E-05
ABC7-4240	-2.39361	0.000183
ABCA11P	0.318564	0.023415
ABCA13	0.937425	0.001505
ABCA2	-0.54422	0.006601
ABCA4	-1.48073	0.00211
ABCA5	-0.49791	0.001828
ABCB1	1.719784	0.000555
ABCB10	0.409647	0.001014
ABCB11	3.793081	0.004287
ABCB6	-0.74482	0.003226
ABCC1	-0.3442	0.005791
ABCC3	-0.54956	6.64E-05
ABCC4	0.583231	0.017305
ABCD3	0.434227	0.014151
ABCD4	-0.38185	0.033231
ABCE1	0.6645	0.001043
ABCF2	0.476919	0.000511
ABCG2	-0.64431	0.025088
ABHD10	0.35894	0.008762
ABHD11	0.738361	5.77E-05
ABHD12	-0.30045	0.020782
ABHD17C	0.952434	3.54E-09
ABHD4	-1.56476	4.61E-19
ABHD5	0.720873	8.97E-05
ABL1	-0.59318	0.003162
ABL2	0.527053	0.007988
ABLIM1	-0.37793	1.73E-05
ABLIM3	-0.2252	0.003692
ABO	-0.53529	0.000626
ABT1	0.353796	0.008227

AC000123.	0.481083	0.007142
AC002066.	-0.95304	0.001553
AC002076.	-1.36533	0.012828
AC002116.	0.818394	0.01273
AC002128.	1.687207	5.04E-05
AC002310.	1.822527	0.023434
AC002454.	-0.82548	0.022202
AC002467.	0.626142	0.01622
AC003006.	-3.95887	0.006724
AC003088.	-3.36273	0.011043
AC003092.	3.534391	3.15E-09
AC003986.	-1.57868	0.035761
AC004381.	-2.70916	0.004925
AC004543.	1.75747	0.005131
AC004947.	4.595602	1.01E-05
AC004985.	-1.4894	0.033787
AC005062.	1.76319	2.40E-05
AC005077.	0.862196	0.009714
AC005083.	1.243279	2.95E-05
AC005224.	0.631531	0.034791
AC005253.	0.36143	0.009699
AC005329.	-3.3536	0.036284
AC005330.	-1.57474	3.67E-07
AC005517.	0.850733	0.00252
AC005519.	-0.5682	0.002087
AC005562.	-0.3248	0.029671
AC005592.	1.403388	5.26E-06
AC005757.	2.603087	0.025176
AC005863.	-0.80129	0.010419
AC005932.	-1.19889	0.0039
AC006262.	-1.22056	1.29E-08
AC006552.	-2.28428	4.28E-05
AC007193.	-1.18453	0.043651
AC007204.	-2.02345	0.03008
AC007228.	1.378471	0.042707
AC007246.	0.532589	0.000458
AC007318.	1.158661	0.027738
AC007326.	-0.86205	0.038802
AC007743.	-0.54075	0.00581
AC007773.	0.419033	0.038
AC008277.	-1.46324	0.013248
AC008440.	0.866925	0.049895
AC009133.	-1.17946	0.000344
AC009133.	0.652711	0.024447
AC009237.	-0.79014	0.041102
AC009487.	0.495322	0.023463
AC009495.	-1.06344	0.035303

AC009506.	-0.57024	0.044384
AC009948.	-0.43444	0.007553
AC010761.	0.864057	0.000137
AC010967.	1.887275	0.006563
AC010969.	3.475782	0.018947
AC011747.	-2.84254	0.025504
AC011747.	-3.08009	3.64E-05
AC011933.	2.470088	0.011387
AC012146.	0.816335	1.21E-05
AC012668.	-2.07573	0.015786
AC013268.	1.886129	0.036152
AC015849.	-1.30263	0.001015
AC015933.	-1.38326	0.007253
AC016582.	-2.51103	1.87E-07
AC016738.	3.059349	0.003758
AC016999.	1.646517	0.001895
AC017006.	-2.13454	0.035563
AC017104.	-2.34519	0.014479
AC020571.	0.855042	0.019486
AC020951.	-0.75085	0.002459
AC024560.	1.297859	9.23E-05
AC024592.	0.964073	0.027064
AC025335.	0.485466	0.014605
AC026150.	-2.66528	0.009274
AC058791.	2.341518	1.20E-15
AC063976.	-0.6279	0.011474
AC068134.	-0.78276	0.024757
AC068279.	1.271135	0.001155
AC068580.	1.588442	4.36E-06
AC069282.	0.405054	0.029349
AC073046.	-1.1016	0.00076
AC073052.	-1.0472	0.048434
AC073130.	-0.85592	0.000763
AC073410.	-1.2129	0.036991
AC073842.	3.028117	0.040334
AC079742.	6.217801	0.040455
AC079922.	0.678415	0.00378
AC091729.	-1.2449	0.032387
AC091729.	-0.77756	1.71E-07
AC092066.	-0.26924	0.035686
AC093162.	-1.05042	0.002639
AC093323.	0.339092	0.00322
AC093724.	-1.57463	2.48E-08
AC093818.	0.674841	0.000146
AC098820.	-2.9597	0.043068
AC098826.	5.235075	9.88E-26
AC099342.	2.013212	6.88E-05

AC100802.	2.313775	0.026843
AC104389.	1.370759	0.03344
AC104777.	2.224414	0.013732
AC105760.	-0.96721	0.008289
AC106786.	-3.42822	0.023785
AC108025.	2.051527	6.66E-06
AC108142.	-0.57295	0.002952
AC112229.	1.517645	1.98E-05
AC114812.	-1.84178	0.015612
AC116366.	-0.95122	0.015704
AC118754.	2.995506	0.008109
AC124789.	0.329397	0.036316
AC124861.	-1.40604	1.06E-05
AC127904.	-0.63957	0.037582
AC129778.	2.137607	0.000104
AC133785.	1.595112	0.009769
AC135048.	-1.15649	0.031421
AC137932.	1.045143	0.025843
AC138969.	-0.35717	0.01468
AC141586.	0.27628	0.021626
AC141928.	-1.52554	0.000144
AC142293.	1.772582	0.011205
AC144652.	-0.62851	0.009393
AC144831.	-0.95998	0.034468
AC145124.	-1.14853	0.020128
AC159540.	-0.4581	0.00101
AC159540.	-1.72567	5.29E-05
ACACB	-0.78193	0.000697
ACAD10	-0.28295	0.00819
ACAD11	-0.28185	0.00671
ACAD8	0.294386	0.000787
ACADL	-1.87013	1.90E-05
ACADM	0.393171	0.016443
ACAP3	-0.62411	0.023613
ACAT1	0.512407	0.003836
ACAT2	-0.75045	1.20E-09
ACHE	-2.93565	7.83E-06
ACKR2	-1.36677	2.23E-07
ACKR3	-4.82135	3.06E-41
ACO1	0.685217	5.86E-05
ACOT1	-1.0674	2.90E-05
ACOT2	-0.67763	0.000529
ACOT4	-0.50355	0.034575
ACOT7	0.352282	0.032098
ACOX2	3.971933	0.001618
ACOX3	-0.89478	2.66E-12
ACOXL	1.083416	1.40E-05

ACP1	0.326344	0.003626
ACP6	0.54411	0.000128
ACPP	1.122801	0.000137
ACR	-3.18443	0.009985
ACRBP	-1.93915	0.002319
ACSF2	-0.85981	6.71E-06
ACSL5	2.038886	1.20E-14
ACSM1	-1.72643	0.029204
ACSM3	-2.48119	1.11E-17
ACSS2	-0.36386	0.029432
ACTA2	-1.76533	0.000853
ACTA2-AS1	1.826975	0.014409
ACTBL2	-1.17617	4.00E-09
ACTC1	-5.07192	6.60E-06
ACTG1	-0.47747	0.000365
ACTG2	-4.78161	1.02E-11
ACTL6A	0.252445	0.024585
ACTL7B	3.394484	0.011526
ACTN1	-0.89612	3.27E-08
ACTN1-AS1	-3.24498	0.004246
ACTR3B	0.25401	0.008399
ACTR6	0.553358	0.001275
ACVR1	-0.3461	0.000718
ACVR1B	-0.2977	0.039019
ACVR2B	0.368299	0.009102
ACY1	0.534256	0.012289
ACYP1	0.499217	0.00487
ADA	-0.61975	0.000335
ADAM12	-1.1734	1.14E-15
ADAM18	-2.67863	0.011899
ADAM22	-0.62462	0.0015
ADAM23	-0.7223	7.59E-05
ADAM8	0.968589	0.008584
ADAMTS10	-2.14933	0.001957
ADAMTS12	-1.51394	1.68E-19
ADAMTS14	-1.15662	0.014743
ADAMTS15	-1.44699	9.38E-05
ADAMTS16	-1.22458	5.14E-06
ADAMTS18	1.566107	0.00921
ADAMTS20	-0.26573	0.046155
ADAMTS6	0.932088	4.26E-05
ADAMTS7	-2.92251	2.03E-05
ADAMTS9	0.967013	0.001206
ADAMTS9-.	1.827135	0.011878
ADAMTSL4	-1.23295	4.79E-05
ADAMTSL5	-1.21593	7.76E-05
ADAP1	0.67698	0.036835

ADAR	-0.19749	0.000645
ADARB2-AS	3.78199	0.016151
ADAT2	0.998453	1.34E-05
ADCY1	0.456529	0.002496
ADCY3	0.351243	0.006163
ADCY6	-0.48286	0.015347
ADCY9	-0.85526	2.42E-07
ADD1	-0.4237	5.99E-06
ADGRA2	-2.45609	2.98E-06
ADGRA3	0.473677	0.010179
ADGRB1	-2.20122	0.043929
ADGRB2	-1.91656	4.79E-09
ADGRE2	1.396824	1.47E-07
ADGRE5	0.688722	2.41E-09
ADGRF1	1.560304	2.52E-05
ADGRF2	2.000232	2.03E-05
ADGRF5P1	-1.02983	0.000728
ADGRG1	-0.81426	0.000532
ADGRG3	-1.76845	0.026905
ADGRL3	-1.67278	5.87E-07
ADGRV1	-1.13198	0.00015
ADH5	-0.21275	0.010037
ADHFE1	-0.66649	0.002012
ADIPOR1	0.243744	0.000356
ADIRF	1.310993	5.09E-07
ADM	-1.41237	3.53E-15
ADM2	-1.47442	1.98E-05
ADNP	-0.25226	0.003337
ADO	0.326949	0.000491
ADORA1	-1.2316	0.026563
ADORA2A	-2.42332	0.004961
ADORA2A-A	-0.9414	0.0095
ADORA2B	0.964566	0.000302
ADPGK	0.246549	0.003782
ADPRH	-2.01134	0.000235
ADPRHL1	-0.69901	0.006434
ADRA1B	-1.45718	2.26E-11
ADRB2	1.430831	4.09E-05
ADRM1	0.397921	0.022926
ADSL	0.241184	0.030038
ADSS	0.266763	0.047199
AEN	0.914092	7.60E-12
AF001548.5	-1.90457	0.009697
AF011889.5	-0.5007	0.008448
AF127577.1	3.106179	0.003261
AFAP1	-0.42674	0.000724
AFAP1L2	-1.6259	2.58E-27

AFDN	0.310471	0.04477
AFG3L2	0.274731	0.005787
AFMID	0.333098	0.016642
AGAP1	0.2861	0.007144
AGAP11	0.735574	0.026063
AGAP6	0.227459	0.021194
AGBL2	-1.00072	0.011761
AGBL5	-0.40413	0.011004
AGGF1P2	-1.00821	0.049194
AGK	0.265188	0.001752
AGMAT	0.908454	1.39E-07
AGO2	0.612707	5.68E-07
AGO4	-1.11085	9.79E-11
AGPAT1	-0.49337	0.037977
AGPAT4	-1.53379	3.53E-33
AGPAT5	0.833604	2.71E-06
AGR2	1.899234	5.16E-07
AGRN	-0.7753	0.047269
AGTPBP1	0.467681	0.030549
AHCTF1	0.629205	3.77E-05
AHCY	0.502461	1.03E-06
AHDC1	-0.95388	0.000988
AHR	0.531265	0.034871
AHRR	-1.09259	8.36E-07
AHSA1	0.550367	0.000111
AIFM1	0.296145	0.028035
AIG1	0.606166	1.75E-09
AIMP1	0.482923	0.002288
AJ239321.3	7.298958	3.77E-13
AJAP1	2.397303	8.62E-11
AJUBA	-1.03002	2.66E-07
AK2	0.539739	2.80E-09
AK3	0.401691	0.002959
AK3P5	1.274623	0.009409
AK4	0.450697	0.038179
AK6	0.668815	2.12E-08
AK8	-1.14718	0.013161
AKAP1	0.619114	1.53E-15
AKAP11	0.380506	0.006123
AKAP12	0.894215	4.96E-17
AKAP2	-1.30172	3.88E-12
AKAP5	-1.60498	5.58E-18
AKAP6	-0.50401	0.008794
AKAP8	0.387289	0.000971
AKIP1	-0.40001	2.33E-05
AKIRIN1	0.276607	0.012615
AKR1C1	0.720209	0.000212

AKR1C3	-0.71255	0.000134
AKR7L	0.84845	0.024408
AKT3	-0.54672	1.88E-06
AL162759.:	-1.45459	5.57E-05
AL591893.:	-0.62687	0.000604
ALAD	-0.44979	0.002305
ALAS1	0.583533	4.98E-16
ALDH18A1	0.18298	0.009033
ALDH1A3	0.829074	1.17E-06
ALDH1L1	-0.50658	0.03601
ALDH1L2	-0.84496	0.004504
ALDH2	-0.87272	0.000191
ALDH3A1	-0.96793	0.009722
ALDH3B1	-0.50738	0.00666
ALDH4A1	-1.28568	2.36E-07
ALDH7A1	-1.0114	8.68E-09
ALDH9A1	0.2887	0.003868
ALG1	0.390207	0.024492
ALG13	0.466519	0.00054
ALG1L	-1.08871	7.35E-08
ALG2	0.221472	0.013485
ALG5	0.364478	0.01022
ALG6	0.401499	0.01603
ALK	1.3081	0.022932
ALKBH1	0.285499	0.005506
ALKBH2	0.432846	0.006521
ALKBH8	0.374078	0.005306
ALOX5	1.241448	9.44E-05
ALOX5AP	-1.49269	0.000145
ALOXE3	1.144912	2.79E-08
ALPK2	-3.95296	9.01E-08
ALPP	-1.20787	8.10E-11
AMD1	0.556676	0.002652
AMD1P4	0.847098	0.043595
AMIGO2	-0.9943	4.50E-23
AMIGO3	-4.14033	0.003069
AMMECR1	0.785119	3.87E-06
AMN1	0.707418	8.75E-05
AMPD3	0.563618	8.04E-06
AMTN	0.93009	0.011125
AMZ1	-1.03211	0.004463
AMZ2P1	-0.51509	0.005944
ANAPC1	0.311232	0.028778
ANAPC10	0.441788	0.005262
ANAPC13	0.46453	0.000101
ANAPC15	-0.26772	0.005467
ANAPC4	0.417993	0.020435

ANAPC5	0.254934	0.007501
ANAPC7	0.381667	0.00141
ANG	-0.99496	0.000932
ANGEL2	0.314871	0.012886
ANGPTL4	1.759845	1.40E-06
ANK1	-2.02849	9.98E-05
ANK3	-1.09518	0.000266
ANKDD1A	0.335783	0.047671
ANKH	-0.24152	0.014019
ANKLE1	-0.67253	0.049108
ANKLE2	-0.26714	0.027315
ANKRD1	-1.80201	5.98E-08
ANKRD17	0.274538	0.009127
ANKRD18A	0.648252	0.00201
ANKRD18B	0.961409	5.24E-07
ANKRD19P	0.871173	0.031927
ANKRD2	-2.3399	4.35E-13
ANKRD20A	1.023926	3.64E-05
ANKRD20A	-2.07581	0.026432
ANKRD20A	0.824087	0.005271
ANKRD20A	1.222736	0.000457
ANKRD22	1.875144	5.03E-08
ANKRD24	-2.28144	0.002212
ANKRD30B	6.176978	2.54E-11
ANKRD34B	-4.58213	1.78E-16
ANKRD37	0.493933	0.02924
ANKRD40	0.163317	0.025418
ANKRD46	0.378225	0.011829
ANKRD50	-0.30287	0.001309
ANKRD52	-0.62997	0.002763
ANKRD6	-0.83737	0.033914
ANKRD65	-1.02893	5.23E-05
ANKRD7	3.362016	0.008125
ANKRD9	-0.9207	0.002496
ANKS6	-0.24653	0.04988
ANO1	0.456096	0.000198
ANO1-AS1	4.509483	0.031974
ANO2	-0.78305	0.03629
ANO6	-0.80508	1.20E-12
ANOS1	-3.4132	4.11E-23
ANP32A	0.29157	0.006638
ANP32B	0.334062	0.000387
ANPEP	1.280111	0.012107
ANTXR1	-1.58341	9.80E-17
ANTXR2	1.624673	5.88E-09
ANXA1	1.080184	8.32E-08
ANXA10	3.323234	4.36E-13

ANXA11	0.409371	0.02403
ANXA2R	-0.90964	0.021175
ANXA4	-0.58456	2.07E-05
ANXA5	-0.57442	1.13E-09
ANXA6	-0.96764	4.24E-05
ANXA8	-1.22525	1.53E-09
ANXA8L1	-1.42468	1.36E-10
AOC2	0.571805	0.012445
AOX1	0.444156	0.024265
AP000240.	1.842908	0.000853
AP000322.	4.483867	2.26E-05
AP000432.	-0.75773	0.018778
AP000648.	0.367122	0.049414
AP000662.	-0.64229	0.036725
AP000692.	2.857692	3.65E-06
AP000695.	-0.74999	0.001137
AP000892.	-2.69266	1.30E-10
AP001048.	-3.18062	0.026525
AP001062.	-0.61111	0.007068
AP001258.	-1.16788	3.66E-06
AP001347.	-0.95525	0.02449
AP001372.	-0.39382	0.033711
AP001615.	6.247103	0.01915
AP001619.	2.138877	0.045392
AP002954.	-1.70348	0.00044
AP003068.	0.941846	1.01E-10
AP003419.	0.782376	0.002545
AP005901.	3.138409	1.40E-22
AP1AR	0.478086	0.006185
AP2A1	-0.64963	0.000238
AP2M1	-0.26398	0.001012
AP3B2	-0.35704	0.041013
AP3D1	-0.19066	0.036865
AP3M2	0.293313	0.008126
AP4B1	0.344922	0.003765
AP4M1	-0.25488	0.032858
APAF1	-0.84629	2.24E-12
APBA1	-2.48325	7.19E-05
APBB1	-0.86353	0.016133
APBB2	-0.84196	0.000209
APCDD1L	-1.34223	1.31E-05
APEX2	0.472135	0.000179
APH1B	-1.51225	1.41E-19
API5	0.291489	0.045593
APIP	0.616832	3.62E-06
APLP1	-1.96743	1.47E-07
APOBEC3A	1.940528	0.000823

APOBEC3B	0.453596	0.016226
APOE	-1.44365	0.004989
APOL1	-0.93254	0.011874
APOL2	-0.59846	2.04E-07
APOL3	-1.24414	0.013143
APOO	0.424974	0.002135
APOOL	0.370857	0.009143
APP	-0.18721	0.033501
APPL2	-0.20358	0.030895
AQP1	-3.59114	0.004935
AQP10	-2.77397	0.038953
AQP3	1.632258	3.95E-07
AQP5	3.012038	0.01168
AQR	0.329726	0.01565
ARC	1.346391	0.018012
AREG	3.05744	4.59E-15
ARF1	0.161155	0.028893
ARF5	-0.46976	0.006938
ARF6	0.215279	0.001902
ARFGAP1	-1.06793	2.73E-05
ARFGEF3	0.275074	0.043472
ARFIP1	0.377028	0.005549
ARG2	0.377773	0.048161
ARHGAP17	-0.28846	0.000467
ARHGAP18	0.984044	1.00E-08
ARHGAP21	-0.41068	0.000271
ARHGAP26	-1.50096	2.18E-21
ARHGAP29	0.412827	0.020809
ARHGAP31	-2.55311	1.30E-09
ARHGAP31	-1.98005	0.035306
ARHGAP32	0.320521	0.008203
ARHGAP33	-1.39279	7.46E-05
ARHGAP35	-0.81721	5.29E-14
ARHGAP4	-2.35716	0.000165
ARHGAP42	-0.66895	8.03E-05
ARHGAP44	-0.78457	0.000279
ARHGAP45	-1.05813	9.48E-05
ARHGAP5-/	0.567484	0.032147
ARHGDIB	-0.51427	6.41E-05
ARHGEF1	0.773506	0.006577
ARHGEF10	-0.54929	3.40E-05
ARHGEF10I	-0.65856	0.005861
ARHGEF17	-1.32039	2.77E-06
ARHGEF19	-1.15167	0.00012
ARHGEF2	1.08858	1.68E-06
ARHGEF25	-0.60164	0.013873
ARHGEF37	-0.53518	0.000345

ARHGEF4	1.573049	1.28E-09
ARHGEF40	-1.58948	1.27E-07
ARID1A	-0.47424	0.002248
ARID1B	-0.46487	2.70E-07
ARID3B	1.417586	8.14E-30
ARID5A	-1.10503	0.008129
ARID5B	-1.68165	1.36E-22
ARIH2	0.277641	2.70E-05
ARL10	-0.39457	0.023954
ARL14	1.508649	4.83E-08
ARL14EP	0.350968	0.01132
ARL14EPL	1.352775	0.0049
ARL15	-1.27386	7.41E-09
ARL3	-0.62483	0.001046
ARL4A	-0.61343	0.001126
ARL4C	-0.42045	1.03E-07
ARL4D	-1.25794	1.51E-05
ARL5A	0.495869	0.000634
ARMC10	0.372745	0.005984
ARMC9	-0.76351	4.72E-12
ARMCX3	-0.41563	9.17E-05
ARMT1	0.425378	0.014877
ARNT	-0.73699	1.05E-09
ARNT2	1.741519	9.07E-14
ARNTL2	1.238383	5.18E-08
ARNTL2-AS	1.618066	8.77E-06
ARPC1A	0.532086	0.000259
ARPC5	-0.26843	0.006426
ARPC5L	0.541021	6.67E-07
ARPIN	-0.47581	0.002267
ARRB1	-0.54402	0.008577
ARRB2	0.451354	0.001986
ARRDC4	0.709412	1.27E-05
ARSA	-0.90555	0.020574
ARSB	-0.5832	4.11E-10
ARSD	-1.11693	8.59E-07
ARSG	-0.49065	5.50E-05
ARSI	-1.38059	2.69E-07
ARSJ	0.407284	0.004053
ARV1	0.833445	0.000824
ARVCF	-0.95025	0.002041
AS3MT	-1.18442	4.86E-06
ASAH2	0.43857	0.047716
ASAH2B	0.526857	0.000363
ASAP3	-1.44668	2.21E-13
ASB1	0.482437	0.000671
ASB13	0.301786	0.01245

ASB16-AS1	-0.43567	0.001493
ASB3	0.30169	0.029755
ASB9	1.325048	7.12E-07
ASCC2	-0.35201	0.007667
ASIC1	-0.53924	0.013488
ASIC3	-1.46706	0.00098
ASIC4	1.364782	0.008715
ASNSD1	0.653632	3.59E-05
ASPH	0.542674	9.67E-07
ASPHD2	-0.59985	2.97E-05
ASXL1	-0.15374	0.036462
ATAD2	0.512756	0.003524
ATAD2B	-0.35134	0.001746
ATAD3B	0.507588	0.048606
ATAD5	0.376899	0.024793
ATAT1	-0.48706	7.14E-05
ATE1	0.464926	0.000558
ATF2	-0.36089	0.008412
ATF3	-0.72635	0.0003
ATG16L1	0.675792	5.90E-11
ATG3	0.306753	0.011493
ATG7	0.552966	8.41E-05
ATG9A	-0.53947	0.003662
ATIC	0.417129	0.002031
ATL1	-0.65085	3.66E-07
ATL2	0.562681	0.000917
ATL3	-0.20169	0.037994
ATOH8	-4.0058	0.0013
ATOX1	0.292525	0.000569
ATP10B	1.450363	0.002037
ATP10D	-1.79769	2.04E-20
ATP13A2	-0.65239	0.045122
ATP1A1	0.292284	9.86E-05
ATP1B1	-0.52588	0.005236
ATP1B3	0.452052	0.002991
ATP2A3	-1.00506	0.002688
ATP2C2	0.842491	1.20E-08
ATP5A1	0.32308	0.011177
ATP5B	0.448939	5.96E-05
ATP5E	0.354576	0.006857
ATP5F1	0.335887	0.011566
ATP5G1	0.683539	4.21E-08
ATP5G3	0.431552	0.005482
ATP5H	0.28168	0.003221
ATP5I	0.335567	0.006955
ATP5J	0.381859	0.023717
ATP5L	0.245516	0.037262

ATP5O	0.388988	0.004456
ATP6AP1	-0.51341	0.000236
ATP6V0A2	0.290579	0.005918
ATP6V0D2	1.348359	1.80E-09
ATP6V1A	-0.43354	0.001999
ATP6V1C1	0.317214	0.023505
ATP6V1D	0.400809	0.001283
ATP6V1G2-	0.718723	0.000397
ATP6V1H	0.210732	0.021298
ATP7B	-0.63826	0.010341
ATP8B1	-0.38766	0.007465
ATP8B2	-1.16549	1.53E-06
ATP8B3	-1.51549	6.64E-07
ATP9A	-0.5754	2.59E-07
ATPAF1	0.379665	0.013009
ATR	0.425808	0.018285
ATRIP	0.620512	0.008972
ATRN	-0.68787	1.05E-11
ATXN1	-1.63219	1.65E-23
ATXN1L	-0.24263	0.0404
ATXN8OS	2.131201	0.040911
AUNIP	0.340366	0.032178
AUP1	0.285833	0.039159
AURKC	-0.67869	0.035778
AUTS2	-1.80234	6.71E-11
AVL9	0.278071	0.006101
AVPI1	0.812529	1.61E-11
AXIN2	-1.83805	3.32E-05
AXL	-0.57141	0.000147
AZGP1	2.780866	0.012182
AZIN2	-0.96652	0.003271
B2M	0.377228	0.037877
B3GALT5	1.14356	0.009928
B3GLCT	-0.79526	3.05E-12
B3GNT2	0.622756	3.03E-09
B3GNT3	1.893456	2.70E-17
B3GNT4	-0.52801	0.023408
B3GNT5	0.898014	4.69E-11
B3GNT7	-0.82842	0.001859
B3GNT8	-1.38143	0.003776
B3GNT9	-1.26558	5.38E-05
B4GALNT1	-1.51433	3.29E-07
B4GALT1	-0.51768	1.07E-13
B4GALT2	-0.52057	0.031127
B4GALT4	0.542266	0.010987
B4GALT5	0.20432	0.009565
B4GALT6	0.920406	2.39E-05

B4GAT1	-0.65452	0.00092
B9D1	-0.46031	0.025435
B9D2	-0.45061	0.021285
BAALC-AS1	-0.72016	0.004614
BACE1	-1.13329	2.12E-08
BACE2	-0.85169	0.0001
BACH1	0.667351	0.00021
BACH2	-2.30933	9.20E-16
BAG1	0.788398	4.01E-18
BAG2	0.560599	0.002748
BAG3	0.477834	0.021083
BAG4	0.309916	0.022239
BAIAP2-AS:	-1.24941	1.37E-07
BAIAP2L2	1.510262	0.007154
BAMBI	-2.26541	4.32E-17
BANCR	-3.33363	0.000705
BANK1	-0.91725	0.042923
BARX1	-2.08733	1.39E-10
BARX2	0.908211	0.000291
BASP1	-0.55635	0.000261
BATF	-2.04261	0.006131
BATF2	1.245952	0.01568
BAZ1A	0.765987	2.65E-05
BAZ2A	0.259549	0.039529
BAZ2B	-0.72524	4.25E-10
BBC3	-1.28129	0.049516
BBOF1	-1.18449	2.73E-11
BBOX1-AS1	-0.26862	0.015391
BBS1	-1.2644	4.40E-06
BBS10	0.46811	0.008539
BBS12	-0.49084	0.003555
BBS2	-0.25476	0.011265
BBS4	-0.29756	0.02703
BBS9	-0.77405	1.17E-08
BCAM	-1.07748	2.50E-05
BCAP29	0.546676	0.000406
BCAP31	0.360172	0.00067
BCAR3	0.491132	1.06E-05
BCAS2	0.472821	0.001325
BCAS3	-0.89682	4.90E-06
BCAS4	-0.91704	6.50E-07
BCAT1	-0.4511	0.022714
BCCIP	0.896513	5.98E-07
BCL10	0.756402	4.18E-05
BCL11B	0.652194	0.021895
BCL2A1	3.724863	2.07E-06
BCL2L11	-0.70837	0.000109

BCL2L13	-0.43455	1.94E-07
BCLAF1	0.44411	0.000222
BCOR	0.197722	0.024217
BCR	-0.53396	5.70E-06
BCRP2	-0.99062	0.0006
BCS1L	0.558451	0.000329
BDH2	-0.6935	5.75E-05
BDKRB2	-0.93138	1.75E-05
BDNF	-2.54066	1.05E-14
BDNF-AS	-2.30404	5.12E-06
BDP1	0.263327	0.028672
BEAN1	-1.44713	0.000407
BECN1	0.281027	0.000486
BEGAIN	-0.85997	0.033839
BEND3P1	-1.40172	0.031573
BEND3P3	-1.10095	0.014282
BEND6	-1.19586	3.00E-07
BEND7	-1.56701	1.17E-30
BEX3	-0.51739	9.97E-05
BHLHA15	-1.10214	0.012341
BHLHB9	-0.52048	0.000425
BHLHE40	-0.47665	0.028563
BHLHE41	1.404787	0.001765
BICC1	-1.07253	1.01E-09
BICDL1	-0.52723	0.024115
BICDL2	0.511214	0.023867
BID	0.256694	0.001833
BIN2	-2.23823	0.00374
BIN3	0.275145	0.042253
BIRC3	1.21547	0.000483
BIRC7	-3.31817	0.000685
BIVM	0.434256	0.000394
BLACAT1	0.62136	0.02323
BLM	0.480497	0.001671
BLVRB	-0.70254	0.001517
BLZF1	0.906493	8.42E-05
BMF	-1.75695	5.43E-11
BMI1	0.296706	0.032414
BMP1	-1.64417	4.01E-09
BMP4	-1.65264	1.43E-09
BMP6	1.888657	3.61E-14
BMP8B	-0.39627	0.02935
BMPR1A	-0.30008	0.007556
BMPR2	-1.47064	1.10E-28
BMS1	0.370798	0.005315
BMT2	-0.44098	0.017481
BNC1	0.562091	0.000547

BNC2	-1.76498	0.001357
BNIP3P2	4.212525	0.006682
BNIP3P3	3.4521	0.030828
BNIP3P4	4.836752	1.27E-14
BNIP3P6	4.2367	0.005625
BNIP3P7	3.457837	0.002972
BOK	-1.21454	0.018201
BOLA2	0.545145	0.00504
BOLA2B	0.770856	1.96E-07
BOLA2P2	1.246995	0.015219
BOLA3	0.669332	5.99E-06
BOLA3-AS1	-0.97889	0.002801
BOP1	0.608895	0.008818
BORCS6	-0.8143	0.041632
BORCS7	0.402292	0.015002
BPGM	-1.04853	6.51E-07
BPHL	-0.58281	2.20E-05
BPNT1	0.405849	0.000577
BRAP	0.259881	0.005067
BRCC3	0.270807	0.049438
BRD2	0.227074	0.035759
BRD3	-0.24229	0.043585
BRD7	0.332156	0.022896
BRD9	0.307697	0.017837
BRI3	-0.37153	0.004295
BRI3BP	0.505225	0.001186
BRICD5	-0.80431	0.029435
BRIX1	0.977001	9.84E-09
BROX	0.314765	0.030663
BRPF3	-0.28251	0.02469
BSN	-0.77491	0.013315
BSPRY	1.380761	4.98E-13
BST2	-1.94192	0.024147
BTBD1	0.419997	0.000714
BTBD10	0.248994	0.025311
BTBD19	2.503698	2.57E-15
BTBD2	-0.54815	0.010296
BTBD7	0.312589	0.011777
BTC	1.204058	4.91E-08
BTD	-0.85126	1.05E-07
BTF3	0.441941	0.000859
BTF3P7	3.438087	0.010038
BTG1	1.158619	4.62E-08
BTG2	-0.87553	3.56E-07
BTG3	0.480718	0.00022
BTN2A2	-1.1494	9.79E-11
BTN2A3P	-0.41606	0.009728

BTN3A1	-1.12705	2.84E-05
BTN3A3	-1.83898	0.000248
BVES	-1.42883	1.58E-10
BVES-AS1	-1.42719	0.007244
BX322557.	-0.67574	0.000949
BX470102.:	1.751403	0.007127
BYSL	0.424492	6.41E-05
BZW1	0.851101	6.49E-07
BZW1P2	0.724612	0.000212
BZW2	0.346265	0.028644
C10orf55	1.687211	2.00E-05
C10orf67	-1.36259	1.62E-11
C10orf91	0.864486	0.015364
C11orf1	0.410901	0.001677
C11orf49	-0.8313	4.18E-05
C11orf54	0.289951	0.0211
C11orf57	0.333189	0.005973
C11orf58	0.232358	0.033844
C11orf63	-0.50706	0.002847
C11orf70	-0.61728	0.015308
C11orf80	-0.25397	0.010966
C11orf91	1.574831	0.025525
C11orf95	-0.62981	0.00808
C11orf96	1.345899	0.028575
C11orf98	0.608766	4.36E-06
C12orf29	0.568597	8.58E-05
C12orf45	0.363909	0.006598
C12orf49	-0.13026	0.045111
C12orf54	-2.47634	1.93E-05
C12orf65	-0.3359	0.000168
C12orf66	0.313905	0.032694
C12orf73	0.320586	0.005081
C14orf1	-0.38357	0.001153
C14orf119	0.316431	0.015652
C14orf132	-2.1801	0.00025
C14orf159	-0.26253	0.020307
C14orf166	0.340666	0.01694
C14orf2	0.473702	0.0018
C14orf28	-0.78903	0.000556
C14orf37	-1.55887	9.88E-07
C15orf39	0.481832	0.021951
C15orf41	0.436923	0.000402
C15orf48	1.727181	7.97E-08
C15orf62	1.263951	5.51E-06
C15orf65	-1.22098	4.56E-05
C16orf45	-0.7862	6.54E-05
C16orf52	1.014119	2.60E-11

C16orf58	-0.51147	0.026246
C17orf100	-0.51701	0.010353
C17orf58	0.504669	0.000757
C17orf67	-0.33491	0.03361
C17orf75	0.26042	0.031184
C17orf97	-0.6455	0.001214
C18orf21	0.429031	0.002694
C18orf8	0.248894	0.031074
C19orf44	0.320209	0.004536
C19orf53	0.389803	6.25E-05
C19orf66	-0.93936	6.26E-05
C1D	0.51747	0.009796
C1GALT1	0.350867	0.00959
C1orf109	0.416248	8.05E-06
C1orf116	1.798498	0.000434
C1orf143	-1.76546	0.006478
C1orf145	-1.32068	0.003113
C1orf198	-0.50382	0.000325
C1orf204	-0.97191	0.007903
C1orf21	-1.2671	8.13E-11
C1orf27	0.386641	0.006764
C1orf50	0.196559	0.033923
C1orf61	1.417789	3.23E-09
C1QBP	0.685829	9.95E-05
C1QL1	-1.42739	0.000508
C1QL3	0.736662	8.83E-05
C1QL4	-0.85164	0.040594
C1QTNF2	-1.97431	8.59E-06
C1QTNF3	-1.88978	9.09E-08
C1QTNF6	-1.47726	1.88E-08
C1R	-3.21626	1.08E-10
C1RL	-0.85374	0.000161
C1S	-2.36461	2.08E-16
C20orf194	-1.16185	3.52E-10
C20orf197	2.392739	2.90E-07
C20orf24	0.532107	3.32E-05
C20orf96	-0.58904	0.00031
C21orf2	-0.90902	0.00375
C21orf58	-0.74468	0.000182
C21orf59	0.577793	3.14E-07
C21orf91	-0.34377	0.038028
C22orf23	-1.08125	0.000198
C2CD2	0.444293	0.000645
C2CD2L	1.022352	1.40E-05
C2CD3	-0.51736	4.51E-06
C2orf16	0.36893	0.040256
C2orf27A	-1.37539	4.30E-17

C2orf42	0.277225	0.031624
C2orf54	1.52181	1.75E-07
C2orf68	-0.32193	0.006055
C2orf69	0.409584	0.013002
C2orf72	-1.19579	0.012709
C2orf91	-1.10243	0.041777
C3	0.66493	0.000422
C3orf33	0.658196	0.000403
C3orf52	0.499092	0.001276
C3orf67	0.912549	1.09E-06
C4BPB	-1.48413	4.20E-18
C4orf19	-1.13451	8.41E-05
C4orf26	-2.89357	1.17E-29
C4orf32	0.521964	6.67E-05
C5	-1.04887	0.001206
C5AR2	2.227051	1.14E-07
C5orf15	-0.35651	0.006332
C5orf30	0.38711	0.006756
C5orf38	-0.92951	0.00211
C5orf42	-0.30611	0.006797
C5orf49	-3.22891	2.50E-06
C5orf51	0.379573	0.01734
C5orf56	-0.53519	0.011562
C5orf66-AS	1.75971	0.038058
C6orf106	-0.48906	0.000131
C6orf136	0.277273	0.013713
C6orf141	0.399298	0.000735
C6orf15	1.371493	0.000144
C6orf203	0.502971	1.89E-05
C6orf3	-0.6857	0.008045
C7orf31	-0.91255	2.26E-10
C8orf31	-0.68549	0.046291
C8orf33	0.260003	0.000607
C8orf4	-1.94414	0.000327
C8orf46	-1.56818	1.59E-05
C8orf48	-0.88419	4.02E-12
C8orf59	0.410233	0.01127
C8orf76	0.29124	0.029149
C9orf170	-2.65021	0.014502
C9orf3	-0.93421	4.92E-29
C9orf40	0.555776	2.40E-05
C9orf47	-1.0239	0.008739
C9orf78	0.181594	0.015856
C9orf85	0.260345	0.034917
CA12	0.972117	0.041886
CA13	0.770068	0.002983
CA2	1.280037	1.00E-05

CA5B	-0.38756	0.004186
CAAP1	0.492042	8.68E-05
CAB39	0.540324	0.001423
CAB39L	0.439888	0.006119
CABIN1	-0.52582	0.001042
CABLES2	-0.37429	0.007866
CABYR	-1.06844	1.47E-09
CACFD1	-1.40462	0.016476
CACHD1	0.222759	0.019419
CACNA1D	-1.1669	0.027606
CACNA1G	-1.22705	0.001614
CACNA1H	-1.0879	0.008931
CACNA2D3	-3.09396	0.000327
CACNB2	0.779081	0.021713
CACNB3	-1.32501	3.46E-06
CACNB4	0.568558	0.044562
CACNG1	-2.71444	0.011565
CACNG4	-3.0421	4.35E-27
CACNG6	-1.47307	3.08E-06
CACYBP	0.520934	0.001169
CADM1	-1.96899	2.61E-17
CADM2	1.253789	0.036076
CADM4	-1.13621	0.000963
CALB1	1.9077	5.83E-11
CALB2	2.978896	1.35E-32
CALCOCO1	-0.50486	0.010527
CALD1	-2.12403	2.79E-35
CALM2	0.351755	0.014974
CALM3	-0.414	0.017667
CALML3-AS	-0.95516	0.000873
CALR	-0.30132	0.035575
CAMK1D	-1.16773	6.98E-06
CAMK1G	1.872668	3.24E-06
CAMK2N1	0.200024	0.007325
CAMK2N2	-1.59432	4.54E-06
CAMK4	-1.23348	0.01079
CAMKV	1.936676	0.046724
CAP2	-0.85545	2.46E-21
CAP2P1	-1.45484	0.041234
CAPN10-AS	-0.4049	0.013569
CAPN13	-4.83083	8.35E-06
CAPN2	-0.47823	2.55E-08
CAPN5	-1.39506	3.84E-05
CAPN8	1.925058	0.000174
CAPRIN1	0.256925	0.008486
CAPRIN2	0.773485	1.71E-07
CAPS	-0.63205	0.023912

CAPS2	-0.44898	0.038411
CAPZA1	0.516947	0.000777
CAPZB	-0.48327	7.37E-09
CARD14	-1.00586	0.01505
CARD19	-0.46917	0.035712
CARD6	0.641905	0.001015
CARD8	-0.20937	0.030872
CARF	-0.32355	0.029279
CARHSP1	-0.58093	0.000119
CARNMT1	0.931664	2.02E-05
CASC10	-2.2658	4.96E-41
CASC15	-0.94319	3.08E-07
CASC19	2.684674	0.003311
CASC8	1.278979	9.68E-06
CASP2	-0.31987	1.46E-05
CASP4	0.651975	3.89E-05
CASP6	-0.3668	0.006019
CASP8	0.429311	0.011184
CASQ2	-3.1232	0.01038
CASZ1	-0.58216	0.000461
CATSPERG	-0.63935	0.02297
CAV1	-1.64089	2.37E-23
CAV2	-0.92254	2.64E-15
CBFA2T2	-0.68838	1.58E-07
CBFB	0.348594	0.006668
CBLB	-0.87335	8.59E-08
CBR3-AS1	-0.82427	6.47E-05
CBR4	0.683281	0.000257
CBS	-0.83679	0.01696
CBSL	-0.63398	0.000919
CBWD1	0.506644	3.03E-05
CBWD3	0.409273	0.002049
CBWD5	0.319366	0.032916
CBX1	-0.8019	1.08E-12
CBX2	-0.95009	0.002888
CBX3	0.430035	0.006738
CBX5	-0.23871	0.047924
CCAR1	0.404179	0.022099
CCAT1	1.175224	5.64E-06
CCDC102A	-0.97959	5.22E-05
CCDC113	-0.62144	0.003237
CCDC125	0.258594	0.021667
CCDC126	-0.50676	6.61E-05
CCDC136	-1.06407	8.93E-06
CCDC138	0.375244	0.040212
CCDC144A	-0.64738	0.000412
CCDC144B	-0.81728	5.40E-06

CCDC144CI	-1.13076	2.49E-09
CCDC144N	-1.80641	4.24E-05
CCDC148	-1.38328	6.91E-05
CCDC149	-0.41466	0.01842
CCDC150	0.609478	0.000301
CCDC150P:	0.498944	0.046544
CCDC157	-0.61768	0.007998
CCDC159	-0.42323	0.035344
CCDC162P	-0.57217	4.43E-06
CCDC167	-0.38721	0.037448
CCDC177	3.099794	0.042654
CCDC180	-1.00448	0.015966
CCDC186	-0.32645	0.00567
CCDC190	-3.87818	0.000648
CCDC191	-0.47061	0.025182
CCDC28B	-0.81486	0.033092
CCDC30	-0.48419	0.014774
CCDC40	-1.20928	3.45E-05
CCDC47	0.435539	0.001668
CCDC50	-0.50722	0.000813
CCDC58	0.824864	1.67E-05
CCDC59	0.562188	0.000871
CCDC66	0.344247	0.024389
CCDC71L	0.532746	0.005122
CCDC74B	-2.42595	0.001724
CCDC80	-0.84239	7.08E-12
CCDC85A	-1.66126	0.00091
CCDC86	0.687642	2.04E-05
CCDC88C	0.235854	0.036191
CCDC89	-2.13535	0.026552
CCDC92	-0.8227	8.73E-09
CCDC97	-0.5	0.018178
CCL20	3.774273	2.96E-14
CCL22	1.914985	1.37E-05
CCL28	-0.38122	0.001326
CCL5	-1.5559	0.000688
CCNA1	2.475595	1.49E-16
CCNB3	1.006254	0.014802
CCNC	0.620715	0.000434
CCND1	0.559371	1.23E-09
CCNE1	1.08184	1.41E-10
CCNE2	0.342459	0.013318
CCNG2	-0.56469	0.000112
CCNH	0.540931	0.00121
CCNJ	0.462058	4.44E-05
CCNJL	-0.54491	0.000392
CCNL1	0.370266	0.027854

CCNT1	0.425587	6.86E-06
CCNY	-0.24278	0.005133
CCNYL1	0.372697	0.030095
CCPG1	-0.45809	0.004247
CCR3	-2.16008	0.002142
CCRL2	1.777882	0.000478
CCSAP	-0.72791	5.32E-05
CCSER1	-0.84483	0.015565
CCSER2	-0.41574	0.004841
CCT2	0.45101	0.00589
CCT3	0.471227	9.17E-06
CCT4	0.491603	0.000202
CCT5	0.42668	0.001311
CCT6A	0.679858	5.10E-05
CCT7	0.579175	6.58E-10
CCT8	0.683331	9.80E-06
CD101	-1.00113	0.000829
CD160	-1.00659	0.000544
CD163L1	1.040763	0.012812
CD274	1.565503	1.08E-13
CD276	-1.28663	1.58E-07
CD33	0.870827	0.01786
CD36	-1.49019	0.000103
CD38	-1.25446	0.036456
CD40	1.606529	0.037838
CD44	0.573585	0.001419
CD46	0.307984	0.032253
CD58	0.61621	2.82E-05
CD59	0.640614	6.18E-05
CD68	1.911746	9.53E-26
CD83	0.532165	4.62E-05
CD86	-1.32764	0.009906
CD9	-0.19669	0.043076
CD99L2	-0.86679	1.41E-06
CDA	-1.01338	1.58E-10
CDC123	0.522586	0.000196
CDC14A	-0.9828	4.82E-17
CDC25A	0.898435	3.94E-06
CDC26	0.484144	2.73E-05
CDC37	0.364455	0.013633
CDC42BPA	-0.27569	0.004401
CDC42BPB	-0.42151	5.75E-08
CDC42BPG	-0.65668	0.028906
CDC42EP3	-1.48632	7.79E-28
CDC42EP4	0.386185	0.043718
CDC42SE2	-0.43064	0.000656
CDC45	0.31776	0.001155

CDC5L	0.271116	0.022234
CDCA4P4	-2.29551	0.045605
CDCA5	0.29937	0.010588
CDCA7L	0.633515	2.42E-06
CDCP1	0.966543	2.41E-09
CDH1	0.478523	0.012678
CDH12P1	-1.36348	0.004179
CDH12P3	-1.36348	0.004179
CDH13	0.797226	0.030291
CDH15	-1.72006	0.006706
CDH2	-2.42951	5.28E-50
CDH23	-0.85111	0.036475
CDH24	-0.75764	0.001103
CDH8	-0.93057	7.18E-07
CDK1	0.53172	0.003029
CDK12	0.305081	0.038804
CDK14	-0.7264	2.48E-14
CDK15	-1.83654	6.19E-08
CDK17	0.671202	4.74E-06
CDK19	-0.46588	0.014849
CDK2AP2	-1.08624	1.20E-06
CDK3	-0.93897	2.91E-06
CDK5R2	-1.03158	0.0034
CDK5RAP1	0.310253	0.002186
CDK7	0.350301	0.02514
CDKN1B	-0.28165	0.004274
CDKN1C	-1.70894	4.59E-06
CDKN2AIP	0.295551	0.015845
CDKN2AIPN	0.33513	0.004644
CDKN2B-AS	-0.90416	1.42E-05
CDKN2C	-1.23647	3.63E-13
CDON	0.569804	0.000243
CDR2	-0.61712	2.88E-08
CDRT1	-0.71312	0.00263
CDS2	-0.23152	0.003972
CDYL2	-0.58054	0.010282
CEACAM1	2.443332	1.81E-30
CEACAM19	-0.87795	0.000467
CEACAM5	1.588431	3.19E-07
CEACAM6	4.370838	3.56E-23
CEBPA	-0.95617	0.01968
CEBPZ	0.779767	1.06E-07
CEBPZOS	0.305848	0.045789
CELF1	0.241437	0.001648
CELSR1	-0.57494	0.008854
CELSR3	-0.64825	0.008307
CEMIP	0.530561	0.000377

CENPBD1	-0.4478	4.91E-06
CENPBD1P	-0.31397	0.007721
CENPH	0.298553	0.049815
CENPK	0.431972	0.024348
CENPQ	0.596157	0.000249
CENPS	0.412964	0.023754
CENPU	0.361363	0.02652
CENPV	0.350031	0.001282
CEP112	-1.05925	0.000574
CEP128	0.592589	7.25E-06
CEP162	-0.29351	0.048421
CEP19	-0.77544	0.00067
CEP250	-0.35236	0.003948
CEP72	0.386339	9.02E-05
CEP76	0.47842	0.005486
CEP83	0.690368	6.57E-05
CEP89	-0.36769	3.40E-05
CERCAM	-1.46165	2.98E-05
CERK	-0.56642	0.000537
CERKL	-1.25983	2.38E-06
CERS2	0.271287	0.008208
CERS4	-1.29535	3.74E-05
CERS5	-0.17245	0.031107
CES2	-0.75939	0.000231
CETN3	0.551565	0.008458
CFAP20	0.217466	0.01319
CFAP206	-1.65008	0.000536
CFAP44	-0.58146	7.61E-05
CFAP45	-0.66756	1.07E-05
CFAP57	-0.67295	0.008571
CFAP58	-1.15834	0.006576
CFAP97	0.443588	0.008093
CFH	-0.78182	0.013894
CFLAR	0.366851	0.004244
CGA	2.159831	0.003681
CGN	1.12269	0.002157
CGNL1	-1.33968	4.03E-09
CH17-172B	-0.89762	0.00087
CH17-195P	-1.10644	0.034857
CH17-296N	0.478016	0.003886
CH17-302N	-1.81329	1.31E-09
CH17-335B	-0.99754	0.018596
CH17-340N	-0.91672	0.019539
CH17-360D	-1.94301	0.032175
CH17-360D	-0.56661	0.006929
CH17-360D	-0.59109	0.018898
CH17-373J:	1.744577	0.014265

CH17-472G	0.345555	0.012993
CH17-80A1	1.62158	0.036253
CH507-216	-1.61359	0.002693
CH507-236	-1.56805	0.003615
CH507-338	-1.56799	0.004034
CH507-396	4.527329	1.77E-05
CH507-42P	-1.18266	0.019741
CH507-42P	-1.6548	0.005578
CH507-42P	-1.16053	0.030243
CH507-513	0.497847	0.006226
CH507-513	0.497746	0.006238
CH507-513	0.497894	0.006225
CH507-9B2	-0.88903	0.000137
CH507-9B2	-2.25833	0.035394
CH507-9B2	0.676584	2.62E-07
CHAC2	1.464644	1.07E-07
CHAD	-1.49299	5.94E-05
CHAMP1	0.350932	0.000969
CHCHD1	0.273436	0.031552
CHCHD10	0.547506	0.004777
CHCHD2	0.41577	7.57E-07
CHCHD3	0.350984	0.000428
CHCHD4	0.970967	2.57E-13
CHCHD7	0.548683	0.001728
CHD1	0.491572	0.000336
CHD1L	0.498248	7.92E-05
CHD3	-0.5877	0.00018
CHD4	0.218208	0.007564
CHD5	-0.74468	0.037833
CHD6	-0.5179	7.17E-07
CHD7	0.555093	3.89E-05
CHD9	-0.43622	7.57E-05
CHEK2	0.4576	0.005153
CHFR	-0.2717	0.002434
CHGB	0.96746	0.045872
CHIC1	0.324213	0.030047
CHIC2	0.611461	0.001272
CHMP4A	0.39099	0.001803
CHMP4C	0.281178	0.031002
CHMP5	0.731681	8.83E-07
CHMP7	0.262186	0.020106
CHN1	-0.55921	0.026631
CHORDC1	0.651254	0.000813
CHRM3	-0.73987	2.19E-07
CHRM4	-1.29185	0.000322
CHRNA9	-1.45751	0.022594
CHRNB1	0.684095	0.003345

CHST10	-0.41352	0.019955
CHST11	-0.67597	1.30E-09
CHST12	-0.37794	0.022412
CHST14	-1.07562	1.99E-05
CHST15	-0.3763	0.027065
CHST3	-0.59472	0.004551
CHST6	0.954201	0.001201
CHSY1	-0.19967	0.008468
CHTOP	0.171191	0.018891
CHUK	0.590634	6.16E-05
CIAO1	0.329356	2.10E-05
CIAPIN1	0.256397	0.020519
CIB1	0.24467	0.001908
CIC	-0.71441	0.037835
CIDECP	-0.32775	0.040817
CILP2	-1.68977	0.001308
CINP	0.393967	0.003805
CIPC	0.285145	0.015714
CIRBP	-0.28373	0.020695
CIT	-0.35048	0.00037
CKAP4	-0.99175	2.73E-17
CKLF	0.339343	0.000584
CLASP1	-0.62239	3.85E-10
CLASP2	0.257139	0.043273
CLCC1	0.340253	0.001171
CLCF1	1.830459	8.80E-09
CLCN2	0.699198	4.04E-05
CLCN5	0.623316	2.15E-06
CLCN6	-0.61628	4.85E-06
CLDN1	-0.53328	0.01359
CLDN12	0.640918	3.67E-05
CLDN14	-1.33691	0.008019
CLDN4	1.093951	0.000148
CLDN7	0.951404	2.67E-07
CLDN9	-0.8656	0.004596
CLDND1	0.439281	0.007593
CLEC12A	5.028464	2.06E-11
CLEC2D	-0.74299	0.020527
CLEC7A	-1.84116	1.51E-05
CLIC3	-2.6366	5.40E-35
CLIC4	-0.55501	0.003552
CLIC5	-0.66979	0.004898
CLINT1	0.377667	0.000758
CLIP2	-1.09759	0.000102
CLIP4	0.675708	0.000871
CLK1	0.498197	0.004672
CLK4	-0.39587	0.025197

CLMP	2.126786	9.06E-13
CLN3	-0.35559	0.010907
CLN6	0.403563	0.013575
CLNS1A	0.529991	7.64E-07
CLP1	0.323509	0.007085
CLPB	0.529199	5.55E-07
CLPTM1L	0.258802	0.007672
CLPX	0.376202	0.005327
CLSPN	0.459121	0.001282
CLSTN1	-0.48954	0.00666
CLSTN3	-0.92289	0.000209
CLTA	0.300024	0.005133
CLTCL1	-1.70304	5.48E-14
CLU	-1.01048	2.07E-05
CLUAP1	-0.38134	0.004705
CLUH	0.768314	0.000404
CLUHP4	4.001105	0.007774
CMAS	0.334727	0.014349
CMB9-55A:	0.687584	0.049922
CMBL	-0.30883	0.0433
CMC1	0.394058	0.000836
CMC2	0.299342	0.013276
CMC4	-0.62851	0.002329
CMIP	-0.46435	5.92E-06
CMPK1	0.258563	0.026914
CMPK2	-1.49202	0.007445
CMSS1	0.906389	6.43E-07
CMTM3	-1.57605	9.19E-10
CMTM4	0.589982	9.30E-07
CMTM7	0.703607	5.22E-10
CMTM8	0.531296	0.026079
CMTR2	0.855664	2.99E-08
CNBP	0.495702	0.000106
CNGB1	-0.55674	0.009781
CNIH1	0.450597	0.001113
CNIH2	-1.49741	6.08E-06
CNKSR3	-0.46347	8.16E-05
CNN1	-3.46433	3.82E-10
CNN2	-1.21477	2.81E-08
CNNM1	1.010653	8.41E-05
CNNM2	-0.53087	0.001101
CNOT10	0.469691	0.000967
CNOT11	0.378471	0.002908
CNOT4	0.256468	0.021459
CNPY4	-1.41231	8.38E-07
CNST	0.420509	0.000972
CNTN1	1.780859	0.002373

CNTN5	-0.95159	1.54E-09
CNTNAP1	-2.02969	1.51E-07
CNTNAP2	-0.57289	0.01152
CNTNAP3	0.258889	0.001338
CNTNAP3B	0.53367	6.82E-07
CNTNAP3P	0.486702	0.00322
COA1	0.167614	0.019834
COA4	0.677613	2.75E-08
COA5	0.304565	0.045296
COA6	0.675092	2.56E-07
COA7	0.674329	6.28E-07
COBL	0.770878	0.001253
COBLL1	-0.34258	0.013249
COG2	0.293444	0.038343
COG5	0.261068	0.035513
COIL	0.422685	0.000335
COL11A1	-1.70161	1.10E-05
COL11A2	-1.25379	0.025261
COL12A1	-1.60946	1.27E-16
COL13A1	1.257958	2.06E-06
COL15A1	-1.72408	5.55E-12
COL16A1	-2.00153	1.27E-05
COL18A1	-1.59386	0.014713
COL1A1	-3.69273	7.33E-09
COL20A1	-3.74364	3.72E-08
COL22A1	1.722253	0.006782
COL26A1	-3.37212	7.92E-05
COL27A1	-0.80998	0.003631
COL28A1	-0.74884	0.000729
COL2A1	-3.32323	0.036301
COL3A1	-7.35274	6.34E-07
COL4A1	-3.23986	5.23E-16
COL4A2	-2.75461	1.27E-06
COL4A2-AS	-1.60544	0.045699
COL4A2-AS	-2.57982	0.028126
COL4A3	-2.83895	1.54E-10
COL4A4	-3.7185	2.46E-16
COL5A1	-4.0844	4.70E-11
COL5A2	-1.39087	7.37E-08
COL6A1	-1.53339	0.000432
COL8A1	1.399247	2.87E-07
COL8A2	-3.26003	0.001771
COL9A2	-1.42536	0.0003
COL9A3	-2.56406	0.024914
COLCA2	-1.57481	0.000151
COLEC11	-0.92071	0.029788
COLGALT1	-0.27891	0.015108

COLGALT2	-1.23033	0.006473
COMMD2	-0.36091	0.003929
COMMD3-I	0.373717	0.037589
COMMD7	-0.27617	0.005438
COMMD9	-0.38445	0.000109
COPA	-0.19856	0.026579
COPG1	-0.31346	0.000157
COPRS	0.214746	0.009507
COPS2	0.525198	0.000749
COPS3	0.375597	0.008914
COPS8	0.243669	0.044636
COPS9	0.283934	0.01726
COPZ2	-3.41166	0.01038
COQ10B	0.390224	0.016821
COQ3	0.366142	0.047655
COQ6	0.270468	0.003938
COQ8A	0.843869	2.57E-14
CORO2A	-1.10066	1.78E-09
CORO2B	1.29524	1.60E-05
CORO6	-1.04053	6.15E-06
CORO7-PAI	4.29022	0.041915
COTL1	0.298474	0.001599
COX10-AS1	0.388635	0.008533
COX15	0.332799	0.004728
COX16	0.432087	0.011633
COX17	0.61592	7.25E-05
COX18	0.444825	0.001906
COX4I1	0.266668	0.002618
COX5A	0.46188	2.13E-05
COX6A1	0.246488	0.003827
COX6B1	0.222081	0.00909
COX6C	0.323356	0.010935
COX7A2	0.402315	0.002716
COX7B	0.522509	0.001356
COX7C	0.435887	0.005129
CP	-1.16548	0.043957
CPA4	-1.87412	5.25E-21
CPAMD8	-1.67726	3.13E-05
CPE	-1.46693	0.001888
CPEB2	-0.77403	5.66E-05
CPEB3	0.749197	0.000946
CPEB4	0.988699	3.83E-14
CPED1	0.524775	0.032094
CPHL1P	4.097335	3.70E-05
CPLX1	1.509212	5.39E-06
CPNE1	-0.29661	0.037544
CPNE2	-0.47038	0.000175

CPNE3	0.383172	0.009507
CPNE4	1.778198	2.58E-09
CPNE5	3.145056	9.70E-10
CPNE8	0.290933	0.02661
CPOX	0.516304	4.51E-06
CPPED1	-0.27874	0.010325
CPS1	-0.72345	0.002815
CPSF2	0.367272	0.015816
CPSF3	0.380209	0.00943
CPSF6	0.307498	0.003468
CPT1C	-1.03237	0.00094
CPVL	-0.70895	0.01968
CPZ	-1.06597	0.000577
CRACR2A	0.494784	0.040052
CRAMP1	-0.28836	0.02105
CRAT	-0.77436	0.000618
CRCP	0.315308	0.001354
CRCT1	4.012792	5.71E-13
CREB3L1	-1.54164	4.77E-08
CREB3L2	-0.54811	3.19E-07
CREB5	-1.62144	0.00017
CREBBP	-0.45584	4.19E-05
CREBL2	0.256919	0.028791
CREM	0.499885	2.02E-05
CRIM1	-1.09269	4.00E-20
CRIP1	-0.85399	5.26E-05
CRIP2	-1.98467	1.95E-08
CRIPT	0.320778	0.02302
CRISPLD1	-0.93567	9.45E-09
CRISPLD2	-0.86529	0.016165
CRK	0.252662	0.00436
CRKL	0.173103	0.000628
CRLF1	-2.68871	3.32E-06
CRLF2	1.368465	0.003533
CRLF3	0.517744	0.000986
CRLS1	0.45123	0.000879
CRMP1	-0.54643	0.006225
CRNDE	-0.8153	0.001233
CRNKL1	0.3899	0.005367
CROCCP3	-0.56128	0.023332
CROT	-0.87725	9.76E-08
CRTC1	-0.71763	0.023366
CRYBB3	-1.22098	0.028626
CRYBG3	0.312248	0.014099
CRYM	2.683568	0.001052
CRYM-AS1	0.683945	0.007144
CSDC2	-5.10377	1.29E-11

CSE1L	0.632843	0.000824
CSF1	-1.09198	2.23E-05
CSF1R	-2.21125	5.76E-07
CSF2	1.047085	0.000779
CSF2RA	2.445739	9.36E-27
CSF2RB	-5.38311	7.82E-07
CSF3	4.532628	1.30E-05
CSGALNAC	0.685035	0.007949
CSNK1A1	0.250512	0.01645
CSNK1G1	-0.29811	0.003378
CSNK2A1	0.385313	2.36E-05
CSNK2A3	-2.07226	1.16E-06
CSNK2B	0.196849	0.03249
CSPG4	-2.04588	0.00261
CSPG4P10	-0.90603	0.032963
CSPG4P11	-1.23363	0.000905
CSPG5	-1.40653	4.21E-09
CSRNP2	-0.57597	1.05E-08
CSRNP3	-2.20432	0.000945
CSRP1	-1.08811	7.23E-09
CSRP2	-0.94218	7.26E-24
CST5	-2.6451	0.046867
CST6	-1.18769	6.42E-06
CSTB	1.313974	1.22E-16
CSTF1	0.378867	0.002151
CSTF2	0.482423	5.89E-05
CSTF3	0.46677	0.009087
CT62	-1.02878	0.029506
CTA-150C2	2.518373	0.026034
CTA-204B4	-0.40854	0.03273
CTA-398F1	-0.90394	0.012421
CTA-963H5	-1.37243	0.011996
CTAGE8	0.754251	0.041657
CTB-119C2	0.636532	0.03691
CTB-131B5	-0.56471	0.048703
CTB-131K1	0.333645	0.004443
CTB-133P2	0.834515	0.038616
CTB-147C2	2.508828	0.00125
CTB-147C2	1.987555	0.000173
CTB-147C2	0.508781	0.034152
CTB-22K14	-1.12276	0.036791
CTB-33O18	-1.52634	0.005842
CTB-50E14	-0.91789	0.019523
CTB-60B18	-1.38141	0.013491
CTB-60B18	-0.60283	0.010523
CTB-88F18	0.847831	0.036472
CTB-89H12	0.413303	0.002684

CTB-92J24.	-1.6365	0.013665
CTC1	0.35798	0.001104
CTC-325H2	-0.6098	0.014907
CTC-353G1	-5.39276	2.67E-07
CTC-420A1	-1.14812	0.037358
CTC-425O2	-0.40725	0.016148
CTC-439O9	-1.59002	0.027157
CTC-444N2	0.520323	4.92E-06
CTC-444N2	0.749769	0.000207
CTC-455F1	0.862878	0.028818
CTC-459F4.	-0.83565	0.00053
CTC-459F4.	0.572842	0.04626
CTC-471J1.	0.603338	0.040359
CTC-471J1.	-1.88061	0.012437
CTC-490E2	-0.99373	0.003428
CTC-490G2	2.235311	0.000157
CTC-490G2	-0.92666	0.04685
CTC-493P1	0.740416	0.010354
CTC-499J9.	-1.07048	0.003047
CTC-518B2	-0.59501	0.015919
CTC-523E2	1.028614	0.010174
CTC-529P8	2.075141	1.13E-06
CTC-534A2	0.576289	0.011202
CTC-542B2	0.751302	0.032009
CTC-559E9	2.312306	0.043508
CTC-756D1	-3.36863	0.004168
CTD-2001J	1.01055	0.031231
CTD-2008L	-0.68531	0.030347
CTD-2008L	-0.96038	0.000288
CTD-2015H	-0.46954	0.008443
CTD-2017D	-0.52596	0.000204
CTD-2020K	-0.94873	0.004889
CTD-2021A	-3.88424	0.000643
CTD-2021A	-1.42371	0.005841
CTD-2021K	0.632164	0.003744
CTD-2024F	1.524496	0.049018
CTD-2026K	-1.56538	2.31E-07
CTD-2027I	-2.89526	0.019486
CTD-2027I	-3.22976	0.046642
CTD-2033D	-1.42071	1.27E-05
CTD-2033D	-1.13656	0.031881
CTD-2035E	-2.59245	0.031122
CTD-2047H	-1.61715	0.037577
CTD-2058B	0.890582	6.61E-05
CTD-2060L	2.550739	0.036359
CTD-2066L	3.560435	0.010016
CTD-2066L	5.177585	3.08E-05

CTD-2066L	1.564847	0.034771
CTD-2083E	1.244525	9.96E-05
CTD-2083E	2.494079	0.008112
CTD-2116N	0.241569	0.044723
CTD-2132N	-0.90466	0.022789
CTD-2144E	0.842021	0.005724
CTD-2162K	0.651661	0.004623
CTD-2165H	-0.9481	0.040947
CTD-2175A	2.333882	0.001128
CTD-2186N	-0.50759	0.008919
CTD-2192J	-1.71287	0.040347
CTD-2192J	2.787727	0.008127
CTD-2227E	-2.07676	6.14E-05
CTD-2228K	-1.017	0.028793
CTD-2245F	-0.35256	0.015718
CTD-2260A	-0.60209	0.018103
CTD-2269F	-1.17741	0.000935
CTD-2270L	-0.615	0.016143
CTD-2286N	0.97073	0.011832
CTD-2297D	-2.8916	0.031568
CTD-2302E	1.208002	0.039461
CTD-2303H	-0.79575	4.88E-05
CTD-2311N	0.414274	0.033448
CTD-2325P	-2.45354	3.45E-12
CTD-2330K	3.739657	0.040709
CTD-2332E	1.034565	0.007724
CTD-2336C	-0.58992	0.038085
CTD-2349P	0.637212	0.043505
CTD-2353F	-2.72605	2.32E-10
CTD-2354A	0.81467	0.003882
CTD-2366F	1.595222	5.93E-07
CTD-2368P	-0.54323	1.19E-06
CTD-2377C	-1.00186	0.046503
CTD-2383N	-1.17297	3.01E-06
CTD-2503C	-3.45709	0.010568
CTD-2514C	-1.1005	0.009408
CTD-2517C	-0.77067	0.034261
CTD-2532D	3.906893	0.015277
CTD-2540L	1.97008	0.001093
CTD-2553L	-0.5434	0.025365
CTD-2554C	-0.50282	0.000806
CTD-2555C	2.145538	0.002263
CTD-2555C	-0.97447	0.001843
CTD-2561B	-0.95954	0.003211
CTD-2562J	-0.95083	0.013437
CTD-2574D	-0.70908	0.031105
CTD-2589N	1.515835	0.018264

CTD-2619J1	-1.33599	0.005513
CTD-2620I2	-0.99261	0.030259
CTD-3006G	-1.06022	0.000746
CTD-3014M	1.162254	6.39E-09
CTD-3018C	-2.85598	0.013098
CTD-3023L	-2.74886	0.024541
CTD-3088G	-1.71815	0.001756
CTD-3092A	-0.66492	0.007529
CTD-3099C	0.825904	0.000773
CTD-3105H	-1.50209	0.004257
CTD-3113P	0.277127	0.043015
CTD-3128G	2.36475	3.74E-17
CTD-3162L	-0.66193	0.032763
CTD-3216D	0.639062	0.030746
CTD-3220F	-1.28222	2.61E-06
CTD-3247F	4.951316	4.31E-06
CTDSP2	-0.24447	0.022001
CTF1	-0.44775	0.045356
CTGF	-1.07143	8.96E-05
CTHRC1	-1.37223	4.02E-07
CTIF	-1.63427	3.56E-09
CTNNA3	-2.10049	0.041675
CTNNB1	-0.23225	0.014978
CTNNBL1	0.212952	0.003856
CTPS1	0.357563	0.02235
CTR9	0.409967	0.000364
CTSC	1.310263	1.15E-08
CTSE	2.004801	2.80E-07
CTSF	-0.88158	0.024728
CTSH	-1.01578	0.000226
CTSK	-0.72879	0.003739
CTSL	0.49478	1.89E-05
CTSO	-0.78506	0.001639
CTSS	1.04352	1.53E-05
CTSV	0.477326	0.044727
CTTNBP2N	-0.28465	0.005838
CUBN	-0.84687	0.011327
CUEDC1	-0.50019	0.025454
CUL1	0.403667	0.009793
CUL2	0.3464	0.011493
CUL4A	0.265247	0.048746
CUL7	-0.42791	0.030913
CUL9	-0.36892	0.032464
CUX1	-0.78394	3.85E-07
CWC22	0.491301	0.001006
CWC25	0.207646	0.004365
CWC27	0.24892	0.043711

CXADR	-0.35564	0.012785
CXCL16	0.594428	4.26E-05
CXCL2	1.442317	0.000278
CXCL3	1.122558	1.12E-05
CXCL5	1.614997	0.003872
CXCL8	1.26121	0.001028
CXCR6	1.681341	1.32E-06
CXorf38	0.273403	0.007414
CXXC5	-1.60788	6.54E-11
CYB561	-0.36408	0.009788
CYB561A3	-0.92237	2.37E-09
CYB5A	0.473189	0.007849
CYB5B	0.253821	0.020095
CYB5D1	0.482624	3.87E-05
CYB5R4	0.414946	0.002796
CYBRD1	-1.71758	8.41E-10
CYCS	0.851406	1.74E-05
CYCSP6	0.879335	7.10E-05
CYMP	3.62554	0.007018
CYP17A1	-3.8332	0.000754
CYP1A2	-0.97773	0.000109
CYP1B1-AS	0.556392	0.022712
CYP26A1	-0.82817	0.013499
CYP2B7P	-1.5272	0.001503
CYP2F2P	-0.92422	0.024872
CYP2S1	-0.32807	0.035759
CYP2T1P	-1.22004	0.020601
CYP2U1	-0.95227	0.000343
CYP39A1	-1.28481	3.23E-05
CYP3A7	-2.9636	0.033885
CYP4B1	-1.2621	0.003163
CYP4F11	0.481627	0.008134
CYP4F26P	1.820125	8.71E-07
CYR61	-0.69354	5.19E-05
CYS1	-1.42181	0.000263
CYSLTR1	0.556426	0.015236
CYSRT1	1.278179	0.000669
CYSTM1	0.758436	1.51E-16
CYTH1	-0.59334	0.000131
CYTH3	-1.35124	1.48E-20
D21S2088E	0.31366	0.042476
DAB1-AS1	-3.5666	0.005473
DAB2	-1.93079	5.61E-20
DAB2IP	-1.46729	2.73E-09
DACT1	-2.07822	1.95E-13
DAD1	-0.34125	0.003475
DAG1	-1.06079	2.44E-13

DAND5	-1.12283	0.000161
DAP3	0.545376	1.12E-06
DAPK1	-1.34744	6.99E-25
DAPK2	-1.32506	3.56E-05
DAPK3	-0.62481	0.001094
DAPP1	0.635173	0.003213
DARS	0.372108	0.01732
DAZAP1	0.305972	0.025719
DBET	1.789459	0.021344
DBF4	0.477662	0.010684
DBN1	-0.66073	0.009251
DBNDD2	0.4991	0.000271
DBR1	0.379789	0.001853
DCAF1	0.436033	7.99E-05
DCAF11	-0.31089	0.031914
DCAF13	0.527697	0.002362
DCAF16	0.354192	0.030707
DCAF4	-0.19469	0.046393
DCAF5	-0.34486	0.00084
DCAF7	-0.20754	0.021944
DCAF8	0.204438	0.03834
DCAKD	-0.63558	0.000253
DCBLD1	-1.25313	7.39E-11
DCBLD2	0.891592	5.04E-13
DCHS2	-1.37976	0.000882
DCLK1	1.215738	3.53E-05
DCLK2	-3.80209	2.95E-13
DCLRE1C	0.428417	0.001437
DCP1A	0.248549	0.009662
DCP1B	-0.5706	0.017086
DCST2	-1.48465	0.030325
DCTD	0.358477	0.002507
DCTN1	-0.38733	0.004931
DCTN6	0.377021	0.002906
DCUN1D1	0.377537	0.014709
DCUN1D3	0.583319	1.87E-05
DCUN1D5	0.548373	7.59E-10
DDAH2	-1.61633	1.01E-05
DDB2	-0.4149	2.00E-05
DDHD1	0.363526	0.003075
DDHD2	-0.18846	0.024394
DDI2	0.53206	2.21E-05
DDIAS	0.715337	2.73E-05
DDTL	0.406729	0.029471
DDX1	0.237965	0.043653
DDX10	0.664184	0.000144
DDX18	0.542699	0.001548

DDX19A	0.261394	0.001958
DDX20	0.316583	0.005415
DDX21	0.824801	5.23E-07
DDX23	0.214284	0.013005
DDX24	0.49071	8.96E-06
DDX27	0.370349	0.000355
DDX28	0.496826	0.00057
DDX31	0.40432	8.22E-05
DDX39A	0.38213	0.019661
DDX39B	0.2395	2.41E-07
DDX3X	0.329901	0.032837
DDX46	0.478162	0.002708
DDX47	0.430626	0.018941
DDX50	0.395744	0.004394
DDX52	0.528841	0.001414
DDX55	0.64002	7.81E-08
DDX56	0.392788	0.00027
DDX60L	-0.51312	0.001252
1-Dec	-1.5783	1.67E-07
DECR1	-0.31759	8.13E-05
DEFB103A	3.345365	0.009126
DEFB103B	2.733666	0.023637
DENND2A	-1.6675	1.26E-09
DENND2C	1.995432	2.62E-08
DENND3	-0.30497	0.001399
DENND4A	0.316658	0.046408
DENND5A	-0.59812	3.79E-05
DENND5B	-0.5632	6.02E-06
DENND6B	-1.34281	6.91E-07
DENR	0.243704	0.040005
DEPDC5	0.296735	0.001666
DESI1	0.681533	3.33E-10
DFFA	0.347148	5.47E-05
DFFB	0.589826	0.0012
DFNA5	-0.88934	3.24E-13
DFNB59	-0.81592	0.016951
DGCR2	-0.8612	0.000478
DGCR5	-1.10944	1.07E-05
DGCR6	-0.8926	0.034785
DGKD	-0.80775	9.15E-06
DGKE	0.356286	0.005181
DGKI	1.357745	0.000359
DGUOK-AS	1.003796	0.006751
DHCR24	-0.54255	4.92E-06
DHCR7	-0.52414	0.000178
DHDH	1.276886	0.005444
DHODH	0.715835	2.68E-07

DHRS11	0.282172	0.038194
DHRS12	-0.63284	0.001404
DHRS2	0.808171	0.000347
DHRS4-AS1	-0.31975	0.005165
DHRS7B	0.313145	0.001934
DHRS9	4.134209	3.57E-25
DHRSX	-0.58629	3.34E-05
DHX15	0.548895	0.000248
DHX30	0.375212	0.004756
DHX33	0.44436	2.80E-05
DHX34	0.435806	0.021894
DHX35	0.243116	0.047148
DHX36	0.396999	0.004187
DHX9	0.444944	0.002665
DIAPH2	0.757776	3.16E-06
DIEXF	0.637867	1.65E-09
DIMT1	0.412534	0.003442
DIP2A	-0.22407	0.044298
DIP2B	-0.44537	5.87E-05
DIP2C	-1.18078	6.03E-06
DIRAS1	-1.76662	7.04E-05
DIRC3	-1.39119	0.00883
DIS3	0.662475	1.57E-06
DISC1	-2.63298	1.47E-08
DISP1	-0.65301	0.004636
DISP2	-0.86415	0.000759
DISP3	-1.41123	0.004378
DKC1	0.756541	2.87E-05
DKK3	-3.53418	7.98E-24
DKKL1	-2.42573	0.031007
DLAT	0.45681	0.000163
DLEU1	0.419416	0.003048
DLEU1_2	1.147839	0.00153
DLEU7	-0.7252	0.000127
DLG3	-0.3378	0.000203
DLG5	-0.53152	6.88E-05
DLGAP1-AS	-0.45282	0.012478
DLGAP4	-0.52674	0.011714
DLK2	-1.21813	7.29E-05
DLL1	-2.43276	1.25E-27
DLL4	1.80117	8.36E-08
DLST	0.292415	0.005323
DLX1	0.522983	0.006999
DLX3	-2.3949	3.71E-09
DLX4	-1.33052	4.16E-07
DMBT1	2.498867	8.02E-15
DMBX1	-0.91915	0.000405

DMPK	-0.87884	0.003651
DMRT2	1.030297	0.001179
DMRTA1	-1.76491	3.93E-06
DMRTC2	3.838097	9.29E-05
DMWD	-0.55999	0.042736
DNA2	0.436606	0.007314
DNAAF2	0.523539	1.04E-06
DNAAF3	-0.70337	0.03484
DNAH10OS	-1.24931	0.000211
DNAH14	0.543059	0.000319
DNAH3	0.971825	0.001121
DNAH5	-1.14908	1.75E-15
DNAI1	-2.02147	0.04509
DNAJA1	0.607504	0.001248
DNAJA2	0.515219	1.50E-06
DNAJA3	0.436159	2.86E-05
DNAJB1	0.646992	1.33E-08
DNAJB2	-0.91791	0.000288
DNAJB3	-1.39994	0.017436
DNAJB4	-0.33689	0.020069
DNAJB5	-1.08777	8.96E-05
DNAJC1	-0.75231	7.83E-05
DNAJC11	0.223404	0.02595
DNAJC15	-0.58146	1.88E-05
DNAJC18	-0.47117	0.004534
DNAJC19	0.592242	5.24E-05
DNAJC2	0.649131	0.000184
DNAJC22	-0.63089	0.006249
DNAJC4	-0.8693	0.001958
DNAJC6	-0.60104	0.007501
DNAJC7	0.328484	0.005892
DNAJC9	0.276386	0.021986
DNAL1	-0.55941	0.000142
DNAL4	-0.91279	8.05E-05
DNASE2	-0.48867	0.001819
DNER	1.16615	1.13E-07
DNM1P35	-1.28912	0.001726
DNM1P51	-1.45855	0.04179
DNMBP	0.510212	2.60E-06
DNMT1	0.32223	9.50E-05
DNMT3A	-0.55886	0.000216
DNMT3L	-1.96552	0.013251
DNTTIP1	0.336753	0.01278
DNTTIP2	0.726821	7.66E-06
DOC2B	-1.36791	0.03584
DOCK1	-0.42524	1.41E-08
DOCK11	-2.08485	1.39E-09

DOCK2	-2.20479	1.14E-10
DOCK3	-1.11432	0.000256
DOCK4	2.484042	1.89E-19
DOK4	-0.57732	0.02211
DONSON	0.358793	0.019945
DOPEY2	-0.56924	0.000181
DPCD	-0.42159	0.005223
DPEP1	2.259776	0.042774
DPF3	1.002036	2.85E-05
DPH2	0.402732	0.00607
DPH3	0.375468	0.008028
DPH5	0.523839	0.003263
DPH6	0.480475	0.021137
DPH6-AS1	-1.4994	0.020592
DPP4	-0.69772	0.04337
DPY19L2	0.676634	0.00054
DPY19L2P2	0.727205	1.96E-05
DPY19L2P4	0.662929	0.04181
DPYD	0.371922	0.011263
DPYSL3	-2.41995	5.35E-16
DPYSL4	-0.95579	0.011979
DPYSL5	-1.71742	6.10E-09
DQX1	-1.1409	0.002666
DRAM1	-1.2936	3.18E-11
DRAXIN	-1.39957	0.001104
DRC3	-0.66772	0.00117
DRG1	0.419399	6.00E-05
DSC3	-0.82864	1.06E-08
DSCAM	2.042603	1.56E-09
DSCC1	0.655502	0.000211
DSCR3	-0.23666	0.045828
DSG3	1.006117	0.001262
DSN1	0.349931	0.007208
DST	-0.50337	0.000225
DSTN	-0.35663	0.004658
DSTNP1	-0.49527	0.038116
DSTNP2	-0.47289	0.028182
DSTYK	-0.61038	1.66E-09
DTD1	0.28327	0.0267
DTD2	0.360814	0.025977
DTL	0.391729	0.002207
DTNB	-1.0976	1.54E-12
DTWD1	0.329406	0.019297
DTX3	-0.51769	0.025556
DUBR	-0.51558	0.009315
DUS2	0.411675	0.003419
DUS3L	0.517062	0.012163

DUS4L	0.33218	0.046582
DUSP1	0.753515	2.94E-09
DUSP10	0.358004	0.000396
DUSP12	0.308138	0.002751
DUSP14	-0.36613	0.020468
DUSP16	-0.58374	0.00026
DUSP18	-0.52788	2.80E-05
DUSP2	0.97959	0.00421
DUSP22	-0.28837	0.004103
DUSP3	-0.57919	2.14E-12
DUSP4	1.534667	5.10E-22
DUSP5	2.721171	2.86E-33
DUSP6	2.003333	2.54E-51
DUSP7	0.688571	0.000603
DUSP8	-1.24174	5.17E-06
DUXAP10	-0.85251	2.26E-07
DUXAP8	-0.935	2.25E-18
DUXAP9	-0.93651	6.41E-12
DVL2	-0.52312	0.039272
DVL3	-0.77667	3.16E-05
DYNAP	0.739296	0.013051
DYNC1H1	-0.33704	0.001093
DYNLL2	-0.15821	0.048325
DYNLRB2	-2.97368	0.00757
DYNLT3	0.316253	0.008068
DYRK1A	-0.28418	0.008851
DYSF	-2.57664	8.76E-12
DYX1C1	-0.97869	5.76E-06
DZANK1	-0.44929	0.024627
E2F3	0.436514	7.92E-06
E2F4	0.441283	0.004072
E2F5	0.564107	4.42E-05
E2F6	0.243192	0.009546
E2F7	0.317975	0.014789
EAF1	0.455596	2.90E-06
EAF2	-0.61609	0.00342
EARS2	0.21556	0.005676
EBF4	-3.2732	0.021455
EBI3	-2.23871	1.67E-08
EBNA1BP2	0.57178	0.000579
EBP	-0.24036	0.011829
EBPL	0.672028	6.32E-05
ECD	0.372321	0.010833
ECE1	-0.84663	2.53E-06
ECE2	0.558896	1.13E-05
ECH1	-0.68541	0.0002
ECHDC1	0.364504	0.001439

ECHS1	0.260249	0.002125
ECM2	-1.20123	0.004922
ECT2	0.511763	0.011776
EDAR	-2.17636	3.70E-07
EDARADD	-1.97334	6.60E-18
EDEM2	-0.53357	0.000391
EDEM3	0.234694	0.01676
EDIL3	-3.03879	7.06E-28
EDN1	-1.21091	1.53E-08
EDN2	-1.09748	0.039898
EDRF1	0.303696	0.019789
EED	0.569422	0.001584
EEF1A1	0.324501	0.019008
EEF1AKMT	0.693721	0.000479
EEF1B2	0.607023	4.41E-08
EEF1E1	0.572029	0.000262
EEF1G	0.251053	0.000526
EEF2K	0.264519	0.002821
EEF2KMT	0.652176	9.05E-05
EEPD1	0.506518	0.047512
EFCAB1	-1.85797	1.21E-08
EFCAB6	-0.7406	0.010283
EFEMP1	-0.77183	1.16E-08
EFHC1	-0.34907	0.04685
EFHC2	-1.15124	0.020214
EFHD2	1.190398	3.14E-09
EFL1	0.332587	0.019772
EFNA1	-0.74388	1.24E-08
EFNA2	-2.59148	1.56E-05
EFNA4	-1.00658	0.00321
EFNA5	-0.91715	1.71E-10
EFNB2	0.614236	5.31E-11
EFNB3	-2.25713	1.40E-05
EFS	-2.10859	0.004307
EFTUD2	0.330641	0.000588
EGF	-2.10942	0.000252
EGLN3	-2.29187	5.07E-06
EGR1	4.357644	4.44E-79
EGR2	2.851989	6.49E-19
EGR3	4.07245	3.02E-33
EGR4	2.962014	2.57E-07
EHD2	-1.05699	0.000324
EHD3	-0.82787	0.000194
EHD4	0.255267	0.045012
EHF	0.980132	1.23E-12
EHHADH	-0.58666	0.000869
EHMT1	-0.26529	0.015196

EID2B	-0.69285	0.01324
EID3	0.739999	0.020241
EIF1AX	0.624918	0.000477
EIF2A	0.574054	0.000202
EIF2AK3	-0.48398	0.001516
EIF2B1	0.32988	0.017673
EIF2B2	0.546668	6.46E-12
EIF2B3	0.548643	1.20E-05
EIF2B5	0.362922	7.10E-09
EIF2S1	0.621955	5.99E-06
EIF2S2	0.394669	0.00219
EIF2S3	0.371734	0.007029
EIF3A	0.426634	3.61E-06
EIF3B	0.483046	0.000146
EIF3C	0.298233	7.34E-05
EIF3D	0.501762	1.49E-05
EIF3E	0.505657	0.001134
EIF3H	0.294561	0.03702
EIF3I	0.366332	0.000868
EIF3J	0.610653	7.40E-06
EIF3K	0.335258	0.000295
EIF3L	0.277713	0.011866
EIF3M	0.67047	2.82E-05
EIF4A1	0.566158	2.25E-06
EIF4A1P10	1.380549	0.014665
EIF4A3	0.266729	0.004785
EIF4B	0.321924	0.003731
EIF4E	0.64248	7.28E-05
EIF4E3	-0.78808	2.31E-07
EIF4G3	0.176116	0.011151
EIF5	0.37175	0.000624
EIF5A	0.51051	0.001891
EIF5A2	0.427272	0.00201
EIF5AL1	0.878833	0.015325
EIF5B	0.619989	3.79E-05
EIF6	0.232888	0.036138
ELAC2	0.349321	0.004784
ELAVL2	-0.60292	1.43E-08
ELF3	1.279883	3.57E-13
ELF3-AS1	1.176236	7.42E-07
ELF4	-0.25313	0.044282
ELF5	3.095542	0.006634
ELFN2	0.877426	0.000291
ELK3	1.492389	8.01E-12
ELL	0.392278	0.033311
ELL2	0.731368	3.39E-06
ELMO2	-0.37541	9.24E-05

ELN	1.376913	0.026251
ELOA	0.438493	2.58E-07
ELOC	0.438704	0.000146
ELOVL1	0.471816	0.010147
ELOVL2	-0.80501	0.000166
ELOVL6	1.195869	2.17E-08
ELOVL7	0.296505	0.030716
ELP2	0.362793	0.004688
ELP3	0.333199	9.25E-05
EMC2	0.342195	0.041355
EMC4	0.364003	0.000303
EMC7	0.403747	0.000242
EMCN	0.986577	0.004562
EMG1	0.488481	3.99E-05
EMID1	-2.16669	0.001583
EML4	0.413241	0.001761
EML5	0.401227	0.014067
EMP1	2.252718	1.34E-16
EMP2	0.699838	2.13E-06
EN1	-1.45829	0.005042
ENAH	-1.36004	1.71E-31
ENDOD1	-0.73653	2.21E-11
ENDOV	-0.61053	0.00372
ENKUR	-0.8741	0.013559
ENO1	0.481349	0.000168
ENO4	-1.06026	0.025806
ENOPH1	0.533842	3.80E-05
ENOSF1	0.472685	1.37E-05
ENOX1	-0.7462	0.00021
ENPP5	-0.7107	0.029924
ENSA	0.183339	0.00633
ENTPD1-AS	0.35917	0.015084
ENTPD3	1.864618	0.000103
ENTPD4	-0.41924	3.55E-06
ENTPD6	0.598934	0.003286
EPAS1	-0.75967	0.00039
EPB41L1	-0.74695	0.000172
EPB41L4A	-1.26099	4.90E-09
EPB41L4B	0.479493	0.010441
EPB41L5	0.653552	1.54E-05
EPB42	-1.48169	0.047368
EPCAM	0.941046	1.11E-07
EPGN	1.418853	0.000926
EPHA2	0.50282	0.008718
EPHA3	-2.04669	0.013223
EPHB2	-1.08383	2.57E-08
EPHB3	0.953193	0.020308

EPHB4	-0.79493	0.002072
EPHX1	-1.57399	9.86E-09
EPHX2	0.819417	0.003783
EPM2A	-0.41573	0.00055
EPOR	-0.40448	0.027652
EPPIN	0.945866	0.020517
EPPK1	-0.80722	0.019978
EPS8L1	0.557552	0.03026
EPS8L2	-0.83004	0.00081
EPSTI1	-2.22048	2.69E-35
ERAP1	-0.55351	1.29E-06
ERBB2	-0.69422	0.002518
ERBB3	0.457166	0.006343
ERC1	-0.33973	0.006059
ERCC2	-0.33086	0.043729
ERCC3	0.148723	0.035705
ERCC4	0.400626	0.006021
ERCC5	-0.29966	0.013959
ERCC6	0.311299	0.018211
ERCC6L	0.47824	0.013775
EREG	2.530119	9.90E-12
ERGIC1	-0.23274	0.039631
ERGIC2	0.354159	0.024481
ERH	0.381999	0.017445
ERI1	0.540552	0.000282
ERI2	0.429252	0.044399
ERICD	-1.29484	2.41E-07
ERICH1	0.278347	0.001654
ERICH5	-0.97601	1.02E-05
ERLIN1	0.222828	0.04874
ERMAP	-0.44892	0.00684
ERN1	0.53523	0.000748
ERO1A	0.443221	0.018388
ERP27	-1.28328	4.68E-09
ERRFI1	1.327182	1.14E-13
ERV3-1	-0.56124	2.68E-10
ERVH-1	1.055967	0.000296
ERVK13-1	0.309903	0.016463
ERVK-28	-1.03332	0.010317
ERVK3-1	0.323342	0.005056
ESCO2	0.667514	0.001533
ESF1	0.793649	1.80E-06
ESM1	7.826141	1.02E-86
ESRP2	0.706587	0.004073
ESRRAP2	1.511737	0.026262
ETF1	0.591607	1.29E-05
ETFDH	0.449921	0.005895

ETFRF1	0.573126	0.002562
ETNK1	0.59682	0.000114
ETNK2	-0.72596	0.002178
ETS2	-0.54323	1.21E-06
ETV1	1.204934	0.000268
ETV3	0.311709	0.000946
ETV4	1.57301	2.13E-22
ETV6	-0.61134	2.49E-08
EVA1A	-1.20967	6.55E-08
EVC	-0.43851	0.000999
EVI2B	2.271703	8.14E-05
EVI5L	-0.90586	0.001456
EVL	-0.9954	7.80E-08
EVPLL	-1.41842	0.012062
EXOC3	0.158188	0.043079
EXOC3L1	-2.85263	9.00E-05
EXOC6	0.647882	0.000117
EXOC6B	-0.48462	1.66E-05
EXOC8	0.173756	0.037524
EXOSC2	0.625472	9.70E-09
EXOSC3	0.711477	2.47E-05
EXOSC4	0.592148	0.015261
EXOSC5	0.38832	0.001003
EXOSC6	0.369493	0.005419
EXOSC7	0.598204	2.84E-08
EXOSC8	0.442684	0.010767
EXOSC9	0.431965	0.003257
EXT1	-1.22465	5.35E-13
EXTL1	-1.82504	0.002357
EXTL2	0.43742	0.000429
EXTL3	-0.37669	0.003981
EYA2	-0.79574	0.000469
EYA3	0.424489	0.001145
EYA4	0.406993	0.022905
EZR	0.603522	0.000216
F10-AS1	-3.60032	0.001122
F2R	-0.6398	2.25E-05
F2RL1	0.898078	0.000163
F3	0.897224	0.001648
FA2H	1.76629	1.96E-12
FABP5	1.239068	1.07E-07
FABP5P7	1.4507	3.32E-09
FADS1	-0.41853	0.003638
FADS2	-1.82114	9.48E-11
FADS3	-1.49327	2.98E-10
FAH	0.336588	0.007174
FAM102B	-0.40527	0.005779

FAM103A1	0.306084	0.004404
FAM106A	-2.29268	8.10E-07
FAM109A	-0.89215	0.033301
FAM109B	-0.75636	0.010846
FAM110A	-0.56461	0.012568
FAM111B	0.426427	0.033169
FAM114A1	-0.88971	2.60E-14
FAM114A2	0.307222	0.023354
FAM117A	-1.14424	7.36E-06
FAM120A	0.219426	0.032956
FAM120B	-0.27757	0.003854
FAM126A	-0.48504	0.001018
FAM126B	0.447454	0.000837
FAM127A	-0.70785	0.00022
FAM127B	-0.46464	0.000707
FAM127C	-1.39874	6.26E-06
FAM129A	-0.67187	0.008472
FAM129B	0.640823	0.011238
FAM131B	-1.43264	0.026419
FAM133B	0.368327	0.000171
FAM134A	-0.40295	0.009295
FAM134B	-2.0112	3.60E-17
FAM135A	0.780497	2.47E-05
FAM136A	0.986557	3.46E-08
FAM13B	-0.26326	0.044357
FAM156A	0.486437	0.013886
FAM160A1	0.551029	0.000224
FAM160B1	-0.29905	0.044953
FAM161A	-0.35921	0.030194
FAM162A	0.885841	5.90E-06
FAM167A	-0.85574	0.00217
FAM167B	-1.26816	0.030711
FAM168A	-0.78303	5.65E-07
FAM169A	-0.36557	0.003672
FAM171A1	-0.71452	4.66E-11
FAM171A2	-2.53336	8.55E-08
FAM171B	-0.47253	0.025691
FAM172BP	1.255463	0.000121
FAM174B	0.603725	0.019271
FAM181B	-1.30999	0.011683
FAM182B	-2.13428	0.028176
FAM183A	-1.39732	2.20E-05
FAM189A2	-1.5942	0.000385
FAM193A	-0.17862	0.027725
FAM198B	-4.36552	1.00E-07
FAM19A2	-1.76559	0.000479
FAM19A3	-1.31428	0.010251

FAM206A	0.425892	0.001474
FAM207A	0.446387	0.047764
FAM208A	0.257769	0.04236
FAM208B	0.386382	0.009281
FAM20B	0.328322	0.002685
FAM20C	-1.28464	4.37E-07
FAM210A	0.490462	0.000349
FAM210B	-0.4263	0.00023
FAM212B	-1.06272	1.05E-09
FAM212B-/	-4.0587	4.73E-05
FAM216A	0.812088	1.37E-05
FAM217B	0.51081	0.0004
FAM220A	-0.49862	0.004687
FAM225A	-0.81547	8.29E-07
FAM225B	-0.76825	0.005618
FAM227A	-0.51235	0.001406
FAM228B	-0.41948	0.001834
FAM229B	-0.96653	4.53E-05
FAM234A	-0.75702	8.90E-05
FAM234B	0.495447	0.000191
FAM24B	0.414292	0.007281
FAM25A	2.828458	4.22E-08
FAM25BP	2.130943	0.029905
FAM25G	3.53235	0.037582
FAM26E	-1.82591	6.30E-07
FAM27E3	0.388733	0.013385
FAM3C	0.887829	4.92E-10
FAM3C2	0.916111	1.23E-07
FAM43B	-2.5794	0.002508
FAM46B	-1.3227	1.14E-09
FAM47E-ST	-0.5638	0.004305
FAM49A	-0.87223	0.002719
FAM53B	-0.66294	0.004897
FAM57A	-0.24527	0.011432
FAM65A	-1.0437	6.07E-06
FAM69A	-0.72986	5.81E-07
FAM71E1	-0.90151	0.003722
FAM72A	0.73305	0.011724
FAM72B	0.769793	0.005563
FAM72D	0.812837	0.021172
FAM76A	-0.35853	0.007773
FAM78A	-0.95529	0.026797
FAM81A	0.433717	0.018832
FAM83A	2.662058	3.78E-20
FAM83A-A:	3.329254	4.49E-25
FAM83B	0.512493	0.000669
FAM84A	0.829333	9.86E-05

FAM84B	0.594042	3.27E-07
FAM86DP	-0.52926	2.10E-05
FAM86JP	-1.14867	2.75E-05
FAM87A	-2.04546	0.002174
FAM89A	-0.48009	0.000307
FAM89B	-1.09019	0.001689
FAM8A1	0.318436	0.003514
FAM90A24	3.368998	0.015753
FAM91A1	0.475243	0.004904
FAM95B1	2.058495	4.44E-05
FAM95C	1.305133	4.22E-05
FAM96A	0.538076	0.000327
FAM98A	0.520303	0.000432
FAM98B	0.374278	0.007646
FAM9B	1.359499	0.014977
FAN1	0.224971	0.00903
FANCA	0.439322	0.001258
FANCB	0.585511	0.000325
FANCE	0.343919	0.000927
FANCF	-0.356	0.001405
FANCI	0.390827	0.013365
FANCL	0.450433	0.015956
FANCM	0.502029	0.005081
FANK1	-1.00311	2.18E-07
FAR2	0.933359	7.99E-08
FARP1	-0.70507	4.79E-07
FARSA	0.46608	0.020213
FARSB	0.587199	0.000387
FASTKD1	0.74814	0.000115
FASTKD2	0.502346	0.002921
FASTKD3	0.516716	0.001948
FASTKD5	0.610242	0.001198
FAT1	-0.80378	4.24E-06
FAT2	-1.65983	2.04E-14
FAT3	-2.92417	3.28E-09
FAT4	-3.25016	5.65E-64
FAU	0.341854	0.006206
FAXC	-0.58153	7.17E-06
FAXDC2	0.541498	0.004912
FBL	0.654302	5.48E-11
FBLIM1	-1.25077	4.46E-08
FBLN1	-1.02479	3.71E-06
FBLN2	-3.63962	0.003975
FBLN5	-3.54828	4.79E-16
FBN1	-4.05827	2.34E-35
FBN2	-0.96891	1.46E-21
FBN3	-2.30331	0.0322

FBP2	-1.66583	0.025367
FBXL12	-0.43254	0.025068
FBXL2	-0.47581	2.75E-06
FBXL20	-0.46876	6.46E-05
FBXL4	0.303064	0.03822
FBXO11	0.5431	4.75E-06
FBXO16	-0.39473	0.020798
FBXO17	-0.87664	0.000209
FBXO2	-1.05957	0.001005
FBXO22	0.346375	0.002544
FBXO28	0.44674	0.000104
FBXO3	0.524001	4.37E-05
FBXO33	0.60701	0.001673
FBXO42	-0.24034	0.017511
FBXO45	0.492535	0.000176
FBXW2	-0.17824	0.014965
FBXW4	-0.44131	0.044574
FBXW8	-0.38427	0.005136
FBXW9	-0.46202	0.018285
FCGRT	-2.16582	0.000723
FCHO2	0.353415	0.020914
FCHSD2	-0.28883	0.001348
FCRLB	0.97525	0.021781
FDFT1	-0.32401	0.023296
FDX1	0.463467	0.00276
FDXACB1	0.648787	9.82E-05
FECH	-0.60525	1.31E-06
FEN1	0.425165	0.004514
FER1L4	0.743116	9.72E-05
FER1L5	-0.49957	0.046248
FERMT1	1.038992	0.000442
FERMT2	-1.15763	2.27E-15
FEZ1	-0.81298	0.000286
FGD3	-1.50605	5.95E-10
FGD4	-0.65117	7.72E-06
FGD5-AS1	-0.24721	0.005693
FGD6	0.655261	0.000455
FGF1	-1.97641	4.16E-09
FGF11	-0.58693	0.01756
FGF18	-1.2168	1.33E-05
FGF2	-0.43943	0.013644
FGFR2	-2.16937	2.86E-43
FGFR3	-0.85918	2.83E-05
FHDC1	-0.30664	0.002847
FHL1	1.03763	1.13E-07
FHOD3	-0.82203	2.59E-10
FIBCD1	-1.65553	0.003037

FIGN	-0.68971	2.65E-05
FILIP1L	-1.11725	5.24E-09
FIP1L1	0.33256	5.69E-05
FIRRE	0.503544	0.006816
FITM1	-2.87546	0.005861
FJX1	-0.57262	0.037444
FKBP1A	0.343947	0.037244
FKBP1B	-1.02238	2.04E-05
FKBP4	0.776425	9.35E-12
FKBP5	0.814811	9.81E-12
FKBP7	-1.21746	0.000101
FKBP9	-0.93227	1.74E-09
FLCN	-0.76813	4.43E-05
FLG	-1.67157	4.01E-06
FLG-AS1	-1.40901	0.010012
FLJ16779	-4.48354	5.78E-05
FLJ22447	-0.79033	5.46E-05
FLJ30679	1.699235	0.023297
FLJ37453	-0.441	0.034941
FLJ42969	-1.05047	0.000818
FLJ46284	-2.54009	8.95E-06
FLNA	-1.15934	1.98E-05
FLNC	-0.85283	0.00054
FLOT1	-0.43258	0.006005
FLOT2	-0.48753	0.008874
FLRT2	-0.94395	0.000119
FLRT3	-0.77825	0.006338
FLVCR2	-1.06864	1.83E-12
FLYWCH1	-0.80644	0.018377
FMN1	0.349096	0.028315
FMN2	-1.95297	2.45E-05
FMNL2	0.77092	7.59E-06
FMO4	-1.30669	0.010684
FMR1-AS1	1.202617	0.007851
FN1	-1.52275	2.05E-09
FNBP1L	-0.79573	3.97E-12
FNBP4	0.250532	0.009467
FNDC3B	-0.57687	8.82E-10
FNDC5	-1.06718	0.007662
FNIP1	-0.28987	0.035937
FNIP2	-1.05021	1.91E-11
FNTA	0.291326	0.010956
FOCAD	0.415267	0.008025
FOLR1	-2.47814	9.30E-05
FOLR3	-1.72264	0.011447
FOS	2.445991	1.70E-38
FOSB	1.124618	3.06E-07

FOSL1	1.970614	4.49E-31
FOSL2	-0.77458	1.23E-05
FOXA1	0.860209	2.76E-08
FOXB1	-1.43755	5.80E-07
FOXC1	-0.58486	0.000912
FOXC2	1.761669	8.36E-11
FOXCUT	-2.0203	2.55E-05
FOXD1	0.620546	0.000476
FOXD1-AS1	1.055046	1.25E-06
FOXD2	-0.67533	0.006892
FOXD3-AS1	-1.32144	9.20E-05
FOXF2	0.410486	8.85E-05
FOXJ2	-0.6853	0.000595
FOXJ3	0.567452	1.85E-07
FOXL1	2.103709	2.32E-13
FOXL2NB	-0.40195	0.000406
FOXM1	-0.20563	0.014688
FOXN2	2.106252	1.96E-14
FOXN3	-1.87431	3.33E-12
FOXO3	0.428806	3.72E-06
FOXO3B	-0.48495	0.02217
FOXO6	-1.0989	0.000438
FOXP1	-1.00091	2.25E-12
FOXP4-AS1	1.081699	0.005856
FOXQ1	2.263736	4.32E-78
FOXS1	-3.19833	0.031205
FPR3	-2.53614	0.000191
FRA10AC1	0.289873	0.035164
FRAS1	-1.70509	2.38E-09
FREM2	-1.37928	0.025006
FRG1GP	1.208651	0.041663
FRG2B	3.034362	0.028281
FRMD4A	-0.35662	0.006607
FRMD5	-1.03367	3.36E-11
FRMD6	-0.86528	2.23E-10
FRMD6-AS:	-0.607	0.002595
FRS2	-0.34223	0.000983
FRY	-1.86016	3.53E-05
FSBP	-0.9726	0.033272
FSCN1	-0.99777	0.004958
FSCN2	-2.3722	0.000657
FSD1	-0.45493	0.005724
FSD1L	-0.40957	0.005378
FSIP2	-0.44965	0.012983
FST	1.917041	0.000997
FSTL1	-1.59088	3.91E-13
FTL	-0.415	0.00019

FTLP14	0.895734	0.016327
FTO	-0.2942	0.017887
FTSJ3	0.440074	0.004437
FTX	-0.29138	0.035777
FUBP3	0.21199	0.016473
FUCA1	0.400565	0.015881
FUT3	1.213719	2.45E-15
FXN	0.479915	0.003178
FXYD3	0.440691	0.00694
FXYD5	0.840881	5.83E-05
FYB	1.584461	4.51E-09
FYN	-1.14686	1.58E-06
FZD1	-1.46676	1.24E-10
FZD10-AS1	-1.11702	5.18E-17
FZD2	-1.72917	6.63E-25
FZD3	0.221498	0.042227
FZD6	-0.334	0.011013
FZD7	-0.8662	8.51E-08
G3BP1	0.336257	0.001015
G6PD	-0.82457	2.30E-07
GAA	-1.19164	0.003898
GABARAP	-0.35459	0.011566
GABARAPL	-1.74263	6.12E-10
GABBR1	-0.74965	2.60E-05
GABBR2	-3.13002	1.85E-10
GABPA	0.384879	0.008721
GABPB2	0.395907	0.001101
GABRA3	-3.41572	0.002891
GABRB3	-0.39175	0.020616
GABRE	0.606879	0.000124
GABRP	0.395492	0.047525
GABRQ	-1.61917	0.001769
GADD45A	-0.79756	6.35E-06
GADD45B	-0.58134	0.000944
GADD45G	-1.13896	0.006058
GAL	1.61238	1.22E-08
GAL3ST4	-1.34838	0.015321
GALK2	0.418554	0.003593
GALNS	-0.48277	0.012517
GALNT10	-1.05125	6.73E-15
GALNT12	0.664971	0.007705
GALNT13	-3.3507	0.00859
GALNT14	0.643255	0.000132
GALNT15	-1.75152	0.000102
GALNT18	-1.91006	3.94E-09
GALNT2	-1.14149	1.03E-11
GALNT5	2.065479	0.012951

GALNT6	-0.88749	3.31E-09
GAMT	-0.81698	0.031316
GAN	0.32511	0.019894
GANAB	-0.29765	0.013745
GAPDHP32	-3.21257	0.015762
GAR1	0.508246	9.39E-06
GAREM1	0.475537	1.02E-05
GAREM2	-0.39659	0.042939
GARNL3	-0.46717	0.001226
GART	0.854731	1.23E-07
GAS1	-2.22573	1.84E-07
GAS2	-1.80397	0.040579
GAS5	0.575771	0.000452
GATA2-AS1	-0.31554	0.044764
GATA3	-0.27831	0.023178
GATA6	-0.5502	0.000118
GATA6-AS1	-1.19197	0.010521
GATAD1	0.152736	0.046816
GATB	0.231466	0.035621
GATC	0.212226	0.032758
GATM	1.019574	2.11E-05
GATS	-0.86617	5.95E-05
GATSL2	-0.46382	0.007486
GATSL3	-1.09269	0.016113
GBA	-0.40108	0.009987
GBAP1	-0.57103	0.011468
GBE1	0.425847	0.014855
GBF1	-0.27365	0.008735
GBP1	-3.19227	1.35E-18
GBP2	-1.52181	3.88E-05
GCC1	-0.15616	0.042336
GCC2	0.416632	0.003569
GCFC2	0.732645	1.99E-07
GCH1	0.593683	1.25E-05
GCKR	1.912622	0.000764
GCLC	0.374576	0.020335
GCN1	0.274516	0.008677
GCNT1	-1.76839	4.11E-19
GCNT4	-2.69176	5.41E-45
GCSAML-A!	1.727225	3.36E-05
GCSH	0.446463	0.004582
GCSHP5	0.47944	0.04029
GDA	1.037155	0.00273
GDAP1	0.588833	0.002095
GDAP2	0.397438	0.003398
GDE1	0.314311	0.000193
GDF11	-0.32918	0.02134

GDI2	0.24681	0.035929
GDNF	-2.45518	2.81E-12
GDNF-AS1	-1.63233	0.004627
GDPD3	1.933225	1.02E-07
GDPD5	-0.64584	0.006949
GEM	1.283747	0.000178
GEMIN4	0.444815	0.000956
GEMIN5	0.796518	1.04E-06
GEMIN6	0.600848	0.001083
GEMIN7	0.37417	0.004222
GFI1	1.627618	2.01E-23
GFI1B	-0.56631	0.008312
GFM1	0.677719	0.000172
GFOD1	-1.02673	3.98E-20
GFPT2	0.950139	5.55E-10
GGACT	0.570156	0.004825
GGCT	0.276436	0.027933
GGCX	-0.18646	0.0126
GGT1	-0.75126	0.048018
GGT2	-5.53832	1.08E-05
GGT3P	-5.09535	3.32E-06
GGT7	-0.96795	0.002489
GGTLC1	-4.60686	0.000692
GGTLC3	-6.23287	2.82E-10
GGTLC5P	-4.15885	0.000626
GHET1	0.596258	0.010352
GHITM	0.375517	0.00228
GHR	-1.04661	0.023342
GHRLOS	0.652968	0.02779
GIGYF2	0.234383	0.001393
GINS2	0.388245	0.003636
GINS3	0.629974	2.01E-07
GIT2	-0.25638	0.016829
GJA1	-3.81805	2.36E-08
GJA3	0.64773	0.004008
GJB2	1.047313	2.52E-05
GJB3	0.635982	0.013095
GJB4	0.957294	0.001392
GJB5	0.521283	0.020512
GJB6	1.248176	0.002134
GK	0.912448	5.07E-08
GK5	0.49574	0.000458
GK-AS1	1.904109	0.008562
GLA	0.626352	3.61E-05
GLB1	-0.42455	0.004421
GLB1L	-0.80013	0.000334
GLB1L2	0.825012	1.64E-07

GLCCI1	0.512771	0.00176
GLDC	1.306308	2.53E-06
GLDN	-1.46871	0.001414
GLE1	0.19524	0.041422
GLG1	-0.26473	0.000354
GLI1	-1.8289	0.002379
GLI2	-1.14853	0.001916
GLI3	-1.19491	6.25E-14
GLI4	-0.63375	0.023361
GLIPR1	-0.80831	7.90E-05
GLIPR2	-2.42226	3.54E-13
GLIS2	-1.79297	1.66E-05
GLIS3	-1.03036	4.23E-07
GLMN	0.725016	0.000226
GLO1	0.280101	0.045982
GLOD4	0.283018	0.019664
GLP2R	-1.23931	0.000111
GLRX2	0.673642	8.20E-06
GLRX3	0.323694	0.014795
GLS	-0.21748	0.024541
GLT8D1	-0.4863	8.21E-08
GLT8D2	-0.84715	0.008313
GLTP	0.365637	0.0178
GLUD1	0.334582	0.000441
GLUD1P3	-0.59522	0.028617
GLUL	-0.81841	1.35E-09
GM2A	-0.45155	0.00055
GMFB	0.313564	0.032919
GMPR	-1.19534	0.0057
GMPR2	0.341969	0.000511
GMPS	0.523042	0.000431
GNA12	-0.49828	6.29E-06
GNA13	-0.35797	0.033795
GNAI1	-0.81629	9.72E-18
GNAI2	-0.4086	0.035492
GNAL	0.568202	0.001832
GNAO1	-2.77153	1.08E-12
GNAQ	-0.78488	2.93E-09
GNAS	-0.53283	8.25E-05
GNAT3	-3.18965	1.30E-06
GNAZ	-0.95607	0.001065
GNB1	-0.181	0.028042
GNB5	-0.26781	0.013234
GNE	2.049168	1.27E-10
GNG12	-0.26631	0.034255
GNG2	-1.57988	1.19E-06
GNG4	0.472334	7.18E-06

GNGT1	-1.00639	0.003091
GNGT2	2.503812	7.10E-06
GNL2	0.550778	3.07E-05
GNL3	0.647592	0.0001
GNL3L	0.616558	3.85E-06
GNPDA1	0.177336	0.025804
GNPNAT1	0.650667	1.12E-05
GNPTAB	-0.70866	2.86E-11
GNS	-0.27651	0.001112
GOLGA2	-0.50834	0.000395
GOLGA2P1	-0.94992	0.001581
GOLGA2P7	-0.73089	0.001918
GOLGA6L1(	-0.62035	2.81E-05
GOLGA6L3	-3.24001	0.001029
GOLGA6L4	-0.47742	0.026625
GOLGA6L5I	-0.85417	2.16E-08
GOLGA6L9	-0.41457	0.000429
GOLGA7B	-0.55694	0.014055
GOLGA8A	0.262618	0.017366
GOLGA8DP	-4.32323	0.001108
GOLGA8F	-5.9686	1.67E-06
GOLGA8G	-3.54581	3.27E-09
GOLGA8N	-1.18598	0.000218
GOLIM4	-0.50908	5.83E-08
GOLM1	0.634764	8.63E-06
GOLPH3L	0.294275	0.041247
GON7	0.528428	0.00133
GOPC	-0.91548	9.80E-13
GORASP2	0.154668	0.030025
GOT1	0.408054	0.005329
GOT2	0.425786	0.00252
GPAM	0.349202	0.010745
GPAT3	2.345758	3.57E-17
GPATCH11	0.306401	0.006707
GPATCH4	0.573983	1.01E-05
GPC1	-1.26895	1.72E-05
GPC2	-1.83165	1.33E-05
GPC4	-2.69728	4.41E-20
GPD1L	0.851909	1.09E-05
GPD2	0.477095	0.005533
GPER1	-2.61913	6.42E-14
GPHA2	2.851734	0.033302
GPHN	0.450243	4.26E-05
GPI	0.528359	7.62E-06
GPM6B	0.54617	0.032434
GPN1	0.219237	0.026291
GPN3	0.611912	0.000251

GPNMB	-1.79729	1.67E-05
GPR1	-2.14728	4.43E-45
GPR107	-0.24964	0.017114
GPR132	0.67105	0.000262
GPR146	-1.98546	0.00094
GPR156	-1.10461	8.11E-06
GPR158	-0.34949	0.027638
GPR160	1.336146	1.22E-12
GPR161	-1.55605	2.89E-20
GPR17	-2.32798	6.99E-10
GPR173	-1.59842	0.002867
GPR176	-1.23554	2.90E-14
GPR183	-1.34825	0.035111
GPR3	0.991963	0.000333
GPR37	-1.35566	0.001264
GPR78	-0.4505	0.012098
GPR89A	0.338092	0.048556
GPRC5A	0.853885	5.35E-06
GPRC5B	-0.62613	0.000399
GPRC5C	-1.56279	2.96E-06
GPRIN2	-2.36018	3.28E-06
GPT2	-0.73434	0.003623
GPX4	-0.67656	0.000114
GPX8	-0.57612	0.00239
GRAMD1B	2.513756	1.02E-22
GRAMD2	-0.86102	7.46E-05
GRAMD3	-0.4815	0.000616
GRB10	-0.56251	0.001534
GRB7	0.85668	7.48E-06
GREB1L	-0.46776	0.000162
GRHL1	1.187546	1.39E-08
GRHL2	-0.54264	5.43E-05
GRHL3	2.418179	0.000162
GRIA4	-1.91363	0.026714
GRID1	-1.36337	4.01E-05
GRIK2	-2.21681	1.43E-05
GRIN2C	-1.9132	0.000517
GRIN2D	-0.51994	0.030127
GRIP1	-1.08336	5.61E-07
GRK5	-0.80624	6.71E-05
GRM4	-1.20357	0.034119
GRPEL1	1.064663	3.83E-12
GRPEL2	0.454676	0.003933
GRSF1	0.477848	0.000194
GRWD1	0.4539	0.044411
GS1-166A2	-2.69066	0.034267
GS1-24F4.2	-2.03629	0.033925

GS1-259H1	-1.42136	0.02394
GSAP	1.173636	2.10E-11
GSDMA	1.165246	0.012394
GSDMB	-0.46428	0.002587
GSDMC	1.775447	5.16E-09
GSG2	0.184203	0.046327
GSN	-0.72028	0.000575
GSPT1	0.516562	6.39E-05
GSTA4	-1.04965	0.01539
GSTCD	0.461507	0.009239
GSTK1	-0.37878	0.002521
GSTM3	-0.74619	0.000312
GSTM4	-4.74588	4.46E-08
GSTO1	0.819735	1.53E-10
GSTP1	-0.44338	0.015266
GSTZ1	-0.36809	0.037379
GTF2A2	0.489784	0.000172
GTF2B	0.709268	4.10E-07
GTF2E1	0.435106	0.000243
GTF2E2	0.371931	0.002593
GTF2H2	0.761678	2.23E-05
GTF2H2B	0.891002	0.00148
GTF2H2C	0.591135	0.001236
GTF2H3	0.536234	0.003168
GTF2IP4	-0.29015	0.01333
GTF2IRD1	-0.4644	0.019943
GTF2IRD2	-0.54145	0.000952
GTF2IRD2B	-0.68432	0.001587
GTF3A	0.397359	0.002495
GTF3C3	0.361471	0.01322
GTF3C6	0.46794	0.006889
GTPBP10	0.811182	2.16E-05
GTPBP4	0.871187	4.90E-07
GTPBP8	0.523199	0.001312
GUCA2A	1.940133	0.00685
GUCY1A2	-1.78251	3.45E-06
GUCY1A3	2.416843	3.43E-05
GUCY1B2	-1.35555	1.73E-05
GUF1	0.475704	0.004067
GUSBP2	-0.46847	0.009247
GUSBP9	-0.6452	0.013598
GXYLT1	0.542519	4.22E-05
GXYLT2	-1.06439	0.002584
GYG1	0.494922	0.00115
GYG2	-1.6918	3.33E-15
H19	-0.81351	0.033096
H1FX	-0.52821	0.030864

H1FX-AS1	-1.55361	2.17E-06
H2AFJ	-0.39989	0.010422
H2AFY	0.227518	1.46E-05
H2AFY2	-0.72641	5.28E-05
H2AFZ	0.32567	0.031703
H3F3AP4	-0.83406	0.001346
H3F3B	0.220405	0.005066
H6PD	-1.00954	1.57E-06
HABP4	-0.66279	5.50E-05
HACD1	-0.59569	2.49E-08
HACD2	-0.49186	3.68E-05
HAL	1.795806	0.006661
HAPLN3	-1.15364	1.87E-05
HARS	0.372669	0.004359
HAS2	4.912203	6.06E-36
HAS2-AS1	3.244548	1.99E-38
HAS3	1.583462	8.83E-14
HAUS2	0.483447	0.000515
HAUS3	0.504245	0.00037
HAUS6	0.548174	0.000178
HAUS7	0.294219	0.020407
HBEGF	2.011662	9.05E-34
HBP1	-0.38212	0.049024
HCAR2	-1.83753	1.11E-14
HCAR3	-1.09309	0.000551
HCCS	0.30445	0.016069
HCFC1R1	-0.75579	0.034388
HCG15	-0.59934	0.032426
HCN3	-0.70993	0.005307
HCN4	-4.45357	0.00236
HDAC1	0.236624	0.043432
HDAC11	-0.91483	0.002908
HDAC2	0.264623	0.013484
HDAC5	-0.92887	9.01E-05
HDAC6	-0.70548	0.000121
HDAC7	-0.52663	0.034086
HDAC9	0.859671	8.73E-05
HDLBP	-0.17139	0.018885
HEATR1	0.47391	0.0033
HEATR3	0.431766	0.009472
HEATR5A	-0.2744	0.013423
HEATR6	-0.25005	0.04876
HECTD1	0.242662	0.03662
HECTD4	-0.45862	0.001259
HECW1	-1.36063	0.003442
HEG1	-3.15159	1.85E-24
HEIH	-0.50083	0.0013

HELB	0.524758	0.001271
HELLS	0.55104	0.006615
HELQ	0.526952	0.002787
HELZ	-0.56825	3.74E-07
HEPHL1	1.185211	0.000339
HERC1	-0.30744	0.001549
HERC2	-0.53966	3.02E-05
HERC3	0.363122	0.005068
HERC4	0.317352	0.047578
HERC5	0.44954	0.043165
HERPUD1	-0.47472	0.01489
HERPUD2	-0.2071	0.02891
HES1	0.757107	7.31E-12
HES6	-1.08822	0.006243
HEXA	-0.24882	0.002508
HFE	-0.60317	0.001841
HGFAC	-2.64994	0.000148
HGSNAT	-0.72297	9.13E-13
HHAT	-1.52142	2.89E-17
HHIP-AS1	-2.71979	0.032685
HHIPL1	-1.2311	0.016017
HHLA1	1.311838	8.38E-07
HHLA3	-0.82373	4.99E-05
HIBADH	-0.29294	0.015569
HIF1A-AS2	0.826236	0.018866
HIGD1A	0.499484	0.002991
HIGD2A	0.280595	0.008729
HILPDA	-0.43223	0.007627
HINFP	-0.26089	0.028742
HIP1	0.603668	5.33E-06
HIP1R	-1.04188	7.66E-06
HIPK2	-0.56388	0.001431
HIST1H1D	1.003578	0.024826
HIST1H2BC	0.404514	0.02647
HIST1H2BD	-0.5	0.006499
HIST1H2BG	0.431226	0.035426
HIST1H2BJ	0.361704	0.039908
HIST1H4H	0.284709	0.04213
HIST2H2BF	0.569062	0.027874
HIST2H4A	1.550003	0.000258
HIST3H2A	0.331779	0.047513
HIST3H3	-2.88234	0.024997
HIVEP1	-0.59745	2.43E-05
HIVEP2	0.359847	0.011162
HIVEP3	-0.51074	0.000153
HK2	0.838107	8.11E-09
HK2P1	0.784307	0.036784

HLA-B	-0.8163	7.80E-05
HLA-C	-0.43497	0.01729
HLA-DOA	0.896242	0.013589
HLA-F	-0.70264	0.000789
HLA-L	-0.48359	0.043648
HLA-V	0.471085	0.028299
HM13	0.385909	0.000237
HMBS	0.250841	0.036064
HMCN1	-4.07179	7.63E-11
HMG20B	-0.53012	0.02102
HMGA1	1.56073	2.18E-10
HMGA2	1.741735	6.30E-07
HMGB3	-0.44233	0.000356
HMGB3P32	2.034754	0.028052
HMGCR	-0.61251	8.58E-05
HMGCS1	-0.66269	0.000163
HMGN1P15	-2.97216	0.032026
HMGN2P4(	-0.50028	0.022051
HMGN3	-0.54581	0.0089
HMMR	0.519729	0.046601
HMOX1	-0.97926	1.40E-05
HMOX2	0.296499	0.026957
HMX3	-0.99837	0.032004
HN1	-0.42898	7.56E-09
HNMT	1.335275	0.017925
HNRNPA1	0.347743	0.002083
HNRNPA1L	-0.31636	0.016885
HNRNPA1P	-0.69679	0.036516
HNRNPA1P	-0.56042	0.013384
HNRNPA1P	-2.91993	0.021601
HNRNPA1P	0.520713	0.044233
HNRNPA2B	0.352655	0.008295
HNRNPA3P	1.091179	0.03369
HNRNPAB	0.435093	0.000124
HNRNPC	0.302956	0.004949
HNRNPCP1	0.956938	0.002971
HNRNPD	0.39448	0.001315
HNRNPF	0.557615	3.75E-10
HNRNPK	0.342321	0.004883
HNRNPLL	-0.21886	0.02702
HNRNPU	0.370505	0.003247
HOGA1	-1.0433	0.002937
HOMER1	0.603372	1.20E-05
HOMER2	-0.23537	0.035931
HOMER3	-0.74844	0.002646
HOOK1	0.664748	0.000176
HOTAIRM1	0.450634	0.004503

HOXA1	0.584805	0.00105
HOXA10	0.519583	9.22E-07
HOXA11	0.409397	0.044515
HOXA13	-0.87103	0.000833
HOXA5	0.773168	0.00735
HOXC10	-0.54475	0.002783
HOXC11	-0.70762	0.003583
HOXC13	-0.90974	1.14E-06
HOXC13-AS	-1.24249	3.58E-09
HOXC4	-0.98828	3.03E-06
HPCAL1	1.041466	2.85E-14
HPDL	1.022854	3.28E-05
HPGD	0.871011	0.028672
HPRT1	0.684576	0.000308
HPS1	-0.41349	0.012128
HPS4	-0.24845	0.007171
HPSE	0.517925	0.018993
HR	0.67463	0.032017
HRAT92	-1.50586	1.03E-05
HRH1	0.798461	3.01E-07
HRK	-1.26641	0.008806
HRNR	-1.52017	0.00537
HS1BP3	-0.58886	0.023375
HS3ST1	1.477677	2.48E-12
HS3ST5	-1.66772	0.000582
HS6ST1	-1.27621	1.56E-05
HS6ST2	0.464037	0.006809
hsa-mir-54	0.693438	0.039006
HSBP1	0.304926	0.011024
HSBP1L1	0.507625	0.000236
HSD11B1	2.084719	0.000854
HSD11B2	0.487688	0.000375
HSD17B1	-0.70155	0.010928
HSD17B12	0.499192	0.001162
HSD17B7P2	-0.43431	0.025993
HSD3B1	1.026677	0.015116
HSD3B7	-0.50701	0.044731
HSDL2	0.421057	0.014612
HSF2BP	-0.46647	0.006097
HSFX1	-3.26436	0.001134
HSH2D	2.76459	1.23E-17
HSP90AA1	0.746984	0.000167
HSP90AB1	0.420073	0.000634
HSPA12A	-0.93031	3.69E-09
HSPA14	0.629444	0.000474
HSPA1A	1.044591	0.002385
HSPA1B	0.839046	0.001564

HSPA2	-1.2522	1.91E-08
HSPA4	0.659167	0.000136
HSPA4L	0.936505	3.11E-05
HSPA6	1.474175	6.94E-05
HSPA8	1.084773	7.66E-07
HSPA8P15	0.782571	0.029479
HSPA9	0.579461	1.36E-05
HSPB1	-1.04144	2.27E-06
HSPB8	-1.028	5.99E-11
HSPBAP1	0.428683	0.000507
HSPD1	0.853306	1.26E-07
HSPD1P11	-1.86641	2.92E-06
HSPD1P6	2.38804	0.000199
HSPE1	0.763801	6.55E-05
HSPG2	-1.40132	7.64E-06
HSPH1	0.64394	0.000637
HTR1D	-1.3743	1.04E-20
HTR2B	1.659776	0.001806
HTR7	0.241107	0.007081
HTR7P1	0.554807	0.036227
HTRA1	-1.32695	0.00042
HTRA2	0.394027	0.006946
HTRA3	-2.46796	1.35E-30
HTT	-0.44419	4.04E-05
HUNK	-1.20858	0.000177
HUS1	0.296296	0.029809
HYAL2	-0.43705	0.041552
HYDIN2	-2.80183	0.001836
HYPK	0.438671	0.001426
IAH1	0.382015	0.042337
IARS	0.357608	0.006203
IARS2	0.38709	0.027727
IBSP	-1.53449	0.03153
ICAM1	-0.51032	0.000202
ICAM4	-3.37551	0.016237
ICAM5	-0.68469	0.022055
ICOSLG	-1.29027	3.17E-06
ID3	-1.93573	4.55E-21
ID4	-2.70999	3.07E-12
IDE	0.246594	0.046107
IDH2	-1.40191	1.17E-08
IDH3A	0.935909	3.80E-07
IDI2	1.81189	0.034553
IDS	0.421075	0.005101
IER2	1.439292	9.56E-11
IER3	0.826418	7.51E-07
IER5	0.967708	9.31E-07

IFFO2	-1.10803	2.12E-08
IFI44	-0.55475	0.001105
IFI44L	-1.5681	0.004627
IFI6	-2.20207	0.003523
IFIT1	-0.8356	0.000188
IFIT2	-0.96663	0.000449
IFIT3	-0.77327	0.002319
IFITM10	-1.36236	0.013938
IFNAR2	-0.39895	1.00E-06
IFNE	1.034601	0.000219
IFNGR2	0.498017	4.16E-05
IFRD2	0.508175	0.000306
IFT122	-0.71271	2.72E-09
IFT140	-1.00585	1.67E-05
IFT172	-0.35766	0.003303
IFT22	-0.549	0.000333
IFT27	-0.66419	5.76E-05
IFT43	-0.48211	0.000254
IFT81	-0.3949	0.00491
IGDCC3	-2.25181	7.28E-07
IGDCC4	-0.93941	0.010904
IGF1R	-0.91426	2.74E-16
IGF2	0.840771	0.000182
IGF2BP2	0.413853	0.000144
IGF2BP2-A:	1.058801	0.0165
IGF2BP3	-0.33336	0.002833
IGF2R	-0.42185	0.001111
IGFBP1	2.443759	5.99E-08
IGFBP3	-0.49498	0.000317
IGFBP4	-0.576	0.030881
IGFBP5	-5.60345	6.17E-11
IGFBP7	-2.96991	2.98E-18
IGFBP7-AS:	-2.87607	0.012377
IGFL1	-1.24738	0.001731
IGFL1P1	-1.96172	0.000546
IGFL2	-1.35301	0.004082
IGFL2-AS1	-1.27909	7.26E-05
IGFLR1	-0.78799	0.009781
IGFN1	3.072486	1.13E-14
IGHMBP2	0.31773	0.041926
IGSF1	-0.95823	4.61E-06
IGSF11	-0.68917	0.001344
IGSF3	-0.82856	6.59E-09
IGSF9	-1.20978	0.000109
IGSF9B	0.44334	0.038421
IK	0.189182	0.023575
IKBIP	-0.62196	7.98E-08

IKBKAP	0.521464	0.000673
IKZF2	0.685237	0.001637
IKZF5	0.362022	0.001274
IL10RA	1.498281	0.041092
IL11	1.559828	4.67E-13
IL12A	0.92549	3.18E-08
IL17D	0.907198	7.56E-10
IL17RB	0.533122	0.009075
IL17RC	-0.70954	0.0025
IL17RD	-0.80148	1.10E-05
IL18	0.452179	0.03826
IL18BP	-1.00644	1.45E-05
IL18R1	1.600625	3.99E-09
IL1A	1.568433	8.58E-06
IL1B	2.192138	2.28E-12
IL1R1	0.965865	1.53E-11
IL1R2	4.956045	3.40E-13
IL1RAPL1	4.334545	3.35E-33
IL1RAPL2	1.315458	0.000723
IL1RL1	8.401451	4.78E-46
IL1RL2	0.931943	1.03E-08
IL1RN	3.200562	3.62E-09
IL20RA	1.599792	1.23E-31
IL23A	1.068168	6.87E-07
IL24	2.935648	2.91E-09
IL2RB	-1.99077	0.004138
IL32	-2.476	3.41E-20
IL36RN	-0.6729	0.021913
IL37	3.732736	1.58E-12
IL6R	1.75129	3.37E-29
ILF2	0.184701	0.014107
ILK	-0.65652	3.74E-05
ILKAP	0.218476	0.028898
IMMP2L	-0.32657	0.040133
IMMT	0.282407	0.019402
IMP3	0.288341	0.030559
IMP4	0.409725	0.00067
IMPA1	0.340948	0.026045
IMPA2	0.503551	0.008476
IMPDH2	0.58958	1.25E-07
IMPG2	-1.13835	0.007502
INAFM1	-0.73432	0.033642
INAFM2	-0.53216	0.000112
ING2	0.330003	0.034003
ING4	-0.96227	2.76E-06
INHA	-1.2287	0.001875
INHBA	-0.74527	2.06E-05

INHBE	-1.61856	0.000251
INIP	0.283462	0.017444
INO80	0.165164	0.029592
INO80B-WI	-0.66589	0.044254
INO80C	0.294687	0.044657
INO80D	0.340136	0.011367
INPP1	0.40407	0.000538
INPP4B	0.293108	0.019973
INPP5A	-0.56861	4.69E-17
INPP5B	-0.52153	1.42E-09
INPP5F	0.335648	0.00552
INPP5J	-1.11441	0.001058
INPPL1	-0.47091	0.0435
INSIG1	-0.64875	0.001494
INSIG2	0.51371	0.001088
INSR	-0.38734	0.035907
INTS10	0.573559	0.000104
INTS12	0.327804	0.026842
INTS13	0.697685	1.50E-05
INTS6	0.425996	0.004723
INTS6L	-0.53278	0.000216
INTS7	0.273353	0.036442
INTS9	0.202638	0.046788
INTU	-0.39111	0.000863
IPO11	0.369614	0.018861
IPO4	0.350512	0.018584
IPO5	0.463198	0.002938
IPO5P1	-1.1461	1.51E-13
IPO7	0.483063	0.00093
IPO8	0.460964	0.014962
IPO9-AS1	-2.06489	0.012758
IPPK	0.611062	0.00237
IQCB1	0.361678	0.009776
IQCE	-0.74756	9.35E-07
IQCG	-0.45442	0.005853
IQCH-AS1	-0.67639	6.37E-05
IQCJ-SCHIP	-1.4799	7.79E-29
IQCK	-0.69389	0.002724
IQGAP2	-1.56988	0.03399
IQGAP3	-0.41001	2.31E-07
IQSEC1	-0.33554	0.031002
IQSEC2	-0.63133	0.022801
IQUB	-1.28782	0.025896
IRAK2	0.78356	1.97E-08
IRF1	-0.59474	0.000333
IRF2BP2	0.366979	0.017422
IRF9	-1.25377	0.000367

IRS1	-0.57004	0.013765
IRS2	-0.65478	0.000106
ISCA1	0.41971	0.000309
ISG15	-0.54969	0.0228
ISG20	1.92801	4.42E-09
ISG20L2	0.360673	0.002577
ISM1	1.513697	2.28E-06
ISM2	-1.00394	0.035287
ISOC1	0.297602	0.016307
ISPD	-0.75132	4.11E-05
ISPD-AS1	-0.96222	0.013537
ISYNA1	-0.55178	0.016323
ITCH	0.306603	0.027329
ITFG1	-0.24889	0.031827
ITGA1	2.012529	6.63E-21
ITGA2	1.29998	1.27E-09
ITGA2B	-1.29794	0.00719
ITGA4	-1.46562	9.75E-13
ITGA7	1.011959	0.001572
ITGAV	-1.13411	3.24E-19
ITGB1BP1	0.288454	0.040192
ITGB2-AS1	0.945995	0.035176
ITGB3	-2.9746	2.05E-19
ITGB3BP	0.39837	0.045411
ITGB5	-0.49644	0.001655
ITGB6	-1.73378	3.30E-35
ITIH5	-1.49867	0.000277
ITM2B	-0.53224	0.000542
ITM2C	-0.68965	0.019114
ITPR2	-0.6049	4.73E-07
ITPR3	0.430817	0.008042
ITPRIPL2	0.294391	0.000332
ITSN1	-0.59904	6.64E-09
IVNS1ABP	-1.3458	4.92E-13
IWS1	0.232811	0.045072
JADE1	-0.76408	8.83E-08
JADE2	-0.27625	0.017843
JADE3	0.224838	0.012209
JAG2	-0.65264	0.012499
JAGN1	0.295908	0.012873
JAK1	-0.43468	0.000349
JAKMIP3	-3.31957	0.00022
JAZF1	-0.75152	5.29E-05
JDP2	-1.5616	2.01E-10
JMJD6	0.535478	0.002256
JPH1	0.362069	2.76E-05
JPH2	-2.24399	3.18E-21

JPH3	-1.19869	5.22E-05
JTB	0.468279	4.57E-06
JUN	-0.71747	0.000545
JUND	-1.07218	2.53E-07
KALRN	-0.69937	0.011603
KANK4	-2.9123	2.17E-24
KANSL1	-0.41301	1.57E-06
KANSL1L	-0.52642	6.40E-05
KANTR	0.509846	0.000888
KARS	0.495577	2.91E-06
KAT14	-0.81817	7.18E-06
KAT2A	0.412333	0.008896
KATNA1	0.263591	0.017734
KATNAL1	-0.82585	3.02E-12
KATNBL1	0.759231	4.12E-06
KAZALD1	-0.64792	0.043464
KAZN	-0.37999	0.015561
KB-1042C1	-1.45045	0.001724
KB-1208A1	0.537752	2.64E-05
KB-1410C5	-2.61345	0.047203
KB-1562D1	-1.64414	0.000331
KB-1732A1	-2.00758	4.99E-06
KB-176G8.:	-0.99158	0.002612
KB-1836B5	-1.46872	0.009661
KB-1995A5	-0.92397	0.001887
KBTBD11	0.401818	0.008677
KBTBD11-C	-1.73482	4.89E-05
KBTBD7	-0.21983	0.046779
KBTBD8	1.470702	2.34E-08
KC6	-0.86257	0.023307
KCNA7	-1.19065	0.000639
KCNC3	-4.29182	0.001756
KCNF1	-1.48262	0.000306
KCNG3	1.819403	0.000103
KCNH1	-4.21959	6.14E-30
KCNH1-IT1	-1.97516	0.000312
KCNH2	0.717969	0.004581
KCNH3	-2.04999	1.37E-10
KCNIP3	-0.68953	0.037488
KCNJ15	-1.76063	9.18E-12
KCNJ4	-1.59362	0.007137
KCNK1	0.853068	2.35E-06
KCNK2	1.602406	0.000369
KCNK3	-1.66343	0.005587
KCNK5	0.39072	0.031718
KCNMA1	-0.65553	0.007328
KCNMB4	-1.30532	1.16E-06

KCNN4	-0.48204	0.039514
KCNQ1	-1.06944	0.042309
KCNQ3	2.107425	0.012503
KCNQ4	2.159993	6.04E-13
KCNQ5	0.439314	0.034246
KCNQ5-IT1	2.192718	0.000509
KCNS3	-1.15075	8.26E-05
KCP	-0.81973	0.044637
KCTD12	1.14658	0.000316
KCTD15	-0.44296	0.004662
KCTD2	-0.30876	0.016674
KCTD20	-0.64694	1.43E-14
KCTD5	0.327918	0.000175
KCTD6	0.479035	0.002384
KCTD7	-0.77981	2.87E-10
KCTD9	0.509762	0.001585
KDELC1	-0.56956	0.000197
KDELC2	-0.57015	1.12E-07
KDELR1	-0.25624	0.029717
KDELR2	-0.1622	0.022281
KDELR3	-0.53082	7.15E-06
KDM1A	-0.3614	0.003893
KDM4A	-0.47474	5.97E-07
KDM4B	-0.83868	0.000517
KDM4D	-0.69155	0.048852
KDM5A	-0.2128	0.020598
KDM5B	-0.45941	2.37E-05
KDM6A	-0.9169	1.40E-08
KDSR	0.330528	0.000191
KHDRBS3	0.64168	0.00872
KIAA0040	0.305716	0.001536
KIAA0141	0.124079	0.049488
KIAA0232	-0.32276	0.005622
KIAA0319	-0.48697	0.008869
KIAA0319L	-0.48688	0.00228
KIAA0355	-0.64758	0.000271
KIAA0368	0.300058	0.034292
KIAA0513	-1.09083	0.000438
KIAA0556	-0.59936	0.001823
KIAA0895	-0.52933	4.53E-07
KIAA0895L	-0.87447	0.002859
KIAA0907	0.275658	0.024081
KIAA0922	-0.55309	2.58E-05
KIAA0930	-0.5787	0.000292
KIAA1024	-1.0631	0.025511
KIAA1109	-0.34984	0.006394
KIAA1143	0.277512	0.040296

KIAA1161	-0.86297	3.48E-05
KIAA1211	-1.06275	0.014161
KIAA1217	-1.1444	1.43E-09
KIAA1324	-0.84845	0.000533
KIAA1324L	-0.43354	0.00196
KIAA1524	0.545457	0.006973
KIAA1549	-0.79742	1.31E-05
KIAA1551	0.350151	0.02706
KIAA1586	0.305022	0.021819
KIAA1614	-1.34809	1.91E-05
KIAA1644	-1.64855	6.83E-11
KIAA1671	-0.63291	6.19E-11
KIAA1683	-2.27087	6.98E-05
KIAA1755	-1.01031	0.001889
KIAA1841	-0.53164	5.80E-06
KIDINS220	-0.34247	1.88E-05
KIF12	-2.31644	7.23E-06
KIF13A	-0.88084	3.02E-07
KIF13B	0.796132	4.54E-28
KIF16B	0.425447	5.92E-05
KIF17	-1.14532	0.009835
KIF1B	-0.47287	4.24E-07
KIF26A	-1.1583	0.005474
KIF26B	-2.10467	4.73E-07
KIF3C	-1.34921	2.11E-12
KIF5C	-1.17965	0.000108
KIF9	0.618979	0.004762
KIF9-AS1	-1.09765	0.000714
KIFAP3	-0.4758	0.000144
KIN	0.299746	0.045364
KIRREL	-0.72316	0.000408
KIT	3.911124	0.005254
KLC3	-0.54487	0.046511
KLC4	-0.83227	0.000129
KLF11	-0.7396	1.74E-07
KLF12	-0.78726	1.09E-08
KLF3	0.688942	3.25E-14
KLF4	0.341969	0.022237
KLF5	0.827452	1.39E-11
KLF7	-0.61289	1.98E-06
KLF7-IT1	1.568586	0.002131
KLF8	-1.17197	2.37E-15
KLHDC3	-0.6722	0.001232
KLHDC4	0.34978	0.037487
KLHDC7A	-2.566	0.000374
KLHDC8B	-1.3988	0.001089
KLHL11	0.519527	0.000255

KLHL13	-0.77143	6.75E-05
KLHL15	0.726296	0.000102
KLHL18	0.411305	0.001169
KLHL2	0.327924	0.018175
KLHL21	0.88715	0.000667
KLHL22	-0.69369	0.004798
KLHL23	0.463477	0.004112
KLHL24	-1.03758	0.000264
KLHL26	-0.72304	0.007427
KLHL29	-1.14045	6.45E-05
KLHL3	-0.87579	0.001591
KLHL30	-1.07269	0.009858
KLHL35	-0.64274	0.026213
KLHL41	-1.26015	0.037158
KLHL7	-0.2589	0.002446
KLHL8	0.496244	0.000446
KLHL9	-0.25657	0.016231
KLK10	0.401462	0.036911
KLK13	1.652744	0.00421
KLK5	-1.38273	0.000241
KLK6	0.590761	9.45E-05
KLK8	-1.55552	0.000421
KLRB1	-2.57113	0.025955
KMO	-1.68558	3.22E-08
KMT2A	-0.23498	0.011539
KMT2C	-0.26157	0.01217
KMT2E	-0.44565	7.65E-05
KNDC1	-1.56124	0.000238
KNSTRN	0.527135	0.00509
KPNA1	0.361778	0.007499
KPNA3	0.309365	0.023228
KPNA4	0.326248	0.014088
KPNA6	0.28618	3.11E-06
KPNA7	1.6683	1.23E-07
KPNB1	0.330506	0.001374
KRBA2	-1.71651	9.73E-06
KRCC1	-0.52229	0.003938
KREMEN1	-1.34123	4.80E-12
KREMEN2	-1.14199	0.012274
KRI1	0.755716	1.52E-08
KRR1	0.832716	4.96E-08
KRT15	1.218662	1.84E-16
KRT16P4	-1.96255	0.011681
KRT16P6	-1.84485	3.83E-06
KRT17	-1.38113	1.62E-07
KRT18	1.16762	5.12E-13
KRT18P11	3.838266	0.021818

KRT19	0.942415	6.97E-10
KRT23	2.197633	0.01409
KRT42P	-1.34626	0.037001
KRT5	-2.33388	1.34E-39
KRT6A	3.547467	4.12E-06
KRT6B	3.620367	6.77E-05
KRT6C	4.942926	4.92E-07
KRT7	0.462628	0.003875
KRT74	-2.57929	0.044868
KRT7-AS	0.715221	0.004273
KRT8	0.952015	6.78E-06
KRT80	-0.50982	0.001894
KRT89P	-1.68299	0.009305
KRT8P3	0.817025	6.80E-05
KRT8P46	-0.95027	0.025405
KRT8P8	1.06264	0.005913
KRTAP1-5	1.411868	0.010812
KRTAP2-3	1.702751	8.46E-05
KRTAP4-12	4.282135	0.001081
KRTAP5-1	-1.93157	0.000128
KRTAP5-AS	-2.35812	9.48E-06
KRTAP7-1	2.938443	0.002685
KRTCAP3	0.656589	7.24E-10
KSR1	-1.93859	2.60E-14
KTI12	0.286894	0.012257
KYNU	1.737136	2.85E-08
L1CAM	-1.05359	0.000927
L2HGDH	0.478992	0.001502
LA16c-13E4	5.438799	6.03E-10
LA16c-380I	-0.47572	0.001073
LA16c-60D	2.057464	3.95E-07
LA16c-60D	3.562002	1.79E-48
LA16c-60H	-1.43864	4.10E-05
LACTB	-0.35008	0.004216
LACTB2	0.491803	0.003534
LAD1	0.509358	0.014809
LAG3	-3.20355	0.003608
LAMA1	0.75344	0.016605
LAMA2	0.604365	0.031153
LAMA3	1.485697	0.000224
LAMA5	-0.91736	0.002515
LAMB1	-0.39007	2.16E-05
LAMB2	-0.97722	2.06E-05
LAMB3	1.232256	4.77E-08
LAMC1	-0.38602	1.02E-05
LAMC2	1.186724	0.000899
LAMC3	-0.88804	0.012171

LAMP1	-0.21823	0.002191
LAMP2	-0.39978	0.001532
LAMTOR5	0.256592	0.007873
LANCL1	-0.31492	0.004252
LAP3	0.406779	8.78E-05
LAPTM4A	-0.65327	2.31E-07
LARGE1	-1.65057	3.39E-05
LARP1	0.224763	0.0403
LARP4	0.57373	0.000237
LARP4B	0.253935	0.005547
LARP7	0.368459	0.024253
LARS	0.31411	0.031927
LASP1	-0.44479	0.004175
LAT2	0.989417	5.18E-06
LAYN	-0.6253	5.54E-06
LBH	-1.67104	2.56E-08
LCA5	-0.81092	0.000115
LCAT	-0.97973	0.001362
LCLAT1	0.282904	0.028278
LCMT2	0.4971	0.012605
LCN2	1.963237	5.92E-24
LCP1	-0.33335	0.003811
LDAH	0.554452	0.000982
LDB1	-0.59153	0.003563
LDB3	-2.67036	1.42E-09
LDHA	0.471378	0.009146
LDHB	0.751844	2.59E-06
LDLR	0.289085	0.004881
LDOC1	-0.36535	0.011651
LDOC1L	-0.70483	0.000222
LEO1	0.388471	0.000397
LETM1	0.549096	3.48E-05
LETM2	0.448085	0.006057
LETMD1	0.37202	0.004905
LFNG	-1.65033	1.17E-07
LGALS1	-0.95425	6.04E-11
LGALS14	-2.76464	0.000151
LGALS2	1.40668	0.021532
LGALS3	0.439121	9.58E-06
LGALS3BP	-1.20423	2.11E-05
LGALS8	0.334652	0.018943
LGALS9B	1.722912	0.00117
LGR5	-2.26778	0.004255
LHB	-1.32679	0.043832
LHFP	-2.33844	2.36E-40
LHFPL2	0.381829	2.52E-10
LHPP	-1.44044	6.08E-07

LHX9	-0.66391	0.04226
LIAS	0.492264	0.000523
LIF	1.270985	6.79E-13
LIFR	-0.48209	0.00979
LIG3	0.204451	0.006715
LIG4	0.655853	0.001061
LILRB5	0.999121	0.014084
LIMD2	-2.08851	2.47E-05
LIMK1	-0.49289	0.030357
LIMK2	-1.07515	3.79E-09
LIMS1	-0.29306	0.031867
LIMS2	-3.03426	4.12E-23
LIN7B	-0.57016	0.002937
LIN7C	0.4045	0.005006
LIN9	0.465583	0.002169
LINC00094	-0.67627	1.91E-06
LINC00205	-0.55032	0.000803
LINC00265	-1.18166	1.33E-14
LINC00271	-1.18018	0.001009
LINC00294	-0.563	2.78E-06
LINC00313	-1.57111	7.11E-05
LINC00314	1.236751	0.013568
LINC00319	-1.26026	0.00347
LINC00326	3.219629	0.02682
LINC00339	0.38096	0.045548
LINC00342	-0.46441	0.01638
LINC00346	-0.62496	0.003726
LINC00365	-1.29058	0.008126
LINC00410	2.272134	0.04653
LINC00452	2.697835	5.12E-08
LINC00460	1.355701	1.09E-05
LINC00475	-1.91214	0.003735
LINC00493	0.609974	1.09E-05
LINC00511	-0.57126	8.43E-06
LINC00513	0.993272	0.000224
LINC00525	-1.2912	0.01265
LINC00536	-1.54903	0.049779
LINC00571	-0.95913	0.006827
LINC00589	2.084277	0.01538
LINC00607	-3.57049	1.93E-08
LINC00623	-0.40415	0.034038
LINC00624	0.788205	0.001608
LINC00626	-3.55234	0.00643
LINC00630	-0.29392	0.039515
LINC00638	-1.39608	0.003697
LINC00640	-1.54407	0.015268
LINC00643	-1.99701	0.0125

LINC00649	-0.34409	0.035843
LINC00662	-0.50911	0.018216
LINC00665	0.382684	0.004797
LINC00667	-0.40534	0.002464
LINC00672	-0.74371	0.003937
LINC00689	-1.40931	0.037931
LINC00702	-1.61941	2.68E-06
LINC00707	1.997251	5.34E-07
LINC00852	0.715472	0.005669
LINC00857	0.485459	0.003137
LINC00863	-0.74393	9.15E-07
LINC00869	-0.78418	3.31E-05
LINC00885	-4.25205	2.26E-23
LINC00893	-0.85615	0.002895
LINC00941	0.943394	0.000742
LINC00942	-1.05305	0.002398
LINC00958	0.625035	0.028156
LINC00960	-0.5622	9.13E-06
LINC00963	-0.61362	0.000194
LINC00973	2.116563	2.12E-08
LINC01013	1.875374	0.020929
LINC01106	1.221959	1.35E-08
LINC01123	0.61357	0.00305
LINC01124	-0.94351	0.038747
LINC01125	-0.40235	0.014206
LINC01127	2.324566	3.39E-08
LINC01128	0.418662	0.002964
LINC01137	-0.64273	0.01924
LINC01165	-2.39061	3.04E-06
LINC01204	1.755179	0.001106
LINC01213	-2.64094	0.0007
LINC01232	-0.53662	0.003173
LINC01234	0.288093	0.010702
LINC01239	-1.91943	3.02E-05
LINC01269	1.047691	0.030779
LINC01278	-0.60143	3.02E-05
LINC01287	-1.47376	4.26E-05
LINC01291	-0.64598	0.010568
LINC01297	0.775641	0.032285
LINC01305	-3.29557	0.00384
LINC01312	1.856193	0.011214
LINC01336	-2.89285	2.91E-10
LINC01348	0.882133	0.014954
LINC01381	-2.28956	0.041917
LINC01389	-1.61769	0.002863
LINC01391	-1.28982	0.000537
LINC01419	1.6577	0.021195

LINC01422	-1.28621	3.90E-05
LINC01451	-2.35756	0.003575
LINC01456	-2.22235	0.000394
LINC01460	-1.49071	0.000233
LINC01468	0.730069	0.04818
LINC01503	-0.99649	7.00E-06
LINC01511	1.281185	0.046299
LINC01515	-0.66137	0.009855
LINC01521	-0.75052	0.002768
LINC01534	0.639269	0.032329
LINC01559	2.23073	0.001244
LINC01568	1.364007	0.023981
LINC01583	-2.282	5.79E-11
LINC01605	0.499358	0.01328
LINC01619	1.512149	0.0012
LINC01629	-0.91476	0.000181
LINC01633	-1.7645	0.029016
LINC01638	-3.91784	8.75E-11
LINC01659	3.660674	0.012795
LINC01669	-1.92673	0.009389
LINC01711	-1.32763	0.000228
LINC01714	2.246673	0.020982
LINC01719	-0.4063	0.035965
LINC01748	-0.56382	0.000175
LINC01764	-0.79888	0.017589
LINC01778	-3.14101	0.022958
LINC01806	0.470497	0.03567
LINC01827	-2.75411	0.005122
LINC01836	-0.88942	0.003942
LINC01873	-0.29889	0.038592
LINC01876	-0.70177	0.000557
LINC01947	3.008457	0.039367
LINC01963	-0.37791	0.001647
LINC01980	-1.02866	6.93E-07
LINC01983	0.441464	0.019109
LINC02001	0.369778	0.007654
LINC02020	1.97022	0.029033
LINC02028	-3.06269	4.36E-08
LINC02035	-0.41401	0.039688
LINC02041	-0.86403	0.045506
LINC02065	2.258903	0.019351
LINC02068	1.529773	0.003302
LINC02132	2.988673	0.002897
LINC02158	2.619727	0.000399
LINC02178	1.966916	0.00142
LINC02204	1.51366	0.000804
LINC-PINT	0.56098	0.025932

LINP1	0.655215	0.000401
LINS1	0.387758	0.014899
LIPC	-1.81996	0.005642
LIPG	0.924276	6.55E-07
LIPH	1.851938	1.34E-16
LIPK	3.182299	3.06E-12
LIPM	1.299883	0.001397
LIPT1	0.587862	0.000256
LIPT2	-0.78368	0.000209
LIX1L	-0.78922	0.004037
LIX1L-AS1	1.042052	0.017767
LL21NC02-[illegible]	-2.58715	4.66E-05
LL22NC03-[illegible]	0.24356	0.04202
LL22NC03-[illegible]	-0.81816	0.001121
LLGL1	-0.43105	0.027682
LLNLF-96A[illegible]	0.314651	0.003459
LLPH	0.515128	0.00756
LMAN2L	-0.67205	2.70E-11
LMCD1	-2.56271	2.72E-14
LMNTD1	-1.4836	0.001673
LMO2	1.386582	7.09E-07
LMO4	-0.47056	0.01273
LMO7	0.835877	1.29E-06
LMO7-AS1	-2.36272	9.57E-08
LMOD1	-3.99578	4.39E-06
LMTK2	0.243354	0.002921
LNP1	-0.56916	0.032373
LNPEP	0.344639	0.01949
LNX2	-0.22944	0.030689
LONRF3	1.422353	6.15E-13
LOX	-1.41799	2.04E-09
LOXL1-AS1	-0.35996	0.011664
LOXL2	-2.50918	6.08E-15
LPAR1	-1.18499	7.34E-06
LPAR2	-0.43845	0.03789
LPAR5	-0.69184	0.009894
LPAR6	1.082739	0.000149
LPCAT1	-0.8317	1.07E-07
LPCAT2	-0.83995	6.45E-12
LPCAT4	1.210678	1.45E-09
LPGAT1	-0.22348	0.014719
LPIN1	0.417426	0.002035
LPP	-0.64658	2.91E-05
LPP-AS2	-0.95796	0.000445
LPXN	0.999655	2.35E-07
LRAT	2.965624	3.25E-33
LRGUK	-1.00856	0.025754

LRIF1	0.451722	0.007783
LRIG2	-0.23028	0.034838
LRIG3	-0.68152	9.82E-07
LRP1	-1.59829	1.49E-07
LRP11	0.499293	6.32E-05
LRP12	-0.2324	0.02712
LRP3	-0.61981	0.045576
LRP4	-1.11588	5.66E-11
LRP8	0.454484	0.000758
LRPPRC	0.675275	3.79E-05
LRR1	0.512774	0.003662
LRRC23	-1.08776	6.30E-06
LRRC27	-0.69166	0.000698
LRRC28	-0.48857	0.000358
LRRC3	-1.74554	5.03E-09
LRRC37A15	-0.69519	0.038276
LRRC37A16	-0.54967	0.000196
LRRC37A17	-0.25946	0.035589
LRRC37A4F	-0.34003	0.003488
LRRC37B	-0.52636	8.99E-05
LRRC37BP1	-0.49152	5.74E-07
LRRC4	1.00825	0.00057
LRRC56	-1.1523	0.011282
LRRC58	0.39792	0.003497
LRRC6	-0.98668	6.29E-06
LRRC61	-0.40423	0.042688
LRRC7	-1.40997	9.16E-10
LRRC73	-1.33705	0.026642
LRRC75A-A	0.50275	8.16E-05
LRRC75B	-1.63449	1.28E-06
LRRC8B	0.642027	3.03E-05
LRRC8C	1.285828	1.24E-06
LRRFIP1	0.504063	8.13E-05
LRRIQ1	-1.1333	1.00E-07
LRRN2	-2.08731	2.71E-07
LRRN4	1.771861	0.004796
LRRTM3	-2.76663	0.010591
LRSAM1	-0.53964	5.24E-06
LRTOMT	-0.31154	0.022638
LSAMP	1.209469	7.29E-05
LSG1	0.399648	0.000142
LSM10	0.397425	0.002662
LSM12	0.401173	0.000113
LSM12P1	0.433955	0.007646
LSM14B	-0.25981	0.006307
LSM3	0.392281	0.010643
LSM5	0.455753	0.007572

LSM6	0.389366	0.005784
LSS	-0.99256	3.83E-06
LTA	-2.34771	0.0029
LTA4H	0.933896	9.81E-11
LTB	-3.08459	1.48E-15
LTBP1	-0.90528	0.00025
LTBP2	-1.3712	3.76E-05
LTBP3	-0.84052	0.026339
LTBP4	-1.14425	0.001858
LTC4S	-2.6913	0.037672
LTN1	0.39319	0.004713
LTV1	0.691415	3.04E-06
LUC7L	0.173295	0.029608
LUC7L3	0.372152	0.007756
LUCAT1	1.249625	1.59E-05
LURAP1L-A	1.437445	0.001831
LUZP1	-0.56526	3.50E-07
LUZP2	-1.34172	0.015524
LY6G6C	0.822074	0.033323
LY6K	1.950312	2.60E-18
LY75-CD30	-1.4582	0.012762
LYAR	1.233438	1.34E-09
LYNX1	-1.57561	8.88E-05
LYPD1	-0.83777	7.28E-06
LYPD3	0.847285	0.001541
LYPD4	3.455241	0.04543
LYPD6	-0.71268	2.37E-05
LYPLA1	1.001576	5.05E-08
LYRM1	0.759278	6.27E-06
LYRM4	0.193565	0.047821
LYRM7	0.803816	1.43E-05
LYSMD2	0.835789	2.17E-06
LZIC	0.303018	0.029944
LZTS1	-1.49442	0.005339
LZTS3	-1.59234	3.71E-05
M6PR	0.183505	0.000166
MACC1	2.017761	0.000403
MACC1-AS:	2.36252	0.000944
MACROD2	-0.5348	0.038115
MAD2L1	0.580565	0.0007
MAD2L1BP	0.336102	0.001139
MAF	-2.69054	2.29E-06
MAFB	-2.16069	1.86E-07
MAFF	1.019454	1.92E-07
MAGEA12	0.340854	0.004213
MAGED1	-1.35539	2.95E-14
MAGED2	-0.96013	1.25E-10

MAGED4	-1.79106	2.46E-06
MAGED4B	-2.17469	1.22E-07
MAGEF1	0.279213	0.03916
MAGI1-IT1	0.711057	0.030831
MAGI2-AS3	1.139712	0.007996
MAGI3	-0.61219	1.84E-09
MAGOH	0.364364	0.005328
MAGOHB	0.404824	0.010959
MAIP1	0.346581	0.004691
MAK16	0.824272	4.12E-09
MAL	0.669203	0.006795
MAL2	1.369567	1.06E-07
MALL	1.418003	4.28E-08
MALSU1	0.335501	0.005451
MALT1	0.241824	0.043891
MAMDC2	-3.23006	1.43E-10
MAML1	-0.30388	0.031192
MAML2	-1.27378	1.09E-18
MAN1A1	-0.72632	0.006486
MAN1B1	-0.38667	0.022758
MAN2A1	0.316129	0.049182
MAN2B1	-0.40331	0.005832
MAN2B2	-0.79037	3.71E-07
MANBA	-0.89876	1.83E-06
MANEA-AS	-1.12994	0.003908
MANSC1	1.206249	6.56E-09
MAOA	1.416367	6.51E-12
MAOB	1.818205	4.28E-05
MAP1A	-0.93395	0.035841
MAP1B	-1.74114	3.32E-14
MAP2	-0.96101	2.33E-05
MAP2K1	0.508484	9.22E-06
MAP2K3	0.760194	2.16E-07
MAP2K5	-0.32588	0.008595
MAP3K1	-0.44329	0.031174
MAP3K13	0.535421	0.002841
MAP3K2	-0.20315	0.045953
MAP3K20	0.254245	0.020275
MAP3K20-A	0.967635	1.64E-05
MAP3K21	0.587206	5.12E-07
MAP3K3	-0.52392	0.003911
MAP3K4	-0.20351	0.00093
MAP3K5	0.526499	0.0001
MAP3K7	0.236557	0.019669
MAP3K7CL	-1.08931	0.000105
MAP3K8	0.503825	0.011639
MAP4	-0.55355	7.49E-09

MAP4K3	0.336711	0.009196
MAP6	-1.27295	0.04818
MAP7	0.259337	0.012893
MAPK10	-0.8681	0.012367
MAPK11	-0.77312	0.009197
MAPK13	0.872705	5.15E-07
MAPK14	-0.39385	7.37E-11
MAPK1IP1I	0.325927	0.002165
MAPK3	-0.32331	0.010109
MAPK4	-4.59326	4.12E-08
MAPK7	-0.78471	0.001076
MAPK8IP1	-0.67787	0.002747
MAPKAPK2	-0.32165	0.008788
MAPKAPK3	0.447911	0.005736
MAPKAPK5	0.139469	0.039476
MAPRE3	-0.69312	9.87E-06
MAPT	-1.09751	0.005312
1-Mar	0.55955	1.31E-05
1-Mar	-0.4391	0.029872
2-Mar	-0.60508	0.000903
3-Mar	0.594459	0.040687
8-Mar	-0.34273	0.005169
MARCKS	-1.13269	6.44E-10
MARCKSL1	-0.74276	0.012553
MARCO	2.473513	0.005021
MARK1	-1.09834	8.05E-10
MARK3	0.216096	0.005258
MARK4	-0.57689	0.01639
MARS2	0.610179	1.72E-06
MARVELD1	-0.87672	2.94E-05
MARVELD2	0.337136	0.003824
MAST1	-0.85449	0.003888
MAST2	-0.46248	0.002894
MAST4	1.975383	2.12E-20
MAST4-AS1	1.032374	0.020747
MAT2A	0.429289	0.03139
MATK	0.80343	0.013305
MATN2	-3.18562	3.33E-31
MATN3	-2.12102	2.04E-25
MATR3	0.270898	0.045089
MAX	0.179803	0.008965
MAZ	-0.85385	0.000851
MB21D1	0.74106	2.42E-05
MBLAC2	0.373418	0.004131
MBNL1-AS1	-1.8334	2.06E-07
MBNL2	-1.04205	0.000115
MBOAT1	0.514879	0.005574

MBOAT2	0.67765	2.64E-05
MBP	0.229935	0.025315
MBTPS1	-0.25102	0.005619
MBTPS2	0.290101	0.029596
MCAM	-2.58665	2.74E-12
MCAT	0.403613	0.003887
MCC	-0.84876	7.58E-09
MCCC2	0.35982	0.009766
MCF2L2	-0.38474	0.013902
MCFD2	0.597861	2.05E-09
MCIDAS	1.277984	1.10E-08
MCL1	0.337153	0.001007
MCM10	0.559112	0.000969
MCM2	0.235122	0.025167
MCM3	0.237791	0.042121
MCM4	0.343738	0.024557
MCM7	0.234546	0.008848
MCM8	0.377673	0.011295
MCMBP	0.363386	0.00478
MCOLN2	0.599453	0.003229
MCOLN3	-0.37973	0.000418
MCPH1	0.39187	0.003468
MCPH1-AS	2.936682	1.75E-14
MCTP1	2.007273	1.92E-29
MCTS1	0.361275	0.021277
MCUB	0.326409	0.010999
MDFIC	-0.32369	0.008998
MDGA1	-1.32231	2.31E-06
MDH2	0.351333	0.000182
MDK	-1.43059	0.000102
MDM4	0.376121	0.009913
MDN1	0.548893	0.001625
ME1	-0.40128	0.001095
ME2	0.625502	0.000496
MECOM	0.654888	5.81E-05
MECP2	-0.3431	0.016853
MED1	0.191132	0.011837
MED10	0.382017	9.47E-06
MED12	-0.28164	0.01687
MED12L	-0.67984	0.001445
MED13L	-0.51356	4.19E-07
MED18	0.513314	0.001182
MED28P3	1.780262	0.0431
MED29	-0.38959	0.0166
MED31	0.680859	0.00171
MED6	0.40645	0.017445
MEF2C	-1.79291	1.62E-07

MEGF6	-2.44762	7.41E-09
MEGF8	-0.76709	0.026601
MEGF9	-0.89191	8.31E-11
MEIS1	-0.76885	2.79E-07
MEIS3	-2.75561	3.56E-09
MEOX1	-3.30254	0.003226
MEST	-1.62878	1.84E-07
MET	-0.31451	0.012535
METAP1	0.473863	4.69E-05
METAP2	0.750095	8.48E-07
METTL1	0.435307	0.003695
METTL12	0.365246	0.024872
METTL13	0.471043	0.005092
METTL16	0.253412	0.017941
METTL18	0.638868	0.003957
METTL21A	0.577435	7.27E-06
METTL22	-0.26109	0.019222
METTL2A	0.530213	0.002952
METTL2B	0.394672	0.000293
METTL5	0.345043	0.022557
METTL7A	-1.34782	0.048879
METTL8	0.387636	0.029261
MEX3A	-1.1173	5.10E-06
MEX3B	-3.03562	2.01E-18
MEX3C	-0.39631	4.42E-06
MEX3D	-1.35383	1.55E-08
MFAP1	0.407175	0.003321
MFAP2	-2.07116	5.04E-08
MFAP3L	-0.51621	0.010944
MFGE8	-1.11006	2.99E-06
MFHAS1	0.312351	0.000265
MFN2	0.233123	0.005934
MFSD2A	1.376437	3.55E-20
MFSD6	-0.22247	0.02906
MGA	0.226796	0.010332
MGAM	-2.42976	3.47E-15
MGAT2	-0.65706	0.043498
MGAT4B	-0.30906	0.040879
MGAT5B	-1.11615	2.18E-05
MGC32805	0.996732	0.000136
MGP	-0.89794	0.015366
MGST3	-0.27981	0.00041
MIA	1.257763	0.027128
MIAT	-1.28418	0.000212
MIATNB	-0.52269	0.000196
MIB1	-0.35875	0.006968
MICA	-0.41601	0.000277

MICAL1	-0.61059	0.04637
MICAL2	-0.69391	8.17E-06
MICAL3	-0.60306	0.003322
MICALCL	-0.8606	1.93E-08
MICALL1	-0.82681	0.000354
MICALL2	-1.06275	0.00046
MICB	-0.44185	0.00371
MICU1	-0.29961	0.008084
MICU2	0.458548	0.000782
MID1	-0.9161	2.97E-21
MID1IP1	0.437694	0.004293
MIEF1	0.235565	0.008556
MIGA1	0.27517	0.02631
MINDY1	-0.68971	0.000164
MINDY3	0.394005	0.001213
MINOS1	0.426616	0.019271
MIR1206	1.353239	0.010083
MIR1249	-2.58637	0.038194
MIR1273D	-2.75847	0.038123
MIR137HG	-0.72667	0.045033
MIR17HG	0.724967	0.001504
MIR181A2I	-0.67194	0.0031
MIR193BH(	-0.87234	5.89E-05
MIR205HG	-0.73982	0.00012
MIR222HG	1.19904	3.44E-06
MIR27B	-1.95307	0.001715
MIR29A	4.760968	0.000495
MIR3651	2.236955	0.030337
MIR4260	1.713339	0.011928
MIR4435-2	0.427886	0.00046
MIR4668	1.768317	0.013715
MIR4737	-0.74182	0.038801
MIR548D1	0.693438	0.039006
MIR6819	-3.06831	0.027446
MIR8485	2.781287	0.013064
MIS18A	0.294861	0.049628
MIS18BP1	0.471785	0.006336
MKKS	0.504825	4.40E-05
MKL1	-1.34466	7.36E-08
MKL2	-0.54685	3.41E-08
MLH3	0.395653	0.00202
MLKL	1.746659	4.74E-13
MLLT10	-0.22693	0.035306
MLLT3	-0.3814	0.049667
MLPH	0.493131	0.000435
MLX	0.628123	9.28E-11
MLXIPL	-0.62655	0.047282

MMAB	-0.46234	0.004386
MMACHC	0.516927	9.89E-06
MME	0.532006	0.039428
MMGT1	0.348772	0.008547
MMP1	7.025326	2.73E-14
MMP10	3.13391	1.24E-08
MMP11	-2.17546	0.000172
MMP12	0.974512	0.024193
MMP13	-1.44811	9.44E-06
MMP14	-2.13147	4.35E-05
MMP2	-2.45664	1.10E-19
MMP20	2.18953	0.000111
MMP24	-3.17007	4.96E-06
MMP25-AS	-1.24896	1.02E-10
MMP27	2.843008	0.006538
MMP3	1.868609	6.10E-07
MMP8	2.316873	7.86E-07
MMP9	1.045675	0.023066
MMRN2	-1.13501	4.62E-05
MN1	-2.18376	1.01E-08
MND1	0.705586	0.002106
MNS1	0.501036	0.03953
MOAP1	0.494601	0.001418
MOB3A	-0.41402	0.003712
MOB3B	-0.97661	2.69E-08
MOB3C	0.452313	0.004234
MOB4	0.527645	0.00505
MOCS2	0.461847	0.004125
MOGAT3	0.881041	0.048439
MON1A	0.962476	1.30E-05
MORF4L1	-0.25468	0.003186
MORF4L1P	-1.83284	0.018959
MORN3	-1.23668	5.51E-06
MORN4	-0.61046	0.000106
MOSPD3	-0.8922	0.004856
MOV10	-0.36362	0.013691
MOV10L1	-2.04124	0.001993
MOXD1	-2.02086	6.53E-07
MPHOSPH1	0.394721	0.005254
MPHOSPH6	0.776367	2.94E-05
MPIG6B	0.837412	0.034695
MPL	-1.32444	0.043175
MPLKIP	0.358034	0.000588
MPP6	0.945596	1.28E-07
MPPE1	0.403396	0.019722
MPRIP	0.434397	0.000828
MPV17L	0.597245	1.36E-05

MPZL1	-0.4906	2.33E-07
MPZL2	0.553854	0.011669
MPZL3	0.361863	0.011781
MR1	-0.47178	0.000261
MRAS	-2.48888	7.07E-16
MRC2	-2.73475	3.31E-06
MREG	0.946225	1.10E-11
MRGBP	0.167217	0.041104
MRM2	0.456826	2.60E-07
MRM3	0.558839	0.001226
MROH2A	-2.92787	0.000737
MRPL1	0.604717	7.09E-05
MRPL10	0.437271	1.30E-05
MRPL11	0.309703	0.002083
MRPL12	0.602541	0.005285
MRPL13	0.493307	0.002457
MRPL15	0.745893	3.28E-06
MRPL16	0.773117	1.30E-09
MRPL17	0.246806	0.001724
MRPL18	0.331559	0.024017
MRPL19	0.502179	0.000314
MRPL20	0.482523	5.72E-05
MRPL21	0.519008	3.82E-07
MRPL22	0.244436	0.040089
MRPL24	0.343124	0.014925
MRPL27	0.210442	0.033042
MRPL3	0.560668	0.000205
MRPL30	0.591381	0.000159
MRPL32	0.619748	6.11E-09
MRPL35	0.321911	0.030787
MRPL36	0.76788	4.94E-09
MRPL37	0.260314	0.00034
MRPL39	0.628976	2.96E-05
MRPL40	0.246492	0.040818
MRPL42	0.578105	0.00035
MRPL44	0.240005	0.042194
MRPL45	0.493993	7.57E-05
MRPL46	0.586418	4.29E-06
MRPL47	0.395538	0.009554
MRPL50	0.757305	0.001585
MRPL52	-0.3672	0.017369
MRPL57	0.48042	0.001103
MRPL58	0.495889	5.05E-05
MRPL9	0.215442	0.001586
MRPS10	0.407408	0.012448
MRPS11	0.316152	0.000253
MRPS12	0.499299	0.047727

MRPS15	0.450012	2.77E-06
MRPS16	0.201948	0.030193
MRPS17	0.958879	2.49E-07
MRPS18B	0.360844	0.000721
MRPS18C	0.536263	0.001608
MRPS2	0.556328	3.62E-07
MRPS21	0.338527	0.00072
MRPS22	0.37186	0.004196
MRPS23	0.577266	1.12E-05
MRPS25	0.56537	0.000311
MRPS27	0.378219	0.002041
MRPS28	0.407149	0.02478
MRPS30	0.581355	9.81E-06
MRPS31	0.55989	0.001503
MRPS33	0.495353	0.007083
MRPS35	0.576994	0.000237
MRPS36	0.335821	0.027668
MRPS5	0.395494	0.000112
MRPS6	0.709932	1.34E-06
MRPS9	0.284999	0.022258
MRRF	0.575423	0.000185
MRS2	0.495324	0.000173
MRTO4	0.630519	0.000341
MSANTD4	0.244968	0.047481
MSC	-2.82514	1.01E-15
MSC-AS1	-2.3598	6.18E-07
MSH2	0.398066	0.010667
MSH6	0.296112	0.017931
MSI1	-1.63685	0.000117
MSMO1	-0.32581	0.047126
MSRA	0.563768	0.012055
MSRB2	-0.47321	0.000186
MSRB3	-2.52065	1.20E-36
MST1R	1.42498	2.84E-06
MSX1	0.60619	0.000572
MSX2	-0.37169	0.027981
MT1F	-0.88843	4.13E-05
MT1X	-0.39793	0.018873
MTCL1	-1.05501	2.02E-11
MTDH	0.268488	0.043948
MTERF1	0.378103	0.038654
MTERF3	0.638266	0.000676
MTERF4	0.252949	0.028298
MTF1	0.185172	0.036108
MTF2	0.231738	0.049238
MTFMT	0.287178	0.011129
MTFR2	0.568085	0.018706

MTHFD1	0.242252	0.032678
MTHFD2L	0.880448	1.57E-07
MTIF2	0.617264	0.000859
MTMR1	0.254057	0.005627
MTMR10	-0.43054	0.000857
MTMR11	-0.69882	8.35E-05
MTMR3	-1.03998	8.83E-09
MTMR4	-0.23277	0.022425
MTMR9LP	-1.37622	2.28E-05
MTND2P28	0.479647	0.007739
MTO1	0.280904	0.022966
MTPAP	0.430507	0.004264
MTPN	0.209826	0.041704
MTRF1L	0.44796	0.000443
MTRNR2L3	1.720148	0.034521
MTRR	0.596804	1.52E-05
MTSS1	-1.22535	5.66E-13
MTSS1L	-1.1004	0.000146
MT-TL1	0.513369	0.046059
MT-TQ	0.651583	0.0108
MT-TT	0.994418	0.030916
MT-TW	1.668339	0.021608
MTURN	0.439989	0.026752
MTUS1	-0.81304	9.54E-05
MTUS2	-1.24696	0.020676
MTX2	0.342839	0.000101
MTX3	0.320731	0.014452
MUC16	1.038519	0.002614
MUC20	1.644046	2.77E-16
MUC20P1	1.731893	4.71E-15
MUC4	-0.97727	0.000128
MUC5AC	2.830858	0.000205
MUS81	-0.44523	0.024152
MVB12B	-0.85501	0.000102
MVD	-0.78756	0.000845
MVK	-0.67684	4.79E-05
MX1	-1.03121	0.009188
MXD1	1.211901	9.77E-22
MXD3	-1.20728	0.00035
MXD4	-1.04776	0.000376
MXI1	0.640606	0.000114
MXRA5	-2.87011	0.002037
MXRA7	-0.34894	0.00045
MYADM	-0.4708	1.00E-06
MYBBP1A	0.561512	0.003372
MYBL1	-0.95375	2.42E-06
MYBL2	-0.55975	0.000847

MYC	1.166744	6.46E-12
MYCBP	0.529463	0.003263
MYCL	-1.22773	4.79E-07
MYD88	0.527784	0.000787
MYEOV	1.876358	3.97E-51
MYH10	-0.3818	3.19E-10
MYH15	1.423955	0.000311
MYH16	2.28074	1.96E-09
MYH9	-1.14489	1.73E-14
MYL12A	-0.35671	0.001351
MYL6	-0.83679	1.22E-07
MYL7	-8.20615	1.93E-16
MYL9	-4.05903	2.16E-36
MYLK	-1.90217	2.60E-23
MYO16-AS	-1.63955	0.010886
MYO18A	0.442384	0.000506
MYO1B	-0.57903	6.89E-05
MYO1D	-0.70366	3.30E-06
MYO3B	-2.14137	5.85E-13
MYO5A	-0.5833	2.84E-06
MYO5B	1.119461	2.75E-10
MYO5C	0.400164	0.039572
MYO6	0.418221	0.015768
MYO7B	-1.57109	0.010911
MYO9A	-0.22532	0.043534
MYO9B	-0.46458	0.00491
MYOCD	-5.68544	6.48E-15
MYOM1	-1.26987	0.024019
MYOZ1	-4.57269	2.57E-13
MYSM1	0.31843	0.008366
MZT1	0.646421	0.000658
N4BP2L1	-0.87939	0.026
N4BP3	-0.98097	0.009663
N6AMT1	0.632515	8.46E-05
NAA10	0.311302	0.014981
NAA15	0.740192	0.000155
NAA16	0.277865	0.025192
NAA20	0.4463	0.000822
NAA25	0.556307	0.000137
NAA35	0.329855	0.014083
NAA50	0.561489	0.00037
NAA60	-0.3374	0.042231
NAB1	0.747519	3.15E-08
NAB2	0.682376	0.024802
NABP1	0.761874	0.002595
NACA	0.466549	0.000708
NACAD	-3.14218	2.98E-13

NACC2	-0.56201	0.011954
NAE1	0.507178	0.001648
NAF1	0.688168	1.43E-06
NAGK	-0.44619	0.028891
NAGLU	-0.61374	0.029435
NAIP	-0.82579	1.01E-07
NAMPT	0.520917	0.003757
NANOS1	-0.84823	7.19E-09
NANP	0.406579	0.005843
NAP1L1P2	3.684906	0.008984
NAP1L3	-1.59131	0.032597
NAP1L4	0.185656	0.002046
NAP1L4P1	0.732689	0.016025
NAPA	-0.349	0.018084
NAPG	0.441794	0.002626
NARS	0.407326	0.006317
NAT1	0.476856	0.022599
NAT10	0.706467	3.93E-07
NATD1	-1.21522	0.000229
NAV1	-0.83836	3.31E-05
NAV2	-2.18249	3.49E-22
NAV2-AS1	-2.08178	1.73E-06
NAV3	1.853332	1.31E-10
NBAT1	-1.98613	1.15E-08
NBEA	-0.44008	0.00691
NBEAL2	0.765214	0.003644
NBEAP3	-0.63937	0.012952
NBPF1	-0.52314	7.57E-06
NBPF11	-0.34242	0.001072
NBPF12	-0.43176	5.47E-07
NBPF15	-0.26196	0.025519
NBPF25P	-0.58955	0.006442
NBPF3	-0.44759	0.00042
NBPF8	0.560832	0.000333
NCALD	-1.03271	0.013926
NCAPD3	0.418186	0.0004
NCAPH	0.267669	0.022099
NCBP1	0.41339	0.018352
NCBP2	0.518928	9.19E-08
NCBP2-AS2	0.40605	0.000837
NCEH1	1.172483	1.55E-06
NCF2	-1.19453	6.54E-09
NCF4	1.780441	0.000217
NCK2	-0.71713	7.24E-07
NCKAP5L	-1.10926	0.007175
NCL	0.79161	8.61E-07
NCOA1	-0.60653	3.68E-09

NCOA2	-0.21518	0.044595
NCOA3	-0.25205	0.023602
NCOA6	-0.21959	0.004016
NCOR1	-0.26339	0.00288
NCOR2	-0.58995	0.026817
NCS1	-0.70119	0.00089
NCSTN	-0.246	0.001015
NDC1	0.662782	0.000721
NDE1	-0.45143	0.000133
NDN	-0.94737	3.47E-06
NDRG2	-0.4454	0.034215
NDST1	-1.67965	2.33E-12
NDUFA1	0.282174	0.008186
NDUFA10	0.223244	0.023763
NDUFA12	0.361581	0.005282
NDUFA4L2	-1.29259	0.003096
NDUFA7	0.293871	0.002508
NDUFA8	0.244192	0.012678
NDUFAB1	0.560391	6.24E-05
NDUFAF1	0.589365	0.000621
NDUFAF2	0.697104	3.84E-06
NDUFAF4	0.864518	1.23E-06
NDUFAF7	0.292597	0.039551
NDUFAF8	0.558258	0.001211
NDUFB1	0.384165	0.01601
NDUFB2	0.333713	0.002717
NDUFB3	0.452639	0.001175
NDUFB5	0.367803	0.006579
NDUFB9	0.338696	0.000377
NDUFC1	0.413971	0.013561
NDUFS1	0.250943	0.016705
NDUFS3	0.308744	0.000604
NDUFV2	0.488748	0.000332
NDUFV3	0.432043	4.37E-06
NEAT1	0.390397	0.025556
NEB	0.467582	0.046335
NEBL	-1.11082	0.00048
NECAP1	0.37156	1.65E-06
NECTIN4	1.207222	2.71E-13
NEDD4L	0.750801	6.42E-06
NEDD8	0.243872	0.009127
NEDD9	-2.05962	4.08E-13
NEK11	-0.4519	0.004322
NEK3	0.460851	0.000442
NEK4	0.306991	0.014818
NEK6	-1.15069	2.80E-09
NEK7	0.520536	0.007647

NELFCD	0.30342	0.000661
NELL2	-0.90307	0.013947
NEMF	0.361635	0.027076
NEMP2	0.380902	0.016179
NEO1	-1.01313	4.56E-05
NEPRO	0.260666	0.045148
NES	-1.75226	4.31E-06
NET1	0.711753	9.02E-15
NETO2	0.457168	0.000421
NEU2	4.394941	0.012729
NEURL1	0.418778	0.02352
NEURL1B	-1.90043	1.11E-15
NEURL2	-1.33609	0.01057
NEURL3	-2.84619	3.04E-05
NEXN	-2.68006	4.30E-46
NF2	-0.32006	0.004613
NFAM1	-2.02242	0.016319
NFATC2	1.696615	4.75E-07
NFE2L1	-0.41501	0.013252
NFIB	-1.085	2.47E-14
NFIX	-0.76122	0.002548
NFKBIZ	0.915528	6.87E-06
NFS1	0.298252	0.00635
NFX1	0.16938	0.037946
NFXL1	0.531669	0.000211
NGDN	0.386299	0.001623
NGEF	0.826169	0.000115
NGF	-3.65533	1.46E-15
NGFR	-1.98798	0.000376
NGLY1	0.367402	0.026233
NHLRC1	-0.4639	0.018362
NHP2	0.37692	0.001247
NHSL1	-0.49831	1.71E-05
NID1	-0.87683	1.05E-05
NIF3L1	0.31042	0.046694
NIFK	0.684746	0.000266
NINJ1	-1.02052	5.27E-05
NINL	-0.90943	6.11E-06
NIP7	0.770418	6.98E-07
NIPA1	0.254473	0.028413
NIPA2	0.473326	0.000115
NIPAL1	0.753768	7.88E-06
NIPAL3	-0.28378	0.000505
NIPSNAP1	-0.62251	0.001121
NIPSNAP3A	-0.43394	0.009496
NISCH	-0.69546	0.000246
NIT1	0.250439	0.025637

NKAIN1	-1.65563	2.72E-05
NKAIN4	-3.44965	5.51E-14
NKD2	2.263728	3.50E-09
NKILA	-0.67994	0.001381
NKIRAS1	0.42289	0.007151
NKIRAS2	-0.41791	0.023697
NKRF	0.609801	0.000232
NKX1-2	-0.71928	6.41E-15
NKX3-1	-1.05636	1.26E-15
NKX3-2	-2.29077	0.002263
NKX6-1	-0.76276	0.007024
NLE1	0.559534	5.85E-07
NLGN2	-1.03715	0.000329
NLRC5	0.449249	0.003714
NLRP1	-1.54593	1.20E-10
NLRP10	2.008883	0.000109
NLRP2	-2.11677	1.88E-06
NLRP3	0.685713	0.036466
NLRP7	-1.82123	1.19E-18
NLRX1	-0.70081	0.014086
NMD3	0.649876	1.37E-05
NME1	0.60847	1.31E-06
NME2	0.291692	0.006222
NMNAT1	-0.26953	0.021752
NMNAT2	1.598899	1.07E-06
NMNAT3	-1.13473	0.020515
NNMT	-1.60182	7.07E-05
NNT	-0.57585	4.50E-09
NOB1	0.507246	1.91E-06
NOC2L	0.594647	0.002034
NOC3L	0.824872	0.00011
NOD1	-0.31143	0.005547
NOG	0.68394	0.01533
NOL10	0.571707	0.000142
NOL11	0.596508	0.000283
NOL12	0.483635	0.004269
NOL4L	-0.61247	0.012322
NOL6	0.726671	0.000402
NOL7	0.494835	5.17E-05
NOL8	0.532442	0.000101
NOL9	0.636614	1.38E-05
NOLC1	0.897719	4.00E-09
NOM1	0.334972	1.57E-06
NOMO3	0.209755	0.048019
NONOP2	-0.59788	0.03773
NOP10	0.81466	3.98E-09
NOP14	0.703685	4.29E-08

NOP16	0.765542	6.27E-08
NOP2	0.710231	2.16E-07
NOP56	0.888875	3.39E-08
NOP58	0.878723	1.76E-06
NORAD	-0.1705	0.034284
NOS2P2	-3.00881	0.018079
NOS3	1.324825	0.001226
NOTCH2	-0.40534	5.27E-05
NOTUM	-4.22187	6.12E-07
NOV	0.587283	0.036476
NPAS2	1.345536	8.09E-16
NPB	-2.68804	0.012821
NPC2	-0.52049	6.35E-05
NPEPPS	0.539434	0.000321
NPHP1	-0.33772	0.042061
NPHP3	-0.34605	0.008909
NPHP4	-0.38205	0.010017
NPM1	0.74726	2.69E-06
NPM3	0.542757	1.03E-05
NPPB	-5.55744	4.29E-09
NPPC	-1.90494	0.00375
NPSR1	-3.61772	0.000589
NPTN	-0.41983	2.20E-05
NPTX1	-0.59662	0.007281
NPTXR	-1.7963	6.54E-09
NPY4R	-1.51377	0.01837
NR1D1	0.529038	0.007468
NR2C1	0.436308	0.00218
NR2F1-AS1	-0.61914	0.001002
NR2F2	-0.46174	0.004794
NR3C2	0.774359	0.036971
NR4A2	1.094091	2.17E-14
NR6A1	0.273777	0.026073
NRARP	1.457093	2.10E-13
NRBF2	0.271932	0.008238
NRBP1	-0.35745	0.000592
NRCAM	-0.72739	3.18E-05
NREP	-3.05251	1.78E-22
NRG4	-1.07171	9.55E-07
NRIP1	0.591019	0.005912
NRIP3	0.238272	0.032354
NRM	-0.69804	0.000101
NRP1	1.190204	7.00E-10
NRROS	0.683296	0.039211
NRSN2	-1.28453	9.40E-07
NRXN1	2.269336	0.002073
NRXN2	-2.79554	0.017343

NRXN3	-1.24602	4.03E-07
NSA2	0.330213	0.018068
NSD1	-0.28453	2.92E-06
NSF	0.415021	0.001936
NSG1	-1.91244	1.29E-05
NSMCE1	0.359443	0.000458
NSRP1	0.445271	0.001013
NSUN2	0.632056	0.000108
NSUN4	0.499292	1.80E-12
NSUN6	0.468584	0.021356
NSUN7	-0.62778	0.003897
NT5C3A	0.520019	0.001471
NT5C3B	0.281993	0.011156
NT5DC2	-0.88945	1.38E-05
NT5DC3	0.262739	0.038652
NT5DC4	0.914534	3.97E-05
NT5E	2.077916	7.29E-14
NT5M	-0.92032	0.013991
NTAN1	-0.2435	0.015431
NTF3	-0.5468	0.024484
NTF4	-0.70431	0.045488
NTHL1	0.832032	0.001634
NTN1	-0.88246	0.000532
NTN3	-3.0333	0.002512
NTNG1	2.067621	0.00249
NTPCR	-0.23901	0.006604
NTRK2	-1.15409	0.000325
NTS	-1.98797	0.016544
NTSR1	2.972957	3.54E-08
NUAK1	-2.30238	1.09E-35
NUAK2	-0.62587	0.008893
NUCB1	-0.68507	0.003478
NUDCD1	0.582647	0.002364
NUDCD2	0.467401	0.001548
NUDT15	0.517464	0.000616
NUDT16	-0.26766	0.039909
NUDT19	0.542726	2.76E-09
NUDT4	0.708272	1.34E-07
NUDT4P2	0.667275	2.16E-10
NUDT5	0.364967	0.000286
NUFIP1	0.431117	0.001006
NUFIP2	0.407497	0.001003
NUMA1	-0.33514	0.020922
NUP107	0.312363	0.039361
NUP133	0.245981	0.046929
NUP155	0.529864	0.001614
NUP188	0.475625	0.000271

NUP205	0.431858	0.003174
NUP214	0.16155	0.023135
NUP35	0.522025	0.018146
NUP37	0.631339	0.003364
NUP43	0.289623	0.034155
NUP50	0.46882	0.01138
NUP54	0.507623	0.000276
NUP88	0.460594	0.00493
NUP98	0.298838	0.012896
NUPL2	0.571363	7.98E-06
NUPR1	-1.7806	3.82E-05
NUS1	0.303909	0.010234
NUTM2D	-0.89504	0.002789
NXF1	0.328824	0.000493
NXN	-1.35405	2.53E-17
NXNL2	-0.55636	0.023933
NXPE3	-0.94789	1.16E-22
NXPH3	-2.80596	0.000639
NXPH4	-0.89151	4.05E-05
NXT1	0.280937	0.013941
NYAP2	-0.95622	0.004552
NYNRIN	-1.92586	3.44E-08
OAF	1.405769	1.33E-09
OAS2	-1.22766	0.027024
OAS3	-0.37594	0.008009
OBSCN	-0.74488	0.036179
OBSL1	-1.57472	6.53E-08
OCIAD1	0.401335	0.004777
OCLM	0.483405	0.012193
OCLN	1.340159	7.33E-07
ODC1	1.716997	9.93E-09
ODF2L	-0.24738	0.037212
OFD1P17	-1.1462	0.007292
OGDHL	-1.05128	0.003623
OGFOD1	0.321381	0.004482
OGFOD3	-0.48687	0.015362
OGFRP1	0.950325	0.01253
OGG1	-0.50699	0.005916
OLA1	0.641726	4.44E-05
OLFM2	-1.5307	5.76E-06
OLFML2A	-2.27049	9.03E-20
OLFML2B	-4.05406	0.000731
OLMALINC	1.25121	9.02E-06
OLR1	-2.2935	3.31E-35
OMA1	0.539022	0.004119
OPA1	0.329568	0.018622
OPN3	-1.75595	2.03E-12

OPTN	-0.50279	3.62E-05
OR10A3	-2.6218	0.037476
OR2A20P	-2.91222	0.016439
OR2A7	-0.66368	0.000159
OR2W3	-3.30397	0.002632
OR5E1P	-2.96941	0.007158
OR7E14P	1.174837	4.89E-09
OR7E62P	2.804263	0.02243
OR7E91P	1.511611	0.036904
ORAI2	-0.97405	4.38E-05
ORAI3	-1.31986	5.54E-05
ORAOV1	0.282716	0.00778
ORC1	0.319282	0.026098
ORC2	0.612185	0.000254
ORC4	0.37675	0.013928
ORC5	0.484304	0.002343
ORC6	0.442271	0.000371
ORMDL3	-0.68959	0.012413
OS9	-0.37489	0.019622
OSBPL10	0.664123	0.000126
OSBPL1A	-0.82616	7.62E-06
OSBPL2	0.190771	0.018914
OSBPL3	0.542818	0.001097
OSBPL5	-1.1047	0.005384
OSBPL7	-0.75519	0.002331
OSBPL9	-0.28303	0.046639
OSCAR	-1.53142	0.048835
OSCP1	-0.76856	3.11E-08
OSER1-AS1	-0.44405	0.018653
OSMR	0.707513	0.000279
OTUB2	1.799346	1.06E-13
OTUD1	-1.52692	1.01E-06
OTUD3	0.400143	8.48E-05
OTUD4	0.289286	0.032035
OTUD6B	0.812553	7.81E-05
OTUD7B	-0.58179	0.000755
OVCA2	0.646822	0.02143
OVCH2	-3.17951	2.49E-07
OVOL1	1.009072	5.14E-06
OVOL1-AS1	1.279161	0.024049
OVOL2	-0.7006	0.011806
OVOS2	-1.38044	0.011396
OXNAD1	0.288393	0.037581
OXR1	0.369741	0.034472
OXSM	0.492414	0.002981
OXSR1	0.479495	0.000486
P2RX4	-0.82377	1.09E-07

P2RX5	0.521952	3.58E-05
P2RY2	-1.4589	7.20E-16
P2RY6	-1.16663	9.61E-09
P3H1	-1.02546	6.15E-06
P3H2	-1.5455	1.85E-39
P3H3	-1.58298	0.001193
P3H4	-0.80037	0.002373
P4HA1	0.279488	0.033242
P4HA2	-0.51594	1.36E-05
P4HB	-0.45361	0.001059
PA2G4	0.76664	5.67E-10
PABPC1	0.345523	0.008431
PABPC1L	0.429079	3.70E-06
PABPC4	0.281383	0.00012
PABPC4L	-1.23171	6.68E-06
PACERR	0.988975	0.006124
PACS1	-1.22093	8.62E-09
PACS2	-0.48393	0.011885
PACSIN2	-0.55556	4.83E-06
PADI1	1.859101	2.56E-23
PADI2	1.104897	0.000215
PAFAH1B1	-0.18475	0.043325
PAG1	1.089285	1.68E-08
PAICS	0.70999	0.000266
PAK1	-0.34226	0.025216
PAK1IP1	0.571577	0.000825
PAK2	-0.36881	0.000876
PAK6	-0.39155	0.04068
PALD1	1.245682	8.96E-14
PALLD	-2.28819	5.57E-57
PALM	-2.05197	0.000347
PALM2	-0.68571	0.000172
PALM2-AK/	-1.86931	1.46E-30
PALM3	-1.59784	0.000237
PALMD	-1.09789	0.005429
PAM	-0.34882	0.000804
PAM16	0.396383	0.022641
PAMR1	-3.56921	1.11E-08
PAN2	-0.29537	0.023822
PANK1	-0.7837	5.08E-10
PANK2	0.202884	0.009345
PANX1	-0.35897	0.000246
PANX2	-0.96939	0.0042
PAPD5	0.439388	0.001683
PAPLN	-1.26481	3.25E-06
PAPOLA	0.329766	0.032317
PAPSS2	-1.25675	1.25E-08

PAQR3	0.444923	0.000302
PAQR6	-0.73578	0.031443
PARD3	0.21486	0.010994
PARD3B	-0.71285	0.045443
PARD6A	0.879296	0.000706
PARD6B	1.833536	5.97E-18
PARD6G	-0.84407	4.01E-08
PARK7	0.343252	0.001053
PARL	0.306503	0.002093
PARM1	0.757991	0.00606
PARP1	0.266885	0.016978
PARP10	-2.01961	0.000261
PARP14	-0.62222	0.03016
PARP3	-0.47379	0.003621
PARP4	0.509812	6.35E-05
PARPBP	0.701015	0.000125
PARVA	-0.69145	1.69E-14
PATZ1	-0.41137	0.020575
PAWR	-0.45652	0.001358
PAXBP1	0.317445	0.039964
PBDC1	0.751151	0.00019
PBK	0.452695	0.020627
PBLD	-0.87613	1.10E-10
PBX1	-0.90598	0.01665
PBXIP1	-1.80414	2.19E-06
PCAT14	-0.29258	0.048338
PCAT7	-1.39251	2.91E-07
PCBD1	0.6599	6.19E-07
PCBD2	0.409753	0.004867
PCBP1-AS1	0.330244	0.018095
PCBP4	-0.98716	0.002482
PCCA	-0.36307	0.043728
PCCA-AS1	-3.31303	0.005204
PCCB	0.264609	0.001584
PCDH1	0.875664	3.10E-06
PCDH12	1.097126	0.03086
PCDH18	-3.65914	0.044493
PCDH7	-2.49966	1.19E-23
PCDHB10	-1.74518	3.55E-07
PCDHB11	-1.69271	3.52E-05
PCDHB12	-1.45437	2.83E-06
PCDHB13	-1.1647	3.92E-16
PCDHB14	-1.38836	3.82E-12
PCDHB15	-1.13783	0.001945
PCDHB19P	-1.47837	0.011944
PCDHB2	-1.48649	0.001569
PCDHB7	-2.69781	0.001593

PCDHB9	-1.83661	1.22E-05
PCDHGA1	-1.98369	3.29E-07
PCDHGA2	-2.21839	0.003771
PCDHGA3	-2.91865	1.86E-06
PCDHGA4	-1.47786	1.22E-05
PCDHGA5	-0.71356	0.031685
PCDHGA6	-0.76223	0.002339
PCDHGA7	-2.03511	0.003424
PCDHGA8	-1.22146	0.024151
PCDHGA9	-1.06823	3.14E-05
PCDHGB1	-1.76797	6.51E-09
PCDHGB2	-1.50649	2.58E-09
PCDHGB3	-1.62326	0.000132
PCDHGB8P	1.516466	0.03435
PCED1B	-0.719	3.90E-06
PCGF1	0.266931	0.005378
PCGF2	-0.52385	0.03341
PCGF3	-0.21523	0.009305
PCGF6	0.36277	0.005095
PCID2	0.328517	0.029077
PCIF1	-0.34567	0.026557
PCK2	-0.84707	0.001957
PCLAF	0.578871	0.010971
PCLO	-0.96857	0.000927
PCMTD2	0.260126	0.005739
PCNP	0.345564	0.02632
PCNX1	-0.71642	4.40E-05
PCOLCE	-1.25916	0.000185
PCSK1N	-1.58879	0.031686
PCSK4	-2.12795	2.55E-06
PCSK5	0.826964	2.28E-05
PCSK6	-0.34768	0.049228
PCSK9	-1.74331	1.05E-08
PCYOX1	-0.25602	0.04657
PCYT1B	-0.91394	5.35E-05
PCYT2	-0.65391	0.003957
PDCD11	0.579371	3.51E-07
PDCD2	0.670054	1.48E-06
PDCD2L	0.75478	2.94E-12
PDCD5	0.609336	0.001012
PDCD6	0.304528	0.015293
PDCD7	0.298909	0.024092
PDE11A	0.840093	0.025019
PDE12	0.718967	1.85E-08
PDE2A	3.896584	2.91E-17
PDE4A	-0.43977	0.011197
PDE5A	-1.5982	1.25E-11

PDE6A	-1.28449	1.83E-06
PDE7A	-0.34917	0.000181
PDE7B	-1.85677	0.003119
PDE8A	0.550904	0.003455
PDE8B	-0.43636	0.038132
PDE9A	0.364843	0.006746
PDGFA	-1.28244	3.73E-12
PDGFB	-1.72468	3.90E-10
PDGFC	-1.03957	2.00E-12
PDGFD	-0.62009	0.013096
PDGFRB	-5.77253	1.79E-11
PDHA1	0.366742	0.004331
PDHX	0.29213	0.011752
PDIA3P1	-1.17375	0.000144
PDK1	0.644084	0.00011
PDK2	-0.6075	0.002003
PDLIM1	-1.7136	1.06E-30
PDLIM7	-0.8817	0.002435
PDP2	0.440749	0.000518
PDPN	-2.24835	5.01E-19
PDSS1	0.820639	4.75E-05
PDXK	-0.5697	0.000167
PDZD4	-1.77891	0.003363
PDZD7	-0.88438	0.009498
PDZK1	-1.57205	3.48E-16
PDZK1IP1	-1.68246	0.000468
PEG10	0.550707	0.026649
PELI1	-0.38872	0.01778
PELO	0.265926	0.002774
PEPD	-0.39957	0.001835
PER3	-0.75811	0.00064
PERM1	1.142418	0.008078
PERP	-0.22233	0.042617
PES1	0.53657	5.27E-05
PEX1	0.367557	0.028684
PEX11B	-0.26745	0.007906
PEX12	0.624577	0.000294
PEX19	0.188614	0.004414
PEX3	0.384712	0.042366
PEX5	0.274122	0.011502
PEX7	0.525794	8.13E-06
PFAS	0.476425	0.011365
PFDN2	0.861096	4.87E-16
PFDN4	0.318084	0.045631
PFDN6	0.396752	0.001567
PFKFB3	-0.49331	0.007512
PFKM	0.624781	6.07E-08

PFKP	-0.28211	0.004548
PFN4	-1.52578	0.001526
PGAM1P7	2.368283	3.38E-06
PGAM5	0.601788	9.81E-10
PGAP1	-0.40861	0.000814
PGAP3	-1.04159	0.000534
PGBD4	0.507203	0.018445
PGF	1.241662	3.56E-05
PGGT1B	0.239645	0.045847
PGK1	0.463849	0.000128
PGLS	-0.50876	0.039975
PGM1	-0.30184	0.040022
PGM2	0.434033	0.014551
PGM2L1	-1.30413	3.66E-14
PGR	-0.30685	0.025148
PGRMC2	-0.83209	7.09E-15
PGS1	-0.61007	2.14E-07
PHACTR3	0.607056	0.006177
PHACTR4	-0.71725	7.25E-10
PHB	0.482388	0.000162
PHB2	0.431305	2.42E-05
PHBP15	4.19919	0.017985
PHC1	-0.59018	1.44E-05
PHEX	-0.64886	0.004761
PHF10	-0.25373	0.020688
PHF12	-0.28723	0.004428
PHF2	-0.3076	0.015892
PHF5A	0.479543	2.51E-09
PHF8	0.340367	0.029049
PHGDH	-0.70851	0.015499
PHKA1	0.833608	3.28E-09
PHKB	-0.3598	0.00372
PHLDA1	2.451703	9.24E-27
PHLDA2	1.638782	1.96E-13
PHLDB1	-2.08199	2.66E-11
PHLPP1	0.540956	0.003983
PHLPP2	-0.25862	0.00427
PHPT1	-0.23463	0.033102
PHTF2	-0.36508	0.007933
PHYHIPL	-1.64594	0.000562
PI15	-1.39883	7.75E-05
PI3	4.194374	4.95E-12
PI4K2A	-0.27493	0.022054
PI4KA	-0.22909	0.034733
PIAS2	0.324068	0.008619
PIAS3	-0.38427	0.015751
PIBF1	0.476303	0.008838

PID1	-2.31749	5.11E-23
PIFO	-2.93735	7.01E-07
PIGCP1	-0.49031	0.044367
PIGF	0.412948	0.009179
PIGH	0.684812	1.01E-06
PIGQ	-0.66517	0.02598
PIGS	-0.63813	2.12E-05
PIGY	1.109129	0.021342
PIGZ	-1.6728	2.46E-05
PIH1D1	0.342637	0.036193
PIK3AP1	0.810219	0.00147
PIK3C2B	-1.08208	4.40E-09
PIK3C2G	-1.62796	3.36E-13
PIK3C3	0.400895	0.010778
PIK3CD	-0.78743	0.000248
PIK3IP1	-1.77516	2.46E-06
PIK3R2	-0.44999	0.031516
PIK3R3	-0.64312	1.43E-05
PIM1	0.570485	0.000487
PINK1	-0.80585	5.69E-08
PINK1-AS	-0.35553	0.003211
PINX1	0.629116	2.57E-06
PIP4K2B	-0.37013	0.009514
PIP5K1B	-2.00211	0.00775
PIP5KL1	-0.99777	0.004528
PITPNB	0.48647	0.000779
PITPNC1	-0.86214	0.001908
PITPNM1	-0.64288	0.007002
PITPNM3	-0.75615	0.001699
PITRM1	0.333316	0.032645
PITX1	0.44956	0.022992
PJA1	0.431404	0.000269
PJA2	-0.64969	0.000489
PKD1L2	-0.41489	0.031088
PKD1L3	2.513448	0.032195
PKD1P6	-0.49196	0.04981
PKD2	-0.54526	1.29E-07
PKIB	2.84328	2.42E-25
PKIG	-0.67103	0.00044
PKN3	-0.45984	0.040677
PKP2	-0.65573	1.81E-06
PKP4	-0.44121	0.000519
PKP4-AS1	-0.98051	0.001429
PLA2G12A	0.238434	0.014627
PLA2G16	-0.56312	0.009971
PLA2G2F	1.883362	4.38E-07
PLA2G4A	0.932983	7.35E-06

PLA2G4C	-0.83685	0.001002
PLA2G4F	1.359847	1.47E-05
PLA2G7	1.468501	3.90E-07
PLA2R1	-0.95249	7.17E-06
PLAA	0.377417	0.003224
PLAC8	-1.43204	8.91E-08
PLAC9	1.954574	0.011117
PLAU	1.721548	1.29E-08
PLAUR	2.403251	4.40E-12
PLB1	0.559486	0.01334
PLBD1	-0.80354	9.07E-05
PLBD2	-0.36444	0.043971
PLCB1	-1.23031	6.95E-05
PLCB4	-0.671	0.011256
PLCD1	-0.78739	0.017413
PLCD4	0.380869	0.045692
PLCE1	-0.57976	0.001108
PLCG1	-0.80219	1.35E-05
PLCG2	-0.51829	0.017243
PLCL2	0.430655	0.011307
PLCXD1	0.514565	0.0077
PLCXD3	0.693538	0.000266
PLD1	-0.29715	0.003798
PLD2	-0.62963	0.004604
PLD3	-0.9026	0.00059
PLD6	0.527388	0.001178
PLEK2	1.190787	1.99E-06
PLEKHA2	-0.52848	4.04E-08
PLEKHA3	0.420194	0.001962
PLEKHA7	0.456133	4.15E-06
PLEKHA8	-0.4427	3.84E-05
PLEKHA8P1	-0.38632	0.018865
PLEKHB2	0.389284	0.000195
PLEKHF2	-0.42301	0.000454
PLEKHG1	-2.52076	5.99E-12
PLEKHG2	-1.19777	1.97E-06
PLEKHG4B	-1.81003	9.08E-07
PLEKHG5	1.090113	0.002884
PLEKHG6	-0.95272	4.36E-06
PLEKHH1	0.469374	0.000898
PLEKHH3	-0.88228	0.016733
PLEKHM3	-0.70688	1.54E-05
PLEKHN1	1.253909	0.005393
PLEKHO1	-1.91766	5.05E-16
PLGLB2	-2.05286	0.02582
PLIN2	1.168867	6.53E-12
PLK2	-0.85664	2.05E-06

PLK3	1.498564	1.14E-22
PLK4	0.327346	0.032656
PLK5	-2.29419	0.001904
PLOD1	-0.41919	0.018975
PLOD2	-0.80466	1.25E-06
PLPP1	-1.20546	7.60E-10
PLPPR3	-1.81381	3.67E-06
PLPPR5	2.575227	0.002672
PLRG1	0.362641	0.006181
PLS1	0.960271	1.16E-05
PLS3	-0.34379	0.012963
PLSCR1	0.783787	1.63E-10
PLXDC2	-1.61059	8.66E-05
PLXNA2	-0.95384	3.79E-10
PLXNA3	-0.51092	0.037112
PLXNA4	-1.40562	0.004831
PLXNB1	-0.6103	0.038919
PLXNB2	-0.44453	0.027034
PLXNB3	-0.77503	0.027593
PLXND1	-1.69858	3.92E-08
PM20D2	0.414	0.00351
PMAIP1	0.414195	0.006475
PMEPA1	-0.96989	6.65E-07
PMF1-BGL/	-0.50414	0.029207
PML	-0.62959	0.000843
PMM1	-0.47995	0.000209
PMP22	-0.49611	0.013394
PMPCA	0.480907	0.000606
PMPCB	0.291743	0.03706
PMS1	0.518798	0.001174
PMS2P1	0.261053	0.021261
PMS2P10	0.884583	0.007406
PMS2P6	-0.44339	0.037085
PNLIPRP3	4.700996	9.38E-07
PNN	0.392588	0.002912
PNO1	0.648823	5.25E-15
PNP	1.530299	3.38E-15
PNPLA2	-0.54461	0.026626
PNPLA3	-1.17902	4.15E-11
PNPO	0.403396	6.82E-05
PNPT1	0.791532	0.000127
PNRC1	-0.76146	4.76E-05
POC1B	0.546477	0.000547
POC5	0.383766	0.019227
PODXL	0.386322	0.011071
POFUT2	-0.52294	0.001594
POGK	-0.17931	0.011591

POGLUT1	0.337514	0.006722
POLA1	0.409911	0.01038
POLB	0.229095	0.01554
POLE2	0.646239	0.006282
POLE3	0.379254	0.000294
POLG2	0.501314	0.001841
POLH	-0.37647	0.002921
POLI	-0.41608	0.000172
POLQ	0.492557	0.014088
POLR1A	0.28619	0.007476
POLR1B	1.111324	1.75E-15
POLR1C	0.526298	1.30E-09
POLR1D	0.380628	0.001267
POLR1E	0.817045	1.99E-08
POLR2A	0.440248	0.005943
POLR2B	0.337305	0.014896
POLR2C	0.353973	2.42E-07
POLR2D	0.596363	1.09E-08
POLR2G	0.28406	0.000627
POLR2H	0.614065	1.57E-10
POLR2I	0.214541	0.04412
POLR2K	0.329883	0.021901
POLR3A	0.386767	0.000483
POLR3E	0.561662	4.63E-11
POLR3F	0.419901	0.00325
POLR3G	1.653558	3.16E-08
POLR3K	0.888912	3.09E-11
POM121L9	-1.7456	0.006314
POMGNT1	-0.4619	0.019814
POMP	0.463138	0.004766
POMT2	-0.45479	0.001575
POP1	0.621782	9.49E-06
POP5	0.373868	0.031896
POPDC3	0.58429	8.21E-06
PORCN	0.456092	0.01717
POU2F1	0.260251	0.011442
POU2F2	0.594374	0.030939
POU5F1	-0.57362	0.040969
POU5F1B	-1.08294	0.028184
POU6F1	-1.89809	1.39E-07
PPA1	0.433708	0.001562
PPA2	0.490858	0.001695
PPAN	0.513937	0.000674
PPARA	-1.21268	4.68E-08
PPARD	0.941454	1.71E-06
PPARG	0.544231	0.00037
PPARGC1B	1.064044	3.45E-11

PPAT	0.72383	8.91E-07
PPBP	4.996635	2.09E-20
PPCDC	0.776867	2.72E-08
PPCS	0.275426	0.016187
PPDPF	-0.73305	0.013075
PPFIA3	-0.88191	0.011058
PPFIBP1	-0.29313	0.004329
PPIA	0.232834	0.047188
PPIC	-1.02411	9.20E-12
PPID	0.31426	0.023094
PPIF	0.683084	8.65E-07
PPIG	0.363975	0.006638
PPIH	0.359085	0.001491
PPIL1	0.427226	0.002798
PPIL4	0.404535	0.002249
PPIP5K1	-0.26102	0.00223
PPM1B	-0.3358	0.019225
PPM1G	0.46996	1.20E-09
PPM1H	-0.89916	7.63E-07
PPME1	-0.22122	0.044356
PPP1CC	0.397975	0.01042
PPP1R10	0.485937	0.033204
PPP1R12B	-0.60517	0.000282
PPP1R13L	-0.8265	0.005389
PPP1R15B	0.318267	0.011455
PPP1R1C	-0.85458	2.33E-07
PPP1R21	0.60145	0.0001
PPP1R26-A	-0.30982	0.025055
PPP1R3B	-0.68529	5.83E-06
PPP1R7	0.179219	0.020453
PPP1R8	0.375957	0.000255
PPP2CA	0.282835	0.019405
PPP2R1B	0.570004	0.00578
PPP2R2A	0.509946	4.89E-05
PPP2R2B	-1.56193	0.001579
PPP2R2C	-0.38126	0.010309
PPP2R5D	-0.22919	0.030263
PPP3CA	-0.32885	0.001265
PPP4R1	0.434819	0.004615
PPP4R1L	-0.47228	6.20E-07
PPP4R3A	0.204983	0.021799
PPP4R3B	0.307583	0.020813
PPP6R3	0.278104	0.018615
PPRC1	0.304754	0.010393
PPTC7	0.760652	3.63E-10
PPWD1	0.373626	0.019171
PQLC3	-0.57588	1.32E-05

PRAF2	-0.72585	0.002444
PRAG1	-1.09751	2.77E-09
PRCP	-0.38515	0.016698
PRDM1	3.099549	4.15E-19
PRDM11	-0.56534	0.025413
PRDM12	1.461917	0.018589
PRDM13	1.00727	6.46E-05
PRDM15	0.239507	0.041041
PRDX1P1	-2.485	0.034353
PRDX4	0.339674	0.002142
PRELID1	0.603983	4.37E-07
PRELID3B	0.784524	4.84E-07
PREP	-0.22567	0.03065
PREX1	-0.36051	0.020103
PRICKLE1	-0.40184	0.015738
PRIM1	0.494729	0.014531
PRIM2	0.368979	0.024235
PRKAB2	-0.6626	2.04E-06
PRKACA	-0.44095	0.015356
PRKACB	-0.51899	0.006643
PRKAG1	0.218635	0.002483
PRKAR2A	0.370208	0.008964
PRKCA	-0.84089	6.58E-14
PRKCD	0.25009	0.003285
PRKCG	-0.89251	2.09E-08
PRKCQ-AS1	0.73715	0.001697
PRKD1	-1.16262	7.07E-07
PRKD3	-0.54601	1.75E-07
PRKG1	1.702859	0.008813
PRKRIP1	-0.35451	0.010109
PRKY	-0.22535	0.013722
PRMT2	-0.49636	4.18E-05
PRMT3	0.971676	8.54E-07
PRMT5	0.836505	2.95E-09
PRMT5-AS1	0.928653	0.005172
PRMT7	0.313489	0.022154
PRNP	0.627259	1.16E-06
PROC	-2.4095	6.49E-09
PROCR	0.383777	0.030941
PRODH	-2.07786	2.31E-05
PROM2	-0.93248	1.09E-07
PROSC	0.404235	0.000935
PROSER2-A	0.515238	0.043923
PROSER3	-0.4526	0.002053
PROX1	-1.08498	3.72E-06
PRPF18	0.255355	0.036346
PRPF19	0.276295	0.003124

PRPF3	0.337054	0.00044
PRPF38A	0.367278	0.002589
PRPF38B	0.480007	2.13E-06
PRPF4	0.585514	1.61E-05
PRPF40A	0.393316	0.005996
PRPF4B	0.272272	0.04008
PRPS2	0.568003	0.000722
PRR13	0.392572	0.000303
PRR15L	2.289861	5.22E-07
PRR34-AS1	0.343144	0.014058
PRR4	-0.90201	7.48E-06
PRR5-ARH(	0.372145	0.005063
PRR5L	-1.62139	3.16E-14
PRR7-AS1	0.780153	0.03481
PRRC1	-0.32219	0.002668
PRRT2	-1.1913	0.048266
PRRT4	1.133558	1.92E-05
PRSS1	2.728118	4.23E-07
PRSS12	0.79764	8.96E-08
PRSS16	-0.35481	0.000691
PRSS2	4.656816	2.89E-08
PRSS22	0.723099	0.013675
PRSS23	-1.39772	1.53E-26
PRTFDC1	-1.43536	0.000138
PRTG	0.524314	0.002144
PRUNE2	-2.30135	6.58E-07
PRX	-0.75759	0.000718
PSAP	-0.44477	0.002485
PSAPL1	-1.8569	0.00154
PSCA	1.559686	0.000134
PSD2	-3.63162	0.003155
PSD3	0.386752	0.006454
PSENEN	-0.43789	0.040025
PSG1	-1.04635	0.001923
PSG11	-1.39277	0.034644
PSG3	-1.08359	0.008732
PSG4	-1.53374	2.91E-08
PSG5	-1.59095	7.98E-05
PSG7	-2.22043	0.000102
PSG9	-0.85659	0.003636
PSMA1	0.364838	0.016968
PSMA2	0.381047	0.016023
PSMA3	0.477082	0.007632
PSMA5	0.488708	9.15E-05
PSMA7	0.275939	0.010428
PSMB1	0.329626	0.008995
PSMB2	0.230119	0.037655

PSMB3	0.253786	0.001023
PSMB5	0.301973	0.038369
PSMB7	0.418876	0.001192
PSMB9	-1.3628	0.01348
PSMC1	0.220669	0.029184
PSMC4	0.285873	0.00956
PSMC5	0.23601	0.009111
PSMC6	0.365095	0.021124
PSMD10P1	-1.44111	0.01402
PSMD11	0.327895	0.014214
PSMD12	0.576218	0.001662
PSMD13	0.294825	0.002433
PSMD14	0.53234	0.000885
PSMD2	0.479443	0.015575
PSMD4	0.244158	0.002273
PSMD5-AS:	-0.3224	0.010584
PSMD6	0.356638	0.00814
PSMD7	0.200318	0.039368
PSME3	0.459244	0.00803
PSME4	0.26517	0.028884
PSMG1	0.508239	0.002531
PSMG3-AS:	-1.11682	0.00013
PSMG4	0.570279	2.40E-06
PSPC1	0.419574	0.038398
PSPHP1	0.702315	4.42E-05
PSPN	-0.50697	0.026139
PTAFR	1.123677	2.22E-16
PTCD2	0.492155	0.002841
PTCD3	0.353085	0.018213
PTCH1	-0.91365	5.78E-14
PTDSS1	0.514177	0.002868
PTDSS2	0.329103	0.047936
PTENP1	-0.7022	0.041194
PTER	0.987525	1.18E-09
PTGES	0.901654	1.11E-07
PTGES3	0.539311	0.002279
PTGES3L	-1.82482	1.54E-15
PTGES3L-A	-1.67486	0.000463
PTGFRN	-0.80493	2.42E-08
PTGR1	-0.4341	0.001135
PTGR2	0.65803	0.003383
PTGS1	0.750492	0.019406
PTGS2	1.706159	9.67E-10
PTK2B	0.347056	0.000918
PTK6	0.771419	3.67E-05
PTK7	-1.27821	1.84E-06
PTMS	-1.14929	1.66E-05

PTOV1	-0.5298	0.00824
PTP4A1	0.547698	0.000292
PTP4A2	0.349633	0.010147
PTPDC1	-0.37017	9.31E-05
PTPN11	0.26136	0.031894
PTPN12	1.042953	2.02E-14
PTPN14	-0.53712	3.76E-05
PTPN2	0.343051	0.024216
PTPN20	0.311231	0.031473
PTPN21	-1.02915	1.66E-14
PTPN22	1.70306	1.44E-06
PTPN6	-0.52986	0.030984
PTPN9	-0.26346	0.004449
PTPRF	-0.47191	0.009774
PTPRG	-0.20852	0.043769
PTPRH	2.457191	2.50E-44
PTPRJ	-0.76474	9.87E-06
PTPRK	-0.90828	4.74E-18
PTPRM	-0.23242	0.032975
PTPRS	-0.85826	0.00061
PTPRZ1	1.291832	0.005879
PTRF	-1.34127	2.56E-09
PTRH2	0.808115	2.22E-06
PTS	0.34953	0.003033
PTTG1IP	-0.38587	1.36E-05
PUDP	0.822837	0.000711
PUM3	0.691813	0.000237
PUS1	0.364363	0.031662
PUS3	0.627777	3.50E-07
PUS7	0.422947	0.009366
PUS7L	0.471338	0.007106
PWP1	0.367464	0.011708
PWP2	0.614123	0.000488
PXDC1	-1.74022	8.54E-19
PXDN	-0.54274	1.78E-05
PXK	-0.35966	0.025293
PXMP2	-0.31949	0.01875
PXMP4	-0.80158	4.11E-10
PXYLP1	0.840659	0.000429
PYCR1	-0.67068	0.020575
PYGB	0.453737	0.046972
PYGL	-0.43466	1.65E-09
PYROXD1	0.788236	2.81E-06
PYROXD2	-1.18768	4.44E-06
QPCT	-1.94891	2.38E-14
QRICH2	-0.56589	0.004709
QRSL1	0.281281	0.04147

QSOX1	-0.40373	0.049751
QSOX2	0.425027	9.86E-05
QTRT2	0.211864	0.048062
RAB11FIP1	0.951828	9.92E-09
RAB11FIP1	-1.15031	0.000838
RAB11FIP2	0.318593	0.018444
RAB11FIP3	-0.66484	0.007315
RAB11FIP4	-0.6013	3.76E-05
RAB11FIP5	-0.72695	0.010902
RAB17	0.810189	9.96E-07
RAB20	-0.84559	0.000567
RAB25	0.431524	0.000142
RAB27B	2.128161	5.47E-17
RAB30	-1.41467	2.47E-15
RAB32	-1.0978	1.19E-10
RAB36	-0.9316	3.61E-09
RAB38	1.678351	0.005888
RAB3IP	0.350681	0.028332
RAB40A	-0.66422	0.00262
RAB40B	-0.35627	0.004013
RAB40C	-0.54593	0.031055
RAB4B-EGL	-2.97522	0.000566
RAB5B	-0.54731	2.59E-07
RAB6A	-0.24563	0.037599
RAB7A	0.325439	0.005616
RAB7B	1.324156	0.000845
RAB9B	-1.39187	0.031922
RABAC1	-0.71156	0.004648
RABEPK	0.318535	0.006117
RABGAP1L	-0.49645	1.10E-09
RABGEF1	0.519085	0.00079
RABGGTA	0.500228	0.000184
RABGGTB	0.566792	0.000341
RABL2A	-0.80044	0.000422
RABL2B	-0.66912	9.64E-09
RAC2	0.40666	0.002616
RAC3	-0.72085	0.001584
RACK1	0.280061	0.000408
RAD1	0.298558	0.004418
RAD18	0.567183	0.000421
RAD23B	0.4352	0.003659
RAD50	0.329591	0.013659
RAD51D	0.277326	0.003113
RAD54L	0.326947	0.001024
RAD9B	-0.74053	0.026658
RADIL	-0.82937	0.021348
RAE1	0.313736	0.002455

RAET1E	2.26562	1.50E-10
RAET1G	1.262963	2.37E-06
RAG1	-0.34826	0.003303
RAI1	-0.71175	0.011813
RAI14	0.391694	0.00056
RALA	-0.18801	0.029983
RALBP1	0.347285	0.001371
RALGPS2	0.420873	0.004879
RAN	0.383107	0.008836
RANBP1	0.521389	0.000174
RANBP2	0.346907	0.008043
RANBP9	0.371218	0.002886
RAP1B	0.245461	0.043827
RAP1GAP2	0.51027	0.01719
RAP2A	-0.82736	1.85E-09
RAP2B	0.410613	0.03277
RAP2C-AS1	-0.80654	0.003901
RAPGEF1	-0.76568	1.72E-06
RAPGEF5	0.889651	3.03E-11
RAPGEF6	0.510287	0.015173
RARA	-0.99196	0.001745
RARA-AS1	-0.904	0.045755
RARB	-1.35659	0.049828
RARG	-0.92581	5.04E-05
RARRES1	1.532384	0.037248
RARS	0.573362	0.000101
RASA1	0.427749	0.00319
RASA2	0.584635	0.000115
RASA3	0.599515	0.012933
RASA4CP	-1.10831	0.014378
RASD2	-3.41361	1.22E-20
RASEF	0.332032	0.005151
RASGEF1B	1.893437	7.56E-11
RASGRF2	0.486086	0.019132
RASGRP1	-3.60704	5.19E-07
RASGRP2	-2.5341	0.00276
RASGRP3	-0.9745	0.00022
RASL11A	0.72546	0.017824
RASSF1	-0.42301	0.000907
RASSF2	-0.47958	0.001743
RASSF3	0.592639	3.84E-05
RASSF4	-1.27679	1.78E-07
RASSF5	0.630842	0.000136
RASSF6	-0.56372	0.000346
RASSF8	-0.45507	5.92E-05
RBBP6	0.298695	0.002103
RBBP8	0.517848	0.000362

RBFA	0.32487	0.002429
RBFADN	-0.55036	0.031398
RBFOX2	-0.5406	5.31E-07
RBL1	0.452203	0.022234
RBM15	0.422636	2.11E-08
RBM15B	-0.5373	0.006016
RBM18	0.461581	0.000738
RBM19	0.493443	5.11E-06
RBM20	-1.31504	1.86E-05
RBM22	0.330185	1.86E-05
RBM23	-0.26224	0.002616
RBM24	-0.65424	0.000373
RBM25	0.519762	0.000657
RBM26	0.514761	0.000103
RBM28	0.710905	1.14E-07
RBM34	0.436012	0.000822
RBM39	0.25157	0.038402
RBM43	-0.83914	0.000427
RBM46	2.10309	0.01202
RBM47	0.387314	0.027938
RBM4B	-0.20766	0.009162
RBM5	-0.17064	0.013686
RBMS3	-2.73277	1.59E-14
RBMX2	0.197232	0.033678
RBMY2JP	-2.67598	0.01947
RBP1	-1.18344	3.52E-07
RBP7	-1.51397	2.54E-09
RBPMS-AS1	1.010666	0.00965
RC3H1	0.277096	0.01179
RCAN2	-2.50558	4.91E-05
RCAN3	-0.57225	6.95E-09
RCBTB1	-0.19805	0.03846
RCBTB2	-0.3926	0.037813
RCC1	0.437473	2.76E-05
RCL1	0.574288	8.20E-08
RCN3	-2.69817	1.58E-05
RCOR1	0.612243	4.85E-05
RCOR2	-1.08073	0.006919
RCOR3	-0.67305	5.24E-05
RDH10	0.491592	0.001074
RECK	-1.36603	4.17E-09
REEP1	-1.22079	0.021447
RELN	2.805552	1.45E-23
RELT	0.589934	0.029662
REN	1.847464	0.001147
REP15	-0.76191	0.018661
REPS1	0.31971	0.012175

REPS2	-0.32164	0.035956
RER1	0.261041	0.004286
RET	-3.12402	0.027953
REV3L	-0.25053	0.025911
REV3L-IT1	-1.60405	0.023223
REXO4	0.239582	0.026608
RFC3	0.647703	0.000758
RFC5	0.276184	0.017365
RFFL	1.118345	3.26E-13
RFK	0.514165	0.000357
RFLNB	-2.08085	9.10E-18
RFPL4A	1.600836	1.91E-05
RFPL4AL1	2.330679	0.00307
RFPL4B	1.469014	0.047249
RFT1	0.439464	0.000325
RFWD3	0.417522	0.00266
RFX2	-1.02494	6.49E-13
RFX8	2.619129	0.027403
RGCC	1.843191	7.00E-11
RGL1	-1.12336	0.000215
RGMA	0.921277	8.89E-05
RGMB-AS1	0.976565	0.005562
RGPD6	0.485897	0.011553
RGS11	-2.23649	0.000762
RGS12	-0.61107	0.007257
RGS16	1.992496	1.34E-09
RGS17	0.913428	1.53E-05
RGS19	0.423855	0.048113
RGS2	2.061493	1.04E-22
RGS20	0.864265	1.72E-05
RGS4	1.846488	0.003012
RGS9	-1.50593	4.25E-08
RHBDD1	-0.32709	0.016672
RHBDD2	-0.45477	0.007021
RHBDL3	-3.18611	0.012212
RHEBL1	-0.59501	0.005075
RHOBTB1	-1.29424	9.23E-17
RHOBTB3	-1.50463	5.48E-12
RHOF	2.491593	6.45E-26
RHOQ	-0.54052	0.0009
RHOU	-1.61162	1.30E-05
RHOV	0.964517	9.46E-08
RHPN2	0.750573	0.000251
RIC1	-0.69134	1.27E-10
RICTOR	0.524313	0.000933
RIDA	0.569182	8.89E-06
RIF1	0.456746	0.001429

RILPL1	-0.77727	0.000152
RIMS4	-1.25959	0.002183
RIN2	-0.95112	3.08E-30
RINL	0.650467	0.014923
RINT1	0.565368	0.000407
RIOK1	0.762367	5.93E-08
RIOK2	0.624754	3.07E-06
RIOK3	0.311591	0.02732
RIOX2	0.250805	0.018789
RIPPLY3	-1.22569	0.000369
RIT1	-0.43111	0.007442
RMDN3	0.409023	0.004403
RMND1	0.562128	4.12E-05
RMND5A	0.376793	0.004767
RMRP	4.392353	0.000154
RN7SKP116	1.041877	0.001682
RN7SKP70	-1.30559	0.027234
RN7SL100F	2.017157	0.047498
RN7SL166F	3.74915	0.019827
RN7SL208F	2.198952	0.01556
RN7SL274F	-1.74852	0.001266
RN7SL368F	1.995057	0.000171
RN7SL38P	-2.51616	0.036499
RN7SL524F	2.167078	0.04475
RN7SL608F	-0.8696	0.037793
RN7SL667F	-1.07734	0.034578
RN7SL862F	-2.7048	0.032191
RN7SL865F	1.159775	0.039278
RN7SL96P	-2.03456	0.016759
RNA5-8S5	0.642553	0.030295
RNA5SP187	1.374415	0.01473
RNASE10	-1.50953	0.001455
RNASE4	-1.62887	0.000705
RNASE7	-0.43834	0.029847
RNASEH1	0.228443	0.049767
RNASEH1-A	0.311788	0.029329
RNASET2	-0.28553	0.04955
RNF103	0.184386	0.010201
RNF103-CH	1.192851	0.019828
RNF112	-2.59584	0.000597
RNF114	0.296457	0.001417
RNF115	-0.34613	0.000835
RNF121	-0.53174	5.79E-05
RNF122	-1.44653	2.84E-05
RNF125	0.463896	0.015975
RNF130	-0.3479	0.002869
RNF138	0.437445	0.004562

RNF139-AS	-0.27527	0.016526
RNF144A	-1.11022	1.14E-07
RNF144B	-1.45861	2.12E-24
RNF146	-0.23964	0.01643
RNF149	0.52475	3.91E-07
RNF150	-1.75778	0.016686
RNF152	-1.49008	1.08E-37
RNF157	-0.66332	0.004534
RNF165	-1.3535	1.49E-06
RNF169	0.311247	0.020299
RNF180	-0.72893	0.016109
RNF182	-0.76784	0.002599
RNF185	-0.45514	0.000187
RNF19A	-0.60619	3.28E-05
RNF213	-0.42461	3.18E-06
RNF217	-1.50176	1.34E-23
RNF217-AS	-1.64223	6.35E-10
RNF219	0.395856	0.006563
RNF24	-0.36076	0.014708
RNF26	-0.40222	0.047922
RNF4	0.184968	0.032785
RNF41	-0.48484	0.025562
RNF43	-1.29813	4.85E-09
RNF5	-0.29537	0.047193
RNF7	0.21465	0.035939
RNF8	0.321334	5.33E-05
RNMT	0.24008	0.024393
RNPEPL1	-0.55617	0.048009
RNU1-59P	4.282942	0.011827
RNU1-72P	2.220749	0.034415
RNU4-25P	1.648369	0.010716
RNU6-1161	0.695034	0.046406
RNU6-1209	2.588674	0.027887
RNU6-476F	1.147303	0.036402
RNU6-577F	-2.48809	0.030023
RNVU1-4	4.005946	0.003151
ROBO1	-0.79456	1.34E-10
ROBO4	2.877234	0.004518
ROCK1P1	0.976444	0.000688
ROGDI	-1.17118	0.000222
ROPN1L	-1.03644	0.01105
ROS1	-2.2737	1.10E-24
RP1	-0.96662	0.032653
RP1-102E2	-1.67817	0.024528
RP1-102K2	0.727145	3.70E-05
RP11-1000	-0.76466	0.036557
RP11-100E	1.683156	0.033899

RP11-100N	2.228937	0.012686
RP11-101E	1.020191	0.006269
RP11-102N	-2.12719	4.26E-05
RP11-1035	1.843793	0.03226
RP11-1036	-1.41428	0.009211
RP11-1038	-1.1951	0.001117
RP11-104N	0.275071	0.038774
RP11-1055	-1.17975	0.005475
RP11-1055	-1.75116	0.013215
RP11-1069	-1.51223	0.01085
RP11-1081	-2.15966	0.039433
RP11-1085	1.826361	0.031895
RP11-1086	-0.73704	0.018246
RP11-108K	-2.79817	0.008126
RP11-108O	-1.14066	0.033114
RP11-10J2	1.431191	0.00015
RP11-10L1	0.966605	0.036405
RP11-10O1	1.466721	0.039973
RP11-10O2	0.913477	0.002523
RP11-1101	3.392631	0.021669
RP11-1109	-1.44829	3.79E-05
RP11-110G	-0.61267	0.016064
RP11-110I1	2.907586	0.003066
RP11-111M	-0.59954	0.003438
RP11-112L	-2.16366	0.022728
RP11-1149	1.187624	3.53E-08
RP11-1149	0.640796	0.000185
RP11-114H	2.207126	0.001944
RP11-114H	3.618908	1.60E-05
RP11-1151	-5.0251	4.97E-05
RP11-1166	-0.43364	0.028501
RP11-11N5	1.87958	0.031069
RP11-121L	-0.83958	0.033949
RP11-123K	-0.97587	0.013344
RP11-1246	-0.94615	0.013133
RP11-124D	-1.96843	0.013494
RP11-1250	1.031744	0.048667
RP11-1275	-0.62495	0.024944
RP11-127I2	1.770972	0.001236
RP11-1280	-1.12283	0.001675
RP11-1281	1.068162	0.02178
RP11-128L	-3.14213	0.005859
RP11-128N	0.939498	0.048787
RP11-1319	-0.64141	0.006053
RP11-131L	1.225193	0.015976
RP11-131M	-2.73015	0.032801
RP11-131M	-0.74189	0.027018

RP11-132A	1.721997	4.30E-13
RP11-135A	1.811764	0.034397
RP11-138B	-0.99902	0.044059
RP11-138I1	0.854549	0.004047
RP11-140L	-1.2018	0.009806
RP11-141C	1.515253	0.007586
RP11-142E	0.53975	0.005112
RP11-145A	-3.56892	6.15E-32
RP11-145A	-3.82708	0.000592
RP11-146E	-1.49218	0.000577
RP11-147L	-0.45423	0.000184
RP11-148B	0.879195	0.011238
RP11-150O	-2.7618	3.76E-09
RP11-151A	0.896625	0.023204
RP11-154H	-2.39215	5.03E-10
RP11-155G	2.493757	0.003758
RP11-155G	-1.70906	0.022651
RP11-156E	0.542722	0.001308
RP11-156K	-3.1446	0.013246
RP11-15B2	2.596349	2.78E-05
RP11-15F1	-1.71948	0.007867
RP11-15H2	-0.59642	0.000143
RP11-15I11	4.105253	0.001672
RP11-161I6	-1.16792	0.012093
RP11-161M	0.850587	0.013931
RP11-162A	-2.10956	0.03294
RP11-164P	0.647955	0.006363
RP11-164P	0.433444	0.002823
RP11-173M	-1.24043	0.019408
RP11-174O	-1.33574	0.033544
RP11-177C	0.969969	0.032374
RP11-177H	-1.42456	4.28E-06
RP11-180C	3.971586	1.06E-11
RP11-180P	-1.51366	0.003656
RP11-182J1	-0.83383	0.005916
RP11-182J1	-0.96703	0.037824
RP11-182J1	-1.66914	0.019849
RP11-182L	-0.70776	0.006427
RP11-184A	3.694437	0.003821
RP11-184A	2.675814	0.026994
RP11-186B	0.852144	1.25E-06
RP11-187C	-1.06357	0.014659
RP11-18A1	1.591966	0.041854
RP1-118J2	-2.80791	6.96E-08
RP11-196G	0.730919	0.015412
RP11-196G	-1.11377	0.048656
RP11-196G	0.938132	0.042436

RP11-1E6.1	2.377871	0.021728
RP11-1H8.5	0.849161	2.52E-05
RP11-203H	-2.80504	0.031293
RP11-206M	2.521619	1.16E-05
RP11-20D1	0.81151	0.000339
RP11-20G6	1.101055	0.03867
RP11-210M	1.900982	0.033653
RP11-211G	1.288165	0.000425
RP11-212P	-0.85125	1.03E-08
RP11-218E	-0.66062	0.001285
RP11-219A	-1.57361	0.00671
RP11-219E	-3.18742	0.014781
RP11-21L1.	-2.64919	0.030738
RP11-21L19	-1.7506	0.024738
RP11-21L2:	-0.54584	0.043064
RP11-221J2	2.480705	0.001033
RP11-221N	3.128802	0.035659
RP11-221N	1.685668	0.000949
RP11-221N	3.234517	0.001459
RP11-223A	3.800913	0.002393
RP11-225B	0.735568	0.000271
RP11-227G	1.011196	0.027501
RP11-228B	-0.95208	0.024236
RP11-22B2	0.508367	0.016508
RP11-22N1	2.841365	0.000183
RP1-122P2	-1.26563	0.007313
RP11-230G	2.071625	0.001171
RP11-231P	1.35569	0.023352
RP11-23J18	-1.68688	0.000947
RP11-23J9.	-0.92692	2.24E-05
RP11-243J1	1.619298	0.007627
RP11-244B	-0.72859	0.012214
RP11-244F	-3.07711	5.12E-10
RP11-244F	-2.70911	1.08E-13
RP11-244H	-3.27249	0.037442
RP11-244H	-1.69374	0.000242
RP11-244H	-0.63403	0.000543
RP11-244O	-1.65963	0.000261
RP11-248J1	0.862897	0.003322
RP1-124C6	-3.59551	8.97E-05
RP11-250B	-1.4331	0.024447
RP11-253M	-1.8485	2.76E-21
RP11-253M	-2.80508	0.001326
RP11-255G	-2.21394	0.015893
RP11-255G	2.066945	0.004346
RP11-255H	-0.62794	3.12E-05
RP11-255H	-2.19894	0.040083

RP11-256I2	-3.13176	0.030861
RP11-256I9	0.533602	0.04897
RP11-259N	0.627318	0.01698
RP11-259O	1.602992	0.030168
RP11-25H1	-1.51527	0.027058
RP11-25K1	-1.64793	4.65E-13
RP11-267L5	-2.55176	0.015748
RP11-272L1	1.698317	1.09E-06
RP11-275N	-2.48651	0.048024
RP11-276H	-0.84104	8.66E-07
RP11-276H	0.909855	0.021194
RP11-277P	-1.28973	8.39E-07
RP11-278A	1.388377	0.031315
RP11-280G	2.667932	0.00317
RP11-280O	2.737341	0.001041
RP11-281P	0.73469	0.007257
RP11-281P	2.206951	0.018504
RP11-283I3	-0.65575	2.73E-05
RP11-284F	1.08147	0.00079
RP11-285E	3.001659	0.028291
RP11-286H	-1.91086	0.000618
RP11-286N	0.399964	0.010645
RP11-288C	-0.51175	0.005009
RP11-290L1	2.173548	0.002264
RP11-290L1	2.422389	2.32E-08
RP11-291I6	-1.67777	0.000485
RP11-292E	3.265399	0.006271
RP11-295D	0.750088	0.000611
RP11-295G	0.982277	2.60E-05
RP11-295K	-0.90916	0.025094
RP11-298I3	-1.10397	0.002972
RP11-298J2	-0.59824	1.03E-05
RP11-299G	-0.71448	4.30E-05
RP11-299G	-1.14208	2.77E-07
RP11-29G8	0.502405	0.026072
RP11-29H2	-0.8452	0.01282
RP11-300E	-2.92777	0.025787
RP11-303E	-0.30276	0.035323
RP11-305L	-0.64965	0.00451
RP11-305M	1.02915	0.00142
RP11-305O	1.265742	0.010049
RP11-307E	-1.18481	5.37E-07
RP11-307O	-1.15151	5.60E-06
RP11-30L15	0.840911	0.026357
RP11-30P6	1.370509	0.017373
RP11-313J2	3.344922	4.84E-12
RP11-314B	-1.75547	3.06E-13

RP11-314C	-1.89629	0.007146
RP11-314D	0.969957	0.005996
RP11-317N	1.066407	0.043742
RP11-322E	-2.65178	0.025549
RP11-323N	-1.18817	1.25E-06
RP11-324I2	1.378054	0.044965
RP11-324O	0.590231	0.049135
RP11-325K	-0.54923	0.034006
RP11-326C	-2.43283	0.000833
RP11-331F	-2.22605	0.010687
RP11-331F	-3.4266	0.010931
RP11-332H	-1.55955	0.029978
RP11-336A	0.9296	0.031311
RP11-336A	1.007942	0.011023
RP11-337C	-1.45887	1.82E-06
RP11-338N	-2.60048	0.027002
RP11-33E1	-1.1292	0.000148
RP11-33N1	2.169817	0.024266
RP11-342M	1.232843	5.91E-05
RP11-343C	0.364598	0.000507
RP11-343C	2.207957	0.010364
RP11-345J4	0.4847	2.11E-06
RP11-345N	0.965149	0.023078
RP11-345P	4.240637	0.000442
RP11-345P	-0.5246	0.013339
RP11-346C	-1.04789	0.007165
RP11-347C	-2.61932	0.045617
RP11-347P	1.204884	0.000114
RP11-348N	-0.81232	0.031669
RP11-349A	-2.82977	0.010999
RP1-134E1	3.873445	0.000634
RP11-34F2	-0.9717	0.01679
RP11-34P1	0.463292	0.04595
RP11-350J2	0.951386	0.000481
RP11-351I2	-0.956	0.00167
RP11-354P	-2.66191	0.035871
RP11-355F	-1.16682	0.032524
RP11-356I2	0.790255	0.007809
RP11-356J5	-1.5233	0.001444
RP11-356O	3.386468	0.04539
RP11-359B	-0.47481	0.038534
RP11-359E	-1.85682	1.51E-07
RP11-359E	-0.51072	0.042489
RP11-35G9	-0.6227	0.025988
RP11-35J1(	-3.10307	0.011698
RP11-35J1(	-2.10367	0.003418
RP11-363E	0.669627	0.039497

RP11-366H	0.58025	0.015797
RP11-366L:	2.062977	2.26E-05
RP11-366L:	3.302697	1.12E-05
RP11-366L!	1.058478	0.002033
RP11-366N	0.953216	0.000131
RP11-367F:	3.835388	0.022964
RP11-367G	-2.12828	1.11E-08
RP11-368N	2.728876	0.009517
RP11-371A	-1.4046	0.000764
RP11-372B	1.651773	0.005107
RP11-373L:	-0.43316	0.031893
RP11-376O	2.983642	0.019965
RP11-379F	0.822554	0.008182
RP11-379H	-0.54758	1.72E-05
RP11-379K	1.773935	0.00416
RP11-37C7	-1.66963	0.046851
RP11-37C7	-0.77333	0.012177
RP11-380J:	-0.74503	0.029953
RP11-380L:	-1.34638	0.025182
RP11-381K	0.589663	0.001263
RP11-381O	1.175536	0.000534
RP11-382A	2.37901	0.003291
RP11-382A	-0.22579	0.036864
RP11-383J:	-1.15074	0.00302
RP11-384P	-3.29924	0.004326
RP11-384P	-0.40575	0.040157
RP11-385D	-0.88165	0.029147
RP11-387N	-2.37706	0.009887
RP11-388C	-1.01827	0.033651
RP11-388P	-2.58087	0.004408
RP11-38L1!	-1.36173	5.13E-05
RP11-390E	0.570513	0.005409
RP11-390F	-1.43058	0.00188
RP11-390P	-1.38325	7.39E-05
RP11-390P	-1.392	0.048239
RP11-392E	0.788862	0.029149
RP11-393K	-1.37094	0.035441
RP11-394B	-0.82695	0.0429
RP11-394J:	2.101945	0.028021
RP11-396B	0.811868	0.016967
RP11-398B	2.213297	0.000496
RP11-398C	0.42927	0.000182
RP11-398K	-0.4487	0.003041
RP11-400K	-1.7252	0.001224
RP11-400N	1.177305	2.83E-06
RP11-401F:	-2.04751	0.016698
RP11-403A	2.176003	0.008946

RP11-403B	-3.25009	0.025921
RP11-403I1	-0.32735	0.006794
RP11-408B	-0.72378	0.007844
RP1-140K8	-4.35679	3.50E-05
RP1-140K8	-3.77396	7.90E-58
RP11-410L	-1.9361	0.019832
RP11-415F	1.966777	0.010027
RP11-415J	-2.61651	1.98E-06
RP11-416I	0.567043	0.012243
RP11-417L	-1.03123	0.003141
RP11-417L	0.758539	0.000888
RP11-420A	-1.51996	3.72E-13
RP11-420L	-3.32029	7.00E-09
RP11-420L	-2.72423	2.65E-35
RP11-422J	-3.4739	0.007418
RP11-423H	-1.10998	0.003879
RP11-424M	1.189665	0.008063
RP11-426C	-1.78987	0.005435
RP11-429G	0.43661	0.013474
RP11-42B7	-3.46777	0.0022
RP11-432M	3.82567	0.001278
RP11-434N	-4.16502	0.000126
RP11-435O	-1.50156	0.017695
RP11-437J	-2.81185	0.046031
RP11-439E	-0.88359	0.0306
RP11-440L	-0.84709	0.004546
RP11-441F	-2.65796	0.027775
RP11-445O	-1.73437	0.013606
RP11-445O	-2.31671	0.001
RP11-448G	-0.75788	9.60E-05
RP11-449P	-1.7717	0.000359
RP11-44F1	-0.53679	0.031625
RP11-44F2	-1.102	9.47E-07
RP11-44N2	-1.16638	0.001418
RP11-44N2	1.664509	0.012627
RP11-452K	-2.25306	0.037059
RP11-452L	-0.95819	0.005448
RP11-452L	-2.20429	0.044566
RP11-454E	-0.88415	0.000118
RP11-454P	4.042893	0.004524
RP11-456P	1.210901	0.008299
RP11-459D	-2.49765	0.002608
RP11-459E	3.777595	0.000381
RP11-45M	0.428556	0.01416
RP11-45M	0.787596	0.003454
RP11-460N	3.527334	6.93E-09
RP11-461A	0.753819	0.040014

RP11-463I2	7.235835	0.004098
RP11-463I2	2.361774	0.040956
RP11-463O	0.719212	4.29E-07
RP11-464C	-3.26359	1.75E-05
RP11-464D	0.975352	0.007149
RP11-465B	0.414074	0.003677
RP11-465L	-1.06326	0.011543
RP11-466H	0.971801	1.36E-06
RP11-467J1	-0.67924	0.041597
RP11-467L	1.290396	2.82E-07
RP11-469H	3.244893	0.000111
RP11-46H1	-0.97299	0.037423
RP11-470M	-4.11305	0.000797
RP11-473M	1.176543	0.019962
RP11-480A	0.451218	0.035111
RP11-480I1	-0.92916	1.26E-06
RP11-480I1	-0.93164	0.016838
RP11-481F	-2.61086	0.000112
RP11-482H	-3.91049	0.003095
RP11-483I1	-2.10839	0.014521
RP11-486B	-1.12676	1.05E-11
RP11-486G	-0.64631	0.040772
RP11-486L	-1.06172	0.036373
RP11-486M	2.295825	0.02494
RP11-486M	3.528454	3.99E-11
RP11-488L	0.368441	0.009747
RP11-488P	2.448478	8.61E-19
RP11-48B3	-1.07551	0.02502
RP11-490B	-0.72661	0.046564
RP11-490M	-1.08719	0.000364
RP11-493E	-3.0505	0.020662
RP11-494M	-1.74128	4.01E-07
RP11-495L	-2.32818	3.73E-05
RP11-495P	-0.63626	0.03966
RP11-496I2	-1.57066	0.000288
RP11-496I2	-0.91509	0.026585
RP11-496N	2.436075	0.000752
RP11-496N	2.932155	0.018253
RP11-497E	-1.06657	0.041189
RP11-498D	2.011322	0.003751
RP11-499F	-0.93235	0.036879
RP11-49I4.	-3.14483	0.003345
RP11-4C20	-0.61839	0.013645
RP11-4O1.	-0.49162	0.002531
RP11-502I4	-1.2534	0.029715
RP11-506K	-1.60658	1.20E-05
RP11-506M	0.514949	0.008101

RP11-507M	-1.34085	0.007941
RP11-508N	-0.38364	0.047234
RP1-150O5	-0.93639	0.048091
RP11-510N	1.797641	0.001495
RP11-514P	-1.06241	0.000714
RP11-517H	-0.52553	0.014863
RP11-517I3	-0.54108	0.031259
RP11-519G	0.464803	0.029234
RP11-519G	1.602776	0.002895
RP11-51F1	-2.56458	1.21E-05
RP1-151F1	-1.50194	2.12E-07
RP1-151F1	-1.3068	8.30E-10
RP11-521I2	0.854734	0.016821
RP11-521L	1.482186	0.000529
RP11-521C	1.044612	0.029614
RP11-525A	3.261498	5.80E-07
RP11-526A	-1.12538	0.008483
RP11-528A	-3.03209	0.016788
RP1-152L7	-0.45112	0.033926
RP11-530C	-1.50713	0.034429
RP11-532M	-0.91071	0.006468
RP11-535M	0.675964	0.045355
RP11-536C	-2.43976	0.046815
RP11-536C	-3.36989	0.029071
RP11-538I1	4.580485	0.027595
RP11-539E	1.762405	0.009526
RP11-53O1	0.576841	0.039059
RP11-540C	-0.92981	0.00851
RP11-544M	-1.09825	0.00016
RP11-546K	-1.70634	0.000893
RP11-548H	1.162685	0.003502
RP11-549J	1.146773	0.023133
RP11-54A9	1.088242	0.022649
RP11-54O7	-3.60218	0.003404
RP11-551L	-0.66778	0.019732
RP11-553A	-1.48187	0.00283
RP11-553K	-3.37095	0.015822
RP11-554D	-1.45489	0.01598
RP11-557N	-1.95855	0.022491
RP11-55K1	-0.94483	0.016834
RP11-560J	-0.75106	0.04837
RP11-563N	1.447032	0.007628
RP11-564D	0.450044	0.021001
RP11-566E	-0.42165	0.004864
RP11-567E	1.997922	0.013252
RP11-572M	-0.95508	0.014708
RP11-574E	2.293921	0.004048

RP11-577H	1.125601	0.017778
RP11-586K	2.236816	8.66E-05
RP11-588H	0.546358	0.023235
RP11-589M	-1.6874	0.000122
RP11-597D	1.225236	0.04741
RP11-59D5	3.52607	2.13E-27
RP11-603J2	0.498414	0.002354
RP11-609N	0.604617	0.043094
RP11-615I2	-2.93955	0.016811
RP11-616K	2.129764	0.009897
RP11-617F1	-0.97066	0.000216
RP11-618P	1.439867	0.003617
RP11-61A1	-0.80698	0.033507
RP11-61K9	-1.27026	0.003593
RP11-61L2	-1.4185	2.36E-06
RP11-61N2	0.914804	0.000635
RP11-620J1	-1.05606	0.006445
RP11-622C	-1.09466	0.020365
RP11-624L	-0.55853	1.93E-05
RP11-626G	0.655318	0.012977
RP11-635N	1.047371	0.007415
RP11-63E9	1.812321	0.002139
RP11-640N	0.84481	0.023095
RP11-641D	0.519624	0.000215
RP11-642A	1.166317	0.032263
RP11-643A	-2.58795	0.014567
RP11-64B1	-1.23365	4.15E-05
RP11-64C1	-0.62424	0.048752
RP11-64D2	2.837606	1.89E-33
RP11-65L3	-1.22519	0.019388
RP11-660L	0.448753	0.018064
RP11-662M	-2.86779	0.000992
RP11-666A	-1.35569	3.11E-05
RP11-666A	-2.14149	0.000432
RP11-66B2	0.469723	0.036082
RP11-66B2	-0.44331	0.003851
RP11-670E	0.918149	0.02089
RP11-676B	-0.45262	0.046992
RP11-676J1	-1.41404	0.004008
RP11-676J1	-1.19142	0.020935
RP11-677M	3.121862	1.19E-11
RP11-67C2	1.922765	0.000528
RP11-67H2	1.707234	0.007241
RP11-681B	-1.61848	1.02E-05
RP11-681H	-2.1471	0.00013
RP11-685M	-0.95206	0.003951
RP11-685N	0.824349	0.042492

RP11-686D	-1.12554	0.002748
RP11-686D	-1.24072	0.011996
RP11-686D	-0.83892	0.001634
RP11-686D	-0.6432	0.016011
RP11-689P	-1.43824	0.037587
RP11-696N	-1.63884	0.004853
RP11-69E1	0.62905	0.008594
RP11-69E1	0.737154	0.027168
RP11-69I8.	1.3667	2.85E-07
RP11-6J21.	-2.41728	0.000585
RP11-700J1	0.732267	0.022697
RP11-702H	-2.54635	0.037639
RP11-705C	-0.40216	0.013801
RP11-707M	-0.92431	0.029111
RP11-715J2	-0.62442	0.013484
RP11-716O	-3.851	0.001027
RP11-722G	-1.755	0.00085
RP11-727F	2.647665	0.031661
RP1-172H2	2.460509	0.036842
RP11-72M1	1.877286	0.011898
RP11-739P	-2.33443	0.003244
RP11-745A	1.099541	0.000122
RP11-747H	0.564657	0.028427
RP11-74M1	-1.83225	0.00068
RP11-755E	-1.99427	0.004109
RP11-755F	-1.61121	7.72E-06
RP11-756P	0.417846	0.002381
RP11-758M	1.766656	1.34E-05
RP11-758M	-0.65489	0.00019
RP11-75I2.	-2.00894	0.002743
RP11-766F	-4.26512	1.99E-08
RP11-773H	1.293188	0.005558
RP11-774O	-2.77618	3.54E-14
RP11-775D	1.327436	0.009977
RP11-783K	0.630252	0.001775
RP11-78A1	1.69894	0.003775
RP1-178F1	-1.74393	0.003882
RP11-78J2	-0.60018	0.006347
RP11-793H	-2.65514	0.026068
RP11-794P	2.755136	0.028224
RP11-797H	-0.95349	0.008502
RP11-79D8	-2.94594	0.022069
RP11-7F17	-0.80027	0.006328
RP11-7F18	-1.23281	0.007237
RP11-800A	-1.74439	3.61E-09
RP11-800A	-1.5768	0.00105
RP11-803D	0.478541	0.037647

RP11-80I15	-1.24233	0.019885
RP11-80I15	-1.23506	0.029343
RP11-814H	-3.27823	0.034889
RP11-817J1	2.323341	9.20E-05
RP11-817J1	6.457556	5.80E-21
RP11-818F	-1.08881	0.039794
RP11-81A2	-0.58515	0.043573
RP11-81H3	1.3495	0.00301
RP11-823E	2.434207	5.80E-05
RP11-82L18	-1.19424	1.07E-09
RP11-83N9	-1.4096	0.001814
RP11-840I1	-1.69233	0.018848
RP11-843B	0.984527	0.008658
RP11-844P	1.441512	0.000487
RP11-844P	2.077034	0.007634
RP11-845M	-1.38425	0.000585
RP11-846F	2.886869	0.037267
RP11-848P	-0.83326	0.001373
RP11-849F	0.646593	0.036782
RP11-84A1	1.640857	0.043552
RP11-854K	0.862368	0.009914
RP11-864J1	-0.63929	0.016894
RP11-864N	0.767577	0.029196
RP11-867G	-4.01674	1.95E-06
RP11-875O	0.786474	0.02399
RP11-876N	0.715566	0.004284
RP11-879F	1.387406	0.007253
RP11-881M	1.252559	0.046718
RP11-885B	-2.55119	0.047065
RP11-890B	-0.41664	0.005798
RP11-893F	-2.42798	0.000877
RP11-894P	-0.31335	0.046071
RP11-89K1	0.536707	0.013154
RP11-89M2	-2.2205	1.15E-06
RP11-8L2.1	-1.21049	4.37E-07
RP11-90J19	-0.36574	0.043392
RP11-91P1	2.225626	0.038533
RP11-92K1	-1.05003	0.002677
RP11-930P	-2.92251	0.038416
RP11-933H	-1.87096	0.035518
RP11-94H1	2.922659	6.18E-05
RP11-958N	-1.15015	0.010201
RP11-95M1	-0.88119	0.042845
RP11-967K	-0.56707	0.008709
RP11-96C2	5.252671	0.00012
RP11-96D1	0.924857	0.006976
RP11-96K1	0.378031	0.027241

RP11-976B	-1.05998	0.000964
RP11-97O1	1.345974	0.010619
RP11-989E	-0.39886	0.022397
RP11-98J2:	-0.99485	0.0031
RP11-99A1	3.093954	0.001512
RP11-9E17	0.758706	0.0085
RP1-272L1(	-2.64253	0.000388
RP1-283E3	-0.92525	3.76E-06
RP1-283E3	-0.40091	0.016062
RP1-28O1C	1.762084	0.021464
RP1-292L2(	-1.38461	0.001281
RP13-128C	-2.62089	0.013986
RP13-279N	-2.58232	0.040729
RP13-463N	1.377829	3.80E-07
RP13-516M	-1.65524	0.033359
RP13-638C	0.594978	0.04943
RP13-766D	-3.21751	0.012249
RP1-5O6.7	0.669987	0.010665
RP1-68D18	0.545162	0.034197
RP1-78O14	-1.00855	0.008499
RP1-7G5.5	3.559454	0.004832
RP3-324O1	2.019814	0.031854
RP3-325F2	0.927063	0.008451
RP3-329A5	-0.58763	0.02899
RP3-331H2	0.628532	0.003929
RP3-335N1	-1.91243	0.027011
RP3-342P2	1.056206	0.01813
RP3-382I1C	-1.18006	1.12E-05
RP3-394A1	-0.60598	2.31E-05
RP3-395M	3.429189	0.042585
RP3-399L1!	-1.31401	2.09E-08
RP3-412A9	2.614309	0.001462
RP3-414A1	-0.69629	0.027147
RP3-424M(	-0.72145	0.038529
RP3-425C1	-0.39886	0.018656
RP3-439F8	-0.85497	0.013025
RP3-449M8	-0.70309	0.022501
RP3-449M8	-1.00213	0.027251
RP3-453C1	1.060302	0.001566
RP3-467D1	0.554403	0.023104
RP3-467K1	0.92394	0.002096
RP3-475N1	-0.50021	0.043904
RP3-486I3.	-1.42137	0.018434
RP3-495K2	-3.08711	0.03925
RP3-512E2	-2.39724	0.011205
RP3-523K2	0.514983	0.002389
RP4-536B2	-1.4658	0.013867

RP4-564F2	0.656946	0.000889
RP4-564F2	0.7042	0.048216
RP4-568C1	0.485117	0.002169
RP4-591C2	0.75622	0.035963
RP4-594A5	-0.76048	0.004601
RP4-614O4	0.951614	0.039354
RP4-621F1	-1.82124	2.04E-05
RP4-622L5	2.058047	0.039197
RP4-625H1	-1.77904	3.12E-06
RP4-635E1	2.758312	0.011296
RP4-639F2	-1.14952	0.032313
RP4-668J2	1.147233	0.031972
RP4-669L1	-1.2917	0.021634
RP4-671O1	0.621819	0.006369
RP4-740C4	-0.83631	1.93E-05
RP4-742C1	-1.05081	0.00504
RP4-756G2	-0.86261	0.00292
RP4-781K5	0.613811	0.000231
RP4-781K5	1.803848	0.00087
RP4-781K5	1.599719	6.82E-05
RP4-781K5	2.261991	0.006998
RP4-784A1	1.430951	0.025895
RP4-794H1	-2.03575	0.000787
RP5-1021I2	0.984762	0.000996
RP5-1021I2	0.59165	0.00594
RP5-1028K	1.194658	0.000247
RP5-1039K	-0.90833	1.11E-06
RP5-1039K	-2.0235	3.82E-05
RP5-1039K	-0.38589	0.000719
RP5-1042K	-1.36157	0.01314
RP5-1049G	-1.68653	0.027935
RP5-1054A	-2.81847	0.02949
RP5-1056L	1.593066	0.034573
RP5-1112D	-0.62727	0.035054
RP5-1120P	0.542781	0.007765
RP5-1142A	-0.7165	0.049665
RP5-1142J1	1.221138	0.02254
RP5-844F9	1.932797	0.004541
RP5-849H1	-1.79875	0.021875
RP5-864K1	-0.64954	0.026567
RP5-875O1	-0.94927	0.016801
RP5-882C2	-0.97603	0.033252
RP5-899E9	4.282018	0.001085
RP5-900K1	0.520075	0.017536
RP5-907D1	-1.35385	0.02984
RP5-908M	-0.42107	0.031644
RP5-937E2	3.310215	0.043275

RP5-956O1	-0.82517	0.011163
RP5-963E2	-1.21333	0.019185
RP5-965G2	0.495897	0.029346
RP5-965G2	1.183835	0.007227
RP6-65G23	0.922124	2.16E-06
RPA1	-0.18243	0.015976
RPA2	0.463171	2.44E-07
RPAP2	0.255248	0.024058
RPAP3	0.353987	0.022874
RPARP-AS1	0.270789	0.023047
RPE	0.382536	0.016119
RPE65	1.911629	0.002359
RPF1	0.610796	6.05E-06
RPF2	0.772471	9.62E-06
RPGR	0.664641	1.54E-05
RPH3AL	-0.76473	0.017532
RPIA	0.854958	6.64E-21
RPL10	0.228572	0.018407
RPL10A	0.436332	0.000145
RPL10P13	3.831803	0.008103
RPL11	0.396906	0.000186
RPL12	0.380539	5.99E-06
RPL12P42	2.367044	0.045148
RPL13A	0.387059	0.000106
RPL14	0.561204	0.000224
RPL15	0.214424	0.025651
RPL17	0.516577	9.89E-05
RPL17-C18	0.977289	0.027954
RPL18	0.434021	2.86E-06
RPL19	0.407874	3.11E-05
RPL21	0.511229	8.25E-05
RPL21P16	0.564695	0.001321
RPL21P89	-3.36111	0.018096
RPL22	0.453733	4.54E-05
RPL22L1	0.533496	0.002218
RPL23	0.376969	6.83E-05
RPL23A	0.510705	0.003846
RPL23AP42	0.454829	0.000893
RPL23AP53	0.51233	0.041798
RPL23AP7	0.507232	0.000414
RPL24	0.478776	0.000437
RPL26	0.601272	0.000574
RPL27	0.433947	3.98E-05
RPL27A	0.276012	0.000445
RPL28	0.268351	0.022719
RPL29	0.358659	1.12E-05
RPL29P19	-3.11637	0.037096

RPL3	0.319104	0.000474
RPL30	0.324625	0.020846
RPL31	0.40361	0.002026
RPL32	0.405644	0.003324
RPL32P29	0.676733	0.028164
RPL32P3	-0.26806	0.030107
RPL34	0.491709	0.000624
RPL35	0.452221	0.00036
RPL35A	0.475298	0.000234
RPL36	0.27976	0.005927
RPL36A	0.355332	0.011616
RPL36AL	0.386035	0.006482
RPL37	0.381447	0.008586
RPL37A	0.487288	1.85E-05
RPL38	0.549016	0.000303
RPL39	0.472004	0.004206
RPL39L	-0.3668	0.005464
RPL4	0.44868	0.000147
RPL41	0.360839	0.011048
RPL4P5	0.729244	0.036701
RPL5	0.461493	3.40E-05
RPL6	0.484086	7.67E-05
RPL7	0.519731	0.000532
RPL7A	0.336594	4.13E-05
RPL7AP6	1.569287	0.032594
RPL7L1	0.464092	5.81E-05
RPL7P26	-1.3372	0.026651
RPL8	0.291967	0.020994
RPL9	0.476268	4.82E-05
RPL9P9	0.58322	0.000776
RPLP0	0.267121	0.001142
RPLP0P2	1.238831	3.12E-09
RPP14	0.294252	0.000235
RPP25	0.564674	0.011407
RPP25L	0.512095	0.006852
RPP30	0.312984	0.018462
RPP38	0.360266	0.009487
RPP40	0.676389	0.000341
RPRD2	0.208663	0.011864
RPS10	0.384457	3.04E-05
RPS10-NUI	0.338264	0.026656
RPS11	0.335692	0.000589
RPS12	0.43817	0.002223
RPS13	0.584641	0.000314
RPS14	0.337709	0.016
RPS15A	0.331038	0.020833
RPS15AP3(	-1.41726	0.023503

RPS16	0.488893	4.92E-06
RPS17	0.395744	0.003147
RPS18	0.525081	3.19E-06
RPS19	0.365047	0.00101
RPS20	0.294455	0.033576
RPS20P4	1.583455	0.028437
RPS21	0.397438	0.012219
RPS23	0.543441	0.000388
RPS24	0.477025	0.001021
RPS25	0.493841	0.000128
RPS27A	0.517948	0.0006
RPS28	0.389631	0.000186
RPS28P7	0.372606	0.000143
RPS29	0.51129	0.005749
RPS29P14	3.649869	0.009505
RPS2P5	0.314034	0.001857
RPS3	0.333504	1.19E-05
RPS3A	0.427075	0.008811
RPS4X	0.291719	0.024728
RPS4Y1	0.300307	0.012043
RPS5	0.381542	0.001254
RPS6	0.532015	0.000133
RPS6KA2	-0.87254	4.62E-09
RPS6KA3	0.534963	0.000484
RPS6KA5	0.808063	8.80E-06
RPS6KB1	0.350447	0.002678
RPS6KC1	-0.26933	0.003927
RPS7	0.389289	0.004891
RPS7P1	1.302982	0.000449
RPS8	0.39819	7.11E-05
RPS9	0.362519	3.67E-05
RPSA	0.24357	0.009812
RPSAP43	-3.08453	0.022818
RPSAP52	2.309045	3.83E-18
RPUSD4	0.347336	0.009842
RRAD	-0.78081	1.98E-05
RRAGB	-0.47366	0.000626
RRAGC	-0.31555	0.004566
RRAS	-1.06365	1.21E-05
RRAS2	-0.51198	0.000113
RRM2	0.80271	1.13E-05
RRN3	0.485639	0.000326
RRP1	0.909555	3.97E-07
RRP12	0.334603	0.00343
RRP15	0.543281	0.001288
RRP1B	0.435156	2.19E-05
RRP8	0.273447	0.006301

RRP9	0.635859	0.000314
RRS1	0.704275	7.61E-05
RSAD2	-1.58307	0.002959
RSC1A1	0.839069	0.000119
RSL1D1	0.871294	1.55E-08
RSL24D1	0.458001	0.00414
RSU1	-0.58036	8.44E-07
RTCA	0.390918	0.007468
RTKN2	0.777739	0.004124
RTN2	-1.01644	0.001516
RTN4R	-1.47529	5.27E-06
RTN4RL1	-2.63794	5.74E-11
RTN4RL2	-1.13319	0.029687
RUBCNL	-2.78473	0.000178
RUNDC3A-/	-0.71724	0.001168
RUNX1	-0.39234	7.64E-09
RUVBL1	0.507646	8.51E-05
RUVBL1-AS	-2.11149	0.047176
RWDD1	0.33571	0.01589
RWDD4	0.413845	0.00318
RXRA	-0.41635	0.030199
RYK	0.242641	0.019712
RYR1	-0.81851	0.00224
RYR2	2.437439	1.48E-23
S100A13	0.562258	8.58E-06
S100A14	1.042636	2.38E-08
S100A2	0.639205	0.015999
S100A4	0.739648	0.013536
S100A6	1.232991	4.81E-06
S100A7	4.013634	0.006965
S100A8	3.2655	0.009605
S100A9	1.669682	1.70E-21
S100P	2.546479	8.06E-17
S1PR2	-0.5843	0.003887
S1PR3	-1.26486	3.53E-12
S1PR5	-1.49584	1.01E-05
SAA1	-1.03794	0.016938
SAAL1	0.470408	0.0009
SACM1L	0.407934	0.025658
SACS	0.376002	0.011814
SAFB	0.250284	0.042309
SAFB2	0.334072	0.001127
SALL2	-1.16199	0.008816
SAMD10	0.74043	0.00763
SAMD11	-1.74786	0.008477
SAMD12-A	0.956007	0.000632
SAMD15	-0.57262	0.004224

SAMD3	0.634546	0.020817
SAMD4A	-0.95524	9.89E-12
SAMD5	-0.64788	0.000389
SAMD9	1.426571	2.89E-12
SAMHD1	0.438324	0.000265
SAMM50	0.277405	0.009101
SAMSN1	3.115295	0.022427
SAP18	0.274369	0.014237
SAP30L	-0.47575	0.00021
SAR1B	0.381593	0.0037
SARAF	-0.23726	0.006876
SARM1	-1.04306	6.89E-06
SART3	0.175894	0.005152
SASH1	-1.13208	9.61E-21
SASS6	0.544749	0.004995
SAT1	1.356755	1.41E-07
SAV1	0.528688	0.006693
SBDS	0.428154	0.000969
SBDSP1	0.373679	0.002661
SBF2	-0.33151	0.047334
SBF2-AS1	0.586979	1.73E-05
SBK1	-2.60702	3.97E-05
SBNO1	0.5852	0.000144
SBSN	4.178322	1.48E-07
SCAF4	0.241073	0.001506
SCAMP5	-0.8132	0.010561
SCARA3	-2.85729	2.00E-21
SCARB2	-0.30975	0.000801
SCARF2	-1.56388	0.005546
SCARNA16	3.67933	0.014676
SCD	-0.43339	0.007078
SCD5	-0.37064	0.000679
SCEL	1.357338	9.65E-08
SCG2	0.580402	0.044069
SCG5	1.64105	0.000433
SCGB3A2	1.675144	5.85E-08
SCHIP1	-1.24103	5.82E-10
SCLY	0.65539	4.51E-06
SCMH1	-0.27684	0.019019
SCML2	0.471266	0.038151
SCN8A	-0.86998	0.001053
SCN9A	-1.77302	1.17E-05
SCNN1A	1.169247	0.002021
SCNN1B	3.822704	0.036157
SCO1	0.479842	0.001896
SCOC	0.323359	0.020929
SCPEP1	-0.65501	1.59E-07

SCUBE3	-1.29099	0.000184
SCX	-1.37922	0.022873
SDAD1	0.497184	0.001676
SDC2	-2.43796	0.00094
SDC3	-0.97344	0.005271
SDC4	0.516024	1.75E-10
SDCBP	0.503654	0.011812
SDCBP2	0.817832	0.001642
SDCCAG3	0.664931	1.61E-07
SDCCAG8	0.446822	0.00072
SDE2	0.463932	1.25E-08
SDHAF3	0.868331	6.96E-06
SDHB	0.485749	5.37E-06
SDHD	0.476907	0.002547
SDK1	-2.90169	4.58E-16
SDPR	-1.2057	0.032936
SEC11C	0.648262	2.71E-05
SEC14L1	-0.25217	0.007532
SEC16A	-0.32091	0.016142
SEC22C	-0.32575	0.000275
SEC23A	-0.39801	0.000414
SEC23B	0.597301	4.35E-05
SEC23IP	0.294759	0.015372
SEC24D	-0.71099	3.33E-07
SEC31B	-1.15762	0.001032
SEH1L	0.602587	8.91E-05
SEL1L3	-0.99956	3.27E-09
SELENOF	0.375155	0.015177
SELENOI	0.600795	0.000306
SELENOM	-1.37136	0.000429
SELENON	-0.69262	0.001246
SELENOT	0.565052	0.001013
SELENOW	-0.51272	0.023199
SEMA3A	1.505025	8.47E-15
SEMA3B-AS	-2.38743	0.014998
SEMA3C	-0.96106	0.000108
SEMA3D	0.301471	0.016487
SEMA3E	-0.97943	0.000191
SEMA3F	-1.19805	5.66E-06
SEMA4D	0.268841	0.044497
SEMA4F	-0.71178	0.001273
SEMA5A	-1.38257	6.38E-10
SEMA6B	-0.94099	0.002106
SEMA6C	-2.10333	6.11E-08
SEMA7A	0.780182	0.003601
SENP3-EIF4	0.859132	0.031939
SENP6	0.290471	0.031974

SENP7	-0.82164	5.95E-07
SEPHS1	-0.22673	0.005539
SEPHS2	0.222125	0.003152
11-Sep	-0.43447	0.000329
3-Sep	-1.42293	0.000416
5-Sep	-1.83799	8.36E-07
6-Sep	-1.06769	0.000262
SEPT7-AS1	0.91027	0.043323
8-Sep	-1.08248	1.90E-15
SERAC1	-0.29592	0.019943
SERBP1	0.45439	3.09E-06
SERHL2	-0.38245	0.013509
SERINC2	-0.5448	0.00517
SERINC5	0.758083	1.49E-11
SERPINA1	0.795621	0.046689
SERPINA3	-1.34649	0.011239
SERPINB1	1.669175	2.32E-22
SERPINB13	-3.67389	3.10E-10
SERPINB2	4.116784	1.59E-05
SERPINB3	2.829331	0.009282
SERPINB5	0.913456	0.000159
SERPINB6	0.552771	1.20E-08
SERPINB7	-0.6659	0.008107
SERPINB8	0.7108	0.005929
SERPINB9	-1.29899	0.019018
SERPIND1	2.293568	0.00236
SERPINE1	-2.11105	9.73E-20
SERPINF1	-1.0911	2.07E-07
SERPINH1	-1.45422	3.86E-13
SERPINI1	0.887317	0.000299
SERTAD1	1.059781	2.56E-12
SERTAD3	-0.44497	0.047562
SERTAD4	-1.57583	1.22E-54
SERTAD4-A	-0.92437	0.000235
SESN1	-0.31661	0.0046
SESTD1	0.59654	9.83E-06
SET	0.479687	0.000285
SETD3	0.303133	0.007698
SETD5	-0.2076	0.006726
SETD6	0.337981	0.009698
SETDB2	0.488065	0.005132
SETP14	0.897869	0.021062
SEZ6L2	-1.08995	0.003551
SF1	0.325348	0.012916
SF3A1	0.202893	0.019241
SF3A3	0.429964	9.15E-05
SF3B3	0.272159	0.000879

SF3B6	0.444022	0.002019
SFMBT1	0.258801	0.040046
SFN	0.579813	0.045392
SFR1	1.150841	2.11E-08
SFT2D1	0.419748	0.000122
SFTA1P	0.845778	0.000821
SFTPD	-3.40994	0.025219
SFXN1	0.516231	0.000563
SFXN3	-0.58626	0.002374
SFXN4	0.945804	3.83E-08
SFXN5	-0.83346	0.000678
SGCA	-2.9006	0.03336
SGCB	-1.00092	6.33E-18
SGCE	-0.49179	2.52E-06
SGK1	-0.52794	0.009604
SGK2	-2.02176	0.006823
SGMS2	0.661528	1.92E-06
SGO1	0.422679	0.026554
SGPL1	-0.99749	7.95E-13
SGTB	-0.7976	9.72E-07
SH2B2	-0.82247	0.006111
SH2D2A	1.165033	5.71E-05
SH2D5	0.928596	0.007991
SH3BGRL	-0.57223	0.006336
SH3BGRL2	-0.39777	0.004919
SH3BP2	-0.73938	0.000158
SH3BP4	-0.60811	8.01E-09
SH3BP5	-0.65959	0.000746
SH3BP5-AS	-0.56314	0.001867
SH3BP5L	-0.83487	0.000737
SH3D19	-0.26584	0.007123
SH3GL1	-0.55696	0.026166
SH3GL3	-1.22114	3.78E-05
SH3KBP1	0.648632	6.10E-06
SH3PXD2A	-0.56646	0.012949
SH3PXD2A-	1.333438	8.21E-05
SH3PXD2B	-1.34866	1.57E-09
SH3RF1	0.23195	0.034204
SH3RF2	-2.32443	1.01E-18
SH3TC2	0.994894	0.000178
SHANK1	-1.78793	0.002344
SHC2	-2.40681	4.34E-05
SHC3	-1.97802	4.75E-11
SHF	-1.76975	1.62E-05
SHH	-0.86295	0.034905
SHISA2	-1.65435	2.45E-24
SHISA4	-1.24929	0.001556

SHMT1	-0.4935	0.001175
SHROOM2	-0.51191	0.000824
SHROOM3	-0.51947	5.54E-08
SHROOM4	-2.93133	0.022665
SIAH2	-0.5858	1.27E-06
SIDT2	-1.38856	1.43E-10
SIGIRR	0.500639	0.00804
SIGLEC15	0.858067	0.010346
SIGMAR1	0.583393	0.00282
SIK1	-1.14372	7.93E-10
SIK2	-0.4959	9.53E-06
SIMC1	0.366997	0.001622
SIPA1L1	0.278366	0.010057
SIPA1L2	-1.29863	6.76E-07
SIPA1L3	-0.70855	0.000173
SIRPA	-1.43351	1.08E-09
SIX1	0.429699	0.001632
SIX5	-1.2301	0.011899
SKA3	0.780356	6.08E-06
SKAP2	0.622762	0.000385
SKIDA1	-1.49915	4.71E-08
SKIL	-0.79769	8.04E-11
SKIV2L2	0.629126	0.000341
SKP2	-0.37383	2.45E-05
SLAIN2	-0.26775	0.0052
SLBP	0.358323	0.005655
SLC10A5	-0.64093	0.042617
SLC12A4	-0.91377	0.000222
SLC12A5	-0.79825	0.000566
SLC12A6	0.808273	1.79E-06
SLC12A7	0.690433	1.12E-05
SLC12A8	-0.24195	0.021112
SLC15A2	-0.75525	0.003256
SLC16A12	-2.213	2.31E-16
SLC16A14	-0.70207	0.004539
SLC16A2	-1.31717	9.70E-07
SLC16A5	-0.6507	0.003997
SLC16A6	0.645969	2.14E-05
SLC16A9	-0.4751	0.003188
SLC17A5	0.342936	0.006502
SLC18A2	-0.57442	0.03806
SLC18B1	0.670935	1.07E-06
SLC1A1	-0.87877	0.001498
SLC1A3	-1.60973	1.01E-05
SLC1A4	-1.38319	5.58E-06
SLC20A1	1.498954	4.83E-22
SLC22A15	-0.59881	0.004426

SLC22A23	-1.70487	1.07E-20
SLC22A3	-0.96387	2.08E-08
SLC22A4	0.597143	0.002136
SLC22A5	-0.33248	0.000204
SLC23A2	-0.45425	6.11E-05
SLC24A1	-0.44548	0.005739
SLC25A1	-0.59994	0.017958
SLC25A15	0.45556	7.19E-05
SLC25A17	0.451957	0.000787
SLC25A19	1.342131	5.57E-21
SLC25A23	-0.71283	7.57E-06
SLC25A25-/	0.986864	1.15E-12
SLC25A26	0.252063	0.008212
SLC25A27	-0.54634	0.006539
SLC25A29	-1.09316	3.04E-06
SLC25A3	0.297674	0.012537
SLC25A32	0.806001	2.04E-06
SLC25A33	0.478539	0.00047
SLC25A35	-0.6691	0.005009
SLC25A42	-1.02791	5.31E-05
SLC25A45	0.66008	0.000518
SLC25A5	0.529034	1.67E-05
SLC26A11	-0.8287	0.016681
SLC26A2	-0.58893	0.000248
SLC26A9	4.497453	3.36E-06
SLC27A3	-1.14239	0.000184
SLC27A5	0.549574	0.0013
SLC28A3	1.586558	3.00E-06
SLC29A1	0.915335	1.60E-07
SLC29A2	0.275239	0.04932
SLC29A3	-0.40945	0.012924
SLC29A4	-1.8014	1.57E-06
SLC2A1	0.295048	0.018057
SLC2A10	-1.07016	8.53E-05
SLC2A11	-0.51517	0.011407
SLC2A14	-3.06086	0.000556
SLC2A3	-3.49241	3.07E-08
SLC2A4RG	-0.89606	0.010111
SLC2A5	-2.60288	6.40E-14
SLC2A6	-1.10264	4.17E-06
SLC2A8	-0.81339	0.000935
SLC2A9	-1.9801	3.33E-07
SLC30A4	-0.72423	4.40E-05
SLC30A5	0.207425	0.04425
SLC30A7	0.247742	0.046298
SLC30A9	0.246323	0.04497
SLC31A2	-1.13381	6.76E-26

SLC35A3	0.447481	0.001334
SLC35B3	0.245998	0.017582
SLC35E1	-0.16551	0.013916
SLC35E2	-0.61085	0.000845
SLC35E2B	-0.57402	6.78E-07
SLC35E4	0.471891	0.035587
SLC35F2	0.292304	0.004628
SLC35G1	0.561014	0.001337
SLC35G2	0.820741	0.000592
SLC36A1	-0.45086	0.0002
SLC37A1	-0.22953	0.020769
SLC37A2	0.444803	0.00546
SLC38A3	-1.08641	0.028676
SLC38A4	-1.05183	0.030172
SLC38A6	-0.33998	0.049134
SLC38A7	-0.49856	0.038608
SLC39A11	-0.39079	0.04293
SLC39A8	0.774141	1.09E-05
SLC3A2	-0.60029	3.71E-07
SLC40A1	-1.56995	6.53E-07
SLC41A1	0.363895	1.97E-05
SLC41A2	-0.83455	4.24E-09
SLC43A2	-1.12334	0.000343
SLC44A5	-0.63834	0.000108
SLC45A3	0.937707	0.000157
SLC45A4	0.543256	0.001646
SLC46A1	-1.30589	4.52E-10
SLC46A3	-2.04662	1.21E-12
SLC47A2	-2.20285	4.77E-06
SLC48A1	-0.44026	0.033309
SLC4A11	1.007978	2.48E-07
SLC4A7	0.475712	0.003078
SLC4A9	2.516218	0.004406
SLC50A1	0.328914	0.009084
SLC51A	-0.6129	0.000294
SLC52A1	-1.17634	0.01739
SLC5A12	-1.20562	0.000909
SLC5A2	-2.22685	0.043618
SLC5A3	0.420813	0.008792
SLC6A11	0.465841	0.021426
SLC6A15	1.077782	2.98E-13
SLC6A2	-2.95995	4.32E-15
SLC6A6	-0.39004	0.019289
SLC6A9	-1.16893	1.09E-06
SLC7A1	-0.61185	6.43E-05
SLC7A11-A	-0.76168	0.015433
SLC7A5	-0.85169	2.43E-07

SLC7A7	-3.43479	2.51E-37
SLC8A1	-1.10429	3.54E-12
SLC9A1	-0.63953	0.002317
SLC9A2	0.762518	0.00088
SLC9A3P2	1.967265	0.009231
SLC9A3R2	0.808077	0.006808
SLC9A7	0.661079	1.26E-10
SLC9A7P1	-2.7665	0.00086
SLC9B2	0.570881	2.77E-06
SLCO1B3	0.88889	0.004394
SLCO2A1	-3.24007	5.73E-05
SLCO3A1	-0.44451	9.77E-06
SLCO4A1	2.488725	2.95E-24
SLCO4A1-A	2.9752	7.09E-07
SLFN12	-0.62015	0.002065
SLIRP	0.721126	0.000309
SLIT2	-0.72351	0.035526
SLIT3	-2.10282	5.18E-10
SLK	0.694555	1.70E-07
SLMAP	0.354118	0.002883
SLPI	2.472108	3.58E-06
SLTM	0.345885	0.029389
SLU7	0.347321	0.000921
SLX1B	-0.62968	0.001104
SMAD1	-0.27777	0.010662
SMAD6	-2.63274	1.04E-11
SMAD7	-0.84359	0.000114
SMAD9	-0.91036	0.000988
SMAGP	0.243429	0.008604
SMAP2	0.304158	0.00699
SMARCA1	-0.60547	6.71E-05
SMARCA2	-0.50862	0.004545
SMARCAD1	0.280419	0.035212
SMARCD2	0.400244	9.43E-05
SMARCD3	-1.26061	5.34E-06
SMC3	0.392609	0.008728
SMC5	0.220726	0.048456
SMC5-AS1	-0.65252	0.007228
SMCHD1	0.363119	0.019836
SMCO2	0.965417	0.003743
SMCO3	-1.46966	2.59E-05
SMCO4	-0.37007	0.012671
SMG1P2	0.418488	0.013852
SMG6	-0.3131	0.0211
SMIM15	0.395551	0.002987
SMIM20	0.350093	0.004837
SMIM25	0.972062	0.002769

SMIM2-AS:	0.947816	0.02809
SMIM4	0.353932	0.001493
SMIM5	0.86596	0.011644
SMIM6	1.637957	0.001668
SMIM8	0.398824	0.018986
SMN1	0.447479	8.85E-06
SMN2	0.526558	0.000166
SMNDC1	0.384007	0.005972
SMO	-1.68544	7.58E-11
SMOC1	3.480743	4.17E-15
SMPD1	-0.49765	0.043744
SMPD2	0.485778	0.003711
SMU1	0.235631	0.042696
SMURF1	0.386858	0.004494
SMYD2	-0.58201	6.83E-10
SNAI1	0.98229	0.000172
SNAI2	-0.85778	1.14E-05
SNAI3-AS1	-1.32184	0.018407
SNAPC5	0.342782	0.003657
SNCAIP	-1.30247	6.46E-06
SNCG	-1.48454	1.86E-05
SNED1	0.707967	0.001093
SNF8	-0.2002	0.044497
SNHG1	0.581197	0.001666
SNHG12	0.539029	0.00026
SNHG15	0.679553	5.23E-11
SNHG16	0.808353	4.06E-11
SNHG17	0.459735	6.71E-06
SNHG18	-0.55436	7.01E-05
SNHG19	0.426928	0.020682
SNHG20	0.415315	0.002622
SNHG25	0.944802	0.000932
SNHG3	0.609282	0.001872
SNHG4	0.922611	5.46E-05
SNHG5	0.710384	0.000128
SNHG6	0.395123	0.011154
SNHG7	0.423535	8.43E-06
SNHG8	0.661714	3.96E-06
SNHG9	0.610666	0.002668
SNIP1	0.25638	0.002219
SNN	-1.78229	6.66E-15
SNORA14A	1.644189	0.038786
SNORA14B	0.74756	0.042802
SNORA19	1.368956	0.021914
SNORA22	0.81294	0.029353
SNORA2B	1.826908	0.012091
SNORA33	0.579509	0.001168

SNORA65	1.018574	0.004137
SNORA69	2.049471	0.022274
SNORA71A	0.712567	0.038321
SNORA71B	0.575431	0.033439
SNORA71C	0.764815	0.008138
SNORA75	0.870204	0.038254
SNORA80B	1.470183	0.030563
SNORD100	0.809728	0.012221
SNORD101	0.831696	0.014065
SNORD12B	0.777671	0.004319
SNORD14A	0.852982	0.009722
SNORD14E	1.401631	0.001348
SNORD17	0.920094	0.001091
SNORD1B	1.960541	0.0175
SNORD20	1.260749	0.002965
SNORD36C	1.09662	0.020534
SNORD3A	1.599238	0.003769
SNORD46	1.171727	0.002148
SNORD72	1.795609	0.002597
SNORD78	1.057434	0.009852
SNORD82	2.483005	0.008806
SNORD93	1.061668	0.0004
SNORD99	0.892464	0.035919
snoU2-30	4.088541	0.026875
SNPH	-0.90469	0.036098
SNRNP25	0.347706	0.009182
SNRNP27	0.30614	0.021685
SNRNP40	0.417652	0.002108
SNRPA	0.205309	0.024534
SNRPA1	0.347927	0.010579
SNRPB2	0.556121	3.46E-05
SNRPD1	0.583651	2.57E-05
SNRPD2	0.267558	0.013458
SNRPD3	0.25486	0.029925
SNRPE	0.463121	0.005713
SNRPF	0.534791	1.87E-05
SNRPG	0.532149	0.000551
SNRPN	-0.59078	0.000509
SNTA1	-1.10602	4.65E-05
SNU13	0.49566	5.85E-07
SNURF	-1.02812	0.023762
SNW1	0.206618	0.040844
SNX1	-0.26448	0.003891
SNX15	-0.54797	0.002028
SNX16	0.381239	0.010007
SNX18	-0.3939	0.024098
SNX18P3	0.847471	0.002659

SNX18P7	1.227356	0.003682
SNX25	0.510511	0.005106
SNX33	-0.91677	0.00123
SNX5	0.539059	0.000334
SNX7	-0.68012	1.23E-09
SNX9	0.641347	1.41E-09
SOAT1	0.361388	0.030016
SOCS1	1.350155	0.021367
SOCS4	0.342075	0.009305
SOD2	0.366574	0.008532
SOGA1	-1.31299	2.01E-12
SORCS2	-1.80797	1.06E-08
SORD	0.485224	7.45E-06
SORD2P	0.65729	1.94E-07
SORL1	-1.08049	0.000127
SORT1	-0.70443	1.31E-06
SOS2	-0.4713	4.35E-05
SOWAHA	-1.87039	3.02E-11
SOWAHB	-0.77707	5.80E-05
SOWAHC	0.87586	4.18E-23
SOWAHD	-3.62743	5.49E-07
SOX12	-1.24925	0.000308
SOX13	0.401056	0.00134
SOX4	-1.07872	1.07E-11
SOX6	-0.75516	0.000538
SOX7	0.335712	0.00227
SOX9	1.150569	3.03E-16
SP1	0.142689	0.037726
SP110	-0.5059	0.001305
SP140	0.963939	1.02E-05
SP5	-1.93639	0.00034
SPACA6	-0.87822	0.012517
SPACA9	-0.45252	0.035456
SPARC	-4.75114	3.43E-09
SPARCL1	-0.92036	0.001516
SPATA13	0.858113	2.57E-07
SPATA17	-1.22764	3.12E-05
SPATA5	0.318186	0.015682
SPATA5L1	0.74726	9.24E-08
SPATA6	-1.04716	4.26E-05
SPATA7	-0.74341	8.39E-05
SPATA9	-1.22238	0.043883
SPCS3	0.37683	0.028213
SPDYE11	1.366696	0.012016
SPDYE9P	1.366696	0.012016
SPECC1	-0.91648	5.72E-15
SPECC1L	-0.19796	0.007512

SPECC1L-AI	-1.3	9.97E-05
SPEG	-1.06527	0.008135
SPEN	-0.20118	0.004732
SPERT	-1.22939	0.035584
SPIN1	-0.2396	0.020324
SPINK1	1.448278	0.002193
SPINK4	2.699985	0.001003
SPINK5	-1.05531	5.98E-11
SPIRE2	0.863588	1.79E-08
SPNS1	-0.59152	0.047931
SPNS2	2.354707	5.99E-20
SPOCK1	-0.57149	0.001466
SPOCK2	-0.75809	0.014801
SPOCK3	-2.38823	0.009449
SPON1	-0.72302	0.003934
SPOPL	0.274911	0.042675
SPOUT1	0.391959	0.017069
SPPL3	-0.20806	0.01845
SPR	0.283	0.028311
SPRED1	0.296225	0.003792
SPRED2	0.422355	8.26E-07
SPRR1A	4.886434	5.00E-27
SPRR1B	5.027454	7.03E-09
SPRR2A	7.41206	2.73E-32
SPRR2D	5.474632	9.39E-13
SPRR2E	4.643135	1.01E-06
SPRR2F	5.463856	4.64E-06
SPRR3	4.411714	3.82E-30
SPRY1	1.117102	8.39E-05
SPRY2	1.276215	1.97E-12
SPRY3	-0.59618	0.034656
SPRY4	2.141898	4.73E-53
SPRY4-IT1	2.048873	3.43E-05
SPRYD7	0.467156	0.001101
SPSB3	-0.63479	0.003177
SPSB4	-4.02908	0.000869
SPTAN1	-0.29249	0.007977
SPTLC3	-0.30582	0.033143
SPTY2D1	0.242907	0.018313
SPX	1.612372	0.016752
SQLE	-0.62025	2.02E-10
SQRDL	0.884714	0.000864
SRA1	0.366817	0.010848
SRBD1	0.456167	0.011018
SRC	-0.84074	0.000303
SRCIN1	-1.01971	0.006792
SRD5A1	-0.42283	4.03E-05

SREBF2	-0.46657	0.004808
SREK1IP1	0.988229	1.04E-12
SRFBP1	0.786486	3.62E-05
SRGAP1	0.29743	0.00827
SRGAP2B	-0.52761	1.57E-05
SRGN	1.101828	1.08E-07
SRM	0.53063	0.005441
SRMS	1.804832	0.006851
SRP19	0.41092	0.002841
SRP54	0.274962	0.043865
SRP68	0.215429	0.021434
SRP68P3	-1.3964	0.047001
SRP72	0.510518	3.83E-05
SRPK1	0.583512	0.000139
SRPRB	0.415488	0.000419
SRPX	0.50667	0.007306
SRPX2	0.925932	1.55E-05
SRRM1	0.353934	0.000897
SRRM2	0.200909	0.012491
SRRM3	-0.97046	0.012749
SRSF11	0.267	0.034723
SRSF2	0.335824	0.042426
SRSF7	0.436031	0.000508
SRSF9	0.288255	0.010522
SRXN1	0.396139	0.007263
SS18L2	0.402319	0.031845
SSB	0.679749	0.000164
SSBP1	0.550374	0.000113
SSBP2	-0.505	0.000171
SSBP3	-0.70206	0.001629
SSC4D	-1.42589	0.000429
SSFA2	0.849709	2.92E-05
SSH1	-0.56431	8.03E-07
SSH2	-0.5846	3.31E-18
SSR2	-0.23385	0.013982
SSR4P1	-1.27705	0.045047
SSRP1	0.239703	0.002125
SSU72	0.283612	0.005503
ST14	0.39574	0.022734
ST18	2.577121	0.045842
ST3GAL1	-1.19196	5.15E-07
ST3GAL2	-0.75592	0.006227
ST3GAL3	-0.81073	0.004149
ST3GAL5	-0.79714	0.000631
ST3GAL6	0.854512	2.73E-05
ST5	-1.48073	3.31E-14
ST6GAL1	-0.89146	0.017299

ST6GAL2	-0.81258	0.044578
ST6GALNA(	-1.50223	1.05E-07
ST6GALNA(	-0.96837	0.008451
ST7-AS1	-1.03384	0.000182
ST7L	0.249093	0.022728
ST8SIA4	0.702277	0.000405
STAC	-0.63778	7.62E-05
STAC3	-1.45719	0.008074
STAG2	0.382573	0.020991
STAG3L5P-	-0.29554	0.041114
STAM	0.505219	0.000485
STAMBP	0.185286	0.019701
STAMBPL1	1.321169	1.49E-06
STARD10	-0.70025	7.31E-05
STARD4	-0.92783	1.20E-05
STARD4-AS	-2.67374	1.61E-13
STARD5	-1.85474	1.07E-18
STARD7	0.322978	0.000591
STARD9	-0.7582	4.20E-07
STAT2	-0.51386	2.21E-05
STAT3	-0.39866	0.000189
STAT4	-1.25715	0.004725
STAT5A	-0.86999	2.91E-06
STAT5B	-0.29543	0.000158
STAT6	0.390038	0.048399
STAU1	0.24721	0.001684
STBD1	-0.67473	0.01719
STC1	1.687179	0.009154
STEAP1	2.421959	3.54E-14
STEAP1B	2.527867	5.73E-07
STEAP2	0.791238	5.69E-05
STEAP4	2.402266	6.77E-10
STIP1	0.483376	1.25E-08
STK17B	0.85345	8.20E-06
STK19	-0.59553	0.000669
STK32A	-1.28181	0.029746
STK38	-0.6009	1.17E-12
STK38L	-0.24555	0.04945
STK39	0.552901	9.46E-05
STK4	0.579804	6.32E-08
STMN1	-0.45709	0.000348
STN1	0.881328	2.08E-23
STOML1	-0.65824	0.013285
STOML2	0.331186	0.000518
STON1	-2.69458	5.76E-10
STON1-GTF	-5.55793	2.67E-05
STON2	-0.37019	6.05E-05

STOX1	0.485719	0.032881
STOX2	-1.76897	5.41E-14
STPG1	-0.3843	0.000666
STPG2	-0.9952	0.028337
STRAP	0.393838	0.001135
STRBP	0.350701	0.005148
STRIP2	1.267708	5.02E-14
STRN3	0.317927	0.013066
STS	1.044675	1.56E-07
STX11	-1.47418	1.16E-08
STX18	0.290922	0.001765
STX19	-0.45395	0.023942
STX1A	0.797832	0.001412
STX1B	-0.98819	0.007685
STX5	-0.40378	0.019568
STX6	0.221634	0.014282
STXBP3	0.327548	0.039308
STXBP5	-0.34668	1.60E-05
STXBP6	-0.67288	0.004887
SUB1	0.393853	0.007788
SUCLG1	0.304712	0.032351
SUCLG2-AS	-0.46994	0.036739
SUCNR1	1.520945	0.026574
SUFU	-0.66781	0.000252
SUGCT	-1.75086	0.000151
SUGT1	0.505693	0.000237
SUGT1P1	0.693846	0.031363
SUGT1P2	1.837422	0.019438
SULF1	-3.41797	0.00226
SULF2	-0.78682	0.000253
SULT1E1	-2.67641	4.90E-18
SULT2B1	1.964239	1.46E-14
SULT4A1	0.223602	0.036333
SUMF1	-0.19912	0.00873
SUMF2	0.225495	0.008539
SUMO2	0.247679	0.022774
SUN2	-1.447	3.46E-08
SUN3	-0.51005	0.007551
SUOX	-0.32117	0.043213
SUPT16H	0.366739	0.001452
SUPV3L1	0.415523	0.000158
SURF2	0.581933	0.003177
SURF6	0.443861	0.012145
SUSD1	-0.40823	1.63E-05
SUSD2	-1.25704	6.95E-06
SUSD5	-1.00504	1.70E-10
SUSD6	0.308185	0.024148

SUV39H2	0.535268	0.005182
SUZ12	0.496767	0.000927
SV2A	-1.45233	4.95E-06
SVEP1	-1.17907	5.70E-09
SVIL	-0.32359	1.65E-05
SVOPL	-1.12083	0.007755
SWAP70	-0.73913	1.72E-15
SYAP1	0.245664	0.016784
SYBU	-0.879	0.000622
SYCE2	-0.76366	0.037799
SYCP2	-0.64032	0.002204
SYDE2	-0.39473	0.010737
SYF2	0.409044	0.005568
SYK	0.352508	0.012511
SYN3	-1.39577	0.042284
SYNC	-2.52102	4.67E-07
SYNCRIP	0.424713	0.00261
SYNDIG1	-2.88667	0.0024
SYNE1	0.819439	0.00024
SYNE2	-0.75924	3.03E-10
SYNE3	-1.18434	1.71E-06
SYNGAP1	-0.48932	0.022203
SYNGR1	-0.89888	0.000258
SYNGR3	-1.83745	1.16E-10
SYNJ1	0.394195	0.005314
SYNJ2	-0.29577	0.010479
SYNJ2BP	0.421336	0.002287
SYNM	-0.40335	0.000426
SYNPO	-2.35157	6.40E-15
SYNPO2L	-1.05263	0.021077
SYS1-DBND	-1.19836	0.037446
SYT1	-0.60411	0.000537
SYT12	-1.2358	0.00083
SYT14	1.593813	3.80E-08
SYT15	-0.74724	0.000169
SYT16	-1.19201	1.09E-07
SYT17	-0.93211	3.93E-09
SYT2	-1.26641	0.009344
SYTL1	-0.90876	0.011444
SYTL2	0.5857	0.040758
SYTL4	1.802167	8.97E-15
TACC1	0.287631	0.023192
TACC2	-0.29821	0.001725
TACO1	0.362936	0.001644
TACSTD2	0.482125	0.030055
TADA3	-0.64912	0.001431
TAF1	0.221832	0.022224

TAF13	0.323808	0.023646
TAF15	0.305676	0.049423
TAF1A	0.784312	2.28E-05
TAF1B	0.422926	0.00263
TAF1D	0.420695	0.003992
TAF4	0.268714	0.007915
TAF4B	1.139421	4.97E-07
TAF5	0.67252	8.23E-06
TAF5L	0.187381	0.019853
TAF7	0.29662	0.016123
TAF9	0.477134	0.001003
TAF9B	0.33168	0.029841
TAGLN	-4.08901	5.15E-39
TAGLN2	-0.8176	1.81E-07
TAGLN3	1.902316	0.033848
TAMM41	0.255076	0.03957
TANC1	-0.22262	0.001029
TANC2	-0.60261	1.80E-07
TANGO2	-0.2724	0.022026
TANGO6	0.429504	0.011813
TAP1	-1.03788	3.05E-11
TAP2	0.738279	1.27E-13
TAPBP	-0.8513	4.76E-11
TAPBPL	-0.70784	0.000326
TARBP1	0.632425	6.10E-05
TARID	1.539069	1.10E-05
TARS	0.288314	0.034021
TARS2	0.279339	0.045657
TASP1	0.399755	0.003076
TATDN1	0.370931	0.00462
TATDN2	0.286343	0.015304
TATDN2P2	0.562387	0.035944
TAX1BP3	-0.41824	0.012119
TBC1D1	-0.28619	0.045938
TBC1D12	-0.40037	0.000254
TBC1D15	0.441485	0.001855
TBC1D16	-0.77614	0.000991
TBC1D17	-0.86312	0.000221
TBC1D19	-0.58457	0.000939
TBC1D2	-0.96823	4.24E-05
TBC1D20	-0.40542	2.72E-05
TBC1D22A	-0.24908	0.037042
TBC1D26	-1.34965	0.042276
TBC1D29	1.376597	0.02642
TBC1D2B	-0.30144	0.01807
TBC1D30	1.415199	3.53E-10
TBC1D31	0.592967	0.002206

TBC1D32	-0.43628	0.005809
TBC1D4	0.874812	2.53E-07
TBC1D5	-0.25383	0.023006
TBC1D7	-0.27394	0.002513
TBC1D8	1.15169	2.10E-08
TBC1D9	-0.92787	6.47E-07
TBCA	0.475998	0.005638
TBK1	0.280191	0.042863
TBL2	0.254002	0.018734
TBL3	0.330681	0.034439
TBP	0.255178	0.030222
TBPL1	0.462589	0.000596
TBRG4	0.36053	1.40E-05
TBX1	-1.27557	0.000431
TBX3	0.55614	0.007507
TC2N	0.649504	0.000306
TCAF2	-1.3609	4.82E-12
TCAF2P1	-1.81953	0.000359
TCEA3	-0.77795	0.018271
TCEAL3	-0.53534	0.000339
TCEAL8	-0.29131	0.007751
TCEAL9	-0.47152	6.09E-05
TCERG1	0.45753	0.000247
TCF20	0.365271	2.86E-05
TCF4	-0.56281	2.72E-05
TCF7L1	-3.58737	1.45E-28
TCHH	-0.75193	0.0259
TCHHL1	-2.97732	2.29E-09
TCHP	0.412887	0.00514
TCN1	3.405408	3.66E-09
TCN2	1.144473	0.031871
TCOF1	0.430257	0.005485
TCP1	0.706672	5.04E-05
TCP11L1	-0.45152	8.74E-05
TCTEX1D2	-0.38194	0.016646
TCTEX1D4	2.177336	0.000266
TCTN1	-0.49009	0.000144
TCTN2	-0.70303	2.23E-12
TDG	-0.23786	0.009095
TDGF1P5	2.485423	0.039773
TDRD9	2.830102	4.93E-07
TDRP	0.293692	0.00642
TEAD1	-0.39833	0.000256
TEAD2	-1.83839	5.77E-13
TEAD3	-0.55239	0.029136
TEC	1.088073	1.46E-08
TECPR2	-0.46041	0.000861

TEF	-1.13604	7.49E-05
TEKT4P2	-0.44047	0.027202
TENM2	-3.62503	4.10E-07
TENM3	-1.48436	6.89E-22
TEP1	-0.43035	0.00696
TERF1	0.377474	0.00958
TERF2	0.274956	0.00542
TERF2IP	0.437933	2.85E-05
TERT	1.264627	0.003195
TES	-0.85527	2.65E-10
TESK1	-0.6449	0.037428
TET1	-2.10223	1.14E-11
TET2	-0.47587	5.27E-05
TET3	-0.67409	1.39E-05
TEX10	0.799575	2.46E-06
TEX2	0.159699	0.032901
TEX30	0.576717	0.000154
TEX40	1.311248	0.020805
TFAM	0.71567	5.28E-06
TFAP4	0.714193	4.87E-07
TFB2M	0.716751	1.02E-05
TFCP2L1	1.049136	3.52E-09
TFDP1	0.502537	0.001463
TFDP2	-0.27672	0.030032
TFEB	-1.31386	0.001436
TFEC	-1.44295	0.008845
TFPI2	1.983547	4.87E-50
TGDS	0.417812	0.003052
TGFA	1.633462	1.96E-07
TGFB1I1	-2.11292	2.84E-11
TGFB2	-4.48602	4.69E-28
TGFB2-AS1	-4.03322	7.00E-12
TGFBI	-1.1053	1.98E-07
TGFBR1	-0.43719	8.29E-05
TGFBR2	0.255083	0.03432
TGFBR3	1.080831	1.70E-05
TGFBRAP1	0.312911	0.009396
TGIF1	-0.69759	3.45E-11
TGM1	-0.78202	0.007387
TGS1	0.560907	0.000172
THADA	0.515969	0.006672
THAP10	0.548696	0.000332
THAP12	0.322654	0.014679
THBS1	-1.7769	6.22E-12
THBS2	-0.87366	0.021938
THBS3	-1.18387	1.24E-05
THEG	3.26333	0.031016

THEMIS2	0.438705	0.028407
THG1L	0.333692	0.00495
THNSL1	0.579662	2.89E-05
THOC1	0.414175	0.013945
THOC2	0.426986	0.004162
THOP1	0.354525	0.029076
THRA	-0.94382	0.000789
THSD1	-0.57069	0.004516
THSD7B	-4.5656	1.78E-06
THUMPD2	0.707888	4.80E-06
THUMPD3	0.455159	0.000329
THY1	2.858127	0.049401
TIA1	-0.41142	0.000121
TIAF1	0.990792	3.45E-06
TIAM1	0.92117	1.16E-08
TIAM2	-1.43835	4.67E-23
TICAM1	0.832691	0.006233
TIGAR	0.335499	0.030517
TIGD2	-0.34089	0.038372
TIMM10	0.384615	0.011517
TIMM17A	0.400694	0.000116
TIMM21	0.608385	0.004699
TIMM23	0.309749	0.000874
TIMM23B	0.373517	0.000105
TIMM50	0.370091	0.000564
TIMM8A	0.571059	4.27E-05
TIMM9	0.440052	0.009741
TIMMDC1	0.351178	0.020853
TIMP1	1.144723	6.21E-09
TIMP2	-1.14567	3.21E-11
TINCR	-1.27506	5.96E-08
TINF2	-0.32065	0.007553
TIPARP	0.616645	0.007957
TIPIN	1.002808	0.000368
TJP3	0.859066	0.009349
TK1	-0.37937	0.006258
TK2	-0.47958	2.22E-06
TKT	-0.25395	0.028523
TLCD1	0.752612	0.000524
TLCD2	-1.31498	1.72E-08
TLE2	-1.47294	1.55E-05
TLE4	-1.52742	1.49E-28
TLL1	-1.04196	0.000487
TLN1	-0.4459	0.001841
TLR1	0.911967	0.032584
TLR2	-0.43546	0.014013
TLR3	-1.01935	0.032743

TLR4	2.380207	8.92E-34
TLX2	-2.02802	0.025209
TM4SF1	1.633807	6.52E-09
TM4SF19	0.586716	0.0005
TM4SF1-AS	1.536192	0.037044
TM7SF2	-0.67882	0.017952
TMA16	0.521909	0.000964
TMA7	0.417753	0.000355
TMC4	-0.48086	0.034168
TMC5	1.30615	2.21E-07
TMCC1	-0.4153	0.001938
TMCC2	-2.67538	9.79E-10
TMCC3	2.33311	3.27E-20
TMCO1	0.346299	0.017612
TMCO3	-0.32571	0.000199
TMED10	-0.25228	0.010928
TMED4	0.506547	2.02E-07
TMED5	0.496787	0.000388
TMEFF1	-1.21814	7.52E-06
TMEM104	-0.60909	0.024253
TMEM106I	0.566895	0.000103
TMEM110	-0.46211	0.003154
TMEM117	-0.48884	0.037331
TMEM125	0.39695	0.000484
TMEM126/	0.399245	0.012697
TMEM126I	0.531729	0.001163
TMEM127	-0.29197	0.008245
TMEM133	-0.94051	1.75E-09
TMEM138	-0.46935	0.001163
TMEM139	-0.54288	0.0098
TMEM140	-0.44369	0.010387
TMEM144	0.518631	0.001932
TMEM145	1.404826	5.09E-09
TMEM147	0.333729	0.001544
TMEM150(	-0.84251	0.048418
TMEM154	1.35341	6.13E-16
TMEM156	0.8061	0.004097
TMEM158	0.726712	0.001879
TMEM164	0.328607	0.002793
TMEM165	-0.33363	0.000429
TMEM170/	0.347125	0.00033
TMEM171	1.610229	1.31E-19
TMEM175	-0.66182	0.010842
TMEM178I	-1.98264	0.036056
TMEM181	0.379789	0.000685
TMEM182	0.538465	9.56E-05
TMEM184/	-0.69912	0.031755

TMEM185A	-0.59006	0.000105
TMEM186	0.41833	0.013177
TMEM189	-0.63165	0.001138
TMEM19	-0.38128	0.0085
TMEM192	0.28243	0.018017
TMEM2	-0.88352	2.41E-07
TMEM200A	1.882578	1.25E-12
TMEM200B	-0.72312	5.60E-05
TMEM201	0.464022	0.02318
TMEM205	0.240461	0.02477
TMEM206	0.338409	0.001732
TMEM209	0.409182	0.004155
TMEM214	-0.30839	0.009147
TMEM217	-0.7091	0.000215
TMEM219	-0.25094	0.048978
TMEM221	-0.75023	0.042703
TMEM230	0.259157	0.010181
TMEM234	-0.30475	0.045081
TMEM237	-0.3218	0.004731
TMEM243	0.301255	0.044797
TMEM246	-0.55104	2.70E-05
TMEM25	-0.72658	0.001796
TMEM251	0.381707	0.008164
TMEM254	-0.35119	0.001872
TMEM254-	-1.21907	3.33E-05
TMEM255A	-0.96686	0.001861
TMEM255B	-0.9004	0.026961
TMEM256-	-4.29012	0.008729
TMEM261	0.426169	0.000116
TMEM263	-0.69598	2.38E-14
TMEM30B	0.295537	0.001479
TMEM33	0.733278	9.59E-07
TMEM35B	-0.37962	0.024736
TMEM38B	0.826799	5.05E-07
TMEM39A	-0.2964	0.003526
TMEM40	-1.1057	2.56E-10
TMEM41A	0.497103	0.000115
TMEM42	-0.23911	0.049111
TMEM43	-0.22126	0.002136
TMEM44	-0.88469	4.64E-05
TMEM45A	-1.1665	3.20E-19
TMEM50B	-0.33281	0.016655
TMEM51	0.542321	3.65E-05
TMEM51-A	0.885573	0.00116
TMEM52B	-2.55889	8.73E-28
TMEM54	-0.41763	0.012863
TMEM55A	0.190482	0.048609

TMEM59L	-2.6578	1.57E-06
TMEM61	-2.34229	0.00363
TMEM63A	0.444252	5.59E-07
TMEM63B	-0.70108	6.43E-05
TMEM63C	-2.5403	0.000176
TMEM64	1.015278	5.75E-13
TMEM65	-0.60725	1.43E-06
TMEM69	0.423232	0.012175
TMEM70	0.546318	8.04E-05
TMEM71	1.608791	0.003089
TMEM75	1.470094	0.001142
TMEM79	0.630548	0.015379
TMEM86A	-1.71948	3.78E-06
TMEM87B	0.404337	0.007925
TMEM8B	-1.35627	3.59E-05
TMEM9	-0.59439	0.001855
TMEM91	-0.92813	0.002778
TMEM92	0.932219	0.002878
TMEM94	-0.70799	0.002351
TMEM98	-0.66512	1.12E-05
TMEM9B	0.264783	0.040468
TMF1	0.298333	0.025897
TMIE	-2.49975	0.001306
TMOD1	-0.61321	0.005142
TMOD2	-0.86226	1.14E-06
TMPO-AS1	-0.82781	8.82E-05
TMPRSS11I	2.442058	1.37E-14
TMPRSS11I	1.377348	0.003647
TMSB15B	-0.84387	0.026773
TMSB4X	-0.48212	0.001516
TMSB4XP6	-1.74987	0.003192
TMUB2	-0.39111	0.048336
TMX1	0.578993	0.000796
TMX2	0.490126	0.000121
TMX3	0.354882	0.009233
TNC	-2.75542	4.81E-07
TNF	-2.764	1.52E-18
TNFAIP1	-0.58727	0.000165
TNFAIP3	0.75136	0.004905
TNFRSF10A	1.013562	1.35E-11
TNFRSF11A	0.559478	0.000411
TNFRSF19	-0.67297	3.46E-08
TNFRSF25	1.233668	0.002782
TNFSF14	1.790935	0.000297
TNFSF15	1.361689	4.37E-13
TNFSF4	-1.69943	1.03E-05
TNFSF8	2.618642	8.41E-11

TNIP1	0.384645	0.000515
TNKS1BP1	-0.759	0.025859
TNNC1	-1.30955	9.33E-13
TNNI1	-1.19706	1.20E-06
TNNT1	-0.79417	0.001217
TNNT2	-1.23554	0.000503
TNNT3	-1.98981	6.04E-08
TNRC6C	-0.73761	0.000426
TNRC6C-AS	-0.6398	9.74E-06
TNS1	-1.33645	3.69E-07
TNS2	-1.34476	0.000437
TNS3	-1.52675	3.21E-22
TOE1	0.495935	0.000135
TOM1	-0.52642	0.025874
TOM1L1	0.447314	0.014832
TOM1L2	-0.44479	0.025923
TOMM20	0.400802	0.000551
TOMM22	0.558534	2.02E-06
TOMM34	0.480446	1.63E-08
TOMM40	0.552673	0.020572
TOMM5	0.724341	3.43E-05
TOMM6	0.475815	0.000803
TOMM7	0.382402	0.00026
TOMM70	0.490729	0.00049
TOP1	0.719326	2.25E-07
TOP1MT	0.486611	2.48E-08
TOPORS	0.302428	0.016926
TOPORS-AS	0.599874	0.00349
TOR1AIP2	0.341928	0.010394
TOX	-3.05256	1.98E-38
TOX2	-0.52957	0.014673
TP53	-0.51118	0.006622
TP53BP1	-0.29235	2.84E-06
TP53BP2	0.712919	1.47E-06
TP53I3	-1.5259	3.04E-08
TP53INP1	-1.50969	1.17E-27
TP53INP2	-1.4781	2.19E-14
TP53RK	0.269675	0.003729
TP53TG1	-1.31729	8.94E-09
TP63	-1.20058	3.14E-12
TP73-AS1	-0.65501	0.014203
TPD52L1	-0.23934	0.028992
TPD52L2	0.2957	0.005846
TPGS2	-0.33103	1.91E-05
TPH1	0.961338	0.029996
TPI1	0.358939	0.003131
TPM1	-2.87876	8.08E-51

TPM2	-0.87281	0.00011
TPM3P9	0.444574	0.036824
TPM4	-0.57832	5.60E-05
TPMT	0.527252	0.000922
TPO	0.996376	0.001017
TPP1	-0.41609	0.000621
TPP2	0.577344	5.15E-05
TPR	0.292087	0.01519
TPRA1	-0.4021	0.039374
TPRG1LP1	-3.9524	0.000391
TPRKB	0.507922	0.000407
TPRN	0.704497	0.017448
TPST1	-0.66591	1.79E-13
TPST2	-0.71687	1.24E-07
TPT1	0.449823	4.58E-05
TPT1-AS1	-0.24836	0.034584
TPTE2P2	0.992168	0.00528
TPTEP1	-0.41793	0.008772
TRA2B	0.346233	0.00897
TRABD	0.504408	0.039383
TRABD2A	1.075996	1.18E-07
TRAF1	-0.60551	0.012549
TRAF3IP2	-0.19767	0.022968
TRAF5	-0.99177	3.36E-14
TRAFD1	-0.46133	0.002557
TRAM1	-0.43986	0.00113
TRAM1L1	-1.1084	0.006271
TRAM2	-0.76289	1.81E-27
TRANK1	-1.14153	4.03E-06
TRAP1	0.793995	2.05E-09
TRAPPC3	0.286625	0.000557
TRAPPC6A	-1.18257	0.002508
TRAPPC9	-0.22582	0.004451
TRBC2	1.975084	0.03189
TRBV30	1.704868	0.030744
TRDC	4.782906	6.33E-05
TREH	-3.91902	0.001592
TREML2	2.621126	9.09E-06
TRHDE	0.370817	0.043306
TRHDE-AS1	0.82477	1.21E-06
TRIAP1	0.255467	0.044442
TRIB1	0.78435	5.33E-15
TRIB2	0.881754	1.32E-08
TRIB3	-0.63805	0.030091
TRIM13	0.249218	0.045787
TRIM14	0.577869	6.70E-05
TRIM16	-1.006	1.73E-20

TRIM16L	-1.24262	4.08E-12
TRIM17	-0.98837	3.32E-05
TRIM2	-0.53213	0.000713
TRIM21	-0.27	0.006877
TRIM22	-1.66222	8.59E-06
TRIM24	-0.66826	0.001935
TRIM25	0.388498	2.48E-05
TRIM29	-0.34658	0.022292
TRIM31	1.730823	0.023202
TRIM32	-0.72403	8.58E-08
TRIM33	0.33044	0.004547
TRIM35	0.500033	8.55E-05
TRIM36	-0.62033	0.003167
TRIM37	-0.35761	0.009922
TRIM47	-1.09451	0.000539
TRIM5	-0.18888	0.040237
TRIM56	-0.85676	7.70E-09
TRIM58	-1.01138	1.50E-07
TRIM59	-0.36649	0.013052
TRIM6	-1.2403	4.97E-34
TRIM62	-0.85156	0.000362
TRIM63	2.184213	4.91E-05
TRIM7	0.593389	0.016221
TRIM8	0.358677	0.000305
TRIO	-0.81951	2.05E-13
TRIOBP	-0.81128	0.000264
TRIP11	0.366977	0.012083
TRIP12	0.33579	0.007095
TRIQK	-0.34017	0.045081
TRMO	0.269878	0.021717
TRMT1	0.500009	0.008284
TRMT10A	0.470457	0.020266
TRMT10C	0.812349	4.12E-05
TRMT11	0.461269	0.004424
TRMT13	0.526419	0.001738
TRMT2B	0.279153	0.036883
TRMT44	0.279886	0.004155
TRMT5	0.541047	0.000135
TRMT6	0.898504	9.97E-07
TRNP1	0.807883	0.00505
TRNT1	0.502083	0.007634
TRPA1	-3.10432	0.01445
TRPC1	-1.11137	5.68E-17
TRPC4	-3.84067	2.74E-20
TRPM2	-1.63056	0.000318
TRPM4	-0.55023	0.044287
TRPM6	-1.2096	0.014417

TRPM8	1.339325	0.002542
TRPS1	-0.92268	2.83E-10
TRPV2	1.978279	0.005911
TRPV3	2.056428	2.82E-13
TRPV4	-0.48977	0.016655
TRPV6	-2.37583	6.45E-07
TRUB2	0.333115	0.005023
TSC22D1	0.582434	6.32E-15
TSC22D1-A	-1.05527	0.003785
TSC22D2	-0.16292	0.023709
TSEN2	0.836393	1.89E-06
TSEN54	0.38358	0.027227
TSFM	0.364703	0.001412
TSHZ2	-1.05824	0.028798
TSHZ3	-0.94578	4.01E-05
TSIX	0.391892	0.019315
TSKU	-0.636	0.003965
TSNAX	0.288834	0.034059
TSPAN1	0.721964	6.45E-07
TSPAN11	-1.91662	0.007481
TSPAN2	-2.15851	1.95E-16
TSPAN3	0.187837	0.012768
TSPAN9	-0.90958	0.001488
TSPYL2	0.238628	0.024828
TSPYL4	-0.53209	3.75E-05
TSR1	0.713263	7.00E-08
TSSC1	0.216863	0.038211
TSSC2	-0.69743	0.001703
TTBK2	-0.65564	6.73E-07
TTC1	0.277746	0.007371
TTC13	0.550748	3.77E-06
TTC14	0.439516	0.002778
TTC21A	-1.28251	1.56E-09
TTC22	-0.31691	0.019715
TTC23	-0.65104	1.69E-06
TTC27	0.391351	0.021044
TTC28	-0.76942	0.002136
TTC3	-0.47191	2.25E-08
TTC30A	-0.55259	0.007748
TTC31	-0.33222	0.010676
TTC3P1	-0.64008	0.032598
TTC4	0.559154	3.16E-06
TTC5	0.311731	0.000794
TTC6	0.459978	0.031712
TTC7A	-0.67573	0.000178
TTC7B	-0.34328	0.0006
TTC9	0.727651	9.78E-09

TTF2	0.391986	0.035469
TTLL1	-1.10009	0.00131
TTLL11	-0.47331	0.03132
TTLL11-IT1	-1.51277	0.000108
TTLL12	0.661463	0.000131
TTLL13P	1.517738	0.034028
TTLL2	-2.76038	0.041264
TTLL3	-0.84354	0.000445
TTLL4	0.385313	0.001006
TTLL7	-1.01531	2.85E-08
TTTY16	-1.18773	0.0169
TTTY4	-2.59383	0.041538
TTTY4B	-2.59383	0.041538
TTTY4C	-2.59383	0.041538
TTYH1	0.787983	0.000366
TTYH3	-1.44709	2.62E-05
TUB	-0.99218	0.001945
TUBA1A	-2.91988	2.25E-33
TUBA8	1.03512	0.034546
TUBB	-0.34487	0.001457
TUBB2A	-0.6349	0.001582
TUBB2B	-1.67823	0.000897
TUBB3	-0.75528	0.001713
TUBG2	-0.38695	0.031378
TUBGCP3	0.260662	0.03224
TUBGCP4	0.351173	0.00541
TUFM	0.44206	0.003649
TUFT1	-0.37109	0.002629
TUG1_1	-0.73185	0.04139
TULP3	-0.39584	6.61E-09
TULP4	-0.47887	2.66E-07
TUSC2	0.483486	0.022057
TUSC3	-0.27337	0.005648
TVP23B	0.3255	0.020227
TVP23C-CD	-1.01264	0.000813
TWF1	0.525021	0.000415
TWIST1	-0.68516	0.001203
TWISTNB	0.623766	5.11E-05
TWNK	0.539188	3.05E-05
TXK	1.139585	8.44E-07
TXLNG	0.821037	6.32E-06
TXN	0.472183	0.00797
TXNDC12	0.237137	0.029902
TXNDC17	0.426749	0.000194
TXNL1	0.421169	0.001815
TXNL4A	0.454451	0.000183
TXNL4B	0.207558	0.017506

TYW1	0.31707	0.013153
TYW1B	0.604076	0.000707
TYW3	0.572352	0.000438
U1	1.452051	6.16E-05
U2AF1L4	-0.35707	0.025835
U2SURP	0.496884	0.000686
UACA	-0.93726	9.13E-14
UAP1L1	-1.44084	0.000249
UBA2	0.563579	6.50E-05
UBA3	0.272273	0.049214
UBA5	0.499349	5.04E-05
UBA52	0.356848	0.000652
UBA6	0.564474	0.000334
UBA7	-1.20808	0.04215
UBALD2	0.440522	0.003785
UBAP2L	0.211193	0.037466
UBASH3B	0.391913	0.02371
UBE2C	0.320143	0.010497
UBE2D3	0.218819	0.0116
UBE2E1	0.335881	0.027662
UBE2F	0.316425	0.006418
UBE2G2	0.522429	2.57E-06
UBE2I	0.192816	0.003882
UBE2J1	-0.70681	0.000111
UBE2K	0.328921	0.014084
UBE2L6	-1.3045	0.008146
UBE2N	0.556741	7.71E-05
UBE2Q1	0.227436	0.001415
UBE2Q2	-0.24001	0.01142
UBE2Q2P1	-0.40327	0.031703
UBE2Q2P2	-0.49953	0.012168
UBE2R2	0.448257	5.35E-10
UBE2T	0.437857	0.005457
UBE2V2	0.31533	0.012542
UBE3A	0.31383	0.008984
UBE3B	-0.32232	1.03E-06
UBE3C	0.320663	0.001647
UBE3D	0.447526	0.016425
UBE4A	0.354783	0.008655
UBE4B	-0.54649	1.52E-14
UBIAD1	0.489666	2.51E-07
UBL3	-1.06927	4.08E-10
UBL7	0.430904	0.000119
UBQLN1	0.324024	0.015249
UBQLN2	-0.2959	0.000921
UBQLN4	0.293137	0.000822
UBR1	0.25077	0.033837

UBR7	0.246123	0.027224
UBTD1	-0.94782	0.001978
UBTD2	-0.46095	1.40E-07
UBXN8	1.228932	2.61E-09
UCA1	0.285097	0.004764
UCHL1-AS1	2.007987	0.042064
UCHL3	0.473196	0.002353
UCHL5	0.914224	4.81E-08
UCN2	-0.87128	0.024226
UCP2	-0.58827	0.001301
UFD1L	0.240276	0.001417
UFL1	0.362248	0.033255
UGP2	-0.37591	0.002497
UGT1A1	-1.87487	5.93E-20
UGT1A2P	-3.23614	0.000566
UGT1A6	-2.01518	1.11E-14
UGT1A7	-0.97373	0.03898
UGT8	2.348715	0.020473
UHMK1	0.259788	0.040513
UHRF1BP1	-0.31911	0.034781
UHRF2	0.585306	6.36E-06
UIMC1	0.356807	0.001707
ULK1	-1.03624	0.000345
ULK2	-0.37047	3.50E-05
ULK3	0.766719	0.000264
UMAD1	0.341212	0.031892
UMPS	0.665894	3.10E-05
UNC119B	-0.13304	0.037974
UNK	-0.41662	0.00746
UPF2	0.351432	0.005588
UPK1B	1.132544	0.006794
UPK3B	-0.92051	0.00373
UPK3BL	-0.92996	9.49E-06
UPP1	1.02599	2.79E-14
UQCRB	0.462122	0.000322
UQCRC2	0.379277	0.014863
UQCRFS1	0.463628	0.000368
UQCRH	0.574216	6.11E-06
UQCRQ	0.273832	0.040438
URAHP	-0.89242	0.027052
URB2	0.561666	0.000235
URGCP	0.368059	0.018415
URI1	0.403091	0.001926
USMG5	0.529742	0.002187
USO1	0.289608	0.034233
USP1	0.275594	0.048178
USP10	0.304601	0.006776

USP12	0.621998	6.26E-05
USP15	0.399856	0.00511
USP16	0.503075	0.002831
USP18	-0.72489	0.015877
USP22	-0.51708	6.46E-07
USP27X	-0.57525	0.023258
USP2-AS1	1.06372	1.32E-05
USP3	0.402889	0.005877
USP30	-0.49118	0.000225
USP31	-0.27674	0.006645
USP32P1	-0.59159	0.000144
USP32P2	-0.81233	7.41E-07
USP32P3	-1.22658	2.39E-06
USP35	-0.59257	0.023111
USP36	0.347694	0.000585
USP38	0.308006	0.007241
USP40	-0.27137	0.0061
USP42	-0.1766	0.01147
USP45	0.350875	0.021549
USP49	-0.57213	3.09E-05
USP51	-1.06683	0.005027
USP53	0.335227	0.027013
USP6NL	0.539613	0.002882
USP7	0.400668	0.002163
UST	-0.67335	0.038786
UTAT33	-0.74421	0.033722
UTP11	0.773777	1.33E-06
UTP14A	0.631748	1.36E-11
UTP14C	-0.42082	1.42E-06
UTP15	0.588763	0.000236
UTP18	0.328713	0.030598
UTP20	0.435164	0.031553
UTP23	0.517751	3.53E-05
UTP3	0.705228	2.06E-05
UTP4	0.663581	2.75E-05
UTP6	0.687129	3.10E-05
UTRN	-1.13918	4.70E-15
VAC14	-0.33586	0.031841
VAMP1	-0.79163	2.38E-05
VANGL1	-0.63416	4.73E-06
VANGL2	-1.45924	2.87E-15
VAPA	0.500719	0.002693
VASH1	-1.51976	3.15E-05
VASH2	-2.24284	1.04E-09
VASN	-0.78524	0.038302
VAT1	-0.35663	0.047038
VAV2	-0.46538	0.031232

VAV3	-1.51131	9.80E-07
VBP1	0.326163	0.035805
VCAN	-2.04438	0.011533
VCL	-0.30215	0.002234
VDAC1	0.617687	5.71E-05
VDAC2	0.271736	0.029158
VDAC3	0.305662	0.043732
VDR	-0.35234	0.016202
VEGFC	-0.81742	3.75E-07
VEZF1	-0.21167	0.028893
VEZT	0.395547	0.00526
VGF	0.695819	0.013176
VGLL4	-0.69569	2.05E-07
VIL1	-5.06325	6.33E-07
VILL	1.705589	6.47E-07
VIM	-1.97842	1.17E-06
VIM-AS1	-0.89161	0.006011
VIP	2.400511	0.000153
VLDLR	-1.16116	0.00427
VMA21	0.477857	0.000709
VMAC	-0.74075	0.03392
VMP1	0.35582	0.017257
VN1R51P	-2.94024	0.032557
VN1R83P	-1.02834	0.005379
VNN1	2.566437	4.55E-09
VNN2	2.149342	0.010189
VOPP1	0.424613	0.0372
VPS13C	-0.40499	0.002511
VPS13D	-0.1821	0.037723
VPS26A	0.320546	0.010419
VPS29	0.326846	0.033477
VPS33A	0.416307	0.004126
VPS37A	0.257795	0.040388
VPS37B	0.66359	0.001783
VPS39	-0.2967	2.53E-05
VPS4B	0.272058	0.031001
VPS53	-0.37504	1.78E-05
VPS9D1	-0.62651	0.045268
VPS9D1-AS	0.799149	0.010939
VRK1	0.71826	0.000226
VRK2	0.424985	0.003233
VSIG10	-0.27373	0.008015
VSIG10L	-0.75443	0.001272
VSNL1	-1.62283	0.038138
VSTM2L	-1.65943	0.010678
VTCN1	-0.86121	0.004164
VTI1A	0.236133	0.034883

VTN	-2.96254	0.002396
VWA2	-1.91898	0.000395
VWA7	-0.78468	0.002673
VWA8	0.270468	0.045848
WAC-AS1	-0.65459	2.67E-11
WAPL	0.313363	0.011757
WARS2	0.352942	0.017816
WARS2-IT1	-0.44208	0.048305
WASF1	-1.07938	2.85E-23
WASHC2C	-0.22168	0.004019
WASHC3	-0.32999	0.024612
WASL	0.342557	0.002306
WBP1	-1.40109	8.35E-05
WBP1L	-0.3652	0.000609
WBP2	-0.33728	0.048674
WBSCR22	0.372842	0.000188
WDCP	0.489085	0.022695
WDFY1	-0.34992	0.002032
WDFY3	-0.49267	1.11E-06
WDHD1	0.373775	0.023658
WDR1	-0.50264	1.85E-06
WDR12	0.710408	1.46E-06
WDR25	-0.43867	0.010995
WDR26	0.189562	0.029969
WDR3	0.832892	4.23E-07
WDR36	0.481092	0.001374
WDR4	0.507341	0.002353
WDR41	-0.44238	0.001454
WDR43	0.791827	1.51E-07
WDR44	-0.34844	0.002706
WDR46	0.319818	0.007479
WDR5	0.270572	0.000234
WDR53	0.290322	0.021725
WDR55	0.435114	9.08E-06
WDR60	-0.33461	0.002859
WDR63	-1.08554	0.011944
WDR66	-0.74804	2.34E-08
WDR72	0.599437	3.71E-05
WDR73	0.237654	0.013189
WDR74	0.587985	0.000145
WDR75	0.597173	0.000343
WDR77	0.464318	0.002602
WDR78	-0.63604	0.027945
WDR89	0.708859	1.70E-05
WDR91	-0.8378	4.01E-07
WDTC1	-0.5626	0.007789
WEE2-AS1	-1.26251	0.049046

WFDC2	1.038161	8.55E-05
WFDC3	0.662128	0.008331
WFIKKN1	-1.49827	0.014025
WFS1	-1.03923	2.09E-09
WHAMMP:	-0.61198	0.000344
WI2-1896C	-0.75745	0.032869
WI2-85898	-3.38853	0.008921
WIPF2	-0.26006	0.003936
WIPI1	-0.72779	5.51E-06
WISP2	-1.46401	0.003033
WNK1	-0.17945	0.044285
WNT10A	-0.96177	6.48E-06
WNT11	-2.65877	1.11E-06
WNT16	1.503471	5.48E-05
WNT2B	-0.41516	0.013021
WNT4	-1.42701	1.22E-09
WNT5A	-2.10124	4.09E-11
WNT5A-AS	-3.15501	6.32E-07
WNT5B	-1.84296	2.74E-09
WNT6	-4.85278	2.45E-11
WNT7B	-0.88881	6.89E-05
WRAP73	0.509521	1.15E-05
WRN	0.392696	0.006737
WRNIP1	0.599021	3.85E-10
WSCD1	3.565967	1.62E-38
WTIP	-1.02411	0.000401
WWC1	-0.49386	1.25E-09
WWC2	-0.24404	0.028152
WWC3	-0.51646	3.10E-09
WWTR1	0.371411	4.69E-05
XIRP2	4.564553	1.14E-05
XKRX	1.374144	5.42E-08
XPA	0.57916	8.20E-05
XPC	-0.50118	0.000409
XPNPEP1	0.337955	0.002684
XPO4	0.541977	0.000595
XPO5	0.366616	0.000851
XPO6	0.207433	0.027503
XPO7	0.25998	0.026276
XPR1	-0.53348	6.93E-09
XRCC1	-0.51178	0.003501
XRCC2	0.560301	0.00294
XRCC5	0.369658	0.00386
XRCC6	0.289194	0.024749
XRRA1	-0.49384	0.000283
XXbac-B46:	-0.69318	0.000111
XXbac-BPG	-0.5787	0.014026

XXbac-BPG	1.306357	0.03305
XXbac-BPG	-0.41106	0.007306
XX-C00717	-2.23798	0.019816
XX-C2158C	-1.30703	0.02204
XXYLT1	-0.38493	0.005353
XYLB	0.534277	4.78E-05
XYLT1	-1.23647	2.83E-06
YARS2	0.268007	0.02073
YBX1	0.508361	3.80E-10
YBX1P4	2.83757	0.03942
YBX3	0.299818	0.000462
YEATS2	-0.23522	0.015903
YEATS4	0.318014	0.039231
YIF1B	-0.63084	0.011778
YIPF3	-0.44032	0.004546
YIPF5	-0.42883	0.000356
YIPF6	0.324017	0.010068
YOD1	0.940645	1.75E-08
YPEL1	-2.34597	0.000527
YPEL2	-1.20314	6.43E-05
YPEL3	-0.75102	0.005943
YPEL5	-0.44983	0.001774
YRDC	0.70847	6.37E-08
YTHDF1	-0.44603	0.000126
YTHDF2	0.386416	0.002022
YWHAZ	0.431135	0.012102
ZADH2	0.329106	0.000538
ZBED1	-0.87298	5.38E-09
ZBED3	-0.94054	0.000195
ZBTB1	-0.25386	0.007118
ZBTB10	-0.90971	0.000259
ZBTB11	0.32769	0.011427
ZBTB12	-0.44365	0.014475
ZBTB18	-0.62992	0.000355
ZBTB2	0.310524	0.013956
ZBTB22	-0.77292	0.014911
ZBTB24	0.25742	0.033468
ZBTB4	-0.85705	0.001275
ZBTB43	0.378262	0.000195
ZBTB45P1	1.323741	0.000653
ZBTB46	-0.69669	2.17E-05
ZBTB5	-0.34602	0.002073
ZBTB6	0.318263	0.032069
ZBTB7A	0.491877	0.020557
ZBTB8A	-0.49854	1.89E-07
ZBTB9	0.18659	0.039373
ZC2HC1A	-0.53505	0.000795

ZC3H12C	0.81261	5.28E-06
ZC3H13	0.188156	0.042879
ZC3H15	0.634968	2.10E-05
ZC3H18	0.300556	0.042168
ZC3H8	0.466648	0.000635
ZCCHC10	0.319727	0.022362
ZCCHC14	-0.35023	0.000947
ZCCHC17	0.296212	0.001378
ZCCHC18	-2.06702	0.002393
ZCCHC24	-0.76621	0.010053
ZCCHC4	0.511264	8.26E-05
ZCCHC6	0.414893	0.000294
ZCCHC9	0.699789	3.99E-07
ZDHHC1	-1.72456	1.67E-08
ZDHHC13	0.530685	0.000879
ZDHHC16	-0.24183	0.007857
ZDHHC17	-0.67587	2.03E-07
ZDHHC22	-2.83156	0.027116
ZDHHC23	0.565187	0.001786
ZDHHC6	0.555095	0.000191
ZDHHC7	-0.4398	1.18E-06
ZDHHC8P1	-2.38841	6.67E-10
ZDHHC9	0.161109	0.0245
ZEB1	0.921907	0.000659
ZER1	-0.57853	0.001834
ZFAND6	0.382664	0.01776
ZFAS1	0.511979	0.003223
ZFHX3	-0.52486	2.82E-05
ZFP14	-0.68308	4.07E-05
ZFP36L1	-0.899	8.31E-08
ZFP36L2	1.3326	6.09E-18
ZFP37	-0.36909	0.014993
ZFP41	-0.41037	0.041093
ZFP64	-0.21816	0.007635
ZFP90	-0.69662	2.95E-06
ZFPM1	-0.96469	0.00325
ZFR	0.311694	0.011497
ZFX-AS1	-3.03769	0.043664
ZFYVE1	-0.63821	7.75E-06
ZFYVE26	-0.2234	0.046563
ZHX2	-0.53919	2.63E-05
ZHX3	-0.38251	0.012752
ZIC2	0.406961	0.002652
ZIC5	0.583917	3.52E-05
ZIK1	-1.11359	0.023733
ZKSCAN1	-0.3047	0.001675
ZKSCAN3	-0.36859	0.018945

ZKSCAN4	-0.28694	0.040667
ZMAT4	1.891749	0.001278
ZMIZ1	-1.1068	3.24E-06
ZMPSTE24	0.562078	0.000356
ZMYM3	-0.66582	7.45E-05
ZMYND10	-1.15744	0.006445
ZMYND8	0.200026	0.040069
ZNF101	0.330646	0.03057
ZNF117	-0.52848	2.28E-08
ZNF121	0.793511	4.68E-07
ZNF134	-0.39993	0.000935
ZNF135	-0.71776	4.49E-08
ZNF141	0.321742	0.02404
ZNF143	0.526992	0.001789
ZNF165	0.682037	0.000863
ZNF169	0.641367	0.00042
ZNF18	-0.35815	0.008649
ZNF180	0.28467	0.021068
ZNF181	0.259195	0.039348
ZNF184	-0.55544	0.003098
ZNF185	0.397973	0.000399
ZNF195	0.317114	0.00904
ZNF20	-0.4584	0.015024
ZNF202	0.16237	0.032519
ZNF207	0.356079	0.008668
ZNF211	-0.59524	2.09E-05
ZNF213-AS	-0.31032	0.023713
ZNF215	1.07588	0.014828
ZNF219	-0.85491	0.032149
ZNF225	-0.31364	0.029587
ZNF230	-0.31477	0.021424
ZNF232	0.262614	0.02567
ZNF239	0.835497	2.63E-11
ZNF248	-0.44657	0.007523
ZNF250	-0.70399	0.000157
ZNF251	-0.70063	0.007521
ZNF260	-0.31416	0.017396
ZNF267	0.799379	6.49E-08
ZNF275	-0.21923	0.012404
ZNF277	0.392864	0.01242
ZNF280C	0.423792	0.033157
ZNF283	0.331072	0.009445
ZNF286A	-0.39726	0.001249
ZNF287	-0.50673	0.033485
ZNF296	0.720983	0.015778
ZNF3	-0.31137	0.000998
ZNF30	0.675973	9.01E-05

ZNF304	-0.23075	0.014055
ZNF318	-0.43812	3.84E-11
ZNF319	-0.37853	0.047033
ZNF32	-0.28945	0.019681
ZNF322	-0.25944	0.008816
ZNF324	-0.5426	0.019018
ZNF326	0.467507	0.009429
ZNF329	-0.31722	0.003316
ZNF330	0.520337	7.16E-05
ZNF331	0.218458	0.032601
ZNF337-AS	0.344416	0.012737
ZNF33A	-0.30643	0.00504
ZNF341	-0.33709	0.048298
ZNF343	0.433418	2.42E-05
ZNF346	-0.32473	0.008191
ZNF347	-0.50307	1.33E-07
ZNF354A	-0.3974	0.01319
ZNF358	-1.7231	4.28E-07
ZNF362	-1.81651	3.84E-10
ZNF365	1.760634	1.81E-06
ZNF37A	0.244453	0.022973
ZNF37BP	0.290082	0.003931
ZNF382	-0.41923	0.026336
ZNF385A	-0.85409	0.041495
ZNF385B	-1.15084	8.93E-06
ZNF391	-0.4631	0.001161
ZNF394	0.286392	0.017827
ZNF395	-0.31682	0.042249
ZNF396	-1.08246	0.000599
ZNF415	-1.44545	0.006476
ZNF416	-0.26593	0.023518
ZNF419	-0.36734	0.023275
ZNF425	-0.42527	0.038977
ZNF436	-0.56792	0.000104
ZNF439	-0.60651	5.93E-07
ZNF440	-0.39375	6.24E-06
ZNF443	-0.31179	0.004121
ZNF446	-0.98557	0.006374
ZNF449	-0.33485	0.017801
ZNF460	0.551397	0.001538
ZNF462	-0.95005	2.32E-10
ZNF467	-2.12961	0.001518
ZNF469	-2.34365	9.43E-05
ZNF470	-0.24425	0.031668
ZNF471	-0.39402	0.002654
ZNF473	0.196573	0.042728
ZNF485	1.075523	5.32E-13

ZNF488	-1.63865	3.20E-14
ZNF496	-0.34877	0.030958
ZNF497	-0.84877	0.008341
ZNF503	-0.74957	0.008687
ZNF516	-1.24905	4.83E-13
ZNF526	-0.3683	0.012591
ZNF527	0.284462	0.025725
ZNF528	-0.57416	1.32E-11
ZNF528-AS	-0.74055	8.12E-06
ZNF529-AS	-0.33455	0.015098
ZNF532	-0.50206	4.80E-10
ZNF541	-3.22414	0.009468
ZNF544	0.333158	0.023222
ZNF546	0.537201	0.045134
ZNF548	-0.31303	0.013232
ZNF551	0.282101	0.011051
ZNF555	-0.71772	0.000508
ZNF558	0.397984	0.002673
ZNF559-ZN	-0.37276	0.023648
ZNF561	-0.46713	7.11E-06
ZNF561-AS	-0.93618	6.69E-13
ZNF562	0.640788	5.62E-09
ZNF566	0.305511	0.010122
ZNF569	-0.2927	0.045587
ZNF570	-0.45377	0.001424
ZNF575	-0.66987	0.027998
ZNF585A	-0.20769	0.02483
ZNF587B	0.633704	0.002121
ZNF589	0.594315	6.52E-05
ZNF593	0.477546	0.04312
ZNF595	0.53681	0.002154
ZNF599	-0.57657	0.000559
ZNF606	-0.415	0.001355
ZNF607	-0.21125	0.028722
ZNF608	-2.78611	2.33E-39
ZNF609	-0.27758	0.028484
ZNF610	-0.81147	0.025011
ZNF613	-0.36715	0.001894
ZNF618	-1.34687	7.66E-20
ZNF620	0.372998	0.024739
ZNF625-ZN	-0.33743	0.008671
ZNF627	-0.48367	0.007192
ZNF629	-0.76447	0.001915
ZNF639	0.260904	0.040963
ZNF652	-0.39116	2.30E-05
ZNF665	-0.60705	8.41E-05
ZNF671	-0.50327	0.006648

ZNF674-AS	0.364405	0.033273
ZNF688	-0.51252	0.029664
ZNF692	0.767594	0.000396
ZNF695	0.371966	0.006443
ZNF697	0.674842	1.25E-09
ZNF699	-0.64116	1.95E-05
ZNF7	0.195301	0.013735
ZNF702P	-0.86436	3.66E-08
ZNF703	-1.40536	8.13E-05
ZNF704	-1.34966	2.53E-07
ZNF705E	-2.19242	0.045602
ZNF706	0.469163	0.002212
ZNF708	-0.52896	0.001003
ZNF709	-0.7805	0.043797
ZNF71	-0.66624	0.00033
ZNF710	0.503407	0.03484
ZNF713	-0.3944	0.001756
ZNF721	0.480659	0.000509
ZNF724	0.371482	0.031776
ZNF736	-0.22564	0.001093
ZNF736P9Y	-0.91095	2.37E-06
ZNF737	-0.55164	6.45E-05
ZNF738	-0.47095	0.000213
ZNF747	-0.40576	0.03977
ZNF750	2.141715	1.24E-05
ZNF76	-0.41014	0.030071
ZNF761	0.242859	0.041633
ZNF763	-0.57319	0.01931
ZNF767P	-0.48809	0.00129
ZNF770	0.53185	0.000143
ZNF771	-0.47693	0.029204
ZNF772	-0.23376	0.046033
ZNF773	-0.37928	0.009098
ZNF774	-0.37731	0.012538
ZNF775	-1.11404	0.007719
ZNF776	-0.36172	0.000621
ZNF778	0.528309	0.005201
ZNF789	-0.33229	0.002387
ZNF790	0.561742	0.000279
ZNF793	-0.40412	0.000299
ZNF793-AS	-0.78623	7.99E-05
ZNF80	1.733208	0.044906
ZNF800	0.465968	0.000463
ZNF812P	-1.79753	0.01855
ZNF821	-1.89588	8.58E-09
ZNF827	-0.90411	2.60E-17
ZNF836	-0.81699	1.21E-09

ZNF84	-0.32899	0.009515
ZNF846	-0.96714	6.39E-05
ZNF850	-0.5194	0.000385
ZNF853	-0.62173	0.003846
ZNF860	0.699929	0.046682
ZNF865	-0.765	0.021606
ZNF878	0.887515	1.62E-05
ZNF90P1	1.036699	0.019016
ZNF91	-0.24308	0.04199
ZNF92	0.531709	0.000101
ZNF92P3	-2.16432	0.00369
ZNF93	-0.51287	0.000132
ZNHIT6	0.553826	0.00049
ZNRD1	0.275016	0.025989
ZNRF1	-0.43816	0.000177
ZPR1	0.477868	1.42E-06
ZRANB1	-0.60414	7.11E-05
ZSCAN12P1	0.395958	0.017607
ZSCAN2	-0.81985	8.50E-06
ZSCAN23	-0.451	0.005257
ZSCAN31	-1.3904	3.19E-16
ZSCAN4	-0.92441	0.031677
ZUFSP	0.448792	0.0017
ZWINT	0.436305	3.89E-05
ZXDB	0.38603	0.009964
ZYX	-0.70143	0.007625

Supplementary Table 5 - SCC25 differentially expressed genes between cetuximab and PBS.

Genes	log2FoldCh	pvalue
AAAS	0.206396	0.011988
AADAC	0.619799	0.000583
AADACP1	0.577236	0.000743
AAK1	-0.30949	9.08E-05
AAMP	0.28913	4.47E-06
AAR2	0.157147	0.032178
AARS2	0.204389	0.037484
AARSD1	0.268212	5.23E-05
AATBC	-0.87642	0.001856
AATF	0.413164	9.24E-18
ABC7-4240	-0.66022	0.000542
ABCA13	0.314583	0.001919
ABCA5	-0.39625	0.000243
ABCA7	-0.42198	0.023204
ABCB10P4	-0.8964	0.000599
ABCB6	-0.36045	0.032022
ABCC1	0.26157	3.49E-05
ABCC3	-0.31405	0.000533
ABCC4	0.189735	0.000689
ABCC6	-0.59541	0.004841
ABCC9	0.2306	0.032643
ABCE1	0.283118	0.000207
ABCF1	0.179991	0.001446
ABCF2	0.193768	0.001011
ABHD10	0.171474	0.039893
ABHD15	-0.42369	0.000227
ABHD17B	-0.20072	0.002645
ABHD17C	0.22863	0.008926
ABHD2	0.168455	0.010303
ABHD4	-0.40824	6.33E-06
ABHD6	-0.25962	0.021904
ABI2	0.192862	0.043282
ABI3BP	-0.42256	0.000995
ABL2	0.283326	0.022797
ABLIM2	0.214235	0.040311
ABLIM3	-0.17753	0.001968
ABO	-0.21985	0.002927
ABR	0.224153	0.030557
ABT1	0.210547	0.018118
ABTB2	0.370665	0.001686
AC002398.	-0.59001	0.018062
AC002480.	-0.43398	0.01241
AC002550.	-1.0171	0.023409
AC003092.	0.96803	0.021092

AC003991.	-0.81086	0.00551
AC004870.	-0.89696	0.013507
AC005154.	-0.3082	7.99E-05
AC005154.	-1.23527	0.019045
AC005523.	0.275479	0.002497
AC005537.	-0.51236	0.024073
AC005618.	-0.77152	0.003875
AC005682.	0.299162	0.006003
AC006014.	-0.62608	0.025769
AC006116.	-0.76269	0.012543
AC006128.	0.544396	0.049204
AC006372.	-3.02082	0.0337
AC006509.	-1.20554	0.016664
AC007285.	-0.61757	0.021708
AC007326.	-0.44166	0.019819
AC007383.	-0.42678	0.016529
AC007743.	-0.44371	0.005455
AC009502.	0.439453	0.022332
AC009948.	-0.34136	0.000154
AC010731.	-1.36632	0.029667
AC010970.	0.282687	0.002884
AC011286.	-1.3696	0.027086
AC011294.	0.884193	0.044009
AC011330.	-0.38001	0.04293
AC011530.	2.779948	0.035291
AC011738.	-0.69807	0.005078
AC012146.	0.412796	0.017797
AC012501.	-0.81546	0.042896
AC015849.	2.990632	0.024478
AC016745.	-1.27014	0.001558
AC016999.	1.024976	0.039633
AC017116.	-2.75949	0.03162
AC020571.	0.972398	0.043507
AC020956.	-1.46552	0.016534
AC024560.	-0.19001	0.028695
AC024937.	-0.54338	0.034097
AC025171.	-0.5835	0.000185
AC034220.	-0.46933	0.015626
AC053503.	-3.63315	0.007078
AC058791.	0.872763	2.29E-05
AC063976.	-0.87519	0.000194
AC068134.	-0.86106	0.00127
AC068134.	-2.58542	0.00246
AC073115.	-3.50053	0.020821
AC073333.	0.964604	0.028512
AC074183.	0.61887	0.01325
AC074286.	-0.35916	0.015549

AC078883.	1.002262	0.03182
AC079610.	-0.49647	0.029209
AC084809.	-0.88635	0.049713
AC091729.	-0.24589	0.028963
AC092066.	-0.43116	0.00078
AC092641.	1.949835	0.048413
AC093162.	-0.45011	0.017049
AC093732.	-1.35719	0.048584
AC093838.	0.361818	0.000345
AC093899.	-2.91085	0.040519
AC098614.	-0.39436	0.013057
AC104389.	0.724252	0.007562
AC105760.	-0.31525	0.042976
AC108142.	0.420206	0.008414
AC108868.	3.001787	0.024158
AC109333.	1.747231	0.011398
AC113189.	0.467255	0.009554
AC114730.	1.436695	0.006981
AC116366.	-0.65004	0.010873
AC124789.	0.801315	0.001171
AC133785.	0.896389	0.004673
AC135048.	-1.04557	6.85E-06
AC137932.	0.801259	0.005604
AC141586.	0.233142	0.001695
AC145124.	-0.60165	0.014346
AC145343.	-0.64939	0.018961
AC159540.	-0.49071	5.45E-06
ACADM	-0.24643	0.010867
ACADSB	-0.40753	1.04E-05
ACAN	0.930348	0.046279
ACAT2	0.19221	0.019114
ACBD4	-0.62784	0.000118
ACBD5	-0.14244	0.040784
ACCS	-0.49774	6.15E-08
ACD	0.441856	0.006534
ACE2	-0.99496	0.005034
ACIN1	0.163746	0.00067
ACKR3	-1.14047	3.54E-15
ACLY	0.185685	0.000143
ACOT1	-0.44423	1.61E-05
ACOT13	-0.24046	0.000492
ACOT4	-0.45116	0.001692
ACOT7	0.569718	1.90E-07
ACOX1	-0.11313	0.033888
ACOXL	0.628251	0.000182
ACP5	-0.49178	0.007249
ACSBG1	-0.58876	0.022396

ACSF2	-0.25767	0.001876
ACSL1	-0.38828	8.71E-08
ACSL3	-0.15518	0.009999
ACSL5	-0.24208	0.000118
ACTA1	-2.74511	0.003851
ACTA2	-1.19024	8.15E-07
ACTBL2	-0.49912	0.003937
ACTG2	-1.45141	1.37E-12
ACTL6A	0.190204	0.002422
ACTR10	-0.14687	0.019586
ACVR1B	0.162145	0.001402
ACY1	0.303738	0.001976
ACYP2	-0.36367	0.000235
ADAL	-0.22089	0.02118
ADAM12	-0.27395	0.012808
ADAM15	0.411276	0.000685
ADAM19	0.822072	1.69E-09
ADAM23	-0.4737	8.95E-07
ADAM28	-0.42777	0.00128
ADAM32	-0.20754	0.034562
ADAM8	1.309405	7.47E-14
ADAMTS12	0.252782	0.020708
ADAMTS15	-0.2001	0.024363
ADAMTS17	0.358941	0.036027
ADAMTS20	-0.23785	0.044185
ADAMTS6	0.660353	1.63E-09
ADAMTS9	-0.87039	7.29E-08
ADAMTSL1	0.633641	1.15E-05
ADAMTSL3	-0.50389	0.019551
ADAT2	0.254804	0.00889
ADCY1	0.374984	0.001374
ADCY3	0.366982	5.50E-05
ADCY4	-1.00495	0.002516
ADCY5	-0.8921	0.027315
ADCY6	-0.64385	6.41E-08
ADCY7	0.423193	3.08E-05
ADCY9	-0.22085	0.018147
ADD2	0.326256	0.000315
ADGRA2	-1.14287	2.71E-08
ADGRE5	0.308607	0.002669
ADGRF1	0.828952	0.000253
ADGRG6	0.409602	3.79E-05
ADGRL3	-0.54872	0.038537
ADGRV1	-0.46847	0.01411
ADH5	-0.12201	0.033609
ADH7	-2.34565	1.43E-12
ADHFE1	-0.98732	9.36E-07

ADM	-0.46471	0.00026
ADORA2B	0.587395	3.45E-15
ADPRH	-0.61478	0.000799
ADRA1B	0.602201	2.07E-06
ADRA1D	1.420734	0.000202
ADRB1	0.543083	0.002199
ADRB2	0.238181	0.010894
ADRM1	0.477278	0.001063
ADSL	0.244867	0.000548
ADSSL1	-0.44754	0.000147
ADTRP	0.442134	0.001624
AEBP1	0.851407	2.61E-07
AEN	0.557023	1.11E-16
AF003625.:	0.889021	1.67E-06
AF131217.:	1.263499	0.016876
AFAP1	0.347676	4.48E-05
AFAP1-AS1	0.164719	0.046682
AFAP1L1	0.349537	8.36E-05
AFF1	-0.26601	0.008341
AFG3L2	0.098302	0.014275
AGA	-0.36477	0.001354
AGAP10P	-0.79703	0.006633
AGBL5	0.190712	0.044837
AGGF1P1	-2.21318	0.015008
AGK	0.174442	0.013215
AGL	-0.28316	4.04E-05
AGMAT	0.503766	3.99E-06
AGO2	0.431734	2.85E-11
AGO4	-0.60574	1.33E-11
AGPAT3	-0.44182	0.000113
AGPAT4	-0.21155	0.000895
AGPAT5	0.362048	9.88E-06
AGPS	-0.18877	0.026456
AGR2	0.842853	5.22E-06
AHCTF1	0.233426	0.001575
AHCY	0.402688	1.99E-06
AHCYL2	-0.38046	7.91E-05
AHSA1	0.280527	1.53E-05
AIF1L	0.345331	1.39E-06
AIFM3	-0.74703	0.004957
AIM1	0.163075	0.003813
AIMP2	0.29582	0.00019
AIP	0.201008	0.039561
AJAP1	0.976149	3.47E-11
AK2	0.289244	2.64E-07
AK4	0.120785	0.033952
AKAP1	0.318198	1.15E-12

AKAP12	1.211928	4.15E-45
AKAP2	0.413284	6.17E-12
AKAP6	-0.60698	9.57E-05
AKAP8L	0.172693	0.03536
AKAP9	-0.27853	0.019767
AKR1B1	0.213174	0.009724
AKR1B10	-0.61024	0.02455
AKR1B15	-0.47376	0.037807
AKR1C2	-0.78658	0.00012
AKR1C3	-0.65716	0.000123
AKR7A3	0.59156	0.04812
AKT2	0.24962	0.016287
AKT3	-0.17868	0.049564
AKTIP	-0.37946	0.000135
AL022344.[illegible]	-0.68116	0.005522
AL050303.[illegible]	-2.65316	0.017359
ALAD	-0.24094	0.000864
ALDH18A1	0.099126	0.018053
ALDH1B1	-0.39432	1.57E-09
ALDH3A1	-1.15097	5.49E-07
ALDH3A2	-0.17389	0.028915
ALDH3B1	-0.42204	0.012697
ALDH3B2	-2.33628	2.81E-05
ALDH5A1	-0.56877	8.36E-09
ALDH6A1	-0.30255	0.001089
ALDH7A1	-0.17379	0.004531
ALG1	0.264863	0.010669
ALG3	0.274164	0.013267
ALKBH2	0.185961	0.048946
ALKBH5	0.121251	0.047949
ALOX12	0.792294	1.49E-11
ALOXE3	1.072208	2.41E-09
ALPK1	-0.58599	1.94E-10
ALPP	-0.98296	0.000647
ALX4	-0.39567	0.023348
ALYREF	0.341379	0.001418
AMACR	-0.32656	7.29E-06
AMBRA1	0.180816	0.011469
AMIGO1	-0.48519	0.001427
AMMECR1	0.303374	1.84E-05
AMOT	-1.22747	0.001918
AMOTL1	0.27557	5.69E-09
AMOTL2	0.224637	0.020857
AMPD2	0.357144	0.001587
AMTN	0.284755	0.008609
ANAPC1	0.154708	0.008082
ANAPC5	0.104213	0.021097

ANAPC7	0.139639	0.004402
ANG	-0.34015	0.011028
ANGEL1	0.140885	0.024042
ANGPT1	-0.86808	4.15E-06
ANGPT2	1.065721	0.000786
ANGPTL2	0.966293	9.90E-05
ANGPTL4	0.545135	7.87E-05
ANGPTL7	-2.06744	0.024992
ANGPTL8	1.69043	0.034662
ANK3	-0.32535	0.00055
ANKEF1	-0.33229	1.91E-06
ANKH	-0.17768	0.036671
ANKMY1	0.268069	0.004498
ANKMY2	-0.19821	0.00733
ANKRA2	-0.2687	0.023586
ANKRD10	0.462312	1.60E-07
ANKRD10-I	0.537119	3.77E-06
ANKRD11	0.215729	0.032416
ANKRD13A	-0.1605	0.047331
ANKRD13B	0.533316	0.000252
ANKRD13C	-0.13225	0.039055
ANKRD18C	-1.18522	0.009259
ANKRD20A	-0.95364	3.91E-06
ANKRD22	0.6968	0.047561
ANKRD29	0.17098	0.037958
ANKRD33B	0.421994	4.94E-05
ANKRD34B	-1.8458	0.009926
ANKRD65	-0.67908	0.002512
ANKRD9	-0.48694	0.000824
ANKS6	0.355118	2.28E-06
ANKZF1	0.15003	0.019393
ANLN	0.219538	0.003484
ANO1	0.799759	4.26E-15
ANO1-AS2	1.872609	0.00473
ANO7	0.526755	0.033795
ANOS1	-0.69676	6.61E-09
ANP32A	0.178834	0.032135
ANP32B	0.233902	0.003925
ANPEP	0.79455	2.04E-13
ANTXR1	-0.36065	1.80E-12
ANTXR2	0.767898	3.28E-08
ANXA1	0.170413	0.019845
ANXA10	1.720885	2.39E-14
ANXA13	-1.22572	0.000371
ANXA3	-0.51047	2.04E-09
ANXA4	-0.23082	0.006391
ANXA5	0.289891	4.57E-05

ANXA6	0.440996	9.71E-05
ANXA8	-0.16202	0.037298
ANXA8L1	-0.18505	0.006094
ANXA9	-0.47582	0.00491
AOX1	0.306074	0.004419
AP000275.	-3.12442	0.013272
AP000350.	-1.62877	0.031601
AP000350.	-0.80977	0.000496
AP000593.	1.106718	0.000334
AP000695.	-0.39268	0.019897
AP000892.	-0.93587	1.40E-08
AP001258.	-0.41458	0.003858
AP001372.	-0.39559	0.0108
AP002954.	-0.70844	0.019777
AP003068.	-0.27658	0.015162
AP003068.	0.809458	0.029252
AP004782.	0.844377	0.033169
AP1M2	-0.19986	0.021333
AP1S2	0.221962	0.023624
AP2B1	0.105758	0.018998
AP2S1	0.244684	0.001004
AP3B2	-0.50929	1.38E-07
AP4B1	0.156117	0.015851
AP4M1	0.247438	0.008804
AP5M1	-0.29172	8.75E-05
APBA2	0.56048	0.005914
APCDD1	-1.69581	0.000359
APCDD1L	0.338107	0.020219
APCDD1L-A	0.344808	0.002547
APEH	0.171097	0.045215
APEX1	0.228796	0.000288
APEX2	0.326038	0.000499
APH1B	-0.36666	0.001207
APIP	0.188202	0.030941
APITD1-CO	0.616881	0.003284
APLF	-0.31055	0.014122
APLN	0.526609	0.000188
APLP1	-0.44306	0.030362
APOB	-1.30499	0.021509
APOBEC3B	0.424554	0.000858
APOBEC3D	-0.32077	0.004994
APOBEC3F	-0.31863	0.000921
APOBR	-1.29443	0.00919
APOE	-0.48108	0.028244
APOL2	-0.39024	0.007932
APOL3	-0.43857	0.000333
APOL6	-0.37981	0.030762

APOM	-0.33012	0.029192
APOO	0.202058	0.023452
APOOL	-0.20525	0.009109
APOPT1	0.115502	0.024281
APP	-0.17378	0.013643
APPBP2	-0.30108	0.0002
APRT	0.378589	0.013738
AQP1	0.467016	0.015826
AQP10	-0.44581	0.047737
AQP11	-0.49756	0.041621
AQP7	-0.59295	0.014225
ARAF	0.236622	0.037336
ARAP1-AS2	0.725963	0.026441
ARAP3	0.511264	8.27E-07
ARC	0.985949	1.98E-06
AREG	1.121364	5.09E-31
ARF1	0.203444	0.019623
ARF6	0.123371	0.010175
ARFGAP2	0.091439	0.032971
ARFIP2	0.127481	0.027496
ARFRP1	0.343096	0.000743
ARG2	0.85027	1.70E-14
ARHGAP11	0.260198	0.020252
ARHGAP17	0.145971	0.018644
ARHGAP18	0.412463	0.000587
ARHGAP19	0.286904	0.002501
ARHGAP22	-0.30702	0.018172
ARHGAP23	0.369108	0.006961
ARHGAP24	0.244043	0.00788
ARHGAP25	0.500111	0.000184
ARHGAP26	-0.53371	1.53E-08
ARHGAP27	-0.37627	0.010475
ARHGAP29	0.460688	6.57E-08
ARHGAP5	-0.2816	0.008245
ARHGDIA	0.321311	0.043925
ARHGDIB	0.24408	0.004515
ARHGEF1	0.524666	7.98E-05
ARHGEF10I	-0.70405	4.19E-06
ARHGEF18	0.281807	0.046667
ARHGEF2	0.605296	1.39E-08
ARHGEF34I	-0.20254	0.014751
ARHGEF37	-0.79658	0.000289
ARHGEF6	-0.22838	0.036032
ARID3B	0.822731	4.84E-14
ARID4A	-0.25778	0.010991
ARID5A	0.304313	0.038667
ARID5B	-0.21946	0.049575

ARIH2	0.122138	0.012292
ARL11	-0.65315	0.001949
ARL14	0.425762	0.01009
ARL15	-0.32271	0.024371
ARL2	0.427454	0.001363
ARL6IP5	-0.32178	6.13E-11
ARL9	0.402034	0.027202
ARMC6	0.437487	0.003269
ARMCX3	-0.3364	2.60E-06
ARMCX4	-0.27683	0.00889
ARNTL	0.395238	1.80E-05
ARNTL2	0.28635	0.000423
ARPC1A	0.109314	0.021477
ARPC1B	0.453461	0.000969
ARPC5	-0.26992	1.18E-05
ARPIN	-0.37508	1.23E-07
ARRDC4	0.319032	0.011089
ARSB	-0.17354	0.019483
ARSD	-0.58016	1.08E-09
ARSG	-0.62088	1.01E-05
ARSJ	0.161681	0.041817
ARV1	-0.274	0.000524
AS3MT	-0.43545	0.012187
ASAH1	-0.64785	1.41E-14
ASAP1	0.255868	5.78E-06
ASAP3	-0.17302	0.046188
ASB1	0.396443	9.94E-10
ASB6	0.264184	0.005884
ASF1B	0.260456	0.022787
ASH1L	-0.16478	0.008839
ASIC2	0.824579	0.008193
ASL	0.220575	0.047208
ASPH	-0.13003	0.032474
ASRGL1	0.67699	0.001006
ASS1	-0.38562	0.048251
ASTL	1.636589	0.047011
ASXL1	0.200409	0.000144
ATAD2	0.16149	0.010949
ATAD2B	-0.1903	0.008687
ATAD3A	0.548576	0.001723
ATAD3B	0.579987	4.05E-05
ATAD5	0.185413	0.02144
ATF1	0.217671	0.005246
ATF4	0.125953	0.043217
ATF7IP2	-0.45165	0.000478
ATG10	-0.24801	0.022464
ATG101	0.281288	0.008405

ATG16L1	0.23961	0.00015
ATG2B	-0.22344	0.00233
ATG4A	-0.1963	0.037699
ATIC	0.343868	2.25E-12
ATL1	-0.86856	3.19E-07
ATL2	0.217451	0.006017
ATL3	-0.13562	0.046547
ATOH8	-1.65898	1.34E-05
ATP11C	0.171048	0.031245
ATP13A5	-1.82208	0.004702
ATP1A3	0.926323	0.025221
ATP1B1	-0.32987	2.71E-11
ATP1B3	-0.17623	0.0044
ATP2A2	-0.16237	0.001123
ATP5A1	0.124233	0.000303
ATP5B	0.158582	0.000443
ATP5F1	0.121169	0.028142
ATP5G1P6	1.040817	0.016262
ATP5G2	0.195681	0.009119
ATP5J2-PT(	0.314516	0.027282
ATP5L2	2.162291	0.013217
ATP6AP1L	-0.65814	0.034378
ATP6AP2	-0.1923	0.009313
ATP6V0A1	-0.3487	3.09E-07
ATP6V0B	0.222546	0.022118
ATP6V0D2	-0.39398	0.000986
ATP6V1A	-0.38413	2.79E-06
ATP6V1B1-	0.550211	0.03182
ATP6V1C2	-0.44267	0.009688
ATP6V1E1	-0.22612	0.000779
ATP7A	-0.3737	5.84E-05
ATP7B	-0.29212	0.044112
ATP8B1	-0.43007	2.45E-06
ATP9A	-0.16543	0.047585
ATRIP	0.312097	0.010091
ATRN	-0.25886	4.71E-09
ATXN1	-0.32017	6.61E-09
ATXN10	-0.16289	0.00022
ATXN1L	-0.09435	0.030431
ATXN2L	0.217598	0.0256
ATXN7L2	0.504801	0.000376
ATXN7L3	0.154237	0.023218
AUNIP	0.253808	0.000379
AUP1	0.225168	0.008081
AURKA	0.370178	0.001246
AURKAPS1	1.427833	0.011602
AURKB	0.521576	3.56E-06

AUTS2	-0.20245	0.012993
AVPI1	0.352683	0.018969
AXDND1	-0.83483	0.000895
AXIN1	0.401618	0.00268
AXIN2	-1.18078	0.000533
AXL	0.62153	1.06E-06
AZGP1	-2.15219	0.032326
B2M	-0.35611	0.00151
B3GALT4	-0.36669	0.029162
B3GLCT	-0.3044	1.63E-05
B3GNT2	-0.26003	0.0054
B3GNT3	0.638515	1.26E-07
B4GALNT2	-0.95163	1.40E-09
B4GALNT3	0.426388	0.000237
B4GALT1	-0.13139	0.008761
B4GALT2	0.285353	0.03765
B4GALT3	0.256849	0.000258
B4GALT4	-0.17468	0.022943
B9D1	0.24737	0.00518
BACE1	-0.13905	0.027539
BAG6	0.342106	0.00022
BAIAP2L2	0.845597	0.000128
BAIAP3	0.658622	0.009367
BAK1	0.201424	0.003327
BAMBI	0.348143	0.036943
BANF1	0.296669	0.000125
BANK1	-0.79018	0.002271
BAP1	0.21232	0.033598
BATF3	0.828235	0.004774
BAX	0.400908	0.000126
BAZ2B	-0.31346	0.008778
BBOF1	-0.24051	0.025727
BBOX1	-1.93257	0.001267
BBS2	-0.20831	0.001342
BBS5	-0.18163	0.015885
BCAP31	0.140524	0.011084
BCAR1	0.423054	0.044139
BCAR3	0.53943	5.03E-08
BCAS3	-0.46269	6.50E-09
BCAT1	0.345531	0.000589
BCCIP	0.305594	4.11E-05
BCDIN3D	-0.24402	0.00606
BCDIN3D-A	-0.51272	0.028458
BCHE	-0.41843	8.07E-05
BCKDHB	-0.35275	1.15E-06
BCL10	0.495036	1.61E-06
BCL2	0.728646	1.78E-05

BCL2L1	0.305122	0.000539
BCL2L10	-0.42148	0.043354
BCL2L12	0.402787	0.00695
BCL2L14	-1.08287	0.000587
BCL2L2-PAI	0.559288	0.003169
BCL7A	-0.20062	0.042118
BCO1	-1.05573	0.040191
BCR	0.225893	0.02547
BCS1L	0.486639	1.70E-12
BDH2	-0.38005	2.30E-07
BEND3	0.266124	0.036372
BEND3P3	0.327078	0.020215
BEND6	-0.35243	2.01E-05
BEND7	-0.2735	0.020618
BEST1	-0.21426	0.022907
BFSP1	-0.23943	0.01242
BHLHB9	-0.23067	0.025775
BHLHE40	0.284575	0.02871
BHLHE41	-0.58704	8.93E-13
BICDL2	-0.67346	0.020952
BIK	0.647148	0.000296
BIN1	0.270246	0.038863
BIN3	0.181018	0.042731
BIRC5	0.334617	0.000178
BISPR	-0.46212	0.004251
BLM	0.360049	0.000194
BLMH	0.203539	0.001739
BLOC1S6	-0.15061	0.03736
BLVRA	-0.22668	0.013369
BLZF2P	-1.72166	0.026417
BMP6	1.27916	0.000187
BMPR1A	-0.16105	0.033276
BMPR1B	0.263103	0.031767
BMPR2	-0.32199	6.85E-05
BMS1	0.14761	0.032419
BMS1P10	0.398868	0.038739
BMT2	-0.34622	0.004887
BNC1	0.244237	8.16E-07
BNIP1	0.251452	0.002331
BNIP3L	-0.1796	0.023748
BOD1	0.226523	0.00014
BOLA1	0.302425	0.04857
BOLA2	0.406448	8.76E-05
BOLA2B	0.455722	0.000497
BOLA3	0.285429	2.20E-05
BOP1	0.689501	2.98E-05
BORA	0.208512	0.005531

BORCS7	-0.34051	2.23E-05
BPHL	-0.20244	0.00305
BPTF	-0.18567	0.005459
BRCA2	0.237502	0.00025
BRD4	0.239944	0.005738
BRD9	0.220831	0.005681
BRI3BP	0.159169	0.031304
BRIX1	0.36047	2.33E-09
BRPF1	0.258014	0.00459
BRWD1	-0.24495	0.035851
BSDC1	-0.14574	0.033275
BTBD16	-1.16905	0.046884
BTBD19	0.328671	0.026684
BTBD2	0.365687	0.045021
BTBD6	0.198544	0.011844
BTBD7	-0.16318	0.012897
BTBD8	-0.63391	7.31E-06
BTBD9	-0.3	0.004967
BTC	-0.61461	0.009464
BTF3	0.135914	0.015947
BTF3P2	2.483348	1.12E-08
BTG2	-0.32388	1.28E-05
BTN2A2	-0.1904	0.01606
BTN3A1	-0.27273	0.002109
BTN3A2	-0.22986	0.004657
BTN3A3	-0.40073	0.000112
BUB1	0.409931	9.34E-05
BUB1B	0.393731	0.001661
BUB3	0.230101	7.81E-05
BUD13	0.188322	0.002265
BVES	-0.35763	6.35E-07
BX322557.	-0.31779	0.004524
BYSL	0.375377	1.18E-08
BZW1	0.221627	0.00637
BZW1P2	0.467293	7.98E-10
BZW2	0.339595	3.16E-13
C10orf67	-0.39974	0.041666
C10orf99	-1.54594	0.025198
C11orf52	-0.93964	1.27E-06
C11orf54	-0.25005	0.027757
C11orf63	-0.25644	0.004235
C11orf68	0.465126	0.000286
C11orf80	-0.28772	0.009333
C11orf84	0.285847	0.034748
C11orf88	-1.12605	0.00093
C11orf98	0.353341	9.07E-06
C12orf10	0.336484	0.003702

C12orf43	0.192871	0.012973
C12orf49	-0.13243	0.00811
C12orf50	-0.3717	0.003709
C12orf54	-0.46815	0.01004
C12orf56	-0.54555	0.025331
C14orf132	0.241219	0.011577
C14orf166	0.16902	0.002498
C14orf28	-0.48296	4.81E-05
C14orf37	-0.35209	0.023904
C15orf38-A	0.230223	0.044053
C15orf39	0.344781	0.022238
C15orf48	-0.93094	0.02487
C15orf52	-0.28698	0.000766
C15orf59	-0.88829	0.000151
C16orf45	0.273244	0.012768
C16orf52	0.395574	0.000836
C16orf62	-0.16775	0.001207
C16orf74	0.492262	0.000263
C16orf91	0.297465	0.019703
C17orf49	0.368687	0.006499
C17orf51	0.472284	3.27E-08
C17orf53	0.387962	0.002049
C18orf32	-0.16816	0.04056
C19orf24	0.495302	0.022364
C19orf25	0.357093	0.008921
C19orf33	0.418166	0.002165
C19orf48	0.527318	6.95E-06
C19orf53	0.263759	0.002906
C19orf66	-0.39799	0.021338
C1GALT1C1	-0.17639	0.018481
C1orf109	0.211775	0.031892
C1orf112	0.198113	0.01663
C1orf132	-0.6909	8.35E-06
C1orf186	-0.52419	8.11E-06
C1orf198	-0.26566	3.66E-05
C1orf21	-0.49043	5.46E-06
C1orf226	0.264028	0.006218
C1orf43	0.11682	0.011528
C1orf61	0.593782	0.031275
C1orf74	-0.14169	0.020809
C1orf94	1.266317	0.02552
C1QBP	0.458609	2.78E-09
C1QTNF2	-1.16027	0.008409
C1QTNF3	-0.94154	0.012638
C1QTNF3-A	1.828971	0.034487
C1R	-0.97035	1.69E-10
C1RL	-0.64202	5.00E-06

C1S	-1.09885	2.07E-17
C2	-0.28359	0.049949
C20orf194	-0.40545	1.07E-08
C20orf197	0.71967	0.000237
C20orf27	0.613341	0.000818
C21orf59	0.208954	0.003302
C21orf91	-0.23798	0.023612
C22orf23	-0.54831	0.004577
C22orf34	1.70928	0.008257
C2orf27A	-0.36399	0.024025
C2orf54	-1.19035	0.047529
C2orf70	1.300722	0.000949
C3	-1.40036	1.40E-06
C3orf52	0.207658	0.015747
C3orf62	-0.21193	0.021415
C3orf67	0.199207	0.037555
C4BPAP1	3.466297	0.026534
C4orf3	-0.17156	0.01645
C4orf33	-0.44838	2.46E-06
C4orf36	-0.35022	0.004624
C4orf47	-0.75764	0.017256
C5AR2	0.399548	0.048971
C5orf15	-0.24638	0.001245
C5orf17	-0.53669	0.035698
C5orf30	0.373175	0.000187
C5orf42	-0.31405	0.002708
C5orf56	-0.471	0.007719
C5orf63	-0.40081	0.000198
C5orf66	0.554217	0.002081
C6orf1	0.558617	9.40E-05
C6orf10	-1.11184	0.000953
C6orf136	0.292208	0.021681
C6orf141	-0.30632	0.036965
C6orf201	-0.99866	0.009604
C6orf62	-0.1846	0.001226
C6orf89	-0.19482	0.000173
C6orf99	0.662758	0.024335
C7orf26	0.262765	0.007084
C7orf31	-0.35814	0.001621
C7orf57	0.311582	0.008848
C7orf65	-1.33564	0.007874
C8orf31	-0.98807	0.00488
C8orf33	0.278873	4.72E-06
C8orf4	-0.64495	0.029512
C8orf44	-0.28766	0.034669
C8orf46	-0.72498	0.02778
C8orf48	-0.35951	0.013481

C9orf142	0.448391	0.031956
C9orf3	-0.34286	1.62E-05
C9orf47	0.621903	0.039202
C9orf78	0.151719	0.002218
CA11	-0.64685	0.000162
CA3-AS1	-1.11127	0.00339
CAAP1	-0.1695	0.046639
CACFD1	-0.47473	0.01031
CACNA1A	-0.36239	0.004329
CACNA1H	1.227809	6.75E-06
CACNG4	-1.33204	2.50E-19
CACTIN	0.399456	0.02766
CACTIN-AS	1.034563	0.039399
CACYBP	0.25058	0.001507
CAD	0.390237	2.46E-06
CADM1	0.441589	0.000492
CADPS2	-0.56645	0.000462
CALB1	0.641071	1.63E-12
CALCOCO1	-0.37808	0.000423
CALCOCO2	-0.21023	0.007787
CALD1	-0.79128	9.10E-24
CALHM2	0.668384	1.02E-05
CALHM3	0.899781	0.002148
CALM3	0.190119	0.031544
CALU	-0.14059	0.038429
CAMK1D	-0.80025	3.25E-08
CAMK2D	0.167173	0.015173
CAMK2N1	0.266881	0.005094
CAMK4	0.841493	2.79E-05
CAMLG	0.163527	0.0092
CAMSAP1	0.154122	0.005459
CANT1	0.206496	0.024374
CAP2	-0.15227	0.044514
CAPN10	0.299858	0.036912
CAPN13	-2.35096	0.002318
CAPN14	-0.55609	0.047851
CAPN3	-0.60509	0.004943
CAPRIN2	0.556025	1.93E-09
CAPS2	-0.40704	0.003526
CARD10	0.510448	0.002845
CARD11	0.540032	3.24E-06
CARD16	-0.45862	2.08E-05
CARD17	-1.51814	5.21E-06
CARD6	-0.26777	0.014919
CARD8	-0.25008	4.23E-05
CARF	-0.55183	2.07E-05
CARM1	0.447032	4.19E-05

CARMIL2	0.657405	0.038339
CARMIL3	-1.08023	0.005172
CARMN	-0.96452	1.93E-08
CARNMT1	0.2951	0.000774
CARNS1	-0.58181	0.020985
CASC8	0.834208	0.000191
CASC9	-0.25714	0.018587
CASP10	-0.41005	0.0004
CASP14	-1.24647	0.001224
CASP1P2	-0.47368	0.039803
CASP6	-0.28472	0.00092
CASP7	-0.34467	0.000228
CASP9	-0.32788	0.011039
CASQ2	-0.69516	0.001635
CAT	-0.33996	5.79E-08
CATSPER2	-0.39849	0.006723
CAV1	0.178044	0.031133
CBFA2T2	-0.29733	2.85E-05
CBFA2T3	-0.63748	0.003742
CBFB	0.220793	0.003519
CBL	0.19481	0.013516
CBLN3	-0.45293	0.006784
CBR1	-0.24301	0.033218
CBR3-AS1	-0.67219	0.000352
CC2D2A	0.232245	0.010697
CCAR2	0.261344	0.001922
CCAT1	0.607586	0.017501
CCDC102B	-0.40407	0.01463
CCDC103	-0.74142	0.004611
CCDC115	-0.1694	0.020939
CCDC12	0.19999	0.032524
CCDC122	-0.22367	0.043427
CCDC124	0.364713	0.047983
CCDC126	-0.41347	0.00015
CCDC138	0.248091	0.002568
CCDC144A	-0.41438	0.000936
CCDC144B	-0.57549	0.000843
CCDC144CI	-0.77312	1.65E-06
CCDC144N	-0.6518	0.018085
CCDC146	-0.96295	1.62E-09
CCDC149	-0.47888	0.00053
CCDC150	0.365163	0.004405
CCDC162P	-0.27521	0.018258
CCDC173	-1.09902	0.013991
CCDC186	-0.25842	0.012951
CCDC189	0.48548	0.016979
CCDC18-AS	-0.41482	1.84E-05

CCDC191	-0.50857	0.010585
CCDC22	0.286997	0.020027
CCDC3	0.578272	1.12E-07
CCDC34	0.229625	0.049724
CCDC39	-0.45911	0.0057
CCDC50	-0.19263	0.014809
CCDC57	0.230677	0.03861
CCDC58	0.285145	0.001484
CCDC59	0.212857	0.011896
CCDC69	1.088982	3.85E-15
CCDC71L	0.426114	0.000721
CCDC74B	0.681594	0.030714
CCDC80	-0.8874	1.27E-07
CCDC85C	0.435469	1.33E-07
CCDC86	0.552823	4.76E-06
CCDC9	0.208205	0.048639
CCDC92	-0.37304	0.003881
CCHCR1	0.257076	0.007578
CCK	1.055894	0.021511
CCL20	1.134802	2.82E-08
CCL28	-0.33548	0.000434
CCL5	-1.25164	0.017902
CCNA1	1.252005	2.44E-23
CCNA2	0.253772	0.015309
CCNB1	0.308282	0.000284
CCNB1IP1	0.141568	0.028908
CCNB2	0.25906	0.000197
CCNB3	0.706749	0.03838
CCND2	0.585914	2.10E-13
CCND3	0.328194	0.00092
CCNDBP1	-0.19637	0.014072
CCNG1	0.232729	0.001727
CCNH	0.214908	0.002709
CCNJL	-0.43723	0.000348
CCNK	0.238241	1.59E-05
CCNO	0.620691	0.002026
CCNY	0.098955	0.049255
CCPG1	-0.39222	0.000788
CCT2	0.243787	0.000504
CCT3	0.233138	1.33E-06
CCT4	0.291094	4.01E-06
CCT5	0.280573	5.22E-07
CCT6A	0.288487	5.40E-08
CCT6B	-0.57468	0.028478
CCT7	0.378112	2.29E-13
CCT8	0.232996	0.00098
CD101	-0.51358	0.004907

CD109	0.186873	0.029944
CD160	-0.43581	0.049176
CD163L1	-0.36683	0.03354
CD24	-0.46957	7.36E-17
CD27-AS1	-0.24328	0.003726
CD320	0.532197	0.002941
CD33	0.960828	0.034089
CD36	-1.13581	7.08E-12
CD37	-0.54455	0.004185
CD38	-0.89417	0.03021
CD3EAP	0.409501	0.000615
CD44	0.367626	5.10E-07
CD46	-0.23654	0.004349
CD47	-0.23559	0.002711
CD55	0.520165	1.67E-06
CD59	0.179856	0.010823
CD6	0.61558	0.013021
CD68	0.318464	0.000577
CD83	0.234372	0.019356
CDA	0.275924	0.002762
CDC123	0.233439	0.0001
CDC20	0.574142	3.25E-06
CDC25A	0.560815	1.63E-06
CDC25B	0.371427	0.001547
CDC34	0.384826	0.007324
CDC37	0.361092	0.000255
CDC42BPA	-0.35106	7.20E-07
CDC42EP1	0.709751	4.50E-05
CDC42EP2	0.529342	0.005032
CDC42EP3	-0.48101	3.11E-08
CDC42SE2	-0.29451	0.0001
CDC45	0.395982	6.70E-09
CDC6	0.249646	1.64E-05
CDCA2	0.300536	0.00259
CDCA3	0.446315	0.000253
CDCA4	0.328889	0.012192
CDCA5	0.409401	5.02E-05
CDCA7	0.212976	0.040753
CDCA7L	0.197715	6.40E-05
CDCA8	0.35423	0.007436
CDCP1	0.265852	9.20E-07
CDH1	-0.43477	0.000858
CDH11	0.798677	3.29E-08
CDH13	-0.2407	0.000448
CDH2	-0.30136	0.000906
CDHR2	-0.83662	0.000119
CDK1	0.289864	0.007905

CDK10	0.293303	0.00823
CDK11B	0.156235	0.039128
CDK12	0.150063	0.009355
CDK17	0.254458	0.001409
CDK19	-0.28493	0.004213
CDK2	0.233068	0.001138
CDK2AP1	0.123529	0.038356
CDK4	0.416238	2.99E-05
CDK5RAP1	0.226581	4.38E-05
CDK5RAP2	0.142881	0.002602
CDK5RAP3	0.166243	0.018381
CDK6	0.283801	0.001689
CDKL3	-0.48833	0.02017
CDKL5	-0.35071	0.00019
CDKN1B	-0.19659	0.007359
CDKN2AIPN	0.195277	0.001384
CDKN2B	-0.72314	1.48E-10
CDKN3	0.159162	0.023505
CDNF	-0.5905	0.005554
CDPF1	0.350077	0.027746
CDRT1	-0.6483	3.04E-05
CDS1	-0.19202	0.007464
CDT1	0.453889	0.026219
CDYL	0.19917	2.94E-05
CEACAM19	-0.46524	0.01525
CEBPZ	0.203698	0.005341
CECR7	-0.3097	0.025651
CELF1	0.175281	0.002519
CELSR3	-0.32694	0.031774
CEMIP	0.191725	0.000808
CENPA	0.332133	0.045487
CENPH	0.31286	0.00013
CENPN	0.199835	0.007761
CENPO	0.262316	0.003877
CENPP	0.133671	0.040393
CENPU	0.212633	0.004163
CENPV	0.415577	4.54E-05
CENPW	0.315171	0.000549
CEP112	-0.40938	0.021662
CEP126	-0.24059	0.003248
CEP128	0.227505	0.008125
CEP131	0.407242	0.017906
CEP135	0.193662	0.047056
CEP162	-0.20373	0.026864
CEP164	0.257613	0.000154
CEP170	0.314607	0.002595
CEP19	-0.38006	0.000537

CEP250	0.245277	0.008711
CEP55	0.307519	1.05E-05
CEP70	-0.36573	0.000278
CEP72	0.389892	0.000405
CEP78	0.162376	0.004808
CEP83	0.278432	0.003828
CEP85	0.161874	0.003623
CEP89	0.14071	0.027083
CERCAM	-0.30512	0.024672
CERKL	-0.40307	5.65E-06
CERS2	0.213204	0.001996
CERS3	-0.42637	0.002887
CERS4	-0.3591	0.027219
CES4A	-0.66109	0.000147
CETP	-0.98571	0.032922
CFAP20	0.201024	0.000775
CFAP206	-0.76214	0.003276
CFAP44	-0.65161	2.18E-06
CFAP46	0.739832	0.003603
CFAP57	-0.22114	0.032067
CFAP70	-0.50249	0.004583
CFAP97	0.172044	0.038606
CFB	-1.01617	0.018698
CFH	-0.18197	0.019403
CFI	-2.25472	3.93E-05
CFL2	-0.20039	0.006499
CFLAR	-0.4613	0.000135
CGB7	0.316543	0.035541
CGB8	0.676258	0.001652
CGNL1	0.264672	0.004091
CH17-195P	-0.44291	0.029569
CH17-264L	-0.40732	0.00189
CH17-296N	0.531698	0.008757
CH507-236	-0.77566	0.034069
CH507-338	-0.77566	0.034069
CH507-513	-0.28867	2.75E-05
CH507-528	-0.2882	2.90E-05
CH507-9B2	-0.51241	0.041887
CH507-9B2	0.399049	6.98E-06
CHAC1	0.299244	0.039536
CHAF1A	0.347479	0.00016
CHAF1B	0.180874	0.004463
CHAMP1	0.165028	0.031447
CHCHD2	0.189172	0.034347
CHCHD3	0.275024	3.69E-07
CHCHD4	0.570867	2.74E-09
CHCHD5	0.234114	0.012734

CHD1	0.211446	0.00406
CHD1L	0.174644	0.010207
CHD4	0.162333	0.004795
CHD6	-0.19093	0.004889
CHD7	0.213804	0.005748
CHEK1	0.214944	0.001444
CHERP	0.412558	0.001719
CHI3L1	-1.19957	0.019007
CHIC1	-0.20418	0.029558
CHIC2	0.336806	0.000425
CHID1	0.189345	0.016725
CHM	-0.1749	0.048384
CHMP1B	-0.22323	0.006013
CHMP1B2F	-0.5132	0.043011
CHMP3	-0.1333	0.013617
CHN1	-0.3276	0.040352
CHN2	-0.27591	0.040068
CHODL	-0.45317	0.036104
CHP1	-0.14183	0.032254
CHRAC1	0.120664	0.018598
CHRNA5	0.440127	0.000891
CHST11	0.144578	0.011484
CHST15	-0.12674	0.049831
CHSY3	-0.44499	0.025414
CHTF18	0.675876	0.002967
CHTOP	0.115476	0.048322
CIAO1	0.099942	0.042341
CIB1	0.31922	0.001079
CIITA	-0.44125	0.006146
CISD3	0.277433	0.032331
CISH	0.386119	0.036874
CIT	0.236777	0.002732
CIZ1	0.263981	0.004791
CKAP2L	0.225748	0.006563
CKAP5	0.174885	0.003686
CKMT1B	-0.20035	0.014244
CKS1B	0.408817	2.78E-06
CKS2	0.270222	0.022819
CLASP1	-0.14983	0.01545
CLCA2	-0.89519	0.00122
CLCF1	0.341728	0.001228
CLCN3	-0.17279	0.005176
CLCN6	0.138235	0.021177
CLDN1	-1.26645	3.87E-41
CLDN16	-1.00609	4.07E-10
CLDN23	-0.89035	1.36E-06
CLDN4	-0.43107	0.025839

CLEC12A	-0.87216	0.001014
CLEC2D	-0.56636	0.000827
CLEC7A	-1.24331	8.07E-06
CLHC1	-0.39998	0.008359
CLIC1	0.180374	0.000193
CLIC3	-1.29536	2.10E-05
CLIC4	-0.37484	0.001067
CLIP1	-0.39151	0.000426
CLIP3	-0.68239	0.000856
CLIP4	-0.21532	0.039746
CLK2	0.176303	0.010671
CLK3	0.179864	0.009184
CLMN	0.318689	0.027694
CLMP	0.684722	7.52E-17
CLN5	-0.21746	0.004781
CLN6	0.318104	0.001764
CLN8	-0.25574	0.000123
CLNS1A	0.195417	0.003225
CLPB	0.364832	7.06E-07
CLPP	0.374203	0.028858
CLPTM1L	0.188429	0.009506
CLU	-0.38549	0.001853
CLUAP1	-0.20731	0.018849
CLUH	0.593618	0.000146
CLUHP3	-0.18839	0.022974
CLYBL	-0.52505	0.004351
CMAHP	-0.55642	0.04996
CMBL	0.273121	0.001917
CMP21-97(	-3.89142	0.002305
CMSS1	0.135744	0.046049
CMTM4	-0.36115	6.18E-12
CMTM7	0.407232	9.04E-05
CMYA5	-0.37562	0.042602
CNBP	0.251867	7.61E-06
CNGB1	-0.64373	0.000253
CNIH3	0.482407	0.004611
CNNM2	-0.25889	0.004658
CNOT10	0.132733	0.015661
CNOT3	0.290533	0.028843
CNOT7	0.14602	0.018561
CNOT9	0.13688	0.01512
CNPY1	-3.25228	0.020018
CNPY4	-0.2416	0.01044
CNTN1	0.225244	0.02842
CNTNAP3	0.256368	0.017027
COA4	0.265432	0.000562
COA5	-0.32862	1.24E-05

COA6	0.10866	0.045106
COBLL1	-0.39954	0.009411
COL12A1	-0.17824	0.004162
COL13A1	0.379901	0.008229
COL17A1	0.25408	0.00016
COL1A1	-0.39562	0.013141
COL1A2	-0.68956	2.84E-06
COL21A1	-0.9611	0.000279
COL3A1	-1.61735	3.27E-14
COL4A1	0.239629	0.002874
COL4A2	0.317346	0.01431
COL4A4	-1.00113	0.035204
COL4A5	-0.50939	2.39E-06
COL4A6	-0.12724	0.036065
COL5A1	-0.42113	0.001121
COL5A1-AS	1.101142	0.044219
COL5A2	-0.92276	7.85E-18
COL5A3	-0.35204	0.013503
COL6A3	0.603284	0.024965
COL8A1	-0.60755	1.22E-11
COL9A2	-0.69957	0.000936
COLCA1	-1.0778	0.000179
COLCA2	-2.08501	4.99E-06
COLGALT2	-1.86099	0.02535
COMMD2	-0.17913	0.010782
COMMD3	-0.1374	0.01746
COMMD4	0.18791	0.020252
COPRS	0.273593	0.012712
COPS3	0.276572	9.38E-08
COPS6	0.178561	0.001765
COPS7A	0.191936	0.018817
COPS8	0.139717	0.016434
COPZ1	0.116464	0.00516
COPZ2	-0.29252	0.023156
COQ2	0.266806	0.019864
COQ8A	0.277418	0.000839
CORO1A	0.630724	1.20E-05
CORO1C	0.096135	0.012637
CORO2A	0.154826	0.003131
COTL1	0.358224	0.000409
COX10	0.287896	0.000696
CPA4	-0.86094	6.44E-06
CPE	-0.59224	1.21E-07
CPEB2	-0.32842	0.035056
CPNE1	0.284464	1.64E-05
CPNE3	-0.16332	0.042393
CPNE7	0.639301	3.77E-05

CPOX	0.151812	0.033772
CPQ	-0.55222	1.80E-05
CPS1	-0.18636	0.000973
CPSF1	0.432043	0.001177
CPSF3	0.169181	0.005384
CPSF4	0.291911	0.00336
CPSF6	0.114631	0.018421
CPT2	-0.25308	0.000497
CPVL	-0.13683	0.00401
CPZ	-0.50503	0.012438
CR1	-0.86223	0.025964
CRACR2A	0.400981	9.83E-08
CRAMP1	0.140846	0.032124
CRCP	0.163767	0.007025
CREB3L2	-0.13427	0.036448
CREB5	-0.43773	0.03399
CREBL2	-0.17125	0.019609
CREG1	-0.46464	5.60E-06
CREG2	-0.3871	9.55E-06
CRELD1	-0.47573	0.006219
CRIM1	-0.24737	0.000212
CRISPLD1	-0.57013	0.000296
CRISPLD2	0.39703	0.027843
CROCC2	1.271823	0.027998
CROT	-0.45318	1.08E-06
CRYAB	-0.74108	7.45E-05
CRYBG3	-0.19856	0.013201
CRYL1	-0.22694	0.037948
CRYZL1	-0.20153	0.014686
CS	0.18177	4.10E-06
CSDC2	-0.96675	0.001683
CSE1L	0.304857	9.47E-07
CSF1	-0.44876	2.00E-05
CSF1R	-0.29721	0.040117
CSF2	0.883607	4.59E-07
CSMD2	0.736498	1.62E-07
CSNK1E	0.452124	1.93E-07
CSNK2A1	0.140207	0.00156
CSNK2A2	0.171676	0.001957
CSNK2B	0.172986	0.029828
CSPG4	0.353738	0.02123
CSRNP1	0.566332	1.37E-05
CSRNP3	-1.22465	2.09E-11
CSRP1	-0.27222	0.000177
CSRP2	0.12899	0.017851
CSTF2	0.267526	5.61E-06
CTA-212A2	-1.45122	0.0011

CTA-293F1	-0.56682	0.008451
CTA-414D7	0.516458	0.037298
CTAGE5	-0.204	0.038256
CTB-113P1	-0.52569	0.031249
CTB-133P2	0.711158	5.32E-05
CTB-151G2	-0.59539	0.000118
CTB-193M1	0.398781	0.000106
CTB-58E17	-0.78213	0.034226
CTB-5E10.3	0.663494	0.02417
CTB-60B18	0.567882	0.00243
CTBP2	0.221407	2.84E-09
CTBS	-0.23031	0.001294
CTC-261N6	-3.584	0.028451
CTC-351M1	-0.39992	0.02132
CTC-429L19	-1.57137	0.047582
CTC-471F3.	-0.6334	0.0058
CTC-479C5	0.468224	0.048784
CTC-490E2	-0.36593	0.009283
CTC-510F1	-0.63315	0.004206
CTCF	0.113045	0.002383
CTD-2008L	-0.43051	0.008562
CTD-2008P	-0.39543	0.043186
CTD-2036P	-0.83597	0.027792
CTD-2047H	-3.32247	0.001238
CTD-2058B	0.568054	1.65E-07
CTD-2066L	0.394521	0.029996
CTD-2192J1	-0.40411	0.007337
CTD-2269F	-0.92671	1.07E-05
CTD-2284J1	-0.68907	0.003017
CTD-2292M	-0.40685	0.012161
CTD-2303H	-0.78827	4.72E-06
CTD-2309C	-1.34772	0.008691
CTD-2319I1	-1.14915	0.004327
CTD-2323K	0.750485	0.007726
CTD-2354A	0.708553	8.63E-09
CTD-2382E	1.152389	0.004526
CTD-2396E	-0.8613	0.000674
CTD-2510F	0.292565	0.014733
CTD-2514C	-0.47617	0.032075
CTD-2521M	-0.85007	0.01454
CTD-2541J1	0.970815	0.007796
CTD-2547L	-0.83201	0.000182
CTD-2547L	-0.4321	0.019043
CTD-2553L	-1.32692	0.027046
CTD-2554C	-0.4231	0.031368
CTD-2562J1	1.521608	0.024403
CTD-2583A	0.47156	0.025475

CTD-2587H	1.049221	0.001836
CTD-2619J:	-0.44796	0.031114
CTD-2623N	-0.83667	0.037825
CTD-2626G	-0.35714	0.001174
CTD-2647L	-0.43385	0.029578
CTD-2666L	-0.86162	0.029009
CTD-3006G	-0.64292	0.018839
CTD-3088G	-0.46835	0.03915
CTD-3105H	-1.70781	0.014965
CTD-3105H	-2.37191	0.018099
CTD-3113P	-0.25583	0.033998
CTD-3131K	-2.28283	0.049858
CTD-3214H	-3.32053	4.42E-05
CTD-3214K	-1.30301	0.036838
CTD-3247H	-1.96634	0.036003
CTDNEP1	0.269964	0.036036
CTF1	-0.50853	0.005479
CTNNAL1	0.597584	2.59E-13
CTNNBL1	0.17796	0.000885
CTPS1	0.267546	2.54E-05
CTSA	-0.26102	0.022606
CTSB	-0.39996	0.00015
CTSF	-0.68683	4.64E-08
CTSH	-0.2645	0.004078
CTSK	-0.36153	2.97E-05
CTSO	-1.016	2.33E-08
CTU2	0.428595	0.007142
CUL2	0.137417	0.016665
CUL4A	0.134877	0.001623
CUL5	-0.18585	0.018963
CUTC	0.174661	0.01461
CWC25	0.13854	0.032474
CWF19L1	0.130389	0.046128
CXADR	-0.46453	0.000803
CXCL1	0.451796	0.006025
CXCL10	-1.91374	0.009711
CXCL11	-0.7519	0.01932
CXCL12	-0.69316	0.02998
CXCL14	-1.20418	0.000142
CXCL16	-0.53452	5.43E-05
CXCL9	-2.25266	0.006199
CXCR2	-2.11892	0.036255
CXorf23	-0.36254	0.00076
CXorf40B	0.155977	0.035017
CXorf57	0.452323	6.39E-05
CXXC1	0.243598	0.033163
CXXC5	-0.87962	2.82E-07

CYB561	-0.1965	0.019155
CYB561A3	-0.33269	0.001231
CYB561D2	0.237743	0.028004
CYB5B	0.127115	0.006664
CYB5D1	0.275652	0.001205
CYB5D2	-0.45901	0.000101
CYB5R1	-0.38137	0.00074
CYB5R2	-0.57944	5.80E-05
CYBRD1	-0.71054	3.51E-19
CYC1	0.285502	0.015478
CYLD	-0.33664	0.00299
CYP1A1	-0.91322	0.000241
CYP1B1-AS	0.260814	0.000583
CYP26B1	0.493398	4.63E-06
CYP27B1	0.949862	2.81E-12
CYP27C1	0.286218	0.002262
CYP2B6	1.09515	1.97E-05
CYP2B7P	0.931738	5.06E-07
CYP2R1	-1.13338	0.012172
CYP2S1	0.343829	0.010955
CYP2U1	-1.07472	3.47E-10
CYP39A1	-0.90591	1.31E-08
CYP3A5	-0.4333	0.008245
CYP3A7	-0.71481	0.001148
CYP3A7-CY	-1.86692	0.007912
CYP4B1	-2.12595	0.003643
CYP4X1	-0.91092	5.38E-11
CYTH2	0.182303	0.023101
CYTH4	1.233503	0.001173
CYTOR	0.349804	0.000289
DAG1	-0.50358	7.50E-06
DAGLB	0.265896	0.002537
DANCR	0.363294	0.00079
DAP3	0.146498	0.013635
DAPK1	-0.68864	3.13E-07
DAPL1	-1.18584	0.000847
DARS	0.163458	0.019295
DARS2	0.213964	0.000418
DAW1	0.795209	0.003323
DAXX	0.195814	0.000544
DAZAP1	0.367171	0.000374
DBF4	0.287269	0.007644
DBF4B	0.254722	0.007174
DBH	0.917213	0.001903
DBNDD2	0.263534	0.035792
DBT	-0.1859	0.029843
DCAF1	0.177855	0.003741

DCAF13	0.270814	0.000433
DCAF15	0.307102	0.048825
DCAF5	-0.24829	9.29E-06
DCAF6	-0.1862	0.000221
DCBLD1	-0.68929	1.42E-22
DCDC1	1.149282	0.02441
DCK	0.162342	0.022972
DCLK2	-0.58516	0.049746
DCLRE1B	0.229673	0.004874
DCN	-0.97742	1.61E-08
DCPS	0.193725	0.017784
DCTD	0.313707	1.23E-07
DCTPP1	0.384788	4.77E-05
DCUN1D2-,	1.823254	0.023296
DCUN1D5	0.256897	0.000115
DDB1	0.101372	0.007693
DDB2	0.167479	0.009609
DDIAS	0.249569	0.005068
DDN	1.153527	4.07E-07
DDO	-0.70011	0.003277
DDX10	0.256611	0.00035
DDX11	0.291713	0.010513
DDX18	0.210932	0.004949
DDX19A	0.127021	0.035145
DDX20	0.140049	0.006626
DDX21	0.367908	3.58E-06
DDX23	0.167844	0.003879
DDX24	0.153312	0.003462
DDX27	0.186649	3.17E-05
DDX28	0.283698	0.012809
DDX31	0.368274	1.97E-11
DDX39A	0.386223	0.000124
DDX39B	0.309822	1.56E-08
DDX41	0.241634	0.007958
DDX49	0.335037	0.00522
DDX50	0.172095	0.012894
DDX50P1	0.584275	0.045057
DDX54	0.444164	0.002883
DDX55	0.182838	0.007629
DDX56	0.313799	4.41E-05
DECR1	-0.53477	8.15E-07
DEDD2	0.405647	0.005058
DEF6	0.293469	0.008636
DEFB1	-1.87885	0.000644
DEGS1	-0.16189	0.037567
DENND1B	-0.48619	2.18E-05
DENND1C	-0.64184	0.004509

DENND2D	-0.22509	0.037578
DENND3	0.246017	0.001095
DENND4C	-0.28291	0.011156
DEPDC1B	0.241179	0.003546
DFFA	0.132796	0.030576
DGKA	0.267414	1.22E-07
DGKD	0.383873	1.03E-09
DGKI	0.401129	0.00022
DGKZ	0.404853	0.012767
DHCR7	0.307727	0.000311
DHODH	0.192905	0.032549
DHPS	0.219519	0.002514
DHRS1	-0.20747	0.012409
DHRS11	0.348362	0.016345
DHRS12	-0.64065	0.000332
DHRS4-AS1	-0.20952	0.004098
DHRS7	-0.2171	0.002302
DHRS9	1.512584	1.23E-07
DHX15	0.194521	0.002852
DHX30	0.3656	0.000318
DHX33	0.476848	2.69E-28
DHX34	0.412087	0.012594
DHX35	0.192	0.000591
DHX37	0.423648	0.001224
DHX38	0.206839	0.000157
DHX40	-0.14707	0.04359
DHX9	0.154724	0.01094
DIABLO	0.1912	0.000233
DIAPH1	0.167816	5.47E-05
DIAPH2	0.242654	0.004905
DIEXF	0.258016	8.83E-06
DIMT1	0.314566	6.77E-06
DIRC2	-0.23028	0.000835
DIRC3	0.783458	0.046096
DIS3	0.205907	0.000906
DISC1	-0.95796	0.000239
DISP1	-0.40544	0.012152
DISP3	0.379851	0.048741
DIXDC1	-0.5235	6.36E-05
DKC1	0.397938	1.74E-07
DKK1	0.299189	2.18E-06
DKK3	-0.33719	4.59E-07
DLAT	0.190043	0.003734
DLC1	0.416154	9.14E-05
DLEC1	-1.63358	0.016864
DLEU1	0.261853	0.026911
DLG1-AS1	-0.44457	0.008585

DLG4	0.640623	0.000678
DLGAP1	-0.7315	0.000527
DLGAP1-AS	-1.30081	0.040838
DLGAP5	0.284151	0.010072
DLST	0.128563	0.004409
DLX1	0.322833	0.03023
DLX2	0.564255	0.019214
DLX3	-0.44331	0.00355
DLX4	-0.61386	0.000115
DMD	-0.1768	0.023163
DMKN	0.183312	0.00917
DMRT2	0.861303	0.020057
DMRT3	-1.43474	0.018255
DMRTC2	2.230182	0.012308
DMXL1	-0.3374	0.000758
DNA2	0.18693	0.0101
DNAAF3	0.633678	0.005164
DNAAF5	0.430821	0.000199
DNAH17	-1.19457	0.000302
DNAH5	-0.37907	0.000655
DNAJA3	0.328903	8.98E-06
DNAJA4	0.258522	0.017149
DNAJB2	-0.15593	0.028372
DNAJB4	-0.37435	0.027674
DNAJB9	-0.29206	0.029182
DNAJC1	-0.33704	7.94E-05
DNAJC11	0.241293	0.000555
DNAJC14	0.156744	0.006777
DNAJC16	-0.18981	0.011295
DNAJC2	0.256906	0.000624
DNAJC3	-0.2089	0.018483
DNAJC5	0.194328	0.012521
DNAJC7	0.156841	0.00092
DNAJC8	0.113482	0.019081
DNAJC9	0.239906	7.91E-08
DNAL1	-0.16725	0.047783
DNAL4	-0.23024	0.021886
DNASE2	-0.32002	0.000155
DNER	0.484932	2.21E-07
DNLZ	0.506209	0.017108
DNM1P35	-0.6561	0.044332
DNM2	0.198239	0.040849
DNM3	0.421556	0.009985
DNMBP	0.468896	1.18E-20
DNMT1	0.34768	3.64E-08
DNMT3L	-3.04462	0.035058
DNTTIP1	0.256403	0.001813

DOCK10	0.484376	4.00E-06
DOCK4	0.818826	1.30E-07
DOCK6	0.282905	0.021948
DOCK8	-0.76086	0.002018
DOCK9	0.236037	8.08E-05
DOPEY2	-0.37236	0.002582
DPAGT1	0.302478	8.51E-07
DPF1	0.446868	0.001198
DPF2	0.193583	0.001313
DPF3	0.450176	0.000966
DPH2	0.340744	0.000638
DPH7	0.298248	1.74E-05
DPM2	0.34486	4.33E-05
DPP4	-0.61558	0.000472
DPP8	-0.16852	0.035752
DPP9	0.434668	0.00085
DQX1	-0.52599	0.040879
DRAM1	-0.78259	8.52E-14
DRAM2	-0.15738	0.014479
DRAP1	0.520587	0.000312
DRD2	0.736999	0.037901
DRG1	0.197057	0.001369
DRG2	0.383123	3.06E-06
DSC1	-1.93571	0.000138
DSC2	-0.48647	0.000718
DSC3	-0.20864	0.023068
DSCR3	-0.13964	0.045821
DSEL	0.232556	0.007114
DSG3	-0.44794	0.006738
DST	-0.34227	1.37E-05
DSTN	-0.3828	2.09E-09
DSTYK	-0.2461	2.98E-06
DTD1	0.260047	0.001585
DTL	0.328195	2.23E-06
DTNA	-0.48231	0.003681
DTWD2	-0.48917	5.88E-05
DTX4	-0.57809	4.33E-06
DTYMK	0.372365	0.004295
DUBR	-0.49369	7.15E-05
DUOX2	-0.44124	0.028099
DUS1L	0.284698	0.026319
DUS2	0.198562	0.001091
DUS3L	0.560661	0.000456
DUSP11	0.193515	0.021771
DUSP12	0.187588	0.001448
DUSP14	-0.17991	0.025741
DUSP16	-0.8121	3.35E-07

DUSP2	0.892248	2.73E-08
DUSP3	-0.26188	0.000778
DUSP4	1.62	1.87E-65
DUSP5	1.341816	5.19E-30
DUSP5P1	-0.68481	0.010145
DUSP6	1.223475	8.96E-51
DUSP7	0.518338	1.91E-05
DUSP8	-1.25416	0.001156
DUSP9	0.500753	0.017125
DUXAP10	-0.20508	0.004069
DUXAP8	-0.23029	0.002689
DVL1	0.622558	0.002397
DYNC1LI1	0.14254	0.00963
DYNC1LI2	-0.16767	0.024144
DYNLT3	-0.29302	0.001116
DYRK3	0.370057	0.00014
DYSF	-0.29267	0.004153
DYX1C1	0.630966	0.010443
DYX1C1-CC	-6.821	2.13E-05
E2F1	0.297554	0.008992
E2F3	0.19434	0.000753
E2F4	0.412249	1.12E-06
E2F6P4	-0.46724	0.018073
E2F7	0.589011	1.58E-10
E2F8	0.199639	0.033944
EARS2	0.203804	0.00638
EBF3	-0.10255	0.016713
EBF4	-0.78027	0.00151
EBNA1BP2	0.406377	2.80E-08
ECE2	0.448933	2.81E-06
ECEL1P1	-1.39428	0.006814
ECEL1P2	-1.58128	0.006173
ECH1	-0.20497	0.019075
ECHDC2	-0.72298	1.14E-06
ECI2	0.151583	0.02913
ECM1	0.43914	0.006643
ECSIT	0.457349	2.32E-05
ECT2	0.220575	0.018465
EDARADD	-0.38825	1.03E-07
EDEM2	-0.16114	0.031872
EDEM3	-0.23402	0.013719
EDIL3	-1.52058	1.03E-13
EDN1	-0.98003	2.58E-24
EDN2	-0.67392	0.013163
EDNRA	0.545524	6.23E-06
EDNRB	-0.24188	0.016963
EEF1A1	0.160406	0.003907

EEF1A1P11	1.244977	0.032234
EEF1B2	0.21759	4.97E-05
EEF1D	0.369466	0.000744
EEF1E1	0.324111	0.001421
EEF1G	0.263776	4.46E-06
EEF2	0.383332	7.26E-05
EEF2KMT	0.253929	0.016912
EFCAB1	0.42332	0.035618
EFCAB14	-0.18066	0.0012
EFCAB2	-0.23982	0.022167
EFCAB6	-0.75024	0.001126
EFEMP1	-0.11078	0.01536
EFHD1	-0.84995	2.99E-09
EFHD2	0.701936	1.96E-05
EFNA1	-0.31209	0.000664
EFNA5	-0.33942	8.64E-07
EFNB1	0.623732	0.000277
EFNB2	0.214289	0.000342
EFTUD2	0.240923	1.87E-06
EGF	-0.41876	3.57E-05
EGFL6	-1.35095	0.002286
EGFR	-0.08277	0.024581
EGLN1	-0.24636	0.002631
EGR1	2.444051	1.50E-42
EGR2	1.974086	4.20E-15
EGR3	1.794795	4.97E-21
EHBP1	-0.19223	0.016482
EHD1	0.3013	0.035324
EHD4	0.179654	0.022926
EHHADH	-0.3854	1.38E-06
EHMT2	0.200526	0.044308
EIF1	0.156396	0.006643
EIF1AD	0.154419	0.020396
EIF2A	0.147878	0.048341
EIF2AK3	-0.23474	0.03218
EIF2B2	0.295367	2.76E-08
EIF2B3	0.140808	0.021847
EIF2B4	0.17456	0.01176
EIF2B5	0.148535	0.004864
EIF2D	0.155625	0.025155
EIF2S2	0.152818	0.012121
EIF3A	0.16138	0.010194
EIF3B	0.316912	1.06E-07
EIF3C	0.332059	3.36E-10
EIF3D	0.329118	3.11E-15
EIF3E	0.210986	0.004911
EIF3G	0.314873	0.003462

EIF3H	0.173839	0.000245
EIF3I	0.238973	7.89E-09
EIF3J	0.142632	0.027232
EIF3K	0.176736	0.001836
EIF3L	0.182931	0.000176
EIF3M	0.185424	0.003992
EIF4A1	0.355481	1.13E-14
EIF4B	0.252085	5.50E-07
EIF4E3	-0.5733	0.000717
EIF4EBP1	0.57625	0.000254
EIF4EBP2	-0.15455	0.021601
EIF4G1	0.238675	0.00126
EIF4H	0.164055	0.001003
EIF5A	0.479559	4.82E-07
EIF5B	0.198812	0.004952
EIF6	0.377617	3.42E-06
ELAC2	0.315822	1.31E-07
ELAVL1	0.148448	0.032231
ELFN2	0.824107	1.04E-07
ELK1	0.355704	0.004024
ELK3	0.561209	1.62E-11
ELL	0.376416	0.005382
ELL3	-0.36464	0.033215
ELMO1	0.155271	0.044715
ELMOD2	-0.33408	0.000509
ELOA	0.12165	0.012439
ELOF1	0.202846	0.008933
ELOVL1	0.232474	0.001399
ELOVL2	1.402872	1.45E-17
ELOVL4	0.444818	3.64E-05
ELOVL6	0.454973	1.22E-09
ELOVL7	-0.67454	0.000221
ELP3	0.127898	0.03735
ELP5	0.300326	0.002413
EMC2	-0.24484	0.01201
EMC3-AS1	0.31071	0.000134
EMC4	0.154554	0.009278
EMC8	0.248535	0.005553
EMG1	0.348215	1.14E-07
EML2-AS1	0.951115	0.032802
EML3	0.309716	0.015044
EMP1	0.630386	1.09E-10
EMP3	0.592357	2.91E-05
EN1	-0.46591	0.001935
EN2	0.406206	0.016637
ENAH	-0.42089	0.000146
ENDOD1	-0.35256	2.29E-07

ENO1	0.278405	4.36E-07
ENOPH1	0.186984	0.00619
ENPP6	-1.42749	0.035247
ENTPD3	-0.59042	0.001161
ENTPD3-AS	-0.6445	0.002635
ENTPD4	-0.15814	0.010497
ENTPD6	0.256709	0.014416
EPB41	-0.23918	0.004806
EPB41L1	-0.38837	0.023861
EPB41L2	0.270142	4.04E-05
EPB41L4A	-0.63451	0.046656
EPB41L4A-.	0.275325	0.010681
EPHA1-AS1	-0.51565	0.024905
EPHA2	0.641366	4.49E-07
EPHA3	-0.1744	0.01249
EPHB2	0.263478	0.016993
EPHX1	-0.27115	0.011386
EPHX2	-0.52601	7.65E-05
EPM2A	-0.27592	0.002787
EPM2AIP1	-0.36258	2.51E-07
EPN2	0.230913	0.010336
EPOR	0.48069	0.000752
EPRS	0.150215	0.025896
EPS15L1	0.160137	0.00364
ERAL1	0.190926	0.006931
ERAP1	-0.23707	0.017081
ERAP2	-0.33411	0.001169
ERBB3	-0.48979	1.46E-12
ERCC1	0.290445	0.000978
ERCC6L	0.258562	0.027736
EREG	1.362277	8.63E-23
ERFE	0.513718	0.000109
ERG	0.62641	1.52E-09
ERH	0.159868	0.041498
ERI1	0.254147	0.000687
ERI3	0.164489	0.036569
ERICH5	-1.3042	0.001286
ERLEC1	-0.1449	0.005836
ERLIN1	0.151725	0.0065
ERMAP	-0.33199	1.77E-05
ERMP1	-0.22845	0.004045
ERO1B	-0.42196	0.000159
ERRFI1	0.972068	7.01E-14
ERV3-1	-0.42605	0.001115
ERVH-1	0.553723	0.000673
ERVMER34	0.299111	0.00046
ERVW-1	-0.408	0.008626

ESD	0.140637	0.024458
ESF1	0.212523	0.040617
ESM1	1.324292	0.000535
ESPL1	0.405441	0.000103
ESR2	-0.36059	0.048737
ESRG	0.263817	3.22E-05
ESRP1	-0.19368	0.001408
ESYT1	0.208952	0.005508
ESYT3	-0.48805	0.013717
ETAA1	-0.29519	0.00017
ETFBKMT	-0.68192	0.007903
ETFDH	-0.24093	0.000506
ETFRF1	-0.31203	0.000653
ETS1	0.550776	6.27E-12
ETV1	0.643846	0.000142
ETV4	1.092004	3.00E-18
ETV5	0.303729	0.000439
ETV7	-0.70212	0.002749
EVA1C	-0.39567	0.038528
EVC2	0.200741	0.019816
EVI2A	0.410357	0.009288
EVPL	0.462827	0.008192
EWSR1	0.150924	0.001507
EXD2	-0.21491	0.000974
EXOC3L4	-0.65995	0.037836
EXOSC1	0.131905	0.026815
EXOSC10	0.180434	0.000232
EXOSC2	0.335237	2.51E-05
EXOSC3	0.221257	0.001755
EXOSC4	0.387818	0.044347
EXOSC5	0.456995	5.89E-05
EXOSC7	0.339664	6.00E-08
EXOSC8	0.309437	0.000318
EXOSC9	0.244103	0.000525
EXT2	-0.12453	0.047384
EZH2	0.190245	0.000543
EZR	0.310739	6.90E-13
F12	0.54687	0.046881
F3	0.144913	0.026061
F8	-0.51724	0.00027
FAAHP1	-0.49396	0.01467
FAAP100	0.402298	0.03216
FABP5	0.537895	2.63E-05
FADS2	-0.30874	0.011317
FAF1	0.207346	0.00194
FAHD2CP	-0.7252	0.000256
FAIM	-0.41416	9.42E-07

FAM106A	-0.70045	0.023031
FAM107B	0.280594	0.005127
FAM110C	-0.32296	0.007753
FAM114A1	-0.39385	2.79E-07
FAM117B	-0.20199	0.030875
FAM120A	0.122433	0.00632
FAM120B	-0.17924	0.002345
FAM129B	0.51383	1.74E-05
FAM131A	0.268799	0.012994
FAM131B	-0.96653	0.035945
FAM134B	-0.94915	5.76E-09
FAM136A	0.266947	0.00065
FAM138E	-3.98813	0.021764
FAM13A-AS	-0.46445	0.005601
FAM13B	-0.37601	1.95E-05
FAM151B	-0.43931	0.044789
FAM161A	-0.19209	0.027724
FAM167A	0.836859	7.64E-07
FAM168A	-0.13231	0.00705
FAM171A1	0.232807	0.006726
FAM172A	-0.36381	2.25E-05
FAM174A	-0.37693	3.18E-05
FAM177A1	-0.16822	0.001473
FAM182B	-0.82941	0.030175
FAM183A	-0.87525	0.028625
FAM185A	-0.34468	0.023162
FAM185BP	-1.49593	0.021848
FAM189B	0.396887	0.033967
FAM196B	0.95672	2.27E-13
FAM198B	-0.58086	1.43E-09
FAM19A2	-0.83085	0.001841
FAM200A	-0.31211	0.001027
FAM204A	0.171976	0.0288
FAM207A	0.508469	0.004404
FAM212B	-0.54213	1.02E-06
FAM212B-A	-0.93053	0.001454
FAM214A	-0.39936	3.29E-07
FAM216A	0.658416	1.06E-06
FAM21EP	-0.7514	0.007436
FAM21FP	-0.59228	0.049186
FAM222A-A	1.156893	0.024623
FAM227A	-0.428	0.004123
FAM228B	-0.28975	0.003364
FAM26E	-0.67982	0.012094
FAM27C	0.72011	0.001501
FAM46A	-0.46996	0.015186
FAM49A	-1.2309	0.003045

FAM49B	0.187416	0.002525
FAM53C	-0.22723	0.032641
FAM58A	0.26424	0.031513
FAM64A	0.491277	0.000204
FAM69A	-0.31312	0.000612
FAM72A	0.414859	0.00951
FAM72B	0.400095	0.007064
FAM72C	0.536199	0.00045
FAM72D	0.428604	0.022185
FAM78A	-0.61902	0.02093
FAM83A	0.692951	4.03E-16
FAM83A-AS	0.663815	3.99E-09
FAM83D	0.375938	0.018702
FAM83G	0.370047	0.000778
FAM8A1	-0.29007	0.003529
FAM90A12	-2.59421	0.016865
FAN1	-0.14993	0.03934
FANCA	0.407406	1.09E-06
FANCC	0.116499	0.012268
FANCD2	0.275752	1.93E-06
FANCE	0.195775	0.026906
FANCG	0.404601	0.000416
FANCI	0.26866	0.000459
FANK1	-0.40912	0.000477
FAP	-0.4766	8.45E-06
FAR2	0.388748	0.010567
FARSA	0.342446	0.003847
FARSB	0.329252	9.95E-09
FAS	-0.21993	0.011437
FASN	0.622518	0.000208
FAT1	-1.67413	0.004281
FAT2	-0.57621	4.15E-18
FAT3	0.585193	0.034551
FAT4	-0.26481	0.013307
FAU	0.332705	0.00093
FAXDC2	-0.78137	2.62E-07
FBL	0.476078	1.93E-07
FBLN1	-0.47923	1.25E-05
FBN1	-0.52305	4.01E-09
FBN2	0.350634	1.58E-05
FBP1	-0.9714	0.038784
FBXL17	-0.80222	3.33E-09
FBXL20	-0.34396	0.000294
FBXL5	-0.28455	0.000177
FBXL6	0.352427	0.027038
FBXO10	-0.37148	0.003658
FBXO17	0.233154	0.010534

FBXO2	-0.30658	0.030199
FBXO27	-0.36325	7.03E-05
FBXO31	0.220128	0.019279
FBXO32	-0.65485	1.51E-12
FBXO45	0.240091	0.024071
FBXO9	-0.2605	0.00498
FBXW10	-0.96549	0.000654
FBXW2	-0.19279	0.002278
FBXW4	-0.27805	0.006526
FBXW7	0.227692	0.009564
FCGBP	-0.74015	0.016203
FCGR1A	-1.11098	0.001808
FCGR1B	-1.03004	0.027679
FCHSD2	0.174966	0.011299
FCMR	0.788076	0.000256
FDPS	0.187313	0.002418
FECH	-0.27321	7.74E-05
FEN1	0.267694	0.011724
FER1L4	-0.5891	0.000522
FERMT1	0.213852	0.001391
FERMT3	0.717296	0.010649
FETUB	-2.90999	0.048123
FGD3	-0.64015	0.000124
FGF11	-0.46408	2.63E-10
FGF5	-0.37945	0.010776
FGFBP1	0.60405	1.24E-09
FGFR1	0.299905	0.002439
FGFR1OP	0.186439	0.013459
FGFR2	-0.28614	1.62E-05
FGFR4	0.613202	0.00294
FH	0.199579	4.59E-05
FHL1	0.33302	4.52E-07
FHL2	0.187715	0.025838
FHL3	0.421125	9.83E-05
FHOD1	0.389622	0.006235
FHOD3	-0.187	0.007979
FIBIN	-0.98649	7.07E-05
FICD	-0.31543	0.005611
FIG4	-0.47196	0.000137
FIGNL1	0.37168	1.97E-06
FILIP1L	-1.58702	0.006191
FIP1L1	0.179036	0.003552
FKBP10	0.301583	0.025004
FKBP11	0.336766	0.000275
FKBP15	0.101962	0.01343
FKBP1A	0.267855	0.000653
FKBP4	0.373703	6.19E-10

FKBP7	-0.32237	0.030788
FKBP9	-0.23849	0.002734
FLAD1	0.185778	0.031396
FLG	0.476562	0.003242
FLI1	0.541519	7.68E-08
FLII	0.208154	0.02921
FLJ16779	-1.48926	4.23E-07
FLJ20021	0.433668	0.005735
FLJ22447	-0.67349	0.0009
FLJ27354	0.809123	0.001232
FLJ31356	-1.09566	2.62E-06
FLJ37035	-0.59112	0.045102
FLNB	0.512989	8.02E-06
FLNB-AS1	0.785667	0.001737
FLNC	0.681577	1.11E-06
FLOT2	-0.29194	0.000992
FLRT2	0.285192	0.035312
FLRT3	-0.27743	0.000704
FLVCR1	0.228864	0.030496
FMNL2	0.188621	0.038098
FMNL3	0.42245	9.23E-06
FMO4	-0.40392	0.002776
FN1	0.561576	9.16E-05
FNBP1	0.225394	0.00039
FNBP1L	-0.17828	0.034194
FNDC3B	-0.22515	0.001574
FOLH1	-0.9591	0.000198
FOLR1	-2.03903	1.02E-12
FOLR3	-0.38049	0.00318
FOS	0.971668	8.37E-06
FOSB	1.064062	8.10E-15
FOSL1	1.111995	2.38E-19
FOSL2	-0.32374	0.000412
FOXA2	0.933364	0.007977
FOXC2	0.515009	0.000135
FOXD2-AS1	0.355494	0.04051
FOXD3-AS1	-1.12859	0.043433
FOXD4L3	-3.72677	0.015836
FOXE3	1.217338	0.015237
FOXF2	0.363477	0.004934
FOXG1-AS1	0.901146	2.12E-05
FOXJ3	0.172223	0.002008
FOXK2	0.193544	0.019479
FOXL1	0.428369	0.005813
FOXL2	-0.50019	0.032123
FOXL2NB	-0.4634	0.038514
FOXM1	0.324669	0.000696

FOXN1	-0.95539	0.003879
FOXN2	0.29179	0.006124
FOXN3	-0.25388	0.000596
FOXO4	-0.52563	3.67E-05
FOXO6	-0.59384	0.025187
FOXP2	-1.18637	1.64E-06
FOXRED1	0.151919	0.040199
FPGT	-0.28462	0.001525
FPR1	0.386329	0.043258
FRAS1	-0.73831	1.15E-09
FRG1	-0.2853	0.031059
FRG1BP	-0.2941	0.014395
FRG2C	-2.88046	0.029969
FRK	-0.52326	2.05E-06
FRMD3	0.407241	0.028729
FRMD5	0.271422	0.011462
FRS2	-0.21702	0.00553
FSCN1	0.591076	0.003294
FSCN2	-0.91797	0.009469
FSD1	2.300387	0.018816
FSD2	-1.00759	0.011219
FSIP2	-0.48257	4.10E-05
FST	0.675321	0.000277
FSTL1	-0.21909	0.019929
FSTL4	-1.15965	3.13E-24
FTO	-0.14294	0.004928
FTSJ1	0.178302	0.034691
FTSJ3	0.197728	0.003611
FTX	-0.52378	1.71E-06
FUBP1	0.175443	0.005439
FUBP3	0.100472	0.026738
FUCA1	-0.30456	2.14E-05
FUOM	0.390809	0.018607
FURIN	0.376395	0.010159
FUS	0.222638	0.005248
FUT2	-0.30618	0.044088
FUT4	0.275307	0.044272
FUT8	0.191609	0.007722
FUT8-AS1	1.24486	0.016712
FXN	0.197064	0.008198
FXR2	0.290323	0.001854
FXYD3	-0.77412	0.000118
FXYD5	0.385293	0.000158
FXYD7	1.401149	0.038611
FYTTD1	-0.23635	0.000653
FZD1	-0.58965	1.02E-08
FZD10	0.542835	0.000215

FZD5	-0.28681	6.15E-05
FZD7	-0.21492	0.020478
FZD9	0.605526	0.017509
G0S2	0.616464	1.54E-05
G6PC3	0.362649	0.002802
GAB1	-0.45033	6.26E-05
GABARAPL	-0.68992	0.000209
GABARAPL	-0.13208	0.033123
GABRA3	-0.61931	0.000779
GABRP	0.341709	0.002469
GAD1	-0.62783	0.000891
GAGE12F	1.148663	0.017227
GAL	1.138128	5.01E-13
GAL3ST4	-0.44539	0.018946
GALC	-0.46544	5.02E-08
GALE	0.370815	0.000126
GALK2	0.14097	0.014337
GALNT1	-0.23507	0.002731
GALNT10	-0.23298	0.000313
GALNT11	-0.27736	1.70E-07
GALNT14	0.398275	1.35E-05
GALNT18	-0.22298	0.009276
GALNT2	-0.26198	0.000898
GALNT5	-0.30009	6.31E-07
GALNT6	-0.1204	0.014804
GAPDH	0.239013	0.001354
GAPDHP62	1.113936	0.005467
GAR1	0.216298	0.031357
GARS	0.199686	0.013182
GART	0.320582	1.57E-07
GAS5	0.237616	0.001323
GAS6-AS2	-0.25055	0.024874
GATA6	0.295468	0.007087
GATAD1	-0.26609	7.03E-05
GATAD2A	0.395281	9.61E-05
GATAD2B	0.167224	0.014049
GATS	-0.83731	8.36E-10
GATSL3	-0.45928	0.008958
GBA	-0.1811	0.029075
GBE1	-0.18495	0.017845
GBGT1	0.441259	0.016885
GBP1	-0.95124	2.31E-07
GBP1P1	-1.09685	4.34E-05
GBP2	-1.45721	4.59E-07
GBP4	-1.15557	0.007406
GBP5	-0.67888	0.038645
GBP6	-0.49154	0.000274

GCA	-0.46434	0.002717
GCAT	0.341452	0.011667
GCDH	0.273688	0.002094
GCFC2	0.197329	0.008371
GCH1	-0.4371	0.002614
GCN1	0.221993	0.000397
GCNT1	-0.31083	8.45E-07
GCNT4	-0.81139	3.87E-08
GDA	0.426424	0.000118
GDF11	0.593257	2.60E-06
GDF15	0.73006	0.049369
GDPD1	-0.65516	0.025787
GDPD2	-0.42959	0.032408
GDPD5	0.375327	0.025801
GEM	0.530234	6.96E-05
GEMIN4	0.356156	2.96E-05
GEMIN5	0.402697	5.82E-15
GEMIN7	0.225445	0.01745
GFM1	0.171952	0.000608
GFM2	-0.23489	0.001265
GFOD1	0.101813	0.038865
GFOD2	0.197593	0.019997
GFPT2	1.064919	8.55E-25
GFY	2.299338	0.04425
GGA1	0.295818	0.005092
GGACT	-0.70673	0.002119
GGCT	0.210581	0.000434
GGPS1	-0.19662	0.034255
GGT6	-0.8067	0.007791
GGT7	-0.31088	0.012859
GHc-857G6	-1.04008	0.026038
GHET1	-0.5379	0.000564
GHR	-0.65559	6.44E-05
GIF	-3.2812	0.044624
GIMAP6	0.420794	0.042162
GIMAP8	1.416711	0.010428
GINM1	-0.23555	0.00118
GINS3	0.179492	0.017222
GINS4	0.261684	0.002997
GIPR	-0.4408	0.024805
GIT1	0.435974	0.000151
GJA1	-0.51686	0.000158
GJA3	1.504617	2.88E-10
GJB2	0.812267	7.52E-31
GJB3	0.655448	1.22E-09
GJB4	0.814618	8.64E-13
GJB5	0.541298	2.61E-12

GJD3	-3.11746	0.03818
GK	0.704696	1.54E-08
GKAP1	-0.67002	0.003323
GLB1L2	0.668403	6.50E-05
GLDN	-0.47907	0.009473
GLIPR2	-0.2876	0.003662
GLIS3	-0.32496	0.016632
GLO1	0.170615	0.014682
GLOD4	0.181271	0.0002
GLP2R	-1.3649	0.00015
GLRB	-1.39411	0.001332
GLRX	-0.48839	0.000636
GLRX3	0.187802	0.000363
GLRX5	0.205362	0.013032
GLS	-0.29455	0.015042
GLT8D2	-0.49808	0.000528
GLTSCR2	0.400074	0.00885
GLUD1	0.300039	8.50E-08
GLYCTK	0.656605	0.000245
GMCL1	-0.19485	0.011348
GMDS	0.265768	0.001525
GMDS-AS1	-0.357	0.041537
GMEB2	0.23597	0.032801
GMIP	0.188756	0.049027
GMPR	-0.61746	0.007289
GMPR2	-0.17056	0.001888
GMPS	0.258738	0.00011
GNA12	0.293493	0.000175
GNA15	0.380835	0.000405
GNAI1	-0.22722	0.001317
GNAO1	-0.47291	0.010028
GNE	0.228574	0.047686
GNG11	0.865886	9.22E-05
GNG2	0.343671	0.025903
GNG5	0.222135	0.03068
GNL1	0.21367	0.006214
GNL2	0.252656	8.04E-06
GNL3	0.287951	4.41E-06
GNL3L	0.295383	0.000265
GNPDA1	0.198694	0.000311
GNPNAT1	0.238079	0.000188
GNPTAB	-0.42136	2.64E-07
GNS	-0.21112	1.34E-06
GOLGA1	-0.17375	0.001541
GOLGA2P5	-0.36473	0.000697
GOLGA6L1	-4.53318	0.000256
GOLGA6L5	-0.48447	0.002185

GOLGA6L9	-0.25145	0.014435
GOLGA8CP	-1.05629	0.026558
GOLGA8EP	-3.596	0.012038
GOLGA8M	-1.44361	0.036833
GOLGA8N	-0.80056	2.20E-10
GOLGA8R	-0.71184	0.005432
GOLGB1	-0.34014	0.000304
GOLIM4	0.116636	0.049747
GOLM1	0.21986	1.28E-05
GOPC	-0.40031	3.64E-08
GOT1	0.306626	1.31E-08
GOT2	0.189706	4.22E-05
GP6	-1.35664	0.000397
GPANK1	0.254167	0.025341
GPAT2	0.403926	7.58E-05
GPAT3	0.901051	5.62E-17
GPAT4	0.139718	0.012295
GPATCH4	0.253154	0.001432
GPHN	0.29692	7.40E-05
GPI	0.202657	0.003024
GPKOW	0.203893	0.002068
GPM6A	-0.76379	0.001542
GPN1	0.194226	0.001581
GPN3	0.173227	0.016115
GPNMB	-0.78947	9.93E-09
GPR1	-0.60354	2.94E-10
GPR107	-0.21367	0.000204
GPR132	-0.62428	0.005586
GPR135	-0.27974	0.02611
GPR137C	-0.39263	0.021583
GPR153	0.927863	1.47E-08
GPR155	-0.74143	1.88E-06
GPR158	-0.29216	0.000511
GPR161	-0.32813	7.41E-05
GPR176	0.253864	0.02327
GPR179	-1.10678	0.035272
GPR3	0.78303	0.007675
GPR50	0.960522	4.65E-12
GPR68	-0.31052	0.029447
GPR87	0.218352	0.007901
GPRASP2	-0.27809	0.005238
GPRC5B	0.211245	0.00999
GPRC5C	-0.31701	0.022666
GPRIN1	0.369819	0.004854
GPRIN2	-1.03287	1.19E-05
GPSM1	0.484125	0.032015
GPSM3	1.056917	1.11E-06

GPX3	0.302442	0.004444
GPX7	-0.4805	0.000188
GRAMD1A	0.362313	0.004802
GRAMD1C	-0.60393	0.000578
GRAMD2	-0.6213	4.91E-09
GRAMD3	-0.42307	1.54E-06
GRB10	0.399251	5.53E-07
GRB14	0.33989	0.029351
GRB2	0.102286	0.048898
GRB7	0.572061	2.97E-05
GREM1	-0.40479	0.001489
GRHL2	-0.20545	0.001371
GRIA1	0.429419	0.001756
GRIK2	-0.3556	0.001331
GRIN2A	-0.97073	0.040277
GRK5	0.60628	1.68E-10
GRK6	0.289729	0.024445
GRK7	-1.77605	0.009625
GRM1	-0.91738	0.005736
GRPEL1	0.401142	1.51E-10
GRSF1	0.175534	0.016626
GRWD1	0.3672	0.002367
GS1-24F4.2	-2.3473	0.044389
GS1-259H1	-0.66101	0.032418
GS1-304P7	-2.76959	0.041417
GS1-358P8	-0.81337	2.03E-06
GSAP	-0.81239	0.000215
GSDMB	-0.45097	0.000545
GSG2	0.416056	8.86E-05
GSK3A	0.281694	0.00555
GSK3B	-0.1429	0.0059
GSPT1	0.219794	2.12E-05
GSPT2	0.237756	0.000338
GSS	0.164129	0.010213
GSTA4	-0.33247	0.000209
GSTK1	-0.389	2.92E-06
GSTM2	-0.55302	1.79E-05
GSTO1	0.472199	1.65E-14
GTF2F1	0.224305	0.016376
GTF2H2B	0.293372	0.049543
GTF2H4	0.190324	0.008173
GTF2I	-0.19031	0.005218
GTF2IP12	-0.60653	0.000153
GTF2IP4	-0.28068	2.45E-05
GTF2IRD2B	-0.54908	3.16E-09
GTF3A	0.254931	1.31E-05
GTF3C2	0.207472	0.007675

GTF3C5	0.302517	0.002926
GTF3C6	0.195404	0.044669
GTPBP1	0.180006	0.019796
GTPBP4	0.272353	2.14E-06
GTSE1	0.34196	0.023132
GUCY1B2	-2.12738	0.001284
GUCY2C	-0.51477	0.034805
GXYLT2	-0.66986	4.44E-07
GYG2	-0.74838	0.041305
H19	-0.80633	0.000342
H2AFV	0.116342	0.014417
H2AFY	0.117383	0.019644
H2AFZ	0.269943	0.000189
H3F3B	0.128573	0.002586
H6PD	-0.28399	0.000268
HACD1	-0.33953	4.28E-07
HACD2	-0.57008	1.99E-17
HACD4	-0.1611	0.041059
HADH	-0.35824	3.54E-10
HAGH	-0.41759	0.002308
HAP1	-0.45788	0.00456
HARS	0.209966	0.000154
HAS2	0.635417	0.001536
HAS2-AS1	0.521015	0.044775
HAUS1	0.273221	0.018137
HAUS2	0.164981	0.009137
HAUS4	0.181172	0.000757
HAUS5	0.415379	0.002801
HAUS7	0.433306	4.67E-05
HAUS8	0.29095	0.002109
HAX1	0.154824	0.001695
HBE1	0.55129	0.04564
HBEGF	1.045006	1.51E-12
HBP1	-0.36144	0.007059
HCAR2	-0.74435	0.039833
HCFC1	0.305493	0.042959
HCG27	-0.67688	0.002366
HCK	-1.1985	0.010512
HCN3	-0.46166	0.04398
HCP5	-0.4765	7.07E-09
HDAC11	-0.2293	0.047277
HDAC2	0.151116	0.017857
HDAC3	0.194372	0.001687
HDAC7	0.237934	0.04759
HDAC9	0.415916	0.000114
HDDC2	0.174616	0.003602
HDDC3	0.256568	0.04955

HDGF	0.196749	0.003993
HDHD5	0.37487	0.000244
HEATR1	0.207787	0.003853
HEATR4	-0.63077	0.0001
HEATR5A	-0.30958	0.034233
HEBP2	-0.16229	0.001842
HECTD4	-0.2342	0.001887
HEG1	-1.15009	1.97E-15
HELLPAR	-0.34105	3.80E-05
HELLS	0.196597	0.015131
HELZ	-0.41219	1.64E-06
HEPHL1	0.390059	0.018983
HERC2	-0.38451	1.63E-14
HERC2P9	-0.31699	0.000269
HERC3	0.227043	0.003711
HERPUD2	-0.17648	0.003435
HES1	0.210358	0.009295
HES2	-0.65685	0.000142
HES6	0.604548	0.029762
HEXA	-0.14386	0.005556
HEXIM1	-0.37282	3.05E-06
HEY1	0.678376	0.006268
HEY2	0.546277	0.012807
HFE	-0.3451	7.28E-08
HGH1	0.389257	0.003663
HGS	0.324283	0.035448
HGSNAT	-0.36832	4.09E-08
HHAT	-0.41973	6.74E-05
HHIP	-0.94055	0.004895
HHLA1	0.550178	1.70E-10
HIBCH	-0.25235	0.004166
HIC2	0.383786	0.005765
HID1	-0.86194	0.029309
HIGD2A	0.262401	0.037031
HINT3	-0.18398	0.027351
HIST1H1C	-0.51102	1.35E-05
HIST1H2AC	-0.49952	2.82E-09
HIST1H2AE	-0.45713	0.012022
HIST1H2AG	-0.34329	0.003055
HIST1H2AI	-0.68877	0.004904
HIST1H2BC	-0.60095	4.85E-06
HIST1H2BD	-0.41529	0.000143
HIST1H2BE	-0.76825	0.002806
HIST1H2BF	-0.94165	0.000105
HIST1H2BG	-0.8343	3.19E-16
HIST1H2BN	-0.66994	4.89E-07
HIST1H3D	-0.38179	0.023137

HIST1H3E	-0.9484	1.68E-11
HIST1H3H	-0.77062	6.98E-07
HIST1H4E	-0.84634	4.97E-05
HIST1H4H	-1.1631	2.07E-09
HIST2H2BE	-0.60841	1.05E-08
HIST2H2BF	-0.55642	3.67E-05
HIST2H4B	-0.52827	6.00E-08
HIST4H4	-0.47303	0.009191
HIVEP2	-0.18309	0.023132
HIVEP3	0.252581	0.017627
HJURP	0.306337	0.0167
HK1	0.186599	0.010506
HK2	0.599366	4.18E-09
HKDC1	0.422359	0.00233
HLA-B	-0.39318	0.005365
HLA-DPB1	0.394048	0.040492
HLA-DRA	-1.76504	0.022387
HLA-F	-0.35296	0.040346
HLA-F-AS1	-0.29208	0.005073
HLCS	-0.36383	2.25E-09
HLX	0.620158	0.001548
HMBS	0.257264	0.001211
HMCES	0.17743	0.003136
HMCN1	-0.40691	0.020265
HMGA1	1.13051	6.65E-15
HMGA2	0.758345	7.20E-07
HMGB1	0.218369	0.012992
HMGB1P6	0.32239	5.85E-05
HMGCL	-0.20813	0.001288
HMGCS2	-3.34373	0.001176
HMGN2	0.253871	0.000293
HMGN3	-0.32029	3.45E-06
HMGN3-AS	-0.34144	0.009288
HMGN5	-0.27786	0.031737
HMGXB3	0.202841	0.001148
HMMR	0.309404	0.006363
HN1	0.193777	0.03559
HNF4A	1.287063	0.027734
HNRNPA1	0.270523	0.000167
HNRNPA1P	1.475313	0.034435
HNRNPAB	0.301138	3.52E-07
HNRNPC	0.166387	0.002924
HNRNPD	0.219086	0.000654
HNRNPDL	0.129906	0.002072
HNRNPF	0.277068	1.20E-13
HNRNPK	0.1565	0.005166
HNRNPL	0.315001	3.45E-07

HNRNPM	0.177265	0.011398
HNRNPR	0.168289	0.020302
HNRNPUL1	0.230183	0.008239
HOMER1	0.241362	0.000954
HOMER2	0.167757	0.007649
HOMEZ	-0.37743	2.69E-07
HOPX	0.715384	0.010328
HOTAIR	-0.46859	0.00511
HOTAIRM1	0.179943	0.020215
HOTTIP	-1.09422	0.001265
HOXA11-AS	-1.30893	0.00882
HOXA13	-0.36332	0.007557
HOXA4	-0.44475	0.02658
HOXA5	-0.41283	0.025064
HOXA-AS2	-0.54666	0.024423
HOXB3	0.200987	0.046392
HOXB8	-0.47583	0.040435
HOXC12	-1.3912	0.024456
HOXC13-AS	-0.29155	0.026994
HOXD10	-0.23906	2.13E-05
HOXD13	0.438644	0.005342
HOXD8	-0.27801	0.023941
HOXD9	-0.26722	0.040122
HPCAL1	0.292318	0.031094
HPDL	0.566638	0.013608
HPRT1	0.25202	0.00099
HPS3	-0.27443	5.05E-05
HR	0.446129	0.010646
HRAS	0.271127	0.022231
HRH1	0.289857	0.002441
HRH2	-0.66642	1.01E-05
HRK	-1.09397	0.006886
HS3ST1	1.295209	1.17E-15
HS3ST2	-0.46838	0.003258
HS3ST3A1	0.635738	0.001367
HS6ST1	-0.43946	0.001635
HS6ST2	0.637067	2.23E-08
HSD11B1	1.071678	0.001323
HSD11B2	0.417522	0.000951
HSD17B2	0.651757	0.00027
HSD17B4	-0.18831	0.008486
HSD17B6	-0.49281	0.00775
HSD3B7	-0.62484	0.003821
HSF1	0.382874	0.012301
HSFX1	-0.46906	0.029953
HSP90AA1	0.257608	0.001353
HSP90AB1	0.254027	0.000226

HSPA12A	-0.3984	1.03E-06
HSPA1A	0.289536	0.02716
HSPA4	0.212049	0.003651
HSPA4L	-0.32721	0.018142
HSPA9	0.254505	3.65E-06
HSPB1	-0.56695	0.007199
HSPB11	-0.19417	0.016254
HSPB3	-0.9734	9.82E-11
HSPB8	-0.4726	1.21E-06
HSPBP1	0.364208	0.019148
HSPD1	0.395913	1.37E-05
HSPE1	0.217051	0.026034
HSPH1	0.25222	0.027899
HTATIP2	-0.24375	0.000737
HTR1D	-0.4399	0.000822
HTR2C	-0.61812	0.001272
HTR7	0.595994	1.24E-07
HTRA1	-0.78711	4.02E-12
HUS1	0.224798	0.019876
HYAL1	-0.37821	0.000822
IAH1	-0.16013	0.036945
IARS	0.297421	1.18E-07
ICAM1	-0.47371	0.008216
ICK	-0.16537	0.01734
ICOSLG	-0.73206	2.35E-09
IDH1	-0.65749	2.65E-06
IDH3A	0.169783	0.017587
IDH3B	0.233287	0.017615
IDNK	-0.44954	0.006023
IDO1	-1.98249	0.018634
IER2	0.451493	0.001643
IER3	0.932557	1.48E-14
IFFO2	-0.41998	0.004157
IFI27L1	-0.19285	0.028678
IFI30	-0.23836	0.003995
IFI35	-0.4193	0.044582
IFIT5	-0.30882	0.034678
IFITM10	-0.50754	0.004184
IFNAR2	-0.22091	0.009108
IFNE	0.354804	0.036675
IFNGR2	0.303292	0.000121
IFNWP19	0.325391	0.025125
IFRD2	0.501156	2.29E-05
IFT43	-0.18268	0.017096
IFT80	-0.27064	0.000725
IFT81	-0.38352	9.43E-06
IGDCC4	0.728416	0.028638

IGF1R	0.111713	0.040173
IGF2	0.674857	0.022582
IGF2BP1	0.208469	0.015371
IGF2BP2	0.184638	0.001959
IGF2BP2-AS	1.086468	3.99E-05
IGF2R	-0.23451	0.000343
IGFBP1	0.922071	0.016324
IGFBP4	0.304883	0.017863
IGFBP5	-0.67761	0.004508
IGFBP7	-0.57519	1.08E-07
IGFL2-AS1	-0.41211	0.007188
IGHMBP2	0.249492	0.006673
IGIP	-0.57684	0.002312
IGSF1	-0.48791	0.001496
IGSF3	-0.30922	3.99E-05
IGSF9B	0.936225	0.002232
IKBKAP	0.134874	0.00469
IKZF2	-0.76939	2.49E-16
IKZF3	0.347073	0.012128
IL10RB	-0.23525	0.000385
IL11	1.174137	1.43E-24
IL11RA	-0.44287	0.009663
IL12A	0.412677	0.048466
IL13RA2	0.644715	0.000176
IL1A	0.548891	0.000131
IL1B	0.530313	0.001031
IL1R1	-0.51628	0.001209
IL1R2	0.925435	0.002834
IL1RAP	-0.16557	0.039826
IL1RL1	1.886944	9.22E-14
IL1RN	0.828387	5.72E-12
IL20	1.784179	7.32E-06
IL24	2.349054	1.95E-30
IL27RA	0.489693	1.46E-08
IL32	-1.25427	2.33E-12
IL37	1.700642	5.91E-06
IL3RA	-0.71692	0.000465
IL4R	0.235304	0.000467
IL6R	0.370221	3.22E-09
IL7	-0.89872	0.002068
IL7R	0.531548	6.81E-07
ILDR1	-1.03809	0.001833
ILF2	0.19969	0.000878
ILF3	0.355272	3.55E-07
ILKAP	0.245525	0.000202
IMMT	0.117951	0.028162
IMP4	0.421207	1.02E-05

IMPA2	-0.22517	0.035121
IMPDH1	0.330923	0.010166
IMPDH2	0.432496	2.42E-21
INAFM2	-0.50733	9.35E-07
INCA1	-0.3934	0.004458
INCENP	0.344021	0.006044
INF2	0.322568	0.047581
ING1	0.206417	0.032066
ING4	-0.47444	4.23E-08
INHA	-0.72033	0.023087
INHBA	0.668588	1.94E-08
INHBB	-0.74126	1.34E-05
INO80E	0.310847	0.001715
INPP1	-0.30063	0.043134
INPP4A	-0.16742	0.015981
INPPL1	-0.31248	0.001486
INSC	0.86305	0.042828
INSIG2	-0.24886	0.004475
INSL3	-1.18503	0.027271
INSR	-0.21842	0.008001
INTS10	0.111244	0.046538
INTS11	0.297223	0.00361
INTS13	0.321039	0.000626
INTS14	0.159342	0.006324
INTS4	0.258297	3.23E-05
INTS6L	-0.29206	0.009506
IPO11	0.23908	0.000102
IPO4	0.61436	3.57E-07
IPO5	0.30307	9.71E-07
IPO5P1	-0.31297	0.020873
IPO7	0.188357	0.024701
IPO9	0.155522	0.001498
IPP	-0.25238	0.001532
IPPK	0.119886	0.039829
IQCA1	-0.63212	1.07E-16
IQCF3	-1.30765	0.030917
IQCH-AS1	-0.36399	0.024614
IQGAP2	-0.42878	0.048014
IRAK1	0.423292	0.000578
IRAK1BP1	-0.26319	0.001749
IRAK2	0.257336	0.004459
IRAK3	-0.36	0.000131
IRF2	-0.18321	0.015706
IRF3	0.262208	0.013435
IRF4	1.003872	1.08E-21
IRGQ	-0.239	0.000193
IRS2	0.316159	0.005425

ISCA1	0.111628	0.047454
ISCA1P1	0.867929	0.001888
ISCU	0.148487	0.010906
ISM1	0.712769	0.000177
ISOC2	0.347143	0.044183
ISPD	-0.43508	0.003277
ISY1-RAB43	0.24652	0.008449
ITCH	0.17855	0.033866
ITFG1	-0.3204	1.53E-05
ITFG2	0.165103	0.028735
ITGA1	-0.32035	0.000215
ITGA11	-0.70718	8.49E-05
ITGA2	0.378965	0.001666
ITGA3	0.288602	0.004825
ITGA4	-0.28002	9.63E-06
ITGA5	0.269014	0.008869
ITGA6	0.176444	0.006793
ITGA7	0.861549	8.70E-05
ITGAV	-0.44293	4.45E-05
ITGB1BP1	0.215851	0.003117
ITGB2	0.423323	0.004184
ITGB3	-0.89145	1.57E-06
ITGB6	-0.49238	3.39E-07
ITGB8	-0.52426	2.92E-06
ITGBL1	-0.28643	5.65E-07
ITM2A	-0.62157	0.001704
ITM2B	-0.44866	0.000214
ITPA	0.254944	0.00124
ITPK1	0.402866	0.000251
ITPR1	0.18045	0.016017
ITPR3	0.368756	9.12E-07
ITPRIP	0.396369	7.32E-05
ITPRIPL1	0.624206	0.021887
ITSN2	-0.1733	0.048681
IVL	-1.22579	0.003469
IYD	-1.1593	0.016059
JADE2	0.30015	0.000261
JAG1	0.547581	1.26E-14
JAK3	-0.4433	0.045412
JAKMIP3	-0.37057	0.002702
JARID2	0.271318	1.19E-08
JKAMP	-0.1866	0.005283
JPH2	-0.79272	3.43E-08
JPX	-0.20029	0.033563
JRKL	-0.25024	0.044251
JUND	-0.64568	0.040011
KANK1	-0.2075	0.001991

KANSL1L	-0.33684	0.01244
KANSL2	0.234499	0.003273
KARS	0.311657	4.35E-10
KAT14	-0.17594	0.01336
KAT2A	0.332661	0.00159
KAT2B	-0.35749	0.000178
KAT5	0.223034	0.009771
KAT6A	0.126893	0.018133
KAT7	0.176294	0.001735
KATNAL1	-0.2199	0.031225
KATNB1	0.434767	0.002092
KB-1460A1	-0.83032	0.035317
KB-1836B5	-0.94362	0.037176
KBTBD3	-0.64409	0.000344
KC6	-0.38216	0.003144
KCMF1	0.220918	8.67E-05
KCNA2	-0.81279	4.31E-15
KCNAB1	-0.61116	0.001434
KCNAB2	0.274661	0.020914
KCND3	-0.88382	0.002823
KCNE4	-0.55016	0.025332
KCNF1	-0.6551	0.013081
KCNG1	0.644066	0.000131
KCNH3	0.944825	0.000801
KCNJ8	1.075708	0.04366
KCNK1	0.361229	0.000138
KCNK3	-0.93717	0.027982
KCNK5	0.712368	1.41E-09
KCNK7	0.977057	0.031939
KCNMA1	0.409339	0.000189
KCNN2	-1.79708	0.015672
KCNN4	0.388943	0.017297
KCNQ1OT1	-0.23795	0.001713
KCNQ5	0.39596	3.08E-05
KCNS3	-0.60238	4.98E-08
KCNV1	-0.5316	0.015265
KCTD10	-0.1379	0.007889
KCTD12	-0.2771	0.016863
KCTD15	0.207817	0.014217
KCTD17	0.387469	0.019911
KCTD18	-0.2998	2.95E-05
KCTD20	-0.207	8.98E-05
KCTD21	-0.34091	0.001876
KCTD5	0.237282	0.00373
KCTD6	-0.29444	0.009918
KCTD7	-0.28438	0.002628
KCTD8	1.408823	0.003107

KDELC2	-0.24708	0.000411
KDM1A	0.140522	0.000954
KDM4C	-0.24315	0.035704
KDM5A	-0.14892	0.049638
KDM5B	-0.36425	4.86E-08
KDM5C	0.152264	0.025356
KDM6A	-0.24857	0.000479
KDM7A	0.492607	0.027585
KDSR	0.2008	9.77E-05
KHDRBS1	0.171634	0.001499
KHDRBS3	0.573362	5.89E-07
KHNYN	-0.1545	0.000275
KHSRP	0.3735	0.002302
KHSRPP1	-1.08934	1.00E-06
KIAA0100	0.157312	0.000516
KIAA0232	-0.37695	0.000715
KIAA0319L	-0.43115	4.62E-07
KIAA0895	-0.51325	1.22E-05
KIAA1109	-0.22632	0.023444
KIAA1147	-0.48455	7.22E-06
KIAA1161	-0.26029	0.000476
KIAA1217	-0.22889	0.00453
KIAA1328	-0.19644	0.026857
KIAA1462	0.380038	0.00027
KIAA1524	0.236087	0.020421
KIAA1549	0.271541	0.000119
KIAA1549L	0.767445	5.33E-05
KIAA1614	-0.71511	0.000251
KIAA1644	-0.23444	0.006994
KIAA1683	-1.54733	3.34E-05
KIAA1841	-0.27189	0.004324
KIAA1958	0.283634	0.016202
KIDINS220	-0.24555	0.001202
KIF11	0.204246	0.038731
KIF12	-0.66401	0.005011
KIF13A	-0.20806	0.002629
KIF14	0.301494	0.007025
KIF15	0.417952	0.000226
KIF17	1.079783	1.60E-11
KIF18A	0.268096	0.014283
KIF18B	0.486162	0.000445
KIF1A	1.8468	1.23E-05
KIF1B	-0.16448	0.040064
KIF1C	0.199889	0.025962
KIF20A	0.303443	0.00036
KIF21B	0.454565	0.000264
KIF22	0.396675	8.04E-05

KIF24	0.231343	0.032786
KIF28P	-1.00258	0.015373
KIF2C	0.40681	7.68E-06
KIF4A	0.262093	0.003462
KIFC1	0.355404	0.008091
KIT	-1.22322	2.76E-05
KITLG	0.297277	0.002908
KL	3.416699	0.026227
KLC2	0.303399	0.01242
KLC3	0.390518	0.020671
KLF16	0.478314	0.006524
KLF4	0.329412	0.023094
KLF5	0.332508	0.001259
KLF7	0.165732	0.033177
KLF8	-0.45707	7.45E-07
KLHDC1	-0.64256	0.005825
KLHDC2	-0.16515	0.006896
KLHDC4	0.230408	0.029522
KLHDC8B	-0.85949	1.20E-06
KLHDC9	-0.62692	0.026203
KLHL13	-0.25958	9.44E-05
KLHL18	0.134956	0.028299
KLHL21	0.206667	0.035677
KLHL23	-0.25146	0.02447
KLHL24	-0.59357	0.000127
KLHL3	-0.34207	0.012109
KLHL33	-0.45598	0.017382
KLHL36	-0.25149	1.73E-05
KLHL41	-1.59909	0.041361
KLHL42	-0.2049	0.004246
KLK13	1.342254	0.000367
KLK5	-0.57568	0.001678
KLRC4-KLRI	-3.93016	0.008694
KMO	-0.82679	1.73E-10
KMT2B	0.348967	0.004069
KMT2C	-0.2507	0.0094
KMT2E	-0.21268	0.005728
KMT5A	0.209022	0.000445
KMT5B	-0.14752	0.017781
KNOP1	0.208589	0.002719
KNSTRN	0.220114	0.011824
KPNA2	0.264932	0.000925
KPNA2P3	-0.64037	0.035935
KPNA5	-0.18984	0.021276
KPNB1	0.219371	9.59E-05
KPTN	0.287071	0.014215
KRBA1	-0.33249	0.009666

KRBA2	-0.5531	0.000264
KRCC1	-0.57271	4.40E-08
KREMEN1	-0.18685	0.035088
KRI1	0.167545	0.016879
KRR1	0.404048	3.31E-07
KRT16P2	1.013897	0.017869
KRT17	-0.73193	0.000291
KRT18	0.481223	1.01E-09
KRT5	-0.20833	0.01983
KRT75	-1.76285	0.000769
KRT8	0.272479	0.018113
KRT80	-0.27329	0.009466
KRTAP10-2	-1.14167	0.049311
KRTAP5-AS	-1.27477	0.016835
KRTCAP3	0.261498	0.020578
KTN1-AS1	0.226809	0.007271
KXD1	0.215262	0.010727
KYAT1	0.318029	0.023881
L1CAM	-0.92941	1.05E-14
L3MBTL1	-0.76446	1.83E-07
L3MBTL4	-0.71085	0.000563
LA16c-358I	0.31375	0.008425
LA16c-380I	0.721879	0.002766
LA16c-390I	0.792842	0.00663
LACC1	-0.27658	0.015804
LACE1	-0.2185	0.020029
LACTB	-0.28355	0.000868
LACTB2	-0.16446	0.015597
LAD1	0.54031	2.70E-05
LAMA3	0.383805	2.79E-11
LAMA4	0.890685	2.55E-11
LAMB3	0.344926	0.00115
LAMC2	0.351216	1.47E-05
LAMC3	0.537218	0.001937
LAMP1	-0.16488	0.014066
LAMP2	-0.29858	0.000437
LAMP3	-0.65718	0.00312
LAMTOR3	-0.18363	0.027254
LANCL1	-0.21509	0.001515
LANCL2	0.152143	0.0369
LAPTM5	0.597844	1.20E-06
LARGE1	-0.55559	0.004921
LARP1	0.140939	0.010163
LARS	0.204264	0.006647
LARS2	0.15474	0.008432
LAS1L	0.315462	5.14E-09
LASP1	0.170911	0.035802

LAYN	-0.45357	3.42E-08
LBH	-0.76261	7.86E-13
LBR	0.18195	0.035833
LCK	1.235339	1.03E-12
LDB3	-1.2541	6.32E-06
LDHB	0.338272	3.36E-05
LDLR	0.373643	8.41E-06
LDLRAD3	0.441912	2.55E-12
LDLRAD4	-1.56217	2.05E-27
LDOC1L	-0.25237	0.004463
LEFTY2	-2.5368	0.009544
LENG1	0.395308	0.002628
LENG9	0.407076	0.047893
LEPROT	-0.33133	3.00E-07
LEPROTL1	-0.17701	0.02196
LETM1	0.260848	0.001024
LETM2	0.41875	2.82E-09
LGALS3	0.335441	0.000414
LGALS8	-0.26938	0.00214
LGALSL	-0.51948	0.000877
LGMN	-0.22667	0.012849
LGR4	-0.3379	2.69E-07
LGR5	-2.24013	0.000108
LHFPL2	0.463457	3.77E-15
LHX1	0.647679	0.005646
LHX2	0.877496	0.031023
LHX9	1.042641	0.036251
LIF	0.444856	0.04264
LIG1	0.354756	0.000833
LIG3	0.240256	1.90E-05
LIG4	-0.45798	7.41E-05
LIMA1	0.15268	0.010434
LIMCH1	-0.85897	1.30E-13
LIMK2	-0.30755	2.32E-05
LIMS2	-0.55003	0.004979
LIN28B	0.538602	0.003499
LINC00205	-0.3571	0.0011
LINC00294	-0.40739	1.66E-05
LINC00314	1.231261	0.026658
LINC00337	0.901349	0.016662
LINC00342	-0.40011	0.008733
LINC00346	-0.55764	6.43E-08
LINC00365	-0.50719	0.044434
LINC00472	0.622483	0.00083
LINC00484	-1.79438	0.021935
LINC00504	-0.33911	0.000678
LINC00511	-0.34046	0.000323

LINC00519	-0.47247	0.000474
LINC00520	1.199398	0.000967
LINC00578	-1.89037	0.022309
LINC00626	-1.69207	0.002902
LINC00638	-0.3941	0.022271
LINC00640	-1.07295	0.00029
LINC00641	-0.29141	0.003843
LINC00649	-0.32263	4.00E-06
LINC00665	-0.36836	0.041863
LINC00667	-0.2042	0.00016
LINC00674	-0.56877	0.00074
LINC00675	-3.31844	0.042276
LINC00707	0.92463	2.64E-21
LINC00863	-0.42773	9.39E-05
LINC00865	-0.60074	6.63E-09
LINC00882	-0.4264	0.019589
LINC00884	-0.69459	0.00729
LINC00885	-1.19399	0.0045
LINC00886	-0.43825	1.17E-05
LINC00888	-0.33236	3.96E-06
LINC00893	-0.64051	0.002444
LINC00898	-0.96013	0.024006
LINC00909	-0.29026	0.002832
LINC00941	0.51379	3.18E-06
LINC00954	-0.83215	0.009674
LINC00958	0.169398	0.020552
LINC00960	-0.8831	1.05E-05
LINC00963	-0.20804	0.002754
LINC01003	-0.68476	4.73E-05
LINC01010	-0.50968	0.00972
LINC01011	-0.5539	0.010237
LINC01021	-0.59541	9.09E-05
LINC01089	-0.31032	0.014706
LINC01116	0.268113	0.029996
LINC01123	0.351413	0.040514
LINC01133	-0.67489	0.007843
LINC01138	-0.26779	0.019748
LINC01139	-0.30004	0.015958
LINC01154	2.917649	0.032079
LINC01204	0.684586	0.020017
LINC01220	-1.37204	0.016807
LINC01232	-0.24125	0.015379
LINC01285	-0.90953	0.000573
LINC01297	-0.45783	0.03757
LINC01322	0.455092	0.000658
LINC01341	-1.07387	0.000188
LINC01345	-2.80456	0.006589

LINC01347	-0.3307	0.023902
LINC01348	-0.53824	0.039136
LINC01355	-0.41643	0.007791
LINC01356	0.464108	0.00202
LINC01376	-0.5283	0.01051
LINC01410	-0.28353	0.02615
LINC01451	-1.22621	0.034815
LINC01460	-0.71973	0.014976
LINC01468	0.352532	0.007388
LINC01474	2.165009	0.046322
LINC01508	-0.61774	0.048163
LINC01537	0.695917	0.001136
LINC01551	1.278283	0.012941
LINC01588	-0.31872	0.002912
LINC01605	0.331664	0.000308
LINC01609	-0.48467	0.005303
LINC01649	-1.55203	0.020345
LINC01653	-0.96733	0.031426
LINC01719	-0.45818	0.006136
LINC01722	-0.42975	0.025094
LINC01727	-1.52156	0.009785
LINC01748	-0.39409	0.0008
LINC01762	-0.68787	0.048161
LINC01833	-0.70719	0.000815
LINC01873	-0.30018	0.006521
LINC01943	-0.678	0.048932
LINC01979	-1.23335	0.011731
LINC02015	-1.08207	8.06E-05
LINC02031	-1.31176	0.043226
LINC02035	-0.76949	1.24E-07
LINC02068	-1.51818	9.98E-05
LINC02154	1.061303	7.69E-05
LINC02178	0.959478	6.76E-05
LINC02186	-0.95369	0.045202
LINC02187	-1.6323	0.000155
LINC02198	-1.83506	0.022401
LINP1	0.272413	0.004741
LIPA	-0.37038	5.66E-05
LIPE	0.451707	0.048235
LITAF	-0.28266	0.00089
LL0XNC01-[illegible]	-0.37471	0.028867
LL22NC03-[illegible]	-0.29917	0.000297
LLGL1	0.349322	0.017593
LLNLR-222[illegible]	-0.38567	0.04238
LLNLR-307[illegible]	2.781687	0.04467
LMAN2L	-0.19782	0.00031
LMBRD1	-0.30584	0.00056

LMBRD2	-0.3968	7.15E-05
LMLN	-0.22473	0.001938
LMNA	0.316365	0.029248
LMNB2	0.383974	0.00347
LMO4	-0.21938	4.28E-05
LMO7	-0.5538	8.20E-08
LMOD1	-1.95931	3.33E-15
LOXL1	-0.52511	0.00801
LOXL3	0.243636	0.048463
LPAR1	0.266292	7.24E-05
LPAR3	0.251864	0.001279
LPAR6	-0.52348	3.78E-05
LPCAT1	0.196299	0.002059
LPCAT4	0.569297	3.04E-08
LPGAT1	-0.16969	0.019243
LPP	-0.41785	1.17E-05
LPP-AS2	-0.52748	0.005907
LPXN	0.751365	4.67E-07
LRCH3	-0.12897	0.027248
LRP11	-0.23923	0.029227
LRP5L	0.243737	0.014446
LRPPRC	0.26902	0.000197
LRR1	0.196756	0.021828
LRRC17	-0.59355	7.81E-05
LRRC27	-0.42527	2.52E-05
LRRC3	-0.2455	0.040379
LRRC32	-1.38482	5.49E-07
LRRC37A16	-0.42795	0.003733
LRRC37A3	-0.4543	3.70E-05
LRRC37B	-0.20364	0.023514
LRRC55	-3.02821	0.016884
LRRC57	-0.1471	0.031947
LRRC59	0.234589	1.53E-05
LRRC7	-1.17674	3.05E-39
LRRC75A-A	0.175742	0.001451
LRRC8A	0.447105	2.00E-05
LRRC8C	0.611559	6.66E-09
LRRC8D	0.107393	0.03242
LRRK1	0.199273	0.000381
LRRN1	-0.74985	0.048897
LRWD1	0.609079	9.31E-05
LSG1	0.176994	5.66E-05
LSM12	0.178805	0.006927
LSM12P1	0.306252	0.00326
LSM2	0.288206	0.011029
LSM5	0.165223	0.02496
LSM6	0.228527	0.014557

LSM7	0.329067	0.001679
LSP1	-0.35253	0.014038
LTA4H	0.155501	0.010662
LTB	-1.0725	1.56E-08
LTBP1	0.504908	1.18E-15
LTV1	0.194898	0.007097
LUC7L	0.268984	0.000132
LUM	-1.52243	5.72E-08
LURAP1L	-0.64678	0.001922
LVCAT1	-0.99414	7.18E-05
LY6K	0.349049	0.00132
LY75	-0.27643	0.025016
LYAR	0.549294	2.80E-10
LYN	-0.49546	4.10E-05
LYNX1	-0.61276	0.020674
LYPLA2	0.249909	0.020567
LYPLAL1	-0.24182	0.003395
LYRM4	0.185262	0.011829
LYRM9	-0.4686	0.019534
LZIC	0.128977	0.047523
LZTS1	0.506359	0.000169
MAB21L3	-0.50017	1.28E-05
MACC1	-0.67287	0.000234
MACROD2	-0.64441	0.000234
MAD1L1	0.484809	0.000511
MAD2L1	0.35421	4.46E-05
MAD2L2	0.436568	0.000976
MAFA-AS1	-1.28329	0.017103
MAFB	-1.20038	7.64E-07
MAFF	0.746804	1.19E-12
MAGED1	-0.17621	0.024296
MAGT1	-0.21287	0.005364
MAIP1	0.239856	5.60E-05
MAK16	0.266129	0.000844
MALAT1	-0.28392	0.01241
MALL	1.109519	2.22E-76
MALSU1	0.17208	0.001552
MAMDC2	-0.44703	0.000722
MAMDC2-A	-0.71515	0.004151
MAN1A2	-0.15097	0.039204
MAN2B2	-0.33722	0.003833
MAN2C1	0.273419	0.041366
MANBA	-0.57168	8.58E-11
MANSC4	-2.60383	0.046724
MAOB	1.055788	3.15E-13
MAP10	-0.4034	0.006659
MAP1B	-0.20097	0.009199

MAP1LC3A	-0.45466	0.00442
MAP2K1	0.330222	7.91E-11
MAP2K3	0.376138	0.000447
MAP2K4	0.15053	0.01678
MAP2K5	-0.16531	0.034817
MAP2K6	-0.82309	0.001691
MAP2K7	0.327051	0.000516
MAP3K13	-0.20586	0.000789
MAP3K14	0.490613	0.000239
MAP3K2	-0.20305	0.028419
MAP3K20	0.14915	0.020858
MAP3K4	-0.1499	0.005808
MAP3K5	-0.39782	0.00941
MAP3K8	-0.33602	0.031004
MAP4K4	0.48096	1.19E-09
MAP6	-0.71349	0.002946
MAP6D1	0.250397	0.038389
MAP7D2	-1.15033	0.000259
MAPK10	-0.59531	0.000104
MAPK13	-0.24596	0.042467
MAPK14	-0.19924	8.07E-06
MAPKAPK2	0.149166	0.030087
MAPKAPK3	0.545083	6.10E-13
MAPKAPK5	0.157147	0.000992
MAPKAPK5	0.14217	0.048402
MAPKBP1	0.317999	5.47E-05
MAPRE2	0.301221	1.17E-05
MAPRE3	-0.26657	0.006552
2-Mar	-0.60381	2.91E-05
2-Mar	-0.45379	0.003257
4-Mar	1.018324	1.09E-06
8-Mar	-0.16249	0.0173
9-Mar	-0.38312	0.005771
MARCKS	-0.46447	2.91E-10
MARCKSL1	0.44572	0.001774
MARK1	-0.49039	1.76E-13
MARK2P16	3.165082	0.039378
MARK4	0.280672	0.040705
MARS	0.220326	0.000535
MARS2	0.30914	5.79E-05
MARVELD2	-0.22399	0.000739
MARVELD3	-0.6501	2.35E-09
MASP1	-0.67136	0.034889
MATN1-AS	0.316657	0.048917
MATN2	-1.33965	1.01E-10
MAVS	-0.07561	0.035992
MBD1	0.215751	0.001402

MBD6	0.299055	0.022882
MBNL1-AS1	-1.03798	9.30E-13
MBNL2	-0.27143	0.019916
MBOAT1	0.209306	0.005662
MBOAT2	0.577084	4.95E-16
MBOAT7	0.295879	0.049133
MBTPS1	-0.19468	0.000509
MCAM	-0.48041	4.84E-06
MCAT	0.269026	0.013797
MCCC1	-0.11892	0.039518
MCCC2	0.12073	0.02036
MCEE	-0.22033	0.019291
MCF2L	0.446654	0.034392
MCF2L2	-0.31059	0.028915
MCFD2	0.134088	0.048817
MCIDAS	0.620404	0.008588
MCM10	0.413256	1.23E-06
MCM2	0.322645	0.003303
MCM3AP	0.114464	0.013874
MCM4	0.299367	1.17E-05
MCM5	0.279068	0.014016
MCM6	0.103769	0.03647
MCM7	0.36924	1.19E-06
MCM8	0.1834	0.025875
MCMBP	0.234104	2.59E-08
MCMDC2	-0.49992	0.014905
MCOLN3	-0.20768	0.040593
MCRIP1	0.333136	0.029125
MCRS1	0.214785	0.011036
MCUB	-0.38312	0.012486
MDC1	0.291523	0.001935
MDFI	0.55093	0.006536
MDH2	0.224876	0.017485
MDN1	0.232803	1.36E-05
ME1	-0.23748	0.00497
ME2	0.231047	0.000435
ME3	0.931991	0.000554
MEA1	0.223556	0.003064
MED1	0.150481	0.035686
MED15	0.266739	0.02566
MED22	0.269972	0.032644
MED24	0.180358	0.037668
MED27	0.183818	0.000331
MED8	0.188914	0.000314
MEG3	0.670928	2.63E-17
MEG8	4.210708	0.002032
MEGF9	-0.47579	1.48E-08

MEIS1	-0.25639	0.003457
MEIS2	-0.23593	0.012716
MELK	0.227796	0.005068
MEMO1	0.240892	0.00015
MEMO1P1	-1.28558	0.004016
MERTK	0.712767	2.86E-06
MEST	-0.33229	0.000244
MET	0.218446	0.000952
METAP1	0.217634	0.000566
METAP1D	-0.23218	0.003523
METAP2	0.222732	0.003146
METRNL	0.431709	0.039123
METTL1	0.261827	0.003134
METTL12	0.276656	0.014757
METTL16	0.276629	2.96E-09
METTL17	0.129831	0.003168
METTL21B	0.237956	0.01372
METTL25	-0.47211	7.67E-06
METTL7A	-1.13819	6.58E-17
METTL9	0.170842	0.001544
MFAP3L	-0.43394	0.037876
MFAP5	-0.30918	0.049882
MFN2	0.205192	0.000372
MFNG	0.450168	0.030055
MFSD1	-0.30587	3.32E-06
MFSD10	0.462358	0.016418
MFSD11	-0.1865	0.005387
MFSD14A	-0.16292	0.028057
MFSD2A	0.568251	4.54E-08
MFSD6	-0.87185	3.82E-09
MFSD8	-0.16782	0.039184
MFSD9	-0.22788	0.010745
MGAM	-1.03933	0.000105
MGAT4A	-0.66196	0.027212
MGAT5	0.218713	1.34E-05
MGC32805	1.452479	1.60E-06
MGST2	-0.17358	0.027424
MGST3	-0.14261	0.012704
MICAL2	0.303249	4.00E-05
MID1	-0.20669	0.000373
MID1IP1	0.337964	0.023757
MID2	-0.17293	0.015138
MIDN	0.440117	0.002727
MIGA1	-0.16745	0.033827
MIGA2	0.276279	0.015193
MIIP	0.596335	7.00E-05
MINDY1	-0.31114	0.001089

MINPP1	-0.13666	0.017629
MIPEP	0.22844	0.001362
MIR100HG	0.661153	1.09E-06
MIR193BH(	-0.41617	0.00615
MIR210HG	-0.40359	0.028556
MIR22HG	-0.55543	9.17E-07
MIR27B	-1.31358	0.001146
MIR31HG	0.362623	0.004769
MIR331	-2.03394	0.017491
MIR4292	0.616225	0.03133
MIR4435-2	0.332486	0.000263
MIR4458H(	-0.1938	0.044927
MIR503HG	-0.46667	0.018766
MIR570	-0.33451	0.021709
MIR635	-0.7981	0.046886
MIRLET7BH	-0.55839	0.000551
MIRLET7D	-0.56558	0.027139
MIS18A	0.255045	0.000133
MIS18BP1	0.178093	0.032913
MISP	0.423259	0.00498
MITF	-0.36594	0.011899
MKI67	0.255238	0.015185
MKL2	-0.14134	0.007078
MKNK2	0.507233	0.000133
MLKL	0.280617	1.28E-05
MLLT1	0.366836	0.014308
MLLT3	-0.27816	0.001874
MLST8	0.533082	0.002834
MLXIPL	-0.5555	0.00529
MLYCD	-0.33092	0.000611
MMACHC	0.210336	0.008203
MME	-0.87907	5.89E-06
MME-AS1	-1.33617	0.022121
MMP12	-1.56342	0.008383
MMP13	-0.5206	1.86E-05
MMP14	-0.28382	0.001582
MMP15	-0.53995	0.000445
MMP2	-0.30407	0.010171
MMP24	-0.56571	0.016053
MMP28	-0.44969	0.029911
MMP3	1.479932	4.93E-10
MMP7	-0.98055	0.000111
MMP9	1.568207	5.28E-05
MMS19	0.327821	5.13E-10
MMS22L	0.186719	0.013269
MOAP1	-0.33283	1.28E-05
MOB1A	-0.13967	0.049921

MOB3A	0.228438	0.032737
MOB3C	-0.20614	0.035255
MOCS1	0.305482	0.009698
MOK	0.543728	6.90E-07
MON1A	0.392868	0.008669
MON1B	-0.13892	0.023206
MORC2	0.098139	0.032576
MORN4	-0.2248	0.006559
MOSPD1	-0.3554	0.000448
MOV10L1	-0.45044	0.003812
MOXD1	-1.11648	1.17E-08
MPDU1	0.273536	0.003239
MPI	0.139621	0.022128
MPP2	0.506521	0.004896
MPP3	0.309514	0.008146
MPP4	-1.30411	0.002087
MPP6	0.557349	3.83E-12
MPP7	-0.31464	6.50E-05
MPPE1	-0.29943	5.73E-06
MPRIP	0.529725	2.01E-11
MPV17	0.184362	0.019993
MPZL1	-0.1056	0.022936
MR1	-0.55576	5.57E-14
MRAS	-1.09254	1.98E-11
MRFAP1	0.100975	0.02755
MRFAP1L1	-0.12388	0.048437
MRGBP	0.291402	0.00149
MRM3	0.315949	0.00145
MROH7	-1.06418	0.00248
MROH8	-0.72342	0.001755
MRPL1	0.268093	0.000211
MRPL10	0.31645	0.000409
MRPL11	0.221456	0.027236
MRPL12	0.396268	0.016915
MRPL14	0.169786	0.008929
MRPL15	0.22315	8.62E-05
MRPL16	0.28478	1.88E-06
MRPL17	0.24654	0.010942
MRPL18	0.197043	0.000321
MRPL2	0.34377	0.000832
MRPL20	0.22667	0.00178
MRPL21	0.25502	0.000638
MRPL22	0.16791	0.019189
MRPL23	0.317306	0.011566
MRPL24	0.203388	0.000996
MRPL27	0.230985	0.000251
MRPL28	0.318072	0.008112

MRPL3	0.270694	3.15E-05
MRPL32	0.214899	0.001053
MRPL36	0.372866	0.000953
MRPL37	0.33029	0.000311
MRPL38	0.331415	0.030811
MRPL39	0.18101	0.037082
MRPL4	0.434134	0.027669
MRPL42	0.191151	0.020513
MRPL45	0.191315	0.000572
MRPL46	0.193186	0.00185
MRPL47	0.150099	0.048939
MRPL48	0.124594	0.031836
MRPL49	0.17727	0.015671
MRPL53	0.224704	0.042556
MRPL58	0.291679	1.50E-05
MRPL9	0.289038	4.11E-05
MRPS10	0.239978	5.32E-06
MRPS11	0.198415	0.031004
MRPS12	0.362752	0.018343
MRPS15	0.186992	0.006198
MRPS16	0.13369	0.022848
MRPS18A	0.30873	0.001757
MRPS18B	0.303192	3.81E-05
MRPS2	0.549823	5.09E-05
MRPS22	0.194811	0.001201
MRPS23	0.225044	0.002217
MRPS25	0.246934	0.000174
MRPS27	0.202648	2.29E-06
MRPS28	0.20031	0.003672
MRPS30	0.249073	1.08E-05
MRPS35	0.194675	0.000684
MRPS5	0.201623	0.001247
MRPS6	0.402341	1.46E-09
MRPS7	0.237692	0.003405
MRRF	0.176755	0.002119
MRTO4	0.433148	3.23E-07
MRVI1	-0.87789	0.001037
MSANTD3	0.186569	0.00716
MSC-AS1	-0.22388	0.005515
MSH2	0.205828	0.008378
MSH5	0.330656	0.000222
MSI2	-0.27337	0.000151
MSRB2	-0.44296	0.000594
MSRB3	-0.47526	2.98E-09
MST1R	0.474491	6.21E-05
MSTO1	0.221402	0.021598
MSX1	0.358701	0.022778

MT1E	0.645834	5.12E-07
MT1L	1.018581	6.39E-10
MT1X	0.406437	2.52E-05
MT2A	1.031482	1.07E-12
MTA1	0.389543	0.006131
MTA2	0.200017	0.000895
MTAP	0.503583	3.60E-11
MTCH2	0.116363	0.040941
MT-CO1	-0.18941	0.018698
MTERF2	-0.23655	0.003388
MTERF3	0.225036	0.014787
MTFP1	0.46896	0.000182
MTFR1L	-0.22042	0.002052
MTFR2	0.288363	0.000483
MTG1	0.327058	0.002962
MTHFD1	0.255012	3.90E-06
MTHFD1L	0.482094	8.71E-14
MTHFD2	0.231535	0.025126
MTMR11	0.49346	1.28E-05
MTMR3	-0.23526	0.023074
MT-ND1	0.211351	0.040264
MT-ND2	0.30851	0.003336
MTND4P23	-3.31201	0.018308
MT-ND5	-0.20837	0.000772
MT-ND6	-0.24479	0.020208
MTOR	0.121501	0.007046
MTPAP	0.159203	0.035811
MTRF1	-0.20454	0.013137
MT-RNR2	-0.31967	2.65E-07
MTRR	0.197414	0.001792
MTSS1	0.388466	0.000252
MT-TA	-0.43311	0.000668
MT-TG	-0.41204	0.017083
MT-TN	-0.4565	0.021282
MT-TP	-0.33568	0.004333
MTURN	0.306655	0.016379
MTUS1	-0.3314	1.98E-05
MTUS2	-1.14932	9.20E-08
MUC1	-0.96784	0.014048
MUC16	-1.76661	0.000148
MUC2	2.105361	0.011588
MUC20	-1.02902	0.000272
MUC4	-1.14677	0.001337
MUCL1	2.974817	0.025023
MUS81	0.232078	0.040158
MUTYH	0.402846	0.000557
MVP	-0.28341	0.042805

MXD4	-0.32996	0.014598
MXI1	-0.15463	0.035111
MXRA5	-1.17758	1.18E-23
MXRA5Y	-1.15509	9.30E-06
MXRA7	-0.1786	2.07E-05
MYB	0.459021	0.013062
MYBBP1A	0.547452	8.92E-06
MYBL2	0.270605	0.008274
MYBPC2	-0.535	0.048083
MYC	1.048231	6.72E-21
MYCL	-0.93356	3.95E-12
MYEF2	0.355783	0.045139
MYEOV	0.730904	5.27E-06
MYH10	0.173584	0.009554
MYH14	-0.43565	0.007765
MYH15	1.228371	3.63E-08
MYH16	0.730068	4.29E-11
MYH3	-0.39605	0.000189
MYL12A	-0.37447	3.66E-06
MYL2	-0.9897	9.15E-05
MYL6	-0.43373	8.83E-09
MYL6B	0.216681	0.029505
MYL7	-2.2241	5.67E-07
MYL9	-1.38446	6.78E-15
MYLIP	-0.52679	0.005563
MYLK	-0.64138	9.08E-10
MYLK3	-0.42358	0.019031
MYO10	0.604106	8.10E-34
MYO16	-0.52954	0.000283
MYO16-AS	-1.16334	0.000234
MYO19	0.317365	6.49E-06
MYO3B	-0.97436	7.96E-21
MYO5C	-0.58891	0.018628
MYO7B	0.611743	7.38E-05
MYOM1	-1.21904	5.21E-05
MYOM2	-0.56059	5.03E-05
MYOSLID	0.358934	4.31E-05
MYPN	0.452408	0.011335
MYRF	0.631178	3.76E-05
MYRIP	-3.4842	0.013842
MZF1-AS1	-0.42069	0.035623
N4BP2L1	-0.66754	0.000165
N4BP2L2	-0.22395	0.005185
N4BP3	0.315349	0.017832
NAA10	0.303746	0.002445
NAA15	0.292285	4.28E-05
NAA25	0.168208	0.024759

NAALAD2	-0.61157	0.01593
NAB2	0.652852	4.37E-10
NABP1	-0.55809	5.14E-08
NACA	0.202151	8.13E-05
NACAD	0.469329	0.01082
NACC1	0.323283	0.026474
NADSYN1	-0.35422	0.000741
NAE1	0.159661	0.012764
NAF1	0.255415	0.001353
NAGS	0.659408	5.77E-06
NAIF1	0.210484	0.014212
NAIP	-0.30313	0.024928
NALCN	-0.41355	0.000286
NAMPT	0.225858	0.033565
NANOS1	-0.28387	0.025302
NANS	0.244143	5.33E-05
NAP1L1	0.281152	3.85E-05
NAP1L2	-0.36657	3.38E-05
NAP1L4	0.118251	0.005115
NAP1L5	-0.49988	0.008263
NAPB	-0.23886	0.012663
NAPEPLD	-0.26232	0.014003
NARS2	0.279991	1.60E-05
NASP	0.19498	0.003913
NAT1	-0.28929	0.014482
NAT10	0.398215	8.32E-11
NAT9	0.223318	0.005131
NATD1	-0.22681	0.043422
NAV2	-0.39886	1.02E-07
NAV3	1.055934	8.08E-31
NBDY	-0.1912	0.003989
NBEAL1	-0.39408	0.001863
NBEAL2	0.308457	0.03656
NBL1	0.339636	0.040551
NBPF12	-0.29344	4.26E-05
NBPF14	-0.14224	0.023932
NBPF19	-0.20808	0.007015
NBPF20	-0.19972	0.033549
NBR2	-0.30468	0.005077
NCAPD2	0.300473	5.20E-05
NCAPD3	0.290832	5.72E-08
NCAPG	0.322225	0.001139
NCAPG2	0.193811	0.007107
NCAPH	0.377671	4.20E-08
NCAPH2	0.476696	3.80E-05
NCBP1	0.188355	0.000869
NCBP3	0.124794	0.038475

NCEH1	0.155664	0.041215
NCF2	-0.35813	0.004944
NCK1-AS1	-0.24909	0.040646
NCKAP5	-0.46308	0.000173
NCKIPSD	0.314118	0.007037
NCL	0.301082	3.99E-05
NCOA1	-0.34673	0.000298
NCOA2	-0.24498	2.12E-07
NCSTN	-0.23387	0.000291
NDC1	0.320241	0.00011
NDC80	0.279931	0.022838
NDE1	0.181325	0.004085
NDFIP1	-0.15402	0.033379
NDFIP2	-0.30593	0.002484
NDRG2	0.172783	0.003523
NDRG3	-0.12745	0.00921
NDST1	-0.2853	0.00132
NDUFAB1	0.224967	0.018665
NDUFAF2	0.25982	0.003845
NDUFAF4	0.245207	0.012276
NDUFAF8	0.351701	0.049472
NDUFB1	-0.12692	0.031112
NDUFB8	0.194107	0.01595
NDUFB9	0.220705	6.63E-06
NDUFC1	0.184389	0.029445
NDUFS2	0.09634	0.033737
NDUFS3	0.23989	0.003248
NDUFV1	0.279108	0.01142
NDUFV2	0.14976	0.001963
NEBL	-0.3143	0.014013
NECTIN1	0.361659	0.000773
NECTIN3	0.343867	0.005476
NEDD1	-0.18716	0.046345
NEDD4L	0.200772	0.017442
NEDD9	-0.34212	2.06E-07
NEFL	-0.9151	1.56E-13
NEIL3	0.200533	0.007821
NEK11	-0.22904	0.028935
NEK2	0.2625	0.014468
NEK9	-0.13928	0.001764
NELFA	0.313924	0.033808
NELFB	0.296758	0.023205
NELFCD	0.273575	2.31E-07
NEMP2	0.401808	1.79E-09
NEO1	0.482826	7.94E-06
NES	0.530188	1.88E-05
NET1	0.194807	0.003322

NETO2	0.235045	0.00028
NEURL1	1.306379	4.84E-08
NEURL1B	-0.62736	1.59E-15
NEURL3	-0.797	0.014926
NEURL4	0.315086	0.000986
NEUROG2	-1.47937	0.018478
NEXN	-0.74601	9.02E-12
NFAM1	-0.71655	0.006344
NFATC2	0.85016	3.99E-15
NFE2L3	0.190496	0.012098
NFKBIE	0.28739	0.012281
NFRKB	0.158133	0.0212
NFS1	0.217331	0.000905
NFX1	0.195453	0.001176
NGDN	0.215076	0.000299
NGEF	0.901793	4.69E-11
NGFR	0.790971	2.75E-06
NHEJ1	0.159486	0.017499
NHLH2	-0.91816	4.05E-11
NHLRC3	-0.42646	3.63E-06
NHP2	0.331161	0.000973
NHSL1	0.168871	0.012556
NHSL2	0.318568	2.41E-05
NICN1	-0.34113	0.012239
NID2	0.418516	0.000255
NIF3L1	0.165026	0.016466
NIFK	0.18858	0.000534
NIFK-AS1	-0.48067	0.007976
NIN	0.190721	0.005988
NINJ1	-0.49663	0.009212
NIP7	0.270252	0.000962
NIPA1	-0.23274	0.02707
NIPAL1	0.401063	1.78E-05
NIPAL2	-0.20165	0.042692
NIPSNAP3A	-0.27803	2.29E-05
NKD2	0.482126	0.041262
NKRF	0.235797	0.008155
NKX2-2-AS1	-4.12565	0.012683
NLE1	0.568745	4.98E-12
NLN	0.223937	0.000754
NLRP10	1.020014	7.61E-08
NLRP3	0.537849	0.000952
NLRP6	-1.27341	0.005302
NLRP7	-0.67499	0.001392
NME1	0.455088	1.52E-08
NME2	0.290854	0.000755
NME9	-0.80437	0.022872

NMI	-0.27979	0.013265
NMRK1	-0.27195	0.000904
NNMT	-0.4578	6.18E-05
NNT	-0.18084	0.002581
NOB1	0.433148	6.29E-15
NOC2L	0.454742	2.46E-05
NOC3L	0.326076	0.001432
NOC4L	0.457665	0.019123
NOD1	0.17847	0.036897
NOD2	-0.64971	0.000234
NOL10	0.223515	0.000304
NOL11	0.208079	0.002415
NOL12	0.290817	0.002436
NOL6	0.55562	6.91E-08
NOL7	0.225858	7.34E-05
NOL8	0.130854	0.042568
NOL9	0.143071	0.021436
NOLC1	0.417982	1.52E-14
NOM1	0.14773	0.044524
NONO	0.154327	6.39E-05
NOP10	0.224061	4.52E-06
NOP14	0.359329	6.08E-13
NOP16	0.468703	2.38E-11
NOP2	0.360963	1.89E-07
NOP56	0.354487	3.04E-05
NOP58	0.226448	0.000253
NOP9	0.124091	0.00625
NORAD	-0.25733	0.000237
NOS2P3	0.785123	0.047417
NOV	0.277261	0.000598
NOXA1	-0.69588	0.032091
NPB	-2.09558	0.020274
NPC1	0.253432	0.014101
NPC1L1	-1.63541	0.023307
NPHP3	-0.15133	0.038052
NPHP4	0.258796	0.01934
NPIPA2	-1.41099	0.002331
NPL	-0.39775	0.02707
NPM1	0.381209	3.12E-05
NPM2	-0.56488	0.022336
NPM3	0.58652	1.54E-08
NPNT	-0.86091	0.012894
NPR3	0.91651	2.95E-14
NPRL3	0.363964	0.003194
NPSR1-AS1	-0.68041	0.041625
NPTN-IT1	0.332512	0.010709
NPTX1	0.925927	0.003116

NPTXR	-0.42587	8.51E-05
NQO1	-0.16709	0.000247
NR1D1	0.648988	2.31E-06
NR2C2AP	0.291962	0.000411
NR2E1	-0.54365	1.64E-06
NR3C1	0.206903	0.01617
NR4A1	0.501513	0.000814
NRBP2	-0.45203	0.000219
NRCAM	-0.46077	7.67E-06
NREP	-0.39218	1.07E-06
NRG1	0.27527	0.000114
NRG3	0.866984	0.044836
NRG4	-0.53762	5.33E-06
NRGN	0.999937	0.004034
NRIP3	0.251494	0.006207
NRM	0.248934	0.041345
NRP2	0.33141	0.000236
NRXN2	-0.29818	0.021048
NSD1	-0.15866	0.001222
NSD2	0.321225	1.06E-06
NSMF	0.424028	0.005052
NSUN2	0.275595	2.12E-10
NSUN5	0.255574	0.021997
NT5C2	-0.36656	1.38E-05
NT5DC1	-0.18093	0.001571
NT5DC3	0.359879	4.90E-07
NT5E	0.619332	1.61E-12
NTF3	0.557009	0.001697
NTHL1	0.427825	0.01437
NTMT1	0.482192	1.34E-08
NTN1	-0.83938	0.001129
NTN4	-0.54992	2.44E-11
NTN5	-0.85113	0.015651
NTNG1	0.497752	0.00242
NTRK1	1.054501	0.020818
NTSR1	2.218346	0.000812
NUB1	-0.26105	0.001694
NUBP1	0.205839	0.00793
NUCB2	-0.18831	0.024653
NUDC	0.311753	0.000912
NUDCD1	0.21267	0.019166
NUDCD2	0.20243	0.001338
NUDT1	0.394624	0.000249
NUDT11	0.191853	0.035283
NUDT15	0.314271	4.32E-07
NUDT16	-0.32538	2.75E-05
NUDT21	0.139227	0.033225

NUDT4P1	1.483783	0.014446
NUDT4P2	0.265758	0.004902
NUDT6	-0.35934	0.020748
NUDT7	-0.25352	0.040075
NUF2	0.185507	0.033872
NUFIP1	0.243656	0.00522
NUMBL	0.362152	0.030564
NUP107	0.214038	0.002911
NUP153	0.198621	0.000305
NUP155	0.220585	0.000514
NUP160	0.17988	0.006092
NUP188	0.358317	7.78E-09
NUP205	0.23428	0.000535
NUP214	0.135651	0.019039
NUP35	0.314859	4.71E-05
NUP37	0.165282	0.02101
NUP50	0.12271	0.020216
NUP58	0.131342	0.029135
NUP62	0.239275	0.029312
NUP62CL	0.541005	7.79E-06
NUP85	0.157817	0.002287
NUP88	0.315941	7.23E-09
NUP93	0.372485	1.94E-10
NUP98	0.177333	3.67E-05
NUPR1	-0.50885	0.005504
NUTF2	0.186636	0.008236
NUTM2D	-0.42926	0.002162
NWD1	-0.86689	0.000186
NXF1	0.182197	0.002609
NXPE3	-0.30277	2.45E-09
NXT1	0.253146	0.006336
NYNRIN	-0.44354	0.009876
OACYLP	-2.79318	0.021597
OAF	0.789164	5.67E-10
OAZ1	0.225772	0.01042
OCIAD1	-0.12534	0.037512
OCIAD2	0.350246	0.000223
OCLN	-0.55753	0.01943
ODAM	-2.1267	0.000914
ODC1	0.801113	2.32E-18
ODF2	0.318201	4.90E-08
OGDHL	0.779424	0.035721
OIP5	0.306021	0.000514
OIP5-AS1	-0.23095	0.001484
OLA1	0.21225	0.005179
OLFML2A	-0.32533	0.005009
OLFML2B	-0.6812	0.002286

OLFML3	-0.77618	8.22E-08
OLMALINC	0.534077	1.86E-07
OLR1	-1.03992	0.00357
OPHN1	-0.21108	0.007106
OPN4	-3.45349	0.010796
OPRD1	-0.60666	0.032383
OPTN	-0.30114	0.000527
OR11H4	-0.75053	0.04642
OR2A1-AS1	-0.39583	0.006765
OR2A9P	-0.56944	0.005953
OR4A14P	1.793311	0.03423
OR7E14P	1.837432	0.009049
OR8R1P	-1.15856	0.016983
ORAI2	0.372536	0.000935
ORAI3	-0.61695	7.29E-06
ORAOV1	-0.11868	0.041406
ORC1	0.435273	6.97E-05
ORC4	-0.21526	0.00253
ORC6	0.155037	0.021901
ORMDL3	-0.26815	7.40E-07
OS9	-0.18877	0.001457
OSBP2	0.264816	0.048208
OSBPL10	0.19567	0.000276
OSBPL1A	-0.16305	0.0179
OSBPL6	0.322506	0.005701
OSGEP	0.1987	0.000482
OSGEPL1	-0.18617	0.046866
OSTM1-AS1	-1.61034	0.016699
OTUD1	-0.37892	0.018071
OVCH2	-0.66279	0.000292
OVGP1	-0.88536	0.003897
OVOL1	0.732007	0.002551
OXA1L	0.129655	0.025041
OXNAD1	0.2846	0.005568
OXSR1	0.209935	0.001748
OXTR	0.439795	0.005966
P2RX4	-0.48749	2.21E-05
P2RX5	0.790179	8.22E-07
P2RX6	-0.72689	0.000367
P2RX6P	-2.20997	0.026726
P2RX7	-0.64983	5.45E-08
P2RY1	-0.31239	0.040297
P2RY6	-0.57188	0.000881
P3H2	-0.24572	0.000901
P4HA2	-0.22332	0.008791
P4HTM	-0.54551	0.009994
PA2G4	0.355286	5.41E-06

PABPC1	0.221648	1.04E-06
PABPC1L	0.451661	3.74E-05
PABPC4	0.333509	5.44E-07
PADI1	0.634051	0.000871
PAF1	0.158873	0.029902
PAFAH1B1	-0.12209	0.004876
PAFAH1B3	0.40231	0.011688
PAFAH2	-0.25393	0.000432
PAG1	0.687874	9.46E-18
PAGR1	0.390924	0.015459
PAICS	0.375816	8.75E-07
PAK1	0.151561	0.007631
PAK1IP1	0.34195	2.45E-07
PAK2	-0.13543	0.04994
PAK3	0.504955	0.006169
PAK4	0.399267	0.042038
PALD1	0.471164	0.035393
PALLD	-0.8677	1.94E-23
PALM	-0.89958	0.006528
PALM2-AK/	-0.19263	0.031355
PALMD	-0.78083	2.28E-09
PAM	-0.57973	4.29E-07
PAM16	0.374926	0.004761
PAMR1	-3.1052	3.29E-12
PANK4	0.167594	0.032303
PAOX	-0.30371	0.040005
PAPD4	-0.16448	0.031243
PAPLN	-0.9852	1.86E-10
PAPPA2	0.530749	0.009162
PAPPA-AS1	-0.98088	0.020533
PAQR5	0.608721	6.72E-09
PAQR7	-0.32466	0.001096
PARD3B	-1.01721	0.005283
PARD6B	0.387097	0.026785
PARL	0.137265	0.00872
PARP1	0.175563	0.003108
PARP14	-0.6133	0.035628
PARP1P1	3.560917	0.008555
PARP2	0.149768	0.035027
PARP3	-0.29833	0.004294
PARP4	-0.15188	0.010527
PARP8	-0.19794	0.016097
PARPBP	0.227832	0.040452
PARVB	0.267743	0.004791
PASK	0.284995	0.000539
PATJ	-0.27118	0.001041
PATL1	0.08883	0.039356

PATL2	-0.4697	0.004395
PAX6	-0.29976	0.000742
PAX8-AS1	-0.19264	0.000207
PAX9	0.588123	6.45E-09
PAXBP1	0.280121	0.001638
PAXIP1-AS1	-0.39016	0.004327
PAXIP1-AS2	-0.50125	1.38E-05
PBDC1	0.138306	0.028727
PBLD	-0.34256	0.000223
PBX1	-1.06773	4.34E-10
PBX2	0.196266	0.010579
PBX3	0.499153	1.50E-10
PBXIP1	-0.58262	9.87E-08
PC	0.518116	6.92E-06
PCAT14	-0.35229	0.007962
PCAT7	-0.50848	3.66E-05
PCBP1	0.19614	0.010775
PCBP2	0.171369	0.000428
PCBP3-OT1	1.241922	0.014718
PCCB	0.182212	0.000465
PCDH1	0.463641	1.39E-06
PCDH10	-0.29675	0.00834
PCDH18	-0.643	0.013376
PCDH7	-0.35258	0.001819
PCDHAC2	0.326516	0.014895
PCDHB10	-0.72476	0.00106
PCDHB12	-0.40332	0.008042
PCDHB13	-0.59792	0.004189
PCDHB14	-0.7108	2.22E-10
PCDHB6	-0.45488	0.003209
PCDHB9	-0.83867	7.84E-06
PCDHGA1	-0.85336	1.50E-08
PCDHGA4	-0.39106	0.024718
PCDHGB1	-0.47459	0.000378
PCDHGC5	0.778728	1.75E-07
PCF11	-0.15501	0.036571
PCGF5	-0.26356	0.012838
PCGF6	0.18914	0.010945
PCID2	0.256284	0.000396
PCLAF	0.278763	0.015089
PCLO	-0.35188	0.00012
PCMT1	0.151015	0.001527
PCMTD1	-0.41913	0.000115
PCMTD2	-0.33216	1.56E-07
PCNA	0.236755	4.60E-06
PCNT	0.212373	0.001899
PCOLCE2	0.223235	0.009273

PCOTH	0.626764	0.000194
PCSK6	-0.25115	0.006694
PCTP	-0.26516	0.00073
PCYOX1	-0.45739	3.67E-15
PCYOX1L	-0.30885	0.003631
PCYT1A	-0.13146	0.011669
PDCD11	0.37616	2.15E-08
PDCD1LG2	-0.4069	0.002354
PDCD2	0.180019	0.000678
PDCD2L	0.406989	1.90E-05
PDCD5	0.199833	0.000528
PDE12	0.172674	0.003934
PDE1A	-0.85033	0.017727
PDE2A	0.352134	0.045872
PDE3A	-1.02904	0.001207
PDE5A	-0.69706	2.86E-05
PDE8B	-0.33201	0.007388
PDE9A	0.949436	0.001661
PDGFB	-0.30796	0.010596
PDGFC	-0.41472	1.46E-08
PDGFRA	0.712157	0.004053
PDHA1	0.193545	0.000574
PDIA3	-0.12963	0.005576
PDIA3P1	-0.29092	0.004688
PDK3	-0.26617	0.002464
PDLIM1	-0.36002	0.000547
PDLIM2	0.575974	0.000764
PDP2	0.187961	0.00485
PDRG1	0.151497	0.013103
PDS5B	0.215812	0.013206
PDSS1	0.234632	0.002043
PEA15	0.160622	0.001272
PEAK1	-0.34983	0.000286
PEAR1	0.513393	1.01E-09
PECR	-0.28848	0.040856
PEF1	0.188569	0.009616
PELP1	0.406083	0.000202
PEMT	0.534311	0.002167
PER1	0.457211	0.001605
PER2	0.22114	0.003804
PERP	-0.39031	2.12E-07
PES1	0.39878	1.40E-07
PEX1	-0.29679	0.000252
PEX10	0.32881	0.016387
PEX11B	-0.17147	0.044472
PEX13	-0.35819	9.47E-05
PEX2	-0.29873	0.000106

PEX5	0.241519	1.85E-07
PF4	1.596807	0.048838
PFAS	0.668615	1.05E-07
PFDN1	0.114963	0.036739
PFDN2	0.339926	3.84E-07
PFKM	0.447725	2.85E-11
PFKP	0.318401	0.000637
PGAM5	0.406532	1.61E-09
PGAP1	-0.28539	0.005062
PGAP2	0.200339	0.013554
PGAP3	-0.32799	0.005087
PGBD1	-0.19818	0.033076
PGF	0.94291	2.74E-05
PGGT1B	-0.16366	0.048389
PGM2L1	-0.27856	0.00879
PGPEP1	-0.36875	0.003779
PGRMC2	-0.27657	3.22E-05
PHACTR3	0.571114	0.011943
PHACTR4	-0.23501	0.000186
PHB	0.318913	5.71E-06
PHB2	0.355544	6.63E-05
PHBP12	1.932919	0.011129
PHC1	0.334367	7.89E-05
PHC1P1	0.356174	0.046883
PHC2	0.466805	7.29E-13
PHF10	-0.17804	0.006581
PHF11	-0.35291	0.001557
PHF19	0.473901	9.68E-06
PHF21A	-0.19307	0.006175
PHF21B	0.910297	0.007231
PHF5A	0.330994	2.38E-05
PHGDH	0.24334	0.039602
PHKA2	0.325459	4.14E-08
PHKB	-0.34502	8.61E-05
PHKG1	0.516432	0.022701
PHLDA1	0.615856	4.61E-16
PHLDB2	-0.32489	0.011294
PHLPP2	-0.17498	0.014774
PHOSPHO2	-0.33634	0.048286
PHRF1	0.336418	0.02578
PHTF1	-0.18179	0.043532
PHYH	-0.37604	1.33E-07
PI15	-1.04178	0.00056
PI4K2B	-0.26278	0.005332
PI4KAP1	0.595024	4.74E-06
PI4KAP2	0.437208	0.00289
PID1	0.504956	9.32E-07

PIF1	0.425709	0.02143
PIGB	-0.18834	0.048077
PIGCP1	-0.36567	0.003331
PIGL	0.2283	0.000952
PIGN	-0.22565	0.000651
PIGP	-0.1841	0.030315
PIGV	-0.18666	0.016329
PIGX	-0.19219	0.003566
PIGY	0.89076	0.017286
PIGZ	-0.52701	0.000187
PIH1D1	0.264519	0.001012
PIK3C2A	-0.18477	0.027439
PIK3C2B	-0.45013	0.001838
PIK3CB	-0.22062	0.005356
PIK3IP1	-0.72533	3.48E-13
PIK3R1	-0.18824	0.008974
PIK3R3	-0.32108	0.013352
PIM1	0.709355	1.83E-09
PIM2	0.262288	0.016574
PINK1-AS	-0.19492	0.014427
PINX1	0.348159	6.26E-05
PIP4K2A	0.115446	0.049611
PIP5K1A	0.107809	0.016039
PISD	0.296305	3.66E-05
PITPNA-AS1	0.405373	0.012731
PITPNC1	0.568981	8.30E-13
PITRM1	0.176763	0.000253
PJA1	0.248321	6.39E-07
PJA2	-0.22543	0.039802
PKD2	-0.2077	0.016098
PKDREJ	-0.72844	0.003101
PKHD1	-0.77109	0.002048
PKMYT1	0.489488	0.026735
PKP2	0.267949	1.01E-08
PKP4	0.143437	0.001285
PLA2G1B	-2.56837	0.040556
PLA2G4A	0.53055	1.38E-06
PLA2G4C	-0.72792	1.36E-06
PLA2G4F	-0.66137	0.025568
PLA2R1	-0.55959	4.57E-10
PLAA	0.140576	0.011852
PLAU	0.298438	0.002777
PLAUR	0.463584	0.000113
PLB1	-0.329	0.013223
PLBD1-AS1	-0.72385	0.008434
PLBD2	-0.16836	0.011916
PLCB1	-0.46302	0.000563

PLCB2	0.849978	0.000352
PLCB4	-0.48686	0.000402
PLCE1	-0.40423	0.001044
PLCL1	-1.18392	0.000884
PLD1	-0.7	6.89E-09
PLD6	0.387954	0.000186
PLEC	0.430027	0.046626
PLEK2	0.671327	8.55E-13
PLEKHA1	-0.27123	0.001601
PLEKHA2	-0.28756	3.00E-05
PLEKHA6	0.347143	0.015829
PLEKHB1	-0.72665	0.020179
PLEKHF2	-0.3042	4.79E-06
PLEKHG1	-0.39952	0.00035
PLEKHG2	0.254213	0.030088
PLEKHG3	0.63201	2.70E-09
PLEKHG4	0.55784	0.002987
PLEKHG5	0.638986	2.44E-05
PLEKHH1	-0.26617	0.012276
PLEKHM3	-0.25249	0.013901
PLEKHO1	-0.33663	0.001212
PLIN3	0.276141	0.002253
PLK1	0.540842	7.47E-05
PLK2	-0.13989	0.046318
PLK3	0.708931	1.11E-11
PLK4	0.30612	1.99E-05
PLOD2	-0.44419	0.001293
PLPP1	-0.67499	8.75E-16
PLPP3	-0.2343	0.038684
PLPP4	-0.58214	1.17E-05
PLPP5	0.1225	0.018739
PLPP6	-0.32481	0.001497
PLPPR5	0.991055	0.017918
PLRG1	0.132876	0.017577
PLS1	0.490421	0.021333
PLS3-AS1	-0.74498	0.016107
PLSCR3	0.398793	0.002268
PLSCR4	-0.86817	3.13E-16
PLXDC2	-0.98921	1.48E-14
PLXNA2	0.525208	2.51E-05
PMF1	0.222188	0.007904
PMFBP1	-0.21334	0.01231
PMP22	0.322793	0.031445
PMPCA	0.277777	0.000593
PMS2P6	-0.48745	0.002032
PNKP	0.37741	0.019885
PNMA1	0.223167	0.001275

PNO1	0.324805	1.38E-07
PNP	0.471623	2.51E-16
PNPO	0.195423	0.01002
PNPT1	0.18917	0.010015
PNRC1	-0.30122	0.025235
POC1A	0.215158	0.047092
PODNL1	-0.7507	0.000269
PODXL	0.492001	2.42E-06
POLA1	0.236749	7.93E-05
POLA2	0.201252	0.01509
POLD1	0.448631	0.024635
POLD2	0.503417	1.11E-06
POLDIP2	0.171322	0.026094
POLDIP3	0.207881	0.000126
POLE	0.337477	0.000963
POLE3	0.242177	2.55E-05
POLE4	0.249743	0.018053
POLG	0.32564	3.40E-06
POLI	-0.27747	0.001509
POLR1A	0.222119	0.000437
POLR1B	0.42684	3.57E-12
POLR1C	0.382294	8.43E-10
POLR1D	0.111335	0.023853
POLR1E	0.455444	1.82E-09
POLR2C	0.178322	5.08E-06
POLR2E	0.278476	0.045516
POLR2F	0.226045	0.013219
POLR2H	0.308345	9.97E-08
POLR2I	0.210697	0.025392
POLR2J	0.225375	0.018111
POLR3A	0.218763	5.51E-07
POLR3C	0.116964	0.04116
POLR3D	0.279065	0.000802
POLR3E	0.298644	0.000203
POLR3G	0.711011	8.79E-16
POLR3GL	-0.17907	0.03006
POLR3K	0.377922	3.07E-05
POLRMT	0.478402	0.006758
POM121C	0.212724	0.014153
POMGNT2	0.388131	0.013596
PON2	-0.20312	0.001227
POP1	0.20226	0.001701
POP5	0.153717	0.033532
POP7	0.332315	0.041112
POR	0.250581	0.03998
POT1-AS1	-0.45045	0.002434
POU2AF1	-0.85618	3.77E-05

POU2F2	0.843127	0.000625
POU6F2	-0.59175	0.003691
POU6F2-AS	-1.04453	0.010845
PP14571	-0.9339	0.011801
PPAN	0.463012	0.001293
PPARA	-0.32418	7.58E-06
PPARD	0.297764	0.021356
PPARG	0.517002	1.77E-09
PPARGC1A	-1.73166	4.12E-17
PPARGC1B	0.202757	0.039024
PPAT	0.352462	9.31E-06
PPCDC	0.277583	0.006271
PPFIBP1	0.188437	0.004886
PPIA	0.140957	0.010374
PPIC	-0.51059	4.92E-09
PPIE	0.113387	0.012157
PPIF	0.312372	4.66E-07
PPIH	0.216876	0.000949
PPIL1	0.17345	0.000424
PPIP5K1	-0.16576	0.000851
PPM1A	-0.14957	0.040318
PPM1B	-0.22404	0.021064
PPM1F	0.379136	0.004838
PPM1G	0.329994	1.02E-08
PPM1H	-0.78191	0.000101
PPM1K	-0.52206	0.003128
PPM1L	-0.41257	0.003892
PPME1	0.124515	0.027379
PPP1CC	0.202566	0.01187
PPP1R11	0.172053	0.010818
PPP1R15A	0.544021	4.34E-08
PPP1R16A	0.449104	0.04331
PPP1R18	0.347655	1.79E-05
PPP1R26	0.252247	0.037152
PPP1R3B	-0.45011	0.00518
PPP1R3D	-0.17451	0.043612
PPP1R8	0.127644	0.007436
PPP2R1A	0.264568	0.007002
PPP2R2B	-1.4095	1.08E-05
PPP2R2D	0.140877	0.005671
PPP2R3A	-0.25271	0.00232
PPP2R5B	-0.25472	0.024431
PPP2R5C	-0.10549	0.020123
PPP2R5D	0.161128	0.04053
PPP4R1	0.200596	9.78E-07
PPP4R4	-0.13587	0.04301
PPP5C	0.34792	1.92E-05

PPP6R1	0.375339	0.00415
PPRC1	0.48109	4.44E-08
PPT1	-0.0855	0.026747
PPT2	0.301978	0.000998
PQBP1	0.347267	0.000179
PQLC3	-0.38381	3.34E-06
PRAP1	-2.62348	0.005219
PRC1	0.230754	0.00011
PRCP	-0.25352	0.000221
PRDM8	0.741065	5.83E-05
PRDM9	1.111444	0.021019
PRDX4	0.146513	0.010778
PREB	0.19618	0.015092
PRELID1	0.374618	0.000565
PRELID3A	0.352231	0.017482
PREX1	0.321826	0.000646
PRICKLE2	-0.44665	3.99E-07
PRIM1	0.358843	8.56E-06
PRIM2	0.175939	0.028156
PRKAB2	-0.23549	0.000979
PRKAR1B	0.276739	0.025137
PRKAR2A	0.104198	0.047074
PRKCA	0.19724	0.002478
PRKCI	-0.16359	0.039496
PRKCSH	0.203029	0.017927
PRKDC	0.212497	3.95E-06
PRKG1	-0.37958	0.017272
PRMT1	0.393699	3.64E-05
PRMT3	0.387594	8.68E-06
PRMT5	0.363435	4.21E-10
PRMT7	0.230604	0.02214
PRMT9	-0.21663	0.016787
PRNP	0.109709	0.029336
PROC	-0.36307	0.023554
PROCR	0.84755	2.02E-17
PRODH	-0.99764	0.001419
PROM2	-0.5727	0.000611
PROS1	-0.49342	2.36E-05
PROSC	0.118061	0.019302
PROSER1	0.155782	0.006718
PROSER2	0.341095	0.008247
PRPF19	0.260414	0.002165
PRPF31	0.259727	0.001799
PRPF38A	0.180688	0.023236
PRPF4	0.284827	1.65E-06
PRPF6	0.153181	0.023135
PRPF8	0.231557	1.60E-05

PRPS1	0.228721	0.00013
PRPS2	0.303245	3.05E-09
PRR11	0.291496	0.001537
PRR5	0.467099	0.01116
PRR5-ARH(	0.651595	0.03299
PRR9	2.687286	0.000233
PRRC2A	0.288358	0.007066
PRRC2B	0.139918	0.039145
PRRC2C	0.116019	0.030993
PRRG2	-0.32836	0.031701
PRRG4	-0.8589	2.10E-05
PRRT2	-1.27085	1.71E-05
PRRT4	0.484776	0.007415
PRRX2	0.652306	0.026501
PRSS16	-0.47906	7.11E-08
PRSS3	2.28468	1.73E-19
PRSS8	-0.51141	0.000133
PRUNE1	-0.14292	0.021246
PRUNE2	-0.65227	0.004347
PSAP	-0.36773	0.000381
PSAT1	0.318225	0.004047
PSD4	0.191528	0.038635
PSEN1	-0.18936	0.002388
PSENEN	0.140841	0.024241
PSG1	-0.7351	0.004834
PSG2	-1.13703	0.021926
PSG3	-0.88041	0.008556
PSG4	-1.01444	2.34E-10
PSG5	-1.02161	0.00011
PSG7	-1.07692	0.001653
PSG8	-1.42745	0.003994
PSG9	-0.8284	0.000494
PSMA3-AS:	-0.22987	0.018241
PSMA7	0.303189	2.55E-05
PSMB2	0.129879	0.002554
PSMB3	0.214397	7.01E-05
PSMB5	0.221573	4.57E-05
PSMB6	0.235985	8.98E-06
PSMB7	0.181051	0.003429
PSMB8-AS:	-0.35018	0.008578
PSMB9	-0.28501	0.006617
PSMC3	0.156275	0.00766
PSMC3IP	0.225168	0.020248
PSMC4	0.199669	0.000101
PSMC5	0.151952	0.005448
PSMD11	0.118773	0.021539
PSMD13	0.202032	1.13E-05

PSMD2	0.311706	1.28E-06
PSMD3	0.158867	0.043151
PSMD4	0.114063	0.034413
PSMD5-AS:	-0.32714	5.04E-06
PSMD6	0.126	0.012204
PSMD8	0.225323	0.007874
PSME1	-0.21111	0.011842
PSME3	0.185674	0.012187
PSMF1	0.177571	0.028841
PSMG1	0.308541	3.09E-07
PSMG2	0.185837	0.011424
PSMG3	0.260218	0.029215
PSMG3-AS:	-0.44912	0.000918
PSMG4	0.361445	0.008894
PSPH	0.147652	0.00442
PSRC1	0.330098	0.03572
PTBP1	0.380242	0.000825
PTCD3	0.206772	9.41E-05
PTDSS1	0.204795	0.002353
PTGER2	0.319125	0.018046
PTGES2	0.406314	0.006666
PTGES3	0.194062	0.006268
PTGES3P1	-1.47975	0.00703
PTGFRN	-0.16103	0.01304
PTGS2	0.512885	0.000302
PTK2B	0.267648	0.014935
PTN	-0.2271	0.044088
PTOV1-AS1	-0.25421	0.005556
PTOV1-AS2	0.490371	0.028202
PTP4A2	0.089851	0.040534
PTPN1	0.226355	0.000194
PTPN14	-0.14657	0.010992
PTPN2	0.178296	0.019735
PTPN21	-0.31627	7.30E-06
PTPN3	-0.1779	0.000257
PTPN4	-0.17057	0.048782
PTPRC	-1.00829	0.041411
PTPRCAP	-2.95225	0.031072
PTPRE	0.494218	3.95E-16
PTPRH	1.49883	2.07E-09
PTPRM	-0.20629	0.004595
PTPRQ	-0.52019	0.048307
PTPRR	0.303955	0.019802
PTTG1	0.208156	0.001362
PUF60	0.361529	0.002495
PUM3	0.322776	1.92E-05
PURB	-0.22447	0.008043

PUS1	0.386921	0.00167
PUS10	-0.44092	1.19E-05
PUS7	0.197095	0.003686
PUSL1	0.422621	0.04387
PVT1	0.361793	7.08E-07
PWP1	0.183517	0.001101
PWWP2A	-0.16562	0.049533
PXMP4	-0.41143	0.002215
PXN	0.347216	0.000297
PYCR1	0.354387	0.001637
PYGB	0.471767	5.00E-07
PYROXD2	-0.51738	1.11E-06
QDPR	0.157085	0.03562
QPRT	-0.35957	0.032463
QRICH2	-0.47373	3.99E-05
QRSL1	0.190021	0.003751
QSOX2	0.332817	1.12E-08
QTRT1	0.423988	0.001511
QTRT2	0.154058	0.021825
R3HCC1	0.235197	0.035051
R3HDM1	0.192074	0.001
R3HDM4	0.359326	0.007782
RAB11FIP1	-0.2456	0.003667
RAB11FIP1	-0.33213	0.047217
RAB11FIP4	-0.20716	0.04893
RAB17	0.332519	0.022635
RAB23	-0.20001	0.001622
RAB25	-0.47872	0.001242
RAB26	-0.70574	7.54E-05
RAB27A	-0.36119	0.000231
RAB30	-0.5587	3.16E-10
RAB31	0.167011	0.008853
RAB32	-0.48648	6.80E-05
RAB34	0.256561	0.040335
RAB38	0.279184	0.004606
RAB3D	-0.28704	0.006235
RAB3IL1	0.376192	0.01558
RAB3IP	-0.3019	0.0013
RAB40A	-0.48955	0.016251
RAB40B	-0.23151	0.042196
RAB43P1	0.542968	0.012193
RAB4A	-0.32782	1.22E-07
RAB5B	-0.28132	1.46E-05
RAB6B	-0.45505	0.044659
RAB8A	0.144322	0.007965
RABEP1	0.16899	0.00558
RABEPK	0.391512	6.48E-08

RABGAP1L	-0.30563	0.002139
RABL2A	-0.27321	0.022011
RABL3	-0.20928	0.005087
RABL6	0.285053	0.041427
RAC2	0.743591	2.77E-10
RAC3	0.386661	0.023691
RACGAP1	0.251399	0.001412
RACK1	0.369221	2.29E-10
RAD1	0.148999	0.00355
RAD18	0.339587	8.15E-05
RAD21	0.161068	0.032056
RAD21-AS1	-0.62162	0.02308
RAD23A	0.189924	0.018384
RAD51AP1	0.21938	0.023547
RAD51-AS1	-0.34855	0.010055
RAD54B	0.297184	0.012324
RAD54L	0.464503	1.50E-07
RAE1	0.154095	0.004318
RAF1	0.103372	0.021737
RAG1	-0.17563	0.047902
RAI14	-0.18513	0.028459
RALA	0.14037	0.030871
RALB	0.187431	0.014987
RALBP1	0.121122	0.006027
RALGAPA2	-0.32589	0.007146
RALGDS	0.273345	0.020159
RALGPS1	-0.50563	0.000312
RALGPS2	0.207076	0.04525
RALY	0.310085	0.007206
RALYL	-0.91225	0.034528
RAN	0.238294	8.44E-05
RANBP1	0.350817	4.92E-05
RANBP3	0.228662	0.045687
RANGAP1	0.450781	1.50E-07
RANGRF	0.327252	0.004074
RAP1GAP2	0.663113	1.03E-13
RAP1GDS1	0.113473	0.048478
RAP2B	0.170759	0.012989
RAP2C-AS1	-0.62729	0.003073
RAPGEF1	0.189883	0.028322
RAPGEF3	0.352942	0.001311
RARA	0.39362	0.028851
RARB	-1.20915	0.000108
RASA3	0.487928	4.91E-07
RASAL2	0.274707	0.004426
RASAL2-AS	0.268222	0.012862
RASD2	-0.85968	1.64E-06

RASGRF1	0.344987	0.024189
RASIP1	0.509565	0.00132
RASL11B	-0.35817	9.38E-05
RASSF5	0.357828	0.038752
RASSF6	-0.70168	9.76E-06
RASSF8	0.274612	0.004336
RBBP7	0.138319	0.031731
RBBP8	0.129119	0.040487
RBFA	0.30725	3.21E-05
RBFADN	-0.62013	0.000867
RBFOX2	-0.10533	0.0475
RBL1	0.176084	0.021465
RBL2	-0.27214	2.41E-07
RBM10	0.270954	0.024182
RBM15	0.20849	0.001148
RBM17	0.122219	0.003477
RBM19	0.308992	2.61E-10
RBM20	-0.60461	0.026143
RBM22	0.172044	0.001037
RBM26-AS1	-0.33647	0.045975
RBM28	0.211453	0.00078
RBM38	0.339219	0.044108
RBMS3	-0.66308	2.92E-10
RBMX	0.18208	0.005715
RBMX2	0.174408	0.002797
RC3H1	-0.21513	0.018178
RCAN1	-0.43441	0.000166
RCAN2	-1.96017	0.001202
RCAN3	-0.18237	0.006908
RCBTB2	-0.30022	0.013548
RCC1	0.458794	1.21E-18
RCC1L	0.246241	0.001628
RCC2	0.163931	0.001193
RCL1	0.446442	1.34E-12
RCN1	-0.15961	0.007981
RCOR3	-0.22074	3.36E-05
RDH13	-0.29663	0.009455
RDH16	-0.52343	0.009544
RECK	-0.43001	1.84E-06
RECQL4	0.549886	0.003782
REEP1	-1.63392	3.67E-08
REEP4	0.326077	0.031355
RELA	0.215009	0.044583
RELN	2.1094	0.006353
RELT	0.472094	0.001416
RERG	-0.94182	0.021945
RET	-0.64232	5.16E-08

REXO4	0.29355	8.99E-08
RFC2	0.261866	0.0003
RFC3	0.226347	0.005776
RFC4	0.166374	0.017822
RFC5	0.270027	1.34E-06
RFLNB	0.273115	0.021121
RFT1	0.313107	3.22E-05
RFTN1	0.16074	0.008506
RFWD3	0.212573	0.00804
RFX2	0.298264	0.012955
RFX5	-0.17767	0.004877
RFX8	1.169235	3.32E-07
RGAG1	0.671365	0.01359
RGCC	1.053317	1.79E-14
RGL2	0.282011	0.005666
RGL4	-0.41875	0.025046
RGS10	0.398108	0.000258
RGS14	0.453558	0.012868
RGS16	1.017049	0.016626
RGS17	0.495962	0.022442
RGS19	0.371232	0.048188
RGS2	-0.26848	0.001465
RGS20	0.319071	7.76E-05
RHBDL2	-0.36967	4.94E-05
RHCG	0.834428	0.046275
RHNO1	0.234572	0.005041
RHOB	0.294161	0.006808
RHOBTB1	-0.41251	8.18E-05
RHOBTB3	-0.456	0.010514
RHOD	0.452657	0.021137
RHOG	0.305942	0.011894
RHOQ	-0.33612	1.35E-05
RHOQP3	3.089849	0.03903
RHOT2	0.342122	0.040277
RHOU	-0.5701	1.43E-12
RHOXF1-AS	-0.83805	2.24E-05
RIC3	-1.42945	0.004548
RIC8B	-0.2006	0.002136
RIMBP2	-0.48412	0.002361
RIMBP3	-0.8899	0.00276
RIMKLB	-0.22042	0.038476
RIMS2	-0.50372	0.000661
RIMS3	-0.68935	9.82E-05
RIN1	0.540118	0.012289
RIN2	0.118977	0.012217
RINT1	0.199615	0.021177
RIOK1	0.301868	2.32E-06

RIPK1	-0.20686	0.001855
RIPK3	-0.50893	0.000526
RIPPLY2	0.630254	0.007677
RIPPLY3	-0.65806	0.031513
RIT1	-0.28308	0.040244
RMDN2	-0.25654	0.021175
RMND1	0.187086	0.016327
RN7SL1	-0.5018	0.016256
RN7SL2	-0.92114	0.005105
RN7SL274F	1.146932	0.04856
RN7SL376F	-2.03414	0.003966
RN7SL45P	-2.30809	0.017021
RN7SL847F	3.696163	0.006865
RNA5SP15!	2.951635	0.034853
RNASE1	-0.35793	0.02295
RNASE10	-0.43803	0.019159
RNASE4	-0.28823	0.029701
RNASE7	0.407939	4.91E-07
RNASEH2A	0.333625	0.004948
RNASEL	-0.51293	3.54E-06
RNASET2	-0.48244	8.83E-05
RND3	-0.42335	0.022572
RNF103	-0.3145	7.34E-06
RNF103-CH	1.057214	0.015172
RNF11	-0.12901	0.036164
RNF115	-0.12823	0.036762
RNF125	0.375029	3.69E-05
RNF13	-0.2239	0.010131
RNF135	-0.27943	0.001378
RNF138	0.24467	0.003619
RNF14	-0.14613	0.011924
RNF141	-0.25061	0.000162
RNF144A	0.830676	3.37E-22
RNF144A-A	0.662261	0.003234
RNF146	-0.40898	0.000324
RNF150	-1.53185	0.001917
RNF152	-0.61757	4.27E-05
RNF167	0.232255	0.028104
RNF169	0.281473	0.000588
RNF170	-0.34444	9.92E-05
RNF182	1.01017	0.0045
RNF19B	0.313497	0.001171
RNF212B	-0.39546	0.006182
RNF213	-0.36953	0.000736
RNF216	0.151057	0.007461
RNF216P1	0.292097	0.000318
RNF217-AS	-0.59336	2.61E-05

RNF26	0.27935	0.038206
RNF40	0.171382	0.049462
RNPC3	-0.26031	0.004553
RNPS1	0.346908	1.43E-06
RNU7-18P	1.350651	0.045225
RNU7-75P	1.935506	0.015166
RNY3	-3.74388	0.003138
ROBO3	0.659087	0.001054
ROCK1P1	-0.83056	0.023396
ROCK2	-0.24116	0.00397
ROS1	-1.93342	1.41E-07
RP11-1000	-0.51454	0.019559
RP11-1020	-0.5987	0.011924
RP11-102N	-1.27692	0.00058
RP11-1033	1.64252	0.015048
RP11-1094	0.579821	0.003131
RP11-1094	-1.71205	0.031759
RP11-10A1	-0.49493	0.006886
RP11-10C2	-0.36674	0.010295
RP11-1102	0.710848	0.00028
RP11-1145	-0.72914	0.034891
RP11-114G	-1.01428	0.019745
RP11-114H	0.861666	5.38E-14
RP11-114H	1.240475	1.07E-20
RP11-1151	1.203639	0.042346
RP11-1152	-3.14478	0.031384
RP11-115D	-0.61766	0.000662
RP11-1167	-2.06396	0.003809
RP11-117L!	-2.72103	0.036566
RP11-119D	-1.00365	0.00095
RP11-1246	-0.45249	0.002202
RP11-12G1	0.456347	9.52E-05
RP11-131M	-1.31105	0.000829
RP11-132A	0.681022	8.76E-08
RP11-1334	-0.48906	0.010146
RP11-134L	-0.5422	0.042555
RP11-13K1	-1.48103	0.043671
RP11-141M	-1.20906	0.000123
RP11-142A	-1.07018	0.048694
RP11-145A	-1.09371	1.71E-08
RP11-145M	-1.69571	0.024549
RP11-147L	-0.52615	7.87E-09
RP11-147L	-0.42871	0.046432
RP11-152K	-1.74906	0.047421
RP11-156P	0.573125	0.000124
RP11-158I1	-2.07353	0.000232
RP11-159F	-0.75051	0.001946

RP11-159N	-0.8784	0.003106
RP11-15A1	-0.92958	0.010577
RP11-15F1	-0.87926	0.031375
RP11-160N	-1.00525	0.005313
RP11-161M	1.470804	0.001716
RP11-164C	-0.88611	0.019519
RP11-164J1	-0.68084	0.001528
RP11-165F	-2.43003	0.019792
RP11-165J3	-1.12822	0.013751
RP11-169D	2.623955	0.022176
RP11-170M	0.734954	0.043573
RP11-171I2	-1.25387	0.036212
RP11-171I2	-0.88014	0.00288
RP11-173A	1.861279	0.049095
RP11-173P	5.382989	0.014957
RP11-174G	-0.60182	6.46E-07
RP11-174O	-2.31223	0.003204
RP11-175P	-0.91071	0.003062
RP11-181B	-2.26281	0.009762
RP11-181C	3.60299	0.048616
RP11-181K	2.069081	0.021125
RP11-186B	0.395258	0.000315
RP11-187C	-0.72633	0.024995
RP11-18H2	1.034953	0.002138
RP11-196G	0.514908	0.039154
RP11-196G	-0.64081	3.01E-09
RP11-196G	-1.0451	0.00852
RP11-196G	-1.11623	6.98E-05
RP11-197K	-0.3646	0.010604
RP11-1E6.1	2.231883	3.33E-12
RP11-206L	-0.41686	0.00202
RP11-20G1	-2.89815	0.039385
RP11-210K	-2.8872	0.049616
RP11-211G	-1.9406	0.007386
RP11-212P	-0.32692	0.002104
RP11-215P	1.200424	0.045599
RP11-217C	1.258483	0.025152
RP11-218M	-0.43336	0.001546
RP11-219A	-0.77927	0.005412
RP11-219A	1.299475	0.005965
RP11-221N	0.772764	0.000599
RP11-228B	-0.69041	0.008034
RP11-229P	1.394531	0.000257
RP11-22B2	0.601796	1.73E-05
RP11-22C1	-0.72324	0.018069
RP11-22D3	-3.02485	0.026334
RP11-22N1	0.93438	0.045775

RP11-234A	1.013178	0.00529
RP11-23J1ε	-0.96476	0.008243
RP11-244F	-1.69189	0.003907
RP11-244H	-0.60818	0.000629
RP11-244C	-0.23269	0.010712
RP11-248G	5.364594	3.61E-05
RP1-124C6	-1.49275	2.72E-05
RP11-250B	-0.9394	0.018606
RP11-250B	-0.4985	0.032008
RP11-253M	-0.65037	0.002891
RP11-254I2	-2.99164	0.016017
RP11-256I9	0.278202	0.042962
RP11-257K	-3.78068	0.005028
RP11-259N	0.272389	0.028031
RP11-259C	-0.58122	0.006317
RP11-259C	-0.27589	0.014708
RP1-125I3.	1.467841	0.001058
RP11-265B	-3.07096	0.028387
RP11-265N	-5.95795	1.64E-06
RP11-266K	-0.37683	0.024808
RP11-266L!	-3.51601	0.003766
RP11-267M	0.579251	0.004389
RP11-272P	1.50148	0.012181
RP11-274B	-0.43059	0.004099
RP11-274H	-0.74472	0.009381
RP11-277P	-0.64695	2.31E-05
RP11-280G	1.944391	0.035985
RP11-281A	-3.33449	0.037199
RP11-283I3	-0.6351	5.81E-07
RP11-285F	-0.5812	0.04008
RP11-286N	1.119396	0.000634
RP11-286N	-0.27772	0.010122
RP11-288L!	-1.12937	0.034706
RP11-290L	1.53418	0.049252
RP11-295D	0.592079	0.000931
RP11-295K	-0.6214	0.00498
RP11-297P	0.929934	1.46E-21
RP11-298I3	-0.49489	1.43E-05
RP11-299G	-0.45357	0.001011
RP11-29B2	-3.90623	0.002355
RP11-2L8.1	-1.90433	0.021545
RP11-305K	-0.82445	0.032309
RP11-306C	-0.49564	0.001914
RP11-307E	-0.22908	0.046152
RP11-309M	1.853594	0.029735
RP11-30P6	0.754302	0.000449
RP11-314N	-0.7952	0.044755

RP11-319G	-0.54464	0.034944
RP11-321N	-1.45472	0.011282
RP11-323P	-0.66914	0.019675
RP11-324E	-0.86879	0.03902
RP11-324I2	-0.95807	0.03337
RP11-325L	-1.11157	0.044258
RP11-326C	0.76866	0.044417
RP11-326G	-0.59067	0.009134
RP11-326I1	-0.42799	0.026422
RP11-326I1	-1.26759	0.015524
RP11-327F	-0.64705	0.00993
RP11-32B5	-1.20647	0.003725
RP11-32B5	-1.06841	0.033764
RP11-334C	-0.43539	0.025089
RP11-335E	1.29833	0.009977
RP11-337C	-0.72508	0.007664
RP11-337C	-0.33738	0.01561
RP11-337N	-0.81585	0.028273
RP11-338K	0.840671	0.047082
RP11-33B1	-0.22417	0.02806
RP11-33B1	1.221873	0.037712
RP11-342K	3.414943	0.035781
RP11-342M	0.455668	0.02345
RP11-343C	0.367883	7.93E-05
RP11-345J	-2.57377	0.043063
RP11-345J4	0.226692	0.000847
RP11-345P	-1.56345	0.022385
RP11-346D	-0.41968	0.00018
RP11-347C	-2.05105	0.042835
RP11-350N	2.505188	0.043447
RP11-350N	0.933476	0.007609
RP11-350N	0.782311	0.0233
RP11-353K	-1.2704	0.045449
RP11-356J5	-0.42233	2.10E-05
RP11-359E	-0.64926	0.006756
RP11-35J1(	-0.96598	0.042513
RP11-360L	2.499473	1.49E-05
RP11-361L	-0.75014	0.011082
RP11-363E	-0.89532	0.034987
RP11-364P	-1.17856	0.000274
RP11-366L	0.969765	0.012938
RP11-367G	-0.64854	6.83E-05
RP11-368N	-0.68968	0.04876
RP11-373L	-0.58196	1.88E-05
RP11-378A	-0.36742	0.04014
RP11-379B	-0.37421	0.000411
RP11-379F	-1.64805	0.016679

RP11-379H	-0.39763	5.03E-05
RP11-37L2.	-1.7091	0.000419
RP11-382B	-0.32465	6.95E-05
RP11-383J2	-0.89493	0.009021
RP11-384C	3.300657	0.040335
RP11-385D	-1.13345	0.000162
RP11-387H	-3.06435	0.033097
RP11-38L1!	-0.5051	5.33E-06
RP11-391H	-2.09011	0.006402
RP11-391L:	-2.14799	0.029477
RP11-395I1	-0.60852	0.040204
RP11-395N	-0.55578	0.002258
RP11-397A	-0.39498	0.012795
RP11-398C	0.358873	7.56E-05
RP11-3B12	-4.05863	0.000632
RP11-400K	-1.15909	0.003263
RP11-400K	-0.65298	2.18E-14
RP11-403A	0.751488	0.040416
RP11-405M	1.383694	0.040575
RP11-406D	-0.66825	0.045088
RP11-408P	0.380356	0.001027
RP1-140K8	-0.49928	0.000455
RP11-412H	2.005412	0.010794
RP11-413N	-0.57053	0.039061
RP11-414H	-3.02035	0.019066
RP11-415J8	0.316145	0.009668
RP11-416I2	1.115488	2.96E-10
RP11-416N	0.514521	0.016592
RP11-420A	-0.87529	0.007086
RP11-420L!	-0.55366	3.31E-05
RP11-420N	-2.77275	0.040971
RP11-420N	3.222507	0.049755
RP11-421F	-0.36772	0.00185
RP11-424C	1.090978	0.001931
RP11-439E	-0.7126	0.015363
RP11-439E	-1.26865	0.001154
RP11-440D	0.265141	0.036784
RP11-445F	0.848191	1.71E-05
RP11-445H	-2.78178	0.009424
RP11-445N	2.967724	0.02403
RP11-446N	-0.72436	0.018799
RP11-448A	-0.49877	0.004876
RP11-449H	0.389827	0.010975
RP11-44B1	-1.65104	0.041486
RP11-44F1	-0.67464	0.021459
RP11-44F2	-0.53	0.000871
RP11-44N2	-0.428	0.013998

RP11-456N	1.397711	0.026006
RP11-456P	0.676113	0.028269
RP11-457M	-0.80908	0.036765
RP11-45M2	0.373045	0.012982
RP11-461O	0.891544	0.040021
RP11-463O	-7.15363	0.0168
RP11-467J1	-0.70817	0.006721
RP11-468E	-0.27717	0.024431
RP11-46A1	-2.19208	0.025967
RP11-46C2	-0.31827	0.016688
RP11-473M	-0.90718	0.039595
RP11-473O	-2.21467	0.036566
RP11-474L	0.996536	0.009802
RP11-478P	-0.90193	0.048942
RP11-481A	-0.47762	0.047411
RP11-481C	0.453132	0.0278
RP11-481F	-1.77608	1.65E-05
RP11-482H	-1.83989	0.02658
RP11-483F	-0.89492	0.043499
RP11-483I1	-0.67335	0.028779
RP11-486L	-0.61397	0.00256
RP11-486O	-1.00001	0.001754
RP11-488P	1.309366	7.25E-21
RP11-489E	-1.47768	0.028661
RP11-494M	-0.4606	0.004816
RP11-495P	0.686571	0.047324
RP11-496H	-0.35502	0.009145
RP11-498B	1.986259	0.037019
RP11-498E	0.690386	0.01111
RP11-499E	-0.82439	0.00778
RP11-499F	-1.67376	0.023226
RP11-49I11	-1.30867	0.00862
RP11-504P	-0.28159	0.030307
RP11-504P	-0.68514	0.018859
RP11-517C	1.444868	0.030494
RP11-517I3	-1.03443	0.011471
RP11-517I3	-0.97939	2.05E-05
RP1-151F1	-0.37545	0.019429
RP11-520H	-1.53507	0.036087
RP11-521B	0.463805	0.000934
RP11-521B	0.429183	1.75E-06
RP11-521C	0.740768	0.007195
RP11-523L	-1.88664	0.042003
RP11-525A	1.5461	0.010527
RP11-529K	0.689287	0.003993
RP11-530N	-0.53499	0.044922
RP11-532F	-0.46141	0.018554

RP11-538D	-0.48032	0.029953
RP11-539G	-0.72967	0.0387
RP11-53B2	-1.71055	4.72E-05
RP11-53B2	-2.05014	0.003361
RP11-541N	-0.51654	0.008116
RP11-544M	-0.39891	0.026698
RP11-545A	-1.60185	0.018632
RP11-546J1	-0.81893	0.005429
RP11-548K	-3.14728	0.034712
RP11-54A9	0.73975	0.001379
RP11-54H7	-0.134	0.020742
RP11-54O7	-2.91607	0.042937
RP11-553A	-0.38369	0.047554
RP11-553L	-0.50106	0.010267
RP11-557H	-0.814	0.011861
RP11-561O	-1.06179	0.028331
RP11-566K	1.197676	0.025513
RP11-567J2	0.705874	0.038242
RP11-56B1	-0.60224	0.037647
RP11-574H	1.126993	0.010567
RP11-575F	-0.40068	0.037321
RP11-578F	-3.66589	0.020735
RP11-57A1	3.404232	0.030187
RP11-57H1	-0.57764	0.035331
RP11-57H1	-0.45732	5.91E-05
RP11-582J1	0.360619	0.017297
RP11-583F	-2.21245	0.003262
RP11-585P	0.416364	0.000386
RP11-585P	-0.76102	0.038641
RP11-587D	1.147485	0.034401
RP11-587P	3.045422	0.027818
RP11-588K	-1.93528	0.022769
RP11-58O9	0.435099	0.001033
RP11-596C	-0.9689	0.0002
RP11-597D	-0.49282	0.003506
RP11-598P	-7.14465	0.018333
RP1-159A1	-2.03625	0.037788
RP11-59C5	0.429864	0.012009
RP11-59D5	1.798825	2.71E-12
RP11-59H7	-0.61532	0.046943
RP11-59H7	-1.00524	0.047932
RP11-5C23	-0.51614	0.019913
RP11-603J2	-2.43762	0.03821
RP11-609N	0.69415	0.000525
RP11-611L	-2.90165	0.027175
RP11-616M	3.092187	0.010171
RP11-617F	-0.59541	0.000353

RP11-618P	1.496999	0.044814
RP11-61N2	0.535238	0.007997
RP11-624A	-0.21385	0.027377
RP11-624L	-0.22504	0.037692
RP11-625H	-0.52918	0.047753
RP11-629E	-1.13626	0.014528
RP11-631N	-0.46229	0.000787
RP11-638I8	2.826176	0.037057
RP11-63P1	2.00371	0.005619
RP11-641D	0.212048	0.001221
RP11-655M	1.816527	0.018105
RP11-65G9	0.902932	1.35E-05
RP11-661A	0.445366	0.004142
RP11-667M	-3.13165	0.023652
RP11-66B2	0.249032	0.037979
RP11-66B2	1.139788	3.66E-06
RP11-677M	-0.49679	0.018345
RP11-677M	1.411436	0.013319
RP11-680A	0.536791	0.016044
RP11-686D	-0.64184	0.018815
RP11-68I3.	3.118022	0.018627
RP11-696N	-0.50786	0.002954
RP11-69C1	-1.12605	0.005751
RP11-69I8.	0.616727	1.56E-09
RP11-701H	-2.4209	0.039922
RP11-701H	-0.93743	0.030954
RP11-701H	-1.44776	0.012974
RP11-703I1	-1.96465	0.001032
RP11-706P	-1.19595	0.018313
RP11-70D2	-1.28049	0.046635
RP11-70L8.	0.797505	0.002831
RP11-719K	-3.26876	0.046794
RP11-729I1	1.739658	0.037202
RP11-731C	-0.66116	0.005301
RP11-734K	-0.48746	0.000531
RP11-736N	-0.74699	0.024529
RP11-739P	-0.81977	0.008725
RP11-739P	-0.6635	0.016561
RP11-73E1	-0.48301	0.012699
RP11-73M1	-0.44224	0.041274
RP11-745L1	-0.85172	0.018297
RP11-74M1	-0.60829	0.029148
RP11-752G	-3.72459	0.007895
RP11-752L1	-3.13365	0.029329
RP11-753A	2.061293	0.04591
RP11-757F	-0.29269	0.044279
RP11-757G	-0.97352	6.54E-05

RP11-75I2.	-3.08039	0.010662
RP11-761I4	-1.73723	0.005189
RP11-762I7	-1.46161	0.038578
RP11-766F	-0.9437	0.001481
RP11-774O	-1.015	8.99E-07
RP11-775C	-1.41277	0.028908
RP11-777F	-2.08898	0.044382
RP11-781P	-2.91876	0.029059
RP11-783K	0.380816	0.007926
RP11-7K24	-0.45472	0.047571
RP11-800A	-1.04274	0.005307
RP11-806O	-1.6516	0.000364
RP11-80I15	-0.99624	0.032784
RP11-810P	-1.26264	0.043062
RP11-81A2	-0.39676	0.014077
RP11-822E	0.650088	0.000439
RP11-823E	1.443114	2.21E-05
RP11-834C	0.718851	0.004008
RP11-834C	-1.52636	0.026877
RP11-834C	0.40952	0.029201
RP11-83A2	-0.26235	0.02978
RP11-83M1	-0.70094	0.006787
RP11-83M1	-0.74465	0.001306
RP11-844P	2.901615	0.008433
RP11-844P	0.566533	0.007371
RP11-845M	-1.01629	0.00098
RP11-848G	-1.50532	0.003536
RP11-848G	-0.43854	0.026541
RP11-848P	-0.43875	0.003752
RP11-849F	-0.52268	0.023912
RP11-849H	-0.35491	0.021496
RP11-867G	-1.47414	0.006489
RP11-879F	2.609088	0.004674
RP11-884K	-0.5376	0.003713
RP11-885B	-1.93974	0.032738
RP11-88I18	-0.36832	0.046564
RP11-890B	-0.43687	0.00032
RP11-903H	-0.71741	0.003104
RP11-90J19	-0.47767	2.47E-07
RP11-90L1.	-0.47822	2.14E-05
RP11-932O	0.857784	0.025858
RP11-932O	-1.04636	0.016443
RP11-967K	-0.47339	0.000572
RP11-96B2	1.840783	0.005209
RP11-96B5	-2.91182	0.033582
RP11-96D1	-0.55571	0.001378
RP1-197B1	-0.49682	0.011799

RP11-989E	0.470498	0.020712
RP1-198K1	0.761372	0.021962
RP1-199J3.	-0.77532	0.017568
RP1-221C1	-0.66996	2.55E-05
RP1-261G2	0.743634	4.79E-05
RP1-27O5.3	0.56706	0.006008
RP13-104F	-0.6049	0.005247
RP13-204A	-1.33099	0.000279
RP13-210D	-1.80056	0.006313
RP13-467H	-1.40857	0.03174
RP1-34B20	-1.00114	0.004055
RP1-34M23	2.528473	0.001429
RP13-766D	-1.15304	0.005707
RP13-942N	0.74596	0.000974
RP1-80N2.3	-0.69298	0.000421
RP1-80N2.4	-0.70461	0.016961
RP1-95L4.4	-0.50702	0.013453
RP1L1	0.440816	0.012162
RP3-331H2	0.477526	8.68E-05
RP3-333H2	2.985463	0.022407
RP3-368A4	-0.51125	0.019406
RP3-395M2	0.936135	0.033792
RP3-395M2	-0.69948	0.01636
RP3-400B1	-0.95502	0.01302
RP3-426I6.	3.666794	0.006098
RP3-428L1	-0.33572	0.018746
RP3-429G5	-1.78572	0.013366
RP3-439F8	-0.55133	0.000707
RP3-465N2	3.502516	0.006975
RP3-467D1	0.286723	0.00435
RP3-473L9.	-2.3544	0.02895
RP3-508I15	1.676066	0.041437
RP4-530I15	0.851305	0.000988
RP4-536B2	-0.84096	0.018594
RP4-545C2	-0.69174	0.00128
RP4-555D2	0.28028	0.019424
RP4-568C1	-0.30674	0.000244
RP4-584D1	-3.29829	0.033973
RP4-614C1	0.237675	0.013141
RP4-635E1	-0.72005	5.32E-05
RP4-663N1	2.527137	0.042538
RP4-665N4	-2.98064	0.022672
RP4-668J24	0.736418	0.018072
RP4-673M1	-1.1638	0.00439
RP4-714D9	-0.27549	0.021109
RP4-758J18	-0.22927	0.034493
RP4-781K5	0.288953	0.012084

RP4-798P1	-0.45992	0.000181
RP4-800G7	-0.43188	0.043025
RP5-1028K	0.71487	8.43E-06
RP5-1037N	1.956362	0.020174
RP5-1039K	-0.40745	0.001257
RP5-1039K	-1.80007	0.002225
RP5-1039K	-0.30215	0.002222
RP5-1042K	0.839454	0.008704
RP5-1061H	-0.57379	0.031433
RP5-1063M	0.482648	0.013635
RP5-1065J2	-0.39765	0.034707
RP5-1074L1	-0.41162	0.000885
RP5-1086K	-0.5677	0.008436
RP5-1148A	-0.45103	0.031703
RP5-821D1	-0.32571	0.032528
RP5-827C2	-0.8455	0.02933
RP5-855D2	-0.67065	0.040445
RP5-855D2	-1.13006	0.024676
RP5-857K2	-0.63988	0.035497
RP5-864K1	0.684461	0.044373
RP5-874C2	-1.42922	0.041069
RP5-875H1	0.57384	0.002009
RP5-882C2	-0.59514	0.010386
RP5-965G2	0.551075	0.000107
RP5-965G2	0.511108	0.044544
RP5-967N2	0.739891	0.010339
RP6-114E2	-0.5665	0.049945
RP6-218J18	-3.29249	0.043903
RP9P	0.221441	0.012826
RPA1	0.148548	0.006832
RPA2	0.1325	0.019863
RPA3	0.174197	0.010233
RPAP1	0.204413	0.012616
RPAP2	-0.16811	0.0309
RPAP3	0.163481	0.048062
RPE65	0.45309	0.000221
RPEP6	-1.03595	0.001212
RPF1	0.177666	0.011981
RPF2	0.319143	0.000111
RPGR	0.186266	0.020385
RPIA	0.314367	3.27E-06
RPL10	0.179508	0.02134
RPL10A	0.252566	4.47E-06
RPL10P13	1.574233	6.49E-07
RPL11	0.221437	7.05E-08
RPL12	0.295855	3.82E-08
RPL13A	0.307605	0.000316

RPL13AP5	0.70628	0.02296
RPL13AP7	0.680226	0.040632
RPL13P12	1.564355	0.007874
RPL14	0.2612	3.87E-06
RPL15	0.158794	0.000196
RPL17	0.215601	0.000439
RPL18	0.369851	6.23E-06
RPL18A	0.327987	0.007941
RPL19	0.224535	0.012433
RPL21P16	0.41407	0.013218
RPL22L1	0.346164	0.000559
RPL23	0.182037	0.007052
RPL23A	0.355616	3.22E-05
RPL24	0.193401	0.00767
RPL26	0.198848	0.013274
RPL27	0.19293	0.000369
RPL27A	0.290279	0.000581
RPL28	0.235358	0.007541
RPL29	0.281066	1.97E-05
RPL3	0.29533	5.63E-11
RPL30	0.119699	0.034427
RPL31	0.170825	0.005601
RPL32	0.27019	4.37E-09
RPL34	0.226854	9.32E-05
RPL35	0.337322	1.48E-05
RPL35A	0.238647	0.000226
RPL36	0.404819	4.98E-05
RPL36A	0.19333	0.009208
RPL37	0.153922	0.013575
RPL37A	0.149354	0.034291
RPL38	0.221829	6.23E-05
RPL39L	0.226164	0.032935
RPL4	0.279991	1.41E-12
RPL41	0.220481	9.68E-06
RPL5	0.226832	0.000881
RPL6	0.249678	9.72E-09
RPL7	0.179846	0.021087
RPL7A	0.300175	1.18E-07
RPL7L1	0.174262	0.002595
RPL8	0.43216	0.001267
RPL9P7	2.442514	0.012456
RPLP0	0.262324	3.41E-06
RPLP0P2	0.522853	0.005638
RPLP0P6	0.96891	0.0043
RPLP2	0.419256	0.001682
RPN1	0.10153	0.042841
RPP21	0.327638	0.005883

RPP25L	0.234718	0.005309
RPP30	0.234655	0.00792
RPP40	0.324181	6.16E-05
RPRM	-1.90103	0.03211
RPS10	0.281617	2.91E-07
RPS11	0.155722	0.017982
RPS12	0.255461	1.68E-07
RPS13	0.258293	4.68E-07
RPS14	0.332518	4.33E-06
RPS15	0.36845	0.031579
RPS15A	0.173938	0.001881
RPS16	0.346631	1.69E-05
RPS17	0.221809	0.000177
RPS18	0.233188	2.96E-05
RPS19	0.276778	0.000437
RPS19BP1	0.320932	0.023939
RPS2	0.410287	0.029252
RPS20	0.176218	0.000333
RPS21	0.281565	4.12E-05
RPS24	0.219734	0.000257
RPS27A	0.151344	0.030974
RPS28	0.305804	0.00305
RPS28P7	0.29446	0.00924
RPS2P46	0.493431	0.000875
RPS2P5	0.242214	0.021279
RPS3	0.268313	0.000149
RPS3A	0.164241	0.041227
RPS4X	0.23733	9.87E-07
RPS4XP16	-0.66419	0.026273
RPS5	0.351743	8.04E-05
RPS6	0.221883	0.001241
RPS6KA1	0.272979	0.002456
RPS6KA2	0.277293	0.010465
RPS6KA2-IT	0.870288	0.008388
RPS6KA4	0.345749	0.02583
RPS7	0.17614	0.002985
RPS7P11	2.139618	0.03196
RPS8	0.298625	1.86E-09
RPS9	0.301849	6.88E-07
RPSA	0.251943	8.86E-05
RPSAP52	1.367317	2.65E-32
RPUSD3	0.311559	0.021036
RPUSD4	0.177826	0.014023
RRAGB	-0.20562	0.004543
RRAGC	-0.167	0.048862
RRM1	0.127283	0.043969
RRM2	0.351478	0.001015

RRP1	0.555862	1.10E-07
RRP12	0.368205	1.20E-06
RRP15	0.178405	0.035214
RRP1B	0.327211	0.0005
RRP7A	0.32801	0.001484
RRP7BP	0.234652	0.035451
RRP8	0.200335	0.000564
RRP9	0.470428	8.58E-05
RRS1	0.306409	0.006078
RSAD1	0.373152	0.000206
RSC1A1	0.286209	0.006627
RSL1D1	0.384557	3.12E-08
RSL24D1	0.204005	0.018707
RSU1	-0.13943	0.010507
RTCB	0.120924	0.04181
RTEL1	0.436976	0.000133
RTKN	0.303573	0.028272
RTKN2	-0.44439	0.000403
RTN1	-0.96065	0.048589
RTP3	0.223916	0.042295
RTP4	-0.82629	0.017469
RTTN	0.330677	0.000202
RUNX3	0.380775	0.008316
RUSC2	0.566413	5.58E-07
RUVBL1	0.39521	1.49E-14
RUVBL2	0.451618	0.000418
RWDD2A	-0.46791	3.67E-05
RWDD3	-0.17894	0.038855
RXRG	-0.87867	0.035797
S100A10	0.177439	0.005712
S100A11	0.225016	0.008356
S100A13	0.141882	0.009446
S100A16	0.310859	0.000262
S100A2	0.284852	5.13E-05
S100A3	0.297929	0.038849
S100A5	1.490497	0.017127
S1PR1	0.304375	0.008789
S1PR2	-0.34206	0.001538
S1PR3	0.25762	0.000724
S1PR5	-0.56835	0.000711
SAA1	-0.75631	1.36E-11
SAA2	-1.00233	1.71E-10
SAA4	-1.69352	0.004001
SAAL1	0.317251	6.79E-06
SACS	0.299329	0.004077
SAE1	0.229083	3.25E-05
SAFB	0.266616	0.000738

SAFB2	0.238869	0.005478
SALL1	0.419734	0.015077
SAMD12	-1.11469	3.81E-07
SAMD4A	-0.53262	0.000138
SAMD4B	0.306952	0.002976
SAMD9L	-0.80073	0.014194
SAMHD1	-0.48757	0.020819
SAP130	0.182115	0.017902
SAP18	0.113352	0.02542
SAP30	0.424496	0.001983
SAP30BP	0.153861	0.001071
SAP30L	-0.20036	0.016926
SAP30L-AS:	-0.38781	0.039611
SAPCD1-AS	-2.07348	0.03517
SAR1B	-0.13766	0.025862
SARDH	0.439238	0.026553
SARM1	-0.50534	0.005928
SARS	0.172859	0.012311
SARS2	0.344423	0.011062
SART1	0.278411	0.006642
SART3	0.223785	3.52E-05
SASH1	-0.336	0.001267
SATB1	-0.61961	0.005096
SATB2	-0.24075	0.015024
SAV1	0.158239	0.021093
SBF1	0.316477	0.027178
SBF2-AS1	-0.29003	0.001869
SCAF4	0.217222	0.000925
SCAF8	0.119069	0.020177
SCAP	0.238396	0.032716
SCARB1	0.344993	0.000659
SCARB2	-0.14594	0.021126
SCARF1	-0.86224	0.00567
SCARNA13	0.509846	0.040068
SCART1	-0.4427	0.035297
SCCPDH	-0.20017	0.000434
SCD	0.216225	0.029589
SCD5	-0.57493	1.51E-06
SCFD2	0.137987	0.029943
SCGB3A2	0.614119	1.53E-12
SCLT1	0.203827	0.005851
SCLY	0.337671	0.001276
SCN1B	-0.51096	0.006396
SCN2A	-0.65613	1.04E-06
SCN3B	-0.50845	0.0142
SCN4B	-0.43065	9.06E-05
SCN8A	-0.3859	0.010004

SCN9A	0.587866	0.000904
SCO1	0.156071	0.005523
SCP2	-0.24699	0.002587
SCPEP1	-0.65671	5.55E-08
SCRN3	-0.39932	0.000131
SCUBE3	-0.42856	0.006317
SCYL1	0.221397	0.041491
SCYL3	-0.22028	0.03878
SDAD1	0.12113	0.017334
SDCCAG3	0.420361	8.37E-09
SDCCAG8	0.149142	0.012865
SDHA	0.188545	0.001853
SDHAF3	0.241558	0.005096
SDHB	0.288209	0.000292
SDHC	-0.11082	0.041575
SDK1	-0.58521	2.06E-05
SDK2	-0.40723	0.001588
SDPR	-0.54278	0.003391
SDR39U1	0.325277	0.04287
SDR42E1	-0.28228	0.009288
SDSL	-0.35033	0.007315
SEC13	0.147658	0.048108
SEC14L1	0.114541	0.021925
SEC14L2	0.387018	2.74E-07
SEC16B	-0.46106	0.00014
SEC1P	-0.85974	0.015866
SEC22A	-0.19763	0.033932
SEC22B	-0.14216	0.044003
SEC23A	-0.21156	0.011282
SECISBP2L	-0.21663	0.030149
SEH1L	0.34521	1.75E-09
SEL1L3	-0.25607	0.021043
SELENOH	0.267085	0.021559
SELENOP	-1.5098	0.000241
SELL	-1.21169	0.000105
SEMA3C	-0.19963	0.026552
SEMA3F	-0.30184	0.020532
SEMA4B	0.311099	0.013092
SEMA4D	0.234175	0.010534
SEMA4G	-0.28717	0.043266
SEMA6D	-0.55692	0.007319
SEMA7A	0.710453	2.14E-09
SENCR	0.660936	0.014682
SENP1	0.211536	0.000381
SENP3	0.341777	0.002639
SENP7	-0.30618	0.011064
SENP8	-0.27244	0.018105

SEPHS1	0.141315	0.035582
SEPSECS-AS	-0.21485	0.048716
11-Sep	0.199956	0.002489
SEPT14P19	-1.47555	0.000878
4-Sep	-0.48596	0.030941
6-Sep	-0.18499	0.005095
8-Sep	-0.16683	0.001972
9-Sep	0.324057	0.009048
SERAC1	-0.24239	0.032159
SERBP1	0.300248	1.56E-13
SERINC3	-0.14975	0.031648
SERPINB13	-0.83196	0.033
SERPINB2	1.804278	8.00E-44
SERPINB5	0.333285	5.80E-05
SERPINB8	0.269631	0.005625
SERPINB9P	-0.70507	3.10E-06
SERPIND1	1.154538	0.028912
SERPINE1	0.314605	8.57E-05
SERPINE2	0.905308	5.06E-06
SERPINF1	-0.20512	0.034484
SERPINF2	-0.49864	0.004829
SERTAD1	0.304939	0.025647
SERTAD2	0.182452	0.004419
SERTAD3	-0.29193	0.000928
SERTAD4	-0.35526	4.82E-05
SESN1	-0.17878	0.0439
SESN2	0.346616	0.012273
SESN3	-0.51354	0.001049
SET	0.240644	0.000455
SETBP1	0.395332	1.96E-06
SETD1A	0.364311	0.009677
SETD3	0.117935	0.019068
SF1	0.246684	0.00952
SF3A1	0.282561	5.07E-07
SF3A2	0.41266	0.041281
SF3A3	0.23024	1.19E-05
SF3B2	0.230871	1.09E-07
SF3B3	0.295716	3.86E-07
SF3B5	0.298314	0.008286
SFMBT2	0.264384	0.006271
SFN	0.416662	0.001438
SFR1	0.182424	0.034047
SFRP1	-0.23253	5.17E-05
SFSWAP	0.263708	0.000251
SFT2D2	-0.2518	0.003984
SFTA1P	1.067599	2.22E-15
SFXN1	0.175209	0.006294

SFXN4	0.365591	7.12E-07
SGCB	-0.2554	7.56E-05
SGF29	0.249151	0.011526
SGK1	-0.16952	0.025159
SGMS1-AS1	-0.81448	3.49E-05
SGMS2	0.190603	0.014985
SGPP1	-0.1904	0.029804
SGTA	0.462872	0.001479
SH2B2	0.717961	0.017055
SH2B3	0.444556	1.68E-12
SH2D2A	1.136017	5.92E-11
SH2D3A	0.516085	0.000229
SH2D3C	-0.64873	0.041908
SH2D5	1.116941	8.64E-16
SH3BGRL	-0.54396	7.61E-11
SH3BGRL3	0.483016	0.00031
SH3BP5-AS	-0.49695	0.001923
SH3D19	-0.21814	0.000248
SH3GL1	0.419286	0.003162
SH3GLB1	-0.1535	0.03421
SH3KBP1	0.35927	1.28E-06
SH3RF1	0.213436	0.003683
SH3RF2	-0.29697	0.000513
SH3RF3	0.440458	4.84E-05
SH3TC1	0.592784	0.000226
SH3TC2	0.37302	8.53E-06
SH3YL1	-0.36607	0.004665
SHANK2	-0.30087	0.002972
SHC1	0.2745	0.000459
SHC2	-0.63456	0.00946
SHC3	-0.30083	0.002692
SHCBP1	0.24518	0.001836
SHISA4	-0.73444	5.59E-06
SHISA9	-0.36255	0.004155
SHMT2	0.404132	8.95E-12
SHQ1P1	-1.70253	0.001799
SHROOM3	0.394473	1.24E-12
SHROOM4	-0.40201	2.34E-07
SHTN1	-0.34837	0.000283
SIAE	-0.61316	5.94E-10
SIDT2	-0.55822	3.60E-07
SIGMAR1	0.457507	0.000898
SIKE1	-0.20531	0.008253
SIMC1	0.409328	0.000606
SIN3A	0.127214	0.016598
SIPA1L3	0.343493	0.014006
SIRPA	-0.2812	0.002155

SIRT5	-0.13112	0.043401
SIX1	0.148802	0.032794
SIX2	0.383798	0.031294
SKA1	0.256472	0.003575
SKA3	0.456931	1.22E-05
SKI	0.210366	0.037742
SKIV2L	0.275795	0.000612
SLAIN2	-0.18997	0.041137
SLBP	0.165142	0.012704
SLC12A7	0.300359	0.012476
SLC12A8	-0.4352	0.000105
SLC13A5	0.571858	0.000253
SLC15A2	-0.30069	0.000131
SLC16A1	0.135202	0.046862
SLC16A13	-0.27769	0.032895
SLC16A14	-0.46203	0.000632
SLC16A4	-0.46287	0.000881
SLC17A5	-0.42003	1.98E-09
SLC19A1	0.494464	0.000703
SLC1A3	-0.18402	0.005432
SLC1A5	0.252055	0.01194
SLC20A1	0.804186	3.07E-28
SLC22A2	0.438385	0.048337
SLC22A23	-0.41023	0.000307
SLC22A5	-0.17362	0.017451
SLC24A3	-0.61233	6.00E-05
SLC25A15	0.293579	9.60E-05
SLC25A19	0.473375	1.23E-09
SLC25A1P5	1.706846	0.017599
SLC25A22	0.534071	0.002487
SLC25A23	-0.37183	0.000837
SLC25A25	0.437608	6.06E-10
SLC25A27	-0.39905	0.001352
SLC25A3	0.112909	0.041129
SLC25A32	0.243046	0.001103
SLC25A33	0.144271	0.035206
SLC25A37	0.334657	2.01E-06
SLC25A39	0.309586	0.007975
SLC25A4	-0.29216	0.001603
SLC25A42	-0.28814	0.010774
SLC25A46	-0.14941	0.024665
SLC25A5	0.276842	1.97E-08
SLC25A52	1.376293	0.017357
SLC25A5-A	0.453586	0.04363
SLC25A6	0.350282	0.005957
SLC26A2	-0.29587	0.010115
SLC26A6	0.194122	0.043917

SLC27A3	-0.38762	0.005377
SLC27A4	0.347342	0.002945
SLC27A6	-2.90641	0.036244
SLC28A3	-0.56094	0.003884
SLC29A1	0.715274	8.34E-11
SLC2A10	-0.72496	0.001767
SLC2A11	-0.4683	0.000106
SLC2A12	-1.74565	6.13E-16
SLC2A1-AS1	-0.67682	0.029127
SLC2A3	1.469032	6.77E-25
SLC2A5	-0.25486	0.020915
SLC2A9	-0.28456	0.000327
SLC30A10	-0.85746	0.005968
SLC30A4	-0.68462	1.62E-09
SLC30A6	-0.19674	0.024099
SLC33A1	-0.24202	0.002467
SLC34A1	1.685756	0.033476
SLC35A5	-0.17413	0.001418
SLC35B3	-0.21907	0.0185
SLC35B4	-0.19618	0.039856
SLC35D1	-0.30059	0.000205
SLC35E2B	-0.17496	0.004587
SLC35E3	-0.13525	0.047544
SLC35F2	0.236026	4.39E-05
SLC35F5	-0.16077	0.040416
SLC37A2	0.349751	0.000846
SLC37A4	0.200209	0.010216
SLC38A4	-0.47481	1.42E-10
SLC38A5	0.329048	0.002685
SLC38A6	-0.21637	0.002193
SLC38A7	-0.17264	0.014019
SLC38A9	-0.15668	0.006981
SLC39A10	0.387483	0.000385
SLC39A11	-0.361	9.04E-07
SLC39A14	0.132138	0.002408
SLC39A4	0.610059	0.003595
SLC40A1	-1.58615	7.57E-05
SLC41A1	0.166162	0.009624
SLC41A2	-0.31904	0.032288
SLC43A1	0.447449	0.020696
SLC43A3	0.468655	6.34E-10
SLC44A2	-0.38371	0.000315
SLC44A3	-0.60441	6.83E-09
SLC44A3-A	-0.44119	9.64E-05
SLC44A5	-0.36115	0.011067
SLC45A1	1.86628	0.034479
SLC45A3	0.382039	0.007899

SLC46A1	-0.53558	8.19E-09
SLC47A2	-0.63621	0.006988
SLC4A11	0.32898	0.038927
SLC4A7	0.333786	0.007877
SLC4A8	-0.36895	0.003622
SLC51A	-0.48981	0.000156
SLC52A1	-0.42831	1.64E-05
SLC5A12	-0.70276	4.97E-09
SLC5A3	0.287289	0.008447
SLC5A5	-1.37252	0.008946
SLC5A6	0.406961	0.000352
SLC6A15	0.388066	3.25E-05
SLC6A2	-1.18395	5.62E-07
SLC6A20	0.436565	0.02894
SLC6A8	0.275513	0.004453
SLC7A2	0.808545	4.21E-16
SLC7A4	-0.98306	0.021735
SLC7A5P1	0.964914	0.041258
SLC7A7	-1.06744	2.47E-14
SLC8A1	-0.25406	0.033961
SLC9A1	0.323475	0.003236
SLC9A3R2	0.638332	0.001647
SLC9A7P1	-0.90747	0.011863
SLC9A8	-0.14967	0.040622
SLC9A9	-0.56575	6.25E-06
SLC9B2	0.354907	0.000527
SLC9C1	-0.92129	0.011203
SLCO1A2	-0.86955	0.000173
SLCO2A1	0.293032	0.028095
SLCO4A1	1.374674	6.21E-36
SLCO4C1	-0.92859	0.034482
SLCO6A1	-1.55932	0.004521
SLFN11	0.439777	1.87E-11
SLFN13	-0.29481	0.015704
SLIRP	0.198797	0.004805
SLIT2	0.652986	0.000468
SLPI	-0.57949	0.021429
SMAD3	0.190624	0.018211
SMAD6	-0.53522	0.021602
SMAGP	0.50144	1.86E-06
SMARCA1	-0.29673	0.000413
SMARCA2	-0.15264	0.008103
SMARCA4	0.377114	0.000454
SMARCB1	0.226602	0.013066
SMARCC1	0.184204	0.0006
SMARCC2	0.159554	0.001909
SMARCD1	0.322468	1.50E-07

SMARCD3	-0.26011	0.039182
SMC1A	0.147306	0.001585
SMC2	0.279395	0.002036
SMC2-AS1	-0.58708	0.001526
SMC4	0.195387	0.033864
SMG1P2	0.376979	0.004471
SMG1P4	0.421903	0.028716
SMG1P7	-0.32563	0.005156
SMG5	0.211263	0.00119
SMG9	0.232	0.0013
SMIM14	-0.54173	0.001637
SMIM20	0.19083	0.02642
SMIM3	0.700766	1.42E-18
SMIM7	-0.12244	0.04947
SMN2	0.214956	0.005243
SMNDC1	0.162759	0.038086
SMO	-0.37211	0.01143
SMOX	0.494937	2.51E-08
SMPD2	0.340189	0.001424
SMPD3	0.553529	0.01528
SMPD4	0.268654	0.005297
SMPDL3A	-0.18606	0.037505
SMPDL3B	-0.65883	0.000256
SMS	0.26297	1.52E-05
SMTN	0.512464	0.000969
SMURF1	0.185637	0.009522
SMURF2	0.170279	0.040629
SMYD5	0.404259	1.79E-08
SNAI1	0.836286	0.000132
SNAI2	0.42696	2.27E-09
SNAPC2	0.368515	0.035531
SNAPC3	-0.17097	0.048308
SNAPC4	0.434259	0.005959
SNCAIP	-0.32576	0.020136
SNCB	1.139291	0.000457
SNCG	-1.18193	7.49E-11
SNHG1	0.366108	1.95E-05
SNHG12	0.22958	0.013891
SNHG14	-0.36625	2.07E-05
SNHG15	0.470374	6.55E-08
SNHG16	0.209468	0.007788
SNHG17	0.37196	0.000118
SNHG23	0.701304	0.00187
SNHG4	0.203943	0.029679
SNHG5	0.256891	0.003152
SNHG6	0.128355	0.031259
SNHG9	0.366463	0.033797

SNN	-0.25966	0.000449
SNORA20	-1.83584	0.028213
SNORA70	0.873252	0.002309
SNORA71C	1.215881	7.51E-05
SNORA73B	0.651287	0.041907
SNORD17	0.634473	0.000214
SNORD46	1.064963	0.043813
SNPH	-0.34431	0.031111
SNRK	0.20348	0.001541
SNRNP200	0.169968	5.64E-05
SNRNP25	0.278882	0.001757
SNRNP40	0.306012	5.22E-08
SNRPA	0.388156	0.004496
SNRPA1	0.248473	2.55E-05
SNRPB	0.439655	2.25E-07
SNRPB2	0.153515	0.035022
SNRPC	0.214285	0.005963
SNRPD1	0.313287	6.07E-05
SNRPD2	0.20045	0.001976
SNRPE	0.250308	0.002264
SNRPF	0.308855	8.06E-06
SNRPG	0.229115	0.001368
SNRPN	-0.18819	0.024339
SNTB2	-0.43214	5.98E-05
SNU13	0.328205	2.49E-06
SNURF	-1.28435	0.000386
SNX12	0.130249	0.008814
SNX13	-0.16275	0.029042
SNX14	-0.19443	0.010278
SNX19	-0.23165	1.58E-05
SNX24	-0.31043	0.001308
SNX29	0.204256	0.017796
SNX33	-0.31453	0.000107
SNX4	-0.25976	2.50E-05
SNX5	0.299883	9.23E-10
SNX7	-0.22412	0.005326
SNX9	0.316896	1.43E-08
SOCS2	0.400662	0.000688
SOCS3	0.601633	0.012175
SOD2	-0.59032	4.66E-05
SOD3	-1.47049	4.20E-05
SOGA1	-0.163	0.049212
SOGA3	-0.42774	0.037186
SORCS3	2.649865	1.29E-05
SORD	0.387735	3.71E-18
SORD2P	0.443839	3.95E-05
SORL1	0.240312	2.81E-05

SORT1	-0.69412	4.54E-11
SOS1	-0.33177	3.61E-05
SOS2	-0.42114	7.93E-07
SOWAHB	-1.29309	0.000321
SOX13	0.567531	2.28E-07
SOX2	-0.39142	0.007156
SOX4	-0.24415	0.001387
SOX6	-0.49617	1.71E-05
SOX7	0.265472	0.011742
SOX9	0.90106	4.92E-21
SP140L	-0.20427	0.001956
SP2	0.231748	0.021944
SPAG16	-0.24787	0.006526
SPAG5	0.249932	0.000543
SPAG7	0.186394	0.010782
SPANXA1	0.984758	0.027389
SPANXA2	0.984758	0.027389
SPANXB1	0.815957	0.027168
SPARC	-0.54203	0.007911
SPATA13	0.408066	6.62E-08
SPATS2L	0.127735	0.007182
SPDL1	0.199978	0.044247
SPECC1	-0.16575	0.000168
SPEF2	-0.5516	0.014871
SPEG	-0.3669	0.014627
SPG11	-0.22658	0.008344
SPG21	0.170922	0.003781
SPIDR	-0.19184	0.008093
SPIN4	0.276197	0.000795
SPINK5	-0.43132	0.000537
SPINT1	-0.19026	0.049272
SPOCK1	0.593239	6.09E-07
SPOCK2	-0.64119	0.015381
SPOUT1	0.334822	0.000246
SPPL2A	-0.32433	1.83E-06
SPPL3	-0.14314	0.016598
SPRR1B	1.255878	1.36E-05
SPRR2A	1.268681	0.00625
SPRR2C	2.897736	0.021295
SPRR2F	3.688058	0.005052
SPRY2	1.049006	3.53E-35
SPRY4	0.681325	4.31E-23
SPRYD3	0.233622	0.000489
SPTB	0.482973	0.016436
SPTBN5	0.768934	0.018155
SPTSSA	-0.32066	0.002765
SQLE	0.307575	6.51E-05

SRA1	0.186262	0.041563
SRBD1	0.139281	0.016838
SRCAP	0.176013	0.010392
SRFBP1	0.209042	0.020617
SRGAP1	0.229784	0.037875
SRGAP2	0.180114	0.001929
SRGAP2D	0.408101	7.64E-05
SRGAP3	-0.38105	0.00013
SRL	-1.87085	0.012785
SRM	0.551433	0.000264
SRP14-AS1	-0.41046	8.08E-05
SRP68	0.173534	0.000678
SRP72	0.213032	0.001454
SRPK1	0.214756	0.007798
SRPRB	0.154845	0.00417
SRPX	0.244827	0.009562
SRRM1	0.140265	0.010103
SRRT	0.307206	0.001002
SRSF7	0.170955	0.032021
SRSF8	-0.16304	0.027762
SRSF9	0.268493	0.000119
SSB	0.198938	0.045289
SSBP1	0.160409	0.041755
SSC5D	-1.11087	0.00555
SSFA2	0.345484	5.94E-07
SSNA1	0.299827	0.015306
SSPN	-0.8891	3.30E-26
SSR2	0.14749	0.011265
SSRP1	0.375719	8.92E-11
SSSCA1	0.370691	0.018155
ST3GAL5	0.365757	0.025112
ST3GAL6	0.3113	0.002085
ST3GAL6-A	0.870343	0.000534
ST6GAL1	-0.25415	0.000286
ST6GALNA(	0.370627	0.027509
ST7	0.142711	0.034487
ST7-OT4	-1.27632	0.027038
ST8SIA4	0.700303	0.005569
ST8SIA6	-1.43538	0.000487
STAB1	2.628852	0.044174
STAC	0.470748	6.52E-06
STAG1	0.193522	0.009679
STAG3L1	-0.32159	0.005938
STAM	0.167111	0.011299
STAMBPL1	0.490441	8.02E-09
STARD10	-0.29103	0.025098
STARD4-AS	-0.29795	0.049276

STARD5	-0.54051	0.013657
STARD7	-0.13903	0.001316
STAT2	-0.45589	0.016218
STAT3	-0.24258	0.047218
STAT5A	-0.32556	0.017714
STAU1	0.145663	0.00017
STC2	0.672554	2.88E-14
STEAP1	0.496115	6.21E-07
STEAP1B	2.279118	0.023819
STIL	0.249747	0.002159
STIP1	0.324485	8.34E-08
STK10	0.359958	6.47E-07
STK11IP	0.243428	0.005623
STK17B	0.479156	4.35E-07
STK24	0.236185	4.60E-07
STK31	-0.77436	8.75E-08
STK32A	-1.23919	0.002954
STK32C	0.317504	0.012221
STK35	0.166707	0.023406
STK38	-0.32649	1.53E-15
STK38L	-0.42582	8.91E-05
STK4	0.254221	0.000579
STMN3	1.169688	1.24E-08
STN1	0.148775	0.006359
STOM	0.15104	0.017807
STOML2	0.388059	5.02E-05
STON1	-0.7529	6.18E-07
STON2	-0.28283	0.002146
STOX2	-0.83102	2.50E-11
STRAP	0.181754	0.000383
STRIP1	0.113135	0.035961
STRIP2	0.194011	0.014322
STS	-0.34925	0.0013
STX10	0.207003	0.048137
STX12	-0.35516	0.000309
STX19	-1.13025	0.004324
STX1A	0.619781	6.52E-06
STX7	-0.23047	0.005219
STXBP5	-0.25255	0.008802
SUCLG1	0.108868	0.023986
SUCLG2-AS	-0.80508	0.047682
SUDS3	-0.14143	0.020543
SUGP2	0.106235	0.03937
SULF1	-0.59798	0.035281
SULT1E1	-0.84594	0.000161
SUMF1	-0.35869	0.000101
SUMO3	0.144596	0.039506

SUN3	0.402299	0.011627
SUOX	-0.2378	0.0243
SUPT16H	0.185301	0.002333
SUPT5H	0.176702	0.005979
SUPT7L	-0.11203	0.02412
SUPV3L1	0.149292	0.02033
SURF2	0.504959	0.000877
SURF6	0.333158	0.020864
SUSD2	-0.69306	0.009696
SUSD4	0.705551	0.000186
SUV39H1	0.350937	0.008995
SVEP1	-0.20572	0.038427
SVIL	-0.18699	0.005189
SWI5	0.211595	0.017707
SYBU	-0.34224	0.0143
SYCP2L	-0.5192	0.041231
SYDE2	-0.34112	0.019621
SYMPK	0.284898	0.000333
SYNC	-0.72826	1.60E-15
SYNCRIP	0.166392	0.0269
SYNE1	-0.28556	6.69E-05
SYNE2	-0.37407	1.38E-05
SYNE3	0.966121	1.80E-35
SYNJ2	0.420771	1.09E-16
SYNJ2BP	-0.28252	4.16E-05
SYNPO	-0.67417	1.12E-06
SYNPO2	-1.56438	2.84E-16
SYS1	0.176785	0.004987
SYT15	-0.67549	1.32E-09
SYT17	-0.47229	0.005134
SYT7	0.382385	0.00047
SYTL2	-0.81822	0.001342
SYTL3	0.30368	1.81E-07
SZRD1	0.231233	0.00022
TACC1	0.186862	0.006343
TACC3	0.359514	0.004671
TACSTD2	-0.81788	5.04E-07
TAF12	0.137309	0.047754
TAF1A	0.301035	0.011006
TAF1D	0.245236	0.00811
TAF3	0.170968	0.018936
TAF4	0.29969	3.70E-06
TAF4B	0.25387	3.40E-05
TAF5	0.241863	0.042447
TAF5L	0.127103	0.033094
TAF6	0.248236	0.026435
TAF9	0.166325	0.011085

TAGLN	-1.18486	6.85E-12
TAGLN3	1.683003	1.92E-15
TAL2	-3.18482	0.0275
TANC1	0.103657	0.040667
TANGO6	0.327493	6.28E-08
TANK	-0.18346	0.048233
TAP1	-0.39802	0.00611
TAP2	0.182376	0.000595
TAPBP	-0.23126	0.014198
TAPBPL	-0.29064	0.022219
TAPT1	-0.23406	0.008435
TARS	0.179967	0.007213
TARS2	0.173912	0.005256
TAS1R3	0.724129	0.034264
TAS2R4	-0.99977	0.021874
TATDN2	0.192881	0.002349
TBC1D1	0.224235	0.000139
TBC1D10C	-0.97763	0.004409
TBC1D12	-0.15858	0.017241
TBC1D20	-0.17653	0.001304
TBC1D23	-0.29296	0.002483
TBC1D26	-2.06352	0.031046
TBC1D31	0.217017	0.005683
TBC1D32	-0.33305	0.007692
TBC1D8	0.349008	2.16E-06
TBC1D8B	-0.36943	7.54E-06
TBC1D9	-0.68961	3.88E-11
TBCD	0.185527	0.032683
TBCEL	-0.55439	2.96E-09
TBCK	-0.27682	0.002282
TBK1	-0.19877	0.018645
TBKBP1	0.524102	0.002615
TBL1X	0.134627	0.047489
TBL1XR1	0.180924	0.02312
TBL3	0.42255	0.015705
TBP	0.135143	0.02707
TBPL1	0.162327	0.013132
TBRG4	0.447737	9.57E-09
TBX2	1.286965	0.017508
TBX21	2.359813	1.41E-17
TCEAL4	-0.32844	0.000471
TCEAL8	-0.19555	0.013759
TCEANC	-0.3624	0.044707
TCERG1	0.259578	9.14E-06
TCERG1L	0.763767	2.83E-11
TCF19	0.205929	0.013217
TCF20	0.228726	1.39E-05

TCF21	0.690502	0.046194
TCF3	0.479455	0.005393
TCF4	-0.18408	0.015898
TCF7	0.389551	0.00093
TCHHL1	-2.38321	0.01805
TCHP	0.217136	0.001808
TCN2	-0.457	0.007526
TCOF1	0.71716	1.44E-19
TCP1	0.308189	4.43E-06
TCP11L1	-0.30422	0.00034
TCP11L2	-0.23781	0.012669
TCTN1	-0.19429	0.003219
TDO2	-0.39394	0.016971
TDP1	0.112218	0.030145
TDRD7	-0.64831	0.004569
TDRP	-0.27987	0.000123
TEAD4	0.53579	5.96E-07
TELO2	0.421472	0.041561
TENM1	-0.67686	0.00019
TENM2	-0.44156	2.92E-12
TENM3	0.262742	0.014729
TENM4	-0.30322	0.005458
TERF2	0.138656	0.044524
TERT	1.784748	8.45E-06
TET1	-1.55809	1.60E-14
TET3	0.176137	0.003084
TEX10	0.35508	1.88E-07
TEX14	-0.78089	0.023086
TEX19	1.211146	0.019352
TEX30	0.232532	0.00176
TEX40	0.640126	1.77E-06
TFAM	0.259055	0.0027
TFAP4	0.486734	0.000414
TFCP2L1	-0.72306	3.12E-18
TFDP1	0.179183	0.000137
TFDP2	-0.24748	4.99E-05
TFPI	-0.27633	0.012763
TFPI2	1.561024	5.93E-23
TFRC	0.276013	0.016342
TG	-0.6021	0.002275
TGFA	0.57661	1.07E-13
TGFB1	0.365226	0.030535
TGFB2	-1.15297	2.26E-26
TGFB2-AS1	-1.17316	3.05E-13
TGFB2-OT1	-7.18399	0.017698
TGFB3	-0.30598	0.037904
TGFBR3	0.580175	1.77E-08

TGIF2-C20c	0.603278	0.016207
TGM5	-1.38112	0.045133
TGS1	0.177126	0.006336
THADA	0.246339	5.89E-05
THAP4	0.50261	6.45E-05
THAP7-AS1	-0.39738	0.001824
THBD	0.406024	0.020019
THBS2	-0.46377	0.03752
THBS3	-0.40237	0.000606
THCAT158	-1.48719	0.027965
THEG	1.102546	0.001644
THEM4	0.152026	0.042726
THOC5	0.220385	1.85E-05
THOP1	0.427621	0.003683
THRB	-0.26545	0.04678
THSD4	-0.29458	0.001964
THY1	0.679379	0.00179
TIAM1	0.630449	1.55E-18
TIAM2	-0.30105	0.009633
TICRR	0.352585	0.00745
TIGD2	-0.35964	3.29E-06
TIGD6	-0.18887	0.027847
TIMELESS	0.28089	1.53E-06
TIMM10	0.440136	3.59E-05
TIMM13	0.330554	0.010234
TIMM17B	0.284386	0.014731
TIMM22	0.241855	0.000552
TIMM23	0.200775	6.46E-05
TIMM23B	0.171599	0.013328
TIMM44	0.349824	0.00064
TIMM50	0.330614	3.61E-05
TIMM8A	0.175791	0.041964
TIMM9	0.17121	0.004234
TIMP1	0.724555	1.31E-05
TIMP2	-0.5531	3.68E-11
TIPIN	0.32929	0.000112
TIRAP	-0.31059	0.000878
TJP2	0.234829	2.31E-07
TJP3	-1.35522	0.014528
TK1	0.314195	0.017131
TK2	-0.15119	0.018274
TKFC	0.236636	0.033612
TKT	0.216658	0.035123
TLCD2	-0.76481	1.26E-07
TLDC2	-1.18642	0.004363
TLE3	0.342918	0.003528
TLE4	-0.31694	0.001396

TLL1	-0.2946	0.000397
TLR2	-0.36234	0.000628
TLR5	-0.54335	0.000655
TM4SF1	0.150052	0.031043
TM4SF19	0.483589	6.84E-07
TM4SF19-A	0.321489	0.018935
TM7SF3	-0.36018	8.94E-05
TM9SF2	-0.17865	0.001687
TM9SF4	-0.18761	0.026429
TMA16	0.192757	0.007828
TMBIM1	-0.14159	0.049048
TMBIM4	-0.17364	0.015144
TMC1	-0.70722	0.030714
TMC3	-0.46799	0.023995
TMC4	-1.10781	8.09E-06
TMC7	0.218073	0.03091
TMCC2	-0.41817	0.015535
TMCC3	0.459035	0.000236
TMCO3	-0.15005	0.003931
TMCO6	0.328725	0.000187
TMED1	0.229279	0.028042
TMED10	-0.12929	0.00687
TMED10P2	-1.32388	0.012155
TMED4	-0.16402	0.028072
TMED7-TIC	-0.53644	0.000299
TMEM106I	-0.30572	0.002145
TMEM106(	0.097053	0.041451
TMEM109	0.188594	0.027916
TMEM11	0.29272	0.000425
TMEM116	-0.24402	0.039576
TMEM117	-0.36907	0.000156
TMEM123	-0.22175	0.001721
TMEM126/	0.191539	0.035759
TMEM128	-0.23132	0.004349
TMEM132I	-1.1621	0.004111
TMEM133	-0.54623	0.000825
TMEM140	-0.45022	0.004355
TMEM141	0.309626	0.00134
TMEM144	-0.27827	0.002736
TMEM145	0.75029	0.004466
TMEM147	0.341592	0.008704
TMEM150(	-0.93535	0.009231
TMEM154	0.227299	0.009443
TMEM156	0.362363	0.012629
TMEM159	-0.35437	0.005984
TMEM161I	-0.22018	0.037457
TMEM164	0.344383	4.81E-08

TMEM165	-0.30658	7.25E-11
TMEM168	-0.3153	0.005963
TMEM170A	0.185634	0.042912
TMEM171	0.314287	0.047949
TMEM177	0.291675	0.004077
TMEM178B	-1.26006	0.000178
TMEM18	0.132946	0.023888
TMEM185A	-0.25174	0.006546
TMEM19	-0.2342	0.00183
TMEM200A	0.992477	8.57E-12
TMEM201	0.680823	0.000144
TMEM204	0.506867	0.003473
TMEM206	0.300104	0.000299
TMEM208	0.133942	0.027529
TMEM209	0.149647	0.032517
TMEM229B	-0.3148	0.00341
TMEM232	-1.24018	0.027072
TMEM237	-0.20025	0.000114
TMEM254	-0.22793	0.000414
TMEM255A	-1.30856	1.62E-17
TMEM258	0.120481	0.032377
TMEM260	-0.28114	0.000154
TMEM261	0.282403	0.007108
TMEM268	0.250642	2.83E-06
TMEM27	-0.46923	6.76E-05
TMEM30B	-0.37996	1.53E-08
TMEM33	0.204823	0.001953
TMEM35B	-0.18859	0.047153
TMEM38B	0.212756	0.035526
TMEM39B	0.292527	0.011
TMEM44-A	-0.3687	0.017566
TMEM45A	-0.24493	0.009628
TMEM5	0.127312	0.015198
TMEM50A	-0.11865	0.018529
TMEM50B	-0.25924	0.001827
TMEM52B	-0.67294	0.013836
TMEM56	-0.40248	0.037724
TMEM59	-0.33947	0.000214
TMEM62	-0.57145	6.96E-07
TMEM63B	-0.31053	0.005329
TMEM63C	-0.50444	0.002649
TMEM69	0.173227	0.005858
TMEM86A	-0.66923	0.000338
TMEM9	-0.21518	0.00915
TMEM92	0.387342	0.001682
TMEM97	0.314039	0.001636
TMEM9B	-0.14045	0.009496

TMEM9B-A	-0.78132	0.006807
TMLHE	-0.22894	0.005889
TMOD1	-0.8558	0.021587
TMOD2	-0.69539	2.03E-07
TMPO	0.202233	0.002978
TMPRSS11I	0.534939	0.009937
TMPRSS13	-0.98973	0.000622
TMSB10	0.240146	4.21E-06
TMTC1	-0.27247	0.030489
TMTC2	-0.32557	0.000687
TMX2	0.156631	0.01452
TNC	-0.92164	7.33E-20
TNFAIP2	-0.92237	0.003197
TNFAIP8L2	-1.8994	0.02252
TNFRSF10A	0.458171	1.16E-08
TNFRSF10E	0.188253	0.004207
TNFRSF10D	0.494149	0.005657
TNFRSF12A	0.402153	0.003213
TNFRSF14	-0.40461	0.02258
TNFRSF19	0.61905	2.27E-17
TNFRSF1B	0.319124	0.006016
TNFRSF25	0.448898	0.004473
TNFRSF6B	1.822481	0.002551
TNFRSF8	1.132568	1.14E-08
TNFRSF9	-0.59671	0.000192
TNFSF10	-1.20118	0.000673
TNFSF13B	-1.29714	0.000205
TNFSF18	0.835492	0.04538
TNIK	0.797997	3.63E-27
TNIP1	0.213307	0.011193
TNIP2	0.315104	0.006272
TNIP3	-0.85607	7.31E-06
TNNT1	0.399694	0.004751
TNNT3	-1.02376	0.003607
TNPO2	0.163944	0.003384
TNRC6A	-0.1609	0.004182
TNRC6C-AS	-0.43301	0.000972
TNS3	-0.35736	0.000147
TNS4	0.622394	9.43E-08
TOE1	0.276721	0.014464
TOMM22	0.243976	0.000117
TOMM34	0.660941	2.71E-13
TOMM40	0.544346	1.69E-05
TOMM5	0.350022	5.48E-06
TOMM6	0.254812	0.001108
TONSL	0.481525	0.010437
TOP1	0.179863	0.008405

TOP1MT	0.461189	1.31E-05
TOP2A	0.250712	0.005771
TOP3A	0.292758	2.71E-06
TOP3B	0.172803	0.036285
TOPBP1	0.172202	0.000469
TOPORS-AS	-0.39349	0.038754
TOR4A	0.590791	0.00687
TP53AIP1	-0.53074	1.48E-06
TP53I3	-0.39714	7.25E-05
TP53INP1	-0.98024	4.04E-11
TP53INP2	-0.73685	1.71E-10
TP53TG1	-0.41451	0.003412
TPBG	0.286458	0.00265
TPD52L1	-0.15564	0.010765
TPD52L2	0.193585	8.98E-05
TPI1	0.16279	0.007392
TPI1P2	-0.66991	0.01033
TPM1	-0.70118	8.68E-26
TPM3	0.139323	0.008719
TPM4	-0.2729	3.59E-08
TPP2	0.143203	0.028831
TPRN	0.461801	0.011307
TPT1	0.170123	0.007436
TPTEP1	-0.36069	0.006998
TPX2	0.345583	5.10E-06
TRABD2A	0.705245	2.44E-14
TRAF2	0.284632	0.018204
TRAF3IP1	-0.17401	0.025748
TRAF3IP2-A	0.353223	0.020211
TRAF3IP3	-0.84011	0.005091
TRAF5	-0.21633	0.019828
TRAF7	0.273105	0.025586
TRAFD1	-0.36504	0.002367
TRAK2	-0.21139	0.002105
TRAM1	-0.35298	0.000537
TRAM2	0.192625	0.005479
TRANK1	-0.89196	0.003913
TRAP1	0.516473	5.65E-11
TRAPPC2	-0.14605	0.023692
TRAPPC6A	-0.55792	0.001318
TRAPPC9	-0.34886	0.006268
TRBC2	1.749956	1.31E-08
TREML2	1.626195	0.003437
TRERF1	0.393388	1.82E-06
TRIB1	0.628944	2.04E-08
TRIB3	0.427623	0.002344
TRIM16	-0.26641	0.00019

TRIM16L	-0.28872	0.005382
TRIM2	-0.76171	1.05E-10
TRIM21	-0.31085	0.00964
TRIM22	-1.13214	0.001847
TRIM24	-0.14899	0.02658
TRIM28	0.39488	0.00112
TRIM29	-0.3643	0.033735
TRIM34	-0.53349	1.33E-08
TRIM36	0.263381	0.027399
TRIM38	-0.2819	0.00285
TRIM4	-0.23103	0.010412
TRIM55	-0.7935	0.005852
TRIM56	-0.41351	7.26E-09
TRIM6	-0.45773	7.47E-09
TRIM68	-0.12788	0.038931
TRIM69	-0.5251	0.00046
TRIM73	-0.37997	0.018303
TRIM8	0.271727	0.010241
TRIO	-0.13083	0.003072
TRIP13	0.397812	6.75E-05
TRIQK	-0.35293	0.002054
TRIT1	0.114851	0.042584
TRMT1	0.530927	3.80E-05
TRMT112	0.302285	0.000343
TRMT1L	-0.22089	0.015132
TRMT2A	0.283071	0.046166
TRMT6	0.31958	2.74E-09
TRMT61A	0.471789	0.011222
TRNP1	0.211586	0.041711
TROAP	0.434073	0.001949
TRPA1	-0.71562	0.025624
TRPM2	0.539778	0.002087
TRPM4	-0.34175	0.012686
TRPM7	-0.15909	0.025128
TRPV1	-0.27929	0.016417
TRPV3	0.612857	0.000947
TRPV6	-1.42047	0.000144
TRRAP	0.206856	0.000349
TRUB2	0.226977	1.19E-05
TSC1	-0.12341	0.025264
TSC22D1	0.304276	7.35E-06
TSC22D3	-0.22487	0.006307
TSEN2	0.270919	0.001675
TSEN54	0.243326	0.042583
TSFM	0.174202	0.005889
TSHZ2	-0.3437	0.012062
TSHZ3	0.172118	0.03371

TSIX	0.259346	0.008474
TSPAN2	-0.53605	4.06E-06
TSPAN3	-0.2114	0.000183
TSPAN31	-0.35902	0.001314
TSPAN32	-1.34721	0.035161
TSPAN5	0.206977	0.002464
TSPEAR-AS	-0.72297	0.010241
TSPEAR-AS	-0.92864	0.005242
TSPOAP1	-0.83902	0.001177
TSPOAP1-A	-1.18372	0.040182
TSPYL2	0.28014	0.002849
TSR1	0.410721	4.16E-18
TSSC1	0.210184	0.008923
TSSK3	-0.75431	0.002854
TSTA3	0.3297	0.001685
TSTD3	-0.45987	0.006278
TTC13	-0.62507	1.64E-05
TTC23	-0.16842	0.0145
TTC27	0.218356	0.003289
TTC28	-0.45685	0.018659
TTC28-AS1	-0.67409	1.16E-10
TTC3	-0.21587	0.000487
TTC30A	-0.41153	2.12E-06
TTC30B	-0.30092	0.005078
TTC34	-0.48846	0.008365
TTC3P1	-0.33753	0.034682
TTC4	0.172735	0.00072
TTC9B	1.209038	0.036082
TTF1	0.123555	0.045228
TTI1	0.23107	0.000475
TTI2	0.166275	0.034074
TTK	0.344484	0.000987
TTL	0.257637	4.41E-07
TTLL1	-0.53241	0.007911
TTLL12	0.591935	2.11E-05
TTLL4	0.39426	1.86E-10
TTLL5	0.106743	0.022925
TTLL7	-0.57852	2.91E-07
TTN	-0.27412	0.004701
TTYH2	0.426778	0.000627
TUB	0.335308	0.005059
TUBA1A	-0.63073	1.36E-09
TUBA1B	0.337865	1.70E-05
TUBA1C	0.229706	0.01363
TUBB	0.182022	0.012944
TUBB3	0.283911	0.041849
TUBB4B	0.3632	0.005697

TUBB6	0.442635	2.95E-05
TUBB8P12	-1.25134	0.032954
TUBG1	0.351193	3.88E-05
TUBGCP2	0.250334	0.004053
TUBGCP3	0.141158	0.003187
TUBGCP4	0.129426	0.036802
TUFM	0.401163	2.30E-06
TUG1	-0.18357	0.005287
TULP4	-0.14031	0.022685
TUT1	0.289161	0.001937
TWIST1	0.339026	0.010457
TWISTNB	0.175318	0.008959
TWNK	0.372334	1.03E-07
TXLNG	0.236155	0.000582
TXNDC12	0.138949	0.00152
TXNDC15	-0.15835	0.014909
TXNDC17	0.236399	0.005876
TXNDC5	0.215228	0.000214
TXNL4A	0.155607	0.014244
TXNRD3	-0.25504	0.007121
TYMS	0.162616	0.037136
U2AF1	0.212577	0.003104
U2AF2	0.373535	0.000317
U47924.27	-0.4368	0.028361
UACA	0.294256	0.000104
UAP1	0.425948	1.56E-06
UBA1	0.191734	0.001335
UBA2	0.249308	5.04E-06
UBA52	0.146183	0.005043
UBA7	-0.51827	0.005419
UBALD2	0.29757	0.005855
UBAP2L	0.109485	0.038186
UBASH3B	0.488501	5.49E-12
UBD	-1.79047	9.06E-05
UBE2C	0.463893	0.000443
UBE2E2	0.198619	0.041062
UBE2H	-0.24369	0.009073
UBE2I	0.235821	0.000272
UBE2J1	-0.1703	0.00804
UBE2M	0.263436	0.005485
UBE2N	0.151606	0.007623
UBE2O	0.21835	0.010591
UBE2Q2P1	-0.54228	0.014308
UBE2QL1	-1.41196	1.14E-08
UBE2R2	0.147055	0.027837
UBE2S	0.453139	0.031221
UBE2T	0.279163	0.000117

UBE3B	-0.15261	0.01848
UBIAD1	0.225265	0.025241
UBL3	-0.35476	4.69E-06
UBL4A	0.203698	0.010398
UBL7	0.247181	0.008515
UBN2	-0.34827	0.000297
UBOX5	0.280089	0.001678
UBQLN2	-0.18112	0.00184
UBQLN4	0.23647	7.24E-05
UBQLN4P1	1.06482	0.009859
UBR4	0.125843	0.008787
UBXN1	0.235866	0.014238
UBXN11	0.314162	0.001603
UBXN2B	-0.39139	1.29E-05
UBXN4	-0.2094	0.0143
UCHL3	0.311221	0.000161
UCHL5	0.152574	0.049059
UCK2	0.442662	1.69E-08
UCKL1	0.236598	0.048262
UCP2	0.483314	0.037983
UEVLD	-0.29637	0.000327
UGCG	0.345408	0.002216
UGDH-AS1	-0.36884	4.32E-05
UGGT2	-0.34788	0.00164
UGP2	-0.18384	0.019998
UGT1A1	-0.30858	0.003555
UGT1A10	-0.74039	2.42E-13
UGT1A2P	-0.70886	0.049284
UGT1A3	-0.66854	0.008005
UGT1A6	-0.41993	1.22E-05
UGT1A7	-0.76422	0.008366
UGT1A8	-0.95738	1.85E-10
UGT2B17	-1.61104	1.55E-06
UGT8	0.60976	9.25E-06
UHRF1	0.546319	1.01E-06
UIMC1	0.24159	0.004682
ULBP3	0.261527	0.031356
ULK2	-0.38315	5.88E-07
UNC13B	-0.2472	0.013637
UNC13C	-0.77059	0.049708
UNC13D	0.507698	0.001162
UNC50	-0.1344	0.045441
UNC93A	-0.72385	4.87E-05
UNG	0.195027	0.022619
UNGP3	-1.31565	0.00152
UPF1	0.229451	0.03383
UPK2	-0.86048	0.005962

UPK3B	-1.10386	0.000116
UPP1	0.959374	2.24E-21
UQCRC1	0.18953	0.045116
UQCRFS1	0.209366	0.000347
URB1	0.265844	1.62E-10
URB2	0.309152	0.000488
URM1	0.243658	0.005493
USE1	0.235401	0.039931
USP1	0.178198	0.033518
USP10	0.129615	0.011298
USP17L4	3.404487	0.002231
USP2-AS1	0.948903	5.03E-07
USP3	0.192499	0.001469
USP30-AS1	-1.00921	0.025624
USP31	-0.28983	0.001008
USP32P1	-0.3552	0.013045
USP32P2	-0.53044	0.001361
USP32P3	-0.44962	0.00081
USP33	-0.24537	0.002856
USP36	0.340447	1.46E-07
USP39	0.165995	0.00347
USP4	0.138352	0.040219
USP46-AS1	-0.74002	4.89E-06
USP49	-0.29199	0.001672
USP9X	-0.12948	0.040515
UST	-0.60228	1.73E-12
UTAT33	-0.97683	0.0434
UTP11	0.250829	0.001107
UTP14A	0.285875	3.56E-06
UTP14C	-0.18593	0.042346
UTP18	0.367198	7.23E-08
UTP20	0.220287	0.002032
UTP4	0.380298	2.73E-19
UTP6	0.187802	0.015699
UTRN	-0.30115	2.37E-06
UXT	0.167726	0.019505
VAC14	0.281028	0.008684
VAMP1	-0.17376	0.046605
VAMP4	-0.19511	0.046835
VAMP8	-0.23967	0.000848
VANGL2	-0.34422	3.38E-06
VARS	0.541136	3.90E-05
VASH1	-0.39461	0.008615
VASN	0.5742	0.027674
VASP	0.434153	0.000226
VAV2	0.228418	0.002905
VAV3	-0.3559	1.57E-06

VCPKMT	-0.16519	0.032094
VDAC1	0.244055	9.88E-05
VEGFA	0.616538	6.78E-09
VEGFC	0.141056	0.019184
VEPH1	-0.3988	0.010566
VGF	1.042971	0.00156
VGLL1	-0.70391	0.004943
VGLL4	-0.14973	0.028338
VIL1	-1.60408	0.008909
VIM	0.474398	0.004237
VIPAS39	-0.11088	0.03914
VIPR1	-0.9762	3.72E-17
VIPR1-AS1	-1.03765	0.0038
VMAC	-0.40715	0.032765
VMP1	-0.16652	0.042294
VOPP1	0.188579	0.005944
VPS13A	-0.20076	0.049862
VPS13B	-0.20451	0.038859
VPS13C	-0.42745	0.000268
VPS13D	-0.19006	0.013199
VPS16	0.177967	0.017477
VPS25	0.120121	0.036724
VPS28	0.224285	0.022539
VPS37B	0.286012	0.025936
VPS39	-0.14445	0.024543
VPS45	-0.20167	0.002063
VPS50	-0.16441	0.047463
VPS52	0.124303	0.027864
VPS54	-0.18939	0.01279
VPS8	-0.1991	0.018012
VPS9D1-AS	0.735607	0.000227
VRK1	0.302373	0.00164
VSIG10	-0.3257	5.05E-05
VSIG10L	-0.35625	0.033698
VSTM2A	2.931255	0.046147
VSTM5	-1.05057	0.044983
VTCN1	-1.69787	0.001866
VWA2	-0.39494	0.004406
VWA3A	-2.68292	0.033201
VWA5A	-0.91804	7.30E-06
VWA8	-0.2419	0.007997
VWA8-AS1	-0.83489	0.021379
VWDE	-0.54603	6.58E-05
VWF	-0.51164	0.017024
WAC-AS1	-0.39987	3.19E-10
WARS2	0.136014	0.025494
WARS2-IT1	-0.42406	0.026615

WASF1	-0.1994	0.003831
WASHC1	0.395602	0.005199
WASHC2C	-0.10244	0.033846
WASHC3	-0.25967	3.84E-05
WBP11	0.192156	0.003544
WBP1L	-0.14885	0.003156
WBP2NL	-0.50249	0.042332
WBSCR22	0.241867	0.000456
WDFY3	-0.14963	0.037598
WDHD1	0.194066	0.019651
WDR11	-0.15338	0.004605
WDR12	0.286452	1.37E-05
WDR3	0.299194	0.000115
WDR31	-0.51981	3.17E-06
WDR36	0.173381	0.033253
WDR37	-0.38556	0.000452
WDR4	0.371646	0.003211
WDR43	0.344787	2.22E-08
WDR44	-0.15436	0.035163
WDR46	0.32728	0.000121
WDR5	0.262662	0.000359
WDR54	0.395413	0.000123
WDR55	0.171221	0.015621
WDR5B	-0.23623	0.046367
WDR62	0.440696	0.001705
WDR64	-2.81751	0.020144
WDR72	-0.23294	0.001703
WDR74	0.44784	2.21E-05
WDR75	0.1673	0.016948
WDR88	-0.5378	0.022713
WDR90	0.552879	0.0037
WDR91	-0.21522	0.005535
WDSUB1	-0.27858	0.006044
WDTC1	-0.18277	0.045022
WFDC2	-1.07351	0.001205
WFDC21P	-0.90602	0.00125
WHAMMP:	-0.35004	0.00437
WHAMMP:	-0.28613	0.028822
WIPI1	-0.64306	5.74E-10
WNK1	-0.11424	0.011281
WNT10A	-0.64258	0.000926
WNT10B	0.621892	0.0164
WNT11	-1.2834	0.007802
WNT2B	-0.54724	1.95E-05
WNT4	-1.15177	1.20E-07
WNT6	-1.23655	0.000126
WRAP53	0.409319	1.35E-05

WRB	-0.21173	0.025971
WRNIP1	0.186176	0.004982
WSCD1	1.322938	4.22E-11
WWC2-AS2	-0.6743	0.035296
WWP1	-0.13853	0.040177
XAB2	0.340838	0.006655
XAF1	-1.03376	0.048418
XCL1	-0.73469	0.00016
XCR1	-1.42385	0.030538
XDH	0.205027	0.008399
XG	-0.74001	6.30E-26
XPO4	0.196701	0.009637
XPO5	0.363685	6.54E-17
XPO6	0.25069	2.12E-07
XPO7	0.170562	0.000884
XPOT	0.215085	0.009298
XPR1	-0.47636	0.000123
XRCC1	0.257117	0.001399
XRCC3	0.44402	0.00189
XRCC5	0.17004	0.002531
XRCC6	0.165801	0.000535
XRN1	-0.22334	0.031602
XRN2	0.190444	0.000319
XRRA1	0.228936	0.038497
XXbac-BPG	-0.33316	0.019176
XXbac-BPG	-0.38534	0.028769
XXyac-YRM	-0.26551	0.041302
XYLB	0.439518	0.002213
XYLT1	0.466776	1.36E-07
YAP1	0.140448	0.031505
YARS	0.256989	2.62E-05
YARS2	0.221455	0.000867
YBX1	0.424077	1.07E-07
YBX1P1	0.515076	0.049572
YBX3	0.185329	0.000253
YEATS2	0.139406	0.020473
YIPF6	-0.2446	0.004203
YKT6	0.169271	0.000739
YOD1	0.334888	0.001683
YPEL3	-0.55921	0.00223
YPEL5	-0.54215	3.14E-07
YRDC	0.409959	1.93E-08
YTHDF3	-0.11588	0.03688
YWHAE	0.165406	0.004579
YWHAG	-0.20092	0.001787
Z84812.4	3.834982	0.001106
ZBED1	-0.38066	0.000511

ZBED2	0.847359	1.93E-08
ZBED3	-0.23789	0.040169
ZBED4	0.204134	0.000191
ZBED5	-0.12855	0.02906
ZBED5-AS1	-0.31599	0.026186
ZBED9	-0.27277	0.001814
ZBTB1	-0.25396	0.001416
ZBTB17	0.413897	0.001666
ZBTB2	0.352896	0.000114
ZBTB38	-0.14854	0.013822
ZBTB39	0.19	0.005181
ZBTB47	-0.28538	0.049521
ZBTB48	0.203423	0.035938
ZBTB5	-0.19945	0.027193
ZBTB9	0.231263	0.002468
ZC3H10	0.265141	0.024953
ZC3H18	0.264334	0.009878
ZC3H7B	0.205405	0.031083
ZC3HAV1	-0.29133	0.010275
ZCCHC2	-0.18196	0.034299
ZCCHC4	0.200473	0.019497
ZCWPW1	-0.21456	0.048548
ZDBF2	-0.28972	0.026405
ZDHHC11B	-0.54209	0.005681
ZDHHC18	0.178343	0.024099
ZDHHC20P	-1.84896	0.033449
ZDHHC21	-0.50555	0.000527
ZDHHC23	-0.44126	0.000735
ZDHHC6	0.110553	0.019049
ZDHHC7	-0.13617	0.016918
ZFAND2B	0.206657	0.024557
ZFAND5	-0.15201	0.006695
ZFAS1	0.192243	0.005942
ZFHX2	-0.88713	0.0059
ZFHX4	-0.24943	0.000966
ZFP1	0.225764	0.005158
ZFP14	-0.59736	4.69E-05
ZFP3	-0.85064	2.33E-05
ZFP30	-0.25029	0.014797
ZFP36	0.38498	0.025604
ZFP36L1	0.482824	3.24E-11
ZFP36L2	0.376225	3.37E-05
ZFP57	-0.7151	0.006325
ZFP69B	0.451804	0.004021
ZFP90	-0.20858	0.000153
ZFPM2-AS1	-0.2966	0.002313
ZFYVE16	-0.17423	0.02955

ZFYVE19	0.193095	0.003042
ZFYVE26	-0.15988	0.006893
ZGPAT	0.355157	0.003293
ZHX1	-0.1771	0.039306
ZIC1	-0.39345	6.92E-05
ZIC2	0.414573	0.001056
ZIC4	-0.7625	8.32E-07
ZKSCAN1	-0.31721	5.58E-06
ZKSCAN3	-0.57181	3.19E-08
ZKSCAN4	-0.26287	0.001933
ZMAT3	-0.21722	0.01879
ZMIZ1-AS1	-0.6615	0.001433
ZMYND19	0.425667	5.60E-06
ZMYND8	0.325337	4.74E-05
ZNF10	-0.44829	6.79E-05
ZNF117	-0.63347	5.38E-06
ZNF124	-0.27742	0.03316
ZNF134	-0.19791	0.004416
ZNF142	0.269813	0.000514
ZNF143	0.212499	0.004996
ZNF182	-0.23957	0.014223
ZNF189	-0.17647	0.031299
ZNF19	-0.41817	0.004335
ZNF202	0.165932	0.007393
ZNF204P	-0.48096	8.32E-06
ZNF211	-0.33004	4.13E-05
ZNF212	0.192752	0.022369
ZNF223	-0.29103	0.030518
ZNF224	-0.26446	0.021028
ZNF226	-0.28031	0.003609
ZNF23	-0.16214	0.038424
ZNF248	-0.40992	5.18E-05
ZNF25	-0.4611	0.00512
ZNF251	-0.2151	0.02755
ZNF252P	-0.27296	0.00461
ZNF253	-0.331	0.024657
ZNF254	-0.47279	0.000108
ZNF260	-0.23445	0.027952
ZNF263	0.162661	0.013045
ZNF264	-0.33188	0.000637
ZNF274	0.159695	0.046766
ZNF276	0.223455	0.022935
ZNF28	-0.21866	0.039202
ZNF280D	-0.27008	0.001403
ZNF281	-0.19464	0.006945
ZNF282	0.437101	0.000951
ZNF30	-0.26776	0.028243

ZNF302	-0.32717	0.000144
ZNF311	-0.32499	0.020241
ZNF319	-0.27636	0.029645
ZNF320	-0.33567	0.004714
ZNF321P	-0.45251	0.00648
ZNF322	-0.22776	0.02458
ZNF329	-0.2244	0.005165
ZNF330	0.24558	0.000299
ZNF334	-0.41448	2.19E-05
ZNF335	0.25023	0.028689
ZNF337-AS	-0.33849	0.00972
ZNF33A	-0.24706	0.001774
ZNF33B	-0.19917	0.007459
ZNF343	0.178233	0.00979
ZNF350	-0.26595	0.013834
ZNF37BP	-0.20905	0.028949
ZNF384	0.210172	0.000219
ZNF385C	-0.65767	0.036806
ZNF391	-0.40764	1.44E-05
ZNF396	-0.71577	0.001096
ZNF416	-0.25915	0.03743
ZNF417	-0.31171	4.15E-05
ZNF420	-0.33156	0.042856
ZNF425	-0.6153	0.000763
ZNF433	-0.34804	0.03646
ZNF436	-0.18611	0.001668
ZNF439	-0.59494	0.030522
ZNF45	-0.17601	0.006609
ZNF474	0.485415	1.97E-05
ZNF48	-0.35262	0.012818
ZNF485	0.252178	0.045496
ZNF496	0.222523	0.014079
ZNF503-AS	-0.60143	0.017652
ZNF506	-0.22091	0.012186
ZNF510	-0.19721	0.026744
ZNF511	0.365014	0.007039
ZNF514	-0.3187	0.000977
ZNF528	-0.33459	1.05E-05
ZNF528-AS	-0.30122	0.010262
ZNF532	-0.1319	0.009029
ZNF540	-0.89245	0.004332
ZNF546	-0.38372	0.042649
ZNF547	-0.38644	0.020667
ZNF552	-0.25174	0.022654
ZNF554	-0.463	0.026282
ZNF561	-0.28108	0.000327
ZNF561-AS	-0.35736	0.004078

ZNF563	-0.39213	0.004243
ZNF570	-0.32915	0.005323
ZNF571	-0.3063	0.032736
ZNF572	-0.29665	0.013704
ZNF575	-0.87831	0.007468
ZNF585A	-0.24554	0.016108
ZNF585B	-0.32514	0.002199
ZNF587	-0.19752	0.022455
ZNF592	0.146873	0.016078
ZNF593	0.447482	0.027021
ZNF594	-0.25398	0.011684
ZNF596	-0.46864	0.009605
ZNF598	0.483051	0.008722
ZNF599	-0.41235	0.000141
ZNF605	-0.28158	0.000641
ZNF606	-0.43664	2.47E-06
ZNF608	-0.31541	0.00106
ZNF611	-0.33478	0.003036
ZNF615	-0.2922	0.004279
ZNF622	0.153835	0.018884
ZNF625-ZN	-0.4383	0.021788
ZNF629	-0.30066	0.000708
ZNF638	-0.19688	0.008033
ZNF641	-0.23322	0.009611
ZNF652	-0.24239	0.00773
ZNF658	-0.35588	0.000607
ZNF658B	-0.55988	0.002708
ZNF692	0.34973	0.001153
ZNF697	0.493713	1.01E-07
ZNF70	-0.2578	0.014099
ZNF704	-0.52232	9.48E-13
ZNF706	0.1874	0.001434
ZNF713	-0.30408	0.011355
ZNF720	-0.32397	0.004078
ZNF74	0.260261	0.014653
ZNF75D	-0.26289	0.00252
ZNF765	-0.27878	0.005647
ZNF767P	-0.21453	0.017739
ZNF772	-0.19228	0.005149
ZNF773	-0.24366	0.016068
ZNF774	-0.54649	2.16E-07
ZNF776	-0.18341	0.022998
ZNF778	0.192781	0.012114
ZNF780A	-0.36708	0.00322
ZNF780B	-0.35037	0.000609
ZNF789	-0.26126	0.006967
ZNF791	-0.2396	0.001624

ZNF81	-0.35586	0.000643
ZNF812P	-1.60881	9.63E-05
ZNF813	-0.22595	0.049038
ZNF814	-0.32415	0.000164
ZNF815P	-0.47054	0.036365
ZNF816	-0.24208	0.044762
ZNF836	-0.31534	0.001853
ZNF84	-0.27347	0.00337
ZNF844	-0.60192	0.015405
ZNF846	-0.73198	2.86E-08
ZNF862	-0.76423	6.43E-07
ZNF879	-0.5522	0.00287
ZNF891	-0.25418	0.032
ZNF91	-0.39037	1.21E-06
ZNHIT6	0.235514	0.018986
ZNRD1	0.469791	2.89E-08
ZNRD1ASP	-0.3931	0.000179
ZPR1	0.311996	1.04E-08
ZRANB2-AS	-1.61763	0.044329
ZSCAN16	-0.43466	0.040123
ZSCAN26	-0.22891	0.013763
ZSCAN30	-0.27589	8.66E-06
ZSCAN31	-0.7196	5.43E-07
ZW10	0.162156	0.01029
ZWILCH	0.264002	0.000595
ZWINT	0.322697	9.98E-06
ZYG11B	-0.28327	0.011301

Supplementary Table 6 - Differential binding affinity between cetuximab and PBS across SCC1, SCC6 and

seqnames	start	end	width	Fold	p.value	FDR	genename	promoterst
chr8	1.24E+08	1.24E+08	465	-0.88	3.74E-12	1.00E-07	FAM83A	1.24E+08
chr8	1.24E+08	1.24E+08	465	-0.88	3.74E-12	1.00E-07	FAM83A	1.24E+08
chr1	67628697	67629065	369	-1.5	1.40E-10	1.07E-06	IL23R	67627168
chr11	27489816	27490486	671	-1.14	1.57E-10	1.12E-06	LOC10537(	27488277
chr11	27489816	27490486	671	-1.14	1.57E-10	1.12E-06	LOC10537(	27488277
chr11	1.23E+08	1.23E+08	274	-1.99	1.38E-08	2.79E-05	CLMP	1.23E+08
chr15	91412871	91413305	435	-0.94	1.87E-08	3.45E-05	FURIN	91409768
chr15	91412871	91413305	435	-0.94	1.87E-08	3.45E-05	FURIN	91406821
chr15	91412871	91413305	435	-0.94	1.87E-08	3.45E-05	FURIN	91411107
chr12	41084233	41084479	247	-1.66	1.95E-08	3.53E-05	CNTN1	41081243
chr12	41084233	41084479	247	-1.66	1.95E-08	3.53E-05	CNTN1	41081243
chr1	1.7E+08	1.7E+08	664	-0.82	1.03E-07	0.000124	SELL	1.7E+08
chr1	1.7E+08	1.7E+08	664	-0.82	1.03E-07	0.000124	SELL	1.7E+08
chr12	95012793	95013041	249	-1.17	4.96E-07	0.000379	TMCC3	95007927
chr14	1.02E+08	1.02E+08	702	-0.91	8.58E-07	0.000585	LINC00239	1.02E+08
chr16	27326810	27327424	615	-1.08	9.24E-07	0.000614	IL4R	27320229
chr16	27326810	27327424	615	-1.08	9.24E-07	0.000614	IL4R	27320229
chr16	27326810	27327424	615	-1.08	9.24E-07	0.000614	IL4R	27320229
chr16	27326810	27327424	615	-1.08	9.24E-07	0.000614	IL4R	27320229
chr17	15009939	15010150	212	-1.12	9.36E-07	0.000618	CDRT8	15007504
chr2	23603673	23604292	620	-0.94	1.01E-06	0.000646	KLHL29	23603297
chr4	54933455	54933698	244	-1.2	1.18E-06	0.000723	CHIC2	54928815
chr6	1.47E+08	1.47E+08	238	-1.02	1.48E-06	0.000838	LUADT1	1.47E+08
chr2	1.55E+08	1.55E+08	217	2.32	1.86E-06	0.00101	GALNT13	1.55E+08
chr3	24565793	24566185	393	-1	2.50E-06	0.00124	MIR4792	24560926
chr16	31366328	31366536	209	-1.36	3.05E-06	0.0014	ITGAX	31361454
chr16	31366328	31366536	209	-1.36	3.05E-06	0.0014	ITGAX	31361454
chr18	12255567	12256025	459	-1.02	3.74E-06	0.00164	CIDEA	12249317
chr18	12255567	12256025	459	-1.02	3.74E-06	0.00164	CIDEA	12249359
chr18	12255567	12256025	459	-1.02	3.74E-06	0.00164	CIDEA	12249359
chr5	1.4E+08	1.4E+08	257	-1.72	4.19E-06	0.00176	HBEGF	1.4E+08
chr3	1.8E+08	1.8E+08	739	-1.17	4.48E-06	0.00182	LOC10050!	1.8E+08
chr11	1.29E+08	1.29E+08	605	-0.82	4.92E-06	0.00195	ARHGAP32	1.29E+08
chr13	76195309	76195660	352	2.59	5.31E-06	0.00207	LMO7	76189569
chr5	1.73E+08	1.73E+08	290	-1.57	5.75E-06	0.0022	LINC01485	1.73E+08
chr1	2.45E+08	2.45E+08	335	-1.07	5.81E-06	0.0022	C1orf100	2.45E+08
chr1	2.45E+08	2.45E+08	335	-1.07	5.81E-06	0.0022	C1orf100	2.45E+08
chr1	2.45E+08	2.45E+08	335	-1.07	5.81E-06	0.0022	C1orf100	2.45E+08
chr11	93450736	93450984	249	4.01	6.56E-06	0.00238	SCARNA9	93449679
chr8	1.24E+08	1.24E+08	246	-0.8	7.05E-06	0.0025	FAM83A	1.24E+08
chr8	1.24E+08	1.24E+08	246	-0.8	7.05E-06	0.0025	FAM83A	1.24E+08
chr8	1.24E+08	1.24E+08	246	-0.8	7.05E-06	0.0025	FAM83A	1.24E+08
chr2	65594709	65594970	262	-1.16	7.46E-06	0.00261	SPRED2	65591931
chr11	8835815	8836167	353	-0.8	1.04E-05	0.00336	ST5	8830224

chr11	8835815	8836167	353	-0.8	1.04E-05	0.00336	ST5	8830882
chr4	71063704	71063913	210	1.92	1.26E-05	0.00377	ODAM	71057243
chr1	2.12E+08	2.12E+08	392	-1.06	1.30E-05	0.00382	LOC10192S	2.12E+08
chr1	2.12E+08	2.12E+08	392	-1.06	1.30E-05	0.00382	LOC10192S	2.12E+08
chr12	51742910	51743165	256	1.99	1.84E-05	0.00475	CELA1	51738463
chr3	1.89E+08	1.89E+08	289	-1.2	1.86E-05	0.00475	TPRG1	1.89E+08
chr11	7986954	7987338	385	-1.97	2.25E-05	0.00537	NLRP10	7983059
chr3	1.37E+08	1.37E+08	793	-0.83	2.52E-05	0.00581	SLC35G2	1.37E+08
chr3	1.37E+08	1.37E+08	793	-0.83	2.52E-05	0.00581	SLC35G2	1.37E+08
chr3	1.37E+08	1.37E+08	793	-0.83	2.52E-05	0.00581	SLC35G2	1.37E+08
chr18	3015276	3015558	283	-1.65	2.63E-05	0.00596	LPIN2	3009945
chr5	64061881	64062308	428	-1.74	2.66E-05	0.00598	CWC27	64059744
chr5	64061881	64062308	428	-1.74	2.66E-05	0.00598	CWC27	64059744
chr5	64061881	64062308	428	-1.74	2.66E-05	0.00598	CWC27	64059744
chr5	64061881	64062308	428	-1.74	2.66E-05	0.00598	CWC27	64059744
chr14	74223246	74223443	198	-0.9	3.14E-05	0.00673	MIR4505	74220449
chr8	74332070	74332552	483	-1.52	3.20E-05	0.00681	STAU2-AS1	74327308
chr12	1.1E+08	1.1E+08	99	-1.34	3.53E-05	0.0072	FAM222A-/	1.1E+08
chr12	1.1E+08	1.1E+08	99	-1.34	3.53E-05	0.0072	FAM222A-/	1.1E+08
chr15	81070584	81070745	162	-1.16	3.71E-05	0.00741	CEMIP	81066683
chr15	81070584	81070745	162	-1.16	3.71E-05	0.00741	CEMIP	81066683
chr15	81070584	81070745	162	-1.16	3.71E-05	0.00741	CEMIP	81066683
chr3	1.19E+08	1.19E+08	458	-0.92	3.90E-05	0.00769	IGSF11-AS1	1.19E+08
chr1	54481946	54482409	464	-0.93	4.64E-05	0.00871	LDLRAD1	54481859
chr1	54481946	54482409	464	-0.93	4.64E-05	0.00871	LDLRAD1	54481859
chr1	54481946	54482409	464	-0.93	4.64E-05	0.00871	LDLRAD1	54481859
chr1	54481946	54482409	464	-0.93	4.64E-05	0.00871	LDLRAD1	54481859
chr1	54481946	54482409	464	-0.93	4.64E-05	0.00871	LDLRAD1	54481859
chr13	96187458	96187978	521	-1.08	5.28E-05	0.00952	CLDN10-AS	96184164
chr7	1.38E+08	1.38E+08	408	3.29	5.59E-05	0.00998	SVOPL	1.38E+08
chr1	2466219	2466742	524	-0.68	6.67E-05	0.0112	HES5	2459684
chr11	1.25E+08	1.25E+08	302	-0.88	6.93E-05	0.0115	ROBO4	1.25E+08
chr11	1.25E+08	1.25E+08	302	-0.88	6.93E-05	0.0115	ROBO4	1.25E+08
chr10	24498896	24499227	332	-1.34	7.55E-05	0.0121	KIAA1217	24492719
chr10	24498896	24499227	332	-1.34	7.55E-05	0.0121	KIAA1217	24492719
chr10	24498896	24499227	332	-1.34	7.55E-05	0.0121	KIAA1217	24492719
chr5	42993329	42993588	260	-1.02	8.44E-05	0.013	FLJ32255	42991435
chr17	19312954	19313202	249	-1.33	8.85E-05	0.0134	RNF112	19309490
chr8	1.29E+08	1.29E+08	346	2.43	9.44E-05	0.014	TMEM75	1.29E+08
chr17	55685197	55685456	260	-1.59	9.59E-05	0.0141	LOC101927	55683773
chr7	38380906	38381264	359	-0.89	1.00E-04	0.0144	TRG-AS1	38376177
chr11	1.25E+08	1.25E+08	1267	-0.45	0.000108	0.0152	CHEK1	1.25E+08
chr11	1.25E+08	1.25E+08	1267	-0.45	0.000108	0.0152	CHEK1	1.25E+08
chr11	1.25E+08	1.25E+08	1267	-0.45	0.000108	0.0152	CHEK1	1.25E+08
chr11	1.25E+08	1.25E+08	1267	-0.45	0.000108	0.0152	CHEK1	1.25E+08
chr11	1.25E+08	1.25E+08	1267	-0.45	0.000108	0.0152	CHEK1	1.25E+08
chr11	1.25E+08	1.25E+08	1267	-0.45	0.000108	0.0152	CHEK1	1.25E+08

chr20	17539186	17539624	439	-0.84	0.000115	0.0158	BFSP1	17537605
chr20	17539186	17539624	439	-0.84	0.000115	0.0158	BFSP1	17537605
chr20	17539186	17539624	439	-0.84	0.000115	0.0158	BFSP1	17537605
chr10	1.12E+08	1.12E+08	285	-0.58	0.000116	0.0159	MXI1	1.12E+08
chr2	1.8E+08	1.8E+08	298	-1.14	0.000129	0.0169	SESTD1	1.8E+08
chr8	57235354	57235609	256	-1.84	0.00013	0.017	SDR16C5	57231335
chr8	57235354	57235609	256	-1.84	0.00013	0.017	SDR16C5	57231335
chr8	57235354	57235609	256	-1.84	0.00013	0.017	SDR16C5	57231335
chrX	1.35E+08	1.35E+08	163	-2.21	0.000142	0.018	MAP7D3	1.35E+08
chr12	1.04E+08	1.04E+08	150	-0.79	0.000147	0.0182	LOC10192§	1.04E+08
chr10	1.04E+08	1.04E+08	690	-0.9	0.000147	0.0183	TRIM8	1.04E+08
chr7	3082693	3082939	247	-1.06	0.00015	0.0185	CARD11	3081579
chr13	1.04E+08	1.04E+08	229	-1.66	0.000158	0.0192	SLC10A2	1.04E+08
chr1	17695954	17696286	333	-0.76	0.000158	0.0192	PADI6	17693690
chr6	1.44E+08	1.44E+08	256	-0.91	0.000171	0.0201	PHACTR2	1.44E+08
chr6	1.44E+08	1.44E+08	256	-0.91	0.000171	0.0201	PHACTR2	1.44E+08
chr11	15096275	15096733	459	-0.69	0.000195	0.0217	CALCB	15090145
chr5	1.7E+08	1.7E+08	293	-1.56	0.000196	0.0218	LINC01366	1.7E+08
chr5	1.7E+08	1.7E+08	293	-1.56	0.000196	0.0218	LINC01366	1.7E+08
chr5	1.7E+08	1.7E+08	293	-1.56	0.000196	0.0218	LINC01366	1.7E+08
chr12	15113719	15114864	1146	-0.53	0.000207	0.0227	ARHGDIB	15112562
chr12	9517482	9517840	359	-1.19	0.000211	0.023	LOC10193(	9515059
chr19	58401838	58402575	738	2.83	0.000214	0.0231	ZNF814	58398442
chr3	54935232	54935570	339	3.38	0.000222	0.0235	CACNA2D3	54933282
chr6	52537273	52537537	265	-1.05	0.000224	0.0237	TMEM14A	52530883
chr13	44773107	44773257	151	-1.75	0.000227	0.0238	MIR8079	44768336
chr1	85085160	85085347	188	-1.06	0.000237	0.0245	LINC01461	85084714
chr15	45923591	45923847	257	-0.68	0.000238	0.0246	SQRDL	45921937
chr15	45923591	45923847	257	-0.68	0.000238	0.0246	SQRDL	45918345
chr7	1.51E+08	1.51E+08	295	-1.84	0.000242	0.0249	NOS3	1.51E+08
chr7	1.51E+08	1.51E+08	295	-1.84	0.000242	0.0249	NOS3	1.51E+08
chr7	1.51E+08	1.51E+08	295	-1.84	0.000242	0.0249	NOS3	1.51E+08
chr7	1.51E+08	1.51E+08	295	-1.84	0.000242	0.0249	NOS3	1.51E+08
chr1	12221876	12222317	442	-0.93	0.000272	0.0266	TNFRSF1B	12222059
chr1	12221876	12222317	442	-0.93	0.000272	0.0266	MIR7846	12221999
chr1	1.6E+08	1.6E+08	241	-0.94	0.000274	0.0266	VANGL2	1.6E+08
chr1	33812610	33812871	262	-1.25	0.000275	0.0267	LOC10192§	33810952
chr1	33226510	33227172	663	-0.56	0.000289	0.0274	KIAA1522	33226234
chr1	33226510	33227172	663	-0.56	0.000289	0.0274	KIAA1522	33226234
chr5	94417202	94417597	396	-0.78	3.00E-04	0.0282	MCTP1	94415570
chr5	94417202	94417597	396	-0.78	3.00E-04	0.0282	MCTP1	94415570
chr20	37054767	37054976	210	-1.01	0.000305	0.0284	SNORA71A	37054086
chr20	37054767	37054976	210	-1.01	0.000305	0.0284	SNORA71B	37051978
chr6	1.3E+08	1.3E+08	223	-0.85	0.000306	0.0284	TMEM244	1.3E+08
chr5	1.34E+08	1.34E+08	472	-0.76	0.000321	0.0293	LOC10192	1.34E+08
chr2	10438113	10438313	201	-1.49	0.000341	0.0304	HPCAL1	10438029
chr11	46718834	46719091	258	2.84	0.000349	0.0308	ZNF408	46717643

chr11	46718834	46719091	258	2.84	0.000349	0.0308	ZNF408	46717316
chr5	1.39E+08	1.39E+08	312	-0.68	0.000351	0.0308	MZB1	1.39E+08
chr6	2877728	2878030	303	-1.18	0.000352	0.0308	SERPINB9P	2874744
chr11	9023429	9023619	191	-1.19	0.000365	0.0314	NRIP3	9023596
chr22	22988325	22988721	397	3.38	0.000371	0.0315	GGTLC2	22983781
chr22	22988325	22988721	397	3.38	0.000371	0.0315	GGTLC2	22982090
chr22	22988325	22988721	397	3.38	0.000371	0.0315	POM121L1	22985012
chr6	1.1E+08	1.1E+08	287	-1.55	0.000373	0.0316	CCDC162P	1.1E+08
chr6	86114064	86114770	707	-0.95	0.000394	0.0327	LOC10192٤	86112520
chr6	1.38E+08	1.38E+08	255	-1.06	0.000408	0.0335	LOC100507	1.38E+08
chr6	1.38E+08	1.38E+08	255	-1.06	0.000408	0.0335	LOC100507	1.38E+08
chr11	60693352	60693673	322	-1.04	0.000464	0.0365	TMEM132/	60686912
chr11	60693352	60693673	322	-1.04	0.000464	0.0365	TMEM132/	60686912
chr5	95414846	95415059	214	-0.91	0.000477	0.0371	MIR583	95409841
chr8	1.24E+08	1.24E+08	542	-0.63	0.000479	0.0371	FAM83A	1.24E+08
chr1	2.1E+08	2.1E+08	220	-0.89	0.00049	0.0376	LAMB3	2.1E+08
chr1	2.1E+08	2.1E+08	220	-0.89	0.00049	0.0376	LAMB3	2.1E+08
chr1	2.1E+08	2.1E+08	220	-0.89	0.00049	0.0376	LAMB3	2.1E+08
chr16	58495100	58495274	175	-1.51	0.000501	0.0381	NDRG4	58492993
chr16	58495100	58495274	175	-1.51	0.000501	0.0381	NDRG4	58492548
chr16	58495100	58495274	175	-1.51	0.000501	0.0381	NDRG4	58493725
chr6	86113315	86113652	338	-0.86	0.000512	0.0385	LOC10192٤	86112520
chr11	1.14E+08	1.14E+08	968	-0.37	0.000514	0.0385	REXO2	1.14E+08
chr4	1.85E+08	1.85E+08	287	-0.74	0.000537	0.0397	LOC10272	1.85E+08
chr4	1.85E+08	1.85E+08	287	-0.74	0.000537	0.0397	LOC10272	1.85E+08
chr12	1.09E+08	1.09E+08	148	-1	0.000568	0.0411	CMKLR1	1.09E+08
chr12	1.09E+08	1.09E+08	148	-1	0.000568	0.0411	CMKLR1	1.09E+08
chr12	1.09E+08	1.09E+08	148	-1	0.000568	0.0411	CMKLR1	1.09E+08
chr16	58032492	58032712	221	-0.95	0.000595	0.0424	USB1	58030276
chr16	58032492	58032712	221	-0.95	0.000595	0.0424	ZNF319	58031762
chr16	58032492	58032712	221	-0.95	0.000595	0.0424	USB1	58030276
chr16	58032492	58032712	221	-0.95	0.000595	0.0424	USB1	58030276
chr7	89782159	89782271	113	-1.11	0.000609	0.0429	STEAP1	89778688
chr1	1.61E+08	1.61E+08	284	-0.79	0.000613	0.043	NIT1	1.61E+08
chr1	1.61E+08	1.61E+08	284	-0.79	0.000613	0.043	NIT1	1.61E+08
chr1	1.61E+08	1.61E+08	284	-0.79	0.000613	0.043	NIT1	1.61E+08
chr1	1.61E+08	1.61E+08	284	-0.79	0.000613	0.043	NIT1	1.61E+08
chr1	1.54E+08	1.54E+08	152	1.8	0.000615	0.0431	AQP10	1.54E+08
chr11	67122109	67122372	264	-0.89	0.000617	0.0431	POLD4	67119067
chr11	67122109	67122372	264	-0.89	0.000617	0.0431	POLD4	67119067
chr11	67122109	67122372	264	-0.89	0.000617	0.0431	POLD4	67119067
chr11	67122109	67122372	264	-0.89	0.000617	0.0431	POLD4	67119067
chr11	67122109	67122372	264	-0.89	0.000617	0.0431	POLD4	67119067
chr8	42360585	42360759	175	-0.73	0.000643	0.0442	SLC20A2	42356978
chr3	1.94E+08	1.94E+08	1007	-0.65	0.000673	0.0454	LINC00884	1.94E+08
chr3	40546191	40546470	280	-1.38	0.000695	0.0463	ZNF620	40542601
chr3	40546191	40546470	280	-1.38	0.000695	0.0463	ZNF620	40542529

chr3	40546191	40546470	280	-1.38	0.000695	0.0463	ZNF620	40542529
chr17	42285836	42285978	143	-1.34	0.000705	0.0467	MIR6782	42283207
chr5	39077237	39077351	115	-1.33	0.000721	0.0473	RICTOR	39072510
chr5	39077237	39077351	115	-1.33	0.000721	0.0473	RICTOR	39072510
chr5	39077237	39077351	115	-1.33	0.000721	0.0473	RICTOR	39072510
chr2	62899801	62900114	314	1.72	0.000748	0.0484	EHBP1	62895985
chr1	62546676	62547048	373	2.46	0.000752	0.0484	MIR3116-2	62542528
chr12	88972993	88973198	206	-1.03	0.000753	0.0484	KITLG	88972250
chr12	88972993	88973198	206	-1.03	0.000753	0.0484	KITLG	88972250
chr1	6551597	6551838	242	-0.96	0.000763	0.0488	PLEKHG5	6548640
chr1	6551597	6551838	242	-0.96	0.000763	0.0488	PLEKHG5	6548654
chr1	6551597	6551838	242	-0.96	0.000763	0.0488	PLEKHG5	6549761
chr20	25227817	25229043	1227	-0.39	0.000778	0.0494	PYGB	25223705
chr9	12817785	12817883	99	-2.25	0.000781	0.0496	LURAP1L-A	12812388
chr10	93373361	93373802	442	-0.94	0.000783	0.0497	HECTD2-AS	93369217
chr6	1.07E+08	1.07E+08	493	-0.8	0.000789	0.0499	PRDM1	1.07E+08

d SCC25.

promoterstop
1.24E+08
1.24E+08
67634168
27495277
27495277
1.23E+08
91416768
91413821
91418107
41088243
41088243
1.7E+08
1.7E+08
95014927
1.02E+08
27327229
27327229
27327229
27327229
15014504
23610297
54935815
1.47E+08
1.55E+08
24567926
31368454
31368454
12256317
12256359
12256359
1.4E+08
1.8E+08
1.29E+08
76196569
1.73E+08
2.45E+08
2.45E+08
2.45E+08
93456679
1.24E+08
1.24E+08
1.24E+08
65598931
8837224

8837882
71064243
2.12E+08
2.12E+08
51745463
1.89E+08
7990059
1.37E+08
1.37E+08
1.37E+08
3016945
64066744
64066744
64066744
64066744
74227449
74334308
1.1E+08
1.1E+08
81073683
81073683
81073683
1.19E+08
54488859
54488859
54488859
54488859
54488859
96191164
1.38E+08
2466684
1.25E+08
1.25E+08
24499719
24499719
24499719
42998435
19316490
1.29E+08
55690773
38383177
1.25E+08
1.25E+08
1.25E+08
1.25E+08
1.25E+08
1.25E+08

17544605
17544605
17544605
1.12E+08
1.8E+08
57238335
57238335
57238335
1.35E+08
1.04E+08
1.04E+08
3088579
1.04E+08
17700690
1.44E+08
1.44E+08
15097145
1.7E+08
1.7E+08
1.7E+08
15119562
9522059
58405442
54940282
52537883
44775336
85091714
45928937
45925345
1.51E+08
1.51E+08
1.51E+08
1.51E+08
12229059
12228999
1.6E+08
33817952
33233234
33233234
94422570
94422570
37061086
37058978
1.3E+08
1.34E+08
10445029
46724643

46724316
1.39E+08
2881744
9030596
22990781
22989090
22992012
1.1E+08
86119520
1.38E+08
1.38E+08
60693912
60693912
95416841
1.24E+08
2.1E+08
2.1E+08
2.1E+08
58499993
58499548
58500725
86119520
1.14E+08
1.85E+08
1.85E+08
1.09E+08
1.09E+08
1.09E+08
58037276
58038762
58037276
58037276
89785688
1.61E+08
1.61E+08
1.61E+08
1.61E+08
1.54E+08
67126067
67126067
67126067
67126067
67126067
42363978
1.94E+08
40549601
40549529

40549529
42290207
39079510
39079510
39079510
62902985
62549528
88979250
88979250
6555640
6555654
6556761
25230705
12819388
93376217
1.07E+08

Supplementary Table 7 - SCC1 differential binding affinity between cetuximab and PBS.

seqnames	start	end	width	Fold	p.value	FDR	genename	promoters†
chr1	2.1E+08	2.1E+08	332	-2.73	1.42E-12	1.10E-09	LAMB3	2.1E+08
chr1	2.1E+08	2.1E+08	332	-2.73	1.42E-12	1.10E-09	LAMB3	2.1E+08
chr8	74332193	74332468	276	-2.64	1.15E-11	6.71E-09	STAU2-AS1	74327308
chr11	1.23E+08	1.23E+08	250	-2.96	4.24E-11	1.94E-08	CLMP	1.23E+08
chr12	30947828	30948298	471	-1.89	1.68E-10	5.51E-08	LINC00941	30943614
chr18	3015278	3015558	281	-2.23	2.50E-10	7.66E-08	LPIN2	3009945
chr11	57192284	57192808	525	-2.58	6.48E-10	1.62E-07	SLC43A3	57192621
chr11	57192284	57192808	525	-2.58	6.48E-10	1.62E-07	SLC43A3	57192621
chr11	57192284	57192808	525	-2.58	6.48E-10	1.62E-07	SLC43A3	57192621
chr11	57192284	57192808	525	-2.58	6.48E-10	1.62E-07	SLC43A3	57192560
chr3	40546200	40546455	256	-2.29	2.42E-09	4.81E-07	ZNF620	40542601
chr3	40546200	40546455	256	-2.29	2.42E-09	4.81E-07	ZNF620	40542529
chr3	40546200	40546455	256	-2.29	2.42E-09	4.81E-07	ZNF620	40542529
chr1	37942901	37943291	391	-1.81	2.97E-09	5.74E-07	MIR6732	37940830
chr1	37942901	37943291	391	-1.81	2.97E-09	5.74E-07	LINC01137	37938044
chr13	1.02E+08	1.02E+08	645	-1.52	9.28E-09	1.56E-06	ITGBL1	1.02E+08
chr13	1.02E+08	1.02E+08	645	-1.52	9.28E-09	1.56E-06	ITGBL1	1.02E+08
chr13	1.02E+08	1.02E+08	645	-1.52	9.28E-09	1.56E-06	ITGBL1	1.02E+08
chr15	80872449	80872979	531	-1.52	1.08E-08	1.76E-06	MIR5572	80868443
chr1	33812626	33812871	246	-2.38	2.00E-08	2.88E-06	LOC10192[illegible]	33810952
chr3	1.8E+08	1.8E+08	633	-2.1	2.23E-08	3.13E-06	LOC10050[illegible]	1.8E+08
chr1	47655754	47656223	470	-1.59	3.13E-08	4.06E-06	PDZK1IP1	47653771
chr2	2.33E+08	2.33E+08	304	2.2	5.40E-08	6.11E-06	ECEL1P2	2.33E+08
chr8	1.04E+08	1.04E+08	286	-1.96	6.31E-08	6.82E-06	CTHRC1	1.04E+08
chr8	1.04E+08	1.04E+08	286	-1.96	6.31E-08	6.82E-06	CTHRC1	1.04E+08
chr3	1.38E+08	1.38E+08	264	-2.54	9.28E-08	9.32E-06	NME9	1.38E+08
chr6	86116375	86116655	281	-1.57	1.31E-07	1.22E-05	LOC10192[illegible]	86112520
chr12	1.09E+08	1.09E+08	198	-2.15	1.38E-07	1.26E-05	SART3	1.09E+08
chr13	95363460	95364092	633	-1.66	1.42E-07	1.28E-05	LOC10192[illegible]	95358740
chr13	95363460	95364092	633	-1.66	1.42E-07	1.28E-05	SOX21-AS1	95359969
chr13	95363460	95364092	633	-1.66	1.42E-07	1.28E-05	SOX21	95362797
chr2	1.99E+08	1.99E+08	175	-2.17	1.48E-07	1.32E-05	LOC10192[illegible]	1.99E+08
chr11	1.14E+08	1.14E+08	477	-1.4	1.52E-07	1.35E-05	NNMT	1.14E+08
chr16	56641171	56641542	372	-1.42	1.66E-07	1.44E-05	MT2A	56637477
chr2	20792907	20793574	668	-1.3	1.75E-07	1.50E-05	HS1BP3-IT[illegible]	20790308
chr8	1.24E+08	1.24E+08	203	-1.58	1.77E-07	1.50E-05	FAM83A	1.24E+08
chr8	1.24E+08	1.24E+08	203	-1.58	1.77E-07	1.50E-05	FAM83A	1.24E+08
chr8	1.24E+08	1.24E+08	203	-1.58	1.77E-07	1.50E-05	FAM83A	1.24E+08
chr6	2877728	2878004	277	-2.21	1.78E-07	1.50E-05	SERPINB9P	2874744
chr15	91412887	91413305	419	-1.37	1.83E-07	1.53E-05	FURIN	91409768
chr15	91412887	91413305	419	-1.37	1.83E-07	1.53E-05	FURIN	91406821
chr15	91412887	91413305	419	-1.37	1.83E-07	1.53E-05	FURIN	91411107
chr6	1.19E+08	1.19E+08	364	-1.46	2.09E-07	1.70E-05	FAM184A	1.19E+08
chr6	1.19E+08	1.19E+08	364	-1.46	2.09E-07	1.70E-05	FAM184A	1.19E+08

chr1	67628697	67628986	290	-1.92	2.12E-07	1.72E-05	IL23R	67627168
chr4	78977565	78978135	571	-1.49	3.04E-07	2.27E-05	FRAS1	78973723
chr4	78977565	78978135	571	-1.49	3.04E-07	2.27E-05	FRAS1	78973723
chr8	1.31E+08	1.31E+08	541	-1.35	3.19E-07	2.33E-05	ASAP1	1.31E+08
chr8	1.31E+08	1.31E+08	541	-1.35	3.19E-07	2.33E-05	ASAP1	1.31E+08
chr4	58290457	58290656	200	-2.13	3.41E-07	2.44E-05	LOC101928	58287037
chr20	17539186	17539591	406	-1.51	3.63E-07	2.56E-05	BFSP1	17537605
chr20	17539186	17539591	406	-1.51	3.63E-07	2.56E-05	BFSP1	17537605
chr20	17539186	17539591	406	-1.51	3.63E-07	2.56E-05	BFSP1	17537605
chr11	1.14E+08	1.14E+08	537	-2.07	3.76E-07	2.62E-05	LOC101928	1.14E+08
chr3	1.57E+08	1.57E+08	324	-1.35	3.96E-07	2.73E-05	LINC00880	1.57E+08
chr5	1.73E+08	1.73E+08	261	-2.43	4.34E-07	2.91E-05	LINC01485	1.73E+08
chr1	2.49E+08	2.49E+08	161	-1.82	7.14E-07	4.26E-05	SH3BP5L	2.49E+08
chr2	1.06E+08	1.06E+08	698	-1.11	7.34E-07	4.36E-05	FHL2	1.06E+08
chr2	1.06E+08	1.06E+08	698	-1.11	7.34E-07	4.36E-05	FHL2	1.06E+08
chr2	1.06E+08	1.06E+08	698	-1.11	7.34E-07	4.36E-05	FHL2	1.06E+08
chr2	1.06E+08	1.06E+08	698	-1.11	7.34E-07	4.36E-05	FHL2	1.06E+08
chr2	1.06E+08	1.06E+08	698	-1.11	7.34E-07	4.36E-05	FHL2	1.06E+08
chr2	1.06E+08	1.06E+08	698	-1.11	7.34E-07	4.36E-05	FHL2	1.06E+08
chr2	1.06E+08	1.06E+08	698	-1.11	7.34E-07	4.36E-05	FHL2	1.06E+08
chr2	1.06E+08	1.06E+08	698	-1.11	7.34E-07	4.36E-05	FHL2	1.06E+08
chr1	2.01E+08	2.01E+08	231	-1.93	8.48E-07	4.91E-05	C1orf106	2.01E+08
chr17	73780657	73781296	640	1.15	1.02E-06	5.61E-05	UNK	73775919
chr17	73780657	73781296	640	1.15	1.02E-06	5.61E-05	H3F3B	73774016
chr17	73780657	73781296	640	1.15	1.02E-06	5.61E-05	MIR4738	73778688
chr17	73780657	73781296	640	1.15	1.02E-06	5.61E-05	UNK	73775919
chr2	1.44E+08	1.44E+08	369	-1.69	1.09E-06	5.93E-05	KYNU	1.44E+08
chr2	1.44E+08	1.44E+08	369	-1.69	1.09E-06	5.93E-05	KYNU	1.44E+08
chr2	1.44E+08	1.44E+08	369	-1.69	1.09E-06	5.93E-05	KYNU	1.44E+08
chr3	1.32E+08	1.32E+08	355	-1.7	1.15E-06	6.19E-05	ACKR4	1.32E+08
chr16	66512775	66513313	539	-1.4	1.24E-06	6.56E-05	BEAN1-AS1	66508305
chr5	1.5E+08	1.5E+08	223	-1.91	1.40E-06	7.15E-05	TNIP1	1.5E+08
chr5	1.5E+08	1.5E+08	223	-1.91	1.40E-06	7.15E-05	TNIP1	1.5E+08
chr5	1.5E+08	1.5E+08	223	-1.91	1.40E-06	7.15E-05	TNIP1	1.5E+08
chr5	1.5E+08	1.5E+08	223	-1.91	1.40E-06	7.15E-05	TNIP1	1.5E+08
chr5	1.5E+08	1.5E+08	223	-1.91	1.40E-06	7.15E-05	TNIP1	1.5E+08
chr5	1.5E+08	1.5E+08	223	-1.91	1.40E-06	7.15E-05	TNIP1	1.5E+08
chr5	1.5E+08	1.5E+08	223	-1.91	1.40E-06	7.15E-05	TNIP1	1.5E+08
chr5	1.5E+08	1.5E+08	223	-1.91	1.40E-06	7.15E-05	TNIP1	1.5E+08
chr3	1.08E+08	1.08E+08	115	-2.53	1.45E-06	7.37E-05	LINC01215	1.08E+08
chr5	1.34E+08	1.34E+08	335	-1.55	1.60E-06	7.91E-05	LOC101927	1.34E+08
chr14	94425758	94426240	483	-1.24	1.68E-06	8.22E-05	ASB2	94421767
chr5	35047009	35047172	164	-3.25	2.32E-06	0.000106	AGXT2	35046240
chr5	35047009	35047172	164	-3.25	2.32E-06	0.000106	AGXT2	35046240
chr10	1.06E+08	1.06E+08	411	-1.35	2.89E-06	0.000127	ITPRIP	1.06E+08
chr18	21451490	21451595	106	-2.61	2.95E-06	0.000129	LAMA3	21447983
chr18	21451490	21451595	106	-2.61	2.95E-06	0.000129	LAMA3	21447983

chrX	56765268	56765456	189	5.14	3.41E-06	0.000145	UQCRBP1	56762017
chr14	71252387	71252594	208	-2.63	3.54E-06	0.000148	MAP3K9	71248169
chr14	71252387	71252594	208	-2.63	3.54E-06	0.000148	MAP3K9	71248169
chr1	12679050	12679391	342	-1.33	3.62E-06	0.000151	DHRS3	12675737
chr5	1.7E+08	1.7E+08	152	-3.15	3.65E-06	0.000152	LINC01366	1.7E+08
chr5	1.7E+08	1.7E+08	152	-3.15	3.65E-06	0.000152	LINC01366	1.7E+08
chr5	1.7E+08	1.7E+08	152	-3.15	3.65E-06	0.000152	LINC01366	1.7E+08
chr4	86697858	86698067	210	-2.55	3.90E-06	0.00016	ARHGAP24	86694850
chr6	1.34E+08	1.34E+08	299	-1.57	3.92E-06	0.00016	SLC2A12	1.34E+08
chr20	56887974	56888183	210	-1.96	3.93E-06	0.00016	PPP4R1L	56882495
chr6	86114110	86114732	623	-1.22	3.98E-06	0.000161	LOC101928	86112520
chr13	31287375	31287532	158	-2.84	4.19E-06	0.000168	ALOX5AP	31282614
chr12	1.05E+08	1.05E+08	405	-1.48	4.59E-06	0.000179	MIR3922	1.05E+08
chr8	62605113	62605346	234	-1.52	5.59E-06	0.000211	ASPH	62600408
chr8	62605113	62605346	234	-1.52	5.59E-06	0.000211	ASPH	62600408
chr8	62605113	62605346	234	-1.52	5.59E-06	0.000211	ASPH	62600408
chr8	62605113	62605346	234	-1.52	5.59E-06	0.000211	ASPH	62600408
chr8	62605113	62605346	234	-1.52	5.59E-06	0.000211	ASPH	62600408
chr8	62605113	62605346	234	-1.52	5.59E-06	0.000211	ASPH	62600408
chr8	62605113	62605346	234	-1.52	5.59E-06	0.000211	ASPH	62600408
chr8	94924612	94924936	325	-1.35	5.79E-06	0.000217	PDP1	94924161
chr8	94924612	94924936	325	-1.35	5.79E-06	0.000217	PDP1	94924174
chr8	94924612	94924936	325	-1.35	5.79E-06	0.000217	PDP1	94924082
chr8	94924612	94924936	325	-1.35	5.79E-06	0.000217	PDP1	94924278
chr18	12255679	12255947	269	-1.75	6.73E-06	0.000242	CIDEA	12249317
chr18	12255679	12255947	269	-1.75	6.73E-06	0.000242	CIDEA	12249359
chr18	12255679	12255947	269	-1.75	6.73E-06	0.000242	CIDEA	12249359
chr12	1.05E+08	1.05E+08	111	-2.21	6.95E-06	0.000248	CHST11	1.05E+08
chr12	1.05E+08	1.05E+08	111	-2.21	6.95E-06	0.000248	CHST11	1.05E+08
chr17	54993173	54993501	329	-1.21	6.95E-06	0.000248	TRIM25	54989409
chr4	1.89E+08	1.89E+08	272	-1.63	8.07E-06	0.00028	TRIML2	1.89E+08
chr4	1.89E+08	1.89E+08	272	-1.63	8.07E-06	0.00028	TRIML2	1.89E+08
chr1	2.24E+08	2.24E+08	880	-1.03	9.38E-06	0.000315	CAPN2	2.24E+08
chr11	10476526	10476778	253	-1.53	9.46E-06	0.000317	AMPD3	10471665
chr11	10476526	10476778	253	-1.53	9.46E-06	0.000317	AMPD3	10472480
chr6	86113315	86113609	295	-1.34	9.86E-06	0.000324	LOC101928	86112520
chr18	3595557	3596065	509	-1.3	1.00E-05	0.000329	DLGAP1-AS	3589111
chr2	1.07E+08	1.07E+08	289	-1.49	1.07E-05	0.000347	UXS1	1.07E+08
chr7	1.55E+08	1.55E+08	164	-2.87	1.12E-05	0.000357	HTR5A	1.55E+08
chr4	79567091	79567816	726	-1.32	1.16E-05	0.000365	LINC01094	79562147
chr4	79567091	79567816	726	-1.32	1.16E-05	0.000365	LINC01094	79562147
chr4	79567091	79567816	726	-1.32	1.16E-05	0.000365	LINC01094	79562147
chr4	79567091	79567816	726	-1.32	1.16E-05	0.000365	LINC01094	79562147
chr4	79567091	79567816	726	-1.32	1.16E-05	0.000365	LINC01094	79562147
chr4	79567091	79567816	726	-1.32	1.16E-05	0.000365	LINC01094	79562147
chrX	1.35E+08	1.35E+08	137	-3.93	1.19E-05	0.000372	MAP7D3	1.35E+08
chr11	17034486	17034768	283	-2.05	1.19E-05	0.000373	PLEKHA7	17033963

chr5	94417269	94417531	263	-1.71	1.26E-05	0.00039	MCTP1	94415570
chr5	94417269	94417531	263	-1.71	1.26E-05	0.00039	MCTP1	94415570
chr12	94964155	94964270	116	-3.48	1.30E-05	0.000399	MIR7844	94963128
chr1	6551613	6551819	207	-1.47	1.56E-05	0.000461	PLEKHG5	6548640
chr1	6551613	6551819	207	-1.47	1.56E-05	0.000461	PLEKHG5	6548654
chr1	6551613	6551819	207	-1.47	1.56E-05	0.000461	PLEKHG5	6549761
chr9	19404291	19404849	559	-1.12	1.57E-05	0.000461	ACER2	19403924
chr1	12221920	12222143	224	-1.63	1.59E-05	0.000467	TNFRSF1B	12222059
chr1	12221920	12222143	224	-1.63	1.59E-05	0.000467	MIR7846	12221999
chr11	60693352	60693672	321	-1.96	1.60E-05	0.000469	TMEM132A	60686912
chr11	60693352	60693672	321	-1.96	1.60E-05	0.000469	TMEM132A	60686912
chr5	10310606	10310843	238	-1.28	1.62E-05	0.000472	CMBL	10306168
chr1	1.81E+08	1.81E+08	242	-1.54	1.62E-05	0.000472	MR1	1.81E+08
chr1	1.81E+08	1.81E+08	242	-1.54	1.62E-05	0.000472	MR1	1.81E+08
chr1	1.81E+08	1.81E+08	242	-1.54	1.62E-05	0.000472	MR1	1.81E+08
chr1	1.81E+08	1.81E+08	242	-1.54	1.62E-05	0.000472	MR1	1.81E+08
chr1	1.81E+08	1.81E+08	242	-1.54	1.62E-05	0.000472	MR1	1.81E+08
chr2	1.8E+08	1.8E+08	213	-1.81	1.79E-05	0.000513	SESTD1	1.8E+08
chr3	1.49E+08	1.49E+08	669	-1.05	1.85E-05	0.000523	CP	1.49E+08
chr3	1.49E+08	1.49E+08	669	-1.05	1.85E-05	0.000523	CP	1.49E+08
chr11	9023429	9023609	181	-2.14	1.85E-05	0.000524	NRIP3	9023596
chr5	76110117	76110291	175	-1.67	2.06E-05	0.000567	F2RL1	76109832
chr4	1.49E+08	1.49E+08	217	-1.64	2.18E-05	0.000592	MIR4799	1.49E+08
chr13	1.04E+08	1.04E+08	229	-1.81	2.26E-05	0.000609	SLC10A2	1.04E+08
chr7	1.35E+08	1.35E+08	388	-1.16	2.31E-05	0.000619	WDR91	1.35E+08
chr8	1.34E+08	1.34E+08	181	-2.29	2.35E-05	0.000626	TMEM71	1.34E+08
chr8	1.34E+08	1.34E+08	181	-2.29	2.35E-05	0.000626	TMEM71	1.34E+08
chr1	86889617	86889872	256	-1.37	2.55E-05	0.000669	CLCA2	86884768
chr12	41300859	41301355	497	-1.2	2.61E-05	0.000681	CNTN1	41297158
chr2	23603673	23604237	565	-1.14	2.74E-05	0.000707	KLHL29	23603297
chr3	1.43E+08	1.43E+08	346	-1.04	2.88E-05	0.000733	PAQR9-AS1	1.43E+08
chr10	1.35E+08	1.35E+08	235	-1.43	3.32E-05	0.000814	ADAM8	1.35E+08
chr10	1.35E+08	1.35E+08	235	-1.43	3.32E-05	0.000814	ADAM8	1.35E+08
chr10	1.35E+08	1.35E+08	235	-1.43	3.32E-05	0.000814	ADAM8	1.35E+08
chr1	1.81E+08	1.81E+08	229	-1.55	3.32E-05	0.000814	STX6	1.81E+08
chr1	1.81E+08	1.81E+08	229	-1.55	3.32E-05	0.000814	STX6	1.81E+08
chr12	41084292	41084475	184	-2.16	3.39E-05	0.000827	CNTN1	41081243
chr12	41084292	41084475	184	-2.16	3.39E-05	0.000827	CNTN1	41081243
chr8	62201249	62201368	120	-2.39	3.42E-05	0.000831	CLVS1	62195524
chr8	86157602	86158394	793	-0.95	3.55E-05	0.000854	CA13	86152715
chr1	2.1E+08	2.1E+08	155	-1.49	3.58E-05	0.000859	LAMB3	2.1E+08
chr1	2.1E+08	2.1E+08	155	-1.49	3.58E-05	0.000859	LAMB3	2.1E+08
chr1	2.1E+08	2.1E+08	155	-1.49	3.58E-05	0.000859	LAMB3	2.1E+08
chr8	1.24E+08	1.24E+08	279	-1.67	3.86E-05	0.000908	MIR4663	1.24E+08
chr10	1.3E+08	1.3E+08	412	-1.38	4.04E-05	0.000943	PTPRE	1.3E+08
chr17	73840533	73841513	981	0.99	4.08E-05	0.000951	UNC13D	73838798
chr2	1.2E+08	1.2E+08	438	-1.26	4.33E-05	0.00099	STEAP3	1.2E+08

chr2	1.2E+08	1.2E+08	438	-1.26	4.33E-05	0.00099	STEAP3	1.2E+08
chr2	1.2E+08	1.2E+08	438	-1.26	4.33E-05	0.00099	STEAP3	1.2E+08
chr2	1.2E+08	1.2E+08	438	-1.26	4.33E-05	0.00099	STEAP3	1.2E+08
chr10	1.06E+08	1.06E+08	239	-1.71	4.34E-05	0.000991	CFAP58	1.06E+08
chr13	47128571	47128930	360	-1.2	4.37E-05	0.000996	LRCH1	47122295
chr13	47128571	47128930	360	-1.2	4.37E-05	0.000996	LRCH1	47122295
chr13	47128571	47128930	360	-1.2	4.37E-05	0.000996	LRCH1	47122295
chr5	1.4E+08	1.4E+08	272	-1.64	4.58E-05	0.00103	CD14	1.4E+08
chr5	1.4E+08	1.4E+08	272	-1.64	4.58E-05	0.00103	CD14	1.4E+08
chr5	1.4E+08	1.4E+08	272	-1.64	4.58E-05	0.00103	CD14	1.4E+08
chr5	1.4E+08	1.4E+08	272	-1.64	4.58E-05	0.00103	CD14	1.4E+08
chr5	59063916	59064666	751	-1.07	4.66E-05	0.00104	PDE4D	59062438
chr3	1.87E+08	1.87E+08	313	-1.61	4.87E-05	0.00107	RPL39L	1.87E+08
chr3	1.33E+08	1.33E+08	133	-1.45	5.34E-05	0.00115	CDV3	1.33E+08
chr3	1.33E+08	1.33E+08	133	-1.45	5.34E-05	0.00115	CDV3	1.33E+08
chr3	1.33E+08	1.33E+08	133	-1.45	5.34E-05	0.00115	CDV3	1.33E+08
chr3	1.33E+08	1.33E+08	133	-1.45	5.34E-05	0.00115	CDV3	1.33E+08
chr3	1.33E+08	1.33E+08	133	-1.45	5.34E-05	0.00115	CDV3	1.33E+08
chr3	1.33E+08	1.33E+08	133	-1.45	5.34E-05	0.00115	CDV3	1.33E+08
chr3	1.33E+08	1.33E+08	133	-1.45	5.34E-05	0.00115	CDV3	1.33E+08
chr5	42993329	42993522	194	-1.36	5.50E-05	0.00118	FLJ32255	42991435
chr11	1.25E+08	1.25E+08	198	-1.96	5.52E-05	0.00119	ROBO4	1.25E+08
chr11	1.25E+08	1.25E+08	198	-1.96	5.52E-05	0.00119	ROBO4	1.25E+08
chr1	1.66E+08	1.66E+08	152	-1.83	5.53E-05	0.00119	TMCO1	1.66E+08
chr1	1.66E+08	1.66E+08	152	-1.83	5.53E-05	0.00119	TMCO1	1.66E+08
chr1	1.66E+08	1.66E+08	152	-1.83	5.53E-05	0.00119	TMCO1	1.66E+08
chr1	1.66E+08	1.66E+08	152	-1.83	5.53E-05	0.00119	TMCO1	1.66E+08
chr19	50145135	50145469	335	1.1	5.87E-05	0.00124	SCAF1	50140381
chr19	50145135	50145469	335	1.1	5.87E-05	0.00124	RRAS	50141400
chr2	1.03E+08	1.03E+08	184	-1.55	5.91E-05	0.00125	IL1R1	1.03E+08
chr2	19562772	19562989	218	-1.36	5.98E-05	0.00126	OSR1	19556372
chr15	89166429	89166667	239	-1.27	6.44E-05	0.00133	AEN	89159526
chr1	2.45E+08	2.45E+08	293	-1.18	6.48E-05	0.00133	C1orf100	2.45E+08
chr1	2.45E+08	2.45E+08	293	-1.18	6.48E-05	0.00133	C1orf100	2.45E+08
chr1	2.45E+08	2.45E+08	293	-1.18	6.48E-05	0.00133	C1orf100	2.45E+08
chr2	2.42E+08	2.42E+08	204	-2.25	6.61E-05	0.00135	BOK	2.42E+08
chr16	56279843	56280350	508	-0.96	6.78E-05	0.00137	MIR3935	56274431
chr3	1.39E+08	1.39E+08	535	-1.26	7.08E-05	0.00142	FOXL2NB	1.39E+08
chr3	1.39E+08	1.39E+08	535	-1.26	7.08E-05	0.00142	FOXL2	1.39E+08
chr3	1.39E+08	1.39E+08	535	-1.26	7.08E-05	0.00142	LINC01391	1.39E+08
chr6	1.64E+08	1.64E+08	359	-1.43	7.33E-05	0.00147	DKFZp451E	1.64E+08
chr11	19266236	19266535	300	-1.37	7.69E-05	0.00152	E2F8	19261167
chr11	19266236	19266535	300	-1.37	7.69E-05	0.00152	E2F8	19261202
chr11	19266236	19266535	300	-1.37	7.69E-05	0.00152	E2F8	19260507
chr8	25203030	25203208	179	-2.28	7.99E-05	0.00156	MIR6876	25197917
chr12	15113796	15114817	1022	-0.91	8.02E-05	0.00156	ARHGDIB	15112562
chr2	10438167	10438313	147	-2.08	8.21E-05	0.00159	HPCAL1	10438029

chr17	48367395	48367748	354	-1.6	8.58E-05	0.00164	TMEM92-A	48363216
chr14	45723099	45723351	253	-1.64	8.59E-05	0.00164	MIS18BP1	45720605
chr2	37417026	37417257	232	-1.56	9.04E-05	0.00171	SULT6B1	37413690
chrX	1.06E+08	1.06E+08	167	-3	9.23E-05	0.00174	CLDN2	1.06E+08
chrX	1.06E+08	1.06E+08	167	-3	9.23E-05	0.00174	CLDN2	1.06E+08
chr10	77188351	77188540	190	-1.45	9.40E-05	0.00176	C10orf11	77186403
chr2	1.5E+08	1.5E+08	230	-1.33	9.42E-05	0.00177	LYPD6B	1.5E+08
chr2	1.5E+08	1.5E+08	230	-1.33	9.42E-05	0.00177	LYPD6B	1.5E+08
chr2	1.5E+08	1.5E+08	230	-1.33	9.42E-05	0.00177	LYPD6B	1.5E+08
chr2	1.5E+08	1.5E+08	230	-1.33	9.42E-05	0.00177	LYPD6B	1.5E+08
chr15	41408485	41409025	541	1.15	9.42E-05	0.00177	INO80	41406444
chr15	41408485	41409025	541	1.15	9.42E-05	0.00177	INO80	41406444
chr11	87907899	87908063	165	-1.49	9.68E-05	0.0018	RAB38	87906635
chr11	87907899	87908063	165	-1.49	9.68E-05	0.0018	MIR3166	87904669
chr12	1.19E+08	1.19E+08	310	-1.18	9.89E-05	0.00183	VSIG10	1.19E+08
chr11	1.24E+08	1.24E+08	131	-1.71	0.000101	0.00187	OR10S1	1.24E+08
chr20	49254861	49255055	195	-3.17	0.000105	0.00193	FAM65C	49251455
chr5	43193560	43193906	347	-1.45	0.000109	0.00198	NIM1K	43187953
chr5	43193560	43193906	347	-1.45	0.000109	0.00198	NIM1K	43187169
chr12	88973020	88973198	179	-1.52	0.00011	0.00199	KITLG	88972250
chr12	88973020	88973198	179	-1.52	0.00011	0.00199	KITLG	88972250
chr2	31637610	31637893	284	-1.17	0.000111	0.00201	XDH	31635611
chr1	2.11E+08	2.11E+08	429	-1.01	0.000112	0.00201	KCNH1	2.11E+08
chr1	2.11E+08	2.11E+08	429	-1.01	0.000112	0.00201	KCNH1	2.11E+08
chr8	1.03E+08	1.03E+08	116	-2.64	0.000114	0.00204	NCALD	1.03E+08
chr8	1.03E+08	1.03E+08	116	-2.64	0.000114	0.00204	NCALD	1.03E+08
chr8	1.03E+08	1.03E+08	116	-2.64	0.000114	0.00204	NCALD	1.03E+08
chr8	1.03E+08	1.03E+08	116	-2.64	0.000114	0.00204	NCALD	1.03E+08
chr8	1.03E+08	1.03E+08	116	-2.64	0.000114	0.00204	NCALD	1.03E+08
chr8	1.03E+08	1.03E+08	116	-2.64	0.000114	0.00204	NCALD	1.03E+08
chr8	1.03E+08	1.03E+08	116	-2.64	0.000114	0.00204	NCALD	1.03E+08
chr6	10431687	10431912	226	-1.19	0.000118	0.00208	MIR5689H(	10429561
chr19	48018300	48018849	550	0.99	0.00012	0.00211	NAPA	48016515
chr19	48018300	48018849	550	0.99	0.00012	0.00211	NAPA	48016515
chr19	48018300	48018849	550	0.99	0.00012	0.00211	NAPA	48016515
chr19	11457040	11457295	256	1.33	0.000121	0.00213	TMEM205	11454981
chr19	11457040	11457295	256	1.33	0.000121	0.00213	TMEM205	11454981
chr19	11457040	11457295	256	1.33	0.000121	0.00213	TMEM205	11454981
chr19	11457040	11457295	256	1.33	0.000121	0.00213	CCDC159	11452180
chr6	2876768	2877095	328	-1.2	0.000122	0.00214	SERPINB9P	2874744
chr18	61925489	61925765	277	-1.36	0.000124	0.00216	LINC01538	61925290
chr2	88839982	88840205	224	-2.04	0.000129	0.00223	LOC101928	88833237
chr3	5019305	5019456	152	-1.52	0.000135	0.00231	BHLHE40	5016096
chr2	18060384	18060835	452	-1.09	0.000136	0.00233	KCNS3	18054923
chr2	18060384	18060835	452	-1.09	0.000136	0.00233	KCNS3	18054113
chr3	1.55E+08	1.55E+08	586	-0.97	0.00014	0.00237	MME	1.55E+08
chr3	1.55E+08	1.55E+08	586	-0.97	0.00014	0.00237	MME	1.55E+08

chr3	1.55E+08	1.55E+08	586	-0.97	0.00014	0.00237	MME	1.55E+08
chr3	1.55E+08	1.55E+08	586	-0.97	0.00014	0.00237	MME	1.55E+08
chr17	7210398	7210987	590	1.05	0.000142	0.0024	EIF5A	7206693
chr17	7210398	7210987	590	1.05	0.000142	0.0024	EIF5A	7206287
chr17	7210398	7210987	590	1.05	0.000142	0.0024	EIF5A	7205317
chr17	7210398	7210987	590	1.05	0.000142	0.0024	EIF5A	7205855
chr5	52283634	52284026	393	-1.15	0.000143	0.0024	ITGA2	52280155
chr5	52283634	52284026	393	-1.15	0.000143	0.0024	ITGA2	52280155
chr5	52283634	52284026	393	-1.15	0.000143	0.0024	ITGA2	52280155
chr5	52283634	52284026	393	-1.15	0.000143	0.0024	ITGA2	52280155
chr5	52283634	52284026	393	-1.15	0.000143	0.0024	ITGA2	52280155
chr5	52283634	52284026	393	-1.15	0.000143	0.0024	ITGA2	52280155
chr10	1.28E+08	1.28E+08	294	-1.63	0.000144	0.00241	DHX32	1.28E+08
chr11	85780868	85781080	213	-1.33	0.000147	0.00246	PICALM	85778139
chr11	85780868	85781080	213	-1.33	0.000147	0.00246	PICALM	85778139
chr11	85780868	85781080	213	-1.33	0.000147	0.00246	PICALM	85778923
chr11	85780868	85781080	213	-1.33	0.000147	0.00246	PICALM	85778139
chr7	24796728	24797606	879	-0.93	0.000149	0.00248	DFNA5	24795639
chr7	24796728	24797606	879	-0.93	0.000149	0.00248	DFNA5	24795639
chr7	24796728	24797606	879	-0.93	0.000149	0.00248	DFNA5	24795083
chr9	1.03E+08	1.03E+08	296	-1.27	0.000155	0.00256	MSANTD3	1.03E+08
chr9	1.03E+08	1.03E+08	296	-1.27	0.000155	0.00256	MSANTD3	1.03E+08
chr4	15002940	15003071	132	-1.3	0.000157	0.00257	CPEB2-AS1	15001669
chr4	15002940	15003071	132	-1.3	0.000157	0.00257	CPEB2	14999297
chr4	15002940	15003071	132	-1.3	0.000157	0.00257	CPEB2	14999297
chr4	15002940	15003071	132	-1.3	0.000157	0.00257	CPEB2	14999297
chr4	15002940	15003071	132	-1.3	0.000157	0.00257	CPEB2	14999297
chr4	15002940	15003071	132	-1.3	0.000157	0.00257	CPEB2	14999297
chr4	15002940	15003071	132	-1.3	0.000157	0.00257	CPEB2	14999297
chr2	2.28E+08	2.28E+08	196	-1.86	0.000163	0.00266	MIR5702	2.28E+08
chr14	78262163	78262408	246	-1.55	0.000163	0.00266	ADCK1	78261425
chr14	78262163	78262408	246	-1.55	0.000163	0.00266	ADCK1	78261425
chr16	90038875	90039223	349	1.31	0.000165	0.00269	AFG3L1P	90033987
chr16	90038875	90039223	349	1.31	0.000165	0.00269	AFG3L1P	90033987
chr16	90038875	90039223	349	1.31	0.000165	0.00269	AFG3L1P	90033987
chr16	90038875	90039223	349	1.31	0.000165	0.00269	CENPBD1	90037240
chr5	1.16E+08	1.16E+08	278	-1.97	0.000168	0.00272	LOC102467	1.16E+08
chr11	1.25E+08	1.25E+08	222	-1.27	0.000168	0.00272	ROBO4	1.25E+08
chr11	1.25E+08	1.25E+08	222	-1.27	0.000168	0.00272	ROBO4	1.25E+08
chr11	1.25E+08	1.25E+08	975	-0.78	0.000171	0.00275	CHEK1	1.25E+08
chr11	1.25E+08	1.25E+08	975	-0.78	0.000171	0.00275	CHEK1	1.25E+08
chr11	1.25E+08	1.25E+08	975	-0.78	0.000171	0.00275	CHEK1	1.25E+08
chr11	1.25E+08	1.25E+08	975	-0.78	0.000171	0.00275	CHEK1	1.25E+08
chr11	1.25E+08	1.25E+08	975	-0.78	0.000171	0.00275	CHEK1	1.25E+08
chr11	1.25E+08	1.25E+08	975	-0.78	0.000171	0.00275	CHEK1	1.25E+08
chr16	89724064	89724517	454	1.07	0.000171	0.00275	CHMP1A	89722193
chr16	89724064	89724517	454	1.07	0.000171	0.00275	CHMP1A	89722193

chr16	89724064	89724517	454	1.07	0.000171	0.00275	CHMP1A	89722193
chr16	89724064	89724517	454	1.07	0.000171	0.00275	SPATA33	89719151
chr16	89724064	89724517	454	1.07	0.000171	0.00275	SPATA33	89719454
chr16	89724064	89724517	454	1.07	0.000171	0.00275	SPATA33	89719211
chr16	89724064	89724517	454	1.07	0.000171	0.00275	SPATA33	89719211
chr16	89724064	89724517	454	1.07	0.000171	0.00275	SPATA33	89719454
chr16	89724064	89724517	454	1.07	0.000171	0.00275	SPATA33	89719211
chr16	89724064	89724517	454	1.07	0.000171	0.00275	SPATA33	89719211
chr13	1.07E+08	1.07E+08	329	-1.29	0.000173	0.00277	LINC00460	1.07E+08
chr14	99738505	99738653	149	-2.11	0.000173	0.00277	BCL11B	99736050
chr14	99738505	99738653	149	-2.11	0.000173	0.00277	BCL11B	99736050
chr14	99738505	99738653	149	-2.11	0.000173	0.00277	BCL11B	99736050
chr14	99738505	99738653	149	-2.11	0.000173	0.00277	BCL11B	99736050
chr11	1.2E+08	1.2E+08	147	-2.28	0.000174	0.00278	GRIK4	1.2E+08
chr11	1.2E+08	1.2E+08	147	-2.28	0.000174	0.00278	GRIK4	1.2E+08
chr1	1.6E+08	1.6E+08	343	-1.03	0.000177	0.00282	DUSP23	1.6E+08
chr11	695115	695780	666	0.9	0.000185	0.00293	DEAF1	693754
chr11	695115	695780	666	0.9	0.000185	0.00293	DEAF1	693754
chr11	695115	695780	666	0.9	0.000185	0.00293	TMEM80	690590
chr11	695115	695780	666	0.9	0.000185	0.00293	TMEM80	690590
chr11	695115	695780	666	0.9	0.000185	0.00293	TMEM80	690590
chr11	695115	695780	666	0.9	0.000185	0.00293	TMEM80	690590
chr4	68751536	68752111	576	-1.31	0.000189	0.00297	TMPRSS11I	68747716
chr7	92748616	92749126	511	-1.23	0.00019	0.00298	SAMD9	92745336
chr7	92748616	92749126	511	-1.23	0.00019	0.00298	SAMD9	92745336
chr8	1.42E+08	1.42E+08	200	-1.17	0.000194	0.00303	SLC45A4	1.42E+08
chr8	1.42E+08	1.42E+08	200	-1.17	0.000194	0.00303	SLC45A4	1.42E+08
chr3	1.87E+08	1.87E+08	255	-1.53	0.000194	0.00303	RTP4	1.87E+08
chr6	49518236	49518497	262	-1.1	0.000194	0.00303	C6orf141	49513112
chr8	1.24E+08	1.24E+08	295	-1.28	0.000194	0.00303	LINC01151	1.24E+08
chr22	51021234	51021586	353	1.24	0.000202	0.00311	CPT1B	51014894
chr22	51021234	51021586	353	1.24	0.000202	0.00311	CPT1B	51015096
chr22	51021234	51021586	353	1.24	0.000202	0.00311	CHKB	51019428
chr22	51021234	51021586	353	1.24	0.000202	0.00311	CHKB-AS1	51016454
chr22	51021234	51021586	353	1.24	0.000202	0.00311	CPT1B	51014506
chr22	51021234	51021586	353	1.24	0.000202	0.00311	CPT1B	51015096
chr22	51021234	51021586	353	1.24	0.000202	0.00311	CPT1B	51015096
chr22	51021234	51021586	353	1.24	0.000202	0.00311	CPT1B	51014894
chr22	51021234	51021586	353	1.24	0.000202	0.00311	CHKB-CPT1	51019428
chr22	51021234	51021586	353	1.24	0.000202	0.00311	CHKB-AS1	51016454
chr11	3695506	3695700	195	-1.94	0.000202	0.00312	CHRNA10	3690614
chr11	3695506	3695700	195	-1.94	0.000202	0.00312	CHRNA10	3690614
chr11	3695506	3695700	195	-1.94	0.000202	0.00312	CHRNA10	3690614
chr6	16239653	16240107	455	-1.22	0.000204	0.00314	GMPR	16233810
chr9	1.14E+08	1.14E+08	207	-1.36	0.000206	0.00316	LPAR1	1.14E+08
chr9	1.14E+08	1.14E+08	207	-1.36	0.000206	0.00316	LPAR1	1.14E+08
chr1	59616715	59617079	365	-1.08	0.00021	0.00319	HSD52	59610479

chr12	32908007	32908387	381	-1.05	0.000213	0.00323	YARS2	32906887
chr3	4533980	4534094	115	-1.48	0.000231	0.00342	ITPR1	4530031
chr3	4533980	4534094	115	-1.48	0.000231	0.00342	ITPR1	4530031
chr3	4533980	4534094	115	-1.48	0.000231	0.00342	ITPR1	4530031
chr3	4533980	4534094	115	-1.48	0.000231	0.00342	ITPR1-AS1	4532847
chr1	1.6E+08	1.6E+08	233	1.93	0.000232	0.00343	LINC01133	1.6E+08
chr1	85744651	85744808	158	-1.12	0.000235	0.00347	BCL10	85740587
chr17	79935017	79935553	537	1.38	0.000238	0.0035	ASPSCR1	79930425
chr17	79935017	79935553	537	1.38	0.000238	0.0035	ASPSCR1	79930425
chr17	79935017	79935553	537	1.38	0.000238	0.0035	ASPSCR1	79930425
chr3	1.34E+08	1.34E+08	170	-2.04	0.000239	0.00351	KY	1.34E+08
chr11	560837	561009	173	1.47	0.000242	0.00353	RASSF7	556449
chr11	560837	561009	173	1.47	0.000242	0.00353	RASSF7	555970
chr11	560837	561009	173	1.47	0.000242	0.00353	RASSF7	556449
chr11	560837	561009	173	1.47	0.000242	0.00353	LMNTD2	558779
chr5	1.48E+08	1.48E+08	278	-1.05	0.000247	0.0036	SH3TC2	1.48E+08
chr5	1.48E+08	1.48E+08	278	-1.05	0.000247	0.0036	LOC255187	1.48E+08
chr5	39272308	39272817	510	-1.17	0.000255	0.00368	FYB	39268759
chr1	78956614	78956837	224	-1.73	0.000256	0.00369	PTGFR	78951727
chr1	78956614	78956837	224	-1.73	0.000256	0.00369	PTGFR	78951727
chr7	1.37E+08	1.37E+08	398	-1.14	0.000256	0.0037	PTN	1.37E+08
chr22	30685597	30685847	251	0.93	0.000265	0.00379	GATSL3	30683616
chr5	1.51E+08	1.51E+08	300	-1.03	0.000267	0.00381	FAT2	1.51E+08
chr10	91062498	91062741	244	-1.68	0.000269	0.00384	IFIT2	91056705
chr7	33016901	33017013	113	-2.83	0.00027	0.00385	FKBP9	33014085
chr6	47012244	47012437	194	-1.5	0.000274	0.00388	ADGRF1	47008099
chr6	47012244	47012437	194	-1.5	0.000274	0.00388	ADGRF1	47008099
chr17	41919720	41920230	511	1.69	0.000279	0.00393	CD300LG	41919515
chr17	41919720	41920230	511	1.69	0.000279	0.00393	CD300LG	41919515
chr17	41919720	41920230	511	1.69	0.000279	0.00393	CD300LG	41919515
chr17	41919720	41920230	511	1.69	0.000279	0.00393	CD300LG	41919515
chr2	68957980	68958178	199	-2.28	0.000279	0.00394	ARHGAP25	68956912
chr2	68957980	68958178	199	-2.28	0.000279	0.00394	ARHGAP25	68956912
chr9	1.4E+08	1.4E+08	341	1.09	0.000282	0.00397	C9orf142	1.4E+08
chr3	1.29E+08	1.29E+08	246	-1.47	0.000285	0.004	H1FOO	1.29E+08
chr7	38726464	38726753	290	-1.67	0.000295	0.00409	FAM183BP	38724689
chr8	1.42E+08	1.42E+08	511	-0.86	0.000295	0.0041	AGO2	1.42E+08
chr8	1.42E+08	1.42E+08	511	-0.86	0.000295	0.0041	AGO2	1.42E+08
chr3	42531753	42532092	340	-1.04	0.000297	0.00411	VIPR1	42525790
chr7	1.45E+08	1.45E+08	540	-0.77	0.000298	0.00412	TPK1	1.45E+08
chr7	1.45E+08	1.45E+08	540	-0.77	0.000298	0.00412	TPK1	1.45E+08
chr1	1.55E+08	1.55E+08	367	1.05	0.000312	0.00427	ZBTB7B	1.55E+08
chr1	1.55E+08	1.55E+08	367	1.05	0.000312	0.00427	ZBTB7B	1.55E+08
chr1	1.55E+08	1.55E+08	367	1.05	0.000312	0.00427	ZBTB7B	1.55E+08
chr1	1.55E+08	1.55E+08	367	1.05	0.000312	0.00427	ZBTB7B	1.55E+08
chr1	1.55E+08	1.55E+08	367	1.05	0.000312	0.00427	ZBTB7B	1.55E+08
chr12	1.1E+08	1.1E+08	99	-2.37	0.000315	0.0043	FAM222A-/	1.1E+08

chr12	1.1E+08	1.1E+08	99	-2.37	0.000315	0.0043	FAM222A-/	1.1E+08
chr19	18632791	18633430	640	0.92	0.00032	0.00436	ELL	18630937
chr11	8835878	8836139	262	-1.24	0.000323	0.00438	ST5	8830224
chr11	8835878	8836139	262	-1.24	0.000323	0.00438	ST5	8830882
chr10	49462094	49462380	287	-1.4	0.000323	0.00438	FRMPD2	49457800
chr13	77464269	77464533	265	-0.86	0.000324	0.00439	KCTD12	77458540
chr19	49457982	49458302	321	1.42	0.000326	0.00441	BAX	49453116
chr19	49457982	49458302	321	1.42	0.000326	0.00441	BAX	49453116
chr19	49457982	49458302	321	1.42	0.000326	0.00441	BAX	49453116
chr19	49457982	49458302	321	1.42	0.000326	0.00441	BAX	49453116
chr19	49457982	49458302	321	1.42	0.000326	0.00441	BAX	49453116
chr19	49457982	49458302	321	1.42	0.000326	0.00441	BAX	49453116
chr19	49457982	49458302	321	1.42	0.000326	0.00441	BAX	49453116
chr19	49457982	49458302	321	1.42	0.000326	0.00441	BAX	49453116
chr19	49457982	49458302	321	1.42	0.000326	0.00441	BAX	49453116
chr5	1.35E+08	1.35E+08	178	-1.6	0.000355	0.0047	TIFAB	1.35E+08
chr19	50179961	50180491	531	0.9	0.000356	0.00471	PRMT1	50175408
chr19	50179961	50180491	531	0.9	0.000356	0.00471	PRMT1	50175408
chr19	50179961	50180491	531	0.9	0.000356	0.00471	PRMT1	50175408
chr19	50179961	50180491	531	0.9	0.000356	0.00471	MIR5088	50180373
chr19	50179961	50180491	531	0.9	0.000356	0.00471	PRMT1	50175408
chr8	1.07E+08	1.07E+08	886	-0.81	0.000364	0.00479	ZFPM2-AS1	1.07E+08
chr8	1.07E+08	1.07E+08	886	-0.81	0.000364	0.00479	ZFPM2-AS1	1.07E+08
chr4	52917742	52918365	624	-0.94	0.000366	0.0048	SPATA18	52912496
chr4	52917742	52918365	624	-0.94	0.000366	0.0048	SPATA18	52912496
chr4	52917742	52918365	624	-0.94	0.000366	0.0048	SPATA18	52912496
chr5	1.42E+08	1.42E+08	201	-1.76	0.000366	0.0048	ARHGAP26	1.42E+08
chr7	1.28E+08	1.28E+08	300	-1.22	0.000372	0.00486	LEP	1.28E+08
chr19	15490406	15490819	414	1.27	0.000374	0.00488	AKAP8	15488612
chr12	1.04E+08	1.04E+08	456	-0.87	0.000393	0.00507	NT5DC3	1.04E+08
chr1	68302536	68302751	216	-1.17	0.000409	0.00524	GNG12	68297155
chr1	1.09E+08	1.09E+08	471	-1.02	0.000429	0.00543	FAM102B	1.09E+08
chr8	54792280	54792644	365	-0.89	0.000439	0.00554	RGS20	54788431
chr8	54792280	54792644	365	-0.89	0.000439	0.00554	RGS20	54788431
chr8	54792280	54792644	365	-0.89	0.000439	0.00554	RGS20	54788431
chr14	94855079	94855255	177	-1.37	0.000442	0.00557	SERPINA1	94855029
chr14	94855079	94855255	177	-1.37	0.000442	0.00557	SERPINA1	94855029
chr14	94855079	94855255	177	-1.37	0.000442	0.00557	SERPINA1	94855029
chr14	94855079	94855255	177	-1.37	0.000442	0.00557	SERPINA1	94853153
chr14	94855079	94855255	177	-1.37	0.000442	0.00557	SERPINA1	94855029
chr14	94855079	94855255	177	-1.37	0.000442	0.00557	SERPINA1	94855029
chr14	94855079	94855255	177	-1.37	0.000442	0.00557	SERPINA1	94855029
chr14	94855079	94855255	177	-1.37	0.000442	0.00557	SERPINA1	94855029
chr14	94855079	94855255	177	-1.37	0.000442	0.00557	SERPINA1	94855029
chr14	94855079	94855255	177	-1.37	0.000442	0.00557	SERPINA1	94855029
chr14	94855079	94855255	177	-1.37	0.000442	0.00557	SERPINA1	94855029
chr8	1.45E+08	1.45E+08	304	1.14	0.000448	0.00561	CYC1	1.45E+08

chr2	1.88E+08	1.88E+08	246	-1.58	0.000466	0.00581	TFPI	1.88E+08
chr2	1.88E+08	1.88E+08	246	-1.58	0.000466	0.00581	TFPI	1.88E+08
chr2	1.88E+08	1.88E+08	246	-1.58	0.000466	0.00581	TFPI	1.88E+08
chr3	1.33E+08	1.33E+08	232	-1.03	0.000469	0.00584	CDV3	1.33E+08
chr3	1.33E+08	1.33E+08	232	-1.03	0.000469	0.00584	CDV3	1.33E+08
chr3	1.33E+08	1.33E+08	232	-1.03	0.000469	0.00584	CDV3	1.33E+08
chr3	52278212	52278488	277	-1.34	0.00047	0.00584	PPM1M	52275185
chr3	52278212	52278488	277	-1.34	0.00047	0.00584	PPM1M	52274808
chr11	64644477	64644792	316	1.39	0.000471	0.00584	EHD1	64644240
chr15	83957181	83957573	393	-0.88	0.000471	0.00585	BNC1	83951468
chr15	83957181	83957573	393	-0.88	0.000471	0.00585	BNC1	83950209
chr20	6034680	6034970	291	-1.21	0.000483	0.00596	LRRN4	6032726
chr10	77189938	77190154	217	-1.29	0.000489	0.00602	C10orf11	77186403
chr19	50269563	50270158	596	0.8	0.000494	0.00606	AP2A1	50265179
chr19	50269563	50270158	596	0.8	0.000494	0.00606	TSKS	50264515
chr19	50269563	50270158	596	0.8	0.000494	0.00606	AP2A1	50265179
chr1	1.63E+08	1.63E+08	184	-2.57	0.000495	0.00608	RGS5	1.63E+08
chr1	1.63E+08	1.63E+08	184	-2.57	0.000495	0.00608	RGS5	1.63E+08
chr1	1.63E+08	1.63E+08	184	-2.57	0.000495	0.00608	RGS5	1.63E+08
chr17	7155099	7155917	819	0.88	0.000497	0.00609	ELP5	7150371
chr17	7155099	7155917	819	0.88	0.000497	0.00609	CTDNEP1	7153259
chr17	7155099	7155917	819	0.88	0.000497	0.00609	CTDNEP1	7152995
chr17	7155099	7155917	819	0.88	0.000497	0.00609	ELP5	7150519
chr17	7155099	7155917	819	0.88	0.000497	0.00609	ELP5	7150519
chr17	7155099	7155917	819	0.88	0.000497	0.00609	ELP5	7150519
chr19	5680539	5680986	448	1.09	0.000499	0.00611	HSD11B1L	5675775
chr19	5680539	5680986	448	1.09	0.000499	0.00611	HSD11B1L	5675775
chr19	5680539	5680986	448	1.09	0.000499	0.00611	HSD11B1L	5675775
chr19	5680539	5680986	448	1.09	0.000499	0.00611	HSD11B1L	5675775
chr19	5680539	5680986	448	1.09	0.000499	0.00611	HSD11B1L	5675775
chr19	5680539	5680986	448	1.09	0.000499	0.00611	HSD11B1L	5675775
chr19	5680539	5680986	448	1.09	0.000499	0.00611	C19orf70	5678907
chr19	5680539	5680986	448	1.09	0.000499	0.00611	HSD11B1L	5675775
chr19	5680539	5680986	448	1.09	0.000499	0.00611	HSD11B1L	5675775
chr19	5680539	5680986	448	1.09	0.000499	0.00611	HSD11B1L	5675775
chr19	5680539	5680986	448	1.09	0.000499	0.00611	HSD11B1L	5675775
chr19	5680539	5680986	448	1.09	0.000499	0.00611	C19orf70	5678907
chr14	92336396	92336584	189	-2.15	0.000499	0.00611	TC2N	92331880
chr7	43944840	43945146	307	-1.13	0.000502	0.00613	URGCP-MR	43944231
chr7	43944840	43945146	307	-1.13	0.000502	0.00613	URGCP	43944668
chr7	43944840	43945146	307	-1.13	0.000502	0.00613	URGCP	43944668
chr7	43944840	43945146	307	-1.13	0.000502	0.00613	URGCP	43944668
chr8	1.21E+08	1.21E+08	900	-0.78	0.000509	0.00619	DSCC1	1.21E+08
chr3	1.19E+08	1.19E+08	736	-0.86	0.000509	0.00619	ARHGAP31	1.19E+08
chr4	74606171	74606474	304	-1.36	0.000531	0.00639	CXCL8	74601222
chr17	4607136	4607522	387	1.28	0.000537	0.00644	PELP1	4605632
chr17	4607136	4607522	387	1.28	0.000537	0.00644	PELP1	4605632

chr17	4607136	4607522	387	1.28	0.000537	0.00644	LOC10155§	4602524
chr7	1.5E+08	1.5E+08	433	-1.27	0.000543	0.00649	GIMAP2	1.5E+08
chr3	1.6E+08	1.6E+08	656	-0.87	0.000545	0.0065	IL12A	1.6E+08
chr12	1.09E+08	1.09E+08	192	-1.31	0.000549	0.00654	CORO1C	1.09E+08
chr12	1.09E+08	1.09E+08	192	-1.31	0.000549	0.00654	CORO1C	1.09E+08
chr9	26949682	26949949	268	-1.36	0.000552	0.00656	PLAA	26945468
chr1	2.48E+08	2.48E+08	745	-0.83	0.000571	0.00671	NLRP3	2.48E+08
chr1	2.48E+08	2.48E+08	745	-0.83	0.000571	0.00671	NLRP3	2.48E+08
chr1	2.48E+08	2.48E+08	745	-0.83	0.000571	0.00671	NLRP3	2.48E+08
chr1	2.48E+08	2.48E+08	745	-0.83	0.000571	0.00671	NLRP3	2.48E+08
chr1	2.48E+08	2.48E+08	745	-0.83	0.000571	0.00671	NLRP3	2.48E+08
chr1	2.48E+08	2.48E+08	745	-0.83	0.000571	0.00671	NLRP3	2.48E+08
chr3	1.6E+08	1.6E+08	926	-0.81	0.000578	0.00678	ARL14	1.6E+08
chr4	71265125	71265259	135	-2.57	0.00058	0.00679	PROL1	71258598
chr4	71265125	71265259	135	-2.57	0.00058	0.00679	PROL1	71258598
chr4	71265125	71265259	135	-2.57	0.00058	0.00679	PROL1	71258598
chr16	89752924	89753421	498	1.12	0.000583	0.00681	CDK10	89748075
chr16	89752924	89753421	498	1.12	0.000583	0.00681	CDK10	89748075
chr16	89752924	89753421	498	1.12	0.000583	0.00681	CDK10	89748075
chr16	89752924	89753421	498	1.12	0.000583	0.00681	CDK10	89748075
chr16	89752924	89753421	498	1.12	0.000583	0.00681	CDK10	89748075
chr16	89752924	89753421	498	1.12	0.000583	0.00681	CDK10	89748075
chr5	40755204	40756224	1021	-0.86	0.000602	0.007	TTC33	40754072
chr1	1.17E+08	1.17E+08	734	-1.03	0.000613	0.00708	MAB21L3	1.17E+08
chr3	11609681	11610160	480	-1.22	0.000631	0.00723	VGLL4	11608398
chr7	36406466	36407071	606	-0.96	0.000632	0.00724	KIAA0895	36404850
chr7	36406466	36407071	606	-0.96	0.000632	0.00724	KIAA0895	36404878
chr7	36406466	36407071	606	-0.96	0.000632	0.00724	KIAA0895	36404782
chr7	36406466	36407071	606	-0.96	0.000632	0.00724	KIAA0895	36404842
chr7	36406466	36407071	606	-0.96	0.000632	0.00724	KIAA0895	36404878
chr8	67089145	67089905	761	-0.89	0.000635	0.00727	CRH	67088880
chr12	1.27E+08	1.27E+08	527	-0.73	0.000646	0.00736	LINC00944	1.27E+08
chr2	2.36E+08	2.36E+08	224	-1.28	0.000649	0.00739	LINC01173	2.36E+08
chr3	61547125	61547292	168	-1.89	0.000657	0.00746	PTPRG	61542242
chr11	1.13E+08	1.13E+08	198	-1.44	0.00069	0.00774	MIR4301	1.13E+08
chr14	1.02E+08	1.02E+08	488	-1.18	0.000722	0.00798	LINC00239	1.02E+08
chr1	2.24E+08	2.24E+08	567	-1.07	0.00073	0.00804	CAPN8	2.24E+08
chr16	85785744	85786077	334	-1	0.000732	0.00805	C16orf74	85782689
chr13	36429766	36430083	318	-1.75	0.000748	0.00819	DCLK1	36427998
chr13	36429766	36430083	318	-1.75	0.000748	0.00819	DCLK1	36427998
chr13	36429766	36430083	318	-1.75	0.000748	0.00819	DCLK1	36427998
chr15	53082247	53082762	516	-1.26	0.00075	0.0082	ONECUT1	53080209
chr5	35852212	35852410	199	-1.4	0.00075	0.0082	IL7R	35851976
chr5	35852212	35852410	199	-1.4	0.00075	0.0082	IL7R	35851976
chr13	96187576	96187817	242	-1.31	0.00077	0.00836	CLDN10-AS	96184164
chr4	26585395	26585884	490	-0.99	0.000773	0.00838	TBC1D19	26580545
chr4	26585395	26585884	490	-0.99	0.000773	0.00838	TBC1D19	26580545

chr12	1.13E+08	1.13E+08	227	-1.22	0.000786	0.00847	OAS3	1.13E+08
chr4	82085844	82086175	332	-1.02	0.000799	0.00859	LOC101928	82081093
chr1	46637059	46637333	275	-1.19	0.00081	0.00869	TSPAN1	46635748
chr9	1.18E+08	1.18E+08	148	-2.08	0.000812	0.00869	TNFSF15	1.18E+08
chr12	58159016	58159433	418	-1.23	0.000813	0.0087	CYP27B1	58158976
chr2	65594709	65594875	167	-1.5	0.000822	0.00878	SPRED2	65591931
chr17	7307251	7307877	627	0.82	0.000854	0.00904	TMEM256-	7305450
chr17	7307251	7307877	627	0.82	0.000854	0.00904	NLGN2	7306501
chr17	7307251	7307877	627	0.82	0.000854	0.00904	TMEM256	7305450
chr2	1.07E+08	1.07E+08	318	-1.16	0.000869	0.00916	UXS1	1.07E+08
chr2	1.07E+08	1.07E+08	318	-1.16	0.000869	0.00916	UXS1	1.07E+08
chr2	1.07E+08	1.07E+08	318	-1.16	0.000869	0.00916	UXS1	1.07E+08
chr13	76056202	76056872	671	-0.83	0.000884	0.00929	TBC1D4	76054304
chr13	76056202	76056872	671	-0.83	0.000884	0.00929	TBC1D4	76054304
chr13	76056202	76056872	671	-0.83	0.000884	0.00929	TBC1D4	76054304
chr5	1.13E+08	1.13E+08	306	-1.05	0.00089	0.00933	MCC	1.13E+08
chr6	36095350	36095561	212	-1.44	0.000892	0.00934	MAPK13	36093260
chr6	36095350	36095561	212	-1.44	0.000892	0.00934	MAPK13	36093260
chr1	1.84E+08	1.84E+08	536	-1.5	0.000894	0.00936	APOBEC4	1.84E+08
chr5	1.72E+08	1.72E+08	252	-1.07	0.000922	0.00958	STK10	1.72E+08
chr9	88742334	88742589	256	-1.29	0.000923	0.00959	LOC101927	88737454
chr9	1.41E+08	1.41E+08	272	1.07	0.00093	0.00963	EHMT1	1.41E+08
chr9	1.41E+08	1.41E+08	272	1.07	0.00093	0.00963	EHMT1	1.41E+08
chr9	1.41E+08	1.41E+08	272	1.07	0.00093	0.00963	ARRDC1-AS	1.41E+08
chr9	1.41E+08	1.41E+08	272	1.07	0.00093	0.00963	ARRDC1-AS	1.41E+08
chr15	44084225	44084542	318	0.89	0.000934	0.00967	MIR1282	44083957
chr15	44084225	44084542	318	0.89	0.000934	0.00967	SERF2	44079039
chr15	44084225	44084542	318	0.89	0.000934	0.00967	SERF2	44079173
chr15	44084225	44084542	318	0.89	0.000934	0.00967	SERF2	44079173
chr15	44084225	44084542	318	0.89	0.000934	0.00967	SERF2	44079574
chr15	44084225	44084542	318	0.89	0.000934	0.00967	SERF2-C15(	44079173
chr15	44084225	44084542	318	0.89	0.000934	0.00967	SERF2	44079173
chr12	70082880	70083170	291	-1.14	0.000943	0.00974	BEST3	70081057
chr12	70082880	70083170	291	-1.14	0.000943	0.00974	BEST3	70081057
chr17	27046563	27047002	440	0.84	0.00096	0.00989	SNORD42B	27042567
chr17	27046563	27047002	440	0.84	0.00096	0.00989	SNORD4A	27044599
chr17	27046563	27047002	440	0.84	0.00096	0.00989	SNORD4B	27045698
chr17	27046563	27047002	440	0.84	0.00096	0.00989	RAB34	27043286
chr17	27046563	27047002	440	0.84	0.00096	0.00989	RPL23A	27041999
chr17	27046563	27047002	440	0.84	0.00096	0.00989	RAB34	27043286
chr17	27046563	27047002	440	0.84	0.00096	0.00989	NARR	27042908
chr17	27046563	27047002	440	0.84	0.00096	0.00989	RAB34	27043286
chr17	27046563	27047002	440	0.84	0.00096	0.00989	RAB34	27043286
chr17	27046563	27047002	440	0.84	0.00096	0.00989	RAB34	27043286
chr17	27046563	27047002	440	0.84	0.00096	0.00989	SNORD42A	27045446
chr17	27046563	27047002	440	0.84	0.00096	0.00989	RAB34	27042908
chr17	27046563	27047002	440	0.84	0.00096	0.00989	RAB34	27043286

chr17	27046563	27047002	440	0.84	0.00096	0.00989	RAB34	27042908
chr1	2.47E+08	2.47E+08	137	-1.4	0.000968	0.00995	C1orf229	2.47E+08
chr20	17674390	17674737	348	-1.47	0.000993	0.0101	BANF2	17669319
chr1	31229661	31229901	241	-1.76	0.000996	0.0102	LAPTM5	31228683
chr11	2011248	2011422	175	1.12	0.000999	0.0102	MRPL23-AS	2009150
chr11	2011248	2011422	175	1.12	0.000999	0.0102	LINC01219	2007325
chr11	2011248	2011422	175	1.12	0.000999	0.0102	HOTS	2008637
chr1	2.32E+08	2.32E+08	353	-1.25	0.001	0.0102	DISC1	2.32E+08
chr1	2.32E+08	2.32E+08	353	-1.25	0.001	0.0102	DISC1	2.32E+08
chr1	2.32E+08	2.32E+08	353	-1.25	0.001	0.0102	DISC1	2.32E+08
chr1	2.32E+08	2.32E+08	353	-1.25	0.001	0.0102	DISC1	2.32E+08
chr1	2.32E+08	2.32E+08	353	-1.25	0.001	0.0102	DISC1	2.32E+08
chr1	2.32E+08	2.32E+08	353	-1.25	0.001	0.0102	DISC1	2.32E+08
chr1	2.32E+08	2.32E+08	353	-1.25	0.001	0.0102	DISC1	2.32E+08
chr1	2.32E+08	2.32E+08	353	-1.25	0.001	0.0102	DISC1	2.32E+08
chr1	2.32E+08	2.32E+08	353	-1.25	0.001	0.0102	DISC1	2.32E+08
chr1	2.32E+08	2.32E+08	353	-1.25	0.001	0.0102	DISC1	2.32E+08
chr1	2.32E+08	2.32E+08	353	-1.25	0.001	0.0102	DISC1	2.32E+08
chr1	2.32E+08	2.32E+08	353	-1.25	0.001	0.0102	DISC1	2.32E+08
chr1	2.32E+08	2.32E+08	353	-1.25	0.001	0.0102	DISC1	2.32E+08
chr1	2.32E+08	2.32E+08	353	-1.25	0.001	0.0102	DISC1	2.32E+08
chr1	2.32E+08	2.32E+08	353	-1.25	0.001	0.0102	DISC1	2.32E+08
chr1	2.32E+08	2.32E+08	353	-1.25	0.001	0.0102	DISC1	2.32E+08
chr1	2.32E+08	2.32E+08	353	-1.25	0.001	0.0102	DISC1	2.32E+08
chr1	2.32E+08	2.32E+08	353	-1.25	0.001	0.0102	DISC1	2.32E+08
chr1	2.32E+08	2.32E+08	353	-1.25	0.001	0.0102	DISC1	2.32E+08
chr1	2.32E+08	2.32E+08	353	-1.25	0.001	0.0102	DISC1	2.32E+08
chr1	2.32E+08	2.32E+08	353	-1.25	0.001	0.0102	DISC1	2.32E+08
chr1	2.32E+08	2.32E+08	353	-1.25	0.001	0.0102	DISC1	2.32E+08
chr14	67881031	67881205	175	-1.34	0.00101	0.0103	PLEK2	67876917
chr13	1.01E+08	1.01E+08	479	-0.99	0.00102	0.0103	GGACT	1.01E+08
chr6	18387272	18387838	567	-0.71	0.00104	0.0105	RNF144B	18382580
chr1	89664137	89664689	553	-1.06	0.00104	0.0105	GBP4	89662633
chr1	1.11E+08	1.11E+08	423	-0.88	0.00105	0.0106	AHCYL1	1.11E+08
chr1	1.11E+08	1.11E+08	423	-0.88	0.00105	0.0106	AHCYL1	1.11E+08
chr8	1.24E+08	1.24E+08	503	-0.77	0.00106	0.0106	LOC105375	1.24E+08
chr17	7232670	7233047	378	1.08	0.00106	0.0106	NEURL4	7230638
chr17	7232670	7233047	378	1.08	0.00106	0.0106	NEURL4	7230638
chr3	1.69E+08	1.69E+08	467	-0.9	0.00107	0.0107	MECOM	1.69E+08
chr3	1.69E+08	1.69E+08	467	-0.9	0.00107	0.0107	MECOM	1.69E+08
chr8	1.35E+08	1.35E+08	391	-0.82	0.00108	0.0108	ST3GAL1	1.35E+08
chr8	1.35E+08	1.35E+08	391	-0.82	0.00108	0.0108	ST3GAL1	1.35E+08
chr16	89882972	89883382	411	0.95	0.00108	0.0108	FANCA	89881065
chr16	89882972	89883382	411	0.95	0.00108	0.0108	FANCA	89881065
chr16	89882972	89883382	411	0.95	0.00108	0.0108	FANCA	89881065
chr8	74268583	74269029	447	-0.85	0.00108	0.0108	RDH10-AS1	74266696

chr17	7760428	7761082	655	0.81	0.0011	0.0109	NAA38	7759172
chr17	7760428	7761082	655	0.81	0.0011	0.0109	CYB5D1	7756063
chr3	40350961	40351384	424	-0.74	0.0011	0.0109	EIF1B-AS1	40349189
chr3	40350961	40351384	424	-0.74	0.0011	0.0109	EIF1B	40346172
chr16	19430987	19431343	357	-1.87	0.0011	0.0109	TMC5	19424017
chr17	42277443	42277875	433	0.87	0.00111	0.011	ATXN7L3	42273529
chr17	42277443	42277875	433	0.87	0.00111	0.011	ATXN7L3	42273529
chr1	1.83E+08	1.83E+08	1032	-0.86	0.00111	0.011	SMG7	1.83E+08
chr1	1.83E+08	1.83E+08	1032	-0.86	0.00111	0.011	SMG7	1.83E+08
chr1	1.83E+08	1.83E+08	1032	-0.86	0.00111	0.011	SMG7	1.83E+08
chr1	1.83E+08	1.83E+08	1032	-0.86	0.00111	0.011	SMG7	1.83E+08
chr1	1.83E+08	1.83E+08	1032	-0.86	0.00111	0.011	SMG7-AS1	1.83E+08
chr2	1.33E+08	1.33E+08	345	-0.98	0.00112	0.011	LYPD1	1.33E+08
chr2	1.33E+08	1.33E+08	345	-0.98	0.00112	0.011	LYPD1	1.33E+08
chr7	18548645	18548974	330	-0.92	0.00112	0.011	HDAC9	18543899
chr7	18548645	18548974	330	-0.92	0.00112	0.011	HDAC9	18543899
chr7	48124466	48124814	349	-0.8	0.00112	0.0111	UPP1	48123199
chr7	48124466	48124814	349	-0.8	0.00112	0.0111	UPP1	48123808
chr7	48124466	48124814	349	-0.8	0.00112	0.0111	UPP1	48123199
chr7	48124466	48124814	349	-0.8	0.00112	0.0111	UPP1	48123850
chr7	48124466	48124814	349	-0.8	0.00112	0.0111	UPP1	48123736
chr7	48124466	48124814	349	-0.8	0.00112	0.0111	UPP1	48123850
chr10	64139822	64140056	235	-0.98	0.00112	0.0111	LOC283045	64132886
chr6	52537286	52537495	210	-1.29	0.00115	0.0113	TMEM14A	52530883
chr5	1.26E+08	1.26E+08	718	-0.8	0.00116	0.0114	GRAMD3	1.26E+08
chr5	1.26E+08	1.26E+08	718	-0.8	0.00116	0.0114	GRAMD3	1.26E+08
chr5	1.26E+08	1.26E+08	718	-0.8	0.00116	0.0114	GRAMD3	1.26E+08
chr3	1.19E+08	1.19E+08	458	-1.39	0.00116	0.0114	IGSF11-AS1	1.19E+08
chr20	52199633	52199807	175	-1.29	0.00119	0.0116	ZNF217	52197636
chr11	65029226	65029538	313	1.24	0.0012	0.0117	POLA2	65024322
chr3	1.32E+08	1.32E+08	224	-1.35	0.0012	0.0117	CPNE4	1.32E+08
chr19	797004	797418	415	1.02	0.00122	0.0118	PTBP1	792391
chr19	797004	797418	415	1.02	0.00122	0.0118	PTBP1	792391
chr19	797004	797418	415	1.02	0.00122	0.0118	PTBP1	792391
chrX	15511326	15511777	452	-1.03	0.00125	0.012	PIR	15509711
chrX	15511326	15511777	452	-1.03	0.00125	0.012	PIR	15509711
chrX	15511326	15511777	452	-1.03	0.00125	0.012	PIR-FIGF	15507432
chr8	1.28E+08	1.28E+08	606	-0.74	0.00126	0.012	CCAT1	1.28E+08
chr16	639487	640046	560	1.03	0.00126	0.012	RAB40C	634668
chr16	639487	640046	560	1.03	0.00126	0.012	RAB40C	634356
chr16	639487	640046	560	1.03	0.00126	0.012	RAB40C	635076
chr16	639487	640046	560	1.03	0.00126	0.012	RAB40C	635076
chr16	639487	640046	560	1.03	0.00126	0.012	RAB40C	635172
chr1	1.82E+08	1.82E+08	984	-0.72	0.00127	0.0121	GLUL	1.82E+08
chr1	1.82E+08	1.82E+08	984	-0.72	0.00127	0.0121	GLUL	1.82E+08
chr1	1.82E+08	1.82E+08	984	-0.72	0.00127	0.0121	GLUL	1.82E+08
chr1	1.83E+08	1.83E+08	405	-1.11	0.00127	0.0121	LOC28464{	1.83E+08

chr19	49588388	49588892	505	0.92	0.00128	0.0122	SNRNP70	49583396
chr19	49588388	49588892	505	0.92	0.00128	0.0122	SNRNP70	49583396
chr9	77501999	77502305	307	-1.31	0.00128	0.0122	TRPM6	77501010
chr9	77501999	77502305	307	-1.31	0.00128	0.0122	TRPM6	77500263
chr9	77501999	77502305	307	-1.31	0.00128	0.0122	TRPM6	77500636
chr15	22956588	22956782	195	-1.52	0.00129	0.0122	CYFIP1	22950754
chr11	1.13E+08	1.13E+08	400	-0.91	0.0013	0.0123	ANKK1	1.13E+08
chr13	47127121	47127335	215	-1.04	0.00131	0.0124	LRCH1	47122295
chr13	47127121	47127335	215	-1.04	0.00131	0.0124	LRCH1	47122295
chr13	47127121	47127335	215	-1.04	0.00131	0.0124	LRCH1	47122295
chr1	22109615	22110206	592	0.98	0.00132	0.0125	USP48	22107688
chr1	22109615	22110206	592	0.98	0.00132	0.0125	USP48	22107688
chr10	75571399	75571858	460	0.9	0.00134	0.0126	NDST2	75569589
chr8	56685382	56686229	848	-0.76	0.00134	0.0126	TMEM68	56683966
chr8	56685382	56686229	848	-0.76	0.00134	0.0126	TMEM68	56683966
chr8	56685382	56686229	848	-0.76	0.00134	0.0126	TMEM68	56683966
chr8	56685382	56686229	848	-0.76	0.00134	0.0126	TGS1	56680790
chr8	56685382	56686229	848	-0.76	0.00134	0.0126	TGS1	56680790
chr1	2.34E+08	2.34E+08	171	-1.4	0.00134	0.0126	SLC35F3	2.34E+08
chr2	1.2E+08	1.2E+08	292	-0.92	0.00135	0.0127	STEAP3-AS	1.2E+08
chr5	1.19E+08	1.19E+08	523	-0.87	0.00135	0.0127	TNFAIP8	1.19E+08
chr5	1.19E+08	1.19E+08	523	-0.87	0.00135	0.0127	TNFAIP8	1.19E+08
chr6	8102177	8103044	868	-0.72	0.00136	0.0127	EEF1E1	8100828
chr6	8102177	8103044	868	-0.72	0.00136	0.0127	EEF1E1	8100828
chr6	8102177	8103044	868	-0.72	0.00136	0.0127	EEF1E1-BL(	8100828
chr6	1.37E+08	1.37E+08	143	-2.13	0.00136	0.0127	LOC10192(	1.37E+08
chr1	2.22E+08	2.22E+08	569	-0.66	0.00136	0.0127	DUSP10	2.22E+08
chr1	2.22E+08	2.22E+08	569	-0.66	0.00136	0.0127	DUSP10	2.22E+08
chr11	506798	507476	679	0.94	0.00143	0.0132	RNH1	505283
chr11	506798	507476	679	0.94	0.00143	0.0132	RNH1	505283
chr11	506798	507476	679	0.94	0.00143	0.0132	RNH1	505283
chr11	506798	507476	679	0.94	0.00143	0.0132	RNH1	505283
chr11	506798	507476	679	0.94	0.00143	0.0132	RNH1	504821
chr11	506798	507476	679	0.94	0.00143	0.0132	RNH1	504821
chr11	506798	507476	679	0.94	0.00143	0.0132	RNH1	504821
chr11	506798	507476	679	0.94	0.00143	0.0132	RNH1	504821
chr18	3602948	3603662	715	-0.72	0.00144	0.0132	DLGAP1-AS	3598734
chr17	79650741	79651078	338	0.91	0.00144	0.0133	ARL16	79648954
chr17	79650741	79651078	338	0.91	0.00144	0.0133	HGS	79645961
chr2	2.17E+08	2.17E+08	217	-1.56	0.00144	0.0133	PKI55	2.17E+08
chr19	49990520	49990838	319	1.06	0.00145	0.0133	SNORD32A	49988222
chr19	49990520	49990838	319	1.06	0.00145	0.0133	RPL13A	49985810
chr19	49990520	49990838	319	1.06	0.00145	0.0133	RPL13A	49985810
chr19	49990520	49990838	319	1.06	0.00145	0.0133	SNORD33	49988873
chr19	49990520	49990838	319	1.06	0.00145	0.0133	SNORD34	49989163
chr19	49990520	49990838	319	1.06	0.00145	0.0133	SNORD35A	49989431
chr19	49990520	49990838	319	1.06	0.00145	0.0133	RPL13AP5	49985864

chr19	49990520	49990838	319	1.06	0.00145	0.0133	RPL13A	49985810
chr10	97054837	97055165	329	-0.96	0.00145	0.0133	PDLIM1	97048905
chr6	10419289	10419569	281	-0.87	0.00146	0.0133	TFAP2A	10417797
chr6	10419289	10419569	281	-0.87	0.00146	0.0133	TFAP2A	10413470
chr12	1.31E+08	1.31E+08	223	-0.98	0.00148	0.0134	RAN	1.31E+08
chr12	1.31E+08	1.31E+08	223	-0.98	0.00148	0.0134	RAN	1.31E+08
chr12	1.31E+08	1.31E+08	223	-0.98	0.00148	0.0134	RAN	1.31E+08
chr10	43187340	43187567	228	-1.21	0.00148	0.0135	LINC01518	43185207
chr10	43187340	43187567	228	-1.21	0.00148	0.0135	LINC01518	43185207
chr10	43187340	43187567	228	-1.21	0.00148	0.0135	LINC01518	43185207
chr10	43187340	43187567	228	-1.21	0.00148	0.0135	LINC01518	43185207
chr5	86414033	86414198	166	-1.37	0.00151	0.0137	LOC10192[illegible]	86410964
chr5	86414033	86414198	166	-1.37	0.00151	0.0137	MIR4280	86408771
chr1	2.17E+08	2.17E+08	207	-1.14	0.00152	0.0138	ESRRG	2.17E+08
chr1	2.17E+08	2.17E+08	207	-1.14	0.00152	0.0138	ESRRG	2.17E+08
chr1	2.17E+08	2.17E+08	207	-1.14	0.00152	0.0138	ESRRG	2.17E+08
chr1	2.17E+08	2.17E+08	207	-1.14	0.00152	0.0138	ESRRG	2.17E+08
chr10	88729173	88729328	156	-1.32	0.00153	0.0138	AGAP11	88725497
chr10	88729173	88729328	156	-1.32	0.00153	0.0138	ADIRF	88723187
chr19	9929811	9930451	641	0.92	0.00153	0.0138	FBXL12	9927779
chr19	9929811	9930451	641	0.92	0.00153	0.0138	FBXL12	9927779
chr19	9929811	9930451	641	0.92	0.00153	0.0138	FBXL12	9927779
chr19	9929811	9930451	641	0.92	0.00153	0.0138	FBXL12	9927779
chr19	9929811	9930451	641	0.92	0.00153	0.0138	FBXL12	9927779
chr19	9929811	9930451	641	0.92	0.00153	0.0138	FBXL12	9927779
chr19	9929811	9930451	641	0.92	0.00153	0.0138	FBXL12	9927779
chr19	9929811	9930451	641	0.92	0.00153	0.0138	FBXL12	9927779
chr8	97247496	97248011	516	-0.71	0.00156	0.014	UQCRB	97245862
chr8	97247496	97248011	516	-0.71	0.00156	0.014	UQCRB	97245862
chr8	97247496	97248011	516	-0.71	0.00156	0.014	UQCRB	97245862
chr8	97247496	97248011	516	-0.71	0.00156	0.014	UQCRB	97245862
chr17	10278599	10278803	205	-1.97	0.00157	0.0141	MYH13	10274322
chr1	2.47E+08	2.47E+08	508	-0.83	0.00158	0.0141	ZNF670	2.47E+08
chr1	2.47E+08	2.47E+08	508	-0.83	0.00158	0.0141	ZNF670-ZN	2.47E+08
chr1	2.47E+08	2.47E+08	508	-0.83	0.00158	0.0141	ZNF670	2.47E+08
chr3	69435088	69435512	425	-1.34	0.00159	0.0142	FRMD4B	69433455
chr22	38902385	38902640	256	1.12	0.00161	0.0143	DDX17	38900345
chr22	38902385	38902640	256	1.12	0.00161	0.0143	DDX17	38900345
chr2	2.33E+08	2.33E+08	424	0.98	0.00161	0.0143	ALPP	2.33E+08
chr19	42806597	42806805	209	1.79	0.00162	0.0144	PAFAH1B3	42804952
chr19	42806597	42806805	209	1.79	0.00162	0.0144	PAFAH1B3	42804796
chr19	42806597	42806805	209	1.79	0.00162	0.0144	PAFAH1B3	42804929
chr19	42806597	42806805	209	1.79	0.00162	0.0144	PRR19	42801283
chr6	16131277	16131487	211	-1.12	0.00162	0.0144	MYLIP	16124316
chr10	1.3E+08	1.3E+08	679	-0.78	0.00165	0.0145	MKI67	1.3E+08
chr10	1.3E+08	1.3E+08	679	-0.78	0.00165	0.0145	MKI67	1.3E+08
chr6	1.32E+08	1.32E+08	183	-1.58	0.00167	0.0146	LINC01013	1.32E+08

chr17	26972057	26972427	371	0.91	0.00169	0.0148	KIAA0100	26970177
chr8	68255550	68256401	852	-0.74	0.00169	0.0148	ARFGEF1	68253912
chr6	24667042	24667460	419	-0.76	0.00169	0.0148	ACOT13	24662262
chr6	24667042	24667460	419	-0.76	0.00169	0.0148	ACOT13	24662262
chr6	24667042	24667460	419	-0.76	0.00169	0.0148	TDP2	24665115
chr17	79633597	79633804	208	1	0.00172	0.015	OXLD1	79631667
chr17	79633597	79633804	208	1	0.00172	0.015	OXLD1	79631667
chr17	79633597	79633804	208	1	0.00172	0.015	OXLD1	79631667
chr17	79633597	79633804	208	1	0.00172	0.015	OXLD1	79631667
chr17	79633597	79633804	208	1	0.00172	0.015	OXLD1	79631176
chr17	79633597	79633804	208	1	0.00172	0.015	OXLD1	79631667
chr17	79633597	79633804	208	1	0.00172	0.015	OXLD1	79631667
chr17	79633597	79633804	208	1	0.00172	0.015	OXLD1	79631667
chr17	79633597	79633804	208	1	0.00172	0.015	CCDC137	79628760
chr8	1.24E+08	1.24E+08	542	-0.69	0.00176	0.0153	FAM83A	1.24E+08
chr19	18668502	18668707	206	1.58	0.00176	0.0153	KXD1	18663571
chr19	18668502	18668707	206	1.58	0.00176	0.0153	KXD1	18663571
chr19	18668502	18668707	206	1.58	0.00176	0.0153	KXD1	18663571
chr10	1.16E+08	1.16E+08	525	-0.84	0.00176	0.0153	ABLIM1	1.16E+08
chr8	57232629	57233338	710	-0.89	0.00178	0.0154	SDR16C5	57231335
chr8	57232629	57233338	710	-0.89	0.00178	0.0154	SDR16C5	57231335
chr8	57232629	57233338	710	-0.89	0.00178	0.0154	SDR16C5	57231335
chr11	66886309	66886799	491	1.01	0.00179	0.0154	KDM2A	66881739
chr11	66886309	66886799	491	1.01	0.00179	0.0154	KDM2A	66881739
chr1	2.21E+08	2.21E+08	316	-1.07	0.00179	0.0154	MARK1	2.21E+08
chr1	2.21E+08	2.21E+08	316	-1.07	0.00179	0.0154	MARK1	2.21E+08
chr1	2.21E+08	2.21E+08	316	-1.07	0.00179	0.0154	MARK1	2.21E+08
chr1	2.21E+08	2.21E+08	316	-1.07	0.00179	0.0154	MARK1	2.21E+08
chr1	2.21E+08	2.21E+08	316	-1.07	0.00179	0.0154	MARK1	2.21E+08
chr1	2.31E+08	2.31E+08	382	-0.96	0.00179	0.0155	MIR1182	2.31E+08
chr11	59435789	59436010	222	-1.2	0.0018	0.0155	PATL1	59434511
chr17	2414971	2415330	360	1	0.0018	0.0155	METTL16	2413200
chr10	62493086	62493453	368	-0.81	0.0018	0.0155	ANK3	62491284
chr19	48673566	48673988	423	0.85	0.00181	0.0156	LIG1	48671860
chr19	48673566	48673988	423	0.85	0.00181	0.0156	LIG1	48671860
chr19	48673566	48673988	423	0.85	0.00181	0.0156	LIG1	48671860
chr19	48673566	48673988	423	0.85	0.00181	0.0156	LIG1	48671860
chr19	48673566	48673988	423	0.85	0.00181	0.0156	C19orf68	48668948
chr14	71249173	71249467	295	-1.21	0.00183	0.0156	MAP3K9	71248169
chr14	71249173	71249467	295	-1.21	0.00183	0.0156	MAP3K9	71248169
chr11	4208705	4209010	306	-1.07	0.00183	0.0157	LOC10050(	4203381
chr10	1.14E+08	1.14E+08	597	-0.88	0.00183	0.0157	GPAM	1.14E+08
chr10	1.14E+08	1.14E+08	597	-0.88	0.00183	0.0157	GPAM	1.14E+08
chr22	21392088	21392373	286	1.19	0.00184	0.0157	MIR649	21386561
chr6	17706290	17706409	120	-1.39	0.00185	0.0158	NUP153	17705065
chr6	17706290	17706409	120	-1.39	0.00185	0.0158	NUP153	17705065
chr6	17706290	17706409	120	-1.39	0.00185	0.0158	NUP153	17705065

chr6	17706290	17706409	120	-1.39	0.00185	0.0158	LOC105374	17701360
chr6	17706290	17706409	120	-1.39	0.00185	0.0158	LOC105374	17702273
chr22	38577750	38578076	327	0.79	0.00186	0.0159	PLA2G6	38575761
chr22	38577750	38578076	327	0.79	0.00186	0.0159	PLA2G6	38575761
chr22	38577750	38578076	327	0.79	0.00186	0.0159	PLA2G6	38575836
chr11	7534528	7535226	699	-0.97	0.00187	0.0159	PPFIBP2	7529995
chr1	2.15E+08	2.15E+08	350	-0.99	0.00188	0.0159	KCNK2	2.15E+08
chr19	11545943	11546389	447	0.89	0.00189	0.016	CCDC151	11543980
chr19	11545943	11546389	447	0.89	0.00189	0.016	CCDC151	11543980
chr19	11545943	11546389	447	0.89	0.00189	0.016	CCDC151	11544603
chr19	11545943	11546389	447	0.89	0.00189	0.016	PRKCSH	11541077
chr19	11545943	11546389	447	0.89	0.00189	0.016	PRKCSH	11541560
chr19	11545943	11546389	447	0.89	0.00189	0.016	PRKCSH	11541077
chr19	11545943	11546389	447	0.89	0.00189	0.016	PRKCSH	11541465
chr19	11545943	11546389	447	0.89	0.00189	0.016	PRKCSH	11541077
chr9	90340868	90341449	582	-0.98	0.00189	0.016	CTSL	90335973
chr9	90340868	90341449	582	-0.98	0.00189	0.016	CTSL	90335973
chr9	90340868	90341449	582	-0.98	0.00189	0.016	CTSL	90335973
chr9	90340868	90341449	582	-0.98	0.00189	0.016	CTSL	90335973
chr9	90340868	90341449	582	-0.98	0.00189	0.016	CTSL	90335973
chr18	11857380	11857720	341	-1.06	0.0019	0.016	GNAL	11852436
chr4	75719097	75719973	877	-0.74	0.00191	0.0162	BTC	75717896
chr4	75719097	75719973	877	-0.74	0.00191	0.0162	BTC	75717896
chr1	1.66E+08	1.66E+08	116	-1.36	0.00192	0.0162	LOC440700	1.66E+08
chr5	1.63E+08	1.63E+08	781	-0.81	0.00193	0.0162	CCNG1	1.63E+08
chr5	1.63E+08	1.63E+08	781	-0.81	0.00193	0.0162	CCNG1	1.63E+08
chr3	13589542	13589753	212	-1.12	0.00193	0.0162	FBLN2	13585624
chr3	13589542	13589753	212	-1.12	0.00193	0.0162	FBLN2	13585624
chr17	41910498	41910629	132	1.57	0.00194	0.0163	MPP3	41908547
chr17	41910498	41910629	132	1.57	0.00194	0.0163	MPP3	41908547
chr3	1.39E+08	1.39E+08	748	-0.68	0.00195	0.0163	LOC100507	1.39E+08
chr3	1.39E+08	1.39E+08	748	-0.68	0.00195	0.0163	COPB2	1.39E+08
chr3	1.39E+08	1.39E+08	748	-0.68	0.00195	0.0163	LOC100507	1.39E+08
chr3	1.39E+08	1.39E+08	748	-0.68	0.00195	0.0163	COPB2	1.39E+08
chr4	10023016	10023486	471	-0.93	0.00195	0.0163	SLC2A9	10021114
chr13	1.14E+08	1.14E+08	210	1.17	0.00196	0.0164	LAMP1	1.14E+08
chr3	1.71E+08	1.71E+08	323	-0.88	0.00199	0.0166	EIF5A2	1.71E+08
chr4	81187522	81188191	670	-0.83	0.002	0.0166	FGF5	81182741
chr4	81187522	81188191	670	-0.83	0.002	0.0166	FGF5	81184418
chr4	81187522	81188191	670	-0.83	0.002	0.0166	FGF5	81182741
chr18	657388	658541	1154	-0.69	0.00201	0.0167	TYMSOS	656340
chr18	657388	658541	1154	-0.69	0.00201	0.0167	TYMS	652603
chr13	88323347	88323777	431	-0.93	0.00201	0.0167	MIR4500H(	88321218
chr13	88323347	88323777	431	-0.93	0.00201	0.0167	SLITRK5	88319869
chr16	74325637	74326080	444	-0.71	0.00203	0.0168	PSMD7	74325672
chr10	1.04E+08	1.04E+08	432	-0.92	0.00203	0.0169	TRIM8	1.04E+08
chr2	2.42E+08	2.42E+08	252	1.02	0.00203	0.0169	HDLBP	2.42E+08

chr2	2.42E+08	2.42E+08	252	1.02	0.00203	0.0169	2-Sep	2.42E+08
chr2	2.42E+08	2.42E+08	252	1.02	0.00203	0.0169	2-Sep	2.42E+08
chr2	2.42E+08	2.42E+08	252	1.02	0.00203	0.0169	2-Sep	2.42E+08
chr2	2.42E+08	2.42E+08	252	1.02	0.00203	0.0169	2-Sep	2.42E+08
chr2	2.42E+08	2.42E+08	252	1.02	0.00203	0.0169	2-Sep	2.42E+08
chr2	2.42E+08	2.42E+08	252	1.02	0.00203	0.0169	2-Sep	2.42E+08
chr8	67036030	67036862	833	-0.7	0.00204	0.0169	TRIM55	67034277
chr8	67036030	67036862	833	-0.7	0.00204	0.0169	TRIM55	67034277
chr8	67036030	67036862	833	-0.7	0.00204	0.0169	TRIM55	67034277
chr8	67036030	67036862	833	-0.7	0.00204	0.0169	TRIM55	67034277
chr1	1.11E+08	1.11E+08	620	-0.95	0.00205	0.017	CYMP	1.11E+08
chr4	1.01E+08	1.01E+08	503	-0.87	0.00206	0.017	DAPP1	1.01E+08
chr4	1.01E+08	1.01E+08	503	-0.87	0.00206	0.017	DAPP1	1.01E+08
chr16	47921489	47921771	283	-1.1	0.00208	0.0171	LOC100507	47919041
chr12	1.02E+08	1.02E+08	1097	-0.68	0.00208	0.0172	ARL1	1.02E+08
chr12	1.02E+08	1.02E+08	1097	-0.68	0.00208	0.0172	ARL1	1.02E+08
chr16	30569680	30569976	297	0.95	0.00209	0.0172	ZNF764	30567642
chr16	30569680	30569976	297	0.95	0.00209	0.0172	ZNF764	30567642
chr17	73717096	73717564	469	0.89	0.0021	0.0173	ITGB4	73712515
chr17	73717096	73717564	469	0.89	0.0021	0.0173	ITGB4	73715775
chr17	73717096	73717564	469	0.89	0.0021	0.0173	ITGB4	73712515
chr9	1.31E+08	1.31E+08	459	0.87	0.00211	0.0173	SWI5	1.31E+08
chr9	1.31E+08	1.31E+08	459	0.87	0.00211	0.0173	GOLGA2	1.31E+08
chr9	1.31E+08	1.31E+08	459	0.87	0.00211	0.0173	SWI5	1.31E+08
chr9	1.31E+08	1.31E+08	459	0.87	0.00211	0.0173	SWI5	1.31E+08
chr6	1.26E+08	1.26E+08	396	-1	0.00212	0.0173	NCOA7	1.26E+08
chr3	1.9E+08	1.9E+08	174	-1	0.00212	0.0173	CLDN16	1.9E+08
chr12	75874381	75874681	301	-1.04	0.00213	0.0174	GLIPR1	75869512
chr10	97205146	97205306	161	-1.69	0.00214	0.0174	SORBS1	97198937
chr10	97205146	97205306	161	-1.69	0.00214	0.0174	SORBS1	97198937
chr10	97205146	97205306	161	-1.69	0.00214	0.0174	SORBS1	97198937
chr10	97205146	97205306	161	-1.69	0.00214	0.0174	SORBS1	97198937
chr10	97205146	97205306	161	-1.69	0.00214	0.0174	SORBS1	97198937
chr10	97205146	97205306	161	-1.69	0.00214	0.0174	SORBS1	97198937
chr10	97205146	97205306	161	-1.69	0.00214	0.0174	SORBS1	97198937
chr10	97205146	97205306	161	-1.69	0.00214	0.0174	SORBS1	97198937
chr6	2857921	2858247	327	-1.17	0.00216	0.0176	MIR4645	2852341
chr10	1.29E+08	1.29E+08	331	-0.79	0.00217	0.0177	DOCK1	1.29E+08
chr10	1.29E+08	1.29E+08	331	-0.79	0.00217	0.0177	DOCK1	1.29E+08
chr10	54078799	54079151	353	-0.83	0.00219	0.0178	PRKG1-AS1	54071888
chr10	1.33E+08	1.33E+08	212	-1.33	0.00222	0.0179	TCERG1L-A	1.33E+08
chr10	1.14E+08	1.14E+08	648	-0.85	0.00226	0.0182	VTI1A	1.14E+08
chr10	1.14E+08	1.14E+08	648	-0.85	0.00226	0.0182	VTI1A	1.14E+08
chr10	1.14E+08	1.14E+08	648	-0.85	0.00226	0.0182	VTI1A	1.14E+08
chr10	1.14E+08	1.14E+08	648	-0.85	0.00226	0.0182	VTI1A	1.14E+08
chr10	1.14E+08	1.14E+08	648	-0.85	0.00226	0.0182	VTI1A	1.14E+08
chr10	1.14E+08	1.14E+08	648	-0.85	0.00226	0.0182	ZDHHC6	1.14E+08

chr10	1.14E+08	1.14E+08	648	-0.85	0.00226	0.0182	VTI1A	1.14E+08
chr10	1.14E+08	1.14E+08	648	-0.85	0.00226	0.0182	ZDHHC6	1.14E+08
chr8	1.31E+08	1.31E+08	807	-0.76	0.00229	0.0183	GSDMC	1.31E+08
chr17	40829101	40829326	226	0.98	0.00233	0.0186	PLEKHH3	40826108
chr17	40829101	40829326	226	0.98	0.00233	0.0186	PLEKHH3	40827048
chr17	40829101	40829326	226	0.98	0.00233	0.0186	PLEKHH3	40827048
chr3	1.19E+08	1.19E+08	505	-0.86	0.00237	0.0188	ARHGAP31	1.19E+08
chr14	70883333	70883966	634	-0.94	0.00237	0.0188	SYNJ2BP-C(	70881807
chr14	70883333	70883966	634	-0.94	0.00237	0.0188	SYNJ2BP-C(	70881807
chr14	70883333	70883966	634	-0.94	0.00237	0.0188	SYNJ2BP-C(	70881807
chr14	70883333	70883966	634	-0.94	0.00237	0.0188	SYNJ2BP	70881807
chr2	24587874	24588207	334	-1.08	0.00241	0.0191	ITSN2	24581397
chr2	24587874	24588207	334	-1.08	0.00241	0.0191	ITSN2	24581397
chr2	24587874	24588207	334	-1.08	0.00241	0.0191	ITSN2	24581397
chr12	28128150	28128380	231	-0.98	0.00241	0.0191	PTHLH	28122916
chr12	28128150	28128380	231	-0.98	0.00241	0.0191	PTHLH	28122916
chr1	55264840	55264994	155	-1.29	0.00242	0.0191	TTC22	55264941
chr1	55264840	55264994	155	-1.29	0.00242	0.0191	TTC22	55264941
chr3	1.61E+08	1.61E+08	495	-0.91	0.00242	0.0191	SPTSSB	1.61E+08
chr10	1.28E+08	1.28E+08	214	-1.45	0.00242	0.0191	FANK1-AS1	1.28E+08
chr19	2427562	2427826	265	1.34	0.00243	0.0192	TIMM13	2425914
chr2	10592541	10592836	296	-0.95	0.00244	0.0192	ODC1	10586680
chr2	10592541	10592836	296	-0.95	0.00244	0.0192	ODC1	10585925
chr2	10592541	10592836	296	-0.95	0.00244	0.0192	ODC1	10585925
chr2	10592541	10592836	296	-0.95	0.00244	0.0192	ODC1	10586680
chr2	2.22E+08	2.22E+08	421	-0.9	0.00245	0.0193	EPHA4	2.22E+08
chr2	2.22E+08	2.22E+08	421	-0.9	0.00245	0.0193	EPHA4	2.22E+08
chr2	2.22E+08	2.22E+08	421	-0.9	0.00245	0.0193	EPHA4	2.22E+08
chr11	1.13E+08	1.13E+08	341	-0.67	0.00246	0.0194	ANKK1	1.13E+08
chr20	19865388	19865605	218	-1.08	0.0025	0.0196	RIN2	19865209
chr20	19865388	19865605	218	-1.08	0.0025	0.0196	RIN2	19862164
chr10	61725046	61725506	461	-0.87	0.00251	0.0196	LINC01553	61718671
chr1	1.13E+08	1.13E+08	517	-0.74	0.00252	0.0197	RHOC	1.13E+08
chr1	1.13E+08	1.13E+08	517	-0.74	0.00252	0.0197	RHOC	1.13E+08
chr1	1.13E+08	1.13E+08	517	-0.74	0.00252	0.0197	RHOC	1.13E+08
chr3	1.57E+08	1.57E+08	895	-0.67	0.00252	0.0197	CCNL1	1.57E+08
chr3	1.57E+08	1.57E+08	895	-0.67	0.00252	0.0197	CCNL1	1.57E+08
chr17	40610713	40610901	189	1.5	0.00254	0.0198	ATP6V0A1	40605861
chr17	40610713	40610901	189	1.5	0.00254	0.0198	ATP6V0A1	40605861
chr17	40610713	40610901	189	1.5	0.00254	0.0198	ATP6V0A1	40605861
chr3	1.52E+08	1.52E+08	235	-0.96	0.00255	0.0198	MBNL1	1.52E+08
chr3	1.52E+08	1.52E+08	235	-0.96	0.00255	0.0198	MBNL1	1.52E+08
chr3	1.52E+08	1.52E+08	235	-0.96	0.00255	0.0198	MBNL1	1.52E+08
chr9	1050150	1050429	280	-0.9	0.00259	0.02	DMRT2	1045353
chr9	1050150	1050429	280	-0.9	0.00259	0.02	DMRT2	1045345
chr9	1050150	1050429	280	-0.9	0.00259	0.02	DMRT2	1045619
chr10	92619325	92619531	207	-1.2	0.0026	0.0201	HTR7	92615671

chr10	92619325	92619531	207	-1.2	0.0026	0.0201	HTR7	92615671
chr10	92619325	92619531	207	-1.2	0.0026	0.0201	HTR7	92615671
chr12	56753863	56754380	518	0.69	0.00261	0.0202	STAT2	56752037
chr12	56753863	56754380	518	0.69	0.00261	0.0202	STAT2	56752037
chr6	1.49E+08	1.49E+08	1039	-0.71	0.00264	0.0203	LOC10537[illegible]	1.49E+08
chr4	15679381	15679545	165	-0.91	0.00267	0.0205	FAM200B	15678351
chr1	1.71E+08	1.71E+08	573	-1.16	0.00268	0.0205	PRRX1	1.71E+08
chr1	1.71E+08	1.71E+08	573	-1.16	0.00268	0.0205	PRRX1	1.71E+08
chr15	91564951	91565911	961	-0.86	0.00271	0.0207	VPS33B	91563851
chr15	91564951	91565911	961	-0.86	0.00271	0.0207	LOC10192[illegible]	91560851
chr15	91564951	91565911	961	-0.86	0.00271	0.0207	VPS33B	91563851
chr15	91564951	91565911	961	-0.86	0.00271	0.0207	VPS33B	91563851
chr10	65136808	65137059	252	-1.71	0.00272	0.0207	MIR1296	65130808
chr17	77813259	77813446	188	1.19	0.00275	0.0209	CBX4	77811213
chr17	79818365	79819124	760	0.74	0.00279	0.0211	P4HB	79816544
chr4	24586031	24586415	385	-0.9	0.0028	0.0212	DHX15	24584184
chr9	1.4E+08	1.4E+08	319	0.83	0.0028	0.0212	TRAF2	1.4E+08
chr9	1.4E+08	1.4E+08	319	0.83	0.0028	0.0212	MIR4479	1.4E+08
chr14	91724511	91724915	405	-1.04	0.00282	0.0213	GPR68	91718224
chr5	16508967	16509427	461	-0.84	0.00283	0.0213	FAM134B	16507107
chr3	1.39E+08	1.39E+08	419	-1.06	0.00285	0.0214	NMNAT3	1.39E+08
chr3	1.39E+08	1.39E+08	419	-1.06	0.00285	0.0214	NMNAT3	1.39E+08
chr2	19550051	19550349	299	-0.94	0.00286	0.0215	MIR4757	19543189
chr16	10832737	10832908	172	-1.66	0.00287	0.0215	NUBP1	10832642
chr16	10832737	10832908	172	-1.66	0.00287	0.0215	NUBP1	10832642
chr3	42304094	42304325	232	-1.36	0.00289	0.0216	CCK	42303439
chr11	1.22E+08	1.22E+08	417	-0.81	0.00291	0.0218	MIR100HG	1.22E+08
chr2	1.36E+08	1.36E+08	505	-0.68	0.00293	0.0219	UBXN4	1.36E+08
chr12	1.07E+08	1.07E+08	263	-1.02	0.00295	0.022	TMEM263	1.07E+08
chr17	47492185	47492485	301	0.83	0.003	0.0223	PHB	47490267
chr17	47492185	47492485	301	0.83	0.003	0.0223	PHB	47490267
chr17	47492185	47492485	301	0.83	0.003	0.0223	PHB	47490220
chr17	47492185	47492485	301	0.83	0.003	0.0223	PHB	47490267
chr6	1.67E+08	1.67E+08	753	-0.65	0.00304	0.0225	MPC1	1.67E+08
chr6	1.67E+08	1.67E+08	753	-0.65	0.00304	0.0225	MPC1	1.67E+08
chr6	1.67E+08	1.67E+08	753	-0.65	0.00304	0.0225	MPC1	1.67E+08
chr20	23117486	23117806	321	-0.82	0.00304	0.0225	LINC00656	23111273
chr4	1.47E+08	1.47E+08	728	-0.89	0.00311	0.0229	LSM6	1.47E+08
chr15	41709043	41709355	313	1.15	0.00311	0.0229	RTF1	41704301
chr11	1.1E+08	1.1E+08	771	-0.6	0.00313	0.023	ZC3H12C	1.1E+08
chr4	1.46E+08	1.46E+08	222	-1.24	0.00313	0.023	SMAD1-AS[illegible]	1.46E+08
chr17	78120978	78121458	481	0.94	0.00313	0.023	EIF4A3	78118982
chr19	49436779	49436954	176	1.22	0.00314	0.023	DHDH	49431938
chr16	68298041	68298504	464	0.9	0.00315	0.0231	SLC7A6	68293418
chr16	68298041	68298504	464	0.9	0.00315	0.0231	SLC7A6	68293418
chr3	1.46E+08	1.46E+08	115	-1.13	0.00316	0.0231	PLOD2	1.46E+08
chr3	1.46E+08	1.46E+08	115	-1.13	0.00316	0.0231	PLOD2	1.46E+08

chr17	36003434	36003612	179	1.2	0.00317	0.0231	DDX52	36001493
chr17	36003434	36003612	179	1.2	0.00317	0.0231	DDX52	36001493
chr3	1.19E+08	1.19E+08	854	-0.69	0.00318	0.0232	IGSF11	1.19E+08
chr14	94595872	94596166	295	-0.92	0.00319	0.0233	IFI27L2	94593957
chr18	32559104	32559418	315	-1.08	0.00321	0.0234	MAPRE2	32553207
chr20	20032934	20033523	590	-0.69	0.00324	0.0235	CFAP61	20028157
chr20	20032934	20033523	590	-0.69	0.00324	0.0235	CFAP61	20032289
chr20	20032934	20033523	590	-0.69	0.00324	0.0235	CRNKL1	20031136
chr2	67624249	67624528	280	-0.86	0.00324	0.0235	ETAA1	67619441
chr2	2.07E+08	2.07E+08	211	-1.13	0.00325	0.0236	NRP2	2.07E+08
chr2	2.07E+08	2.07E+08	211	-1.13	0.00325	0.0236	NRP2	2.07E+08
chr2	2.07E+08	2.07E+08	211	-1.13	0.00325	0.0236	NRP2	2.07E+08
chr2	2.07E+08	2.07E+08	211	-1.13	0.00325	0.0236	NRP2	2.07E+08
chr2	2.07E+08	2.07E+08	211	-1.13	0.00325	0.0236	NRP2	2.07E+08
chr2	2.07E+08	2.07E+08	211	-1.13	0.00325	0.0236	NRP2	2.07E+08
chr12	57482407	57482574	168	1.05	0.00325	0.0236	NAB2	57477676
chr2	66660569	66660857	289	-0.96	0.00326	0.0236	MEIS1	66657531
chr2	66660569	66660857	289	-0.96	0.00326	0.0236	MEIS1-AS3	66658602
chr11	1.25E+08	1.25E+08	251	-1.14	0.00326	0.0236	LOC403312	1.25E+08
chr11	1.25E+08	1.25E+08	251	-1.14	0.00326	0.0236	FEZ1	1.25E+08
chr11	1.25E+08	1.25E+08	251	-1.14	0.00326	0.0236	FEZ1	1.25E+08
chr17	7142811	7143194	384	0.77	0.00326	0.0236	PHF23	7140249
chr17	7142811	7143194	384	0.77	0.00326	0.0236	PHF23	7140825
chr17	7142811	7143194	384	0.77	0.00326	0.0236	PHF23	7140825
chr17	7142811	7143194	384	0.77	0.00326	0.0236	DVL2	7135863
chr3	40566135	40566547	413	-0.87	0.00331	0.0239	ZNF621	40561368
chr3	40566135	40566547	413	-0.87	0.00331	0.0239	ZNF621	40561368
chr3	40566135	40566547	413	-0.87	0.00331	0.0239	ZNF621	40561368
chr1	23810721	23811261	541	0.91	0.00332	0.0239	ASAP3	23808750
chr1	23810721	23811261	541	0.91	0.00332	0.0239	ASAP3	23808750
chr3	1.39E+08	1.39E+08	358	-1.04	0.00333	0.024	RBP1	1.39E+08
chr3	1.39E+08	1.39E+08	358	-1.04	0.00333	0.024	RBP1	1.39E+08
chr3	1.39E+08	1.39E+08	358	-1.04	0.00333	0.024	RBP1	1.39E+08
chr1	1.71E+08	1.71E+08	231	-0.98	0.00334	0.024	PRRC2C	1.71E+08
chr4	2420477	2420803	327	0.89	0.00335	0.024	ZFYVE28	2418370
chr4	2420477	2420803	327	0.89	0.00335	0.024	ZFYVE28	2418370
chr4	2420477	2420803	327	0.89	0.00335	0.024	ZFYVE28	2418370
chr4	2420477	2420803	327	0.89	0.00335	0.024	ZFYVE28	2418370
chr4	2420477	2420803	327	0.89	0.00335	0.024	CFAP99	2415671
chr10	1.23E+08	1.23E+08	397	-0.89	0.00335	0.024	WDR11-AS	1.23E+08
chr10	1.23E+08	1.23E+08	397	-0.89	0.00335	0.024	WDR11	1.23E+08
chr6	1.38E+08	1.38E+08	255	-1.72	0.00336	0.0241	LOC100507	1.38E+08
chr6	1.38E+08	1.38E+08	255	-1.72	0.00336	0.0241	LOC100507	1.38E+08
chr1	89530930	89531122	193	-1.17	0.00337	0.0241	GBP1	89529043
chrX	1.07E+08	1.07E+08	559	-1.11	0.00338	0.0242	LOC101928	1.07E+08
chr19	41256340	41257046	707	0.83	0.00339	0.0242	SNRPA	41251758
chr19	41256340	41257046	707	0.83	0.00339	0.0242	C19orf54	41253828

chr8	1.36E+08	1.36E+08	137	-1.38	0.00342	0.0244	MIR30B	1.36E+08
chr4	1.44E+08	1.44E+08	198	-1.39	0.00342	0.0244	GUSBP5	1.44E+08
chr15	45926856	45927648	793	-0.69	0.00343	0.0244	SQRDL	45921937
chr10	1.15E+08	1.15E+08	303	-0.78	0.00345	0.0245	CASP7	1.15E+08
chr10	1.15E+08	1.15E+08	303	-0.78	0.00345	0.0245	CASP7	1.15E+08
chr10	1.15E+08	1.15E+08	303	-0.78	0.00345	0.0245	CASP7	1.15E+08
chr10	1.15E+08	1.15E+08	303	-0.78	0.00345	0.0245	CASP7	1.15E+08
chr10	1.15E+08	1.15E+08	303	-0.78	0.00345	0.0245	CASP7	1.15E+08
chr10	1.15E+08	1.15E+08	303	-0.78	0.00345	0.0245	CASP7	1.15E+08
chr19	45542163	45542648	486	1.27	0.00345	0.0245	CLASRP	45537297
chr19	45542163	45542648	486	1.27	0.00345	0.0245	CLASRP	45537297
chr19	45542163	45542648	486	1.27	0.00345	0.0245	CLASRP	45537297
chr12	1.23E+08	1.23E+08	121	-2.16	0.00347	0.0246	LRRC43	1.23E+08
chr11	1.12E+08	1.12E+08	817	-0.67	0.0035	0.0248	PTS	1.12E+08
chr11	706974	707445	472	0.81	0.00354	0.025	EPS8L2	701119
chr15	77287330	77287511	182	-1.18	0.00355	0.025	PSTPIP1	77282464
chr15	45018909	45019034	126	-1.22	0.00356	0.0251	LOC100419	45016185
chr7	1.34E+08	1.34E+08	184	-3.16	0.00357	0.0252	AKR1B10	1.34E+08
chr16	81527334	81527659	326	-0.84	0.00358	0.0252	CMIP	81523953
chr20	1869918	1870156	239	-1.38	0.0036	0.0253	SIRPA	1869812
chr6	1.16E+08	1.16E+08	601	-0.82	0.00361	0.0253	FRK	1.16E+08
chr16	2205418	2205758	341	0.82	0.00362	0.0254	SNORD60	2203106
chr16	2205418	2205758	341	0.82	0.00362	0.0254	TRAF7	2200798
chr16	2205418	2205758	341	0.82	0.00362	0.0254	SNHG19	2203359
chr3	1.84E+08	1.84E+08	146	-1.18	0.00366	0.0256	FAM131A	1.84E+08
chr3	1.84E+08	1.84E+08	146	-1.18	0.00366	0.0256	FAM131A	1.84E+08
chr12	97300809	97301458	650	-0.76	0.00369	0.0257	NEDD1	97296243
chr12	97300809	97301458	650	-0.76	0.00369	0.0257	NEDD1	97296000
chr12	97300809	97301458	650	-0.76	0.00369	0.0257	NEDD1	97296000
chr12	97300809	97301458	650	-0.76	0.00369	0.0257	NEDD1	97296000
chr17	79869252	79869575	324	0.88	0.0037	0.0258	PCYT2	79867353
chr17	79869252	79869575	324	0.88	0.0037	0.0258	PCYT2	79867353
chr17	79869252	79869575	324	0.88	0.0037	0.0258	PCYT2	79867353
chr17	79869252	79869575	324	0.88	0.0037	0.0258	PCYT2	79867353
chr17	79869252	79869575	324	0.88	0.0037	0.0258	PCYT2	79867353
chr17	79869252	79869575	324	0.88	0.0037	0.0258	PCYT2	79867115
chr17	79869252	79869575	324	0.88	0.0037	0.0258	PCYT2	79867115
chr15	86313704	86313907	204	-1.21	0.00371	0.0259	MIR1276	86311809
chr1	86620414	86620547	134	-1.45	0.00373	0.0259	COL24A1	86620121
chr22	39898208	39898382	175	1.74	0.00375	0.026	MIEF1	39893234
chr22	39898208	39898382	175	1.74	0.00375	0.026	MIEF1	39893234
chr22	39898208	39898382	175	1.74	0.00375	0.026	MIEF1	39893234
chr11	36531497	36532225	729	-0.78	0.00378	0.0262	TRAF6	36529863
chr11	36531497	36532225	729	-0.78	0.00378	0.0262	TRAF6	36529863
chr6	1.53E+08	1.53E+08	321	-1.11	0.0038	0.0263	SYNE1-AS1	1.53E+08
chr20	33463781	33464325	545	0.72	0.00383	0.0264	ACSS2	33457765
chr20	33463781	33464325	545	0.72	0.00383	0.0264	ACSS2	33459327

chr20	33463781	33464325	545	0.72	0.00383	0.0264	GGT7	33458661
chr20	33463781	33464325	545	0.72	0.00383	0.0264	ACSS2	33459327
chr1	1.59E+08	1.59E+08	202	-1.58	0.00384	0.0265	AIM2	1.59E+08
chr6	1.5E+08	1.5E+08	113	-1.43	0.00385	0.0265	RAET1E-AS	1.5E+08
chr12	1.07E+08	1.07E+08	239	-0.85	0.00388	0.0267	CRY1	1.07E+08
chr10	43892427	43892919	493	-0.79	0.00391	0.0269	HNRNPF	43890279
chr10	43892427	43892919	493	-0.79	0.00391	0.0269	HNRNPF	43890794
chr10	6821050	6821266	217	-1.13	0.00394	0.027	LINC00707	6816559
chr10	6821050	6821266	217	-1.13	0.00394	0.027	LINC00706	6819142
chr2	14774518	14774813	296	-0.84	0.00394	0.027	FAM84A	14767809
chr2	14774518	14774813	296	-0.84	0.00394	0.027	LOC65360	14770214
chr19	8070528	8070640	113	1.67	0.00395	0.0271	ELAVL1	8068529
chr12	1.15E+08	1.15E+08	694	-0.68	0.00396	0.0272	TBX3	1.15E+08
chr12	1.15E+08	1.15E+08	694	-0.68	0.00396	0.0272	TBX3	1.15E+08
chr10	90640402	90640764	363	-0.79	0.00397	0.0272	STAMBPL1	90634943
chr9	1.39E+08	1.39E+08	519	0.96	0.00398	0.0272	MIR4674	1.39E+08
chr9	1.39E+08	1.39E+08	519	0.96	0.00398	0.0272	NALT1	1.39E+08
chr9	1.39E+08	1.39E+08	519	0.96	0.00398	0.0272	NOTCH1	1.39E+08
chr19	12847722	12848115	394	0.78	0.00398	0.0273	C19orf43	12843529
chr19	12847722	12848115	394	0.78	0.00398	0.0273	ASNA1	12843305
chr3	33839960	33840752	793	-0.72	0.00404	0.0275	PDCD6IP	33835062
chr3	33839960	33840752	793	-0.72	0.00404	0.0275	PDCD6IP	33835062
chr3	33839960	33840752	793	-0.72	0.00404	0.0275	PDCD6IP	33835062
chr14	97263470	97263794	325	-0.74	0.00406	0.0276	VRK1	97258683
chr3	1.92E+08	1.92E+08	364	-1.02	0.00408	0.0277	FGF12	1.92E+08
chr16	67240765	67241147	383	-0.96	0.0041	0.0278	MIR328	67234298
chr3	1.34E+08	1.34E+08	795	-0.72	0.00411	0.0278	CEP63	1.34E+08
chr3	1.34E+08	1.34E+08	795	-0.72	0.00411	0.0278	CEP63	1.34E+08
chr3	1.34E+08	1.34E+08	795	-0.72	0.00411	0.0278	CEP63	1.34E+08
chr3	1.34E+08	1.34E+08	795	-0.72	0.00411	0.0278	CEP63	1.34E+08
chr3	1.34E+08	1.34E+08	795	-0.72	0.00411	0.0278	ANAPC13	1.34E+08
chr3	1.34E+08	1.34E+08	795	-0.72	0.00411	0.0278	ANAPC13	1.34E+08
chr3	1.34E+08	1.34E+08	795	-0.72	0.00411	0.0278	ANAPC13	1.34E+08
chr1	2.42E+08	2.42E+08	349	-0.9	0.00411	0.0278	MAP1LC3C	2.42E+08
chr5	14871796	14872124	329	-0.97	0.00414	0.0279	ANKH	14869887
chr19	2900644	2901059	416	0.87	0.00418	0.0281	ZNF57	2895895
chr17	43138193	43138981	789	0.67	0.00421	0.0283	DCAKD	43136477
chr17	43138193	43138981	789	0.67	0.00421	0.0283	DCAKD	43136477
chr17	43138193	43138981	789	0.67	0.00421	0.0283	NMT1	43133666
chr6	75915558	75915988	431	-0.72	0.00423	0.0284	COL12A1	75913623
chr6	75915558	75915988	431	-0.72	0.00423	0.0284	COL12A1	75913623
chr8	97657322	97657891	570	-0.74	0.00425	0.0285	CPQ	97652454
chr20	18568432	18569078	647	-0.8	0.00432	0.0289	DTD1	18563536
chr20	18568432	18569078	647	-0.8	0.00432	0.0289	DTD1	18563536
chr3	69135411	69135889	479	-0.73	0.00436	0.029	ARL6IP5	69129089
chr17	1945010	1945372	363	0.96	0.00436	0.029	OVCA2	1940276
chr16	86609442	86609619	178	-1.21	0.00437	0.029	FOXL1	86607114

chr2	18059702	18060221	520	-0.91	0.00439	0.0292	KCNS3	18054923
chr2	18059702	18060221	520	-0.91	0.00439	0.0292	KCNS3	18054113
chr7	1.5E+08	1.5E+08	265	-0.97	0.00439	0.0292	ATP6V0E2	1.5E+08
chr7	1.5E+08	1.5E+08	265	-0.97	0.00439	0.0292	ATP6V0E2	1.5E+08
chr7	1.5E+08	1.5E+08	265	-0.97	0.00439	0.0292	ATP6V0E2	1.5E+08
chr7	1.5E+08	1.5E+08	265	-0.97	0.00439	0.0292	ATP6V0E2	1.5E+08
chr7	1.5E+08	1.5E+08	265	-0.97	0.00439	0.0292	ATP6V0E2-	1.5E+08
chr4	1.39E+08	1.39E+08	339	-0.88	0.0044	0.0292	SLC7A11	1.39E+08
chr10	97849472	97850142	671	-0.76	0.00441	0.0292	ENTPD1-AS	97847992
chr10	97849472	97850142	671	-0.76	0.00441	0.0292	ENTPD1-AS	97847992
chr10	97849472	97850142	671	-0.76	0.00441	0.0292	ENTPD1-AS	97847992
chr10	97849472	97850142	671	-0.76	0.00441	0.0292	ENTPD1-AS	97847992
chr8	1.24E+08	1.24E+08	286	-0.8	0.00447	0.0295	FAM83A	1.24E+08
chr8	1.24E+08	1.24E+08	286	-0.8	0.00447	0.0295	FAM83A	1.24E+08
chr10	38265179	38265860	682	-0.72	0.00447	0.0295	ZNF25	38263453
chr6	1.34E+08	1.34E+08	247	-0.88	0.00448	0.0296	SGK1	1.34E+08
chr6	1.34E+08	1.34E+08	247	-0.88	0.00448	0.0296	SGK1	1.34E+08
chr6	1.34E+08	1.34E+08	247	-0.88	0.00448	0.0296	SGK1	1.34E+08
chr6	1.34E+08	1.34E+08	247	-0.88	0.00448	0.0296	SGK1	1.34E+08
chr7	33080464	33080697	234	-1.05	0.00449	0.0296	NT5C3A	33078777
chr7	33080464	33080697	234	-1.05	0.00449	0.0296	NT5C3A	33078777
chr22	19434888	19435384	497	0.83	0.0045	0.0297	C22orf39	19433755
chr22	19434888	19435384	497	0.83	0.0045	0.0297	C22orf39	19433755
chr17	62207231	62207773	543	0.79	0.00456	0.03	ERN1	62205502
chr11	18655844	18656424	581	-0.72	0.0046	0.0301	SPTY2D1	18654020
chr18	54318281	54318742	462	-0.9	0.00461	0.0302	WDR7	54313615
chr18	54318281	54318742	462	-0.9	0.00461	0.0302	WDR7	54313615
chr1	89488475	89488583	109	-1.42	0.00461	0.0302	GBP3	89486549
chr8	1.09E+08	1.09E+08	478	-1.1	0.00463	0.0302	ANGPT1	1.09E+08
chr8	1.09E+08	1.09E+08	478	-1.1	0.00463	0.0302	ANGPT1	1.09E+08
chr19	1812485	1813004	520	0.85	0.00466	0.0305	ATP8B3	1810236
chr19	1812485	1813004	520	0.85	0.00466	0.0305	ATP8B3	1810275
chr19	1812485	1813004	520	0.85	0.00466	0.0305	ATP8B3	1810275
chr12	39298699	39299010	312	-0.87	0.00473	0.0307	CPNE8	39297420
chr8	1.41E+08	1.41E+08	277	-0.78	0.00475	0.0308	KCNK9	1.41E+08
chr8	1.41E+08	1.41E+08	277	-0.78	0.00475	0.0308	KCNK9	1.41E+08
chr4	56261736	56262189	454	-0.92	0.00475	0.0309	TMEM165	56257079
chr4	56261736	56262189	454	-0.92	0.00475	0.0309	TMEM165	56257079
chr3	1.6E+08	1.6E+08	1321	-0.58	0.00479	0.0311	KPNA4	1.6E+08
chr22	30819919	30820298	380	0.7	0.00485	0.0314	MTFP1	30816610
chr22	30819919	30820298	380	0.7	0.00485	0.0314	MTFP1	30816610
chr9	1.4E+08	1.4E+08	617	0.7	0.00485	0.0314	SSNA1	1.4E+08
chr9	1.4E+08	1.4E+08	617	0.7	0.00485	0.0314	ANAPC2	1.4E+08
chr8	72274019	72274415	397	-1.15	0.00486	0.0314	EYA1	72272467
chr8	72274019	72274415	397	-1.15	0.00486	0.0314	EYA1	72272467
chr8	72274019	72274415	397	-1.15	0.00486	0.0314	EYA1	72272467
chr7	65540643	65540952	310	0.8	0.0049	0.0315	ASL	65535775

chr7	65540643	65540952	310	0.8	0.0049	0.0315	ASL	65535833
chr7	65540643	65540952	310	0.8	0.0049	0.0315	ASL	65535833
chr7	65540643	65540952	310	0.8	0.0049	0.0315	ASL	65535833
chr15	82554890	82555282	393	-0.77	0.00491	0.0316	SAXO2	82550151
chr15	82554890	82555282	393	-0.77	0.00491	0.0316	EFL1	82553104
chr15	82554890	82555282	393	-0.77	0.00491	0.0316	EFL1	82553104
chr16	53086466	53086858	393	-0.68	0.00496	0.0318	CHD9	53083944
chr22	30278984	30279719	736	0.81	0.00496	0.0318	MTMR3	30274157
chr22	30278984	30279719	736	0.81	0.00496	0.0318	MTMR3	30274157
chr22	30278984	30279719	736	0.81	0.00496	0.0318	MTMR3	30274157
chr9	1.39E+08	1.39E+08	344	0.77	0.00501	0.032	SEC16A	1.39E+08
chr9	1.39E+08	1.39E+08	344	0.77	0.00501	0.032	C9orf163	1.39E+08
chr9	1.39E+08	1.39E+08	344	0.77	0.00501	0.032	SEC16A	1.39E+08
chr1	2.24E+08	2.24E+08	641	-0.74	0.00502	0.0321	CAPN2	2.24E+08
chr16	53407297	53407863	567	-0.93	0.00503	0.0321	LOC64380	53403041
chr16	53407297	53407863	567	-0.93	0.00503	0.0321	LOC10272	53402404
chr5	36151924	36152321	398	-0.68	0.00504	0.0322	SKP2	36147144
chr5	36151924	36152321	398	-0.68	0.00504	0.0322	SKP2	36147144
chr5	36151924	36152321	398	-0.68	0.00504	0.0322	SKP2	36147144
chr5	36151924	36152321	398	-0.68	0.00504	0.0322	LMBRD2	36150015
chr5	36151924	36152321	398	-0.68	0.00504	0.0322	MIR580	36146090
chr9	90477588	90477739	152	-1.58	0.00507	0.0323	LOC39236	90471971
chr11	6502407	6502790	384	-0.73	0.00508	0.0323	ARFIP2	6500709
chr11	6502407	6502790	384	-0.73	0.00508	0.0323	ARFIP2	6500709
chr11	6502407	6502790	384	-0.73	0.00508	0.0323	TIMM10B	6497676
chr11	6502407	6502790	384	-0.73	0.00508	0.0323	ARFIP2	6500709
chr11	6502407	6502790	384	-0.73	0.00508	0.0323	ARFIP2	6500709
chr17	74260952	74261372	421	0.67	0.00508	0.0323	UBALD2	74256285
chr5	60457961	60458467	507	-0.83	0.00509	0.0323	SMIM15	60456302
chr5	60457961	60458467	507	-0.83	0.00509	0.0323	CTC-436P1	60453142
chr1	1.83E+08	1.83E+08	556	-0.74	0.00509	0.0323	RNASEL	1.83E+08
chr6	42946813	42947033	221	1.15	0.00509	0.0323	PEX6	42944981
chr6	42946813	42947033	221	1.15	0.00509	0.0323	PEX6	42944981
chr6	42946813	42947033	221	1.15	0.00509	0.0323	PEX6	42944981
chr2	1.77E+08	1.77E+08	254	-0.8	0.0051	0.0324	HOXD11	1.77E+08
chr8	1.28E+08	1.28E+08	1030	-0.63	0.00511	0.0324	FAM84B	1.28E+08
chr9	1.18E+08	1.18E+08	212	-0.96	0.00511	0.0324	TNFSF15	1.18E+08
chr11	1.24E+08	1.24E+08	260	-0.96	0.00513	0.0325	VWA5A	1.24E+08
chr11	1.24E+08	1.24E+08	260	-0.96	0.00513	0.0325	VWA5A	1.24E+08
chr11	1.24E+08	1.24E+08	260	-0.96	0.00513	0.0325	VWA5A	1.24E+08
chr6	18264532	18265114	583	-0.75	0.00513	0.0325	DEK	18262799
chr6	18264532	18265114	583	-0.75	0.00513	0.0325	DEK	18262799
chr15	92396034	92396900	867	-0.63	0.00514	0.0325	SLCO3A1	92391937
chr15	92396034	92396900	867	-0.63	0.00514	0.0325	SLCO3A1	92391937
chr1	1.13E+08	1.13E+08	432	-0.78	0.00515	0.0326	CTTNBP2N	1.13E+08
chr2	96812800	96812962	163	1.26	0.00521	0.0329	DUSP2	96809179
chr6	1.67E+08	1.67E+08	538	-0.64	0.00524	0.033	RPS6KA2	1.67E+08

chr19	6737618	6737918	301	0.87	0.0053	0.0333	GPR108	6735633
chr19	6737618	6737918	301	0.87	0.0053	0.0333	TRIP10	6734692
chr19	6737618	6737918	301	0.87	0.0053	0.0333	TRIP10	6734692
chr19	6737618	6737918	301	0.87	0.0053	0.0333	TRIP10	6734692
chr19	6737618	6737918	301	0.87	0.0053	0.0333	TRIP10	6734692
chr19	6737618	6737918	301	0.87	0.0053	0.0333	MIR6791	6734789
chr10	43048026	43048479	454	-0.71	0.0053	0.0333	ZNF37BP	43046318
chr6	1.5E+08	1.5E+08	167	-1.48	0.00532	0.0333	RAET1E	1.5E+08
chr15	40635794	40635976	183	-1.02	0.00532	0.0334	C15orf52	40631168
chr4	1.44E+08	1.44E+08	710	-0.62	0.00538	0.0337	SMARCA5	1.44E+08
chr4	1.44E+08	1.44E+08	710	-0.62	0.00538	0.0337	SMARCA5-/	1.44E+08
chr2	1.62E+08	1.62E+08	542	-0.87	0.0054	0.0338	TANK	1.62E+08
chr2	1.62E+08	1.62E+08	542	-0.87	0.0054	0.0338	TANK	1.62E+08
chr6	1.26E+08	1.26E+08	697	-0.72	0.00542	0.0338	HDDC2	1.26E+08
chr12	1.07E+08	1.07E+08	545	-0.72	0.00542	0.0338	TMEM263	1.07E+08
chr5	1.39E+08	1.39E+08	217	-1.09	0.00543	0.0339	NRG2	1.39E+08
chr5	1.39E+08	1.39E+08	217	-1.09	0.00543	0.0339	NRG2	1.39E+08
chr5	1.39E+08	1.39E+08	217	-1.09	0.00543	0.0339	NRG2	1.39E+08
chr5	1.39E+08	1.39E+08	217	-1.09	0.00543	0.0339	NRG2	1.39E+08
chr5	1.39E+08	1.39E+08	217	-1.09	0.00543	0.0339	NRG2	1.39E+08
chr15	39871932	39872119	188	-1.01	0.00544	0.0339	THBS1	39868279
chr17	7819106	7819282	177	1.04	0.00548	0.0341	LOC28402З	7817265
chr11	1.29E+08	1.29E+08	354	-0.97	0.00548	0.0341	ARHGAP32	1.29E+08
chr16	1661989	1662314	326	0.97	0.0055	0.0342	IFT140	1660109
chr16	1661989	1662314	326	0.97	0.0055	0.0342	CRAMP1	1659640
chr22	41985727	41986198	472	0.84	0.00553	0.0343	PMM1	41983871
chr5	35229562	35229813	252	-1.28	0.00553	0.0344	PRLR	35228691
chr1	89487502	89487722	221	-0.96	0.00555	0.0345	GBP3	89486549
chr16	46607375	46607806	432	-0.7	0.00556	0.0345	ANKRD26P	46601009
chr7	1.51E+08	1.51E+08	424	-0.72	0.00558	0.0346	CDK5	1.51E+08
chr7	1.51E+08	1.51E+08	424	-0.72	0.00558	0.0346	SLC4A2	1.51E+08
chr7	1.51E+08	1.51E+08	424	-0.72	0.00558	0.0346	SLC4A2	1.51E+08
chr7	1.51E+08	1.51E+08	424	-0.72	0.00558	0.0346	SLC4A2	1.51E+08
chr7	1.51E+08	1.51E+08	424	-0.72	0.00558	0.0346	SLC4A2	1.51E+08
chr7	1.51E+08	1.51E+08	424	-0.72	0.00558	0.0346	CDK5	1.51E+08
chr1	1.79E+08	1.79E+08	536	-0.74	0.00566	0.0349	C1orf220	1.79E+08
chr17	80231376	80231769	394	1.09	0.00566	0.0349	CSNK1D	80229594
chr17	80231376	80231769	394	1.09	0.00566	0.0349	CSNK1D	80229594
chr17	80231376	80231769	394	1.09	0.00566	0.0349	CSNK1D	80229618
chr11	1.11E+08	1.11E+08	223	-1.05	0.00568	0.035	C11orf88	1.11E+08
chr11	1.11E+08	1.11E+08	223	-1.05	0.00568	0.035	C11orf88	1.11E+08
chr11	1.11E+08	1.11E+08	223	-1.05	0.00568	0.035	MIR34B	1.11E+08
chr11	1.11E+08	1.11E+08	223	-1.05	0.00568	0.035	MIR34C	1.11E+08
chr11	1.11E+08	1.11E+08	223	-1.05	0.00568	0.035	BTG4	1.11E+08
chr4	18023288	18024029	742	-0.7	0.00569	0.0351	LCORL	18021483
chr4	18023288	18024029	742	-0.7	0.00569	0.0351	LCORL	18021483
chr16	88636537	88636877	341	0.9	0.00569	0.0351	ZC3H18	88631788

chr16	88636537	88636877	341	0.9	0.00569	0.0351	ZC3H18	88631788
chr1	1.84E+08	1.84E+08	705	-0.72	0.00574	0.0353	TSEN15	1.84E+08
chr1	1.84E+08	1.84E+08	705	-0.72	0.00574	0.0353	TSEN15	1.84E+08
chr1	1.84E+08	1.84E+08	705	-0.72	0.00574	0.0353	TSEN15	1.84E+08
chr1	1.84E+08	1.84E+08	705	-0.72	0.00574	0.0353	TSEN15	1.84E+08
chr1	1.84E+08	1.84E+08	705	-0.72	0.00574	0.0353	TSEN15	1.84E+08
chr1	1.84E+08	1.84E+08	705	-0.72	0.00574	0.0353	TSEN15	1.84E+08
chr17	41150107	41150523	417	0.79	0.0058	0.0356	RPL27	41145445
chr14	96672164	96672364	201	-1.17	0.00581	0.0356	BDKRB2	96666134
chr10	99894199	99894846	648	-0.73	0.00582	0.0357	R3HCC1L	99889380
chr10	99894199	99894846	648	-0.73	0.00582	0.0357	R3HCC1L	99889380
chr10	99894199	99894846	648	-0.73	0.00582	0.0357	R3HCC1L	99889380
chr10	99894199	99894846	648	-0.73	0.00582	0.0357	R3HCC1L	99889380
chr10	99894199	99894846	648	-0.73	0.00582	0.0357	R3HCC1L	99889380
chr9	1.37E+08	1.37E+08	369	0.94	0.00585	0.0358	LINC00094	1.37E+08
chr8	67976331	67976760	430	-0.7	0.00585	0.0358	CSPP1	67971602
chr8	67976331	67976760	430	-0.7	0.00585	0.0358	COPS5	67972562
chr6	1.55E+08	1.55E+08	383	0.99	0.00587	0.0358	TIAM2	1.55E+08
chr20	43988565	43988698	134	-1.09	0.00587	0.0358	SYS1	43985576
chr20	43988565	43988698	134	-1.09	0.00587	0.0358	SYS1	43986739
chr20	43988565	43988698	134	-1.09	0.00587	0.0358	SYS1-DBND	43986808
chr20	43988565	43988698	134	-1.09	0.00587	0.0358	SYS1	43986739
chr15	89455121	89455332	212	-0.92	0.00587	0.0358	MFGE8	89454700
chr15	89455121	89455332	212	-0.92	0.00587	0.0358	MFGE8	89453993
chr15	89455121	89455332	212	-0.92	0.00587	0.0358	MFGE8	89454700
chr15	89455121	89455332	212	-0.92	0.00587	0.0358	MFGE8	89454700
chr15	89455121	89455332	212	-0.92	0.00587	0.0358	MFGE8	89454700
chr16	54962902	54963344	443	-0.74	0.00587	0.0358	IRX5	54960110
chr16	54962902	54963344	443	-0.74	0.00587	0.0358	IRX5	54960110
chr16	54962902	54963344	443	-0.74	0.00587	0.0358	CRNDE	54960690
chr16	54962902	54963344	443	-0.74	0.00587	0.0358	CRNDE	54961101
chr16	54962902	54963344	443	-0.74	0.00587	0.0358	CRNDE	54960690
chr16	54962902	54963344	443	-0.74	0.00587	0.0358	CRNDE	54960690
chr16	54962902	54963344	443	-0.74	0.00587	0.0358	CRNDE	54961101
chr11	1.02E+08	1.02E+08	403	-0.81	0.00589	0.0359	BIRC3	1.02E+08
chr11	1.02E+08	1.02E+08	403	-0.81	0.00589	0.0359	BIRC3	1.02E+08
chr11	93474577	93475192	616	-0.64	0.00591	0.036	C11orf54	93469759
chr11	93474577	93475192	616	-0.64	0.00591	0.036	TAF1D	93472703
chr11	93474577	93475192	616	-0.64	0.00591	0.036	C11orf54	93469759
chr11	93474577	93475192	616	-0.64	0.00591	0.036	C11orf54	93469759
chr11	93474577	93475192	616	-0.64	0.00591	0.036	C11orf54	93469759
chr11	93474577	93475192	616	-0.64	0.00591	0.036	C11orf54	93469759
chr11	93474577	93475192	616	-0.64	0.00591	0.036	C11orf54	93469759
chr17	77889972	77890122	151	1.01	0.00592	0.036	LOC10192ɛ	77888155
chr6	1.67E+08	1.67E+08	270	-0.86	0.00592	0.036	MPC1	1.67E+08
chr6	1.67E+08	1.67E+08	270	-0.86	0.00592	0.036	MPC1	1.67E+08
chr6	1.67E+08	1.67E+08	270	-0.86	0.00592	0.036	MPC1	1.67E+08

chr10	1.15E+08	1.15E+08	481	-0.73	0.00593	0.0361	TCF7L2	1.15E+08
chr10	1.15E+08	1.15E+08	481	-0.73	0.00593	0.0361	TCF7L2	1.15E+08
chr10	1.15E+08	1.15E+08	481	-0.73	0.00593	0.0361	TCF7L2	1.15E+08
chr10	1.15E+08	1.15E+08	481	-0.73	0.00593	0.0361	TCF7L2	1.15E+08
chr10	1.15E+08	1.15E+08	481	-0.73	0.00593	0.0361	TCF7L2	1.15E+08
chr10	1.15E+08	1.15E+08	481	-0.73	0.00593	0.0361	TCF7L2	1.15E+08
chr10	1.15E+08	1.15E+08	481	-0.73	0.00593	0.0361	TCF7L2	1.15E+08
chr10	1.15E+08	1.15E+08	481	-0.73	0.00593	0.0361	TCF7L2	1.15E+08
chr10	1.15E+08	1.15E+08	481	-0.73	0.00593	0.0361	TCF7L2	1.15E+08
chr10	1.15E+08	1.15E+08	481	-0.73	0.00593	0.0361	TCF7L2	1.15E+08
chr10	1.15E+08	1.15E+08	481	-0.73	0.00593	0.0361	TCF7L2	1.15E+08
chr10	1.15E+08	1.15E+08	481	-0.73	0.00593	0.0361	TCF7L2	1.15E+08
chr10	1.15E+08	1.15E+08	481	-0.73	0.00593	0.0361	TCF7L2	1.15E+08
chr3	1.9E+08	1.9E+08	113	-1.3	0.00595	0.0361	CLDN1	1.9E+08
chr6	39895045	39895147	103	-1.19	0.00598	0.0363	MOCS1	39893455
chr12	95012867	95013021	155	-1.14	0.00599	0.0363	TMCC3	95007927
chr1	3615588	3615913	326	-0.89	0.00601	0.0364	TP73	3609609
chr6	35656608	35656911	304	0.85	0.00603	0.0365	FKBP5	35654719
chr6	35656608	35656911	304	0.85	0.00603	0.0365	FKBP5	35654692
chr6	35656608	35656911	304	0.85	0.00603	0.0365	FKBP5	35654719
chr7	42276727	42276991	265	-1.1	0.00603	0.0365	GLI3	42274618
chr1	26232812	26233031	220	1.22	0.00603	0.0365	STMN1	26230993
chr1	26232812	26233031	220	1.22	0.00603	0.0365	STMN1	26231368
chr1	26232812	26233031	220	1.22	0.00603	0.0365	STMN1	26230644
chr1	26232812	26233031	220	1.22	0.00603	0.0365	MIR3917	26230945
chr1	26232812	26233031	220	1.22	0.00603	0.0365	STMN1	26230993
chr15	79296782	79297090	309	-0.89	0.00604	0.0365	RASGRF1	79296002
chr3	1.23E+08	1.23E+08	217	-1.03	0.00606	0.0366	DIRC2	1.23E+08
chr3	1.23E+08	1.23E+08	217	-1.03	0.00606	0.0366	HSPBAP1	1.23E+08
chr17	27717179	27717490	312	0.9	0.00606	0.0366	TAOK1	27712942
chr17	27717179	27717490	312	0.9	0.00606	0.0366	TAOK1	27712942
chr17	27717179	27717490	312	0.9	0.00606	0.0366	MIR4523	27712679
chr10	93373528	93373700	173	-1.21	0.00612	0.0368	HECTD2-AS	93369217
chr7	1.4E+08	1.4E+08	936	-0.63	0.00612	0.0368	NDUFB2-AS	1.4E+08
chr7	1.4E+08	1.4E+08	936	-0.63	0.00612	0.0368	NDUFB2	1.4E+08
chr3	1.41E+08	1.41E+08	564	-0.57	0.00612	0.0368	SLC25A36	1.41E+08
chr3	1.41E+08	1.41E+08	564	-0.57	0.00612	0.0368	SLC25A36	1.41E+08
chr6	1.07E+08	1.07E+08	304	-0.88	0.00614	0.0369	PRDM1	1.07E+08
chr10	1.32E+08	1.32E+08	1236	-0.62	0.00616	0.037	LINC00959	1.32E+08
chr11	88071592	88071777	186	-1.01	0.00617	0.037	CTSC	88068955
chr11	88071592	88071777	186	-1.01	0.00617	0.037	CTSC	88068955
chr11	88071592	88071777	186	-1.01	0.00617	0.037	CTSC	88068955
chr4	1.03E+08	1.03E+08	455	-1	0.00618	0.0371	BANK1	1.03E+08
chr4	1.03E+08	1.03E+08	455	-1	0.00618	0.0371	BANK1	1.03E+08
chr8	66556643	66557540	898	-0.61	0.00624	0.0374	MTFR1	66551887
chr8	66556643	66557540	898	-0.61	0.00624	0.0374	MTFR1	66551887
chr17	47865836	47866744	909	0.62	0.00626	0.0375	KAT7	47860980

chr17	47865836	47866744	909	0.62	0.00626	0.0375	KAT7	47860980
chr17	47865836	47866744	909	0.62	0.00626	0.0375	KAT7	47860980
chr17	47865836	47866744	909	0.62	0.00626	0.0375	KAT7	47860980
chr17	47865836	47866744	909	0.62	0.00626	0.0375	KAT7	47860980
chr17	1587819	1588403	585	0.8	0.00627	0.0375	PRPF8	1586176
chr14	50100792	50101297	506	-0.73	0.00628	0.0376	DNAAF2	50099948
chr14	50100792	50101297	506	-0.73	0.00628	0.0376	DNAAF2	50099948
chr3	1.79E+08	1.79E+08	542	-0.61	0.00628	0.0376	MRPL47	1.79E+08
chr3	1.79E+08	1.79E+08	542	-0.61	0.00628	0.0376	MRPL47	1.79E+08
chr3	1.79E+08	1.79E+08	542	-0.61	0.00628	0.0376	NDUFB5	1.79E+08
chr3	1.79E+08	1.79E+08	542	-0.61	0.00628	0.0376	NDUFB5	1.79E+08
chr3	1.79E+08	1.79E+08	542	-0.61	0.00628	0.0376	NDUFB5	1.79E+08
chr2	2.31E+08	2.31E+08	266	-0.98	0.00629	0.0376	SP140L	2.31E+08
chr2	2.31E+08	2.31E+08	266	-0.98	0.00629	0.0376	SP140L	2.31E+08
chr2	2.31E+08	2.31E+08	266	-0.98	0.00629	0.0376	SP140L	2.31E+08
chr3	1.84E+08	1.84E+08	406	0.67	0.00629	0.0376	AP2M1	1.84E+08
chr3	1.84E+08	1.84E+08	406	0.67	0.00629	0.0376	AP2M1	1.84E+08
chr3	1.84E+08	1.84E+08	406	0.67	0.00629	0.0376	AP2M1	1.84E+08
chr3	1.89E+08	1.89E+08	257	-0.94	0.00629	0.0376	TPRG1	1.89E+08
chr1	1.53E+08	1.53E+08	175	-1.1	0.00631	0.0377	IVL	1.53E+08
chr11	1.22E+08	1.22E+08	229	-1.24	0.00636	0.0379	MIRLET7A2	1.22E+08
chr1	63832883	63833658	776	-0.55	0.00638	0.038	ALG6	63828260
chr1	2.37E+08	2.37E+08	571	-0.81	0.00641	0.0381	EDARADD	2.37E+08
chr1	2.37E+08	2.37E+08	571	-0.81	0.00641	0.0381	EDARADD	2.37E+08
chr20	18477518	18478004	487	-0.82	0.00642	0.0381	RBBP9	18475887
chr17	37026064	37026303	240	0.97	0.00647	0.0383	LASP1	37021111
chr17	37026064	37026303	240	0.97	0.00647	0.0383	LASP1	37021111
chr17	37026064	37026303	240	0.97	0.00647	0.0383	LASP1	37021111
chr10	1.3E+08	1.3E+08	265	-0.79	0.00648	0.0383	PTPRE	1.3E+08
chr10	1.3E+08	1.3E+08	265	-0.79	0.00648	0.0383	PTPRE	1.3E+08
chr1	1.65E+08	1.65E+08	337	-0.72	0.00652	0.0385	LOC100505	1.65E+08
chr9	95736540	95736905	366	1.03	0.00657	0.0387	FGD3	95731717
chr3	39447911	39448402	492	-0.68	0.00657	0.0387	SNORA62	39447544
chr3	39447911	39448402	492	-0.68	0.00657	0.0387	RPSA	39443179
chr3	39447911	39448402	492	-0.68	0.00657	0.0387	RPSA	39443179
chr3	39447911	39448402	492	-0.68	0.00657	0.0387	SNORA6	39444881
chr12	27234677	27234923	247	-0.88	0.00659	0.0388	C12orf71	27233455
chr1	2.05E+08	2.05E+08	386	-1.05	0.00665	0.039	MIR135B	2.05E+08
chr14	62217670	62218084	415	-0.78	0.00666	0.0391	HIF1A-AS2	62213807
chr11	6628261	6628445	185	-0.84	0.00667	0.0391	RRP8	6622880
chr7	17409509	17409893	385	-0.99	0.00668	0.0391	KCCAT333	17409540
chr7	17409509	17409893	385	-0.99	0.00668	0.0391	KCCAT333	17409540
chr14	1.04E+08	1.04E+08	316	0.97	0.00668	0.0391	BAG5	1.04E+08
chr14	1.04E+08	1.04E+08	316	0.97	0.00668	0.0391	APOPT1	1.04E+08
chr14	1.04E+08	1.04E+08	316	0.97	0.00668	0.0391	BAG5	1.04E+08
chr14	1.04E+08	1.04E+08	316	0.97	0.00668	0.0391	APOPT1	1.04E+08
chr14	1.04E+08	1.04E+08	316	0.97	0.00668	0.0391	APOPT1	1.04E+08

chr14	1.04E+08	1.04E+08	316	0.97	0.00668	0.0391	APOPT1	1.04E+08
chr14	1.04E+08	1.04E+08	316	0.97	0.00668	0.0391	BAG5	1.04E+08
chr14	1.04E+08	1.04E+08	316	0.97	0.00668	0.0391	APOPT1	1.04E+08
chr14	1.04E+08	1.04E+08	316	0.97	0.00668	0.0391	APOPT1	1.04E+08
chr5	1.54E+08	1.54E+08	241	-1.09	0.00671	0.0393	MIR378H	1.54E+08
chr1	1.55E+08	1.55E+08	620	0.79	0.00676	0.0395	PMVK	1.55E+08
chr4	1.65E+08	1.65E+08	112	-1.52	0.00677	0.0395	1-Mar	1.65E+08
chr5	1.12E+08	1.12E+08	111	-1.42	0.00677	0.0395	EPB41L4A	1.12E+08
chr5	1.12E+08	1.12E+08	111	-1.42	0.00677	0.0395	EPB41L4A-/	1.12E+08
chr12	49716730	49717081	352	0.75	0.00678	0.0395	TROAP	49711970
chr12	49716730	49717081	352	0.75	0.00678	0.0395	TROAP	49711970
chr12	49716730	49717081	352	0.75	0.00678	0.0395	TROAP	49711970
chr12	49716730	49717081	352	0.75	0.00678	0.0395	LOC101927	49716359
chr11	68227536	68228166	631	0.69	0.0068	0.0396	PPP6R3	68223185
chr11	68227536	68228166	631	0.69	0.0068	0.0396	PPP6R3	68223185
chr11	68227536	68228166	631	0.69	0.0068	0.0396	PPP6R3	68223185
chr11	68227536	68228166	631	0.69	0.0068	0.0396	PPP6R3	68223185
chr11	68227536	68228166	631	0.69	0.0068	0.0396	PPP6R3	68223185
chr11	68227536	68228166	631	0.69	0.0068	0.0396	PPP6R3	68223185
chrX	1.23E+08	1.23E+08	269	-1.03	0.00681	0.0397	THOC2	1.23E+08
chr7	1.4E+08	1.4E+08	253	-0.97	0.00685	0.0398	RAB19	1.4E+08
chr7	1.34E+08	1.34E+08	129	-1.24	0.00685	0.0398	AKR1B15	1.34E+08
chr17	4634589	4635173	585	0.77	0.00687	0.0399	MED11	4629702
chr17	4634589	4635173	585	0.77	0.00687	0.0399	MED11	4629702
chr6	1.26E+08	1.26E+08	705	-0.63	0.00688	0.04	NCOA7	1.26E+08
chr6	1.26E+08	1.26E+08	705	-0.63	0.00688	0.04	NCOA7	1.26E+08
chr6	1.26E+08	1.26E+08	705	-0.63	0.00688	0.04	NCOA7	1.26E+08
chr14	53167436	53167751	316	-0.99	0.00694	0.0402	ERO1A	53160649
chr9	78505324	78505571	248	-1.2	0.007	0.0405	PCSK5	78500559
chr9	78505324	78505571	248	-1.2	0.007	0.0405	PCSK5	78500559
chr9	78505324	78505571	248	-1.2	0.007	0.0405	PCSK5	78500559
chr2	2.09E+08	2.09E+08	611	-0.64	0.00704	0.0407	FZD5	2.09E+08
chr22	20067459	20067850	392	0.71	0.00708	0.0409	DGCR8	20062754
chr22	20067459	20067850	392	0.71	0.00708	0.0409	DGCR8	20062754
chr7	38380929	38381160	232	-1.16	0.00708	0.0409	TRG-AS1	38376177
chr10	1.24E+08	1.24E+08	365	-0.75	0.00712	0.041	ATE1	1.24E+08
chr10	1.24E+08	1.24E+08	365	-0.75	0.00712	0.041	ATE1	1.24E+08
chr10	1.24E+08	1.24E+08	365	-0.75	0.00712	0.041	ATE1	1.24E+08
chr10	1.24E+08	1.24E+08	365	-0.75	0.00712	0.041	ATE1	1.24E+08
chr10	1.24E+08	1.24E+08	365	-0.75	0.00712	0.041	ATE1	1.24E+08
chr10	1.24E+08	1.24E+08	365	-0.75	0.00712	0.041	ATE1	1.24E+08
chr10	1.24E+08	1.24E+08	365	-0.75	0.00712	0.041	ATE1-AS1	1.24E+08
chr19	3557588	3557727	140	1.29	0.00718	0.0413	MFSD12	3555582
chr19	3557588	3557727	140	1.29	0.00718	0.0413	MFSD12	3555582
chr1	2.15E+08	2.15E+08	477	-0.64	0.00721	0.0414	CENPF	2.15E+08
chr16	15707712	15707796	85	-1.88	0.00721	0.0414	MIR6506	15702952
chr1	92496518	92496673	156	-1.56	0.00722	0.0415	EPHX4	92490532

chr22	20004367	20004587	221	0.88	0.00722	0.0415	TANGO2	20003591
chr22	20004367	20004587	221	0.88	0.00722	0.0415	ARVCF	20002309
chr22	20004367	20004587	221	0.88	0.00722	0.0415	TANGO2	20003591
chr22	20004367	20004587	221	0.88	0.00722	0.0415	TANGO2	20003591
chr22	20004367	20004587	221	0.88	0.00722	0.0415	TANGO2	20003591
chr22	20004367	20004587	221	0.88	0.00722	0.0415	TANGO2	20003591
chr22	20004367	20004587	221	0.88	0.00722	0.0415	TANGO2	20003591
chr22	20004367	20004587	221	0.88	0.00722	0.0415	TANGO2	19999522
chr22	20004367	20004587	221	0.88	0.00722	0.0415	TANGO2	20003591
chr22	20004367	20004587	221	0.88	0.00722	0.0415	TANGO2	20003591
chr22	20004367	20004587	221	0.88	0.00722	0.0415	TANGO2	20003591
chr22	20004367	20004587	221	0.88	0.00722	0.0415	TANGO2	20003591
chr22	20004367	20004587	221	0.88	0.00722	0.0415	TANGO2	19999522
chr22	20004367	20004587	221	0.88	0.00722	0.0415	TANGO2	20003591
chr1	1.85E+08	1.85E+08	603	-0.87	0.00724	0.0415	FAM129A	1.85E+08
chr16	87984503	87985193	691	0.66	0.00726	0.0416	BANP	87980037
chr16	87984503	87985193	691	0.66	0.00726	0.0416	BANP	87980037
chr16	87984503	87985193	691	0.66	0.00726	0.0416	BANP	87980037
chr16	87984503	87985193	691	0.66	0.00726	0.0416	BANP	87980037
chr16	87984503	87985193	691	0.66	0.00726	0.0416	BANP	87980037
chr11	67572762	67573554	793	0.72	0.00726	0.0416	FAM86C2P	67570807
chr20	48937762	48938145	384	-0.73	0.00728	0.0417	LINC01271	48935879
chr13	97875995	97876296	302	-0.97	0.00729	0.0417	MBNL2	97869542
chr13	97875995	97876296	302	-0.97	0.00729	0.0417	MBNL2	97869549
chr13	97875995	97876296	302	-0.97	0.00729	0.0417	MBNL2	97869549
chr15	1.01E+08	1.01E+08	296	-0.84	0.00731	0.0418	LRRK1	1.01E+08
chr1	90098429	90099102	674	-0.75	0.00732	0.0418	LRRC8C	90093643
chr1	90098429	90099102	674	-0.75	0.00732	0.0418	FLJ27354	90096453
chr20	40246942	40247318	377	-0.67	0.00733	0.0418	CHD6	40245133
chr9	5509684	5509904	221	-0.96	0.00733	0.0419	PDCD1LG2	5505544
chr17	8023521	8023745	225	0.92	0.00734	0.0419	ALOXE3	8020234
chr17	8023521	8023745	225	0.92	0.00734	0.0419	ALOXE3	8019860
chr5	1.54E+08	1.54E+08	369	-0.71	0.00734	0.0419	SAP30L-AS1	1.54E+08
chr5	1.54E+08	1.54E+08	369	-0.71	0.00734	0.0419	SAP30L	1.54E+08
chr5	1.54E+08	1.54E+08	369	-0.71	0.00734	0.0419	SAP30L	1.54E+08
chr5	1.54E+08	1.54E+08	369	-0.71	0.00734	0.0419	SAP30L	1.54E+08
chr5	1.54E+08	1.54E+08	369	-0.71	0.00734	0.0419	SAP30L	1.54E+08
chr12	1.21E+08	1.21E+08	250	1.07	0.00738	0.0421	SPPL3	1.21E+08
chr12	1.21E+08	1.21E+08	250	1.07	0.00738	0.0421	XLOC_0099	1.21E+08
chr14	99740919	99741263	345	-0.94	0.0074	0.0421	BCL11B	99736050
chr14	99740919	99741263	345	-0.94	0.0074	0.0421	BCL11B	99736050
chr14	99740919	99741263	345	-0.94	0.0074	0.0421	BCL11B	99736050
chr14	99740919	99741263	345	-0.94	0.0074	0.0421	BCL11B	99736050
chr7	1.21E+08	1.21E+08	349	-0.96	0.00741	0.0421	WNT16	1.21E+08
chr19	18263344	18264080	737	0.75	0.00751	0.0426	PIK3R2	18258987
chr19	18263344	18264080	737	0.75	0.00751	0.0426	PIK3R2	18258987
chr16	50733794	50733981	188	-1.15	0.00753	0.0426	NOD2	50728260

chr2	1.31E+08	1.31E+08	431	-0.68	0.00754	0.0427	PTPN18	1.31E+08
chr2	1.31E+08	1.31E+08	431	-0.68	0.00754	0.0427	PTPN18	1.31E+08
chr10	1.25E+08	1.25E+08	198	-0.97	0.00754	0.0427	C10orf88	1.25E+08
chr7	39772771	39773210	440	-0.71	0.00756	0.0428	LINC00265	39768166
chr19	39390084	39390612	529	0.79	0.00756	0.0428	SIRT2	39388502
chr19	39390084	39390612	529	0.79	0.00756	0.0428	SIRT2	39388502
chr19	39390084	39390612	529	0.79	0.00756	0.0428	NFKBIB	39385339
chr19	39390084	39390612	529	0.79	0.00756	0.0428	NFKBIB	39385569
chr19	39390084	39390612	529	0.79	0.00756	0.0428	NFKBIB	39385569
chr19	39390084	39390612	529	0.79	0.00756	0.0428	SIRT2	39388463
chr22	30987876	30988318	443	0.78	0.00757	0.0428	PES1	30985927
chr22	30987876	30988318	443	0.78	0.00757	0.0428	PES1	30985927
chr19	13049171	13049518	348	0.95	0.00761	0.043	FARSA	13042558
chr19	13049171	13049518	348	0.95	0.00761	0.043	CALR	13044413
chr19	13049171	13049518	348	0.95	0.00761	0.043	MIR6515	13046297
chr11	15991625	15991733	109	-1.22	0.00762	0.043	MIR6073	15989167
chr5	1.15E+08	1.15E+08	728	-0.84	0.00762	0.043	PGGT1B	1.15E+08
chr16	2932589	2933321	733	0.73	0.00762	0.043	FLYWCH2	2928195
chr16	2932589	2933321	733	0.73	0.00762	0.043	FLYWCH2	2928195
chr16	2932589	2933321	733	0.73	0.00762	0.043	FLYWCH2	2928195
chr10	64577679	64577947	269	-0.82	0.00763	0.043	EGR2	64574126
chr10	64577679	64577947	269	-0.82	0.00763	0.043	EGR2	64574126
chr10	64577679	64577947	269	-0.82	0.00763	0.043	EGR2	64576927
chr10	64577679	64577947	269	-0.82	0.00763	0.043	EGR2	64574126
chr19	50169084	50169253	170	1.11	0.00767	0.0431	IRF3	50167132
chr19	50169084	50169253	170	1.11	0.00767	0.0431	IRF3	50167132
chr19	50169084	50169253	170	1.11	0.00767	0.0431	BCL2L12	50163398
chr19	50169084	50169253	170	1.11	0.00767	0.0431	BCL2L12	50163398
chr19	50169084	50169253	170	1.11	0.00767	0.0431	IRF3	50167132
chr19	50169084	50169253	170	1.11	0.00767	0.0431	IRF3	50167132
chr19	50169084	50169253	170	1.11	0.00767	0.0431	IRF3	50167132
chr19	50169084	50169253	170	1.11	0.00767	0.0431	IRF3	50167132
chr19	50169084	50169253	170	1.11	0.00767	0.0431	BCL2L12	50163398
chr19	50169084	50169253	170	1.11	0.00767	0.0431	IRF3	50167132
chr19	50169084	50169253	170	1.11	0.00767	0.0431	IRF3	50167132
chr19	50169084	50169253	170	1.11	0.00767	0.0431	BCL2L12	50163398
chr19	50169084	50169253	170	1.11	0.00767	0.0431	IRF3	50167132
chr19	50169084	50169253	170	1.11	0.00767	0.0431	BCL2L12	50163398
chr19	50169084	50169253	170	1.11	0.00767	0.0431	BCL2L12	50163398
chr19	50169084	50169253	170	1.11	0.00767	0.0431	BCL2L12	50163398
chr19	50169084	50169253	170	1.11	0.00767	0.0431	BCL2L12	50163398
chr19	50169084	50169253	170	1.11	0.00767	0.0431	BCL2L12	50163398
chr19	50169084	50169253	170	1.11	0.00767	0.0431	BCL2L12	50163398
chr19	50169084	50169253	170	1.11	0.00767	0.0431	BCL2L12	50163398
chr19	50169084	50169253	170	1.11	0.00767	0.0431	BCL2L12	50163398
chr19	50169084	50169253	170	1.11	0.00767	0.0431	BCL2L12	50163398
chr1	1.53E+08	1.53E+08	261	-0.83	0.00772	0.0434	SPRR1B	1.53E+08

chr11	15096341	15096733	393	-0.94	0.00772	0.0434	CALCB	15090145
chr17	18163750	18163978	229	0.81	0.00774	0.0435	FLII	18160230
chr17	18163750	18163978	229	0.81	0.00774	0.0435	MIEF2	18158847
chr17	18163750	18163978	229	0.81	0.00774	0.0435	FLII	18160230
chr17	18163750	18163978	229	0.81	0.00774	0.0435	FLII	18159870
chr17	18163750	18163978	229	0.81	0.00774	0.0435	MIEF2	18158847
chr17	18163750	18163978	229	0.81	0.00774	0.0435	MIEF2	18159423
chr6	1.32E+08	1.32E+08	550	-0.63	0.00774	0.0435	CTGF	1.32E+08
chr19	4791300	4791783	484	0.75	0.00776	0.0435	FEM1A	4786727
chr18	12200307	12200489	183	-1.83	0.00777	0.0436	C18orf61	12195777
chr20	6103590	6104328	739	-0.6	0.00778	0.0436	FERMT1	6102191
chr2	2.19E+08	2.19E+08	221	-0.93	0.00782	0.0438	PNKD	2.19E+08
chr7	24795341	24795523	183	-0.88	0.00783	0.0438	DFNA5	24795083
chr5	16935496	16935784	289	-0.98	0.00784	0.0439	MYO10	16934385
chr8	1.18E+08	1.18E+08	759	-0.6	0.00788	0.0441	UTP23	1.18E+08
chr14	57857021	57857381	361	-0.81	0.0079	0.0441	NAA30	57852270
chr19	9879238	9879512	275	1.11	0.00791	0.0441	ZNF846	9877410
chr16	48399699	48400031	333	-0.79	0.00793	0.0442	SIAH1	48397784
chr17	4710188	4710514	327	0.87	0.00794	0.0442	PLD2	4705395
chr17	4710188	4710514	327	0.87	0.00794	0.0442	PLD2	4705395
chr7	1.55E+08	1.55E+08	289	-0.72	0.00796	0.0443	INSIG1	1.55E+08
chr7	1.55E+08	1.55E+08	289	-0.72	0.00796	0.0443	INSIG1	1.55E+08
chr7	1.55E+08	1.55E+08	289	-0.72	0.00796	0.0443	INSIG1	1.55E+08
chr10	44069887	44070150	264	-1.05	0.00797	0.0443	ZNF239	44068066
chr10	44069887	44070150	264	-1.05	0.00797	0.0443	ZNF239	44068066
chr10	44069887	44070150	264	-1.05	0.00797	0.0443	ZNF239	44068065
chr17	40306919	40307268	350	0.8	0.00797	0.0443	RAB5C	40305062
chr17	40306919	40307268	350	0.8	0.00797	0.0443	RAB5C	40305062
chr17	40306919	40307268	350	0.8	0.00797	0.0443	RAB5C	40305062
chr19	49496454	49497165	712	0.69	0.00798	0.0444	RUVBL2	49492155
chr19	49496454	49497165	712	0.69	0.00798	0.0444	GYS1	49494610
chr19	49496454	49497165	712	0.69	0.00798	0.0444	GYS1	49494610
chr19	49496454	49497165	712	0.69	0.00798	0.0444	GYS1	49494610
chr4	1.46E+08	1.46E+08	713	-0.61	0.00803	0.0446	OTUD4	1.46E+08
chr1	2.48E+08	2.48E+08	172	-1.33	0.00804	0.0446	GCSAML-A!	2.48E+08
chr20	43803318	43803530	213	-1.23	0.00805	0.0447	PI3	43798539
chr1	2.08E+08	2.08E+08	172	-0.97	0.00806	0.0447	PLXNA2	2.08E+08
chrX	74493777	74494473	697	-0.68	0.00809	0.0448	UPRT	74488893
chrX	74493777	74494473	697	-0.68	0.00809	0.0448	UPRT	74488893
chrX	74493777	74494473	697	-0.68	0.00809	0.0448	UPRT	74488893
chr1	1.12E+08	1.12E+08	566	-0.77	0.0081	0.0448	PIFO	1.12E+08
chr1	1.12E+08	1.12E+08	566	-0.77	0.0081	0.0448	PIFO	1.12E+08
chr1	1.12E+08	1.12E+08	566	-0.77	0.0081	0.0448	PIFO	1.12E+08
chr19	4402302	4402569	268	1.06	0.00813	0.0449	CHAF1A	4397659
chr19	4402302	4402569	268	1.06	0.00813	0.0449	SH3GL1	4398415
chr19	4402302	4402569	268	1.06	0.00813	0.0449	SH3GL1	4398565
chr19	4402302	4402569	268	1.06	0.00813	0.0449	SH3GL1	4398565

chr8	1.34E+08	1.34E+08	191	-1.13	0.00816	0.0451	MIR7848	1.34E+08
chr7	27169777	27170019	243	-1.04	0.00817	0.0451	HOXA3	27164639
chr7	27169777	27170019	243	-1.04	0.00817	0.0451	HOXA4	27168399
chr11	1.18E+08	1.18E+08	156	-1.15	0.00818	0.0451	TMPRSS4	1.18E+08
chr11	1.18E+08	1.18E+08	156	-1.15	0.00818	0.0451	TMPRSS4	1.18E+08
chr11	1.18E+08	1.18E+08	156	-1.15	0.00818	0.0451	TMPRSS4	1.18E+08
chr11	1.18E+08	1.18E+08	156	-1.15	0.00818	0.0451	TMPRSS4	1.18E+08
chr11	1.18E+08	1.18E+08	156	-1.15	0.00818	0.0451	TMPRSS4	1.18E+08
chr11	1.18E+08	1.18E+08	156	-1.15	0.00818	0.0451	TMPRSS4	1.18E+08
chr11	1.18E+08	1.18E+08	156	-1.15	0.00818	0.0451	TMPRSS4	1.18E+08
chr11	1.14E+08	1.14E+08	456	-0.69	0.0082	0.0452	ZW10	1.14E+08
chr11	1.14E+08	1.14E+08	456	-0.69	0.0082	0.0452	CLDN25	1.14E+08
chr8	9045582	9045841	260	-1.13	0.00833	0.0457	LOC10192‹	9041508
chr17	74023329	74023709	381	0.75	0.00834	0.0457	EVPL	74021507
chr8	1.11E+08	1.11E+08	384	-0.74	0.00834	0.0457	KCNV1	1.11E+08
chr15	84115723	84116179	457	-0.83	0.00834	0.0458	SH3GL3	84110979
chr15	84115723	84116179	457	-0.83	0.00834	0.0458	SH3GL3	84110979
chr15	84115723	84116179	457	-0.83	0.00834	0.0458	SH3GL3	84110979
chr15	84115723	84116179	457	-0.83	0.00834	0.0458	SH3GL3	84110979
chr17	2206714	2207268	555	0.73	0.00842	0.0461	SMG6	2205069
chr17	2206714	2207268	555	0.73	0.00842	0.0461	SRR	2202236
chr17	2206714	2207268	555	0.73	0.00842	0.0461	SRR	2202236
chr11	4208253	4208439	187	-1.19	0.00844	0.0462	LOC100506	4203381
chr19	16582777	16583001	225	1.05	0.00845	0.0462	EPS15L1	16580823
chr19	16582777	16583001	225	1.05	0.00845	0.0462	EPS15L1	16580823
chr19	16582777	16583001	225	1.05	0.00845	0.0462	EPS15L1	16580823
chr19	16582777	16583001	225	1.05	0.00845	0.0462	EPS15L1	16580823
chr19	16582777	16583001	225	1.05	0.00845	0.0462	EPS15L1	16580823
chr19	16582777	16583001	225	1.05	0.00845	0.0462	EPS15L1	16580823
chr6	35420002	35420163	162	1	0.00846	0.0463	FANCE	35415137
chr3	1.87E+08	1.87E+08	174	-0.87	0.00849	0.0464	BCL6	1.87E+08
chr3	1.87E+08	1.87E+08	174	-0.87	0.00849	0.0464	BCL6	1.87E+08
chr2	1.09E+08	1.09E+08	732	-0.66	0.00849	0.0464	LIMS1	1.09E+08
chr15	89181942	89182412	471	-0.61	0.00851	0.0465	ISG20	89176973
chr15	89181942	89182412	471	-0.61	0.00851	0.0465	ISG20	89176973
chr15	89181942	89182412	471	-0.61	0.00851	0.0465	ISG20	89176973
chr17	4269859	4270277	419	0.76	0.00853	0.0465	UBE2G1	4267969
chr15	89164357	89164610	254	-0.76	0.00857	0.0466	AEN	89159526
chr21	45759143	45759371	229	1.26	0.00857	0.0467	C21orf2	45756221
chr21	45759143	45759371	229	1.26	0.00857	0.0467	C21orf2	45757285
chr21	45759143	45759371	229	1.26	0.00857	0.0467	C21orf2	45757285
chr21	45759143	45759371	229	1.26	0.00857	0.0467	C21orf2	45757285
chr12	53448105	53448352	248	0.97	0.00859	0.0467	MIR6757	53445727
chr12	53448105	53448352	248	0.97	0.00859	0.0467	LOC28333‹	53446222
chr10	1.19E+08	1.19E+08	770	-0.64	0.00861	0.0468	PDZD8	1.19E+08
chr10	1.27E+08	1.27E+08	171	-1.23	0.00864	0.0469	MIR4296	1.27E+08
chr7	1.16E+08	1.16E+08	140	-1.14	0.00864	0.0469	LOC10272‹	1.16E+08

chr7	1.16E+08	1.16E+08	140	-1.14	0.00864	0.0469	LOC10272	1.16E+08
chr6	1.25E+08	1.25E+08	364	-0.88	0.00865	0.0469	TPD52L1	1.25E+08
chr6	1.25E+08	1.25E+08	364	-0.88	0.00865	0.0469	TPD52L1	1.25E+08
chr6	1.25E+08	1.25E+08	364	-0.88	0.00865	0.0469	TPD52L1	1.25E+08
chr6	1.25E+08	1.25E+08	364	-0.88	0.00865	0.0469	TPD52L1	1.25E+08
chr6	1.25E+08	1.25E+08	364	-0.88	0.00865	0.0469	TPD52L1	1.25E+08
chr6	1.25E+08	1.25E+08	364	-0.88	0.00865	0.0469	TPD52L1	1.25E+08
chr6	1.25E+08	1.25E+08	364	-0.88	0.00865	0.0469	TPD52L1	1.25E+08
chr5	1.16E+08	1.16E+08	319	-0.88	0.00868	0.047	SEMA6A	1.16E+08
chr5	1.16E+08	1.16E+08	319	-0.88	0.00868	0.047	SEMA6A	1.16E+08
chr1	2.21E+08	2.21E+08	755	-0.6	0.0087	0.0471	2-Mar	2.21E+08
chr1	2.21E+08	2.21E+08	755	-0.6	0.0087	0.0471	2-Mar	2.21E+08
chr9	27528994	27529154	161	-1.03	0.00876	0.0473	MOB3B	27527850
chr12	1.14E+08	1.14E+08	324	-0.87	0.00877	0.0474	TPCN1	1.14E+08
chr1	1.12E+08	1.12E+08	191	-1.14	0.00877	0.0474	DENND2D	1.12E+08
chr1	1.12E+08	1.12E+08	191	-1.14	0.00877	0.0474	DENND2D	1.12E+08
chr17	42422434	42422703	270	0.74	0.00878	0.0474	GRN	42417453
chr17	79870694	79870855	162	0.94	0.00879	0.0474	PCYT2	79867353
chr17	79870694	79870855	162	0.94	0.00879	0.0474	PCYT2	79867353
chr17	79870694	79870855	162	0.94	0.00879	0.0474	PCYT2	79867353
chr17	79870694	79870855	162	0.94	0.00879	0.0474	PCYT2	79867353
chr17	79870694	79870855	162	0.94	0.00879	0.0474	PCYT2	79867353
chr17	79870694	79870855	162	0.94	0.00879	0.0474	PCYT2	79867115
chr17	79870694	79870855	162	0.94	0.00879	0.0474	PCYT2	79867115
chr9	80850842	80851176	335	-0.89	0.00882	0.0475	CEP78	80845990
chr9	80850842	80851176	335	-0.89	0.00882	0.0475	CEP78	80845990
chr11	64072562	64073080	519	0.71	0.00883	0.0476	ESRRA	64068698
chr11	64072562	64073080	519	0.71	0.00883	0.0476	ESRRA	64067999
chr11	64072562	64073080	519	0.71	0.00883	0.0476	ESRRA	64067999
chr7	1.23E+08	1.23E+08	1124	-0.54	0.00883	0.0476	CADPS2	1.23E+08
chr7	1.23E+08	1.23E+08	1124	-0.54	0.00883	0.0476	CADPS2	1.23E+08
chr7	1.23E+08	1.23E+08	1124	-0.54	0.00883	0.0476	CADPS2	1.23E+08
chr19	36545017	36545813	797	0.69	0.00884	0.0476	WDR62	36540782
chr19	36545017	36545813	797	0.69	0.00884	0.0476	WDR62	36540782
chr19	36545017	36545813	797	0.69	0.00884	0.0476	THAP8	36543664
chr9	1.4E+08	1.4E+08	266	0.93	0.00885	0.0476	EDF1	1.4E+08
chr9	1.4E+08	1.4E+08	266	0.93	0.00885	0.0476	EDF1	1.4E+08
chr9	1.4E+08	1.4E+08	266	0.93	0.00885	0.0476	EDF1	1.4E+08
chr9	1.4E+08	1.4E+08	266	0.93	0.00885	0.0476	EDF1	1.4E+08
chr9	1.4E+08	1.4E+08	266	0.93	0.00885	0.0476	EDF1	1.4E+08
chr11	5617304	5617565	262	-0.72	0.00885	0.0476	TRIM6-TRI	5612864
chr11	5617304	5617565	262	-0.72	0.00885	0.0476	TRIM6	5612864
chr11	5617304	5617565	262	-0.72	0.00885	0.0476	TRIM6	5612864
chr11	5617304	5617565	262	-0.72	0.00885	0.0476	TRIM6	5612864
chr11	5617304	5617565	262	-0.72	0.00885	0.0476	TRIM6	5612330
chr19	48246389	48247051	663	0.68	0.00885	0.0476	GLTSCR2	48243792
chr19	39832614	39833069	456	0.89	0.00886	0.0476	SAMD4B	39828098

chr19	39832614	39833069	456	0.89	0.00886	0.0476	SAMD4B	39828098
chr20	7999696	8000591	896	-0.68	0.00887	0.0477	TMX4	7998476
chr5	49736498	49737396	899	-0.73	0.00887	0.0477	EMB	49735234
chr17	4736245	4736821	577	0.81	0.0089	0.0478	MINK1	4731634
chr17	4736245	4736821	577	0.81	0.0089	0.0478	MINK1	4731634
chr17	4736245	4736821	577	0.81	0.0089	0.0478	MINK1	4731634
chr17	4736245	4736821	577	0.81	0.0089	0.0478	MINK1	4731634
chr10	1.05E+08	1.05E+08	154	-1.07	0.00891	0.0478	NEURL1	1.05E+08
chr17	12920884	12921813	930	-0.63	0.00893	0.0479	ELAC2	12919381
chr17	12920884	12921813	930	-0.63	0.00893	0.0479	ELAC2	12919381
chr17	12920884	12921813	930	-0.63	0.00893	0.0479	ELAC2	12919381
chr7	1.34E+08	1.34E+08	420	-0.61	0.00894	0.0479	BPGM	1.34E+08
chr7	1.34E+08	1.34E+08	420	-0.61	0.00894	0.0479	BPGM	1.34E+08
chr7	1.34E+08	1.34E+08	420	-0.61	0.00894	0.0479	BPGM	1.34E+08
chr4	40336063	40336188	126	-1.6	0.00894	0.0479	CHRNA9	40332345
chr4	74733651	74733767	117	-1.15	0.00895	0.0479	CXCL1	74730108
chr4	74733651	74733767	117	-1.15	0.00895	0.0479	CXCL1	74730108
chr1	1.66E+08	1.66E+08	291	-0.78	0.00896	0.048	MIR3658	1.66E+08
chr9	1.4E+08	1.4E+08	554	0.61	0.00897	0.048	TUBB4B	1.4E+08
chr11	11643548	11643861	314	-0.78	0.00898	0.0481	GALNT18	11641561
chr9	1.4E+08	1.4E+08	359	0.88	0.00899	0.0481	C9orf173-A	1.4E+08
chr9	1.4E+08	1.4E+08	359	0.88	0.00899	0.0481	NELFB	1.4E+08
chr11	1.31E+08	1.31E+08	406	-0.7	0.009	0.0481	SNX19	1.31E+08
chr11	1.31E+08	1.31E+08	406	-0.7	0.009	0.0481	SNX19	1.31E+08
chr19	11485288	11485828	541	0.68	0.00904	0.0483	SWSAP1	11480382
chr3	36985660	36986347	688	-1.09	0.00905	0.0483	TRANK1	36984548
chr1	26185912	26186115	204	1.17	0.00906	0.0484	AUNIP	26183949
chr1	26185912	26186115	204	1.17	0.00906	0.0484	AUNIP	26183949
chr6	18156257	18156526	270	-0.78	0.00908	0.0484	KDM1B	18150618
chr6	18156257	18156526	270	-0.78	0.00908	0.0484	TPMT	18153396
chr12	1.07E+08	1.07E+08	772	-0.62	0.00908	0.0484	LOC100287	1.07E+08
chr12	1.07E+08	1.07E+08	772	-0.62	0.00908	0.0484	RIC8B	1.07E+08
chr11	43942232	43942596	365	-0.88	0.00911	0.0485	ALKBH3-AS	43940494
chr3	49823633	49824204	572	0.81	0.00912	0.0485	IP6K1	49821627
chr3	49823633	49824204	572	0.81	0.00912	0.0485	IP6K1	49821973
chr3	49823633	49824204	572	0.81	0.00912	0.0485	IP6K1	49821973
chr12	48213737	48213935	199	1.11	0.00914	0.0486	HDAC7	48211763
chr12	48213737	48213935	199	1.11	0.00914	0.0486	HDAC7	48211763
chr12	48213737	48213935	199	1.11	0.00914	0.0486	HDAC7	48211763
chr6	1.62E+08	1.62E+08	452	-0.64	0.00915	0.0486	AGPAT4	1.62E+08
chr8	1.42E+08	1.42E+08	224	-0.93	0.00915	0.0487	SLC45A4	1.42E+08
chr8	1.42E+08	1.42E+08	224	-0.93	0.00915	0.0487	SLC45A4	1.42E+08
chr1	1.83E+08	1.83E+08	141	-1.43	0.00916	0.0487	SHCBP1L	1.83E+08
chr19	42901069	42901389	321	0.8	0.00921	0.0489	LIPE-AS1	42896299
chr19	42901069	42901389	321	0.8	0.00921	0.0489	LOC10193(	42896279
chr5	1.38E+08	1.38E+08	541	-0.68	0.00922	0.0489	BRD8	1.38E+08
chr5	1.38E+08	1.38E+08	541	-0.68	0.00922	0.0489	BRD8	1.38E+08

chr5	1.38E+08	1.38E+08	541	-0.68	0.00922	0.0489	BRD8	1.38E+08
chr5	1.38E+08	1.38E+08	541	-0.68	0.00922	0.0489	BRD8	1.38E+08
chr5	1.38E+08	1.38E+08	541	-0.68	0.00922	0.0489	BRD8	1.38E+08
chr5	1.38E+08	1.38E+08	541	-0.68	0.00922	0.0489	BRD8	1.38E+08
chr5	1.38E+08	1.38E+08	541	-0.68	0.00922	0.0489	KIF20A	1.38E+08
chr4	53578337	53578813	477	-0.62	0.00925	0.049	SNORA26	53574415
chr4	53578337	53578813	477	-0.62	0.00925	0.049	MIR4449	53573848
chr4	53578337	53578813	477	-0.62	0.00925	0.049	DANCR	53573620
chr1	1.2E+08	1.2E+08	84	-1.74	0.00928	0.0491	HAO2	1.2E+08
chr1	1.2E+08	1.2E+08	84	-1.74	0.00928	0.0491	HAO2	1.2E+08
chr1	1.2E+08	1.2E+08	84	-1.74	0.00928	0.0491	HAO2-IT1	1.2E+08
chr16	2587754	2588117	364	1.01	0.00928	0.0491	PDPK1	2582964
chr16	2587754	2588117	364	1.01	0.00928	0.0491	PDPK1	2582964
chr16	2587754	2588117	364	1.01	0.00928	0.0491	PDPK1	2582964
chr3	1.61E+08	1.61E+08	430	-1	0.00928	0.0492	SPTSSB	1.61E+08
chr2	2.42E+08	2.42E+08	326	0.86	0.00933	0.0494	STK25	2.42E+08
chr2	2.42E+08	2.42E+08	326	0.86	0.00933	0.0494	STK25	2.42E+08
chr2	2.42E+08	2.42E+08	326	0.86	0.00933	0.0494	STK25	2.42E+08
chr2	2.42E+08	2.42E+08	326	0.86	0.00933	0.0494	STK25	2.42E+08
chr2	2.42E+08	2.42E+08	326	0.86	0.00933	0.0494	STK25	2.42E+08
chr2	2.42E+08	2.42E+08	326	0.86	0.00933	0.0494	STK25	2.42E+08
chr2	2.42E+08	2.42E+08	326	0.86	0.00933	0.0494	STK25	2.42E+08
chr2	2.42E+08	2.42E+08	326	0.86	0.00933	0.0494	STK25	2.42E+08
chr2	2.42E+08	2.42E+08	326	0.86	0.00933	0.0494	STK25	2.42E+08
chr12	48551214	48551549	336	0.89	0.00935	0.0495	ASB8	48549377
chr9	1.4E+08	1.4E+08	656	0.61	0.00938	0.0496	MAN1B1	1.4E+08
chr9	1.4E+08	1.4E+08	656	0.61	0.00938	0.0496	MAN1B1	1.4E+08
chr9	1.4E+08	1.4E+08	656	0.61	0.00938	0.0496	MAN1B1	1.4E+08
chr9	1.4E+08	1.4E+08	656	0.61	0.00938	0.0496	MAN1B1-A	1.4E+08
chr17	80056080	80056876	797	0.72	0.0094	0.0497	FASN	80054106
chr7	1E+08	1E+08	390	0.78	0.00941	0.0497	POP7	1E+08
chr1	1.81E+08	1.81E+08	432	-0.7	0.00941	0.0497	IER5	1.81E+08
chr11	64085056	64085666	611	0.66	0.00946	0.0499	TRMT112	64083556
chr11	64085056	64085666	611	0.66	0.00946	0.0499	PRDX5	64080559
chr11	64085056	64085666	611	0.66	0.00946	0.0499	PRDX5	64080559
chr11	64085056	64085666	611	0.66	0.00946	0.0499	PRDX5	64080559
chr11	64085056	64085666	611	0.66	0.00946	0.0499	TRMT112	64083556
chr11	64085056	64085666	611	0.66	0.00946	0.0499	TRMT112	64083556

promoterstop
2.1E+08
2.1E+08
74334308
1.23E+08
30950614
3016945
57199621
57199621
57199621
57199560
40549601
40549529
40549529
37947830
37945044
1.02E+08
1.02E+08
1.02E+08
80875443
33817952
1.8E+08
47660771
2.33E+08
1.04E+08
1.04E+08
1.38E+08
86119520
1.09E+08
95365740
95366969
95369797
1.99E+08
1.14E+08
56644477
20797308
1.24E+08
1.24E+08
1.24E+08
2881744
91416768
91413821
91418107
1.19E+08
1.19E+08

67634168
78980723
78980723
1.31E+08
1.31E+08
58294037
17544605
17544605
17544605
1.14E+08
1.57E+08
1.73E+08
2.49E+08
1.06E+08
1.06E+08
1.06E+08
1.06E+08
1.06E+08
1.06E+08
1.06E+08
1.06E+08
2.01E+08
73782919
73781016
73785688
73782919
1.44E+08
1.44E+08
1.44E+08
1.32E+08
66515305
1.5E+08
1.5E+08
1.5E+08
1.5E+08
1.5E+08
1.5E+08
1.5E+08
1.5E+08
1.08E+08
1.34E+08
94428767
35053240
35053240
1.06E+08
21454983
21454983

56769017
71255169
71255169
12682737
1.7E+08
1.7E+08
1.7E+08
86701850
1.34E+08
56889495
86119520
31289614
1.05E+08
62607408
62607408
62607408
62607408
62607408
62607408
62607408
94931161
94931174
94931082
94931278
12256317
12256359
12256359
1.05E+08
1.05E+08
54996409
1.89E+08
1.89E+08
2.24E+08
10478665
10479480
86119520
3596111
1.07E+08
1.55E+08
79569147
79569147
79569147
79569147
79569147
79569147
1.35E+08
17040963

94422570
94422570
94970128
6555640
6555654
6556761
19410924
12229059
12228999
60693912
60693912
10313168
1.81E+08
1.81E+08
1.81E+08
1.81E+08
1.81E+08
1.8E+08
1.49E+08
1.49E+08
9030596
76116832
1.49E+08
1.04E+08
1.35E+08
1.34E+08
1.34E+08
86891768
41304158
23610297
1.43E+08
1.35E+08
1.35E+08
1.35E+08
1.81E+08
1.81E+08
41088243
41088243
62202524
86159715
2.1E+08
2.1E+08
2.1E+08
1.24E+08
1.3E+08
73845798
1.2E+08

1.2E+08
1.2E+08
1.2E+08
1.06E+08
47129295
47129295
47129295
1.4E+08
1.4E+08
1.4E+08
1.4E+08
59069438
1.87E+08
1.33E+08
1.33E+08
1.33E+08
1.33E+08
1.33E+08
1.33E+08
1.33E+08
42998435
1.25E+08
1.25E+08
1.66E+08
1.66E+08
1.66E+08
1.66E+08
50147381
50148400
1.03E+08
19563372
89166526
2.45E+08
2.45E+08
2.45E+08
2.43E+08
56281431
1.39E+08
1.39E+08
1.39E+08
1.64E+08
19268167
19268202
19267507
25204917
15119562
10445029

48370216
45727605
37420690
1.06E+08
1.06E+08
77193403
1.5E+08
1.5E+08
1.5E+08
1.5E+08
41413444
41413444
87913635
87911669
1.19E+08
1.24E+08
49258455
43194953
43194169
88979250
88979250
31642611
2.11E+08
2.11E+08
1.03E+08
1.03E+08
1.03E+08
1.03E+08
1.03E+08
1.03E+08
1.03E+08
10436561
48023515
48023515
48023515
11461981
11461981
11461981
11459180
2881744
61932290
88840237
5023096
18061923
18061113
1.55E+08
1.55E+08

1.55E+08
1.55E+08
7213693
7213287
7212317
7212855
52287155
52287155
52287155
52287155
52287155
52287155
1.28E+08
85785139
85785139
85785923
85785139
24802639
24802639
24802083
1.03E+08
1.03E+08
15008669
15006297
15006297
15006297
15006297
15006297
15006297
2.28E+08
78268425
78268425
90040987
90040987
90040987
90044240
1.16E+08
1.25E+08
1.25E+08
1.25E+08
1.25E+08
1.25E+08
1.25E+08
1.25E+08
1.25E+08
89729193
89729193

89729193
89726151
89726454
89726211
89726211
89726454
89726211
89726211
1.07E+08
99743050
99743050
99743050
99743050
1.2E+08
1.2E+08
1.6E+08
700754
700754
697590
697590
697590
697590
68754716
92752336
92752336
1.42E+08
1.42E+08
1.87E+08
49520112
1.24E+08
51021894
51022096
51026428
51023454
51021506
51022096
51022096
51021894
51026428
51023454
3697614
3697614
3697614
16240810
1.14E+08
1.14E+08
59617479

32913887
4537031
4537031
4537031
4539847
1.6E+08
85747587
79937425
79937425
79937425
1.34E+08
563449
562970
563449
565779
1.48E+08
1.48E+08
39275759
78958727
78958727
1.37E+08
30690616
1.51E+08
91063705
33021085
47015099
47015099
41926515
41926515
41926515
41926515
68963912
68963912
1.4E+08
1.29E+08
38731689
1.42E+08
1.42E+08
42532790
1.45E+08
1.45E+08
1.55E+08
1.55E+08
1.55E+08
1.55E+08
1.55E+08
1.1E+08

1.1E+08
18637937
8837224
8837882
49464800
77465540
49460116
49460116
49460116
49460116
49460116
49460116
49460116
49460116
49460116
1.35E+08
50182408
50182408
50182408
50187373
50182408
1.07E+08
1.07E+08
52919496
52919496
52919496
1.42E+08
1.28E+08
15495612
1.04E+08
68304155
1.09E+08
54795431
54795431
54795431
94862029
94862029
94862029
94860153
94862029
94862029
94862029
94862029
94862029
94862029
94862029
1.45E+08

1.88E+08
1.88E+08
1.88E+08
1.33E+08
1.33E+08
1.33E+08
52282185
52281808
64651240
83958468
83957209
6039726
77193403
50272179
50271515
50272179
1.63E+08
1.63E+08
1.63E+08
7157371
7160259
7159995
7157519
7157519
7157519
5682775
5682775
5682775
5682775
5682775
5682775
5685907
5682775
5682775
5682775
5682775
5685907
92338880
43951231
43951668
43951668
43951668
1.21E+08
1.19E+08
74608222
4612632
4612632

4609524
1.5E+08
1.6E+08
1.09E+08
1.09E+08
26952468
2.48E+08
2.48E+08
2.48E+08
2.48E+08
2.48E+08
2.48E+08
1.6E+08
71265598
71265598
71265598
89755075
89755075
89755075
89755075
89755075
89755075
40761072
1.17E+08
11615398
36411850
36411878
36411782
36411842
36411878
67095880
1.27E+08
2.36E+08
61549242
1.13E+08
1.02E+08
2.24E+08
85789689
36434998
36434998
36434998
53087209
35858976
35858976
96191164
26587545
26587545

1.13E+08
82088093
46642748
1.18E+08
58165976
65598931
7312450
7313501
7312450
1.07E+08
1.07E+08
1.07E+08
76061304
76061304
76061304
1.13E+08
36100260
36100260
1.84E+08
1.72E+08
88744454
1.41E+08
1.41E+08
1.41E+08
1.41E+08
44090957
44086039
44086173
44086173
44086574
44086173
44086173
70088057
70088057
27049567
27051599
27052698
27050286
27048999
27050286
27049908
27050286
27050286
27050286
27052446
27049908
27050286

27049908
2.47E+08
17676319
31235683
2016150
2014325
2015637
2.32E+08
2.32E+08
2.32E+08
2.32E+08
2.32E+08
2.32E+08
2.32E+08
2.32E+08
2.32E+08
2.32E+08
2.32E+08
2.32E+08
2.32E+08
2.32E+08
2.32E+08
2.32E+08
2.32E+08
2.32E+08
2.32E+08
2.32E+08
2.32E+08
2.32E+08
67883917
1.01E+08
18389580
89669633
1.11E+08
1.11E+08
1.24E+08
7237638
7237638
1.69E+08
1.69E+08
1.35E+08
1.35E+08
89888065
89888065
89888065
74273696

7766172
7763063
40356189
40353172
19431017
42280529
42280529
1.83E+08
1.83E+08
1.83E+08
1.83E+08
1.83E+08
1.33E+08
1.33E+08
18550899
18550899
48130199
48130808
48130199
48130850
48130736
48130850
64139886
52537883
1.26E+08
1.26E+08
1.26E+08
1.19E+08
52204636
65031322
1.32E+08
799391
799391
799391
15516711
15516711
15514432
1.28E+08
641668
641356
642076
642076
642172
1.82E+08
1.82E+08
1.82E+08
1.83E+08

49590396
49590396
77508010
77507263
77507636
22957754
1.13E+08
47129295
47129295
47129295
22114688
22114688
75576589
56690966
56690966
56690966
56687790
56687790
2.34E+08
1.2E+08
1.19E+08
1.19E+08
8107828
8107828
8107828
1.37E+08
2.22E+08
2.22E+08
512283
512283
512283
512283
511821
511821
511821
511821
3605734
79655954
79652961
2.17E+08
49995222
49992810
49992810
49995873
49996163
49996431
49992864

49992810
97055905
10424797
10420470
1.31E+08
1.31E+08
1.31E+08
43192207
43192207
43192207
43192207
86417964
86415771
2.17E+08
2.17E+08
2.17E+08
2.17E+08
88732497
88730187
9934779
9934779
9934779
9934779
9934779
9934779
9934779
9934779
97252862
97252862
97252862
97252862
10281322
2.47E+08
2.47E+08
2.47E+08
69440455
38907345
38907345
2.33E+08
42811952
42811796
42811929
42808283
16131316
1.3E+08
1.3E+08
1.32E+08

26977177
68260912
24669262
24669262
24672115
79638667
79638667
79638667
79638667
79638176
79638667
79638667
79638667
79635760
1.24E+08
18670571
18670571
18670571
1.16E+08
57238335
57238335
57238335
66888739
66888739
2.21E+08
2.21E+08
2.21E+08
2.21E+08
2.21E+08
2.31E+08
59441511
2420200
62498284
48678860
48678860
48678860
48678860
48675948
71255169
71255169
4210381
1.14E+08
1.14E+08
21393561
17712065
17712065
17712065

17708360
17709273
38582761
38582761
38582836
7536995
2.15E+08
11550980
11550980
11551603
11548077
11548560
11548077
11548465
11548077
90342973
90342973
90342973
90342973
90342973
11859436
75724896
75724896
1.66E+08
1.63E+08
1.63E+08
13592624
13592624
41915547
41915547
1.39E+08
1.39E+08
1.39E+08
1.39E+08
10028114
1.14E+08
1.71E+08
81189741
81191418
81189741
663340
659603
88328218
88326869
74332672
1.04E+08
2.42E+08

2.42E+08
2.42E+08
2.42E+08
2.42E+08
2.42E+08
2.42E+08
67041277
67041277
67041277
67041277
1.11E+08
1.01E+08
1.01E+08
47926041
1.02E+08
1.02E+08
30574642
30574642
73719515
73722775
73719515
1.31E+08
1.31E+08
1.31E+08
1.31E+08
1.26E+08
1.9E+08
75876512
97205937
97205937
97205937
97205937
97205937
97205937
97205937
97205937
2859341
1.29E+08
1.29E+08
54078888
1.33E+08
1.14E+08
1.14E+08
1.14E+08
1.14E+08
1.14E+08
1.14E+08

1.14E+08
1.14E+08
1.31E+08
40833108
40834048
40834048
1.19E+08
70888807
70888807
70888807
70888807
24588397
24588397
24588397
28129916
28129916
55271941
55271941
1.61E+08
1.28E+08
2432914
10593680
10592925
10592925
10593680
2.22E+08
2.22E+08
2.22E+08
1.13E+08
19872209
19869164
61725671
1.13E+08
1.13E+08
1.13E+08
1.57E+08
1.57E+08
40612861
40612861
40612861
1.52E+08
1.52E+08
1.52E+08
1052353
1052345
1052619
92622671

92622671
92622671
56759037
56759037
1.49E+08
15685351
1.71E+08
1.71E+08
91570851
91567851
91570851
91570851
65137808
77818213
79823544
24591184
1.4E+08
1.4E+08
91725224
16514107
1.39E+08
1.39E+08
19550189
10839642
10839642
42310439
1.22E+08
1.37E+08
1.07E+08
47497267
47497267
47497220
47497267
1.67E+08
1.67E+08
1.67E+08
23118273
1.47E+08
41711301
1.1E+08
1.46E+08
78125982
49438938
68300418
68300418
1.46E+08
1.46E+08

36008493
36008493
1.19E+08
94600957
32560207
20035157
20039289
20038136
67626441
2.07E+08
2.07E+08
2.07E+08
2.07E+08
2.07E+08
2.07E+08
57484676
66664531
66665602
1.25E+08
1.25E+08
1.25E+08
7147249
7147825
7147825
7142863
40568368
40568368
40568368
23815750
23815750
1.39E+08
1.39E+08
1.39E+08
1.71E+08
2425370
2425370
2425370
2425370
2422671
1.23E+08
1.23E+08
1.38E+08
1.38E+08
89536043
1.07E+08
41258758
41260828

1.36E+08
1.44E+08
45928937
1.15E+08
1.15E+08
1.15E+08
1.15E+08
1.15E+08
1.15E+08
45544297
45544297
45544297
1.23E+08
1.12E+08
708119
77289464
45023185
1.34E+08
81530953
1876812
1.16E+08
2210106
2207798
2210359
1.84E+08
1.84E+08
97303243
97303000
97303000
97303000
79874353
79874353
79874353
79874353
79874353
79874115
79874115
86318809
86627121
39900234
39900234
39900234
36536863
36536863
1.53E+08
33464765
33466327

33465661
33466327
1.59E+08
1.5E+08
1.07E+08
43897279
43897794
6823559
6826142
14774809
14777214
8075529
1.15E+08
1.15E+08
90641943
1.39E+08
1.39E+08
1.39E+08
12850529
12850305
33842062
33842062
33842062
97265683
1.92E+08
67241298
1.34E+08
1.34E+08
1.34E+08
1.34E+08
1.34E+08
1.34E+08
1.34E+08
2.42E+08
14876887
2902895
43143477
43143477
43140666
75920623
75920623
97659454
18570536
18570536
69136089
1947276
86614114

18061923
18061113
1.5E+08
1.5E+08
1.5E+08
1.5E+08
1.5E+08
1.39E+08
97854992
97854992
97854992
97854992
1.24E+08
1.24E+08
38270453
1.35E+08
1.35E+08
1.35E+08
1.35E+08
33085777
33085777
19440755
19440755
62212502
18661020
54320615
54320615
89493549
1.09E+08
1.09E+08
1817236
1817275
1817275
39304420
1.41E+08
1.41E+08
56264079
56264079
1.6E+08
30823610
30823610
1.4E+08
1.4E+08
72279467
72279467
72279467
65542775

65542833
65542833
65542833
82557151
82560104
82560104
53090944
30281157
30281157
30281157
1.39E+08
1.39E+08
1.39E+08
2.24E+08
53410041
53409404
36154144
36154144
36154144
36157015
36153090
90478971
6507709
6507709
6504676
6507709
6507709
74263285
60463302
60460142
1.83E+08
42951981
42951981
42951981
1.77E+08
1.28E+08
1.18E+08
1.24E+08
1.24E+08
1.24E+08
18269799
18269799
92398937
92398937
1.13E+08
96816179
1.67E+08

6742633
6741692
6741692
6741692
6741692
6741789
43053318
1.5E+08
40638168
1.44E+08
1.44E+08
1.62E+08
1.62E+08
1.26E+08
1.07E+08
1.39E+08
1.39E+08
1.39E+08
1.39E+08
1.39E+08
39875279
7824265
1.29E+08
1667109
1666640
41990871
35235691
89493549
46608009
1.51E+08
1.51E+08
1.51E+08
1.51E+08
1.51E+08
1.51E+08
1.79E+08
80236594
80236594
80236618
1.11E+08
1.11E+08
1.11E+08
1.11E+08
1.11E+08
18028483
18028483
88638788

88638788
1.84E+08
1.84E+08
1.84E+08
1.84E+08
1.84E+08
1.84E+08
41152445
96673134
99896380
99896380
99896380
99896380
99896380
1.37E+08
67978602
67979562
1.55E+08
43992576
43993739
43993808
43993739
89461700
89460993
89461700
89461700
89461700
54967110
54967110
54967690
54968101
54967690
54967690
54968101
1.02E+08
1.02E+08
93476759
93479703
93476759
93476759
93476759
93476759
93476759
77895155
1.67E+08
1.67E+08
1.67E+08

1.15E+08
1.15E+08
1.15E+08
1.15E+08
1.15E+08
1.15E+08
1.15E+08
1.15E+08
1.15E+08
1.15E+08
1.15E+08
1.15E+08
1.15E+08
1.9E+08
39900455
95014927
3616609
35661719
35661692
35661719
42281618
26237993
26238368
26237644
26237945
26237993
79303002
1.23E+08
1.23E+08
27719942
27719942
27719679
93376217
1.4E+08
1.4E+08
1.41E+08
1.41E+08
1.07E+08
1.32E+08
88075955
88075955
88075955
1.03E+08
1.03E+08
66558887
66558887
47867980

47867980
47867980
47867980
47867980
1593176
50106948
50106948
1.79E+08
1.79E+08
1.79E+08
1.79E+08
1.79E+08
2.31E+08
2.31E+08
2.31E+08
1.84E+08
1.84E+08
1.84E+08
1.89E+08
1.53E+08
1.22E+08
63835260
2.37E+08
2.37E+08
18482887
37028111
37028111
37028111
1.3E+08
1.3E+08
1.65E+08
95738717
39454544
39450179
39450179
39451881
27240455
2.05E+08
62220807
6629880
17416540
17416540
1.04E+08
1.04E+08
1.04E+08
1.04E+08
1.04E+08

1.04E+08
1.04E+08
1.04E+08
1.04E+08
1.54E+08
1.55E+08
1.65E+08
1.12E+08
1.12E+08
49718970
49718970
49718970
49723359
68230185
68230185
68230185
68230185
68230185
68230185
1.23E+08
1.4E+08
1.34E+08
4636702
4636702
1.26E+08
1.26E+08
1.26E+08
53167649
78507559
78507559
78507559
2.09E+08
20069754
20069754
38383177
1.24E+08
1.24E+08
1.24E+08
1.24E+08
1.24E+08
1.24E+08
1.24E+08
3562582
3562582
2.15E+08
15709952
92497532

20010591
20009309
20010591
20010591
20010591
20010591
20010591
20006522
20010591
20010591
20010591
20010591
20006522
20010591
1.85E+08
87987037
87987037
87987037
87987037
87987037
67577807
48942879
97876542
97876549
97876549
1.01E+08
90100643
90103453
40252133
5512544
8027234
8026860
1.54E+08
1.54E+08
1.54E+08
1.54E+08
1.54E+08
1.21E+08
1.21E+08
99743050
99743050
99743050
99743050
1.21E+08
18265987
18265987
50735260

1.31E+08
1.31E+08
1.25E+08
39775166
39395502
39395502
39392339
39392569
39392569
39395463
30992927
30992927
13049558
13051413
13053297
15996167
1.15E+08
2935195
2935195
2935195
64581126
64581126
64583927
64581126
50174132
50174132
50170398
50170398
50174132
50174132
50174132
50174132
50170398
50174132
50174132
50170398
50174132
50170398
50170398
50170398
50170398
50170398
50170398
50170398
50170398
50170398
1.53E+08

15097145
18167230
18165847
18167230
18166870
18165847
18166423
1.32E+08
4793727
12202777
6109191
2.19E+08
24802083
16941385
1.18E+08
57859270
9884410
48404784
4712395
4712395
1.55E+08
1.55E+08
1.55E+08
44075066
44075066
44075065
40312062
40312062
40312062
49499155
49501610
49501610
49501610
1.46E+08
2.48E+08
43805539
2.08E+08
74495893
74495893
74495893
1.12E+08
1.12E+08
1.12E+08
4404659
4405415
4405565
4405565

1.34E+08
27171639
27175399
1.18E+08
1.18E+08
1.18E+08
1.18E+08
1.18E+08
1.18E+08
1.18E+08
1.14E+08
1.14E+08
9048508
74028507
1.11E+08
84117979
84117979
84117979
84117979
2212069
2209236
2209236
4210381
16587823
16587823
16587823
16587823
16587823
16587823
35422137
1.87E+08
1.87E+08
1.09E+08
89183973
89183973
89183973
4274969
89166526
45763221
45764285
45764285
45764285
53452727
53453222
1.19E+08
1.27E+08
1.16E+08

1.16E+08
1.25E+08
1.25E+08
1.25E+08
1.25E+08
1.25E+08
1.25E+08
1.25E+08
1.16E+08
1.16E+08
2.21E+08
2.21E+08
27534850
1.14E+08
1.12E+08
1.12E+08
42424453
79874353
79874353
79874353
79874353
79874353
79874115
79874115
80852990
80852990
64075698
64074999
64074999
1.23E+08
1.23E+08
1.23E+08
36547782
36547782
36550664
1.4E+08
1.4E+08
1.4E+08
1.4E+08
1.4E+08
5619864
5619864
5619864
5619864
5619330
48250792
39835098

39835098
8005476
49742234
4738634
4738634
4738634
4738634
1.05E+08
12926381
12926381
12926381
1.34E+08
1.34E+08
1.34E+08
40339345
74737108
74737108
1.66E+08
1.4E+08
11648561
1.4E+08
1.4E+08
1.31E+08
1.31E+08
11487382
36991548
26190949
26190949
18157618
18160396
1.07E+08
1.07E+08
43947494
49828627
49828973
49828973
48218763
48218763
48218763
1.62E+08
1.42E+08
1.42E+08
1.83E+08
42903299
42903279
1.38E+08
1.38E+08

1.38E+08
1.38E+08
1.38E+08
1.38E+08
1.38E+08
53581415
53580848
53580620
1.2E+08
1.2E+08
1.2E+08
2589964
2589964
2589964
1.61E+08
2.42E+08
2.42E+08
2.42E+08
2.42E+08
2.42E+08
2.42E+08
2.42E+08
2.42E+08
2.42E+08
48556377
1.4E+08
1.4E+08
1.4E+08
1.4E+08
80061106
1E+08
1.81E+08
64090556
64087559
64087559
64087559
64090556
64090556

Supplementary Table 8 - SCC6 differential binding affinity between cetuximab and PBS.

seqnames	start	end	width	Fold	p.value	FDR	genename	promoterst
chr20	61863315	61864179	865	2.39	3.38E-47	1.07E-43	BIRC7	61862234
chr20	61863315	61864179	865	2.39	3.38E-47	1.07E-43	BIRC7	61862234
chr1	2.31E+08	2.31E+08	486	2.91	1.15E-36	1.82E-33	FAM89A	2.31E+08
chr2	48758263	48758798	536	1.98	1.81E-30	1.43E-27	STON1	48752307
chr2	48758263	48758798	536	1.98	1.81E-30	1.43E-27	STON1	48752307
chr2	48758263	48758798	536	1.98	1.81E-30	1.43E-27	STON1-GTF	48752063
chr7	1.38E+08	1.38E+08	408	4.28	2.03E-29	1.41E-26	SVOPL	1.38E+08
chr12	8831217	8831659	443	2.74	8.55E-28	5.31E-25	RIMKLB	8829272
chr3	1.83E+08	1.83E+08	328	3.69	9.78E-27	5.53E-24	KLHL6-AS1	1.83E+08
chr8	1.29E+08	1.29E+08	346	2.9	8.52E-26	4.39E-23	TMEM75	1.29E+08
chr17	79533487	79534131	645	2.22	1.82E-22	6.42E-20	NPLOC4	79531661
chr4	56251411	56251859	449	2.32	7.91E-22	2.63E-19	SRD5A3-AS	56249747
chr12	54656281	54656659	379	3.29	8.19E-22	2.71E-19	CBX5	54651329
chr12	54656281	54656659	379	3.29	8.19E-22	2.71E-19	CBX5	54651370
chr9	23674178	23674644	467	3.63	1.91E-21	5.89E-19	LOC10192	23670397
chr8	71522563	71522945	383	2.35	2.03E-21	6.25E-19	LACTB2-AS	71515811
chr8	71522563	71522945	383	2.35	2.03E-21	6.25E-19	TRAM1	71518694
chr8	71522563	71522945	383	2.35	2.03E-21	6.25E-19	TRAM1	71517926
chr8	71522563	71522945	383	2.35	2.03E-21	6.25E-19	TRAM1	71518694
chr11	1.01E+08	1.01E+08	416	1.46	2.95E-21	8.91E-19	TMEM133	1.01E+08
chr11	1.03E+08	1.03E+08	328	1.49	7.48E-21	2.13E-18	MMP13	1.03E+08
chr5	36156360	36156597	238	3.34	8.68E-20	2.10E-17	LMBRD2	36150015
chr1	68302428	68302745	318	2.99	1.50E-19	3.55E-17	GNG12	68297155
chr11	20072077	20072722	646	1.64	2.25E-19	5.16E-17	NAV2-AS2	20068849
chr16	75251148	75251490	343	3.22	1.20E-18	2.49E-16	CTRB1	75247883
chr8	1.28E+08	1.28E+08	772	1.08	3.39E-18	6.57E-16	CCAT2	1.28E+08
chr7	43561967	43562426	460	2.51	2.89E-17	4.80E-15	LOC10050	43560141
chr11	1.03E+08	1.03E+08	340	-1.55	4.67E-17	7.65E-15	MMP1	1.03E+08
chr11	1.03E+08	1.03E+08	340	-1.55	4.67E-17	7.65E-15	MMP1	1.03E+08
chr9	86087219	86087500	282	-2.47	4.90E-17	8.02E-15	FRMD3	86080657
chr7	7140038	7140396	359	1.69	1.45E-16	2.17E-14	LOC100131	7134417
chr17	12452799	12453283	485	2.52	1.73E-16	2.54E-14	LINC00670	12448284
chr17	12452799	12453283	485	2.52	1.73E-16	2.54E-14	LINC00670	12448284
chr8	57235354	57235609	256	-2.44	2.02E-16	2.91E-14	SDR16C5	57231335
chr8	57235354	57235609	256	-2.44	2.02E-16	2.91E-14	SDR16C5	57231335
chr8	57235354	57235609	256	-2.44	2.02E-16	2.91E-14	SDR16C5	57231335
chr16	56640609	56641552	944	1.17	2.30E-16	3.27E-14	MT2A	56637477
chr3	1.43E+08	1.43E+08	358	2.27	4.24E-16	5.77E-14	LOC100507	1.43E+08
chr13	67944073	67944403	331	4.14	9.35E-16	1.19E-13	LINC00364	67941518
chr6	1105439	1105813	375	3.69	1.24E-15	1.53E-13	LINC01622	1099567
chr6	1105439	1105813	375	3.69	1.24E-15	1.53E-13	LINC01622	1099567
chr19	58401838	58402575	738	3.7	1.49E-15	1.82E-13	ZNF814	58398442
chr7	1599440	1600050	611	1.38	1.58E-15	1.92E-13	TMEM184A	1594066
chr2	1.14E+08	1.14E+08	342	3	5.57E-15	6.19E-13	IL1F10	1.14E+08

chr8	74332070	74332552	483	-1.55	5.96E-15	6.59E-13	STAU2-AS1	74327308
chr11	7987012	7987338	327	-3.12	7.93E-15	8.53E-13	NLRP10	7983059
chr1	1.13E+08	1.13E+08	480	1.61	1.14E-14	1.19E-12	WNT2B	1.13E+08
chr1	1.13E+08	1.13E+08	480	1.61	1.14E-14	1.19E-12	WNT2B	1.13E+08
chr1	1.13E+08	1.13E+08	480	1.61	1.14E-14	1.19E-12	MIR4256	1.13E+08
chr9	12771781	12772248	468	1.62	1.95E-14	1.95E-12	LURAP1L	12770011
chr8	97505752	97506143	392	2.06	2.34E-14	2.30E-12	SDC2	97500881
chr10	11940238	11940983	746	0.96	3.02E-14	2.90E-12	PROSER2-A	11934709
chr12	1.17E+08	1.17E+08	256	3.25	3.26E-14	3.10E-12	MIR620	1.17E+08
chr2	2.11E+08	2.11E+08	229	3.51	3.38E-14	3.21E-12	ACADL	2.11E+08
chr11	19736121	19736580	460	1.6	5.47E-14	5.00E-12	NAV2	19729880
chr11	19736121	19736580	460	1.6	5.47E-14	5.00E-12	NAV2	19729880
chr11	19736121	19736580	460	1.6	5.47E-14	5.00E-12	NAV2	19729880
chr11	19736121	19736580	460	1.6	5.47E-14	5.00E-12	LOC100126	19734218
chr2	2.27E+08	2.27E+08	979	1.13	9.30E-14	8.03E-12	LOC646736	2.27E+08
chr2	30454798	30455271	474	1.97	1.15E-13	9.80E-12	LBH	30449396
chr2	74652577	74653050	474	1.81	1.42E-13	1.19E-11	C2orf81	74646757
chr2	74652577	74653050	474	1.81	1.42E-13	1.19E-11	C2orf81	74646757
chr2	74652577	74653050	474	1.81	1.42E-13	1.19E-11	C2orf81	74646757
chr10	1.05E+08	1.05E+08	325	2.35	1.48E-13	1.23E-11	WBP1L	1.05E+08
chr5	64061963	64062240	278	-2.67	1.82E-13	1.49E-11	CWC27	64059744
chr5	64061963	64062240	278	-2.67	1.82E-13	1.49E-11	CWC27	64059744
chr5	64061963	64062240	278	-2.67	1.82E-13	1.49E-11	CWC27	64059744
chr5	64061963	64062240	278	-2.67	1.82E-13	1.49E-11	CWC27	64059744
chr1	2.29E+08	2.29E+08	584	2.4	2.13E-13	1.72E-11	RAB4A	2.29E+08
chr1	2.29E+08	2.29E+08	584	2.4	2.13E-13	1.72E-11	RAB4A	2.29E+08
chr1	2.29E+08	2.29E+08	584	2.4	2.13E-13	1.72E-11	RAB4A	2.29E+08
chr1	3615686	3615963	278	3.65	2.48E-13	1.98E-11	TP73	3609609
chr8	20128489	20128801	313	2.84	2.76E-13	2.18E-11	LZTS1-AS1	20128283
chr3	1.57E+08	1.57E+08	340	2.35	4.74E-13	3.59E-11	PA2G4P4	1.57E+08
chr2	56219616	56220034	419	1.31	6.38E-13	4.73E-11	MIR216A	56214194
chr19	46583852	46584295	444	1.95	6.79E-13	5.00E-11	LOC400706	46578887
chr7	1.01E+08	1.01E+08	915	1.34	7.28E-13	5.34E-11	SERPINE1	1.01E+08
chr5	1.14E+08	1.14E+08	321	2.49	7.31E-13	5.35E-11	KCNN2	1.14E+08
chr5	1.14E+08	1.14E+08	321	2.49	7.31E-13	5.35E-11	KCNN2	1.14E+08
chr7	76052600	76053145	546	1.06	9.04E-13	6.47E-11	ZP3	76049271
chr5	1.42E+08	1.42E+08	557	2.55	1.42E-12	9.74E-11	FGF1	1.42E+08
chr5	1.42E+08	1.42E+08	557	2.55	1.42E-12	9.74E-11	FGF1	1.42E+08
chr5	1.42E+08	1.42E+08	557	2.55	1.42E-12	9.74E-11	FGF1	1.42E+08
chr5	1.42E+08	1.42E+08	557	2.55	1.42E-12	9.74E-11	FGF1	1.42E+08
chr5	1.42E+08	1.42E+08	557	2.55	1.42E-12	9.74E-11	FGF1	1.42E+08
chr5	1.42E+08	1.42E+08	557	2.55	1.42E-12	9.74E-11	FGF1	1.42E+08
chr19	29457725	29458284	560	2.32	1.72E-12	1.16E-10	LOC102724	29454374
chr19	29457725	29458284	560	2.32	1.72E-12	1.16E-10	LINC00906	29451037
chr2	1.22E+08	1.22E+08	374	1.75	2.16E-12	1.44E-10	GLI2	1.22E+08
chr20	7328100	7328444	345	2.11	2.43E-12	1.60E-10	LOC101929	7323079
chr11	61064932	61065558	627	1.52	2.55E-12	1.67E-10	VWCE	61060788

chr12	48162228	48162606	379	1.66	2.99E-12	1.94E-10	SLC48A1	48161966
chr2	69198774	69199445	672	1.05	4.04E-12	2.57E-10	GKN1	69196704
chr9	79056488	79057079	592	1.19	5.85E-12	3.60E-10	GCNT1	79051581
chr15	83410798	83411392	595	2.13	6.10E-12	3.74E-10	ACTG1P17	83406532
chr10	49608109	49608681	573	2.04	8.14E-12	4.88E-10	MAPK8	49604654
chr10	49608109	49608681	573	2.04	8.14E-12	4.88E-10	MAPK8	49604654
chr10	49608109	49608681	573	2.04	8.14E-12	4.88E-10	MAPK8	49604654
chr10	49608109	49608681	573	2.04	8.14E-12	4.88E-10	MAPK8	49604654
chr19	57789813	57790280	468	0.97	8.53E-12	5.10E-10	ZNF460	57786852
chr14	24099174	24099501	328	2.39	9.64E-12	5.71E-10	DHRS2	24094323
chr3	1.9E+08	1.9E+08	445	1.4	1.32E-11	7.56E-10	P3H2-AS1	1.9E+08
chr12	741277	741846	570	1.51	1.34E-11	7.65E-10	LOC10004(	735056
chr1	54481946	54482381	436	-1.78	1.34E-11	7.67E-10	LDLRAD1	54481859
chr1	54481946	54482381	436	-1.78	1.34E-11	7.67E-10	LDLRAD1	54481859
chr1	54481946	54482381	436	-1.78	1.34E-11	7.67E-10	LDLRAD1	54481859
chr1	54481946	54482381	436	-1.78	1.34E-11	7.67E-10	LDLRAD1	54481859
chr1	54481946	54482381	436	-1.78	1.34E-11	7.67E-10	LDLRAD1	54481859
chr22	22988325	22988721	397	4.09	1.36E-11	7.76E-10	GGTLC2	22983781
chr22	22988325	22988721	397	4.09	1.36E-11	7.76E-10	GGTLC2	22982090
chr22	22988325	22988721	397	4.09	1.36E-11	7.76E-10	POM121L1	22985012
chr8	15394511	15394820	310	2.94	1.63E-11	9.13E-10	TUSC3	15392595
chr8	15394511	15394820	310	2.94	1.63E-11	9.13E-10	TUSC3	15392595
chr8	39439294	39439574	281	2.31	1.92E-11	1.06E-09	ADAM18	39437086
chr8	39439294	39439574	281	2.31	1.92E-11	1.06E-09	ADAM18	39437086
chr7	41017150	41017746	597	-1.72	2.22E-11	1.21E-09	LINC01450	41017537
chr10	76967873	76968288	416	1.65	2.26E-11	1.23E-09	VDAC2	76965299
chr10	76967873	76968288	416	1.65	2.26E-11	1.23E-09	VDAC2	76965299
chr10	76967873	76968288	416	1.65	2.26E-11	1.23E-09	VDAC2	76965299
chr10	76967873	76968288	416	1.65	2.26E-11	1.23E-09	VDAC2	76964911
chr20	55901712	55902121	410	1.38	2.36E-11	1.28E-09	SPO11	55899830
chr20	55901712	55902121	410	1.38	2.36E-11	1.28E-09	SPO11	55899830
chr16	53086471	53087057	587	1.05	2.44E-11	1.32E-09	CHD9	53083944
chr8	1.45E+08	1.45E+08	303	4.4	2.57E-11	1.38E-09	MAFA-AS1	1.44E+08
chr12	1.22E+08	1.22E+08	542	-1.65	2.65E-11	1.42E-09	RHOF	1.22E+08
chr16	27327076	27327409	334	-1.73	2.68E-11	1.43E-09	IL4R	27320229
chr16	27327076	27327409	334	-1.73	2.68E-11	1.43E-09	IL4R	27320229
chr16	27327076	27327409	334	-1.73	2.68E-11	1.43E-09	IL4R	27320229
chr16	27327076	27327409	334	-1.73	2.68E-11	1.43E-09	IL4R	27320229
chr12	1.09E+08	1.09E+08	370	2.38	2.90E-11	1.54E-09	CORO1C	1.09E+08
chr11	46718834	46719091	258	4.96	3.21E-11	1.69E-09	ZNF408	46717643
chr11	46718834	46719091	258	4.96	3.21E-11	1.69E-09	ZNF408	46717316
chr15	31455205	31455652	448	2.04	3.51E-11	1.83E-09	TRPM1	31451476
chr5	1.4E+08	1.4E+08	243	-2.18	4.60E-11	2.33E-09	HBEGF	1.4E+08
chr11	89229143	89229498	356	1.66	5.06E-11	2.54E-09	NOX4	89222733
chr11	89229143	89229498	356	1.66	5.06E-11	2.54E-09	NOX4	89222146
chr11	89229143	89229498	356	1.66	5.06E-11	2.54E-09	NOX4	89222733
chr11	89229143	89229498	356	1.66	5.06E-11	2.54E-09	NOX4	89223387

chr11	89229143	89229498	356	1.66	5.06E-11	2.54E-09	NOX4	89222733
chr11	89229143	89229498	356	1.66	5.06E-11	2.54E-09	NOX4	89222733
chr7	1.49E+08	1.49E+08	200	2.35	5.22E-11	2.61E-09	KRBA1	1.49E+08
chr7	1.49E+08	1.49E+08	200	2.35	5.22E-11	2.61E-09	KRBA1	1.49E+08
chr1	43208982	43209365	384	2.17	5.41E-11	2.70E-09	CLDN19	43203925
chr1	43208982	43209365	384	2.17	5.41E-11	2.70E-09	CLDN19	43203925
chr1	43208982	43209365	384	2.17	5.41E-11	2.70E-09	CLDN19	43203925
chr6	47755914	47756314	401	1.08	6.47E-11	3.20E-09	OPN5	47749803
chr7	44180929	44181209	281	3.69	6.71E-11	3.30E-09	MYL7	44178916
chr2	1.14E+08	1.14E+08	483	1.89	6.73E-11	3.31E-09	IL1F10	1.14E+08
chr2	1.14E+08	1.14E+08	483	1.89	6.73E-11	3.31E-09	IL1F10	1.14E+08
chr9	80064311	80064948	638	1.28	7.38E-11	3.59E-09	GNA14-AS1	80057564
chr10	27789900	27790119	220	2.05	7.79E-11	3.77E-09	RAB18	27788102
chr10	27789900	27790119	220	2.05	7.79E-11	3.77E-09	RAB18	27788102
chr10	27789900	27790119	220	2.05	7.79E-11	3.77E-09	RAB18	27788102
chr10	27789900	27790119	220	2.05	7.79E-11	3.77E-09	RAB18	27788102
chr10	27789900	27790119	220	2.05	7.79E-11	3.77E-09	RAB18	27788102
chr10	27789900	27790119	220	2.05	7.79E-11	3.77E-09	RAB18	27788102
chr13	77499333	77499649	317	2.19	8.30E-11	3.99E-09	BTF3P11	77497584
chr12	51742910	51743165	256	2.95	9.47E-11	4.52E-09	CELA1	51738463
chr2	43268976	43269435	460	1.24	1.09E-10	5.13E-09	LOC10272[illegible]	43264682
chr1	1.66E+08	1.66E+08	254	1.71	1.09E-10	5.13E-09	MIR3658	1.66E+08
chr2	86121012	86121361	350	3.45	1.20E-10	5.59E-09	ST3GAL5	86114157
chr16	31524463	31524774	312	1.92	1.24E-10	5.75E-09	C16orf58	31517740
chr3	1.36E+08	1.36E+08	336	1.99	1.30E-10	6.01E-09	PPP2R3A	1.36E+08
chr10	30496432	30496970	539	1.34	1.43E-10	6.54E-09	LOC10192[illegible]	30495780
chr19	17907393	17907689	297	-1.92	1.45E-10	6.63E-09	B3GNT3	17900918
chr8	1.34E+08	1.34E+08	458	2.96	2.11E-10	9.38E-09	SLA	1.34E+08
chr3	54935232	54935570	339	4.23	2.14E-10	9.48E-09	CACNA2D3	54933282
chr2	37571684	37572445	762	1.6	2.20E-10	9.74E-09	QPCT	37566752
chr1	2.49E+08	2.49E+08	321	1.5	2.32E-10	1.02E-08	MIR3124	2.49E+08
chr3	1.89E+08	1.89E+08	289	-1.57	2.46E-10	1.08E-08	TPRG1	1.89E+08
chr19	44283503	44283947	445	1.37	2.58E-10	1.13E-08	KCNN4	44283409
chr7	1.35E+08	1.35E+08	244	3.97	2.59E-10	1.14E-08	CALD1	1.35E+08
chr7	1.35E+08	1.35E+08	244	3.97	2.59E-10	1.14E-08	CALD1	1.35E+08
chr11	93450736	93450984	249	5.58	2.68E-10	1.17E-08	SCARNA9	93449679
chr15	94653893	94654328	436	1.75	2.77E-10	1.21E-08	LINC01581	94649167
chr6	40989704	40990362	659	1.69	3.42E-10	1.47E-08	LOC10192[illegible]	40989019
chr3	1.96E+08	1.96E+08	524	1.14	3.51E-10	1.50E-08	LINC00885	1.96E+08
chr12	51236101	51236935	835	1.15	3.68E-10	1.56E-08	TMPRSS12	51231700
chr11	1.03E+08	1.03E+08	454	0.82	4.46E-10	1.86E-08	WTAPP1	1.03E+08
chr11	1.03E+08	1.03E+08	454	0.82	4.46E-10	1.86E-08	MMP10	1.03E+08
chr6	47667794	47668184	391	1.08	4.54E-10	1.89E-08	ADGRF4	47661288
chr22	27579401	27579780	380	4.53	4.59E-10	1.91E-08	LOC10537[illegible]	27578477
chr20	31098601	31099214	614	1.86	5.12E-10	2.11E-08	LOC10192[illegible]	31092761
chr2	2.16E+08	2.16E+08	717	1.25	5.55E-10	2.27E-08	FN1	2.16E+08
chr2	2.16E+08	2.16E+08	717	1.25	5.55E-10	2.27E-08	FN1	2.16E+08

chr2	2.16E+08	2.16E+08	717	1.25	5.55E-10	2.27E-08	FN1	2.16E+08
chr2	2.16E+08	2.16E+08	717	1.25	5.55E-10	2.27E-08	FN1	2.16E+08
chr2	2.16E+08	2.16E+08	717	1.25	5.55E-10	2.27E-08	FN1	2.16E+08
chr2	2.16E+08	2.16E+08	717	1.25	5.55E-10	2.27E-08	FN1	2.16E+08
chr2	2.16E+08	2.16E+08	717	1.25	5.55E-10	2.27E-08	FN1	2.16E+08
chr2	2.16E+08	2.16E+08	717	1.25	5.55E-10	2.27E-08	FN1	2.16E+08
chr2	2.16E+08	2.16E+08	717	1.25	5.55E-10	2.27E-08	FN1	2.16E+08
chr2	2.16E+08	2.16E+08	717	1.25	5.55E-10	2.27E-08	FN1	2.16E+08
chr2	62899801	62900114	314	2.45	6.13E-10	2.48E-08	EHBP1	62895985
chr1	46684183	46684632	450	2.24	7.47E-10	2.96E-08	POMGNT1	46683977
chr1	13913836	13914314	479	2.07	7.74E-10	3.05E-08	PDPN	13906966
chr1	13913836	13914314	479	2.07	7.74E-10	3.05E-08	PDPN	13906966
chr3	1.5E+08	1.5E+08	510	1.36	7.77E-10	3.06E-08	LINC01213	1.5E+08
chr3	1.5E+08	1.5E+08	510	1.36	7.77E-10	3.06E-08	LINC01213	1.5E+08
chr10	44353064	44353334	271	1.56	8.10E-10	3.17E-08	LINC00840	44349858
chr1	41952007	41952333	327	1.89	8.15E-10	3.18E-08	EDN2	41948354
chr1	41952007	41952333	327	1.89	8.15E-10	3.18E-08	EDN2	41948354
chr1	41952007	41952333	327	1.89	8.15E-10	3.18E-08	EDN2	41948354
chr3	1.6E+08	1.6E+08	216	2.36	8.35E-10	3.25E-08	TRIM59	1.6E+08
chr10	88718658	88719244	587	0.92	9.77E-10	3.74E-08	MMRN2	88715425
chr10	88718658	88719244	587	0.92	9.77E-10	3.74E-08	SNCG	88713287
chr6	1.55E+08	1.55E+08	544	1.38	1.01E-09	3.85E-08	TIAM2	1.55E+08
chr2	1.07E+08	1.07E+08	254	2.25	1.02E-09	3.88E-08	UXS1	1.07E+08
chr2	1.07E+08	1.07E+08	254	2.25	1.02E-09	3.88E-08	UXS1	1.07E+08
chr2	1.07E+08	1.07E+08	254	2.25	1.02E-09	3.88E-08	UXS1	1.07E+08
chr10	1.04E+08	1.04E+08	505	-1.31	1.02E-09	3.90E-08	TRIM8	1.04E+08
chr11	94296445	94297102	658	1.89	1.08E-09	4.09E-08	PIWIL4	94295473
chr5	1.39E+08	1.39E+08	317	1.94	1.20E-09	4.49E-08	CXXC5	1.39E+08
chr5	1.39E+08	1.39E+08	317	1.94	1.20E-09	4.49E-08	CXXC5	1.39E+08
chr19	51509673	51510036	364	1.5	1.59E-09	5.78E-08	KLK8	51502958
chr19	51509673	51510036	364	1.5	1.59E-09	5.78E-08	KLK8	51502958
chr19	51509673	51510036	364	1.5	1.59E-09	5.78E-08	KLK8	51502958
chr19	51509673	51510036	364	1.5	1.59E-09	5.78E-08	KLK8	51502958
chr19	51509673	51510036	364	1.5	1.59E-09	5.78E-08	KLK8	51502958
chr19	51509673	51510036	364	1.5	1.59E-09	5.78E-08	KLK8	51502958
chr8	1.25E+08	1.25E+08	201	2.75	1.85E-09	6.65E-08	LOC101927	1.25E+08
chr8	1.29E+08	1.29E+08	230	2.48	2.16E-09	7.65E-08	MIR1208	1.29E+08
chr14	21510078	21510570	493	1.45	2.17E-09	7.68E-08	RNASE7	21505384
chr7	1196390	1196755	366	-1.28	2.18E-09	7.71E-08	LOC101927	1195420
chr7	1196390	1196755	366	-1.28	2.18E-09	7.71E-08	LOC101927	1195009
chr7	1196390	1196755	366	-1.28	2.18E-09	7.71E-08	LOC101927	1195009
chr7	1196390	1196755	366	-1.28	2.18E-09	7.71E-08	LOC101927	1195009
chr7	1196390	1196755	366	-1.28	2.18E-09	7.71E-08	LOC101927	1195009
chr7	1196390	1196755	366	-1.28	2.18E-09	7.71E-08	LOC101927	1195009
chr3	1.5E+08	1.5E+08	410	1.43	2.32E-09	8.13E-08	LOC101928	1.5E+08
chr2	1.97E+08	1.97E+08	750	1.07	2.39E-09	8.35E-08	LOC101927	1.97E+08
chr2	1.97E+08	1.97E+08	750	1.07	2.39E-09	8.35E-08	LOC101927	1.97E+08

chr7	1.51E+08	1.51E+08	295	-2.18	2.42E-09	8.45E-08	NOS3	1.51E+08
chr7	1.51E+08	1.51E+08	295	-2.18	2.42E-09	8.45E-08	NOS3	1.51E+08
chr7	1.51E+08	1.51E+08	295	-2.18	2.42E-09	8.45E-08	NOS3	1.51E+08
chr7	1.51E+08	1.51E+08	295	-2.18	2.42E-09	8.45E-08	NOS3	1.51E+08
chr17	20839125	20839392	268	2.35	2.47E-09	8.61E-08	LOC33926(	20836832
chr1	36616130	36616784	655	0.94	2.56E-09	8.89E-08	MAP7D1	36616565
chr1	36616130	36616784	655	0.94	2.56E-09	8.89E-08	MAP7D1	36616565
chr1	36616130	36616784	655	0.94	2.56E-09	8.89E-08	TRAPPC3	36613115
chr1	36616130	36616784	655	0.94	2.56E-09	8.89E-08	TRAPPC3	36613115
chr1	36616130	36616784	655	0.94	2.56E-09	8.89E-08	TRAPPC3	36613115
chr1	36616130	36616784	655	0.94	2.56E-09	8.89E-08	TRAPPC3	36613115
chr1	36616130	36616784	655	0.94	2.56E-09	8.89E-08	TRAPPC3	36613115
chr1	36616130	36616784	655	0.94	2.56E-09	8.89E-08	MAP7D1	36616565
chr1	36616130	36616784	655	0.94	2.56E-09	8.89E-08	TRAPPC3	36613115
chr21	27512492	27512890	399	-1.61	2.68E-09	9.24E-08	APP	27510708
chr22	23519466	23519865	400	1.15	2.82E-09	9.68E-08	BCR	23517551
chr22	23519466	23519865	400	1.15	2.82E-09	9.68E-08	BCR	23517551
chr3	99721681	99722147	467	1.4	2.96E-09	1.01E-07	MIR548G	99715059
chr5	86409682	86410006	325	1.58	2.97E-09	1.01E-07	MIR4280	86408771
chr8	1.28E+08	1.28E+08	334	-1.25	3.46E-09	1.17E-07	PRNCR1	1.28E+08
chr8	1.28E+08	1.28E+08	334	-1.25	3.46E-09	1.17E-07	PCAT2	1.28E+08
chr17	55978724	55979011	288	1.68	4.08E-09	1.36E-07	CUEDC1	55978750
chr3	1.96E+08	1.96E+08	353	1.56	4.45E-09	1.46E-07	LINC00885	1.96E+08
chr7	43944801	43945148	348	-1.26	4.53E-09	1.49E-07	URGCP-MR	43944231
chr7	43944801	43945148	348	-1.26	4.53E-09	1.49E-07	URGCP	43944668
chr7	43944801	43945148	348	-1.26	4.53E-09	1.49E-07	URGCP	43944668
chr7	43944801	43945148	348	-1.26	4.53E-09	1.49E-07	URGCP	43944668
chr9	1.25E+08	1.25E+08	231	-2.61	5.72E-09	1.84E-07	PTGS1	1.25E+08
chr9	1.25E+08	1.25E+08	231	-2.61	5.72E-09	1.84E-07	PTGS1	1.25E+08
chr9	1.25E+08	1.25E+08	231	-2.61	5.72E-09	1.84E-07	PTGS1	1.25E+08
chr9	1.25E+08	1.25E+08	231	-2.61	5.72E-09	1.84E-07	PTGS1	1.25E+08
chr9	1.25E+08	1.25E+08	231	-2.61	5.72E-09	1.84E-07	PTGS1	1.25E+08
chr9	1.25E+08	1.25E+08	231	-2.61	5.72E-09	1.84E-07	PTGS1	1.25E+08
chr6	1.47E+08	1.47E+08	267	1.47	6.52E-09	2.07E-07	ADGB	1.47E+08
chr12	41084290	41084474	185	-1.85	9.93E-09	3.01E-07	CNTN1	41081243
chr12	41084290	41084474	185	-1.85	9.93E-09	3.01E-07	CNTN1	41081243
chr16	8810466	8810779	314	1.45	9.94E-09	3.01E-07	ABAT	8809572
chr5	14660491	14660706	216	1.8	1.02E-08	3.06E-07	OTULIN	14659782
chr22	45676451	45676749	299	2.49	1.12E-08	3.34E-07	UPK3A	45675867
chr22	45676451	45676749	299	2.49	1.12E-08	3.34E-07	UPK3A	45675867
chr9	27520060	27520491	432	1.78	1.12E-08	3.34E-07	IFNK	27519311
chr14	50474200	50474501	302	-1.2	1.12E-08	3.35E-07	LINC01588	50472238
chr14	50474200	50474501	302	-1.2	1.12E-08	3.35E-07	LINC01588	50471381
chr14	50474200	50474501	302	-1.2	1.12E-08	3.35E-07	LINC01588	50471237
chr14	50474200	50474501	302	-1.2	1.12E-08	3.35E-07	LINC01588	50471381
chr19	9417148	9417370	223	2.3	1.19E-08	3.53E-07	ZNF699	9413795
chr8	75912863	75913041	179	3.21	1.21E-08	3.59E-07	CRISPLD1	75907388

chr8	75912863	75913041	179	3.21	1.21E-08	3.59E-07	CRISPLD1	75907388
chr12	47473577	47474162	586	0.85	1.23E-08	3.63E-07	PCED1B	47468385
chr12	47473577	47474162	586	0.85	1.23E-08	3.63E-07	PCED1B	47468385
chr12	47473577	47474162	586	0.85	1.23E-08	3.63E-07	AMIGO2	47471734
chr12	47473577	47474162	586	0.85	1.23E-08	3.63E-07	AMIGO2	47471734
chr12	1.09E+08	1.09E+08	384	2.63	1.26E-08	3.70E-07	CMKLR1	1.09E+08
chr6	1.34E+08	1.34E+08	280	1.79	1.31E-08	3.84E-07	SLC2A12	1.34E+08
chr19	54288491	54288776	286	1.75	1.32E-08	3.86E-07	MIR372	54286143
chr19	54288491	54288776	286	1.75	1.32E-08	3.86E-07	MIR371A	54285928
chr19	54288491	54288776	286	1.75	1.32E-08	3.86E-07	MIR373	54286958
chr1	1.81E+08	1.81E+08	461	1.61	1.33E-08	3.87E-07	OVAAL	1.81E+08
chr21	30502618	30502887	270	2.13	1.38E-08	4.01E-07	MAP3K7CL	30497805
chr6	73974202	73974462	261	1.49	1.41E-08	4.08E-07	KHDC1	73970919
chr1	1.53E+08	1.53E+08	296	2.57	1.49E-08	4.31E-07	LOC10192	1.53E+08
chr6	1.53E+08	1.53E+08	424	1.17	1.72E-08	4.90E-07	VIP	1.53E+08
chr6	1.53E+08	1.53E+08	424	1.17	1.72E-08	4.90E-07	VIP	1.53E+08
chr11	19529686	19529997	312	-1.77	1.77E-08	5.02E-07	NAV2-AS5	19526776
chr7	87563274	87563885	612	0.9	1.81E-08	5.11E-07	ADAM22	87558565
chr7	87563274	87563885	612	0.9	1.81E-08	5.11E-07	ADAM22	87558565
chr7	87563274	87563885	612	0.9	1.81E-08	5.11E-07	ADAM22	87558565
chr7	87563274	87563885	612	0.9	1.81E-08	5.11E-07	ADAM22	87558565
chr7	87563274	87563885	612	0.9	1.81E-08	5.11E-07	ADAM22	87558565
chr6	33718658	33719193	536	1.52	1.86E-08	5.22E-07	IP6K3	33712762
chr6	33718658	33719193	536	1.52	1.86E-08	5.22E-07	IP6K3	33712762
chr13	76195309	76195660	352	4.69	1.88E-08	5.27E-07	LMO7	76189569
chr20	56270082	56270480	399	0.73	1.90E-08	5.30E-07	PMEPA1	56263680
chr1	2.01E+08	2.01E+08	306	2.17	1.95E-08	5.43E-07	TNNT2	2.01E+08
chr1	2.01E+08	2.01E+08	306	2.17	1.95E-08	5.43E-07	TNNT2	2.01E+08
chr1	2.01E+08	2.01E+08	306	2.17	1.95E-08	5.43E-07	TNNT2	2.01E+08
chr1	2.01E+08	2.01E+08	306	2.17	1.95E-08	5.43E-07	TNNT2	2.01E+08
chr1	2.01E+08	2.01E+08	306	2.17	1.95E-08	5.43E-07	TNNT2	2.01E+08
chr1	2.01E+08	2.01E+08	306	2.17	1.95E-08	5.43E-07	TNNT2	2.01E+08
chr1	2.01E+08	2.01E+08	306	2.17	1.95E-08	5.43E-07	TNNT2	2.01E+08
chr11	12306711	12307066	356	1.29	2.38E-08	6.50E-07	MICALCL	12303446
chr1	1.6E+08	1.6E+08	1569	0.59	2.41E-08	6.55E-07	TAGLN2	1.6E+08
chr1	1.6E+08	1.6E+08	1569	0.59	2.41E-08	6.55E-07	TAGLN2	1.6E+08
chr1	1.6E+08	1.6E+08	1569	0.59	2.41E-08	6.55E-07	TAGLN2	1.6E+08
chr4	1.83E+08	1.83E+08	629	1.03	2.54E-08	6.86E-07	TENM3	1.83E+08
chr9	1.14E+08	1.14E+08	247	1.97	2.85E-08	7.62E-07	LPAR1	1.14E+08
chr9	1.14E+08	1.14E+08	247	1.97	2.85E-08	7.62E-07	LPAR1	1.14E+08
chr2	1.98E+08	1.98E+08	312	1.67	3.25E-08	8.58E-07	ANKRD44	1.98E+08
chr2	1.98E+08	1.98E+08	312	1.67	3.25E-08	8.58E-07	ANKRD44	1.98E+08
chr13	77464164	77464554	391	1.07	3.34E-08	8.81E-07	KCTD12	77458540
chr10	14427983	14428477	495	1.72	3.41E-08	8.96E-07	MIR4293	14423276
chr6	3061678	3062161	484	0.99	3.48E-08	9.14E-07	RIPK1	3059121
chr6	3061678	3062161	484	0.99	3.48E-08	9.14E-07	RIPK1	3059121
chr3	1.09E+08	1.09E+08	257	1.54	3.67E-08	9.58E-07	GUCA1C	1.09E+08

chr13	1.04E+08	1.04E+08	134	-2.82	3.89E-08	1.01E-06	SLC10A2	1.04E+08
chr8	1.02E+08	1.02E+08	812	0.79	3.97E-08	1.03E-06	FLJ42969	1.02E+08
chr19	30154935	30155424	490	1.23	5.25E-08	1.32E-06	PLEKHF1	30151326
chr16	74325687	74326054	368	1.25	5.39E-08	1.35E-06	PSMD7	74325672
chr9	1.32E+08	1.32E+08	316	2	5.51E-08	1.38E-06	NTMT1	1.32E+08
chr1	2.42E+08	2.42E+08	277	1.71	5.99E-08	1.49E-06	CHML	2.42E+08
chr2	1.4E+08	1.4E+08	313	1.38	6.76E-08	1.66E-06	NXPH2	1.4E+08
chr11	1.17E+08	1.17E+08	433	1.31	7.06E-08	1.73E-06	TAGLN	1.17E+08
chr11	1.17E+08	1.17E+08	433	1.31	7.06E-08	1.73E-06	TAGLN	1.17E+08
chr1	2.26E+08	2.26E+08	352	1.24	7.33E-08	1.78E-06	PYCR2	2.26E+08
chr1	2.26E+08	2.26E+08	352	1.24	7.33E-08	1.78E-06	PYCR2	2.26E+08
chr1	2.26E+08	2.26E+08	352	1.24	7.33E-08	1.78E-06	MIR6741	2.26E+08
chr1	59481473	59481797	325	1.73	7.57E-08	1.83E-06	LINC01358	59481147
chr22	23743388	23743658	271	1.38	7.63E-08	1.84E-06	ZDHHC8P1	23742799
chr6	1.1E+08	1.1E+08	273	-1.55	7.70E-08	1.86E-06	CCDC162P	1.1E+08
chr7	1.51E+08	1.51E+08	481	1.14	7.81E-08	1.88E-06	ASIC3	1.51E+08
chr7	1.51E+08	1.51E+08	481	1.14	7.81E-08	1.88E-06	ASIC3	1.51E+08
chr7	1.51E+08	1.51E+08	481	1.14	7.81E-08	1.88E-06	ASIC3	1.51E+08
chr7	1.51E+08	1.51E+08	481	1.14	7.81E-08	1.88E-06	ASIC3	1.51E+08
chr19	44257188	44257459	272	1.39	8.85E-08	2.10E-06	SMG9	44257142
chr14	51800836	51801127	292	2.7	8.99E-08	2.13E-06	LINC00640	51795110
chr1	57284829	57285057	229	2.74	9.10E-08	2.16E-06	C1orf168	57283369
chr7	2556729	2557075	347	1.16	9.40E-08	2.22E-06	LFNG	2552496
chr7	2556729	2557075	347	1.16	9.40E-08	2.22E-06	LFNG	2554478
chr7	2556729	2557075	347	1.16	9.40E-08	2.22E-06	LFNG	2554478
chr6	1.26E+08	1.26E+08	253	2.82	1.03E-07	2.41E-06	NCOA7-AS1	1.26E+08
chr6	42161244	42161585	342	1.71	1.11E-07	2.59E-06	GUCA1B	42160694
chr19	7559571	7559780	210	3.26	1.14E-07	2.65E-06	C19orf45	7557444
chr2	2.36E+08	2.36E+08	280	2.06	1.17E-07	2.72E-06	LINC01173	2.36E+08
chr1	85100320	85100892	573	0.99	1.30E-07	2.99E-06	LINC01555	85098703
chr1	41201837	41202164	328	3.31	1.35E-07	3.07E-06	NFYC	41199507
chr8	56677074	56677322	249	2.53	1.36E-07	3.11E-06	TMEM68	56674539
chr11	1.02E+08	1.02E+08	687	-0.42	1.40E-07	3.19E-06	BIRC3	1.02E+08
chr11	1.02E+08	1.02E+08	687	-0.42	1.40E-07	3.19E-06	BIRC3	1.02E+08
chr5	1.39E+08	1.39E+08	409	1.42	1.43E-07	3.23E-06	LOC10192	1.39E+08
chr5	1.39E+08	1.39E+08	409	1.42	1.43E-07	3.23E-06	LOC10192	1.39E+08
chr6	19835958	19836067	110	2.21	1.58E-07	3.54E-06	ID4	19832600
chr3	19978998	19979432	435	1.08	1.72E-07	3.81E-06	EFHB	19973706
chr9	99328736	99329378	643	-0.92	1.78E-07	3.93E-06	CDC14B	99327203
chr5	1514879	1515203	325	1.2	2.00E-07	4.37E-06	MIR6075	1508971
chr3	1.94E+08	1.94E+08	261	1.86	2.27E-07	4.89E-06	TMEM44-A	1.94E+08
chr3	1.94E+08	1.94E+08	261	1.86	2.27E-07	4.89E-06	TMEM44-A	1.94E+08
chr3	1.94E+08	1.94E+08	261	1.86	2.27E-07	4.89E-06	TMEM44-A	1.94E+08
chr21	37850568	37850978	411	1.63	2.30E-07	4.94E-06	CLDN14	37850388
chr21	37850568	37850978	411	1.63	2.30E-07	4.94E-06	CLDN14	37850388
chr8	80996388	80996968	581	0.64	2.40E-07	5.12E-06	TPD52	80991066
chr8	80996388	80996968	581	0.64	2.40E-07	5.12E-06	TPD52	80991066

chr8	80996388	80996968	581	0.64	2.40E-07	5.12E-06	TPD52	80991066
chr8	80996388	80996968	581	0.64	2.40E-07	5.12E-06	TPD52	80991066
chr8	80996388	80996968	581	0.64	2.40E-07	5.12E-06	TPD52	80991066
chr2	2.36E+08	2.36E+08	351	1.43	2.46E-07	5.23E-06	SH3BP4	2.36E+08
chr3	1.34E+08	1.34E+08	195	3.03	2.66E-07	5.61E-06	MIR4788	1.34E+08
chr20	39317737	39318898	1162	0.57	2.69E-07	5.67E-06	MAFB	39315880
chr3	1.75E+08	1.75E+08	557	1.38	2.93E-07	6.11E-06	NAALADL2-	1.75E+08
chr14	21998755	21998988	234	3.24	3.20E-07	6.61E-06	SALL2	21992542
chr14	21998755	21998988	234	3.24	3.20E-07	6.61E-06	SALL2	21992542
chr2	2.09E+08	2.09E+08	334	1.43	3.35E-07	6.89E-06	CRYGC	2.09E+08
chr5	1.77E+08	1.77E+08	333	1.06	3.51E-07	7.17E-06	PDLIM7	1.77E+08
chr5	1.77E+08	1.77E+08	333	1.06	3.51E-07	7.17E-06	PDLIM7	1.77E+08
chr5	1.77E+08	1.77E+08	333	1.06	3.51E-07	7.17E-06	PDLIM7	1.77E+08
chr5	1.77E+08	1.77E+08	333	1.06	3.51E-07	7.17E-06	PDLIM7	1.77E+08
chr11	1.14E+08	1.14E+08	541	0.86	3.69E-07	7.51E-06	NNMT	1.14E+08
chr5	1.5E+08	1.5E+08	350	0.95	3.97E-07	8.02E-06	NDST1	1.5E+08
chr6	1.14E+08	1.14E+08	400	1	3.99E-07	8.05E-06	LOC101927	1.14E+08
chr1	1.54E+08	1.54E+08	266	3.79	4.11E-07	8.26E-06	AQP10	1.54E+08
chr1	1.54E+08	1.54E+08	266	3.79	4.11E-07	8.26E-06	ATP8B2	1.54E+08
chr1	13910319	13910745	427	2.06	4.46E-07	8.89E-06	PDPN	13905251
chr1	13910319	13910745	427	2.06	4.46E-07	8.89E-06	PDPN	13905251
chr1	13910319	13910745	427	2.06	4.46E-07	8.89E-06	PDPN	13906966
chr1	13910319	13910745	427	2.06	4.46E-07	8.89E-06	PDPN	13906966
chr4	11430309	11431103	795	-0.75	4.59E-07	9.13E-06	HS3ST1	11428537
chr5	1.32E+08	1.32E+08	235	1.74	4.65E-07	9.22E-06	8-Sep	1.32E+08
chr5	1.32E+08	1.32E+08	235	1.74	4.65E-07	9.22E-06	8-Sep	1.32E+08
chr5	1.32E+08	1.32E+08	235	1.74	4.65E-07	9.22E-06	8-Sep	1.32E+08
chr5	1.32E+08	1.32E+08	235	1.74	4.65E-07	9.22E-06	8-Sep	1.32E+08
chr5	1.32E+08	1.32E+08	235	1.74	4.65E-07	9.22E-06	8-Sep	1.32E+08
chr2	1.8E+08	1.8E+08	268	-1.26	4.65E-07	9.23E-06	SESTD1	1.8E+08
chr14	23294147	23294562	416	1.79	4.73E-07	9.37E-06	MRPL52	23294091
chr14	23294147	23294562	416	1.79	4.73E-07	9.37E-06	MRPL52	23294091
chr14	23294147	23294562	416	1.79	4.73E-07	9.37E-06	MRPL52	23294091
chr14	23294147	23294562	416	1.79	4.73E-07	9.37E-06	MRPL52	23294091
chr14	23294147	23294562	416	1.79	4.73E-07	9.37E-06	MRPL52	23294091
chr14	23294147	23294562	416	1.79	4.73E-07	9.37E-06	MRPL52	23294091
chr14	23294147	23294562	416	1.79	4.73E-07	9.37E-06	MRPL52	23294091
chr4	83675090	83675280	191	2.48	4.92E-07	9.69E-06	MIR575	83672583
chr19	35896143	35896549	407	2.77	5.08E-07	9.95E-06	LINC01531	35891508
chr11	1851915	1852322	408	1.06	5.23E-07	1.02E-05	SYT8	1850539
chr11	1851915	1852322	408	1.06	5.23E-07	1.02E-05	SYT8	1850539
chr11	1851915	1852322	408	1.06	5.23E-07	1.02E-05	SYT8	1850539
chr11	1851915	1852322	408	1.06	5.23E-07	1.02E-05	SYT8	1850539
chr12	10453807	10454064	258	1.5	5.25E-07	1.03E-05	KLRD1	10452049
chr13	96187458	96187978	521	-0.96	5.51E-07	1.07E-05	CLDN10-AS	96184164
chr6	1.5E+08	1.5E+08	200	-1.21	5.53E-07	1.07E-05	ULBP3	1.5E+08
chr10	45475783	45476061	279	1.21	5.68E-07	1.10E-05	C10orf10	45472330

chr1	1356660	1357151	492	1.32	6.33E-07	1.21E-05	TMEM88B	1356507
chr1	1356660	1357151	492	1.32	6.33E-07	1.21E-05	ANKRD65	1354824
chr1	1356660	1357151	492	1.32	6.33E-07	1.21E-05	ANKRD65	1354824
chr1	1356660	1357151	492	1.32	6.33E-07	1.21E-05	ANKRD65	1354824
chr22	45596920	45597235	316	1.56	7.11E-07	1.34E-05	MIR1249	45594900
chr2	38055285	38055591	307	1.6	7.26E-07	1.37E-05	LINC00211	38054854
chr17	5487802	5488511	710	1.21	8.06E-07	1.50E-05	NLRP1	5485832
chr17	5487802	5488511	710	1.21	8.06E-07	1.50E-05	NLRP1	5485832
chr17	5487802	5488511	710	1.21	8.06E-07	1.50E-05	NLRP1	5485832
chr17	5487802	5488511	710	1.21	8.06E-07	1.50E-05	NLRP1	5485832
chr17	5487802	5488511	710	1.21	8.06E-07	1.50E-05	NLRP1	5485832
chr3	63843118	63843349	232	2.57	8.19E-07	1.52E-05	THOC7-AS1	63841320
chr4	1.25E+08	1.25E+08	414	1.53	8.24E-07	1.53E-05	LINC01091	1.25E+08
chr12	10328888	10329146	259	1.99	8.26E-07	1.53E-05	OLR1	10322790
chr12	10328888	10329146	259	1.99	8.26E-07	1.53E-05	OLR1	10322790
chr12	10328888	10329146	259	1.99	8.26E-07	1.53E-05	TMEM52B	10326556
chr12	10328888	10329146	259	1.99	8.26E-07	1.53E-05	OLR1	10322790
chr5	77175780	77176134	355	1.34	8.32E-07	1.54E-05	LOC10192‹	77175479
chr15	43511604	43511849	246	2.25	8.43E-07	1.56E-05	EPB42	43511323
chr15	43511604	43511849	246	2.25	8.43E-07	1.56E-05	EPB42	43511323
chr4	32027772	32027999	228	2.74	8.49E-07	1.57E-05	LOC10537‹	32022044
chr3	38034213	38034472	260	-1.54	8.68E-07	1.60E-05	VILL	38030077
chr19	33789474	33789766	293	0.93	8.72E-07	1.61E-05	CEBPA-AS1	33788762
chr3	12800713	12801287	575	0.94	9.07E-07	1.66E-05	TMEM40	12798808
chr3	12800713	12801287	575	0.94	9.07E-07	1.66E-05	TMEM40	12798808
chr3	12800713	12801287	575	0.94	9.07E-07	1.66E-05	TMEM40	12798808
chr10	77054360	77055270	911	0.73	9.14E-07	1.67E-05	ZNF503-AS	77051140
chr10	77054360	77055270	911	0.73	9.14E-07	1.67E-05	ZNF503-AS	77051140
chr10	77054360	77055270	911	0.73	9.14E-07	1.67E-05	ZNF503-AS	77051140
chr10	77054360	77055270	911	0.73	9.14E-07	1.67E-05	ZNF503-AS	77051140
chr7	5630890	5631549	660	0.62	9.26E-07	1.69E-05	FSCN1	5627435
chr17	48287721	48288282	562	1.29	9.38E-07	1.71E-05	LOC10192‹	48287054
chr3	1.53E+08	1.53E+08	1810	0.46	9.76E-07	1.77E-05	RAP2B	1.53E+08
chr18	59564069	59564383	315	1.21	1.03E-06	1.85E-05	RNF152	59558304
chr1	2.1E+08	2.1E+08	952	0.66	1.09E-06	1.96E-05	MIR205	2.1E+08
chr8	79472953	79473185	233	1.41	1.13E-06	2.02E-05	PKIA-AS1	79468738
chr6	26152873	26153226	354	1.75	1.13E-06	2.02E-05	HIST1H1E	26151558
chr2	75800405	75800766	362	3.19	1.13E-06	2.03E-05	EVA1A	75794848
chr12	8974955	8975250	296	-1.21	1.14E-06	2.04E-05	A2ML1	8970067
chr8	1.44E+08	1.44E+08	524	0.93	1.17E-06	2.08E-05	C8orf31	1.44E+08
chr8	1.44E+08	1.44E+08	524	0.93	1.17E-06	2.08E-05	C8orf31	1.44E+08
chr8	1.44E+08	1.44E+08	524	0.93	1.17E-06	2.08E-05	C8orf31	1.44E+08
chr20	61423495	61423842	348	1.25	1.19E-06	2.12E-05	MRGBP	61422804
chr16	75592764	75592970	207	1.76	1.19E-06	2.12E-05	TMEM231	75588184
chr16	75592764	75592970	207	1.76	1.19E-06	2.12E-05	TMEM231	75588184
chr16	75592764	75592970	207	1.76	1.19E-06	2.12E-05	TMEM231	75588184
chr3	1.96E+08	1.96E+08	526	1.53	1.31E-06	2.30E-05	TM4SF19-T	1.96E+08

chr3	1.96E+08	1.96E+08	526	1.53	1.31E-06	2.30E-05	TM4SF19	1.96E+08
chr3	1.96E+08	1.96E+08	526	1.53	1.31E-06	2.30E-05	TM4SF19	1.96E+08
chr3	1.96E+08	1.96E+08	526	1.53	1.31E-06	2.30E-05	TM4SF19	1.96E+08
chr16	83835760	83835961	202	-1.98	1.37E-06	2.39E-05	LOC102724	83835581
chr7	22707349	22707879	531	-0.82	1.40E-06	2.44E-05	LOC401312	22703152
chr13	1.12E+08	1.12E+08	179	1.62	1.42E-06	2.48E-05	ANKRD10	1.12E+08
chr13	1.12E+08	1.12E+08	179	1.62	1.42E-06	2.48E-05	ANKRD10	1.12E+08
chr13	1.12E+08	1.12E+08	179	1.62	1.42E-06	2.48E-05	ANKRD10	1.12E+08
chr13	1.12E+08	1.12E+08	179	1.62	1.42E-06	2.48E-05	ANKRD10	1.12E+08
chr9	12817785	12817883	99	-2.59	1.52E-06	2.63E-05	LURAP1L-A	12812388
chr12	64216210	64216516	307	2.19	1.54E-06	2.65E-05	TMEM5-AS	64213936
chr2	1.06E+08	1.06E+08	1009	0.61	1.55E-06	2.68E-05	FHL2	1.06E+08
chr2	1.06E+08	1.06E+08	1009	0.61	1.55E-06	2.68E-05	FHL2	1.06E+08
chr2	1.06E+08	1.06E+08	1009	0.61	1.55E-06	2.68E-05	FHL2	1.06E+08
chr2	1.06E+08	1.06E+08	1009	0.61	1.55E-06	2.68E-05	FHL2	1.06E+08
chr2	1.06E+08	1.06E+08	1009	0.61	1.55E-06	2.68E-05	FHL2	1.06E+08
chr2	1.06E+08	1.06E+08	1009	0.61	1.55E-06	2.68E-05	FHL2	1.06E+08
chr2	1.06E+08	1.06E+08	1009	0.61	1.55E-06	2.68E-05	FHL2	1.06E+08
chr2	1.06E+08	1.06E+08	1009	0.61	1.55E-06	2.68E-05	FHL2	1.06E+08
chr9	33621103	33621383	281	1.53	1.57E-06	2.70E-05	ANXA2P2	33619222
chr2	1.61E+08	1.61E+08	980	0.86	1.64E-06	2.80E-05	LOC100505	1.61E+08
chr11	27490256	27490466	211	-1.18	1.65E-06	2.82E-05	LOC105376	27488277
chr11	27490256	27490466	211	-1.18	1.65E-06	2.82E-05	LOC105376	27488277
chr3	8542385	8542603	219	1.33	1.67E-06	2.86E-05	LMCD1-AS1	8541344
chr3	8542385	8542603	219	1.33	1.67E-06	2.86E-05	LMCD1	8538492
chr3	8542385	8542603	219	1.33	1.67E-06	2.86E-05	LMCD1	8538492
chr3	8542385	8542603	219	1.33	1.67E-06	2.86E-05	LMCD1	8538492
chr3	8542385	8542603	219	1.33	1.67E-06	2.86E-05	LMCD1	8538492
chr10	73272542	73272962	421	1.05	1.68E-06	2.86E-05	LOC102723	73269677
chr6	25263863	25264088	226	2.18	1.71E-06	2.91E-05	LOC101928	25259635
chr6	25263863	25264088	226	2.18	1.71E-06	2.91E-05	LOC101928	25259635
chr15	71984157	71984442	286	2.35	1.72E-06	2.93E-05	THSD4-AS2	71981878
chr17	41522236	41522521	286	2.29	1.74E-06	2.96E-05	MIR2117	41517173
chr6	10431686	10431907	222	1.4	1.74E-06	2.96E-05	MIR5689H(	10429561
chr22	30643061	30643270	210	1.95	1.81E-06	3.06E-05	LIF	30640840
chr22	30643061	30643270	210	1.95	1.81E-06	3.06E-05	LIF	30640840
chr11	62367450	62367636	187	2.2	1.84E-06	3.11E-05	MTA2	62367312
chr22	24974704	24975061	358	1.67	1.90E-06	3.19E-05	GGT1	24974717
chr7	1.1E+08	1.1E+08	324	-1.16	1.94E-06	3.26E-05	EIF3IP1	1.1E+08
chr7	30329959	30330272	314	-1.6	1.97E-06	3.29E-05	MIR550B1	30327506
chr7	30329959	30330272	314	-1.6	1.97E-06	3.29E-05	MIR550A1	30324409
chr5	1.51E+08	1.51E+08	233	1.59	1.97E-06	3.29E-05	FAT2	1.51E+08
chr8	10512047	10512560	514	0.86	2.00E-06	3.34E-05	RP1L1	10510617
chr2	1.71E+08	1.71E+08	438	2.01	2.08E-06	3.47E-05	MYO3B	1.71E+08
chr2	1.71E+08	1.71E+08	438	2.01	2.08E-06	3.47E-05	MYO3B	1.71E+08
chr2	1.71E+08	1.71E+08	438	2.01	2.08E-06	3.47E-05	MYO3B	1.71E+08
chr2	1.71E+08	1.71E+08	438	2.01	2.08E-06	3.47E-05	MYO3B	1.71E+08

chr2	1.71E+08	1.71E+08	438	2.01	2.08E-06	3.47E-05	MYO3B	1.71E+08
chr6	56507708	56507985	278	1.19	2.12E-06	3.52E-05	DST	56505694
chr6	56507708	56507985	278	1.19	2.12E-06	3.52E-05	DST	56505694
chr17	63054828	63055029	202	1.88	2.26E-06	3.72E-05	GNA13	63049892
chr17	63054828	63055029	202	1.88	2.26E-06	3.72E-05	GNA13	63050920
chr20	54934478	54935073	596	0.74	2.37E-06	3.89E-05	FAM210B	54928982
chr17	68066420	68066797	378	2.17	2.47E-06	4.03E-05	KCNJ16	68066365
chr17	68066420	68066797	378	2.17	2.47E-06	4.03E-05	KCNJ16	68066365
chr17	68066420	68066797	378	2.17	2.47E-06	4.03E-05	KCNJ16	68066365
chr17	68066420	68066797	378	2.17	2.47E-06	4.03E-05	KCNJ16	68066365
chr14	24732756	24733227	472	1.4	2.62E-06	4.24E-05	TGM1	24730416
chr14	27289376	27290131	756	-0.93	2.64E-06	4.28E-05	LOC101927	27289326
chr9	1.16E+08	1.16E+08	360	1.27	2.71E-06	4.37E-05	SLC31A2	1.16E+08
chr5	1.22E+08	1.22E+08	240	2.54	2.72E-06	4.39E-05	LOC101927	1.22E+08
chr1	2.07E+08	2.07E+08	185	2.09	2.74E-06	4.42E-05	C4BPB	2.07E+08
chr1	2.07E+08	2.07E+08	185	2.09	2.74E-06	4.42E-05	C4BPB	2.07E+08
chr1	2.07E+08	2.07E+08	185	2.09	2.74E-06	4.42E-05	C4BPB	2.07E+08
chr1	2.07E+08	2.07E+08	185	2.09	2.74E-06	4.42E-05	C4BPB	2.07E+08
chr1	2.07E+08	2.07E+08	185	2.09	2.74E-06	4.42E-05	C4BPB	2.07E+08
chr6	1.17E+08	1.17E+08	189	1.91	2.79E-06	4.48E-05	TSPYL4	1.17E+08
chr11	86502217	86502456	240	2.86	2.82E-06	4.53E-05	PRSS23	86497100
chr1	67628699	67628971	273	-1.53	2.83E-06	4.53E-05	IL23R	67627168
chr9	71785994	71786454	461	0.65	2.94E-06	4.69E-05	TJP2	71783970
chr9	71785994	71786454	461	0.65	2.94E-06	4.69E-05	TJP2	71783970
chr9	71785994	71786454	461	0.65	2.94E-06	4.69E-05	TJP2	71783970
chr4	36280560	36280741	182	2.84	2.97E-06	4.74E-05	DTHD1	36278236
chr4	36280560	36280741	182	2.84	2.97E-06	4.74E-05	DTHD1	36280643
chr11	1.03E+08	1.03E+08	341	0.51	3.00E-06	4.78E-05	WTAPP1	1.03E+08
chr11	1.03E+08	1.03E+08	341	0.51	3.00E-06	4.78E-05	MMP10	1.03E+08
chr10	1.06E+08	1.06E+08	338	1.54	3.04E-06	4.84E-05	ITPRIP	1.06E+08
chr1	15272122	15272328	207	2	3.10E-06	4.93E-05	KAZN	15267414
chr4	1.7E+08	1.7E+08	318	1.27	3.11E-06	4.94E-05	PALLD	1.7E+08
chr8	89339595	89340084	490	0.72	3.29E-06	5.19E-05	MMP16	89337717
chr10	1.14E+08	1.14E+08	452	-1.2	3.32E-06	5.23E-05	ACSL5	1.14E+08
chr10	1.14E+08	1.14E+08	452	-1.2	3.32E-06	5.23E-05	ACSL5	1.14E+08
chr10	1.14E+08	1.14E+08	452	-1.2	3.32E-06	5.23E-05	ACSL5	1.14E+08
chr18	3015276	3015558	283	-1.33	3.34E-06	5.25E-05	LPIN2	3009945
chr16	56279875	56280479	605	0.58	3.47E-06	5.45E-05	MIR3935	56274431
chr20	48883898	48884493	596	-1.04	3.53E-06	5.51E-05	LINC01272	48879022
chr14	24101077	24101476	400	0.96	3.65E-06	5.69E-05	DHRS2	24100572
chr14	24101077	24101476	400	0.96	3.65E-06	5.69E-05	DHRS2	24100572
chr14	24101077	24101476	400	0.96	3.65E-06	5.69E-05	DHRS2	24094323
chr3	1.71E+08	1.71E+08	319	2.18	3.66E-06	5.70E-05	SLC2A2	1.71E+08
chr3	1.71E+08	1.71E+08	319	2.18	3.66E-06	5.70E-05	SLC2A2	1.71E+08
chr3	1.71E+08	1.71E+08	319	2.18	3.66E-06	5.70E-05	SLC2A2	1.71E+08
chr1	95391226	95391530	305	1.03	3.67E-06	5.71E-05	LOC72997(	95388583
chr1	95391226	95391530	305	1.03	3.67E-06	5.71E-05	CNN3	95390779

chr1	95391226	95391530	305	1.03	3.67E-06	5.71E-05	CNN3	95390779
chr1	95391226	95391530	305	1.03	3.67E-06	5.71E-05	CNN3	95389364
chr6	1.23E+08	1.23E+08	231	1.43	3.73E-06	5.79E-05	PKIB	1.23E+08
chr1	1.54E+08	1.54E+08	360	1.33	3.79E-06	5.86E-05	TPM3	1.54E+08
chr1	1.54E+08	1.54E+08	360	1.33	3.79E-06	5.86E-05	TPM3	1.54E+08
chr1	1.54E+08	1.54E+08	360	1.33	3.79E-06	5.86E-05	MIR190B	1.54E+08
chr1	62787192	62787596	405	1.09	3.84E-06	5.93E-05	KANK4	62783083
chr12	51159369	51159668	300	1.55	4.05E-06	6.22E-05	ATF1	51152788
chr1	37937349	37937727	379	1.08	4.15E-06	6.35E-05	ZC3H12A	37935118
chr2	2.08E+08	2.08E+08	596	0.71	4.15E-06	6.36E-05	LOC101927	2.08E+08
chr10	60933285	60933507	223	1.68	4.31E-06	6.57E-05	PHYHIPL	60931347
chr10	60933285	60933507	223	1.68	4.31E-06	6.57E-05	PHYHIPL	60932226
chr5	10513781	10514154	374	2.41	4.35E-06	6.61E-05	LOC38927	10507306
chr1	1.93E+08	1.93E+08	317	1.65	4.40E-06	6.69E-05	B3GALT2	1.93E+08
chr14	1.02E+08	1.02E+08	702	-1.11	4.45E-06	6.74E-05	LINC00239	1.02E+08
chr6	1.26E+08	1.26E+08	707	0.61	4.47E-06	6.78E-05	TPD52L1	1.26E+08
chr1	1.61E+08	1.61E+08	344	1.14	4.47E-06	6.78E-05	ARHGAP30	1.61E+08
chr1	1.61E+08	1.61E+08	344	1.14	4.47E-06	6.78E-05	ARHGAP30	1.61E+08
chr1	1.61E+08	1.61E+08	344	1.14	4.47E-06	6.78E-05	ARHGAP30	1.61E+08
chr1	1.61E+08	1.61E+08	344	1.14	4.47E-06	6.78E-05	ARHGAP30	1.61E+08
chrX	56765153	56765488	336	1.26	4.68E-06	7.05E-05	UQCRBP1	56762017
chr22	36018506	36018974	469	-0.86	4.79E-06	7.19E-05	MB	36011555
chr22	36018506	36018974	469	-0.86	4.79E-06	7.19E-05	MB	36017401
chr14	94637628	94637929	302	1.62	5.05E-06	7.55E-05	PPP4R4	94635648
chr14	94637628	94637929	302	1.62	5.05E-06	7.55E-05	PPP4R4	94635648
chr7	3340390	3341004	615	-0.7	5.25E-06	7.82E-05	SDK1	3336079
chr17	56479433	56479730	298	1.68	5.34E-06	7.94E-05	RNF43	56478082
chr20	56268042	56268659	618	0.49	5.37E-06	7.98E-05	PMEPA1	56263680
chr22	39824432	39825028	597	1.01	5.46E-06	8.10E-05	LOC10050	39823164
chr1	1.18E+08	1.18E+08	354	1.1	5.48E-06	8.13E-05	MIR942	1.18E+08
chr6	1.23E+08	1.23E+08	255	1.52	5.58E-06	8.26E-05	PKIB	1.23E+08
chr4	70725870	70726620	751	1	5.66E-06	8.35E-05	SULT1E1	70723870
chr3	1.51E+08	1.51E+08	116	1.71	5.88E-06	8.64E-05	GPR87	1.51E+08
chr1	36952359	36952854	496	-1.37	5.98E-06	8.77E-05	CSF3R	36946915
chr1	36952359	36952854	496	-1.37	5.98E-06	8.77E-05	CSF3R	36946915
chr1	36952359	36952854	496	-1.37	5.98E-06	8.77E-05	CSF3R	36946915
chr11	77738937	77739132	196	1.8	6.39E-06	9.30E-05	KCTD14	77732320
chr3	1.23E+08	1.23E+08	310	1.52	6.49E-06	9.42E-05	MYLK	1.23E+08
chr3	1.23E+08	1.23E+08	310	1.52	6.49E-06	9.42E-05	MYLK	1.23E+08
chr14	23305513	23305849	337	1.03	7.11E-06	0.000102	MMP14	23300741
chr10	82301821	82302133	313	1.25	7.13E-06	0.000102	SH2D4B	82295574
chr3	1.23E+08	1.23E+08	462	0.69	7.26E-06	0.000104	SEMA5B	1.23E+08
chr1	1.53E+08	1.53E+08	531	0.71	7.54E-06	0.000107	IVL	1.53E+08
chr13	36271429	36271948	520	1.46	7.60E-06	0.000108	LINC00445	36266660
chr13	36271429	36271948	520	1.46	7.60E-06	0.000108	LINC00445	36266863
chr10	1.06E+08	1.06E+08	299	1.02	7.79E-06	0.00011	ITPRIP	1.06E+08
chr20	17543489	17543800	312	1.33	7.88E-06	0.000111	BFSP1	17537605

chr20	17543489	17543800	312	1.33	7.88E-06	0.000111	BFSP1	17537605
chr20	17543489	17543800	312	1.33	7.88E-06	0.000111	BFSP1	17537605
chr7	1.32E+08	1.32E+08	274	1.6	8.02E-06	0.000113	PLXNA4	1.32E+08
chr7	1.32E+08	1.32E+08	274	1.6	8.02E-06	0.000113	FLJ40288	1.32E+08
chr22	31640005	31640260	256	0.97	8.27E-06	0.000116	LIMK2	31639347
chr22	31640005	31640260	256	0.97	8.27E-06	0.000116	LIMK2	31639347
chr9	1.4E+08	1.4E+08	738	0.67	9.10E-06	0.000126	CLIC3	1.4E+08
chr8	38642973	38643182	210	2.05	9.40E-06	0.00013	TACC1	38639721
chr8	38642973	38643182	210	2.05	9.40E-06	0.00013	TACC1	38639721
chr20	44633460	44633822	363	1.08	9.88E-06	0.000136	MMP9	44632546
chr1	65883501	65883694	194	1.21	1.00E-05	0.000137	LEPR	65881334
chr1	65883501	65883694	194	1.21	1.00E-05	0.000137	LEPR	65881334
chr1	65883501	65883694	194	1.21	1.00E-05	0.000137	LEPR	65881334
chr1	65883501	65883694	194	1.21	1.00E-05	0.000137	LEPROT	65881398
chr1	65883501	65883694	194	1.21	1.00E-05	0.000137	LEPROT	65881130
chr1	65883501	65883694	194	1.21	1.00E-05	0.000137	LEPROT	65881130
chr3	1.31E+08	1.31E+08	553	1.04	1.02E-05	0.000139	ATP2C1	1.31E+08
chr3	1.31E+08	1.31E+08	553	1.04	1.02E-05	0.000139	ATP2C1	1.31E+08
chr3	1.31E+08	1.31E+08	553	1.04	1.02E-05	0.000139	ATP2C1	1.31E+08
chr11	1.11E+08	1.11E+08	217	1.92	1.04E-05	0.000142	C11orf88	1.11E+08
chr11	1.11E+08	1.11E+08	217	1.92	1.04E-05	0.000142	C11orf88	1.11E+08
chr11	1.11E+08	1.11E+08	217	1.92	1.04E-05	0.000142	MIR34B	1.11E+08
chr11	1.11E+08	1.11E+08	217	1.92	1.04E-05	0.000142	MIR34C	1.11E+08
chr11	1.11E+08	1.11E+08	217	1.92	1.04E-05	0.000142	BTG4	1.11E+08
chr2	1.78E+08	1.78E+08	331	1.14	1.07E-05	0.000146	LOC10013(	1.78E+08
chr15	1.01E+08	1.01E+08	212	-1.32	1.10E-05	0.000149	SPATA41	1.01E+08
chr15	1.01E+08	1.01E+08	212	-1.32	1.10E-05	0.000149	SPATA41	1.01E+08
chr5	32176426	32176769	344	0.96	1.13E-05	0.000152	GOLPH3	32172425
chr1	68148388	68148763	376	0.91	1.16E-05	0.000156	GADD45A	68145859
chr1	68148388	68148763	376	0.91	1.16E-05	0.000156	GADD45A	68145859
chr1	68148388	68148763	376	0.91	1.16E-05	0.000156	GADD45A	68145859
chr10	52381719	52382052	334	0.89	1.23E-05	0.000164	SGMS1	52381737
chr10	52381719	52382052	334	0.89	1.23E-05	0.000164	SGMS1-AS:	52379710
chr7	20259262	20259827	566	-0.71	1.25E-05	0.000166	MACC1	20255013
chr19	14636651	14636887	237	1.21	1.26E-05	0.000168	MIR639	14635354
chr19	14636651	14636887	237	1.21	1.26E-05	0.000168	TECR	14635378
chr19	14636651	14636887	237	1.21	1.26E-05	0.000168	TECR	14635378
chr19	14636651	14636887	237	1.21	1.26E-05	0.000168	TECR	14635378
chr21	37843693	37843992	300	2.34	1.29E-05	0.000172	CLDN14	37836739
chr2	1.62E+08	1.62E+08	646	1.22	1.32E-05	0.000174	TANK	1.62E+08
chr2	1.62E+08	1.62E+08	646	1.22	1.32E-05	0.000174	TANK	1.62E+08
chr16	55602130	55602366	237	1.56	1.32E-05	0.000175	CAPNS2	55595583
chr19	49567365	49567615	251	1.37	1.34E-05	0.000178	NTF4	49565124
chr12	1.25E+08	1.25E+08	325	1.87	1.36E-05	0.000179	FAM101A	1.25E+08
chr1	1.53E+08	1.53E+08	163	4.24	1.37E-05	0.000181	C1orf68	1.53E+08
chr7	22600971	22601733	763	0.67	1.41E-05	0.000186	LOC10050(	22597955
chr5	1.57E+08	1.57E+08	558	1	1.43E-05	0.000188	ADAM19	1.57E+08

chr6	1.69E+08	1.69E+08	234	2.37	1.45E-05	0.00019	LOC10192¢	1.69E+08
chr4	71063704	71063913	210	2.46	1.53E-05	0.000199	ODAM	71057243
chr5	42993361	42993588	228	-1.16	1.56E-05	0.000203	FLJ32255	42991435
chr11	1.02E+08	1.02E+08	311	0.48	1.59E-05	0.000207	BIRC2	1.02E+08
chr11	1.02E+08	1.02E+08	311	0.48	1.59E-05	0.000207	BIRC2	1.02E+08
chr11	1.02E+08	1.02E+08	311	0.48	1.59E-05	0.000207	BIRC2	1.02E+08
chr16	21532826	21533299	474	1.21	1.61E-05	0.000209	SLC7A5P2	21529765
chr3	1.91E+08	1.91E+08	322	-1.17	1.71E-05	0.00022	OSTN-AS1	1.91E+08
chr1	62546676	62547048	373	3.13	1.74E-05	0.000224	MIR3116-2	62542528
chr19	47332508	47332755	248	1.73	1.84E-05	0.000235	SNAR-E	47331961
chr9	99182812	99183371	560	0.77	1.85E-05	0.000236	ZNF367	99178669
chr11	60691581	60692137	557	0.89	1.87E-05	0.000238	TMEM132/	60686912
chr11	60691581	60692137	557	0.89	1.87E-05	0.000238	TMEM132/	60686912
chr13	76334335	76334849	515	-0.78	1.88E-05	0.00024	LMO7	76329796
chr3	1.49E+08	1.49E+08	317	-1.12	1.90E-05	0.000242	TM4SF4	1.49E+08
chr2	1.61E+08	1.61E+08	192	1.8	1.92E-05	0.000244	BAZ2B	1.61E+08
chr2	1.61E+08	1.61E+08	192	1.8	1.92E-05	0.000244	7-Mar	1.61E+08
chr2	1.61E+08	1.61E+08	192	1.8	1.92E-05	0.000244	7-Mar	1.61E+08
chr2	1.61E+08	1.61E+08	192	1.8	1.92E-05	0.000244	7-Mar	1.61E+08
chr3	1.51E+08	1.51E+08	280	-1	1.93E-05	0.000245	AADACP1	1.51E+08
chr14	38064916	38065561	646	-0.52	2.03E-05	0.000257	TTC6	38059403
chr14	38064916	38065561	646	-0.52	2.03E-05	0.000257	FOXA1	38062325
chr18	33513999	33514261	263	-1.75	2.07E-05	0.000261	MIR3929	33512105
chr12	1.07E+08	1.07E+08	764	-0.5	2.07E-05	0.000261	LOC100287	1.07E+08
chr12	1.07E+08	1.07E+08	764	-0.5	2.07E-05	0.000261	RIC8B	1.07E+08
chr10	45459815	45460097	283	1.04	2.08E-05	0.000262	TMEM72-A	45453137
chr8	1.24E+08	1.24E+08	330	-0.94	2.12E-05	0.000266	FAM83A	1.24E+08
chr8	1.24E+08	1.24E+08	330	-0.94	2.12E-05	0.000266	FAM83A	1.24E+08
chr22	24245515	24245742	228	2.25	2.15E-05	0.000269	MIF-AS1	24239117
chr17	38085560	38085844	285	1.45	2.18E-05	0.000273	ORMDL3	38081884
chr4	84034105	84034420	316	1.1	2.26E-05	0.000281	PLAC8	84029012
chr4	84034105	84034420	316	1.1	2.26E-05	0.000281	PLAC8	84033911
chr4	84034105	84034420	316	1.1	2.26E-05	0.000281	PLAC8	84033911
chr6	1.5E+08	1.5E+08	355	-1.27	2.26E-05	0.000282	RAET1E	1.5E+08
chr6	1.5E+08	1.5E+08	355	-1.27	2.26E-05	0.000282	RAET1E	1.5E+08
chr6	1.5E+08	1.5E+08	355	-1.27	2.26E-05	0.000282	RAET1E	1.5E+08
chr6	1.5E+08	1.5E+08	355	-1.27	2.26E-05	0.000282	RAET1E	1.5E+08
chr5	1.5E+08	1.5E+08	390	1.25	2.37E-05	0.000292	LOC10254€	1.5E+08
chr1	1.12E+08	1.12E+08	580	0.92	2.45E-05	0.000301	CHI3L2	1.12E+08
chr1	1.12E+08	1.12E+08	580	0.92	2.45E-05	0.000301	CHI3L2	1.12E+08
chr7	2439576	2439963	388	0.9	2.50E-05	0.000307	CHST12	2438194
chr7	2439576	2439963	388	0.9	2.50E-05	0.000307	CHST12	2438194
chr7	2439576	2439963	388	0.9	2.50E-05	0.000307	CHST12	2438194
chr9	72289765	72290180	416	1.18	2.52E-05	0.000309	APBA1	72285275
chr3	1.11E+08	1.11E+08	315	1.06	2.53E-05	0.00031	PHLDB2	1.11E+08
chr11	9779697	9780621	925	0.75	2.57E-05	0.000315	SBF2-AS1	9774839
chr11	9779697	9780621	925	0.75	2.57E-05	0.000315	LOC44002ٰ8	9779080

chr20	62184135	62185512	1378	-0.68	2.59E-05	0.000317	C20orf195	62179372
chr19	41724550	41725219	670	0.78	2.61E-05	0.000319	AXL	41720103
chr19	41724550	41725219	670	0.78	2.61E-05	0.000319	AXL	41720103
chr1	6445838	6446209	372	-1.38	2.62E-05	0.00032	ACOT7	6443883
chr14	94494688	94495064	377	-0.96	2.69E-05	0.000328	OTUB2	94487723
chr1	15269654	15269730	77	2.58	2.92E-05	0.000351	KAZN	15267414
chr3	1.08E+08	1.08E+08	217	2.55	2.97E-05	0.000357	LINC00636	1.08E+08
chr1	1.54E+08	1.54E+08	152	3.29	2.98E-05	0.000358	AQP10	1.54E+08
chr2	1.55E+08	1.55E+08	217	2.45	3.01E-05	0.000362	GALNT13	1.55E+08
chr11	65149319	65150395	1077	0.52	3.03E-05	0.000363	SLC25A45	65148145
chr11	65149319	65150395	1077	0.52	3.03E-05	0.000363	SLC25A45	65149172
chr11	65149319	65150395	1077	0.52	3.03E-05	0.000363	SLC25A45	65147857
chr11	65149319	65150395	1077	0.52	3.03E-05	0.000363	SLC25A45	65147857
chr11	65149319	65150395	1077	0.52	3.03E-05	0.000363	SLC25A45	65148145
chr11	65149319	65150395	1077	0.52	3.03E-05	0.000363	FRMD8	65149040
chr11	65149319	65150395	1077	0.52	3.03E-05	0.000363	FRMD8	65149040
chr11	65149319	65150395	1077	0.52	3.03E-05	0.000363	FRMD8	65149040
chr1	1.66E+08	1.66E+08	214	-1.54	3.04E-05	0.000364	LRRC52	1.66E+08
chr11	27061776	27062512	737	0.83	3.04E-05	0.000365	BBOX1	27057508
chr22	26964207	26964849	643	0.6	3.09E-05	0.000369	TPST2	26959370
chr13	36429680	36430108	429	-0.98	3.12E-05	0.000373	DCLK1	36427998
chr13	36429680	36430108	429	-0.98	3.12E-05	0.000373	DCLK1	36427998
chr13	36429680	36430108	429	-0.98	3.12E-05	0.000373	DCLK1	36427998
chr3	39192427	39193342	916	0.59	3.12E-05	0.000373	CSRNP1	39193102
chr12	53301620	53302362	743	0.74	3.16E-05	0.000377	KRT8	53296868
chr10	1.35E+08	1.35E+08	283	-1.38	3.19E-05	0.000379	CYP2E1	1.35E+08
chr16	67560448	67560613	166	1.57	3.21E-05	0.000382	FAM65A	67557719
chr16	67560448	67560613	166	1.57	3.21E-05	0.000382	FAM65A	67557716
chr16	67560448	67560613	166	1.57	3.21E-05	0.000382	FAM65A	67558539
chr5	95064389	95064650	262	1.19	3.26E-05	0.000387	RHOBTB3	95061849
chr2	1.7E+08	1.7E+08	255	-1.13	3.29E-05	0.00039	BBS5	1.7E+08
chr1	95290779	95290978	200	1.07	3.29E-05	0.00039	LINC01057	95283837
chr4	14890435	14890712	278	1.99	3.44E-05	0.000406	LINC00504	14887793
chr1	2.45E+08	2.45E+08	335	-0.92	3.62E-05	0.000425	C1orf100	2.45E+08
chr1	2.45E+08	2.45E+08	335	-0.92	3.62E-05	0.000425	C1orf100	2.45E+08
chr1	2.45E+08	2.45E+08	335	-0.92	3.62E-05	0.000425	C1orf100	2.45E+08
chr3	1.11E+08	1.11E+08	303	1.03	3.63E-05	0.000426	PHLDB2	1.11E+08
chr13	1.02E+08	1.02E+08	607	-1.05	3.72E-05	0.000436	ITGBL1	1.02E+08
chr11	628569	628841	273	1.43	3.91E-05	0.000454	CDHR5	623067
chr11	628569	628841	273	1.43	3.91E-05	0.000454	CDHR5	623067
chr11	628569	628841	273	1.43	3.91E-05	0.000454	SCT	625173
chr11	628569	628841	273	1.43	3.91E-05	0.000454	CDHR5	623067
chr1	1.81E+08	1.81E+08	745	1	3.99E-05	0.000462	KIAA1614	1.81E+08
chr5	1.34E+08	1.34E+08	227	1.59	4.28E-05	0.000492	C5orf24	1.34E+08
chr5	1.34E+08	1.34E+08	227	1.59	4.28E-05	0.000492	C5orf24	1.34E+08
chr5	1.34E+08	1.34E+08	227	1.59	4.28E-05	0.000492	C5orf24	1.34E+08
chr11	28500491	28500690	200	0.98	4.40E-05	0.000504	MIR8068	28497095

chr5	17805509	17805868	360	1.04	4.45E-05	0.00051	LOC646241	17802382
chr5	17805509	17805868	360	1.04	4.45E-05	0.00051	LOC646241	17802382
chr7	2883840	2884388	549	-0.54	4.55E-05	0.000519	GNA12	2881959
chr7	2883840	2884388	549	-0.54	4.55E-05	0.000519	GNA12	2881959
chr17	72711347	72711606	260	-1.36	4.61E-05	0.000525	CD300LF	72707139
chr17	72711347	72711606	260	-1.36	4.61E-05	0.000525	CD300LF	72707139
chr17	72711347	72711606	260	-1.36	4.61E-05	0.000525	CD300LF	72707139
chr17	72711347	72711606	260	-1.36	4.61E-05	0.000525	CD300LF	72707139
chr17	72711347	72711606	260	-1.36	4.61E-05	0.000525	CD300LF	72707139
chr17	72711347	72711606	260	-1.36	4.61E-05	0.000525	CD300LF	72707139
chr17	72711347	72711606	260	-1.36	4.61E-05	0.000525	CD300LF	72707139
chr17	72711347	72711606	260	-1.36	4.61E-05	0.000525	CD300LF	72707139
chr15	86860670	86860857	188	-2.57	4.62E-05	0.000526	AGBL1-AS1	86858199
chr2	99797321	99797989	669	0.78	4.74E-05	0.000538	MITD1	99795492
chr2	99797321	99797989	669	0.78	4.74E-05	0.000538	MRPL30	99792541
chr2	99797321	99797989	669	0.78	4.74E-05	0.000538	MRPL30	99792541
chr2	2.11E+08	2.11E+08	232	1.61	4.80E-05	0.000544	MYL1	2.11E+08
chr6	52537326	52537536	211	-1.04	4.82E-05	0.000546	TMEM14A	52530883
chr13	99849140	99849616	477	-0.84	4.84E-05	0.000547	UBAC2	99847678
chr13	99849140	99849616	477	-0.84	4.84E-05	0.000547	UBAC2	99848162
chr13	99849140	99849616	477	-0.84	4.84E-05	0.000547	UBAC2	99848162
chr12	1.02E+08	1.02E+08	200	0.94	5.06E-05	0.000569	SPIC	1.02E+08
chr1	1.57E+08	1.57E+08	477	0.75	5.10E-05	0.000573	BCAN	1.57E+08
chr1	1.57E+08	1.57E+08	477	0.75	5.10E-05	0.000573	BCAN	1.57E+08
chr10	1.21E+08	1.21E+08	180	1.29	5.11E-05	0.000573	NANOS1	1.21E+08
chr7	1.51E+08	1.51E+08	714	0.51	5.28E-05	0.000591	AGAP3	1.51E+08
chr9	1.4E+08	1.4E+08	146	-1.13	5.33E-05	0.000595	RNF208	1.4E+08
chr9	1.4E+08	1.4E+08	146	-1.13	5.33E-05	0.000595	RNF224	1.4E+08
chr9	1.4E+08	1.4E+08	146	-1.13	5.33E-05	0.000595	CYSRT1	1.4E+08
chr3	49841349	49841461	113	2.41	5.36E-05	0.000598	FAM212A	49835686
chr3	49841349	49841461	113	2.41	5.36E-05	0.000598	CDHR4	49835268
chr6	17389644	17389987	344	0.88	5.47E-05	0.000609	CAP2	17388735
chr8	332877	333161	285	1.65	5.72E-05	0.000633	FAM87A	331174
chr20	43883069	43883718	650	-0.77	5.78E-05	0.000639	SLPI	43881205
chr18	61254249	61254509	261	1.73	5.79E-05	0.00064	SERPINB13	61249533
chr18	61254249	61254509	261	1.73	5.79E-05	0.00064	SERPINB13	61249533
chr1	1.67E+08	1.67E+08	445	1.27	5.93E-05	0.000654	DUSP27	1.67E+08
chr1	1.67E+08	1.67E+08	445	1.27	5.93E-05	0.000654	GPA33	1.67E+08
chr10	99474512	99474863	352	0.89	6.04E-05	0.000663	MARVELD1	99468464
chr16	57653490	57654501	1012	0.58	6.07E-05	0.000667	ADGRG1	57648604
chr16	57653490	57654501	1012	0.58	6.07E-05	0.000667	ADGRG1	57648604
chr16	57653490	57654501	1012	0.58	6.07E-05	0.000667	ADGRG1	57648909
chr16	57653490	57654501	1012	0.58	6.07E-05	0.000667	ADGRG1	57648909
chr2	2.2E+08	2.2E+08	255	-1.65	6.11E-05	0.000671	SPEG	2.2E+08
chr17	64413497	64413716	220	2.95	6.13E-05	0.000673	PRKCA-AS1	64410972
chr3	71119008	71119195	188	1.33	6.14E-05	0.000674	FOXP1	71112074
chr6	1.09E+08	1.09E+08	337	0.81	6.22E-05	0.000681	LINC00222	1.09E+08

chr14	23446878	23447341	464	0.63	6.37E-05	0.000696	AJUBA	23444432
chr4	1.04E+08	1.04E+08	132	-2.41	6.52E-05	0.00071	LOC10192‹	1.04E+08
chr19	12897020	12897560	541	0.54	6.62E-05	0.00072	JUNB	12897309
chr10	99331409	99331801	393	0.72	6.71E-05	0.000729	ANKRD2	99327197
chr10	99331409	99331801	393	0.72	6.71E-05	0.000729	ANKRD2	99327197
chr10	99331409	99331801	393	0.72	6.71E-05	0.000729	ANKRD2	99327197
chr10	99331409	99331801	393	0.72	6.71E-05	0.000729	ANKRD2	99327197
chr6	46922887	46923187	301	1.73	6.73E-05	0.000731	ADGRF5	46920675
chr11	12309222	12309826	605	0.65	6.84E-05	0.000741	MICALCL	12303446
chr10	1.06E+08	1.06E+08	452	-1.06	7.01E-05	0.000757	ITPRIP	1.06E+08
chr2	1.99E+08	1.99E+08	388	0.53	7.01E-05	0.000757	PLCL1	1.99E+08
chr2	2.2E+08	2.2E+08	918	0.61	7.02E-05	0.000758	DNAJB2	2.2E+08
chr2	2.2E+08	2.2E+08	918	0.61	7.02E-05	0.000758	DNAJB2	2.2E+08
chr11	1.02E+08	1.02E+08	366	-0.47	7.08E-05	0.000763	MMP7	1.02E+08
chr12	27234645	27234987	343	1.23	7.08E-05	0.000763	C12orf71	27233455
chr11	1.14E+08	1.14E+08	369	1.19	7.11E-05	0.000766	ZW10	1.14E+08
chr11	1.14E+08	1.14E+08	369	1.19	7.11E-05	0.000766	CLDN25	1.14E+08
chr2	1.28E+08	1.28E+08	138	3.12	7.13E-05	0.000768	LOC10537‹	1.28E+08
chr7	55086350	55087022	673	0.58	7.32E-05	0.000787	EGFR	55081724
chr7	55086350	55087022	673	0.58	7.32E-05	0.000787	EGFR	55081724
chr7	55086350	55087022	673	0.58	7.32E-05	0.000787	EGFR	55081724
chr7	55086350	55087022	673	0.58	7.32E-05	0.000787	EGFR	55081724
chr12	1.33E+08	1.33E+08	776	0.74	7.36E-05	0.00079	ANKLE2	1.33E+08
chr2	20422964	20423407	444	0.82	7.58E-05	0.000811	SDC1	20423194
chr2	20422964	20423407	444	0.82	7.58E-05	0.000811	SDC1	20422927
chr3	87039653	87040809	1157	0.55	7.67E-05	0.00082	VGLL3	87038257
chr7	44203189	44203640	452	0.75	7.78E-05	0.00083	GCK	44196887
chr7	44203189	44203640	452	0.75	7.78E-05	0.00083	GCK	44196887
chr6	40991619	40991797	179	1.39	8.00E-05	0.000852	LOC10192‹	40989019
chr9	74920029	74920367	339	-1.32	8.05E-05	0.000856	LINC01504	74915351
chr9	74920029	74920367	339	-1.32	8.05E-05	0.000856	LINC01504	74915351
chr12	1.27E+08	1.27E+08	201	1.15	8.24E-05	0.000873	LOC10099‹	1.27E+08
chr12	1.27E+08	1.27E+08	201	1.15	8.24E-05	0.000873	LOC10099‹	1.27E+08
chr12	1.27E+08	1.27E+08	201	1.15	8.24E-05	0.000873	LOC10099‹	1.27E+08
chr20	35490424	35490738	315	0.92	8.28E-05	0.000877	SOGA1	35486276
chr20	35490424	35490738	315	0.92	8.28E-05	0.000877	SOGA1	35490087
chr8	1.2E+08	1.2E+08	1523	-0.43	8.51E-05	0.000897	SAMD12	1.2E+08
chr8	1.2E+08	1.2E+08	1523	-0.43	8.51E-05	0.000897	SAMD12	1.2E+08
chr8	1.2E+08	1.2E+08	1523	-0.43	8.51E-05	0.000897	SAMD12	1.2E+08
chr8	1.2E+08	1.2E+08	1523	-0.43	8.51E-05	0.000897	SAMD12-A	1.2E+08
chr11	76495662	76495853	192	1.18	8.71E-05	0.000915	TSKU	76489284
chr11	76495662	76495853	192	1.18	8.71E-05	0.000915	LOC10192‹	76492106
chr11	76495662	76495853	192	1.18	8.71E-05	0.000915	LOC10192‹	76492106
chr11	76495662	76495853	192	1.18	8.71E-05	0.000915	TSKU	76490191
chr11	76495662	76495853	192	1.18	8.71E-05	0.000915	TSKU	76490191
chr8	1.28E+08	1.28E+08	277	2.23	8.71E-05	0.000915	PRNCR1	1.28E+08
chr3	1.96E+08	1.96E+08	224	1.3	8.82E-05	0.000926	TNK2	1.96E+08

chr10	75668855	75669186	332	-1.06	8.94E-05	0.000935	PLAU	75665861
chr10	75668855	75669186	332	-1.06	8.94E-05	0.000935	PLAU	75665861
chr3	1.3E+08	1.3E+08	238	1.43	9.22E-05	0.000962	TMCC1	1.3E+08
chr9	90477507	90477977	471	-0.85	9.72E-05	0.00101	LOC39236	90471971
chr16	60575	61175	601	-3.3	9.76E-05	0.00101	DDX11L10	56554
chr16	57125335	57125683	349	1.04	9.94E-05	0.00103	CPNE2	57121454
chr5	1.18E+08	1.18E+08	199	0.89	9.95E-05	0.00103	LOC10537	1.18E+08
chr17	56569857	56570228	372	0.96	9.95E-05	0.00103	HSF5	56563762
chr5	1.32E+08	1.32E+08	315	0.95	9.97E-05	0.00103	SLC22A5	1.32E+08
chr5	1.32E+08	1.32E+08	315	0.95	9.97E-05	0.00103	SLC22A5	1.32E+08
chr5	1.32E+08	1.32E+08	315	0.95	9.97E-05	0.00103	MIR3936	1.32E+08
chr21	39715576	39716057	482	2.84	0.000101	0.00104	LINC01423	39715998
chr2	1.44E+08	1.44E+08	466	-0.8	0.000101	0.00104	KYNU	1.44E+08
chr2	1.44E+08	1.44E+08	466	-0.8	0.000101	0.00104	KYNU	1.44E+08
chr2	1.44E+08	1.44E+08	466	-0.8	0.000101	0.00104	KYNU	1.44E+08
chr14	23448447	23448712	266	0.9	0.000102	0.00105	AJUBA	23444432
chr2	2.41E+08	2.41E+08	402	0.97	0.000103	0.00105	GPC1	2.41E+08
chr19	21326813	21326947	135	-1.39	0.000104	0.00107	ZNF431	21319839
chr4	1.54E+08	1.54E+08	267	-1.08	0.000105	0.00107	KIAA0922	1.54E+08
chr4	1.54E+08	1.54E+08	267	-1.08	0.000105	0.00107	KIAA0922	1.54E+08
chr3	1.49E+08	1.49E+08	1075	0.44	0.000105	0.00107	WWTR1	1.49E+08
chr3	1.49E+08	1.49E+08	1075	0.44	0.000105	0.00107	WWTR1	1.49E+08
chr3	1.49E+08	1.49E+08	1075	0.44	0.000105	0.00107	WWTR1-AS	1.49E+08
chr2	1.97E+08	1.97E+08	847	0.51	0.000107	0.00109	SLC39A10	1.97E+08
chr2	1.97E+08	1.97E+08	847	0.51	0.000107	0.00109	SLC39A10	1.97E+08
chr9	1.26E+08	1.26E+08	202	2.05	0.000108	0.0011	OR5C1	1.26E+08
chr5	1.49E+08	1.49E+08	440	1.35	0.00011	0.00112	MIR145	1.49E+08
chr5	1.49E+08	1.49E+08	440	1.35	0.00011	0.00112	MIR143	1.49E+08
chr1	1.75E+08	1.75E+08	416	0.74	0.00011	0.00112	RABGAP1L	1.75E+08
chr7	45964004	45964413	410	2.11	0.000111	0.00113	IGFBP3	45958871
chr7	45964004	45964413	410	2.11	0.000111	0.00113	IGFBP3	45958871
chr17	39678045	39678690	646	-0.74	0.000112	0.00113	KRT15	39673270
chr17	39678045	39678690	646	-0.74	0.000112	0.00113	MIR6510	39671469
chr4	1.18E+08	1.18E+08	200	1.02	0.000113	0.00115	LINC01378	1.18E+08
chr17	39780778	39781257	480	0.98	0.000114	0.00115	KRT17	39778882
chr20	56197635	56197958	324	0.77	0.000116	0.00117	ZBP1	56193632
chr20	56197635	56197958	324	0.77	0.000116	0.00117	ZBP1	56193632
chr20	56197635	56197958	324	0.77	0.000116	0.00117	ZBP1	56193632
chr20	56197635	56197958	324	0.77	0.000116	0.00117	ZBP1	56193632
chr2	45236472	45237091	620	0.54	0.000117	0.00117	SIX2	45234542
chr1	61719174	61719301	128	-2.25	0.000117	0.00117	NFIA-AS1	61717190
chrX	57168106	57168300	195	1.32	0.000118	0.00119	SPIN2A	57162058
chr17	47049642	47050096	455	0.75	0.000121	0.00121	GIP	47043955
chr2	77754374	77754573	200	0.81	0.000125	0.00124	LRRTM4	77747502
chr2	77754374	77754573	200	0.81	0.000125	0.00124	LRRTM4	77747502
chr2	77754374	77754573	200	0.81	0.000125	0.00124	LRRTM4	77747502
chr2	77754374	77754573	200	0.81	0.000125	0.00124	LRRTM4	77747494

chr16	15738155	15738347	193	1.29	0.000127	0.00126	KIAA0430	15735023
chr16	15738155	15738347	193	1.29	0.000127	0.00126	KIAA0430	15735023
chr16	15738155	15738347	193	1.29	0.000127	0.00126	KIAA0430	15735023
chr16	15738155	15738347	193	1.29	0.000127	0.00126	NDE1	15732123
chr16	15738155	15738347	193	1.29	0.000127	0.00126	MIR484	15732150
chr20	31071480	31071992	513	0.98	0.000128	0.00127	NOL4L	31069385
chr12	10456745	10457042	298	1.31	0.000128	0.00127	KLRD1	10452049
chr12	10456745	10457042	298	1.31	0.000128	0.00127	KLRD1	10455416
chr12	10456745	10457042	298	1.31	0.000128	0.00127	KLRD1	10455416
chr8	1.22E+08	1.22E+08	289	1.32	0.00013	0.00129	LOC101927	1.22E+08
chr10	1.27E+08	1.27E+08	264	1.23	0.00013	0.00129	MIR4296	1.27E+08
chr9	1.38E+08	1.38E+08	517	0.65	0.000132	0.0013	COL5A1	1.38E+08
chr9	1.38E+08	1.38E+08	517	0.65	0.000132	0.0013	COL5A1	1.38E+08
chr11	94882848	94883156	309	0.79	0.000133	0.00131	LOC101929	94878702
chr9	1.02E+08	1.02E+08	534	-0.85	0.000133	0.00131	GALNT12	1.02E+08
chr10	1.03E+08	1.03E+08	509	0.85	0.000134	0.00132	LZTS2	1.03E+08
chr10	1.03E+08	1.03E+08	509	0.85	0.000134	0.00132	LZTS2	1.03E+08
chr10	1.03E+08	1.03E+08	509	0.85	0.000134	0.00132	LZTS2	1.03E+08
chr10	1.03E+08	1.03E+08	509	0.85	0.000134	0.00132	LZTS2	1.03E+08
chr11	35551540	35552073	534	0.97	0.000135	0.00133	PAMR1	35545579
chr11	35551540	35552073	534	0.97	0.000135	0.00133	PAMR1	35545579
chr11	35551540	35552073	534	0.97	0.000135	0.00133	PAMR1	35549848
chr11	35551540	35552073	534	0.97	0.000135	0.00133	PAMR1	35545579
chr19	52191077	52191627	551	0.75	0.000136	0.00134	MIRLET7E	52191038
chr19	52191077	52191627	551	0.75	0.000136	0.00134	SPACA6	52191592
chr19	52191077	52191627	551	0.75	0.000136	0.00134	MIR99B	52190864
chr19	52191077	52191627	551	0.75	0.000136	0.00134	MIR125A	52191506
chr19	52191077	52191627	551	0.75	0.000136	0.00134	SPACA6	52187686
chr19	52191077	52191627	551	0.75	0.000136	0.00134	SPACA6	52191596
chr8	1.35E+08	1.35E+08	272	1.43	0.000136	0.00134	LOC101927	1.35E+08
chr20	30307126	30307480	355	0.63	0.000137	0.00134	ABALON	30304309
chr7	79763714	79764383	670	0.56	0.000137	0.00135	GNAI1	79759139
chr7	79763714	79764383	670	0.56	0.000137	0.00135	GNAI1	79760070
chr15	83738188	83738648	461	0.83	0.000137	0.00135	BTBD1	83734106
chr15	83738188	83738648	461	0.83	0.000137	0.00135	BTBD1	83734106
chr7	6316532	6316836	305	1.39	0.000141	0.00138	CYTH3	6310242
chr12	8833740	8834199	460	0.87	0.000141	0.00138	RIMKLB	8829272
chr1	72750222	72750924	703	0.62	0.000142	0.00139	NEGR1	72746277
chr20	23967373	23967653	281	2.97	0.000142	0.00139	GGTLC1	23965512
chr20	23967373	23967653	281	2.97	0.000142	0.00139	GGTLC1	23967416
chr3	1.94E+08	1.94E+08	528	-0.79	0.000143	0.00139	LINC00884	1.94E+08
chr5	7850107	7851340	1234	0.68	0.000144	0.0014	C5orf49	7849603
chr2	1.88E+08	1.88E+08	405	1.35	0.000147	0.00143	ZSWIM2	1.88E+08
chr19	52229305	52229686	382	0.93	0.000147	0.00143	HAS1	52225245
chr19	52229305	52229686	382	0.93	0.000147	0.00143	HAS1	52225245
chr22	24645581	24645887	307	3.01	0.000147	0.00143	GGT5	24639110
chr22	24645581	24645887	307	3.01	0.000147	0.00143	GGT5	24639110

chr22	24645581	24645887	307	3.01	0.000147	0.00143	GGT5	24639110
chr22	24645581	24645887	307	3.01	0.000147	0.00143	GGT5	24639110
chr22	24645581	24645887	307	3.01	0.000147	0.00143	GGT5	24639110
chr22	24645581	24645887	307	3.01	0.000147	0.00143	POM121L9	24642588
chr10	6246316	6246565	250	1.19	0.000153	0.00148	PFKFB3	6239839
chr10	6246316	6246565	250	1.19	0.000153	0.00148	PFKFB3	6239839
chr2	2.25E+08	2.25E+08	760	-0.45	0.000154	0.00149	CUL3	2.25E+08
chr2	2.25E+08	2.25E+08	760	-0.45	0.000154	0.00149	CUL3	2.25E+08
chr17	12568874	12569384	511	0.89	0.000154	0.00149	MYOCD	12564206
chr17	12568874	12569384	511	0.89	0.000154	0.00149	MYOCD	12564206
chr4	56416042	56416415	374	0.82	0.000155	0.0015	CLOCK	56411076
chr4	56416042	56416415	374	0.82	0.000155	0.0015	CLOCK	56410099
chr3	1.77E+08	1.77E+08	224	-1.41	0.000158	0.00152	LINC00501	1.77E+08
chr1	2.12E+08	2.12E+08	225	-1.27	0.000161	0.00155	LOC10192(	2.12E+08
chr1	2.12E+08	2.12E+08	225	-1.27	0.000161	0.00155	LOC10192(	2.12E+08
chr1	2.22E+08	2.22E+08	623	0.56	0.000164	0.00157	C1orf140	2.22E+08
chr7	39773592	39773859	268	0.76	0.000165	0.00158	LINC00265	39768166
chr2	23604040	23604256	217	-1.03	0.000165	0.00158	KLHL29	23603297
chr7	50801306	50801637	332	0.7	0.000167	0.00159	GRB10	50798050
chr7	50801306	50801637	332	0.7	0.000167	0.00159	GRB10	50798050
chr13	51793523	51793723	201	1.27	0.000168	0.0016	FAM124A	51791469
chr13	51793523	51793723	201	1.27	0.000168	0.0016	FAM124A	51791469
chr17	11982149	11982373	225	1.47	0.000169	0.00161	MIR744	11980215
chr12	91403081	91403504	424	2.09	0.000169	0.00161	EPYC	91396803
chr1	17571837	17572063	227	1.92	0.000171	0.00163	PADI3	17570592
chr6	658394	658508	115	3.36	0.000173	0.00165	HUS1B	654964
chr5	1.41E+08	1.41E+08	238	0.99	0.000174	0.00165	PCDHGB2	1.41E+08
chr5	1.41E+08	1.41E+08	238	0.99	0.000174	0.00165	PCDHGA5	1.41E+08
chr5	1.41E+08	1.41E+08	238	0.99	0.000174	0.00165	PCDHGB2	1.41E+08
chr5	1.41E+08	1.41E+08	238	0.99	0.000174	0.00165	PCDHGA5	1.41E+08
chr2	1.89E+08	1.89E+08	218	-2.44	0.000175	0.00166	MIR561	1.89E+08
chr3	1.7E+08	1.7E+08	499	-0.79	0.000178	0.00168	GPR160	1.7E+08
chr5	43560272	43560487	216	0.9	0.000178	0.00168	PAIP1	43555195
chr5	43560272	43560487	216	0.9	0.000178	0.00168	PAIP1	43555195
chr5	43560272	43560487	216	0.9	0.000178	0.00168	PAIP1	43555521
chr8	89340854	89341197	344	0.76	0.00018	0.0017	MMP16	89337717
chr20	23860492	23860915	424	0.83	0.000181	0.00171	CST5	23858380
chr5	1.73E+08	1.73E+08	112	-2.4	0.000181	0.00171	LINC01485	1.73E+08
chr1	1.52E+08	1.52E+08	705	0.65	0.000182	0.00171	MIR554	1.52E+08
chr14	92413786	92414068	283	1.35	0.000182	0.00171	FBLN5	92412046
chr2	1.1E+08	1.1E+08	606	1.04	0.000184	0.00173	EDAR	1.1E+08
chr2	1.74E+08	1.74E+08	201	0.97	0.000185	0.00174	RAPGEF4	1.74E+08
chr17	35718593	35718745	153	1.49	0.000187	0.00176	ACACA	35714059
chr5	95066656	95067083	428	0.83	0.000188	0.00176	RHOBTB3	95061849
chr19	55721490	55721828	339	-0.83	0.000188	0.00176	PTPRH	55718874
chr19	55721490	55721828	339	-0.83	0.000188	0.00176	PTPRH	55718874
chr19	10654220	10654760	541	-0.63	0.00019	0.00178	ATG4D	10649569

chr19	10654220	10654760	541	-0.63	0.00019	0.00178	ATG4D	10649569
chr19	10654220	10654760	541	-0.63	0.00019	0.00178	ATG4D	10649569
chr19	10654220	10654760	541	-0.63	0.00019	0.00178	ATG4D	10649569
chr7	16844557	16845038	482	-0.85	0.000192	0.00179	AGR2	16842738
chr1	17311222	17311548	327	1.23	0.000193	0.0018	MFAP2	17306081
chr1	17311222	17311548	327	1.23	0.000193	0.0018	MFAP2	17305173
chr1	17311222	17311548	327	1.23	0.000193	0.0018	MFAP2	17305173
chr1	17311222	17311548	327	1.23	0.000193	0.0018	MFAP2	17306081
chr1	2.24E+08	2.24E+08	125	-1.7	0.000197	0.00184	CAPN8	2.24E+08
chr11	60634491	60634758	268	1.85	2.00E-04	0.00186	ZP1	60630014
chr3	27257948	27258079	132	2.46	0.000201	0.00187	NEK10	27256014
chr3	27257948	27258079	132	2.46	0.000201	0.00187	NEK10	27256014
chr8	12802981	12803111	131	2.66	0.000201	0.00187	KIAA1456	12798182
chr10	21187895	21188101	207	2.11	0.000205	0.0019	NEBL	21184531
chr19	48763078	48763611	534	-1.52	0.000208	0.00192	CARD8	48757203
chr19	48763078	48763611	534	-1.52	0.000208	0.00192	CARD8	48757203
chr15	51202647	51202903	257	1.49	0.000209	0.00193	AP4E1	51195868
chr15	51202647	51202903	257	1.49	0.000209	0.00193	AP4E1	51195868
chr15	94649108	94649434	327	0.82	0.00021	0.00194	LINC01581	94649167
chr3	48632940	48633478	539	0.83	0.00021	0.00194	COL7A1	48630593
chr17	39781969	39782171	203	1.55	0.00021	0.00194	KRT17	39778882
chr7	96653801	96654265	465	1.07	0.000213	0.00196	DLX5	96652143
chr16	68268058	68268514	457	-0.67	0.000216	0.00199	MIR6773	68265402
chr16	68268058	68268514	457	-0.67	0.000216	0.00199	ESRP2	68268136
chr16	84152967	84153359	393	-0.88	0.000219	0.00201	MBTPS1	84148548
chrX	92928269	92928896	628	1.14	0.000221	0.00203	FAM133A	92924011
chrX	92928269	92928896	628	1.14	0.000221	0.00203	FAM133A	92924011
chrX	92928269	92928896	628	1.14	0.000221	0.00203	FAM133A	92924011
chrX	92928269	92928896	628	1.14	0.000221	0.00203	FAM133A	92924011
chrX	92928269	92928896	628	1.14	0.000221	0.00203	NAP1L3	92926682
chr12	10332373	10332797	425	1.19	0.000222	0.00204	TMEM52B	10326556
chr3	97690544	97691434	891	0.49	0.000223	0.00205	MINA	97689295
chr3	97690544	97691434	891	0.49	0.000223	0.00205	MINA	97689295
chr3	97690544	97691434	891	0.49	0.000223	0.00205	MINA	97689295
chr3	97690544	97691434	891	0.49	0.000223	0.00205	MINA	97689295
chr9	35669389	35669632	244	1.27	0.000225	0.00206	ARHGEF39	35663278
chr9	35669389	35669632	244	1.27	0.000225	0.00206	CA9	35668914
chr8	11429408	11429592	185	-2.32	0.000225	0.00206	LINC00208	11429043
chr7	1.16E+08	1.16E+08	561	0.54	0.000227	0.00207	CAV1	1.16E+08
chr7	1.16E+08	1.16E+08	561	0.54	0.000227	0.00207	CAV1	1.16E+08
chr7	1.16E+08	1.16E+08	561	0.54	0.000227	0.00207	CAV1	1.16E+08
chr7	1.16E+08	1.16E+08	561	0.54	0.000227	0.00207	CAV1	1.16E+08
chr1	35544004	35544204	201	1.26	0.000227	0.00208	ZMYM1	35539963
chr1	35544004	35544204	201	1.26	0.000227	0.00208	ZMYM1	35539963
chr1	35544004	35544204	201	1.26	0.000227	0.00208	ZMYM1	35539963
chr1	35544004	35544204	201	1.26	0.000227	0.00208	ZMYM1	35539963
chr5	1.6E+08	1.6E+08	134	1.33	0.000228	0.00209	PTTG1	1.6E+08

chr5	1.6E+08	1.6E+08	134	1.33	0.000228	0.00209	PTTG1	1.6E+08
chr5	1.6E+08	1.6E+08	134	1.33	0.000228	0.00209	SLU7	1.6E+08
chr5	1.6E+08	1.6E+08	134	1.33	0.000228	0.00209	PTTG1	1.6E+08
chr7	45925001	45925423	423	-1.03	0.000234	0.00213	IGFBP1	45922958
chr14	90420876	90421348	473	-0.53	0.000235	0.00213	EFCAB11	90419121
chr14	90420876	90421348	473	-0.53	0.000235	0.00213	TDP1	90417245
chr14	90420876	90421348	473	-0.53	0.000235	0.00213	EFCAB11	90419121
chr14	90420876	90421348	473	-0.53	0.000235	0.00213	TDP1	90417245
chr14	90420876	90421348	473	-0.53	0.000235	0.00213	EFCAB11	90418901
chr14	90420876	90421348	473	-0.53	0.000235	0.00213	EFCAB11	90418901
chr14	90420876	90421348	473	-0.53	0.000235	0.00213	EFCAB11	90419121
chr14	59104265	59105157	893	0.79	0.000237	0.00215	DACT1	59099756
chr14	59104265	59105157	893	0.79	0.000237	0.00215	DACT1	59100554
chr14	59104265	59105157	893	0.79	0.000237	0.00215	DACT1	59099756
chr1	2.35E+08	2.35E+08	201	1.13	0.000241	0.00218	LOC10192	2.35E+08
chr1	2.35E+08	2.35E+08	201	1.13	0.000241	0.00218	LOC10192	2.35E+08
chr4	71339536	71339774	239	1.87	0.000241	0.00219	MUC7	71332674
chr4	71339536	71339774	239	1.87	0.000241	0.00219	MUC7	71332833
chr4	1.83E+08	1.83E+08	285	0.91	0.000251	0.00226	TENM3	1.83E+08
chr7	1.16E+08	1.16E+08	271	0.99	0.000251	0.00226	CAV2	1.16E+08
chr7	1.16E+08	1.16E+08	271	0.99	0.000251	0.00226	CAV2	1.16E+08
chr7	1.16E+08	1.16E+08	271	0.99	0.000251	0.00226	CAV2	1.16E+08
chr7	1.16E+08	1.16E+08	271	0.99	0.000251	0.00226	CAV2	1.16E+08
chr1	2.28E+08	2.28E+08	261	1.49	0.000254	0.00229	MIR3620	2.28E+08
chr13	1.15E+08	1.15E+08	201	0.86	0.000259	0.00232	CHAMP1	1.15E+08
chr13	1.15E+08	1.15E+08	201	0.86	0.000259	0.00232	CHAMP1	1.15E+08
chr13	1.15E+08	1.15E+08	201	0.86	0.000259	0.00232	CHAMP1	1.15E+08
chrX	28516859	28517111	253	1.69	0.000259	0.00233	MIR6134	28511780
chr15	56536564	56536828	265	1	0.000263	0.00236	RFX7	56533483
chr6	1.14E+08	1.14E+08	531	0.67	0.000263	0.00236	MARCKS	1.14E+08
chr5	89316992	89317341	350	-1.02	0.000266	0.00238	MIR3660	89310537
chr2	2.08E+08	2.08E+08	283	0.95	0.000268	0.00239	LOC10192	2.08E+08
chr1	1.78E+08	1.78E+08	1313	0.53	0.000271	0.00242	RASAL2	1.78E+08
chr1	1.78E+08	1.78E+08	1313	0.53	0.000271	0.00242	RASAL2-AS	1.78E+08
chr17	38182475	38182912	438	0.98	0.000272	0.00242	MIR6884	38180662
chr17	38182475	38182912	438	0.98	0.000272	0.00242	SNORD124	38181898
chr20	15908608	15908818	211	1.55	0.000273	0.00243	LOC61326	15901959
chr3	1.5E+08	1.5E+08	150	1.06	0.000277	0.00247	SELT	1.5E+08
chr19	9731744	9732694	951	0.62	0.000284	0.00252	ZNF561	9729916
chr19	9731744	9732694	951	0.62	0.000284	0.00252	ZNF561-AS	9727157
chr1	33435206	33435506	301	1.04	0.000286	0.00253	RNF19B	33428414
chr1	33435206	33435506	301	1.04	0.000286	0.00253	RNF19B	33428414
chr1	33435206	33435506	301	1.04	0.000286	0.00253	RNF19B	33428414
chr5	1.47E+08	1.47E+08	291	1.14	0.000286	0.00253	C5orf46	1.47E+08
chr8	1.03E+08	1.03E+08	311	0.78	0.000287	0.00254	LOC10405	1.03E+08
chr7	22396488	22397592	1105	0.5	0.000289	0.00255	RAPGEF5	22394533
chr1	1.62E+08	1.62E+08	499	1.26	0.000303	0.00265	FCRLA	1.62E+08

chr1	1.62E+08	1.62E+08	499	1.26	0.000303	0.00265	FCRLA	1.62E+08
chr1	1.62E+08	1.62E+08	499	1.26	0.000303	0.00265	FCRLA	1.62E+08
chr1	1.62E+08	1.62E+08	499	1.26	0.000303	0.00265	FCRLA	1.62E+08
chr1	1.62E+08	1.62E+08	499	1.26	0.000303	0.00265	FCRLA	1.62E+08
chr1	1.62E+08	1.62E+08	499	1.26	0.000303	0.00265	FCRLA	1.62E+08
chr1	1.62E+08	1.62E+08	499	1.26	0.000303	0.00265	FCRLA	1.62E+08
chr12	49582726	49583051	326	1.36	0.000304	0.00266	TUBA1A	49581107
chr12	49582726	49583051	326	1.36	0.000304	0.00266	TUBA1A	49581107
chr12	49582726	49583051	326	1.36	0.000304	0.00266	TUBA1A	49581107
chr8	10924439	10924813	375	0.82	0.000305	0.00266	LOC10192	10922599
chr2	1.08E+08	1.08E+08	392	1.13	0.000307	0.00268	ST6GAL2	1.08E+08
chr2	1.08E+08	1.08E+08	392	1.13	0.000307	0.00268	ST6GAL2	1.08E+08
chr2	1.08E+08	1.08E+08	392	1.13	0.000307	0.00268	ST6GAL2	1.08E+08
chr3	1.7E+08	1.7E+08	392	0.64	0.000309	0.00269	SKIL	1.7E+08
chr3	1.7E+08	1.7E+08	392	0.64	0.000309	0.00269	SKIL	1.7E+08
chr3	1.7E+08	1.7E+08	392	0.64	0.000309	0.00269	SKIL	1.7E+08
chr3	1.7E+08	1.7E+08	392	0.64	0.000309	0.00269	SKIL	1.7E+08
chr19	45504548	45505545	998	-0.65	0.000311	0.00271	RELB	45499706
chr19	28284036	28285307	1272	0.36	0.000312	0.00271	LOC101927	28279374
chr19	28284036	28285307	1272	0.36	0.000312	0.00271	LOC101927	28279802
chr19	28284036	28285307	1272	0.36	0.000312	0.00271	LOC101927	28279374
chr19	28284036	28285307	1272	0.36	0.000312	0.00271	LOC101927	28279374
chr19	28284036	28285307	1272	0.36	0.000312	0.00271	LOC101927	28279802
chr19	28284036	28285307	1272	0.36	0.000312	0.00271	LINC00662	28282848
chr8	91013046	91014043	998	0.49	0.000314	0.00273	DECR1	91008579
chr11	89439422	89439621	200	1.15	0.000314	0.00273	TRIM77	89438466
chr11	89439422	89439621	200	1.15	0.000314	0.00273	TRIM77	89438466
chr6	1.16E+08	1.16E+08	390	0.68	0.000314	0.00273	LOC105377	1.16E+08
chr10	1.05E+08	1.05E+08	324	-1	0.000318	0.00276	CALHM2	1.05E+08
chr10	1.05E+08	1.05E+08	324	-1	0.000318	0.00276	CALHM2	1.05E+08
chr10	1.05E+08	1.05E+08	324	-1	0.000318	0.00276	CALHM2	1.05E+08
chr6	72113989	72114740	752	0.72	0.000325	0.00281	MIR30A	72111324
chr10	88428021	88428388	368	0.75	0.000326	0.00282	LDB3	88423205
chr10	88428021	88428388	368	0.75	0.000326	0.00282	LDB3	88423425
chr10	88428021	88428388	368	0.75	0.000326	0.00282	LDB3	88423425
chr10	88428021	88428388	368	0.75	0.000326	0.00282	LDB3	88423425
chr10	88428021	88428388	368	0.75	0.000326	0.00282	LDB3	88423320
chr10	88428021	88428388	368	0.75	0.000326	0.00282	LDB3	88423425
chr10	99343804	99344185	382	0.81	0.000334	0.00287	HOGA1	99339101
chr10	99343804	99344185	382	0.81	0.000334	0.00287	HOGA1	99339101
chr5	94952737	94953028	292	1.2	0.00034	0.00292	GPR150	94950979
chr5	1.32E+08	1.32E+08	641	-0.85	0.000341	0.00292	C5orf56	1.32E+08
chr6	37521459	37521797	339	0.67	0.000348	0.00298	MIR4462	37521198
chr22	29468634	29469078	445	-0.47	0.00035	0.00299	KREMEN1	29464065
chr22	29468634	29469078	445	-0.47	0.00035	0.00299	KREMEN1	29464065
chr6	1.07E+08	1.07E+08	493	-1.01	0.000359	0.00305	PRDM1	1.07E+08
chr8	80997396	80997821	426	0.68	0.000362	0.00307	TPD52	80991066

chr8	80997396	80997821	426	0.68	0.000362	0.00307	TPD52	80991066
chr8	80997396	80997821	426	0.68	0.000362	0.00307	TPD52	80991066
chr8	80997396	80997821	426	0.68	0.000362	0.00307	TPD52	80991066
chr8	80997396	80997821	426	0.68	0.000362	0.00307	TPD52	80991066
chr18	46479081	46479335	255	1.05	0.000362	0.00308	SMAD7	46475081
chr18	46479081	46479335	255	1.05	0.000362	0.00308	SMAD7	46475081
chr18	46479081	46479335	255	1.05	0.000362	0.00308	SMAD7	46473703
chr6	99282157	99282690	534	0.54	0.000369	0.00313	POU3F2	99277579
chr8	95231783	95232209	427	-0.77	0.000372	0.00315	CDH17	95227531
chr1	1.12E+08	1.12E+08	158	1.02	0.000372	0.00315	FAM212B	1.12E+08
chr1	1.12E+08	1.12E+08	158	1.02	0.000372	0.00315	FAM212B-/	1.12E+08
chr1	1.12E+08	1.12E+08	158	1.02	0.000372	0.00315	FAM212B-/	1.12E+08
chr1	2.07E+08	2.07E+08	345	0.93	0.000376	0.00318	IL24	2.07E+08
chr1	2.07E+08	2.07E+08	345	0.93	0.000376	0.00318	IL24	2.07E+08
chr1	2.07E+08	2.07E+08	345	0.93	0.000376	0.00318	IL24	2.07E+08
chr1	2.07E+08	2.07E+08	345	0.93	0.000376	0.00318	IL24	2.07E+08
chr6	1.53E+08	1.53E+08	320	1	0.000379	0.0032	MYCT1	1.53E+08
chr15	31456485	31456710	226	1.24	0.000381	0.00322	TRPM1	31451476
chr16	87904082	87904575	494	0.72	0.000389	0.00327	SLC7A5	87901100
chr19	41224899	41225164	266	0.75	0.000391	0.00328	ADCK4	41220790
chr19	41224899	41225164	266	0.75	0.000391	0.00328	ADCK4	41219011
chr19	41224899	41225164	266	0.75	0.000391	0.00328	ITPKC	41218007
chr11	1.03E+08	1.03E+08	177	-0.91	0.000391	0.00329	MMP8	1.03E+08
chr11	1.03E+08	1.03E+08	177	-0.91	0.000391	0.00329	MMP8	1.03E+08
chr11	1.03E+08	1.03E+08	177	-0.91	0.000391	0.00329	MMP8	1.03E+08
chr1	45308839	45309136	298	1.12	0.000392	0.00329	PTCH2	45306616
chr1	45308839	45309136	298	1.12	0.000392	0.00329	PTCH2	45306616
chr8	1.14E+08	1.14E+08	198	0.71	0.000398	0.00334	CSMD3	1.14E+08
chr4	1.39E+08	1.39E+08	475	0.75	0.000406	0.00339	LINC00499	1.39E+08
chr2	1.06E+08	1.06E+08	522	0.43	0.000407	0.0034	UTAT33	1.06E+08
chr12	53318926	53320429	1504	0.35	0.000417	0.00347	KRT8	53318253
chr18	46579334	46579663	330	1.12	0.000419	0.00348	MIR4744	46574138
chr20	42815760	42816553	794	0.5	0.00042	0.00349	JPH2	42814218
chr20	42815760	42816553	794	0.5	0.00042	0.00349	JPH2	42814218
chr2	2.01E+08	2.01E+08	500	0.69	0.000422	0.0035	SPATS2L	2.01E+08
chr2	2.01E+08	2.01E+08	500	0.69	0.000422	0.0035	SPATS2L	2.01E+08
chr2	2.01E+08	2.01E+08	500	0.69	0.000422	0.0035	SPATS2L	2.01E+08
chr3	25467963	25468161	199	2.46	0.000422	0.0035	RARB	25464753
chr3	25467963	25468161	199	2.46	0.000422	0.0035	RARB	25464753
chr3	25467963	25468161	199	2.46	0.000422	0.0035	RARB	25464753
chr3	25467963	25468161	199	2.46	0.000422	0.0035	RARB	25464753
chr3	25467963	25468161	199	2.46	0.000422	0.0035	RARB	25464753
chr3	25467963	25468161	199	2.46	0.000422	0.0035	RARB	25464753
chr3	25467963	25468161	199	2.46	0.000422	0.0035	RARB	25464753
chr14	77735586	77735832	247	1.46	0.000424	0.00352	NGB	77735655
chr17	39771699	39772004	306	1.13	0.000427	0.00354	KRT16	39767079
chr5	57878655	57878975	321	0.69	0.000428	0.00354	RAB3C	57873870

chr5	57878655	57878975	321	0.69	0.000428	0.00354	RAB3C	57873014
chr5	34686887	34687625	739	0.49	0.000434	0.00358	RAI14	34682663
chr19	46714506	46714991	486	0.82	0.000438	0.00361	DKFZp434J	46708498
chr2	2.26E+08	2.26E+08	287	-1.23	0.000438	0.00361	MIR4439	2.26E+08
chr15	33360311	33360577	267	-0.87	0.000441	0.00363	FMN1	33358233
chr5	33894745	33895026	282	0.9	0.000447	0.00367	ADAMTS12	33890124
chr3	13588303	13588599	297	1.3	0.00045	0.00369	FBLN2	13585624
chr3	13588303	13588599	297	1.3	0.00045	0.00369	FBLN2	13585624
chr12	55817408	55817608	201	1.02	0.000455	0.00372	OR6C76	55815037
chr1	1.55E+08	1.55E+08	584	0.62	0.000465	0.00379	ZBTB7B	1.55E+08
chr1	1.55E+08	1.55E+08	584	0.62	0.000465	0.00379	ZBTB7B	1.55E+08
chr1	1.55E+08	1.55E+08	584	0.62	0.000465	0.00379	ZBTB7B	1.55E+08
chr1	1.55E+08	1.55E+08	584	0.62	0.000465	0.00379	ZBTB7B	1.55E+08
chr1	1.55E+08	1.55E+08	584	0.62	0.000465	0.00379	ZBTB7B	1.55E+08
chr9	1.24E+08	1.24E+08	567	-0.64	0.000467	0.00381	PHF19	1.24E+08
chr9	1.24E+08	1.24E+08	567	-0.64	0.000467	0.00381	PHF19	1.24E+08
chr9	1.24E+08	1.24E+08	567	-0.64	0.000467	0.00381	PHF19	1.24E+08
chr1	1.62E+08	1.62E+08	696	0.87	0.00047	0.00383	OLFML2B	1.62E+08
chr1	1.62E+08	1.62E+08	696	0.87	0.00047	0.00383	OLFML2B	1.62E+08
chr10	73496097	73496202	106	-2.23	0.000477	0.00387	C10orf105	73495581
chr8	24150584	24150782	199	1.39	0.000478	0.00388	ADAM28	24146552
chr8	24150584	24150782	199	1.39	0.000478	0.00388	ADAM28	24146552
chr8	24150584	24150782	199	1.39	0.000478	0.00388	ADAM28	24146552
chr8	24150584	24150782	199	1.39	0.000478	0.00388	ADAM28	24146552
chr8	24150584	24150782	199	1.39	0.000478	0.00388	ADAM28	24146552
chr13	1.15E+08	1.15E+08	256	1.32	0.000478	0.00388	GAS6-AS2	1.15E+08
chr10	49459692	49460156	465	0.93	0.000481	0.0039	FRMPD2	49457800
chr3	64172741	64173614	874	0.93	0.000488	0.00395	PRICKLE2-A	64168219
chr5	1.39E+08	1.39E+08	180	1.19	0.000497	0.00401	CXXC5	1.39E+08
chr5	1.39E+08	1.39E+08	180	1.19	0.000497	0.00401	CXXC5	1.39E+08
chr13	80055116	80055599	484	0.46	0.000504	0.00406	NDFIP2	80050258
chr13	80055116	80055599	484	0.46	0.000504	0.00406	NDFIP2	80050258
chr13	80055116	80055599	484	0.46	0.000504	0.00406	NDFIP2-AS1	80053366
chr16	54880768	54881430	663	0.51	0.00051	0.0041	LOC101927	54880145
chr6	1.47E+08	1.47E+08	167	-1.34	0.000518	0.00416	LUADT1	1.47E+08
chr14	77425653	77425963	311	0.67	0.00052	0.00418	LINC01629	77420980
chr1	1.7E+08	1.7E+08	214	1.66	0.000522	0.00419	METTL11B	1.7E+08
chr9	1.36E+08	1.36E+08	514	-0.66	0.000526	0.00421	RALGDS	1.36E+08
chr6	1.7E+08	1.7E+08	382	-1	0.000526	0.00421	LINC01615	1.7E+08
chr6	1.7E+08	1.7E+08	382	-1	0.000526	0.00421	LINC01615	1.7E+08
chr10	68684817	68685012	196	1.16	0.000528	0.00422	LRRTM3	68680791
chr2	2.33E+08	2.33E+08	247	1.22	0.000539	0.00431	NPPC	2.33E+08
chr14	23290136	23290320	185	1.32	0.000542	0.00432	SLC7A7	23287020
chr1	46807924	46808091	168	1.06	0.000543	0.00433	NSUN4	46801849
chr1	46807924	46808091	168	1.06	0.000543	0.00433	NSUN4	46801849
chr1	1.8E+08	1.8E+08	1168	0.41	0.000554	0.00441	TOR1AIP2	1.8E+08
chr1	1.8E+08	1.8E+08	1168	0.41	0.000554	0.00441	TOR1AIP2	1.8E+08

chr1	1.8E+08	1.8E+08	1168	0.41	0.000554	0.00441	TOR1AIP2	1.8E+08
chr1	1.8E+08	1.8E+08	1168	0.41	0.000554	0.00441	TOR1AIP1	1.8E+08
chr1	1.8E+08	1.8E+08	1168	0.41	0.000554	0.00441	TOR1AIP1	1.8E+08
chr9	99620273	99620612	340	0.69	0.000559	0.00444	ZNF782	99614389
chr5	41925158	41926039	882	0.37	0.000559	0.00444	FBXO4	41920355
chr5	41925158	41926039	882	0.37	0.000559	0.00444	FBXO4	41920355
chr5	41925158	41926039	882	0.37	0.000559	0.00444	FBXO4	41920353
chr6	11234291	11234657	367	0.79	0.000563	0.00446	NEDD9	11230915
chr6	11234291	11234657	367	0.79	0.000563	0.00446	NEDD9	11230915
chr6	11234291	11234657	367	0.79	0.000563	0.00446	NEDD9	11230915
chr15	55488717	55489472	756	0.47	0.000563	0.00447	RSL24D1	55487231
chr5	40842557	40842711	155	-1.44	0.000569	0.00451	CARD6	40836409
chr2	2.33E+08	2.33E+08	511	0.66	0.000576	0.00455	ECEL1P2	2.33E+08
chr3	1.51E+08	1.51E+08	372	0.61	0.000579	0.00457	GPR87	1.51E+08
chr7	77325444	77326388	945	0.42	0.000581	0.00459	RSBN1L	77320742
chr7	77325444	77326388	945	0.42	0.000581	0.00459	APTR	77324662
chr7	77325444	77326388	945	0.42	0.000581	0.00459	APTR	77323582
chr7	77325444	77326388	945	0.42	0.000581	0.00459	APTR	77323582
chr7	77325444	77326388	945	0.42	0.000581	0.00459	APTR	77323582
chr2	98346855	98347101	247	1.44	0.00059	0.00465	ZAP70	98345868
chr6	1.19E+08	1.19E+08	675	-0.45	0.000591	0.00466	FAM184A	1.19E+08
chr6	1.19E+08	1.19E+08	675	-0.45	0.000591	0.00466	FAM184A	1.19E+08
chr6	26110144	26110347	204	2.06	0.000593	0.00467	HIST1H1T	26106364
chr5	21459344	21459890	547	0.73	0.000604	0.00474	GUSBP1	21454588
chr5	21459344	21459890	547	0.73	0.000604	0.00474	GUSBP1	21454588
chr5	21459344	21459890	547	0.73	0.000604	0.00474	GUSBP1	21454588
chr12	31476859	31477341	483	0.84	0.000614	0.00481	FAM60A	31477121
chr12	31476859	31477341	483	0.84	0.000614	0.00481	FAM60A	31477159
chr12	31476859	31477341	483	0.84	0.000614	0.00481	FAM60A	31477121
chr12	31476859	31477341	483	0.84	0.000614	0.00481	FLJ13224	31472249
chr2	1.39E+08	1.39E+08	794	0.7	0.00062	0.00485	LOC10192{	1.39E+08
chr1	26680576	26680920	345	-1.05	0.000623	0.00487	AIM1L	26678621
chr12	10545080	10545536	457	0.96	0.000639	0.00497	KLRK1	10540653
chr8	22454643	22455320	678	0.56	0.000649	0.00503	C8orf58	22452113
chr8	22454643	22455320	678	0.56	0.000649	0.00503	C8orf58	22452113
chr8	22454643	22455320	678	0.56	0.000649	0.00503	C8orf58	22452113
chr3	24565865	24566105	241	-0.85	0.00065	0.00503	MIR4792	24560926
chr2	1.01E+08	1.01E+08	946	0.33	0.000652	0.00505	PDCL3	1.01E+08
chr1	1.14E+08	1.14E+08	314	0.74	0.000653	0.00505	MAGI3	1.14E+08
chr1	1.14E+08	1.14E+08	314	0.74	0.000653	0.00505	MAGI3	1.14E+08
chr3	1.75E+08	1.75E+08	201	1.02	0.000656	0.00507	MIR548AY	1.75E+08
chr7	47691059	47691507	449	1.59	0.000659	0.00509	C7orf65	47689841
chr12	52915077	52915317	241	1.22	0.000659	0.00509	KRT5	52912243
chr18	3593444	3594247	804	0.58	0.000663	0.00511	DLGAP1-AS	3589111
chr15	1.01E+08	1.01E+08	633	-0.57	0.000663	0.00511	LRRK1	1.01E+08
chr5	1.61E+08	1.61E+08	201	0.88	0.00067	0.00516	LINC01202	1.61E+08
chr19	32082809	32083004	196	0.79	0.000676	0.0052	THEG5	32078186

chr11	46736057	46736263	207	1.52	0.000677	0.0052	F2	46735715
chr11	46736057	46736263	207	1.52	0.000677	0.0052	F2	46735715
chr12	80836701	80836877	177	1.41	0.000684	0.00524	PTPRQ	80833125
chr6	69344949	69345310	362	1.24	0.000684	0.00525	ADGRB3	69340631
chr6	69344949	69345310	362	1.24	0.000684	0.00525	LOC10192ξ	69342216
chr11	834774	835239	466	0.78	0.000695	0.00532	CD151	827951
chr11	834774	835239	466	0.78	0.000695	0.00532	CD151	827951
chr11	834774	835239	466	0.78	0.000695	0.00532	CD151	827951
chr11	834774	835239	466	0.78	0.000695	0.00532	CD151	827951
chr9	1.32E+08	1.32E+08	744	0.59	7.00E-04	0.00535	LOC10192S	1.32E+08
chr13	78104892	78105089	198	1.28	0.000708	0.0054	SCEL	78104808
chr13	78104892	78105089	198	1.28	0.000708	0.0054	SCEL	78104808
chr13	78104892	78105089	198	1.28	0.000708	0.0054	SCEL	78104808
chr5	95063637	95063906	270	0.93	0.000719	0.00547	RHOBTB3	95061849
chr11	82756045	82756271	227	1.24	0.000723	0.0055	SNORA70E	82750640
chr3	59033804	59034002	199	0.71	0.000726	0.00551	C3orf67	59033715
chr1	1.11E+08	1.11E+08	765	0.67	0.00073	0.00554	LAMTOR5	1.11E+08
chr1	1.11E+08	1.11E+08	765	0.67	0.00073	0.00554	LAMTOR5-/	1.11E+08
chr10	44349511	44349911	401	0.56	0.000743	0.00562	LINC00840	44349858
chr1	1.17E+08	1.17E+08	335	1	0.000751	0.00567	PTGFRN	1.17E+08
chr8	22419352	22420022	671	0.88	0.000757	0.00571	SORBS3	22418178
chr22	21391986	21392416	431	1.14	0.000758	0.00571	MIR649	21386561
chr6	64283350	64283775	426	0.41	0.000759	0.00572	PTP4A1	64276916
chr3	1.3E+08	1.3E+08	273	0.73	0.000763	0.00574	TMCC1-AS:	1.3E+08
chr2	1.21E+08	1.21E+08	695	0.47	0.000763	0.00574	PTPN4	1.21E+08
chr21	46847383	46847944	562	0.86	0.000763	0.00574	COL18A1-A	46842955
chr21	46847383	46847944	562	0.86	0.000763	0.00574	COL18A1-A	46842985
chr10	82173305	82173675	371	0.64	0.000769	0.00578	FAM213A	82168574
chr10	82173305	82173675	371	0.64	0.000769	0.00578	FAM213A	82168574
chr13	41711480	41711705	226	0.97	0.000777	0.00582	KBTBD6	41704936
chr11	58940271	58940521	251	-0.84	0.000789	0.0059	DTX4	58934811
chr11	58940271	58940521	251	-0.84	0.000789	0.0059	DTX4	58933902
chr19	33560584	33561103	520	0.74	0.000792	0.00591	RHPN2	33553824
chr17	33390546	33390945	400	0.62	0.000814	0.00605	RFFL	33388759
chr1	1.55E+08	1.55E+08	200	0.86	0.000817	0.00607	ADAR	1.55E+08
chr1	1.55E+08	1.55E+08	200	0.86	0.000817	0.00607	ADAR	1.55E+08
chr1	1.55E+08	1.55E+08	200	0.86	0.000817	0.00607	ADAR	1.55E+08
chr10	1.05E+08	1.05E+08	629	-0.8	0.000823	0.0061	RPEL1	1.05E+08
chr6	44042194	44042485	292	-1.08	0.000827	0.00613	LOC10192S	44040389
chr19	12476299	12476917	619	0.37	0.000829	0.00615	ZNF442	12474475
chr5	17216365	17217814	1450	0.54	0.000831	0.00615	LOC28569€	17215531
chr5	17216365	17217814	1450	0.54	0.000831	0.00615	BASP1	17212669
chr5	17216365	17217814	1450	0.54	0.000831	0.00615	BASP1	17211931
chr5	1.32E+08	1.32E+08	125	1.67	0.000832	0.00616	P4HA2	1.32E+08
chr5	1.32E+08	1.32E+08	125	1.67	0.000832	0.00616	P4HA2	1.32E+08
chr8	80520158	80520358	201	1.21	0.000836	0.00619	STMN2	80518048
chr8	80520158	80520358	201	1.21	0.000836	0.00619	STMN2	80518048

chr6	46652537	46652794	258	1.3	0.000841	0.00622	TDRD6	46650611
chr6	46652537	46652794	258	1.3	0.000841	0.00622	TDRD6	46650611
chr1	1.72E+08	1.72E+08	461	-0.69	0.000843	0.00622	PIGC	1.72E+08
chr1	1.72E+08	1.72E+08	461	-0.69	0.000843	0.00622	C1orf105	1.72E+08
chr1	1.72E+08	1.72E+08	461	-0.69	0.000843	0.00622	PIGC	1.72E+08
chr21	44753302	44753878	577	0.85	0.00086	0.00633	LINC00322	44749919
chr22	41959149	41959638	490	0.96	0.000863	0.00635	LOC10537:	41954887
chr2	24587974	24588226	253	1.1	0.000863	0.00636	ITSN2	24581397
chr2	24587974	24588226	253	1.1	0.000863	0.00636	ITSN2	24581397
chr2	24587974	24588226	253	1.1	0.000863	0.00636	ITSN2	24581397
chr12	10025339	10025671	333	1.23	0.000866	0.00637	CLEC2B	10020458
chr12	52624245	52624988	744	0.62	0.000866	0.00637	KRT7	52621953
chr17	15009991	15010150	160	-1.37	0.000869	0.00638	CDRT8	15007504
chr16	71557805	71558004	200	0.76	0.000873	0.00641	CHST4	71555022
chr16	71557805	71558004	200	0.76	0.000873	0.00641	CHST4	71555153
chr10	92980167	92980649	483	-0.75	0.000887	0.00649	PCGF5	92975358
chr10	92980167	92980649	483	-0.75	0.000887	0.00649	PCGF5	92974907
chr10	92980167	92980649	483	-0.75	0.000887	0.00649	PCGF5	92975358
chr15	22956598	22956819	222	1.37	0.000887	0.0065	CYFIP1	22950754
chr6	73939797	73939997	201	0.77	0.000896	0.00655	KHDC1L	73933175
chr19	41933210	41933527	318	1	0.000908	0.00662	B3GNT8	41932635
chr7	48124412	48124849	438	0.39	0.000918	0.00668	UPP1	48123199
chr7	48124412	48124849	438	0.39	0.000918	0.00668	UPP1	48123808
chr7	48124412	48124849	438	0.39	0.000918	0.00668	UPP1	48123199
chr7	48124412	48124849	438	0.39	0.000918	0.00668	UPP1	48123850
chr7	48124412	48124849	438	0.39	0.000918	0.00668	UPP1	48123736
chr7	48124412	48124849	438	0.39	0.000918	0.00668	UPP1	48123850
chr12	95012857	95012992	136	-1.21	0.000925	0.00673	TMCC3	95007927
chr11	48118700	48118979	280	1.01	0.000948	0.00687	MIR3161	48113333
chr16	10832664	10832933	270	-0.83	0.000954	0.0069	NUBP1	10832642
chr16	10832664	10832933	270	-0.83	0.000954	0.0069	NUBP1	10832642
chr2	1.98E+08	1.98E+08	201	1.16	0.000961	0.00695	ANKRD44	1.98E+08
chr2	1.98E+08	1.98E+08	201	1.16	0.000961	0.00695	ANKRD44	1.98E+08
chr10	87187937	87188135	199	0.99	0.000974	0.00702	LOC10192!	87187618
chr19	10631678	10631864	187	1.16	0.000977	0.00704	S1PR5	10626668
chr19	10631678	10631864	187	1.16	0.000977	0.00704	S1PR5	10626126
chr14	59296373	59296583	211	-1.09	0.000983	0.00708	LINC01500	59290005
chr3	1.95E+08	1.95E+08	819	-0.85	0.000988	0.0071	MUC20	1.95E+08
chr3	1.95E+08	1.95E+08	819	-0.85	0.000988	0.0071	MUC20	1.95E+08
chr3	1.95E+08	1.95E+08	819	-0.85	0.000988	0.0071	MUC20	1.95E+08
chr3	1.95E+08	1.95E+08	819	-0.85	0.000988	0.0071	MUC20	1.95E+08
chr8	1.24E+08	1.24E+08	920	0.4	0.000989	0.00711	LOC10537!	1.24E+08
chr22	39150350	39150630	281	0.98	0.000996	0.00715	SUN2	39150024
chr22	39150350	39150630	281	0.98	0.000996	0.00715	SUN2	39149467
chr22	39150350	39150630	281	0.98	0.000996	0.00715	SUN2	39150024
chr11	64644318	64644802	485	0.74	0.000999	0.00716	EHD1	64644240
chr14	52183354	52183575	222	1.32	0.001	0.00718	FRMD6	52179000

chr2	1.73E+08	1.73E+08	946	0.62	0.001	0.00719	DLX1	1.73E+08
chr2	1.73E+08	1.73E+08	946	0.62	0.001	0.00719	DLX1	1.73E+08
chr1	90286215	90286856	642	0.56	0.00102	0.00726	LRRC8D	90282479
chr1	90286215	90286856	642	0.56	0.00102	0.00726	LRRC8D	90281572
chr1	2.1E+08	2.1E+08	171	-0.75	0.00102	0.00728	LAMB3	2.1E+08
chr1	2.1E+08	2.1E+08	171	-0.75	0.00102	0.00728	LAMB3	2.1E+08
chr1	2.1E+08	2.1E+08	171	-0.75	0.00102	0.00728	LAMB3	2.1E+08
chr10	1.3E+08	1.3E+08	283	-0.82	0.00104	0.00739	PTPRE	1.3E+08
chr1	2.32E+08	2.32E+08	259	1.19	0.00104	0.00741	DISC2	2.32E+08
chr7	47802763	47803510	748	-0.47	0.00104	0.00742	LINC00525	47796073
chr2	1.28E+08	1.28E+08	507	1.12	0.00105	0.00744	LIMS2	1.28E+08
chr2	1.28E+08	1.28E+08	507	1.12	0.00105	0.00744	LIMS2	1.28E+08
chr10	88779796	88780079	284	-1.25	0.00105	0.00745	FAM25A	88775045
chr15	90461013	90461263	251	-1.42	0.00105	0.00746	C15orf38-A	90454222
chr15	90461013	90461263	251	-1.42	0.00105	0.00746	ARPIN	90454222
chrX	27480458	27480657	200	1.11	0.00105	0.00748	PPP4R3CP	27479458
chr9	1.12E+08	1.12E+08	476	0.61	0.00107	0.0076	CTNNAL1	1.12E+08
chr9	1.12E+08	1.12E+08	476	0.61	0.00107	0.0076	CTNNAL1	1.12E+08
chr7	1.44E+08	1.44E+08	206	-0.99	0.00107	0.0076	OR2A14	1.44E+08
chr2	65594709	65594948	240	-1.07	0.00108	0.00762	SPRED2	65591931
chr15	63334624	63334989	366	0.8	0.00108	0.00766	TPM1	63329837
chr15	63334624	63334989	366	0.8	0.00108	0.00766	TPM1	63329837
chr15	63334624	63334989	366	0.8	0.00108	0.00766	TPM1	63329837
chr15	63334624	63334989	366	0.8	0.00108	0.00766	TPM1	63329837
chr15	63334624	63334989	366	0.8	0.00108	0.00766	TPM1	63329837
chr15	63334624	63334989	366	0.8	0.00108	0.00766	TPM1	63329837
chr15	63334624	63334989	366	0.8	0.00108	0.00766	TPM1	63329837
chr9	85677039	85678298	1260	-0.39	0.00108	0.00766	RASEF	85676043
chr11	61658059	61658297	239	1.01	0.0011	0.00778	FADS3	61657017
chr1	1.75E+08	1.75E+08	309	-0.58	0.00111	0.00779	KIAA0040	1.75E+08
chr1	1.75E+08	1.75E+08	309	-0.58	0.00111	0.00779	KIAA0040	1.75E+08
chr1	1.75E+08	1.75E+08	309	-0.58	0.00111	0.00779	KIAA0040	1.75E+08
chr1	1.75E+08	1.75E+08	309	-0.58	0.00111	0.00779	KIAA0040	1.75E+08
chr16	89571838	89572014	177	-1.07	0.00111	0.00779	SPG7	89569795
chr16	89571838	89572014	177	-1.07	0.00111	0.00779	SPG7	89569795
chr16	24737620	24737819	200	0.81	0.00112	0.0079	TNRC6A	24736048
chr3	1.68E+08	1.68E+08	1230	0.27	0.00114	0.00801	GOLIM4	1.68E+08
chr3	1.68E+08	1.68E+08	1230	0.27	0.00114	0.00801	GOLIM4	1.68E+08
chr5	1.33E+08	1.33E+08	199	0.78	0.00115	0.00806	LOC10537‹	1.33E+08
chr21	30517685	30517995	311	-1.27	0.00116	0.00808	MAP3K7CL	30512879
chr1	2466219	2466646	428	-0.74	0.00116	0.00809	HES5	2459684
chr12	1.09E+08	1.09E+08	362	0.74	0.00116	0.00811	CORO1C	1.09E+08
chr2	85359985	85360715	731	0.63	0.00116	0.00811	TCF7L1	85355582
chr8	1.29E+08	1.29E+08	295	0.77	0.00116	0.00812	PVT1	1.29E+08
chr8	1.29E+08	1.29E+08	295	0.77	0.00116	0.00812	MIR1204	1.29E+08
chr10	1.12E+08	1.12E+08	409	0.99	0.00116	0.00812	RBM20	1.12E+08
chr17	7482619	7482966	348	-0.84	0.00117	0.00813	CD68	7477804

chr17	7482619	7482966	348	-0.84	0.00117	0.00813	SNORA67	7476272
chr17	7482619	7482966	348	-0.84	0.00117	0.00813	CD68	7477805
chr17	7482619	7482966	348	-0.84	0.00117	0.00813	MPDU1	7481964
chr17	7482619	7482966	348	-0.84	0.00117	0.00813	MPDU1	7481964
chr11	1.02E+08	1.02E+08	423	0.35	0.00118	0.00818	BIRC2	1.02E+08
chr11	1.02E+08	1.02E+08	423	0.35	0.00118	0.00818	BIRC2	1.02E+08
chr11	1.02E+08	1.02E+08	423	0.35	0.00118	0.00818	BIRC2	1.02E+08
chr13	73301392	73302241	850	0.33	0.00118	0.00819	BORA	73296886
chr13	73301392	73302241	850	0.33	0.00118	0.00819	BORA	73297041
chr13	73301392	73302241	850	0.33	0.00118	0.00819	MZT1	73299938
chr13	73301392	73302241	850	0.33	0.00118	0.00819	BORA	73296886
chr15	91412871	91413200	330	-0.7	0.00118	0.0082	FURIN	91409768
chr15	91412871	91413200	330	-0.7	0.00118	0.0082	FURIN	91406821
chr15	91412871	91413200	330	-0.7	0.00118	0.0082	FURIN	91411107
chr10	75401481	75401728	248	2.76	0.00118	0.00821	MYOZ1	75399515
chr5	56778473	56778921	449	0.96	0.00119	0.00829	ACTBL2	56776636
chr6	41701592	41702058	467	0.83	0.0012	0.00834	TFEB	41701997
chr6	41701592	41702058	467	0.83	0.0012	0.00834	TFEB	41700798
chr6	41701592	41702058	467	0.83	0.0012	0.00834	TFEB	41700139
chr6	41701592	41702058	467	0.83	0.0012	0.00834	TFEB	41701265
chr6	41701592	41702058	467	0.83	0.0012	0.00834	TFEB	41701265
chr1	1.98E+08	1.98E+08	240	0.78	0.0012	0.00835	NEK7	1.98E+08
chr17	7620229	7621033	805	0.59	0.00122	0.00846	DNAH2	7615671
chr17	7620229	7621033	805	0.59	0.00122	0.00846	DNAH2	7616077
chr9	1.12E+08	1.12E+08	152	1.38	0.00123	0.00848	PALM2	1.12E+08
chr14	30174917	30175117	201	1.22	0.00123	0.00852	MIR548AI	30172608
chr1	1.53E+08	1.53E+08	133	-1.61	0.00124	0.00856	SPRR4	1.53E+08
chr1	1.6E+08	1.6E+08	341	0.7	0.00124	0.00856	LINC01133	1.6E+08
chr5	1.5E+08	1.5E+08	248	1.05	0.00124	0.00858	SMIM3	1.5E+08
chr19	11876955	11877153	199	1.17	0.00125	0.00863	ZNF441	11872814
chr22	28314864	28315659	796	-0.54	0.00125	0.00863	PITPNB	28313294
chr22	28314864	28315659	796	-0.54	0.00125	0.00863	PITPNB	28313202
chr22	28314864	28315659	796	-0.54	0.00125	0.00863	PITPNB	28313294
chr22	28314864	28315659	796	-0.54	0.00125	0.00863	MIR3199-2	28311513
chr22	28314864	28315659	796	-0.54	0.00125	0.00863	MIR3199-1	28314600
chr22	28314864	28315659	796	-0.54	0.00125	0.00863	TTC28-AS1	28310363
chr22	28314864	28315659	796	-0.54	0.00125	0.00863	TTC28-AS1	28310363
chr19	42746682	42747567	886	0.41	0.00125	0.00863	GSK3A	42744736
chr15	90541670	90541885	216	-1.1	0.00126	0.00866	ZNF710	90539722
chr2	85637443	85637667	225	-0.9	0.00127	0.0087	CAPG	85635820
chr2	85637443	85637667	225	-0.9	0.00127	0.0087	CAPG	85635820
chr14	81898637	81898926	290	1.31	0.00127	0.00873	STON2	81891748
chr20	49545986	49546155	170	-1.14	0.00127	0.00874	ADNP	49545527
chr20	49545986	49546155	170	-1.14	0.00127	0.00874	ADNP	49545527
chr20	49545986	49546155	170	-1.14	0.00127	0.00874	ADNP	49545527
chr20	49545986	49546155	170	-1.14	0.00127	0.00874	ADNP-AS1	49542520
chr20	49545986	49546155	170	-1.14	0.00127	0.00874	ADNP-AS1	49542520

chr20	49545986	49546155	170	-1.14	0.00127	0.00874	ADNP-AS1	49542520
chr3	1.72E+08	1.72E+08	369	0.84	0.00128	0.00875	TMEM212	1.72E+08
chr1	76081457	76081807	351	1.05	0.00128	0.00877	SLC44A5	76074799
chr1	76081457	76081807	351	1.05	0.00128	0.00877	SLC44A5	76074799
chr3	1.37E+08	1.37E+08	662	-0.49	0.00129	0.00881	IL20RB	1.37E+08
chr7	1.05E+08	1.05E+08	236	1.38	0.0013	0.00888	LINC01004	1.05E+08
chr2	52931648	52932125	478	1.36	0.0013	0.00889	MIR4431	52927753
chr9	71790090	71790296	207	0.7	0.0013	0.0089	TJP2	71783970
chr9	71790090	71790296	207	0.7	0.0013	0.0089	TJP2	71783970
chr9	71790090	71790296	207	0.7	0.0013	0.0089	TJP2	71783970
chr4	95128625	95129324	700	0.64	0.0013	0.00891	SMARCAD1	95124234
chr4	95128625	95129324	700	0.64	0.0013	0.00891	SMARCAD1	95123995
chr4	95128625	95129324	700	0.64	0.0013	0.00891	SMARCAD1	95123995
chr4	95128625	95129324	700	0.64	0.0013	0.00891	SMARCAD1	95123758
chr4	95128625	95129324	700	0.64	0.0013	0.00891	LOC10192‍9	95126707
chr14	60337371	60337776	406	0.82	0.00131	0.00894	RTN1	60335557
chr5	1.22E+08	1.22E+08	468	-0.61	0.00131	0.00895	SNX24	1.22E+08
chr11	62538536	62539226	691	0.49	0.00133	0.00904	TAF6L	62533774
chr7	23286226	23286531	306	0.95	0.00133	0.00905	GPNMB	23281315
chr7	23286226	23286531	306	0.95	0.00133	0.00905	GPNMB	23281315
chr12	1.04E+08	1.04E+08	1358	-0.39	0.00133	0.00905	NT5DC3	1.04E+08
chr9	72287175	72288143	969	0.47	0.00134	0.00906	APBA1	72285275
chr2	2.35E+08	2.35E+08	319	0.76	0.00134	0.00909	UGT1A6	2.35E+08
chr2	2.35E+08	2.35E+08	319	0.76	0.00134	0.00909	UGT1A6	2.35E+08
chr4	2820583	2820783	201	1.04	0.00134	0.00911	SH3BP2	2815540
chr4	2820583	2820783	201	1.04	0.00134	0.00911	SH3BP2	2814911
chr7	24612667	24613859	1193	0.37	0.00135	0.00914	MPP6	24607964
chr7	24612667	24613859	1193	0.37	0.00135	0.00914	MPP6	24607964
chr7	66767126	66768070	945	0.33	0.00136	0.0092	PMS2P4	66765429
chr7	66767126	66768070	945	0.33	0.00136	0.0092	PMS2P4	66765429
chr7	66767126	66768070	945	0.33	0.00136	0.0092	PMS2P4	66765429
chr7	66767126	66768070	945	0.33	0.00136	0.0092	STAG3L4	66762624
chr7	66767126	66768070	945	0.33	0.00136	0.0092	STAG3L4	66762624
chr1	23523204	23523649	446	0.8	0.00137	0.00925	HTR1D	23519222
chr6	1.33E+08	1.33E+08	383	0.7	0.00138	0.00929	VNN2	1.33E+08
chr8	1.24E+08	1.24E+08	375	-0.64	0.00138	0.00929	FAM83A	1.24E+08
chr3	1.25E+08	1.25E+08	335	0.84	0.00138	0.00931	HEG1	1.25E+08
chr8	61655071	61655265	195	1.71	0.00138	0.00931	CHD7	61648817
chr3	1.84E+08	1.84E+08	545	-0.73	0.00139	0.00937	MIR1224	1.84E+08
chr16	27437744	27437989	246	1.7	0.0014	0.00941	IL21R	27433578
chr16	29911345	29911867	523	-0.82	0.0014	0.00943	SEZ6L2	29908585
chr16	29911345	29911867	523	-0.82	0.0014	0.00943	SEZ6L2	29908585
chr16	29911345	29911867	523	-0.82	0.0014	0.00943	SEZ6L2	29908585
chr16	29911345	29911867	523	-0.82	0.0014	0.00943	SEZ6L2	29908585
chr16	29911345	29911867	523	-0.82	0.0014	0.00943	SEZ6L2	29908585
chr16	29911345	29911867	523	-0.82	0.0014	0.00943	SEZ6L2	29908585
chr16	29911345	29911867	523	-0.82	0.0014	0.00943	ASPHD1	29907146

chr17	20370827	20371187	361	-1.83	0.00143	0.00958	LGALS9B	20368848
chr1	1690234	1690659	426	-0.83	0.00143	0.0096	NADK	1688081
chr7	1.4E+08	1.4E+08	486	-0.89	0.00144	0.00964	RAB19	1.4E+08
chr7	96635395	96635792	398	0.83	0.00144	0.00965	DLX6	96630289
chr3	85007805	85008378	574	0.6	0.00145	0.00968	CADM2	85003132
chr3	85007805	85008378	574	0.6	0.00145	0.00968	CADM2	85003132
chr5	10479257	10479862	606	0.49	0.00145	0.00969	MIR6131	10473148
chr8	1.19E+08	1.19E+08	255	0.71	0.00146	0.00975	EXT1	1.19E+08
chr5	1.46E+08	1.46E+08	200	0.87	0.00147	0.0098	LARS	1.46E+08
chr5	1.46E+08	1.46E+08	200	0.87	0.00147	0.0098	LARS	1.46E+08
chr5	1.46E+08	1.46E+08	200	0.87	0.00147	0.0098	LARS	1.46E+08
chr5	1.46E+08	1.46E+08	200	0.87	0.00147	0.0098	LARS	1.46E+08
chr11	1.31E+08	1.31E+08	200	1.02	0.00147	0.0098	MIR8052	1.31E+08
chr7	32799151	32799351	201	1.2	0.00147	0.00981	LINC00997	32792897
chr12	69974588	69974766	179	1.37	0.00148	0.00982	CCT2	69974207
chr12	69974588	69974766	179	1.37	0.00148	0.00982	CCT2	69974461
chr12	69974588	69974766	179	1.37	0.00148	0.00982	MIR3913-2	69973502
chr1	1.81E+08	1.81E+08	789	-0.49	0.00149	0.00992	XPR1	1.81E+08
chr1	1.81E+08	1.81E+08	789	-0.49	0.00149	0.00992	XPR1	1.81E+08
chr2	1.01E+08	1.01E+08	300	1.35	0.0015	0.00995	NPAS2	1.01E+08
chr5	1.51E+08	1.51E+08	646	0.68	0.00151	0.01	FAT2	1.51E+08
chr16	67311206	67311582	377	0.73	0.00151	0.01	PLEKHG4	67307059
chr16	67311206	67311582	377	0.73	0.00151	0.01	PLEKHG4	67308426
chr16	67311206	67311582	377	0.73	0.00151	0.01	PLEKHG4	67308049
chr16	67311206	67311582	377	0.73	0.00151	0.01	PLEKHG4	67308426
chr1	2.48E+08	2.48E+08	636	-0.49	0.00155	0.0102	GCSAML-A!	2.48E+08
chr19	13263661	13263984	324	-0.64	0.00156	0.0103	STX10	13259188
chr19	13263661	13263984	324	-0.64	0.00156	0.0103	STX10	13259188
chr19	13263661	13263984	324	-0.64	0.00156	0.0103	STX10	13259188
chr19	13263661	13263984	324	-0.64	0.00156	0.0103	STX10	13259188
chr10	69459758	69460085	328	1.48	0.00157	0.0103	CTNNA3	69453949
chr21	28215307	28215980	674	0.66	0.00158	0.0104	ADAMTS1	28215728
chr1	12221913	12222317	405	-0.84	0.00159	0.0104	TNFRSF1B	12222059
chr1	12221913	12222317	405	-0.84	0.00159	0.0104	MIR7846	12221999
chr5	1.31E+08	1.31E+08	448	0.81	0.00161	0.0105	CDC42SE2	1.31E+08
chr5	1.31E+08	1.31E+08	448	0.81	0.00161	0.0105	CDC42SE2	1.31E+08
chr1	99127007	99127766	760	0.46	0.00162	0.0106	SNX7	99122235
chr1	99127007	99127766	760	0.46	0.00162	0.0106	SNX7	99122235
chr1	99127007	99127766	760	0.46	0.00162	0.0106	SNX7	99122235
chr3	1.21E+08	1.21E+08	753	0.52	0.00162	0.0106	POLQ	1.21E+08
chr13	22177715	22178536	822	0.38	0.00164	0.0107	MICU2	22176355
chr12	16034485	16034684	200	0.74	0.00164	0.0107	STRAP	16030287
chr11	1.2E+08	1.2E+08	383	-0.96	0.00167	0.0109	TRIM29	1.2E+08
chr5	50678713	50679456	744	0.44	0.00167	0.0109	LOC64236(	50677166
chr5	50678713	50679456	744	0.44	0.00167	0.0109	ISL1	50673957
chr3	1.11E+08	1.11E+08	617	0.36	0.00168	0.0109	PLCXD2	1.11E+08
chr3	1.11E+08	1.11E+08	617	0.36	0.00168	0.0109	PLCXD2	1.11E+08

chr9	1.27E+08	1.27E+08	368	0.85	0.00168	0.0109	NEK6	1.27E+08
chr9	1.27E+08	1.27E+08	368	0.85	0.00168	0.0109	NEK6	1.27E+08
chr9	1.27E+08	1.27E+08	368	0.85	0.00168	0.0109	NEK6	1.27E+08
chr9	1.27E+08	1.27E+08	368	0.85	0.00168	0.0109	NEK6	1.27E+08
chr9	1.2E+08	1.2E+08	891	-0.48	0.00168	0.0109	TLR4	1.2E+08
chr9	1.2E+08	1.2E+08	891	-0.48	0.00168	0.0109	TLR4	1.2E+08
chr9	1.2E+08	1.2E+08	891	-0.48	0.00168	0.0109	TLR4	1.2E+08
chr4	1.52E+08	1.52E+08	268	-0.94	0.00168	0.0109	FAM160A1	1.52E+08
chr2	73522982	73523170	189	1.6	0.00168	0.0109	EGR4	73518829
chr9	98640446	98640646	201	1.06	0.00168	0.0109	LINC00476	98636259
chr9	98640446	98640646	201	1.06	0.00168	0.0109	LINC00476	98636259
chrX	99986496	99986630	135	-2.06	0.00168	0.0109	SYTL4	99984522
chrX	99986496	99986630	135	-2.06	0.00168	0.0109	SYTL4	99984522
chrX	99986496	99986630	135	-2.06	0.00168	0.0109	SYTL4	99985135
chr2	97171035	97171451	417	0.61	0.00169	0.011	NEURL3	97169122
chr2	97171035	97171451	417	0.61	0.00169	0.011	NEURL3	97169122
chr13	36793163	36793363	201	0.71	0.00169	0.011	SOHLH2	36786752
chr13	36793163	36793363	201	0.71	0.00169	0.011	SOHLH2	36786752
chr14	35590893	35591952	1060	0.36	0.0017	0.011	KIAA0391	35586757
chr14	35590893	35591952	1060	0.36	0.0017	0.011	KIAA0391	35586757
chr14	35590893	35591952	1060	0.36	0.0017	0.011	KIAA0391	35586526
chr14	35590893	35591952	1060	0.36	0.0017	0.011	KIAA0391	35586526
chr14	35590893	35591952	1060	0.36	0.0017	0.011	KIAA0391	35586526
chr14	35590893	35591952	1060	0.36	0.0017	0.011	PPP2R3C	35589748
chr14	35590893	35591952	1060	0.36	0.0017	0.011	PPP2R3C	35589749
chr14	35590893	35591952	1060	0.36	0.0017	0.011	PPP2R3C	35589748
chr14	35590893	35591952	1060	0.36	0.0017	0.011	PPP2R3C	35589749
chr8	24377330	24377530	201	1.05	0.00171	0.0111	LOC10192‌9	24370381
chr9	1.32E+08	1.32E+08	331	-0.72	0.00172	0.0111	LINC00963	1.32E+08
chr12	56111232	56111552	321	-0.72	0.00172	0.0112	BLOC1S1	56105075
chr12	56111232	56111552	321	-0.72	0.00172	0.0112	BLOC1S1	56105075
chr12	56111232	56111552	321	-0.72	0.00172	0.0112	BLOC1S1	56104817
chr12	56111232	56111552	321	-0.72	0.00172	0.0112	RDH5	56109150
chr12	56111232	56111552	321	-0.72	0.00172	0.0112	RDH5	56109150
chr12	56111232	56111552	321	-0.72	0.00172	0.0112	BLOC1S1	56104817
chr12	56111232	56111552	321	-0.72	0.00172	0.0112	BLOC1S1-R	56104817
chr15	62599799	62600096	298	1.4	0.00173	0.0112	MIR8067	62594933
chr5	72793966	72794681	716	0.77	0.00175	0.0113	BTF3	72789249
chr5	72793966	72794681	716	0.77	0.00175	0.0113	BTF3	72789249
chr18	32559019	32559434	416	-0.59	0.00175	0.0113	MAPRE2	32553207
chr20	61449980	61450176	197	1.2	0.00177	0.0114	COL9A3	61443413
chr12	1.17E+08	1.17E+08	498	0.65	0.0018	0.0115	MIR4472-2	1.17E+08
chr8	1.46E+08	1.46E+08	194	0.82	0.00181	0.0116	C8orf33	1.46E+08
chr2	2.02E+08	2.02E+08	390	0.77	0.00188	0.0119	LOC101927	2.02E+08
chr2	2.02E+08	2.02E+08	390	0.77	0.00188	0.0119	BZW1	2.02E+08
chr2	2.02E+08	2.02E+08	390	0.77	0.00188	0.0119	BZW1	2.02E+08
chr2	2.02E+08	2.02E+08	390	0.77	0.00188	0.0119	BZW1	2.02E+08

chr2	2.02E+08	2.02E+08	390	0.77	0.00188	0.0119	BZW1	2.02E+08
chr12	11699967	11700415	449	1.1	0.00188	0.0119	LINC01252	11695963
chr3	1.36E+08	1.36E+08	200	0.72	0.00188	0.0119	MSL2	1.36E+08
chr3	1.36E+08	1.36E+08	200	0.72	0.00188	0.0119	MSL2	1.36E+08
chr14	77647842	77648279	438	0.62	0.00189	0.012	TMEM63C	77643101
chr22	33834214	33834642	429	0.73	0.00189	0.012	MIR4764	33830655
chr12	55413567	55413871	305	0.86	0.00192	0.0121	NEUROD4	55408728
chr19	11196120	11196474	355	0.56	0.00192	0.0121	LDLR	11195037
chr19	11196120	11196474	355	0.56	0.00192	0.0121	LDLR	11195037
chr19	11196120	11196474	355	0.56	0.00192	0.0121	LDLR	11195037
chr19	11196120	11196474	355	0.56	0.00192	0.0121	LDLR	11195037
chr19	11196120	11196474	355	0.56	0.00192	0.0121	LDLR	11195037
chr7	46739304	46739504	201	0.84	0.00193	0.0122	LOC730338	46734720
chr11	77897028	77897156	129	-1.01	0.00193	0.0122	USP35	77894857
chr6	13711772	13712582	811	-0.52	0.00194	0.0122	RANBP9	13709796
chr1	2.13E+08	2.13E+08	429	-1.07	0.00194	0.0122	BATF3	2.13E+08
chr8	32405497	32406773	1277	0.5	0.00194	0.0122	NRG1	32400727
chr8	32405497	32406773	1277	0.5	0.00194	0.0122	NRG1	32400727
chr8	32405497	32406773	1277	0.5	0.00194	0.0122	NRG1	32400727
chr8	32405497	32406773	1277	0.5	0.00194	0.0122	NRG1	32400727
chr8	32405497	32406773	1277	0.5	0.00194	0.0122	NRG1	32400727
chr8	32405497	32406773	1277	0.5	0.00194	0.0122	NRG1	32400727
chr8	32405497	32406773	1277	0.5	0.00194	0.0122	NRG1	32400727
chr8	32405497	32406773	1277	0.5	0.00194	0.0122	NRG1	32400727
chr8	32405497	32406773	1277	0.5	0.00194	0.0122	NRG1	32400727
chr8	32405497	32406773	1277	0.5	0.00194	0.0122	NRG1	32400727
chr8	32405497	32406773	1277	0.5	0.00194	0.0122	NRG1	32400727
chr3	1.5E+08	1.5E+08	1145	0.35	0.00197	0.0124	SIAH2	1.5E+08
chr2	1.44E+08	1.44E+08	179	1.75	0.00197	0.0124	KYNU	1.44E+08
chr2	1.44E+08	1.44E+08	179	1.75	0.00197	0.0124	KYNU	1.44E+08
chr2	1.44E+08	1.44E+08	179	1.75	0.00197	0.0124	KYNU	1.44E+08
chr2	1.53E+08	1.53E+08	893	0.43	0.00198	0.0125	STAM2	1.53E+08
chr17	48277548	48278430	883	0.59	0.002	0.0125	COL1A1	48277000
chr1	1.52E+08	1.52E+08	389	0.82	0.00202	0.0126	TCHHL1	1.52E+08
chr22	25709360	25709558	199	1.25	0.00203	0.0127	IGLL3P	25709223
chr11	7988675	7988882	208	1.33	0.00203	0.0127	NLRP10	7983059
chr6	57178626	57178968	343	0.69	0.00204	0.0127	PRIM2	57177414
chr6	57178626	57178968	343	0.69	0.00204	0.0127	PRIM2	57177414
chr6	57178626	57178968	343	0.69	0.00204	0.0127	PRIM2	57174602
chr4	79472750	79473198	449	-0.83	0.00204	0.0127	ANXA3	79467741
chr8	1.46E+08	1.46E+08	1036	-0.47	0.00204	0.0127	MFSD3	1.46E+08
chr8	1.46E+08	1.46E+08	1036	-0.47	0.00204	0.0127	MFSD3	1.46E+08
chr2	2.07E+08	2.07E+08	487	0.46	0.00206	0.0128	NRP2	2.07E+08
chr2	2.07E+08	2.07E+08	487	0.46	0.00206	0.0128	NRP2	2.07E+08
chr2	2.07E+08	2.07E+08	487	0.46	0.00206	0.0128	NRP2	2.07E+08
chr2	2.07E+08	2.07E+08	487	0.46	0.00206	0.0128	NRP2	2.07E+08
chr2	2.07E+08	2.07E+08	487	0.46	0.00206	0.0128	NRP2	2.07E+08

chr2	2.07E+08	2.07E+08	487	0.46	0.00206	0.0128	NRP2	2.07E+08
chr19	32896082	32897179	1098	0.34	0.00206	0.0128	DPY19L3	32892021
chr19	32896082	32897179	1098	0.34	0.00206	0.0128	DPY19L3	32891654
chr19	32896082	32897179	1098	0.34	0.00206	0.0128	LOC400684	32894445
chr10	1.13E+08	1.13E+08	860	0.65	0.00207	0.0128	ADRA2A	1.13E+08
chr12	53339303	53340055	753	0.49	0.00208	0.0129	KRT18	53337842
chr12	53339303	53340055	753	0.49	0.00208	0.0129	KRT18	53337654
chr11	85397173	85397489	317	0.87	0.00209	0.013	CCDC89	85395320
chr12	27925683	27925935	253	1.17	0.0021	0.013	MANSC4	27922209
chr2	1.39E+08	1.39E+08	242	-1.1	0.0021	0.013	HNMT	1.39E+08
chr2	1.39E+08	1.39E+08	242	-1.1	0.0021	0.013	HNMT	1.39E+08
chr2	1.39E+08	1.39E+08	242	-1.1	0.0021	0.013	HNMT	1.39E+08
chr11	67120800	67121376	577	0.57	0.0021	0.013	POLD4	67119067
chr11	67120800	67121376	577	0.57	0.0021	0.013	POLD4	67119067
chr11	67120800	67121376	577	0.57	0.0021	0.013	POLD4	67119067
chr11	67120800	67121376	577	0.57	0.0021	0.013	POLD4	67119067
chr11	67120800	67121376	577	0.57	0.0021	0.013	POLD4	67119067
chr16	58032492	58032660	169	-1.2	0.00211	0.0131	USB1	58030276
chr16	58032492	58032660	169	-1.2	0.00211	0.0131	ZNF319	58031762
chr16	58032492	58032660	169	-1.2	0.00211	0.0131	USB1	58030276
chr16	58032492	58032660	169	-1.2	0.00211	0.0131	USB1	58030276
chr1	2.26E+08	2.26E+08	628	-0.62	0.00211	0.0131	LIN9	2.26E+08
chr1	2.26E+08	2.26E+08	628	-0.62	0.00211	0.0131	LIN9	2.26E+08
chr1	2.26E+08	2.26E+08	628	-0.62	0.00211	0.0131	LIN9	2.26E+08
chr9	86957973	86958374	402	0.78	0.00212	0.0131	SLC28A3	86953694
chr9	86957973	86958374	402	0.78	0.00212	0.0131	SLC28A3	86953694
chr7	33080441	33080730	290	-0.59	0.00213	0.0132	NT5C3A	33078777
chr7	33080441	33080730	290	-0.59	0.00213	0.0132	NT5C3A	33078777
chr10	1.02E+08	1.02E+08	844	0.62	0.00214	0.0132	ABCC2	1.02E+08
chr16	10673930	10674732	803	-0.47	0.00214	0.0132	EMP2	10672573
chr19	57149462	57149766	305	0.85	0.00214	0.0132	SMIM17	57149526
chr6	2180862	2181060	199	0.74	0.00214	0.0132	GMDS	2174225
chr16	27275363	27275776	414	0.52	0.00215	0.0133	FLJ21408	27274525
chr12	54777061	54777170	110	-1.83	0.00215	0.0133	ZNF385A	54776514
chr12	54777061	54777170	110	-1.83	0.00215	0.0133	ZNF385A	54776514
chr5	1.57E+08	1.57E+08	163	1.14	0.00216	0.0133	THG1L	1.57E+08
chr5	1.57E+08	1.57E+08	163	1.14	0.00216	0.0133	THG1L	1.57E+08
chr5	1.57E+08	1.57E+08	163	1.14	0.00216	0.0133	THG1L	1.57E+08
chr5	1.57E+08	1.57E+08	163	1.14	0.00216	0.0133	THG1L	1.57E+08
chr11	18721929	18722207	279	0.75	0.00218	0.0134	TMEM86A	18715281
chr13	24475472	24475916	445	0.54	0.00219	0.0135	C1QTNF9B	24469145
chr13	24475472	24475916	445	0.54	0.00219	0.0135	C1QTNF9B	24469641
chr14	61203282	61203487	206	1.05	0.00219	0.0135	MNAT1	61196458
chr14	61203282	61203487	206	1.05	0.00219	0.0135	MNAT1	61196458
chr5	35994207	35994406	200	0.88	0.00221	0.0135	UGT3A1	35989535
chr14	21539508	21539884	377	0.67	0.00221	0.0136	NDRG2	21537031
chr14	21539508	21539884	377	0.67	0.00221	0.0136	ARHGEF40	21533418

chr14	21539508	21539884	377	0.67	0.00221	0.0136	ARHGEF40	21533418
chr14	21539508	21539884	377	0.67	0.00221	0.0136	ARHGEF40	21533418
chr5	73936418	73936720	303	0.85	0.00223	0.0136	ENC1	73935249
chr5	73936418	73936720	303	0.85	0.00223	0.0136	ENC1	73935249
chr5	73936418	73936720	303	0.85	0.00223	0.0136	ENC1	73935249
chr5	73936418	73936720	303	0.85	0.00223	0.0136	ENC1	73935249
chr5	73936418	73936720	303	0.85	0.00223	0.0136	ENC1	73935249
chr5	73936418	73936720	303	0.85	0.00223	0.0136	HEXB	73930847
chr4	84456730	84457447	718	-0.73	0.00223	0.0137	GPAT3	84452066
chr4	84456730	84457447	718	-0.73	0.00223	0.0137	GPAT3	84452259
chr4	84456730	84457447	718	-0.73	0.00223	0.0137	GPAT3	84452469
chr5	1.12E+08	1.12E+08	200	0.7	0.00224	0.0137	LOC102467	1.12E+08
chr21	27541120	27541498	379	0.78	0.00225	0.0137	APP	27541138
chr21	27541120	27541498	379	0.78	0.00225	0.0137	APP	27541138
chr21	27541120	27541498	379	0.78	0.00225	0.0137	APP	27541138
chr21	27541120	27541498	379	0.78	0.00225	0.0137	APP	27541138
chr21	27541120	27541498	379	0.78	0.00225	0.0137	APP	27541446
chr21	27541120	27541498	379	0.78	0.00225	0.0137	APP	27541138
chr21	27541120	27541498	379	0.78	0.00225	0.0137	APP	27541138
chr21	27541120	27541498	379	0.78	0.00225	0.0137	APP	27541138
chr21	27541120	27541498	379	0.78	0.00225	0.0137	APP	27541138
chr6	76783701	76783896	196	0.74	0.00225	0.0138	IMPG1	76780395
chr6	76783701	76783896	196	0.74	0.00225	0.0138	IMPG1	76780395
chr3	1.41E+08	1.41E+08	345	-0.64	0.00225	0.0138	RASA2	1.41E+08
chr3	1.41E+08	1.41E+08	345	-0.64	0.00225	0.0138	RASA2	1.41E+08
chr3	1.41E+08	1.41E+08	345	-0.64	0.00225	0.0138	RASA2	1.41E+08
chr15	81292143	81292260	118	-1.08	0.00226	0.0138	MIR4514	81287814
chr15	81292143	81292260	118	-1.08	0.00226	0.0138	MESDC1	81288294
chr15	89922858	89923456	599	0.45	0.00227	0.0138	MIR9-3HG	89916272
chr12	51739502	51739761	260	0.92	0.00227	0.0139	CELA1	51738463
chr9	1.13E+08	1.13E+08	597	-0.48	0.00227	0.0139	TXN	1.13E+08
chr9	1.13E+08	1.13E+08	597	-0.48	0.00227	0.0139	TXN	1.13E+08
chr1	1.81E+08	1.81E+08	207	0.92	0.00228	0.0139	MR1	1.81E+08
chr1	1.81E+08	1.81E+08	207	0.92	0.00228	0.0139	MR1	1.81E+08
chr1	1.81E+08	1.81E+08	207	0.92	0.00228	0.0139	MR1	1.81E+08
chr1	1.81E+08	1.81E+08	207	0.92	0.00228	0.0139	MR1	1.81E+08
chr1	1.81E+08	1.81E+08	207	0.92	0.00228	0.0139	MR1	1.81E+08
chr8	1.2E+08	1.2E+08	208	-0.57	0.00229	0.0139	MAL2	1.2E+08
chr15	30373090	30373336	247	1.42	0.00229	0.014	GOLGA8J	30370157
chr12	32830948	32831030	83	-1.23	0.0023	0.014	DNM1L	32827133
chr12	32830948	32831030	83	-1.23	0.0023	0.014	DNM1L	32827133
chr12	32830948	32831030	83	-1.23	0.0023	0.014	DNM1L	32827133
chr12	32830948	32831030	83	-1.23	0.0023	0.014	DNM1L	32827133
chr12	32830948	32831030	83	-1.23	0.0023	0.014	DNM1L	32827133
chr12	32830948	32831030	83	-1.23	0.0023	0.014	DNM1L	32827133
chr12	32830948	32831030	83	-1.23	0.0023	0.014	DNM1L	32827133
chr21	45663945	45664152	208	-0.97	0.0023	0.014	ICOSLG	45658887

chr21	45663945	45664152	208	-0.97	0.0023	0.014	ICOSLG	45658887
chr21	45663945	45664152	208	-0.97	0.0023	0.014	ICOSLG	45658887
chr21	45663945	45664152	208	-0.97	0.0023	0.014	ICOSLG	45658887
chr4	86697858	86698080	223	-1.35	0.0023	0.014	ARHGAP24	86694850
chr1	1.85E+08	1.85E+08	207	-0.93	0.00231	0.014	IVNS1ABP	1.85E+08
chr5	87733781	87734223	443	0.62	0.00232	0.0141	LOC10254(	87732907
chr10	92922404	92922654	251	-1.17	0.00232	0.0141	PCGF5	92917768
chr10	92922404	92922654	251	-1.17	0.00232	0.0141	PCGF5	92917768
chr18	53119596	53119708	113	2.42	0.00233	0.0141	TCF4-AS1	53114763
chrX	1.1E+08	1.1E+08	200	0.65	0.00234	0.0142	AMMECR1	1.1E+08
chr12	72665226	72666315	1090	0.5	0.00235	0.0142	TRHDE	72661528
chr12	72665226	72666315	1090	0.5	0.00235	0.0142	TRHDE-AS1	72665289
chr12	72665226	72666315	1090	0.5	0.00235	0.0142	TRHDE-AS1	72665289
chr4	1.35E+08	1.35E+08	868	0.76	0.00236	0.0143	PABPC4L	1.35E+08
chr21	43135870	43136317	448	-0.65	0.00236	0.0143	LINC00112	43131595
chr21	43135870	43136317	448	-0.65	0.00236	0.0143	LINC00479	43133935
chrX	47244568	47244768	201	1.3	0.00239	0.0144	SNORA11C	47243048
chr1	1.51E+08	1.51E+08	788	0.96	0.00239	0.0144	PSMB4	1.51E+08
chr1	2.04E+08	2.04E+08	519	0.68	0.00239	0.0145	PIK3C2B	2.04E+08
chr9	35907901	35908388	488	1.13	0.00239	0.0145	HRCT1	35901188
chr9	35907901	35908388	488	1.13	0.00239	0.0145	LINC00961	35904479
chr19	55983867	55984095	229	-0.92	0.0024	0.0145	ZNF628	55982698
chr5	87564510	87565279	770	0.38	0.0024	0.0145	TMEM161I	87562696
chr5	87564510	87565279	770	0.38	0.0024	0.0145	TMEM161I	87562696
chr5	87564510	87565279	770	0.38	0.0024	0.0145	TMEM161I	87562696
chr5	87564510	87565279	770	0.38	0.0024	0.0145	TMEM161I	87559698
chr5	87564510	87565279	770	0.38	0.0024	0.0145	TMEM161I	87559698
chr5	87564510	87565279	770	0.38	0.0024	0.0145	TMEM161I	87559832
chr5	87564510	87565279	770	0.38	0.0024	0.0145	TMEM161I	87559832
chr5	87564510	87565279	770	0.38	0.0024	0.0145	TMEM161I	87562696
chr2	2.38E+08	2.38E+08	227	-0.75	0.00241	0.0145	MLPH	2.38E+08
chr2	2.38E+08	2.38E+08	227	-0.75	0.00241	0.0145	MLPH	2.38E+08
chr2	2.38E+08	2.38E+08	227	-0.75	0.00241	0.0145	MLPH	2.38E+08
chr2	2.38E+08	2.38E+08	227	-0.75	0.00241	0.0145	MLPH	2.38E+08
chr2	2.38E+08	2.38E+08	227	-0.75	0.00241	0.0145	MLPH	2.38E+08
chr19	47633862	47634300	439	-0.72	0.00241	0.0145	SAE1	47629079
chr19	47633862	47634300	439	-0.72	0.00241	0.0145	SAE1	47629079
chr19	47633862	47634300	439	-0.72	0.00241	0.0145	SAE1	47629079
chr19	47633862	47634300	439	-0.72	0.00241	0.0145	SAE1	47629114
chr3	40142871	40143071	201	0.7	0.00241	0.0145	MYRIP	40136501
chr3	52484040	52484319	280	1.05	0.00242	0.0146	SEMA3G	52477043
chr4	1.06E+08	1.06E+08	201	0.98	0.00245	0.0147	ARHGEF38-	1.06E+08
chr11	65208932	65209132	201	0.92	0.00246	0.0147	MIR612	65206928
chr9	35071886	35072972	1087	0.45	0.00247	0.0148	VCP	35070739
chr7	1.24E+08	1.24E+08	200	0.73	0.00248	0.0149	GPR37	1.24E+08
chr7	66146185	66146362	178	0.81	0.00249	0.0149	RABGEF1	66142077
chr7	66146185	66146362	178	0.81	0.00249	0.0149	RABGEF1	66142077

chr15	51390177	51390504	328	0.92	0.00249	0.0149	TNFAIP8L3	51384902
chr5	1.21E+08	1.21E+08	331	0.69	0.0025	0.0149	LOX	1.21E+08
chr5	1.21E+08	1.21E+08	331	0.69	0.0025	0.0149	LOX	1.21E+08
chr5	1.21E+08	1.21E+08	331	0.69	0.0025	0.0149	LOX	1.21E+08
chr16	2844654	2844832	179	-1.13	0.0025	0.0149	PRSS41	2843485
chr1	1.09E+08	1.09E+08	740	0.52	0.00252	0.015	HENMT1	1.09E+08
chr1	1.09E+08	1.09E+08	740	0.52	0.00252	0.015	HENMT1	1.09E+08
chr12	15942165	15943055	891	0.42	0.00253	0.0151	EPS8	15940510
chrX	15512746	15512946	201	0.87	0.00255	0.0152	PIR	15509711
chrX	15512746	15512946	201	0.87	0.00255	0.0152	PIR	15509711
chrX	15512746	15512946	201	0.87	0.00255	0.0152	PIR-FIGF	15507432
chr14	64968424	64968716	293	0.75	0.00256	0.0152	ZBTB1	64966291
chr14	64968424	64968716	293	0.75	0.00256	0.0152	ZBTB25	64968563
chr14	64968424	64968716	293	0.75	0.00256	0.0152	ZBTB1	64966291
chr20	47448230	47448539	310	-0.72	0.00257	0.0153	PREX1	47442420
chr8	96089593	96089834	242	1.01	0.00258	0.0153	MIR3150B	96083224
chr8	96089593	96089834	242	1.01	0.00258	0.0153	LOC10537‡	96083148
chr8	96089593	96089834	242	1.01	0.00258	0.0153	LOC10537‡	96083410
chr5	85913554	85914294	741	0.42	0.00259	0.0154	MIR3607	85911313
chr5	85913554	85914294	741	0.42	0.00259	0.0154	COX7C	85908783
chr1	97559572	97560304	733	0.59	0.00262	0.0155	DPYD-AS1	97556478
chr14	24834954	24835481	528	0.69	0.00262	0.0155	NFATC4	24831116
chr14	24834954	24835481	528	0.69	0.00262	0.0155	NFATC4	24833287
chr14	24834954	24835481	528	0.69	0.00262	0.0155	NFATC4	24832225
chr14	24834954	24835481	528	0.69	0.00262	0.0155	NFATC4	24831116
chr14	24834954	24835481	528	0.69	0.00262	0.0155	NFATC4	24833358
chr14	24834954	24835481	528	0.69	0.00262	0.0155	NFATC4	24832225
chr5	1.8E+08	1.8E+08	345	1.03	0.00263	0.0156	LINC00847	1.8E+08
chr5	1.8E+08	1.8E+08	345	1.03	0.00263	0.0156	LINC00847	1.8E+08
chr5	1.8E+08	1.8E+08	345	1.03	0.00263	0.0156	LINC00847	1.8E+08
chr4	56718104	56718422	319	0.74	0.00263	0.0156	EXOC1	56714815
chr4	56718104	56718422	319	0.74	0.00263	0.0156	EXOC1	56714815
chr4	56718104	56718422	319	0.74	0.00263	0.0156	EXOC1	56714815
chr8	1.25E+08	1.25E+08	258	-0.7	0.00264	0.0156	LOC101927	1.25E+08
chr8	1.04E+08	1.04E+08	884	0.33	0.00264	0.0156	SLC25A32	1.04E+08
chr8	1.04E+08	1.04E+08	884	0.33	0.00264	0.0156	DCAF13	1.04E+08
chr8	1.04E+08	1.04E+08	884	0.33	0.00264	0.0156	SLC25A32	1.04E+08
chr8	1.04E+08	1.04E+08	884	0.33	0.00264	0.0156	SLC25A32	1.04E+08
chr1	1.19E+08	1.19E+08	200	0.72	0.00266	0.0157	SPAG17	1.19E+08
chr1	18806930	18807645	716	0.57	0.0027	0.0159	KLHDC7A	18802423
chr20	56284963	56285803	841	0.38	0.00272	0.016	PMEPA1	56283670
chr20	56284963	56285803	841	0.38	0.00272	0.016	PMEPA1	56283670
chr20	56284963	56285803	841	0.38	0.00272	0.016	PMEPA1	56284592
chr20	56284963	56285803	841	0.38	0.00272	0.016	PMEPA1	56283031
chr20	56284963	56285803	841	0.38	0.00272	0.016	NKILA	56280238
chr22	18116004	18116433	430	0.42	0.00273	0.016	ATP6V1E1	18109588
chr22	18116004	18116433	430	0.42	0.00273	0.016	ATP6V1E1	18109588

chr22	18116004	18116433	430	0.42	0.00273	0.016	ATP6V1E1	18109588
chr22	18116004	18116433	430	0.42	0.00273	0.016	BCL2L13	18116349
chr22	18116004	18116433	430	0.42	0.00273	0.016	BCL2L13	18116349
chr22	18116004	18116433	430	0.42	0.00273	0.016	BCL2L13	18116349
chr22	18116004	18116433	430	0.42	0.00273	0.016	BCL2L13	18116349
chr22	18116004	18116433	430	0.42	0.00273	0.016	BCL2L13	18116349
chr22	18116004	18116433	430	0.42	0.00273	0.016	BCL2L13	18116349
chr22	18116004	18116433	430	0.42	0.00273	0.016	BCL2L13	18116349
chr22	18116004	18116433	430	0.42	0.00273	0.016	BCL2L13	18116349
chr22	18116004	18116433	430	0.42	0.00273	0.016	BCL2L13	18116349
chr22	18116004	18116433	430	0.42	0.00273	0.016	BCL2L13	18116349
chr9	1.28E+08	1.28E+08	151	1.3	0.00273	0.016	OLFML2A	1.28E+08
chr2	54751437	54751656	220	1.07	0.00275	0.0161	RPL23AP32	54751358
chr7	1.37E+08	1.37E+08	201	1.22	0.00275	0.0161	LOC349160	1.37E+08
chr13	40176667	40177493	827	0.55	0.00276	0.0162	LHFP	40175356
chr19	15940236	15940474	239	0.96	0.00276	0.0162	UCA1	15934756
chr8	24408047	24408242	196	1.06	0.00277	0.0162	LOC101929	24404131
chr11	94880712	94880879	168	0.9	0.00279	0.0163	LOC101929	94878702
chr1	1.71E+08	1.71E+08	901	0.36	0.0028	0.0163	GORAB	1.7E+08
chr1	1.71E+08	1.71E+08	901	0.36	0.0028	0.0163	GORAB	1.7E+08
chr1	1.71E+08	1.71E+08	901	0.36	0.0028	0.0163	LOC101928	1.7E+08
chr1	1.71E+08	1.71E+08	901	0.36	0.0028	0.0163	GORAB	1.7E+08
chr5	1.4E+08	1.4E+08	170	1.14	0.0028	0.0164	SLC4A9	1.4E+08
chr5	1.4E+08	1.4E+08	170	1.14	0.0028	0.0164	SLC4A9	1.4E+08
chr5	1.4E+08	1.4E+08	170	1.14	0.0028	0.0164	SLC4A9	1.4E+08
chr5	1.4E+08	1.4E+08	170	1.14	0.0028	0.0164	SLC4A9	1.4E+08
chr3	1.81E+08	1.81E+08	711	0.36	0.00281	0.0164	DNAJC19	1.81E+08
chr3	1.81E+08	1.81E+08	711	0.36	0.00281	0.0164	DNAJC19	1.81E+08
chr3	1.81E+08	1.81E+08	711	0.36	0.00281	0.0164	DNAJC19	1.81E+08
chr3	1.81E+08	1.81E+08	711	0.36	0.00281	0.0164	DNAJC19	1.81E+08
chr3	1.81E+08	1.81E+08	711	0.36	0.00281	0.0164	DNAJC19	1.81E+08
chr3	1.81E+08	1.81E+08	711	0.36	0.00281	0.0164	DNAJC19	1.81E+08
chr22	35932149	35932459	311	0.82	0.00286	0.0166	RASD2	35932351
chr18	61571872	61572072	201	1.11	0.00287	0.0167	SERPINB10	61570223
chr2	96804260	96804658	399	-0.91	0.00287	0.0167	ASTL	96802175
chr15	36883163	36883465	303	0.78	0.0029	0.0168	C15orf41	36882069
chr15	36883163	36883465	303	0.78	0.0029	0.0168	C15orf41	36882069
chr8	1.24E+08	1.24E+08	215	0.92	0.00292	0.0169	WDYHV1	1.24E+08
chr8	1.24E+08	1.24E+08	215	0.92	0.00292	0.0169	WDYHV1	1.24E+08
chr8	1.24E+08	1.24E+08	215	0.92	0.00292	0.0169	WDYHV1	1.24E+08
chr8	1.24E+08	1.24E+08	215	0.92	0.00292	0.0169	WDYHV1	1.24E+08
chr11	64577955	64578323	369	0.58	0.00293	0.0169	MEN1	64576188
chr11	64577955	64578323	369	0.58	0.00293	0.0169	MEN1	64576035
chr11	64577955	64578323	369	0.58	0.00293	0.0169	MEN1	64576188
chr11	64577955	64578323	369	0.58	0.00293	0.0169	MEN1	64576766
chr11	64577955	64578323	369	0.58	0.00293	0.0169	MEN1	64576766
chr11	64577955	64578323	369	0.58	0.00293	0.0169	MEN1	64576213

chr11	64577955	64578323	369	0.58	0.00293	0.0169	MEN1	64576035
chr11	7531251	7531451	201	0.97	0.00294	0.017	PPFIBP2	7529995
chr19	49528944	49529258	315	1.71	0.00298	0.0172	CGB3	49525632
chr5	1.34E+08	1.34E+08	199	0.78	0.003	0.0173	SEC24A	1.34E+08
chr5	1.34E+08	1.34E+08	199	0.78	0.003	0.0173	SEC24A	1.34E+08
chr2	40661894	40662424	531	0.43	0.00301	0.0173	SLC8A1	40655444
chr2	40661894	40662424	531	0.43	0.00301	0.0173	SLC8A1	40655444
chr2	40661894	40662424	531	0.43	0.00301	0.0173	SLC8A1	40655444
chr2	40661894	40662424	531	0.43	0.00301	0.0173	SLC8A1	40655444
chr2	1.5E+08	1.5E+08	321	0.95	0.00301	0.0173	LYPD6B	1.5E+08
chr19	17190857	17191127	271	0.89	0.00301	0.0173	HAUS8	17184343
chr19	17190857	17191127	271	0.89	0.00301	0.0173	HAUS8	17184343
chr9	79114363	79114559	197	0.75	0.00302	0.0174	GCNT1	79110548
chr7	51383150	51383503	354	-0.76	0.00303	0.0174	COBL	51382515
chr7	51383150	51383503	354	-0.76	0.00303	0.0174	COBL	51382515
chr7	51383150	51383503	354	-0.76	0.00303	0.0174	COBL	51382515
chr15	42130653	42130966	314	-0.99	0.00305	0.0175	PLA2G4B	42126010
chr7	69063053	69063606	554	0.66	0.00306	0.0175	AUTS2	69058904
chr7	69063053	69063606	554	0.66	0.00306	0.0175	AUTS2	69058904
chr7	69063053	69063606	554	0.66	0.00306	0.0175	AUTS2	69058904
chr7	69063053	69063606	554	0.66	0.00306	0.0175	LOC100507	69060481
chr6	46702809	46703870	1062	0.55	0.00307	0.0176	PLA2G7	46701151
chr6	46702809	46703870	1062	0.55	0.00307	0.0176	PLA2G7	46701430
chr8	1.11E+08	1.11E+08	384	-0.65	0.00307	0.0176	KCNV1	1.11E+08
chr10	22535484	22536191	708	-0.5	0.00308	0.0176	LOC10013(	22536000
chr5	41922103	41922236	134	0.83	0.00309	0.0177	FBXO4	41920355
chr5	41922103	41922236	134	0.83	0.00309	0.0177	FBXO4	41920355
chr5	41922103	41922236	134	0.83	0.00309	0.0177	FBXO4	41920353
chr17	47585450	47586240	791	0.47	0.0031	0.0177	MIR6165	47583178
chr3	1.77E+08	1.77E+08	243	1	0.00313	0.0179	TBL1XR1	1.77E+08
chr7	38380906	38381264	359	-0.91	0.00316	0.018	TRG-AS1	38376177
chr10	1.03E+08	1.03E+08	201	1.24	0.00317	0.018	POLL	1.03E+08
chr10	1.03E+08	1.03E+08	201	1.24	0.00317	0.018	POLL	1.03E+08
chr10	1.03E+08	1.03E+08	201	1.24	0.00317	0.018	DPCD	1.03E+08
chr10	1.03E+08	1.03E+08	201	1.24	0.00317	0.018	POLL	1.03E+08
chr10	1.03E+08	1.03E+08	201	1.24	0.00317	0.018	POLL	1.03E+08
chr10	1.03E+08	1.03E+08	201	1.24	0.00317	0.018	POLL	1.03E+08
chr19	35598452	35598614	163	-1.26	0.00318	0.0181	HPN-AS1	35595208
chr19	35598452	35598614	163	-1.26	0.00318	0.0181	HPN-AS1	35595175
chr2	2.28E+08	2.28E+08	796	-0.47	0.00318	0.0181	MFF	2.28E+08
chr2	2.28E+08	2.28E+08	796	-0.47	0.00318	0.0181	MFF	2.28E+08
chr2	2.28E+08	2.28E+08	796	-0.47	0.00318	0.0181	MFF	2.28E+08
chr2	2.28E+08	2.28E+08	796	-0.47	0.00318	0.0181	MFF	2.28E+08
chr2	2.28E+08	2.28E+08	796	-0.47	0.00318	0.0181	MFF	2.28E+08
chr2	2.28E+08	2.28E+08	796	-0.47	0.00318	0.0181	MFF	2.28E+08
chr2	2.28E+08	2.28E+08	796	-0.47	0.00318	0.0181	MFF	2.28E+08
chr2	2.28E+08	2.28E+08	796	-0.47	0.00318	0.0181	MFF	2.28E+08

chr2	2.28E+08	2.28E+08	796	-0.47	0.00318	0.0181	MFF	2.28E+08
chr2	2.28E+08	2.28E+08	796	-0.47	0.00318	0.0181	MFF	2.28E+08
chr2	2.28E+08	2.28E+08	796	-0.47	0.00318	0.0181	LOC654841	2.28E+08
chr5	1.41E+08	1.41E+08	294	0.64	0.00319	0.0181	LOC100505	1.41E+08
chr2	263914	265159	1246	-0.38	0.00319	0.0182	SH3YL1	262097
chr2	263914	265159	1246	-0.38	0.00319	0.0182	SH3YL1	262097
chr2	263914	265159	1246	-0.38	0.00319	0.0182	SH3YL1	260786
chr2	263914	265159	1246	-0.38	0.00319	0.0182	SH3YL1	262866
chr2	263914	265159	1246	-0.38	0.00319	0.0182	SH3YL1	262743
chr2	263914	265159	1246	-0.38	0.00319	0.0182	SH3YL1	262392
chr2	263914	265159	1246	-0.38	0.00319	0.0182	SH3YL1	262866
chr2	263914	265159	1246	-0.38	0.00319	0.0182	SH3YL1	259130
chr2	263914	265159	1246	-0.38	0.00319	0.0182	ACP1	259868
chr2	263914	265159	1246	-0.38	0.00319	0.0182	ACP1	259868
chr2	263914	265159	1246	-0.38	0.00319	0.0182	ACP1	259868
chr2	263914	265159	1246	-0.38	0.00319	0.0182	ACP1	259868
chr17	57920305	57921895	1591	0.34	0.00321	0.0182	MIR21	57913626
chr11	57508657	57509263	607	0.37	0.00322	0.0183	C11orf31	57503721
chr4	1.47E+08	1.47E+08	563	0.71	0.00324	0.0184	ZNF827	1.47E+08
chr4	1.47E+08	1.47E+08	563	0.71	0.00324	0.0184	ZNF827	1.47E+08
chr9	1.25E+08	1.25E+08	199	1.06	0.00324	0.0184	OR1L4	1.25E+08
chr1	32478980	32479530	551	0.6	0.00325	0.0184	KHDRBS1	32474294
chr1	32478980	32479530	551	0.6	0.00325	0.0184	KHDRBS1	32474294
chr1	32478980	32479530	551	0.6	0.00325	0.0184	KHDRBS1	32474294
chr1	32478980	32479530	551	0.6	0.00325	0.0184	KHDRBS1	32474294
chr19	41873755	41874015	261	0.84	0.00325	0.0184	B9D2	41868078
chr21	39574461	39574659	199	0.65	0.00326	0.0184	DSCR10	39573249
chr2	2.41E+08	2.41E+08	706	0.72	0.00326	0.0185	MIR149	2.41E+08
chr2	2.41E+08	2.41E+08	706	0.72	0.00326	0.0185	PP14571	2.41E+08
chr5	31190461	31190661	201	1.05	0.00326	0.0185	CDH6	31188761
chr12	15081942	15082220	279	0.7	0.00327	0.0185	ERP27	15080059
chr2	2.28E+08	2.28E+08	939	-0.41	0.00328	0.0185	RHBDD1	2.28E+08
chr2	2.28E+08	2.28E+08	939	-0.41	0.00328	0.0185	RHBDD1	2.28E+08
chr2	54784921	54785823	903	0.53	0.00334	0.0188	SPTBN1	54780530
chr5	1.34E+08	1.34E+08	326	1.11	0.00334	0.0188	SAR1B	1.34E+08
chr5	1.34E+08	1.34E+08	326	1.11	0.00334	0.0188	SAR1B	1.34E+08
chr10	10834637	10834947	311	0.58	0.00337	0.0189	SFTA1P	10834877
chr22	25506736	25507664	929	0.39	0.00337	0.0189	LOC100128	25506659
chr1	1.55E+08	1.55E+08	479	0.65	0.00337	0.019	SHC1	1.55E+08
chr1	1.55E+08	1.55E+08	479	0.65	0.00337	0.019	SHC1	1.55E+08
chr1	1.55E+08	1.55E+08	479	0.65	0.00337	0.019	SHC1	1.55E+08
chr1	1.55E+08	1.55E+08	479	0.65	0.00337	0.019	SHC1	1.55E+08
chr1	1.55E+08	1.55E+08	479	0.65	0.00337	0.019	MIR4258	1.55E+08
chr1	1.55E+08	1.55E+08	479	0.65	0.00337	0.019	CKS1B	1.55E+08
chr1	1.55E+08	1.55E+08	479	0.65	0.00337	0.019	CKS1B	1.55E+08
chr1	1.55E+08	1.55E+08	479	0.65	0.00337	0.019	SHC1	1.55E+08
chr10	5637931	5638370	440	0.63	0.00338	0.019	LOC105376	5636081

chr5	32443712	32445012	1301	-0.34	0.0034	0.0191	ZFR	32442844
chr11	63702091	63702288	198	0.91	0.00341	0.0191	NAA40	63701441
chr11	63702091	63702288	198	0.91	0.00341	0.0191	NAA40	63701802
chr15	75493141	75493376	236	-0.82	0.00345	0.0193	C15orf39	75489220
chr21	43171771	43171967	197	1.36	0.00345	0.0193	MIR6814	43165001
chr1	1.92E+08	1.92E+08	198	1.48	0.00346	0.0193	RGS18	1.92E+08
chr2	2.2E+08	2.2E+08	526	0.67	0.00346	0.0193	ABCB6	2.2E+08
chr3	69811638	69811931	294	-0.99	0.00346	0.0193	MITF	69807961
chr3	69811638	69811931	294	-0.99	0.00346	0.0193	MITF	69807706
chr10	1.28E+08	1.28E+08	703	-0.57	0.00347	0.0194	BCCIP	1.28E+08
chr10	1.28E+08	1.28E+08	703	-0.57	0.00347	0.0194	BCCIP	1.28E+08
chr10	1.28E+08	1.28E+08	703	-0.57	0.00347	0.0194	BCCIP	1.28E+08
chr10	1.28E+08	1.28E+08	703	-0.57	0.00347	0.0194	UROS	1.28E+08
chr9	74525976	74526621	646	0.3	0.0035	0.0195	C9orf85	74521422
chr9	74525976	74526621	646	0.3	0.0035	0.0195	ABHD17B	74524161
chr9	74525976	74526621	646	0.3	0.0035	0.0195	ABHD17B	74524161
chr2	2.38E+08	2.39E+08	1096	-0.6	0.00351	0.0196	RAB17	2.38E+08
chr2	2.38E+08	2.39E+08	1096	-0.6	0.00351	0.0196	RAB17	2.38E+08
chr9	1.24E+08	1.24E+08	321	0.72	0.00351	0.0196	GSN-AS1	1.24E+08
chr10	77307153	77307371	219	1.28	0.00352	0.0196	MIR606	77307215
chr4	47832410	47832610	201	0.96	0.00353	0.0196	LOC101927	47828361
chr4	47832410	47832610	201	0.96	0.00353	0.0196	LOC101927	47828361
chr2	2.31E+08	2.31E+08	153	0.97	0.00355	0.0197	SP110	2.31E+08
chr2	2.31E+08	2.31E+08	153	0.97	0.00355	0.0197	SP110	2.31E+08
chr2	2.31E+08	2.31E+08	153	0.97	0.00355	0.0197	SP110	2.31E+08
chr2	2.31E+08	2.31E+08	153	0.97	0.00355	0.0197	SP140	2.31E+08
chr2	2.31E+08	2.31E+08	153	0.97	0.00355	0.0197	SP140	2.31E+08
chr2	2.31E+08	2.31E+08	153	0.97	0.00355	0.0197	SP140	2.31E+08
chr2	2.31E+08	2.31E+08	153	0.97	0.00355	0.0197	SP140	2.31E+08
chr2	2.31E+08	2.31E+08	153	0.97	0.00355	0.0197	SP140	2.31E+08
chr1	92764158	92764786	629	0.47	0.00355	0.0197	RPAP2	92759521
chr1	92764158	92764786	629	0.47	0.00355	0.0197	GLMN	92762566
chr13	61989191	61989525	335	0.67	0.00355	0.0197	PCDH20	61987655
chr8	49838189	49838358	170	0.88	0.00356	0.0198	SNAI2	49831999
chr19	58906204	58907086	883	-0.57	0.00358	0.0198	RNF225	58902456
chr1	1.62E+08	1.62E+08	280	-0.86	0.00358	0.0198	C1orf226	1.62E+08
chr1	1.62E+08	1.62E+08	280	-0.86	0.00358	0.0198	C1orf111	1.62E+08
chr7	1.16E+08	1.16E+08	208	1.75	0.00361	0.02	LOC102724	1.16E+08
chr7	1.16E+08	1.16E+08	208	1.75	0.00361	0.02	LOC102724	1.16E+08
chr7	29020292	29020776	485	0.73	0.00362	0.02	LOC100506	29014582
chr11	1.11E+08	1.11E+08	199	0.62	0.00364	0.0201	MIR4491	1.11E+08
chr11	58910058	58910807	750	0.35	0.00367	0.0202	FAM111A	58905218
chr11	58910058	58910807	750	0.35	0.00367	0.0202	FAM111A	58905218
chr11	58910058	58910807	750	0.35	0.00367	0.0202	FAM111A	58905218
chr11	58910058	58910807	750	0.35	0.00367	0.0202	LOC101927	58908281
chr11	58910058	58910807	750	0.35	0.00367	0.0202	FAM111A	58905218
chr11	58910058	58910807	750	0.35	0.00367	0.0202	FAM111A	58907251

chr11	58910058	58910807	750	0.35	0.00367	0.0202	FAM111A	58905218
chr11	58910058	58910807	750	0.35	0.00367	0.0202	FAM111A	58907251
chr11	58910058	58910807	750	0.35	0.00367	0.0202	FAM111A	58907251
chr14	62161775	62162357	583	0.35	0.00367	0.0202	HIF1A	62157118
chr14	62161775	62162357	583	0.35	0.00367	0.0202	HIF1A	62159339
chr14	62161775	62162357	583	0.35	0.00367	0.0202	HIF1A	62157118
chr14	62161775	62162357	583	0.35	0.00367	0.0202	HIF1A-AS1	62160541
chr3	1.79E+08	1.79E+08	198	0.75	0.00369	0.0203	ZMAT3	1.79E+08
chr3	1.79E+08	1.79E+08	198	0.75	0.00369	0.0203	ZMAT3	1.79E+08
chr15	85523621	85524830	1210	-0.4	0.00371	0.0204	PDE8A	85520204
chr15	85523621	85524830	1210	-0.4	0.00371	0.0204	PDE8A	85520204
chr15	85523621	85524830	1210	-0.4	0.00371	0.0204	PDE8A	85518743
chr19	46145478	46145926	449	-0.63	0.00372	0.0205	EML2	46140675
chr19	46145478	46145926	449	-0.63	0.00372	0.0205	EML2	46144985
chr19	46145478	46145926	449	-0.63	0.00372	0.0205	EML2	46140675
chr19	46145478	46145926	449	-0.63	0.00372	0.0205	MIR330	46140345
chr19	46145478	46145926	449	-0.63	0.00372	0.0205	EML2-AS1	46139751
chr19	46145478	46145926	449	-0.63	0.00372	0.0205	EML2-AS1	46139751
chr4	1.56E+08	1.56E+08	584	0.45	0.00374	0.0206	MAP9	1.56E+08
chr17	59489002	59490045	1044	0.51	0.00376	0.0206	C17orf82	59484111
chr20	10635242	10635595	354	1.02	0.00376	0.0206	MIR6870	10628343
chr6	1.7E+08	1.7E+08	625	-0.56	0.00379	0.0207	WDR27	1.7E+08
chr6	1.7E+08	1.7E+08	625	-0.56	0.00379	0.0207	WDR27	1.7E+08
chr6	1.7E+08	1.7E+08	625	-0.56	0.00379	0.0207	C6orf120	1.7E+08
chr6	1.7E+08	1.7E+08	625	-0.56	0.00379	0.0207	C6orf120	1.7E+08
chr5	59064326	59064743	418	0.87	0.00383	0.021	PDE4D	59062438
chr22	25839771	25839970	200	0.65	0.00384	0.021	CRYBB2P1	25839053
chr22	25839771	25839970	200	0.65	0.00384	0.021	CRYBB2P1	25839053
chr16	57661439	57661757	319	0.56	0.00387	0.0211	ADGRG1	57657137
chr16	57661439	57661757	319	0.56	0.00387	0.0211	ADGRG1	57657137
chr16	57661439	57661757	319	0.56	0.00387	0.0211	ADGRG1	57657137
chr16	57661439	57661757	319	0.56	0.00387	0.0211	ADGRG1	57657137
chr16	57661439	57661757	319	0.56	0.00387	0.0211	ADGRG1	57657137
chr1	77328252	77328449	198	1.48	0.00387	0.0211	ST6GALNA(	77328185
chr12	14994785	14995050	266	0.77	0.00389	0.0212	ART4	14994413
chr5	39077237	39077345	109	-1.08	0.00392	0.0213	RICTOR	39072510
chr5	39077237	39077345	109	-1.08	0.00392	0.0213	RICTOR	39072510
chr5	39077237	39077345	109	-1.08	0.00392	0.0213	RICTOR	39072510
chr5	57880062	57880260	199	0.92	0.00393	0.0214	RAB3C	57873870
chr8	9598233	9598306	74	1.27	0.00394	0.0214	MIR597	9594181
chr9	1.16E+08	1.16E+08	339	-0.59	0.00395	0.0215	RGS3	1.16E+08
chr9	1.16E+08	1.16E+08	339	-0.59	0.00395	0.0215	RGS3	1.16E+08
chr6	17706848	17707351	504	-0.45	0.00396	0.0215	NUP153	17705065
chr6	17706848	17707351	504	-0.45	0.00396	0.0215	NUP153	17705065
chr6	17706848	17707351	504	-0.45	0.00396	0.0215	NUP153	17705065
chr6	17706848	17707351	504	-0.45	0.00396	0.0215	LOC10537(	17701360
chr6	17706848	17707351	504	-0.45	0.00396	0.0215	LOC10537(	17702273

chr4	1.1E+08	1.1E+08	550	0.51	0.00397	0.0216	SEC24B	1.1E+08
chr4	1.1E+08	1.1E+08	550	0.51	0.00397	0.0216	SEC24B	1.1E+08
chr4	1.1E+08	1.1E+08	550	0.51	0.00397	0.0216	SEC24B	1.1E+08
chr4	1.1E+08	1.1E+08	550	0.51	0.00397	0.0216	SEC24B	1.1E+08
chr4	1.1E+08	1.1E+08	550	0.51	0.00397	0.0216	SEC24B	1.1E+08
chr4	1.1E+08	1.1E+08	550	0.51	0.00397	0.0216	SEC24B-AS	1.1E+08
chr1	1.71E+08	1.71E+08	201	1.15	0.00397	0.0216	FMO1	1.71E+08
chr10	17171215	17171414	200	0.61	0.00399	0.0216	CUBN	17169816
chr12	56136701	56136920	220	0.92	0.004	0.0217	GDF11	56132063
chr15	25378735	25378934	200	0.61	0.00401	0.0217	PWAR1	25375788
chr11	1.14E+08	1.14E+08	517	-0.89	0.00401	0.0217	HTR3A	1.14E+08
chr11	1.14E+08	1.14E+08	517	-0.89	0.00401	0.0217	HTR3A	1.14E+08
chr11	1.14E+08	1.14E+08	517	-0.89	0.00401	0.0217	HTR3A	1.14E+08
chr11	1.14E+08	1.14E+08	517	-0.89	0.00401	0.0217	HTR3A	1.14E+08
chr2	2.17E+08	2.17E+08	577	-0.45	0.00403	0.0218	SMARCAL1	2.17E+08
chr2	2.17E+08	2.17E+08	577	-0.45	0.00403	0.0218	SMARCAL1	2.17E+08
chr9	35114151	35114384	234	0.83	0.00404	0.0218	FAM214B	35113908
chr9	35114151	35114384	234	0.83	0.00404	0.0218	FAM214B	35109371
chr9	35114151	35114384	234	0.83	0.00404	0.0218	FAM214B	35113969
chr11	6633328	6634176	849	0.33	0.00405	0.0219	TAF10	6631475
chr5	1.11E+08	1.11E+08	201	0.95	0.00406	0.0219	STARD4-AS	1.11E+08
chr7	76022369	76023192	824	0.44	0.00409	0.022	ZP3	76021840
chr8	81786163	81787131	969	0.5	0.00411	0.0221	ZNF704	81785016
chr2	1.63E+08	1.63E+08	439	0.46	0.00412	0.0221	FAP	1.63E+08
chr2	1.63E+08	1.63E+08	439	0.46	0.00412	0.0221	FAP	1.63E+08
chr7	2353936	2354421	486	-0.52	0.00413	0.0222	SNX8	2352110
chr3	1.68E+08	1.68E+08	634	0.44	0.00413	0.0222	EGFEM1P	1.68E+08
chr11	43959074	43959263	190	-1.05	0.00415	0.0223	C11orf96	43959105
chr3	1.57E+08	1.57E+08	241	-0.61	0.00416	0.0223	PA2G4P4	1.57E+08
chr1	1E+08	1E+08	476	0.79	0.00416	0.0223	PALMD	1E+08
chr2	2.42E+08	2.42E+08	500	-0.54	0.00418	0.0224	C2orf54	2.42E+08
chr2	2.42E+08	2.42E+08	500	-0.54	0.00418	0.0224	C2orf54	2.42E+08
chr7	82076176	82076374	199	1.06	0.00418	0.0224	CACNA2D1	82071122
chr7	82076176	82076374	199	1.06	0.00418	0.0224	CACNA2D1	82071122
chr2	1.29E+08	1.29E+08	239	0.76	0.00418	0.0224	POLR2D	1.29E+08
chr19	45174800	45175141	342	0.83	0.00421	0.0225	CEACAM19	45169723
chr19	45174800	45175141	342	0.83	0.00421	0.0225	CEACAM19	45169723
chr7	1.3E+08	1.3E+08	526	0.57	0.00422	0.0225	CPA4	1.3E+08
chr7	1.3E+08	1.3E+08	526	0.57	0.00422	0.0225	CPA4	1.3E+08
chr1	41174570	41175316	747	0.53	0.00422	0.0226	NFYC	41169994
chr7	3082738	3082883	146	-1.54	0.00422	0.0226	CARD11	3081579
chr12	1.02E+08	1.02E+08	703	-0.35	0.00422	0.0226	GNPTAB	1.02E+08
chr19	49559291	49559643	353	0.72	0.00431	0.0229	CGB7	49556997
chr8	66934479	66934719	241	-0.75	0.00433	0.023	DNAJC5B	66928790
chr16	56895132	56895519	388	0.85	0.00433	0.023	SLC12A3	56894118
chr16	56895132	56895519	388	0.85	0.00433	0.023	SLC12A3	56894118
chr16	56895132	56895519	388	0.85	0.00433	0.023	SLC12A3	56894118

chr10	1.3E+08	1.3E+08	201	1.15	0.00434	0.0231	PTPRE	1.3E+08
chr22	29279048	29279732	685	-0.53	0.00435	0.0231	ZNRF3	29274754
chr17	5187363	5187463	101	1.69	0.00436	0.0231	RABEP1	5180557
chr17	5187363	5187463	101	1.69	0.00436	0.0231	RABEP1	5180557
chr17	5187363	5187463	101	1.69	0.00436	0.0231	RABEP1	5180557
chr17	5187363	5187463	101	1.69	0.00436	0.0231	RABEP1	5180557
chr1	2.24E+08	2.24E+08	659	0.38	0.0044	0.0233	CAPN2	2.24E+08
chr19	14317677	14318458	782	0.64	0.00441	0.0234	ADGRL1	14314997
chr19	14317677	14318458	782	0.64	0.00441	0.0234	ADGRL1	14314997
chr9	78505129	78506014	886	0.4	0.00442	0.0234	PCSK5	78500559
chr9	78505129	78506014	886	0.4	0.00442	0.0234	PCSK5	78500559
chr9	78505129	78506014	886	0.4	0.00442	0.0234	PCSK5	78500559
chr4	69963966	69964039	74	1.26	0.00442	0.0234	UGT2B7	69957192
chr5	15271620	15272133	514	0.69	0.00446	0.0235	LOC10192!	15264669
chr3	1.6E+08	1.6E+08	680	-0.47	0.00446	0.0236	IL12A	1.6E+08
chr6	1.7E+08	1.7E+08	815	-0.55	0.00446	0.0236	TCTE3	1.7E+08
chr6	1.7E+08	1.7E+08	815	-0.55	0.00446	0.0236	ERMARD	1.7E+08
chr6	1.7E+08	1.7E+08	815	-0.55	0.00446	0.0236	ERMARD	1.7E+08
chr6	1.7E+08	1.7E+08	815	-0.55	0.00446	0.0236	ERMARD	1.7E+08
chr6	1.7E+08	1.7E+08	815	-0.55	0.00446	0.0236	ERMARD	1.7E+08
chr16	48418504	48419810	1307	0.45	0.00448	0.0236	SIAH1	48417229
chr10	23727570	23728328	759	0.32	0.00448	0.0237	OTUD1	23723197
chr15	25683195	25684503	1309	0.32	0.00449	0.0237	UBE3A	25682175
chr15	25683195	25684503	1309	0.32	0.00449	0.0237	UBE3A	25682175
chr13	99630278	99630837	560	0.75	0.0045	0.0237	DOCK9	99628338
chr13	99630278	99630837	560	0.75	0.0045	0.0237	DOCK9	99628338
chr1	2E+08	2E+08	201	1.52	0.00453	0.0239	NR5A2	2E+08
chr1	2E+08	2E+08	201	1.52	0.00453	0.0239	NR5A2	2E+08
chr3	1.01E+08	1.01E+08	775	0.39	0.00453	0.0239	NXPE3	1.01E+08
chr3	1.01E+08	1.01E+08	775	0.39	0.00453	0.0239	NXPE3	1.01E+08
chr8	1.28E+08	1.28E+08	1096	-0.22	0.00454	0.0239	FAM84B	1.28E+08
chr20	48429051	48429643	593	-0.56	0.00456	0.024	SLC9A8	48424249
chr20	48429051	48429643	593	-0.56	0.00456	0.024	SLC9A8	48424249
chr20	48429051	48429643	593	-0.56	0.00456	0.024	SLC9A8	48424249
chr20	48429051	48429643	593	-0.56	0.00456	0.024	SLC9A8	48424249
chr20	48429051	48429643	593	-0.56	0.00456	0.024	SLC9A8	48424249
chr20	48429051	48429643	593	-0.56	0.00456	0.024	SLC9A8	48424249
chr3	57208868	57209068	201	1.13	0.00456	0.024	IL17RD	57202345
chr2	13149270	13149429	160	1.33	0.00456	0.024	LOC10050€	13145138
chr4	87812923	87813712	790	0.52	0.0046	0.0241	C4orf36	87811575
chr13	62784114	62784313	200	1.46	0.0046	0.0241	LINC01075	62781414
chr3	1.79E+08	1.79E+08	1169	0.4	0.00461	0.0241	USP13	1.79E+08
chr6	1.5E+08	1.5E+08	302	-0.83	0.00461	0.0242	RAET1E-AS	1.5E+08
chr14	21566721	21567060	340	0.71	0.00461	0.0242	ZNF219	21564808
chr14	21566721	21567060	340	0.71	0.00461	0.0242	ZNF219	21565173
chr14	21566721	21567060	340	0.71	0.00461	0.0242	TMEM253	21562095
chr1	26678444	26678623	180	-1.06	0.00463	0.0242	AIM1L	26678621

chr1	1.99E+08	1.99E+08	277	0.59	0.00463	0.0242	MIR181A1I	1.99E+08
chr2	17721654	17721983	330	0.82	0.00463	0.0242	VSNL1	17716806
chr9	3490487	3490686	200	0.81	0.00464	0.0243	RFX3	3487487
chr5	86563318	86564192	875	0.41	0.00466	0.0244	RASA1	86559740
chr5	86563318	86564192	875	0.41	0.00466	0.0244	RASA1	86559069
chr12	49075578	49076320	743	0.39	0.00467	0.0244	KANSL2	49074035
chr20	749158	749548	391	-1.04	0.00468	0.0244	SLC52A3	747228
chr5	40755190	40756339	1150	0.33	0.00471	0.0246	TTC33	40754072
chr15	78426254	78426483	230	1	0.00473	0.0246	CIB2	78421877
chr15	78426254	78426483	230	1	0.00473	0.0246	CIB2	78421877
chr15	78426254	78426483	230	1	0.00473	0.0246	CIB2	78421877
chr15	78426254	78426483	230	1	0.00473	0.0246	CIB2	78421877
chr15	78426254	78426483	230	1	0.00473	0.0246	CIB2	78421877
chr12	49350030	49350642	613	0.53	0.00477	0.0248	ARF3	49349252
chr18	78009866	78010065	200	1.18	0.00482	0.025	PARD6G	78003397
chr10	25402122	25402875	754	-0.67	0.00483	0.025	LINC01516	25396986
chr5	8460088	8460288	201	1.03	0.00484	0.0251	LOC72950(	8455677
chr5	8460088	8460288	201	1.03	0.00484	0.0251	MIR4458	8456037
chrX	13582605	13582805	201	0.98	0.00485	0.0251	EGFL6	13582693
chrX	13582605	13582805	201	0.98	0.00485	0.0251	EGFL6	13582693
chr12	10513743	10513889	147	0.83	0.00488	0.0252	LOC10192{	10511367
chr16	70562663	70562863	201	0.72	0.00488	0.0252	SNORD111	70558401
chr11	534664	534832	169	0.89	0.00488	0.0252	HRAS	533567
chr11	534664	534832	169	0.89	0.00488	0.0252	HRAS	533567
chr11	534664	534832	169	0.89	0.00488	0.0252	HRAS	533567
chr11	534664	534832	169	0.89	0.00488	0.0252	HRAS	533567
chr11	534664	534832	169	0.89	0.00488	0.0252	LRRC56	532521
chr12	1.24E+08	1.24E+08	863	-0.46	0.0049	0.0253	ZNF664-FA	1.24E+08
chr12	1.24E+08	1.24E+08	863	-0.46	0.0049	0.0253	ZNF664	1.24E+08
chr12	1.24E+08	1.24E+08	863	-0.46	0.0049	0.0253	CCDC92	1.24E+08
chr12	1.24E+08	1.24E+08	863	-0.46	0.0049	0.0253	ZNF664	1.24E+08
chr12	1.24E+08	1.24E+08	863	-0.46	0.0049	0.0253	CCDC92	1.24E+08
chr12	1.24E+08	1.24E+08	863	-0.46	0.0049	0.0253	CCDC92	1.24E+08
chr12	1.24E+08	1.24E+08	863	-0.46	0.0049	0.0253	CCDC92	1.24E+08
chr12	1.24E+08	1.24E+08	863	-0.46	0.0049	0.0253	CCDC92	1.24E+08
chr12	1.24E+08	1.24E+08	863	-0.46	0.0049	0.0253	CCDC92	1.24E+08
chr6	27106525	27106724	200	1.05	0.00491	0.0254	HIST1H4I	27102087
chr4	1.03E+08	1.03E+08	201	0.67	0.00491	0.0254	BANK1	1.03E+08
chr1	2.3E+08	2.3E+08	577	-0.36	0.00492	0.0254	GALNT2	2.3E+08
chr1	2.3E+08	2.3E+08	577	-0.36	0.00492	0.0254	GALNT2	2.3E+08
chr12	98985148	98985347	200	0.59	0.00492	0.0254	SLC25A3	98982402
chr12	98985148	98985347	200	0.59	0.00492	0.0254	SLC25A3	98982402
chr12	98985148	98985347	200	0.59	0.00492	0.0254	SLC25A3	98982402
chr5	1.24E+08	1.24E+08	284	-0.88	0.00493	0.0254	ZNF608	1.24E+08
chr9	32550790	32551136	347	0.63	0.00493	0.0254	TOPORS	32550626
chr9	32550790	32551136	347	0.63	0.00493	0.0254	TOPORS	32550626
chr9	32550790	32551136	347	0.63	0.00493	0.0254	TOPORS-AS	32547231

chr9	32550790	32551136	347	0.63	0.00493	0.0254	TOPORS-AS	32547231
chr9	32550790	32551136	347	0.63	0.00493	0.0254	TOPORS-AS	32546714
chr9	32550790	32551136	347	0.63	0.00493	0.0254	TOPORS-AS	32546141
chr10	65190070	65190342	273	1.32	0.00493	0.0254	LOC105378	65185334
chr7	1.32E+08	1.32E+08	199	1.16	0.00494	0.0255	PLXNA4	1.32E+08
chr20	44462183	44462686	504	0.55	0.00495	0.0255	SNX21	44457469
chr20	44462183	44462686	504	0.55	0.00495	0.0255	SNX21	44457469
chr20	44462183	44462686	504	0.55	0.00495	0.0255	SNX21	44457469
chr20	44462183	44462686	504	0.55	0.00495	0.0255	SNX21	44457469
chr6	1.35E+08	1.35E+08	463	0.44	0.00497	0.0256	HBS1L	1.35E+08
chr6	1.35E+08	1.35E+08	463	0.44	0.00497	0.0256	HBS1L	1.35E+08
chr6	1.35E+08	1.35E+08	463	0.44	0.00497	0.0256	HBS1L	1.35E+08
chr6	41471633	41472022	390	1.08	0.00498	0.0256	LINC01276	41465181
chr1	16485644	16485758	115	-1.29	0.00499	0.0256	EPHA2	16480582
chr14	35516962	35517159	198	0.57	0.00499	0.0256	FAM177A1	35510458
chr14	35516962	35517159	198	0.57	0.00499	0.0256	FAM177A1	35510609
chr2	2.19E+08	2.19E+08	211	-0.8	0.00499	0.0257	AAMP	2.19E+08
chr2	2.19E+08	2.19E+08	211	-0.8	0.00499	0.0257	AAMP	2.19E+08
chr5	1512182	1512432	251	-0.69	0.00501	0.0257	MIR6075	1508971
chr15	33602926	33603161	236	1.25	0.00501	0.0257	RYR3	33598162
chr15	33602926	33603161	236	1.25	0.00501	0.0257	RYR3	33598162
chr15	33602926	33603161	236	1.25	0.00501	0.0257	LOC101928	33600446
chr15	33602926	33603161	236	1.25	0.00501	0.0257	LOC101928	33600860
chr13	1.15E+08	1.15E+08	379	1.06	0.00502	0.0257	LINC00565	1.15E+08
chrX	38663141	38663453	313	-0.51	0.00502	0.0258	MID1IP1-A	38661136
chr1	1.82E+08	1.82E+08	866	0.4	0.00503	0.0258	GLUL	1.82E+08
chr1	1.82E+08	1.82E+08	866	0.4	0.00503	0.0258	GLUL	1.82E+08
chr1	1.82E+08	1.82E+08	866	0.4	0.00503	0.0258	GLUL	1.82E+08
chr10	1.1E+08	1.1E+08	201	0.76	0.00505	0.0259	LINC01435	1.1E+08
chr3	93781511	93782231	721	0.31	0.00505	0.0259	DHFRL1	93780067
chr3	93781511	93782231	721	0.31	0.00505	0.0259	DHFRL1	93779831
chr3	93781511	93782231	721	0.31	0.00505	0.0259	NSUN3	93776854
chr3	98107128	98107255	128	2.09	0.00506	0.0259	OR5K3	98104509
chr5	40052972	40053261	290	-0.81	0.0051	0.0261	LINC00603	40047392
chr2	99757452	99758303	852	0.34	0.0051	0.0261	TSGA10	99756037
chr2	99757452	99758303	852	0.34	0.0051	0.0261	C2orf15	99753075
chr2	99757452	99758303	852	0.34	0.0051	0.0261	C2orf15	99753075
chr5	1.76E+08	1.76E+08	310	0.76	0.00511	0.0261	CLTB	1.76E+08
chr5	1.76E+08	1.76E+08	310	0.76	0.00511	0.0261	CLTB	1.76E+08
chr5	1.76E+08	1.76E+08	310	0.76	0.00511	0.0261	CLTB	1.76E+08
chr7	27195683	27196445	763	0.47	0.00512	0.0261	HOXA7	27194296
chr6	20211487	20211688	202	-0.63	0.00513	0.0262	MBOAT1	20210695
chr6	20211487	20211688	202	-0.63	0.00513	0.0262	MBOAT1	20210695
chr20	44539371	44539939	569	0.39	0.00516	0.0263	PLTP	44539003
chr20	44539371	44539939	569	0.39	0.00516	0.0263	PLTP	44537633
chr20	44539371	44539939	569	0.39	0.00516	0.0263	PLTP	44539003
chr20	44539371	44539939	569	0.39	0.00516	0.0263	PLTP	44539003

chr3	40498662	40498995	334	0.72	0.00516	0.0263	RPL14	40493829
chr3	40498662	40498995	334	0.72	0.00516	0.0263	RPL14	40493782
chr3	40498662	40498995	334	0.72	0.00516	0.0263	ENTPD3-AS	40492799
chr12	65002757	65002964	208	-1.06	0.00518	0.0264	RASSF3	64999292
chr12	65002757	65002964	208	-1.06	0.00518	0.0264	RASSF3	64999292
chr1	1.58E+08	1.58E+08	201	0.69	0.0052	0.0265	FCRL5	1.58E+08
chr1	1.58E+08	1.58E+08	201	0.69	0.0052	0.0265	FCRL5	1.58E+08
chr1	1.1E+08	1.1E+08	438	0.81	0.0052	0.0265	GNAT2	1.1E+08
chr17	33760140	33760947	808	0.41	0.00521	0.0265	SLFN12	33757543
chr17	33760140	33760947	808	0.41	0.00521	0.0265	SLFN12	33758195
chr7	93346590	93346802	213	-0.86	0.00523	0.0265	MIR4652	93341239
chr11	10476499	10476726	228	-0.73	0.00524	0.0266	AMPD3	10471665
chr11	10476499	10476726	228	-0.73	0.00524	0.0266	AMPD3	10472480
chr10	1.12E+08	1.12E+08	259	-0.63	0.00526	0.0267	MXI1	1.12E+08
chr8	79428228	79428786	559	0.46	0.00527	0.0267	PKIA	79423335
chr8	79428228	79428786	559	0.46	0.00527	0.0267	PKIA	79423335
chr18	2570723	2571686	964	0.57	0.00529	0.0268	NDC80	2566509
chr18	2570723	2571686	964	0.57	0.00529	0.0268	METTL4	2569502
chr18	2570723	2571686	964	0.57	0.00529	0.0268	METTL4	2569502
chr14	75469205	75469709	505	0.41	0.00531	0.0269	EIF2B2	75464611
chr1	1.53E+08	1.53E+08	410	0.66	0.00532	0.0269	S100A12	1.53E+08
chr6	1.71E+08	1.71E+08	726	-0.54	0.00533	0.0269	PDCD2	1.71E+08
chr6	1.71E+08	1.71E+08	726	-0.54	0.00533	0.0269	PDCD2	1.71E+08
chr6	1.71E+08	1.71E+08	726	-0.54	0.00533	0.0269	PDCD2	1.71E+08
chr6	1.71E+08	1.71E+08	726	-0.54	0.00533	0.0269	PDCD2	1.71E+08
chr6	1.71E+08	1.71E+08	726	-0.54	0.00533	0.0269	PDCD2	1.71E+08
chr6	1.71E+08	1.71E+08	726	-0.54	0.00533	0.0269	PDCD2	1.71E+08
chr17	18849845	18850222	378	1.05	0.00534	0.027	SLC5A10	18848988
chr11	1.02E+08	1.02E+08	182	0.44	0.00535	0.027	TMEM123	1.02E+08
chr15	99190558	99190901	344	-0.72	0.00538	0.0271	IGF1R	99186767
chr15	99190558	99190901	344	-0.72	0.00538	0.0271	IGF1R	99186767
chr11	65188728	65188942	215	0.7	0.0054	0.0272	NEAT1	65185268
chr11	65188728	65188942	215	0.7	0.0054	0.0272	NEAT1	65185268
chr5	72140347	72140543	197	0.84	0.0054	0.0272	TNPO1	72138929
chr2	75750645	75751082	438	0.52	0.00541	0.0272	LOC101927	75746193
chr2	1.01E+08	1.01E+08	453	0.71	0.00542	0.0273	CHST10	1.01E+08
chr7	21581590	21581788	199	0.81	0.00542	0.0273	DNAH11	21577832
chr13	21476781	21477289	509	0.5	0.00542	0.0273	XPO4	21474913
chr3	96532732	96533158	427	0.59	0.00542	0.0273	EPHA6	96528424
chr3	96532732	96533158	427	0.59	0.00542	0.0273	EPHA6	96528424
chr10	70092531	70092785	255	0.92	0.00546	0.0274	PBLD	70090684
chr10	70092531	70092785	255	0.92	0.00546	0.0274	HNRNPH3	70086767
chr10	70092531	70092785	255	0.92	0.00546	0.0274	PBLD	70090684
chr10	70092531	70092785	255	0.92	0.00546	0.0274	HNRNPH3	70086767
chr3	97978686	97978849	164	-1.65	0.00546	0.0274	OR5H6	97978128
chr20	14529950	14530149	200	0.66	0.00549	0.0275	MACROD2-	14530029
chr7	1.55E+08	1.55E+08	973	-0.45	0.00553	0.0277	PAXIP1-AS:	1.55E+08

chr7	1.55E+08	1.55E+08	973	-0.45	0.00553	0.0277	PAXIP1	1.55E+08
chr2	2.04E+08	2.04E+08	660	0.57	0.00553	0.0277	ABI2	2.04E+08
chr2	2.04E+08	2.04E+08	660	0.57	0.00553	0.0277	ABI2	2.04E+08
chr2	2.04E+08	2.04E+08	660	0.57	0.00553	0.0277	ABI2	2.04E+08
chr2	2.04E+08	2.04E+08	660	0.57	0.00553	0.0277	ABI2	2.04E+08
chr1	91486871	91488022	1152	0.31	0.00554	0.0277	ZNF644	91485812
chr1	91486871	91488022	1152	0.31	0.00554	0.0277	ZNF644	91485065
chr1	91486871	91488022	1152	0.31	0.00554	0.0277	ZNF644	91485812
chr18	6729494	6730041	548	0.74	0.00554	0.0277	LOC101927	6727861
chr1	1.7E+08	1.7E+08	459	-0.8	0.00554	0.0277	SELL	1.7E+08
chr1	1.7E+08	1.7E+08	459	-0.8	0.00554	0.0277	SELL	1.7E+08
chr4	88740429	88740691	263	0.86	0.00554	0.0277	MEPE	88737549
chr2	1.93E+08	1.93E+08	357	1.04	0.00554	0.0277	SDPR	1.93E+08
chr1	74663485	74664174	690	0.33	0.00556	0.0278	LRRIQ3	74661871
chr1	74663485	74664174	690	0.33	0.00556	0.0278	FPGT-TNNI	74658895
chr1	74663485	74664174	690	0.33	0.00556	0.0278	FPGT-TNNI	74658895
chr1	74663485	74664174	690	0.33	0.00556	0.0278	FPGT	74658895
chr1	74663485	74664174	690	0.33	0.00556	0.0278	FPGT	74658895
chr1	74663485	74664174	690	0.33	0.00556	0.0278	FPGT	74658895
chr11	12308630	12308896	267	0.69	0.00557	0.0278	MICALCL	12303446
chr17	78193231	78193513	283	0.86	0.00558	0.0279	SLC26A11	78189199
chr17	78193231	78193513	283	0.86	0.00558	0.0279	SLC26A11	78189199
chr17	78193231	78193513	283	0.86	0.00558	0.0279	SGSH	78192199
chr17	78193231	78193513	283	0.86	0.00558	0.0279	SLC26A11	78189199
chr17	78193231	78193513	283	0.86	0.00558	0.0279	SLC26A11	78189199
chr12	30944865	30945064	200	0.9	0.0056	0.0279	LINC00941	30943614
chr17	33418146	33418346	201	0.79	0.0056	0.0279	RFFL	33414348
chr2	1.79E+08	1.79E+08	210	0.74	0.00565	0.0281	PLEKHA3	1.79E+08
chr2	1.79E+08	1.79E+08	210	0.74	0.00565	0.0281	FKBP7	1.79E+08
chr2	1.79E+08	1.79E+08	210	0.74	0.00565	0.0281	FKBP7	1.79E+08
chr3	1.56E+08	1.56E+08	775	0.28	0.00566	0.0281	TIPARP	1.56E+08
chr3	1.56E+08	1.56E+08	775	0.28	0.00566	0.0281	TIPARP	1.56E+08
chr3	1.56E+08	1.56E+08	775	0.28	0.00566	0.0281	TIPARP	1.56E+08
chr3	1.56E+08	1.56E+08	775	0.28	0.00566	0.0281	TIPARP-AS1	1.56E+08
chr12	52585687	52586247	561	0.48	0.00572	0.0284	KRT80	52583784
chr12	52585687	52586247	561	0.48	0.00572	0.0284	KRT80	52583784
chr11	16759596	16760525	930	0.37	0.00574	0.0285	C11orf58	16755147
chr12	24100804	24101004	201	0.9	0.00576	0.0286	SOX5	24100637
chr6	2885703	2885903	201	0.75	0.00577	0.0286	LOC101927	2879221
chr15	55698425	55698614	190	1.09	0.00582	0.0288	C15orf65	55695722
chr15	55698425	55698614	190	1.09	0.00582	0.0288	CCPG1	55698574
chr13	20013250	20013491	242	-1.88	0.00582	0.0288	TPTE2	20010615
chr10	44340695	44340969	275	-0.91	0.00584	0.0289	LINC00619	44335753
chr13	44682632	44682858	227	-1.3	0.00587	0.029	SMIM2-AS1	44679684
chr2	2.28E+08	2.28E+08	318	-0.67	0.00589	0.029	MIR5702	2.28E+08
chr10	31323560	31323743	184	0.81	0.00591	0.0291	ZNF438	31318866
chr10	31323560	31323743	184	0.81	0.00591	0.0291	ZNF438	31318866

chr10	31323560	31323743	184	0.81	0.00591	0.0291	ZNF438	31318866
chr10	31323560	31323743	184	0.81	0.00591	0.0291	ZNF438	31318866
chr10	31323560	31323743	184	0.81	0.00591	0.0291	ZNF438	31318866
chr10	31323560	31323743	184	0.81	0.00591	0.0291	ZNF438	31318866
chr10	31323560	31323743	184	0.81	0.00591	0.0291	ZNF438	31318866
chr7	1.05E+08	1.05E+08	190	1.24	0.00591	0.0291	SRPK2	1.05E+08
chr11	87912331	87912740	410	0.48	0.00592	0.0291	RAB38	87906635
chr19	18549316	18549775	460	0.69	0.00593	0.0292	ISYNA1	18547111
chr19	18549316	18549775	460	0.69	0.00593	0.0292	ISYNA1	18547111
chr19	18549316	18549775	460	0.69	0.00593	0.0292	ISYNA1	18547111
chr19	18549316	18549775	460	0.69	0.00593	0.0292	ISYNA1	18546119
chr19	18549316	18549775	460	0.69	0.00593	0.0292	ISYNA1	18546972
chr1	1.51E+08	1.51E+08	199	0.63	0.00595	0.0293	SEMA6C	1.51E+08
chr1	1.51E+08	1.51E+08	199	0.63	0.00595	0.0293	SEMA6C	1.51E+08
chr1	1.51E+08	1.51E+08	199	0.63	0.00595	0.0293	SEMA6C	1.51E+08
chr16	66964207	66964366	160	-0.79	0.00596	0.0293	CES2	66963346
chr16	66964207	66964366	160	-0.79	0.00596	0.0293	CES2	66963346
chr16	66964207	66964366	160	-0.79	0.00596	0.0293	CES2	66963373
chr16	66964207	66964366	160	-0.79	0.00596	0.0293	RRAD	66957439
chr16	66964207	66964366	160	-0.79	0.00596	0.0293	RRAD	66957439
chr1	1.79E+08	1.79E+08	605	0.4	0.00596	0.0293	SOAT1	1.79E+08
chr1	1.79E+08	1.79E+08	605	0.4	0.00596	0.0293	SOAT1	1.79E+08
chr1	1.79E+08	1.79E+08	605	0.4	0.00596	0.0293	SOAT1	1.79E+08
chr1	1.79E+08	1.79E+08	605	0.4	0.00596	0.0293	SOAT1	1.79E+08
chr6	7389598	7390512	915	-0.37	0.00596	0.0293	CAGE1	7387942
chr6	7389598	7390512	915	-0.37	0.00596	0.0293	CAGE1	7387942
chr6	7389598	7390512	915	-0.37	0.00596	0.0293	CAGE1	7387942
chr6	7389598	7390512	915	-0.37	0.00596	0.0293	RIOK1	7385061
chr10	1.02E+08	1.02E+08	722	0.49	0.00597	0.0294	DNMBP	1.02E+08
chr17	8268455	8268724	270	1.12	0.00603	0.0296	LOC100128	8261859
chr8	41516579	41516857	279	0.91	0.00605	0.0297	MIR486-1	41516026
chr8	41516579	41516857	279	0.91	0.00605	0.0297	MIR486-2	41512961
chr2	1.02E+08	1.02E+08	612	0.68	0.00609	0.0298	CREG2	1.02E+08
chr19	44289499	44289645	147	-0.97	0.0061	0.0299	KCNN4	44283409
chr20	32307945	32308385	441	0.56	0.00611	0.0299	PXMP4	32306136
chr20	32307945	32308385	441	0.56	0.00611	0.0299	PXMP4	32306136
chr10	93683259	93683865	607	-0.6	0.00615	0.03	BTAF1	93678735
chr1	1.08E+08	1.08E+08	1013	0.41	0.00615	0.0301	PRMT6	1.08E+08
chr13	1.15E+08	1.15E+08	980	0.6	0.00616	0.0301	GAS6-AS1	1.15E+08
chr10	6822586	6822785	200	0.78	0.00617	0.0301	LINC00707	6816559
chr10	6822586	6822785	200	0.78	0.00617	0.0301	LINC00706	6819142
chr3	1.22E+08	1.22E+08	398	0.75	0.00618	0.0302	CSTA	1.22E+08
chr2	1.68E+08	1.68E+08	319	0.49	0.00619	0.0302	XIRP2-AS1	1.68E+08
chr8	1.28E+08	1.28E+08	204	1.13	0.0062	0.0302	CASC19	1.28E+08
chr7	1.03E+08	1.03E+08	760	0.47	0.0062	0.0302	NAPEPLD	1.03E+08
chr7	1.03E+08	1.03E+08	760	0.47	0.0062	0.0302	NAPEPLD	1.03E+08
chr10	64576107	64576645	539	-0.4	0.0062	0.0302	EGR2	64574126

chr10	64576107	64576645	539	-0.4	0.0062	0.0302	EGR2	64574126
chr10	64576107	64576645	539	-0.4	0.0062	0.0302	EGR2	64574126
chr3	1.31E+08	1.31E+08	547	0.38	0.0062	0.0302	MRPL3	1.31E+08
chr10	1.03E+08	1.03E+08	239	0.78	0.00623	0.0303	SFXN3	1.03E+08
chr10	1.03E+08	1.03E+08	239	0.78	0.00623	0.0303	PDZD7	1.03E+08
chr10	1.03E+08	1.03E+08	239	0.78	0.00623	0.0303	PDZD7	1.03E+08
chr15	45814604	45815282	679	0.5	0.00624	0.0304	SLC30A4	45813002
chrX	46184810	46185263	454	0.88	0.00625	0.0304	LINC01186	46185109
chr10	28966168	28966600	433	-0.58	0.00628	0.0305	BAMBI	28961423
chr8	1.45E+08	1.45E+08	604	-0.49	0.00629	0.0305	GSDMD	1.45E+08
chr8	1.45E+08	1.45E+08	604	-0.49	0.00629	0.0305	GSDMD	1.45E+08
chr20	43733584	43733797	214	1.41	0.0063	0.0306	KCNS1	43727753
chr16	72206241	72206459	219	-1.03	0.0063	0.0306	PMFBP1	72204349
chr16	72206241	72206459	219	-1.03	0.0063	0.0306	PMFBP1	72204064
chr2	70528549	70529321	773	0.59	0.0063	0.0306	FAM136A	70527220
chr7	95111174	95111357	184	1.28	0.00632	0.0307	ASB4	95110212
chr7	95111174	95111357	184	1.28	0.00632	0.0307	ASB4	95110212
chr13	42708315	42708864	550	0.87	0.00632	0.0307	DGKH	42707177
chr13	42708315	42708864	550	0.87	0.00632	0.0307	DGKH	42707177
chr13	42708315	42708864	550	0.87	0.00632	0.0307	DGKH	42707177
chr13	42708315	42708864	550	0.87	0.00632	0.0307	DGKH	42707177
chr13	42708315	42708864	550	0.87	0.00632	0.0307	DGKH	42707177
chr12	1.05E+08	1.05E+08	256	0.82	0.00633	0.0307	MIR7641-2	1.05E+08
chr11	17756610	17756743	134	1.04	0.00636	0.0308	KCNC1	17752494
chr11	17756610	17756743	134	1.04	0.00636	0.0308	KCNC1	17752494
chr6	28351044	28351601	558	0.34	0.00636	0.0308	ZSCAN12	28349216
chr6	28351044	28351601	558	0.34	0.00636	0.0308	ZSCAN12	28349216
chr1	51983790	51983963	174	-0.94	0.00638	0.0309	EPS15	51982995
chr8	1.44E+08	1.44E+08	200	0.92	0.00641	0.031	LYNX1	1.44E+08
chr8	1.44E+08	1.44E+08	200	0.92	0.00641	0.031	LYNX1	1.44E+08
chr8	1.44E+08	1.44E+08	200	0.92	0.00641	0.031	LYNX1	1.44E+08
chr8	1.44E+08	1.44E+08	200	0.92	0.00641	0.031	LYNX1	1.44E+08
chr9	1.4E+08	1.4E+08	130	-1.17	0.00641	0.031	ENTPD8	1.4E+08
chr9	1.4E+08	1.4E+08	130	-1.17	0.00641	0.031	ENTPD8	1.4E+08
chr6	1.05E+08	1.05E+08	199	0.96	0.00642	0.031	LINC00577	1.05E+08
chr12	49212124	49212416	293	0.82	0.00643	0.0311	CACNB3	49207253
chr12	49212124	49212416	293	0.82	0.00643	0.0311	CACNB3	49207253
chr3	1.89E+08	1.89E+08	219	-0.63	0.00645	0.0311	TPRG1-AS1	1.89E+08
chr1	98675688	98675900	213	0.92	0.00652	0.0314	LOC729987	98671266
chr8	80941815	80942725	911	0.33	0.00653	0.0314	MRPS28	80940506
chr6	86114485	86114696	212	-0.86	0.00654	0.0315	LOC101928	86112520
chr2	11672659	11672827	169	1.15	0.00655	0.0315	GREB1	11669241
chr2	29117258	29117689	432	0.51	0.00657	0.0316	WDR43	29112508
chr20	42189212	42189412	201	0.73	0.0066	0.0317	SGK2	42188754
chr20	42189212	42189412	201	0.73	0.0066	0.0317	SGK2	42182634
chr10	27541317	27541870	554	0.54	0.0067	0.0321	LRRC37A6F	27539235
chr19	34286327	34287312	986	0.45	0.0067	0.0321	KCTD15	34282750

chr19	34286327	34287312	986	0.45	0.0067	0.0321	KCTD15	34282750
chr19	34286327	34287312	986	0.45	0.0067	0.0321	KCTD15	34282750
chr11	1.25E+08	1.25E+08	541	0.63	0.00671	0.0321	ESAM	1.25E+08
chr11	1.25E+08	1.25E+08	541	0.63	0.00671	0.0321	LOC10192[illegible]	1.25E+08
chr1	1.2E+08	1.2E+08	200	0.8	0.00673	0.0322	HSD3B2	1.2E+08
chr1	1.2E+08	1.2E+08	200	0.8	0.00673	0.0322	HSD3B2	1.2E+08
chr20	58569824	58570048	225	-0.77	0.00676	0.0323	CDH26	58566416
chr6	35177762	35177898	137	1.71	0.00676	0.0323	SCUBE3	35176838
chr6	35177762	35177898	137	1.71	0.00676	0.0323	SCUBE3	35176838
chr7	56173953	56174536	584	0.3	0.00676	0.0323	CHCHD2	56172187
chr1	46215960	46216673	714	0.41	0.00677	0.0323	IPP	46214485
chr1	46215960	46216673	714	0.41	0.00677	0.0323	IPP	46214485
chr12	21654186	21654937	752	0.34	0.00678	0.0323	RECQL	21652603
chr12	21654186	21654937	752	0.34	0.00678	0.0323	RECQL	21652603
chr12	21654186	21654937	752	0.34	0.00678	0.0323	GOLT1B	21649698
chr19	7456082	7456568	487	0.54	0.00678	0.0323	ARHGEF18	7454998
chr10	6019259	6020140	882	-0.49	0.00679	0.0324	IL15RA	6017574
chr10	6019259	6020140	882	-0.49	0.00679	0.0324	IL15RA	6017574
chr10	6019259	6020140	882	-0.49	0.00679	0.0324	IL15RA	6017574
chr10	6019259	6020140	882	-0.49	0.00679	0.0324	IL15RA	6018150
chr10	6019259	6020140	882	-0.49	0.00679	0.0324	IL15RA	6018150
chr7	21984324	21984495	172	-0.72	0.00681	0.0325	CDCA7L	21983542
chr7	21984324	21984495	172	-0.72	0.00681	0.0325	CDCA7L	21983542
chr7	21984324	21984495	172	-0.72	0.00681	0.0325	CDCA7L	21983542
chr4	1.86E+08	1.86E+08	454	0.76	0.00682	0.0325	MIR3945H[illegible]	1.86E+08
chr1	70819396	70819657	262	0.69	0.00685	0.0326	ANKRD13C	70818417
chr1	70819396	70819657	262	0.69	0.00685	0.0326	HHLA3	70815492
chr1	70819396	70819657	262	0.69	0.00685	0.0326	HHLA3	70815492
chr1	70819396	70819657	262	0.69	0.00685	0.0326	HHLA3	70815492
chr1	70819396	70819657	262	0.69	0.00685	0.0326	HHLA3	70815492
chr19	34972401	34972848	448	0.61	0.00686	0.0326	WTIP	34967879
chr1	2.01E+08	2.01E+08	391	0.61	0.00691	0.0328	PHLDA3	2.01E+08
chr1	2.01E+08	2.01E+08	391	0.61	0.00691	0.0328	PHLDA3	2.01E+08
chr13	39258034	39258234	201	0.49	0.00691	0.0328	FREM2	39256172
chr15	27111700	27111945	246	1.51	0.00694	0.0329	GABRA5	27107272
chr15	27111700	27111945	246	1.51	0.00694	0.0329	GABRA5	27106865
chr1	63832885	63833712	828	0.31	0.00695	0.0329	ALG6	63828260
chr2	2.3E+08	2.3E+08	188	0.83	0.00695	0.0329	PID1	2.3E+08
chr2	2.3E+08	2.3E+08	188	0.83	0.00695	0.0329	PID1	2.3E+08
chr19	55838961	55839197	237	-0.97	0.00699	0.0331	TMEM150[illegible]	55834708
chr19	55838961	55839197	237	-0.97	0.00699	0.0331	TMEM150[illegible]	55834708
chr19	55838961	55839197	237	-0.97	0.00699	0.0331	TMEM150[illegible]	55834708
chr19	55838961	55839197	237	-0.97	0.00699	0.0331	TMEM150[illegible]	55834708
chr2	1.98E+08	1.98E+08	656	0.36	0.007	0.0331	PGAP1	1.98E+08
chr17	20978777	20979013	237	1.61	0.007	0.0331	LINC01563	20973868
chr17	20978777	20979013	237	1.61	0.007	0.0331	LINC01563	20973868
chr7	20369074	20370668	1595	0.26	0.00701	0.0332	ITGB8	20365724

chr7	20369074	20370668	1595	0.26	0.00701	0.0332	LOC101927	20369385
chr7	20369074	20370668	1595	0.26	0.00701	0.0332	LOC101927	20369385
chr12	62588435	62588775	341	0.65	0.00704	0.0333	FAM19A2	62584620
chr12	1.03E+08	1.03E+08	201	0.82	0.00704	0.0333	PAH	1.03E+08
chr4	89377820	89378649	830	-0.66	0.00707	0.0334	HERC5	89373267
chr6	89853265	89853465	201	1.67	0.00708	0.0334	PM20D2	89850768
chr9	3524309	3524505	197	0.76	0.00717	0.0337	RFX3	3524001
chr9	3524309	3524505	197	0.76	0.00717	0.0337	RFX3	3524001
chr7	66206314	66206590	277	0.91	0.00719	0.0338	RABGEF1	66200642
chr7	66206314	66206590	277	0.91	0.00719	0.0338	RABGEF1	66200642
chr19	44668895	44669427	533	0.51	0.00721	0.0339	ZNF226	44664248
chr19	44668895	44669427	533	0.51	0.00721	0.0339	ZNF226	44664214
chr19	44668895	44669427	533	0.51	0.00721	0.0339	ZNF226	44664248
chr19	44668895	44669427	533	0.51	0.00721	0.0339	ZNF226	44664316
chr19	44668895	44669427	533	0.51	0.00721	0.0339	ZNF226	44664248
chr19	44668895	44669427	533	0.51	0.00721	0.0339	ZNF226	44664214
chr19	44668895	44669427	533	0.51	0.00721	0.0339	ZNF226	44664214
chr19	44668895	44669427	533	0.51	0.00721	0.0339	ZNF226	44664214
chr19	44668895	44669427	533	0.51	0.00721	0.0339	ZNF226	44664307
chr12	1.09E+08	1.09E+08	499	0.73	0.00722	0.0339	WSCD2	1.09E+08
chr12	1.09E+08	1.09E+08	499	0.73	0.00722	0.0339	WSCD2	1.09E+08
chr2	2.2E+08	2.2E+08	853	0.56	0.00722	0.034	OBSL1	2.2E+08
chr2	2.2E+08	2.2E+08	853	0.56	0.00722	0.034	INHA	2.2E+08
chr2	2.2E+08	2.2E+08	853	0.56	0.00722	0.034	OBSL1	2.2E+08
chr2	2.2E+08	2.2E+08	853	0.56	0.00722	0.034	OBSL1	2.2E+08
chr3	1.01E+08	1.01E+08	328	-0.73	0.00724	0.034	FAM172BP	1.01E+08
chr1	1.6E+08	1.6E+08	209	0.54	0.00724	0.034	TAGLN2	1.6E+08
chr1	1.6E+08	1.6E+08	209	0.54	0.00724	0.034	TAGLN2	1.6E+08
chr4	57301587	57302572	986	0.34	0.00724	0.034	PPAT	57299802
chr4	57301587	57302572	986	0.34	0.00724	0.034	PAICS	57297268
chr4	57301587	57302572	986	0.34	0.00724	0.034	PAICS	57297268
chr4	57301587	57302572	986	0.34	0.00724	0.034	PAICS	57296914
chr12	33054440	33054688	249	0.65	0.00725	0.034	PKP2	33047780
chr12	33054440	33054688	249	0.65	0.00725	0.034	PKP2	33047780
chr22	29427388	29427614	227	1.35	0.00727	0.0341	ZNRF3-AS1	29425464
chr16	24855908	24856135	228	-1.01	0.00729	0.0342	SLC5A11	24852183
chr16	24855908	24856135	228	-1.01	0.00729	0.0342	SLC5A11	24852183
chr16	24855908	24856135	228	-1.01	0.00729	0.0342	SLC5A11	24852183
chr16	24855908	24856135	228	-1.01	0.00729	0.0342	SLC5A11	24852183
chr16	24855908	24856135	228	-1.01	0.00729	0.0342	SLC5A11	24852551
chr4	1.13E+08	1.13E+08	968	0.49	0.0073	0.0342	C4orf32	1.13E+08
chr10	21822542	21823341	800	0.39	0.00733	0.0343	MLLT10	21818100
chr10	21822542	21823341	800	0.39	0.00733	0.0343	MLLT10	21818573
chr10	21822542	21823341	800	0.39	0.00733	0.0343	MLLT10	21818100
chr10	21822542	21823341	800	0.39	0.00733	0.0343	MLLT10	21818100
chr10	21822542	21823341	800	0.39	0.00733	0.0343	MLLT10	21818100
chr10	63806362	63806578	217	0.77	0.00733	0.0343	ARID5B	63803969

chr3	1.97E+08	1.97E+08	197	1.04	0.00737	0.0345	DLG1	1.97E+08
chr3	1.97E+08	1.97E+08	197	1.04	0.00737	0.0345	DLG1	1.97E+08
chr1	1.61E+08	1.61E+08	231	-0.67	0.00738	0.0345	NIT1	1.61E+08
chr1	1.61E+08	1.61E+08	231	-0.67	0.00738	0.0345	NIT1	1.61E+08
chr1	1.61E+08	1.61E+08	231	-0.67	0.00738	0.0345	NIT1	1.61E+08
chr1	1.61E+08	1.61E+08	231	-0.67	0.00738	0.0345	NIT1	1.61E+08
chr12	1.21E+08	1.21E+08	585	-0.6	0.00739	0.0345	GCN1	1.21E+08
chr9	1.03E+08	1.03E+08	977	0.31	0.0074	0.0345	LOC101928	1.03E+08
chr9	1.03E+08	1.03E+08	977	0.31	0.0074	0.0345	NR4A3	1.03E+08
chr9	1.03E+08	1.03E+08	977	0.31	0.0074	0.0345	NR4A3	1.03E+08
chr9	1.03E+08	1.03E+08	977	0.31	0.0074	0.0345	NR4A3	1.03E+08
chr3	44521837	44522037	201	0.83	0.00742	0.0346	ZNF445	44517162
chr1	1.61E+08	1.61E+08	780	0.6	0.00748	0.0349	B4GALT3	1.61E+08
chr1	1.61E+08	1.61E+08	780	0.6	0.00748	0.0349	B4GALT3	1.61E+08
chr1	1.61E+08	1.61E+08	780	0.6	0.00748	0.0349	B4GALT3	1.61E+08
chr2	47167439	47168439	1001	0.43	0.00757	0.0351	TTC7A	47163312
chr2	47167439	47168439	1001	0.43	0.00757	0.0351	TTC7A	47163312
chr2	47167439	47168439	1001	0.43	0.00757	0.0351	TTC7A	47163312
chr2	47167439	47168439	1001	0.43	0.00757	0.0351	MCFD2	47166994
chr2	47167439	47168439	1001	0.43	0.00757	0.0351	MCFD2	47166994
chr14	74106394	74106650	257	0.96	0.00759	0.0352	DNAL1	74106577
chr14	74106394	74106650	257	0.96	0.00759	0.0352	DNAL1	74106577
chr14	68140834	68141888	1055	0.45	0.00761	0.0353	VTI1B	68139602
chr1	2.37E+08	2.37E+08	568	0.61	0.00762	0.0353	EDARADD	2.37E+08
chr1	2.37E+08	2.37E+08	568	0.61	0.00762	0.0353	EDARADD	2.37E+08
chr7	25018686	25019345	660	0.44	0.00764	0.0354	OSBPL3	25017831
chr12	13043482	13044597	1116	0.35	0.00767	0.0355	GPRC5A	13038955
chr6	26206228	26206415	188	0.96	0.00767	0.0355	HIST1H4E	26199872
chr10	27443999	27444315	317	0.49	0.00769	0.0356	MASTL	27438752
chr10	27443999	27444315	317	0.49	0.00769	0.0356	MASTL	27438752
chr10	27443999	27444315	317	0.49	0.00769	0.0356	YME1L1	27441349
chr10	27443999	27444315	317	0.49	0.00769	0.0356	MASTL	27438752
chr10	27443999	27444315	317	0.49	0.00769	0.0356	YME1L1	27441349
chr10	27443999	27444315	317	0.49	0.00769	0.0356	YME1L1	27441349
chr9	79520993	79521439	447	0.49	0.00771	0.0356	PRUNE2	79519003
chr9	79520993	79521439	447	0.49	0.00771	0.0356	PRUNE2	79519003
chr9	79520993	79521439	447	0.49	0.00771	0.0356	PRUNE2	79519003
chr9	79520993	79521439	447	0.49	0.00771	0.0356	PRUNE2	79519003
chr1	2.49E+08	2.49E+08	426	0.41	0.00772	0.0357	ZNF692	2.49E+08
chr1	2.49E+08	2.49E+08	426	0.41	0.00772	0.0357	ZNF692	2.49E+08
chr1	2.49E+08	2.49E+08	426	0.41	0.00772	0.0357	ZNF692	2.49E+08
chr11	3695485	3695747	263	0.96	0.00776	0.0358	CHRNA10	3690614
chr11	3695485	3695747	263	0.96	0.00776	0.0358	CHRNA10	3690614
chr11	3695485	3695747	263	0.96	0.00776	0.0358	CHRNA10	3690614
chr5	1.39E+08	1.39E+08	361	0.82	0.00776	0.0358	LOC101929	1.39E+08
chr5	1.39E+08	1.39E+08	361	0.82	0.00776	0.0358	LOC101929	1.39E+08
chr11	67122163	67122343	181	-0.88	0.00779	0.0359	POLD4	67119067

chr11	67122163	67122343	181	-0.88	0.00779	0.0359	POLD4	67119067
chr11	67122163	67122343	181	-0.88	0.00779	0.0359	POLD4	67119067
chr11	67122163	67122343	181	-0.88	0.00779	0.0359	POLD4	67119067
chr11	67122163	67122343	181	-0.88	0.00779	0.0359	POLD4	67119067
chr19	49057056	49058047	992	0.49	0.00783	0.036	SULT2B1	49050428
chr16	66995043	66995156	114	-1.53	0.00783	0.036	CES3	66990131
chr16	66995043	66995156	114	-1.53	0.00783	0.036	CES3	66990131
chr16	58059378	58059704	327	-0.63	0.00785	0.0361	MMP15	58054469
chr5	54529408	54529672	265	-0.64	0.00787	0.0362	CCNO	54527545
chr5	54529408	54529672	265	-0.64	0.00787	0.0362	CCNO	54527545
chr5	54529408	54529672	265	-0.64	0.00787	0.0362	CCNO	54526819
chr5	54529408	54529672	265	-0.64	0.00787	0.0362	CCNO	54527545
chr1	1.77E+08	1.77E+08	295	-0.92	0.00789	0.0362	LOC102724	1.77E+08
chr11	1.25E+08	1.25E+08	164	-0.99	0.00791	0.0363	ROBO4	1.25E+08
chr11	1.25E+08	1.25E+08	164	-0.99	0.00791	0.0363	ROBO4	1.25E+08
chr14	67653965	67654251	287	-0.76	0.00791	0.0363	FAM71D	67651109
chr1	59616685	59617102	418	0.51	0.00793	0.0364	HSD52	59610479
chr1	53019100	53019514	415	0.66	0.00794	0.0364	ZCCHC11	53016762
chr1	53019100	53019514	415	0.66	0.00794	0.0364	ZCCHC11	53016762
chr9	92788169	92788443	275	0.94	0.00795	0.0365	MIR4290	92783817
chr14	1.06E+08	1.06E+08	371	0.59	0.00797	0.0365	LOC10272З	1.06E+08
chr19	45279617	45280198	582	-0.54	0.00802	0.0367	CBLC	45276125
chr19	45279617	45280198	582	-0.54	0.00802	0.0367	CBLC	45276125
chr2	2.03E+08	2.03E+08	199	0.92	0.00805	0.0368	ALS2	2.03E+08
chr2	2.03E+08	2.03E+08	199	0.92	0.00805	0.0368	ALS2	2.03E+08
chr5	1.5E+08	1.5E+08	407	0.66	0.00809	0.0369	SYNPO	1.5E+08
chr5	1.5E+08	1.5E+08	407	0.66	0.00809	0.0369	SYNPO	1.5E+08
chr4	88141345	88142001	657	0.6	0.00809	0.0369	KLHL8	88139760
chr4	88141345	88142001	657	0.6	0.00809	0.0369	KLHL8	88139760
chr4	88141345	88142001	657	0.6	0.00809	0.0369	KLHL8	88139420
chr4	88141345	88142001	657	0.6	0.00809	0.0369	KLHL8	88139760
chr4	88141345	88142001	657	0.6	0.00809	0.0369	KLHL8	88139760
chr2	1.83E+08	1.83E+08	146	1.12	0.00816	0.0372	PPP1R1C	1.83E+08
chr17	7740174	7740319	146	-0.77	0.00818	0.0373	KDM6B	7738234
chr6	1.25E+08	1.25E+08	464	0.55	0.00819	0.0373	RNF217	1.25E+08
chr6	12290224	12290909	686	0.37	0.00821	0.0374	EDN1	12285528
chr6	12290224	12290909	686	0.37	0.00821	0.0374	EDN1	12285528
chr10	49863562	49864624	1063	0.4	0.00826	0.0375	ARHGAP22	49858561
chr10	49863562	49864624	1063	0.4	0.00826	0.0375	ARHGAP22	49862310
chr6	1.1E+08	1.1E+08	605	0.64	0.00826	0.0375	MICAL1	1.1E+08
chr6	1.1E+08	1.1E+08	605	0.64	0.00826	0.0375	MICAL1	1.1E+08
chr12	9517507	9517815	309	-0.86	0.00829	0.0376	LOC10193(	9515059
chr11	72980836	72981080	245	0.85	0.00829	0.0376	P2RY6	72975938
chr11	72980836	72981080	245	0.85	0.00829	0.0376	P2RY6	72978186
chr11	72980836	72981080	245	0.85	0.00829	0.0376	P2RY6	72978186
chr10	1.02E+08	1.02E+08	1096	0.31	0.00839	0.038	OLMALINC	1.02E+08
chr1	1.16E+08	1.16E+08	201	0.72	0.00842	0.0381	TSHB	1.16E+08

chr19	2051216	2051431	216	-0.87	0.00845	0.0382	MKNK2	2049243
chr19	2051216	2051431	216	-0.87	0.00845	0.0382	MKNK2	2049243
chr1	2.12E+08	2.12E+08	730	-0.5	0.00848	0.0383	NEK2	2.12E+08
chr1	2.12E+08	2.12E+08	730	-0.5	0.00848	0.0383	NEK2	2.12E+08
chr1	2.12E+08	2.12E+08	730	-0.5	0.00848	0.0383	NEK2	2.12E+08
chr16	50730657	50730772	116	-1.17	0.00849	0.0383	NOD2	50726049
chr16	50730657	50730772	116	-1.17	0.00849	0.0383	NOD2	50728260
chr9	66553098	66554140	1043	2.27	0.0085	0.0384	LOC72867:	66548199
chr9	66553098	66554140	1043	2.27	0.0085	0.0384	LOC40332:	66551911
chr9	66553098	66554140	1043	2.27	0.0085	0.0384	LOC40332:	66551911
chr18	44679684	44679884	201	0.88	0.0085	0.0384	HDHD2	44674871
chr18	44679684	44679884	201	0.88	0.0085	0.0384	HDHD2	44674891
chr19	12889049	12889417	369	0.53	0.00851	0.0384	HOOK2	12884434
chr19	12889049	12889417	369	0.53	0.00851	0.0384	HOOK2	12884434
chr4	80993865	80994569	705	0.45	0.00852	0.0384	ANTXR2	80992477
chr4	80993865	80994569	705	0.45	0.00852	0.0384	ANTXR2	80992477
chr4	80993865	80994569	705	0.45	0.00852	0.0384	ANTXR2	80992626
chr4	80993865	80994569	705	0.45	0.00852	0.0384	ANTXR2	80992477
chr19	3481799	3481993	195	1.44	0.00853	0.0384	SMIM24	3478540
chr2	2.39E+08	2.39E+08	198	0.71	0.00853	0.0385	LINC01107	2.39E+08
chr4	96007805	96008071	267	0.83	0.00854	0.0385	BMPR1B	96007641
chr22	39493064	39493474	411	0.67	0.00856	0.0386	APOBEC3H	39488228
chr22	39493064	39493474	411	0.67	0.00856	0.0386	APOBEC3H	39488228
chr22	39493064	39493474	411	0.67	0.00856	0.0386	APOBEC3H	39488228
chr22	39493064	39493474	411	0.67	0.00856	0.0386	APOBEC3H	39488228
chr2	27850887	27851125	239	0.83	0.00857	0.0386	CCDC121	27849745
chr2	27850887	27851125	239	0.83	0.00857	0.0386	GPN1	27846514
chr2	27850887	27851125	239	0.83	0.00857	0.0386	GPN1	27846862
chr2	27850887	27851125	239	0.83	0.00857	0.0386	GPN1	27846514
chr2	27850887	27851125	239	0.83	0.00857	0.0386	GPN1	27846514
chr2	27850887	27851125	239	0.83	0.00857	0.0386	CCDC121	27849898
chr2	27850887	27851125	239	0.83	0.00857	0.0386	GPN1	27846862
chr22	18393398	18393892	495	0.86	0.00863	0.0388	MICAL3	18387652
chr22	18393398	18393892	495	0.86	0.00863	0.0388	MICAL3	18387652
chr2	2.02E+08	2.02E+08	753	0.46	0.00864	0.0388	LOC101927	2.02E+08
chr2	2.02E+08	2.02E+08	753	0.46	0.00864	0.0388	BZW1	2.02E+08
chr2	2.02E+08	2.02E+08	753	0.46	0.00864	0.0388	BZW1	2.02E+08
chr2	2.02E+08	2.02E+08	753	0.46	0.00864	0.0388	BZW1	2.02E+08
chr2	2.02E+08	2.02E+08	753	0.46	0.00864	0.0388	BZW1	2.02E+08
chr8	1.09E+08	1.09E+08	1166	0.26	0.00864	0.0388	EMC2	1.09E+08
chr13	78111618	78112036	419	0.46	0.00872	0.0391	SCEL	78104808
chr13	78111618	78112036	419	0.46	0.00872	0.0391	SCEL	78104808
chr13	78111618	78112036	419	0.46	0.00872	0.0391	SCEL	78104808
chr10	38644963	38645798	836	0.74	0.00872	0.0391	HSD17B7P:	38640307
chr2	47629956	47630675	720	0.46	0.00877	0.0392	MSH2	47625205
chr2	47629956	47630675	720	0.46	0.00877	0.0392	MSH2	47625205
chr20	36661658	36662294	637	-0.5	0.00878	0.0393	RPRD1B	36656947

chr20	36661658	36662294	637	-0.5	0.00878	0.0393	TTI1	36659870
chr20	36661658	36662294	637	-0.5	0.00878	0.0393	TTI1	36659870
chr6	4775731	4776772	1042	-0.45	0.00879	0.0393	CDYL	4771679
chr11	59577755	59578473	719	0.39	0.00881	0.0394	MRPL16	59576345
chr10	1.23E+08	1.23E+08	611	-0.38	0.00882	0.0394	WDR11-AS	1.23E+08
chr10	1.23E+08	1.23E+08	611	-0.38	0.00882	0.0394	WDR11	1.23E+08
chrX	99991691	99991890	200	0.69	0.00883	0.0394	SYTL4	99985135
chr16	53464007	53464597	591	0.61	0.00883	0.0395	RBL2	53463350
chr21	48052144	48052333	190	1.06	0.00884	0.0395	PRMT2	48050506
chr21	48052144	48052333	190	1.06	0.00884	0.0395	PRMT2	48050506
chr21	48052144	48052333	190	1.06	0.00884	0.0395	PRMT2	48050506
chr21	48052144	48052333	190	1.06	0.00884	0.0395	PRMT2	48050506
chr21	48052144	48052333	190	1.06	0.00884	0.0395	PRMT2	48050506
chr21	48052144	48052333	190	1.06	0.00884	0.0395	PRMT2	48050506
chr21	48052144	48052333	190	1.06	0.00884	0.0395	PRMT2	48050506
chr21	48052144	48052333	190	1.06	0.00884	0.0395	PRMT2	48050506
chr20	44146952	44147322	371	0.48	0.00885	0.0395	SPINT3	44142264
chr1	1.6E+08	1.6E+08	331	1.06	0.0089	0.0397	KCNJ10	1.6E+08
chr14	67707429	67708611	1183	0.28	0.0089	0.0397	MPP5	67703011
chr14	67707429	67708611	1183	0.28	0.0089	0.0397	MPP5	67703011
chr1	1.86E+08	1.86E+08	201	0.91	0.00893	0.0398	PRG4	1.86E+08
chr1	1.86E+08	1.86E+08	201	0.91	0.00893	0.0398	PRG4	1.86E+08
chr1	1.86E+08	1.86E+08	201	0.91	0.00893	0.0398	PRG4	1.86E+08
chr1	1.86E+08	1.86E+08	201	0.91	0.00893	0.0398	PRG4	1.86E+08
chr1	1.86E+08	1.86E+08	201	0.91	0.00893	0.0398	PRG4	1.86E+08
chr6	24803676	24803940	265	-0.72	0.00893	0.0398	C6orf229	24797124
chr7	96656978	96657216	239	0.88	0.009	0.04	DLX5	96652143
chr6	1.68E+08	1.68E+08	220	-1.02	0.00902	0.0401	LOC40128(	1.68E+08
chr6	1.68E+08	1.68E+08	220	-1.02	0.00902	0.0401	LOC44117{	1.68E+08
chr3	1.41E+08	1.41E+08	222	0.93	0.00905	0.0401	ZBTB38	1.41E+08
chr8	1.29E+08	1.29E+08	416	0.39	0.00905	0.0402	CASC11	1.29E+08
chr8	1.29E+08	1.29E+08	416	0.39	0.00905	0.0402	CASC11	1.29E+08
chr8	1.29E+08	1.29E+08	416	0.39	0.00905	0.0402	MYC	1.29E+08
chr3	4534000	4535220	1221	0.36	0.00905	0.0402	ITPR1	4530031
chr3	4534000	4535220	1221	0.36	0.00905	0.0402	ITPR1	4530031
chr3	4534000	4535220	1221	0.36	0.00905	0.0402	ITPR1	4530031
chr3	4534000	4535220	1221	0.36	0.00905	0.0402	ITPR1-AS1	4532847
chr11	8928249	8928475	227	0.8	0.00909	0.0403	AKIP1	8927700
chr11	8928249	8928475	227	0.8	0.00909	0.0403	AKIP1	8927700
chr11	8928249	8928475	227	0.8	0.00909	0.0403	AKIP1	8927700
chr11	8928249	8928475	227	0.8	0.00909	0.0403	AKIP1	8927700
chr11	8928249	8928475	227	0.8	0.00909	0.0403	AKIP1	8927738
chr11	8928249	8928475	227	0.8	0.00909	0.0403	AKIP1	8927738
chr13	21750320	21751167	848	0.42	0.00912	0.0404	SKA3	21748741
chr13	21750320	21751167	848	0.42	0.00912	0.0404	SKA3	21748741
chr13	21750320	21751167	848	0.42	0.00912	0.0404	MRPL57	21745371
chr7	86274935	86275132	198	0.88	0.00913	0.0404	GRM3	86268229

chr6	42847267	42847880	614	0.56	0.00914	0.0405	RPL7L1	42842353
chr6	42847267	42847880	614	0.56	0.00914	0.0405	RPL7L1	42842353
chr6	42847267	42847880	614	0.56	0.00914	0.0405	RPL7L1	42842353
chr12	75905226	75905694	469	0.55	0.00914	0.0405	KRR1	75903418
chr9	93563847	93564428	582	-0.6	0.00918	0.0406	SYK	93559208
chr9	93563847	93564428	582	-0.6	0.00918	0.0406	SYK	93558961
chr9	93563847	93564428	582	-0.6	0.00918	0.0406	SYK	93558961
chr16	86609403	86609972	570	-0.55	0.0092	0.0407	FOXL1	86607114
chr8	1.21E+08	1.21E+08	656	0.29	0.00923	0.0407	DEPTOR	1.21E+08
chr8	1.21E+08	1.21E+08	656	0.29	0.00923	0.0407	DEPTOR	1.21E+08
chr5	94890294	94891092	799	0.42	0.00926	0.0409	ARSK	94885824
chr5	94890294	94891092	799	0.42	0.00926	0.0409	TTC37	94888709
chrX	69271711	69271909	199	0.72	0.00928	0.0409	AWAT2	69267788
chr17	79844099	79844410	312	0.84	0.0093	0.041	ANAPC11	79843665
chr7	5085254	5085938	685	-0.44	0.00931	0.041	RBAK-RBAK	5080451
chr7	5085254	5085938	685	-0.44	0.00931	0.041	RBAK	5080451
chr7	5085254	5085938	685	-0.44	0.00931	0.041	RBAK	5080552
chr11	1.05E+08	1.05E+08	270	-1.13	0.00935	0.0411	CASP4	1.05E+08
chr6	1.53E+08	1.53E+08	683	0.28	0.00936	0.0412	MTRF1L	1.53E+08
chr6	1.53E+08	1.53E+08	683	0.28	0.00936	0.0412	MTRF1L	1.53E+08
chr6	1.53E+08	1.53E+08	683	0.28	0.00936	0.0412	MTRF1L	1.53E+08
chr6	1.53E+08	1.53E+08	683	0.28	0.00936	0.0412	MTRF1L	1.53E+08
chr6	1.53E+08	1.53E+08	683	0.28	0.00936	0.0412	MTRF1L	1.53E+08
chr6	1.53E+08	1.53E+08	683	0.28	0.00936	0.0412	MTRF1L	1.53E+08
chr6	1.53E+08	1.53E+08	683	0.28	0.00936	0.0412	MTRF1L	1.53E+08
chr2	1.06E+08	1.06E+08	807	0.48	0.00939	0.0413	NCK2	1.06E+08
chr2	1.06E+08	1.06E+08	807	0.48	0.00939	0.0413	NCK2	1.06E+08
chr10	1.06E+08	1.06E+08	560	-0.66	0.00947	0.0415	SH3PXD2A-	1.06E+08
chrX	24665363	24665649	287	0.78	0.0095	0.0416	PCYT1B	24663455
chrX	24665363	24665649	287	0.78	0.0095	0.0416	PCYT1B	24663455
chrX	24665363	24665649	287	0.78	0.0095	0.0416	PCYT1B-AS	24663189
chr8	93029812	93030012	201	1.08	0.00951	0.0417	RUNX1T1	93027908
chr15	35662379	35662577	199	0.86	0.00952	0.0417	MIR3942	35662565
chr6	97372067	97373035	969	-0.36	0.00954	0.0417	KLHL32	97367495
chr6	97372067	97373035	969	-0.36	0.00954	0.0417	KLHL32	97367495
chr6	97372067	97373035	969	-0.36	0.00954	0.0417	KLHL32	97367495
chr6	97372067	97373035	969	-0.36	0.00954	0.0417	KLHL32	97367495
chr6	97372067	97373035	969	-0.36	0.00954	0.0417	KLHL32	97367495
chr6	1.5E+08	1.5E+08	269	0.71	0.00954	0.0417	RAET1E-AS	1.5E+08
chr6	1.5E+08	1.5E+08	269	0.71	0.00954	0.0417	LRP11	1.5E+08
chr10	1.3E+08	1.3E+08	332	0.84	0.00965	0.0421	FOXI2	1.3E+08
chr14	50159771	50160265	495	0.65	0.00966	0.0421	KLHDC1	50154822
chr14	50159771	50160265	495	0.65	0.00966	0.0421	POLE2	50153098
chr14	50159771	50160265	495	0.65	0.00966	0.0421	POLE2	50153098
chr14	50159771	50160265	495	0.65	0.00966	0.0421	POLE2	50153098
chr5	1.5E+08	1.5E+08	212	1.03	0.00967	0.0421	TNIP1	1.5E+08
chr5	1.5E+08	1.5E+08	212	1.03	0.00967	0.0421	TNIP1	1.5E+08

chr19	49137757	49137973	217	-0.69	0.00967	0.0421	SEC1P	49136295
chr12	45585928	45586465	538	0.65	0.00972	0.0423	RNY5	45579252
chr7	20256975	20257569	595	-0.36	0.00973	0.0423	MACC1	20255013
chr7	20256975	20257569	595	-0.36	0.00973	0.0423	LOC10050€	20252199
chr7	20256975	20257569	595	-0.36	0.00973	0.0423	LOC10050€	20252199
chr7	20256975	20257569	595	-0.36	0.00973	0.0423	LOC10050€	20252199
chr19	57791148	57792004	857	0.32	0.00975	0.0424	ZNF460	57786852
chr11	65837484	65837874	391	0.76	0.00975	0.0424	PACS1	65832823
chr5	86416315	86416670	356	0.53	0.00978	0.0425	LOC101929	86410964
chr16	8954146	8954261	116	0.96	0.00979	0.0425	CARHSP1	8953667
chr16	8954146	8954261	116	0.96	0.00979	0.0425	CARHSP1	8953667
chr16	8954146	8954261	116	0.96	0.00979	0.0425	CARHSP1	8953667
chr4	39183754	39184249	496	0.41	0.0098	0.0425	WDR19	39179023
chr4	39183754	39184249	496	0.41	0.0098	0.0425	WDR19	39179023
chr11	1.29E+08	1.29E+08	353	-0.84	0.00993	0.043	ARHGAP32	1.29E+08
chr20	42295405	42295842	438	-0.57	0.00996	0.043	MYBL2	42290658
chr20	42295405	42295842	438	-0.57	0.00996	0.043	MYBL2	42290658
chr20	6034691	6034962	272	0.8	0.01	0.0432	LRRN4	6032726
chr3	79068478	79068903	426	0.56	0.01	0.0432	ROBO1	79066609
chr3	79068478	79068903	426	0.56	0.01	0.0432	ROBO1	79066609
chr9	1.4E+08	1.4E+08	265	0.65	0.01	0.0432	MAN1B1	1.4E+08
chr9	1.4E+08	1.4E+08	265	0.65	0.01	0.0432	MAN1B1	1.4E+08
chr9	1.4E+08	1.4E+08	265	0.65	0.01	0.0432	MAN1B1	1.4E+08
chr2	2.41E+08	2.41E+08	346	0.56	0.01	0.0432	MIR149	2.41E+08
chr1	47600014	47600213	200	0.61	0.01	0.0432	CYP4A22	47598096
chr1	47600014	47600213	200	0.61	0.01	0.0432	CYP4A22	47598096
chr1	45801718	45801864	147	0.84	0.01	0.0432	TOE1	45800341
chr1	32930388	32930731	344	0.73	0.0101	0.0434	ZBTB8B	32925657
chr3	1.59E+08	1.59E+08	201	0.77	0.0101	0.0435	IQCJ-SCHIP	1.59E+08
chr3	1.59E+08	1.59E+08	201	0.77	0.0101	0.0435	IQCJ-SCHIP	1.59E+08
chr3	1.59E+08	1.59E+08	201	0.77	0.0101	0.0435	IQCJ	1.59E+08
chr3	1.59E+08	1.59E+08	201	0.77	0.0101	0.0435	IQCJ	1.59E+08
chr3	1.59E+08	1.59E+08	201	0.77	0.0101	0.0435	IQCJ	1.59E+08
chr12	19592323	19592775	453	0.44	0.0101	0.0435	AEBP2	19587607
chr12	19592323	19592775	453	0.44	0.0101	0.0435	AEBP2	19587607
chr12	19592323	19592775	453	0.44	0.0101	0.0435	AEBP2	19588514
chr11	59436421	59437303	883	0.31	0.0101	0.0435	PATL1	59434511
chr12	1.13E+08	1.13E+08	201	1.24	0.0101	0.0436	OAS2	1.13E+08
chr12	1.13E+08	1.13E+08	201	1.24	0.0101	0.0436	OAS2	1.13E+08
chr12	1.13E+08	1.13E+08	201	1.24	0.0101	0.0436	OAS2	1.13E+08
chr14	20937342	20938511	1170	-0.35	0.0102	0.0437	PNP	20932537
chr7	2671123	2671672	550	-0.48	0.0102	0.0437	TTYH3	2666602
chr22	24643501	24643853	353	0.62	0.0102	0.0437	GGT5	24639110
chr22	24643501	24643853	353	0.62	0.0102	0.0437	GGT5	24639110
chr22	24643501	24643853	353	0.62	0.0102	0.0437	GGT5	24639110
chr22	24643501	24643853	353	0.62	0.0102	0.0437	GGT5	24639110
chr22	24643501	24643853	353	0.62	0.0102	0.0437	GGT5	24639110

chr22	24643501	24643853	353	0.62	0.0102	0.0437	POM121L9	24642588
chr1	97186934	97188051	1118	0.37	0.0102	0.0438	PTBP2	97182160
chr1	97186934	97188051	1118	0.37	0.0102	0.0438	PTBP2	97182160
chr1	97186934	97188051	1118	0.37	0.0102	0.0438	PTBP2	97182160
chr1	97186934	97188051	1118	0.37	0.0102	0.0438	PTBP2	97182160
chr1	97186934	97188051	1118	0.37	0.0102	0.0438	PTBP2	97182160
chr1	97186934	97188051	1118	0.37	0.0102	0.0438	PTBP2	97182160
chr1	97186934	97188051	1118	0.37	0.0102	0.0438	PTBP2	97182160
chr1	97186934	97188051	1118	0.37	0.0102	0.0438	PTBP2	97182160
chr1	97186934	97188051	1118	0.37	0.0102	0.0438	PTBP2	97183193
chr9	1.27E+08	1.27E+08	289	0.79	0.0102	0.0438	MIR181B2	1.27E+08
chr3	1.12E+08	1.12E+08	811	0.46	0.0102	0.0439	PHLDB2	1.12E+08
chr3	1.12E+08	1.12E+08	811	0.46	0.0102	0.0439	PHLDB2	1.12E+08
chr3	1.12E+08	1.12E+08	811	0.46	0.0102	0.0439	PHLDB2	1.12E+08
chr17	33571444	33571866	423	0.54	0.0102	0.044	SLFN5	33565085
chr1	1.02E+08	1.02E+08	201	0.85	0.0103	0.0441	LOC101928	1.02E+08
chr6	52149111	52149931	821	0.24	0.0103	0.0441	MCM3	52147679
chr6	52149111	52149931	821	0.24	0.0103	0.0441	MCM3	52147679
chr20	57465990	57466296	307	0.58	0.0103	0.0442	GNAS	57459179
chr20	57465990	57466296	307	0.58	0.0103	0.0442	LOC101927	57461865
chr20	57465990	57466296	307	0.58	0.0103	0.0442	GNAS	57461357
chr20	57465990	57466296	307	0.58	0.0103	0.0442	GNAS	57461357
chr20	57465990	57466296	307	0.58	0.0103	0.0442	GNAS	57461357
chr20	57465990	57466296	307	0.58	0.0103	0.0442	GNAS	57462218
chr20	57465990	57466296	307	0.58	0.0103	0.0442	GNAS	57461357
chr20	57465990	57466296	307	0.58	0.0103	0.0442	GNAS	57461357
chr12	1.24E+08	1.24E+08	474	-0.64	0.0103	0.0442	TCTN2	1.24E+08
chr12	1.24E+08	1.24E+08	474	-0.64	0.0103	0.0442	TCTN2	1.24E+08
chr1	2.49E+08	2.49E+08	1044	-0.24	0.0104	0.0443	PGBD2	2.49E+08
chr1	2.49E+08	2.49E+08	1044	-0.24	0.0104	0.0443	PGBD2	2.49E+08
chr9	1.31E+08	1.31E+08	529	-0.4	0.0104	0.0443	LCN2	1.31E+08
chr2	2.24E+08	2.24E+08	445	-0.49	0.0104	0.0445	ACSL3	2.24E+08
chr2	2.24E+08	2.24E+08	445	-0.49	0.0104	0.0445	ACSL3	2.24E+08
chr10	1.05E+08	1.05E+08	297	-0.76	0.0104	0.0446	CALHM3	1.05E+08
chr7	2594972	2595630	659	-0.45	0.0105	0.0448	IQCE	2593605
chr7	2594972	2595630	659	-0.45	0.0105	0.0448	IQCE	2593605
chr7	2594972	2595630	659	-0.45	0.0105	0.0448	IQCE	2593605
chr7	2594972	2595630	659	-0.45	0.0105	0.0448	IQCE	2593605
chr7	2594972	2595630	659	-0.45	0.0105	0.0448	IQCE	2593605
chr7	2594972	2595630	659	-0.45	0.0105	0.0448	BRAT1	2593392
chr11	62559347	62559592	246	0.7	0.0105	0.0449	TMEM223	62557486
chr11	62559347	62559592	246	0.7	0.0105	0.0449	MIR6514	62558243
chr8	25042064	25042392	329	0.59	0.0105	0.0449	DOCK5	25037286
chr1	2.17E+08	2.17E+08	239	1.32	0.0106	0.0449	ESRRG	2.17E+08
chr1	2.17E+08	2.17E+08	239	1.32	0.0106	0.0449	ESRRG	2.17E+08
chr11	10594308	10594508	201	0.7	0.0106	0.0449	LYVE1	10588365
chr19	7580765	7581002	238	0.88	0.0106	0.045	ZNF358	7576003

chr3	40515532	40515731	200	0.71	0.0107	0.0453	ZNF619	40513603
chr3	40515532	40515731	200	0.71	0.0107	0.0453	ZNF619	40513603
chr3	40515532	40515731	200	0.71	0.0107	0.0453	ZNF619	40513632
chr3	40515532	40515731	200	0.71	0.0107	0.0453	ZNF619	40513603
chr16	66614552	66614826	275	0.73	0.0107	0.0453	CMTM2	66608350
chr16	66614552	66614826	275	0.73	0.0107	0.0453	CMTM2	66608350
chr4	78977625	78978564	940	0.46	0.0107	0.0453	FRAS1	78973723
chr4	78977625	78978564	940	0.46	0.0107	0.0453	FRAS1	78973723
chr12	1.14E+08	1.14E+08	646	-0.36	0.0107	0.0453	RBM19	1.14E+08
chr12	1.14E+08	1.14E+08	646	-0.36	0.0107	0.0453	RBM19	1.14E+08
chr12	1.14E+08	1.14E+08	646	-0.36	0.0107	0.0453	RBM19	1.14E+08
chr4	1.59E+08	1.59E+08	263	1.03	0.0107	0.0455	FAM198B	1.59E+08
chr4	1.59E+08	1.59E+08	263	1.03	0.0107	0.0455	FAM198B	1.59E+08
chr4	1.59E+08	1.59E+08	263	1.03	0.0107	0.0455	FAM198B	1.59E+08
chr1	1.53E+08	1.53E+08	200	1.93	0.0107	0.0455	SPRR2D	1.53E+08
chr3	1.5E+08	1.5E+08	445	0.39	0.0107	0.0455	SELT	1.5E+08
chr16	31454279	31454676	398	0.61	0.0108	0.0456	ZNF843	31452348
chr15	81286358	81286556	199	0.6	0.0108	0.0457	MESDC2	81280219
chr15	81286358	81286556	199	0.6	0.0108	0.0457	MESDC2	81280219
chr15	81286358	81286556	199	0.6	0.0108	0.0457	MESDC2	81280219
chr10	46089794	46090527	734	0.37	0.0108	0.0458	8-Mar	46088354
chr5	1.38E+08	1.38E+08	199	0.88	0.0108	0.0458	BRD8	1.38E+08
chr5	1.38E+08	1.38E+08	199	0.88	0.0108	0.0458	BRD8	1.38E+08
chr5	1.38E+08	1.38E+08	199	0.88	0.0108	0.0458	BRD8	1.38E+08
chr5	1.38E+08	1.38E+08	199	0.88	0.0108	0.0458	BRD8	1.38E+08
chr5	1.38E+08	1.38E+08	199	0.88	0.0108	0.0458	BRD8	1.38E+08
chr5	1.38E+08	1.38E+08	199	0.88	0.0108	0.0458	BRD8	1.38E+08
chr11	68938243	68938435	193	1.16	0.0108	0.0458	LOC338694	68937030
chr7	37776471	37776664	194	0.9	0.0108	0.0459	GPR141	37774995
chr1	1.85E+08	1.85E+08	313	-0.91	0.0108	0.0459	FAM129A	1.85E+08
chr3	1.12E+08	1.12E+08	242	0.78	0.0109	0.0459	TMPRSS7	1.12E+08
chr3	1.12E+08	1.12E+08	242	0.78	0.0109	0.0459	TMPRSS7	1.12E+08
chr8	1.09E+08	1.09E+08	200	0.81	0.0109	0.0459	ANGPT1	1.09E+08
chr8	1.09E+08	1.09E+08	200	0.81	0.0109	0.0459	ANGPT1	1.09E+08
chr6	1.7E+08	1.7E+08	200	1.04	0.0109	0.046	WDR27	1.7E+08
chr6	1.7E+08	1.7E+08	200	1.04	0.0109	0.046	WDR27	1.7E+08
chr4	1.85E+08	1.85E+08	217	-0.84	0.0109	0.046	LOC102723	1.85E+08
chr4	1.85E+08	1.85E+08	217	-0.84	0.0109	0.046	LOC102723	1.85E+08
chr4	7436607	7436823	217	-0.81	0.0109	0.046	PSAPL1	7434700
chr11	32605095	32605607	513	0.38	0.0109	0.0461	EIF3M	32600312
chr11	32605095	32605607	513	0.38	0.0109	0.0461	EIF3M	32600312
chr2	1.31E+08	1.31E+08	1245	0.25	0.0109	0.0462	IMP4	1.31E+08
chr2	1.31E+08	1.31E+08	1245	0.25	0.0109	0.0462	CCDC115	1.31E+08
chr2	1.31E+08	1.31E+08	1245	0.25	0.0109	0.0462	CCDC115	1.31E+08
chr19	6464166	6464282	117	-0.92	0.011	0.0464	CRB3	6459259
chr19	6464166	6464282	117	-0.92	0.011	0.0464	CRB3	6459259
chr19	6464166	6464282	117	-0.92	0.011	0.0464	SLC25A23	6457781

chr3	1.84E+08	1.84E+08	273	0.56	0.011	0.0465	AP2M1	1.84E+08
chr3	1.84E+08	1.84E+08	273	0.56	0.011	0.0465	AP2M1	1.84E+08
chr3	1.84E+08	1.84E+08	273	0.56	0.011	0.0465	AP2M1	1.84E+08
chr13	33160244	33160723	480	0.48	0.0111	0.0466	PDS5B	33155563
chr22	19132078	19132461	384	-0.47	0.0111	0.0466	DGCR14	19130190
chr22	19132078	19132461	384	-0.47	0.0111	0.0466	DGCR14	19130190
chr3	1.96E+08	1.96E+08	238	-0.68	0.0111	0.0466	TM4SF19-T	1.96E+08
chr3	1.96E+08	1.96E+08	238	-0.68	0.0111	0.0466	TM4SF19	1.96E+08
chr3	1.96E+08	1.96E+08	238	-0.68	0.0111	0.0466	TM4SF19	1.96E+08
chr3	1.96E+08	1.96E+08	238	-0.68	0.0111	0.0466	TM4SF19	1.96E+08
chr1	2.15E+08	2.15E+08	147	1.61	0.0111	0.0467	KCNK2	2.15E+08
chr1	1.93E+08	1.93E+08	929	0.28	0.0111	0.0468	TROVE2	1.93E+08
chr1	1.93E+08	1.93E+08	929	0.28	0.0111	0.0468	TROVE2	1.93E+08
chr1	1.93E+08	1.93E+08	929	0.28	0.0111	0.0468	TROVE2	1.93E+08
chr1	1.93E+08	1.93E+08	929	0.28	0.0111	0.0468	TROVE2	1.93E+08
chr1	1.93E+08	1.93E+08	929	0.28	0.0111	0.0468	TROVE2	1.93E+08
chr1	1.93E+08	1.93E+08	929	0.28	0.0111	0.0468	UCHL5	1.93E+08
chr1	1.93E+08	1.93E+08	929	0.28	0.0111	0.0468	UCHL5	1.93E+08
chr1	1.93E+08	1.93E+08	929	0.28	0.0111	0.0468	UCHL5	1.93E+08
chr1	1.93E+08	1.93E+08	929	0.28	0.0111	0.0468	TROVE2	1.93E+08
chr1	1.93E+08	1.93E+08	929	0.28	0.0111	0.0468	UCHL5	1.93E+08
chr1	1.93E+08	1.93E+08	929	0.28	0.0111	0.0468	UCHL5	1.93E+08
chr11	93276429	93276790	362	-0.45	0.0112	0.0469	SMCO4	93274546
chr10	30642868	30643136	269	1.09	0.0112	0.0469	MTPAP	30636267
chr8	1.26E+08	1.26E+08	925	-0.31	0.0112	0.0469	NDUFB9	1.26E+08
chr8	1.26E+08	1.26E+08	925	-0.31	0.0112	0.0469	TATDN1	1.26E+08
chr8	1.26E+08	1.26E+08	925	-0.31	0.0112	0.0469	NDUFB9	1.26E+08
chr8	1.26E+08	1.26E+08	925	-0.31	0.0112	0.0469	NDUFB9	1.26E+08
chr8	1.26E+08	1.26E+08	925	-0.31	0.0112	0.0469	TATDN1	1.26E+08
chr8	1.26E+08	1.26E+08	925	-0.31	0.0112	0.0469	NDUFB9	1.26E+08
chr8	1.26E+08	1.26E+08	925	-0.31	0.0112	0.0469	TATDN1	1.26E+08
chr8	1.26E+08	1.26E+08	925	-0.31	0.0112	0.0469	TATDN1	1.26E+08
chr8	1.26E+08	1.26E+08	925	-0.31	0.0112	0.0469	TATDN1	1.26E+08
chr8	1.26E+08	1.26E+08	925	-0.31	0.0112	0.0469	TATDN1	1.26E+08
chr5	35857957	35858238	282	0.62	0.0112	0.0469	IL7R	35851976
chr5	35857957	35858238	282	0.62	0.0112	0.0469	IL7R	35851976
chr5	1.27E+08	1.27E+08	306	0.66	0.0112	0.047	CCDC192	1.27E+08
chr7	42972437	42972645	209	0.82	0.0112	0.0471	PSMA2	42969805
chr7	42972437	42972645	209	0.82	0.0112	0.0471	MRPL32	42966938
chr14	56261456	56261667	212	-0.83	0.0112	0.0471	LINC00520	56261392
chr14	56261456	56261667	212	-0.83	0.0112	0.0471	LINC00520	56261392
chr10	1E+08	1E+08	599	0.4	0.0112	0.0471	LOXL4	1E+08
chr6	13407897	13408431	535	-0.46	0.0113	0.0473	GFOD1	13406369
chr1	1.52E+08	1.52E+08	341	0.65	0.0113	0.0473	RPTN	1.52E+08
chr15	49338168	49339002	835	0.37	0.0113	0.0473	SECISBP2L	49336760
chr15	49338168	49339002	835	0.37	0.0113	0.0473	SECISBP2L	49336760
chr15	83879404	83879735	332	1.06	0.0113	0.0474	HDGFRP3	83874770

chr12	52609392	52609777	386	0.51	0.0113	0.0474	C12orf80	52602639
chr12	80084249	80085297	1049	0.34	0.0114	0.0475	PAWR	80082790
chr6	53791964	53792351	388	0.56	0.0114	0.0476	LOC101927	53789779
chr7	24795329	24795513	185	0.64	0.0114	0.0477	DFNA5	24795083
chr5	89825191	89826124	934	0.31	0.0115	0.0477	LYSMD3	89823401
chr5	89825191	89826124	934	0.31	0.0115	0.0477	LYSMD3	89823401
chr4	1.84E+08	1.84E+08	803	-0.33	0.0115	0.0477	LOC389247	1.84E+08
chr4	1.84E+08	1.84E+08	803	-0.33	0.0115	0.0477	ING2	1.84E+08
chr4	1.84E+08	1.84E+08	803	-0.33	0.0115	0.0477	ING2	1.84E+08
chr8	27748411	27748586	176	0.92	0.0115	0.0478	MIR4287	27741633
chr11	68227544	68228295	752	0.36	0.0115	0.0478	PPP6R3	68223185
chr11	68227544	68228295	752	0.36	0.0115	0.0478	PPP6R3	68223185
chr11	68227544	68228295	752	0.36	0.0115	0.0478	PPP6R3	68223185
chr11	68227544	68228295	752	0.36	0.0115	0.0478	PPP6R3	68223185
chr11	68227544	68228295	752	0.36	0.0115	0.0478	PPP6R3	68223185
chr11	68227544	68228295	752	0.36	0.0115	0.0478	PPP6R3	68223185
chr22	17565589	17566355	767	0.29	0.0115	0.0478	IL17RA	17560848
chr22	17565589	17566355	767	0.29	0.0115	0.0478	IL17RA	17560848
chr2	1.67E+08	1.67E+08	485	0.38	0.0115	0.0479	SCN9A	1.67E+08
chr8	52811486	52812342	857	0.27	0.0115	0.0479	PCMTD1	52809746
chr8	52811486	52812342	857	0.27	0.0115	0.0479	PCMTD1	52809746
chr8	52811486	52812342	857	0.27	0.0115	0.0479	PCMTD1	52809746
chr2	1.28E+08	1.28E+08	521	0.69	0.0115	0.0479	PROC	1.28E+08
chr1	1.12E+08	1.12E+08	1022	0.25	0.0115	0.0479	LRIF1	1.12E+08
chr1	1.12E+08	1.12E+08	1022	0.25	0.0115	0.0479	LRIF1	1.12E+08
chr1	64937444	64937644	201	0.72	0.0115	0.048	CACHD1	64931475
chr11	12398956	12399929	974	0.48	0.0115	0.048	PARVA	12394025
chr1	1.6E+08	1.6E+08	201	0.71	0.0116	0.0481	FCRL6	1.6E+08
chr1	1.6E+08	1.6E+08	201	0.71	0.0116	0.0481	FCRL6	1.6E+08
chr9	1.17E+08	1.17E+08	198	0.88	0.0116	0.0482	C9orf91	1.17E+08
chr8	1.25E+08	1.25E+08	1382	-0.22	0.0116	0.0482	RNF139	1.25E+08
chr8	1.25E+08	1.25E+08	1382	-0.22	0.0116	0.0482	RNF139-AS	1.25E+08
chr8	1.25E+08	1.25E+08	1382	-0.22	0.0116	0.0482	RNF139-AS	1.25E+08
chr1	2.31E+08	2.31E+08	423	-0.61	0.0117	0.0484	FAM89A	2.31E+08
chr2	1.79E+08	1.79E+08	222	-0.68	0.0117	0.0484	PDE11A	1.79E+08
chr19	52254953	52255212	260	-1.25	0.0117	0.0485	FPR1	52253150
chr19	52254953	52255212	260	-1.25	0.0117	0.0485	FPR1	52253150
chr9	88968714	88969748	1035	-0.29	0.0117	0.0485	ZCCHC6	88967402
chr9	88968714	88969748	1035	-0.29	0.0117	0.0485	ZCCHC6	88967402
chr9	88968714	88969748	1035	-0.29	0.0117	0.0485	ZCCHC6	88967402
chr1	1.56E+08	1.56E+08	786	0.51	0.0117	0.0485	DAP3	1.56E+08
chr1	1.56E+08	1.56E+08	786	0.51	0.0117	0.0485	DAP3	1.56E+08
chr1	1.56E+08	1.56E+08	786	0.51	0.0117	0.0485	DAP3	1.56E+08
chr1	1.56E+08	1.56E+08	786	0.51	0.0117	0.0485	DAP3	1.56E+08
chr1	1.56E+08	1.56E+08	786	0.51	0.0117	0.0485	YY1AP1	1.56E+08
chr1	1.56E+08	1.56E+08	786	0.51	0.0117	0.0485	YY1AP1	1.56E+08
chr1	1.56E+08	1.56E+08	786	0.51	0.0117	0.0485	YY1AP1	1.56E+08

chr1	1.56E+08	1.56E+08	786	0.51	0.0117	0.0485	YY1AP1	1.56E+08
chr1	1.56E+08	1.56E+08	786	0.51	0.0117	0.0485	YY1AP1	1.56E+08
chr1	1.56E+08	1.56E+08	786	0.51	0.0117	0.0485	YY1AP1	1.56E+08
chr1	1.56E+08	1.56E+08	786	0.51	0.0117	0.0485	YY1AP1	1.56E+08
chr1	1.56E+08	1.56E+08	786	0.51	0.0117	0.0485	YY1AP1	1.56E+08
chr1	1.56E+08	1.56E+08	786	0.51	0.0117	0.0485	DAP3	1.56E+08
chr1	1.56E+08	1.56E+08	786	0.51	0.0117	0.0485	YY1AP1	1.56E+08
chr1	1.56E+08	1.56E+08	786	0.51	0.0117	0.0485	YY1AP1	1.56E+08
chr1	1.56E+08	1.56E+08	786	0.51	0.0117	0.0485	YY1AP1	1.56E+08
chr16	2896709	2897143	435	0.72	0.0117	0.0486	PRSS30P	2890752
chr6	49518132	49518766	635	0.31	0.0118	0.0486	C6orf141	49513112
chr9	75767474	75767605	132	0.92	0.0118	0.0487	ANXA1	75761646
chr18	39532991	39533190	200	0.78	0.0118	0.0489	PIK3C3	39530162
chr18	39532991	39533190	200	0.78	0.0118	0.0489	PIK3C3	39530162
chr17	70024587	70024787	201	0.76	0.0118	0.0489	LINC01152	70021794
chr17	70024587	70024787	201	0.76	0.0118	0.0489	LINC01152	70021794
chr17	70024587	70024787	201	0.76	0.0118	0.0489	LOC10272[illegible]	70020120
chr2	37455875	37456074	200	0.87	0.0118	0.0489	NDUFAF7	37453773
chr2	37455875	37456074	200	0.87	0.0118	0.0489	NDUFAF7	37453773
chr13	27699587	27700348	762	0.39	0.0119	0.0491	USP12-AS1	27694669
chr22	50353393	50354481	1089	-0.35	0.0119	0.0491	PIM3	50349142
chr22	50353393	50354481	1089	-0.35	0.0119	0.0491	MIR6821	50351513
chr2	1.28E+08	1.28E+08	221	0.8	0.0119	0.0492	ERCC3	1.28E+08
chr2	1.28E+08	1.28E+08	221	0.8	0.0119	0.0492	ERCC3	1.28E+08
chr2	1.28E+08	1.28E+08	221	0.8	0.0119	0.0492	ERCC3	1.28E+08
chr3	27258616	27258917	302	0.95	0.0119	0.0492	NEK10	27256014
chr3	27258616	27258917	302	0.95	0.0119	0.0492	NEK10	27256014
chr17	46696089	46696258	170	1.33	0.012	0.0494	HOXB8	46690301
chr12	47578699	47578955	257	0.78	0.012	0.0494	MIR4698	47576594
chr7	23634731	23634977	247	-0.56	0.012	0.0494	CCDC126	23631997
chr7	1.44E+08	1.44E+08	140	-1.01	0.0121	0.0496	ARHGEF5	1.44E+08
chr7	1.35E+08	1.35E+08	666	-0.33	0.0121	0.0496	C7orf49	1.35E+08
chr7	1.35E+08	1.35E+08	666	-0.33	0.0121	0.0496	C7orf49	1.35E+08
chr7	1.35E+08	1.35E+08	666	-0.33	0.0121	0.0496	C7orf49	1.35E+08
chr7	1.35E+08	1.35E+08	666	-0.33	0.0121	0.0496	C7orf49	1.35E+08
chr7	1.35E+08	1.35E+08	666	-0.33	0.0121	0.0496	C7orf49	1.35E+08
chr7	1.35E+08	1.35E+08	666	-0.33	0.0121	0.0496	C7orf49	1.35E+08
chr7	1.35E+08	1.35E+08	666	-0.33	0.0121	0.0496	C7orf49	1.35E+08
chr7	1.35E+08	1.35E+08	666	-0.33	0.0121	0.0496	C7orf49	1.35E+08
chr7	1.35E+08	1.35E+08	666	-0.33	0.0121	0.0496	C7orf49	1.35E+08
chr22	29196043	29196866	824	-0.42	0.0121	0.0496	XBP1	29194560
chr22	29196043	29196866	824	-0.42	0.0121	0.0496	XBP1	29194560
chr1	43123699	43124497	799	0.44	0.0121	0.0497	PPIH	43119047
chr6	74019396	74020162	767	0.61	0.0121	0.0497	KHDC1	74017938
chr6	74019396	74020162	767	0.61	0.0121	0.0497	KHDC1	74018088
chr13	96742243	96742738	496	0.82	0.0121	0.0497	HS6ST3	96738092
chr20	48531938	48532477	540	-0.47	0.0122	0.0498	SPATA2	48530080

chr20	48531938	48532477	540	-0.47	0.0122	0.0498	SPATA2	48528276
chr20	48531938	48532477	540	-0.47	0.0122	0.0498	LOC105372	48527358
chr9	1.31E+08	1.31E+08	199	0.66	0.0122	0.0499	MIR2861	1.31E+08
chr9	1.31E+08	1.31E+08	199	0.66	0.0122	0.0499	CDK9	1.31E+08
chr9	1.31E+08	1.31E+08	199	0.66	0.0122	0.0499	MIR3960	1.31E+08
chr12	67662253	67663459	1207	0.25	0.0122	0.05	CAND1	67658060

promoterstop
61869234
61869234
2.31E+08
48759307
48759307
48759063
1.38E+08
8836272
1.83E+08
1.29E+08
79538661
56256747
54658329
54658370
23677397
71522811
71525694
71524926
71525694
1.01E+08
1.03E+08
36157015
68304155
20075849
75254883
1.28E+08
43567141
1.03E+08
1.03E+08
86087657
7141417
12455284
12455284
57238335
57238335
57238335
56644477
1.43E+08
67948518
1106567
1106567
58405442
1601066
1.14E+08

74334308
7990059
1.13E+08
1.13E+08
1.13E+08
12777011
97507881
11941709
1.17E+08
2.11E+08
19736880
19736880
19736880
19741218
2.27E+08
30456396
74653757
74653757
74653757
1.05E+08
64066744
64066744
64066744
64066744
2.29E+08
2.29E+08
2.29E+08
3616609
20135283
1.57E+08
56221194
46585887
1.01E+08
1.14E+08
1.14E+08
76056271
1.42E+08
1.42E+08
1.42E+08
1.42E+08
1.42E+08
1.42E+08
29461374
29458037
1.22E+08
7330079
61067788

48168966
69203704
79058581
83413532
49611654
49611654
49611654
49611654
57793852
24101323
1.9E+08
742056
54488859
54488859
54488859
54488859
54488859
22990781
22989090
22992012
15399595
15399595
39444086
39444086
41024537
76972299
76972299
76972299
76971911
55906830
55906830
53090944
1.45E+08
1.22E+08
27327229
27327229
27327229
27327229
1.09E+08
46724643
46724316
31458476
1.4E+08
89229733
89229146
89229733
89230387

89229733
89229733
1.49E+08
1.49E+08
43210925
43210925
43210925
47756803
44185916
1.14E+08
1.14E+08
80064564
27795102
27795102
27795102
27795102
27795102
27795102
77504584
51745463
43271682
1.66E+08
86121157
31524740
1.36E+08
30502780
17907918
1.34E+08
54940282
37573752
2.49E+08
1.89E+08
44290409
1.35E+08
1.35E+08
93456679
94656167
40996019
1.96E+08
51238700
1.03E+08
1.03E+08
47668288
27585477
31099761
2.16E+08
2.16E+08

2.16E+08
2.16E+08
2.16E+08
2.16E+08
2.16E+08
2.16E+08
2.16E+08
2.16E+08
62902985
46690977
13913966
13913966
1.5E+08
1.5E+08
44356858
41955354
41955354
41955354
1.6E+08
88722425
88720287
1.55E+08
1.07E+08
1.07E+08
1.07E+08
1.04E+08
94302473
1.39E+08
1.39E+08
51509958
51509958
51509958
51509958
51509958
51509958
1.25E+08
1.29E+08
21512384
1202420
1202009
1202009
1202009
1202009
1202009
1.5E+08
1.97E+08
1.97E+08

1.51E+08
1.51E+08
1.51E+08
1.51E+08
20843832
36623565
36623565
36620115
36620115
36620115
36620115
36620115
36623565
36620115
27517708
23524551
23524551
99722059
86415771
1.28E+08
1.28E+08
55985750
1.96E+08
43951231
43951668
43951668
43951668
1.25E+08
1.25E+08
1.25E+08
1.25E+08
1.25E+08
1.25E+08
1.47E+08
41088243
41088243
8816572
14666782
45682867
45682867
27526311
50479238
50478381
50478237
50478381
9420795
75914388

75914388
47475385
47475385
47478734
47478734
1.09E+08
1.34E+08
54293143
54292928
54293958
1.81E+08
30504805
73977919
1.53E+08
1.53E+08
1.53E+08
19533776
87565565
87565565
87565565
87565565
87565565
33719762
33719762
76196569
56270680
2.01E+08
2.01E+08
2.01E+08
2.01E+08
2.01E+08
2.01E+08
2.01E+08
12310446
1.6E+08
1.6E+08
1.6E+08
1.83E+08
1.14E+08
1.14E+08
1.98E+08
1.98E+08
77465540
14430276
3066121
3066121
1.09E+08

1.04E+08
1.02E+08
30158326
74332672
1.32E+08
2.42E+08
1.4E+08
1.17E+08
1.17E+08
2.26E+08
2.26E+08
2.26E+08
59488147
23749799
1.1E+08
1.51E+08
1.51E+08
1.51E+08
1.51E+08
44264142
51802110
57290369
2559496
2561478
2561478
1.26E+08
42167694
7564444
2.36E+08
85105703
41206507
56681539
1.02E+08
1.02E+08
1.39E+08
1.39E+08
19839600
19980706
99334203
1515971
1.94E+08
1.94E+08
1.94E+08
37857388
37857388
80998066
80998066

80998066
80998066
80998066
2.36E+08
1.34E+08
39322880
1.75E+08
21999542
21999542
2.09E+08
1.77E+08
1.77E+08
1.77E+08
1.77E+08
1.14E+08
1.5E+08
1.14E+08
1.54E+08
1.54E+08
13912251
13912251
13913966
13913966
11435537
1.32E+08
1.32E+08
1.32E+08
1.32E+08
1.32E+08
1.8E+08
23301091
23301091
23301091
23301091
23301091
23301091
23301091
83679583
35898508
1857539
1857539
1857539
1857539
10459049
96191164
1.5E+08
45479330

1363507
1361824
1361824
1361824
45601900
38061854
5492832
5492832
5492832
5492832
5492832
63848320
1.25E+08
10329790
10329790
10333556
10329790
77182479
43518323
43518323
32029044
38037077
33795762
12805808
12805808
12805808
77058140
77058140
77058140
77058140
5634435
48294054
1.53E+08
59565304
2.1E+08
79475738
26158558
75801848
8977067
1.44E+08
1.44E+08
1.44E+08
61429804
75595184
75595184
75595184
1.96E+08

1.96E+08
1.96E+08
1.96E+08
83842581
22710152
1.12E+08
1.12E+08
1.12E+08
1.12E+08
12819388
64220936
1.06E+08
1.06E+08
1.06E+08
1.06E+08
1.06E+08
1.06E+08
1.06E+08
1.06E+08
33626222
1.61E+08
27495277
27495277
8548344
8545492
8545492
8545492
8545492
73276677
25266635
25266635
71988878
41524173
10436561
30647840
30647840
62374312
24981717
1.1E+08
30334506
30331409
1.51E+08
10517617
1.71E+08
1.71E+08
1.71E+08
1.71E+08

1.71E+08
56512694
56512694
63056892
63057920
54935982
68073365
68073365
68073365
68073365
24737416
27296326
1.16E+08
1.22E+08
2.07E+08
2.07E+08
2.07E+08
2.07E+08
2.07E+08
1.17E+08
86504100
67634168
71790970
71790970
71790970
36285236
36287643
1.03E+08
1.03E+08
1.06E+08
15274414
1.7E+08
89344717
1.14E+08
1.14E+08
1.14E+08
3016945
56281431
48886022
24107572
24107572
24101323
1.71E+08
1.71E+08
1.71E+08
95395583
95397779

95397779
95396364
1.23E+08
1.54E+08
1.54E+08
1.54E+08
62790083
51159788
37942118
2.08E+08
60938347
60939226
10514306
1.93E+08
1.02E+08
1.26E+08
1.61E+08
1.61E+08
1.61E+08
1.61E+08
56769017
36018555
36024401
94642648
94642648
3343079
56485082
56270680
39830164
1.18E+08
1.23E+08
70730870
1.51E+08
36953915
36953915
36953915
77739320
1.23E+08
1.23E+08
23307741
82302574
1.23E+08
1.53E+08
36273660
36273863
1.06E+08
17544605

17544605
17544605
1.32E+08
1.32E+08
31646347
31646347
1.4E+08
38646721
38646721
44639546
65888334
65888334
65888334
65888398
65888130
65888130
1.31E+08
1.31E+08
1.31E+08
1.11E+08
1.11E+08
1.11E+08
1.11E+08
1.11E+08
1.78E+08
1.01E+08
1.01E+08
32179425
68152859
68152859
68152859
52388737
52386710
20262013
14642354
14642378
14642378
14642378
37843739
1.62E+08
1.62E+08
55602583
49572124
1.25E+08
1.53E+08
22604955
1.57E+08

1.69E+08
71064243
42998435
1.02E+08
1.02E+08
1.02E+08
21536765
1.91E+08
62549528
47338961
99185669
60693912
60693912
76336796
1.49E+08
1.61E+08
1.61E+08
1.61E+08
1.61E+08
1.51E+08
38066403
38069325
33519105
1.07E+08
1.07E+08
45460137
1.24E+08
1.24E+08
24246117
38088884
84036012
84040911
84040911
1.5E+08
1.5E+08
1.5E+08
1.5E+08
1.5E+08
1.12E+08
1.12E+08
2445194
2445194
2445194
72292275
1.11E+08
9781839
9786080

62186372
41727103
41727103
6450883
94494723
15274414
1.08E+08
1.54E+08
1.55E+08
65155145
65156172
65154857
65154857
65155145
65156040
65156040
65156040
1.66E+08
27064508
26966370
36434998
36434998
36434998
39200102
53303868
1.35E+08
67564719
67564716
67565539
95068849
1.7E+08
95290837
14894793
2.45E+08
2.45E+08
2.45E+08
1.11E+08
1.02E+08
630067
630067
632173
630067
1.81E+08
1.34E+08
1.34E+08
1.34E+08
28504095

17809382
17809382
2888959
2888959
72714139
72714139
72714139
72714139
72714139
72714139
72714139
72714139
86865199
99802492
99799541
99799541
2.11E+08
52537883
99854678
99855162
99855162
1.02E+08
1.57E+08
1.57E+08
1.21E+08
1.51E+08
1.4E+08
1.4E+08
1.4E+08
49842686
49842268
17395735
338174
43888205
61256533
61256533
1.67E+08
1.67E+08
99475464
57655604
57655604
57655909
57655909
2.2E+08
64417972
71119074
1.09E+08

23451432
1.04E+08
12904309
99334197
99334197
99334197
99334197
46927675
12310446
1.06E+08
1.99E+08
2.2E+08
2.2E+08
1.02E+08
27240455
1.14E+08
1.14E+08
1.28E+08
55088724
55088724
55088724
55088724
1.33E+08
20430194
20429927
87045257
44203887
44203887
40996019
74922351
74922351
1.27E+08
1.27E+08
1.27E+08
35493276
35497087
1.2E+08
1.2E+08
1.2E+08
1.2E+08
76496284
76499106
76499106
76497191
76497191
1.28E+08
1.96E+08

75672861
75672861
1.3E+08
90478971
63554
57128454
1.18E+08
56570762
1.32E+08
1.32E+08
1.32E+08
39722998
1.44E+08
1.44E+08
1.44E+08
23451432
2.41E+08
21326839
1.54E+08
1.54E+08
1.49E+08
1.49E+08
1.49E+08
1.97E+08
1.97E+08
1.26E+08
1.49E+08
1.49E+08
1.75E+08
45965871
45965871
39680270
39678469
1.18E+08
39785882
56200632
56200632
56200632
56200632
45241542
61724190
57169058
47050955
77754502
77754502
77754502
77754494

15742023
15742023
15742023
15739123
15739150
31076385
10459049
10462416
10462416
1.22E+08
1.27E+08
1.38E+08
1.38E+08
94885702
1.02E+08
1.03E+08
1.03E+08
1.03E+08
1.03E+08
35552579
35552579
35556848
35552579
52198038
52198592
52197864
52198506
52194686
52198596
1.35E+08
30311309
79766139
79767070
83741106
83741106
6317242
8836272
72753277
23972512
23974416
1.94E+08
7856603
1.88E+08
52232245
52232245
24646110
24646110

24646110
24646110
24646110
24649588
6246839
6246839
2.25E+08
2.25E+08
12571206
12571206
56418076
56417099
1.77E+08
2.12E+08
2.12E+08
2.22E+08
39775166
23610297
50805050
50805050
51798469
51798469
11987215
91403803
17577592
661964
1.41E+08
1.41E+08
1.41E+08
1.41E+08
1.89E+08
1.7E+08
43562195
43562195
43562521
89344717
23865380
1.73E+08
1.52E+08
92419046
1.1E+08
1.74E+08
35721059
95068849
55725874
55725874
10656569

10656569
10656569
10656569
16849738
17313081
17312173
17312173
17313081
2.24E+08
60637014
27263014
27263014
12805182
21191531
48764203
48764203
51202868
51202868
94656167
48637593
39785882
96659143
68272402
68275136
84155548
92931011
92931011
92931011
92931011
92933682
10333556
97696295
97696295
97696295
97696295
35670278
35675914
11436043
1.16E+08
1.16E+08
1.16E+08
1.16E+08
35546963
35546963
35546963
35546963
1.6E+08

1.6E+08
1.6E+08
1.6E+08
45929958
90426121
90424245
90426121
90424245
90425901
90425901
90426121
59106756
59107554
59106756
2.35E+08
2.35E+08
71339674
71339833
1.83E+08
1.16E+08
1.16E+08
1.16E+08
1.16E+08
2.28E+08
1.15E+08
1.15E+08
1.15E+08
28518780
56540483
1.14E+08
89317537
2.08E+08
1.78E+08
1.78E+08
38187662
38188898
15908959
1.5E+08
9736916
9734157
33435414
33435414
33435414
1.47E+08
1.03E+08
22401533
1.62E+08

1.62E+08
1.62E+08
1.62E+08
1.62E+08
1.62E+08
1.62E+08
49588107
49588107
49588107
10929599
1.08E+08
1.08E+08
1.08E+08
1.7E+08
1.7E+08
1.7E+08
1.7E+08
45506706
28286374
28286802
28286374
28286374
28286802
28289848
91015579
89445466
89445466
1.16E+08
1.05E+08
1.05E+08
1.05E+08
72118324
88430205
88430425
88430425
88430425
88430320
88430425
99346101
99346101
94957979
1.32E+08
37528198
29471065
29471065
1.07E+08
80998066

80998066
80998066
80998066
80998066
46482081
46482081
46480703
99284579
95234531
1.12E+08
1.12E+08
1.12E+08
2.07E+08
2.07E+08
2.07E+08
2.07E+08
1.53E+08
31458476
87908100
41227790
41226011
41225007
1.03E+08
1.03E+08
1.03E+08
45313616
45313616
1.14E+08
1.39E+08
1.06E+08
53325253
46581138
42821218
42821218
2.01E+08
2.01E+08
2.01E+08
25471753
25471753
25471753
25471753
25471753
25471753
25471753
77742655
39774079
57880870

57880014
34689663
46715498
2.26E+08
33365233
33897124
13592624
13592624
55822037
1.55E+08
1.55E+08
1.55E+08
1.55E+08
1.55E+08
1.24E+08
1.24E+08
1.24E+08
1.62E+08
1.62E+08
73502581
24153552
24153552
24153552
24153552
24153552
1.15E+08
49464800
64175219
1.39E+08
1.39E+08
80057258
80057258
80060366
54887145
1.47E+08
77427980
1.7E+08
1.36E+08
1.7E+08
1.7E+08
68687791
2.33E+08
23294020
46808849
46808849
1.8E+08
1.8E+08

1.8E+08
1.8E+08
1.8E+08
99621389
41927355
41927355
41927353
11237915
11237915
11237915
55494231
40843409
2.33E+08
1.51E+08
77327742
77331662
77330582
77330582
77330582
98352868
1.19E+08
1.19E+08
26113364
21461588
21461588
21461588
31484121
31484159
31484121
31479249
1.39E+08
26685621
10547653
22459113
22459113
22459113
24567926
1.01E+08
1.14E+08
1.14E+08
1.75E+08
47696841
52919243
3596111
1.01E+08
1.61E+08
32085186

46742715
46742715
80840125
69347631
69349216
834951
834951
834951
834951
1.32E+08
78111808
78111808
78111808
95068849
82757640
59040715
1.11E+08
1.11E+08
44356858
1.17E+08
22425178
21393561
64283916
1.3E+08
1.21E+08
46849955
46849985
82175574
82175574
41711936
58941811
58940902
33560824
33395759
1.55E+08
1.55E+08
1.55E+08
1.05E+08
44047389
12481475
17222531
17219669
17218931
1.32E+08
1.32E+08
80525048
80525048

46657611
46657611
1.72E+08
1.72E+08
1.72E+08
44756919
41961887
24588397
24588397
24588397
10027458
52628953
15014504
71562022
71562153
92982358
92981907
92982358
22957754
73940175
41939635
48130199
48130808
48130199
48130850
48130736
48130850
95014927
48120333
10839642
10839642
1.98E+08
1.98E+08
87194618
10633668
10633126
59297005
1.95E+08
1.95E+08
1.95E+08
1.95E+08
1.24E+08
39157024
39156467
39157024
64651240
52186000

1.73E+08
1.73E+08
90289479
90288572
2.1E+08
2.1E+08
2.1E+08
1.3E+08
2.32E+08
47803073
1.28E+08
1.28E+08
88782045
90461222
90461222
27486458
1.12E+08
1.12E+08
1.44E+08
65598931
63336837
63336837
63336837
63336837
63336837
63336837
63336837
85683043
61664017
1.75E+08
1.75E+08
1.75E+08
1.75E+08
89576795
89576795
24743048
1.68E+08
1.68E+08
1.33E+08
30519879
2466684
1.09E+08
85362582
1.29E+08
1.29E+08
1.12E+08
7484804

7483272
7484805
7488964
7488964
1.02E+08
1.02E+08
1.02E+08
73303886
73304041
73306938
73303886
91416768
91413821
91418107
75406515
56783636
41708997
41707798
41707139
41708265
41708265
1.98E+08
7622671
7623077
1.12E+08
30179608
1.53E+08
1.6E+08
1.5E+08
11879814
28320294
28320202
28320294
28318513
28321600
28317363
28317363
42751736
90546722
85642820
85642820
81898748
49552527
49552527
49552527
49549520
49549520

49549520
1.72E+08
76081799
76081799
1.37E+08
1.05E+08
52934753
71790970
71790970
71790970
95131234
95130995
95130995
95130758
95133707
60342557
1.22E+08
62540774
23288315
23288315
1.04E+08
72292275
2.35E+08
2.35E+08
2822540
2821911
24614964
24614964
66772429
66772429
66772429
66769624
66769624
23526222
1.33E+08
1.24E+08
1.25E+08
61655817
1.84E+08
27440578
29915585
29915585
29915585
29915585
29915585
29915585
29914146

20375848
1695081
1.4E+08
96637289
85010132
85010132
10480148
1.19E+08
1.46E+08
1.46E+08
1.46E+08
1.46E+08
1.31E+08
32799897
69981207
69981461
69980502
1.81E+08
1.81E+08
1.01E+08
1.51E+08
67314059
67315426
67315049
67315426
2.48E+08
13266188
13266188
13266188
13266188
69460949
28222728
12229059
12228999
1.31E+08
1.31E+08
99129235
99129235
99129235
1.21E+08
22183355
16037287
1.2E+08
50684166
50680957
1.11E+08
1.11E+08

1.27E+08
1.27E+08
1.27E+08
1.27E+08
1.2E+08
1.2E+08
1.2E+08
1.52E+08
73525829
98643259
98643259
99991522
99991522
99992135
97176122
97176122
36793752
36793752
35593757
35593757
35593526
35593526
35593526
35596748
35596749
35596748
35596749
24377381
1.32E+08
56112075
56112075
56111817
56116150
56116150
56111817
56111817
62601933
72796249
72796249
32560207
61450413
1.17E+08
1.46E+08
2.02E+08
2.02E+08
2.02E+08
2.02E+08

2.02E+08
11702963
1.36E+08
1.36E+08
77650101
33837655
55415728
11202037
11202037
11202037
11202037
11202037
46741720
77901857
13716796
2.13E+08
32407727
32407727
32407727
32407727
32407727
32407727
32407727
32407727
32407727
32407727
32407727
1.5E+08
1.44E+08
1.44E+08
1.44E+08
1.53E+08
48284000
1.52E+08
25716223
7990059
57184414
57184414
57181602
79474741
1.46E+08
1.46E+08
2.07E+08
2.07E+08
2.07E+08
2.07E+08
2.07E+08

2.07E+08
32899021
32898654
32901445
1.13E+08
53344842
53344654
85402320
27929209
1.39E+08
1.39E+08
1.39E+08
67126067
67126067
67126067
67126067
67126067
58037276
58038762
58037276
58037276
2.27E+08
2.27E+08
2.27E+08
86960694
86960694
33085777
33085777
1.02E+08
10679573
57156526
2181225
27281525
54783514
54783514
1.57E+08
1.57E+08
1.57E+08
1.57E+08
18722281
24476145
24476641
61203458
61203458
35996535
21544031
21540418

21540418
21540418
73942249
73942249
73942249
73942249
73942249
73937847
84459066
84459259
84459469
1.12E+08
27548138
27548138
27548138
27548138
27548446
27548138
27548138
27548138
27548138
76787395
76787395
1.41E+08
1.41E+08
1.41E+08
81294814
81295294
89923272
51745463
1.13E+08
1.13E+08
1.81E+08
1.81E+08
1.81E+08
1.81E+08
1.81E+08
1.2E+08
30377157
32834133
32834133
32834133
32834133
32834133
32834133
32834133
45665887

45665887
45665887
45665887
86701850
1.85E+08
87739907
92924768
92924768
53121763
1.1E+08
72668528
72672289
72672289
1.35E+08
43138595
43140935
47250048
1.51E+08
2.04E+08
35908188
35911479
55989698
87569696
87569696
87569696
87566698
87566698
87566832
87566832
87569696
2.38E+08
2.38E+08
2.38E+08
2.38E+08
2.38E+08
47636079
47636079
47636079
47636114
40143501
52484043
1.06E+08
65213928
35077739
1.24E+08
66149077
66149077

51391902
1.21E+08
1.21E+08
1.21E+08
2850485
1.09E+08
1.09E+08
15947510
15516711
15516711
15514432
64973291
64975563
64973291
47449420
96090224
96090148
96090410
85918313
85915783
97563478
24838116
24840287
24839225
24838116
24840358
24839225
1.8E+08
1.8E+08
1.8E+08
56721815
56721815
56721815
1.25E+08
1.04E+08
1.04E+08
1.04E+08
1.04E+08
1.19E+08
18809423
56290670
56290670
56291592
56290031
56287238
18116588
18116588

18116588
18123349
18123349
18123349
18123349
18123349
18123349
18123349
18123349
18123349
18123349
1.28E+08
54758358
1.37E+08
40182356
15941756
24411131
94885702
1.71E+08
1.71E+08
1.71E+08
1.71E+08
1.4E+08
1.4E+08
1.4E+08
1.4E+08
1.81E+08
1.81E+08
1.81E+08
1.81E+08
1.81E+08
1.81E+08
35939351
61577223
96809175
36889069
36889069
1.24E+08
1.24E+08
1.24E+08
1.24E+08
64583188
64583035
64583188
64583766
64583766
64583213

64583035
7536995
49532632
1.34E+08
1.34E+08
40662444
40662444
40662444
40662444
1.5E+08
17191343
17191343
79117548
51389515
51389515
51389515
42133010
69065904
69065904
69065904
69067481
46708151
46708430
1.11E+08
22543000
41927355
41927355
41927353
47590178
1.77E+08
38383177
1.03E+08
1.03E+08
1.03E+08
1.03E+08
1.03E+08
1.03E+08
35602208
35602175
2.28E+08
2.28E+08
2.28E+08
2.28E+08
2.28E+08
2.28E+08
2.28E+08
2.28E+08

2.28E+08
2.28E+08
2.28E+08
1.41E+08
269097
269097
267786
269866
269743
269392
269866
266130
266868
266868
266868
266868
57920626
57510721
1.47E+08
1.47E+08
1.25E+08
32481294
32481294
32481294
32481294
41875078
39580249
2.41E+08
2.41E+08
31195761
15087059
2.28E+08
2.28E+08
54787530
1.34E+08
1.34E+08
10841877
25513659
1.55E+08
1.55E+08
1.55E+08
1.55E+08
1.55E+08
1.55E+08
1.55E+08
1.55E+08
5643081

32449844
63708441
63708802
75496220
43172001
1.92E+08
2.2E+08
69814961
69814706
1.28E+08
1.28E+08
1.28E+08
1.28E+08
74528422
74531161
74531161
2.39E+08
2.39E+08
1.24E+08
77314215
47835361
47835361
2.31E+08
2.31E+08
2.31E+08
2.31E+08
2.31E+08
2.31E+08
2.31E+08
2.31E+08
92766521
92769566
61994655
49838999
58909456
1.62E+08
1.62E+08
1.16E+08
1.16E+08
29021582
1.11E+08
58912218
58912218
58912218
58915281
58912218
58914251

58912218
58914251
58914251
62164118
62166339
62164118
62167541
1.79E+08
1.79E+08
85527204
85527204
85525743
46147675
46151985
46147675
46147345
46146751
46146751
1.56E+08
59491111
10635343
1.7E+08
1.7E+08
1.7E+08
1.7E+08
59069438
25846053
25846053
57664137
57664137
57664137
57664137
57664137
77335185
15001413
39079510
39079510
39079510
57880870
9601181
1.16E+08
1.16E+08
17712065
17712065
17712065
17708360
17709273

1.1E+08
1.1E+08
1.1E+08
1.1E+08
1.1E+08
1.1E+08
1.71E+08
17176816
56139063
25382788
1.14E+08
1.14E+08
1.14E+08
1.14E+08
2.17E+08
2.17E+08
35120908
35116371
35120969
6638475
1.11E+08
76028840
81792016
1.63E+08
1.63E+08
2359110
1.68E+08
43966105
1.57E+08
1E+08
2.42E+08
2.42E+08
82078122
82078122
1.29E+08
45176723
45176723
1.3E+08
1.3E+08
41176994
3088579
1.02E+08
49563997
66935790
56901118
56901118
56901118

1.3E+08
29281754
5187557
5187557
5187557
5187557
2.24E+08
14321997
14321997
78507559
78507559
78507559
69964192
15271669
1.6E+08
1.7E+08
1.7E+08
1.7E+08
1.7E+08
1.7E+08
48424229
23730197
25689175
25689175
99635338
99635338
2E+08
2E+08
1.02E+08
1.02E+08
1.28E+08
48431249
48431249
48431249
48431249
48431249
48431249
57209345
13152138
87818575
62788414
1.79E+08
1.5E+08
21571808
21572173
21569095
26685621

1.99E+08
17723806
3494487
86566740
86566069
49081035
754228
40761072
78428877
78428877
78428877
78428877
78428877
49356252
78010397
25403986
8462677
8463037
13589693
13589693
10518367
70565401
540567
540567
540567
540567
539521
1.24E+08
1.24E+08
1.24E+08
1.24E+08
1.24E+08
1.24E+08
1.24E+08
1.24E+08
1.24E+08
27109087
1.03E+08
2.3E+08
2.3E+08
98989402
98989402
98989402
1.24E+08
32557626
32557626
32554231

32554231
32553714
32553141
65192334
1.32E+08
44464469
44464469
44464469
44464469
1.35E+08
1.35E+08
1.35E+08
41472181
16487582
35517458
35517609
2.19E+08
2.19E+08
1515971
33605162
33605162
33607446
33607860
1.15E+08
38668136
1.82E+08
1.82E+08
1.82E+08
1.1E+08
93787067
93786831
93783854
98111509
40054392
99763037
99760075
99760075
1.76E+08
1.76E+08
1.76E+08
27201296
20217695
20217695
44546003
44544633
44546003
44546003

40500829
40500782
40499799
65006292
65006292
1.58E+08
1.58E+08
1.1E+08
33764543
33765195
93348239
10478665
10479480
1.12E+08
79430335
79430335
2573509
2576502
2576502
75471611
1.53E+08
1.71E+08
1.71E+08
1.71E+08
1.71E+08
1.71E+08
1.71E+08
18855988
1.02E+08
99193767
99193767
65192268
65192268
72145929
75753193
1.01E+08
21584832
21481913
96535424
96535424
70097684
70093767
70097684
70093767
97985128
14537029
1.55E+08

1.55E+08
2.04E+08
2.04E+08
2.04E+08
2.04E+08
91492812
91492065
91492812
6734861
1.7E+08
1.7E+08
88744549
1.93E+08
74668871
74665895
74665895
74665895
74665895
74665895
12310446
78196199
78196199
78199199
78196199
78196199
30950614
33421348
1.79E+08
1.79E+08
1.79E+08
1.56E+08
1.56E+08
1.56E+08
1.56E+08
52590784
52590784
16762147
24107637
2886221
55702722
55705574
20017615
44342753
44686684
2.28E+08
31325866
31325866

31325866
31325866
31325866
31325866
31325866
1.05E+08
87913635
18554111
18554111
18554111
18553119
18553972
1.51E+08
1.51E+08
1.51E+08
66970346
66970346
66970373
66964439
66964439
1.79E+08
1.79E+08
1.79E+08
1.79E+08
7394942
7394942
7394942
7392061
1.02E+08
8268859
41523026
41519961
1.02E+08
44290409
32313136
32313136
93685735
1.08E+08
1.15E+08
6823559
6826142
1.22E+08
1.68E+08
1.28E+08
1.03E+08
1.03E+08
64581126

64581126
64581126
1.31E+08
1.03E+08
1.03E+08
1.03E+08
45820002
46192109
28968423
1.45E+08
1.45E+08
43734753
72211349
72211064
70534220
95117212
95117212
42714177
42714177
42714177
42714177
42714177
1.05E+08
17759494
17759494
28356216
28356216
51989995
1.44E+08
1.44E+08
1.44E+08
1.44E+08
1.4E+08
1.4E+08
1.05E+08
49214253
49214253
1.89E+08
98678266
80947506
86119520
11676241
29119508
42195754
42189634
27546235
34289750

34289750
34289750
1.25E+08
1.25E+08
1.2E+08
1.2E+08
58573416
35183838
35183838
56179187
46221485
46221485
21659603
21659603
21656698
7461998
6024574
6024574
6024574
6025150
6025150
21990542
21990542
21990542
1.86E+08
70825417
70822492
70822492
70822492
70822492
34974879
2.01E+08
2.01E+08
39263172
27114272
27113865
63835260
2.3E+08
2.3E+08
55841708
55841708
55841708
55841708
1.98E+08
20980868
20980868
20372724

20376385
20376385
62591620
1.03E+08
89380267
89857768
3531001
3531001
66207642
66207642
44671248
44671214
44671248
44671316
44671248
44671214
44671214
44671214
44671307
1.09E+08
1.09E+08
2.2E+08
2.2E+08
2.2E+08
2.2E+08
1.01E+08
1.6E+08
1.6E+08
57306802
57304268
57304268
57303914
33054780
33054780
29432464
24859183
24859183
24859183
24859183
24859551
1.13E+08
21825100
21825573
21825100
21825100
21825100
63810969

1.97E+08
1.97E+08
1.61E+08
1.61E+08
1.61E+08
1.61E+08
1.21E+08
1.03E+08
1.03E+08
1.03E+08
1.03E+08
44524162
1.61E+08
1.61E+08
1.61E+08
47170312
47170312
47170312
47173994
47173994
74113577
74113577
68146602
2.37E+08
2.37E+08
25024831
13045955
26206872
27445752
27445752
27448349
27445752
27448349
27448349
79526003
79526003
79526003
79526003
2.49E+08
2.49E+08
2.49E+08
3697614
3697614
3697614
1.39E+08
1.39E+08
67126067

67126067
67126067
67126067
67126067
49057428
66997131
66997131
58061469
54534545
54534545
54533819
54534545
1.77E+08
1.25E+08
1.25E+08
67658109
59617479
53023762
53023762
92790817
1.06E+08
45283125
45283125
2.03E+08
2.03E+08
1.5E+08
1.5E+08
88146760
88146760
88146420
88146760
88146760
1.83E+08
7745234
1.25E+08
12292528
12292528
49865561
49869310
1.1E+08
1.1E+08
9522059
72982938
72985186
72985186
1.02E+08
1.16E+08

2056243
2056243
2.12E+08
2.12E+08
2.12E+08
50733049
50735260
66555199
66558911
66558911
44681871
44681891
12891434
12891434
80999477
80999477
80999626
80999477
3485540
2.39E+08
96014641
39495228
39495228
39495228
39495228
27856745
27853514
27853862
27853514
27853514
27856898
27853862
18394652
18394652
2.02E+08
2.02E+08
2.02E+08
2.02E+08
2.02E+08
1.09E+08
78111808
78111808
78111808
38647307
47632205
47632205
36663947

36666870
36666870
4778679
59583345
1.23E+08
1.23E+08
99992135
53470350
48057506
48057506
48057506
48057506
48057506
48057506
48057506
48057506
44149264
1.6E+08
67710011
67710011
1.86E+08
1.86E+08
1.86E+08
1.86E+08
1.86E+08
24804124
96659143
1.68E+08
1.68E+08
1.41E+08
1.29E+08
1.29E+08
1.29E+08
4537031
4537031
4537031
4539847
8934700
8934700
8934700
8934700
8934738
8934738
21755741
21755741
21752371
86275229

42849353
42849353
42849353
75910418
93566208
93565961
93565961
86614114
1.21E+08
1.21E+08
94892824
94895709
69274788
79850665
5087451
5087451
5087552
1.05E+08
1.53E+08
1.53E+08
1.53E+08
1.53E+08
1.53E+08
1.53E+08
1.53E+08
1.06E+08
1.06E+08
1.06E+08
24670455
24670455
24670189
93034908
35669565
97374495
97374495
97374495
97374495
97374495
1.5E+08
1.5E+08
1.3E+08
50161822
50160098
50160098
50160098
1.5E+08
1.5E+08

49143295
45586252
20262013
20259199
20259199
20259199
57793852
65839823
86417964
8960667
8960667
8960667
39186023
39186023
1.29E+08
42297658
42297658
6039726
79073609
79073609
1.4E+08
1.4E+08
1.4E+08
2.41E+08
47605096
47605096
45807341
32932657
1.59E+08
1.59E+08
1.59E+08
1.59E+08
1.59E+08
19594607
19594607
19595514
59441511
1.13E+08
1.13E+08
1.13E+08
20939537
2673602
24646110
24646110
24646110
24646110
24646110

24649588
97189160
97189160
97189160
97189160
97189160
97189160
97189160
97189160
97190193
1.27E+08
1.12E+08
1.12E+08
1.12E+08
33572085
1.02E+08
52154679
52154679
57466179
57468865
57468357
57468357
57468357
57469218
57468357
57468357
1.24E+08
1.24E+08
2.49E+08
2.49E+08
1.31E+08
2.24E+08
2.24E+08
1.05E+08
2600605
2600605
2600605
2600605
2600605
2600392
62564486
62565243
25044286
2.17E+08
2.17E+08
10595365
7583003

40520603
40520603
40520632
40520603
66615350
66615350
78980723
78980723
1.14E+08
1.14E+08
1.14E+08
1.59E+08
1.59E+08
1.59E+08
1.53E+08
1.5E+08
31459348
81287219
81287219
81287219
46095354
1.38E+08
1.38E+08
1.38E+08
1.38E+08
1.38E+08
1.38E+08
68944030
37781995
1.85E+08
1.12E+08
1.12E+08
1.09E+08
1.09E+08
1.7E+08
1.7E+08
1.85E+08
1.85E+08
7441700
32607312
32607312
1.31E+08
1.31E+08
1.31E+08
6466259
6466259
6464781

1.84E+08
1.84E+08
1.84E+08
33162563
19137190
19137190
1.96E+08
1.96E+08
1.96E+08
1.96E+08
2.15E+08
1.93E+08
1.93E+08
1.93E+08
1.93E+08
1.93E+08
1.93E+08
1.93E+08
1.93E+08
1.93E+08
1.93E+08
1.93E+08
93281546
30643267
1.26E+08
1.26E+08
1.26E+08
1.26E+08
1.26E+08
1.26E+08
1.26E+08
1.26E+08
1.26E+08
1.26E+08
35858976
35858976
1.27E+08
42976805
42973938
56268392
56268392
1E+08
13413369
1.52E+08
49343760
49343760
83881770

52609639
80089790
53796779
24802083
89830401
89830401
1.84E+08
1.84E+08
1.84E+08
27748633
68230185
68230185
68230185
68230185
68230185
68230185
17567848
17567848
1.67E+08
52816746
52816746
52816746
1.28E+08
1.12E+08
1.12E+08
64938475
12401025
1.6E+08
1.6E+08
1.17E+08
1.25E+08
1.25E+08
1.25E+08
2.31E+08
1.79E+08
52260150
52260150
88974402
88974402
88974402
1.56E+08
1.56E+08
1.56E+08
1.56E+08
1.56E+08
1.56E+08
1.56E+08

1.56E+08
1.56E+08
1.56E+08
1.56E+08
1.56E+08
1.56E+08
1.56E+08
1.56E+08
1.56E+08
2897752
49520112
75768646
39537162
39537162
70028794
70028794
70027120
37460773
37460773
27701669
50356142
50358513
1.28E+08
1.28E+08
1.28E+08
27263014
27263014
46697301
47583594
23638997
1.44E+08
1.35E+08
1.35E+08
1.35E+08
1.35E+08
1.35E+08
1.35E+08
1.35E+08
1.35E+08
1.35E+08
29201560
29201560
43126047
74024938
74025088
96745092
48537080

48535276
48534358
1.31E+08
1.31E+08
1.31E+08
67665060

Supplementary Table 9 - SCC25 differential binding affinity between cetuximab and PBS.

seqnames	start	end	width	Fold	p.value	FDR	genename	promoterst
chr9	71814826	71815357	532	-1.13	8.54E-12	1.74E-08	TJP2	71815077
chr9	71814826	71815357	532	-1.13	8.54E-12	1.74E-08	TJP2	71814926
chr9	1.03E+08	1.03E+08	534	-0.91	1.79E-11	3.32E-08	MSANTD3	1.03E+08
chr9	1.03E+08	1.03E+08	534	-0.91	1.79E-11	3.32E-08	MSANTD3	1.03E+08
chr8	67579313	67579889	577	-0.91	5.73E-11	7.78E-08	C8orf44-SG	67574786
chr8	67579313	67579889	577	-0.91	5.73E-11	7.78E-08	C8orf44	67574786
chr8	67579313	67579889	577	-0.91	5.73E-11	7.78E-08	VCPIP1	67577452
chr6	1.19E+08	1.19E+08	612	-1.04	9.48E-11	1.05E-07	FAM184A	1.19E+08
chr6	1.19E+08	1.19E+08	612	-1.04	9.48E-11	1.05E-07	FAM184A	1.19E+08
chr5	41903966	41904640	675	-0.71	3.90E-10	2.77E-07	C5orf51	41899445
chr9	71785995	71786381	387	-0.97	3.95E-10	2.77E-07	TJP2	71783970
chr9	71785995	71786381	387	-0.97	3.95E-10	2.77E-07	TJP2	71783970
chr9	71785995	71786381	387	-0.97	3.95E-10	2.77E-07	TJP2	71783970
chr5	13944721	13945306	586	-0.9	6.19E-10	3.67E-07	DNAH5	13942589
chr9	74525539	74526774	1236	-0.68	1.11E-09	5.82E-07	C9orf85	74521422
chr9	74525539	74526774	1236	-0.68	1.11E-09	5.82E-07	ABHD17B	74524161
chr9	74525539	74526774	1236	-0.68	1.11E-09	5.82E-07	ABHD17B	74524161
chr3	1.46E+08	1.46E+08	866	-0.87	1.86E-09	8.14E-07	PLSCR1	1.46E+08
chr1	89458121	89458948	828	-0.61	2.46E-09	9.87E-07	RBMXL1	89456643
chr1	89458121	89458948	828	-0.61	2.46E-09	9.87E-07	CCBL2	89456643
chr1	89458121	89458948	828	-0.61	2.46E-09	9.87E-07	CCBL2	89456643
chr1	89458121	89458948	828	-0.61	2.46E-09	9.87E-07	RBMXL1	89456643
chr1	92409659	92410086	428	-0.94	2.78E-09	1.07E-06	BRDT	92409927
chr1	92409659	92410086	428	-0.94	2.78E-09	1.07E-06	BRDT	92409927
chr1	92409659	92410086	428	-0.94	2.78E-09	1.07E-06	BRDT	92409927
chr1	92409659	92410086	428	-0.94	2.78E-09	1.07E-06	BRDT	92409927
chr1	92409659	92410086	428	-0.94	2.78E-09	1.07E-06	BRDT	92409927
chr1	92409659	92410086	428	-0.94	2.78E-09	1.07E-06	BRDT	92409927
chr5	1.68E+08	1.68E+08	693	-0.93	3.22E-09	1.19E-06	PANK3	1.68E+08
chr9	1.04E+08	1.04E+08	825	-0.67	4.14E-09	1.44E-06	RNF20	1.04E+08
chr11	22646645	22647626	982	-0.75	5.14E-09	1.72E-06	FANCF	22645387
chr14	67880731	67881389	659	-0.94	5.26E-09	1.75E-06	PLEK2	67876917
chr3	1.13E+08	1.13E+08	398	-0.84	5.28E-09	1.75E-06	USF3	1.13E+08
chr3	1.13E+08	1.13E+08	398	-0.84	5.28E-09	1.75E-06	USF3	1.13E+08
chr7	1.12E+08	1.12E+08	611	-0.83	8.84E-09	2.58E-06	DOCK4	1.12E+08
chr7	1.12E+08	1.12E+08	611	-0.83	8.84E-09	2.58E-06	ZNF277	1.12E+08
chr18	32558930	32559490	561	-0.84	2.13E-08	4.70E-06	MAPRE2	32553207
chr4	1.01E+08	1.01E+08	611	-0.91	2.37E-08	5.15E-06	DAPP1	1.01E+08
chr4	1.01E+08	1.01E+08	611	-0.91	2.37E-08	5.15E-06	DAPP1	1.01E+08
chr20	40246819	40247342	524	-0.74	2.50E-08	5.32E-06	CHD6	40245133
chr1	71551971	71552398	428	-1.21	2.61E-08	5.47E-06	ZRANB2	71544972
chr1	71551971	71552398	428	-1.21	2.61E-08	5.47E-06	ZRANB2	71544972
chr9	1.08E+08	1.08E+08	1421	-0.66	2.98E-08	5.97E-06	SLC44A1	1.08E+08
chr9	1.08E+08	1.08E+08	1421	-0.66	2.98E-08	5.97E-06	SLC44A1	1.08E+08

chr14	24779070	24779496	427	-0.93	3.01E-08	6.00E-06	LTB4R2	24774356
chr14	24779070	24779496	427	-0.93	3.01E-08	6.00E-06	CIDEB	24778576
chr14	24779070	24779496	427	-0.93	3.01E-08	6.00E-06	LTB4R	24777312
chr14	24779070	24779496	427	-0.93	3.01E-08	6.00E-06	LTB4R	24775704
chr14	24779070	24779496	427	-0.93	3.01E-08	6.00E-06	LTB4R2	24774356
chr14	24779070	24779496	427	-0.93	3.01E-08	6.00E-06	CIDEB	24778583
chr1	51986038	51986446	409	-1.06	3.20E-08	6.29E-06	EPS15	51982995
chr10	1.13E+08	1.13E+08	1339	-0.63	3.39E-08	6.50E-06	SHOC2	1.13E+08
chr10	1.13E+08	1.13E+08	1339	-0.63	3.39E-08	6.50E-06	BBIP1	1.13E+08
chr10	1.13E+08	1.13E+08	1339	-0.63	3.39E-08	6.50E-06	BBIP1	1.13E+08
chr10	1.13E+08	1.13E+08	1339	-0.63	3.39E-08	6.50E-06	BBIP1	1.13E+08
chr10	1.13E+08	1.13E+08	1339	-0.63	3.39E-08	6.50E-06	BBIP1	1.13E+08
chr10	1.13E+08	1.13E+08	1339	-0.63	3.39E-08	6.50E-06	BBIP1	1.13E+08
chr1	1.65E+08	1.65E+08	562	-0.83	3.50E-08	6.61E-06	PBX1	1.65E+08
chr1	1.65E+08	1.65E+08	562	-0.83	3.50E-08	6.61E-06	PBX1	1.65E+08
chr1	1.65E+08	1.65E+08	562	-0.83	3.50E-08	6.61E-06	PBX1	1.65E+08
chr8	1.21E+08	1.21E+08	154	-1.5	3.61E-08	6.79E-06	DEPTOR	1.21E+08
chr8	1.21E+08	1.21E+08	154	-1.5	3.61E-08	6.79E-06	DEPTOR	1.21E+08
chr9	1.03E+08	1.03E+08	1581	-0.67	3.66E-08	6.83E-06	ERP44	1.03E+08
chr9	1.03E+08	1.03E+08	1581	-0.67	3.66E-08	6.83E-06	INVS	1.03E+08
chr9	1.03E+08	1.03E+08	1581	-0.67	3.66E-08	6.83E-06	INVS	1.03E+08
chr9	1.03E+08	1.03E+08	1581	-0.67	3.66E-08	6.83E-06	INVS	1.03E+08
chr9	1.03E+08	1.03E+08	1581	-0.67	3.66E-08	6.83E-06	INVS	1.03E+08
chr14	45430944	45431727	784	-0.74	4.10E-08	7.44E-06	FAM179B	45426392
chr14	45430944	45431727	784	-0.74	4.10E-08	7.44E-06	FAM179B	45426392
chr14	45430944	45431727	784	-0.74	4.10E-08	7.44E-06	FAM179B	45426392
chr14	45430944	45431727	784	-0.74	4.10E-08	7.44E-06	KLHL28	45429179
chr14	45430944	45431727	784	-0.74	4.10E-08	7.44E-06	KLHL28	45428335
chr20	6103531	6104372	842	-0.83	4.10E-08	7.44E-06	FERMT1	6102191
chr1	89828822	89829990	1169	-0.68	4.80E-08	8.39E-06	GBP6	89824435
chr7	34877327	34877826	500	-0.91	5.47E-08	9.21E-06	NPSR1-AS1	34871943
chr11	59382820	59383961	1142	-0.63	5.56E-08	9.30E-06	OSBP	59381617
chr9	1.08E+08	1.08E+08	874	-0.75	5.88E-08	9.71E-06	FSD1L	1.08E+08
chr9	1.08E+08	1.08E+08	874	-0.75	5.88E-08	9.71E-06	FSD1L	1.08E+08
chr9	1.08E+08	1.08E+08	874	-0.75	5.88E-08	9.71E-06	FSD1L	1.08E+08
chr9	1.08E+08	1.08E+08	874	-0.75	5.88E-08	9.71E-06	FSD1L	1.08E+08
chr9	80850691	80851619	929	-0.8	7.29E-08	1.12E-05	CEP78	80845990
chr9	80850691	80851619	929	-0.8	7.29E-08	1.12E-05	CEP78	80845990
chr12	96878937	96879304	368	-1.13	7.87E-08	1.18E-05	CFAP54	96878348
chr15	98503437	98504047	611	-0.78	7.98E-08	1.20E-05	ARRDC4	98498932
chr11	1.08E+08	1.08E+08	580	-0.78	8.05E-08	1.20E-05	EXPH5	1.08E+08
chr11	1.08E+08	1.08E+08	580	-0.78	8.05E-08	1.20E-05	EXPH5	1.08E+08
chr1	76262028	76263072	1045	-1.07	8.10E-08	1.21E-05	MSH4	76257555
chr17	58754641	58755421	781	-0.85	8.43E-08	1.25E-05	BCAS3	58750171
chr17	58754641	58755421	781	-0.85	8.43E-08	1.25E-05	BCAS3	58750171
chr14	20810877	20811842	966	-0.6	8.79E-08	1.28E-05	PARP2	20806772
chr14	20810877	20811842	966	-0.6	8.79E-08	1.28E-05	PARP2	20806772

chr14	20810877	20811842	966	-0.6	8.79E-08	1.28E-05	RPPH1	20809570
chr19	58892079	58892754	676	-0.92	9.04E-08	1.31E-05	ZNF837	58890389
chr19	58892079	58892754	676	-0.92	9.04E-08	1.31E-05	ZNF837	58890389
chr2	1.97E+08	1.97E+08	556	-1.32	9.22E-08	1.32E-05	LOC101927	1.97E+08
chr2	1.97E+08	1.97E+08	556	-1.32	9.22E-08	1.32E-05	LOC101927	1.97E+08
chr15	52945244	52945960	717	-1.11	9.84E-08	1.39E-05	FAM214A	52942247
chr8	1.19E+08	1.19E+08	661	-0.82	1.09E-07	1.49E-05	MED30	1.19E+08
chr8	1.19E+08	1.19E+08	661	-0.82	1.09E-07	1.49E-05	MED30	1.19E+08
chr12	30947927	30948662	736	-0.8	1.15E-07	1.55E-05	LINC00941	30943614
chr7	30544035	30544825	791	-0.75	1.16E-07	1.56E-05	GGCT	30542457
chr7	30544035	30544825	791	-0.75	1.16E-07	1.56E-05	GGCT	30542457
chr7	30544035	30544825	791	-0.75	1.16E-07	1.56E-05	GGCT	30542457
chr7	30544035	30544825	791	-0.75	1.16E-07	1.56E-05	GGCT	30542457
chr7	30544035	30544825	791	-0.75	1.16E-07	1.56E-05	GGCT	30542457
chr9	72374331	72375204	874	-0.74	1.27E-07	1.65E-05	PTAR1	72372876
chr6	1.17E+08	1.17E+08	748	-0.74	1.29E-07	1.68E-05	RWDD1	1.17E+08
chr6	1.17E+08	1.17E+08	748	-0.74	1.29E-07	1.68E-05	RWDD1	1.17E+08
chr6	1.17E+08	1.17E+08	748	-0.74	1.29E-07	1.68E-05	RWDD1	1.17E+08
chr14	81999987	82000577	591	-0.6	1.31E-07	1.69E-05	SEL1L	81998205
chr14	81999987	82000577	591	-0.6	1.31E-07	1.69E-05	SEL1L	81998205
chr8	1.1E+08	1.1E+08	824	-0.65	1.31E-07	1.69E-05	TMEM74	1.1E+08
chr15	49103154	49103906	753	-0.73	1.36E-07	1.74E-05	CEP152	49101343
chr15	49103154	49103906	753	-0.73	1.36E-07	1.74E-05	CEP152	49101343
chr10	1.06E+08	1.06E+08	483	-0.81	1.42E-07	1.80E-05	ITPRIP	1.06E+08
chr14	24583199	24584514	1316	-0.6	1.45E-07	1.83E-05	DCAF11	24578905
chr14	24583199	24584514	1316	-0.6	1.45E-07	1.83E-05	DCAF11	24578985
chr14	24583199	24584514	1316	-0.6	1.45E-07	1.83E-05	DCAF11	24579491
chr14	24583199	24584514	1316	-0.6	1.45E-07	1.83E-05	DCAF11	24578985
chr14	24583199	24584514	1316	-0.6	1.45E-07	1.83E-05	DCAF11	24579493
chr2	17994303	17994609	307	-1.32	1.51E-07	1.88E-05	MSGN1	17992785
chr7	1.38E+08	1.38E+08	507	-0.86	1.57E-07	1.93E-05	CREB3L2	1.38E+08
chr7	1.38E+08	1.38E+08	507	-0.86	1.57E-07	1.93E-05	CREB3L2	1.38E+08
chr13	96187485	96187875	391	-0.98	1.70E-07	2.05E-05	CLDN10-AS	96184164
chr7	1.15E+08	1.15E+08	666	-0.89	1.74E-07	2.10E-05	LINC01392	1.15E+08
chr16	48645604	48646029	426	-0.96	1.95E-07	2.29E-05	N4BP1	48642120
chr17	19312975	19313202	228	-1.87	1.99E-07	2.33E-05	RNF112	19309490
chr20	39798384	39798720	337	-1.08	2.05E-07	2.37E-05	MIR6871	39792662
chr14	73925115	73925605	491	-0.84	2.23E-07	2.52E-05	NUMB	73923286
chr14	73925115	73925605	491	-0.84	2.23E-07	2.52E-05	NUMB	73923286
chr14	73925115	73925605	491	-0.84	2.23E-07	2.52E-05	NUMB	73923286
chr14	73925115	73925605	491	-0.84	2.23E-07	2.52E-05	NUMB	73923286
chr15	75744036	75744664	629	-0.7	2.23E-07	2.52E-05	SIN3A	75742087
chr15	75744036	75744664	629	-0.7	2.23E-07	2.52E-05	SIN3A	75741926
chr9	85881551	85882016	466	-1.26	2.23E-07	2.52E-05	FRMD3	85880202
chr9	86535643	86536628	986	-0.69	2.64E-07	2.81E-05	KIF27	86534380
chr9	86535643	86536628	986	-0.69	2.64E-07	2.81E-05	KIF27	86534380
chr9	86535643	86536628	986	-0.69	2.64E-07	2.81E-05	KIF27	86534380

chr9	1.04E+08	1.04E+08	580	-0.59	2.82E-07	2.94E-05	ZNF189	1.04E+08
chr9	1.04E+08	1.04E+08	580	-0.59	2.82E-07	2.94E-05	ZNF189	1.04E+08
chr9	1.04E+08	1.04E+08	580	-0.59	2.82E-07	2.94E-05	ZNF189	1.04E+08
chr9	1.04E+08	1.04E+08	580	-0.59	2.82E-07	2.94E-05	ZNF189	1.04E+08
chr9	1.04E+08	1.04E+08	580	-0.59	2.82E-07	2.94E-05	ZNF189	1.04E+08
chr9	1.04E+08	1.04E+08	580	-0.59	2.82E-07	2.94E-05	ZNF189	1.04E+08
chr9	1.04E+08	1.04E+08	580	-0.59	2.82E-07	2.94E-05	MRPL50	1.04E+08
chr15	77223765	77224292	528	-0.84	2.91E-07	3.01E-05	RCN2	77218961
chr15	77223765	77224292	528	-0.84	2.91E-07	3.01E-05	RCN2	77218961
chr9	79791907	79792682	776	-0.6	3.06E-07	3.12E-05	VPS13A	79787360
chr9	79791907	79792682	776	-0.6	3.06E-07	3.12E-05	VPS13A	79787360
chr9	79791907	79792682	776	-0.6	3.06E-07	3.12E-05	VPS13A	79787360
chr9	79791907	79792682	776	-0.6	3.06E-07	3.12E-05	VPS13A	79787360
chr9	79791907	79792682	776	-0.6	3.06E-07	3.12E-05	VPS13A-AS	79790833
chr4	52708934	52709535	602	-0.67	3.16E-07	3.19E-05	DCUN1D4	52704744
chr4	52708934	52709535	602	-0.67	3.16E-07	3.19E-05	DCUN1D4	52704165
chr4	52708934	52709535	602	-0.67	3.16E-07	3.19E-05	DCUN1D4	52704165
chr4	52708934	52709535	602	-0.67	3.16E-07	3.19E-05	DCUN1D4	52704165
chr16	53537027	53537720	694	-0.63	3.20E-07	3.22E-05	AKTIP	53535216
chr16	53537027	53537720	694	-0.63	3.20E-07	3.22E-05	AKTIP	53535216
chr16	53537027	53537720	694	-0.63	3.20E-07	3.22E-05	AKTIP	53535216
chr6	80816028	80816908	881	-0.67	3.30E-07	3.31E-05	BCKDHB	80811326
chr6	80816028	80816908	881	-0.67	3.30E-07	3.31E-05	BCKDHB	80811326
chr6	80816028	80816908	881	-0.67	3.30E-07	3.31E-05	BCKDHB	80811326
chr6	80816028	80816908	881	-0.67	3.30E-07	3.31E-05	BCKDHB	80811326
chr3	1.79E+08	1.79E+08	1178	-0.59	3.42E-07	3.38E-05	ACTL6A	1.79E+08
chr3	1.79E+08	1.79E+08	1178	-0.59	3.42E-07	3.38E-05	ACTL6A	1.79E+08
chr3	1.79E+08	1.79E+08	1178	-0.59	3.42E-07	3.38E-05	ACTL6A	1.79E+08
chr9	88896965	88898163	1199	-0.65	3.54E-07	3.46E-05	ISCA1	88895490
chr10	28591654	28592335	682	-0.71	3.55E-07	3.46E-05	MPP7	28589995
chr10	28591654	28592335	682	-0.71	3.55E-07	3.46E-05	MPP7	28589995
chr10	28591654	28592335	682	-0.71	3.55E-07	3.46E-05	MPP7	28589995
chr1	63156058	63156432	375	-1.36	3.65E-07	3.52E-05	DOCK7	63152039
chr1	63156058	63156432	375	-1.36	3.65E-07	3.52E-05	DOCK7	63152039
chr1	63156058	63156432	375	-1.36	3.65E-07	3.52E-05	DOCK7	63152039
chr1	63156058	63156432	375	-1.36	3.65E-07	3.52E-05	DOCK7	63152039
chr1	63156058	63156432	375	-1.36	3.65E-07	3.52E-05	DOCK7	63152039
chr9	4792569	4793352	784	-0.8	3.66E-07	3.52E-05	RCL1	4787833
chr9	4792569	4793352	784	-0.8	3.66E-07	3.52E-05	RCL1	4787833
chr9	4792569	4793352	784	-0.8	3.66E-07	3.52E-05	RCL1	4787833
chr3	24565793	24566185	393	-1.16	3.72E-07	3.56E-05	MIR4792	24560926
chr3	1.15E+08	1.15E+08	946	-0.56	4.33E-07	3.93E-05	ZBTB20	1.15E+08
chr11	27489816	27490486	671	-0.98	4.54E-07	4.08E-05	LOC10537(	27488277
chr11	27489816	27490486	671	-0.98	4.54E-07	4.08E-05	LOC10537(	27488277
chr11	6340980	6342370	1391	-0.66	4.56E-07	4.08E-05	PRKCDBP	6339740
chr14	51561691	51562366	676	-0.76	5.42E-07	4.70E-05	TRIM9	51560422
chr14	51561691	51562366	676	-0.76	5.42E-07	4.70E-05	TRIM9	51560422

chr17	56595060	56596185	1126	-0.74	5.49E-07	4.73E-05	SEPT4-AS1	56592197
chr17	56595060	56596185	1126	-0.74	5.49E-07	4.73E-05	MTMR4	56593251
chr1	1.72E+08	1.72E+08	708	-0.55	5.62E-07	4.79E-05	METTL13	1.72E+08
chr1	1.72E+08	1.72E+08	708	-0.55	5.62E-07	4.79E-05	METTL13	1.72E+08
chr1	1.72E+08	1.72E+08	708	-0.55	5.62E-07	4.79E-05	METTL13	1.72E+08
chr10	91461039	91461659	621	-0.82	5.71E-07	4.83E-05	KIF20B	91456346
chr10	91461039	91461659	621	-0.82	5.71E-07	4.83E-05	KIF20B	91456346
chr10	91461039	91461659	621	-0.82	5.71E-07	4.83E-05	FLJ37201	91455685
chr13	98085153	98086395	1243	-0.58	5.80E-07	4.89E-05	RAP2A	98081474
chr3	1.67E+08	1.67E+08	1396	-0.53	5.97E-07	4.99E-05	SERPINI1	1.67E+08
chr3	1.67E+08	1.67E+08	1396	-0.53	5.97E-07	4.99E-05	SERPINI1	1.67E+08
chr3	1.67E+08	1.67E+08	1396	-0.53	5.97E-07	4.99E-05	PDCD10	1.67E+08
chr3	1.67E+08	1.67E+08	1396	-0.53	5.97E-07	4.99E-05	PDCD10	1.67E+08
chr3	1.67E+08	1.67E+08	1396	-0.53	5.97E-07	4.99E-05	PDCD10	1.67E+08
chr11	1.08E+08	1.08E+08	834	-0.63	6.10E-07	5.07E-05	ATM	1.08E+08
chr11	1.08E+08	1.08E+08	834	-0.63	6.10E-07	5.07E-05	NPAT	1.08E+08
chr7	12726203	12727297	1095	-0.64	6.38E-07	5.24E-05	ARL4A	12721910
chr7	12726203	12727297	1095	-0.64	6.38E-07	5.24E-05	ARL4A	12721910
chr7	12726203	12727297	1095	-0.64	6.38E-07	5.24E-05	ARL4A	12721451
chr7	12726203	12727297	1095	-0.64	6.38E-07	5.24E-05	ARL4A	12721451
chr5	35856754	35857278	525	-0.96	6.77E-07	5.45E-05	IL7R	35851976
chr5	35856754	35857278	525	-0.96	6.77E-07	5.45E-05	IL7R	35851976
chr19	55850525	55851272	748	-0.7	6.91E-07	5.53E-05	KMT5C	55846220
chr1	1.74E+08	1.74E+08	523	-1.05	7.01E-07	5.59E-05	SNORD47	1.74E+08
chr1	1.74E+08	1.74E+08	523	-1.05	7.01E-07	5.59E-05	SNORD81	1.74E+08
chr1	1.74E+08	1.74E+08	523	-1.05	7.01E-07	5.59E-05	GAS5-AS1	1.74E+08
chr1	89487487	89487742	256	-1.08	7.10E-07	5.63E-05	GBP3	89486549
chr11	1.06E+08	1.06E+08	565	-0.66	7.18E-07	5.67E-05	MSANTD4	1.06E+08
chr11	1.06E+08	1.06E+08	565	-0.66	7.18E-07	5.67E-05	MSANTD4	1.06E+08
chr11	1.06E+08	1.06E+08	565	-0.66	7.18E-07	5.67E-05	MSANTD4	1.06E+08
chr11	1.06E+08	1.06E+08	565	-0.66	7.18E-07	5.67E-05	MSANTD4	1.06E+08
chr11	1.06E+08	1.06E+08	565	-0.66	7.18E-07	5.67E-05	MSANTD4	1.06E+08
chr9	88358157	88358739	583	-0.72	7.33E-07	5.74E-05	AGTPBP1	88354944
chr9	88358157	88358739	583	-0.72	7.33E-07	5.74E-05	AGTPBP1	88354944
chr9	88358157	88358739	583	-0.72	7.33E-07	5.74E-05	AGTPBP1	88354944
chr11	31832597	31833063	467	-0.71	7.36E-07	5.75E-05	PAX6	31830901
chr11	31832597	31833063	467	-0.71	7.36E-07	5.75E-05	PAX6	31830901
chr11	31832597	31833063	467	-0.71	7.36E-07	5.75E-05	PAX6	31830901
chr11	31832597	31833063	467	-0.71	7.36E-07	5.75E-05	PAX6	31831731
chr11	31832597	31833063	467	-0.71	7.36E-07	5.75E-05	PAX6	31830690
chr11	31832597	31833063	467	-0.71	7.36E-07	5.75E-05	PAX6	31831890
chr11	31832597	31833063	467	-0.71	7.36E-07	5.75E-05	PAX6	31826473
chr3	1.56E+08	1.56E+08	631	-0.74	7.43E-07	5.77E-05	SSR3	1.56E+08
chr3	1.56E+08	1.56E+08	631	-0.74	7.43E-07	5.77E-05	SSR3	1.56E+08
chr3	1.56E+08	1.56E+08	631	-0.74	7.43E-07	5.77E-05	SSR3	1.56E+08
chr3	1.56E+08	1.56E+08	631	-0.74	7.43E-07	5.77E-05	SSR3	1.56E+08
chr6	75915499	75916036	538	-0.86	7.84E-07	6.00E-05	COL12A1	75913623

chr6	75915499	75916036	538	-0.86	7.84E-07	6.00E-05	COL12A1	75913623
chr14	24733317	24734056	740	-0.77	7.92E-07	6.05E-05	TGM1	24730416
chr1	3773604	3774366	763	-0.65	8.04E-07	6.08E-05	DFFB	3768830
chr1	3773604	3774366	763	-0.65	8.04E-07	6.08E-05	DFFB	3768830
chr1	3773604	3774366	763	-0.65	8.04E-07	6.08E-05	DFFB	3768830
chr1	3773604	3774366	763	-0.65	8.04E-07	6.08E-05	CEP104	3771797
chr14	53019043	53019621	579	-0.68	8.18E-07	6.15E-05	TXNDC16	53017301
chr14	53019043	53019621	579	-0.68	8.18E-07	6.15E-05	GPR137C	53014865
chr14	53019043	53019621	579	-0.68	8.18E-07	6.15E-05	TXNDC16	53017301
chr10	76585756	76586647	892	-0.61	9.07E-07	6.67E-05	KAT6B	76581170
chr10	76585756	76586647	892	-0.61	9.07E-07	6.67E-05	KAT6B	76581170
chr10	76585756	76586647	892	-0.61	9.07E-07	6.67E-05	KAT6B	76581170
chr5	10479228	10479878	651	-0.72	9.22E-07	6.74E-05	MIR6131	10473148
chr3	1.68E+08	1.68E+08	1809	-0.52	9.68E-07	6.96E-05	GOLIM4	1.68E+08
chr3	1.68E+08	1.68E+08	1809	-0.52	9.68E-07	6.96E-05	GOLIM4	1.68E+08
chr14	81407733	81408366	634	-0.63	9.82E-07	7.01E-05	CEP128	81403884
chr1	1.01E+08	1.01E+08	552	-0.65	9.93E-07	7.03E-05	CDC14A	1.01E+08
chr1	1.01E+08	1.01E+08	552	-0.65	9.93E-07	7.03E-05	CDC14A	1.01E+08
chr1	1.01E+08	1.01E+08	552	-0.65	9.93E-07	7.03E-05	CDC14A	1.01E+08
chr11	71791396	71792150	755	-0.56	9.95E-07	7.03E-05	LRTOMT	71786376
chr11	71791396	71792150	755	-0.56	9.95E-07	7.03E-05	LRTOMT	71786376
chr11	71791396	71792150	755	-0.56	9.95E-07	7.03E-05	NUMA1	71789739
chr11	71791396	71792150	755	-0.56	9.95E-07	7.03E-05	LRTOMT	71786376
chr11	71791396	71792150	755	-0.56	9.95E-07	7.03E-05	LRTOMT	71786376
chr11	71791396	71792150	755	-0.56	9.95E-07	7.03E-05	LRTOMT	71786376
chr11	71791396	71792150	755	-0.56	9.95E-07	7.03E-05	LRTOMT	71786376
chr11	71791396	71792150	755	-0.56	9.95E-07	7.03E-05	LRTOMT	71786376
chr11	71791396	71792150	755	-0.56	9.95E-07	7.03E-05	LRTOMT	71786376
chr11	71791396	71792150	755	-0.56	9.95E-07	7.03E-05	NUMA1	71789739
chr11	71791396	71792150	755	-0.56	9.95E-07	7.03E-05	NUMA1	71789520
chr11	71791396	71792150	755	-0.56	9.95E-07	7.03E-05	LRTOMT	71786376
chr11	71791396	71792150	755	-0.56	9.95E-07	7.03E-05	LRTOMT	71786376
chr16	50775527	50776091	565	-0.65	1.12E-06	7.65E-05	CYLD	50771028
chr16	50775527	50776091	565	-0.65	1.12E-06	7.65E-05	CYLD	50770960
chr16	50775527	50776091	565	-0.65	1.12E-06	7.65E-05	CYLD	50770960
chr16	50775527	50776091	565	-0.65	1.12E-06	7.65E-05	MIR3181	50771215
chr10	1.06E+08	1.06E+08	477	-0.8	1.16E-06	7.83E-05	ITPRIP	1.06E+08
chr15	42162847	42163207	361	-1.27	1.19E-06	7.98E-05	MIR4310	42156749
chr19	32595366	32596107	742	-0.8	1.19E-06	8.01E-05	LOC101927	32594822
chr16	85785700	85786077	378	-1.29	1.20E-06	8.04E-05	C16orf74	85782689
chr3	1.61E+08	1.61E+08	353	-1.13	1.21E-06	8.09E-05	B3GALNT1	1.61E+08
chr3	1.61E+08	1.61E+08	353	-1.13	1.21E-06	8.09E-05	B3GALNT1	1.61E+08
chr3	1.61E+08	1.61E+08	353	-1.13	1.21E-06	8.09E-05	B3GALNT1	1.61E+08
chr3	1.61E+08	1.61E+08	353	-1.13	1.21E-06	8.09E-05	B3GALNT1	1.61E+08
chr3	1.61E+08	1.61E+08	353	-1.13	1.21E-06	8.09E-05	B3GALNT1	1.61E+08
chr8	67036152	67036848	697	-0.72	1.24E-06	8.20E-05	TRIM55	67034277
chr8	67036152	67036848	697	-0.72	1.24E-06	8.20E-05	TRIM55	67034277

chr8	67036152	67036848	697	-0.72	1.24E-06	8.20E-05	TRIM55	67034277
chr8	67036152	67036848	697	-0.72	1.24E-06	8.20E-05	TRIM55	67034277
chr14	80682073	80682433	361	-0.91	1.26E-06	8.25E-05	DIO2	80675970
chr14	80682073	80682433	361	-0.91	1.26E-06	8.25E-05	DIO2	80675864
chr14	80682073	80682433	361	-0.91	1.26E-06	8.25E-05	DIO2	80675864
chr14	80682073	80682433	361	-0.91	1.26E-06	8.25E-05	DIO2	80675864
chr6	47755803	47756288	486	-0.86	1.26E-06	8.25E-05	OPN5	47749803
chr11	58912135	58912528	394	-0.88	1.30E-06	8.45E-05	FAM111A	58905218
chr11	58912135	58912528	394	-0.88	1.30E-06	8.45E-05	FAM111A	58905218
chr11	58912135	58912528	394	-0.88	1.30E-06	8.45E-05	FAM111A	58905218
chr11	58912135	58912528	394	-0.88	1.30E-06	8.45E-05	LOC101927	58908281
chr11	58912135	58912528	394	-0.88	1.30E-06	8.45E-05	FAM111A	58905218
chr11	58912135	58912528	394	-0.88	1.30E-06	8.45E-05	FAM111A	58907251
chr11	58912135	58912528	394	-0.88	1.30E-06	8.45E-05	FAM111A	58905218
chr11	58912135	58912528	394	-0.88	1.30E-06	8.45E-05	FAM111A	58907251
chr11	58912135	58912528	394	-0.88	1.30E-06	8.45E-05	FAM111A	58907251
chr16	55702306	55703262	957	-0.78	1.30E-06	8.49E-05	SLC6A2	55700582
chr5	1.42E+08	1.42E+08	483	-0.95	1.32E-06	8.55E-05	FGF1	1.42E+08
chr3	1.11E+08	1.11E+08	341	-0.96	1.33E-06	8.59E-05	PLCXD2-AS	1.11E+08
chr10	61668289	61668434	146	-1.51	1.35E-06	8.68E-05	CCDC6	61664414
chr11	6704354	6704946	593	-0.76	1.38E-06	8.79E-05	MRPL17	6702632
chr8	67340925	67341329	405	-0.79	1.44E-06	9.06E-05	ADHFE1	67339717
chr8	67340925	67341329	405	-0.79	1.44E-06	9.06E-05	RRS1	67336262
chr8	67340925	67341329	405	-0.79	1.44E-06	9.06E-05	RRS1-AS1	67339212
chr12	41223457	41223878	422	-1.05	1.45E-06	9.10E-05	CNTN1	41216739
chr16	25122727	25123240	514	-0.69	1.50E-06	9.35E-05	LCMT1	25118046
chr16	25122727	25123240	514	-0.69	1.50E-06	9.35E-05	LCMT1	25118046
chr16	25122727	25123240	514	-0.69	1.50E-06	9.35E-05	LCMT1-AS1	25120876
chr14	24911230	24912247	1018	-0.64	1.51E-06	9.39E-05	SDR39U1	24910009
chr14	24911230	24912247	1018	-0.64	1.51E-06	9.39E-05	SDR39U1	24910007
chr14	24911230	24912247	1018	-0.64	1.51E-06	9.39E-05	SDR39U1	24910111
chr14	24911230	24912247	1018	-0.64	1.51E-06	9.39E-05	SDR39U1	24910009
chr14	24911230	24912247	1018	-0.64	1.51E-06	9.39E-05	LOC101927	24907158
chr7	20369870	20370783	914	-0.87	1.57E-06	9.63E-05	ITGB8	20365724
chr7	20369870	20370783	914	-0.87	1.57E-06	9.63E-05	LOC101927	20369385
chr7	20369870	20370783	914	-0.87	1.57E-06	9.63E-05	LOC101927	20369385
chr10	22535397	22536177	781	-0.88	1.58E-06	9.66E-05	LOC100130	22536000
chr19	12847642	12848309	668	-0.68	1.66E-06	1.00E-04	C19orf43	12843529
chr19	12847642	12848309	668	-0.68	1.66E-06	1.00E-04	ASNA1	12843305
chr3	98449237	98449437	201	-1.71	1.67E-06	1.00E-04	ST3GAL6	98446547
chr3	98449237	98449437	201	-1.71	1.67E-06	1.00E-04	ST3GAL6	98446079
chr3	98449237	98449437	201	-1.71	1.67E-06	1.00E-04	ST3GAL6	98446079
chr3	98449237	98449437	201	-1.71	1.67E-06	1.00E-04	ST3GAL6	98446079
chr3	98449237	98449437	201	-1.71	1.67E-06	1.00E-04	ST3GAL6	98446547
chr3	98449237	98449437	201	-1.71	1.67E-06	1.00E-04	ST3GAL6	98446079
chr14	24422759	24423462	704	-0.86	1.69E-06	0.000101	DHRS4	24417943
chr14	24422759	24423462	704	-0.86	1.69E-06	0.000101	DHRS4	24417943

chr14	24422759	24423462	704	-0.86	1.69E-06	0.000101	DHRS4	24417943
chr14	24422759	24423462	704	-0.86	1.69E-06	0.000101	DHRS4	24417943
chr14	24422759	24423462	704	-0.86	1.69E-06	0.000101	DHRS4	24417943
chr14	24422759	24423462	704	-0.86	1.69E-06	0.000101	DHRS4	24417943
chr14	24422759	24423462	704	-0.86	1.69E-06	0.000101	DHRS4-AS1	24421406
chr14	24422759	24423462	704	-0.86	1.69E-06	0.000101	DHRS4-AS1	24421406
chr14	24422759	24423462	704	-0.86	1.69E-06	0.000101	DHRS4-AS1	24422321
chr14	24422759	24423462	704	-0.86	1.69E-06	0.000101	DHRS4-AS1	24421406
chr8	56685044	56686392	1349	-0.63	1.70E-06	0.000102	TMEM68	56683966
chr8	56685044	56686392	1349	-0.63	1.70E-06	0.000102	TMEM68	56683966
chr8	56685044	56686392	1349	-0.63	1.70E-06	0.000102	TMEM68	56683966
chr8	56685044	56686392	1349	-0.63	1.70E-06	0.000102	TGS1	56680790
chr8	56685044	56686392	1349	-0.63	1.70E-06	0.000102	TGS1	56680790
chr12	1.15E+08	1.15E+08	895	-0.65	1.70E-06	0.000102	TBX3	1.15E+08
chr12	1.15E+08	1.15E+08	895	-0.65	1.70E-06	0.000102	TBX3	1.15E+08
chr10	1.34E+08	1.34E+08	427	-0.85	1.70E-06	0.000102	INPP5A	1.34E+08
chr5	1.48E+08	1.48E+08	1139	-0.8	1.73E-06	0.000103	SH3TC2	1.48E+08
chr5	1.48E+08	1.48E+08	1139	-0.8	1.73E-06	0.000103	LOC255187	1.48E+08
chr9	80911706	80912603	898	-0.61	1.73E-06	0.000103	PSAT1	80906990
chr9	80911706	80912603	898	-0.61	1.73E-06	0.000103	PSAT1	80906990
chr9	73033146	73035939	2794	-0.53	1.74E-06	0.000103	KLF9	73027573
chr6	1.18E+08	1.18E+08	834	-0.76	1.75E-06	0.000103	GOPC	1.18E+08
chr6	1.18E+08	1.18E+08	834	-0.76	1.75E-06	0.000103	GOPC	1.18E+08
chr5	1.18E+08	1.18E+08	965	-0.67	1.79E-06	0.000105	DMXL1	1.18E+08
chr5	1.18E+08	1.18E+08	965	-0.67	1.79E-06	0.000105	DMXL1	1.18E+08
chr5	1.18E+08	1.18E+08	965	-0.67	1.79E-06	0.000105	DMXL1	1.18E+08
chr5	1.18E+08	1.18E+08	965	-0.67	1.79E-06	0.000105	LOC10537	1.18E+08
chr10	16859231	16859928	698	-0.59	1.80E-06	0.000105	RSU1	16857453
chr10	16859231	16859928	698	-0.59	1.80E-06	0.000105	RSU1	16857453
chr18	6836365	6836736	372	-1.4	1.82E-06	0.000106	ARHGAP28	6829431
chr2	1.03E+08	1.03E+08	397	-1.37	1.89E-06	0.000109	IL1RL1	1.03E+08
chr2	1.03E+08	1.03E+08	397	-1.37	1.89E-06	0.000109	IL1RL1	1.03E+08
chr6	1.52E+08	1.52E+08	617	-0.72	1.91E-06	0.000109	ARMT1	1.52E+08
chr6	1.52E+08	1.52E+08	617	-0.72	1.91E-06	0.000109	ARMT1	1.52E+08
chr6	1.52E+08	1.52E+08	617	-0.72	1.91E-06	0.000109	RMND1	1.52E+08
chr6	1.52E+08	1.52E+08	617	-0.72	1.91E-06	0.000109	RMND1	1.52E+08
chr8	96280833	96282400	1568	-0.72	1.94E-06	0.00011	C8orf37-AS	96276063
chr8	96280833	96282400	1568	-0.72	1.94E-06	0.00011	C8orf37-AS	96276063
chr8	96280833	96282400	1568	-0.72	1.94E-06	0.00011	C8orf37-AS	96276063
chr8	96280833	96282400	1568	-0.72	1.94E-06	0.00011	C8orf37-AS	96276063
chr8	96280833	96282400	1568	-0.72	1.94E-06	0.00011	C8orf37-AS	96276063
chr8	96280833	96282400	1568	-0.72	1.94E-06	0.00011	C8orf37-AS	96276063
chr8	96280833	96282400	1568	-0.72	1.94E-06	0.00011	C8orf37-AS	96276063
chr8	96280833	96282400	1568	-0.72	1.94E-06	0.00011	C8orf37-AS	96276063
chr8	96280833	96282400	1568	-0.72	1.94E-06	0.00011	C8orf37-AS	96276063
chr8	96280833	96282400	1568	-0.72	1.94E-06	0.00011	C8orf37	96279462
chr8	1.24E+08	1.24E+08	451	-0.9	1.96E-06	0.000111	FAM83A	1.24E+08

chr8	1.24E+08	1.24E+08	451	-0.9	1.96E-06	0.000111	FAM83A	1.24E+08
chr7	1.23E+08	1.23E+08	887	-0.81	1.99E-06	0.000112	IQUB	1.23E+08
chr7	1.23E+08	1.23E+08	887	-0.81	1.99E-06	0.000112	IQUB	1.23E+08
chr7	1.23E+08	1.23E+08	887	-0.81	1.99E-06	0.000112	IQUB	1.23E+08
chr7	1.23E+08	1.23E+08	887	-0.81	1.99E-06	0.000112	IQUB	1.23E+08
chr13	96328789	96329781	993	-0.74	2.03E-06	0.000114	DNAJC3	96324392
chr13	96328789	96329781	993	-0.74	2.03E-06	0.000114	DNAJC3-AS	96327179
chr3	1.58E+08	1.58E+08	718	-0.8	2.07E-06	0.000115	RSRC1	1.58E+08
chr3	1.58E+08	1.58E+08	718	-0.8	2.07E-06	0.000115	RSRC1	1.58E+08
chr3	1.58E+08	1.58E+08	718	-0.8	2.07E-06	0.000115	RSRC1	1.58E+08
chr3	1.58E+08	1.58E+08	718	-0.8	2.07E-06	0.000115	SHOX2	1.58E+08
chr3	1.58E+08	1.58E+08	718	-0.8	2.07E-06	0.000115	SHOX2	1.58E+08
chr3	1.58E+08	1.58E+08	718	-0.8	2.07E-06	0.000115	SHOX2	1.58E+08
chr9	1.24E+08	1.24E+08	719	-0.74	2.07E-06	0.000115	GSN-AS1	1.24E+08
chr9	1.24E+08	1.24E+08	719	-0.74	2.07E-06	0.000115	GSN	1.24E+08
chr9	1.24E+08	1.24E+08	719	-0.74	2.07E-06	0.000115	GSN	1.24E+08
chr20	7327141	7327643	503	-1.03	2.17E-06	0.000119	LOC10192‹	7323079
chr12	1.26E+08	1.26E+08	1319	-0.63	2.20E-06	0.00012	LINC00939	1.26E+08
chr8	70378785	70379173	389	-1	2.21E-06	0.00012	SULF1	70373858
chr8	70378785	70379173	389	-1	2.21E-06	0.00012	SULF1	70373858
chr20	56268052	56268584	533	-0.73	2.21E-06	0.00012	PMEPA1	56263680
chr7	7221880	7222961	1082	-0.65	2.32E-06	0.000125	C1GALT1	7217245
chr4	1.04E+08	1.04E+08	1556	-0.62	2.34E-06	0.000126	UBE2D3	1.04E+08
chr4	1.04E+08	1.04E+08	1556	-0.62	2.34E-06	0.000126	UBE2D3	1.04E+08
chr4	1.04E+08	1.04E+08	1556	-0.62	2.34E-06	0.000126	UBE2D3	1.04E+08
chr4	1.04E+08	1.04E+08	1556	-0.62	2.34E-06	0.000126	UBE2D3	1.04E+08
chr4	1.04E+08	1.04E+08	1556	-0.62	2.34E-06	0.000126	UBE2D3	1.04E+08
chr4	1.04E+08	1.04E+08	1556	-0.62	2.34E-06	0.000126	UBE2D3	1.04E+08
chr4	1.04E+08	1.04E+08	1556	-0.62	2.34E-06	0.000126	UBE2D3	1.04E+08
chr4	1.04E+08	1.04E+08	1556	-0.62	2.34E-06	0.000126	LOC105377	1.04E+08
chr4	1.04E+08	1.04E+08	1556	-0.62	2.34E-06	0.000126	LOC105377	1.04E+08
chr4	1.04E+08	1.04E+08	1556	-0.62	2.34E-06	0.000126	UBE2D3	1.04E+08
chr1	1.08E+08	1.08E+08	317	-0.97	2.37E-06	0.000127	NTNG1	1.08E+08
chr1	1.08E+08	1.08E+08	317	-0.97	2.37E-06	0.000127	NTNG1	1.08E+08
chr10	62537932	62538671	740	-0.74	2.41E-06	0.000128	CDK1	62533211
chr10	62537932	62538671	740	-0.74	2.41E-06	0.000128	CDK1	62533088
chr10	62537932	62538671	740	-0.74	2.41E-06	0.000128	CDK1	62533211
chr10	62537932	62538671	740	-0.74	2.41E-06	0.000128	CDK1	62533211
chr11	65683220	65683585	366	-0.87	2.45E-06	0.00013	DRAP1	65681727
chr1	1.7E+08	1.7E+08	495	-0.57	2.60E-06	0.000136	KIFAP3	1.7E+08
chr1	1.7E+08	1.7E+08	495	-0.57	2.60E-06	0.000136	KIFAP3	1.7E+08
chr1	1.7E+08	1.7E+08	495	-0.57	2.60E-06	0.000136	KIFAP3	1.7E+08
chr17	10278580	10278994	415	-1.13	2.63E-06	0.000137	MYH13	10274322
chr1	90101711	90102210	500	-0.84	2.64E-06	0.000137	FLJ27354	90096453
chr2	22758945	22759388	444	-1.07	2.69E-06	0.000139	LOC10272:	22754350
chr9	92249943	92250292	350	-1.15	2.71E-06	0.00014	UNQ6494	92249697
chr15	49258920	49259338	419	-0.87	2.71E-06	0.00014	SHC4	49253641

chr3	1.57E+08	1.57E+08	332	-0.89	2.77E-06	0.000142	LINC00880	1.57E+08
chr5	1.49E+08	1.49E+08	461	-0.92	2.84E-06	0.000144	PCYOX1L	1.49E+08
chr5	1.49E+08	1.49E+08	461	-0.92	2.84E-06	0.000144	GRPEL2-AS	1.49E+08
chr5	1.49E+08	1.49E+08	461	-0.92	2.84E-06	0.000144	PCYOX1L	1.49E+08
chr5	1.49E+08	1.49E+08	461	-0.92	2.84E-06	0.000144	PCYOX1L	1.49E+08
chr7	94287144	94287940	797	-0.84	2.85E-06	0.000144	SGCE	94283521
chr7	94287144	94287940	797	-0.84	2.85E-06	0.000144	SGCE	94283521
chr7	94287144	94287940	797	-0.84	2.85E-06	0.000144	SGCE	94283521
chr7	94287144	94287940	797	-0.84	2.85E-06	0.000144	SGCE	94283521
chr7	94287144	94287940	797	-0.84	2.85E-06	0.000144	PEG10	94280636
chr7	94287144	94287940	797	-0.84	2.85E-06	0.000144	PEG10	94280636
chr7	94287144	94287940	797	-0.84	2.85E-06	0.000144	PEG10	94280636
chr7	94287144	94287940	797	-0.84	2.85E-06	0.000144	PEG10	94280636
chr7	94287144	94287940	797	-0.84	2.85E-06	0.000144	PEG10	94280636
chr7	94287144	94287940	797	-0.84	2.85E-06	0.000144	PEG10	94280636
chr9	82186330	82186982	653	-0.48	2.98E-06	0.00015	TLE4	82181687
chr9	82186330	82186982	653	-0.48	2.98E-06	0.00015	TLE4	82181687
chr9	82186330	82186982	653	-0.48	2.98E-06	0.00015	TLE4	82181687
chr9	82186330	82186982	653	-0.48	2.98E-06	0.00015	TLE4	82181687
chr9	82186330	82186982	653	-0.48	2.98E-06	0.00015	TLE4	82181687
chr9	82186330	82186982	653	-0.48	2.98E-06	0.00015	TLE4	82181687
chr16	78133167	78134025	859	-0.63	2.98E-06	0.00015	WWOX	78128309
chr16	78133167	78134025	859	-0.63	2.98E-06	0.00015	WWOX	78128309
chr16	78133167	78134025	859	-0.63	2.98E-06	0.00015	WWOX	78128309
chr16	78133167	78134025	859	-0.63	2.98E-06	0.00015	WWOX	78128309
chr16	78133167	78134025	859	-0.63	2.98E-06	0.00015	WWOX	78128309
chr8	74268585	74269091	507	-0.82	3.05E-06	0.000152	RDH10-AS1	74266696
chr16	72127341	72127945	605	-0.75	3.06E-06	0.000152	TXNL4B	72126215
chr16	72127341	72127945	605	-0.75	3.06E-06	0.000152	TXNL4B	72125768
chr16	72127341	72127945	605	-0.75	3.06E-06	0.000152	TXNL4B	72125539
chr16	72127341	72127945	605	-0.75	3.06E-06	0.000152	DHX38	72122614
chr9	1E+08	1E+08	923	-0.59	3.07E-06	0.000153	NCBP1	1E+08
chr9	1E+08	1E+08	923	-0.59	3.07E-06	0.000153	TSTD2	1E+08
chr8	66546130	66546702	573	-0.62	3.08E-06	0.000153	ARMC1	66544452
chr8	66546130	66546702	573	-0.62	3.08E-06	0.000153	ARMC1	66544452
chr4	1.12E+08	1.12E+08	452	-0.72	3.10E-06	0.000154	PITX2	1.12E+08
chr1	1.66E+08	1.66E+08	236	-1.1	3.13E-06	0.000155	TMCO1	1.66E+08
chr1	1.66E+08	1.66E+08	236	-1.1	3.13E-06	0.000155	TMCO1	1.66E+08
chr1	1.66E+08	1.66E+08	236	-1.1	3.13E-06	0.000155	TMCO1	1.66E+08
chr1	1.66E+08	1.66E+08	236	-1.1	3.13E-06	0.000155	TMCO1	1.66E+08
chr3	1.86E+08	1.86E+08	568	-0.65	3.15E-06	0.000155	ETV5	1.86E+08
chr15	42867316	42867904	589	-0.79	3.30E-06	0.000161	STARD9	42862856
chr12	32907968	32909370	1403	-0.72	3.30E-06	0.000161	YARS2	32906887
chr9	74383376	74384941	1566	-0.54	3.34E-06	0.000162	TMEM2	74381800
chr9	74383376	74384941	1566	-0.54	3.34E-06	0.000162	TMEM2	74381800
chr1	1.18E+08	1.18E+08	536	-0.71	3.34E-06	0.000162	LOC101929	1.18E+08
chr1	1.18E+08	1.18E+08	536	-0.71	3.34E-06	0.000162	TTF2	1.18E+08

chr9	72287777	72288212	436	-0.8	3.38E-06	0.000164	APBA1	72285275
chr1	2.05E+08	2.05E+08	531	-0.97	3.40E-06	0.000164	MIR135B	2.05E+08
chr20	56197334	56197973	640	-0.98	3.43E-06	0.000165	ZBP1	56193632
chr20	56197334	56197973	640	-0.98	3.43E-06	0.000165	ZBP1	56193632
chr20	56197334	56197973	640	-0.98	3.43E-06	0.000165	ZBP1	56193632
chr20	56197334	56197973	640	-0.98	3.43E-06	0.000165	ZBP1	56193632
chr10	1.32E+08	1.32E+08	1261	-0.57	3.50E-06	0.000167	LINC00959	1.32E+08
chr16	4545167	4545524	358	-0.98	3.51E-06	0.000167	HMOX2	4540819
chr16	4545167	4545524	358	-0.98	3.51E-06	0.000167	HMOX2	4540858
chr7	43561993	43562445	453	-0.85	3.56E-06	0.000169	LOC10050(	43560141
chr9	93404991	93405459	469	-1.09	3.72E-06	0.000175	DIRAS2	93403387
chr11	65382702	65383267	566	-0.58	3.79E-06	0.000177	PCNX3	65378782
chr11	65382702	65383267	566	-0.58	3.79E-06	0.000177	MAP3K11	65379720
chr3	98241136	98241996	861	-0.64	3.88E-06	0.00018	CLDND1	98239910
chr3	98241136	98241996	861	-0.64	3.88E-06	0.00018	CLDND1	98239910
chr3	98241136	98241996	861	-0.64	3.88E-06	0.00018	CLDND1	98239910
chr3	98241136	98241996	861	-0.64	3.88E-06	0.00018	CLDND1	98239910
chr3	98241136	98241996	861	-0.64	3.88E-06	0.00018	CLDND1	98239910
chr3	98241136	98241996	861	-0.64	3.88E-06	0.00018	CLDND1	98239910
chr1	1.6E+08	1.6E+08	589	-0.7	3.93E-06	0.000182	DUSP23	1.6E+08
chr4	82085791	82086227	437	-1.05	3.94E-06	0.000182	LOC10192{	82081093
chr7	21466637	21468041	1405	-0.59	3.95E-06	0.000182	SP4	21462688
chr5	1.16E+08	1.16E+08	363	-0.85	4.03E-06	0.000185	LOC102467	1.16E+08
chr5	1.51E+08	1.51E+08	536	-0.83	4.09E-06	0.000186	FAT2	1.51E+08
chr14	20904189	20904832	644	-0.9	4.10E-06	0.000186	KLHL33	20901801
chr20	25604278	25605056	779	-0.61	4.19E-06	0.00019	NANP	25602648
chr20	25604278	25605056	779	-0.61	4.19E-06	0.00019	ZNF337-AS	25599680
chr20	25604278	25605056	779	-0.61	4.19E-06	0.00019	ZNF337-AS	25599680
chr20	25604278	25605056	779	-0.61	4.19E-06	0.00019	ZNF337-AS	25599860
chr2	55746514	55746974	461	-0.87	4.20E-06	0.00019	CFAP36	55741730
chr2	55746514	55746974	461	-0.87	4.20E-06	0.00019	CFAP36	55741730
chr13	1.11E+08	1.11E+08	511	-0.69	4.25E-06	0.000192	ING1	1.11E+08
chr13	1.11E+08	1.11E+08	511	-0.69	4.25E-06	0.000192	ING1	1.11E+08
chr3	1.21E+08	1.21E+08	675	-0.65	4.26E-06	0.000192	GOLGB1	1.21E+08
chr3	1.21E+08	1.21E+08	675	-0.65	4.26E-06	0.000192	GOLGB1	1.21E+08
chr3	1.21E+08	1.21E+08	675	-0.65	4.26E-06	0.000192	GOLGB1	1.21E+08
chr3	1.21E+08	1.21E+08	675	-0.65	4.26E-06	0.000192	GOLGB1	1.21E+08
chr5	1.23E+08	1.23E+08	653	-0.77	4.29E-06	0.000193	CSNK1G3	1.23E+08
chr5	1.23E+08	1.23E+08	653	-0.77	4.29E-06	0.000193	CSNK1G3	1.23E+08
chr5	1.23E+08	1.23E+08	653	-0.77	4.29E-06	0.000193	CSNK1G3	1.23E+08
chr14	55658100	55658745	646	-0.73	4.32E-06	0.000193	DLGAP5	55656396
chr14	55658100	55658745	646	-0.73	4.32E-06	0.000193	DLGAP5	55656396
chr20	39968650	39969632	983	-0.71	4.35E-06	0.000195	LPIN3	39964492
chr20	39968650	39969632	983	-0.71	4.35E-06	0.000195	LPIN3	39964492
chr3	1.72E+08	1.72E+08	548	-0.63	4.49E-06	0.000198	NCEH1	1.72E+08
chr3	1.72E+08	1.72E+08	548	-0.63	4.49E-06	0.000198	NCEH1	1.72E+08
chr3	1.72E+08	1.72E+08	548	-0.63	4.49E-06	0.000198	NCEH1	1.72E+08

chr3	1.72E+08	1.72E+08	548	-0.63	4.49E-06	0.000198	NCEH1	1.72E+08
chr2	75750681	75751031	351	-0.98	4.56E-06	0.000201	LOC101927	75746193
chr1	1.75E+08	1.75E+08	406	-0.73	4.57E-06	0.000201	RABGAP1L	1.75E+08
chr11	7986954	7987296	343	-1.27	4.57E-06	0.000201	NLRP10	7983059
chr15	1.01E+08	1.01E+08	666	-1	4.58E-06	0.000201	SPATA41	1.01E+08
chr15	1.01E+08	1.01E+08	666	-1	4.58E-06	0.000201	SPATA41	1.01E+08
chr5	1.18E+08	1.18E+08	754	-0.64	4.60E-06	0.000201	DTWD2	1.18E+08
chr5	1.18E+08	1.18E+08	754	-0.64	4.60E-06	0.000201	DTWD2	1.18E+08
chr1	35218868	35219251	384	-1	4.61E-06	0.000202	GJB5	35215647
chr11	3862032	3863071	1040	-0.73	4.62E-06	0.000202	RHOG	3860213
chr9	1.31E+08	1.31E+08	670	-0.75	4.71E-06	0.000204	SET	1.31E+08
chr9	1.31E+08	1.31E+08	670	-0.75	4.71E-06	0.000204	SET	1.31E+08
chr9	1.31E+08	1.31E+08	670	-0.75	4.71E-06	0.000204	SET	1.31E+08
chr9	1.31E+08	1.31E+08	670	-0.75	4.71E-06	0.000204	SET	1.31E+08
chr1	2.45E+08	2.45E+08	309	-1.16	4.72E-06	0.000204	C1orf100	2.45E+08
chr1	2.45E+08	2.45E+08	309	-1.16	4.72E-06	0.000204	C1orf100	2.45E+08
chr1	2.45E+08	2.45E+08	309	-1.16	4.72E-06	0.000204	C1orf100	2.45E+08
chr8	93977672	93978561	890	-0.63	4.73E-06	0.000204	TRIQK	93976372
chr8	93977672	93978561	890	-0.63	4.73E-06	0.000204	TRIQK	93976372
chr8	93977672	93978561	890	-0.63	4.73E-06	0.000204	TRIQK	93976372
chr8	93977672	93978561	890	-0.63	4.73E-06	0.000204	TRIQK	93976372
chr8	93977672	93978561	890	-0.63	4.73E-06	0.000204	TRIQK	93976372
chr8	93977672	93978561	890	-0.63	4.73E-06	0.000204	TRIQK	93975817
chr8	93977672	93978561	890	-0.63	4.73E-06	0.000204	TRIQK	93976265
chr1	2.48E+08	2.48E+08	1031	-0.92	4.82E-06	0.000207	OR2M5	2.48E+08
chr9	78505069	78506153	1085	-0.58	4.92E-06	0.00021	PCSK5	78500559
chr9	78505069	78506153	1085	-0.58	4.92E-06	0.00021	PCSK5	78500559
chr9	78505069	78506153	1085	-0.58	4.92E-06	0.00021	PCSK5	78500559
chr9	1.24E+08	1.24E+08	822	-0.81	5.03E-06	0.000212	STOM	1.24E+08
chr9	1.24E+08	1.24E+08	822	-0.81	5.03E-06	0.000212	STOM	1.24E+08
chr9	1.24E+08	1.24E+08	822	-0.81	5.03E-06	0.000212	STOM	1.24E+08
chr9	1.24E+08	1.24E+08	822	-0.81	5.03E-06	0.000212	STOM	1.24E+08
chr9	1.24E+08	1.24E+08	822	-0.81	5.03E-06	0.000212	STOM	1.24E+08
chr1	90460289	90461168	880	-0.52	5.03E-06	0.000212	ZNF326	90455677
chr1	90460289	90461168	880	-0.52	5.03E-06	0.000212	ZNF326	90455677
chr1	90460289	90461168	880	-0.52	5.03E-06	0.000212	GEMIN8P4	90458525
chr5	1.6E+08	1.6E+08	716	-0.89	5.05E-06	0.000213	ZBED8	1.6E+08
chr5	1.6E+08	1.6E+08	716	-0.89	5.05E-06	0.000213	ZBED8	1.6E+08
chr7	8008046	8008552	507	-0.67	5.07E-06	0.000213	LOC10050!	8006383
chr7	8008046	8008552	507	-0.67	5.07E-06	0.000213	GLCCI1	8003373
chr12	39298686	39299533	848	-0.75	5.09E-06	0.000214	CPNE8	39297420
chr19	50528605	50529316	712	-0.84	5.13E-06	0.000215	ZNF473	50524062
chr19	50528605	50529316	712	-0.84	5.13E-06	0.000215	VRK3	50526805
chr19	50528605	50529316	712	-0.84	5.13E-06	0.000215	VRK3	50526805
chr19	50528605	50529316	712	-0.84	5.13E-06	0.000215	ZNF473	50524062
chr19	50528605	50529316	712	-0.84	5.13E-06	0.000215	ZNF473	50524062
chr19	50528605	50529316	712	-0.84	5.13E-06	0.000215	VRK3	50526643

chr11	5705962	5706558	597	-0.67	5.24E-06	0.000218	TRIM22	5705816
chr11	5705962	5706558	597	-0.67	5.24E-06	0.000218	TRIM5	5704339
chr11	5705962	5706558	597	-0.67	5.24E-06	0.000218	TRIM5	5704339
chr11	5705962	5706558	597	-0.67	5.24E-06	0.000218	TRIM22	5705816
chr11	5705962	5706558	597	-0.67	5.24E-06	0.000218	TRIM5	5704339
chr16	20911589	20912361	773	-0.68	5.25E-06	0.000218	LYRM1	20906189
chr16	20911589	20912361	773	-0.68	5.25E-06	0.000218	LYRM1	20906987
chr16	20911589	20912361	773	-0.68	5.25E-06	0.000218	LYRM1	20906987
chr16	20911589	20912361	773	-0.68	5.25E-06	0.000218	LYRM1	20906619
chr16	20911589	20912361	773	-0.68	5.25E-06	0.000218	LYRM1	20907425
chr16	20911589	20912361	773	-0.68	5.25E-06	0.000218	DCUN1D3	20909561
chr16	20911589	20912361	773	-0.68	5.25E-06	0.000218	LYRM1	20906619
chr16	20911589	20912361	773	-0.68	5.25E-06	0.000218	LYRM1	20907394
chr16	20911589	20912361	773	-0.68	5.25E-06	0.000218	LYRM1	20906987
chr16	20911589	20912361	773	-0.68	5.25E-06	0.000218	LYRM1	20906619
chr16	20911589	20912361	773	-0.68	5.25E-06	0.000218	LYRM1	20906987
chr7	50599085	50599867	783	-0.93	5.30E-06	0.000219	DDC-AS1	50594456
chr15	93425530	93426086	557	-0.8	5.33E-06	0.00022	LINC01578	93421525
chr15	93425530	93426086	557	-0.8	5.33E-06	0.00022	LINC01578	93421072
chr15	93425530	93426086	557	-0.8	5.33E-06	0.00022	LINC01578	93421072
chr16	17565165	17565637	473	-0.95	5.45E-06	0.000223	XYLT1	17562738
chr1	77522304	77522577	274	-1.64	5.48E-06	0.000224	MIR7156	77520827
chr9	1.23E+08	1.23E+08	414	-0.68	5.53E-06	0.000225	CDK5RAP2	1.23E+08
chr9	1.23E+08	1.23E+08	414	-0.68	5.53E-06	0.000225	CDK5RAP2	1.23E+08
chr9	1.23E+08	1.23E+08	414	-0.68	5.53E-06	0.000225	CDK5RAP2	1.23E+08
chr9	1.23E+08	1.23E+08	414	-0.68	5.53E-06	0.000225	CDK5RAP2	1.23E+08
chr9	1.23E+08	1.23E+08	414	-0.68	5.53E-06	0.000225	CDK5RAP2	1.23E+08
chr9	1.23E+08	1.23E+08	414	-0.68	5.53E-06	0.000225	CDK5RAP2	1.23E+08
chr9	1.23E+08	1.23E+08	414	-0.68	5.53E-06	0.000225	CDK5RAP2	1.23E+08
chr9	1.23E+08	1.23E+08	414	-0.68	5.53E-06	0.000225	CDK5RAP2	1.23E+08
chr1	1.01E+08	1.01E+08	582	-0.61	5.55E-06	0.000226	DBT	1.01E+08
chr3	37034359	37035288	930	-0.62	5.67E-06	0.000229	MLH1	37030267
chr3	37034359	37035288	930	-0.62	5.67E-06	0.000229	MLH1	37029840
chr3	37034359	37035288	930	-0.62	5.67E-06	0.000229	MLH1	37030267
chr3	37034359	37035288	930	-0.62	5.67E-06	0.000229	MLH1	37030267
chr3	37034359	37035288	930	-0.62	5.67E-06	0.000229	EPM2AIP1	37032795
chr3	37034359	37035288	930	-0.62	5.67E-06	0.000229	MLH1	37030267
chr3	37034359	37035288	930	-0.62	5.67E-06	0.000229	MLH1	37029840
chr3	37034359	37035288	930	-0.62	5.67E-06	0.000229	MLH1	37029840
chr7	1.08E+08	1.08E+08	1236	-0.66	5.67E-06	0.000229	PNPLA8	1.08E+08
chr7	1.08E+08	1.08E+08	1236	-0.66	5.67E-06	0.000229	PNPLA8	1.08E+08
chr7	1.08E+08	1.08E+08	1236	-0.66	5.67E-06	0.000229	PNPLA8	1.08E+08
chr7	1.08E+08	1.08E+08	1236	-0.66	5.67E-06	0.000229	PNPLA8	1.08E+08
chr7	1.08E+08	1.08E+08	1236	-0.66	5.67E-06	0.000229	PNPLA8	1.08E+08
chr7	1.08E+08	1.08E+08	1236	-0.66	5.67E-06	0.000229	PNPLA8	1.08E+08
chr1	1.01E+08	1.01E+08	577	-0.66	5.68E-06	0.000229	CDC14A	1.01E+08
chr1	1.01E+08	1.01E+08	577	-0.66	5.68E-06	0.000229	CDC14A	1.01E+08

chr1	1.01E+08	1.01E+08	577	-0.66	5.68E-06	0.000229	CDC14A	1.01E+08
chr8	1.11E+08	1.11E+08	1157	-0.52	5.70E-06	0.00023	EBAG9	1.11E+08
chr8	1.11E+08	1.11E+08	1157	-0.52	5.70E-06	0.00023	EBAG9	1.11E+08
chr8	1.11E+08	1.11E+08	1157	-0.52	5.70E-06	0.00023	EBAG9	1.11E+08
chr7	1.38E+08	1.38E+08	745	-0.98	5.77E-06	0.000232	MIR4468	1.38E+08
chr14	45722235	45723312	1078	-0.55	5.80E-06	0.000233	MIS18BP1	45720605
chr3	1.61E+08	1.61E+08	1356	-0.74	5.86E-06	0.000234	SPTSSB	1.61E+08
chr20	13765415	13766190	776	-0.92	5.90E-06	0.000235	ESF1	13763532
chr20	13765415	13766190	776	-0.92	5.90E-06	0.000235	ESF1	13763579
chr20	13765415	13766190	776	-0.92	5.90E-06	0.000235	NDUFAF5	13760671
chr20	13765415	13766190	776	-0.92	5.90E-06	0.000235	NDUFAF5	13760671
chr20	13765415	13766190	776	-0.92	5.90E-06	0.000235	NDUFAF5	13760671
chr9	84304285	84304980	696	-0.62	6.02E-06	0.000238	TLE1	84302450
chr9	84304285	84304980	696	-0.62	6.02E-06	0.000238	TLE1	84302450
chr9	84304285	84304980	696	-0.62	6.02E-06	0.000238	TLE1	84302450
chr9	84304285	84304980	696	-0.62	6.02E-06	0.000238	LOC101927	84299627
chr3	1.59E+08	1.59E+08	1317	-0.64	6.07E-06	0.000239	MFSD1	1.59E+08
chr3	1.59E+08	1.59E+08	1317	-0.64	6.07E-06	0.000239	MFSD1	1.59E+08
chr3	1.59E+08	1.59E+08	1317	-0.64	6.07E-06	0.000239	MFSD1	1.59E+08
chr3	1.59E+08	1.59E+08	1317	-0.64	6.07E-06	0.000239	MFSD1	1.59E+08
chr3	1.59E+08	1.59E+08	1317	-0.64	6.07E-06	0.000239	MFSD1	1.59E+08
chr3	1.61E+08	1.61E+08	837	-0.71	6.08E-06	0.000239	B3GALNT1	1.61E+08
chr3	1.61E+08	1.61E+08	837	-0.71	6.08E-06	0.000239	B3GALNT1	1.61E+08
chr3	1.61E+08	1.61E+08	837	-0.71	6.08E-06	0.000239	B3GALNT1	1.61E+08
chr3	1.61E+08	1.61E+08	837	-0.71	6.08E-06	0.000239	B3GALNT1	1.61E+08
chr3	1.61E+08	1.61E+08	837	-0.71	6.08E-06	0.000239	B3GALNT1	1.61E+08
chr2	48133228	48134193	966	-0.7	6.22E-06	0.000244	FBXO11	48130932
chr7	23053594	23054006	413	-0.68	6.32E-06	0.000247	FAM126A	23051770
chr6	86114064	86114770	707	-0.88	6.40E-06	0.000249	LOC101928	86112520
chr16	8891361	8892085	725	-0.77	6.42E-06	0.00025	PMM2	8886669
chr16	8891361	8892085	725	-0.77	6.42E-06	0.00025	TMEM186	8889505
chr1	1.1E+08	1.1E+08	741	-0.59	6.46E-06	0.000251	PSMA5	1.1E+08
chr1	1.1E+08	1.1E+08	741	-0.59	6.46E-06	0.000251	PSMA5	1.1E+08
chr1	1.1E+08	1.1E+08	741	-0.59	6.46E-06	0.000251	PSMA5	1.1E+08
chr1	1.1E+08	1.1E+08	741	-0.59	6.46E-06	0.000251	PSMA5	1.1E+08
chr1	2.49E+08	2.49E+08	1248	-0.58	6.76E-06	0.000259	ZNF672	2.49E+08
chr8	1.07E+08	1.07E+08	914	-0.66	6.81E-06	0.00026	ZFPM2-AS1	1.07E+08
chr8	1.07E+08	1.07E+08	914	-0.66	6.81E-06	0.00026	ZFPM2-AS1	1.07E+08
chr10	24498896	24499227	332	-1.34	6.84E-06	0.00026	KIAA1217	24492719
chr10	24498896	24499227	332	-1.34	6.84E-06	0.00026	KIAA1217	24492719
chr10	24498896	24499227	332	-1.34	6.84E-06	0.00026	KIAA1217	24492719
chr8	74884306	74884837	532	-0.63	6.85E-06	0.00026	TCEB1	74882522
chr8	74884306	74884837	532	-0.63	6.85E-06	0.00026	TCEB1	74882143
chr8	74884306	74884837	532	-0.63	6.85E-06	0.00026	TCEB1	74882143
chr8	74884306	74884837	532	-0.63	6.85E-06	0.00026	TMEM70	74883376
chr8	74884306	74884837	532	-0.63	6.85E-06	0.00026	TCEB1	74882522
chr8	74884306	74884837	532	-0.63	6.85E-06	0.00026	TCEB1	74882522

chr8	74884306	74884837	532	-0.63	6.85E-06	0.00026	TCEB1	74882522
chr8	74884306	74884837	532	-0.63	6.85E-06	0.00026	TCEB1	74882522
chr8	74884306	74884837	532	-0.63	6.85E-06	0.00026	TMEM70	74883376
chr8	74884306	74884837	532	-0.63	6.85E-06	0.00026	TMEM70	74883376
chr8	74884306	74884837	532	-0.63	6.85E-06	0.00026	TCEB1	74882522
chr8	74884306	74884837	532	-0.63	6.85E-06	0.00026	TCEB1	74882522
chr9	1.26E+08	1.26E+08	778	-0.66	6.90E-06	0.000262	RABGAP1	1.26E+08
chr1	2.49E+08	2.49E+08	1085	-0.58	7.01E-06	0.000266	PGBD2	2.49E+08
chr1	2.49E+08	2.49E+08	1085	-0.58	7.01E-06	0.000266	PGBD2	2.49E+08
chr15	93443418	93443631	214	-1.34	7.02E-06	0.000266	CHD2	93438550
chr15	93443418	93443631	214	-1.34	7.02E-06	0.000266	CHD2	93438550
chr15	93443418	93443631	214	-1.34	7.02E-06	0.000266	MIR3175	93442628
chr9	1.24E+08	1.24E+08	367	-0.92	7.04E-06	0.000266	PHF19	1.24E+08
chr6	75994323	75994865	543	-0.63	7.08E-06	0.000267	TMEM30A	75992632
chr6	75994323	75994865	543	-0.63	7.08E-06	0.000267	LOC10050€	75989729
chr6	75994323	75994865	543	-0.63	7.08E-06	0.000267	TMEM30A	75992632
chr9	35071963	35072927	965	-0.95	7.22E-06	0.000271	VCP	35070739
chr6	1.44E+08	1.44E+08	596	-1	7.52E-06	0.00028	LTV1	1.44E+08
chr12	67490517	67491157	641	-1.01	7.67E-06	0.000283	LOC102724	67488064
chr11	3818556	3819321	766	-0.56	7.78E-06	0.000285	NUP98	3817022
chr11	3818556	3819321	766	-0.56	7.78E-06	0.000285	NUP98	3817022
chr11	3818556	3819321	766	-0.56	7.78E-06	0.000285	NUP98	3817022
chr11	3818556	3819321	766	-0.56	7.78E-06	0.000285	NUP98	3817022
chr11	3818556	3819321	766	-0.56	7.78E-06	0.000285	PGAP2	3814048
chr11	3818556	3819321	766	-0.56	7.78E-06	0.000285	PGAP2	3814048
chr11	3818556	3819321	766	-0.56	7.78E-06	0.000285	PGAP2	3813953
chr11	3818556	3819321	766	-0.56	7.78E-06	0.000285	PGAP2	3814048
chr11	3818556	3819321	766	-0.56	7.78E-06	0.000285	PGAP2	3814048
chr11	3818556	3819321	766	-0.56	7.78E-06	0.000285	PGAP2	3814048
chr11	3818556	3819321	766	-0.56	7.78E-06	0.000285	PGAP2	3814048
chr11	3818556	3819321	766	-0.56	7.78E-06	0.000285	PGAP2	3814048
chr11	3818556	3819321	766	-0.56	7.78E-06	0.000285	PGAP2	3814188
chr11	3818556	3819321	766	-0.56	7.78E-06	0.000285	PGAP2	3814048
chr11	3818556	3819321	766	-0.56	7.78E-06	0.000285	PGAP2	3814048
chr11	3818556	3819321	766	-0.56	7.78E-06	0.000285	PGAP2	3814048
chr11	3818556	3819321	766	-0.56	7.78E-06	0.000285	PGAP2	3814048
chr11	3818556	3819321	766	-0.56	7.78E-06	0.000285	PGAP2	3814048
chr20	52684125	52684556	432	-0.64	7.94E-06	0.000289	MIR4756	52683024
chr5	40679198	40680171	974	-0.59	7.96E-06	0.000289	PTGER4	40675031
chr9	71735549	71737405	1857	-0.63	8.06E-06	0.000291	TJP2	71731179
chr11	94964349	94964826	478	-0.71	8.21E-06	0.000295	LOC100129	94958349
chr11	94964349	94964826	478	-0.71	8.21E-06	0.000295	SESN3	94962246
chr11	94964349	94964826	478	-0.71	8.21E-06	0.000295	SESN3	94963705
chr18	29598203	29599004	802	-0.61	8.21E-06	0.000295	RNF125	29593444
chr16	10479696	10480226	531	-0.78	8.38E-06	0.000301	ATF7IP2	10474911
chr16	10479696	10480226	531	-0.78	8.38E-06	0.000301	ATF7IP2	10474911
chr16	10479696	10480226	531	-0.78	8.38E-06	0.000301	ATF7IP2	10474911

chr6	52149366	52149939	574	-0.65	8.50E-06	0.000304	MCM3	52147679
chr6	52149366	52149939	574	-0.65	8.50E-06	0.000304	MCM3	52147679
chr6	57178530	57179063	534	-0.79	8.69E-06	0.000309	PRIM2	57177414
chr6	57178530	57179063	534	-0.79	8.69E-06	0.000309	PRIM2	57177414
chr6	57178530	57179063	534	-0.79	8.69E-06	0.000309	PRIM2	57174602
chr14	96000833	96001499	667	-0.59	8.75E-06	0.00031	SNHG10	95999209
chr14	96000833	96001499	667	-0.59	8.75E-06	0.00031	SCARNA13	95997966
chr14	96000833	96001499	667	-0.59	8.75E-06	0.00031	GLRX5	95996322
chr14	96000833	96001499	667	-0.59	8.75E-06	0.00031	SNHG10	95999209
chr6	72114408	72114757	350	-0.93	8.87E-06	0.000312	MIR30A	72111324
chr8	38853903	38854479	577	-0.61	9.01E-06	0.000316	ADAM9	38849504
chr8	38853903	38854479	577	-0.61	9.01E-06	0.000316	ADAM9	38849504
chr8	38853903	38854479	577	-0.61	9.01E-06	0.000316	ADAM9	38849504
chr8	38853903	38854479	577	-0.61	9.01E-06	0.000316	ADAM9	38849504
chr8	38853903	38854479	577	-0.61	9.01E-06	0.000316	TM2D2	38852041
chr8	38853903	38854479	577	-0.61	9.01E-06	0.000316	TM2D2	38852041
chr8	38853903	38854479	577	-0.61	9.01E-06	0.000316	TM2D2	38852041
chr8	38853903	38854479	577	-0.61	9.01E-06	0.000316	TM2D2	38852041
chr2	58273563	58274415	853	-0.6	9.02E-06	0.000317	VRK2	58268776
chr2	58273563	58274415	853	-0.6	9.02E-06	0.000317	VRK2	58268776
chr2	58273563	58274415	853	-0.6	9.02E-06	0.000317	VRK2	58268776
chr2	58273563	58274415	853	-0.6	9.02E-06	0.000317	VRK2	58268776
chr2	58273563	58274415	853	-0.6	9.02E-06	0.000317	VRK2	58268776
chr2	58273563	58274415	853	-0.6	9.02E-06	0.000317	VRK2	58268846
chr2	58273563	58274415	853	-0.6	9.02E-06	0.000317	VRK2	58268776
chr9	77702647	77703710	1064	-0.51	9.18E-06	0.000321	OSTF1	77698397
chr9	77702647	77703710	1064	-0.51	9.18E-06	0.000321	NMRK1	77701133
chr9	77702647	77703710	1064	-0.51	9.18E-06	0.000321	NMRK1	77701133
chr9	77702647	77703710	1064	-0.51	9.18E-06	0.000321	NMRK1	77701133
chr10	90611488	90611799	312	-1.28	9.35E-06	0.000325	ANKRD22	90609732
chr9	72873395	72874456	1062	-0.57	9.38E-06	0.000325	SMC5	72868877
chr9	72873395	72874456	1062	-0.57	9.38E-06	0.000325	SMC5-AS1	72871790
chr5	1.31E+08	1.31E+08	479	-0.66	9.40E-06	0.000326	LYRM7	1.31E+08
chr5	1.31E+08	1.31E+08	479	-0.66	9.40E-06	0.000326	LYRM7	1.31E+08
chr5	1.31E+08	1.31E+08	479	-0.66	9.40E-06	0.000326	LYRM7	1.31E+08
chr8	37552586	37553322	737	-0.71	9.50E-06	0.000328	ZNF703	37548268
chr6	97730588	97731417	830	-0.75	9.57E-06	0.00033	MMS22L	97729052
chr8	1.08E+08	1.08E+08	281	-0.88	9.60E-06	0.000331	OXR1	1.08E+08
chr8	1.08E+08	1.08E+08	281	-0.88	9.60E-06	0.000331	OXR1	1.08E+08
chr16	27326810	27327424	615	-0.75	9.72E-06	0.000333	IL4R	27320229
chr16	27326810	27327424	615	-0.75	9.72E-06	0.000333	IL4R	27320229
chr16	27326810	27327424	615	-0.75	9.72E-06	0.000333	IL4R	27320229
chr16	27326810	27327424	615	-0.75	9.72E-06	0.000333	IL4R	27320229
chr3	1.94E+08	1.94E+08	857	-0.57	9.81E-06	0.000335	LOC64732[illegible]	1.94E+08
chr10	99449362	99449757	396	-0.85	1.00E-05	0.00034	AVPI1	99445015
chr19	36505101	36505464	364	-0.85	1.01E-05	0.000341	ALKBH6	36503141
chr19	36505101	36505464	364	-0.85	1.01E-05	0.000341	ALKBH6	36503141

chr19	36505101	36505464	364	-0.85	1.01E-05	0.000341	LOC101927	36500400
chr19	36505101	36505464	364	-0.85	1.01E-05	0.000341	ALKBH6	36503145
chr11	8008549	8009024	476	-0.77	1.02E-05	0.000344	EIF3F	8003866
chr20	21551728	21552471	744	-0.89	1.02E-05	0.000346	LOC101929	21545661
chr9	4984684	4985364	681	-0.79	1.03E-05	0.000347	JAK2	4980244
chr3	1.32E+08	1.32E+08	1086	-0.64	1.04E-05	0.000348	DNAJC13	1.32E+08
chr5	1.26E+08	1.26E+08	636	-0.72	1.05E-05	0.000351	LOC102723	1.26E+08
chr5	1.26E+08	1.26E+08	636	-0.72	1.05E-05	0.000351	LMNB1	1.26E+08
chr5	1.26E+08	1.26E+08	636	-0.72	1.05E-05	0.000351	LMNB1	1.26E+08
chr5	1.26E+08	1.26E+08	636	-0.72	1.05E-05	0.000351	LMNB1	1.26E+08
chr2	63815445	63816183	739	-0.7	1.06E-05	0.000354	MDH1	63810742
chr2	63815445	63816183	739	-0.7	1.06E-05	0.000354	WDPCP	63813867
chr2	63815445	63816183	739	-0.7	1.06E-05	0.000354	MDH1	63811284
chr2	63815445	63816183	739	-0.7	1.06E-05	0.000354	MDH1	63811284
chr2	63815445	63816183	739	-0.7	1.06E-05	0.000354	MDH1	63810742
chr14	24711432	24712415	984	-0.55	1.06E-05	0.000354	TINF2	24709880
chr14	24711432	24712415	984	-0.55	1.06E-05	0.000354	TINF2	24709880
chr12	1.02E+08	1.02E+08	753	-0.63	1.06E-05	0.000354	GNPTAB	1.02E+08
chr5	1.51E+08	1.51E+08	865	-0.55	1.07E-05	0.000355	G3BP1	1.51E+08
chr5	1.51E+08	1.51E+08	865	-0.55	1.07E-05	0.000355	G3BP1	1.51E+08
chr5	1.51E+08	1.51E+08	865	-0.55	1.07E-05	0.000355	LOC100652	1.51E+08
chr5	1.51E+08	1.51E+08	865	-0.55	1.07E-05	0.000355	LOC100652	1.51E+08
chr5	1.34E+08	1.34E+08	472	-0.86	1.08E-05	0.000359	LOC101927	1.34E+08
chr19	11266442	11266762	321	-0.84	1.10E-05	0.000363	SPC24	11264488
chr19	11266442	11266762	321	-0.84	1.10E-05	0.000363	SPC24	11264488
chr19	11266442	11266762	321	-0.84	1.10E-05	0.000363	SPC24	11264488
chr19	11266442	11266762	321	-0.84	1.10E-05	0.000363	SPC24	11264488
chr4	83295161	83295714	554	-0.78	1.10E-05	0.000364	HNRNPD	83293149
chr4	83295161	83295714	554	-0.78	1.10E-05	0.000364	HNRNPD	83293149
chr4	83295161	83295714	554	-0.78	1.10E-05	0.000364	HNRNPD	83293149
chr4	83295161	83295714	554	-0.78	1.10E-05	0.000364	HNRNPD	83293149
chr1	2466304	2466742	439	-0.97	1.13E-05	0.00037	HES5	2459684
chr9	1.26E+08	1.26E+08	636	-0.73	1.14E-05	0.000373	PDCL	1.26E+08
chr11	1.25E+08	1.25E+08	532	-0.76	1.14E-05	0.000374	ROBO4	1.25E+08
chr11	1.25E+08	1.25E+08	532	-0.76	1.14E-05	0.000374	ROBO4	1.25E+08
chr6	86388425	86389200	776	-0.76	1.15E-05	0.000375	SNHG5	86386451
chr6	86388425	86389200	776	-0.76	1.15E-05	0.000375	SNORD50A	86385086
chr6	86388425	86389200	776	-0.76	1.15E-05	0.000375	SNORD50B	86385377
chr1	1.66E+08	1.66E+08	309	-0.97	1.15E-05	0.000375	MIR3658	1.66E+08
chr5	1.28E+08	1.28E+08	841	-0.97	1.16E-05	0.000377	FBN2	1.28E+08
chr6	86113409	86113652	244	-1.12	1.18E-05	0.000381	LOC101928	86112520
chr2	74652495	74653073	579	-0.84	1.23E-05	0.000394	C2orf81	74646757
chr2	74652495	74653073	579	-0.84	1.23E-05	0.000394	C2orf81	74646757
chr2	74652495	74653073	579	-0.84	1.23E-05	0.000394	C2orf81	74646757
chr12	1.17E+08	1.17E+08	542	-0.68	1.23E-05	0.000395	RNFT2	1.17E+08
chr12	1.17E+08	1.17E+08	542	-0.68	1.23E-05	0.000395	RNFT2	1.17E+08
chr12	1.17E+08	1.17E+08	542	-0.68	1.23E-05	0.000395	C12orf49	1.17E+08

chr12	1.03E+08	1.03E+08	657	-0.62	1.23E-05	0.000395	NUP37	1.03E+08
chr12	1.03E+08	1.03E+08	657	-0.62	1.23E-05	0.000395	PARPBP	1.03E+08
chr13	78111533	78111995	463	-0.84	1.24E-05	0.000397	SCEL	78104808
chr13	78111533	78111995	463	-0.84	1.24E-05	0.000397	SCEL	78104808
chr13	78111533	78111995	463	-0.84	1.24E-05	0.000397	SCEL	78104808
chr16	56648496	56648841	346	-1.19	1.25E-05	0.000397	MT1L	56646372
chr8	37962522	37963491	970	-0.68	1.25E-05	0.000397	ASH2L	37958010
chr8	37962522	37963491	970	-0.68	1.25E-05	0.000397	ASH2L	37958310
chr8	37962522	37963491	970	-0.68	1.25E-05	0.000397	ASH2L	37958310
chr8	37962522	37963491	970	-0.68	1.25E-05	0.000397	ASH2L	37958010
chr3	1.79E+08	1.79E+08	654	-0.71	1.26E-05	4.00E-04	MFN1	1.79E+08
chr16	62072176	62072937	762	-0.77	1.26E-05	4.00E-04	CDH8	62068739
chr6	1.7E+08	1.7E+08	450	-1.01	1.26E-05	0.000401	LINC01615	1.7E+08
chr6	1.7E+08	1.7E+08	450	-1.01	1.26E-05	0.000401	LINC01615	1.7E+08
chr19	12444348	12444752	405	-0.82	1.26E-05	0.000401	ZNF563	12442534
chr5	1.27E+08	1.27E+08	276	-1.09	1.27E-05	0.000401	CTXN3	1.27E+08
chr5	1.27E+08	1.27E+08	276	-1.09	1.27E-05	0.000401	CTXN3	1.27E+08
chr7	1.12E+08	1.12E+08	496	-0.97	1.27E-05	0.000403	IFRD1	1.12E+08
chr7	1.12E+08	1.12E+08	496	-0.97	1.27E-05	0.000403	IFRD1	1.12E+08
chr21	31537096	31537524	429	-0.91	1.28E-05	0.000404	CLDN17	31536971
chr6	1.67E+08	1.67E+08	951	-0.56	1.29E-05	0.000405	RPS6KA2	1.67E+08
chr11	6255716	6256523	808	-0.56	1.29E-05	0.000406	FAM160A2	6253941
chr11	6255716	6256523	808	-0.56	1.29E-05	0.000406	CNGA4	6255321
chr11	6255716	6256523	808	-0.56	1.29E-05	0.000406	FAM160A2	6253941
chr9	1.19E+08	1.19E+08	331	-1.17	1.33E-05	0.000413	ASTN2-AS1	1.19E+08
chr12	34175196	34175721	526	-0.9	1.35E-05	0.000418	ALG10	34170215
chr9	1.27E+08	1.27E+08	163	-1.42	1.39E-05	0.000426	NEK6	1.27E+08
chr9	1.27E+08	1.27E+08	163	-1.42	1.39E-05	0.000426	NEK6	1.27E+08
chr9	1.27E+08	1.27E+08	163	-1.42	1.39E-05	0.000426	NEK6	1.27E+08
chr5	1.13E+08	1.13E+08	443	-0.82	1.39E-05	0.000426	MCC	1.13E+08
chr12	1.2E+08	1.2E+08	624	-0.79	1.40E-05	0.000428	CIT	1.2E+08
chr12	1.2E+08	1.2E+08	624	-0.79	1.40E-05	0.000428	CIT	1.2E+08
chr5	36151901	36152337	437	-0.63	1.41E-05	0.000428	SKP2	36147144
chr5	36151901	36152337	437	-0.63	1.41E-05	0.000428	SKP2	36147144
chr5	36151901	36152337	437	-0.63	1.41E-05	0.000428	SKP2	36147144
chr5	36151901	36152337	437	-0.63	1.41E-05	0.000428	LMBRD2	36150015
chr5	36151901	36152337	437	-0.63	1.41E-05	0.000428	MIR580	36146090
chr8	1.24E+08	1.24E+08	489	-0.61	1.42E-05	0.00043	LOC10537	1.24E+08
chr1	12079198	12079636	439	-0.8	1.44E-05	0.000435	MIIP	12074298
chr6	24775085	24775819	735	-0.73	1.45E-05	0.000436	GMNN	24770158
chr6	24775085	24775819	735	-0.73	1.45E-05	0.000436	GMNN	24770640
chr6	24775085	24775819	735	-0.73	1.45E-05	0.000436	GMNN	24770158
chr6	24775085	24775819	735	-0.73	1.45E-05	0.000436	GMNN	24770158
chr8	1.1E+08	1.1E+08	1014	-0.51	1.45E-05	0.000437	ENY2	1.1E+08
chr8	1.1E+08	1.1E+08	1014	-0.51	1.45E-05	0.000437	ENY2	1.1E+08
chr8	1.1E+08	1.1E+08	1014	-0.51	1.45E-05	0.000437	NUDCD1	1.1E+08
chr8	1.1E+08	1.1E+08	1014	-0.51	1.45E-05	0.000437	NUDCD1	1.1E+08

chr8	1.1E+08	1.1E+08	1014	-0.51	1.45E-05	0.000437	ENY2	1.1E+08
chr8	1.1E+08	1.1E+08	1014	-0.51	1.45E-05	0.000437	ENY2	1.1E+08
chr13	73355885	73356431	547	-0.79	1.47E-05	0.00044	PIBF1	73351229
chr13	73355885	73356431	547	-0.79	1.47E-05	0.00044	DIS3	73354266
chr13	73355885	73356431	547	-0.79	1.47E-05	0.00044	DIS3	73354266
chr14	50052976	50053759	784	-0.56	1.47E-05	0.000441	RPS29	50051134
chr14	50052976	50053759	784	-0.56	1.47E-05	0.000441	RPS29	50051134
chr9	1.28E+08	1.28E+08	493	-0.75	1.47E-05	0.000441	SCAI	1.28E+08
chr9	1.28E+08	1.28E+08	493	-0.75	1.47E-05	0.000441	SCAI	1.28E+08
chr9	1.25E+08	1.25E+08	246	-0.85	1.50E-05	0.000445	PTGS1	1.25E+08
chr9	1.25E+08	1.25E+08	246	-0.85	1.50E-05	0.000445	PTGS1	1.25E+08
chr9	1.25E+08	1.25E+08	246	-0.85	1.50E-05	0.000445	PTGS1	1.25E+08
chr9	1.25E+08	1.25E+08	246	-0.85	1.50E-05	0.000445	PTGS1	1.25E+08
chr9	1.25E+08	1.25E+08	246	-0.85	1.50E-05	0.000445	PTGS1	1.25E+08
chr9	1.25E+08	1.25E+08	246	-0.85	1.50E-05	0.000445	PTGS1	1.25E+08
chr3	1.6E+08	1.6E+08	911	-0.58	1.51E-05	0.000448	KPNA4	1.6E+08
chr8	82644500	82645213	714	-0.81	1.52E-05	0.00045	CHMP4C	82639687
chr1	1.11E+08	1.11E+08	1185	-0.51	1.55E-05	0.000454	RBM15	1.11E+08
chr1	1.11E+08	1.11E+08	1185	-0.51	1.55E-05	0.000454	RBM15	1.11E+08
chr1	1.11E+08	1.11E+08	1185	-0.51	1.55E-05	0.000454	LOC44060(	1.11E+08
chr12	15113719	15114864	1146	-0.7	1.55E-05	0.000455	ARHGDIB	15112562
chr4	1.24E+08	1.24E+08	475	-0.73	1.55E-05	0.000455	BBS12	1.24E+08
chr4	1.24E+08	1.24E+08	475	-0.73	1.55E-05	0.000455	CETN4P	1.24E+08
chr4	1.24E+08	1.24E+08	475	-0.73	1.55E-05	0.000455	BBS12	1.24E+08
chr9	79073875	79074958	1084	-0.56	1.61E-05	0.000467	GCNT1	79069067
chr9	79073875	79074958	1084	-0.56	1.61E-05	0.000467	GCNT1	79069067
chr1	1.6E+08	1.6E+08	571	-0.84	1.61E-05	0.000467	TAGLN2	1.6E+08
chr1	1.6E+08	1.6E+08	571	-0.84	1.61E-05	0.000467	TAGLN2	1.6E+08
chr1	1.6E+08	1.6E+08	571	-0.84	1.61E-05	0.000467	TAGLN2	1.6E+08
chr1	1.98E+08	1.98E+08	1672	-0.57	1.61E-05	0.000467	DENND1B	1.98E+08
chr1	1.98E+08	1.98E+08	1672	-0.57	1.61E-05	0.000467	DENND1B	1.98E+08
chr1	1.98E+08	1.98E+08	1672	-0.57	1.61E-05	0.000467	DENND1B	1.98E+08
chr1	1.98E+08	1.98E+08	1672	-0.57	1.61E-05	0.000467	DENND1B	1.98E+08
chr1	1.98E+08	1.98E+08	1672	-0.57	1.61E-05	0.000467	DENND1B	1.98E+08
chr6	1.17E+08	1.17E+08	641	-0.88	1.62E-05	0.000468	ZUFSP	1.17E+08
chr20	6034627	6034986	360	-0.83	1.63E-05	0.000469	LRRN4	6032726
chr14	31931648	31931976	329	-0.91	1.65E-05	0.000474	DTD2	31924680
chr7	77427645	77428252	608	-0.67	1.65E-05	0.000474	PHTF2	77423108
chr7	77427645	77428252	608	-0.67	1.65E-05	0.000474	PHTF2	77423108
chr7	77427645	77428252	608	-0.67	1.65E-05	0.000474	TMEM60	77425747
chr20	56887893	56888266	374	-0.84	1.68E-05	0.000479	PPP4R1L	56882495
chr9	1.24E+08	1.24E+08	942	-0.54	1.68E-05	0.00048	PSMD5	1.24E+08
chr9	1.24E+08	1.24E+08	942	-0.54	1.68E-05	0.00048	PSMD5	1.24E+08
chr9	1.24E+08	1.24E+08	942	-0.54	1.68E-05	0.00048	PSMD5-AS:	1.24E+08
chr1	2.47E+08	2.47E+08	981	-0.57	1.70E-05	0.000482	AHCTF1	2.47E+08
chr2	62932314	62932937	624	-0.65	1.72E-05	0.000486	EHBP1	62928000
chr2	62932314	62932937	624	-0.65	1.72E-05	0.000486	EHBP1	62929031

chr2	62932314	62932937	624	-0.65	1.72E-05	0.000486	EHBP1	62928000
chr8	80941796	80942680	885	-0.7	1.72E-05	0.000486	MRPS28	80940506
chr2	62080949	62082201	1253	-0.66	1.72E-05	0.000487	FAM161A	62079278
chr2	62080949	62082201	1253	-0.66	1.72E-05	0.000487	FAM161A	62079278
chr2	62080949	62082201	1253	-0.66	1.72E-05	0.000487	FAM161A	62079278
chr17	14204009	14204494	486	-0.58	1.73E-05	0.000489	HS3ST3B1	14199366
chr17	14204009	14204494	486	-0.58	1.73E-05	0.000489	MGC12916	14202056
chr17	14204009	14204494	486	-0.58	1.73E-05	0.000489	HS3ST3B1	14199366
chr8	1.07E+08	1.07E+08	750	-0.57	1.74E-05	0.00049	OXR1	1.07E+08
chr8	1.07E+08	1.07E+08	750	-0.57	1.74E-05	0.00049	OXR1	1.07E+08
chr10	28580770	28581211	442	-1.15	1.76E-05	0.000494	MIR8086	28576279
chr5	42423376	42424032	657	-0.71	1.79E-05	5.00E-04	GHR	42419553
chr5	42423376	42424032	657	-0.71	1.79E-05	5.00E-04	GHR	42419553
chr5	42423376	42424032	657	-0.71	1.79E-05	5.00E-04	GHR	42418876
chr5	42423376	42424032	657	-0.71	1.79E-05	5.00E-04	GHR	42420093
chr5	1.5E+08	1.5E+08	352	-1.02	1.80E-05	5.00E-04	SMIM3	1.5E+08
chr2	18741225	18742167	943	-0.65	1.80E-05	0.000502	RDH14	18739959
chr6	1.16E+08	1.16E+08	838	-0.6	1.81E-05	0.000502	NT5DC1	1.16E+08
chr3	1.6E+08	1.6E+08	1298	-0.48	1.81E-05	0.000503	PPM1L	1.6E+08
chr16	74700466	74701024	559	-0.73	1.82E-05	0.000505	RFWD3	74698779
chr1	6551597	6551838	242	-0.99	1.83E-05	0.000506	PLEKHG5	6548640
chr1	6551597	6551838	242	-0.99	1.83E-05	0.000506	PLEKHG5	6548654
chr1	6551597	6551838	242	-0.99	1.83E-05	0.000506	PLEKHG5	6549761
chr7	18548138	18549021	884	-0.71	1.83E-05	0.000506	HDAC9	18543899
chr7	18548138	18549021	884	-0.71	1.83E-05	0.000506	HDAC9	18543899
chr11	82996681	82997581	901	-0.58	1.86E-05	0.000511	CCDC90B	82995450
chr11	82996681	82997581	901	-0.58	1.86E-05	0.000511	CCDC90B	82995450
chr11	82996681	82997581	901	-0.58	1.86E-05	0.000511	CCDC90B	82995450
chr11	82996681	82997581	901	-0.58	1.86E-05	0.000511	CCDC90B	82995450
chr11	82996681	82997581	901	-0.58	1.86E-05	0.000511	CCDC90B	82995450
chr11	82996681	82997581	901	-0.58	1.86E-05	0.000511	CCDC90B	82995450
chr16	58663543	58664560	1018	-0.62	1.88E-05	0.000515	CNOT1	58661790
chr16	58663543	58664560	1018	-0.62	1.88E-05	0.000515	CNOT1	58661790
chr16	58663543	58664560	1018	-0.62	1.88E-05	0.000515	CNOT1	58661790
chr16	58663543	58664560	1018	-0.62	1.88E-05	0.000515	CNOT1	58661790
chr15	44719123	44719937	815	-0.74	1.91E-05	0.000523	CTDSPL2	44714578
chr18	9474874	9475748	875	-0.69	1.91E-05	0.000523	RALBP1	9470529
chr2	24270223	24270540	318	-0.86	1.94E-05	0.000528	FKBP1B	24267583
chr2	24270223	24270540	318	-0.86	1.94E-05	0.000528	C2orf44	24268296
chr2	24270223	24270540	318	-0.86	1.94E-05	0.000528	FKBP1B	24267583
chr2	24270223	24270540	318	-0.86	1.94E-05	0.000528	C2orf44	24268296
chr5	1.57E+08	1.57E+08	571	-0.72	1.94E-05	0.000528	THG1L	1.57E+08
chr5	1.57E+08	1.57E+08	571	-0.72	1.94E-05	0.000528	THG1L	1.57E+08
chr5	1.57E+08	1.57E+08	571	-0.72	1.94E-05	0.000528	THG1L	1.57E+08
chr5	1.57E+08	1.57E+08	571	-0.72	1.94E-05	0.000528	THG1L	1.57E+08
chr17	5487735	5488511	777	-0.68	1.95E-05	0.00053	NLRP1	5485832
chr17	5487735	5488511	777	-0.68	1.95E-05	0.00053	NLRP1	5485832

chr17	5487735	5488511	777	-0.68	1.95E-05	0.00053	NLRP1	5485832
chr17	5487735	5488511	777	-0.68	1.95E-05	0.00053	NLRP1	5485832
chr17	5487735	5488511	777	-0.68	1.95E-05	0.00053	NLRP1	5485832
chr2	2.07E+08	2.07E+08	846	-0.7	1.96E-05	0.00053	NRP2	2.07E+08
chr2	2.07E+08	2.07E+08	846	-0.7	1.96E-05	0.00053	NRP2	2.07E+08
chr2	2.07E+08	2.07E+08	846	-0.7	1.96E-05	0.00053	NRP2	2.07E+08
chr2	2.07E+08	2.07E+08	846	-0.7	1.96E-05	0.00053	NRP2	2.07E+08
chr2	2.07E+08	2.07E+08	846	-0.7	1.96E-05	0.00053	NRP2	2.07E+08
chr2	2.07E+08	2.07E+08	846	-0.7	1.96E-05	0.00053	NRP2	2.07E+08
chr5	64061881	64062308	428	-1.15	1.96E-05	0.00053	CWC27	64059744
chr5	64061881	64062308	428	-1.15	1.96E-05	0.00053	CWC27	64059744
chr5	64061881	64062308	428	-1.15	1.96E-05	0.00053	CWC27	64059744
chr5	64061881	64062308	428	-1.15	1.96E-05	0.00053	CWC27	64059744
chr2	1.91E+08	1.91E+08	680	-0.74	1.98E-05	0.000536	ANKAR	1.91E+08
chr11	1.02E+08	1.02E+08	314	-1.01	1.98E-05	0.000536	YAP1	1.02E+08
chr11	1.02E+08	1.02E+08	314	-1.01	1.98E-05	0.000536	YAP1	1.02E+08
chr11	1.02E+08	1.02E+08	314	-1.01	1.98E-05	0.000536	YAP1	1.02E+08
chr11	1.02E+08	1.02E+08	314	-1.01	1.98E-05	0.000536	YAP1	1.02E+08
chr11	1.02E+08	1.02E+08	314	-1.01	1.98E-05	0.000536	YAP1	1.02E+08
chr11	1.02E+08	1.02E+08	314	-1.01	1.98E-05	0.000536	YAP1	1.02E+08
chr11	1.02E+08	1.02E+08	314	-1.01	1.98E-05	0.000536	YAP1	1.02E+08
chr11	1.02E+08	1.02E+08	314	-1.01	1.98E-05	0.000536	YAP1	1.02E+08
chr11	1.02E+08	1.02E+08	314	-1.01	1.98E-05	0.000536	YAP1	1.02E+08
chr7	1.44E+08	1.44E+08	892	-0.67	2.05E-05	0.00055	TCAF1	1.44E+08
chr7	1.44E+08	1.44E+08	892	-0.67	2.05E-05	0.00055	TCAF1	1.44E+08
chr11	28129534	28130094	561	-0.72	2.06E-05	0.000551	METTL15	28124797
chr11	28129534	28130094	561	-0.72	2.06E-05	0.000551	METTL15	28124797
chr11	28129534	28130094	561	-0.72	2.06E-05	0.000551	METTL15	28124797
chr11	28129534	28130094	561	-0.72	2.06E-05	0.000551	KIF18A	28127746
chr14	62040810	62041278	469	-1.06	2.07E-05	0.000553	LOC101927	62034912
chr14	62040810	62041278	469	-1.06	2.07E-05	0.000553	LOC101927	62035371
chr2	2.2E+08	2.2E+08	680	-0.67	2.08E-05	0.000554	GLB1L	2.2E+08
chr2	2.2E+08	2.2E+08	680	-0.67	2.08E-05	0.000554	TUBA4B	2.2E+08
chr2	2.2E+08	2.2E+08	680	-0.67	2.08E-05	0.000554	GLB1L	2.2E+08
chr16	54962605	54963655	1051	-0.56	2.09E-05	0.000556	IRX5	54960110
chr16	54962605	54963655	1051	-0.56	2.09E-05	0.000556	IRX5	54960110
chr16	54962605	54963655	1051	-0.56	2.09E-05	0.000556	CRNDE	54960690
chr16	54962605	54963655	1051	-0.56	2.09E-05	0.000556	CRNDE	54961101
chr16	54962605	54963655	1051	-0.56	2.09E-05	0.000556	CRNDE	54960690
chr16	54962605	54963655	1051	-0.56	2.09E-05	0.000556	CRNDE	54960690
chr16	54962605	54963655	1051	-0.56	2.09E-05	0.000556	CRNDE	54961101
chr7	1.13E+08	1.13E+08	572	-0.65	2.09E-05	0.000557	C7orf60	1.13E+08
chr19	45873819	45874132	314	-1.14	2.12E-05	0.000562	ERCC2	45871845
chr19	45873819	45874132	314	-1.14	2.12E-05	0.000562	ERCC2	45871845
chr14	94492513	94492776	264	-0.93	2.15E-05	0.000567	OTUB2	94487723
chr15	86313547	86313975	429	-0.92	2.15E-05	0.000568	MIR1276	86311809
chr3	1.2E+08	1.2E+08	689	-0.66	2.18E-05	0.000574	NDUFB4	1.2E+08

chr3	1.2E+08	1.2E+08	689	-0.66	2.18E-05	0.000574	NDUFB4	1.2E+08
chr19	40476673	40477122	450	-0.84	2.19E-05	0.000576	PSMC4	40471911
chr19	40476673	40477122	450	-0.84	2.19E-05	0.000576	PSMC4	40471911
chr5	14660427	14660758	332	-0.68	2.19E-05	0.000576	OTULIN	14659782
chr20	17539186	17539624	439	-0.86	2.20E-05	0.000576	BFSP1	17537605
chr20	17539186	17539624	439	-0.86	2.20E-05	0.000576	BFSP1	17537605
chr20	17539186	17539624	439	-0.86	2.20E-05	0.000576	BFSP1	17537605
chr5	1.27E+08	1.27E+08	352	-1.13	2.20E-05	0.000577	CCDC192	1.27E+08
chr13	95253799	95254492	694	-0.72	2.22E-05	0.00058	GPR180	95249103
chr2	1.22E+08	1.22E+08	1300	-0.64	2.23E-05	0.000583	RNU4ATAC	1.22E+08
chr9	79520986	79521443	458	-0.73	2.25E-05	0.000585	PRUNE2	79519003
chr9	79520986	79521443	458	-0.73	2.25E-05	0.000585	PRUNE2	79519003
chr9	79520986	79521443	458	-0.73	2.25E-05	0.000585	PRUNE2	79519003
chr9	79520986	79521443	458	-0.73	2.25E-05	0.000585	PRUNE2	79519003
chr5	17431861	17432075	215	-1.41	2.26E-05	0.000589	LOC10192!	17431845
chr5	1.23E+08	1.23E+08	675	-0.72	2.27E-05	0.00059	CEP120	1.23E+08
chr5	1.23E+08	1.23E+08	675	-0.72	2.27E-05	0.00059	CEP120	1.23E+08
chr9	27519498	27520580	1083	-0.76	2.28E-05	0.000591	IFNK	27519311
chr8	1.04E+08	1.04E+08	593	-0.66	2.28E-05	0.000591	AZIN1-AS1	1.04E+08
chr8	1.04E+08	1.04E+08	593	-0.66	2.28E-05	0.000591	AZIN1-AS1	1.04E+08
chr8	1.04E+08	1.04E+08	593	-0.66	2.28E-05	0.000591	AZIN1	1.04E+08
chr8	1.04E+08	1.04E+08	593	-0.66	2.28E-05	0.000591	AZIN1	1.04E+08
chr8	1.04E+08	1.04E+08	593	-0.66	2.28E-05	0.000591	AZIN1	1.04E+08
chr5	74632237	74633078	842	-0.6	2.30E-05	0.000594	HMGCR	74627992
chr5	74632237	74633078	842	-0.6	2.30E-05	0.000594	HMGCR	74627992
chr2	96814258	96814544	287	-1.14	2.32E-05	0.000598	DUSP2	96809179
chr2	54557291	54558126	836	-0.74	2.35E-05	0.000605	C2orf73	54553070
chr9	1.01E+08	1.01E+08	722	-0.61	2.36E-05	0.000607	NANS	1.01E+08
chr8	1.24E+08	1.24E+08	521	-0.73	2.37E-05	0.000608	FAM83A	1.24E+08
chr12	9102093	9102757	665	-0.68	2.37E-05	0.000608	M6PR	9100357
chr12	9102093	9102757	665	-0.68	2.37E-05	0.000608	M6PR	9100357
chr8	66556629	66557556	928	-0.61	2.38E-05	0.00061	MTFR1	66551887
chr8	66556629	66557556	928	-0.61	2.38E-05	0.00061	MTFR1	66551887
chr11	1.28E+08	1.28E+08	719	-0.57	2.39E-05	0.000612	LOC10192!	1.28E+08
chr11	1.28E+08	1.28E+08	719	-0.57	2.39E-05	0.000612	ETS1	1.28E+08
chr11	1.28E+08	1.28E+08	719	-0.57	2.39E-05	0.000612	ETS1	1.28E+08
chr11	1.28E+08	1.28E+08	719	-0.57	2.39E-05	0.000612	MIR6090	1.28E+08
chr11	8835815	8836160	346	-0.84	2.40E-05	0.000614	ST5	8830224
chr11	8835815	8836160	346	-0.84	2.40E-05	0.000614	ST5	8830882
chr5	59788873	59789103	231	-1.52	2.41E-05	0.000617	PDE4D	59781925
chr11	73309020	73309792	773	-0.6	2.46E-05	0.000625	FAM168A	73307234
chr11	73309020	73309792	773	-0.6	2.46E-05	0.000625	FAM168A	73307234
chr11	73309020	73309792	773	-0.6	2.46E-05	0.000625	FAM168A	73307234
chr3	1.31E+08	1.31E+08	545	-0.6	2.47E-05	0.000627	LOC339874	1.31E+08
chr3	1.31E+08	1.31E+08	545	-0.6	2.47E-05	0.000627	NUDT16	1.31E+08
chr3	1.31E+08	1.31E+08	545	-0.6	2.47E-05	0.000627	NUDT16	1.31E+08
chr3	1.31E+08	1.31E+08	545	-0.6	2.47E-05	0.000627	NUDT16	1.31E+08

chr3	1.31E+08	1.31E+08	545	-0.6	2.47E-05	0.000627	NUDT16	1.31E+08
chr12	1.05E+08	1.05E+08	714	-1.03	2.50E-05	0.000633	MIR3922	1.05E+08
chr6	1.23E+08	1.23E+08	562	-0.51	2.54E-05	0.000639	HSF2	1.23E+08
chr6	1.23E+08	1.23E+08	562	-0.51	2.54E-05	0.000639	HSF2	1.23E+08
chr6	1.23E+08	1.23E+08	562	-0.51	2.54E-05	0.000639	HSF2	1.23E+08
chr9	1.1E+08	1.1E+08	721	-0.57	2.55E-05	0.00064	RAD23B	1.1E+08
chr9	1.1E+08	1.1E+08	721	-0.57	2.55E-05	0.00064	RAD23B	1.1E+08
chr9	1.1E+08	1.1E+08	721	-0.57	2.55E-05	0.00064	RAD23B	1.1E+08
chr3	98620004	98620922	919	-0.61	2.55E-05	0.00064	DCBLD2	98618533
chr4	79698004	79698344	341	-1.79	2.55E-05	0.00064	BMP2K	79692531
chr4	79698004	79698344	341	-1.79	2.55E-05	0.00064	BMP2K	79692531
chr9	3525785	3526892	1108	-0.79	2.59E-05	0.000646	RFX3	3524001
chr9	3525785	3526892	1108	-0.79	2.59E-05	0.000646	RFX3	3524001
chr14	54863382	54863862	481	-0.62	2.60E-05	0.000648	CDKN3	54858672
chr14	54863382	54863862	481	-0.62	2.60E-05	0.000648	CDKN3	54858672
chr4	1.02E+08	1.02E+08	207	-1.13	2.61E-05	0.000649	PPP3CA	1.02E+08
chr4	1.02E+08	1.02E+08	207	-1.13	2.61E-05	0.000649	PPP3CA	1.02E+08
chr4	1.02E+08	1.02E+08	207	-1.13	2.61E-05	0.000649	PPP3CA	1.02E+08
chr4	1.02E+08	1.02E+08	207	-1.13	2.61E-05	0.000649	FLJ20021	1.02E+08
chr2	2.17E+08	2.17E+08	290	-1.22	2.63E-05	0.000651	4-Mar	2.17E+08
chr6	88410514	88410797	284	-0.87	2.63E-05	0.000652	AKIRIN2	88409985
chr6	73974096	73974513	418	-0.67	2.64E-05	0.000653	KHDC1	73970919
chr15	89181759	89182401	643	-0.72	2.64E-05	0.000653	ISG20	89176973
chr15	89181759	89182401	643	-0.72	2.64E-05	0.000653	ISG20	89176973
chr15	89181759	89182401	643	-0.72	2.64E-05	0.000653	ISG20	89176973
chr13	28000090	28000542	453	-1	2.65E-05	0.000655	GTF3A	27993680
chr7	7679755	7680639	885	-0.5	2.69E-05	0.000662	UMAD1	7675266
chr7	7679755	7680639	885	-0.5	2.69E-05	0.000662	UMAD1	7675266
chr7	7679755	7680639	885	-0.5	2.69E-05	0.000662	UMAD1	7675266
chr3	1.57E+08	1.57E+08	766	-0.63	2.73E-05	0.000669	LINC00881	1.57E+08
chr13	60737895	60738549	655	-0.73	2.74E-05	0.00067	DIAPH3	60736119
chr13	60737895	60738549	655	-0.73	2.74E-05	0.00067	DIAPH3	60736119
chr13	60737895	60738549	655	-0.73	2.74E-05	0.00067	DIAPH3	60736119
chr13	60737895	60738549	655	-0.73	2.74E-05	0.00067	DIAPH3	60736119
chr13	60737895	60738549	655	-0.73	2.74E-05	0.00067	DIAPH3	60736119
chr1	11322536	11322947	412	-0.72	2.79E-05	0.00068	MTOR	11320608
chr4	48018416	48019549	1134	-0.65	2.79E-05	0.00068	CNGA1	48012961
chr4	48018416	48019549	1134	-0.65	2.79E-05	0.00068	NIPAL1	48013788
chr7	86974503	86975117	615	-0.66	2.80E-05	0.000682	CROT	86969950
chr7	86974503	86975117	615	-0.66	2.80E-05	0.000682	CROT	86969950
chr7	86974503	86975117	615	-0.66	2.80E-05	0.000682	TP53TG1	86972808
chr7	86974503	86975117	615	-0.66	2.80E-05	0.000682	CROT	86969950
chr17	15245933	15246252	320	-1.2	2.83E-05	0.000687	TEKT3	15242958
chr16	80603305	80603824	520	-0.82	2.84E-05	0.000687	LINC01227	80601507
chr8	1.2E+08	1.2E+08	1126	-0.6	2.86E-05	0.000693	NOV	1.2E+08
chr11	69926843	69927266	424	-0.6	2.87E-05	0.000694	ANO1	69926515
chr6	1.05E+08	1.05E+08	645	-0.71	2.90E-05	0.000699	HACE1	1.05E+08

chr6	1.05E+08	1.05E+08	645	-0.71	2.90E-05	0.000699	HACE1	1.05E+08
chr12	9517482	9517840	359	-1.22	2.91E-05	0.000699	LOC10193(	9515059
chr21	26946111	26946508	398	-0.84	2.94E-05	0.000707	MIR155	26941291
chr9	86571184	86571928	745	-0.76	2.95E-05	0.000707	C9orf64	86569657
chr9	86571184	86571928	745	-0.76	2.95E-05	0.000707	C9orf64	86569901
chr14	65409365	65409794	430	-0.72	3.02E-05	0.000721	GPX2	65407623
chr14	65409365	65409794	430	-0.72	3.02E-05	0.000721	GPX2	65407623
chr14	65409365	65409794	430	-0.72	3.02E-05	0.000721	GPX2	65407623
chr7	33943852	33944809	958	-0.8	3.04E-05	0.000725	BMPER	33939522
chr2	1.99E+08	1.99E+08	263	-1.13	3.06E-05	0.000727	LOC101927	1.99E+08
chr3	1.56E+08	1.56E+08	544	-0.51	3.06E-05	0.000727	SLC33A1	1.56E+08
chr3	1.56E+08	1.56E+08	544	-0.51	3.06E-05	0.000727	SLC33A1	1.56E+08
chr7	1.21E+08	1.21E+08	826	-0.62	3.06E-05	0.000727	ING3	1.21E+08
chr7	1.21E+08	1.21E+08	826	-0.62	3.06E-05	0.000727	ING3	1.21E+08
chr11	19138400	19139265	866	-0.7	3.08E-05	0.000728	ZDHHC13	19133691
chr11	19138400	19139265	866	-0.7	3.08E-05	0.000728	ZDHHC13	19133691
chr6	1.56E+08	1.56E+08	790	-0.45	3.08E-05	0.000729	TFB1M	1.56E+08
chr13	42845713	42846159	447	-1.08	3.14E-05	0.000738	AKAP11	42841288
chr4	1.09E+08	1.09E+08	881	-0.57	3.17E-05	0.000744	PAPSS1	1.09E+08
chr12	44152222	44153406	1185	-0.57	3.18E-05	0.000745	IRAK4	44147746
chr12	44152222	44153406	1185	-0.57	3.18E-05	0.000745	IRAK4	44147746
chr12	44152222	44153406	1185	-0.57	3.18E-05	0.000745	IRAK4	44147746
chr12	44152222	44153406	1185	-0.57	3.18E-05	0.000745	IRAK4	44147746
chr12	44152222	44153406	1185	-0.57	3.18E-05	0.000745	IRAK4	44147746
chr12	44152222	44153406	1185	-0.57	3.18E-05	0.000745	PUS7L	44150576
chr12	44152222	44153406	1185	-0.57	3.18E-05	0.000745	PUS7L	44150620
chr12	44152222	44153406	1185	-0.57	3.18E-05	0.000745	PUS7L	44150561
chr12	44152222	44153406	1185	-0.57	3.18E-05	0.000745	PUS7L	44150620
chr4	1.78E+08	1.78E+08	668	-0.61	3.18E-05	0.000746	AGA	1.78E+08
chr4	1.78E+08	1.78E+08	668	-0.61	3.18E-05	0.000746	AGA	1.78E+08
chr4	1.78E+08	1.78E+08	668	-0.61	3.18E-05	0.000746	AGA	1.78E+08
chr7	1.38E+08	1.38E+08	644	-0.63	3.20E-05	0.000748	TRIM24	1.38E+08
chr7	1.38E+08	1.38E+08	644	-0.63	3.20E-05	0.000748	TRIM24	1.38E+08
chr8	1.19E+08	1.19E+08	353	-0.95	3.27E-05	0.000762	EXT1	1.19E+08
chr5	1.5E+08	1.5E+08	479	-0.92	3.27E-05	0.000762	GPX3	1.5E+08
chr1	92496376	92496704	329	-0.74	3.29E-05	0.000765	EPHX4	92490532
chr10	97453506	97454097	592	-0.54	3.30E-05	0.000766	TCTN3	97451900
chr10	97453506	97454097	592	-0.54	3.30E-05	0.000766	TCTN3	97451900
chr12	1.02E+08	1.02E+08	735	-0.79	3.33E-05	0.00077	CHPT1	1.02E+08
chr8	92082190	92082898	709	-0.54	3.41E-05	0.000785	OTUD6B	92077423
chr8	92082190	92082898	709	-0.54	3.41E-05	0.000785	OTUD6B	92077423
chr8	92082190	92082898	709	-0.54	3.41E-05	0.000785	OTUD6B-A!	92080417
chr8	92082190	92082898	709	-0.54	3.41E-05	0.000785	OTUD6B-A!	92080417
chr6	75953136	75953815	680	-0.8	3.48E-05	0.000794	COX7A2	75951644
chr6	75953136	75953815	680	-0.8	3.48E-05	0.000794	COX7A2	75951644
chr13	73301401	73302215	815	-0.61	3.48E-05	0.000794	BORA	73296886
chr13	73301401	73302215	815	-0.61	3.48E-05	0.000794	BORA	73297041

chr13	73301401	73302215	815	-0.61	3.48E-05	0.000794	MZT1	73299938
chr13	73301401	73302215	815	-0.61	3.48E-05	0.000794	BORA	73296886
chr5	1.38E+08	1.38E+08	613	-0.7	3.52E-05	0.000801	CDC25C	1.38E+08
chr5	1.38E+08	1.38E+08	613	-0.7	3.52E-05	0.000801	CDC25C	1.38E+08
chr5	1.38E+08	1.38E+08	613	-0.7	3.52E-05	0.000801	CDC25C	1.38E+08
chr2	2.14E+08	2.14E+08	670	-0.68	3.54E-05	0.000805	SPAG16	2.14E+08
chr2	2.14E+08	2.14E+08	670	-0.68	3.54E-05	0.000805	SPAG16	2.14E+08
chr2	2.14E+08	2.14E+08	670	-0.68	3.54E-05	0.000805	SPAG16	2.14E+08
chr2	2.14E+08	2.14E+08	670	-0.68	3.54E-05	0.000805	SPAG16	2.14E+08
chr2	2.14E+08	2.14E+08	670	-0.68	3.54E-05	0.000805	LOC10013(	2.14E+08
chr11	45114782	45115060	279	-1.11	3.57E-05	0.000809	PRDM11	45110563
chr3	1.49E+08	1.49E+08	555	-0.8	3.59E-05	0.000811	HLTF	1.49E+08
chr3	1.49E+08	1.49E+08	555	-0.8	3.59E-05	0.000811	HLTF	1.49E+08
chr3	1.49E+08	1.49E+08	555	-0.8	3.59E-05	0.000811	HLTF	1.49E+08
chr3	1.49E+08	1.49E+08	555	-0.8	3.59E-05	0.000811	HLTF	1.49E+08
chr3	1.49E+08	1.49E+08	555	-0.8	3.59E-05	0.000811	HLTF-AS1	1.49E+08
chr6	1.07E+08	1.07E+08	598	-0.78	3.59E-05	0.000811	AIM1	1.07E+08
chr10	25348860	25349275	416	-0.7	3.59E-05	0.000811	ENKUR	25349208
chr11	7621562	7621989	428	-0.67	3.65E-05	0.00082	PPFIBP2	7621964
chr11	1.34E+08	1.34E+08	510	-0.62	3.65E-05	0.00082	ACAD8	1.34E+08
chr11	1.34E+08	1.34E+08	510	-0.62	3.65E-05	0.00082	THYN1	1.34E+08
chr11	1.34E+08	1.34E+08	510	-0.62	3.65E-05	0.00082	THYN1	1.34E+08
chr11	1.34E+08	1.34E+08	510	-0.62	3.65E-05	0.00082	THYN1	1.34E+08
chr11	1.34E+08	1.34E+08	510	-0.62	3.65E-05	0.00082	THYN1	1.34E+08
chr11	1.34E+08	1.34E+08	510	-0.62	3.65E-05	0.00082	THYN1	1.34E+08
chr14	77923758	77924461	704	-0.59	3.66E-05	0.000823	VIPAS39	77921983
chr14	77923758	77924461	704	-0.59	3.66E-05	0.000823	VIPAS39	77921983
chr14	77923758	77924461	704	-0.59	3.66E-05	0.000823	VIPAS39	77921983
chr14	77923758	77924461	704	-0.59	3.66E-05	0.000823	AHSA1	77919372
chr14	77923758	77924461	704	-0.59	3.66E-05	0.000823	VIPAS39	77921983
chr14	77923758	77924461	704	-0.59	3.66E-05	0.000823	VIPAS39	77921983
chr3	1.79E+08	1.79E+08	1211	-0.59	3.68E-05	0.000824	PIK3CA	1.79E+08
chr3	1.79E+08	1.79E+08	1211	-0.59	3.68E-05	0.000824	LOC101928	1.79E+08
chr11	1.25E+08	1.25E+08	1267	-0.51	3.68E-05	0.000824	CHEK1	1.25E+08
chr11	1.25E+08	1.25E+08	1267	-0.51	3.68E-05	0.000824	CHEK1	1.25E+08
chr11	1.25E+08	1.25E+08	1267	-0.51	3.68E-05	0.000824	CHEK1	1.25E+08
chr11	1.25E+08	1.25E+08	1267	-0.51	3.68E-05	0.000824	CHEK1	1.25E+08
chr11	1.25E+08	1.25E+08	1267	-0.51	3.68E-05	0.000824	CHEK1	1.25E+08
chr11	1.25E+08	1.25E+08	1267	-0.51	3.68E-05	0.000824	CHEK1	1.25E+08
chr9	1.03E+08	1.03E+08	936	-0.47	3.79E-05	0.000845	LOC101928	1.03E+08
chr9	1.03E+08	1.03E+08	936	-0.47	3.79E-05	0.000845	NR4A3	1.03E+08
chr9	1.03E+08	1.03E+08	936	-0.47	3.79E-05	0.000845	NR4A3	1.03E+08
chr9	1.03E+08	1.03E+08	936	-0.47	3.79E-05	0.000845	NR4A3	1.03E+08
chr11	65149349	65150349	1001	-0.55	3.80E-05	0.000846	SLC25A45	65148145
chr11	65149349	65150349	1001	-0.55	3.80E-05	0.000846	SLC25A45	65149172
chr11	65149349	65150349	1001	-0.55	3.80E-05	0.000846	SLC25A45	65147857
chr11	65149349	65150349	1001	-0.55	3.80E-05	0.000846	SLC25A45	65147857

chr11	65149349	65150349	1001	-0.55	3.80E-05	0.000846	SLC25A45	65148145
chr11	65149349	65150349	1001	-0.55	3.80E-05	0.000846	FRMD8	65149040
chr11	65149349	65150349	1001	-0.55	3.80E-05	0.000846	FRMD8	65149040
chr11	65149349	65150349	1001	-0.55	3.80E-05	0.000846	FRMD8	65149040
chr4	53578281	53579061	781	-0.61	3.80E-05	0.000846	SNORA26	53574415
chr4	53578281	53579061	781	-0.61	3.80E-05	0.000846	MIR4449	53573848
chr4	53578281	53579061	781	-0.61	3.80E-05	0.000846	DANCR	53573620
chr17	19622642	19622893	252	-1.28	3.82E-05	0.000848	SLC47A2	19618043
chr17	19622642	19622893	252	-1.28	3.82E-05	0.000848	SLC47A2	19618043
chr17	19622642	19622893	252	-1.28	3.82E-05	0.000848	SLC47A2	19618043
chr7	14030783	14031288	506	-0.57	3.83E-05	0.000849	ETV1	14024139
chr7	14030783	14031288	506	-0.57	3.83E-05	0.000849	ETV1	14024139
chr7	14030783	14031288	506	-0.57	3.83E-05	0.000849	ETV1	14024139
chr7	14030783	14031288	506	-0.57	3.83E-05	0.000849	ETV1	14028865
chr7	14030783	14031288	506	-0.57	3.83E-05	0.000849	ETV1	14029050
chr7	14030783	14031288	506	-0.57	3.83E-05	0.000849	ETV1	14027642
chr7	14030783	14031288	506	-0.57	3.83E-05	0.000849	ETV1	14027642
chr7	14030783	14031288	506	-0.57	3.83E-05	0.000849	ETV1	14028327
chr5	1.6E+08	1.6E+08	612	-0.52	3.83E-05	0.000851	PWWP2A	1.6E+08
chr5	1.6E+08	1.6E+08	612	-0.52	3.83E-05	0.000851	PWWP2A	1.6E+08
chr5	1.6E+08	1.6E+08	612	-0.52	3.83E-05	0.000851	PWWP2A	1.6E+08
chr6	1.33E+08	1.33E+08	653	-0.77	3.84E-05	0.000851	SNORA33	1.33E+08
chr3	15643008	15643537	530	-0.78	3.88E-05	0.000857	BTD	15638251
chr3	15643008	15643537	530	-0.78	3.88E-05	0.000857	HACL1	15641359
chr3	15643008	15643537	530	-0.78	3.88E-05	0.000857	HACL1	15641359
chr3	15643008	15643537	530	-0.78	3.88E-05	0.000857	HACL1	15641359
chr3	15643008	15643537	530	-0.78	3.88E-05	0.000857	BTD	15638251
chr3	15643008	15643537	530	-0.78	3.88E-05	0.000857	BTD	15638475
chr3	15643008	15643537	530	-0.78	3.88E-05	0.000857	BTD	15638251
chr3	15643008	15643537	530	-0.78	3.88E-05	0.000857	BTD	15637858
chr3	15643008	15643537	530	-0.78	3.88E-05	0.000857	HACL1	15641359
chr3	15643008	15643537	530	-0.78	3.88E-05	0.000857	HACL1	15641359
chr20	21105950	21106815	866	-0.63	3.91E-05	0.000861	KIZ	21101623
chr20	21105950	21106815	866	-0.63	3.91E-05	0.000861	KIZ	21101623
chr20	21105950	21106815	866	-0.63	3.91E-05	0.000861	KIZ	21101623
chr20	21105950	21106815	866	-0.63	3.91E-05	0.000861	KIZ	21101623
chr20	39657042	39657628	587	-0.66	3.93E-05	0.000866	TOP1	39652461
chr10	97054832	97055224	393	-0.68	3.93E-05	0.000866	PDLIM1	97048905
chr15	89089497	89090072	576	-0.61	3.96E-05	0.00087	DET1	89087912
chr15	89089497	89090072	576	-0.61	3.96E-05	0.00087	DET1	89087912
chr15	89089497	89090072	576	-0.61	3.96E-05	0.00087	DET1	89087912
chr11	67795547	67795824	278	-1.04	4.06E-05	0.000887	NDUFS8	67793083
chr11	67795547	67795824	278	-1.04	4.06E-05	0.000887	MIR7113	67795330
chr12	1.2E+08	1.2E+08	599	-0.57	4.07E-05	0.000888	PRKAB1	1.2E+08
chr1	87793868	87794638	771	-0.5	4.14E-05	0.000897	LMO4	87789150
chr13	77565658	77566399	742	-0.77	4.15E-05	0.000899	CLN5	77561058
chr11	6639847	6640875	1029	-0.68	4.17E-05	0.000903	TPP1	6638692

chr2	88990623	88991536	914	-0.59	4.18E-05	0.000903	RPIA	88986175
chr1	1.97E+08	1.97E+08	452	-0.97	4.23E-05	0.000912	CFH	1.97E+08
chr1	1.97E+08	1.97E+08	452	-0.97	4.23E-05	0.000912	CFH	1.97E+08
chr18	12255567	12256020	454	-1.06	4.25E-05	0.000915	CIDEA	12249317
chr18	12255567	12256020	454	-1.06	4.25E-05	0.000915	CIDEA	12249359
chr18	12255567	12256020	454	-1.06	4.25E-05	0.000915	CIDEA	12249359
chr15	89631093	89631791	699	-0.58	4.26E-05	0.000917	ABHD2	89626380
chr15	89631093	89631791	699	-0.58	4.26E-05	0.000917	ABHD2	89626380
chr6	1.11E+08	1.11E+08	843	-0.54	4.28E-05	0.00092	AMD1	1.11E+08
chr6	1.11E+08	1.11E+08	843	-0.54	4.28E-05	0.00092	CDK19	1.11E+08
chr6	1.11E+08	1.11E+08	843	-0.54	4.28E-05	0.00092	CDK19	1.11E+08
chr6	1.11E+08	1.11E+08	843	-0.54	4.28E-05	0.00092	CDK19	1.11E+08
chr6	1.11E+08	1.11E+08	843	-0.54	4.28E-05	0.00092	CDK19	1.11E+08
chr9	1.18E+08	1.18E+08	621	-0.71	4.29E-05	0.00092	TNC	1.18E+08
chr8	95486915	95487526	612	-0.73	4.35E-05	0.00093	RAD54B	95485343
chr8	95486915	95487526	612	-0.73	4.35E-05	0.00093	RAD54B	95485343
chr5	43514814	43515446	633	-0.8	4.43E-05	0.000942	C5orf34	43513273
chr20	20032776	20033552	777	-0.69	4.44E-05	0.000943	CFAP61	20028157
chr20	20032776	20033552	777	-0.69	4.44E-05	0.000943	CFAP61	20032289
chr20	20032776	20033552	777	-0.69	4.44E-05	0.000943	CRNKL1	20031136
chr14	39643951	39644493	543	-0.67	4.48E-05	0.000949	PNN	39639386
chr14	39643951	39644493	543	-0.67	4.48E-05	0.000949	TRAPPC6B	39637634
chr14	39643951	39644493	543	-0.67	4.48E-05	0.000949	TRAPPC6B	39637634
chr14	60558424	60559055	632	-0.56	4.56E-05	0.000959	PCNX4	60553628
chr5	36606554	36607094	541	-0.86	4.56E-05	0.000959	SLC1A3	36601456
chr5	36606554	36607094	541	-0.86	4.56E-05	0.000959	SLC1A3	36601456
chr5	36606554	36607094	541	-0.86	4.56E-05	0.000959	SLC1A3	36601456
chr5	36606554	36607094	541	-0.86	4.56E-05	0.000959	SLC1A3	36601456
chr5	36606554	36607094	541	-0.86	4.56E-05	0.000959	SLC1A3	36601456
chr18	68051413	68052114	702	-0.5	4.61E-05	0.000967	LOC10106(	68049827
chr16	19566560	19567043	484	-0.62	4.61E-05	0.000967	C16orf62	19561736
chr16	19566560	19567043	484	-0.62	4.61E-05	0.000967	C16orf62	19561736
chr5	86416315	86416780	466	-0.81	4.67E-05	0.000976	LOC10192‹	86410964
chr11	1.25E+08	1.25E+08	494	-0.63	4.68E-05	0.000979	TMEM218	1.25E+08
chr11	1.25E+08	1.25E+08	494	-0.63	4.68E-05	0.000979	TMEM218	1.25E+08
chr11	1.25E+08	1.25E+08	494	-0.63	4.68E-05	0.000979	TMEM218	1.25E+08
chr11	1.25E+08	1.25E+08	494	-0.63	4.68E-05	0.000979	TMEM218	1.25E+08
chr11	1.25E+08	1.25E+08	494	-0.63	4.68E-05	0.000979	TMEM218	1.25E+08
chr11	1.25E+08	1.25E+08	494	-0.63	4.68E-05	0.000979	TMEM218	1.25E+08
chr11	1.25E+08	1.25E+08	494	-0.63	4.68E-05	0.000979	TMEM218	1.25E+08
chr11	1.25E+08	1.25E+08	494	-0.63	4.68E-05	0.000979	TMEM218	1.25E+08
chr11	1.25E+08	1.25E+08	494	-0.63	4.68E-05	0.000979	TMEM218	1.25E+08
chr11	1.25E+08	1.25E+08	494	-0.63	4.68E-05	0.000979	TMEM218	1.25E+08
chr11	1.25E+08	1.25E+08	494	-0.63	4.68E-05	0.000979	TMEM218	1.25E+08
chr11	1.25E+08	1.25E+08	494	-0.63	4.68E-05	0.000979	TMEM218	1.25E+08
chr11	1.25E+08	1.25E+08	494	-0.63	4.68E-05	0.000979	TMEM218	1.25E+08
chr11	1.25E+08	1.25E+08	494	-0.63	4.68E-05	0.000979	TMEM218	1.25E+08

chr11	1.25E+08	1.25E+08	494	-0.63	4.68E-05	0.000979	TMEM218	1.25E+08
chr11	1.25E+08	1.25E+08	494	-0.63	4.68E-05	0.000979	TMEM218	1.25E+08
chr11	1.25E+08	1.25E+08	494	-0.63	4.68E-05	0.000979	TMEM218	1.25E+08
chr11	1.25E+08	1.25E+08	494	-0.63	4.68E-05	0.000979	TMEM218	1.25E+08
chr16	1832496	1833041	546	-0.65	4.69E-05	0.000979	NUBP2	1827923
chr16	1832496	1833041	546	-0.65	4.69E-05	0.000979	NUBP2	1827923
chr16	1832496	1833041	546	-0.65	4.69E-05	0.000979	NUBP2	1827923
chr16	1832496	1833041	546	-0.65	4.69E-05	0.000979	NUBP2	1827923
chr16	1832496	1833041	546	-0.65	4.69E-05	0.000979	SPSB3	1830581
chr12	10364820	10365504	685	-0.83	4.71E-05	0.000981	GABARAPL	10360488
chr6	1.6E+08	1.6E+08	677	-0.61	4.75E-05	0.000986	LOC10012S	1.6E+08
chr6	1.6E+08	1.6E+08	677	-0.61	4.75E-05	0.000986	ACAT2	1.6E+08
chr6	1.6E+08	1.6E+08	677	-0.61	4.75E-05	0.000986	ACAT2	1.6E+08
chr9	15552505	15553468	964	-0.75	4.77E-05	0.000988	CCDC171	15548096
chr12	1.04E+08	1.04E+08	574	-0.76	4.80E-05	0.000994	TDG	1.04E+08
chr16	77224371	77225137	767	-0.57	4.84E-05	0.000999	MON1B	77219815
chr16	77224371	77225137	767	-0.57	4.84E-05	0.000999	MON1B	77219815
chr16	77224371	77225137	767	-0.57	4.84E-05	0.000999	MON1B	77219815
chr12	1.17E+08	1.17E+08	422	-0.89	4.90E-05	0.00101	MIR4472-2	1.17E+08
chr7	1607049	1607329	281	-0.98	4.91E-05	0.00101	PSMG3-AS:	1604708
chr7	1607049	1607329	281	-0.98	4.91E-05	0.00101	PSMG3-AS:	1604708
chr7	1607049	1607329	281	-0.98	4.91E-05	0.00101	PSMG3-AS:	1604708
chr10	14995709	14996551	843	-0.65	4.92E-05	0.00101	DCLRE1C	14994431
chr10	14995709	14996551	843	-0.65	4.92E-05	0.00101	DCLRE1C	14994431
chr10	14995709	14996551	843	-0.65	4.92E-05	0.00101	DCLRE1C	14994431
chr10	14995709	14996551	843	-0.65	4.92E-05	0.00101	MEIG1	14996437
chr10	14995709	14996551	843	-0.65	4.92E-05	0.00101	DCLRE1C	14994431
chr10	14995709	14996551	843	-0.65	4.92E-05	0.00101	DCLRE1C	14994431
chr10	14995709	14996551	843	-0.65	4.92E-05	0.00101	DCLRE1C	14994431
chr10	14995709	14996551	843	-0.65	4.92E-05	0.00101	DCLRE1C	14994431
chr10	14995709	14996551	843	-0.65	4.92E-05	0.00101	DCLRE1C	14994431
chr10	14995709	14996551	843	-0.65	4.92E-05	0.00101	DCLRE1C	14994431
chr3	4344910	4345610	701	-0.84	4.92E-05	0.00101	SETMAR	4339987
chr3	4344910	4345610	701	-0.84	4.92E-05	0.00101	SETMAR	4339987
chr3	4344910	4345610	701	-0.84	4.92E-05	0.00101	SETMAR	4340296
chr3	4344910	4345610	701	-0.84	4.92E-05	0.00101	SETMAR	4339987
chr3	4344910	4345610	701	-0.84	4.92E-05	0.00101	SETMAR	4339987
chr1	1.93E+08	1.93E+08	956	-0.52	4.98E-05	0.00101	TROVE2	1.93E+08
chr1	1.93E+08	1.93E+08	956	-0.52	4.98E-05	0.00101	TROVE2	1.93E+08
chr1	1.93E+08	1.93E+08	956	-0.52	4.98E-05	0.00101	TROVE2	1.93E+08
chr1	1.93E+08	1.93E+08	956	-0.52	4.98E-05	0.00101	TROVE2	1.93E+08
chr1	1.93E+08	1.93E+08	956	-0.52	4.98E-05	0.00101	TROVE2	1.93E+08
chr1	1.93E+08	1.93E+08	956	-0.52	4.98E-05	0.00101	UCHL5	1.93E+08
chr1	1.93E+08	1.93E+08	956	-0.52	4.98E-05	0.00101	UCHL5	1.93E+08
chr1	1.93E+08	1.93E+08	956	-0.52	4.98E-05	0.00101	UCHL5	1.93E+08
chr1	1.93E+08	1.93E+08	956	-0.52	4.98E-05	0.00101	TROVE2	1.93E+08
chr1	1.93E+08	1.93E+08	956	-0.52	4.98E-05	0.00101	UCHL5	1.93E+08

chr1	1.93E+08	1.93E+08	956	-0.52	4.98E-05	0.00101	UCHL5	1.93E+08
chr20	13619255	13619950	696	-0.58	4.98E-05	0.00101	TASP1	13617583
chr17	59530990	59531180	191	-1.92	5.01E-05	0.00102	TBX4	59528806
chr8	95907347	95908214	868	-0.62	5.02E-05	0.00102	CCNE2	95905482
chr3	28282783	28283304	522	-0.78	5.02E-05	0.00102	CMC1	28278123
chr7	26904002	26904694	693	-0.67	5.03E-05	0.00102	SKAP2	26902362
chr19	56652022	56652645	624	-0.58	5.05E-05	0.00102	ZNF444	56647534
chr19	56652022	56652645	624	-0.58	5.05E-05	0.00102	ZNF444	56647534
chr15	1.01E+08	1.01E+08	603	-0.61	5.10E-05	0.00103	ASB7	1.01E+08
chr15	1.01E+08	1.01E+08	603	-0.61	5.10E-05	0.00103	ASB7	1.01E+08
chr15	1.01E+08	1.01E+08	603	-0.61	5.10E-05	0.00103	LINS1	1.01E+08
chr7	33168688	33169316	629	-0.52	5.11E-05	0.00103	BBS9	33164151
chr7	33168688	33169316	629	-0.52	5.11E-05	0.00103	BBS9	33164151
chr7	33168688	33169316	629	-0.52	5.11E-05	0.00103	BBS9	33164151
chr7	33168688	33169316	629	-0.52	5.11E-05	0.00103	BBS9	33164151
chr1	98519422	98520076	655	-0.58	5.12E-05	0.00103	MIR137HG	98513249
chr16	81129633	81130310	678	-0.68	5.12E-05	0.00103	GCSH	81127980
chr16	81129633	81130310	678	-0.68	5.12E-05	0.00103	GCSH	81127980
chr20	23030252	23030894	643	-0.65	5.18E-05	0.00104	THBD	23028301
chr11	19266196	19266536	341	-0.85	5.21E-05	0.00104	E2F8	19261167
chr11	19266196	19266536	341	-0.85	5.21E-05	0.00104	E2F8	19261202
chr11	19266196	19266536	341	-0.85	5.21E-05	0.00104	E2F8	19260507
chr7	90893469	90894158	690	-0.66	5.21E-05	0.00105	FZD1	90888782
chr11	1.07E+08	1.07E+08	548	-0.86	5.22E-05	0.00105	GUCY1A2	1.07E+08
chr11	1.07E+08	1.07E+08	548	-0.86	5.22E-05	0.00105	GUCY1A2	1.07E+08
chr3	1.85E+08	1.85E+08	526	-0.68	5.22E-05	0.00105	SENP2	1.85E+08
chr1	59245479	59245901	423	-1.06	5.24E-05	0.00105	LINC01135	59245822
chr1	59245479	59245901	423	-1.06	5.24E-05	0.00105	LINC01135	59245822
chr1	59245479	59245901	423	-1.06	5.24E-05	0.00105	LINC01135	59245822
chr10	52381698	52382055	358	-0.77	5.25E-05	0.00105	SGMS1	52381737
chr10	52381698	52382055	358	-0.77	5.25E-05	0.00105	SGMS1-AS:	52379710
chr6	5003719	5004457	739	-0.66	5.26E-05	0.00105	LYRM4-AS1	4999049
chr6	5003719	5004457	739	-0.66	5.26E-05	0.00105	LYRM4-AS1	4999049
chr6	5003719	5004457	739	-0.66	5.26E-05	0.00105	LYRM4-AS1	4999049
chr6	5003719	5004457	739	-0.66	5.26E-05	0.00105	RPP40	5002297
chr6	5003719	5004457	739	-0.66	5.26E-05	0.00105	RPP40	5002297
chr6	5003719	5004457	739	-0.66	5.26E-05	0.00105	RPP40	5002297
chr10	1.02E+08	1.02E+08	343	-1.04	5.32E-05	0.00106	PAX2	1.02E+08
chr18	34408432	34409515	1084	-0.49	5.34E-05	0.00106	KIAA1328	34404079
chr18	34408432	34409515	1084	-0.49	5.34E-05	0.00106	TPGS2	34407179
chr18	34408432	34409515	1084	-0.49	5.34E-05	0.00106	TPGS2	34407179
chr18	34408432	34409515	1084	-0.49	5.34E-05	0.00106	TPGS2	34407179
chr18	34408432	34409515	1084	-0.49	5.34E-05	0.00106	TPGS2	34407179
chr18	34408432	34409515	1084	-0.49	5.34E-05	0.00106	TPGS2	34407179
chr18	34408432	34409515	1084	-0.49	5.34E-05	0.00106	TPGS2	34407179
chr18	34408432	34409515	1084	-0.49	5.34E-05	0.00106	TPGS2	34407179
chr18	34408432	34409515	1084	-0.49	5.34E-05	0.00106	TPGS2	34407179

chr18	34408432	34409515	1084	-0.49	5.34E-05	0.00106	TPGS2	34407179
chr18	29666851	29667150	300	-1.05	5.35E-05	0.00106	RNF138	29666817
chr5	1.54E+08	1.54E+08	846	-0.7	5.36E-05	0.00106	MIR378H	1.54E+08
chr14	91526735	91527550	816	-0.5	5.41E-05	0.00107	RPS6KA5	91524993
chr14	91526735	91527550	816	-0.5	5.41E-05	0.00107	RPS6KA5	91524993
chr8	37350721	37351740	1020	-0.59	5.41E-05	0.00107	LINC01605	37349431
chr16	46917836	46918352	517	-0.57	5.44E-05	0.00108	GPT2	46914025
chr16	46917836	46918352	517	-0.57	5.44E-05	0.00108	GPT2	46913291
chr13	1.04E+08	1.04E+08	613	-0.74	5.46E-05	0.00108	MIR548AS	1.04E+08
chr5	32312481	32313471	991	-0.51	5.49E-05	0.00108	MTMR12	32311114
chr5	32312481	32313471	991	-0.51	5.49E-05	0.00108	MTMR12	32311114
chr5	32312481	32313471	991	-0.51	5.49E-05	0.00108	MTMR12	32311114
chr1	86173860	86174444	585	-0.8	5.54E-05	0.00109	ZNHIT6	86172116
chr1	86173860	86174444	585	-0.8	5.54E-05	0.00109	ZNHIT6	86172116
chr10	54230084	54230378	295	-1.26	5.55E-05	0.00109	LINC01468	54228293
chr11	64102559	64103250	692	-0.64	5.60E-05	0.0011	CCDC88B	64102689
chr8	1.24E+08	1.24E+08	995	-0.66	5.60E-05	0.0011	DERL1	1.24E+08
chr8	1.24E+08	1.24E+08	995	-0.66	5.60E-05	0.0011	DERL1	1.24E+08
chr3	94654946	94655436	491	-0.8	5.63E-05	0.0011	LINC00879	94652106
chr5	1.15E+08	1.15E+08	1217	-0.59	5.65E-05	0.0011	TRIM36	1.15E+08
chr6	1.55E+08	1.55E+08	762	-0.67	5.69E-05	0.00111	CNKSR3	1.55E+08
chr7	36192234	36193020	787	-0.52	5.70E-05	0.00111	EEPD1	36187835
chr19	9945558	9946023	466	-0.69	5.77E-05	0.00112	PIN1	9940882
chr19	9945558	9946023	466	-0.69	5.77E-05	0.00112	PIN1	9940882
chr19	9945558	9946023	466	-0.69	5.77E-05	0.00112	PIN1	9940882
chr2	14772581	14772962	382	-0.74	5.84E-05	0.00113	FAM84A	14767809
chr2	14772581	14772962	382	-0.74	5.84E-05	0.00113	LOC653602	14770214
chr1	85156032	85156486	455	-0.97	5.84E-05	0.00113	SSX2IP	85154151
chr1	85156032	85156486	455	-0.97	5.84E-05	0.00113	SSX2IP	85154151
chr1	85156032	85156486	455	-0.97	5.84E-05	0.00113	SSX2IP	85154240
chr1	85156032	85156486	455	-0.97	5.84E-05	0.00113	SSX2IP	85154240
chr1	85156032	85156486	455	-0.97	5.84E-05	0.00113	SSX2IP	85154240
chr16	54964385	54965069	685	-0.65	5.85E-05	0.00113	IRX5	54960110
chr16	54964385	54965069	685	-0.65	5.85E-05	0.00113	IRX5	54960110
chr16	54964385	54965069	685	-0.65	5.85E-05	0.00113	CRNDE	54960690
chr16	54964385	54965069	685	-0.65	5.85E-05	0.00113	CRNDE	54961101
chr16	54964385	54965069	685	-0.65	5.85E-05	0.00113	CRNDE	54960690
chr16	54964385	54965069	685	-0.65	5.85E-05	0.00113	CRNDE	54960690
chr16	54964385	54965069	685	-0.65	5.85E-05	0.00113	CRNDE	54961101
chr6	87864656	87865437	782	-0.56	5.88E-05	0.00114	ZNF292	87860268
chr2	1.76E+08	1.76E+08	989	-0.53	5.90E-05	0.00114	ATF2	1.76E+08
chr2	1.76E+08	1.76E+08	989	-0.53	5.90E-05	0.00114	ATF2	1.76E+08
chr2	1.76E+08	1.76E+08	989	-0.53	5.90E-05	0.00114	ATF2	1.76E+08
chr2	1.76E+08	1.76E+08	989	-0.53	5.90E-05	0.00114	ATF2	1.76E+08
chr2	1.76E+08	1.76E+08	989	-0.53	5.90E-05	0.00114	ATF2	1.76E+08
chr2	1.76E+08	1.76E+08	989	-0.53	5.90E-05	0.00114	ATF2	1.76E+08
chr2	1.76E+08	1.76E+08	989	-0.53	5.90E-05	0.00114	ATF2	1.76E+08

chr2	1.76E+08	1.76E+08	989	-0.53	5.90E-05	0.00114	ATF2	1.76E+08
chr2	1.76E+08	1.76E+08	989	-0.53	5.90E-05	0.00114	ATF2	1.76E+08
chr2	1.76E+08	1.76E+08	989	-0.53	5.90E-05	0.00114	ATF2	1.76E+08
chr2	1.76E+08	1.76E+08	989	-0.53	5.90E-05	0.00114	ATF2	1.76E+08
chr2	1.76E+08	1.76E+08	989	-0.53	5.90E-05	0.00114	ATF2	1.76E+08
chr2	1.76E+08	1.76E+08	989	-0.53	5.90E-05	0.00114	ATF2	1.76E+08
chr2	1.76E+08	1.76E+08	989	-0.53	5.90E-05	0.00114	MIR933	1.76E+08
chr13	77600507	77601691	1185	-0.67	5.97E-05	0.00115	FBXL3	77599331
chr1	2.42E+08	2.42E+08	526	-0.69	5.98E-05	0.00115	FH	2.42E+08
chr20	20693112	20693738	627	-0.59	5.98E-05	0.00115	RALGAPA2	20691266
chrX	1.32E+08	1.32E+08	938	-0.77	5.98E-05	0.00115	MBNL3	1.32E+08
chr6	90539230	90539903	674	-0.59	6.00E-05	0.00116	CASP8AP2	90534618
chr6	90539230	90539903	674	-0.59	6.00E-05	0.00116	CASP8AP2	90534618
chr6	90539230	90539903	674	-0.59	6.00E-05	0.00116	CASP8AP2	90534618
chr1	2.47E+08	2.47E+08	600	-0.66	6.02E-05	0.00116	ZNF670	2.47E+08
chr1	2.47E+08	2.47E+08	600	-0.66	6.02E-05	0.00116	ZNF670-ZN	2.47E+08
chr1	2.47E+08	2.47E+08	600	-0.66	6.02E-05	0.00116	ZNF670	2.47E+08
chr13	60970241	60971324	1084	-0.58	6.02E-05	0.00116	TDRD3	60966426
chr13	60970241	60971324	1084	-0.58	6.02E-05	0.00116	TDRD3	60965590
chr13	60970241	60971324	1084	-0.58	6.02E-05	0.00116	TDRD3	60965590
chr9	77642666	77643629	964	-0.55	6.04E-05	0.00116	CARNMT1	77641310
chr6	73933937	73934380	444	-0.87	6.04E-05	0.00116	KHDC1L	73933175
chr12	95012793	95013041	249	-1.17	6.07E-05	0.00116	TMCC3	95007927
chr15	85923438	85923940	503	-0.87	6.08E-05	0.00116	AKAP13	85918846
chr15	85923438	85923940	503	-0.87	6.08E-05	0.00116	AKAP13	85918846
chr15	85923438	85923940	503	-0.87	6.08E-05	0.00116	MIR7706	85918826
chr19	47551842	47552155	314	-0.98	6.21E-05	0.00119	TMEM160	47549882
chr8	1.44E+08	1.44E+08	658	-0.62	6.22E-05	0.00119	LY6D	1.44E+08
chr3	1.05E+08	1.05E+08	681	-0.47	6.24E-05	0.00119	ALCAM	1.05E+08
chr3	1.05E+08	1.05E+08	681	-0.47	6.24E-05	0.00119	ALCAM	1.05E+08
chr3	1.05E+08	1.05E+08	681	-0.47	6.24E-05	0.00119	ALCAM	1.05E+08
chr3	1.05E+08	1.05E+08	681	-0.47	6.24E-05	0.00119	ALCAM	1.05E+08
chr11	17297914	17298421	508	-0.63	6.24E-05	0.00119	NUCB2	17293285
chr6	1.46E+08	1.46E+08	549	-0.67	6.29E-05	0.0012	SHPRH	1.46E+08
chr6	1.46E+08	1.46E+08	549	-0.67	6.29E-05	0.0012	SHPRH	1.46E+08
chr8	82598162	82598759	598	-0.85	6.30E-05	0.0012	IMPA1	82596589
chr8	82598162	82598759	598	-0.85	6.30E-05	0.0012	IMPA1	82596589
chr8	82598162	82598759	598	-0.85	6.30E-05	0.0012	IMPA1	82596589
chr12	31812067	31812562	496	-0.69	6.31E-05	0.0012	ETFBKMT	31807608
chr12	31812067	31812562	496	-0.69	6.31E-05	0.0012	ETFBKMT	31807120
chr7	39664179	39664279	101	-1.54	6.32E-05	0.0012	RALA	39658151
chr15	78286401	78287087	687	-0.74	6.40E-05	0.00121	LOC91450	78284567
chr9	22008719	22009614	896	-0.85	6.41E-05	0.00121	CDKN2B	22007312
chr9	22008719	22009614	896	-0.85	6.41E-05	0.00121	CDKN2B	22007312
chr16	23568493	23569140	648	-0.58	6.48E-05	0.00122	EARS2	23566696
chr16	23568493	23569140	648	-0.58	6.48E-05	0.00122	UBFD1	23563861
chr16	23568493	23569140	648	-0.58	6.48E-05	0.00122	EARS2	23566696

chr16	23568493	23569140	648	-0.58	6.48E-05	0.00122	EARS2	23566696
chr3	1.33E+08	1.33E+08	589	-0.51	6.48E-05	0.00122	CDV3	1.33E+08
chr3	1.33E+08	1.33E+08	589	-0.51	6.48E-05	0.00122	CDV3	1.33E+08
chr3	1.33E+08	1.33E+08	589	-0.51	6.48E-05	0.00122	CDV3	1.33E+08
chr3	1.33E+08	1.33E+08	589	-0.51	6.48E-05	0.00122	CDV3	1.33E+08
chr3	1.33E+08	1.33E+08	589	-0.51	6.48E-05	0.00122	CDV3	1.33E+08
chr3	1.33E+08	1.33E+08	589	-0.51	6.48E-05	0.00122	CDV3	1.33E+08
chr3	1.33E+08	1.33E+08	589	-0.51	6.48E-05	0.00122	CDV3	1.33E+08
chr3	1.12E+08	1.12E+08	751	-0.83	6.55E-05	0.00123	PHLDB2	1.12E+08
chr3	1.12E+08	1.12E+08	751	-0.83	6.55E-05	0.00123	PHLDB2	1.12E+08
chr3	1.12E+08	1.12E+08	751	-0.83	6.55E-05	0.00123	PHLDB2	1.12E+08
chr7	22707369	22707895	527	-0.83	6.57E-05	0.00123	LOC401312	22703152
chr18	51750862	51751243	382	-1.01	6.62E-05	0.00124	MBD2	51749158
chr18	51750862	51751243	382	-1.01	6.62E-05	0.00124	MBD2	51749158
chr18	51750862	51751243	382	-1.01	6.62E-05	0.00124	SNORA37	51746782
chr3	1.42E+08	1.42E+08	796	-0.69	6.63E-05	0.00124	GK5	1.42E+08
chr3	1.42E+08	1.42E+08	796	-0.69	6.63E-05	0.00124	GK5	1.42E+08
chr4	99064078	99064852	775	-0.65	6.65E-05	0.00124	STPG2	99062391
chr20	16710372	16710997	626	-0.66	6.68E-05	0.00124	SNRPB2	16705608
chr20	16710372	16710997	626	-0.66	6.68E-05	0.00124	SNRPB2	16705608
chr11	95522650	95523719	1070	-0.6	6.70E-05	0.00125	CEP57	95518624
chr11	95522650	95523719	1070	-0.6	6.70E-05	0.00125	CEP57	95518624
chr11	95522650	95523719	1070	-0.6	6.70E-05	0.00125	CEP57	95518624
chr11	95522650	95523719	1070	-0.6	6.70E-05	0.00125	FAM76B	95520954
chr7	48124453	48124833	381	-0.66	6.71E-05	0.00125	UPP1	48123199
chr7	48124453	48124833	381	-0.66	6.71E-05	0.00125	UPP1	48123808
chr7	48124453	48124833	381	-0.66	6.71E-05	0.00125	UPP1	48123199
chr7	48124453	48124833	381	-0.66	6.71E-05	0.00125	UPP1	48123850
chr7	48124453	48124833	381	-0.66	6.71E-05	0.00125	UPP1	48123736
chr7	48124453	48124833	381	-0.66	6.71E-05	0.00125	UPP1	48123850
chr1	1.75E+08	1.75E+08	352	-0.62	6.75E-05	0.00125	KIAA0040	1.75E+08
chr1	1.75E+08	1.75E+08	352	-0.62	6.75E-05	0.00125	KIAA0040	1.75E+08
chr1	1.75E+08	1.75E+08	352	-0.62	6.75E-05	0.00125	KIAA0040	1.75E+08
chr1	1.75E+08	1.75E+08	352	-0.62	6.75E-05	0.00125	KIAA0040	1.75E+08
chr4	1.29E+08	1.29E+08	996	-0.67	6.75E-05	0.00125	PLK4	1.29E+08
chr4	1.29E+08	1.29E+08	996	-0.67	6.75E-05	0.00125	PLK4	1.29E+08
chr4	1.29E+08	1.29E+08	996	-0.67	6.75E-05	0.00125	PLK4	1.29E+08
chr2	1.91E+08	1.91E+08	923	-0.82	6.77E-05	0.00126	OSGEPL1-A	1.91E+08
chr2	1.91E+08	1.91E+08	923	-0.82	6.77E-05	0.00126	OSGEPL1	1.91E+08
chr3	1.56E+08	1.56E+08	713	-0.52	6.80E-05	0.00126	TIPARP	1.56E+08
chr3	1.56E+08	1.56E+08	713	-0.52	6.80E-05	0.00126	TIPARP	1.56E+08
chr3	1.56E+08	1.56E+08	713	-0.52	6.80E-05	0.00126	TIPARP	1.56E+08
chr3	1.56E+08	1.56E+08	713	-0.52	6.80E-05	0.00126	TIPARP-AS1	1.56E+08
chr11	77530472	77530668	197	-1.92	6.83E-05	0.00127	AAMDC	77527159
chr11	77530472	77530668	197	-1.92	6.83E-05	0.00127	AAMDC	77527159
chr11	77530472	77530668	197	-1.92	6.83E-05	0.00127	AAMDC	77527159
chr11	77530472	77530668	197	-1.92	6.83E-05	0.00127	RSF1	77529880

chr11	77530472	77530668	197	-1.92	6.83E-05	0.00127	AAMDC	77527159
chr11	77530472	77530668	197	-1.92	6.83E-05	0.00127	AAMDC	77527159
chr11	77530472	77530668	197	-1.92	6.83E-05	0.00127	AAMDC	77527159
chr11	77530472	77530668	197	-1.92	6.83E-05	0.00127	AAMDC	77527159
chr12	1.04E+08	1.04E+08	866	-0.61	6.86E-05	0.00127	HSP90B1	1.04E+08
chr12	1.04E+08	1.04E+08	866	-0.61	6.86E-05	0.00127	MIR3652	1.04E+08
chr12	1.04E+08	1.04E+08	866	-0.61	6.86E-05	0.00127	TTC41P	1.04E+08
chr3	1.14E+08	1.14E+08	762	-0.54	6.90E-05	0.00127	CCDC191	1.14E+08
chr3	1.14E+08	1.14E+08	762	-0.54	6.90E-05	0.00127	QTRTD1	1.14E+08
chr3	1.14E+08	1.14E+08	762	-0.54	6.90E-05	0.00127	QTRTD1	1.14E+08
chr3	1.14E+08	1.14E+08	762	-0.54	6.90E-05	0.00127	QTRTD1	1.14E+08
chr3	1.14E+08	1.14E+08	762	-0.54	6.90E-05	0.00127	QTRTD1	1.14E+08
chr10	82171545	82171878	334	-0.99	6.92E-05	0.00128	FAM213A	82168574
chr10	82171545	82171878	334	-0.99	6.92E-05	0.00128	FAM213A	82168574
chr8	1.46E+08	1.46E+08	402	-0.69	6.94E-05	0.00128	LRRC14	1.46E+08
chr8	1.46E+08	1.46E+08	402	-0.69	6.94E-05	0.00128	LRRC14	1.46E+08
chr15	77712415	77712824	410	-0.7	7.02E-05	0.00129	PEAK1	77710446
chr15	77712415	77712824	410	-0.7	7.02E-05	0.00129	HMG20A	77707992
chr15	77712415	77712824	410	-0.7	7.02E-05	0.00129	HMG20A	77707992
chr15	77712415	77712824	410	-0.7	7.02E-05	0.00129	HMG20A	77707992
chr3	1.35E+08	1.35E+08	780	-0.56	7.10E-05	0.0013	EPHB1	1.35E+08
chr11	1.29E+08	1.29E+08	1075	-0.68	7.15E-05	0.0013	TP53AIP1	1.29E+08
chr11	1.29E+08	1.29E+08	1075	-0.68	7.15E-05	0.0013	TP53AIP1	1.29E+08
chr11	1.29E+08	1.29E+08	1075	-0.68	7.15E-05	0.0013	TP53AIP1	1.29E+08
chr11	1.29E+08	1.29E+08	1075	-0.68	7.15E-05	0.0013	TP53AIP1	1.29E+08
chr5	6638433	6638600	168	-1.31	7.16E-05	0.0013	NSUN2	6631473
chr5	6638433	6638600	168	-1.31	7.16E-05	0.0013	NSUN2	6631473
chr5	6638433	6638600	168	-1.31	7.16E-05	0.0013	NSUN2	6631473
chr8	37378875	37379315	441	-0.84	7.17E-05	0.0013	LINC01605	37376904
chr11	68776224	68776607	384	-0.62	7.17E-05	0.0013	MRGPRF-A	68774821
chr20	25677118	25677729	612	-0.59	7.18E-05	0.0013	ZNF337	25675540
chr3	1.08E+08	1.08E+08	848	-0.64	7.21E-05	0.00131	DZIP3	1.08E+08
chr3	1.08E+08	1.08E+08	848	-0.64	7.21E-05	0.00131	KIAA1524	1.08E+08
chr11	13030246	13030963	718	-0.74	7.24E-05	0.00131	RASSF10	13025969
chr1	1.73E+08	1.73E+08	857	-0.53	7.26E-05	0.00132	SUCO	1.72E+08
chr1	1.73E+08	1.73E+08	857	-0.53	7.26E-05	0.00132	SUCO	1.72E+08
chr1	1.73E+08	1.73E+08	857	-0.53	7.26E-05	0.00132	SUCO	1.72E+08
chr1	1.73E+08	1.73E+08	857	-0.53	7.26E-05	0.00132	SUCO	1.72E+08
chr4	68566403	68567158	756	-0.85	7.30E-05	0.00132	UBA6	68564889
chr4	68566403	68567158	756	-0.85	7.30E-05	0.00132	UBA6-AS1	68561995
chr12	88535584	88536269	686	-0.63	7.33E-05	0.00133	CEP290	88533993
chr12	88535584	88536269	686	-0.63	7.33E-05	0.00133	TMTC3	88531072
chr10	63808895	63809196	302	-0.86	7.38E-05	0.00133	ARID5B	63803969
chr2	25194502	25195047	546	-0.78	7.44E-05	0.00134	DNAJC27	25192963
chr2	25194502	25195047	546	-0.78	7.44E-05	0.00134	DNAJC27	25192963
chr2	25194502	25195047	546	-0.78	7.44E-05	0.00134	DNAJC27-A	25189980
chr1	91966194	91967208	1015	-0.48	7.44E-05	0.00134	CDC7	91961664

chr1	91966194	91967208	1015	-0.48	7.44E-05	0.00134	CDC7	91961403
chr1	91966194	91967208	1015	-0.48	7.44E-05	0.00134	CDC7	91961403
chr3	1.81E+08	1.81E+08	552	-0.53	7.45E-05	0.00134	FXR1	1.81E+08
chr3	1.81E+08	1.81E+08	552	-0.53	7.45E-05	0.00134	FXR1	1.81E+08
chr3	1.81E+08	1.81E+08	552	-0.53	7.45E-05	0.00134	FXR1	1.81E+08
chr1	1.13E+08	1.13E+08	623	-0.58	7.54E-05	0.00135	RHOC	1.13E+08
chr1	1.13E+08	1.13E+08	623	-0.58	7.54E-05	0.00135	RHOC	1.13E+08
chr1	1.13E+08	1.13E+08	623	-0.58	7.54E-05	0.00135	RHOC	1.13E+08
chr9	1.24E+08	1.24E+08	198	-1.11	7.55E-05	0.00135	DAB2IP	1.24E+08
chr5	1.07E+08	1.07E+08	1070	-0.68	7.60E-05	0.00136	EFNA5	1.07E+08
chr9	67292928	67294101	1174	-0.58	7.65E-05	0.00137	AQP7P1	67287492
chr1	1.7E+08	1.7E+08	658	-1.04	7.75E-05	0.00138	SELL	1.7E+08
chr1	1.7E+08	1.7E+08	658	-1.04	7.75E-05	0.00138	SELL	1.7E+08
chr1	90098255	90099144	890	-0.57	7.75E-05	0.00138	LRRC8C	90093643
chr1	90098255	90099144	890	-0.57	7.75E-05	0.00138	FLJ27354	90096453
chr19	13001609	13002090	482	-0.51	7.98E-05	0.00141	GCDH	12996942
chr19	13001609	13002090	482	-0.51	7.98E-05	0.00141	GCDH	12996942
chr19	13001609	13002090	482	-0.51	7.98E-05	0.00141	KLF1	12996017
chr19	13001609	13002090	482	-0.51	7.98E-05	0.00141	GCDH	12996942
chr19	13001609	13002090	482	-0.51	7.98E-05	0.00141	GCDH	12996942
chr9	1.26E+08	1.26E+08	994	-0.52	8.00E-05	0.00141	RC3H2	1.26E+08
chr9	1.26E+08	1.26E+08	994	-0.52	8.00E-05	0.00141	RC3H2	1.26E+08
chr1	1.93E+08	1.93E+08	1067	-0.58	8.02E-05	0.00142	GLRX2	1.93E+08
chr1	1.93E+08	1.93E+08	1067	-0.58	8.02E-05	0.00142	GLRX2	1.93E+08
chr1	1.93E+08	1.93E+08	1067	-0.58	8.02E-05	0.00142	GLRX2	1.93E+08
chr1	45965608	45966031	424	-0.79	8.03E-05	0.00142	MMACHC	45960855
chr1	45965608	45966031	424	-0.79	8.03E-05	0.00142	CCDC163P	45963751
chr1	45965608	45966031	424	-0.79	8.03E-05	0.00142	CCDC163P	45963751
chr1	45965608	45966031	424	-0.79	8.03E-05	0.00142	CCDC163P	45963751
chr6	76206007	76206460	454	-1.02	8.04E-05	0.00142	FILIP1	76201545
chr6	76206007	76206460	454	-1.02	8.04E-05	0.00142	FILIP1	76201545
chr6	76206007	76206460	454	-1.02	8.04E-05	0.00142	FILIP1	76201545
chr6	76206007	76206460	454	-1.02	8.04E-05	0.00142	FILIP1	76201545
chr15	78799584	78800059	476	-0.72	8.04E-05	0.00142	HYKK	78794905
chr15	78799584	78800059	476	-0.72	8.04E-05	0.00142	HYKK	78794905
chr5	34046197	34046516	320	-1.1	8.08E-05	0.00142	C1QTNF3	34041371
chr5	34046197	34046516	320	-1.1	8.08E-05	0.00142	C1QTNF3	34041371
chr9	1.12E+08	1.12E+08	578	-0.62	8.13E-05	0.00142	CTNNAL1	1.12E+08
chr9	1.12E+08	1.12E+08	578	-0.62	8.13E-05	0.00142	CTNNAL1	1.12E+08
chr11	64684614	64685044	431	-0.64	8.13E-05	0.00142	ATG2A	64682722
chr3	31573483	31574489	1007	-0.62	8.14E-05	0.00142	STT3B	31568992
chr2	20251245	20252004	760	-0.7	8.14E-05	0.00142	LAPTM4A	20249789
chr11	1.03E+08	1.03E+08	648	-0.89	8.17E-05	0.00143	WTAPP1	1.03E+08
chr11	1.03E+08	1.03E+08	648	-0.89	8.17E-05	0.00143	MMP10	1.03E+08
chr8	90996260	90997015	756	-0.57	8.18E-05	0.00143	NBN	90994899
chr8	90996260	90997015	756	-0.57	8.18E-05	0.00143	NBN	90994952
chr20	18447497	18448229	733	-0.67	8.20E-05	0.00143	MIR3192	18446258

chr20	18447497	18448229	733	-0.67	8.20E-05	0.00143	DZANK1	18445829
chr20	18447497	18448229	733	-0.67	8.20E-05	0.00143	POLR3F	18443032
chr20	18447497	18448229	733	-0.67	8.20E-05	0.00143	POLR3F	18443032
chr20	18447497	18448229	733	-0.67	8.20E-05	0.00143	POLR3F	18443032
chr17	18280676	18281115	440	-0.89	8.24E-05	0.00144	EVPLL	18276078
chr20	18487999	18488689	691	-0.58	8.26E-05	0.00144	SEC23B	18483187
chr20	18487999	18488689	691	-0.58	8.26E-05	0.00144	SEC23B	18483209
chr20	18487999	18488689	691	-0.58	8.26E-05	0.00144	SEC23B	18483187
chr20	18487999	18488689	691	-0.58	8.26E-05	0.00144	SEC23B	18483606
chr20	18487999	18488689	691	-0.58	8.26E-05	0.00144	SEC23B	18483187
chr9	1.25E+08	1.25E+08	333	-0.74	8.26E-05	0.00144	NDUFA8	1.25E+08
chr9	1.25E+08	1.25E+08	333	-0.74	8.26E-05	0.00144	NDUFA8	1.25E+08
chr9	1.25E+08	1.25E+08	333	-0.74	8.26E-05	0.00144	MORN5	1.25E+08
chr9	1.25E+08	1.25E+08	333	-0.74	8.26E-05	0.00144	MORN5	1.25E+08
chr11	5295654	5295893	240	-1.47	8.28E-05	0.00144	HBE1	5289373
chr17	8093421	8093734	314	-0.81	8.29E-05	0.00144	BORCS6	8091564
chr17	18086356	18087260	905	-0.6	8.30E-05	0.00144	ALKBH5	18081866
chr19	19174331	19174821	491	-0.66	8.30E-05	0.00144	SLC25A42	19169802
chr11	58345270	58346688	1419	-0.49	8.31E-05	0.00144	LPXN	58343712
chr11	58345270	58346688	1419	-0.49	8.31E-05	0.00144	LPXN	58341390
chr11	58345270	58346688	1419	-0.49	8.31E-05	0.00144	LPXN	58343712
chr11	58345270	58346688	1419	-0.49	8.31E-05	0.00144	ZFP91	58341586
chr11	58345270	58346688	1419	-0.49	8.31E-05	0.00144	ZFP91-CNT	58341586
chr11	58345270	58346688	1419	-0.49	8.31E-05	0.00144	ZFP91	58341586
chr7	90032607	90033366	760	-0.65	8.33E-05	0.00145	CLDN12	90027647
chr7	90032607	90033366	760	-0.65	8.33E-05	0.00145	CLDN12	90027647
chr7	90032607	90033366	760	-0.65	8.33E-05	0.00145	CLDN12	90027647
chr10	1.06E+08	1.06E+08	457	-0.62	8.41E-05	0.00146	GSTO2	1.06E+08
chr10	1.06E+08	1.06E+08	457	-0.62	8.41E-05	0.00146	GSTO2	1.06E+08
chr10	1.06E+08	1.06E+08	457	-0.62	8.41E-05	0.00146	MIR4482	1.06E+08
chr14	24036647	24037130	484	-0.72	8.47E-05	0.00146	AP1G2	24035045
chr14	24036647	24037130	484	-0.72	8.47E-05	0.00146	AP1G2	24034733
chr14	24036647	24037130	484	-0.72	8.47E-05	0.00146	AP1G2	24035045
chr3	64174768	64175125	358	-1.08	8.51E-05	0.00147	PRICKLE2-A	64168219
chr11	1.11E+08	1.11E+08	369	-1	8.51E-05	0.00147	C11orf88	1.11E+08
chr11	1.11E+08	1.11E+08	369	-1	8.51E-05	0.00147	C11orf88	1.11E+08
chr11	1.11E+08	1.11E+08	369	-1	8.51E-05	0.00147	MIR34B	1.11E+08
chr11	1.11E+08	1.11E+08	369	-1	8.51E-05	0.00147	MIR34C	1.11E+08
chr11	1.11E+08	1.11E+08	369	-1	8.51E-05	0.00147	BTG4	1.11E+08
chr5	98108771	98109678	908	-0.48	8.53E-05	0.00147	RGMB-AS1	98106788
chr7	11012636	11013660	1025	-0.46	8.54E-05	0.00147	PHF14	11008498
chr7	11012636	11013660	1025	-0.46	8.54E-05	0.00147	PHF14	11008498
chr7	11012636	11013660	1025	-0.46	8.54E-05	0.00147	PHF14	11008498
chr3	1.84E+08	1.84E+08	185	-1.11	8.56E-05	0.00147	FAM131A	1.84E+08
chr3	1.84E+08	1.84E+08	185	-1.11	8.56E-05	0.00147	FAM131A	1.84E+08
chr11	33279525	33280310	786	-0.85	8.58E-05	0.00147	HIPK3	33273867
chr11	33279525	33280310	786	-0.85	8.58E-05	0.00147	HIPK3	33273217

chr11	33279525	33280310	786	-0.85	8.58E-05	0.00147	HIPK3	33274876
chr11	33279525	33280310	786	-0.85	8.58E-05	0.00147	HIPK3	33273867
chr16	31470378	31471429	1052	-0.53	8.66E-05	0.00149	ARMC5	31465316
chr16	31470378	31471429	1052	-0.53	8.66E-05	0.00149	ARMC5	31464593
chr16	31470378	31471429	1052	-0.53	8.66E-05	0.00149	ARMC5	31465316
chr16	31470378	31471429	1052	-0.53	8.66E-05	0.00149	ARMC5	31465174
chr10	1.04E+08	1.04E+08	690	-0.56	8.66E-05	0.00149	TRIM8	1.04E+08
chr9	98273020	98273516	497	-0.65	8.67E-05	0.00149	PTCH1	98268831
chr9	98273020	98273516	497	-0.65	8.67E-05	0.00149	PTCH1	98268322
chr9	98273020	98273516	497	-0.65	8.67E-05	0.00149	PTCH1	98267481
chr9	98273020	98273516	497	-0.65	8.67E-05	0.00149	PTCH1	98267699
chr9	98273020	98273516	497	-0.65	8.67E-05	0.00149	PTCH1	98268322
chr6	1.58E+08	1.58E+08	304	-0.96	8.70E-05	0.00149	ZDHHC14	1.58E+08
chr6	1.58E+08	1.58E+08	304	-0.96	8.70E-05	0.00149	ZDHHC14	1.58E+08
chr7	1.12E+08	1.12E+08	1095	-0.59	8.72E-05	0.00149	IFRD1	1.12E+08
chr7	1.12E+08	1.12E+08	1095	-0.59	8.72E-05	0.00149	IFRD1	1.12E+08
chr7	1.12E+08	1.12E+08	1095	-0.59	8.72E-05	0.00149	IFRD1	1.12E+08
chr1	1.67E+08	1.67E+08	880	-0.53	8.77E-05	0.0015	TADA1	1.67E+08
chr11	1.25E+08	1.25E+08	602	-0.73	8.77E-05	0.0015	NRGN	1.25E+08
chr11	1.25E+08	1.25E+08	602	-0.73	8.77E-05	0.0015	NRGN	1.25E+08
chr11	4840828	4841062	235	-1.55	8.79E-05	0.0015	OR51F2	4837615
chr9	80064419	80064976	558	-0.57	8.85E-05	0.00151	GNA14-AS1	80057564
chr20	34330107	34330575	469	-0.72	8.98E-05	0.00152	RBM39	34328258
chr20	34330107	34330575	469	-0.72	8.98E-05	0.00152	RBM39	34328258
chr20	34330107	34330575	469	-0.72	8.98E-05	0.00152	RBM39	34328258
chr20	34330107	34330575	469	-0.72	8.98E-05	0.00152	RBM39	34328258
chr20	34330107	34330575	469	-0.72	8.98E-05	0.00152	RBM39	34328258
chr20	34330107	34330575	469	-0.72	8.98E-05	0.00152	RBM39	34328258
chr20	34330107	34330575	469	-0.72	8.98E-05	0.00152	RBM39	34328258
chr9	71783845	71784117	273	-0.77	8.99E-05	0.00152	TJP2	71783970
chr9	71783845	71784117	273	-0.77	8.99E-05	0.00152	TJP2	71783970
chr9	71783845	71784117	273	-0.77	8.99E-05	0.00152	TJP2	71783970
chr9	23820684	23821933	1250	-0.73	9.00E-05	0.00152	ELAVL2	23819478
chr9	23820684	23821933	1250	-0.73	9.00E-05	0.00152	ELAVL2	23819843
chr5	44807449	44807672	224	-0.98	9.00E-05	0.00152	MRPS30	44804026
chr5	44807449	44807672	224	-0.98	9.00E-05	0.00152	BRCAT54	44806895
chr5	44807449	44807672	224	-0.98	9.00E-05	0.00152	BRCAT54	44806895
chr5	44807449	44807672	224	-0.98	9.00E-05	0.00152	BRCAT54	44806895
chr5	44807449	44807672	224	-0.98	9.00E-05	0.00152	BRCAT54	44806895
chr3	1.12E+08	1.12E+08	861	-0.6	9.01E-05	0.00152	SLC35A5	1.12E+08
chr3	1.12E+08	1.12E+08	861	-0.6	9.01E-05	0.00152	ATG3	1.12E+08
chr3	1.12E+08	1.12E+08	861	-0.6	9.01E-05	0.00152	ATG3	1.12E+08
chr6	83902755	83903226	472	-0.8	9.01E-05	0.00153	PGM3	83901655
chr6	83902755	83903226	472	-0.8	9.01E-05	0.00153	PGM3	83901012
chr6	83902755	83903226	472	-0.8	9.01E-05	0.00153	PGM3	83901012
chr6	83902755	83903226	472	-0.8	9.01E-05	0.00153	PGM3	83901012
chr6	83902755	83903226	472	-0.8	9.01E-05	0.00153	RWDD2A	83898031

chrX	1.31E+08	1.31E+08	562	-0.71	9.05E-05	0.00153	FIRRE	1.31E+08
chr6	1.63E+08	1.63E+08	660	-0.51	9.09E-05	0.00153	PACRG	1.63E+08
chr6	1.63E+08	1.63E+08	660	-0.51	9.09E-05	0.00153	PACRG	1.63E+08
chr6	1.63E+08	1.63E+08	660	-0.51	9.09E-05	0.00153	PARK2	1.63E+08
chr6	1.63E+08	1.63E+08	660	-0.51	9.09E-05	0.00153	PARK2	1.63E+08
chr6	1.63E+08	1.63E+08	660	-0.51	9.09E-05	0.00153	PARK2	1.63E+08
chr6	1.63E+08	1.63E+08	660	-0.51	9.09E-05	0.00153	PACRG	1.63E+08
chr4	1.44E+08	1.44E+08	738	-0.69	9.10E-05	0.00153	USP38	1.44E+08
chr4	1.44E+08	1.44E+08	738	-0.69	9.10E-05	0.00153	USP38	1.44E+08
chr4	1.44E+08	1.44E+08	738	-0.69	9.10E-05	0.00153	USP38	1.44E+08
chr1	89149299	89150829	1531	-0.5	9.10E-05	0.00153	PKN2-AS1	89148887
chr1	89149299	89150829	1531	-0.5	9.10E-05	0.00153	PKN2	89144921
chr1	1.67E+08	1.67E+08	969	-0.58	9.15E-05	0.00154	POU2F1	1.67E+08
chr1	1.67E+08	1.67E+08	969	-0.58	9.15E-05	0.00154	POU2F1	1.67E+08
chr1	1.67E+08	1.67E+08	969	-0.58	9.15E-05	0.00154	POU2F1	1.67E+08
chr9	73028758	73029346	589	-0.63	9.17E-05	0.00154	KLF9	73027573
chr15	80872469	80873000	532	-0.84	9.19E-05	0.00154	MIR5572	80868443
chr12	1.02E+08	1.02E+08	536	-0.62	9.22E-05	0.00155	CCDC53	1.02E+08
chr12	1.02E+08	1.02E+08	536	-0.62	9.22E-05	0.00155	CCDC53	1.02E+08
chr10	27149705	27150254	550	-0.6	9.23E-05	0.00155	ABI1	27148016
chr10	27149705	27150254	550	-0.6	9.23E-05	0.00155	ABI1	27148016
chr10	27149705	27150254	550	-0.6	9.23E-05	0.00155	ABI1	27148016
chr10	27149705	27150254	550	-0.6	9.23E-05	0.00155	ABI1	27148016
chr10	27149705	27150254	550	-0.6	9.23E-05	0.00155	ABI1	27148016
chr10	27149705	27150254	550	-0.6	9.23E-05	0.00155	ABI1	27148016
chr10	27149705	27150254	550	-0.6	9.23E-05	0.00155	ABI1	27148016
chr10	27149705	27150254	550	-0.6	9.23E-05	0.00155	ABI1	27148016
chr10	27149705	27150254	550	-0.6	9.23E-05	0.00155	ABI1	27148016
chr10	27149705	27150254	550	-0.6	9.23E-05	0.00155	ABI1	27148016
chr10	27149705	27150254	550	-0.6	9.23E-05	0.00155	ABI1	27148016
chr10	27149705	27150254	550	-0.6	9.23E-05	0.00155	ABI1	27148016
chr11	66383658	66384160	503	-0.7	9.26E-05	0.00155	RBM14	66379052
chr11	66383658	66384160	503	-0.7	9.26E-05	0.00155	RBM14	66379052
chr11	66383658	66384160	503	-0.7	9.26E-05	0.00155	RBM14-RBI	66379052
chr11	66383658	66384160	503	-0.7	9.26E-05	0.00155	RBM14-RBI	66379052
chr11	66383658	66384160	503	-0.7	9.26E-05	0.00155	RBM14	66379052
chr13	95363368	95364030	663	-0.8	9.32E-05	0.00156	LOC101927	95358740
chr13	95363368	95364030	663	-0.8	9.32E-05	0.00156	SOX21-AS1	95359969
chr13	95363368	95364030	663	-0.8	9.32E-05	0.00156	SOX21	95362797
chr1	1.86E+08	1.86E+08	782	-0.54	9.37E-05	0.00156	C1orf27	1.86E+08
chr1	1.86E+08	1.86E+08	782	-0.54	9.37E-05	0.00156	C1orf27	1.86E+08
chr1	1.86E+08	1.86E+08	782	-0.54	9.37E-05	0.00156	C1orf27	1.86E+08
chr1	1.86E+08	1.86E+08	782	-0.54	9.37E-05	0.00156	TPR	1.86E+08
chr5	5419496	5419649	154	-1.47	9.39E-05	0.00156	ICE1	5417785
chr6	54711078	54711916	839	-0.64	9.47E-05	0.00157	FAM83B	54706568
chr3	1.94E+08	1.94E+08	556	-0.82	9.50E-05	0.00157	FAM43A	1.94E+08
chr5	1.12E+08	1.12E+08	688	-0.67	9.53E-05	0.00158	DCP2	1.12E+08

chr5	1.12E+08	1.12E+08	688	-0.67	9.53E-05	0.00158	DCP2	1.12E+08
chr5	1.12E+08	1.12E+08	688	-0.67	9.53E-05	0.00158	DCP2	1.12E+08
chr2	1.6E+08	1.6E+08	671	-0.67	9.62E-05	0.00159	MIR6888	1.6E+08
chr9	77501907	77502263	357	-0.95	9.62E-05	0.00159	TRPM6	77501010
chr9	77501907	77502263	357	-0.95	9.62E-05	0.00159	TRPM6	77500263
chr9	77501907	77502263	357	-0.95	9.62E-05	0.00159	TRPM6	77500636
chr1	89358165	89358802	638	-0.55	9.65E-05	0.00159	GTF2B	89355301
chr5	1.46E+08	1.46E+08	547	-0.58	9.69E-05	0.0016	RBM27	1.46E+08
chr5	86708572	86709080	509	-0.62	9.69E-05	0.0016	CCNH	86706850
chr5	86708572	86709080	509	-0.62	9.69E-05	0.0016	CCNH	86706721
chr1	2.49E+08	2.49E+08	970	-0.6	9.73E-05	0.0016	SH3BP5L	2.49E+08
chr1	2.49E+08	2.49E+08	970	-0.6	9.73E-05	0.0016	MIR3124	2.49E+08
chr11	27384562	27385046	485	-0.71	9.74E-05	0.0016	CCDC34	27382795
chr11	27384562	27385046	485	-0.71	9.74E-05	0.0016	CCDC34	27382795
chr1	2.36E+08	2.36E+08	527	-0.64	9.76E-05	0.00161	TBCE	2.36E+08
chr1	2.36E+08	2.36E+08	527	-0.64	9.76E-05	0.00161	TBCE	2.36E+08
chr1	2.36E+08	2.36E+08	527	-0.64	9.76E-05	0.00161	TBCE	2.36E+08
chr1	2.36E+08	2.36E+08	527	-0.64	9.76E-05	0.00161	TBCE	2.36E+08
chr6	80713967	80714653	687	-0.61	9.80E-05	0.00161	TTK	80709321
chr6	80713967	80714653	687	-0.61	9.80E-05	0.00161	TTK	80709321
chr16	57769203	57769719	517	-0.59	9.83E-05	0.00161	KATNB1	57764659
chr1	1.97E+08	1.97E+08	926	-0.71	9.95E-05	0.00163	ASPM	1.97E+08
chr1	1.97E+08	1.97E+08	926	-0.71	9.95E-05	0.00163	ASPM	1.97E+08
chr11	36394007	36394226	220	-1.39	9.95E-05	0.00163	PRR5L	36392534
chr11	36394007	36394226	220	-1.39	9.95E-05	0.00163	PRR5L	36392534
chr14	70883497	70883916	420	-0.84	9.98E-05	0.00163	SYNJ2BP-C(	70881807
chr14	70883497	70883916	420	-0.84	9.98E-05	0.00163	SYNJ2BP-C(	70881807
chr14	70883497	70883916	420	-0.84	9.98E-05	0.00163	SYNJ2BP-C(	70881807
chr14	70883497	70883916	420	-0.84	9.98E-05	0.00163	SYNJ2BP	70881807
chr14	20929517	20930158	642	-0.49	1.00E-04	0.00164	TMEM55B	20927771
chr14	20929517	20930158	642	-0.49	1.00E-04	0.00164	TMEM55B	20927771
chr14	23755202	23755814	613	-0.59	0.000101	0.00165	HOMEZ	23753309
chr2	1.33E+08	1.33E+08	1009	-0.49	0.000101	0.00165	GPR39	1.33E+08
chr11	1.22E+08	1.22E+08	1036	-0.73	0.000101	0.00165	MIR100HG	1.22E+08
chr16	27214758	27215585	828	-0.63	0.000102	0.00165	KDM8	27210295
chr16	27214758	27215585	828	-0.63	0.000102	0.00165	KDM8	27209806
chr11	18417384	18417908	525	-0.71	0.000102	0.00166	LDHA	18410935
chr11	18417384	18417908	525	-0.71	0.000102	0.00166	LDHA	18410935
chr11	18417384	18417908	525	-0.71	0.000102	0.00166	LDHA	18412812
chr11	18417384	18417908	525	-0.71	0.000102	0.00166	LDHA	18410935
chr11	18417384	18417908	525	-0.71	0.000102	0.00166	LDHA	18410935
chr11	18417384	18417908	525	-0.71	0.000102	0.00166	LDHA	18410935
chr10	1.17E+08	1.17E+08	630	-0.74	0.000102	0.00166	FAM160B1	1.17E+08
chr10	1.17E+08	1.17E+08	630	-0.74	0.000102	0.00166	FAM160B1	1.17E+08
chr11	14541572	14542252	681	-0.67	0.000105	0.00169	PSMA1	14539991
chr11	14541572	14542252	681	-0.67	0.000105	0.00169	PSMA1	14539991
chr5	1.5E+08	1.5E+08	423	-0.89	0.000105	0.0017	SYNPO	1.5E+08

chr5	1.5E+08	1.5E+08	423	-0.89	0.000105	0.0017	SYNPO	1.5E+08
chr11	47429563	47430197	635	-0.58	0.000106	0.0017	SLC39A13	47425045
chr11	47429563	47430197	635	-0.58	0.000106	0.0017	SLC39A13	47425045
chr11	47429563	47430197	635	-0.58	0.000106	0.0017	SLC39A13	47423826
chr2	85812157	85812440	284	-1.23	0.000106	0.00171	VAMP5	85806530
chr15	43802841	43803404	564	-0.84	0.000106	0.00171	TP53BP1	43800707
chr14	23398567	23399025	459	-0.55	0.000106	0.00171	PRMT5	23396794
chr14	23398567	23399025	459	-0.55	0.000106	0.00171	LOC10192€	23393817
chr14	23398567	23399025	459	-0.55	0.000106	0.00171	PRMT5	23396794
chr14	23398567	23399025	459	-0.55	0.000106	0.00171	PRMT5	23396794
chr14	23398567	23399025	459	-0.55	0.000106	0.00171	PRMT5	23396794
chr14	23398567	23399025	459	-0.55	0.000106	0.00171	PRMT5	23396794
chr14	23398567	23399025	459	-0.55	0.000106	0.00171	PRMT5	23396794
chr16	19078734	19079313	580	-0.66	0.000107	0.00172	COQ7	19074238
chr16	19078734	19079313	580	-0.66	0.000107	0.00172	LOC10272З	19076805
chr16	19078734	19079313	580	-0.66	0.000107	0.00172	LOC10272З	19077013
chr16	19078734	19079313	580	-0.66	0.000107	0.00172	LOC10272З	19077013
chr16	19078734	19079313	580	-0.66	0.000107	0.00172	COQ7	19073916
chr16	19078734	19079313	580	-0.66	0.000107	0.00172	LOC10272З	19077013
chr2	3381251	3381927	677	-0.65	0.000107	0.00172	TSSC1	3379653
chr2	3381251	3381927	677	-0.65	0.000107	0.00172	TRAPPC12	3378445
chr4	1.03E+08	1.03E+08	612	-0.66	0.000109	0.00174	SLC39A8	1.03E+08
chr4	1.03E+08	1.03E+08	612	-0.66	0.000109	0.00174	SLC39A8	1.03E+08
chr4	1.03E+08	1.03E+08	612	-0.66	0.000109	0.00174	SLC39A8	1.03E+08
chr12	6485260	6485973	714	-0.63	0.000109	0.00174	SCNN1A	6482390
chr12	6485260	6485973	714	-0.63	0.000109	0.00174	SCNN1A	6484523
chr12	6485260	6485973	714	-0.63	0.000109	0.00174	LTBR	6479533
chr12	6485260	6485973	714	-0.63	0.000109	0.00174	SCNN1A	6482905
chr14	66974250	66975269	1020	-0.42	0.000109	0.00175	GPHN	66969124
chr14	66974250	66975269	1020	-0.42	0.000109	0.00175	GPHN	66969124
chr11	73471817	73472620	804	-0.42	0.000109	0.00175	RAB6A	73470201
chr11	73471817	73472620	804	-0.42	0.000109	0.00175	RAB6A	73470201
chr11	73471817	73472620	804	-0.42	0.000109	0.00175	RAB6A	73469138
chr11	73471817	73472620	804	-0.42	0.000109	0.00175	RAB6A	73470201
chr7	99070258	99070537	280	-1.22	0.00011	0.00176	ZNF789	99065514
chr7	99070258	99070537	280	-1.22	0.00011	0.00176	ZNF789	99065514
chr10	64892850	64893558	709	-0.73	0.00011	0.00176	NRBF2	64888006
chr10	64892850	64893558	709	-0.73	0.00011	0.00176	NRBF2	64888006
chr5	8457408	8458201	794	-0.42	0.000111	0.00176	LOC72950€	8455677
chr5	8457408	8458201	794	-0.42	0.000111	0.00176	MIR4458	8456037
chr5	8457408	8458201	794	-0.42	0.000111	0.00176	MIR4458H(	8452799
chr17	67323066	67323617	552	-0.64	0.000111	0.00177	ABCA5	67321323
chr6	88182387	88183315	929	-0.7	0.000112	0.00177	SLC35A1	88177642
chr6	88182387	88183315	929	-0.7	0.000112	0.00177	SLC35A1	88177642
chr9	1.02E+08	1.02E+08	1139	-0.5	0.000112	0.00178	TGFBR1	1.02E+08
chr9	1.02E+08	1.02E+08	1139	-0.5	0.000112	0.00178	TGFBR1	1.02E+08
chr9	1.02E+08	1.02E+08	1139	-0.5	0.000112	0.00178	TGFBR1	1.02E+08

chr15	91497976	91498599	624	-0.69	0.000112	0.00178	RCCD1	91493105
chr15	91497976	91498599	624	-0.69	0.000112	0.00178	RCCD1	91493105
chr18	73141975	73142277	303	-1.15	0.000112	0.00178	SMIM21	73137658
chr18	73141975	73142277	303	-1.15	0.000112	0.00178	SMIM21	73137658
chr1	31229444	31229900	457	-0.68	0.000113	0.00179	LAPTM5	31228683
chr11	1.23E+08	1.23E+08	200	-1.3	0.000115	0.00181	CLMP	1.23E+08
chr4	1.01E+08	1.01E+08	561	-0.69	0.000116	0.00182	LOC25688(	1.01E+08
chr4	1.01E+08	1.01E+08	561	-0.69	0.000116	0.00182	DNAJB14	1.01E+08
chr4	1.01E+08	1.01E+08	561	-0.69	0.000116	0.00182	DNAJB14	1.01E+08
chr4	1.01E+08	1.01E+08	561	-0.69	0.000116	0.00182	DNAJB14	1.01E+08
chr3	1.12E+08	1.12E+08	236	-1.26	0.000116	0.00182	CCDC80	1.12E+08
chr3	1.12E+08	1.12E+08	236	-1.26	0.000116	0.00182	CCDC80	1.12E+08
chr7	1.22E+08	1.22E+08	669	-0.64	0.000116	0.00182	AASS	1.22E+08
chr5	1.08E+08	1.08E+08	823	-0.67	0.000116	0.00182	LINC01023	1.08E+08
chr12	72148365	72149491	1127	-0.67	0.000117	0.00183	RAB21	72143642
chr8	1.31E+08	1.31E+08	815	-0.59	0.000117	0.00183	FAM49B	1.31E+08
chr8	1.31E+08	1.31E+08	815	-0.59	0.000117	0.00183	FAM49B	1.31E+08
chr8	1.31E+08	1.31E+08	815	-0.59	0.000117	0.00183	FAM49B	1.31E+08
chr6	92403769	92404177	409	-0.67	0.000117	0.00183	CASC6	92398146
chr12	57029965	57030974	1010	-0.64	0.000118	0.00184	BAZ2A	57028163
chr3	8545158	8545524	367	-1	0.000118	0.00184	LMCD1-AS:	8541344
chr3	8545158	8545524	367	-1	0.000118	0.00184	LMCD1	8538492
chr3	8545158	8545524	367	-1	0.000118	0.00184	LMCD1	8538492
chr3	8545158	8545524	367	-1	0.000118	0.00184	LMCD1	8538492
chr3	8545158	8545524	367	-1	0.000118	0.00184	LMCD1	8538492
chr11	4208236	4209120	885	-0.58	0.000119	0.00185	LOC10050(	4203381
chr6	89790146	89790662	517	-0.66	0.000119	0.00185	PNRC1	89785428
chr14	24701345	24702157	813	-0.58	0.00012	0.00186	NEDD8	24699576
chr14	24701345	24702157	813	-0.58	0.00012	0.00186	GMPR2	24696627
chr14	24701345	24702157	813	-0.58	0.00012	0.00186	GMPR2	24697077
chr14	24701345	24702157	813	-0.58	0.00012	0.00186	GMPR2	24697077
chr14	24701345	24702157	813	-0.58	0.00012	0.00186	NEDD8-MD	24699576
chr14	24701345	24702157	813	-0.58	0.00012	0.00186	GMPR2	24696627
chr14	24701345	24702157	813	-0.58	0.00012	0.00186	GMPR2	24696627
chr14	24701345	24702157	813	-0.58	0.00012	0.00186	GMPR2	24696627
chr14	24701345	24702157	813	-0.58	0.00012	0.00186	GMPR2	24697077
chr14	24701345	24702157	813	-0.58	0.00012	0.00186	GMPR2	24697077
chr1	67390470	67391367	898	-0.52	0.00012	0.00186	WDR78	67388570
chr1	67390470	67391367	898	-0.52	0.00012	0.00186	WDR78	67388570
chr1	67390470	67391367	898	-0.52	0.00012	0.00186	MIER1	67385577
chr1	67390470	67391367	898	-0.52	0.00012	0.00186	MIER1	67385577
chr1	67390470	67391367	898	-0.52	0.00012	0.00186	MIER1	67385577
chr1	67390470	67391367	898	-0.52	0.00012	0.00186	MIER1	67390925
chr1	67390470	67391367	898	-0.52	0.00012	0.00186	MIER1	67385577
chr1	67390470	67391367	898	-0.52	0.00012	0.00186	MIER1	67385577
chr1	67390470	67391367	898	-0.52	0.00012	0.00186	MIER1	67385577
chr1	67390470	67391367	898	-0.52	0.00012	0.00186	MIER1	67390925

chr1	67390470	67391367	898	-0.52	0.00012	0.00186	MIER1	67385577
chr1	67390470	67391367	898	-0.52	0.00012	0.00186	MIER1	67385577
chr1	67390470	67391367	898	-0.52	0.00012	0.00186	MIER1	67385577
chr16	53737595	53738162	568	-0.5	0.00012	0.00186	FTO	53732874
chr16	53737595	53738162	568	-0.5	0.00012	0.00186	RPGRIP1L	53735846
chr16	53737595	53738162	568	-0.5	0.00012	0.00186	RPGRIP1L	53735846
chr16	53737595	53738162	568	-0.5	0.00012	0.00186	RPGRIP1L	53735846
chr15	31456504	31456696	193	-1.52	0.000121	0.00187	TRPM1	31451476
chr15	83478116	83478963	848	-0.73	0.000121	0.00187	WHAMM	83473379
chr15	83478116	83478963	848	-0.73	0.000121	0.00187	FSD2	83472822
chr15	83478116	83478963	848	-0.73	0.000121	0.00187	FSD2	83472822
chr15	83478116	83478963	848	-0.73	0.000121	0.00187	FSD2	83472822
chr12	1.1E+08	1.1E+08	695	-0.57	0.000122	0.00188	MMAB	1.1E+08
chr12	1.1E+08	1.1E+08	695	-0.57	0.000122	0.00188	MVK	1.1E+08
chr12	1.1E+08	1.1E+08	695	-0.57	0.000122	0.00188	MVK	1.1E+08
chr12	1.1E+08	1.1E+08	695	-0.57	0.000122	0.00188	MVK	1.1E+08
chr12	1.1E+08	1.1E+08	695	-0.57	0.000122	0.00188	MMAB	1.1E+08
chr12	1.01E+08	1.01E+08	312	-0.84	0.000123	0.00189	ACTR6	1.01E+08
chr12	1.01E+08	1.01E+08	312	-0.84	0.000123	0.00189	ACTR6	1.01E+08
chr12	1.01E+08	1.01E+08	312	-0.84	0.000123	0.00189	ACTR6	1.01E+08
chr6	73073580	73073887	308	-0.83	0.000123	0.00189	RIMS1	73071431
chr14	94425708	94426644	937	-0.64	0.000123	0.00189	ASB2	94421767
chr2	85134156	85134944	789	-0.67	0.000124	0.0019	TMSB10	85127762
chr3	1.13E+08	1.13E+08	524	-0.76	0.000125	0.00191	SPICE1	1.13E+08
chr16	50058822	50059328	507	-0.59	0.000126	0.00192	CNEP1R1	50054116
chr16	50058822	50059328	507	-0.59	0.000126	0.00192	CNEP1R1	50054116
chr16	50058822	50059328	507	-0.59	0.000126	0.00192	CNEP1R1	50054116
chr16	50058822	50059328	507	-0.59	0.000126	0.00192	CNEP1R1	50054116
chr11	1.02E+08	1.02E+08	635	-0.57	0.000127	0.00193	CEP126	1.02E+08
chr11	1.02E+08	1.02E+08	635	-0.57	0.000127	0.00193	ANGPTL5	1.02E+08
chr10	43133509	43134269	761	-0.57	0.000127	0.00193	ZNF33B	43132285
chr10	43133509	43134269	761	-0.57	0.000127	0.00193	ZNF33B	43132285
chr10	43133509	43134269	761	-0.57	0.000127	0.00193	ZNF33B	43132285
chr10	43133509	43134269	761	-0.57	0.000127	0.00193	ZNF33B	43132285
chr10	43133509	43134269	761	-0.57	0.000127	0.00193	ZNF33B	43132285
chr10	43133509	43134269	761	-0.57	0.000127	0.00193	ZNF33B	43132285
chr10	43133509	43134269	761	-0.57	0.000127	0.00193	ZNF33B	43132285
chr10	43133509	43134269	761	-0.57	0.000127	0.00193	ZNF33B	43132285
chr10	43133509	43134269	761	-0.57	0.000127	0.00193	ZNF33B	43132285
chr10	43133509	43134269	761	-0.57	0.000127	0.00193	ZNF33B	43132285
chr10	43133509	43134269	761	-0.57	0.000127	0.00193	ZNF33B	43132285
chr10	43133509	43134269	761	-0.57	0.000127	0.00193	ZNF33B	43132285
chr9	1.31E+08	1.31E+08	310	-0.89	0.000127	0.00193	SLC25A25	1.31E+08
chr9	1.31E+08	1.31E+08	310	-0.89	0.000127	0.00193	SLC25A25	1.31E+08
chr13	50018072	50018722	651	-0.66	0.000127	0.00193	CAB39L	50016262
chr13	50018072	50018722	651	-0.66	0.000127	0.00193	CAB39L	50016262
chr13	50018072	50018722	651	-0.66	0.000127	0.00193	SETDB2	50013428

chr6	1.59E+08	1.59E+08	801	-0.69	0.000127	0.00193	RSPH3	1.59E+08
chr2	1.19E+08	1.19E+08	426	-0.82	0.000127	0.00193	INSIG2	1.19E+08
chr16	31150067	31150383	317	-0.88	0.000128	0.00194	PRSS8	31145151
chr11	1.13E+08	1.13E+08	357	-0.74	0.000128	0.00194	ANKK1	1.13E+08
chr11	65101130	65101633	504	-0.94	0.000129	0.00196	DPF2	65096224
chr13	1.03E+08	1.03E+08	798	-0.65	0.00013	0.00196	TPP2	1.03E+08
chr11	12306707	12307074	368	-0.77	0.00013	0.00196	MICALCL	12303446
chrX	13670720	13671708	989	-0.55	0.000131	0.00197	TCEANC	13666309
chrX	13670720	13671708	989	-0.55	0.000131	0.00197	TCEANC	13666224
chrX	13670720	13671708	989	-0.55	0.000131	0.00197	TCEANC	13666224
chr12	92539572	92540167	596	-0.66	0.000131	0.00197	LINC01619	92534607
chr12	92539572	92540167	596	-0.66	0.000131	0.00197	BTG1	92537673
chr12	92539572	92540167	596	-0.66	0.000131	0.00197	LINC01619	92534607
chr12	92539572	92540167	596	-0.66	0.000131	0.00197	LINC01619	92533489
chr12	92539572	92540167	596	-0.66	0.000131	0.00197	LINC01619	92534607
chr12	92539572	92540167	596	-0.66	0.000131	0.00197	LINC01619	92534607
chr7	39988977	39989872	896	-0.57	0.000131	0.00197	CDK13	39984958
chr7	39988977	39989872	896	-0.57	0.000131	0.00197	CDK13	39984958
chr6	86303151	86304032	882	-0.5	0.000132	0.00198	SNX14	86301874
chr6	86303151	86304032	882	-0.5	0.000132	0.00198	SNX14	86301874
chr6	86303151	86304032	882	-0.5	0.000132	0.00198	SNX14	86301874
chr6	86303151	86304032	882	-0.5	0.000132	0.00198	SNX14	86301874
chr6	10838510	10838913	404	-0.8	0.000132	0.00198	MAK	10836788
chr6	10838510	10838913	404	-0.8	0.000132	0.00198	MAK	10836716
chr6	10838510	10838913	404	-0.8	0.000132	0.00198	MAK	10836788
chr6	1.5E+08	1.5E+08	558	-0.74	0.000132	0.00198	ULBP2	1.5E+08
chr20	814046	814525	480	-0.79	0.000133	0.00199	FAM110A	811710
chr20	814046	814525	480	-0.79	0.000133	0.00199	FAM110A	809339
chr20	814046	814525	480	-0.79	0.000133	0.00199	FAM110A	809339
chr20	814046	814525	480	-0.79	0.000133	0.00199	FAM110A	809339
chr6	1.2E+08	1.2E+08	452	-0.7	0.000134	0.002	MAN1A1	1.2E+08
chr10	31320466	31321544	1079	-0.52	0.000134	0.002	ZNF438	31318866
chr10	31320466	31321544	1079	-0.52	0.000134	0.002	ZNF438	31318866
chr10	31320466	31321544	1079	-0.52	0.000134	0.002	ZNF438	31318866
chr10	31320466	31321544	1079	-0.52	0.000134	0.002	ZNF438	31318866
chr10	31320466	31321544	1079	-0.52	0.000134	0.002	ZNF438	31318866
chr10	31320466	31321544	1079	-0.52	0.000134	0.002	ZNF438	31318866
chr10	31320466	31321544	1079	-0.52	0.000134	0.002	ZNF438	31318866
chr4	1.66E+08	1.66E+08	1201	-0.64	0.000134	0.002	TMEM192	1.66E+08
chr1	70876468	70876992	525	-0.76	0.000134	0.002	CTH	70871900
chr1	70876468	70876992	525	-0.76	0.000134	0.002	CTH	70871900
chr1	70876468	70876992	525	-0.76	0.000134	0.002	CTH	70871900
chr20	62169092	62169489	398	-0.64	0.000134	0.00201	PTK6	62166723
chr20	62169092	62169489	398	-0.64	0.000134	0.00201	PTK6	62166723
chr10	99052007	99052655	649	-0.64	0.000135	0.00201	ARHGAP19	99050430
chr10	99052007	99052655	649	-0.64	0.000135	0.00201	ARHGAP19	99050430
chr10	99052007	99052655	649	-0.64	0.000135	0.00201	ARHGAP19	99050430

chr10	1.02E+08	1.02E+08	995	-0.55	0.000135	0.00202	OLMALINC	1.02E+08
chr15	75660215	75660954	740	-0.88	0.000136	0.00202	MAN2C1	75658968
chr15	75660215	75660954	740	-0.88	0.000136	0.00202	MAN2C1	75658968
chr15	75660215	75660954	740	-0.88	0.000136	0.00202	MAN2C1	75658968
chr15	75660215	75660954	740	-0.88	0.000136	0.00202	MAN2C1	75658968
chr14	39901273	39902312	1040	-0.52	0.000136	0.00203	FBXO33	39899704
chr1	1.51E+08	1.51E+08	723	-0.64	0.000136	0.00203	ENSA	1.51E+08
chr1	1.51E+08	1.51E+08	723	-0.64	0.000136	0.00203	ENSA	1.51E+08
chr1	1.51E+08	1.51E+08	723	-0.64	0.000136	0.00203	ENSA	1.51E+08
chr1	1.51E+08	1.51E+08	723	-0.64	0.000136	0.00203	ENSA	1.51E+08
chr1	1.51E+08	1.51E+08	723	-0.64	0.000136	0.00203	ENSA	1.51E+08
chr1	1.51E+08	1.51E+08	723	-0.64	0.000136	0.00203	ENSA	1.51E+08
chr1	1.51E+08	1.51E+08	723	-0.64	0.000136	0.00203	ENSA	1.51E+08
chr1	1.51E+08	1.51E+08	723	-0.64	0.000136	0.00203	ENSA	1.51E+08
chr14	24900968	24901181	214	-0.73	0.000136	0.00203	KHNYN	24894140
chr14	24900968	24901181	214	-0.73	0.000136	0.00203	KHNYN	24894140
chr14	24900968	24901181	214	-0.73	0.000136	0.00203	CBLN3	24896731
chr12	66563087	66563891	805	-0.58	0.000137	0.00203	TMBIM4	66561852
chr12	66563087	66563891	805	-0.58	0.000137	0.00203	TMBIM4	66561852
chr12	66563087	66563891	805	-0.58	0.000137	0.00203	TMBIM4	66561852
chr12	66563087	66563891	805	-0.58	0.000137	0.00203	TMBIM4	66561852
chr12	14956068	14956856	789	-0.71	0.000137	0.00203	WBP11	14954401
chr12	14956068	14956856	789	-0.71	0.000137	0.00203	C12orf60	14951505
chrX	1.36E+08	1.36E+08	386	-0.78	0.000137	0.00203	ARHGEF6	1.36E+08
chr1	1.09E+08	1.09E+08	635	-0.57	0.000138	0.00204	FAM102B	1.09E+08
chr10	30980531	30980885	355	-1.17	0.000138	0.00204	SVILP1	30976202
chr6	49518162	49518578	417	-0.64	0.000139	0.00205	C6orf141	49513112
chr5	96036860	96037095	236	-1.08	0.000139	0.00205	CAST	96033492
chr5	96036860	96037095	236	-1.08	0.000139	0.00205	CAST	96033492
chr5	96036860	96037095	236	-1.08	0.000139	0.00205	CAST	96033492
chr9	1.36E+08	1.36E+08	663	-0.59	0.000141	0.00207	GTF3C5	1.36E+08
chr9	1.36E+08	1.36E+08	663	-0.59	0.000141	0.00207	GTF3C5	1.36E+08
chr9	1.36E+08	1.36E+08	663	-0.59	0.000141	0.00207	GTF3C5	1.36E+08
chr12	41300810	41301330	521	-0.71	0.000141	0.00208	CNTN1	41297158
chr19	12250833	12251392	560	-0.75	0.000142	0.00208	ZNF20	12249222
chr19	12250833	12251392	560	-0.75	0.000142	0.00208	ZNF20	12249222
chr7	1.29E+08	1.29E+08	430	-0.96	0.000144	0.0021	SMO	1.29E+08
chr6	88875636	88876183	548	-0.97	0.000144	0.0021	CNR1	88873767
chr6	88875636	88876183	548	-0.97	0.000144	0.0021	CNR1	88873767
chr6	88875636	88876183	548	-0.97	0.000144	0.0021	CNR1	88873767
chr6	88875636	88876183	548	-0.97	0.000144	0.0021	CNR1	88873767
chr9	1.26E+08	1.26E+08	723	-0.51	0.000144	0.00211	ZBTB26	1.26E+08
chr9	1.26E+08	1.26E+08	723	-0.51	0.000144	0.00211	ZBTB26	1.26E+08
chr9	1.26E+08	1.26E+08	723	-0.51	0.000144	0.00211	ZBTB26	1.26E+08
chr12	69753413	69754190	778	-0.79	0.000145	0.00212	YEATS4	69748489
chr12	69753413	69754190	778	-0.79	0.000145	0.00212	YEATS4	69748489
chr11	68816132	68816527	396	-0.66	0.000145	0.00212	TPCN2	68811349

chr12	21654244	21654928	685	-0.55	0.000145	0.00212	RECQL	21652603
chr12	21654244	21654928	685	-0.55	0.000145	0.00212	RECQL	21652603
chr12	21654244	21654928	685	-0.55	0.000145	0.00212	GOLT1B	21649698
chr11	43701920	43702558	639	-0.58	0.000146	0.00212	HSD17B12	43697142
chr17	7482551	7482992	442	-0.73	0.000146	0.00212	CD68	7477804
chr17	7482551	7482992	442	-0.73	0.000146	0.00212	SNORA67	7476272
chr17	7482551	7482992	442	-0.73	0.000146	0.00212	CD68	7477805
chr17	7482551	7482992	442	-0.73	0.000146	0.00212	MPDU1	7481964
chr17	7482551	7482992	442	-0.73	0.000146	0.00212	MPDU1	7481964
chr4	47485980	47486471	492	-0.97	0.000146	0.00213	ATP10D	47482409
chr14	58764516	58765388	873	-0.45	0.000148	0.00214	PSMA3-AS:	58762855
chr14	58764516	58765388	873	-0.45	0.000148	0.00214	PSMA3-AS:	58762855
chr14	58764516	58765388	873	-0.45	0.000148	0.00214	ARID4A	58760221
chr14	58764516	58765388	873	-0.45	0.000148	0.00214	ARID4A	58760221
chr14	58764516	58765388	873	-0.45	0.000148	0.00214	ARID4A	58760221
chr10	76967914	76968294	381	-0.85	0.000149	0.00216	VDAC2	76965299
chr10	76967914	76968294	381	-0.85	0.000149	0.00216	VDAC2	76965299
chr10	76967914	76968294	381	-0.85	0.000149	0.00216	VDAC2	76965299
chr10	76967914	76968294	381	-0.85	0.000149	0.00216	VDAC2	76964911
chr11	36615840	36616444	605	-0.73	0.000149	0.00216	C11orf74	36611042
chr11	36615840	36616444	605	-0.73	0.000149	0.00216	C11orf74	36611042
chr11	36615840	36616444	605	-0.73	0.000149	0.00216	C11orf74	36611042
chr11	36615840	36616444	605	-0.73	0.000149	0.00216	C11orf74	36611042
chr11	36615840	36616444	605	-0.73	0.000149	0.00216	C11orf74	36611321
chr11	36615840	36616444	605	-0.73	0.000149	0.00216	C11orf74	36611042
chr11	36615840	36616444	605	-0.73	0.000149	0.00216	C11orf74	36611042
chr11	36615840	36616444	605	-0.73	0.000149	0.00216	C11orf74	36611042
chr12	56367262	56367990	729	-0.57	0.00015	0.00216	RAB5B	56362794
chr12	56367262	56367990	729	-0.57	0.00015	0.00216	RAB5B	56362794
chr12	56367262	56367990	729	-0.57	0.00015	0.00216	RAB5B	56362696
chr20	25388071	25388640	570	-0.84	0.00015	0.00216	GINS1	25383318
chr20	25388071	25388640	570	-0.84	0.00015	0.00216	GINS1	25383318
chr7	1.02E+08	1.02E+08	454	-0.76	0.00015	0.00217	FAM185A	1.02E+08
chr7	1.02E+08	1.02E+08	454	-0.76	0.00015	0.00217	FAM185A	1.02E+08
chr7	1.02E+08	1.02E+08	454	-0.76	0.00015	0.00217	FAM185A	1.02E+08
chr20	42219148	42219803	656	-0.59	0.000152	0.00219	IFT52	42214252
chr20	42219148	42219803	656	-0.59	0.000152	0.00219	IFT52	42214252
chr20	42219148	42219803	656	-0.59	0.000152	0.00219	IFT52	42214252
chr3	1.72E+08	1.72E+08	682	-0.47	0.000152	0.00219	ECT2	1.72E+08
chr3	1.72E+08	1.72E+08	682	-0.47	0.000152	0.00219	ECT2	1.72E+08
chr3	1.72E+08	1.72E+08	682	-0.47	0.000152	0.00219	ECT2	1.72E+08
chr7	30323800	30324664	865	-0.49	0.000152	0.00219	ZNRF2	30318922
chr7	30323800	30324664	865	-0.49	0.000152	0.00219	MIR550A1	30324409
chr6	97284901	97285659	759	-0.56	0.000152	0.00219	GPR63	97283353
chr6	97284901	97285659	759	-0.56	0.000152	0.00219	GPR63	97283353
chr6	86159312	86160237	926	-0.62	0.000153	0.00219	NT5E	86154301
chr6	86159312	86160237	926	-0.62	0.000153	0.00219	NT5E	86154301

chr12	68726027	68726449	423	-0.67	0.000154	0.0022	MDM1	68724161
chr12	68726027	68726449	423	-0.67	0.000154	0.0022	MDM1	68724161
chr12	68726027	68726449	423	-0.67	0.000154	0.0022	MDM1	68724161
chr12	68726027	68726449	423	-0.67	0.000154	0.0022	MDM1	68724161
chr2	56219433	56220172	740	-0.73	0.000154	0.00221	MIR216A	56214194
chr19	11456899	11457412	514	-0.65	0.000154	0.00221	TMEM205	11454981
chr19	11456899	11457412	514	-0.65	0.000154	0.00221	TMEM205	11454981
chr19	11456899	11457412	514	-0.65	0.000154	0.00221	TMEM205	11454981
chr19	11456899	11457412	514	-0.65	0.000154	0.00221	CCDC159	11452180
chr3	1.5E+08	1.5E+08	282	-1.17	0.000154	0.00221	RNF13	1.5E+08
chr3	1.5E+08	1.5E+08	282	-1.17	0.000154	0.00221	RNF13	1.5E+08
chr4	1.07E+08	1.07E+08	1134	-0.61	0.000155	0.00222	TBCK	1.07E+08
chr4	1.07E+08	1.07E+08	1134	-0.61	0.000155	0.00222	TBCK	1.07E+08
chr4	1.07E+08	1.07E+08	1134	-0.61	0.000155	0.00222	TBCK	1.07E+08
chr4	1.07E+08	1.07E+08	1134	-0.61	0.000155	0.00222	TBCK	1.07E+08
chr4	1.07E+08	1.07E+08	1134	-0.61	0.000155	0.00222	TBCK	1.07E+08
chr4	1.07E+08	1.07E+08	1134	-0.61	0.000155	0.00222	AIMP1	1.07E+08
chr4	1.07E+08	1.07E+08	1134	-0.61	0.000155	0.00222	AIMP1	1.07E+08
chr4	1.07E+08	1.07E+08	1134	-0.61	0.000155	0.00222	AIMP1	1.07E+08
chrX	1.06E+08	1.06E+08	774	-0.72	0.000156	0.00222	TBC1D8B	1.06E+08
chrX	1.06E+08	1.06E+08	774	-0.72	0.000156	0.00222	TBC1D8B	1.06E+08
chr20	32902251	32902670	420	-0.79	0.000156	0.00222	AHCY	32897608
chr11	96122525	96123500	976	-0.67	0.000156	0.00222	CCDC82	96121083
chr11	96122525	96123500	976	-0.67	0.000156	0.00222	JRKL	96118157
chr11	96122525	96123500	976	-0.67	0.000156	0.00222	JRKL	96118157
chr11	96122525	96123500	976	-0.67	0.000156	0.00222	CCDC82	96121083
chr11	96122525	96123500	976	-0.67	0.000156	0.00222	CCDC82	96121083
chr5	42756625	42757646	1022	-0.57	0.000156	0.00222	CCDC152	42751919
chr6	52528974	52529593	620	-0.7	0.000156	0.00222	LOC730101	52524198
chr6	52528974	52529593	620	-0.7	0.000156	0.00222	LOC730101	52524198
chr6	84569189	84569666	478	-0.71	0.000157	0.00223	CYB5R4	84564369
chr8	1.46E+08	1.46E+08	860	-0.54	0.000157	0.00223	ZNF34	1.46E+08
chr8	1.46E+08	1.46E+08	860	-0.54	0.000157	0.00223	ZNF34	1.46E+08
chr8	1.46E+08	1.46E+08	860	-0.54	0.000157	0.00223	ZNF34	1.46E+08
chr20	7999774	8000682	909	-0.61	0.000157	0.00223	TMX4	7998476
chr13	78109652	78109924	273	-0.92	0.000158	0.00224	SCEL	78104808
chr13	78109652	78109924	273	-0.92	0.000158	0.00224	SCEL	78104808
chr13	78109652	78109924	273	-0.92	0.000158	0.00224	SCEL	78104808
chr15	78591581	78592165	585	-0.97	0.000158	0.00224	WDR61	78590068
chr15	78591581	78592165	585	-0.97	0.000158	0.00224	WDR61	78590068
chr15	78591581	78592165	585	-0.97	0.000158	0.00224	WDR61	78590068
chr11	27061626	27062418	793	-0.71	0.000159	0.00224	BBOX1	27057508
chr6	1.27E+08	1.27E+08	733	-0.82	0.000159	0.00225	CENPW	1.27E+08
chr6	1.27E+08	1.27E+08	733	-0.82	0.000159	0.00225	CENPW	1.27E+08
chr6	1.27E+08	1.27E+08	733	-0.82	0.000159	0.00225	CENPW	1.27E+08
chr6	1.27E+08	1.27E+08	733	-0.82	0.000159	0.00225	CENPW	1.27E+08
chr11	65657902	65658832	931	-0.52	0.000159	0.00225	CCDC85B	65652874

chr11	65657902	65658832	931	-0.52	0.000159	0.00225	FIBP	65654010
chr11	65657902	65658832	931	-0.52	0.000159	0.00225	FIBP	65654010
chr11	18128246	18128474	229	-1.79	0.00016	0.00226	SAAL1	18125638
chr17	55685197	55685456	260	-1.95	0.00016	0.00226	LOC101927	55683773
chr16	67969522	67969961	440	-0.68	0.000162	0.00227	CTRL	67963778
chr16	67969522	67969961	440	-0.68	0.000162	0.00227	PSMB10	67968780
chr11	47788789	47789275	487	-0.56	0.000162	0.00227	FNBP4	47787030
chr11	47788789	47789275	487	-0.56	0.000162	0.00227	FNBP4	47787030
chr1	2.21E+08	2.21E+08	659	-0.74	0.000162	0.00228	HLX	2.21E+08
chr1	2.21E+08	2.21E+08	659	-0.74	0.000162	0.00228	HLX-AS1	2.21E+08
chr7	1.25E+08	1.25E+08	591	-0.53	0.000163	0.00228	POT1-AS1	1.25E+08
chr7	1.25E+08	1.25E+08	591	-0.53	0.000163	0.00228	POT1-AS1	1.25E+08
chr7	1.25E+08	1.25E+08	591	-0.53	0.000163	0.00228	POT1	1.25E+08
chr7	1.25E+08	1.25E+08	591	-0.53	0.000163	0.00228	POT1	1.25E+08
chr7	1.25E+08	1.25E+08	591	-0.53	0.000163	0.00228	POT1	1.25E+08
chr7	1.25E+08	1.25E+08	591	-0.53	0.000163	0.00228	POT1	1.25E+08
chr7	1.25E+08	1.25E+08	591	-0.53	0.000163	0.00228	POT1	1.25E+08
chrX	18693054	18693510	457	-0.89	0.000163	0.00228	RS1	18688223
chr3	1.46E+08	1.46E+08	306	-0.81	0.000163	0.00228	PLOD2	1.46E+08
chr3	1.46E+08	1.46E+08	306	-0.81	0.000163	0.00228	PLOD2	1.46E+08
chr9	1.03E+08	1.03E+08	812	-0.6	0.000165	0.0023	STX17	1.03E+08
chr9	1.03E+08	1.03E+08	812	-0.6	0.000165	0.0023	STX17-AS1	1.03E+08
chr9	19048951	19049769	819	-0.9	0.000166	0.00231	SAXO1	19047352
chr9	19048951	19049769	819	-0.9	0.000166	0.00231	RRAGA	19044371
chr11	71639307	71640196	890	-0.56	0.000166	0.00231	RNF121	71634767
chr11	71639307	71640196	890	-0.56	0.000166	0.00231	RNF121	71634767
chr11	71639307	71640196	890	-0.56	0.000166	0.00231	RNF121	71634767
chr11	71639307	71640196	890	-0.56	0.000166	0.00231	RNF121	71634767
chr11	71639307	71640196	890	-0.56	0.000166	0.00231	LOC10013	71637493
chr3	1.13E+08	1.13E+08	635	-0.75	0.000166	0.00231	C3orf17	1.13E+08
chr3	1.13E+08	1.13E+08	635	-0.75	0.000166	0.00231	C3orf17	1.13E+08
chr3	1.13E+08	1.13E+08	635	-0.75	0.000166	0.00231	C3orf17	1.13E+08
chr3	1.13E+08	1.13E+08	635	-0.75	0.000166	0.00231	C3orf17	1.13E+08
chr3	1.13E+08	1.13E+08	635	-0.75	0.000166	0.00231	C3orf17	1.13E+08
chr3	1.13E+08	1.13E+08	635	-0.75	0.000166	0.00231	C3orf17	1.13E+08
chr3	1.13E+08	1.13E+08	635	-0.75	0.000166	0.00231	C3orf17	1.13E+08
chr3	1.13E+08	1.13E+08	635	-0.75	0.000166	0.00231	C3orf17	1.13E+08
chr3	1.13E+08	1.13E+08	635	-0.75	0.000166	0.00231	C3orf17	1.13E+08
chr3	1.13E+08	1.13E+08	635	-0.75	0.000166	0.00231	C3orf17	1.13E+08
chr20	13975813	13976283	471	-0.65	0.000167	0.00232	MACROD2	13971145
chr20	13975813	13976283	471	-0.65	0.000167	0.00232	SEL1L2	13973979
chr20	13975813	13976283	471	-0.65	0.000167	0.00232	SEL1L2	13969265
chr20	13975813	13976283	471	-0.65	0.000167	0.00232	SEL1L2	13969265
chr16	34745124	34745408	285	-1.3	0.000167	0.00232	TP53TG3HI	34739541
chr16	34745124	34745408	285	-1.3	0.000167	0.00232	TP53TG3HI	34739541
chr8	1.42E+08	1.42E+08	391	-0.8	0.000168	0.00232	LINC01300	1.42E+08
chr5	1.63E+08	1.63E+08	886	-0.55	0.000169	0.00234	CCNG1	1.63E+08

chr5	1.63E+08	1.63E+08	886	-0.55	0.000169	0.00234	CCNG1	1.63E+08
chr4	1.15E+08	1.15E+08	1445	-0.59	0.000169	0.00234	CAMK2D	1.15E+08
chr4	1.15E+08	1.15E+08	1445	-0.59	0.000169	0.00234	CAMK2D	1.15E+08
chr4	1.15E+08	1.15E+08	1445	-0.59	0.000169	0.00234	CAMK2D	1.15E+08
chr4	1.15E+08	1.15E+08	1445	-0.59	0.000169	0.00234	CAMK2D	1.15E+08
chr4	1.15E+08	1.15E+08	1445	-0.59	0.000169	0.00234	CAMK2D	1.15E+08
chr4	1.15E+08	1.15E+08	1445	-0.59	0.000169	0.00234	CAMK2D	1.15E+08
chr5	5422183	5422909	727	-0.51	0.00017	0.00234	ICE1	5417785
chr10	1.16E+08	1.16E+08	707	-0.75	0.00017	0.00234	AFAP1L2	1.16E+08
chr10	1.16E+08	1.16E+08	707	-0.75	0.00017	0.00234	AFAP1L2	1.16E+08
chr10	1.16E+08	1.16E+08	707	-0.75	0.00017	0.00234	AFAP1L2	1.16E+08
chr9	1.23E+08	1.23E+08	582	-0.53	0.00017	0.00234	MEGF9	1.23E+08
chr16	56485140	56485661	522	-0.65	0.000172	0.00236	OGFOD1	56480423
chr16	56485140	56485661	522	-0.65	0.000172	0.00236	NUDT21	56483261
chr1	2.12E+08	2.12E+08	598	-0.81	0.000172	0.00236	NEK2	2.12E+08
chr1	2.12E+08	2.12E+08	598	-0.81	0.000172	0.00236	NEK2	2.12E+08
chr1	2.12E+08	2.12E+08	598	-0.81	0.000172	0.00236	NEK2	2.12E+08
chr16	54880674	54881439	766	-0.59	0.000172	0.00237	LOC101927	54880145
chr5	79234301	79234729	429	-0.73	0.000173	0.00237	LINC01455	79227401
chr10	1.22E+08	1.22E+08	516	-0.94	0.000174	0.00238	PLPP4	1.22E+08
chr10	1.22E+08	1.22E+08	516	-0.94	0.000174	0.00238	PLPP4	1.22E+08
chr10	1.22E+08	1.22E+08	516	-0.94	0.000174	0.00238	PLPP4	1.22E+08
chr10	1.22E+08	1.22E+08	516	-0.94	0.000174	0.00238	PLPP4	1.22E+08
chr10	1.22E+08	1.22E+08	516	-0.94	0.000174	0.00238	PLPP4	1.22E+08
chr10	1.22E+08	1.22E+08	516	-0.94	0.000174	0.00238	PLPP4	1.22E+08
chr16	46654811	46655467	657	-0.81	0.000174	0.00238	SHCBP1	46653311
chr4	1.09E+08	1.09E+08	609	-0.74	0.000176	0.0024	HADH	1.09E+08
chr4	1.09E+08	1.09E+08	609	-0.74	0.000176	0.0024	HADH	1.09E+08
chr6	1.07E+08	1.07E+08	601	-0.68	0.000176	0.0024	ATG5	1.07E+08
chr6	1.07E+08	1.07E+08	601	-0.68	0.000176	0.0024	ATG5	1.07E+08
chr6	1.07E+08	1.07E+08	601	-0.68	0.000176	0.0024	ATG5	1.07E+08
chr6	1.07E+08	1.07E+08	601	-0.68	0.000176	0.0024	ATG5	1.07E+08
chr6	1.07E+08	1.07E+08	601	-0.68	0.000176	0.0024	ATG5	1.07E+08
chr7	20336161	20336379	219	-1.55	0.000177	0.00241	LOC101927	20331330
chr7	48210816	48211638	823	-0.57	0.000177	0.00241	ABCA13	48206056
chr8	38033633	38034569	937	-0.56	0.000179	0.00243	LSM1	38032248
chr8	38033633	38034569	937	-0.56	0.000179	0.00243	BAG4	38029105
chr8	38033633	38034569	937	-0.56	0.000179	0.00243	LSM1	38032248
chr8	38033633	38034569	937	-0.56	0.000179	0.00243	BAG4	38029105
chr8	38033633	38034569	937	-0.56	0.000179	0.00243	LSM1	38032248
chr15	55700234	55700840	607	-0.53	0.000179	0.00243	C15orf65	55695722
chr15	55700234	55700840	607	-0.53	0.000179	0.00243	CCPG1	55698708
chr15	55700234	55700840	607	-0.53	0.000179	0.00243	CCPG1	55698574
chr15	55700234	55700840	607	-0.53	0.000179	0.00243	CCPG1	55698708
chr15	55700234	55700840	607	-0.53	0.000179	0.00243	CCPG1	55698708
chr1	2.15E+08	2.15E+08	505	-0.59	0.000179	0.00243	CENPF	2.15E+08
chr11	7949186	7949554	369	-1.16	0.00018	0.00244	OR10A6	7948209

chr11	86666315	86667233	919	-0.79	0.00018	0.00244	FZD4	86664440
chr11	86666315	86667233	919	-0.79	0.00018	0.00244	LOC10050(	86661662
chr13	1.11E+08	1.11E+08	681	-0.64	0.00018	0.00244	ING1	1.11E+08
chr13	1.11E+08	1.11E+08	681	-0.64	0.00018	0.00244	ING1	1.11E+08
chr13	1.11E+08	1.11E+08	681	-0.64	0.00018	0.00244	ING1	1.11E+08
chr13	1.11E+08	1.11E+08	681	-0.64	0.00018	0.00244	ING1	1.11E+08
chr13	1.11E+08	1.11E+08	681	-0.64	0.00018	0.00244	ING1	1.11E+08
chr1	47779656	47780218	563	-0.5	0.000181	0.00245	STIL	47777819
chr1	47779656	47780218	563	-0.5	0.000181	0.00245	STIL	47777819
chr1	47779656	47780218	563	-0.5	0.000181	0.00245	STIL	47777819
chr1	47779656	47780218	563	-0.5	0.000181	0.00245	STIL	47777819
chr1	47779656	47780218	563	-0.5	0.000181	0.00245	STIL	47777819
chr1	47779656	47780218	563	-0.5	0.000181	0.00245	STIL	47777819
chr6	47012258	47012455	198	-1.08	0.000183	0.00246	ADGRF1	47008099
chr6	47012258	47012455	198	-1.08	0.000183	0.00246	ADGRF1	47008099
chr5	1.77E+08	1.77E+08	824	-0.59	0.000183	0.00247	GRK6	1.77E+08
chr5	1.77E+08	1.77E+08	824	-0.59	0.000183	0.00247	GRK6	1.77E+08
chr5	1.77E+08	1.77E+08	824	-0.59	0.000183	0.00247	GRK6	1.77E+08
chr7	42971744	42972153	410	-0.7	0.000184	0.00247	PSMA2	42969805
chr7	42971744	42972153	410	-0.7	0.000184	0.00247	MRPL32	42966938
chr15	90895342	90895760	419	-0.77	0.000184	0.00247	ZNF774	90890476
chr15	90895342	90895760	419	-0.77	0.000184	0.00247	GABARAPL	90890679
chr3	1.57E+08	1.57E+08	683	-0.54	0.000185	0.00248	PTX3	1.57E+08
chr11	1.29E+08	1.29E+08	769	-0.79	0.000186	0.00248	TP53AIP1	1.29E+08
chr16	5147685	5148729	1045	-0.46	0.000186	0.00249	EEF2KMT	5145821
chr16	5147685	5148729	1045	-0.46	0.000186	0.00249	EEF2KMT	5145821
chr16	5147685	5148729	1045	-0.46	0.000186	0.00249	EEF2KMT	5145821
chr10	76995590	76995903	314	-0.99	0.000186	0.00249	COMTD1	76993770
chr7	42276780	42277761	982	-0.61	0.000186	0.00249	GLI3	42274618
chr6	1.17E+08	1.17E+08	867	-0.71	0.000187	0.0025	DSE	1.17E+08
chr6	1.17E+08	1.17E+08	867	-0.71	0.000187	0.0025	TSPYL1	1.17E+08
chr7	1.41E+08	1.41E+08	554	-0.69	0.000187	0.0025	MRPS33	1.41E+08
chr7	1.41E+08	1.41E+08	554	-0.69	0.000187	0.0025	MRPS33	1.41E+08
chr17	7210266	7210969	704	-0.58	0.000187	0.0025	EIF5A	7206693
chr17	7210266	7210969	704	-0.58	0.000187	0.0025	EIF5A	7206287
chr17	7210266	7210969	704	-0.58	0.000187	0.0025	EIF5A	7205317
chr17	7210266	7210969	704	-0.58	0.000187	0.0025	EIF5A	7205855
chr15	34659357	34659956	600	-0.78	0.000187	0.0025	LPCAT4	34657395
chr13	53191164	53191807	644	-0.72	0.000188	0.0025	HNRNPA1L	53186604
chr13	53191164	53191807	644	-0.72	0.000188	0.0025	HNRNPA1L	53186604
chr2	85637481	85637854	374	-1.31	0.000188	0.0025	CAPG	85635820
chr2	85637481	85637854	374	-1.31	0.000188	0.0025	CAPG	85635820
chr2	2.01E+08	2.01E+08	247	-0.92	0.000188	0.00251	SPATS2L	2.01E+08
chr2	2.01E+08	2.01E+08	247	-0.92	0.000188	0.00251	SPATS2L	2.01E+08
chr2	2.01E+08	2.01E+08	247	-0.92	0.000188	0.00251	SPATS2L	2.01E+08
chr2	2.01E+08	2.01E+08	247	-0.92	0.000188	0.00251	SPATS2L	2.01E+08
chr2	2.01E+08	2.01E+08	247	-0.92	0.000188	0.00251	SPATS2L	2.01E+08

chr2	2.01E+08	2.01E+08	247	-0.92	0.000188	0.00251	SPATS2L	2.01E+08
chr9	12814219	12814848	630	-0.97	0.000188	0.00251	LURAP1L-A	12812388
chr3	1.57E+08	1.57E+08	559	-0.6	0.000189	0.00252	VEPH1	1.57E+08
chr3	1.57E+08	1.57E+08	559	-0.6	0.000189	0.00252	VEPH1	1.57E+08
chr10	60028551	60029398	848	-0.54	0.00019	0.00252	CISD1	60023861
chr10	60028551	60029398	848	-0.54	0.00019	0.00252	IPMK	60025694
chr14	74550961	74551842	882	-0.48	0.00019	0.00252	LIN52	74546655
chr14	74550961	74551842	882	-0.48	0.00019	0.00252	ALDH6A1	74549196
chr14	74550961	74551842	882	-0.48	0.00019	0.00252	ALDH6A1	74549196
chr14	74550961	74551842	882	-0.48	0.00019	0.00252	ALDH6A1	74549196
chr6	1.11E+08	1.11E+08	890	-0.61	0.00019	0.00252	WASF1	1.1E+08
chr6	1.11E+08	1.11E+08	890	-0.61	0.00019	0.00252	WASF1	1.1E+08
chr6	1.11E+08	1.11E+08	890	-0.61	0.00019	0.00252	WASF1	1.1E+08
chr6	1.11E+08	1.11E+08	890	-0.61	0.00019	0.00252	WASF1	1.1E+08
chr6	1.11E+08	1.11E+08	890	-0.61	0.00019	0.00252	CDC40	1.1E+08
chr17	53811852	53812218	367	-1.22	0.000192	0.00254	TMEM100	53807482
chr1	1.53E+08	1.53E+08	460	-0.86	0.000193	0.00254	SPRR1B	1.53E+08
chr15	89166429	89166671	243	-0.99	0.000193	0.00255	AEN	89159526
chr10	82173288	82173689	402	-0.61	0.000194	0.00255	FAM213A	82168574
chr10	82173288	82173689	402	-0.61	0.000194	0.00255	FAM213A	82168574
chr3	1.7E+08	1.7E+08	970	-0.52	0.000194	0.00255	PRKCI	1.7E+08
chr3	1.57E+08	1.57E+08	189	-0.86	0.000194	0.00255	LINC00880	1.57E+08
chr17	8533993	8534852	860	-0.51	0.000196	0.00257	MYH10	8532079
chr17	8533993	8534852	860	-0.51	0.000196	0.00257	MYH10	8532079
chr17	8533993	8534852	860	-0.51	0.000196	0.00257	MYH10	8532079
chr4	1.79E+08	1.79E+08	356	-0.76	0.000197	0.00258	LINC01098	1.79E+08
chr12	1.27E+08	1.27E+08	619	-0.55	0.000197	0.00258	LINC00944	1.27E+08
chr10	1.12E+08	1.12E+08	248	-0.74	0.000197	0.00258	MXI1	1.12E+08
chr1	1.01E+08	1.01E+08	976	-0.56	0.000198	0.00258	RTCA	1.01E+08
chr1	1.01E+08	1.01E+08	976	-0.56	0.000198	0.00258	RTCA	1.01E+08
chr1	1.01E+08	1.01E+08	976	-0.56	0.000198	0.00258	RTCA-AS1	1.01E+08
chr1	1.01E+08	1.01E+08	976	-0.56	0.000198	0.00258	RTCA-AS1	1.01E+08
chr1	1.93E+08	1.93E+08	679	-0.61	0.000198	0.00259	CDC73	1.93E+08
chr12	30907510	30908292	783	-0.53	0.000198	0.00259	CAPRIN2	30905448
chr12	30907510	30908292	783	-0.53	0.000198	0.00259	CAPRIN2	30905448
chr12	30907510	30908292	783	-0.53	0.000198	0.00259	CAPRIN2	30905448
chr12	30907510	30908292	783	-0.53	0.000198	0.00259	CAPRIN2	30905448
chr12	30907510	30908292	783	-0.53	0.000198	0.00259	CAPRIN2	30905448
chr8	86132430	86133128	699	-0.5	0.000198	0.00259	C8orf59	86130651
chr8	86132430	86133128	699	-0.5	0.000198	0.00259	C8orf59	86130651
chr8	86132430	86133128	699	-0.5	0.000198	0.00259	C8orf59	86130651
chr8	86132430	86133128	699	-0.5	0.000198	0.00259	C8orf59	86130651
chr8	86132430	86133128	699	-0.5	0.000198	0.00259	C8orf59	86130651
chr8	86132430	86133128	699	-0.5	0.000198	0.00259	C8orf59	86130651
chr16	71598817	71599454	638	-0.77	0.000199	0.00259	TAT-AS1	71593918
chr16	71598817	71599454	638	-0.77	0.000199	0.00259	TAT-AS1	71594691
chr10	15138887	15139692	806	-0.61	2.00E-04	0.0026	RPP38	15134178

chr10	15138887	15139692	806	-0.61	2.00E-04	0.0026	RPP38	15134178
chr10	15138887	15139692	806	-0.61	2.00E-04	0.0026	C10orf111	15137318
chr10	15138887	15139692	806	-0.61	2.00E-04	0.0026	RPP38	15134178
chr10	15138887	15139692	806	-0.61	2.00E-04	0.0026	RPP38	15134178
chr19	52254976	52255162	187	-1.8	2.00E-04	0.00261	FPR1	52253150
chr19	52254976	52255162	187	-1.8	2.00E-04	0.00261	FPR1	52253150
chr6	64354796	64355236	441	-0.83	0.000202	0.00262	PHF3	64351430
chr4	1.06E+08	1.06E+08	1138	-0.53	0.000202	0.00263	PPA2	1.06E+08
chr4	1.06E+08	1.06E+08	1138	-0.53	0.000202	0.00263	PPA2	1.06E+08
chr4	1.06E+08	1.06E+08	1138	-0.53	0.000202	0.00263	PPA2	1.06E+08
chr4	1.06E+08	1.06E+08	1138	-0.53	0.000202	0.00263	PPA2	1.06E+08
chr10	61666178	61666858	681	-0.52	0.000203	0.00263	CCDC6	61664414
chr14	64108217	64109230	1014	-0.44	0.000203	0.00263	WDR89	64106641
chr14	64108217	64109230	1014	-0.44	0.000203	0.00263	WDR89	64106641
chr14	64108217	64109230	1014	-0.44	0.000203	0.00263	WDR89	64106641
chr5	1.26E+08	1.26E+08	313	-0.73	0.000203	0.00263	LOC10272	1.26E+08
chr5	1.26E+08	1.26E+08	313	-0.73	0.000203	0.00263	LMNB1	1.26E+08
chr5	1.26E+08	1.26E+08	313	-0.73	0.000203	0.00263	LMNB1	1.26E+08
chr5	1.26E+08	1.26E+08	313	-0.73	0.000203	0.00263	LMNB1	1.26E+08
chr7	76751382	76751970	589	-0.72	0.000203	0.00263	CCDC146	76746933
chr17	1303363	1303780	418	-0.86	0.000204	0.00264	YWHAE	1301556
chr17	1303363	1303780	418	-0.86	0.000204	0.00264	YWHAE	1301556
chr6	46458917	46459854	938	-0.74	0.000205	0.00264	RCAN2	46457060
chr6	46458917	46459854	938	-0.74	0.000205	0.00264	RCAN2	46457804
chr6	46458917	46459854	938	-0.74	0.000205	0.00264	LOC10192	46454788
chr1	2.12E+08	2.12E+08	987	-0.53	0.000206	0.00266	INTS7	2.12E+08
chr1	2.12E+08	2.12E+08	987	-0.53	0.000206	0.00266	INTS7	2.12E+08
chr1	2.12E+08	2.12E+08	987	-0.53	0.000206	0.00266	INTS7	2.12E+08
chr1	2.12E+08	2.12E+08	987	-0.53	0.000206	0.00266	INTS7	2.12E+08
chr1	2.12E+08	2.12E+08	987	-0.53	0.000206	0.00266	INTS7	2.12E+08
chr1	2.12E+08	2.12E+08	987	-0.53	0.000206	0.00266	DTL	2.12E+08
chr1	2.12E+08	2.12E+08	987	-0.53	0.000206	0.00266	DTL	2.12E+08
chr1	2.12E+08	2.12E+08	987	-0.53	0.000206	0.00266	DTL	2.12E+08
chr16	31711723	31712276	554	-0.68	0.000206	0.00266	CLUHP3	31706933
chr15	89923036	89923453	418	-0.84	0.000207	0.00267	MIR9-3HG	89916272
chr11	1.34E+08	1.34E+08	1187	-0.49	0.000208	0.00267	NCAPD3	1.34E+08
chr11	1.34E+08	1.34E+08	1187	-0.49	0.000208	0.00267	VPS26B	1.34E+08
chr3	93698717	93699413	697	-0.54	0.000208	0.00267	ARL13B	93693982
chr3	93698717	93699413	697	-0.54	0.000208	0.00267	ARL13B	93693982
chr3	93698717	93699413	697	-0.54	0.000208	0.00267	ARL13B	93693982
chr3	93698717	93699413	697	-0.54	0.000208	0.00267	ARL13B	93693982
chr3	93698717	93699413	697	-0.54	0.000208	0.00267	ARL13B	93693982
chr6	20401523	20402106	584	-0.49	0.000208	0.00267	E2F3	20398909
chr6	20401523	20402106	584	-0.49	0.000208	0.00267	E2F3	20397136
chr1	1.63E+08	1.63E+08	620	-0.84	0.000208	0.00267	CCDC190	1.63E+08
chr14	36295289	36296199	911	-0.48	0.000208	0.00267	BRMS1L	36290596
chr7	1.33E+08	1.33E+08	412	-0.87	0.000208	0.00267	EXOC4	1.33E+08

chr7	1.33E+08	1.33E+08	412	-0.87	0.000208	0.00267	EXOC4	1.33E+08
chr15	91412888	91413288	401	-0.79	0.00021	0.00269	FURIN	91409768
chr15	91412888	91413288	401	-0.79	0.00021	0.00269	FURIN	91406821
chr15	91412888	91413288	401	-0.79	0.00021	0.00269	FURIN	91411107
chr15	72520809	72521267	459	-0.91	0.000211	0.00269	PKM	72520063
chr8	28747454	28747930	477	-0.71	0.000212	0.0027	HMBOX1	28742910
chr8	28747454	28747930	477	-0.71	0.000212	0.0027	INTS9	28745432
chr8	28747454	28747930	477	-0.71	0.000212	0.0027	INTS9	28745698
chr8	28747454	28747930	477	-0.71	0.000212	0.0027	HMBOX1	28743224
chr8	28747454	28747930	477	-0.71	0.000212	0.0027	INTS9	28745698
chr8	28747454	28747930	477	-0.71	0.000212	0.0027	INTS9	28745698
chr5	35857952	35858248	297	-0.72	0.000212	0.00271	IL7R	35851976
chr5	35857952	35858248	297	-0.72	0.000212	0.00271	IL7R	35851976
chr12	75728201	75728831	631	-0.75	0.000212	0.00271	GLIPR1L1	75723418
chr12	75728201	75728831	631	-0.75	0.000212	0.00271	GLIPR1L1	75723418
chr12	75728201	75728831	631	-0.75	0.000212	0.00271	CAPS2	75721836
chr12	75728201	75728831	631	-0.75	0.000212	0.00271	CAPS2	75721836
chr16	56688211	56688484	274	-1.09	0.000213	0.00271	MT1F	56686854
chr16	56688211	56688484	274	-1.09	0.000213	0.00271	MT1F	56686854
chr4	1.49E+08	1.49E+08	578	-0.76	0.000214	0.00271	TMEM184(	1.49E+08
chr1	1.74E+08	1.74E+08	1296	-0.48	0.000214	0.00272	RABGAP1L	1.74E+08
chr1	1.74E+08	1.74E+08	1296	-0.48	0.000214	0.00272	LOC10272	1.74E+08
chr12	1.19E+08	1.19E+08	531	-0.62	0.000214	0.00272	TAOK3	1.19E+08
chr12	1.19E+08	1.19E+08	531	-0.62	0.000214	0.00272	SUDS3	1.19E+08
chr16	14013580	14014201	622	-0.61	0.000215	0.00272	ERCC4	14009013
chr14	21467092	21467488	397	-0.79	0.000215	0.00272	SLC39A2	21462413
chr14	21467092	21467488	397	-0.79	0.000215	0.00272	SLC39A2	21462413
chr14	21467092	21467488	397	-0.79	0.000215	0.00272	LOC10192	21465322
chr19	54370649	54371034	386	-0.81	0.000216	0.00273	MYADM	54366118
chr19	54370649	54371034	386	-0.81	0.000216	0.00273	MYADM	54364411
chr19	54370649	54371034	386	-0.81	0.000216	0.00273	MYADM	54366118
chr19	54370649	54371034	386	-0.81	0.000216	0.00273	MYADM	54366118
chr19	54370649	54371034	386	-0.81	0.000216	0.00273	MYADM	54367644
chr19	54370649	54371034	386	-0.81	0.000216	0.00273	MYADM	54366118
chr19	54370649	54371034	386	-0.81	0.000216	0.00273	MYADM	54363837
chr19	54370649	54371034	386	-0.81	0.000216	0.00273	MYADM	54366118
chr19	54370649	54371034	386	-0.81	0.000216	0.00273	MYADM	54367644
chr19	54370649	54371034	386	-0.81	0.000216	0.00273	MYADM	54366798
chr19	54370649	54371034	386	-0.81	0.000216	0.00273	MYADM	54364411
chr19	54370649	54371034	386	-0.81	0.000216	0.00273	MYADM	54367644
chr10	97320539	97321418	880	-0.55	0.000216	0.00274	SORBS1	97319177
chr10	97320539	97321418	880	-0.55	0.000216	0.00274	SORBS1	97319177
chr10	97320539	97321418	880	-0.55	0.000216	0.00274	SORBS1	97319177
chr10	97320539	97321418	880	-0.55	0.000216	0.00274	SORBS1	97319177
chr1	2.37E+08	2.37E+08	738	-0.49	0.000216	0.00274	MTR	2.37E+08
chr1	2.37E+08	2.37E+08	738	-0.49	0.000216	0.00274	MTR	2.37E+08
chr1	2.37E+08	2.37E+08	738	-0.49	0.000216	0.00274	MTR	2.37E+08

chr4	1.44E+08	1.44E+08	929	-0.52	0.000216	0.00274	SMARCA5	1.44E+08
chr4	1.44E+08	1.44E+08	929	-0.52	0.000216	0.00274	SMARCA5-,	1.44E+08
chr14	23938456	23938963	508	-0.54	0.000217	0.00275	NGDN	23933897
chr14	23938456	23938963	508	-0.54	0.000217	0.00275	NGDN	23933897
chr4	1.77E+08	1.77E+08	250	-1.1	0.000217	0.00275	SPATA4	1.77E+08
chr12	29533516	29534435	920	-0.61	0.000219	0.00277	ERGIC2	29532143
chr6	79786630	79786879	250	-0.88	0.000219	0.00277	PHIP	79786011
chr19	12551495	12552124	630	-0.75	0.00022	0.00277	ZNF443	12549926
chr21	43916303	43916723	421	-0.71	0.00022	0.00277	RSPH1	43914464
chr21	43916303	43916723	421	-0.71	0.00022	0.00277	RSPH1	43914464
chr21	43916303	43916723	421	-0.71	0.00022	0.00277	SLC37A1	43914741
chr14	61190915	61191260	346	-0.62	0.00022	0.00278	SIX4	61188852
chr11	82782757	82783313	557	-0.6	0.000221	0.00278	RAB30	82780965
chr11	82782757	82783313	557	-0.6	0.000221	0.00278	RAB30	82780504
chr11	82782757	82783313	557	-0.6	0.000221	0.00278	RAB30	82780965
chr11	82782757	82783313	557	-0.6	0.000221	0.00278	RAB30-AS1	82778107
chr1	55230089	55230543	455	-0.87	0.000222	0.00279	PARS2	55228226
chr11	13689730	13690363	634	-0.5	0.000222	0.00279	FAR1	13685205
chr1	91869805	91870370	566	-0.64	0.000223	0.00279	HFM1	91868426
chr20	3646546	3646755	210	-1.4	0.000223	0.0028	GFRA4	3642046
chr20	3646546	3646755	210	-1.4	0.000223	0.0028	GFRA4	3642046
chr6	99282147	99282600	454	-0.68	0.000224	0.00281	POU3F2	99277579
chr12	57039719	57040155	437	-0.68	0.000224	0.00281	SNORD59A	57036885
chr12	57039719	57040155	437	-0.68	0.000224	0.00281	SNORD59B	57035538
chr12	57039719	57040155	437	-0.68	0.000224	0.00281	ATP5B	57037852
chr14	79745425	79745955	531	-0.52	0.000224	0.00281	NRXN3	79740681
chr14	79745425	79745955	531	-0.52	0.000224	0.00281	NRXN3	79740681
chr14	79745425	79745955	531	-0.52	0.000224	0.00281	NRXN3	79740681
chr14	79745425	79745955	531	-0.52	0.000224	0.00281	NRXN3	79740681
chr2	66660128	66660788	661	-0.8	0.000225	0.00281	MEIS1	66657531
chr2	66660128	66660788	661	-0.8	0.000225	0.00281	MEIS1-AS3	66658602
chr11	62494630	62495588	959	-0.58	0.000225	0.00281	TTC9C	62490951
chr11	62494630	62495588	959	-0.58	0.000225	0.00281	HNRNPUL2	62492857
chr11	62494630	62495588	959	-0.58	0.000225	0.00281	HNRNPUL2	62492856
chr11	62494630	62495588	959	-0.58	0.000225	0.00281	TTC9C	62490544
chr11	62494630	62495588	959	-0.58	0.000225	0.00281	TTC9C	62490951
chr11	62494630	62495588	959	-0.58	0.000225	0.00281	TTC9C	62490951
chr11	62494630	62495588	959	-0.58	0.000225	0.00281	TTC9C	62490951
chr11	62494630	62495588	959	-0.58	0.000225	0.00281	TTC9C	62490951
chr9	1.3E+08	1.3E+08	696	-0.68	0.000227	0.00283	RALGPS1	1.3E+08
chr9	1.4E+08	1.4E+08	513	-0.52	0.000227	0.00283	MAN1B1	1.4E+08
chr9	1.4E+08	1.4E+08	513	-0.52	0.000227	0.00283	MAN1B1	1.4E+08
chr9	1.4E+08	1.4E+08	513	-0.52	0.000227	0.00283	MAN1B1	1.4E+08
chr9	1.4E+08	1.4E+08	513	-0.52	0.000227	0.00283	MAN1B1-A	1.4E+08
chr11	57424790	57425286	497	-0.52	0.000227	0.00283	CLP1	57420215
chr11	57424790	57425286	497	-0.52	0.000227	0.00283	CLP1	57420215
chr12	1.2E+08	1.2E+08	275	-0.71	0.000228	0.00284	HSPB8	1.2E+08

chr8	38126090	38127122	1033	-0.58	0.000229	0.00285	PLPP5	38124738
chr8	38126090	38127122	1033	-0.58	0.000229	0.00285	PLPP5	38124738
chr8	38126090	38127122	1033	-0.58	0.000229	0.00285	PLPP5	38124738
chr5	40755184	40756342	1159	-0.57	0.000229	0.00285	TTC33	40754072
chr16	58767715	58768479	765	-0.74	0.00023	0.00286	GOT2	58766261
chr16	58767715	58768479	765	-0.74	0.00023	0.00286	GOT2	58766261
chr12	72057445	72058319	875	-0.55	0.00023	0.00286	ZFC3H1	72055749
chr12	72057445	72058319	875	-0.55	0.00023	0.00286	THAP2	72052676
chr7	1.06E+08	1.06E+08	547	-0.73	0.000231	0.00286	NAMPT	1.06E+08
chr12	54817037	54817335	299	-0.94	0.000231	0.00286	ITGA5	54811244
chr1	1.57E+08	1.57E+08	541	-0.74	0.000231	0.00287	ETV3	1.57E+08
chr1	1.57E+08	1.57E+08	541	-0.74	0.000231	0.00287	ETV3	1.57E+08
chr3	38206364	38207126	763	-0.63	0.000232	0.00287	OXSR1	38202025
chr15	74753390	74753798	409	-0.95	0.000232	0.00287	UBL7	74751529
chr15	74753390	74753798	409	-0.95	0.000232	0.00287	UBL7-AS1	74748605
chr15	74753390	74753798	409	-0.95	0.000232	0.00287	UBL7-AS1	74748605
chr15	74753390	74753798	409	-0.95	0.000232	0.00287	UBL7	74751529
chr15	74753390	74753798	409	-0.95	0.000232	0.00287	UBL7	74751529
chr15	74753390	74753798	409	-0.95	0.000232	0.00287	UBL7	74751529
chr15	74753390	74753798	409	-0.95	0.000232	0.00287	UBL7	74751529
chr15	74753390	74753798	409	-0.95	0.000232	0.00287	UBL7	74751529
chr15	74753390	74753798	409	-0.95	0.000232	0.00287	UBL7-AS1	74748605
chr20	749111	749577	467	-0.73	0.000233	0.00288	SLC52A3	747228
chr1	74663607	74664133	527	-0.7	0.000233	0.00288	LRRIQ3	74661871
chr1	74663607	74664133	527	-0.7	0.000233	0.00288	FPGT-TNNI	74658895
chr1	74663607	74664133	527	-0.7	0.000233	0.00288	FPGT-TNNI	74658895
chr1	74663607	74664133	527	-0.7	0.000233	0.00288	FPGT	74658895
chr1	74663607	74664133	527	-0.7	0.000233	0.00288	FPGT	74658895
chr1	74663607	74664133	527	-0.7	0.000233	0.00288	FPGT	74658895
chr7	33148374	33149396	1023	-0.66	0.000234	0.00289	RP9	33147002
chr8	97505717	97506347	631	-0.6	0.000235	0.0029	SDC2	97500881
chr3	1.41E+08	1.41E+08	594	-0.53	0.000235	0.0029	SLC25A36	1.41E+08
chr3	1.41E+08	1.41E+08	594	-0.53	0.000235	0.0029	SLC25A36	1.41E+08
chr2	1.36E+08	1.36E+08	567	-0.59	0.000236	0.00291	UBXN4	1.36E+08
chr8	95731733	95732573	841	-0.73	0.000236	0.00291	DPY19L4	95727102
chr18	71814915	71815887	973	-0.51	0.000237	0.00291	FBXO15	71812999
chr18	71814915	71815887	973	-0.51	0.000237	0.00291	TIMM21	71810745
chr18	71814915	71815887	973	-0.51	0.000237	0.00291	FBXO15	71813100
chr1	1.53E+08	1.53E+08	470	-0.86	0.000238	0.00292	IVL	1.53E+08
chr3	1.53E+08	1.53E+08	1007	-0.5	0.000238	0.00292	RAP2B	1.53E+08
chr2	1.06E+08	1.06E+08	770	-0.48	0.000239	0.00292	LOC10272	1.06E+08
chr2	1.06E+08	1.06E+08	770	-0.48	0.000239	0.00292	MRPS9	1.06E+08
chr7	39772754	39773277	524	-0.6	0.000239	0.00292	LINC00265	39768166
chr11	64545967	64546648	682	-0.5	0.000239	0.00293	SF1	64544316
chr11	64545967	64546648	682	-0.5	0.000239	0.00293	SF1	64543232
chr11	64545967	64546648	682	-0.5	0.000239	0.00293	SF1	64543938
chr11	64545967	64546648	682	-0.5	0.000239	0.00293	SF1	64544316

chr11	64545967	64546648	682	-0.5	0.000239	0.00293	SF1	64544316
chr11	64545967	64546648	682	-0.5	0.000239	0.00293	SF1	64544316
chr11	64545967	64546648	682	-0.5	0.000239	0.00293	SF1	64544316
chr11	64545967	64546648	682	-0.5	0.000239	0.00293	SF1	64544316
chr13	37007214	37007349	136	-1.98	0.00024	0.00294	CCNA1	37000966
chr13	37007214	37007349	136	-1.98	0.00024	0.00294	CCNA1	37001408
chr13	37007214	37007349	136	-1.98	0.00024	0.00294	CCNA1	37000966
chr13	37007214	37007349	136	-1.98	0.00024	0.00294	CCNA1	37001408
chr4	1.2E+08	1.2E+08	584	-0.76	0.000241	0.00294	LOC64551	1.2E+08
chr10	1.12E+08	1.12E+08	1510	-0.64	0.000242	0.00295	ADD3	1.12E+08
chr10	1.12E+08	1.12E+08	1510	-0.64	0.000242	0.00295	ADD3	1.12E+08
chr10	1.12E+08	1.12E+08	1510	-0.64	0.000242	0.00295	ADD3	1.12E+08
chr10	1.12E+08	1.12E+08	1510	-0.64	0.000242	0.00295	ADD3-AS1	1.12E+08
chr9	91266897	91267577	681	-0.78	0.000242	0.00295	LOC28623	91265075
chr8	77912213	77913109	897	-0.51	0.000242	0.00295	PEX2	77910524
chr8	77912213	77913109	897	-0.51	0.000242	0.00295	PEX2	77911280
chr8	77912213	77913109	897	-0.51	0.000242	0.00295	PEX2	77911280
chr8	77912213	77913109	897	-0.51	0.000242	0.00295	PEX2	77910524
chr1	6085410	6086467	1058	-0.74	0.000244	0.00297	KCNAB2	6081072
chr1	6085410	6086467	1058	-0.74	0.000244	0.00297	KCNAB2	6081072
chr5	1.15E+08	1.15E+08	857	-0.56	0.000244	0.00297	TMED7-TIC	1.15E+08
chr5	1.15E+08	1.15E+08	857	-0.56	0.000244	0.00297	TMED7-TIC	1.15E+08
chr5	1.15E+08	1.15E+08	857	-0.56	0.000244	0.00297	TMED7	1.15E+08
chr4	1.4E+08	1.4E+08	968	-0.61	0.000245	0.00297	RAB33B	1.4E+08
chr5	41869753	41870838	1086	-0.49	0.000245	0.00298	OXCT1	41868791
chr5	41869753	41870838	1086	-0.49	0.000245	0.00298	OXCT1-AS1	41865131
chr13	36429715	36430124	410	-0.89	0.000246	0.00298	DCLK1	36427998
chr13	36429715	36430124	410	-0.89	0.000246	0.00298	DCLK1	36427998
chr13	36429715	36430124	410	-0.89	0.000246	0.00298	DCLK1	36427998
chr4	81256722	81257217	496	-0.85	0.000246	0.00298	C4orf22	81251873
chr4	81256722	81257217	496	-0.85	0.000246	0.00298	C4orf22	81251873
chr3	99355931	99356402	472	-1.18	0.000246	0.00298	COL8A1	99352439
chr3	99355931	99356402	472	-1.18	0.000246	0.00298	COL8A1	99352439
chr13	99849135	99849621	487	-0.91	0.000247	0.00299	UBAC2	99847678
chr13	99849135	99849621	487	-0.91	0.000247	0.00299	UBAC2	99848162
chr13	99849135	99849621	487	-0.91	0.000247	0.00299	UBAC2	99848162
chr20	4129230	4129618	389	-0.93	0.00025	0.00301	SMOX	4124425
chr20	4129230	4129618	389	-0.93	0.00025	0.00301	SMOX	4124425
chr20	4129230	4129618	389	-0.93	0.00025	0.00301	SMOX	4124425
chr20	4129230	4129618	389	-0.93	0.00025	0.00301	SMOX	4124425
chr20	4129230	4129618	389	-0.93	0.00025	0.00301	SMOX	4124425
chr2	1.67E+08	1.67E+08	842	-0.55	0.000251	0.00302	LOC10272	1.67E+08
chr2	1.67E+08	1.67E+08	842	-0.55	0.000251	0.00302	TTC21B	1.67E+08
chr14	58666473	58667173	701	-0.58	0.000252	0.00303	ACTR10	58661832
chr3	1.79E+08	1.79E+08	1128	-0.45	0.000253	0.00303	ZNF639	1.79E+08
chr3	1.79E+08	1.79E+08	1128	-0.45	0.000253	0.00303	ZNF639	1.79E+08
chr3	1.79E+08	1.79E+08	1128	-0.45	0.000253	0.00303	ZNF639	1.79E+08

chr12	49716730	49717093	364	-0.74	0.000253	0.00303	TROAP	49711970
chr12	49716730	49717093	364	-0.74	0.000253	0.00303	TROAP	49711970
chr12	49716730	49717093	364	-0.74	0.000253	0.00303	TROAP	49711970
chr12	49716730	49717093	364	-0.74	0.000253	0.00303	LOC101927	49716359
chr19	11485208	11485869	662	-0.49	0.000253	0.00304	SWSAP1	11480382
chr8	53853299	53854428	1130	-0.48	0.000254	0.00304	NPBWR1	53847467
chr1	1.11E+08	1.11E+08	363	-0.95	0.000254	0.00304	SLC16A4	1.11E+08
chr1	1.11E+08	1.11E+08	363	-0.95	0.000254	0.00304	SLC16A4	1.11E+08
chr1	1.11E+08	1.11E+08	363	-0.95	0.000254	0.00304	SLC16A4	1.11E+08
chr1	1.11E+08	1.11E+08	363	-0.95	0.000254	0.00304	SLC16A4	1.11E+08
chr1	1.11E+08	1.11E+08	363	-0.95	0.000254	0.00304	SLC16A4	1.11E+08
chr14	64009769	64010515	747	-0.51	0.000255	0.00305	PPP2R5E	64008079
chr14	64009769	64010515	747	-0.51	0.000255	0.00305	PPP2R5E	64008079
chr14	64009769	64010515	747	-0.51	0.000255	0.00305	PPP2R5E	64008079
chr14	64009769	64010515	747	-0.51	0.000255	0.00305	PPP2R5E	64008079
chr4	15682986	15683550	565	-0.67	0.000255	0.00305	FAM200B	15678351
chr11	1.26E+08	1.26E+08	1272	-0.43	0.000255	0.00305	HYLS1	1.26E+08
chr7	39660888	39661118	231	-0.91	0.000257	0.00306	RALA	39658151
chr15	49462182	49462691	510	-0.63	0.000257	0.00306	GALK2	49457212
chr15	49462182	49462691	510	-0.63	0.000257	0.00306	GALK2	49457212
chr15	49462182	49462691	510	-0.63	0.000257	0.00306	GALK2	49457212
chr15	49462182	49462691	510	-0.63	0.000257	0.00306	MIR4716	49459350
chr6	74776031	74776528	498	-0.83	0.000257	0.00306	LOC101928	74774166
chr1	1.83E+08	1.83E+08	1172	-0.74	0.000258	0.00307	SMG7	1.83E+08
chr1	1.83E+08	1.83E+08	1172	-0.74	0.000258	0.00307	SMG7	1.83E+08
chr1	1.83E+08	1.83E+08	1172	-0.74	0.000258	0.00307	SMG7	1.83E+08
chr1	1.83E+08	1.83E+08	1172	-0.74	0.000258	0.00307	SMG7	1.83E+08
chr1	1.83E+08	1.83E+08	1172	-0.74	0.000258	0.00307	SMG7-AS1	1.83E+08
chr7	93188109	93188385	277	-1.49	0.000258	0.00307	CALCR	93187122
chr17	79856303	79856753	451	-0.99	0.000258	0.00307	NPB	79854984
chr7	33080456	33080754	299	-0.9	0.000258	0.00307	NT5C3A	33078777
chr7	33080456	33080754	299	-0.9	0.000258	0.00307	NT5C3A	33078777
chr9	1.31E+08	1.31E+08	330	-0.7	0.000258	0.00307	SLC25A25	1.31E+08
chr9	1.31E+08	1.31E+08	330	-0.7	0.000258	0.00307	SLC25A25	1.31E+08
chr11	1.01E+08	1.01E+08	1131	-0.59	0.000258	0.00307	PGR	1.01E+08
chr11	1.01E+08	1.01E+08	1131	-0.59	0.000258	0.00307	PGR	1.01E+08
chr11	1.01E+08	1.01E+08	1131	-0.59	0.000258	0.00307	PGR	1.01E+08
chr11	1.01E+08	1.01E+08	1131	-0.59	0.000258	0.00307	PGR	1.01E+08
chr11	1.01E+08	1.01E+08	1131	-0.59	0.000258	0.00307	PGR	1.01E+08
chr11	1.01E+08	1.01E+08	1131	-0.59	0.000258	0.00307	PGR	1.01E+08
chr11	1.01E+08	1.01E+08	1131	-0.59	0.000258	0.00307	PGR	1.01E+08
chr11	1.01E+08	1.01E+08	1131	-0.59	0.000258	0.00307	LOC101054	1.01E+08
chr2	54950405	54950945	541	-0.67	0.000259	0.00307	EML6	54947148
chr3	1.85E+08	1.85E+08	488	-0.94	0.00026	0.00308	MAP3K13	1.85E+08
chr19	51227822	51228517	696	-0.75	0.000262	0.00309	CLEC11A	51221604
chr2	1.98E+08	1.98E+08	943	-0.51	0.000262	0.0031	COQ10B	1.98E+08
chrX	1.3E+08	1.3E+08	586	-0.66	0.000264	0.00312	ENOX2	1.3E+08

chrX	1.3E+08	1.3E+08	586	-0.66	0.000264	0.00312	ENOX2	1.3E+08
chrX	1.3E+08	1.3E+08	586	-0.66	0.000264	0.00312	ENOX2	1.3E+08
chr8	81083357	81083994	638	-0.68	0.000264	0.00312	TPD52	81081894
chr8	81083357	81083994	638	-0.68	0.000264	0.00312	TPD52	81081894
chr8	81083357	81083994	638	-0.68	0.000264	0.00312	TPD52	81081894
chr8	81083357	81083994	638	-0.68	0.000264	0.00312	TPD52	81081435
chr8	81083357	81083994	638	-0.68	0.000264	0.00312	TPD52	81081894
chr8	81083357	81083994	638	-0.68	0.000264	0.00312	TPD52	81081435
chr8	81083357	81083994	638	-0.68	0.000264	0.00312	TPD52	81081894
chr5	16508931	16509407	477	-0.67	0.000265	0.00312	FAM134B	16507107
chr9	20684055	20684861	807	-0.64	0.000266	0.00312	FOCAD-AS1	20682749
chr8	49835955	49836291	337	-0.64	0.000266	0.00313	SNAI2	49831999
chr5	52405296	52405850	555	-0.82	0.000266	0.00313	LOC25739(	52400671
chr5	52405296	52405850	555	-0.82	0.000266	0.00313	MOCS2	52403602
chr5	52405296	52405850	555	-0.82	0.000266	0.00313	MOCS2	52403602
chr5	52405296	52405850	555	-0.82	0.000266	0.00313	LOC25739(	52400671
chr12	13025181	13026157	977	-0.55	0.000266	0.00313	RPL13AP2(	13023410
chr16	16040139	16040547	409	-0.9	0.000266	0.00313	ABCC1	16038433
chr6	1.49E+08	1.49E+08	999	-0.64	0.000267	0.00313	LOC10537{	1.49E+08
chr15	85144010	85144339	330	-0.91	0.000268	0.00314	ZSCAN2	85139248
chr15	85144010	85144339	330	-0.91	0.000268	0.00314	ZSCAN2	85139248
chr15	85144010	85144339	330	-0.91	0.000268	0.00314	ZSCAN2	85139248
chr19	50269554	50270200	647	-0.56	0.000268	0.00314	AP2A1	50265179
chr19	50269554	50270200	647	-0.56	0.000268	0.00314	TSKS	50264515
chr19	50269554	50270200	647	-0.56	0.000268	0.00314	AP2A1	50265179
chr9	1.16E+08	1.16E+08	768	-0.53	0.000269	0.00314	CDC26	1.16E+08
chr9	1.16E+08	1.16E+08	768	-0.53	0.000269	0.00314	PRPF4	1.16E+08
chr9	1.16E+08	1.16E+08	768	-0.53	0.000269	0.00314	PRPF4	1.16E+08
chr19	41873693	41874006	314	-1.09	0.000271	0.00316	B9D2	41868078
chr2	88926788	88927725	938	-0.43	0.000271	0.00316	EIF2AK3	88925094
chr2	88926788	88927725	938	-0.43	0.000271	0.00316	EIF2AK3	88922447
chr11	65819424	65819901	478	-0.63	0.000272	0.00316	SF3B2	65814815
chr11	65819424	65819901	478	-0.63	0.000272	0.00316	GAL3ST3	65814651
chr3	1.44E+08	1.44E+08	321	-0.78	0.000272	0.00316	SLC9A9	1.44E+08
chr10	1.3E+08	1.3E+08	390	-0.58	0.000272	0.00316	PTPRE	1.3E+08
chr15	1E+08	1E+08	506	-0.8	0.000273	0.00317	LYSMD4	1E+08
chr15	1E+08	1E+08	506	-0.8	0.000273	0.00317	LYSMD4	1E+08
chr15	1E+08	1E+08	506	-0.8	0.000273	0.00317	LYSMD4	1E+08
chr15	1E+08	1E+08	506	-0.8	0.000273	0.00317	LYSMD4	1E+08
chr15	1E+08	1E+08	506	-0.8	0.000273	0.00317	LYSMD4	1E+08
chr15	1E+08	1E+08	506	-0.8	0.000273	0.00317	LYSMD4	1E+08
chr15	1E+08	1E+08	506	-0.8	0.000273	0.00317	LYSMD4	1E+08
chr13	1.07E+08	1.07E+08	349	-0.99	0.000273	0.00317	LINC00443	1.07E+08
chr4	1.01E+08	1.01E+08	568	-0.58	0.000274	0.00318	LOC25688(	1.01E+08
chr4	1.01E+08	1.01E+08	568	-0.58	0.000274	0.00318	DNAJB14	1.01E+08
chr4	1.01E+08	1.01E+08	568	-0.58	0.000274	0.00318	H2AFZ	1.01E+08
chr4	1.01E+08	1.01E+08	568	-0.58	0.000274	0.00318	DNAJB14	1.01E+08

chr4	1.01E+08	1.01E+08	568	-0.58	0.000274	0.00318	DNAJB14	1.01E+08
chr1	68962408	68963054	647	-0.72	0.000274	0.00318	DEPDC1	68960904
chr1	68962408	68963054	647	-0.72	0.000274	0.00318	DEPDC1	68960904
chr1	68962408	68963054	647	-0.72	0.000274	0.00318	DEPDC1-AS	68957358
chr12	58087517	58088003	487	-0.63	0.000274	0.00318	OS9	58082737
chr12	58087517	58088003	487	-0.63	0.000274	0.00318	OS9	58082737
chr12	58087517	58088003	487	-0.63	0.000274	0.00318	OS9	58082737
chr12	58087517	58088003	487	-0.63	0.000274	0.00318	OS9	58082737
chr12	58087517	58088003	487	-0.63	0.000274	0.00318	OS9	58082737
chr12	58087517	58088003	487	-0.63	0.000274	0.00318	OS9	58082737
chr12	58087517	58088003	487	-0.63	0.000274	0.00318	OS9	58082737
chr12	58087517	58088003	487	-0.63	0.000274	0.00318	OS9	58082737
chr14	21571812	21572873	1062	-0.54	0.000275	0.00319	ZNF219	21565173
chr14	21571812	21572873	1062	-0.54	0.000275	0.00319	ZNF219	21570863
chr8	25315847	25316677	831	-0.53	0.000275	0.00319	KCTD9	25313984
chr8	25315847	25316677	831	-0.53	0.000275	0.00319	CDCA2	25311446
chr8	25315847	25316677	831	-0.53	0.000275	0.00319	CDCA2	25311446
chr8	25315847	25316677	831	-0.53	0.000275	0.00319	CDCA2	25311446
chr6	1.14E+08	1.14E+08	418	-0.97	0.000278	0.00321	LOC101927	1.14E+08
chr11	1.34E+08	1.34E+08	332	-0.68	0.00028	0.00323	LOC100128	1.34E+08
chr19	42772415	42773429	1015	-0.46	0.000282	0.00324	CIC	42767688
chr12	38710313	38710843	531	-0.69	0.000283	0.00325	ALG10B	38705556
chr12	38710313	38710843	531	-0.69	0.000283	0.00325	ALG10B	38705556
chr15	44487379	44487782	404	-0.72	0.000284	0.00326	FRMD5	44485492
chr15	44487379	44487782	404	-0.72	0.000284	0.00326	FRMD5	44485492
chr15	44487379	44487782	404	-0.72	0.000284	0.00326	FRMD5	44484644
chr15	44487379	44487782	404	-0.72	0.000284	0.00326	FRMD5	44485492
chr1	1.09E+08	1.09E+08	337	-0.89	0.000285	0.00327	VAV3	1.09E+08
chr1	1.09E+08	1.09E+08	337	-0.89	0.000285	0.00327	VAV3-AS1	1.09E+08
chr1	2.13E+08	2.13E+08	657	-0.69	0.000286	0.00328	ANGEL2	2.13E+08
chr1	2.13E+08	2.13E+08	657	-0.69	0.000286	0.00328	ANGEL2	2.13E+08
chr1	2.13E+08	2.13E+08	657	-0.69	0.000286	0.00328	ANGEL2	2.13E+08
chr1	2.13E+08	2.13E+08	657	-0.69	0.000286	0.00328	ANGEL2	2.13E+08
chr1	2.13E+08	2.13E+08	657	-0.69	0.000286	0.00328	ANGEL2	2.13E+08
chr1	2.13E+08	2.13E+08	657	-0.69	0.000286	0.00328	ANGEL2	2.13E+08
chr12	27234640	27234909	270	-1.14	0.000288	0.00329	C12orf71	27233455
chr12	42876722	42877668	947	-0.74	0.000289	0.0033	PRICKLE1	42875797
chr12	42876722	42877668	947	-0.74	0.000289	0.0033	PRICKLE1	42875416
chr12	42876722	42877668	947	-0.74	0.000289	0.0033	PRICKLE1	42875797
chr3	1.94E+08	1.94E+08	799	-0.78	0.000291	0.00331	LINC00884	1.94E+08
chr1	28974908	28975977	1070	-0.63	0.000291	0.00331	RNU11	28970111
chr16	46722206	46723912	1707	-0.42	0.000293	0.00332	ORC6	46718557
chr16	46722206	46723912	1707	-0.42	0.000293	0.00332	VPS35	46721144
chr16	46722206	46723912	1707	-0.42	0.000293	0.00332	ORC6	46718557
chr4	1.6E+08	1.6E+08	545	-0.55	0.000294	0.00333	ETFDH	1.6E+08
chr4	1.6E+08	1.6E+08	545	-0.55	0.000294	0.00333	ETFDH	1.6E+08
chr4	1.6E+08	1.6E+08	545	-0.55	0.000294	0.00333	C4orf46	1.6E+08

chr4	1.6E+08	1.6E+08	545	-0.55	0.000294	0.00333	C4orf46	1.6E+08
chr4	1.6E+08	1.6E+08	545	-0.55	0.000294	0.00333	C4orf46	1.6E+08
chr6	96025176	96026047	872	-0.56	0.000294	0.00333	MANEA-AS	96023327
chr6	96025176	96026047	872	-0.56	0.000294	0.00333	MANEA	96020372
chr6	96025176	96026047	872	-0.56	0.000294	0.00333	MANEA-AS	96023327
chr20	25990312	25990636	325	-0.9	0.000295	0.00334	LOC10013[illegible]	25985434
chr1	1.71E+08	1.71E+08	435	-0.6	0.000296	0.00335	FMO4	1.71E+08
chr9	19102801	19103203	403	-1.03	0.000296	0.00335	HAUS6	19100940
chr9	19102801	19103203	403	-1.03	0.000296	0.00335	HAUS6	19100940
chr3	13589519	13589789	271	-1.33	0.000298	0.00336	FBLN2	13585624
chr3	13589519	13589789	271	-1.33	0.000298	0.00336	FBLN2	13585624
chr14	73493585	73494147	563	-0.62	0.000298	0.00336	ZFYVE1	73491920
chr14	73493585	73494147	563	-0.62	0.000298	0.00336	ZFYVE1	73491920
chr3	1.09E+08	1.09E+08	409	-0.79	0.000299	0.00337	GUCA1C	1.09E+08
chr1	1.12E+08	1.12E+08	880	-0.57	0.000299	0.00337	LRIF1	1.12E+08
chr1	1.12E+08	1.12E+08	880	-0.57	0.000299	0.00337	LRIF1	1.12E+08
chr9	99381782	99382455	674	-0.56	0.000301	0.00338	CDC14B	99380112
chr9	99381782	99382455	674	-0.56	0.000301	0.00338	CDC14B	99380112
chr1	95538082	95538802	721	-0.51	0.000301	0.00338	ALG14	95536507
chr1	95538082	95538802	721	-0.51	0.000301	0.00338	ALG14	95536507
chr1	95538082	95538802	721	-0.51	0.000301	0.00338	ALG14	95536507
chr8	1.21E+08	1.21E+08	347	-0.71	0.000302	0.00339	ENPP2	1.21E+08
chr8	1.21E+08	1.21E+08	347	-0.71	0.000302	0.00339	ENPP2	1.21E+08
chr8	1.21E+08	1.21E+08	347	-0.71	0.000302	0.00339	ENPP2	1.21E+08
chr1	1.11E+08	1.11E+08	792	-0.57	0.000302	0.00339	CYMP	1.11E+08
chr7	25990728	25991295	568	-0.76	0.000303	0.0034	MIR148A	25987606
chr10	64576089	64576645	557	-0.65	0.000303	0.0034	EGR2	64574126
chr10	64576089	64576645	557	-0.65	0.000303	0.0034	EGR2	64574126
chr10	64576089	64576645	557	-0.65	0.000303	0.0034	EGR2	64574126
chr14	36538089	36538887	799	-0.65	0.000303	0.0034	LINC00609	36534632
chr21	30448169	30448514	346	-1.01	0.000304	0.00341	MAP3K7CL	30444791
chr21	30448169	30448514	346	-1.01	0.000304	0.00341	MAP3K7CL	30444791
chr21	30448169	30448514	346	-1.01	0.000304	0.00341	MAP3K7CL	30444791
chr21	30448169	30448514	346	-1.01	0.000304	0.00341	MAP3K7CL	30444791
chr21	30448169	30448514	346	-1.01	0.000304	0.00341	MAP3K7CL	30444791
chr21	30448169	30448514	346	-1.01	0.000304	0.00341	CCT8	30444118
chr21	30448169	30448514	346	-1.01	0.000304	0.00341	CCT8	30444118
chr21	30448169	30448514	346	-1.01	0.000304	0.00341	MAP3K7CL	30444791
chr21	30448169	30448514	346	-1.01	0.000304	0.00341	CCT8	30444118
chr21	30448169	30448514	346	-1.01	0.000304	0.00341	CCT8	30444118
chr1	15853000	15853654	655	-0.57	0.000304	0.00341	DNAJC16	15848307
chr1	15853000	15853654	655	-0.57	0.000304	0.00341	DNAJC16	15848307
chr1	15853000	15853654	655	-0.57	0.000304	0.00341	DNAJC16	15848307
chr1	15853000	15853654	655	-0.57	0.000304	0.00341	CASP9	15848940
chr1	15853000	15853654	655	-0.57	0.000304	0.00341	CASP9	15849285
chr1	15853000	15853654	655	-0.57	0.000304	0.00341	CASP9	15848940
chr1	15853000	15853654	655	-0.57	0.000304	0.00341	CASP9	15848940

chr1	15853000	15853654	655	-0.57	0.000304	0.00341	CASP9	15848940
chr2	2.03E+08	2.03E+08	566	-0.75	0.000305	0.00341	SNORD70	2.03E+08
chr16	75301825	75302040	216	-1.11	0.000305	0.00341	BCAR1	75298707
chr16	75301825	75302040	216	-1.11	0.000305	0.00341	BCAR1	75299951
chr16	75301825	75302040	216	-1.11	0.000305	0.00341	BCAR1	75297905
chr16	75301825	75302040	216	-1.11	0.000305	0.00341	BCAR1	75297846
chr16	75301825	75302040	216	-1.11	0.000305	0.00341	BCAR1	75297905
chr9	15308610	15308765	156	-1.2	0.000305	0.00341	TTC39B	15305358
chr9	15308610	15308765	156	-1.2	0.000305	0.00341	TTC39B	15305358
chr9	15308610	15308765	156	-1.2	0.000305	0.00341	TTC39B	15305358
chr9	15308610	15308765	156	-1.2	0.000305	0.00341	TTC39B	15305358
chr6	18387259	18387857	599	-0.66	0.000305	0.00341	RNF144B	18382580
chr10	77189900	77190263	364	-0.82	0.000306	0.00342	C10orf11	77186403
chr16	84676774	84677184	411	-1.04	0.000306	0.00342	KLHL36	84677116
chr16	84676774	84677184	411	-1.04	0.000306	0.00342	KLHL36	84677116
chr10	43249701	43250240	540	-0.82	0.000306	0.00342	LOC105378	43245589
chr14	24896504	24896679	176	-0.88	0.000306	0.00342	KHNYN	24894140
chr14	24896504	24896679	176	-0.88	0.000306	0.00342	KHNYN	24894140
chr14	24896504	24896679	176	-0.88	0.000306	0.00342	KHNYN	24893491
chr20	57617451	57618198	748	-0.62	0.000307	0.00343	PRELID3B	57615901
chr20	57617451	57618198	748	-0.62	0.000307	0.00343	PRELID3B	57615901
chr20	57617451	57618198	748	-0.62	0.000307	0.00343	SLMO2-ATI	57615901
chr20	57617451	57618198	748	-0.62	0.000307	0.00343	SLMO2-ATI	57615901
chr8	1.07E+08	1.07E+08	260	-0.97	0.000308	0.00343	OXR1	1.07E+08
chr2	1.67E+08	1.67E+08	330	-0.94	0.000308	0.00343	GALNT3	1.67E+08
chr14	55518061	55518849	789	-0.58	0.000308	0.00343	MAPK1IP1L	55513361
chr10	1.25E+08	1.25E+08	679	-0.61	0.000309	0.00344	LOC399815	1.25E+08
chr10	1.25E+08	1.25E+08	679	-0.61	0.000309	0.00344	FAM24B	1.25E+08
chr10	1.25E+08	1.25E+08	679	-0.61	0.000309	0.00344	FAM24B	1.25E+08
chr10	1.25E+08	1.25E+08	679	-0.61	0.000309	0.00344	FAM24B	1.25E+08
chr10	1.25E+08	1.25E+08	679	-0.61	0.000309	0.00344	FAM24B-CU	1.25E+08
chr5	40677254	40677396	143	-1.29	0.00031	0.00345	PTGER4	40675031
chr19	42579946	42580476	531	-0.7	0.00031	0.00345	ZNF574	42575289
chr5	54040121	54040585	465	-0.82	0.000311	0.00346	LOC102467	54038086
chr16	72698811	72699090	280	-1.09	0.000311	0.00346	LINC01572	72696908
chr4	1.12E+08	1.12E+08	410	-0.78	0.000312	0.00346	PITX2	1.12E+08
chr2	1.97E+08	1.97E+08	868	-0.51	0.000313	0.00347	SLC39A10	1.97E+08
chr2	1.97E+08	1.97E+08	868	-0.51	0.000313	0.00347	SLC39A10	1.97E+08
chr2	37416981	37417249	269	-1.06	0.000313	0.00347	SULT6B1	37413690
chr3	1.79E+08	1.79E+08	588	-0.62	0.000313	0.00347	MRPL47	1.79E+08
chr3	1.79E+08	1.79E+08	588	-0.62	0.000313	0.00347	MRPL47	1.79E+08
chr3	1.79E+08	1.79E+08	588	-0.62	0.000313	0.00347	NDUFB5	1.79E+08
chr3	1.79E+08	1.79E+08	588	-0.62	0.000313	0.00347	NDUFB5	1.79E+08
chr3	1.79E+08	1.79E+08	588	-0.62	0.000313	0.00347	NDUFB5	1.79E+08
chr4	1.2E+08	1.2E+08	213	-1.13	0.000313	0.00347	USP53	1.2E+08
chr11	6343350	6343561	212	-0.79	0.000313	0.00347	PRKCDBP	6339740
chr20	30160328	30161289	962	-0.48	0.000314	0.00348	HM13-AS1	30159066

chr15	45926825	45927681	857	-0.72	0.000317	0.0035	SQRDL	45921937
chr7	1.08E+08	1.08E+08	621	-0.55	0.000321	0.00353	THAP5	1.08E+08
chr7	1.08E+08	1.08E+08	621	-0.55	0.000321	0.00353	THAP5	1.08E+08
chr7	1.08E+08	1.08E+08	621	-0.55	0.000321	0.00353	THAP5	1.08E+08
chr7	1.08E+08	1.08E+08	621	-0.55	0.000321	0.00353	THAP5	1.08E+08
chr7	1.08E+08	1.08E+08	621	-0.55	0.000321	0.00353	THAP5	1.08E+08
chr7	1.08E+08	1.08E+08	621	-0.55	0.000321	0.00353	DNAJB9	1.08E+08
chr1	1.09E+08	1.09E+08	456	-0.63	0.000321	0.00353	GPSM2	1.09E+08
chr13	53226270	53227246	977	-0.55	0.000321	0.00353	SUGT1	53221830
chr13	53226270	53227246	977	-0.55	0.000321	0.00353	SUGT1	53221830
chr2	2.09E+08	2.09E+08	871	-0.57	0.000322	0.00353	CCNYL1	2.09E+08
chr2	2.09E+08	2.09E+08	871	-0.57	0.000322	0.00353	CCNYL1	2.09E+08
chr10	27388937	27389693	757	-0.64	0.000322	0.00354	ANKRD26	27387427
chr10	27388937	27389693	757	-0.64	0.000322	0.00354	ANKRD26	27387427
chr7	1.16E+08	1.16E+08	340	-0.7	0.000325	0.00356	CAV1	1.16E+08
chr7	1.16E+08	1.16E+08	340	-0.7	0.000325	0.00356	CAV1	1.16E+08
chr7	1.16E+08	1.16E+08	340	-0.7	0.000325	0.00356	CAV1	1.16E+08
chr7	1.16E+08	1.16E+08	340	-0.7	0.000325	0.00356	CAV1	1.16E+08
chr5	1.34E+08	1.34E+08	258	-0.96	0.000325	0.00356	LOC10254(	1.34E+08
chr12	8395507	8396449	943	-0.55	0.000327	0.00357	FAM86FP	8393542
chr4	79567100	79567852	753	-0.85	0.000327	0.00358	LINC01094	79562147
chr4	79567100	79567852	753	-0.85	0.000327	0.00358	LINC01094	79562147
chr4	79567100	79567852	753	-0.85	0.000327	0.00358	LINC01094	79562147
chr4	79567100	79567852	753	-0.85	0.000327	0.00358	LINC01094	79562147
chr4	79567100	79567852	753	-0.85	0.000327	0.00358	LINC01094	79562147
chr4	79567100	79567852	753	-0.85	0.000327	0.00358	LINC01094	79562147
chr9	1.24E+08	1.24E+08	771	-0.52	0.000329	0.00359	C5	1.24E+08
chr11	17036828	17037217	390	-1.05	0.000329	0.00359	PLEKHA7	17033963
chr12	69979071	69979735	665	-0.85	0.000329	0.00359	CCT2	69974207
chr12	69979071	69979735	665	-0.85	0.000329	0.00359	CCT2	69974461
chr12	69979071	69979735	665	-0.85	0.000329	0.00359	MIR3913-2	69973502
chr12	69979071	69979735	665	-0.85	0.000329	0.00359	MIR3913-1	69976603
chr19	49955063	49955257	195	-1.04	0.00033	0.0036	PIH1D1	49953115
chr19	49955063	49955257	195	-1.04	0.00033	0.0036	ALDH16A1	49951472
chr19	49955063	49955257	195	-1.04	0.00033	0.0036	ALDH16A1	49951472
chr11	85339188	85339767	580	-0.64	0.00033	0.0036	DLG2	85336314
chr11	85339188	85339767	580	-0.64	0.00033	0.0036	TMEM126I	85334616
chr11	85339188	85339767	580	-0.64	0.00033	0.0036	TMEM126I	85334616
chr11	85339188	85339767	580	-0.64	0.00033	0.0036	TMEM126I	85334616
chr11	85339188	85339767	580	-0.64	0.00033	0.0036	TMEM126I	85334616
chr11	85339188	85339767	580	-0.64	0.00033	0.0036	TMEM126I	85334616
chr6	70571871	70572217	347	-0.88	0.00033	0.0036	COL19A1	70571447
chr9	86087207	86087500	294	-0.75	0.000331	0.0036	FRMD3	86080657
chr12	56211405	56212032	628	-0.53	0.000335	0.00364	SARNP	56209540
chr12	56211405	56212032	628	-0.53	0.000335	0.00364	ORMDL2	56206805
chr12	56211405	56212032	628	-0.53	0.000335	0.00364	SARNP	56209540
chr12	56211405	56212032	628	-0.53	0.000335	0.00364	SARNP	56209540

chr16	85648689	85648929	241	-0.87	0.000335	0.00364	GSE1	85641841
chr16	85648689	85648929	241	-0.87	0.000335	0.00364	GSE1	85641841
chr16	58163137	58163672	536	-0.64	0.000335	0.00364	CFAP20	58161296
chr14	71248937	71249473	537	-0.64	0.000336	0.00364	MAP3K9	71248169
chr14	71248937	71249473	537	-0.64	0.000336	0.00364	MAP3K9	71248169
chr5	1.09E+08	1.09E+08	1064	-0.49	0.000336	0.00364	PJA2	1.09E+08
chr12	1.21E+08	1.21E+08	898	-0.49	0.000336	0.00365	MLEC	1.21E+08
chr12	1.21E+08	1.21E+08	898	-0.49	0.000336	0.00365	MLEC	1.21E+08
chr12	1.21E+08	1.21E+08	898	-0.49	0.000336	0.00365	MLEC	1.21E+08
chr1	1.85E+08	1.85E+08	588	-0.65	0.000337	0.00365	EDEM3	1.85E+08
chr9	27528307	27528577	271	-1.17	0.000338	0.00366	MOB3B	27527850
chr12	53318903	53319469	567	-0.79	0.000339	0.00366	KRT8	53318253
chr8	95652615	95653383	769	-0.57	0.000339	0.00367	ESRP1	95648363
chr8	95652615	95653383	769	-0.57	0.000339	0.00367	ESRP1	95648363
chr8	95652615	95653383	769	-0.57	0.000339	0.00367	ESRP1	95648363
chr8	95652615	95653383	769	-0.57	0.000339	0.00367	ESRP1	95648363
chr8	95652615	95653383	769	-0.57	0.000339	0.00367	ESRP1	95648363
chr8	95652615	95653383	769	-0.57	0.000339	0.00367	LOC10028ε	95649695
chr8	1.36E+08	1.36E+08	657	-0.62	0.000342	0.00368	MIR30B	1.36E+08
chr1	1.11E+08	1.11E+08	617	-0.52	0.000343	0.0037	AHCYL1	1.11E+08
chr1	1.11E+08	1.11E+08	617	-0.52	0.000343	0.0037	AHCYL1	1.11E+08
chr9	91925409	91926256	848	-0.46	0.000346	0.00372	MIR3153	91922139
chr9	91925409	91926256	848	-0.46	0.000346	0.00372	CKS2	91921109
chr2	676140	677595	1456	-0.47	0.000346	0.00372	TMEM18	675439
chr4	1.74E+08	1.74E+08	1175	-0.58	0.000346	0.00372	HMGB2	1.74E+08
chr4	1.74E+08	1.74E+08	1175	-0.58	0.000346	0.00372	HMGB2	1.74E+08
chr4	1.74E+08	1.74E+08	1175	-0.58	0.000346	0.00372	HMGB2	1.74E+08
chr1	2.15E+08	2.15E+08	1133	-0.53	0.000346	0.00372	PTPN14	2.15E+08
chr14	35590875	35591925	1051	-0.58	0.000347	0.00373	KIAA0391	35586757
chr14	35590875	35591925	1051	-0.58	0.000347	0.00373	KIAA0391	35586757
chr14	35590875	35591925	1051	-0.58	0.000347	0.00373	KIAA0391	35586526
chr14	35590875	35591925	1051	-0.58	0.000347	0.00373	KIAA0391	35586526
chr14	35590875	35591925	1051	-0.58	0.000347	0.00373	KIAA0391	35586526
chr14	35590875	35591925	1051	-0.58	0.000347	0.00373	PPP2R3C	35589748
chr14	35590875	35591925	1051	-0.58	0.000347	0.00373	PPP2R3C	35589749
chr14	35590875	35591925	1051	-0.58	0.000347	0.00373	PPP2R3C	35589748
chr14	35590875	35591925	1051	-0.58	0.000347	0.00373	PPP2R3C	35589749
chr10	25305166	25306083	918	-0.6	0.000349	0.00374	ENKUR	25303085
chr10	25305166	25306083	918	-0.6	0.000349	0.00374	ENKUR	25303085
chr10	25305166	25306083	918	-0.6	0.000349	0.00374	ENKUR	25303085
chr10	25305166	25306083	918	-0.6	0.000349	0.00374	THNSL1	25300507
chr16	22308416	22309071	656	-0.62	0.000349	0.00374	POLR3E	22303695
chr16	22308416	22309071	656	-0.62	0.000349	0.00374	POLR3E	22303695
chr16	22308416	22309071	656	-0.62	0.000349	0.00374	POLR3E	22303695
chr16	22308416	22309071	656	-0.62	0.000349	0.00374	POLR3E	22303695
chr16	22308416	22309071	656	-0.62	0.000349	0.00374	POLR3E	22303695
chr16	22308416	22309071	656	-0.62	0.000349	0.00374	POLR3E	22303695

chr12	99038102	99039246	1145	-0.52	0.00035	0.00375	APAF1	99034077
chr12	99038102	99039246	1145	-0.52	0.00035	0.00375	APAF1	99034077
chr12	99038102	99039246	1145	-0.52	0.00035	0.00375	APAF1	99034077
chr12	99038102	99039246	1145	-0.52	0.00035	0.00375	APAF1	99034077
chr12	99038102	99039246	1145	-0.52	0.00035	0.00375	APAF1	99034077
chr12	99038102	99039246	1145	-0.52	0.00035	0.00375	IKBIP	99036829
chr12	99038102	99039246	1145	-0.52	0.00035	0.00375	IKBIP	99036829
chr12	99038102	99039246	1145	-0.52	0.00035	0.00375	IKBIP	99036829
chr7	55260276	55260738	463	-0.66	0.00035	0.00375	EGFR-AS1	55254642
chr5	89705101	89705973	873	-0.57	0.000351	0.00376	CETN3	89703603
chr5	89705101	89705973	873	-0.57	0.000351	0.00376	LOC731157	89700816
chr5	89705101	89705973	873	-0.57	0.000351	0.00376	CETN3	89703603
chr5	89705101	89705973	873	-0.57	0.000351	0.00376	CETN3	89703603
chr2	2.04E+08	2.04E+08	986	-0.5	0.000352	0.00376	RAPH1	2.04E+08
chr2	2.04E+08	2.04E+08	986	-0.5	0.000352	0.00376	RAPH1	2.04E+08
chr3	1.11E+08	1.11E+08	555	-0.54	0.000352	0.00377	PLCXD2	1.11E+08
chr3	1.11E+08	1.11E+08	555	-0.54	0.000352	0.00377	PLCXD2	1.11E+08
chr2	1.98E+08	1.98E+08	528	-0.69	0.000356	0.0038	SF3B1	1.98E+08
chr2	1.98E+08	1.98E+08	528	-0.69	0.000356	0.0038	SF3B1	1.98E+08
chr2	1.98E+08	1.98E+08	528	-0.69	0.000356	0.0038	SF3B1	1.98E+08
chr8	95834977	95835865	889	-0.65	0.00036	0.00383	INTS8	95830517
chr8	95834977	95835865	889	-0.65	0.00036	0.00383	INTS8	95830517
chr8	95834977	95835865	889	-0.65	0.00036	0.00383	INTS8	95830517
chr1	6445731	6446231	501	-0.6	0.00036	0.00383	ACOT7	6443883
chr13	40176745	40177488	744	-0.7	0.00036	0.00383	LHFP	40175356
chr13	1.01E+08	1.01E+08	659	-0.75	0.000361	0.00384	PCCA	1.01E+08
chr13	1.01E+08	1.01E+08	659	-0.75	0.000361	0.00384	PCCA	1.01E+08
chr13	1.01E+08	1.01E+08	659	-0.75	0.000361	0.00384	PCCA	1.01E+08
chr13	1.01E+08	1.01E+08	659	-0.75	0.000361	0.00384	LOC10537(	1.01E+08
chr19	36135278	36135649	372	-0.82	0.000364	0.00386	COX6B1	36134124
chr1	94311439	94312335	897	-0.5	0.000364	0.00386	BCAR3	94310706
chr1	94311439	94312335	897	-0.5	0.000364	0.00386	MIR760	94307387
chr1	1.75E+08	1.75E+08	440	-0.53	0.000366	0.00387	RABGAP1L	1.75E+08
chr9	23826253	23826607	355	-0.62	0.000367	0.00387	ELAVL2	23824063
chr9	23826253	23826607	355	-0.62	0.000367	0.00387	ELAVL2	23819478
chr9	23826253	23826607	355	-0.62	0.000367	0.00387	ELAVL2	23819843
chr6	5082812	5083254	443	-0.86	0.000367	0.00387	PPP1R3G	5080719
chr9	85677153	85678259	1107	-0.51	0.000367	0.00387	RASEF	85676043
chr3	1.98E+08	1.98E+08	669	-0.6	0.000368	0.00389	IQCG	1.98E+08
chr3	1.98E+08	1.98E+08	669	-0.6	0.000368	0.00389	LMLN	1.98E+08
chr3	1.98E+08	1.98E+08	669	-0.6	0.000368	0.00389	LMLN	1.98E+08
chr3	1.98E+08	1.98E+08	669	-0.6	0.000368	0.00389	LMLN	1.98E+08
chr3	1.98E+08	1.98E+08	669	-0.6	0.000368	0.00389	LMLN	1.98E+08
chr1	45769133	45769856	724	-0.65	0.000369	0.00389	LINC01144	45764581
chr12	58158971	58159511	541	-0.72	0.00037	0.00389	CYP27B1	58158976
chr21	29909928	29910366	439	-1.11	0.000372	0.00391	LINC00161	29906639
chr21	29909928	29910366	439	-1.11	0.000372	0.00391	LINC00161	29906639

chr3	1.36E+08	1.36E+08	791	-0.49	0.000374	0.00392	STAG1	1.36E+08
chr2	84743340	84743690	351	-0.67	0.000374	0.00393	DNAH6	84738578
chr10	27792935	27793573	639	-0.55	0.000378	0.00396	RAB18	27788102
chr10	27792935	27793573	639	-0.55	0.000378	0.00396	RAB18	27788102
chr10	27792935	27793573	639	-0.55	0.000378	0.00396	RAB18	27788102
chr10	27792935	27793573	639	-0.55	0.000378	0.00396	RAB18	27788102
chr10	27792935	27793573	639	-0.55	0.000378	0.00396	RAB18	27788102
chr10	27792935	27793573	639	-0.55	0.000378	0.00396	RAB18	27788102
chr3	52739798	52740278	481	-1.19	0.00038	0.00398	GLT8D1	52735714
chr3	52739798	52740278	481	-1.19	0.00038	0.00398	GLT8D1	52738099
chr3	52739798	52740278	481	-1.19	0.00038	0.00398	SPCS1	52734856
chr3	52739798	52740278	481	-1.19	0.00038	0.00398	GLT8D1	52738099
chr3	52739798	52740278	481	-1.19	0.00038	0.00398	GLT8D1	52738099
chr3	52739798	52740278	481	-1.19	0.00038	0.00398	GLT8D1	52738099
chr10	81963345	81964100	756	-0.58	0.00038	0.00398	ANXA11	81963328
chr10	81963345	81964100	756	-0.58	0.00038	0.00398	ANXA11	81963328
chr10	81963345	81964100	756	-0.58	0.00038	0.00398	ANXA11	81963328
chr10	81963345	81964100	756	-0.58	0.00038	0.00398	ANXA11	81963328
chr10	81963345	81964100	756	-0.58	0.00038	0.00398	ANXA11	81962247
chr10	81963345	81964100	756	-0.58	0.00038	0.00398	ANXA11	81963433
chr10	81963345	81964100	756	-0.58	0.00038	0.00398	LINC00857	81962465
chr15	1.01E+08	1.01E+08	433	-0.68	0.000381	0.00399	ADAMTS17	1.01E+08
chr11	43942191	43942641	451	-0.61	0.000382	0.00399	ALKBH3-AS	43940494
chr3	1.8E+08	1.8E+08	739	-0.72	0.000383	0.004	LOC10050!	1.8E+08
chr10	12110630	12111110	481	-0.58	0.000384	0.00401	DHTKD1	12105915
chr7	1.24E+08	1.24E+08	573	-0.66	0.000384	0.00401	GPR37	1.24E+08
chr18	60382217	60382780	564	-0.87	0.000386	0.00402	PHLPP1	60377671
chr8	1.35E+08	1.35E+08	749	-0.45	0.000386	0.00402	ST3GAL1	1.35E+08
chr8	1.35E+08	1.35E+08	749	-0.45	0.000386	0.00402	ST3GAL1	1.35E+08
chr19	49999271	49999676	406	-0.61	0.000387	0.00403	SNORD35B	49995975
chr19	49999271	49999676	406	-0.61	0.000387	0.00403	RPS11	49994621
chr1	1.85E+08	1.85E+08	611	-0.72	0.000388	0.00403	SWT1	1.85E+08
chr1	1.85E+08	1.85E+08	611	-0.72	0.000388	0.00403	SWT1	1.85E+08
chr1	1.85E+08	1.85E+08	611	-0.72	0.000388	0.00403	TRMT1L	1.85E+08
chr1	1.85E+08	1.85E+08	611	-0.72	0.000388	0.00403	TRMT1L	1.85E+08
chr14	51706682	51707095	414	-0.61	0.000391	0.00405	TMX1	51701885
chr7	1.12E+08	1.12E+08	245	-0.82	0.000393	0.00407	TMEM168	1.12E+08
chr7	1.12E+08	1.12E+08	245	-0.82	0.000393	0.00407	TMEM168	1.12E+08
chr7	1.12E+08	1.12E+08	245	-0.82	0.000393	0.00407	TMEM168	1.12E+08
chr20	32699757	32700306	550	-0.78	0.000393	0.00407	EIF2S2	32698162
chr20	32699757	32700306	550	-0.78	0.000393	0.00407	EIF2S2	32698162
chr20	32699757	32700306	550	-0.78	0.000393	0.00407	EIF2S2	32698162
chr6	1.48E+08	1.48E+08	545	-0.69	0.000395	0.00408	STXBP5	1.48E+08
chr6	1.48E+08	1.48E+08	545	-0.69	0.000395	0.00408	STXBP5	1.48E+08
chr6	1.48E+08	1.48E+08	545	-0.69	0.000395	0.00408	STXBP5-AS	1.48E+08
chr1	2322850	2323383	534	-0.61	0.000395	0.00409	MORN1	2321190
chr1	2322850	2323383	534	-0.61	0.000395	0.00409	RER1	2318213

chr1	2322850	2323383	534	-0.61	0.000395	0.00409	MORN1	2321190
chr1	2322850	2323383	534	-0.61	0.000395	0.00409	MORN1	2321190
chr3	38178538	38178939	402	-0.76	0.000396	0.00409	ACAA1	38176733
chr3	38178538	38178939	402	-0.76	0.000396	0.00409	MYD88	38174968
chr3	38178538	38178939	402	-0.76	0.000396	0.00409	MYD88	38174968
chr3	38178538	38178939	402	-0.76	0.000396	0.00409	MYD88	38174968
chr3	38178538	38178939	402	-0.76	0.000396	0.00409	MYD88	38174968
chr3	38178538	38178939	402	-0.76	0.000396	0.00409	ACAA1	38176733
chr3	38178538	38178939	402	-0.76	0.000396	0.00409	ACAA1	38176733
chr3	38178538	38178939	402	-0.76	0.000396	0.00409	MYD88	38174968
chr5	1.35E+08	1.35E+08	403	-0.79	0.000397	0.00409	VTRNA2-1	1.35E+08
chr8	1.2E+08	1.2E+08	322	-0.59	0.000397	0.00409	MAL2	1.2E+08
chr14	77786662	77787881	1220	-0.43	0.000398	0.0041	POMT2	77785225
chr14	77786662	77787881	1220	-0.43	0.000398	0.0041	GSTZ1	77782229
chr14	77786662	77787881	1220	-0.43	0.000398	0.0041	GSTZ1	77782229
chr14	77786662	77787881	1220	-0.43	0.000398	0.0041	GSTZ1	77782229
chr11	1.27E+08	1.27E+08	416	-0.64	0.000398	0.0041	LOC10192!	1.27E+08
chr1	1E+08	1E+08	956	-0.51	0.000398	0.0041	SLC35A3	1E+08
chr1	1E+08	1E+08	956	-0.51	0.000398	0.0041	SLC35A3	1E+08
chr1	1E+08	1E+08	956	-0.51	0.000398	0.0041	SLC35A3	1E+08
chr12	1.29E+08	1.29E+08	1001	-0.48	0.000398	0.0041	SLC15A4	1.29E+08
chr4	1.49E+08	1.49E+08	521	-0.74	0.000401	0.00412	PRMT9	1.49E+08
chr4	1.49E+08	1.49E+08	521	-0.74	0.000401	0.00412	PRMT9	1.49E+08
chr15	51057428	51058059	632	-0.78	0.000402	0.00413	SPPL2A	51055910
chr5	1.32E+08	1.32E+08	1012	-0.58	0.000403	0.00413	PDLIM4	1.32E+08
chr5	1.32E+08	1.32E+08	1012	-0.58	0.000403	0.00413	PDLIM4	1.32E+08
chr4	1.48E+08	1.48E+08	407	-0.68	0.000403	0.00414	TTC29	1.48E+08
chr4	1.48E+08	1.48E+08	407	-0.68	0.000403	0.00414	TTC29	1.48E+08
chr4	1.48E+08	1.48E+08	407	-0.68	0.000403	0.00414	TTC29	1.48E+08
chr4	1.48E+08	1.48E+08	407	-0.68	0.000403	0.00414	TTC29	1.48E+08
chr9	1.08E+08	1.08E+08	1210	-0.52	0.000405	0.00415	TMEM38B	1.08E+08
chr2	20792901	20793557	657	-0.72	0.000405	0.00416	HS1BP3-IT1	20790308
chr11	31531062	31531704	643	-0.55	0.000406	0.00416	IMMP1L	31529175
chr11	31531062	31531704	643	-0.55	0.000406	0.00416	IMMP1L	31529175
chr11	31531062	31531704	643	-0.55	0.000406	0.00416	ELP4	31526275
chr11	31531062	31531704	643	-0.55	0.000406	0.00416	ELP4	31526275
chr11	31531062	31531704	643	-0.55	0.000406	0.00416	ELP4	31526275
chr5	1.46E+08	1.46E+08	739	-0.59	0.000406	0.00416	TCERG1	1.46E+08
chr5	1.46E+08	1.46E+08	739	-0.59	0.000406	0.00416	TCERG1	1.46E+08
chr15	76605313	76605486	174	-1.55	0.000407	0.00417	ETFA	76601810
chr15	76605313	76605486	174	-1.55	0.000407	0.00417	ETFA	76601810
chr19	14062953	14063326	374	-0.78	0.000411	0.00419	PODNL1	14062204
chr19	14062953	14063326	374	-0.78	0.000411	0.00419	PODNL1	14062204
chr19	14062953	14063326	374	-0.78	0.000411	0.00419	DCAF15	14058318
chr8	73920742	73921504	763	-0.45	0.000411	0.0042	TERF1	73916096
chr8	73920742	73921504	763	-0.45	0.000411	0.0042	TERF1	73916096
chr6	1.53E+08	1.53E+08	659	-0.58	0.000412	0.0042	MTRF1L	1.53E+08

chr6	1.53E+08	1.53E+08	659	-0.58	0.000412	0.0042	MTRF1L	1.53E+08
chr6	1.53E+08	1.53E+08	659	-0.58	0.000412	0.0042	MTRF1L	1.53E+08
chr6	1.53E+08	1.53E+08	659	-0.58	0.000412	0.0042	MTRF1L	1.53E+08
chr6	1.53E+08	1.53E+08	659	-0.58	0.000412	0.0042	MTRF1L	1.53E+08
chr6	1.53E+08	1.53E+08	659	-0.58	0.000412	0.0042	MTRF1L	1.53E+08
chr6	1.53E+08	1.53E+08	659	-0.58	0.000412	0.0042	MTRF1L	1.53E+08
chr3	1.54E+08	1.54E+08	410	-0.83	0.000412	0.0042	GPR149	1.54E+08
chr3	70048509	70049348	840	-0.73	0.000413	0.00421	LINC01212	70043894
chr3	99979534	99980207	674	-0.71	0.000414	0.00422	TBC1D23	99974660
chr3	99979534	99980207	674	-0.71	0.000414	0.00422	TBC1D23	99974660
chr14	51800688	51801119	432	-1.19	0.000414	0.00422	LINC00640	51795110
chr16	56641169	56641599	431	-0.7	0.000415	0.00422	MT2A	56637477
chr10	22291623	22292880	1258	-0.53	0.000416	0.00423	DNAJC1	22290650
chr11	64738294	64738555	262	-1.07	0.000416	0.00423	C11orf85	64737563
chr11	64738294	64738555	262	-1.07	0.000416	0.00423	C11orf85	64737563
chr11	64738294	64738555	262	-1.07	0.000416	0.00423	C11orf85	64737563
chr11	82867533	82868189	657	-0.54	0.000417	0.00423	PCF11	82863136
chr1	78469784	78470633	850	-0.93	0.000417	0.00424	DNAJB4	78465511
chr1	78469784	78470633	850	-0.93	0.000417	0.00424	DNAJB4	78465511
chr8	1.46E+08	1.46E+08	1051	-0.48	0.000419	0.00425	ZNF250	1.46E+08
chr8	1.46E+08	1.46E+08	1051	-0.48	0.000419	0.00425	ZNF250	1.46E+08
chr4	18023267	18024016	750	-0.57	0.00042	0.00426	LCORL	18021483
chr4	18023267	18024016	750	-0.57	0.00042	0.00426	LCORL	18021483
chr15	45315215	45315650	436	-0.99	0.00042	0.00426	SORD	45310301
chr15	45315215	45315650	436	-0.99	0.00042	0.00426	SORD	45310301
chr3	1E+08	1E+08	596	-0.7	0.000421	0.00426	LNP1	1E+08
chr3	1E+08	1E+08	596	-0.7	0.000421	0.00426	TOMM70A	1E+08
chr3	1.6E+08	1.6E+08	1157	-0.57	0.000421	0.00427	ARL14	1.6E+08
chr9	21335069	21335503	435	-1.02	0.000423	0.00428	KLHL9	21333429
chr2	2.09E+08	2.09E+08	1002	-0.47	0.000425	0.00429	PTH2R	2.09E+08
chr5	59063778	59064729	952	-0.6	0.000426	0.0043	PDE4D	59062438
chr5	33440549	33441455	907	-0.54	0.000427	0.00431	TARS	33435881
chr5	33440549	33441455	907	-0.54	0.000427	0.00431	TARS	33435881
chr5	33440549	33441455	907	-0.54	0.000427	0.00431	TARS	33435881
chr5	33440549	33441455	907	-0.54	0.000427	0.00431	TARS	33435881
chr5	33440549	33441455	907	-0.54	0.000427	0.00431	TARS	33435881
chr5	33440549	33441455	907	-0.54	0.000427	0.00431	TARS	33435801
chr12	31231564	31231953	390	-0.77	0.000428	0.00431	DDX11-AS1	31224781
chr4	1.55E+08	1.55E+08	737	-0.61	0.000428	0.00431	PLRG1	1.55E+08
chr4	1.55E+08	1.55E+08	737	-0.61	0.000428	0.00431	PLRG1	1.55E+08
chr11	46942350	46942553	204	-1.16	0.000429	0.00432	LRP4	46938173
chr14	21032685	21032983	299	-1.15	0.000429	0.00432	RNASE9	21027090
chr14	21032685	21032983	299	-1.15	0.000429	0.00432	RNASE9	21027090
chr14	21032685	21032983	299	-1.15	0.000429	0.00432	RNASE9	21027090
chr14	21032685	21032983	299	-1.15	0.000429	0.00432	RNASE9	21027090
chr14	21032685	21032983	299	-1.15	0.000429	0.00432	RNASE9	21027090
chr14	21032685	21032983	299	-1.15	0.000429	0.00432	RNASE9	21027090

chr14	21032685	21032983	299	-1.15	0.000429	0.00432	RNASE9	21027090
chr14	21032685	21032983	299	-1.15	0.000429	0.00432	RNASE9	21027090
chr11	77705634	77706145	512	-0.69	0.00043	0.00433	INTS4	77703717
chr4	68995496	68995889	394	-0.9	0.000431	0.00433	TMPRSS11I	68993587
chr12	45270025	45270787	763	-0.68	0.000432	0.00434	NELL2	45267444
chr12	45270025	45270787	763	-0.68	0.000432	0.00434	NELL2	45268247
chr12	45270025	45270787	763	-0.68	0.000432	0.00434	NELL2	45268633
chr12	45270025	45270787	763	-0.68	0.000432	0.00434	NELL2	45268247
chr11	8832267	8832891	625	-0.67	0.000432	0.00434	ST5	8830224
chr11	8832267	8832891	625	-0.67	0.000432	0.00434	ST5	8830882
chr9	1050145	1050507	363	-0.68	0.000434	0.00436	DMRT2	1045353
chr9	1050145	1050507	363	-0.68	0.000434	0.00436	DMRT2	1045345
chr9	1050145	1050507	363	-0.68	0.000434	0.00436	DMRT2	1045619
chr11	1.03E+08	1.03E+08	324	-0.85	0.000435	0.00437	MMP1	1.03E+08
chr11	1.03E+08	1.03E+08	324	-0.85	0.000435	0.00437	MMP1	1.03E+08
chr8	1.24E+08	1.24E+08	549	-0.53	0.000437	0.00438	TBC1D31	1.24E+08
chr8	1.24E+08	1.24E+08	549	-0.53	0.000437	0.00438	TBC1D31	1.24E+08
chr6	20211487	20211703	217	-0.96	0.000437	0.00438	MBOAT1	20210695
chr6	20211487	20211703	217	-0.96	0.000437	0.00438	MBOAT1	20210695
chrX	11777492	11777783	292	-0.77	0.000437	0.00438	MSL3	11771700
chrX	11777492	11777783	292	-0.77	0.000437	0.00438	MSL3	11772734
chrX	11777492	11777783	292	-0.77	0.000437	0.00438	MSL3	11771277
chrX	11777492	11777783	292	-0.77	0.000437	0.00438	MSL3	11771277
chrX	11777492	11777783	292	-0.77	0.000437	0.00438	MSL3	11771277
chr20	37229924	37230567	644	-0.85	0.000438	0.00439	ARHGAP40	37225576
chr9	2014776	2015431	656	-0.58	0.000439	0.00439	SMARCA2	2010218
chr9	2014776	2015431	656	-0.58	0.000439	0.00439	SMARCA2	2010218
chr9	2014776	2015431	656	-0.58	0.000439	0.00439	SMARCA2	2010218
chr8	75262530	75263034	505	-0.69	0.000439	0.00439	GDAP1	75257617
chr8	75262530	75263034	505	-0.69	0.000439	0.00439	GDAP1	75257617
chr8	75262530	75263034	505	-0.69	0.000439	0.00439	GDAP1	75257617
chr7	23636831	23637441	611	-0.77	0.00044	0.0044	CCDC126	23631997
chr19	45393725	45394510	786	-0.47	0.00044	0.0044	TOMM40	45389476
chr19	45393725	45394510	786	-0.47	0.00044	0.0044	TOMM40	45389476
chr19	45393725	45394510	786	-0.47	0.00044	0.0044	TOMM40	45389476
chr8	1.05E+08	1.05E+08	1262	-0.46	0.00044	0.0044	RIMS2	1.05E+08
chr11	65327540	65328236	697	-0.65	0.000442	0.00441	LTBP3	65323699
chr11	65327540	65328236	697	-0.65	0.000442	0.00441	LTBP3	65323699
chr11	65327540	65328236	697	-0.65	0.000442	0.00441	LTBP3	65323699
chr5	74807310	74807924	615	-0.79	0.000444	0.00442	COL4A3BP	74805454
chr5	74807310	74807924	615	-0.79	0.000444	0.00442	COL4A3BP	74805454
chr5	74807310	74807924	615	-0.79	0.000444	0.00442	POLK	74802656
chr5	74807310	74807924	615	-0.79	0.000444	0.00442	COL4A3BP	74805806
chr8	74332115	74332499	385	-0.79	0.000444	0.00442	STAU2-AS1	74327308
chr11	1.14E+08	1.14E+08	560	-0.66	0.000444	0.00442	ZW10	1.14E+08
chr11	1.14E+08	1.14E+08	560	-0.66	0.000444	0.00442	CLDN25	1.14E+08
chr15	85259090	85259945	856	-0.62	0.000446	0.00443	SEC11A	85257691

chr15	85259090	85259945	856	-0.62	0.000446	0.00443	SEC11A	85257691
chr15	85259090	85259945	856	-0.62	0.000446	0.00443	SEC11A	85257691
chr15	85259090	85259945	856	-0.62	0.000446	0.00443	SEC11A	85257691
chr15	85259090	85259945	856	-0.62	0.000446	0.00443	SEC11A	85257691
chr15	85259090	85259945	856	-0.62	0.000446	0.00443	SEC11A	85257691
chr15	85259090	85259945	856	-0.62	0.000446	0.00443	SEC11A	85257691
chr3	1.44E+08	1.44E+08	1714	-0.46	0.000446	0.00444	C3orf58	1.44E+08
chr3	1.44E+08	1.44E+08	1714	-0.46	0.000446	0.00444	C3orf58	1.44E+08
chr11	33722173	33722827	655	-0.71	0.000446	0.00444	C11orf91	33720286
chr9	1.4E+08	1.4E+08	446	-0.57	0.000447	0.00444	MAN1B1	1.4E+08
chr9	1.4E+08	1.4E+08	446	-0.57	0.000447	0.00444	MAN1B1	1.4E+08
chr9	1.4E+08	1.4E+08	446	-0.57	0.000447	0.00444	MAN1B1	1.4E+08
chr8	87526451	87527190	740	-0.56	0.000447	0.00444	CPNE3	87521655
chr8	87526451	87527190	740	-0.56	0.000447	0.00444	RMDN1	87524577
chr3	1.69E+08	1.69E+08	839	-0.61	0.00045	0.00446	MECOM	1.69E+08
chr3	1.69E+08	1.69E+08	839	-0.61	0.00045	0.00446	MECOM	1.69E+08
chr2	1.2E+08	1.2E+08	400	-0.95	0.000451	0.00447	TMEM37	1.2E+08
chr18	28622546	28623101	556	-0.5	0.000451	0.00447	DSC3	28620781
chr18	28622546	28623101	556	-0.5	0.000451	0.00447	DSC3	28620781
chr18	30050404	30051221	818	-0.42	0.000452	0.00448	GAREM1	30048447
chr18	30050404	30051221	818	-0.42	0.000452	0.00448	GAREM1	30048447
chr4	11430419	11431035	617	-0.58	0.000452	0.00448	HS3ST1	11428537
chr19	12791967	12792760	794	-0.55	0.000453	0.00449	DHPS	12790025
chr19	12791967	12792760	794	-0.55	0.000453	0.00449	DHPS	12790701
chr19	12791967	12792760	794	-0.55	0.000453	0.00449	DHPS	12790701
chr19	12791967	12792760	794	-0.55	0.000453	0.00449	DHPS	12790701
chr3	98312023	98312825	803	-0.6	0.000454	0.0045	CPOX	98310455
chr10	1.25E+08	1.25E+08	933	-0.46	0.000455	0.0045	BUB3	1.25E+08
chr10	1.25E+08	1.25E+08	933	-0.46	0.000455	0.0045	BUB3	1.25E+08
chr1	1.01E+08	1.01E+08	870	-0.47	0.000456	0.00451	SASS6	1.01E+08
chr1	1.01E+08	1.01E+08	870	-0.47	0.000456	0.00451	SASS6	1.01E+08
chr1	1.01E+08	1.01E+08	870	-0.47	0.000456	0.00451	TRMT13	1.01E+08
chr5	16467579	16468185	607	-0.85	0.000458	0.00452	ZNF622	16463894
chr11	73587621	73588222	602	-0.43	0.000459	0.00452	PAAF1	73583013
chr11	73587621	73588222	602	-0.43	0.000459	0.00452	PAAF1	73582743
chr11	73587621	73588222	602	-0.43	0.000459	0.00452	COA4	73585890
chr11	73587621	73588222	602	-0.43	0.000459	0.00452	PAAF1	73583013
chr11	73587621	73588222	602	-0.43	0.000459	0.00452	PAAF1	73583013
chr11	73587621	73588222	602	-0.43	0.000459	0.00452	PAAF1	73583013
chr21	32931219	32931937	719	-0.61	0.000461	0.00454	TIAM1	32929290
chr19	50030977	50031293	317	-0.89	0.000463	0.00455	RCN3	50025874
chr16	53086456	53087117	662	-0.54	0.000463	0.00455	CHD9	53083944
chr9	92806836	92807474	639	-0.79	0.000465	0.00456	MIR4290H(	92801781
chr3	1.5E+08	1.5E+08	788	-0.6	0.000465	0.00456	RNF13	1.5E+08
chr3	1.5E+08	1.5E+08	788	-0.6	0.000465	0.00456	RNF13	1.5E+08
chr6	24646225	24646612	388	-0.91	0.000466	0.00457	KIAA0319	24644249
chr6	24646225	24646612	388	-0.91	0.000466	0.00457	KIAA0319	24644383

chr6	24646225	24646612	388	-0.91	0.000466	0.00457	KIAA0319	24644383
chr6	24646225	24646612	388	-0.91	0.000466	0.00457	KIAA0319	24644249
chr6	24646225	24646612	388	-0.91	0.000466	0.00457	KIAA0319	24644383
chr8	67089112	67089933	822	-0.7	0.000466	0.00457	CRH	67088880
chr11	1.13E+08	1.13E+08	320	-0.98	0.000467	0.00458	MIR4301	1.13E+08
chr2	2.07E+08	2.07E+08	214	-0.76	0.000472	0.00461	NRP2	2.07E+08
chr2	2.07E+08	2.07E+08	214	-0.76	0.000472	0.00461	NRP2	2.07E+08
chr2	2.07E+08	2.07E+08	214	-0.76	0.000472	0.00461	NRP2	2.07E+08
chr2	2.07E+08	2.07E+08	214	-0.76	0.000472	0.00461	NRP2	2.07E+08
chr2	2.07E+08	2.07E+08	214	-0.76	0.000472	0.00461	NRP2	2.07E+08
chr2	2.07E+08	2.07E+08	214	-0.76	0.000472	0.00461	NRP2	2.07E+08
chr1	2.12E+08	2.12E+08	172	-1.11	0.000472	0.00461	DTL	2.12E+08
chr1	2.12E+08	2.12E+08	172	-1.11	0.000472	0.00461	DTL	2.12E+08
chr1	2.12E+08	2.12E+08	172	-1.11	0.000472	0.00461	DTL	2.12E+08
chr1	22778155	22778647	493	-1.07	0.000473	0.00462	ZBTB40	22773343
chr1	22778155	22778647	493	-1.07	0.000473	0.00462	ZBTB40	22773343
chr20	58515020	58516001	982	-0.61	0.000473	0.00462	FAM217B	58510443
chr20	58515020	58516001	982	-0.61	0.000473	0.00462	FAM217B	58510443
chr20	58515020	58516001	982	-0.61	0.000473	0.00462	PPP1R3D	58513352
chr5	67511007	67511909	903	-0.52	0.000475	0.00463	PIK3R1	67506583
chr19	12511636	12512310	675	-0.62	0.000476	0.00463	ZNF799	12510088
chr10	1.05E+08	1.05E+08	381	-0.78	0.000476	0.00463	USMG5	1.05E+08
chr10	1.05E+08	1.05E+08	381	-0.78	0.000476	0.00463	USMG5	1.05E+08
chr10	1.05E+08	1.05E+08	381	-0.78	0.000476	0.00463	PDCD11	1.05E+08
chr10	1.05E+08	1.05E+08	381	-0.78	0.000476	0.00463	MIR1307	1.05E+08
chr10	1.05E+08	1.05E+08	381	-0.78	0.000476	0.00463	USMG5	1.05E+08
chr17	55927274	55927838	565	-0.74	0.000477	0.00464	MRPS23	55925433
chr14	73392904	73393775	872	-0.47	0.000481	0.00466	DCAF4	73388039
chr14	73392904	73393775	872	-0.47	0.000481	0.00466	DCAF4	73388039
chr14	73392904	73393775	872	-0.47	0.000481	0.00466	DCAF4	73388039
chr14	73392904	73393775	872	-0.47	0.000481	0.00466	DCAF4	73388039
chr14	73392904	73393775	872	-0.47	0.000481	0.00466	DCAF4	73388039
chr12	7341531	7342476	946	-0.75	0.000481	0.00467	PEX5	7337281
chr12	7341531	7342476	946	-0.75	0.000481	0.00467	PEX5	7337957
chr12	7341531	7342476	946	-0.75	0.000481	0.00467	PEX5	7337281
chr12	7341531	7342476	946	-0.75	0.000481	0.00467	PEX5	7337281
chr12	7341531	7342476	946	-0.75	0.000481	0.00467	PEX5	7336758
chr12	7341531	7342476	946	-0.75	0.000481	0.00467	PEX5	7337440
chr1	2.37E+08	2.37E+08	482	-0.7	0.000485	0.00469	HEATR1	2.37E+08
chr11	2421383	2422045	663	-0.59	0.000486	0.0047	TSSC4	2416717
chr11	2421383	2422045	663	-0.59	0.000486	0.0047	TSSC4	2417247
chr11	2421383	2422045	663	-0.59	0.000486	0.0047	TSSC4	2416717
chr11	2421383	2422045	663	-0.59	0.000486	0.0047	TSSC4	2416717
chr11	2421383	2422045	663	-0.59	0.000486	0.0047	TSSC4	2416717
chr20	57556077	57556541	465	-0.96	0.000487	0.0047	NELFCD	57551262
chr15	65336699	65336837	139	-1.66	0.000491	0.00474	SLC51B	65332707
chr11	1.11E+08	1.11E+08	506	-0.65	0.000491	0.00474	POU2AF1	1.11E+08

chr14	31495502	31496300	799	-0.48	0.000491	0.00474	STRN3	31493607
chr14	31495502	31496300	799	-0.48	0.000491	0.00474	STRN3	31493607
chr14	31495502	31496300	799	-0.48	0.000491	0.00474	AP4S1	31489675
chr14	31495502	31496300	799	-0.48	0.000491	0.00474	AP4S1	31489675
chr14	31495502	31496300	799	-0.48	0.000491	0.00474	AP4S1	31489311
chr14	31495502	31496300	799	-0.48	0.000491	0.00474	AP4S1	31490848
chr14	31495502	31496300	799	-0.48	0.000491	0.00474	AP4S1	31489675
chr14	31495502	31496300	799	-0.48	0.000491	0.00474	AP4S1	31489675
chr17	1588007	1588360	354	-0.86	0.000491	0.00474	PRPF8	1586176
chr6	8435410	8436193	784	-0.65	0.000493	0.00475	LOC10050€	8430855
chr6	8435410	8436193	784	-0.65	0.000493	0.00475	LOC10050€	8430855
chr6	8435410	8436193	784	-0.65	0.000493	0.00475	SLC35B3	8433800
chr6	8435410	8436193	784	-0.65	0.000493	0.00475	SLC35B3	8433800
chr6	8435410	8436193	784	-0.65	0.000493	0.00475	SLC35B3	8433800
chr6	8435410	8436193	784	-0.65	0.000493	0.00475	SLC35B3	8433800
chr12	77272224	77273420	1197	-0.57	0.000494	0.00476	CSRP2	77270820
chr12	77272224	77273420	1197	-0.57	0.000494	0.00476	CSRP2	77270820
chr14	65878413	65879500	1088	-0.43	0.000496	0.00477	FUT8	65872309
chr14	65878413	65879500	1088	-0.43	0.000496	0.00477	FUT8	65872309
chr14	65878413	65879500	1088	-0.43	0.000496	0.00477	FUT8	65874447
chr14	65878413	65879500	1088	-0.43	0.000496	0.00477	FUT8	65872309
chr14	65878413	65879500	1088	-0.43	0.000496	0.00477	FUT8	65872309
chr14	65878413	65879500	1088	-0.43	0.000496	0.00477	FUT8-AS1	65877335
chr12	25151066	25151384	319	-0.89	0.000497	0.00478	C12orf77	25148373
chr2	2.04E+08	2.04E+08	767	-0.57	0.000499	0.00479	CYP20A1	2.04E+08
chr4	775497	776060	564	-0.64	5.00E-04	0.00479	LOC10012⁹	773636
chr4	775497	776060	564	-0.64	5.00E-04	0.00479	LOC10012⁹	773636
chr11	1.08E+08	1.08E+08	550	-0.65	5.00E-04	0.0048	C11orf65	1.08E+08
chr12	16034815	16035651	837	-0.68	5.00E-04	0.0048	STRAP	16030287
chr2	74684779	74685643	865	-0.49	0.000501	0.00481	WBP1	74680526
chr12	22199071	22200018	948	-0.71	0.000504	0.00482	CMAS	22194109
chr11	1.22E+08	1.22E+08	299	-1.03	0.000506	0.00483	MIRLET7A2	1.22E+08
chr4	1.13E+08	1.13E+08	1095	-0.61	0.000508	0.00485	AP1AR	1.13E+08
chr4	1.13E+08	1.13E+08	1095	-0.61	0.000508	0.00485	AP1AR	1.13E+08
chr11	8703848	8704366	519	-0.81	0.000511	0.00487	RPL27A	8698994
chr11	8703848	8704366	519	-0.81	0.000511	0.00487	SNORA3A	8700773
chr11	8703848	8704366	519	-0.81	0.000511	0.00487	SNORA3B	8701985
chr1	2.14E+08	2.14E+08	837	-0.63	0.000511	0.00487	PROX1	2.14E+08
chr1	2.14E+08	2.14E+08	837	-0.63	0.000511	0.00487	PROX1	2.14E+08
chr1	2.14E+08	2.14E+08	837	-0.63	0.000511	0.00487	PROX1-AS1	2.14E+08
chr8	1.42E+08	1.42E+08	277	-0.71	0.000512	0.00488	DENND3	1.42E+08
chr17	8279681	8280208	528	-0.74	0.000513	0.00488	KRBA2	8272858
chr17	8279681	8280208	528	-0.74	0.000513	0.00488	KRBA2	8278029
chr20	44539454	44539986	533	-0.82	0.000514	0.00489	PLTP	44539003
chr20	44539454	44539986	533	-0.82	0.000514	0.00489	PLTP	44537633
chr20	44539454	44539986	533	-0.82	0.000514	0.00489	PLTP	44539003
chr20	44539454	44539986	533	-0.82	0.000514	0.00489	PLTP	44539003

chr13	80055151	80055585	435	-0.67	0.000515	0.00489	NDFIP2	80050258
chr13	80055151	80055585	435	-0.67	0.000515	0.00489	NDFIP2	80050258
chr13	80055151	80055585	435	-0.67	0.000515	0.00489	NDFIP2-AS:	80053366
chr2	2.02E+08	2.02E+08	586	-0.64	0.000515	0.00489	ORC2	2.02E+08
chr2	2.02E+08	2.02E+08	586	-0.64	0.000515	0.00489	ORC2	2.02E+08
chr9	71790040	71790303	264	-0.66	0.000515	0.00489	TJP2	71783970
chr9	71790040	71790303	264	-0.66	0.000515	0.00489	TJP2	71783970
chr9	71790040	71790303	264	-0.66	0.000515	0.00489	TJP2	71783970
chr4	1.39E+08	1.39E+08	531	-0.61	0.000516	0.0049	LINC00499	1.39E+08
chr10	1.04E+08	1.04E+08	954	-0.56	0.000516	0.0049	NOLC1	1.04E+08
chr10	1.04E+08	1.04E+08	954	-0.56	0.000516	0.0049	NOLC1	1.04E+08
chr10	1.04E+08	1.04E+08	954	-0.56	0.000516	0.0049	NOLC1	1.04E+08
chr10	1.01E+08	1.01E+08	1229	-0.47	0.000516	0.0049	SLC25A28	1.01E+08
chr17	72868998	72869664	667	-0.66	0.000516	0.0049	FDXR	72867156
chr17	72868998	72869664	667	-0.66	0.000516	0.0049	FDXR	72867156
chr17	72868998	72869664	667	-0.66	0.000516	0.0049	FDXR	72867156
chr17	72868998	72869664	667	-0.66	0.000516	0.0049	FDXR	72867156
chr17	72868998	72869664	667	-0.66	0.000516	0.0049	FDXR	72862876
chr17	72868998	72869664	667	-0.66	0.000516	0.0049	FDXR	72867156
chr17	72868998	72869664	667	-0.66	0.000516	0.0049	FDXR	72867156
chr17	72868998	72869664	667	-0.66	0.000516	0.0049	FDXR	72867156
chr12	13349205	13349776	572	-0.65	0.000516	0.0049	EMP1	13344601
chr9	1.35E+08	1.35E+08	212	-1.44	0.000518	0.00491	MED27	1.35E+08
chr9	1.35E+08	1.35E+08	212	-1.44	0.000518	0.00491	MED27	1.35E+08
chr9	1.35E+08	1.35E+08	212	-1.44	0.000518	0.00491	MED27	1.35E+08
chr1	4710534	4710921	388	-0.67	0.000519	0.00492	AJAP1	4710104
chr1	4710534	4710921	388	-0.67	0.000519	0.00492	AJAP1	4710104
chr4	1.04E+08	1.04E+08	465	-0.68	0.00052	0.00492	SLC9B1	1.04E+08
chr4	1.04E+08	1.04E+08	465	-0.68	0.00052	0.00492	SLC9B1	1.04E+08
chr4	1.04E+08	1.04E+08	465	-0.68	0.00052	0.00492	SLC9B1	1.04E+08
chr4	1.04E+08	1.04E+08	465	-0.68	0.00052	0.00492	SLC9B1	1.04E+08
chr11	1.17E+08	1.17E+08	147	-1.79	0.000521	0.00492	APOA5	1.17E+08
chr11	1.17E+08	1.17E+08	147	-1.79	0.000521	0.00492	APOA5	1.17E+08
chr14	50100328	50102023	1696	-0.45	0.000521	0.00493	DNAAF2	50099948
chr14	50100328	50102023	1696	-0.45	0.000521	0.00493	DNAAF2	50099948
chr19	45681469	45682251	783	-0.63	0.000522	0.00493	TRAPPC6A	45679501
chr19	45681469	45682251	783	-0.63	0.000522	0.00493	TRAPPC6A	45679501
chr19	45681469	45682251	783	-0.63	0.000522	0.00493	BLOC1S3	45677002
chr19	45681469	45682251	783	-0.63	0.000522	0.00493	TRAPPC6A	45679501
chr19	45681469	45682251	783	-0.63	0.000522	0.00493	TRAPPC6A	45679501
chr17	43138289	43138989	701	-0.7	0.000522	0.00493	DCAKD	43136477
chr17	43138289	43138989	701	-0.7	0.000522	0.00493	DCAKD	43136477
chr17	43138289	43138989	701	-0.7	0.000522	0.00493	NMT1	43133666
chr12	14518301	14519008	708	-0.53	0.000522	0.00493	ATF7IP	14513565
chr12	14518301	14519008	708	-0.53	0.000522	0.00493	ATF7IP	14513565
chr12	14518301	14519008	708	-0.53	0.000522	0.00493	ATF7IP	14513565
chr6	1.34E+08	1.34E+08	279	-1.05	0.000523	0.00493	SGK1	1.34E+08

chr6	1.34E+08	1.34E+08	279	-1.05	0.000523	0.00493	SGK1	1.34E+08
chr6	1.34E+08	1.34E+08	279	-1.05	0.000523	0.00493	SGK1	1.34E+08
chr6	1.34E+08	1.34E+08	279	-1.05	0.000523	0.00493	SGK1	1.34E+08
chr1	2.02E+08	2.02E+08	395	-1	0.000524	0.00494	MIR5191	2.02E+08
chr16	68279068	68279403	336	-1	0.000524	0.00494	PLA2G15	68274246
chr2	2.09E+08	2.09E+08	505	-0.68	0.000524	0.00494	PIKFYVE	2.09E+08
chr2	2.09E+08	2.09E+08	505	-0.68	0.000524	0.00494	PIKFYVE	2.09E+08
chr2	2.09E+08	2.09E+08	505	-0.68	0.000524	0.00494	PIKFYVE	2.09E+08
chr1	1.1E+08	1.1E+08	256	-0.86	0.000524	0.00494	AMPD2	1.1E+08
chr1	1.1E+08	1.1E+08	256	-0.86	0.000524	0.00494	AMPD2	1.1E+08
chr1	1.1E+08	1.1E+08	256	-0.86	0.000524	0.00494	AMPD2	1.1E+08
chr1	1.1E+08	1.1E+08	256	-0.86	0.000524	0.00494	GNAT2	1.1E+08
chr1	1.1E+08	1.1E+08	256	-0.86	0.000524	0.00494	AMPD2	1.1E+08
chr10	60144823	60145742	920	-0.62	0.000524	0.00494	TFAM	60139902
chr10	60144823	60145742	920	-0.62	0.000524	0.00494	TFAM	60139902
chr10	60144823	60145742	920	-0.62	0.000524	0.00494	TFAM	60139902
chr5	1.26E+08	1.26E+08	716	-0.63	0.000526	0.00495	3-Mar	1.26E+08
chr1	3816665	3818098	1434	-0.51	0.000526	0.00496	LINC01134	3811967
chr1	3816665	3818098	1434	-0.51	0.000526	0.00496	C1orf174	3814857
chr13	1.03E+08	1.03E+08	500	-0.59	0.000527	0.00496	BIVM	1.03E+08
chr13	1.03E+08	1.03E+08	500	-0.59	0.000527	0.00496	BIVM	1.03E+08
chr13	1.03E+08	1.03E+08	500	-0.59	0.000527	0.00496	KDELC1	1.03E+08
chr13	1.03E+08	1.03E+08	500	-0.59	0.000527	0.00496	KDELC1	1.03E+08
chr1	6844608	6845515	908	-0.43	0.000527	0.00496	CAMTA1	6840383
chr1	6844608	6845515	908	-0.43	0.000527	0.00496	CAMTA1	6840383
chr1	6844608	6845515	908	-0.43	0.000527	0.00496	CAMTA1	6840383
chr1	6844608	6845515	908	-0.43	0.000527	0.00496	CAMTA1	6840383
chr10	77054631	77055171	541	-0.71	0.000529	0.00498	ZNF503-AS	77051140
chr10	77054631	77055171	541	-0.71	0.000529	0.00498	ZNF503-AS	77051140
chr10	77054631	77055171	541	-0.71	0.000529	0.00498	ZNF503-AS	77051140
chr10	77054631	77055171	541	-0.71	0.000529	0.00498	ZNF503-AS	77051140
chr1	1.61E+08	1.61E+08	382	-0.71	0.00053	0.00498	TOMM40L	1.61E+08
chr1	1.61E+08	1.61E+08	382	-0.71	0.00053	0.00498	MIR5187	1.61E+08
chr1	1.61E+08	1.61E+08	382	-0.71	0.00053	0.00498	TOMM40L	1.61E+08
chr1	1.61E+08	1.61E+08	382	-0.71	0.00053	0.00498	APOA2	1.61E+08
chr1	1.61E+08	1.61E+08	382	-0.71	0.00053	0.00498	TOMM40L	1.61E+08
chr15	52860987	52861490	504	-0.74	0.000531	0.00498	ARPP19	52859329
chr15	52860987	52861490	504	-0.74	0.000531	0.00498	ARPP19	52859329
chr15	52860987	52861490	504	-0.74	0.000531	0.00498	ARPP19	52859418
chr15	52860987	52861490	504	-0.74	0.000531	0.00498	ARPP19	52859643
chr12	83079517	83081075	1559	-0.46	0.000531	0.00499	TMTC2	83075933
chr11	59436438	59437178	741	-0.54	0.000532	0.005	PATL1	59434511
chr2	3130491	3130801	311	-1.53	0.000535	0.00501	LINC01250	3127798
chr1	85098738	85098895	158	-1.47	0.000535	0.00502	LINC01555	85098703
chr10	77188248	77188555	308	-0.76	0.000536	0.00502	C10orf11	77186403
chr14	21944719	21945682	964	-0.5	0.000536	0.00502	RAB2B	21942832
chr14	21944719	21945682	964	-0.5	0.000536	0.00502	TOX4	21940334

chr14	21944719	21945682	964	-0.5	0.000536	0.00502	RAB2B	21943132
chr14	21944719	21945682	964	-0.5	0.000536	0.00502	TOX4	21940334
chr14	21944719	21945682	964	-0.5	0.000536	0.00502	RAB2B	21943132
chr8	1.18E+08	1.18E+08	852	-0.48	0.000538	0.00503	RAD21	1.18E+08
chr8	1.18E+08	1.18E+08	852	-0.48	0.000538	0.00503	MIR3610	1.18E+08
chr8	1.18E+08	1.18E+08	852	-0.48	0.000538	0.00503	RAD21-AS1	1.18E+08
chr20	47804718	47805192	475	-0.92	0.000539	0.00504	STAU1	47802904
chr20	47804718	47805192	475	-0.92	0.000539	0.00504	STAU1	47802904
chr20	47804718	47805192	475	-0.92	0.000539	0.00504	STAU1	47802904
chr20	47804718	47805192	475	-0.92	0.000539	0.00504	STAU1	47802904
chr20	47804718	47805192	475	-0.92	0.000539	0.00504	STAU1	47802904
chr11	32109628	32110278	651	-0.56	0.000539	0.00504	RCN1	32107476
chr11	70245586	70246164	579	-0.46	0.00054	0.00504	CTTN	70239611
chr11	70245586	70246164	579	-0.46	0.00054	0.00504	CTTN	70239611
chr11	70245586	70246164	579	-0.46	0.00054	0.00504	CTTN	70239611
chr7	1.34E+08	1.34E+08	371	-0.58	0.00054	0.00504	BPGM	1.34E+08
chr7	1.34E+08	1.34E+08	371	-0.58	0.00054	0.00504	BPGM	1.34E+08
chr7	1.34E+08	1.34E+08	371	-0.58	0.00054	0.00504	BPGM	1.34E+08
chr11	94226648	94227355	708	-0.71	0.000541	0.00505	ANKRD49	94222152
chr11	94226648	94227355	708	-0.71	0.000541	0.00505	MRE11A	94225040
chr11	94226648	94227355	708	-0.71	0.000541	0.00505	MRE11A	94225040
chr2	55459262	55459814	553	-0.77	0.000542	0.00506	CLHC1	55457699
chr2	55459262	55459814	553	-0.77	0.000542	0.00506	CLHC1	55457449
chr2	55459262	55459814	553	-0.77	0.000542	0.00506	RPS27A	55454813
chr2	55459262	55459814	553	-0.77	0.000542	0.00506	MIR4426	55456299
chr2	55459262	55459814	553	-0.77	0.000542	0.00506	RPS27A	55454540
chr2	55459262	55459814	553	-0.77	0.000542	0.00506	RPS27A	55454038
chr1	59616667	59617085	419	-0.68	0.000543	0.00506	HSD52	59610479
chr12	1.07E+08	1.07E+08	533	-0.61	0.000543	0.00507	CRY1	1.07E+08
chr11	62607773	62609299	1527	-0.49	0.000543	0.00507	WDR74	62605131
chr11	62607773	62609299	1527	-0.49	0.000543	0.00507	WDR74	62605628
chr12	72234240	72234588	349	-0.94	0.000544	0.00507	TBC1D15	72228486
chr12	72234240	72234588	349	-0.94	0.000544	0.00507	TBC1D15	72228486
chr12	72234240	72234588	349	-0.94	0.000544	0.00507	TBC1D15	72228486
chr12	72234240	72234588	349	-0.94	0.000544	0.00507	TBC1D15	72228486
chr14	20881187	20881770	584	-0.62	0.000544	0.00507	TEP1	20879579
chr14	20881187	20881770	584	-0.62	0.000544	0.00507	TEP1	20879579
chr1	36914377	36914608	232	-1.02	0.000547	0.00509	OSCP1	36914086
chr1	36914377	36914608	232	-1.02	0.000547	0.00509	OSCP1	36914086
chr20	17662812	17663388	577	-0.47	0.000547	0.00509	RRBP1	17660928
chr20	17662812	17663388	577	-0.47	0.000547	0.00509	RRBP1	17660928
chr5	42564841	42565292	452	-0.78	0.000552	0.00512	GHR	42560965
chr5	42564841	42565292	452	-0.78	0.000552	0.00512	GHR	42560965
chr5	42564841	42565292	452	-0.78	0.000552	0.00512	GHR	42560562
chr1	1.61E+08	1.61E+08	256	-0.99	0.000552	0.00513	F11R	1.61E+08
chr8	97247583	97248026	444	-0.71	0.000552	0.00513	UQCRB	97245862
chr8	97247583	97248026	444	-0.71	0.000552	0.00513	UQCRB	97245862

chr8	97247583	97248026	444	-0.71	0.000552	0.00513	UQCRB	97245862
chr8	97247583	97248026	444	-0.71	0.000552	0.00513	UQCRB	97245862
chr1	92950204	92951693	1490	-0.46	0.000554	0.00514	GFI1	92949628
chr1	92950204	92951693	1490	-0.46	0.000554	0.00514	GFI1	92947356
chr1	92950204	92951693	1490	-0.46	0.000554	0.00514	GFI1	92950433
chr14	54423375	54424001	627	-0.51	0.000554	0.00514	BMP4	54421580
chr14	54423375	54424001	627	-0.51	0.000554	0.00514	BMP4	54421580
chr14	81894138	81894286	149	-1.38	0.000555	0.00515	STON2	81891556
chr14	81894138	81894286	149	-1.38	0.000555	0.00515	STON2	81891748
chr3	1.22E+08	1.22E+08	367	-1.1	0.000558	0.00516	PARP15	1.22E+08
chr3	1.22E+08	1.22E+08	367	-1.1	0.000558	0.00516	PARP15	1.22E+08
chr2	1.87E+08	1.87E+08	617	-0.54	0.000559	0.00517	ITGAV	1.87E+08
chr2	1.87E+08	1.87E+08	617	-0.54	0.000559	0.00517	ITGAV	1.87E+08
chr2	1.52E+08	1.52E+08	567	-1.04	0.000559	0.00517	NMI	1.52E+08
chr16	58495100	58495274	175	-1.12	0.00056	0.00517	NDRG4	58492993
chr16	58495100	58495274	175	-1.12	0.00056	0.00517	NDRG4	58492548
chr16	58495100	58495274	175	-1.12	0.00056	0.00517	NDRG4	58493725
chr5	43483543	43484703	1161	-0.48	0.000561	0.00518	C5orf28	43481992
chr3	1.2E+08	1.2E+08	1421	-0.47	0.000561	0.00518	GSK3B	1.2E+08
chr3	1.2E+08	1.2E+08	1421	-0.47	0.000561	0.00518	GSK3B	1.2E+08
chr7	54614324	54614765	442	-0.73	0.000562	0.00518	VSTM2A	54608856
chr1	1.04E+08	1.04E+08	238	-1.11	0.000563	0.00519	COL11A1	1.04E+08
chr1	1.04E+08	1.04E+08	238	-1.11	0.000563	0.00519	COL11A1	1.04E+08
chr1	1.04E+08	1.04E+08	238	-1.11	0.000563	0.00519	COL11A1	1.04E+08
chr1	1.04E+08	1.04E+08	238	-1.11	0.000563	0.00519	COL11A1	1.04E+08
chr3	1.83E+08	1.83E+08	453	-0.55	0.000564	0.0052	MCCC1	1.83E+08
chr3	1.83E+08	1.83E+08	453	-0.55	0.000564	0.0052	MCCC1	1.83E+08
chr3	1.83E+08	1.83E+08	453	-0.55	0.000564	0.0052	MCCC1	1.83E+08
chr3	1.83E+08	1.83E+08	453	-0.55	0.000564	0.0052	MCCC1	1.83E+08
chr22	41777413	41777940	528	-0.76	0.000567	0.00522	TEF	41772932
chr6	19839259	19839498	240	-0.8	0.000567	0.00522	ID4	19832600
chr2	85581564	85582069	506	-0.75	0.000568	0.00522	ELMOD3	85576842
chr2	85581564	85582069	506	-0.75	0.000568	0.00522	ELMOD3	85576842
chr2	85581564	85582069	506	-0.75	0.000568	0.00522	ELMOD3	85576842
chr2	85581564	85582069	506	-0.75	0.000568	0.00522	ELMOD3	85576842
chr2	85581564	85582069	506	-0.75	0.000568	0.00522	RETSAT	85579821
chr14	59655042	59655789	748	-0.47	0.000568	0.00522	DAAM1	59650380
chr11	72145239	72145814	576	-0.5	0.000571	0.00524	CLPB	72143728
chr11	72145239	72145814	576	-0.5	0.000571	0.00524	CLPB	72143728
chr11	72145239	72145814	576	-0.5	0.000571	0.00524	CLPB	72143728
chr11	72145239	72145814	576	-0.5	0.000571	0.00524	CLPB	72143728
chr3	32611640	32612666	1027	-0.78	0.000572	0.00524	DYNC1LI1	32610366
chr2	1.07E+08	1.07E+08	324	-1.33	0.000572	0.00525	UXS1	1.07E+08
chr14	50090678	50091086	409	-0.64	0.000574	0.00526	RPL36AL	50085403
chr15	86304858	86305228	371	-0.94	0.000574	0.00526	LOC10192[illegible]	86299956
chr4	76598583	76598889	307	-0.94	0.000577	0.00527	G3BP2	76596343
chr4	76598583	76598889	307	-0.94	0.000577	0.00527	G3BP2	76596667

chr4	76598583	76598889	307	-0.94	0.000577	0.00527	G3BP2	76596667
chr12	33049716	33050249	534	-0.59	0.00058	0.00529	PKP2	33047780
chr12	33049716	33050249	534	-0.59	0.00058	0.00529	PKP2	33047780
chr2	1.7E+08	1.7E+08	328	-0.96	0.000581	0.00529	FASTKD1	1.7E+08
chr2	1.7E+08	1.7E+08	328	-0.96	0.000581	0.00529	FASTKD1	1.7E+08
chr2	1.7E+08	1.7E+08	328	-0.96	0.000581	0.00529	FASTKD1	1.7E+08
chr3	1.6E+08	1.6E+08	697	-0.51	0.000581	0.00529	TRIM59	1.6E+08
chr5	1.69E+08	1.69E+08	524	-0.8	0.000582	0.0053	SLIT3	1.69E+08
chr5	1.69E+08	1.69E+08	524	-0.8	0.000582	0.0053	SLIT3	1.69E+08
chr8	1.45E+08	1.45E+08	1251	-0.47	0.000582	0.0053	MAF1	1.45E+08
chr8	1.45E+08	1.45E+08	1251	-0.47	0.000582	0.0053	SHARPIN	1.45E+08
chr8	1.45E+08	1.45E+08	1251	-0.47	0.000582	0.0053	SHARPIN	1.45E+08
chr8	1.45E+08	1.45E+08	1251	-0.47	0.000582	0.0053	WDR97	1.45E+08
chr1	1.6E+08	1.6E+08	820	-0.62	0.000584	0.00531	TAGLN2	1.6E+08
chr1	1.6E+08	1.6E+08	820	-0.62	0.000584	0.00531	TAGLN2	1.6E+08
chr1	1.6E+08	1.6E+08	820	-0.62	0.000584	0.00531	TAGLN2	1.6E+08
chr8	62626750	62627548	799	-0.46	0.000585	0.00531	ASPH	62625199
chr8	62626750	62627548	799	-0.46	0.000585	0.00531	ASPH	62625199
chr8	62626750	62627548	799	-0.46	0.000585	0.00531	ASPH	62625199
chr8	62626750	62627548	799	-0.46	0.000585	0.00531	ASPH	62625199
chr8	62626750	62627548	799	-0.46	0.000585	0.00531	ASPH	62625199
chr8	62626750	62627548	799	-0.46	0.000585	0.00531	MIR4470	62622346
chr4	1.02E+08	1.02E+08	886	-0.53	0.000587	0.00532	PPP3CA	1.02E+08
chr4	1.02E+08	1.02E+08	886	-0.53	0.000587	0.00532	PPP3CA	1.02E+08
chr4	1.02E+08	1.02E+08	886	-0.53	0.000587	0.00532	PPP3CA	1.02E+08
chr4	1.02E+08	1.02E+08	886	-0.53	0.000587	0.00532	FLJ20021	1.02E+08
chr14	45603315	45603990	676	-0.52	0.000588	0.00533	FANCM	45600141
chr14	45603315	45603990	676	-0.52	0.000588	0.00533	FANCM	45600141
chr14	45603315	45603990	676	-0.52	0.000588	0.00533	FANCM	45600141
chr14	45603315	45603990	676	-0.52	0.000588	0.00533	FKBP3	45601732
chr1	1.61E+08	1.61E+08	646	-0.65	0.000589	0.00533	PPOX	1.61E+08
chr1	1.61E+08	1.61E+08	646	-0.65	0.000589	0.00533	PPOX	1.61E+08
chr6	1.54E+08	1.54E+08	218	-1.84	0.00059	0.00534	OPRM1	1.54E+08
chr6	1.54E+08	1.54E+08	218	-1.84	0.00059	0.00534	OPRM1	1.54E+08
chr6	1.54E+08	1.54E+08	218	-1.84	0.00059	0.00534	OPRM1	1.54E+08
chr6	1.54E+08	1.54E+08	218	-1.84	0.00059	0.00534	OPRM1	1.54E+08
chr4	1.23E+08	1.23E+08	727	-0.58	0.000591	0.00536	BBS7	1.23E+08
chr4	1.23E+08	1.23E+08	727	-0.58	0.000591	0.00536	BBS7	1.23E+08
chr8	37887777	37889104	1328	-0.5	0.000595	0.00538	EIF4EBP1	37883019
chr14	24766033	24766246	214	-1.12	6.00E-04	0.00541	NOP9	24764059
chr14	24766033	24766246	214	-1.12	6.00E-04	0.00541	NOP9	24764059
chr1	89591458	89591971	514	-0.63	0.000602	0.00542	GBP2	89589842
chr1	62901930	62902628	699	-0.6	0.000604	0.00544	USP1	62896974
chr1	62901930	62902628	699	-0.6	0.000604	0.00544	USP1	62897382
chr1	62901930	62902628	699	-0.6	0.000604	0.00544	USP1	62896974
chr10	1.06E+08	1.06E+08	512	-0.64	0.000605	0.00544	CFAP43	1.06E+08
chr1	1.54E+08	1.54E+08	819	-0.64	0.000607	0.00545	ILF2	1.54E+08

chr1	1.54E+08	1.54E+08	819	-0.64	0.000607	0.00545	ILF2	1.54E+08
chr18	25757002	25758102	1101	-0.67	0.000607	0.00545	CDH2	25755410
chr6	64345434	64346235	802	-0.58	0.000607	0.00545	PHF3	64341385
chr6	64345434	64346235	802	-0.58	0.000607	0.00545	PHF3	64340706
chr10	24498217	24498574	358	-1.08	0.00061	0.00547	KIAA1217	24492719
chr10	24498217	24498574	358	-1.08	0.00061	0.00547	KIAA1217	24492719
chr10	24498217	24498574	358	-1.08	0.00061	0.00547	KIAA1217	24492719
chr1	47903090	47903838	749	-0.53	0.00061	0.00547	FOXD2-AS1	47898313
chr1	47903090	47903838	749	-0.53	0.00061	0.00547	LINC01389	47900989
chr1	47903090	47903838	749	-0.53	0.00061	0.00547	FOXD2	47896688
chrX	1.03E+08	1.03E+08	811	-0.54	0.000611	0.00548	MORF4L2	1.03E+08
chrX	1.03E+08	1.03E+08	811	-0.54	0.000611	0.00548	MORF4L2	1.03E+08
chrX	1.03E+08	1.03E+08	811	-0.54	0.000611	0.00548	MORF4L2	1.03E+08
chrX	1.03E+08	1.03E+08	811	-0.54	0.000611	0.00548	MORF4L2	1.03E+08
chrX	1.03E+08	1.03E+08	811	-0.54	0.000611	0.00548	MORF4L2	1.03E+08
chrX	1.03E+08	1.03E+08	811	-0.54	0.000611	0.00548	MORF4L2	1.03E+08
chrX	1.03E+08	1.03E+08	811	-0.54	0.000611	0.00548	MORF4L2	1.03E+08
chrX	1.03E+08	1.03E+08	811	-0.54	0.000611	0.00548	MORF4L2	1.03E+08
chrX	1.03E+08	1.03E+08	811	-0.54	0.000611	0.00548	MORF4L2	1.03E+08
chrX	1.03E+08	1.03E+08	811	-0.54	0.000611	0.00548	MORF4L2	1.03E+08
chrX	1.03E+08	1.03E+08	811	-0.54	0.000611	0.00548	MORF4L2	1.03E+08
chrX	1.03E+08	1.03E+08	811	-0.54	0.000611	0.00548	MORF4L2	1.03E+08
chrX	1.03E+08	1.03E+08	811	-0.54	0.000611	0.00548	MORF4L2	1.03E+08
chrX	1.03E+08	1.03E+08	811	-0.54	0.000611	0.00548	MORF4L2	1.03E+08
chrX	1.03E+08	1.03E+08	811	-0.54	0.000611	0.00548	MORF4L2	1.03E+08
chrX	1.03E+08	1.03E+08	811	-0.54	0.000611	0.00548	MORF4L2	1.03E+08
chrX	1.03E+08	1.03E+08	811	-0.54	0.000611	0.00548	MORF4L2-/	1.03E+08
chr5	82373064	82373725	662	-0.61	0.000612	0.00549	XRCC4	82368227
chr5	82373064	82373725	662	-0.61	0.000612	0.00549	XRCC4	82368227
chr5	82373064	82373725	662	-0.61	0.000612	0.00549	XRCC4	82368227
chr5	82373064	82373725	662	-0.61	0.000612	0.00549	XRCC4	82368227
chr5	82373064	82373725	662	-0.61	0.000612	0.00549	XRCC4	82368227
chr5	82373064	82373725	662	-0.61	0.000612	0.00549	TMEM167/	82371272
chr17	7117656	7118528	873	-0.63	0.000612	0.00549	ACADVL	7118149
chr17	7117656	7118528	873	-0.63	0.000612	0.00549	ACADVL	7118149
chr17	7117656	7118528	873	-0.63	0.000612	0.00549	ACADVL	7118149
chr17	7117656	7118528	873	-0.63	0.000612	0.00549	ACADVL	7115443
chr1	1.2E+08	1.2E+08	733	-0.53	0.000615	0.0055	WARS2	1.2E+08
chr1	1.2E+08	1.2E+08	733	-0.53	0.000615	0.0055	WARS2	1.2E+08
chr1	1.2E+08	1.2E+08	733	-0.53	0.000615	0.0055	LOC10192S	1.2E+08
chr1	1.2E+08	1.2E+08	733	-0.53	0.000615	0.0055	LOC10192S	1.2E+08
chr1	1.2E+08	1.2E+08	733	-0.53	0.000615	0.0055	LOC10192S	1.2E+08
chr1	1.2E+08	1.2E+08	733	-0.53	0.000615	0.0055	LOC10192S	1.2E+08
chr16	1132622	1133328	707	-0.76	0.000616	0.00551	SSTR5-AS1	1126731
chr7	1.12E+08	1.12E+08	369	-1.01	0.000616	0.00551	LSMEM1	1.12E+08
chr7	1.12E+08	1.12E+08	369	-1.01	0.000616	0.00551	LSMEM1	1.12E+08
chr15	91475025	91475286	262	-0.99	0.000617	0.00551	UNC45A	91473214

chr15	91475025	91475286	262	-0.99	0.000617	0.00551	UNC45A	91468409
chr15	91475025	91475286	262	-0.99	0.000617	0.00551	HDDC3	91473799
chr15	91475025	91475286	262	-0.99	0.000617	0.00551	HDDC3	91473799
chr15	91475025	91475286	262	-0.99	0.000617	0.00551	HDDC3	91473799
chr2	7057284	7057949	666	-0.77	0.000617	0.00551	RNF144A	7052522
chr2	7057284	7057949	666	-0.77	0.000617	0.00551	RNF144A-A	7056813
chr5	40842547	40842760	214	-1.22	0.000617	0.00551	CARD6	40836409
chr6	26537541	26538103	563	-0.57	0.000619	0.00552	HMGN4	26533571
chr8	1.09E+08	1.09E+08	815	-0.46	0.000621	0.00553	EMC2	1.09E+08
chr12	62653610	62654955	1346	-0.46	0.000622	0.00554	USP15	62649120
chr12	62653610	62654955	1346	-0.46	0.000622	0.00554	USP15	62649120
chr12	62653610	62654955	1346	-0.46	0.000622	0.00554	MIR6125	62649139
chr12	62653610	62654955	1346	-0.46	0.000622	0.00554	USP15	62649120
chr15	48623208	48624274	1067	-0.58	0.000625	0.00556	DUT	48618620
chr15	48623208	48624274	1067	-0.58	0.000625	0.00556	DUT	48618620
chr15	48623208	48624274	1067	-0.58	0.000625	0.00556	DUT	48619364
chr3	4533842	4535200	1359	-0.53	0.000625	0.00556	ITPR1	4530031
chr3	4533842	4535200	1359	-0.53	0.000625	0.00556	ITPR1	4530031
chr3	4533842	4535200	1359	-0.53	0.000625	0.00556	ITPR1	4530031
chr3	4533842	4535200	1359	-0.53	0.000625	0.00556	ITPR1-AS1	4532847
chr3	1.58E+08	1.58E+08	300	-0.65	0.000625	0.00556	MLF1	1.58E+08
chr3	1.58E+08	1.58E+08	300	-0.65	0.000625	0.00556	MLF1	1.58E+08
chr3	1.58E+08	1.58E+08	300	-0.65	0.000625	0.00556	MLF1	1.58E+08
chr3	1.58E+08	1.58E+08	300	-0.65	0.000625	0.00556	MLF1	1.58E+08
chr3	1.58E+08	1.58E+08	300	-0.65	0.000625	0.00556	MLF1	1.58E+08
chr3	1.58E+08	1.58E+08	300	-0.65	0.000625	0.00556	LOC10099€	1.58E+08
chr3	1.58E+08	1.58E+08	300	-0.65	0.000625	0.00556	MLF1	1.58E+08
chr15	84115790	84116235	446	-0.87	0.000626	0.00556	SH3GL3	84110979
chr15	84115790	84116235	446	-0.87	0.000626	0.00556	SH3GL3	84110979
chr15	84115790	84116235	446	-0.87	0.000626	0.00556	SH3GL3	84110979
chr15	84115790	84116235	446	-0.87	0.000626	0.00556	SH3GL3	84110979
chr17	7475758	7476173	416	-0.64	0.000626	0.00556	EIF4A1	7471023
chr17	7475758	7476173	416	-0.64	0.000626	0.00556	SNORA48	7473030
chr17	7475758	7476173	416	-0.64	0.000626	0.00556	EIF4A1	7471023
chr17	7475758	7476173	416	-0.64	0.000626	0.00556	SNORD10	7475128
chr10	1.12E+08	1.12E+08	444	-0.59	0.000627	0.00557	MXI1	1.12E+08
chr7	1.49E+08	1.49E+08	370	-0.82	0.000627	0.00557	LOC15506C	1.49E+08
chr5	1.73E+08	1.73E+08	370	-0.7	0.000629	0.00558	BOD1	1.73E+08
chr5	1.73E+08	1.73E+08	370	-0.7	0.000629	0.00558	BOD1	1.73E+08
chr10	49462044	49462433	390	-0.73	0.000631	0.00559	FRMPD2	49457800
chr3	42003505	42004043	539	-0.58	0.000631	0.00559	ULK4	42001660
chr1	85742025	85742663	639	-0.52	0.000632	0.0056	BCL10	85740587
chr1	85742025	85742663	639	-0.52	0.000632	0.0056	LOC64662€	85737040
chr5	61601689	61602171	483	-0.62	0.000633	0.0056	KIF2A	61596988
chr5	61601689	61602171	483	-0.62	0.000633	0.0056	KIF2A	61596988
chr5	61601689	61602171	483	-0.62	0.000633	0.0056	KIF2A	61596988
chr5	61601689	61602171	483	-0.62	0.000633	0.0056	KIF2A	61596988

chr3	57094421	57094834	414	-0.87	0.000635	0.00561	SPATA12	57089468
chr4	54933455	54933698	244	-1.01	0.000639	0.00563	CHIC2	54928815
chr1	1.18E+08	1.18E+08	992	-0.51	0.000639	0.00564	WDR3	1.18E+08
chr1	1.18E+08	1.18E+08	992	-0.51	0.000639	0.00564	GDAP2	1.18E+08
chr1	1.18E+08	1.18E+08	992	-0.51	0.000639	0.00564	GDAP2	1.18E+08
chr9	1.08E+08	1.08E+08	910	-0.52	0.00064	0.00564	NIPSNAP3A	1.08E+08
chr6	1.18E+08	1.18E+08	588	-0.54	0.000642	0.00566	NUS1	1.18E+08
chr6	1.18E+08	1.18E+08	588	-0.54	0.000642	0.00566	LOC101927	1.18E+08
chr2	2.11E+08	2.11E+08	577	-0.65	0.000643	0.00566	CPS1	2.11E+08
chr2	2.11E+08	2.11E+08	577	-0.65	0.000643	0.00566	LANCL1	2.11E+08
chr2	2.11E+08	2.11E+08	577	-0.65	0.000643	0.00566	LANCL1	2.11E+08
chr2	2.11E+08	2.11E+08	577	-0.65	0.000643	0.00566	LANCL1	2.11E+08
chr5	1.12E+08	1.12E+08	662	-0.54	0.000644	0.00567	APC	1.12E+08
chr9	99620287	99620629	343	-0.66	0.000645	0.00567	ZNF782	99614389
chr1	1.68E+08	1.68E+08	778	-0.51	0.000647	0.00569	DCAF6	1.68E+08
chr1	1.68E+08	1.68E+08	778	-0.51	0.000647	0.00569	MPC2	1.68E+08
chr1	1.68E+08	1.68E+08	778	-0.51	0.000647	0.00569	DCAF6	1.68E+08
chr1	1.68E+08	1.68E+08	778	-0.51	0.000647	0.00569	DCAF6	1.68E+08
chr1	1.68E+08	1.68E+08	778	-0.51	0.000647	0.00569	MPC2	1.68E+08
chr1	1.68E+08	1.68E+08	778	-0.51	0.000647	0.00569	MPC2	1.68E+08
chr1	1.68E+08	1.68E+08	778	-0.51	0.000647	0.00569	DCAF6	1.68E+08
chr14	24615866	24616801	936	-0.44	0.000648	0.00569	PSME2	24613855
chr14	24615866	24616801	936	-0.44	0.000648	0.00569	RNF31	24611658
chr14	24615866	24616801	936	-0.44	0.000648	0.00569	RNF31	24611083
chr14	24615866	24616801	936	-0.44	0.000648	0.00569	MIR7703	24610774
chr10	25013264	25013897	634	-0.58	0.000648	0.00569	ARHGAP21	25010597
chr2	1.98E+08	1.98E+08	644	-0.58	0.000652	0.00572	PGAP1	1.98E+08
chr11	1.25E+08	1.25E+08	823	-0.65	0.000657	0.00574	MSANTD2	1.25E+08
chr11	1.25E+08	1.25E+08	823	-0.65	0.000657	0.00574	MSANTD2	1.25E+08
chr11	1.25E+08	1.25E+08	823	-0.65	0.000657	0.00574	MSANTD2	1.25E+08
chr11	1.25E+08	1.25E+08	823	-0.65	0.000657	0.00574	MSANTD2	1.25E+08
chr11	1.25E+08	1.25E+08	823	-0.65	0.000657	0.00574	LOC100507	1.25E+08
chr12	41086034	41086311	278	-0.94	0.00066	0.00576	CNTN1	41081243
chr12	41086034	41086311	278	-0.94	0.00066	0.00576	CNTN1	41081243
chr6	1.44E+08	1.44E+08	482	-0.65	0.000663	0.00578	SF3B5	1.44E+08
chr9	1.01E+08	1.01E+08	196	-1.76	0.000663	0.00578	MIR6854	1.01E+08
chr6	1.01E+08	1.01E+08	550	-0.66	0.000663	0.00578	SIM1	1.01E+08
chr2	32288364	32288949	586	-0.56	0.000664	0.00579	SPAST	32283679
chr2	32288364	32288949	586	-0.56	0.000664	0.00579	SPAST	32283679
chr16	90038784	90039399	616	-0.97	0.000666	0.0058	AFG3L1P	90033987
chr16	90038784	90039399	616	-0.97	0.000666	0.0058	AFG3L1P	90033987
chr16	90038784	90039399	616	-0.97	0.000666	0.0058	AFG3L1P	90033987
chr16	90038784	90039399	616	-0.97	0.000666	0.0058	CENPBD1	90037240
chr15	51202502	51202975	474	-0.76	0.000666	0.0058	AP4E1	51195868
chr15	51202502	51202975	474	-0.76	0.000666	0.0058	AP4E1	51195868
chr1	32859985	32860551	567	-0.65	0.000666	0.0058	BSDC1	32858062
chr1	32859985	32860551	567	-0.65	0.000666	0.0058	BSDC1	32858062

chr1	32859985	32860551	567	-0.65	0.000666	0.0058	BSDC1	32858062
chr1	32859985	32860551	567	-0.65	0.000666	0.0058	BSDC1	32858062
chr1	32859985	32860551	567	-0.65	0.000666	0.0058	BSDC1	32858062
chr1	32859985	32860551	567	-0.65	0.000666	0.0058	BSDC1	32858062
chr19	46405861	46406477	617	-0.69	0.000667	0.0058	MYPOP	46403862
chr1	2.07E+08	2.07E+08	822	-0.56	0.000667	0.0058	CD55	2.07E+08
chr1	2.07E+08	2.07E+08	822	-0.56	0.000667	0.0058	CD55	2.07E+08
chr1	2.07E+08	2.07E+08	822	-0.56	0.000667	0.0058	CD55	2.07E+08
chr1	2.07E+08	2.07E+08	822	-0.56	0.000667	0.0058	CD55	2.07E+08
chr1	2.07E+08	2.07E+08	822	-0.56	0.000667	0.0058	CD55	2.07E+08
chr1	2.07E+08	2.07E+08	822	-0.56	0.000667	0.0058	CD55	2.07E+08
chr7	87856296	87856872	577	-0.6	0.00067	0.00582	SRI	87854308
chr7	87856296	87856872	577	-0.6	0.00067	0.00582	SRI	87854308
chr18	3015294	3015456	163	-1.45	0.000671	0.00583	LPIN2	3009945
chr4	1.3E+08	1.3E+08	700	-0.54	0.000674	0.00585	C4orf33	1.3E+08
chr4	1.3E+08	1.3E+08	700	-0.54	0.000674	0.00585	C4orf33	1.3E+08
chr4	1.3E+08	1.3E+08	700	-0.54	0.000674	0.00585	SCLT1	1.3E+08
chr4	1.3E+08	1.3E+08	700	-0.54	0.000674	0.00585	SCLT1	1.3E+08
chr4	1.3E+08	1.3E+08	700	-0.54	0.000674	0.00585	SCLT1	1.3E+08
chr2	86422071	86422783	713	-0.56	0.000675	0.00585	IMMT	86420893
chr2	86422071	86422783	713	-0.56	0.000675	0.00585	IMMT	86420893
chr2	86422071	86422783	713	-0.56	0.000675	0.00585	IMMT	86420893
chr2	86422071	86422783	713	-0.56	0.000675	0.00585	MRPL35	86421555
chr2	86422071	86422783	713	-0.56	0.000675	0.00585	MIR4779	86418231
chr2	86422071	86422783	713	-0.56	0.000675	0.00585	MRPL35	86421555
chr9	40791391	40792291	901	-0.68	0.000682	0.0059	ZNF658	40787480
chr9	40791391	40792291	901	-0.68	0.000682	0.0059	ZNF658	40790116
chr9	40791391	40792291	901	-0.68	0.000682	0.0059	ZNF658	40790116
chr10	16478656	16479372	717	-0.48	0.000683	0.0059	PTER	16473941
chr10	16478656	16479372	717	-0.48	0.000683	0.0059	PTER	16473941
chr10	16478656	16479372	717	-0.48	0.000683	0.0059	PTER	16473941
chr10	16478656	16479372	717	-0.48	0.000683	0.0059	PTER	16473941
chr10	16478656	16479372	717	-0.48	0.000683	0.0059	PTER	16473941
chr19	36139036	36139256	221	-0.96	0.000683	0.0059	COX6B1	36134124
chr19	44506896	44507153	258	-1.26	0.000684	0.00591	ZNF230	44502076
chr19	44506896	44507153	258	-1.26	0.000684	0.00591	LOC101928	44505022
chr5	98264579	98265534	956	-0.57	0.000686	0.00592	CHD1	98260238
chr5	98264579	98265534	956	-0.57	0.000686	0.00592	LOC100289	98259837
chr11	82611382	82611706	325	-0.65	0.000686	0.00592	PRCP	82609557
chr11	82611382	82611706	325	-0.65	0.000686	0.00592	PRCP	82609557
chr11	82611382	82611706	325	-0.65	0.000686	0.00592	DDIAS	82607736
chr8	1.18E+08	1.18E+08	911	-0.51	0.000687	0.00592	UTP23	1.18E+08
chr11	6633325	6634109	785	-0.56	0.000687	0.00593	TAF10	6631475
chr9	1.36E+08	1.36E+08	556	-0.53	0.00069	0.00595	RALGDS	1.36E+08
chr11	4414524	4415185	662	-0.52	0.000694	0.00597	TRIM21	4412926
chr4	1.29E+08	1.29E+08	881	-0.52	0.000695	0.00597	MFSD8	1.29E+08
chr4	1.29E+08	1.29E+08	881	-0.52	0.000695	0.00597	ABHD18	1.29E+08

chr15	67419614	67419932	319	-1.01	0.000696	0.00598	SMAD3	67413053
chr2	1.84E+08	1.84E+08	619	-0.64	0.000696	0.00598	DUSP19	1.84E+08
chr2	1.84E+08	1.84E+08	619	-0.64	0.000696	0.00598	DUSP19	1.84E+08
chr14	37125241	37125668	428	-0.79	0.000697	0.00598	PAX9	37121772
chr3	1.12E+08	1.12E+08	319	-1.33	0.000697	0.00598	LINC01279	1.12E+08
chr10	43904184	43904854	671	-0.56	0.000698	0.00599	HNRNPF	43902332
chr10	43904184	43904854	671	-0.56	0.000698	0.00599	HNRNPF	43902696
chr10	43904184	43904854	671	-0.56	0.000698	0.00599	HNRNPF	43902656
chr10	43904184	43904854	671	-0.56	0.000698	0.00599	HNRNPF	43901299
chr8	96036989	96037548	560	-0.61	0.000698	0.00599	NDUFAF6	96032213
chr17	12920985	12921823	839	-0.49	0.000699	0.006	ELAC2	12919381
chr17	12920985	12921823	839	-0.49	0.000699	0.006	ELAC2	12919381
chr17	12920985	12921823	839	-0.49	0.000699	0.006	ELAC2	12919381
chr10	10836863	10837173	311	-0.82	0.000699	0.006	SFTA1P	10834877
chr9	77700662	77700952	291	-0.54	0.000701	0.00601	OSTF1	77698397
chr5	1.8E+08	1.8E+08	330	-1.04	0.000701	0.00601	LINC00847	1.8E+08
chr5	1.8E+08	1.8E+08	330	-1.04	0.000701	0.00601	LINC00847	1.8E+08
chr5	1.8E+08	1.8E+08	330	-1.04	0.000701	0.00601	LINC00847	1.8E+08
chr5	90675856	90676299	444	-0.62	0.000703	0.00602	ARRDC3-AS	90671163
chr8	86375493	86376283	791	-0.63	0.000704	0.00602	CA2	86371130
chr8	86375493	86376283	791	-0.63	0.000704	0.00602	CA3-AS1	86374562
chr8	86375493	86376283	791	-0.63	0.000704	0.00602	CA3-AS1	86375144
chr8	86375493	86376283	791	-0.63	0.000704	0.00602	CA2	86371130
chr8	86375493	86376283	791	-0.63	0.000704	0.00602	CA3-AS1	86373283
chr8	86375493	86376283	791	-0.63	0.000704	0.00602	CA3-AS1	86373283
chr7	22397244	22397590	347	-0.71	0.000706	0.00603	RAPGEF5	22394533
chr1	92764144	92765002	859	-0.5	0.000707	0.00603	RPAP2	92759521
chr1	92764144	92765002	859	-0.5	0.000707	0.00603	GLMN	92762566
chr2	1.9E+08	1.9E+08	958	-0.63	0.000709	0.00604	WDR75	1.9E+08
chr2	1.9E+08	1.9E+08	958	-0.63	0.000709	0.00604	WDR75	1.9E+08
chr3	64671443	64671826	384	-0.7	0.00071	0.00605	ADAMTS9	64671365
chr3	64671443	64671826	384	-0.7	0.00071	0.00605	ADAMTS9-.	64665545
chr3	64671443	64671826	384	-0.7	0.00071	0.00605	ADAMTS9	64671365
chr2	1.78E+08	1.78E+08	818	-0.7	0.00071	0.00605	TTC30B	1.78E+08
chr1	2.43E+08	2.43E+08	1233	-0.43	0.000712	0.00606	CEP170	2.43E+08
chr1	2.43E+08	2.43E+08	1233	-0.43	0.000712	0.00606	CEP170	2.43E+08
chr1	2.43E+08	2.43E+08	1233	-0.43	0.000712	0.00606	SDCCAG8	2.43E+08
chr1	2.43E+08	2.43E+08	1233	-0.43	0.000712	0.00606	CEP170	2.43E+08
chr1	1.12E+08	1.12E+08	1341	-0.49	0.000713	0.00607	FAM212B	1.12E+08
chr1	1.12E+08	1.12E+08	1341	-0.49	0.000713	0.00607	FAM212B-/	1.12E+08
chr1	1.12E+08	1.12E+08	1341	-0.49	0.000713	0.00607	FAM212B-/	1.12E+08
chr11	1.07E+08	1.07E+08	512	-0.74	0.000714	0.00607	ELMOD1	1.07E+08
chr11	1.07E+08	1.07E+08	512	-0.74	0.000714	0.00607	ELMOD1	1.07E+08
chr11	1.07E+08	1.07E+08	512	-0.74	0.000714	0.00607	ELMOD1	1.07E+08
chr11	1.07E+08	1.07E+08	512	-0.74	0.000714	0.00607	LOC64392:	1.07E+08
chr19	33462835	33463240	406	-0.62	0.000714	0.00607	CEP89	33460935
chr19	33462835	33463240	406	-0.62	0.000714	0.00607	FAAP24	33458122

chr19	33462835	33463240	406	-0.62	0.000714	0.00607	FAAP24	33458122
chr3	1.37E+08	1.37E+08	692	-0.98	0.000716	0.00608	SLC35G2	1.37E+08
chr3	1.37E+08	1.37E+08	692	-0.98	0.000716	0.00608	SLC35G2	1.37E+08
chr3	1.37E+08	1.37E+08	692	-0.98	0.000716	0.00608	SLC35G2	1.37E+08
chr7	1.51E+08	1.51E+08	751	-0.6	0.000717	0.00609	ABCB8	1.51E+08
chr7	1.51E+08	1.51E+08	751	-0.6	0.000717	0.00609	ABCB8	1.51E+08
chr7	1.51E+08	1.51E+08	751	-0.6	0.000717	0.00609	ABCB8	1.51E+08
chr7	1.51E+08	1.51E+08	751	-0.6	0.000717	0.00609	ABCB8	1.51E+08
chr7	1.51E+08	1.51E+08	751	-0.6	0.000717	0.00609	ATG9B	1.51E+08
chr7	1.51E+08	1.51E+08	751	-0.6	0.000717	0.00609	ATG9B	1.51E+08
chr7	1.51E+08	1.51E+08	751	-0.6	0.000717	0.00609	ATG9B	1.51E+08
chr4	56251457	56251861	405	-0.84	0.000718	0.00609	SRD5A3-AS	56249747
chr13	1.14E+08	1.14E+08	612	-0.77	0.000718	0.00609	ADPRHL1	1.14E+08
chr8	57906030	57906912	883	-0.53	0.000718	0.00609	IMPAD1	57904430
chr1	17577230	17577336	107	-1.67	0.00072	0.0061	PADI3	17570592
chr11	27494236	27494574	339	-0.58	0.000721	0.0061	LGR4	27492334
chr11	27494236	27494574	339	-0.58	0.000721	0.0061	LOC10537(	27488277
chr11	27494236	27494574	339	-0.58	0.000721	0.0061	LOC10537(	27488277
chr3	1.33E+08	1.33E+08	583	-0.69	0.000723	0.00612	CDV3	1.33E+08
chr3	1.33E+08	1.33E+08	583	-0.69	0.000723	0.00612	CDV3	1.33E+08
chr3	1.33E+08	1.33E+08	583	-0.69	0.000723	0.00612	CDV3	1.33E+08
chr6	80246726	80247571	846	-0.56	0.000723	0.00612	LCA5	80245147
chr6	80246726	80247571	846	-0.56	0.000723	0.00612	LCA5	80245147
chr14	61789244	61790000	757	-0.67	0.000723	0.00612	PRKCH	61783160
chr9	1.27E+08	1.27E+08	353	-0.64	0.000725	0.00613	LHX2	1.27E+08
chr3	20227552	20228052	501	-0.7	0.000726	0.00614	SGOL1	20225725
chr3	20227552	20228052	501	-0.7	0.000726	0.00614	SGOL1	20225725
chr3	20227552	20228052	501	-0.7	0.000726	0.00614	SGOL1	20225725
chr3	20227552	20228052	501	-0.7	0.000726	0.00614	SGOL1	20225725
chr3	20227552	20228052	501	-0.7	0.000726	0.00614	SGOL1	20225725
chr3	20227552	20228052	501	-0.7	0.000726	0.00614	SGOL1	20225725
chr3	20227552	20228052	501	-0.7	0.000726	0.00614	SGOL1	20225725
chr3	20227552	20228052	501	-0.7	0.000726	0.00614	SGOL1	20225725
chr3	20227552	20228052	501	-0.7	0.000726	0.00614	SGOL1	20225725
chr3	20227552	20228052	501	-0.7	0.000726	0.00614	SGOL1	20225725
chr3	20227552	20228052	501	-0.7	0.000726	0.00614	SGOL1	20225725
chr3	20227552	20228052	501	-0.7	0.000726	0.00614	SGOL1	20225725
chr3	20227552	20228052	501	-0.7	0.000726	0.00614	SGOL1	20225725
chr3	20227552	20228052	501	-0.7	0.000726	0.00614	SGOL1	20225725
chr3	20227552	20228052	501	-0.7	0.000726	0.00614	SGOL1	20225725
chr12	90102557	90103721	1165	-0.49	0.000731	0.00617	LINC00936	90097731
chr11	1.08E+08	1.08E+08	1142	-0.47	0.000732	0.00617	SLC35F2	1.08E+08
chr5	43018004	43018374	371	-0.66	0.000732	0.00617	LOC648987	43017745
chr5	43018004	43018374	371	-0.66	0.000732	0.00617	LOC648987	43016913
chr5	43018004	43018374	371	-0.66	0.000732	0.00617	LOC648987	43016913
chr5	43018004	43018374	371	-0.66	0.000732	0.00617	LOC648987	43016913
chr14	61189163	61189402	240	-0.92	0.000734	0.00618	SIX4	61188852

chr3	1.85E+08	1.85E+08	393	-0.84	0.000736	0.00619	EHHADH	1.85E+08
chr3	1.85E+08	1.85E+08	393	-0.84	0.000736	0.00619	EHHADH	1.85E+08
chr3	1.85E+08	1.85E+08	393	-0.84	0.000736	0.00619	MIR5588	1.85E+08
chr3	1.95E+08	1.95E+08	582	-0.52	0.000736	0.0062	ACAP2	1.95E+08
chr6	1.12E+08	1.12E+08	196	-1.25	0.000739	0.00621	MFSD4B	1.12E+08
chr11	13483949	13485204	1256	-0.61	0.000741	0.00623	BTBD10	13482844
chr11	13483949	13485204	1256	-0.61	0.000741	0.00623	BTBD10	13482844
chr6	57036964	57037652	689	-0.58	0.000742	0.00624	LOC101927	57035011
chr6	57036964	57037652	689	-0.58	0.000742	0.00624	BAG2	57032103
chr9	1.08E+08	1.08E+08	434	-0.9	0.000743	0.00624	ABCA1	1.08E+08
chr4	22516933	22517909	977	-0.67	0.000744	0.00624	ADGRA3	22515677
chr15	40531328	40531983	656	-0.72	0.000745	0.00624	PAK6	40526997
chr15	40531328	40531983	656	-0.72	0.000745	0.00624	PAK6	40526291
chr5	39424933	39425481	549	-0.67	0.000746	0.00625	DAB2	39423335
chr5	39424933	39425481	549	-0.67	0.000746	0.00625	DAB2	39423335
chrX	19002291	19003341	1051	-0.55	0.000746	0.00625	PHKA2	19000480
chr6	1.14E+08	1.14E+08	517	-0.62	0.000748	0.00626	MARCKS	1.14E+08
chr20	30326716	30327234	519	-0.63	0.000748	0.00626	TPX2	30321903
chr1	38325163	38325994	832	-0.66	0.000749	0.00627	MTF1	38323292
chr8	1.04E+08	1.04E+08	1060	-0.41	0.000749	0.00627	SLC25A32	1.04E+08
chr8	1.04E+08	1.04E+08	1060	-0.41	0.000749	0.00627	DCAF13	1.04E+08
chr8	1.04E+08	1.04E+08	1060	-0.41	0.000749	0.00627	SLC25A32	1.04E+08
chr8	1.04E+08	1.04E+08	1060	-0.41	0.000749	0.00627	SLC25A32	1.04E+08
chr19	12992167	12992504	338	-0.64	0.00075	0.00627	DNASE2	12990335
chr15	50716274	50716727	454	-0.77	0.00075	0.00627	USP8	50711573
chr15	50716274	50716727	454	-0.77	0.00075	0.00627	USP8	50711573
chr15	50716274	50716727	454	-0.77	0.00075	0.00627	USP8	50711573
chr4	52710758	52711098	341	-0.68	0.00075	0.00627	DCUN1D4	52704744
chr4	52710758	52711098	341	-0.68	0.00075	0.00627	DCUN1D4	52704165
chr4	52710758	52711098	341	-0.68	0.00075	0.00627	DCUN1D4	52704165
chr4	52710758	52711098	341	-0.68	0.00075	0.00627	DCUN1D4	52704165
chr14	23770545	23771651	1107	-0.4	0.000752	0.00628	BCL2L2	23771068
chr14	23770545	23771651	1107	-0.4	0.000752	0.00628	BCL2L2-PAI	23770970
chr14	23770545	23771651	1107	-0.4	0.000752	0.00628	PPP1R3E	23770057
chr14	23770545	23771651	1107	-0.4	0.000752	0.00628	BCL2L2	23770970
chr3	1.46E+08	1.46E+08	573	-0.74	0.000754	0.00629	PLSCR2	1.46E+08
chr5	1.16E+08	1.16E+08	382	-0.73	0.000754	0.0063	LOC102467	1.16E+08
chr17	63096091	63096887	797	-0.84	0.000756	0.00631	LOC100507	63091929
chr10	25351341	25351738	398	-0.56	0.000757	0.00632	ENKUR	25349208
chr13	60590403	60590650	248	-1.14	0.000762	0.00634	DIAPH3	60585343
chr13	60590403	60590650	248	-1.14	0.000762	0.00634	DIAPH3	60585343
chr11	72463424	72463976	553	-0.67	0.000763	0.00635	ARAP1	72461434
chr9	32526108	32526657	550	-0.8	0.000768	0.00638	DDX58	32524322
chr10	1.25E+08	1.25E+08	649	-0.65	0.000769	0.00638	PSTK	1.25E+08
chr5	82768982	82769562	581	-0.72	0.000769	0.00638	VCAN	82762492
chr5	82768982	82769562	581	-0.72	0.000769	0.00638	VCAN	82762492
chr5	82768982	82769562	581	-0.72	0.000769	0.00638	VCAN	82762492

chr5	82768982	82769562	581	-0.72	0.000769	0.00638	VCAN	82762492
chr5	38599067	38599317	251	-1.02	0.000769	0.00638	LIFR	38593507
chr13	76124403	76124617	215	-1.02	0.00077	0.00639	UCHL3	76118885
chr13	76124403	76124617	215	-1.02	0.00077	0.00639	UCHL3	76118615
chr13	76124403	76124617	215	-1.02	0.00077	0.00639	COMMD6	76121575
chr12	85306304	85306833	530	-0.7	0.000776	0.00642	SLC6A15	85304608
chr12	85306304	85306833	530	-0.7	0.000776	0.00642	SLC6A15	85304608
chr12	85306304	85306833	530	-0.7	0.000776	0.00642	SLC6A15	85304608
chr1	2.42E+08	2.42E+08	527	-0.59	0.000776	0.00642	EXO1	2.42E+08
chr1	2.42E+08	2.42E+08	527	-0.59	0.000776	0.00642	EXO1	2.42E+08
chr1	2.42E+08	2.42E+08	527	-0.59	0.000776	0.00642	EXO1	2.42E+08
chr6	1.33E+08	1.33E+08	951	-0.59	0.000777	0.00643	SLC18B1	1.33E+08
chr7	99746224	99746700	477	-0.73	0.000779	0.00644	LAMTOR4	99741521
chr7	99746224	99746700	477	-0.73	0.000779	0.00644	LAMTOR4	99741521
chr7	99746224	99746700	477	-0.73	0.000779	0.00644	LAMTOR4	99741521
chr7	99746224	99746700	477	-0.73	0.000779	0.00644	LAMTOR4	99741521
chr7	99746224	99746700	477	-0.73	0.000779	0.00644	LAMTOR4	99741521
chr7	99746224	99746700	477	-0.73	0.000779	0.00644	LAMTOR4	99741600
chr7	99746224	99746700	477	-0.73	0.000779	0.00644	LAMTOR4	99741600
chr7	99746224	99746700	477	-0.73	0.000779	0.00644	LAMTOR4	99741600
chr8	52811509	52812570	1062	-0.49	0.00078	0.00645	PCMTD1	52809746
chr8	52811509	52812570	1062	-0.49	0.00078	0.00645	PCMTD1	52809746
chr8	52811509	52812570	1062	-0.49	0.00078	0.00645	PCMTD1	52809746
chr4	3076065	3076413	349	-0.87	0.000781	0.00645	HTT	3071236
chr4	3076065	3076413	349	-0.87	0.000781	0.00645	HTT-AS	3074241
chr20	55901702	55902208	507	-0.55	0.000781	0.00645	SPO11	55899830
chr20	55901702	55902208	507	-0.55	0.000781	0.00645	SPO11	55899830
chr14	24895570	24896146	577	-0.67	0.000782	0.00645	KHNYN	24894140
chr14	24895570	24896146	577	-0.67	0.000782	0.00645	KHNYN	24894140
chr14	24895570	24896146	577	-0.67	0.000782	0.00645	KHNYN	24893491
chr2	1.77E+08	1.77E+08	857	-0.55	0.000783	0.00646	KIAA1715	1.77E+08
chr2	1.77E+08	1.77E+08	857	-0.55	0.000783	0.00646	KIAA1715	1.77E+08
chr2	1.77E+08	1.77E+08	857	-0.55	0.000783	0.00646	KIAA1715	1.77E+08
chr2	1.77E+08	1.77E+08	857	-0.55	0.000783	0.00646	KIAA1715	1.77E+08
chr2	1.77E+08	1.77E+08	857	-0.55	0.000783	0.00646	KIAA1715	1.77E+08
chr2	1.77E+08	1.77E+08	857	-0.55	0.000783	0.00646	KIAA1715	1.77E+08
chr1	60280112	60280645	534	-0.66	0.000784	0.00647	HOOK1	60275532
chr3	9437829	9439367	1539	-0.53	0.000784	0.00647	THUMPD3-	9437178
chr3	9437829	9439367	1539	-0.53	0.000784	0.00647	THUMPD3-	9436307
chr3	9437829	9439367	1539	-0.53	0.000784	0.00647	THUMPD3-	9437178
chr3	9437829	9439367	1539	-0.53	0.000784	0.00647	SETD5	9434383
chr3	9437829	9439367	1539	-0.53	0.000784	0.00647	SETD5	9434383
chr7	1.49E+08	1.49E+08	1079	-0.63	0.000785	0.00647	ZNF786	1.49E+08
chr9	1.15E+08	1.15E+08	787	-0.52	0.000787	0.00648	INIP	1.15E+08
chr20	34542151	34542711	561	-0.57	0.000789	0.00649	SCAND1	34540548
chr20	34542151	34542711	561	-0.57	0.000789	0.00649	SCAND1	34541281
chr10	1.21E+08	1.21E+08	687	-0.54	0.000791	0.0065	TIAL1	1.21E+08

chr10	1.21E+08	1.21E+08	687	-0.54	0.000791	0.0065	TIAL1	1.21E+08
chr13	47128476	47128955	480	-0.76	0.000791	0.00651	LRCH1	47122295
chr13	47128476	47128955	480	-0.76	0.000791	0.00651	LRCH1	47122295
chr13	47128476	47128955	480	-0.76	0.000791	0.00651	LRCH1	47122295
chr19	50093399	50093681	283	-0.88	0.000792	0.00651	PRR12	50089911
chr4	39183754	39184193	440	-0.63	0.000793	0.00651	WDR19	39179023
chr4	39183754	39184193	440	-0.63	0.000793	0.00651	WDR19	39179023
chr2	1.9E+08	1.9E+08	193	-2.11	0.000797	0.00654	DIRC1	1.9E+08
chr5	1.57E+08	1.57E+08	967	-0.48	0.000797	0.00654	MED7	1.57E+08
chr5	1.57E+08	1.57E+08	967	-0.48	0.000797	0.00654	MED7	1.57E+08
chr16	24740747	24741324	578	-0.86	0.000798	0.00654	TNRC6A	24736048
chr12	1.05E+08	1.05E+08	1145	-0.48	0.000798	0.00655	TXNRD1	1.05E+08
chr12	1.05E+08	1.05E+08	1145	-0.48	0.000798	0.00655	TXNRD1	1.05E+08
chr12	1.05E+08	1.05E+08	1145	-0.48	0.000798	0.00655	TXNRD1	1.05E+08
chr12	1.05E+08	1.05E+08	1145	-0.48	0.000798	0.00655	TXNRD1	1.05E+08
chr12	1.05E+08	1.05E+08	1145	-0.48	0.000798	0.00655	TXNRD1	1.05E+08
chr12	1.05E+08	1.05E+08	1145	-0.48	0.000798	0.00655	TXNRD1	1.05E+08
chr2	1.01E+08	1.01E+08	884	-0.49	0.000802	0.00657	PDCL3	1.01E+08
chr8	1.08E+08	1.08E+08	864	-0.72	0.000805	0.00658	ABRA	1.08E+08
chr3	1.56E+08	1.56E+08	564	-0.45	0.000808	0.0066	GMPS	1.56E+08
chr22	29999216	29999641	426	-0.75	0.000809	0.0066	NF2	29994544
chr22	29999216	29999641	426	-0.75	0.000809	0.0066	NF2	29994544
chr22	29999216	29999641	426	-0.75	0.000809	0.0066	NF2	29994544
chr22	29999216	29999641	426	-0.75	0.000809	0.0066	NF2	29994544
chr22	29999216	29999641	426	-0.75	0.000809	0.0066	NF2	29994544
chr22	29999216	29999641	426	-0.75	0.000809	0.0066	NF2	29994544
chr22	29999216	29999641	426	-0.75	0.000809	0.0066	NF2	29994544
chr22	29999216	29999641	426	-0.75	0.000809	0.0066	NF2	29994544
chr22	29999216	29999641	426	-0.75	0.000809	0.0066	NF2	29994544
chr2	1.91E+08	1.91E+08	910	-0.51	0.000809	0.0066	PMS1	1.91E+08
chr2	1.91E+08	1.91E+08	910	-0.51	0.000809	0.0066	PMS1	1.91E+08
chr2	1.91E+08	1.91E+08	910	-0.51	0.000809	0.0066	PMS1	1.91E+08
chr2	1.91E+08	1.91E+08	910	-0.51	0.000809	0.0066	PMS1	1.91E+08
chr2	1.91E+08	1.91E+08	910	-0.51	0.000809	0.0066	PMS1	1.91E+08
chr2	1.91E+08	1.91E+08	910	-0.51	0.000809	0.0066	PMS1	1.91E+08
chr2	1.91E+08	1.91E+08	910	-0.51	0.000809	0.0066	ORMDL1	1.91E+08
chr2	1.91E+08	1.91E+08	910	-0.51	0.000809	0.0066	ORMDL1	1.91E+08
chr11	58873875	58874760	886	-0.53	0.00081	0.0066	FAM111B	58869657
chr11	58873875	58874760	886	-0.53	0.00081	0.0066	FAM111B	58869657
chr11	58873875	58874760	886	-0.53	0.00081	0.0066	FAM111B	58869657
chr3	1.57E+08	1.57E+08	922	-0.52	0.000811	0.00661	LEKR1	1.57E+08
chr3	1.57E+08	1.57E+08	922	-0.52	0.000811	0.00661	LEKR1	1.57E+08
chr1	90456641	90456768	128	-0.8	0.000812	0.00662	ZNF326	90455677
chr1	90456641	90456768	128	-0.8	0.000812	0.00662	ZNF326	90455677
chr12	48157396	48157753	358	-0.74	0.000813	0.00662	RAPGEF3	48150889
chr6	52535730	52536051	322	-0.83	0.000814	0.00663	TMEM14A	52530883
chr1	54518829	54519331	503	-0.67	0.000814	0.00663	TCEANC2	54514244

chr1	54518829	54519331	503	-0.67	0.000814	0.00663	TCEANC2	54514244
chr1	54518829	54519331	503	-0.67	0.000814	0.00663	TMEM59	54517111
chr1	54518829	54519331	503	-0.67	0.000814	0.00663	TMEM59	54517111
chr1	54518829	54519331	503	-0.67	0.000814	0.00663	TMEM59	54517246
chr1	54518829	54519331	503	-0.67	0.000814	0.00663	TMEM59	54517246
chr1	54518829	54519331	503	-0.67	0.000814	0.00663	TMEM59	54517111
chr1	54518829	54519331	503	-0.67	0.000814	0.00663	TMEM59	54517246
chr1	54518829	54519331	503	-0.67	0.000814	0.00663	TMEM59	54517111
chr1	54518829	54519331	503	-0.67	0.000814	0.00663	MIR4781	54514751
chr14	1.04E+08	1.04E+08	489	-0.47	0.000815	0.00663	XRCC3	1.04E+08
chr14	1.04E+08	1.04E+08	489	-0.47	0.000815	0.00663	XRCC3	1.04E+08
chr14	1.04E+08	1.04E+08	489	-0.47	0.000815	0.00663	ZFYVE21	1.04E+08
chr14	1.04E+08	1.04E+08	489	-0.47	0.000815	0.00663	XRCC3	1.04E+08
chr14	1.04E+08	1.04E+08	489	-0.47	0.000815	0.00663	ZFYVE21	1.04E+08
chr2	11884207	11884499	293	-0.62	0.000816	0.00663	LPIN1	11881721
chr9	1.16E+08	1.16E+08	353	-0.6	0.000817	0.00664	RGS3	1.16E+08
chr9	1.16E+08	1.16E+08	353	-0.6	0.000817	0.00664	RGS3	1.16E+08
chr9	88555707	88556580	874	-0.45	0.000817	0.00664	NAA35	88551056
chr12	57351173	57351476	304	-1.03	0.000822	0.00667	RDH16	57349418
chr14	60714715	60714966	252	-0.67	0.000823	0.00668	PPM1A	60710965
chr14	60714715	60714966	252	-0.67	0.000823	0.00668	PPM1A	60710965
chr1	1.76E+08	1.76E+08	1421	-0.4	0.000823	0.00668	RFWD2	1.76E+08
chr1	1.76E+08	1.76E+08	1421	-0.4	0.000823	0.00668	RFWD2	1.76E+08
chr1	1.76E+08	1.76E+08	1421	-0.4	0.000823	0.00668	RFWD2	1.76E+08
chr10	35415526	35416602	1077	-0.55	0.000825	0.00668	CREM	35410768
chr10	35415526	35416602	1077	-0.55	0.000825	0.00668	CREM	35411384
chr10	35415526	35416602	1077	-0.55	0.000825	0.00668	CREM	35411384
chr10	35415526	35416602	1077	-0.55	0.000825	0.00668	CREM	35410768
chr10	35415526	35416602	1077	-0.55	0.000825	0.00668	CREM	35410768
chr10	35415526	35416602	1077	-0.55	0.000825	0.00668	CREM	35410768
chr10	35415526	35416602	1077	-0.55	0.000825	0.00668	CREM	35410768
chr10	35415526	35416602	1077	-0.55	0.000825	0.00668	CREM	35410768
chr10	35415526	35416602	1077	-0.55	0.000825	0.00668	CREM	35410768
chr2	2.03E+08	2.03E+08	489	-0.58	0.000826	0.00669	FAM117B	2.03E+08
chr7	1.39E+08	1.39E+08	549	-0.82	0.000826	0.00669	UBN2	1.39E+08
chr5	43120934	43121871	938	-0.48	0.000827	0.00669	ZNF131	43116609
chr5	43120934	43121871	938	-0.48	0.000827	0.00669	ZNF131	43116595
chr5	43120934	43121871	938	-0.48	0.000827	0.00669	ZNF131	43116595
chr5	43120934	43121871	938	-0.48	0.000827	0.00669	ZNF131	43116002
chr11	45939413	45939834	422	-0.68	0.000827	0.0067	PEX16	45937674
chr11	45939413	45939834	422	-0.68	0.000827	0.0067	GYLTL1B	45938171
chr11	45939413	45939834	422	-0.68	0.000827	0.0067	GYLTL1B	45938171
chr11	45939413	45939834	422	-0.68	0.000827	0.0067	GYLTL1B	45939226
chr11	45939413	45939834	422	-0.68	0.000827	0.0067	PEX16	45937674
chr12	54776871	54777195	325	-0.93	0.000828	0.0067	ZNF385A	54776514
chr12	54776871	54777195	325	-0.93	0.000828	0.0067	ZNF385A	54776514
chr3	40429592	40429994	403	-1.17	0.00083	0.00671	ENTPD3	40423646

chr3	40429592	40429994	403	-1.17	0.00083	0.00671	ENTPD3	40423646
chr3	40429592	40429994	403	-1.17	0.00083	0.00671	ENTPD3	40423735
chr14	1.06E+08	1.06E+08	224	-1.32	0.000831	0.00671	GPR132	1.06E+08
chr14	1.06E+08	1.06E+08	224	-1.32	0.000831	0.00671	GPR132	1.06E+08
chr14	1.06E+08	1.06E+08	224	-1.32	0.000831	0.00671	GPR132	1.06E+08
chr14	1.06E+08	1.06E+08	224	-1.32	0.000831	0.00671	GPR132	1.06E+08
chrX	14891144	14891705	562	-0.5	0.000832	0.00672	FANCB	14889184
chrX	14891144	14891705	562	-0.5	0.000832	0.00672	FANCB	14889184
chrX	14891144	14891705	562	-0.5	0.000832	0.00672	MOSPD2	14886526
chr7	17411851	17412149	299	-0.78	0.000833	0.00673	KCCAT333	17409540
chr7	17411851	17412149	299	-0.78	0.000833	0.00673	KCCAT333	17409540
chr15	92396134	92397014	881	-0.61	0.000836	0.00674	SLCO3A1	92391937
chr15	92396134	92397014	881	-0.61	0.000836	0.00674	SLCO3A1	92391937
chr1	1.85E+08	1.85E+08	847	-0.72	0.000836	0.00674	IVNS1ABP	1.85E+08
chr6	1.06E+08	1.06E+08	368	-1.2	0.000837	0.00675	POPDC3	1.06E+08
chr17	47841464	47841907	444	-0.64	0.000838	0.00676	FAM117A	47839518
chr1	45272869	45273819	951	-0.47	0.000842	0.00678	BTBD19	45269153
chr1	45272869	45273819	951	-0.47	0.000842	0.00678	TCTEX1D4	45270957
chr17	15009939	15010150	212	-1.1	0.000842	0.00678	CDRT8	15007504
chr7	7140058	7140398	341	-0.57	0.000844	0.00679	LOC100131	7134417
chr11	73087220	73087667	448	-0.45	0.000845	0.0068	RELT	73082712
chr11	73087220	73087667	448	-0.45	0.000845	0.0068	RELT	73082404
chr11	1.26E+08	1.26E+08	539	-0.88	0.000848	0.00681	DDX25	1.26E+08
chr11	1.26E+08	1.26E+08	539	-0.88	0.000848	0.00681	PUS3	1.26E+08
chr11	1.26E+08	1.26E+08	539	-0.88	0.000848	0.00681	PUS3	1.26E+08
chr19	47776763	47777584	822	-0.68	0.000848	0.00682	INAFM1	47773141
chr19	47776763	47777584	822	-0.68	0.000848	0.00682	INAFM1	47772658
chr7	1.35E+08	1.35E+08	382	-0.96	0.000848	0.00682	WDR91	1.35E+08
chr15	90548077	90548683	607	-0.99	0.000854	0.00685	MIR3174	90544986
chr2	1.57E+08	1.57E+08	350	-0.69	0.000854	0.00685	NR4A2	1.57E+08
chr16	67429673	67429883	211	-0.99	0.000855	0.00686	TPPP3	67425438
chr16	67429673	67429883	211	-0.99	0.000855	0.00686	TPPP3	67425438
chr6	10722958	10723451	494	-0.63	0.000856	0.00686	TMEM14C	10718147
chr6	10722958	10723451	494	-0.63	0.000856	0.00686	TMEM14C	10718147
chr16	4817111	4817437	327	-0.77	0.000857	0.00686	ZNF500	4815219
chr16	4817111	4817437	327	-0.77	0.000857	0.00686	ZNF500	4815219
chr9	1.1E+08	1.1E+08	275	-0.63	0.000857	0.00686	RAD23B	1.1E+08
chr9	1.1E+08	1.1E+08	275	-0.63	0.000857	0.00686	RAD23B	1.1E+08
chr9	1.1E+08	1.1E+08	275	-0.63	0.000857	0.00686	RAD23B	1.1E+08
chr8	90914031	90915059	1029	-0.54	0.000859	0.00687	OSGIN2	90909095
chr8	90914031	90915059	1029	-0.54	0.000859	0.00687	OSGIN2	90909761
chr1	70671092	70671735	644	-0.87	0.000859	0.00688	LRRC40	70669361
chr1	70671092	70671735	644	-0.87	0.000859	0.00688	SRSF11	70666364
chr1	70671092	70671735	644	-0.87	0.000859	0.00688	SRSF11	70666364
chr5	1.08E+08	1.08E+08	877	-0.53	0.000859	0.00688	FER	1.08E+08
chr5	1.08E+08	1.08E+08	877	-0.53	0.000859	0.00688	FER	1.08E+08
chr5	1.08E+08	1.08E+08	877	-0.53	0.000859	0.00688	FER	1.08E+08

chr11	66103901	66104536	636	-0.7	0.000864	0.00691	RIN1	66102000
chr10	75936132	75936942	811	-0.61	0.000866	0.00692	ADK	75931246
chr10	75936132	75936942	811	-0.61	0.000866	0.00692	ADK	75931246
chr16	31724344	31724845	502	-0.59	0.000868	0.00693	ZNF720	31719549
chrX	18443311	18443827	517	-0.56	0.000868	0.00693	CDKL5	18438724
chr7	16793046	16793474	429	-0.61	0.000874	0.00697	TSPAN13	16788350
chr12	9600081	9601011	931	-0.86	0.000874	0.00697	DDX12P	9598768
chr16	8962238	8963162	925	-0.5	0.000876	0.00697	CARHSP1	8959285
chr16	8962238	8963162	925	-0.5	0.000876	0.00697	CARHSP1	8960218
chr16	8962238	8963162	925	-0.5	0.000876	0.00697	CARHSP1	8960576
chr16	8962238	8963162	925	-0.5	0.000876	0.00697	CARHSP1	8960869
chr16	8962238	8963162	925	-0.5	0.000876	0.00697	CARHSP1	8960869
chr16	8962238	8963162	925	-0.5	0.000876	0.00697	CARHSP1	8960258
chr19	3971106	3971483	378	-0.88	0.000877	0.00698	DAPK3	3969121
chr9	99540131	99540548	418	-0.79	0.000877	0.00698	ZNF510	99538411
chr9	99540131	99540548	418	-0.79	0.000877	0.00698	ZNF510	99538411
chr9	99540131	99540548	418	-0.79	0.000877	0.00698	ZNF510	99538348
chr1	51800452	51800663	212	-1.03	0.000878	0.00698	TTC39A	51794236
chr1	51800452	51800663	212	-1.03	0.000878	0.00698	TTC39A	51794236
chr1	51800452	51800663	212	-1.03	0.000878	0.00698	TTC39A	51793874
chr2	1.09E+08	1.09E+08	386	-0.74	0.000878	0.00698	LIMS1	1.09E+08
chr2	2.11E+08	2.11E+08	601	-0.57	0.000879	0.00699	RPE	2.11E+08
chr2	2.11E+08	2.11E+08	601	-0.57	0.000879	0.00699	RPE	2.11E+08
chr2	2.11E+08	2.11E+08	601	-0.57	0.000879	0.00699	RPE	2.11E+08
chr2	2.11E+08	2.11E+08	601	-0.57	0.000879	0.00699	RPE	2.11E+08
chr2	2.11E+08	2.11E+08	601	-0.57	0.000879	0.00699	RPE	2.11E+08
chr2	2.11E+08	2.11E+08	601	-0.57	0.000879	0.00699	RPE	2.11E+08
chr2	2.11E+08	2.11E+08	601	-0.57	0.000879	0.00699	RPE	2.11E+08
chr2	2.11E+08	2.11E+08	601	-0.57	0.000879	0.00699	RPE	2.11E+08
chr2	2.11E+08	2.11E+08	601	-0.57	0.000879	0.00699	RPE	2.11E+08
chr2	2.11E+08	2.11E+08	601	-0.57	0.000879	0.00699	RPE	2.11E+08
chr2	2.11E+08	2.11E+08	601	-0.57	0.000879	0.00699	RPE	2.11E+08
chr2	2.11E+08	2.11E+08	601	-0.57	0.000879	0.00699	RPE	2.11E+08
chr2	2.11E+08	2.11E+08	601	-0.57	0.000879	0.00699	RPE	2.11E+08
chr2	2.11E+08	2.11E+08	601	-0.57	0.000879	0.00699	RPE	2.11E+08
chr10	1.34E+08	1.34E+08	140	-1.08	0.000885	0.00702	C10orf91	1.34E+08
chr11	1.19E+08	1.19E+08	622	-0.78	0.000888	0.00704	MCAM	1.19E+08
chr1	1.7E+08	1.7E+08	832	-0.45	0.00089	0.00705	SCYL3	1.7E+08
chr1	1.7E+08	1.7E+08	832	-0.45	0.00089	0.00705	SCYL3	1.7E+08
chr9	1.12E+08	1.12E+08	421	-0.57	0.000891	0.00705	TMEM245	1.12E+08
chr5	1.12E+08	1.12E+08	373	-0.68	0.000891	0.00706	APC	1.12E+08
chr5	1.12E+08	1.12E+08	373	-0.68	0.000891	0.00706	APC	1.12E+08
chr7	32534767	32535554	788	-0.53	0.000892	0.00706	LSM5	32532870
chr7	32534767	32535554	788	-0.53	0.000892	0.00706	AVL9	32530037
chr7	32534767	32535554	788	-0.53	0.000892	0.00706	LSM5	32528475
chr7	32534767	32535554	788	-0.53	0.000892	0.00706	LSM5	32532870
chr7	32534767	32535554	788	-0.53	0.000892	0.00706	LSM5	32528023

chr14	34931362	34931869	508	-0.59	0.000893	0.00707	SPTSSA	34929468
chr14	65006903	65007474	572	-0.48	0.000894	0.00707	HSPA2	65002185
chr14	65006903	65007474	572	-0.48	0.000894	0.00707	LOC10272з	65005086
chr1	34628657	34629060	404	-0.72	0.000895	0.00708	CSMD2	34628875
chr1	34628657	34629060	404	-0.72	0.000895	0.00708	C1orf94	34627483
chr15	40226065	40226398	334	-0.93	0.000896	0.00708	EIF2AK4	40221324
chr18	657452	658438	987	-0.62	0.000899	0.0071	TYMSOS	656340
chr18	657452	658438	987	-0.62	0.000899	0.0071	TYMS	652603
chr17	46045287	46045521	235	-1.18	9.00E-04	0.00711	CDK5RAP3	46042893
chr17	46045287	46045521	235	-1.18	9.00E-04	0.00711	CDK5RAP3	46043321
chr17	46045287	46045521	235	-1.18	9.00E-04	0.00711	CDK5RAP3	46043321
chr17	46045287	46045521	235	-1.18	9.00E-04	0.00711	CDK5RAP3	46043321
chr17	46045287	46045521	235	-1.18	9.00E-04	0.00711	CDK5RAP3	46043321
chr3	1E+08	1E+08	559	-0.69	0.000901	0.00711	TMEM45A	1E+08
chr10	1.16E+08	1.16E+08	667	-0.51	0.000901	0.00711	TDRD1	1.16E+08
chr10	1.16E+08	1.16E+08	667	-0.51	0.000901	0.00711	MIR2110	1.16E+08
chr10	1.16E+08	1.16E+08	667	-0.51	0.000901	0.00711	CCDC186	1.16E+08
chr19	55629021	55629302	282	-0.86	0.000902	0.00711	PPP1R12C	55626968
chr19	55629021	55629302	282	-0.86	0.000902	0.00711	PPP1R12C	55626968
chr13	31735974	31736656	683	-0.56	0.000906	0.00714	HSPH1	31734158
chr13	31735974	31736656	683	-0.56	0.000906	0.00714	HSPH1	31734525
chr13	31735974	31736656	683	-0.56	0.000906	0.00714	HSPH1	31734525
chr13	31735974	31736656	683	-0.56	0.000906	0.00714	HSPH1	31734158
chr17	79980717	79981170	454	-0.55	0.000907	0.00714	STRA13	79978785
chr17	79980717	79981170	454	-0.55	0.000907	0.00714	STRA13	79978785
chr17	79980717	79981170	454	-0.55	0.000907	0.00714	STRA13	79978785
chr17	79980717	79981170	454	-0.55	0.000907	0.00714	LRRC45	79976177
chr19	6424829	6425592	764	-0.58	0.000908	0.00715	KHSRP	6422822
chr12	75874367	75874683	317	-0.75	0.00091	0.00716	GLIPR1	75869512
chr17	21191150	21191637	488	-0.68	0.000912	0.00717	MAP2K3	21186347
chr8	97507425	97507634	210	-0.85	0.000914	0.00718	SDC2	97500881
chr9	1.01E+08	1.01E+08	1441	-0.43	0.000915	0.00719	CORO2A	1.01E+08
chr10	35930808	35931676	869	-0.55	0.000916	0.00719	FZD8	35928362
chr10	35930808	35931676	869	-0.55	0.000916	0.00719	MIR4683	35928180
chr15	35261754	35262234	481	-0.74	0.000917	0.0072	AQR	35259995
chr20	17549682	17551058	1377	-0.42	0.000917	0.0072	DSTN	17545598
chr20	17549682	17551058	1377	-0.42	0.000917	0.0072	DSTN	17545598
chr20	17549682	17551058	1377	-0.42	0.000917	0.0072	BFSP1	17547865
chr11	1.3E+08	1.3E+08	585	-0.59	0.000919	0.00721	ADAMTS15	1.3E+08
chr1	85086356	85086807	452	-0.85	0.00092	0.00721	LINC01461	85084714
chr19	39340637	39341409	773	-0.44	0.000921	0.00721	HNRNPL	39340979
chr19	39340637	39341409	773	-0.44	0.000921	0.00721	HNRNPL	39338617
chr10	14879899	14880395	497	-0.57	0.000921	0.00721	HSPA14	14875158
chr10	14879899	14880395	497	-0.57	0.000921	0.00721	CDNF	14877983
chr10	14879899	14880395	497	-0.57	0.000921	0.00721	HSPA14	14875158
chr10	14879899	14880395	497	-0.57	0.000921	0.00721	HSPA14	14875158
chr9	89656853	89657414	562	-0.66	0.000922	0.00722	LOC44017з	89655041

chr1	67628707	67629065	359	-1.07	0.000923	0.00723	IL23R	67627168
chr10	1.13E+08	1.13E+08	817	-0.54	0.000925	0.00724	PDCD4	1.13E+08
chr10	1.13E+08	1.13E+08	817	-0.54	0.000925	0.00724	PDCD4-AS1	1.13E+08
chr10	1.13E+08	1.13E+08	817	-0.54	0.000925	0.00724	PDCD4	1.13E+08
chr10	1.13E+08	1.13E+08	817	-0.54	0.000925	0.00724	PDCD4	1.13E+08
chr5	1.26E+08	1.26E+08	186	-0.95	0.000926	0.00725	PHAX	1.26E+08
chr5	1.26E+08	1.26E+08	186	-0.95	0.000926	0.00725	ALDH7A1	1.26E+08
chr5	1.26E+08	1.26E+08	186	-0.95	0.000926	0.00725	ALDH7A1	1.26E+08
chr5	1.26E+08	1.26E+08	186	-0.95	0.000926	0.00725	ALDH7A1	1.26E+08
chr12	1.14E+08	1.14E+08	285	-0.91	0.000927	0.00725	SDSL	1.14E+08
chr12	1.14E+08	1.14E+08	285	-0.91	0.000927	0.00725	SDSL	1.14E+08
chr14	94412851	94413349	499	-0.7	0.000928	0.00726	MIR4506	94412648
chr19	42829429	42829785	357	-0.73	0.000929	0.00726	MEGF8	42824760
chr19	42829429	42829785	357	-0.73	0.000929	0.00726	MEGF8	42824760
chr20	13201869	13202568	700	-0.75	0.00093	0.00727	ISM1	13197417
chr10	1.19E+08	1.19E+08	973	-0.54	0.000931	0.00728	SHTN1	1.19E+08
chr10	1.19E+08	1.19E+08	973	-0.54	0.000931	0.00728	SHTN1	1.19E+08
chr10	1.19E+08	1.19E+08	973	-0.54	0.000931	0.00728	SHTN1	1.19E+08
chr10	1.19E+08	1.19E+08	973	-0.54	0.000931	0.00728	SHTN1	1.19E+08
chr5	49736491	49737373	883	-0.57	0.000933	0.00728	EMB	49735234
chr10	52384256	52385198	943	-0.49	0.000936	0.0073	SGMS1	52381737
chr10	52384256	52385198	943	-0.49	0.000936	0.0073	SGMS1-AS1	52379710
chr14	21150568	21151669	1102	-0.49	0.000938	0.00731	ANG	21147335
chr14	21150568	21151669	1102	-0.49	0.000938	0.00731	RNASE4	21147371
chr14	21150568	21151669	1102	-0.49	0.000938	0.00731	RNASE4	21147371
chr14	21150568	21151669	1102	-0.49	0.000938	0.00731	RNASE4	21147371
chr3	1.79E+08	1.79E+08	872	-0.49	0.000938	0.00731	GNB4	1.79E+08
chr7	64023264	64023772	509	-0.9	0.000939	0.00732	ZNF680	64021505
chr7	64023264	64023772	509	-0.9	0.000939	0.00732	ZNF680	64021505
chr6	1.62E+08	1.62E+08	459	-0.75	0.00094	0.00732	AGPAT4	1.62E+08
chr5	95297069	95298093	1025	-0.5	0.000947	0.00736	LOC10192	95292704
chr5	95297069	95298093	1025	-0.5	0.000947	0.00736	ELL2	95295775
chr4	56412895	56413377	483	-0.75	0.00095	0.00738	CLOCK	56411076
chr4	56412895	56413377	483	-0.75	0.00095	0.00738	CLOCK	56410099
chr7	1.34E+08	1.34E+08	545	-0.74	0.000953	0.0074	CALD1	1.34E+08
chr7	1.34E+08	1.34E+08	545	-0.74	0.000953	0.0074	CALD1	1.34E+08
chr7	1.34E+08	1.34E+08	545	-0.74	0.000953	0.0074	CALD1	1.34E+08
chr8	1.34E+08	1.34E+08	859	-0.5	0.000955	0.00741	PHF20L1	1.34E+08
chr8	1.34E+08	1.34E+08	859	-0.5	0.000955	0.00741	PHF20L1	1.34E+08
chr8	1.34E+08	1.34E+08	859	-0.5	0.000955	0.00741	PHF20L1	1.34E+08
chr13	50366781	50367361	581	-0.66	0.000955	0.00741	KPNA3	50365057
chr9	92788097	92788438	342	-0.62	0.000958	0.00742	MIR4290	92783817
chr5	1.31E+08	1.31E+08	352	-0.65	0.000959	0.00743	CSF2	1.31E+08
chr16	30773258	30773675	418	-0.73	0.000959	0.00743	CCDC189	30771565
chr16	30773258	30773675	418	-0.73	0.000959	0.00743	RNF40	30767932
chr16	30773258	30773675	418	-0.73	0.000959	0.00743	RNF40	30768609
chr16	30773258	30773675	418	-0.73	0.000959	0.00743	RNF40	30767932

chr16	30773258	30773675	418	-0.73	0.000959	0.00743	CCDC189	30771565
chr16	30773258	30773675	418	-0.73	0.000959	0.00743	RNF40	30767932
chr3	1.32E+08	1.32E+08	1067	-0.62	0.00096	0.00743	ACPP	1.32E+08
chr3	1.32E+08	1.32E+08	1067	-0.62	0.00096	0.00743	ACPP	1.32E+08
chr3	1.32E+08	1.32E+08	1067	-0.62	0.00096	0.00743	ACPP	1.32E+08
chr16	3073340	3074386	1047	-0.46	0.000961	0.00743	HCFC1R1	3072287
chr16	3073340	3074386	1047	-0.46	0.000961	0.00743	HCFC1R1	3072287
chr16	3073340	3074386	1047	-0.46	0.000961	0.00743	THOC6	3069031
chr16	3073340	3074386	1047	-0.46	0.000961	0.00743	HCFC1R1	3072287
chr16	3073340	3074386	1047	-0.46	0.000961	0.00743	HCFC1R1	3072287
chr16	3073340	3074386	1047	-0.46	0.000961	0.00743	HCFC1R1	3072287
chr16	3073340	3074386	1047	-0.46	0.000961	0.00743	HCFC1R1	3072287
chr16	3073340	3074386	1047	-0.46	0.000961	0.00743	HCFC1R1	3072287
chr16	3073340	3074386	1047	-0.46	0.000961	0.00743	HCFC1R1	3072287
chr16	3073340	3074386	1047	-0.46	0.000961	0.00743	THOC6	3069031
chr19	46195233	46195947	715	-0.63	0.000963	0.00745	QPCTL	46190740
chr19	46195233	46195947	715	-0.63	0.000963	0.00745	SNRPD2	46193443
chr19	46195233	46195947	715	-0.63	0.000963	0.00745	QPCTL	46190740
chr19	46195233	46195947	715	-0.63	0.000963	0.00745	SNRPD2	46193443
chr1	2.1E+08	2.1E+08	336	-0.72	0.000964	0.00745	IRF6	2.1E+08
chr1	2.1E+08	2.1E+08	336	-0.72	0.000964	0.00745	IRF6	2.1E+08
chr11	86748659	86749143	485	-0.66	0.000967	0.00747	TMEM135	86743885
chr11	86748659	86749143	485	-0.66	0.000967	0.00747	TMEM135	86743885
chr11	86748659	86749143	485	-0.66	0.000967	0.00747	TMEM135	86743885
chr12	72079741	72080317	577	-0.6	0.000971	0.00749	TMEM19	72074877
chr12	94954644	94955019	376	-0.75	0.000971	0.00749	MIR5700	94950564
chr13	20534261	20534490	230	-0.97	0.000971	0.00749	ZMYM2	20527847
chr13	20534261	20534490	230	-0.97	0.000971	0.00749	ZMYM2	20527973
chr13	20534261	20534490	230	-0.97	0.000971	0.00749	ZMYM2	20527809
chr13	20534261	20534490	230	-0.97	0.000971	0.00749	ZMYM2	20527809
chr15	56285444	56285933	490	-0.76	0.000974	0.0075	NEDD4	56283944
chr16	54407468	54408057	590	-0.72	0.000975	0.00751	LOC10099€	54402370
chr16	54407468	54408057	590	-0.72	0.000975	0.00751	LOC10099€	54402370
chr16	54407468	54408057	590	-0.72	0.000975	0.00751	LOC10099€	54402611
chr8	1.01E+08	1.01E+08	170	-1.44	0.000976	0.00752	RGS22	1.01E+08
chr8	1.01E+08	1.01E+08	170	-1.44	0.000976	0.00752	RGS22	1.01E+08
chr8	1.01E+08	1.01E+08	170	-1.44	0.000976	0.00752	RGS22	1.01E+08
chr12	6643069	6643681	613	-0.62	0.000981	0.00755	GAPDH	6638570
chr12	6643069	6643681	613	-0.62	0.000981	0.00755	GAPDH	6639384
chr12	6643069	6643681	613	-0.62	0.000981	0.00755	GAPDH	6638824
chr12	6643069	6643681	613	-0.62	0.000981	0.00755	GAPDH	6638570
chr1	91486838	91488021	1184	-0.37	0.000984	0.00756	ZNF644	91485812
chr1	91486838	91488021	1184	-0.37	0.000984	0.00756	ZNF644	91485065
chr1	91486838	91488021	1184	-0.37	0.000984	0.00756	ZNF644	91485812
chr17	17875618	17876227	610	-0.45	0.000984	0.00756	TOM1L2	17873784
chr17	17875618	17876227	610	-0.45	0.000984	0.00756	TOM1L2	17873784
chr17	17875618	17876227	610	-0.45	0.000984	0.00756	TOM1L2	17873784

chr17	17875618	17876227	610	-0.45	0.000984	0.00756	TOM1L2	17873784
chr17	17875618	17876227	610	-0.45	0.000984	0.00756	TOM1L2	17873784
chr17	17875618	17876227	610	-0.45	0.000984	0.00756	TOM1L2	17873784
chr17	17875618	17876227	610	-0.45	0.000984	0.00756	DRC3	17871126
chr17	17875618	17876227	610	-0.45	0.000984	0.00756	DRC3	17871126
chr17	17875618	17876227	610	-0.45	0.000984	0.00756	DRC3	17871126
chr17	17875618	17876227	610	-0.45	0.000984	0.00756	DRC3	17871126
chr11	63949144	63949270	127	-0.88	0.000986	0.00757	STIP1	63947743
chr11	63949144	63949270	127	-0.88	0.000986	0.00757	STIP1	63948586
chr11	63949144	63949270	127	-0.88	0.000986	0.00757	STIP1	63948586
chr20	22564788	22565675	888	-0.66	0.000987	0.00758	FOXA2	22563101
chr20	22564788	22565675	888	-0.66	0.000987	0.00758	FOXA2	22564101
chr8	72273921	72274595	675	-0.61	0.000987	0.00758	EYA1	72272467
chr8	72273921	72274595	675	-0.61	0.000987	0.00758	EYA1	72266924
chr8	72273921	72274595	675	-0.61	0.000987	0.00758	EYA1	72266924
chr8	72273921	72274595	675	-0.61	0.000987	0.00758	EYA1	72272467
chr8	72273921	72274595	675	-0.61	0.000987	0.00758	EYA1	72272467
chr3	1.93E+08	1.93E+08	836	-0.53	0.000988	0.00758	MB21D2	1.93E+08
chr10	82167695	82168357	663	-0.82	0.000988	0.00758	FAM213A	82163241
chr10	82167695	82168357	663	-0.82	0.000988	0.00758	FAM213A	82163241
chr10	82167695	82168357	663	-0.82	0.000988	0.00758	FAM213A	82163241
chr10	82167695	82168357	663	-0.82	0.000988	0.00758	FAM213A	82162584
chr18	32923827	32924557	731	-0.61	0.00099	0.00759	ZNF24	32922431
chr18	32923827	32924557	731	-0.61	0.00099	0.00759	ZNF24	32922431
chr6	1.44E+08	1.44E+08	402	-0.87	0.00099	0.00759	STX11	1.44E+08
chr3	33840001	33840721	721	-0.64	0.000991	0.00759	PDCD6IP	33835062
chr3	33840001	33840721	721	-0.64	0.000991	0.00759	PDCD6IP	33835062
chr3	33840001	33840721	721	-0.64	0.000991	0.00759	PDCD6IP	33835062
chr16	19533231	19533544	314	-0.69	0.000994	0.0076	GDE1	19531450
chr16	19533231	19533544	314	-0.69	0.000994	0.0076	CCP110	19530178
chr16	19533231	19533544	314	-0.69	0.000994	0.0076	CCP110	19530178
chr2	2.02E+08	2.02E+08	224	-1.02	0.000995	0.00761	CASP8	2.02E+08
chr2	2.02E+08	2.02E+08	224	-1.02	0.000995	0.00761	CASP8	2.02E+08
chr2	2.02E+08	2.02E+08	224	-1.02	0.000995	0.00761	CASP8	2.02E+08
chr2	2.02E+08	2.02E+08	224	-1.02	0.000995	0.00761	CASP8	2.02E+08
chr19	40931542	40932076	535	-0.65	0.000996	0.00762	SERTAD1	40929932
chr6	53659055	53659835	781	-0.61	0.000997	0.00762	LRRC1	53654777
chrX	1.06E+08	1.06E+08	922	-0.69	0.001	0.00763	CLDN2	1.06E+08
chr5	89769840	89770797	958	-0.51	0.001	0.00763	POLR3G	89765680
chr5	89769840	89770797	958	-0.51	0.001	0.00763	MBLAC2	89768585
chr20	45946078	45946486	409	-0.65	0.001	0.00764	LOC100131	45942245
chr6	76458355	76459342	988	-0.68	0.001	0.00765	MYO6	76453908
chr6	76458355	76459342	988	-0.68	0.001	0.00765	MYO6	76453892
chr12	30948945	30949553	609	-0.59	0.001	0.00766	LINC00941	30943614
chr14	50320365	50320766	402	-1.18	0.00101	0.00767	NEMF	50317791
chr14	50320365	50320766	402	-1.18	0.00101	0.00767	NEMF	50317791
chr10	1.15E+08	1.15E+08	354	-0.91	0.00101	0.00768	SNORA87	1.15E+08

chr10	1.06E+08	1.06E+08	568	-0.69	0.00101	0.00768	LOC101927	1.06E+08
chrX	1.03E+08	1.03E+08	303	-1.26	0.00101	0.00768	BEX2	1.03E+08
chrX	1.03E+08	1.03E+08	303	-1.26	0.00101	0.00768	BEX2	1.03E+08
chrX	1.03E+08	1.03E+08	303	-1.26	0.00101	0.00768	BEX2	1.03E+08
chrX	1.03E+08	1.03E+08	303	-1.26	0.00101	0.00768	BEX2	1.03E+08
chr10	15001270	15002120	851	-0.65	0.00101	0.00769	DCLRE1C	14994431
chr10	15001270	15002120	851	-0.65	0.00101	0.00769	DCLRE1C	14994431
chr10	15001270	15002120	851	-0.65	0.00101	0.00769	DCLRE1C	14994431
chr10	15001270	15002120	851	-0.65	0.00101	0.00769	MEIG1	14996437
chr10	15001270	15002120	851	-0.65	0.00101	0.00769	DCLRE1C	14994431
chr10	15001270	15002120	851	-0.65	0.00101	0.00769	DCLRE1C	14994431
chr10	15001270	15002120	851	-0.65	0.00101	0.00769	DCLRE1C	14994431
chr10	15001270	15002120	851	-0.65	0.00101	0.00769	DCLRE1C	14994431
chr10	15001270	15002120	851	-0.65	0.00101	0.00769	DCLRE1C	14994431
chr10	15001270	15002120	851	-0.65	0.00101	0.00769	DCLRE1C	14994431
chr7	96635383	96635735	353	-1.07	0.00101	0.00769	DLX6	96630289
chr14	75469205	75469673	469	-0.48	0.00102	0.00771	EIF2B2	75464611
chr14	53258005	53258811	807	-0.48	0.00102	0.00771	GNPNAT1	53256386
chr11	1.19E+08	1.19E+08	461	-0.63	0.00102	0.00772	HINFP	1.19E+08
chr11	1.19E+08	1.19E+08	461	-0.63	0.00102	0.00772	HINFP	1.19E+08
chr11	1.19E+08	1.19E+08	461	-0.63	0.00102	0.00772	HINFP	1.19E+08
chr11	1.2E+08	1.2E+08	238	-1.17	0.00102	0.00772	GRIK4	1.2E+08
chr11	1.2E+08	1.2E+08	238	-1.17	0.00102	0.00772	GRIK4	1.2E+08
chrX	1.06E+08	1.06E+08	336	-0.95	0.00102	0.00772	RNF128	1.06E+08
chr18	14944049	14944269	221	-1.27	0.00102	0.00772	LINC01443	14941265
chr1	2.47E+08	2.47E+08	622	-0.55	0.00102	0.00772	ZNF669	2.47E+08
chr1	2.47E+08	2.47E+08	622	-0.55	0.00102	0.00772	ZNF669	2.47E+08
chr8	9413175	9413585	411	-0.83	0.00102	0.00772	TNKS	9408444
chr7	1.23E+08	1.23E+08	523	-0.71	0.00102	0.00773	NDUFA5	1.23E+08
chr7	1.23E+08	1.23E+08	523	-0.71	0.00102	0.00773	NDUFA5	1.23E+08
chr7	1.23E+08	1.23E+08	523	-0.71	0.00102	0.00773	NDUFA5	1.23E+08
chr7	1.23E+08	1.23E+08	523	-0.71	0.00102	0.00773	NDUFA5	1.23E+08
chr7	1.23E+08	1.23E+08	523	-0.71	0.00102	0.00773	NDUFA5	1.23E+08
chr7	1.23E+08	1.23E+08	523	-0.71	0.00102	0.00773	NDUFA5	1.23E+08
chr7	1.23E+08	1.23E+08	523	-0.71	0.00102	0.00773	NDUFA5	1.23E+08
chr7	1.23E+08	1.23E+08	523	-0.71	0.00102	0.00773	NDUFA5	1.23E+08
chrX	15755878	15756493	616	-0.57	0.00102	0.00773	CA5B	15751411
chr17	26368135	26368860	726	-0.54	0.00102	0.00774	NLK	26364687
chr17	13504959	13505745	787	-0.76	0.00103	0.00776	HS3ST3A1	13503259
chr1	1.66E+08	1.66E+08	970	-0.53	0.00103	0.00779	LOC440700	1.66E+08
chr1	1.66E+08	1.66E+08	970	-0.53	0.00103	0.00779	ALDH9A1	1.66E+08
chr8	1.42E+08	1.42E+08	853	-0.63	0.00103	0.00779	SLC45A4	1.42E+08
chr8	1.42E+08	1.42E+08	853	-0.63	0.00103	0.00779	SLC45A4	1.42E+08
chr3	1.57E+08	1.57E+08	668	-0.55	0.00103	0.00779	PA2G4P4	1.57E+08
chr11	1.08E+08	1.08E+08	1047	-0.64	0.00104	0.00781	KDELC2	1.08E+08
chr12	41084233	41084479	247	-1.07	0.00104	0.00782	CNTN1	41081243
chr12	41084233	41084479	247	-1.07	0.00104	0.00782	CNTN1	41081243

chr3	1.11E+08	1.11E+08	739	-0.57	0.00104	0.00782	PVRL3	1.11E+08
chr3	1.11E+08	1.11E+08	739	-0.57	0.00104	0.00782	PVRL3	1.11E+08
chr3	1.11E+08	1.11E+08	739	-0.57	0.00104	0.00782	PVRL3	1.11E+08
chr3	1.11E+08	1.11E+08	739	-0.57	0.00104	0.00782	PVRL3-AS1	1.11E+08
chr6	52537273	52537537	265	-0.9	0.00104	0.00783	TMEM14A	52530883
chr18	12990983	12991780	798	-0.46	0.00104	0.00784	CEP192	12986360
chr6	45345213	45346149	937	-0.55	0.00104	0.00784	SUPT3H	45343788
chr6	45345213	45346149	937	-0.55	0.00104	0.00784	SUPT3H	45343788
chr6	45345213	45346149	937	-0.55	0.00104	0.00784	SUPT3H	45343788
chr13	53424777	53425472	696	-0.8	0.00104	0.00784	PCDH8	53420775
chr13	53424777	53425472	696	-0.8	0.00104	0.00784	PCDH8	53420775
chr7	96634628	96634899	272	-0.98	0.00105	0.00785	DLX6	96630289
chr15	91478244	91478838	595	-0.63	0.00105	0.00785	UNC45A	91473214
chr15	91478244	91478838	595	-0.63	0.00105	0.00785	HDDC3	91473799
chr15	91478244	91478838	595	-0.63	0.00105	0.00785	HDDC3	91473799
chr15	91478244	91478838	595	-0.63	0.00105	0.00785	HDDC3	91473799
chr5	1.31E+08	1.31E+08	809	-0.48	0.00105	0.00786	RAPGEF6	1.31E+08
chr5	1.31E+08	1.31E+08	809	-0.48	0.00105	0.00786	RAPGEF6	1.31E+08
chr5	1.31E+08	1.31E+08	809	-0.48	0.00105	0.00786	RAPGEF6	1.31E+08
chr5	1.31E+08	1.31E+08	809	-0.48	0.00105	0.00786	RAPGEF6	1.31E+08
chr5	1.31E+08	1.31E+08	809	-0.48	0.00105	0.00786	RAPGEF6	1.31E+08
chr5	1.31E+08	1.31E+08	809	-0.48	0.00105	0.00786	RAPGEF6	1.31E+08
chr10	1.05E+08	1.05E+08	390	-0.75	0.00105	0.00788	INA	1.05E+08
chr11	1.26E+08	1.26E+08	301	-0.72	0.00105	0.00789	ST3GAL4	1.26E+08
chr11	1.26E+08	1.26E+08	301	-0.72	0.00105	0.00789	ST3GAL4	1.26E+08
chr4	68751529	68751871	343	-0.78	0.00105	0.00789	TMPRSS11I	68747716
chr9	1.31E+08	1.31E+08	483	-0.54	0.00105	0.00789	SH2D3C	1.31E+08
chr9	1.31E+08	1.31E+08	483	-0.54	0.00105	0.00789	SH2D3C	1.31E+08
chr1	70820135	70820581	447	-0.94	0.00105	0.0079	ANKRD13C	70818417
chr1	70820135	70820581	447	-0.94	0.00105	0.0079	HHLA3	70815492
chr1	70820135	70820581	447	-0.94	0.00105	0.0079	HHLA3	70815492
chr1	70820135	70820581	447	-0.94	0.00105	0.0079	HHLA3	70815492
chr1	70820135	70820581	447	-0.94	0.00105	0.0079	HHLA3	70815492
chr2	1.78E+08	1.78E+08	527	-0.61	0.00106	0.0079	NFE2L2	1.78E+08
chr2	1.78E+08	1.78E+08	527	-0.61	0.00106	0.0079	NFE2L2	1.78E+08
chr2	1.78E+08	1.78E+08	527	-0.61	0.00106	0.0079	NFE2L2	1.78E+08
chr2	1.78E+08	1.78E+08	527	-0.61	0.00106	0.0079	NFE2L2	1.78E+08
chr2	1.78E+08	1.78E+08	527	-0.61	0.00106	0.0079	NFE2L2	1.78E+08
chr2	1.78E+08	1.78E+08	527	-0.61	0.00106	0.0079	NFE2L2	1.78E+08
chr2	1.78E+08	1.78E+08	527	-0.61	0.00106	0.0079	NFE2L2	1.78E+08
chr2	1.78E+08	1.78E+08	527	-0.61	0.00106	0.0079	NFE2L2	1.78E+08
chr1	36851491	36852634	1144	-0.45	0.00106	0.0079	STK40	36849528
chr1	36851491	36852634	1144	-0.45	0.00106	0.0079	STK40	36849528
chr1	36851491	36852634	1144	-0.45	0.00106	0.0079	STK40	36849528
chr16	89382561	89382939	379	-0.81	0.00106	0.00791	LOC100287	89382540
chr5	93446978	93447598	621	-0.47	0.00106	0.00794	FAM172A	93445404
chr5	93446978	93447598	621	-0.47	0.00106	0.00794	FAM172A	93445404

chr5	93446978	93447598	621	-0.47	0.00106	0.00794	FAM172A	93445404
chr5	93446978	93447598	621	-0.47	0.00106	0.00794	FAM172A	93445404
chr9	1.03E+08	1.03E+08	773	-0.46	0.00106	0.00794	MSANTD3	1.03E+08
chr9	1.03E+08	1.03E+08	773	-0.46	0.00106	0.00794	MSANTD3	1.03E+08
chr9	1.03E+08	1.03E+08	773	-0.46	0.00106	0.00794	MSANTD3	1.03E+08
chr9	1.03E+08	1.03E+08	773	-0.46	0.00106	0.00794	MSANTD3	1.03E+08
chr8	38239755	38240447	693	-0.52	0.00107	0.00795	WHSC1L1	38237790
chr8	38239755	38240447	693	-0.52	0.00107	0.00795	LETM2	38238958
chr8	38239755	38240447	693	-0.52	0.00107	0.00795	LETM2	38238958
chr8	38239755	38240447	693	-0.52	0.00107	0.00795	LETM2	38238958
chr8	38239755	38240447	693	-0.52	0.00107	0.00795	LETM2	38239648
chr8	38239755	38240447	693	-0.52	0.00107	0.00795	LETM2	38238958
chr8	38239755	38240447	693	-0.52	0.00107	0.00795	WHSC1L1	38237790
chr8	38239755	38240447	693	-0.52	0.00107	0.00795	LETM2	38238958
chr9	1.04E+08	1.04E+08	1210	-0.43	0.00107	0.00797	TMEM246	1.04E+08
chr9	1.04E+08	1.04E+08	1210	-0.43	0.00107	0.00797	TMEM246	1.04E+08
chr9	1.04E+08	1.04E+08	1210	-0.43	0.00107	0.00797	TMEM246	1.04E+08
chr4	48782172	48782779	608	-0.61	0.00107	0.00797	FRYL	48780316
chr1	12678970	12679469	500	-0.55	0.00107	0.00797	DHRS3	12675737
chr3	17783541	17784537	997	-0.61	0.00107	0.00798	TBC1D5	17780399
chr3	1.94E+08	1.94E+08	751	-0.6	0.00107	0.00799	LINC00887	1.94E+08
chr9	96215813	96216169	357	-0.73	0.00107	0.00799	FAM120A	96208977
chr9	96215813	96216169	357	-0.73	0.00107	0.00799	FAM120A	96208977
chr9	96215813	96216169	357	-0.73	0.00107	0.00799	FAM120A	96208977
chr9	96215813	96216169	357	-0.73	0.00107	0.00799	FAM120A	96208977
chr9	96215813	96216169	357	-0.73	0.00107	0.00799	FAM120AC	96213874
chr12	4429931	4430502	572	-0.6	0.00107	0.00799	TIGAR	4425358
chr19	12721517	12721875	359	-0.72	0.00107	0.008	ZNF490	12719623
chr19	12721517	12721875	359	-0.72	0.00107	0.008	ZNF791	12716731
chr17	79818456	79819001	546	-0.55	0.00108	0.00802	P4HB	79816544
chr9	1.26E+08	1.26E+08	695	-0.52	0.00108	0.00803	STRBP	1.26E+08
chr9	1.26E+08	1.26E+08	695	-0.52	0.00108	0.00803	STRBP	1.26E+08
chr9	1.26E+08	1.26E+08	695	-0.52	0.00108	0.00803	STRBP	1.26E+08
chr1	54665653	54665967	315	-0.89	0.00108	0.00803	CYB5RL	54663746
chr1	54665653	54665967	315	-0.89	0.00108	0.00803	MRPL37	54660839
chr16	28961914	28962335	422	-0.56	0.00108	0.00805	NFATC2IP	28957317
chr8	60032148	60032347	200	-1.16	0.00109	0.00805	TOX	60029767
chr11	48118755	48118969	215	-1.26	0.00109	0.00808	MIR3161	48113333
chr12	1.01E+08	1.01E+08	377	-0.67	0.00109	0.00808	ACTR6	1.01E+08
chr12	1.01E+08	1.01E+08	377	-0.67	0.00109	0.00808	ACTR6	1.01E+08
chr12	1.01E+08	1.01E+08	377	-0.67	0.00109	0.00808	ACTR6	1.01E+08
chr19	14247391	14248304	914	-0.5	0.00109	0.00808	ASF1B	14245440
chr19	14247391	14248304	914	-0.5	0.00109	0.00808	LOC100507	14242963
chr19	13905916	13906250	335	-0.79	0.00109	0.00808	ZSWIM4	13901273
chr6	1.23E+08	1.23E+08	872	-0.55	0.00109	0.00809	PKIB	1.23E+08
chr6	1.23E+08	1.23E+08	872	-0.55	0.00109	0.00809	PKIB	1.23E+08
chrX	15353553	15353969	417	-0.55	0.00109	0.00809	PIGA	15351676

chrX	15353553	15353969	417	-0.55	0.00109	0.00809	PIGA	15351676
chrX	15353553	15353969	417	-0.55	0.00109	0.00809	PIGA	15351676
chrX	15353553	15353969	417	-0.55	0.00109	0.00809	PIGA	15351676
chr11	1.05E+08	1.05E+08	521	-0.66	0.00109	0.0081	CARD16	1.05E+08
chr11	1.05E+08	1.05E+08	521	-0.66	0.00109	0.0081	CARD16	1.05E+08
chr8	1.42E+08	1.42E+08	589	-0.5	0.0011	0.00811	AGO2	1.42E+08
chr8	1.42E+08	1.42E+08	589	-0.5	0.0011	0.00811	AGO2	1.42E+08
chr1	63249364	63249996	633	-0.69	0.0011	0.00811	ATG4C	63244776
chr1	63249364	63249996	633	-0.69	0.0011	0.00811	ATG4C	63244776
chr11	6411119	6411857	739	-0.45	0.0011	0.00812	SMPD1	6406643
chr11	6411119	6411857	739	-0.45	0.0011	0.00812	SMPD1	6406643
chr11	6411119	6411857	739	-0.45	0.0011	0.00812	SMPD1	6406643
chr11	6411119	6411857	739	-0.45	0.0011	0.00812	SMPD1	6406643
chr11	6411119	6411857	739	-0.45	0.0011	0.00812	SMPD1	6406643
chr11	6411119	6411857	739	-0.45	0.0011	0.00812	SMPD1	6406643
chr19	8578562	8579220	659	-0.78	0.0011	0.00814	ZNF414	8577048
chr19	8578562	8579220	659	-0.78	0.0011	0.00814	ZNF414	8577048
chr3	1.53E+08	1.53E+08	1007	-0.64	0.0011	0.00814	P2RY1	1.53E+08
chr9	77112057	77112434	378	-0.7	0.0011	0.00814	RORB	77107251
chr9	77112057	77112434	378	-0.7	0.0011	0.00814	RORB-AS1	77111469
chr11	57434822	57435762	941	-0.41	0.00111	0.00815	ZDHHC5	57430473
chr5	88175739	88175887	149	-1.56	0.00111	0.00816	MEF2C-AS1	88174146
chr15	75746304	75746842	539	-0.59	0.00111	0.00816	SIN3A	75742087
chr15	75746304	75746842	539	-0.59	0.00111	0.00816	SIN3A	75746124
chr15	75746304	75746842	539	-0.59	0.00111	0.00816	SIN3A	75741926
chr2	24299191	24300123	933	-0.47	0.00111	0.00817	FAM228B	24294743
chr2	24299191	24300123	933	-0.47	0.00111	0.00817	FAM228B	24294743
chr2	24299191	24300123	933	-0.47	0.00111	0.00817	SF3B6	24297314
chr7	36429058	36429478	421	-0.58	0.00111	0.00817	ANLN	36424411
chr7	36429058	36429478	421	-0.58	0.00111	0.00817	ANLN	36424411
chr7	36429058	36429478	421	-0.58	0.00111	0.00817	ANLN	36424411
chr7	36429058	36429478	421	-0.58	0.00111	0.00817	KIAA0895	36427734
chr22	19132089	19132398	310	-0.91	0.00112	0.00819	DGCR14	19130190
chr22	19132089	19132398	310	-0.91	0.00112	0.00819	DGCR14	19130190
chr11	31391136	31391710	575	-0.57	0.00112	0.00819	DCDC1	31389357
chr11	31391136	31391710	575	-0.57	0.00112	0.00819	DNAJC24	31386376
chr6	1.38E+08	1.38E+08	552	-0.72	0.00112	0.00822	IFNGR1	1.38E+08
chr1	1.08E+08	1.08E+08	187	-0.86	0.00112	0.00823	VAV3	1.08E+08
chr14	20937375	20938531	1157	-0.52	0.00112	0.00823	PNP	20932537
chr7	44121940	44122604	665	-0.5	0.00112	0.00823	POLM	44120139
chr7	44121940	44122604	665	-0.5	0.00112	0.00823	POLM	44120139
chr7	44121940	44122604	665	-0.5	0.00112	0.00823	POLM	44120139
chr7	44121940	44122604	665	-0.5	0.00112	0.00823	POLM	44120139
chr1	1.6E+08	1.6E+08	783	-0.46	0.00113	0.00826	IGSF9	1.6E+08
chr1	1.6E+08	1.6E+08	783	-0.46	0.00113	0.00826	IGSF9	1.6E+08
chr1	65775108	65775769	662	-1.06	0.00113	0.00829	DNAJC6	65770217
chr9	1.31E+08	1.31E+08	422	-0.54	0.00113	0.0083	ST6GALNA(	1.31E+08

chr9	1.31E+08	1.31E+08	422	-0.54	0.00113	0.0083	ST6GALNA(	1.31E+08
chr15	89455133	89455333	201	-1.14	0.00114	0.0083	MFGE8	89454700
chr15	89455133	89455333	201	-1.14	0.00114	0.0083	MFGE8	89453993
chr15	89455133	89455333	201	-1.14	0.00114	0.0083	MFGE8	89454700
chr15	89455133	89455333	201	-1.14	0.00114	0.0083	MFGE8	89454700
chr15	89455133	89455333	201	-1.14	0.00114	0.0083	MFGE8	89454700
chr19	42746767	42747478	712	-0.55	0.00114	0.00834	GSK3A	42744736
chr1	16485543	16485855	313	-0.84	0.00114	0.00835	EPHA2	16480582
chr8	1.25E+08	1.25E+08	829	-0.42	0.00115	0.00837	TMEM65	1.25E+08
chr20	48770221	48770524	304	-0.81	0.00115	0.00838	TMEM189	48768335
chr20	48770221	48770524	304	-0.81	0.00115	0.00838	TMEM189	48768335
chr20	48770221	48770524	304	-0.81	0.00115	0.00838	TMEM189-	48768335
chr20	48770221	48770524	304	-0.81	0.00115	0.00838	TMEM189	48768335
chr19	49541969	49542152	184	-1.68	0.00115	0.00839	SNAR-G1	49535276
chr19	49541969	49542152	184	-1.68	0.00115	0.00839	CGB1	49538191
chr19	49541969	49542152	184	-1.68	0.00115	0.00839	CGB5	49542101
chr2	1.14E+08	1.14E+08	933	-0.77	0.00116	0.00841	IL36G	1.14E+08
chr2	1.14E+08	1.14E+08	933	-0.77	0.00116	0.00841	IL36G	1.14E+08
chr11	830270	830500	231	-0.85	0.00116	0.00841	CD151	827951
chr11	830270	830500	231	-0.85	0.00116	0.00841	CD151	827951
chr11	830270	830500	231	-0.85	0.00116	0.00841	CD151	827951
chr11	830270	830500	231	-0.85	0.00116	0.00841	CD151	827951
chr5	1.54E+08	1.54E+08	1193	-0.5	0.00116	0.00841	CNOT8	1.54E+08
chr5	1.54E+08	1.54E+08	1193	-0.5	0.00116	0.00841	CNOT8	1.54E+08
chr5	1.54E+08	1.54E+08	1193	-0.5	0.00116	0.00841	CNOT8	1.54E+08
chr5	1.54E+08	1.54E+08	1193	-0.5	0.00116	0.00841	CNOT8	1.54E+08
chr5	1.54E+08	1.54E+08	1193	-0.5	0.00116	0.00841	CNOT8	1.54E+08
chr5	1.54E+08	1.54E+08	1193	-0.5	0.00116	0.00841	CNOT8	1.54E+08
chr5	1.54E+08	1.54E+08	1193	-0.5	0.00116	0.00841	CNOT8	1.54E+08
chr5	1.54E+08	1.54E+08	1193	-0.5	0.00116	0.00841	CNOT8	1.54E+08
chr5	1.54E+08	1.54E+08	1193	-0.5	0.00116	0.00841	CNOT8	1.54E+08
chr12	6862167	6862986	820	-0.57	0.00116	0.00843	MLF2	6860636
chr12	6862167	6862986	820	-0.57	0.00116	0.00843	MLF2	6860082
chr14	64854219	64855167	949	-0.49	0.00116	0.00843	MTHFD1	64849758
chr20	5100028	5100965	938	-0.49	0.00116	0.00843	PCNA-AS1	5095231
chr20	5100028	5100965	938	-0.49	0.00116	0.00843	PCNA	5098647
chr10	1.06E+08	1.06E+08	174	-0.94	0.00117	0.00846	ITPRIP	1.06E+08
chr11	62521332	62521968	637	-0.54	0.00117	0.00847	ZBTB3	62519656
chr12	39836238	39837420	1183	-0.44	0.00117	0.00849	KIF21A	39835192
chr12	39836238	39837420	1183	-0.44	0.00117	0.00849	KIF21A	39835192
chr12	39836238	39837420	1183	-0.44	0.00117	0.00849	KIF21A	39835192
chr12	39836238	39837420	1183	-0.44	0.00117	0.00849	KIF21A	39835192
chr6	96969419	96970191	773	-0.48	0.00117	0.0085	UFL1	96964701
chr8	1.2E+08	1.2E+08	214	-0.87	0.00118	0.00851	MAL2	1.2E+08
chr16	67693851	67694887	1037	-0.66	0.00118	0.00851	PARD6A	67689850
chr16	67693851	67694887	1037	-0.66	0.00118	0.00851	ACD	67692718
chr16	67693851	67694887	1037	-0.66	0.00118	0.00851	ACD	67692718

chr16	67693851	67694887	1037	-0.66	0.00118	0.00851	ACD	67692718
chr16	67693851	67694887	1037	-0.66	0.00118	0.00851	PARD6A	67689850
chr2	48758263	48758840	578	-0.53	0.00118	0.00852	STON1	48752307
chr2	48758263	48758840	578	-0.53	0.00118	0.00852	STON1	48752307
chr2	48758263	48758840	578	-0.53	0.00118	0.00852	STON1-GTF	48752063
chr16	22385769	22386349	581	-0.57	0.00118	0.00852	CDR2	22383938
chr16	74325591	74326089	499	-0.64	0.00118	0.00854	PSMD7	74325672
chr1	2.08E+08	2.08E+08	619	-0.63	0.00119	0.00856	CD46	2.08E+08
chr1	2.08E+08	2.08E+08	619	-0.63	0.00119	0.00856	CD46	2.08E+08
chr1	2.08E+08	2.08E+08	619	-0.63	0.00119	0.00856	CD46	2.08E+08
chr1	2.08E+08	2.08E+08	619	-0.63	0.00119	0.00856	CD46	2.08E+08
chr1	2.08E+08	2.08E+08	619	-0.63	0.00119	0.00856	CD46	2.08E+08
chr1	2.08E+08	2.08E+08	619	-0.63	0.00119	0.00856	CD46	2.08E+08
chr1	2.08E+08	2.08E+08	619	-0.63	0.00119	0.00856	CD46	2.08E+08
chr1	2.08E+08	2.08E+08	619	-0.63	0.00119	0.00856	CD46	2.08E+08
chr13	1.01E+08	1.01E+08	453	-0.54	0.00119	0.00856	TMTC4	1.01E+08
chr13	1.01E+08	1.01E+08	453	-0.54	0.00119	0.00856	TMTC4	1.01E+08
chr13	1.01E+08	1.01E+08	453	-0.54	0.00119	0.00856	TMTC4	1.01E+08
chr17	8090105	8090594	490	-0.61	0.00119	0.00858	MIR4521	8085262
chr10	1.12E+08	1.12E+08	635	-0.55	0.00119	0.0086	MXI1	1.12E+08
chr6	1.37E+08	1.37E+08	916	-0.52	0.0012	0.00862	MAP3K5	1.37E+08
chr10	1.19E+08	1.19E+08	564	-0.84	0.0012	0.00862	ENO4	1.19E+08
chr16	25042832	25043244	413	-0.6	0.0012	0.00863	LOC554206	25038061
chr14	39583302	39583754	453	-0.7	0.0012	0.00863	GEMIN2	39578487
chr14	39583302	39583754	453	-0.7	0.0012	0.00863	GEMIN2	39578487
chr14	39583302	39583754	453	-0.7	0.0012	0.00863	GEMIN2	39578487
chr11	1.18E+08	1.18E+08	344	-0.66	0.0012	0.00865	TMPRSS4	1.18E+08
chr11	1.18E+08	1.18E+08	344	-0.66	0.0012	0.00865	TMPRSS4	1.18E+08
chr11	1.18E+08	1.18E+08	344	-0.66	0.0012	0.00865	TMPRSS4	1.18E+08
chr11	1.18E+08	1.18E+08	344	-0.66	0.0012	0.00865	TMPRSS4	1.18E+08
chr11	1.18E+08	1.18E+08	344	-0.66	0.0012	0.00865	TMPRSS4	1.18E+08
chr11	1.18E+08	1.18E+08	344	-0.66	0.0012	0.00865	TMPRSS4	1.18E+08
chr11	1.18E+08	1.18E+08	344	-0.66	0.0012	0.00865	TMPRSS4	1.18E+08
chr3	30647793	30648175	383	-0.66	0.0012	0.00865	TGFBR2	30642993
chr3	30647793	30648175	383	-0.66	0.0012	0.00865	TGFBR2	30642993
chr11	9482207	9483053	847	-0.47	0.00121	0.00869	ZNF143	9477511
chr11	9482207	9483053	847	-0.47	0.00121	0.00869	ZNF143	9477511
chr11	9482207	9483053	847	-0.47	0.00121	0.00869	LOC644656	9480245
chr11	9482207	9483053	847	-0.47	0.00121	0.00869	ZNF143	9477511
chr8	38756611	38756830	220	-1.03	0.00121	0.00869	PLEKHA2	38753752
chr19	50145129	50145439	311	-0.69	0.00121	0.00869	SCAF1	50140381
chr19	50145129	50145439	311	-0.69	0.00121	0.00869	RRAS	50141400
chr5	1.11E+08	1.11E+08	629	-0.64	0.00122	0.00871	STARD4-AS	1.11E+08
chr5	1.11E+08	1.11E+08	629	-0.64	0.00122	0.00871	STARD4	1.11E+08
chr5	1.11E+08	1.11E+08	629	-0.64	0.00122	0.00871	STARD4	1.11E+08
chr5	1.11E+08	1.11E+08	629	-0.64	0.00122	0.00871	STARD4	1.11E+08
chr5	1.11E+08	1.11E+08	629	-0.64	0.00122	0.00871	STARD4	1.11E+08

chr5	1.11E+08	1.11E+08	629	-0.64	0.00122	0.00871	STARD4	1.11E+08
chr5	1.11E+08	1.11E+08	629	-0.64	0.00122	0.00871	STARD4	1.11E+08
chr5	1.11E+08	1.11E+08	629	-0.64	0.00122	0.00871	STARD4	1.11E+08
chr5	1.11E+08	1.11E+08	629	-0.64	0.00122	0.00871	STARD4	1.11E+08
chr5	1.11E+08	1.11E+08	629	-0.64	0.00122	0.00871	STARD4	1.11E+08
chr5	1.11E+08	1.11E+08	629	-0.64	0.00122	0.00871	STARD4	1.11E+08
chr1	1.1E+08	1.1E+08	526	-0.56	0.00123	0.00876	CYB561D1	1.1E+08
chr1	1.1E+08	1.1E+08	526	-0.56	0.00123	0.00876	CYB561D1	1.1E+08
chr1	1.1E+08	1.1E+08	526	-0.56	0.00123	0.00876	CYB561D1	1.1E+08
chr1	1.1E+08	1.1E+08	526	-0.56	0.00123	0.00876	CYB561D1	1.1E+08
chr1	1.1E+08	1.1E+08	526	-0.56	0.00123	0.00876	CYB561D1	1.1E+08
chr11	1.14E+08	1.14E+08	968	-0.45	0.00123	0.00876	REXO2	1.14E+08
chr2	2.34E+08	2.34E+08	274	-0.81	0.00123	0.00877	ATG16L1	2.34E+08
chr2	2.34E+08	2.34E+08	274	-0.81	0.00123	0.00877	ATG16L1	2.34E+08
chr2	2.34E+08	2.34E+08	274	-0.81	0.00123	0.00877	ATG16L1	2.34E+08
chr2	2.34E+08	2.34E+08	274	-0.81	0.00123	0.00877	ATG16L1	2.34E+08
chr2	2.34E+08	2.34E+08	274	-0.81	0.00123	0.00877	ATG16L1	2.34E+08
chr14	94461345	94461932	588	-0.74	0.00123	0.00877	LINC00521	94458641
chr14	94461345	94461932	588	-0.74	0.00123	0.00877	LINC00521	94458641
chr14	94461345	94461932	588	-0.74	0.00123	0.00877	LINC00521	94458615
chr6	70506605	70507184	580	-0.48	0.00123	0.00877	LMBRD1	70505049
chr1	2.31E+08	2.31E+08	665	-0.66	0.00123	0.00878	EXOC8	2.31E+08
chr1	2.31E+08	2.31E+08	665	-0.66	0.00123	0.00878	SPRTN	2.31E+08
chr1	2.31E+08	2.31E+08	665	-0.66	0.00123	0.00878	SPRTN	2.31E+08
chr1	2.31E+08	2.31E+08	665	-0.66	0.00123	0.00878	SPRTN	2.31E+08
chr13	96296403	96297126	724	-0.66	0.00123	0.0088	DZIP1	96294960
chr13	96296403	96297126	724	-0.66	0.00123	0.0088	DZIP1	96294960
chr19	50979380	50980149	770	-0.68	0.00124	0.00881	FAM71E1	50978003
chr19	50979380	50980149	770	-0.68	0.00124	0.00881	FAM71E1	50978003
chr19	50979380	50980149	770	-0.68	0.00124	0.00881	EMC10	50974733
chr19	50979380	50980149	770	-0.68	0.00124	0.00881	EMC10	50974733
chr3	1.8E+08	1.8E+08	492	-0.86	0.00124	0.00884	LOC10050	1.8E+08
chr10	1.08E+08	1.08E+08	300	-0.67	0.00124	0.00884	LOC10537	1.08E+08
chr10	1.08E+08	1.08E+08	300	-0.67	0.00124	0.00884	LOC10537	1.08E+08
chr10	1.08E+08	1.08E+08	300	-0.67	0.00124	0.00884	LOC10537	1.08E+08
chr22	38092812	38093067	256	-1.03	0.00124	0.00885	TRIOBP	38087994
chr11	43380256	43380845	590	-0.55	0.00125	0.00887	TTC17	43375434
chr11	43380256	43380845	590	-0.55	0.00125	0.00887	TTC17	43375434
chr9	1.15E+08	1.15E+08	679	-0.45	0.00125	0.00888	HSDL2	1.15E+08
chr9	1.15E+08	1.15E+08	679	-0.45	0.00125	0.00888	HSDL2	1.15E+08
chr9	1.15E+08	1.15E+08	679	-0.45	0.00125	0.00888	HSDL2	1.15E+08
chr1	89989967	89990963	997	-0.45	0.00125	0.00889	LRRC8B	89985396
chr11	82612459	82612870	412	-0.72	0.00125	0.00891	PRCP	82609557
chr11	82612459	82612870	412	-0.72	0.00125	0.00891	PRCP	82609557
chr11	82612459	82612870	412	-0.72	0.00125	0.00891	DDIAS	82607736
chr14	32545621	32546592	972	-0.43	0.00126	0.00893	ARHGAP5	32541494
chr14	32545621	32546592	972	-0.43	0.00126	0.00893	ARHGAP5	32541494

chr14	32545621	32546592	972	-0.43	0.00126	0.00893	ARHGAP5-/	32543905
chr19	41256436	41257055	620	-0.62	0.00126	0.00893	SNRPA	41251758
chr19	41256436	41257055	620	-0.62	0.00126	0.00893	C19orf54	41253828
chr3	1.81E+08	1.81E+08	362	-0.68	0.00126	0.00895	SOX2	1.81E+08
chr11	64902124	64902760	637	-0.66	0.00126	0.00895	SYVN1	64900003
chr11	64902124	64902760	637	-0.66	0.00126	0.00895	SYVN1	64900003
chr11	64902124	64902760	637	-0.66	0.00126	0.00895	MIR6751	64895450
chr8	82754005	82755062	1058	-0.56	0.00127	0.00896	SNX16	82752521
chr8	82754005	82755062	1058	-0.56	0.00127	0.00896	SNX16	82752521
chr8	82754005	82755062	1058	-0.56	0.00127	0.00896	SNX16	82752521
chr3	16306227	16306841	615	-0.76	0.00127	0.00896	DPH3	16304496
chr3	16306227	16306841	615	-0.76	0.00127	0.00896	DPH3	16304496
chr3	16306227	16306841	615	-0.76	0.00127	0.00896	OXNAD1	16301666
chr3	1.12E+08	1.12E+08	587	-0.6	0.00127	0.00896	C3orf52	1.12E+08
chr3	1.12E+08	1.12E+08	587	-0.6	0.00127	0.00896	C3orf52	1.12E+08
chr16	30381742	30382543	802	-0.43	0.00127	0.00897	TBC1D10B	30379522
chr16	30381742	30382543	802	-0.43	0.00127	0.00897	MYLPF	30381122
chr1	1.65E+08	1.65E+08	379	-0.53	0.00127	0.00897	LOC10050!	1.65E+08
chr6	52929561	52930194	634	-0.63	0.00127	0.00897	FBXO9	52925256
chr6	52929561	52930194	634	-0.63	0.00127	0.00897	FBXO9	52924795
chr6	52929561	52930194	634	-0.63	0.00127	0.00897	ICK	52924600
chr6	52929561	52930194	634	-0.63	0.00127	0.00897	ICK	52924600
chr9	1.28E+08	1.28E+08	539	-0.6	0.00127	0.00897	WDR38	1.28E+08
chr9	1.28E+08	1.28E+08	539	-0.6	0.00127	0.00897	WDR38	1.28E+08
chr9	1.28E+08	1.28E+08	539	-0.6	0.00127	0.00897	WDR38	1.28E+08
chr9	1.28E+08	1.28E+08	539	-0.6	0.00127	0.00897	WDR38	1.28E+08
chr8	1.44E+08	1.44E+08	152	-1	0.00127	0.00898	ARC	1.44E+08
chr11	66725711	66726381	671	-0.5	0.00127	0.00898	PC	66723847
chr11	66725711	66726381	671	-0.5	0.00127	0.00898	PC	66723847
chr12	1.07E+08	1.07E+08	688	-0.46	0.00127	0.00899	LOC10028/	1.07E+08
chr12	1.07E+08	1.07E+08	688	-0.46	0.00127	0.00899	RIC8B	1.07E+08
chr2	1.53E+08	1.53E+08	647	-0.59	0.00127	0.009	ARL5A	1.53E+08
chr2	1.53E+08	1.53E+08	647	-0.59	0.00127	0.009	ARL5A	1.53E+08
chr2	1.53E+08	1.53E+08	647	-0.59	0.00127	0.009	ARL5A	1.53E+08
chr11	8985609	8986649	1041	-0.52	0.00128	0.009	TMEM9B	8984558
chr11	8985609	8986649	1041	-0.52	0.00128	0.009	TMEM9B	8984320
chr11	8985609	8986649	1041	-0.52	0.00128	0.009	TMEM9B-A	8981221
chr11	8985609	8986649	1041	-0.52	0.00128	0.009	TMEM9B	8984320
chr13	20207497	20208141	645	-0.59	0.00128	0.009	MPHOSPH8	20202787
chr9	1E+08	1E+08	738	-0.5	0.00129	0.00905	XPA	1E+08
chr9	1E+08	1E+08	738	-0.5	0.00129	0.00905	XPA	1E+08
chr6	99962934	99964034	1101	-0.54	0.00129	0.00906	USP45	99961252
chr6	99962934	99964034	1101	-0.54	0.00129	0.00906	TSTD3	99963869
chr19	5903974	5904880	907	-0.45	0.00129	0.00907	NDUFA11	5902024
chr19	5903974	5904880	907	-0.45	0.00129	0.00907	NDUFA11	5902024
chr19	5903974	5904880	907	-0.45	0.00129	0.00907	NDUFA11	5902024
chr19	5903974	5904880	907	-0.45	0.00129	0.00907	VMAC	5899851

chr10	1.04E+08	1.04E+08	1070	-0.44	0.00129	0.00907	SUFU	1.04E+08
chr10	1.04E+08	1.04E+08	1070	-0.44	0.00129	0.00907	SUFU	1.04E+08
chr10	1.04E+08	1.04E+08	1070	-0.44	0.00129	0.00907	ACTR1A	1.04E+08
chr5	54529413	54529696	284	-1.03	0.00129	0.00908	CCNO	54527545
chr5	54529413	54529696	284	-1.03	0.00129	0.00908	CCNO	54527545
chr5	54529413	54529696	284	-1.03	0.00129	0.00908	CCNO	54526819
chr5	54529413	54529696	284	-1.03	0.00129	0.00908	CCNO	54527545
chr2	29199348	29199821	474	-0.65	0.00129	0.00909	FAM179A	29199163
chr11	1.1E+08	1.1E+08	693	-0.64	0.0013	0.00912	FDX1	1.1E+08
chr20	57607298	57607942	645	-0.54	0.0013	0.00913	ATP5E	57605422
chr7	56173955	56174501	547	-0.53	0.0013	0.00913	CHCHD2	56172187
chr7	7139132	7139497	366	-0.68	0.00131	0.00914	LOC100131	7134417
chr4	1.77E+08	1.77E+08	335	-0.53	0.00131	0.00915	ASB5	1.77E+08
chr12	11513205	11513441	237	-1.26	0.00131	0.00916	PRB1	11506524
chr12	11513205	11513441	237	-1.26	0.00131	0.00916	PRB1	11506524
chr12	11513205	11513441	237	-1.26	0.00131	0.00916	PRB1	11506524
chr7	23513704	23514305	602	-0.54	0.00131	0.00918	IGF2BP3	23507995
chr11	1.12E+08	1.12E+08	385	-0.65	0.00132	0.00918	LOC101928	1.12E+08
chr7	36644639	36645392	754	-0.58	0.00132	0.00919	AOAH-IT1	36637726
chr7	79763650	79764246	597	-0.62	0.00132	0.00919	GNAI1	79759139
chr7	79763650	79764246	597	-0.62	0.00132	0.00919	GNAI1	79760070
chr2	10091569	10091946	378	-0.86	0.00132	0.0092	GRHL1	10086791
chr12	71150028	71150253	226	-1	0.00132	0.00921	PTPRR	71146539
chr12	71150028	71150253	226	-1	0.00132	0.00921	PTPRR	71146388
chr12	71150028	71150253	226	-1	0.00132	0.00921	PTPRR	71146388
chr3	1.77E+08	1.77E+08	240	-1.24	0.00132	0.00922	LINC00578	1.77E+08
chr11	62599110	62599819	710	-0.51	0.00132	0.00923	STX5	62597563
chr11	62599110	62599819	710	-0.51	0.00132	0.00923	STX5	62597563
chr20	17660727	17661173	447	-0.7	0.00133	0.00923	RRBP1	17660928
chr20	17660727	17661173	447	-0.7	0.00133	0.00923	RRBP1	17660928
chr5	43313204	43313980	777	-0.41	0.00133	0.00924	HMGCS1	43311614
chr5	43313204	43313980	777	-0.41	0.00133	0.00924	HMGCS1	43311614
chr7	87849196	87849962	767	-0.57	0.00133	0.00924	SRI	87847399
chr7	87849196	87849962	767	-0.57	0.00133	0.00924	SRI	87847399
chr15	40526449	40526606	158	-1.11	0.00133	0.00925	PAK6	40526291
chr2	96931637	96932086	450	-0.62	0.00133	0.00925	CIAO1	96926883
chr2	96931637	96932086	450	-0.62	0.00133	0.00925	TMEM127	96929751
chr2	96931637	96932086	450	-0.62	0.00133	0.00925	TMEM127	96929751
chr6	89673176	89673674	499	-0.52	0.00133	0.00925	RNGTT	89671348
chr6	89673176	89673674	499	-0.52	0.00133	0.00925	RNGTT	89671348
chr6	89673176	89673674	499	-0.52	0.00133	0.00925	RNGTT	89671348
chr3	1.9E+08	1.9E+08	634	-0.45	0.00133	0.00926	IL1RAP	1.9E+08
chr3	1.9E+08	1.9E+08	634	-0.45	0.00133	0.00926	IL1RAP	1.9E+08
chr3	1.9E+08	1.9E+08	634	-0.45	0.00133	0.00926	IL1RAP	1.9E+08
chr3	1.9E+08	1.9E+08	634	-0.45	0.00133	0.00926	IL1RAP	1.9E+08
chr3	1.9E+08	1.9E+08	634	-0.45	0.00133	0.00926	IL1RAP	1.9E+08
chr3	1.9E+08	1.9E+08	634	-0.45	0.00133	0.00926	IL1RAP	1.9E+08

chr11	36531249	36532260	1012	-0.6	0.00133	0.00926	TRAF6	36529863
chr11	36531249	36532260	1012	-0.6	0.00133	0.00926	TRAF6	36529863
chr2	26256426	26257170	745	-0.62	0.00134	0.00927	RAB10	26251728
chr4	85219988	85220538	551	-1.1	0.00134	0.00927	LOC10192ε	85218322
chr5	1.15E+08	1.15E+08	763	-0.52	0.00134	0.00929	FEM1C	1.15E+08
chr17	33915467	33915840	374	-0.84	0.00134	0.0093	AP2B1	33909281
chr17	33915467	33915840	374	-0.84	0.00134	0.0093	AP2B1	33909281
chr19	38878104	38878736	633	-0.66	0.00135	0.00934	SPRED3	38875839
chr19	38878104	38878736	633	-0.66	0.00135	0.00934	GGN	38876668
chr19	38878104	38878736	633	-0.66	0.00135	0.00934	SPRED3	38875931
chr2	1.98E+08	1.98E+08	638	-0.61	0.00136	0.00938	ANKRD44	1.98E+08
chr2	1.98E+08	1.98E+08	638	-0.61	0.00136	0.00938	ANKRD44	1.98E+08
chr6	1.12E+08	1.12E+08	539	-0.63	0.00136	0.0094	MFSD4B	1.12E+08
chr3	1.41E+08	1.41E+08	1231	-0.58	0.00136	0.0094	RASA2	1.41E+08
chr3	1.41E+08	1.41E+08	1231	-0.58	0.00136	0.0094	RASA2	1.41E+08
chr3	1.41E+08	1.41E+08	1231	-0.58	0.00136	0.0094	RASA2	1.41E+08
chr16	84652414	84652728	315	-0.87	0.00137	0.00942	COTL1	84649702
chr2	37193385	37194091	707	-0.56	0.00137	0.00943	STRN	37191615
chr20	11871231	11872157	927	-0.55	0.00137	0.00943	BTBD3	11868140
chr20	11871231	11872157	927	-0.55	0.00137	0.00943	BTBD3	11866370
chr20	11871231	11872157	927	-0.55	0.00137	0.00943	BTBD3	11866370
chr14	24682265	24682846	582	-0.51	0.00137	0.00943	CHMP4A	24681036
chr3	1.01E+08	1.01E+08	304	-0.91	0.00137	0.00945	TRMT10C	1.01E+08
chr7	23338732	23339128	397	-0.71	0.00138	0.00947	MALSU1	23333939
chr12	85305064	85305531	468	-0.69	0.00138	0.00948	SLC6A15	85304608
chr12	85305064	85305531	468	-0.69	0.00138	0.00948	SLC6A15	85304608
chr12	85305064	85305531	468	-0.69	0.00138	0.00948	SLC6A15	85304608
chr12	75782994	75783200	207	-0.92	0.00138	0.0095	CAPS2	75782702
chr12	75782994	75783200	207	-0.92	0.00138	0.0095	CAPS2	75782702
chr12	75782994	75783200	207	-0.92	0.00138	0.0095	GLIPR1L2	75779849
chr12	75782994	75783200	207	-0.92	0.00138	0.0095	GLIPR1L2	75779849
chr12	75782994	75783200	207	-0.92	0.00138	0.0095	GLIPR1L2	75779849
chr1	2.22E+08	2.22E+08	1127	-0.44	0.00138	0.0095	DUSP10	2.22E+08
chr1	2.22E+08	2.22E+08	1127	-0.44	0.00138	0.0095	DUSP10	2.22E+08
chr8	95961547	95961929	383	-0.61	0.00139	0.00951	TP53INP1	95959615
chr8	95961547	95961929	383	-0.61	0.00139	0.00951	TP53INP1	95959615
chr3	1.54E+08	1.54E+08	311	-0.73	0.00139	0.00951	GPR149	1.54E+08
chr11	1.14E+08	1.14E+08	855	-0.53	0.00139	0.00951	RBM7	1.14E+08
chr11	1.14E+08	1.14E+08	855	-0.53	0.00139	0.00951	C11orf71	1.14E+08
chr11	1.14E+08	1.14E+08	855	-0.53	0.00139	0.00951	RBM7	1.14E+08
chr11	1.14E+08	1.14E+08	855	-0.53	0.00139	0.00951	C11orf71	1.14E+08
chr11	1.14E+08	1.14E+08	855	-0.53	0.00139	0.00951	RBM7	1.14E+08
chr11	1.14E+08	1.14E+08	855	-0.53	0.00139	0.00951	RBM7	1.14E+08
chr11	1.14E+08	1.14E+08	855	-0.53	0.00139	0.00951	RBM7	1.14E+08
chr8	82633030	82633823	794	-0.56	0.00139	0.00953	ZFAND1	82631539
chr8	82633030	82633823	794	-0.56	0.00139	0.00953	ZFAND1	82631539
chr8	82633030	82633823	794	-0.56	0.00139	0.00953	ZFAND1	82631539

chr8	82633030	82633823	794	-0.56	0.00139	0.00953	ZFAND1	82631539
chr8	82633030	82633823	794	-0.56	0.00139	0.00953	ZFAND1	82631539
chr8	82633030	82633823	794	-0.56	0.00139	0.00953	ZFAND1	82631539
chr8	82633030	82633823	794	-0.56	0.00139	0.00953	ZFAND1	82631539
chr12	1.02E+08	1.02E+08	971	-0.48	0.00139	0.00953	DRAM1	1.02E+08
chr5	1.18E+08	1.18E+08	342	-1.02	0.00139	0.00953	LOC10537‹	1.18E+08
chr7	16684985	16685890	906	-0.49	0.00139	0.00954	ANKMY2	16683442
chr7	16684985	16685890	906	-0.49	0.00139	0.00954	BZW2	16680758
chr7	16684985	16685890	906	-0.49	0.00139	0.00954	BZW2	16680758
chr7	16684985	16685890	906	-0.49	0.00139	0.00954	BZW2	16680758
chr3	1.91E+08	1.91E+08	406	-0.62	0.0014	0.00956	GMNC	1.91E+08
chr2	1.37E+08	1.37E+08	927	-0.55	0.0014	0.00958	MCM6	1.37E+08
chr12	70636591	70637601	1011	-0.7	0.0014	0.00958	CNOT2	70632493
chr12	70636591	70637601	1011	-0.7	0.0014	0.00958	CNOT2	70632493
chr12	70636591	70637601	1011	-0.7	0.0014	0.00958	CNOT2	70631809
chr12	70636591	70637601	1011	-0.7	0.0014	0.00958	CNOT2	70631773
chr12	70636591	70637601	1011	-0.7	0.0014	0.00958	LINC01481	70635140
chr12	70636591	70637601	1011	-0.7	0.0014	0.00958	LINC01481	70635140
chr12	70636591	70637601	1011	-0.7	0.0014	0.00958	LINC01481	70635140
chr10	1.28E+08	1.28E+08	392	-0.7	0.0014	0.00958	DHX32	1.28E+08
chr12	28406105	28406475	371	-1.26	0.0014	0.00959	CCDC91	28405132
chr8	95565521	95566074	554	-0.55	0.00141	0.0096	KIAA1429	95563746
chr8	95565521	95566074	554	-0.55	0.00141	0.0096	KIAA1429	95563746
chr17	21187624	21188036	413	-0.53	0.00141	0.0096	MAP2K3	21186347
chr17	21187624	21188036	413	-0.53	0.00141	0.0096	MAP2K3	21182967
chr17	21187624	21188036	413	-0.53	0.00141	0.0096	MAP2K3	21182967
chr1	95582286	95583363	1078	-0.54	0.00141	0.00962	TMEM56-R	95578478
chr1	95582286	95583363	1078	-0.54	0.00141	0.00962	TMEM56	95577893
chr11	3876134	3877011	878	-0.5	0.00141	0.00962	STIM1	3871932
chr11	3876134	3877011	878	-0.5	0.00141	0.00962	STIM1	3871932
chr11	3876134	3877011	878	-0.5	0.00141	0.00962	STIM1	3871932
chr11	3876134	3877011	878	-0.5	0.00141	0.00962	MIR4687	3872291
chr8	25902859	25903095	237	-0.93	0.00141	0.00963	EBF2	25900640
chr6	11093711	11094571	861	-0.54	0.00141	0.00964	SMIM13	11089265
chr6	38670714	38671133	420	-0.65	0.00142	0.00964	GLO1	38668952
chr2	70141647	70142265	619	-0.5	0.00142	0.00965	MXD1	70137172
chr2	70141647	70142265	619	-0.5	0.00142	0.00965	MXD1	70137172
chr2	70141647	70142265	619	-0.5	0.00142	0.00965	MXD1	70137172
chr1	1.6E+08	1.6E+08	203	-1.18	0.00142	0.00965	FCRL6	1.6E+08
chr1	1.6E+08	1.6E+08	203	-1.18	0.00142	0.00965	FCRL6	1.6E+08
chr7	56118995	56119610	616	-0.42	0.00142	0.00965	PSPH	56117268
chr7	56118995	56119610	616	-0.42	0.00142	0.00965	CCT6A	56114377
chr7	56118995	56119610	616	-0.42	0.00142	0.00965	CCT6A	56114377
chr6	42896858	42897474	617	-0.58	0.00142	0.00965	CNPY3	42890739
chr6	42896858	42897474	617	-0.58	0.00142	0.00965	CNPY3	42891867
chr6	42896858	42897474	617	-0.58	0.00142	0.00965	CNPY3	42891867
chr6	42896858	42897474	617	-0.58	0.00142	0.00965	CNPY3	42891867

chr6	42896858	42897474	617	-0.58	0.00142	0.00965	CNPY3	42891867
chr6	42896858	42897474	617	-0.58	0.00142	0.00965	CNPY3	42891867
chr6	42896858	42897474	617	-0.58	0.00142	0.00965	CNPY3	42891867
chr6	42896858	42897474	617	-0.58	0.00142	0.00965	CNPY3	42891867
chr6	42896858	42897474	617	-0.58	0.00142	0.00965	CNPY3	42891867
chr6	42896858	42897474	617	-0.58	0.00142	0.00965	CNPY3	42891867
chr6	42896858	42897474	617	-0.58	0.00142	0.00965	CNPY3	42891867
chr6	42896858	42897474	617	-0.58	0.00142	0.00965	CNPY3-GNI	42891867
chr6	42896858	42897474	617	-0.58	0.00142	0.00965	CNPY3-GNI	42891867
chr6	42896858	42897474	617	-0.58	0.00142	0.00965	CNPY3-GNI	42891867
chr6	42896858	42897474	617	-0.58	0.00142	0.00965	CNPY3-GNI	42891867
chr6	42896858	42897474	617	-0.58	0.00142	0.00965	CNPY3-GNI	42891867
chr6	42896858	42897474	617	-0.58	0.00142	0.00965	CNPY3-GNI	42891867
chr6	83777272	83777915	644	-0.53	0.00142	0.00966	UBE3D	83773560
chr6	83777272	83777915	644	-0.53	0.00142	0.00966	UBE3D	83773560
chr6	83777272	83777915	644	-0.53	0.00142	0.00966	DOPEY1	83772384
chr6	83777272	83777915	644	-0.53	0.00142	0.00966	DOPEY1	83772384
chr12	77459072	77459933	862	-0.46	0.00143	0.00969	E2F7	77457360
chr12	46122520	46122898	379	-0.58	0.00143	0.00969	ARID2	46118619
chr12	46122520	46122898	379	-0.58	0.00143	0.00969	LINC00938	46119704
chr9	15307104	15307569	466	-0.64	0.00143	0.0097	TTC39B	15305358
chr9	15307104	15307569	466	-0.64	0.00143	0.0097	TTC39B	15305358
chr9	15307104	15307569	466	-0.64	0.00143	0.0097	TTC39B	15305358
chr9	15307104	15307569	466	-0.64	0.00143	0.0097	TTC39B	15305358
chr17	54670764	54671437	674	-0.79	0.00143	0.00972	NOG	54666059
chr6	52441398	52442318	921	-0.54	0.00143	0.00972	TRAM2	52439862
chr6	52441398	52442318	921	-0.54	0.00143	0.00972	TRAM2-AS:	52437095
chr6	52441398	52442318	921	-0.54	0.00143	0.00972	TRAM2-AS:	52436999
chr14	1E+08	1E+08	657	-0.52	0.00144	0.00975	CYP46A1	1E+08
chr3	66551293	66551897	605	-0.54	0.00144	0.00975	LRIG1	66548845
chr9	1.14E+08	1.14E+08	685	-0.5	0.00144	0.00976	DNAJC25-G	1.14E+08
chr9	1.14E+08	1.14E+08	685	-0.5	0.00144	0.00976	DNAJC25	1.14E+08
chr9	1.14E+08	1.14E+08	685	-0.5	0.00144	0.00976	DNAJC25	1.14E+08
chr16	58549223	58550233	1011	-0.61	0.00144	0.00976	SETD6	58544382
chr16	58549223	58550233	1011	-0.61	0.00144	0.00976	SETD6	58544382
chr16	58549223	58550233	1011	-0.61	0.00144	0.00976	SETD6	58544382
chr11	1.23E+08	1.23E+08	592	-0.6	0.00145	0.00978	UBASH3B	1.23E+08
chr1	71546391	71547137	747	-0.63	0.00145	0.00978	ZRANB2-AS	71542006
chr1	71546391	71547137	747	-0.63	0.00145	0.00978	ZRANB2	71544972
chr1	71546391	71547137	747	-0.63	0.00145	0.00978	ZRANB2	71544972
chr2	71454120	71454740	621	-0.63	0.00145	0.00979	PAIP2B	71452233
chr1	2.1E+08	2.1E+08	428	-0.69	0.00146	0.00983	C1orf74	2.1E+08
chr15	59062922	59063542	621	-0.65	0.00146	0.00984	FAM63B	59058392
chr15	59062922	59063542	621	-0.65	0.00146	0.00984	FAM63B	59058392
chr19	57988723	57989176	454	-0.95	0.00146	0.00986	ZNF772	57986938
chr19	57988723	57989176	454	-0.95	0.00146	0.00986	ZNF772	57986938
chrX	1.2E+08	1.2E+08	574	-0.58	0.00146	0.00987	C1GALT1C1	1.2E+08

chrX	1.2E+08	1.2E+08	574	-0.58	0.00146	0.00987	C1GALT1C1	1.2E+08
chr17	17744171	17744474	304	-0.72	0.00147	0.00987	SREBF1	17738325
chr17	17744171	17744474	304	-0.72	0.00147	0.00987	SREBF1	17738325
chrX	16804256	16804774	519	-0.53	0.00147	0.00987	TXLNG	16799554
chrX	16804256	16804774	519	-0.53	0.00147	0.00987	TXLNG	16799554
chr7	1.49E+08	1.49E+08	642	-0.51	0.00147	0.00989	PDIA4	1.49E+08
chr2	9983415	9983973	559	-0.68	0.00147	0.0099	TAF1B	9978570
chr2	9983415	9983973	559	-0.68	0.00147	0.0099	TAF1B	9978570
chr2	9983415	9983973	559	-0.68	0.00147	0.0099	TAF1B	9978570
chr1	1.87E+08	1.87E+08	833	-0.53	0.00147	0.0099	PTGS2	1.87E+08
chr1	1.87E+08	1.87E+08	833	-0.53	0.00147	0.0099	PACERR	1.87E+08
chr1	59481377	59481797	421	-1.04	0.00148	0.00992	LINC01358	59481147
chr12	8833731	8834273	543	-0.66	0.00148	0.00992	RIMKLB	8829272
chr6	99280647	99281162	516	-0.64	0.00148	0.00993	POU3F2	99277579
chr1	1.66E+08	1.66E+08	707	-0.48	0.00148	0.00993	MGST3	1.66E+08
chr19	4723836	4724112	277	-0.91	0.00149	0.00997	DPP9	4721855
chr1	51435053	51435863	811	-0.65	0.00149	0.00998	CDKN2C	51429366
chr1	51435053	51435863	811	-0.65	0.00149	0.00998	CDKN2C	51430641
chr19	54484219	54484794	576	-0.61	0.00149	0.00998	MIR935	54480560
chr2	1.06E+08	1.06E+08	199	-1.32	0.00149	0.00999	GPR45	1.06E+08
chr1	1.15E+08	1.15E+08	619	-0.71	0.00149	0.01	OLFML3	1.15E+08
chr1	1.15E+08	1.15E+08	619	-0.71	0.00149	0.01	OLFML3	1.15E+08
chr1	1.15E+08	1.15E+08	619	-0.71	0.00149	0.01	OLFML3	1.15E+08
chr5	81574079	81574616	538	-0.77	0.00149	0.01	RPS23	81572235
chr5	81574079	81574616	538	-0.77	0.00149	0.01	ATP6AP1L	81570280
chr6	90062017	90062739	723	-0.64	0.0015	0.01	UBE2J1	90060619
chr8	99956402	99956823	422	-0.63	0.0015	0.01	STK3	99952799
chr8	99956402	99956823	422	-0.63	0.0015	0.01	OSR2	99951630
chr8	99956402	99956823	422	-0.63	0.0015	0.01	OSR2	99951630
chr8	99956402	99956823	422	-0.63	0.0015	0.01	OSR2	99951630
chr12	13251221	13251882	662	-0.54	0.0015	0.01	GSG1	13246740
chr12	13251221	13251882	662	-0.54	0.0015	0.01	GSG1	13246740
chr12	13251221	13251882	662	-0.54	0.0015	0.01	GSG1	13246740
chr12	13251221	13251882	662	-0.54	0.0015	0.01	GSG1	13246740
chr6	73331785	73332498	714	-0.86	0.0015	0.01	KCNQ5	73326570
chr6	73331785	73332498	714	-0.86	0.0015	0.01	KCNQ5	73326570
chr6	73331785	73332498	714	-0.86	0.0015	0.01	KCNQ5	73326570
chr6	73331785	73332498	714	-0.86	0.0015	0.01	KCNQ5	73326570
chr6	73331785	73332498	714	-0.86	0.0015	0.01	KCNQ5	73326570
chr5	94890362	94891163	802	-0.55	0.0015	0.01	ARSK	94885824
chr5	94890362	94891163	802	-0.55	0.0015	0.01	TTC37	94888709
chr15	45571322	45571748	427	-0.62	0.0015	0.0101	LOC10192ε	45569420
chr6	1.52E+08	1.52E+08	175	-1.54	0.00151	0.0101	AKAP12	1.52E+08
chr4	1.41E+08	1.41E+08	742	-0.57	0.00151	0.0101	ELMOD2	1.41E+08
chr12	1.21E+08	1.21E+08	1405	-0.55	0.00151	0.0101	GCN1	1.21E+08
chr3	1.19E+08	1.19E+08	559	-0.54	0.00152	0.0101	B4GALT4	1.19E+08
chr3	1.19E+08	1.19E+08	559	-0.54	0.00152	0.0101	B4GALT4	1.19E+08

chr20	10654588	10655122	535	-0.52	0.00152	0.0101	JAG1	10652694
chr2	1.27E+08	1.27E+08	769	-0.53	0.00152	0.0101	GYPC	1.27E+08
chr2	1.27E+08	1.27E+08	769	-0.53	0.00152	0.0101	GYPC	1.27E+08
chr2	1.27E+08	1.27E+08	769	-0.53	0.00152	0.0101	GYPC	1.27E+08
chr13	52585859	52586583	725	-0.59	0.00152	0.0101	ATP7B	52583630
chr13	52585859	52586583	725	-0.59	0.00152	0.0101	ATP7B	52583630
chr13	52585859	52586583	725	-0.59	0.00152	0.0101	ATP7B	52583630
chr13	52585859	52586583	725	-0.59	0.00152	0.0101	ALG11	52581522
chr13	52585859	52586583	725	-0.59	0.00152	0.0101	ALG11	52581522
chr2	40661966	40662400	435	-0.71	0.00153	0.0102	SLC8A1	40655444
chr2	40661966	40662400	435	-0.71	0.00153	0.0102	SLC8A1	40655444
chr2	40661966	40662400	435	-0.71	0.00153	0.0102	SLC8A1	40655444
chr2	40661966	40662400	435	-0.71	0.00153	0.0102	SLC8A1	40655444
chr13	76055941	76056854	914	-0.62	0.00153	0.0102	TBC1D4	76054304
chr13	76055941	76056854	914	-0.62	0.00153	0.0102	TBC1D4	76054304
chr13	76055941	76056854	914	-0.62	0.00153	0.0102	TBC1D4	76054304
chr5	34929279	34930295	1017	-0.42	0.00153	0.0102	DNAJC21	34924697
chr5	34929279	34930295	1017	-0.42	0.00153	0.0102	DNAJC21	34924697
chr4	80413293	80413572	280	-1.21	0.00153	0.0102	LINC00989	80408746
chr2	1.31E+08	1.31E+08	1083	-0.45	0.00154	0.0102	IMP4	1.31E+08
chr2	1.31E+08	1.31E+08	1083	-0.45	0.00154	0.0102	CCDC115	1.31E+08
chr2	1.31E+08	1.31E+08	1083	-0.45	0.00154	0.0102	CCDC115	1.31E+08
chr5	96212846	96213202	357	-0.82	0.00154	0.0102	ERAP2	96206643
chr5	96212846	96213202	357	-0.82	0.00154	0.0102	ERAP2	96206643
chr14	85997961	85998672	712	-0.48	0.00156	0.0103	FLRT2	85991487
chr6	1.1E+08	1.1E+08	212	-1.2	0.00156	0.0103	CCDC162P	1.1E+08
chr1	2.07E+08	2.07E+08	567	-0.77	0.00156	0.0103	IL24	2.07E+08
chr1	2.07E+08	2.07E+08	567	-0.77	0.00156	0.0103	IL24	2.07E+08
chr1	2.07E+08	2.07E+08	567	-0.77	0.00156	0.0103	IL24	2.07E+08
chr1	2.07E+08	2.07E+08	567	-0.77	0.00156	0.0103	IL24	2.07E+08
chr9	1.1E+08	1.1E+08	230	-1.2	0.00156	0.0103	LINC01509	1.1E+08
chr2	38978143	38978801	659	-0.74	0.00156	0.0103	SRSF7	38976636
chr2	38978143	38978801	659	-0.74	0.00156	0.0103	SRSF7	38976636
chr9	1.31E+08	1.31E+08	729	-0.53	0.00157	0.0103	FAM102A	1.31E+08
chr9	1.08E+08	1.08E+08	560	-0.58	0.00157	0.0103	FKTN	1.08E+08
chr9	1.08E+08	1.08E+08	560	-0.58	0.00157	0.0103	FKTN	1.08E+08
chr9	1.08E+08	1.08E+08	560	-0.58	0.00157	0.0103	FKTN	1.08E+08
chr8	1.24E+08	1.24E+08	239	-0.65	0.00157	0.0103	FAM83A	1.24E+08
chr8	1.24E+08	1.24E+08	239	-0.65	0.00157	0.0103	FAM83A	1.24E+08
chr8	1.24E+08	1.24E+08	239	-0.65	0.00157	0.0103	FAM83A	1.24E+08
chr4	1.03E+08	1.03E+08	426	-0.84	0.00157	0.0103	SLC39A8	1.03E+08
chr4	1.03E+08	1.03E+08	426	-0.84	0.00157	0.0103	SLC39A8	1.03E+08
chr4	1.03E+08	1.03E+08	426	-0.84	0.00157	0.0103	SLC39A8	1.03E+08
chr9	21559526	21560092	567	-0.86	0.00157	0.0104	MIR31HG	21557697
chr16	10673937	10674701	765	-0.6	0.00157	0.0104	EMP2	10672573
chr1	1.01E+08	1.01E+08	785	-0.44	0.00158	0.0104	SLC30A7	1.01E+08
chr1	1.01E+08	1.01E+08	785	-0.44	0.00158	0.0104	EXTL2	1.01E+08

chr1	1.01E+08	1.01E+08	785	-0.44	0.00158	0.0104	EXTL2	1.01E+08
chr1	1.01E+08	1.01E+08	785	-0.44	0.00158	0.0104	EXTL2	1.01E+08
chr1	1.01E+08	1.01E+08	785	-0.44	0.00158	0.0104	EXTL2	1.01E+08
chr1	1.01E+08	1.01E+08	785	-0.44	0.00158	0.0104	EXTL2	1.01E+08
chr1	1.01E+08	1.01E+08	785	-0.44	0.00158	0.0104	EXTL2	1.01E+08
chr1	1.01E+08	1.01E+08	785	-0.44	0.00158	0.0104	SLC30A7	1.01E+08
chr3	33481833	33482590	758	-0.87	0.00158	0.0104	UBP1	33479870
chr3	33481833	33482590	758	-0.87	0.00158	0.0104	UBP1	33479870
chr3	33481833	33482590	758	-0.87	0.00158	0.0104	UBP1	33479897
chr11	1.29E+08	1.29E+08	1294	-0.56	0.00159	0.0104	FLI1	1.29E+08
chr11	1.29E+08	1.29E+08	1294	-0.56	0.00159	0.0104	FLI1	1.29E+08
chr11	1.29E+08	1.29E+08	1294	-0.56	0.00159	0.0104	FLI1	1.29E+08
chr11	1.29E+08	1.29E+08	1294	-0.56	0.00159	0.0104	SENCR	1.29E+08
chr12	1.11E+08	1.11E+08	1169	-0.54	0.00159	0.0104	GPN3	1.11E+08
chr12	1.11E+08	1.11E+08	1169	-0.54	0.00159	0.0104	GPN3	1.11E+08
chr12	1.11E+08	1.11E+08	1169	-0.54	0.00159	0.0104	GPN3	1.11E+08
chr12	1.11E+08	1.11E+08	1169	-0.54	0.00159	0.0104	FAM216A	1.11E+08
chr3	1.96E+08	1.96E+08	245	-0.65	0.00159	0.0104	TNK2	1.96E+08
chr12	3598162	3598545	384	-0.81	0.0016	0.0105	PRMT8	3595363
chr6	88117733	88118546	814	-0.54	0.0016	0.0105	CFAP206	88112689
chr5	1.31E+08	1.31E+08	1065	-0.44	0.0016	0.0105	HINT1	1.3E+08
chr5	1.31E+08	1.31E+08	1065	-0.44	0.0016	0.0105	HINT1	1.3E+08
chr5	1.31E+08	1.31E+08	1065	-0.44	0.0016	0.0105	HINT1	1.3E+08
chr5	1.31E+08	1.31E+08	1065	-0.44	0.0016	0.0105	HINT1	1.3E+08
chr5	1.31E+08	1.31E+08	1065	-0.44	0.0016	0.0105	HINT1	1.3E+08
chr5	1.31E+08	1.31E+08	1065	-0.44	0.0016	0.0105	HINT1	1.3E+08
chr7	1.56E+08	1.56E+08	251	-1.17	0.0016	0.0105	LINC01006	1.56E+08
chr8	80677507	80677682	176	-1.09	0.0016	0.0105	HEY1	80676710
chr8	80677507	80677682	176	-1.09	0.0016	0.0105	LINC01607	80675376
chr6	64281701	64282544	844	-0.45	0.0016	0.0105	PTP4A1	64276916
chr16	30076421	30077180	760	-0.46	0.00161	0.0105	ALDOA	30070817
chr16	30076421	30077180	760	-0.46	0.00161	0.0105	ALDOA	30070599
chr16	30076421	30077180	760	-0.46	0.00161	0.0105	ALDOA	30071993
chr16	30076421	30077180	760	-0.46	0.00161	0.0105	ALDOA	30071993
chr1	1.52E+08	1.52E+08	605	-0.77	0.00161	0.0105	MIR554	1.52E+08
chr11	71497664	71498666	1003	-0.33	0.00161	0.0105	FAM86C1	71493556
chr11	71497664	71498666	1003	-0.33	0.00161	0.0105	FAM86C1	71493556
chr11	71497664	71498666	1003	-0.33	0.00161	0.0105	FAM86C1	71493556
chr2	1.67E+08	1.67E+08	1016	-0.52	0.00162	0.0105	GALNT3	1.67E+08
chr16	4666160	4666656	497	-0.62	0.00162	0.0105	UBALD1	4662927
chr15	69884338	69884500	163	-1.19	0.00162	0.0106	PCAT29	69879538
chr15	69884338	69884500	163	-1.19	0.00162	0.0106	PCAT29	69879538
chr6	72129950	72130812	863	-0.54	0.00163	0.0106	LINC00472	72128448
chr6	72129950	72130812	863	-0.54	0.00163	0.0106	LINC01626	72125729
chr6	72129950	72130812	863	-0.54	0.00163	0.0106	LINC00472	72128448
chr6	72129950	72130812	863	-0.54	0.00163	0.0106	LINC00472	72128448
chr6	72129950	72130812	863	-0.54	0.00163	0.0106	LINC00472	72128448

chr15	59949471	59949940	470	-0.8	0.00163	0.0106	GTF2A2	59947737
chr3	1.11E+08	1.11E+08	290	-0.73	0.00163	0.0106	ZBED2	1.11E+08
chr4	1.86E+08	1.86E+08	347	-0.73	0.00163	0.0106	ACSL1	1.86E+08
chr19	40949006	40949332	327	-0.74	0.00163	0.0106	SERTAD3	40948282
chr19	40949006	40949332	327	-0.74	0.00163	0.0106	SERTAD3	40946505
chr17	16945396	16945882	487	-0.75	0.00163	0.0106	MPRIP	16941073
chr17	16945396	16945882	487	-0.75	0.00163	0.0106	MPRIP	16941073
chr9	95055908	95056294	387	-0.62	0.00164	0.0106	IARS	95054038
chr9	95055908	95056294	387	-0.62	0.00164	0.0106	IARS	95054038
chr9	95055908	95056294	387	-0.62	0.00164	0.0106	IARS	95054038
chr9	95055908	95056294	387	-0.62	0.00164	0.0106	SNORA84	95052875
chr9	95055908	95056294	387	-0.62	0.00164	0.0106	MIR3651	95052829
chr10	46167915	46168551	637	-0.49	0.00164	0.0106	ZFAND4	46166261
chr10	46167915	46168551	637	-0.49	0.00164	0.0106	ZFAND4	46166261
chr10	46167915	46168551	637	-0.49	0.00164	0.0106	ZFAND4	46166180
chr10	46167915	46168551	637	-0.49	0.00164	0.0106	ZFAND4	46166261
chr10	46167915	46168551	637	-0.49	0.00164	0.0106	ZFAND4	46166180
chr14	91976474	91977171	698	-0.53	0.00164	0.0107	PPP4R3A	91974824
chr14	91976474	91977171	698	-0.53	0.00164	0.0107	PPP4R3A	91974824
chr20	5107119	5107711	593	-0.47	0.00164	0.0107	CDS2	5102406
chr20	5107119	5107711	593	-0.47	0.00164	0.0107	PCNA	5105268
chr9	1.01E+08	1.01E+08	532	-0.61	0.00164	0.0107	TRIM14	1.01E+08
chr9	1.01E+08	1.01E+08	532	-0.61	0.00164	0.0107	TRIM14	1.01E+08
chr5	1.21E+08	1.21E+08	411	-0.61	0.00164	0.0107	SRFBP1	1.21E+08
chr16	734202	734715	514	-0.68	0.00164	0.0107	JMJD8	732439
chr9	91794931	91795495	565	-0.59	0.00165	0.0107	SHC3	91791682
chr1	1.71E+08	1.71E+08	1211	-0.43	0.00165	0.0107	PRRC2C	1.71E+08
chr7	21985161	21986160	1000	-0.52	0.00165	0.0107	CDCA7L	21983542
chr7	21985161	21986160	1000	-0.52	0.00165	0.0107	CDCA7L	21983542
chr7	21985161	21986160	1000	-0.52	0.00165	0.0107	CDCA7L	21983542
chr14	21737544	21738124	581	-0.52	0.00165	0.0107	HNRNPC	21735638
chr14	21737544	21738124	581	-0.52	0.00165	0.0107	HNRNPC	21735638
chr14	21737544	21738124	581	-0.52	0.00165	0.0107	HNRNPC	21735638
chr14	21737544	21738124	581	-0.52	0.00165	0.0107	HNRNPC	21735638
chr2	1.63E+08	1.63E+08	532	-0.58	0.00166	0.0107	FAP	1.63E+08
chr2	1.63E+08	1.63E+08	532	-0.58	0.00166	0.0107	FAP	1.63E+08
chr1	1.54E+08	1.54E+08	761	-0.62	0.00166	0.0107	C1orf43	1.54E+08
chr1	1.54E+08	1.54E+08	761	-0.62	0.00166	0.0107	C1orf43	1.54E+08
chr1	1.54E+08	1.54E+08	761	-0.62	0.00166	0.0107	C1orf43	1.54E+08
chr1	1.54E+08	1.54E+08	761	-0.62	0.00166	0.0107	UBAP2L	1.54E+08
chr1	1.54E+08	1.54E+08	761	-0.62	0.00166	0.0107	C1orf43	1.54E+08
chr1	1.54E+08	1.54E+08	761	-0.62	0.00166	0.0107	C1orf43	1.54E+08
chr1	1.54E+08	1.54E+08	761	-0.62	0.00166	0.0107	UBAP2L	1.54E+08
chr1	1.54E+08	1.54E+08	761	-0.62	0.00166	0.0107	C1orf43	1.54E+08
chr1	1.54E+08	1.54E+08	761	-0.62	0.00166	0.0107	UBAP2L	1.54E+08
chr1	1.54E+08	1.54E+08	761	-0.62	0.00166	0.0107	C1orf43	1.54E+08
chr1	1.54E+08	1.54E+08	761	-0.62	0.00166	0.0107	UBAP2L	1.54E+08

chr1	1.54E+08	1.54E+08	761	-0.62	0.00166	0.0107	C1orf43	1.54E+08
[truncated: 547,746 more chars]
